# Epigenetic signature of chronic low back pain in human T cells

**DOI:** 10.1097/PR9.0000000000000960

**Published:** 2021-11-03

**Authors:** Stéphanie Grégoire, David Cheishvili, Mali Salmon-Divon, Sergiy Dymov, Lucas Topham, Virginie Calderon, Yoram Shir, Moshe Szyf, Laura S. Stone

**Affiliations:** a Alan Edwards Centre for Research on Pain, McGill University, Montreal, QC, Canada; b Faculty of Dentistry, McGill University, Montreal, QC, Canada; c HKG Epitherapeutics, Honk Kong, China; d Department of Oncology, McGill University, Montreal, QC, Canada; e Department of Molecular Biology and Adelson School of Medicine, Ariel University, Ariel, Israel; f Department of Pharmacology and Therapeutics, McGill University, Montreal, QC, Canada; g Institut de Recherches Cliniques de Montréal, Montreal, QC, Canada; h Department of Anesthesiology, McGill University, Montreal, QC, Canada; i Alan Edwards Pain Management Unit, Montreal General Hospital, Montreal, QC, Canada; j Sackler Program for Epigenetics and Psychobiology, McGill University, Montreal, QC, Canada; k Department of Neurology and Neurosurgery, McGill University, Montreal, QC, Canada; l Department of Anesthesiology, University of Minnesota, Minneapolis, MN, USA

**Keywords:** DNA methylation, Low back pain, T cells, Biomarker, Epigenetics

## Abstract

**Objective:**

Determine if chronic low back pain (LBP) is associated with DNA methylation signatures in human T cells that will reveal novel mechanisms and potential therapeutic targets and explore the feasibility of epigenetic diagnostic markers for pain-related pathophysiology.

**Methods:**

Genome-wide DNA methylation analysis of 850,000 CpG sites in women and men with chronic LBP and pain-free controls was performed. T cells were isolated *(*discovery cohort, n = 32) and used to identify differentially methylated CpG sites, and gene ontologies and molecular pathways were identified. A polygenic DNA methylation score for LBP was generated in both women and men. Validation was performed in an independent cohort *(*validation cohort, n = 63) of chronic LBP and healthy controls.

**Results:**

Analysis with the discovery cohort revealed a total of 2,496 and 419 differentially methylated CpGs in women and men, respectively. In women, most of these sites were hypomethylated and enriched in genes with functions in the extracellular matrix, in the immune system (ie, cytokines), or in epigenetic processes. In men, a unique chronic LBP DNA methylation signature was identified characterized by significant enrichment for genes from the major histocompatibility complex. Sex-specific polygenic DNA methylation scores were generated to estimate the pain status of each individual and confirmed in the validation cohort using pyrosequencing.

**Conclusion:**

This study reveals sex-specific DNA methylation signatures in human T cells that discriminates chronic LBP participants from healthy controls.

## Abstract 2

Supplemental Digital Content is Available in the Text.

This study reveals sex-specific DNA methylation signatures in human T cells that discriminate chronic low back pain participants from healthy controls.

## 1. Introduction

Chronic pain is debilitating, difficult to treat, and often due to unknown causes. With a life-time prevalence of >80%, low back pain (LBP) is a leading cause of global years lived with disability, with other musculoskeletal conditions, neck pain, and migraine joining in the top 10.^22^ Therapeutic interventions are often inadequate or associated with undesired consequences; these limitations are amplified by concerns related to opioid misuse. Roadblocks to the development of nonopioid pain medications include a paucity of validated targets, the lack of diagnostic markers for pain-related pathologies, and a high degree of interindividual variation in response to interventions.

Chronic pain is associated with long-term changes in gene expression that are, at least in part, under epigenetic control.^39^ Epigenetics refers to modifications that alter gene expression without changing the genetic code. Epigenetic mechanisms include DNA methylation, modulation of the chromatin structure, and noncoding RNAs.^39^ Understanding epigenetic signatures provides insights into underlying disease mechanisms and are emerging as diagnostic biomarkers for complex diseases.^31^ For example, studies have demonstrated broad changes in DNA methylation in blood cells that are sensitive to life experiences including maternal stress, depression, early life abuse, and social economic status in humans^9,11,35^ as well as maternal deprivation in rhesus monkeys.^40^ We and others previously reported chronic pain–related changes in DNA methylation in animal models and in chronic LBP patients,^1,3,30,44,45^ and differential DNA methylation in blood is related to pain sensitivity in healthy subjects.^8^

Chronic pain is associated with the activation of the immune system including increased levels of circulating proinflammatory cytokines, and a role for the adaptative immune system—comprised of B and T cells—is emerging.^28^ In a proof-of-concept study in rats with peripheral nerve injury, we reported widespread changes in DNA methylation in T cells that partially overlapped with changes in the prefrontal cortex and were predictive of the presence and the severity of neuropathic pain.^30^

We hypothesize that chronic pain in humans is associated with DNA methylation signatures that will (1) reveal novel therapeutic targets, (2) provide diagnostic markers for pain-related pathophysiology, and (3) offer insights into the high degree of interindividual variability in chronic pain populations. Genome-wide DNA methylation of 850,000 cytosine-guanine CpG sites in T cells from female and male participants with chronic LBP were compared with pain-free controls *(*discovery cohort, n = 32). Differentially methylated CpG sites were identified and used to generate a polygenic methylation score for LBP in women and men. An independent cohort (validation cohort, n = 63) of LBP participants and healthy controls was used for validation. Finally, molecular, biological, and cellular functions that are epigenetically dysregulated in individuals living with chronic LBP were identified. These data implicate epigenetic regulation in LBP in humans, identify potential pathological drivers, and raise the potential for clinically useful DNA methylation markers for chronic pain in humans.

## 2. Methods

### 2.1. Study design and participants

This study was approved by the Research Ethics Board of the McGill University Health Center (#MP-CUSM-13-402). To minimize selection bias, participants were recruited from community advertisements and all eligible participants were accepted. Participation consisted of a single visit to the Montreal General Hospital (recruitment from 2016 to 2019) during which subjects completed questionnaires and a blood sample was collected. This study consisted of 2 cohorts (Table 1): the discovery cohort (n = 32; women: 8 controls and 8 LBP; men: 8 controls and 8 LBP) and the validation cohort (n = 63; women: 13 controls and 19 LBP; men: 13 controls and 18 LBP). The discovery cohort was selected from the first 75 participants and was intentionally balanced for age and sex. No formal sample size analysis was performed a priori because no data were available on T-cell DNA methylation in patients with chronic LBP. However, given the actual average methylation variance of 3%, n = 8 would enable the detection of differences of 10% or greater at individual CpG at α = 0.05 and power of 0.8 after correcting for multiple comparisons. Inclusion criteria for LBP included self-reported LBP for at least 1 year with an average daily pain score of at least 3/10. Etiology was not addressed in this study. Exclusion criteria for all participants were body mass index >35, pregnancy, age outside of 25 to 65, presence of other chronic pain conditions, and previous back surgery.

**Table 1:** Demographic and clinical characteristics of controls and low back pain participants in the (A) discovery and (B) validation cohorts*.

Parameters	Groups					
	Control women	LBP women	*P*	Control men	LBP men	*P*
(A) Discovery cohort						
Number	8	8	—	8	8	—
Age	43.8 ± 4.6	41.3 ± 3.8	n.s.	43.8 ± 4.0	42.6 ± 3.6	n.s.
Height	162.1 ± 2.0	162.9 ± 2.4	n.s.	178.1 ± 1.7	176.8 ± 2.4	n.s.
Weight	64.1 ± 3.5	71.8 ± 5.1	n.s.	79.9 ± 4.3	81.8 ± 2.4	n.s.
BMI	24.4 ± 1.4	26.9 ± 1.5	n.s.	25.2 ± 1.3	26.2 ± 0.5	n.s.
Pain intensity (0-10)	0 ± 0	5.6 ± 0.6	***P* < 0.001**	0.3 (0.3)	5.4 (0.8)	***P* < 0.001**
Pain duration (%)						
1–5 y	—	50	—	—	62.5	—
More than 5 y	—	50	—	—	37.5	—
Living condition (%)						
Alone	12.5	12.5	n.s.	62.5	62.5	n.s.
Not alone	87.5	87.5	n.s.	37.5	37.5	n.s.
Race (%)						
White	87.5	62.5	n.s.	100	62.5	n.s.
Asian or Pacific Islander	12.5	0	n.s.	0	12.5	n.s.
Black	0	37.5	n.s.	0	12.5	n.s.
Others	0	0	n.s.	0	12.5	n.s.
Education (%)						
Less than high school	0	0	n.s.	0	0	n.s.
High school	0	0	n.s.	0	0	n.s.
College, certificate	37.5	62.5	n.s.	25	25	n.s.
Bachelor's degree	25	12.5	n.s.	37.5	12.5	n.s.
Master's degree, certificate above bachelor	12.5	25	n.s.	12.5	50	n.s.
Higher than master's degree	25	0	n.s.	25	12.5	n.s.
ODI (/50)	0.1 ± 0.1	9.1 ± 1.7	***P* < 0.001**	0.4 ± 0.4	6.5 ± 1.5	***P* < 0.001**
Anxiety score (/21)	3.1 ± 0.6	4.9 ± 1.6	n.s.	2.3 ± 1.1	5.5 ± 0.8	***P* < 0.05**
Depression score (/21)	0.8 ± 0.4	3.8 ± 1.1	***P* < 0.05**	0.8 ± 0.5	3.5 ± 0.6	***P* < 0.01**
DN4 (/7)	0.0 ± 0.0	2.8 ± 0.8	***P* < 0.01**	0.0 ± 0.0	2.3 ± 0.8	***P* < 0.01**
EQ-5D-5L (/25)	5.0 ± 0.0	8.9 ± 0.7	***P* < 0.001**	5.1 ± 0.1	8.1 ± 0.5	***P* < 0.001**
SCL-90-R (/48)	1.3 ± 0.3	7.3 ± 1.6	***P* < 0.01**	1.1 ± 0.7	7.0 ± 1.7	***P* < 0.01**
PCS (/52)	5.4 ± 2.5	15.3 ± 3.7	***P* < 0.05**	6.1 ± 3.1	12.9 ± 4.0	n.s.

Parameters	Groups					
	Control women	LBP women	*P*	Control men	LBP men	*P*
(B) Validation cohort						
Number	13	19	—	13	18	—
Age	38.5 ± 3.5	46.1 ± 2.7	n.s.	43.1 ± 3.2	48.4 ± 2.6	n.s.
Height	165.5 ± 1.7	163.2 ± 1.7	n.s.	175.5 ± 2.2	175.8 ± 1.9	n.s.
Weight	64.6 ± 2.9	64.1 ± 2.6	n.s.	77.5 ± 3.4	84.2 ± 2.5	n.s.
BMI	23.6 ± 1.0	23.9 ± 0.9	n.s.	25.1 ± 0.9	27.3 ± 0.7	n.s.
Pain intensity (0-10)	0.1 ± 0.1	6.9 ± 0.5	***P* < 0001**	0 (0)	6.6 (0.4)	***P* < 0.0001**
Pain duration (%)						
1–5 y	—	47.4	—	—	44.4	—
More than 5 y	—	52.6	—	—	55.6	—
Living condition (%)						
Alone	23.1	15.8	n.s.	53.8	27.8	n.s.
Not alone	76.9	84.2	n.s.	46.2	72.2	n.s.
Race (%)						
White	46.1	68.4	n.s.	69.2	44.45	n.s.
Asian or Pacific Islander	15.4	5.3	n.s.	15.4	0	n.s.
Black	15.4	15.8	n.s.	0	11.1	n.s.
Others	23.1	10.5	n.s.	15.4	44.45	n.s.
Education (%)						
Less than high school	0	0	n.s.	0	0	n.s.
High school	0	10.5	n.s.	7.7	22.2	n.s.
College, certificate	15.4	42.1	n.s.	30.8	27.8	n.s.
Bachelor's degree	15.4	15.8	n.s.	38.4	22.2	n.s.
Master's degree certificate above bachelor	53.8	21.1	n.s.	7.7	27.8	n.s.
Higher than master's degree	15.4	10.5	n.s.	15.4	0	n.s.
ODI (/50)	0.5 ± 0.3	14.2 ± 1.2	***P* < 0.001**	0.1 ± 0.1	11.1 ± 1.3	***P* < 0.0001**
Anxiety score (/21)	2.5 ± 0.7	8.9 ± 1.0	***P* < 0.001**	3.1 ± 0.7	7.3 ± 0.9	***P* < 0.01**
Depression score (/21)	1.6 ± 0.8	6.6 ± 0.9	***P* < 0.0001**	1.5 ± 0.6	5.9 ± 1.0	***P* < 0.0001**
DN4 (/7)	0.0 ± 0.0	3.2 ± 0.5	***P* < 0.0001**	0.0 ± 0.0	2.3 ± 0.4	***P* < 0.0001**
EQ-5D-5L (/25)	5.3 ± 0.2	10.6 ± 0.8	***P* < 0.0001**	5.2 ± 0.1	9.6 ± 0.5	***P* < 0.0001**
SCL-90-R (/48)	1.5 ± 0.4	13.5 ± 1.9	***P* < 0.0001**	0.3 ± 0.2	9.9 ± 1.6	***P* < 0.0001**
PCS (/52)	7.8 ± 2.2	25.8 ± 3.2	***P* < 0.01**	3.1 ± 1.4	19.2 ± 2.8	***P* < 0.0001**

Categorical variables were analyzed using the Fisher exact test. Continuous variables are expressed as mean ± SEM, and an unpaired *t* test or the Mann–Whitney *U* test was used to compare control and LBP participants.

BMI, body mass index; DN4, Douleur Neuropathique 4; EQ, EuroQol; LBP, low back pain; ODI, Oswestry Disability Index; PCS, Pain Catastrophizing Scale; SCL, symptoms checklist.

### 2.2. Self-reported measures

Questionnaires included the Canadian adaptation of the NIH Low Back Pain Taskforce minimum recommended data set and standardized, validated questionnaires measuring neuropathic pain (*DN4*), global health (*EQ-5D-5L*), disability (*ODI2.1a*), somatization (*SCL90R, somatization subscale)*, mood (*HADS*), pain catastrophizing (*PCS*), and a Self-Administered Comorbidity Questionnaire.^37^ Participant characteristics (Table 1) are expressed as mean ± SEM or as percentage (%). As pain intensity, disability, depressive symptoms, global health, and somatization do not follow normal distribution (*P* < 0.05 in the Kolmogorov–Smirnov test), the Mann–Whitney *U* test was used. For the other variables with a normal distribution, unpaired *t tests* were used to compare control and LBP participants using GraphPad Prism and changes were considered significant if *P* < 0.05. Categorical variables were analyzed using the Fisher exact test.

### 2.3. T-cell isolation, DNA extraction, and bisulfite conversion

CD3^+^ T cells were isolated from 8 mL blood collected in EDTA (Ethylenediaminetetraacetic acid) tubes, and leukocytes were freshly isolated using Ficoll gradient separation. T cells were positively isolated using Dynabeads CD3 isolation kit (Invitrogen), and pellets were immediately frozen at −80°C. DNA was isolated using AllPrep DNA/RNA Mini Kit (Qiagen, Canada). Bisulfite conversion was performed (EZ-DNA methylation Gold Kit; Zymo Research, Irvine, CA), and DNA methylation profiles were generated by Genome Quebec according to standard protocols using Illumina 850 K bead arrays (Infinium MethylationEPIC Kit).

### 2.4. DNA methylation data processing

**Figure 1.**
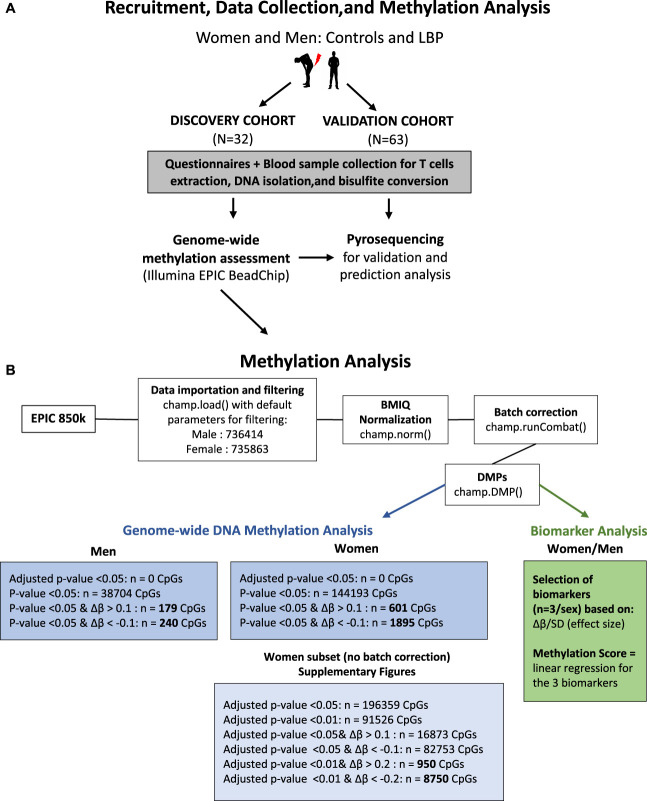
Overview of the study design including (A) the recruitment, data collection, and analysis, as well as (B) the flowchart for the genome-wide methylation analysis study*. *LBP, low back pain; ∆β, absolute difference in methylation value between control and LBP groups.

Illumina arrays were analyzed using the ChAMP Bioconductor package in R.^47^ Figure 1 illustrates the different steps of the analysis. Samples were randomized on arrays to mitigate batch effects. In brief, IDAT files were imported and filtered with champ.load() with default settings. Men and women were analyzed separately, and remaining probes were normalized with champ.norm() using the BMIQ (Beta MIxture Quantile dilation) method. Batch effects from slides were corrected with champ.runCombat(). Differentially methylated positions (DMPs) were obtained using champ.DMP() (Benjamini–Hochberg FDR correction). Differential methylation was calculated as the ratio (β-value) of intensities between methylated cytosines at each CpG site and total locus signal intensity.

The data have been deposited in NCBI's Gene Expression Omnibus (GEO)^19^ and are accessible through GEO Series accession number GSE 162350.

### 2.5. Identification of a polygenic methylation score

Using the discovery cohort, a polygenic methylation score was developed in women and men as a proof-of-concept potential biomarker for LBP. Polygenic scores are used in cases when one single CpG site cannot achieve the desired significance.^26^ A Cohen score was calculated for each probe to shortlist probes with the highest effect size (Cohen D > 2) then shortlisted by the percentage of differential methylation. Top probes were subjected to penalized regression and a set of 3 probes was identified. For each individual, the combined weight of these 3 probes based on the regression coefficient was used to calculate a polygenic methylation score as a single variable that measures a sample's membership in either the control or the LBP group (0 for control and 1 for LBP).

Female Polygenic Methylation Score = ((β1x (−1.77) + β2 × 2.46 + β3 × (−1.36)) −1.32 (β1 = methylation level of cg26114124, β2 = cg07420274, β3 = cg20331269).

Male Polygenic Methylation Score = ((β1x (−1.98) + β2 × 0.23 + β3 × (−2.92)) +2.89 (β1 = methylation level of cg21149944, β2 = cg22831726, β3 = cg25393494).

Two-way analysis of variance was used to compare methylation scores (women vs men, control vs LBP) followed by a Sidak test for multiple comparisons.

### 2.6. Validation using pyrosequencing and an independent cohort

T cells were collected and processed as described above from participants in the validation cohort. Pyrosequencing of selected genes was performed on all participants (discovery and validation cohorts); primers are listed in Supplementary Table 1 (available at http://links.lww.com/PR9/A131). PCR amplification and pyrosequencing were performed using standard methods.^12^ In brief, biotinylated PCR products were incubated with Streptavidin Sepharose beads (GE Healthcare, Mississauga, Canada), followed by denaturation. Pyrosequencing was performed using PyroMark Q24 and analyzed with PyroMark Q24 Software (Qiagen, Toronto, ON, Canada). Data were expressed as mean ± SEM and analyzed by the Student t test. Owing to poor sample quality or quantity, pyrosequencing data for 4 women and 4 men are missing.

Statistical analyses were performed using logistic regression at CpG sites validated with pyrosequencing to evaluate the relationship between CpG methylation value (independent variable) and chronic pain status (dependent variable). There was no age effect, and adding age as a covariate did not change the results.

### 2.7. Genome-wide identification of differentially methylated positions

Genome-wide methylation analysis was performed with the Montreal Clinical Research Institute for quality control, bioinformatics, and statistical analysis. As no significant DMPs were found using adjusted *P*-value <0.05, DMPs were defined with a nominal *P*-value <0.05 and a (∆β) ≥ 10% between the 2 groups. After log2 transformation, β values were then mean and centered for heatmap representation with hierarchical clustering applied on probes and samples using the complete linkage method. A subset analysis was performed on 2 distinct subclusters of LBP and control women (n = 4 each). For this analysis, DMPs were defined with adjusted *P*-value<0.01 and a (∆β) ≥ 20%.

Differences in the genomic distribution of the DMPs in both women and men and between hypomethylated and hypermethylated DMPs were assessed using a Fisher exact test on DMP feature (eg, TSS200, TSS1500, …) as extracted from the Infinium MethylationEPIC v1.0 B4 Manifest.

To assess the overlap between DMPs in our study and publicly available data, Venn diagrams were constructed.^7^ To compare results between human, mouse, and rat, BioMart database was used to identify human gene orthologs. A hypergeometric test was used to test for enrichment of LBP-related genes identified here with the public databases.

### 2.8. Functional enrichment analysis

Gene Ontology (GO) and pathway enrichment analysis were performed on DMPs using g:Profiler with g:SCS multiple testing correction method.^41^ Enriched GO terms were categorized into molecular function, biological processes, and cellular components. Enriched GO terms and KEGG pathways with an adjusted *P*-value < 0.05 are represented as enriched scores expressed as −log10 (adjusted *P*-value). Enriched KEGG maps were drawn with Pathview Web.

## 3. Results

### 3.1. Differential methylation analysis in the discovery cohort

**Figure 2.**
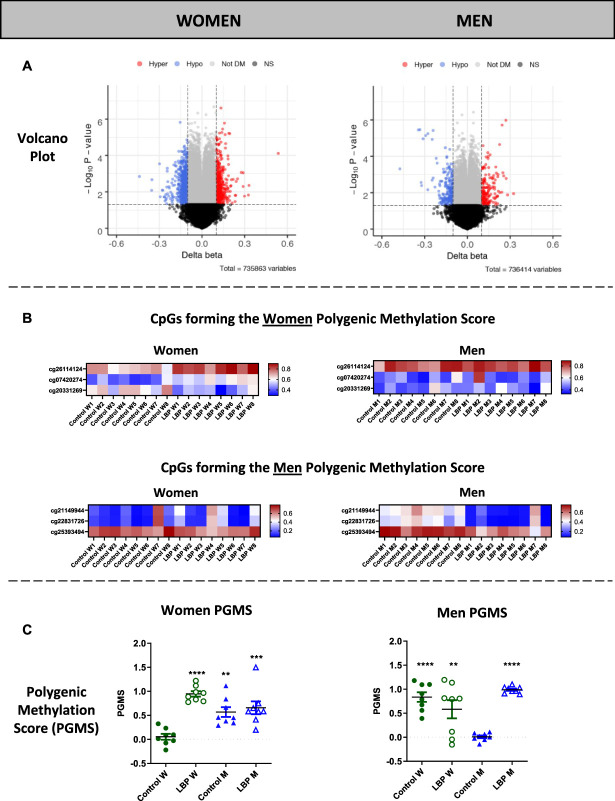
(A) Volcano plots of the differentially methylated positions with x and y axes displaying, respectively, the delta beta values (effect size) and the log10 of the *P* values for each CpGs site. Hypermethylated and hypomethylated CpGs in LBP vs controls (delta beta > 10% and *P* < 0.05) are represented in red and blue, respectively. (B) Heatmap representation of the methylation profile of the selected CpGs in both women and men used to construct the (C) polygenic methylation score. Two-way analysis of variance (ANOVA) followed by Sidak multiple comparisons, ** = *P* < 0.01, *** = *P* < 0.001, **** = *P* < 0.0001. *LBP, low back pain; Not DM = not differentially methylated, NS, no significant; W, women; M, men.

Of the 736,414 CpGs identified in men, 179 were hypermethylated and 240 were hypomethylated. Of the 735,863 CpGs identified in women, 601 were hypermethylated and 1895 were hypomethylated (*P*-value <0.05 and a (∆β) ≥10%, Figure 2A and Supplementary Table 2, available at http://links.lww.com/PR9/A131). In the subset of women analyzed separately, 950 hypermethylated and 8750 hypomethylated CpGs were found (adjusted *P*-value <0.01 and a (∆β) ≥20%, Supplementary Figure 1A and Supplementary Table 3, available at http://links.lww.com/PR9/A131). Using the discovery cohort, we calculated a polygenic methylation score from circulating T cells that requires only 3 CpGs sites (Fig. 2B) to predict the pain status of each individual (0 = no pain and 1 = pain, Fig. 2C). The individual CpGs and the composite methylation scores were sex specific. In men, 2 of the CpGs in the composite score correspond to the same gene, ZNF718.

### 3.2. Confirmation of differential methylation using pyrosequencing and the validation cohort

**Figure 3.**
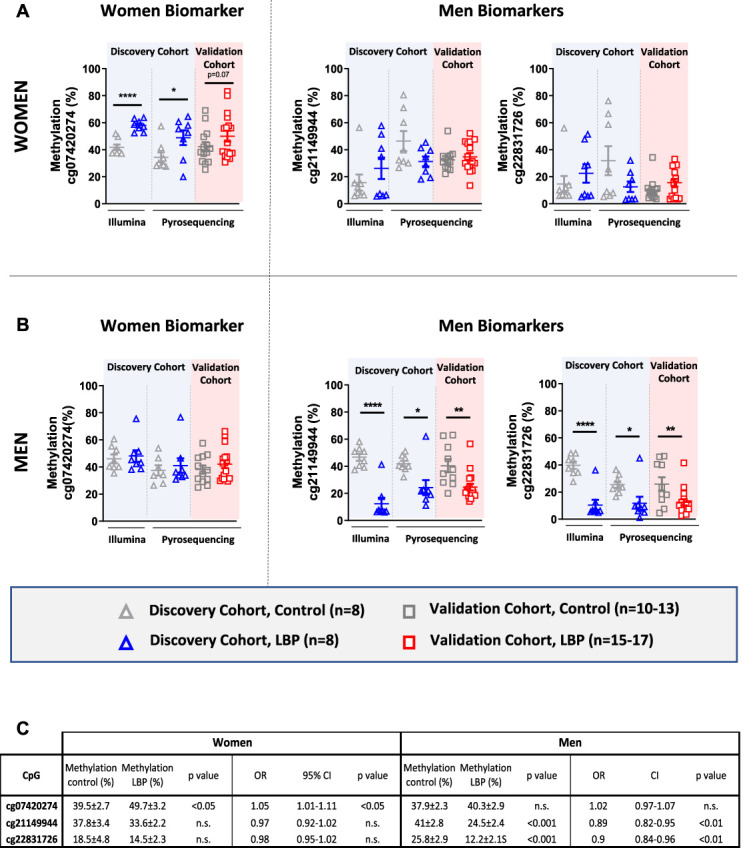
Percentage of methylation using Illumina (850K Epic Array) or targeted pyrosequencing of the 3 CpGs selected for validation in the discovery and validation cohorts in (A) women and (B) men. Association of the 3 CpGs methylation with the risk of having chronic low back pain using a logistic regression (C). Unpaired two-tailed (discovery cohort) or one-tailed (validation cohort) *t* test, * = *P* < 0.05, ** = *P* < 0.01, *** = *P* < 0.001.

To validate results from the array-based analysis, probes covering 3 DMPs from the polygenic methylation score's analysis (cg07420274 for women and cg21149944 and cg22831726 for men) were selected for measurement of DNA methylation at single-nucleotide resolution using pyrosequencing (Fig. 3). In the discovery cohort*,* the differences between LBP and controls obtained with Illumina were confirmed with pyrosequencing in both women and men (Fig. 3A, B, respectively). In the independent validation cohort*,* both DMPs of interest in men were validated by pyrosequencing. Although differential methylation of cg07420274 in women was not statistically significant by pyrosequencing (*P* = 0.07), the direction of change was consistent with the array-based analysis. Results from the array-based analysis correlated with pyrosequencing data (r = 0.44, *P* < 0.05, Supplementary Figure 2, available at http://links.lww.com/PR9/A131).

### 3.3. Association between methylation status and probability of having chronic low back pain

In women, the percentage of methylation at position cg07420274 was 39.5 ± 2.7% and 49.7 ± 3.2% in control (n = 21) and LBP groups (n = 25), respectively (*P* < 0.05, Fig. 3C). A statistically significant association between methylation at cg07420274 and LBP was observed (OR = 1.05, 95% CI: 1.01–1.11, *P* = 0.03).

In men, a statistically significant association was found between LBP and cg21149944 methylation (OR = 0.89, 95% CI: 0.82–0.95, *P* = 0.0015) as well as cg22831726 methylation (OR = 0.9, 95% CI: 0.84–0.96, *P* = 0.0036, Fig. 3C).

### 3.4. Distribution analysis of differentially methylated positions

Although DNA methylation within the transcription start site (TSS) is often predicted to silence the respective gene, the impact of methylation in other genomic regions is less clear. The distribution of hypermethylated and hypomethylated DMPs was therefore investigated per genetic feature.

**Figure 4.**
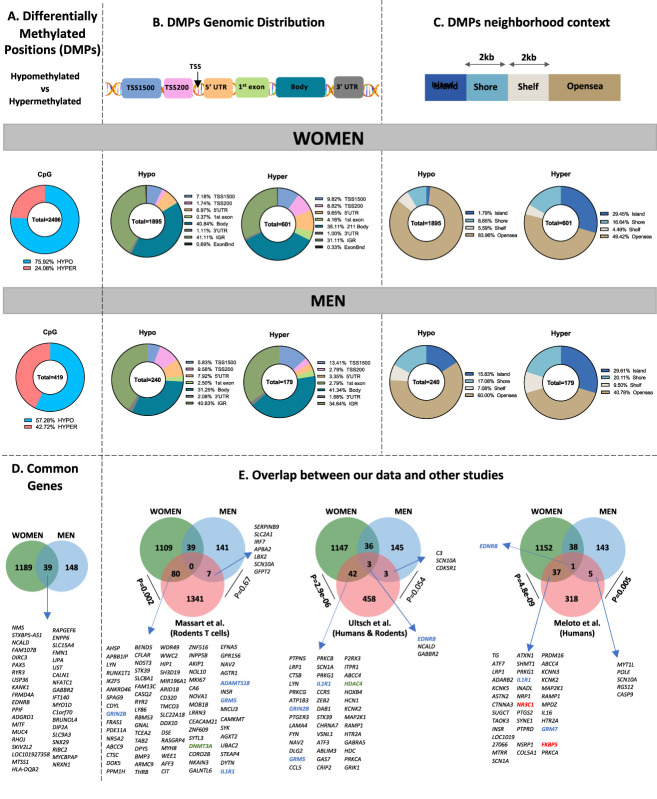
Characteristics of the differentially methylated positions (DMPs) between healthy controls and LBP participants*. Representation of (A) the hypermethylated and hypomethylated DMPs, (B) the genomic distribution, and (C) the neighborhood context of the DMPs detected in LBP participants compared with controls. Venn diagrams representing (D) the common differentially methylated genes between women and men in our study and (E) the overlap between our data and other studies on rat T cells (Massart et al), human pain genes (Meloto et al.), and rodent pain-related genes (Ultsh et al). For overlaps, pain, stress, and epigenetic-related genes mentioned in the text were highlighted in blue, red, and green, respectively. Significance of overlap between 2 groups was determined using the hypergeometric test. *LBP, low back pain.

Overall, 75.9% of DMPs in women and 57.3% in men were hypomethylated (Fig. 4A). Statistically significant differences between the proportions of hypermethylated vs hypomethylated DMPs were observed for the TSS1500 and TSS200, the 5′ untranslated region (5′UTR), the gene body, the intergenic region, and the first exon (women only) (Fig. 4B and Supplementary Table 4, available at http://links.lww.com/PR9/A131). The same profile was observed in the subset analysis of women (Supplementary Figure 1B, available at http://links.lww.com/PR9/A131). Interestingly, although most DMPs in women were hypomethylated, only a small percentage (1.79%) were in CpG islands, compared with 29% of all hypermethylated CpGs in the islands (Fig. 4C).

### 3.5. Overlaps between women and men, humans and rodents, and previously identified pain-relevant genes

Women and men had 39 differentially methylated genes in common (Fig. 4D). Genes that were differentially methylated in both this study and our previous epigenome-wide rodent study are shown in Figure 4E, with a significant enrichment between rat T cells after nerve injury^30^ and female human LBP genes (80 genes, *P* = 0.002).

We compared our current findings with pain literature (The Pain Genes Database: Ultsch et al.; http://www.jbldesign.com/jmogil/enter.html and^27,48^ The Human Pain Genetics Database; http://links.lww.com/PAIN/A520); common^32^ genes are shown in Figure 4E. Significant enrichment between women in our study and the Pain Genes Database were observed (42 genes, *P* = 9.9e-06). Significant enrichment was also found for both women and men in our study and the Human Pain Genetics Database (37 genes, *P* = 4.8e-09 and 5 genes, *P* = 0.005, respectively).

### 3.6. Broad signature of DNA methylation in chronic low back pain

**Figure 5.**
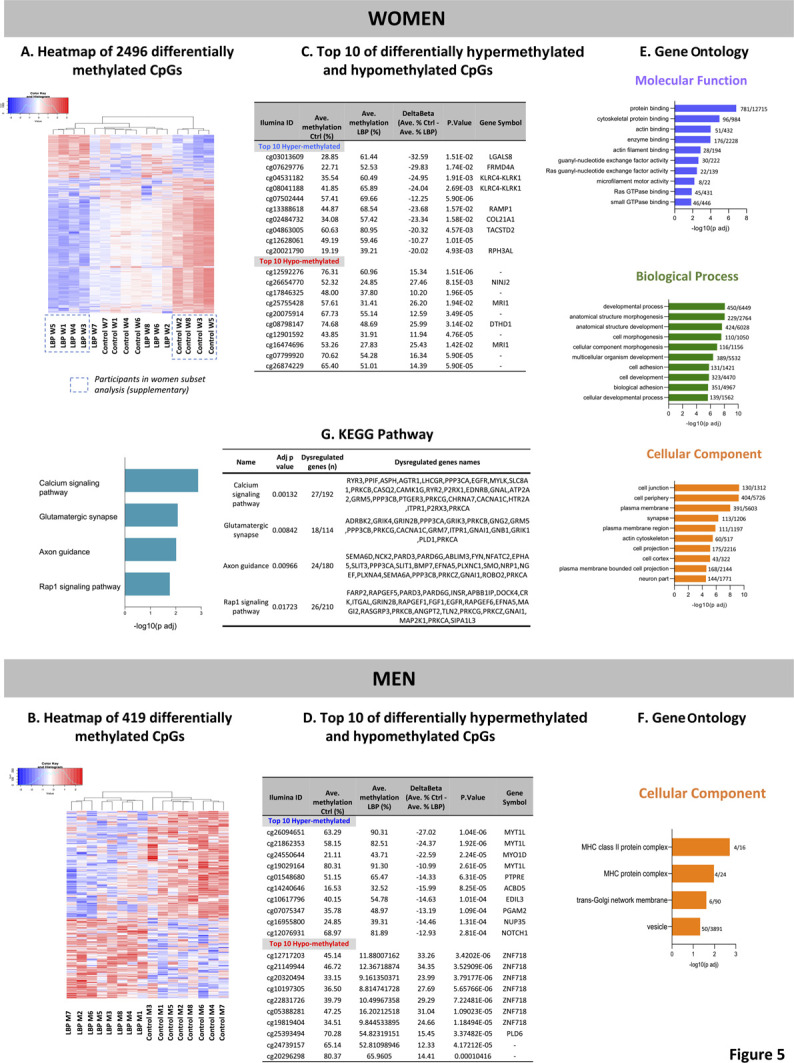
Heatmaps showing methylation signatures of (A) 2496 CpGs sites in women and (B) 419 in men. B values (after log2 transformation) are depicted using a red (hypermethylated in LBP) to blue (hypomethylated in LBP) methylation gradient. The top 10 differentially hypermethylated and hypomethylated CpGs are listed in both women (C) and men (D). Finally, the top 10 enriched Gene Ontology terms and KEGG pathways are shown in women (E and G) and men (F). Data are presented as enriched scores expressed as −log10 (adjusted *P*-value) with adj *P*-value <0.05. LBP, low back pain.

Methylation analysis in women revealed 2496 DMPs between controls and chronic LBP participants with partial separation between groups (Fig. 5A). In males, clustering analysis for the 419 DMPs between controls and LBP participants generated complete separation between groups (Fig. 5B). The top 10 hypermethylated and hypomethylated DMPs for both women and men are listed (Fig. 5C, D, respectively) as well as for the subset group of women (Supplementary Figure 1D, available at http://links.lww.com/PR9/A131). In men, 7 of the 10 most hypomethylated DMPs correspond to the gene ZNF718.

Analysis of the clusters within the female LBP participants (Supplementary Figure 1C, available at http://links.lww.com/PR9/A131) revealed differences in clinical presentation (Supplementary Figure 3, available at http://links.lww.com/PR9/A131). Specifically, although pain intensity was similar in all LBP women (*P* = 0.85), those belonging to the highly clustered group showed significant increases in disability (ODI, *P* = 0.004) and depression (HADS, *P* = 0.03) compared with the other LBP participants. This suggests it may be possible to link methylation signatures to clinical subgroups of LBP patients.

### 3.7. Gene Ontology enrichment analysis

To gain functional insights, the list of gene-annotated DMPs was subjected to Gene Ontology (GO) enrichment analysis (Supplementary Table 5, available at http://links.lww.com/PR9/A131).

In women, the top 10 GO terms categorized into molecular function, biological processes, and cellular component are illustrated in Figure 5E. Cell junction (GO:0030054; adj-p = 4.9 × 10^−10^), cell periphery (GO:0071944: adj-p = 5.6 × 10^−10^), and development process (GO:0032502: adj-p = 8.1 × 10^−9^) had the highest enrichment scores. Different methylation of known epigenetic regulators including DNA methyltransferases (DNMT3a) and histone deacetylase (HDAC4 and HDAC11) were identified in this analysis. Enrichment analysis for the subset group of women is illustrated in Supplementary Figure 4A, available at http://links.lww.com/PR9/A131.

In men, analysis revealed only 4 enrichment GO terms (Fig. 5F), all belonging to the cellular component, the first 2 were MHC class II protein complex and MHC protein complex, representing the major histocompatibility complex that plays a central role in the immune system.

For both woman and men, GO enrichment analysis was also performed on differentially hypermethylated and hypomethylated CpGs separately (Supplementary Figures 5 and Supplementary Table 5, available at http://links.lww.com/PR9/A131).

### 3.8. Pathway enrichment analysis

To generate further insight into functional pathways, we also performed KEGG pathway annotation. In women, 4 enriched pathways were found (Fig. 5G): calcium signaling, glutamatergic synapse, axon guidance, and Rap1 signaling pathway (Supplementary Figure 6, available at http://links.lww.com/PR9/A131). When pathway analysis was performed for hypermethylated or hypomethylated DMPs separately, the hypomethylated CpGs generated similar results to the grouped analysis (Supplementary Figure 5C, available at http://links.lww.com/PR9/A131). Similar pathways were also observed in women subset analysis (Supplementary Figure 4B, available at http://links.lww.com/PR9/A131). No significant pathway enrichment was found in men.

## 4. Discussion

Epigenetic programming provides a mechanism by which previous life experience influences our response to new challenges. These challenges (eg, disease onset and physical or psychological stress), in turn, embed maladaptive states by reprogramming the epigenome. In contrast to genetics, epigenetics is dynamically regulated and can be modulated therapeutically.^24,36^

We examined the association between LBP and epigenome-wide DNA methylation in T cells isolated from patients and pain-free controls. Key observations include divergent epigenetic signatures of LBP in men and women, proof-of-concept evidence for epigenetic pain biomarkers in lymphocytes and identification of individual genes and gene families with a possible role in LBP pathology. Understanding the role of epigenetics in health and disease in general and chronic pain in particular may generate insights into new therapeutic targets, provide diagnostic markers for risk and recovery, and deliver insights into interindividual differences to support personalized medical approaches.

Chronic LBP is a complex and multifactorial disease to which both genetic and environmental factors contribute.^21^ Many environmental factors, such as early trauma, low socioeconomic status, or depression, are mediated, in part, by long-term epigenetic reprogramming.^9,11,35^ Using reduced representation bisulfite sequencing, Aroke et al. demonstrated differential methylation in the whole blood between chronic LBP participants and pain-free controls.^4^ This study adds to other reports linking DNA methylation in human peripheral blood to chronic pain, including postsurgical pain,^13,14^ fibromyalgia,^15^ chronic widespread musculoskeletal pain,^29^ and neuropathic pain.^46^

We identified a polygenic DNA methylation score from circulating T cells that requires only 3 DMPs to measure the pain status of each individual; several of these were validated in an independent cohort using pyrosequencing. The DMPs and scores were sex specific. This is consistent with the growing literature, suggesting sex-specific effects in pain-induced epigenetic changes,^23^ in the immune component of pain,^42^ and in stress responses.^6^ Thus, although LBP affects both sexes,^33^ our data revealed striking sex differences in DNA methylation signatures, suggesting fundamentally different underlying mechanisms and the possibility of sex-specific epigenetic biomarkers and therapeutic approaches.

In women with LBP, we identified 2496 CpGs that met our criteria for differential methylation, more than 3 quarters being hypomethylated. This persisted in promoter regions (TSS200 and 1500) where hypomethylated CpGs represented 60% of the total changes. This is consistent with findings in women with interstitial cystitis or bladder pain syndrome, where DNA methylation analysis of pelleted urine sediment showed decreased methylation in multiple CpGs in the MAPK pathway.^10^ In utopic endometrial tissue samples from patients with severe endometriosis, interrogated CpG sites were also hypomethylated.^25^

In women, Gene Ontology enrichment analysis revealed DMPs associated with processes at the cellular and molecular levels including those involved in cell junction, cell periphery, and development. Some of the genes in the enriched ontologies include pain genes *(EGFR, GRIN2B, GRM5,* and *GAD1)*, genes involved in the extracellular matrix (ECM) such as proteases *(*eg, *MMP15, ADAMTS18,* and *ADAMTS16)*, collagen *(COL12A1)*, glycoproteins *(LAMA1)*, integrins *(ITGB6)*, and growth factors *(*eg, *BMP3, BMP7,* and *TGFBR2)*, interleukins and their receptors (eg, *IL1R1)*, and genes associated with epigenetic processes *(*eg, *HDAC4, HDAC11,* and *DNMT3a)*. Although LBP is multifactorial, intervertebral disc degeneration is a potential underlying cause and is associated with altered ECM turnover and inflammation.^5^ A recent study suggesting a role for ECM reorganization in the nervous system in chronic pain.^38^ Furthermore, the changes observed in the current study may be secondary to LBP but rather reflect the unrelenting stress of living with chronic pain. This is supported by the overlap with *The Human Pain Genetics Database* at well-known stress-related genes including the glucocorticoid receptor (NR3C1) and the FK506 binding protein 5 (FKBP5).^20^ Interestingly, the endothelin receptor type B (EDNRB), which was previously linked to pain,^32,34,48^ was differentially methylated in both women and men with LBP, making EDNRB a potential candidate for further investigation.

In men with LBP, 419 CpGs were found to be differentially methylated, which is 5 times fewer than in women. As in women, DMPs tended towards hypomethylation compared with hypermethylation (57% vs 43%).

In men, we identified a unique LBP DNA methylation signature characterized by significant enrichment for genes from the major histocompatibility complex (MHC) class II, including HLA-DQA1 and HLA-DQB2. Consistent with this, a recent study on the transition from acute to chronic LBP showed significant upregulation of mRNA in blood for antigen presentation pathways (MHC class I and II).^18^ MHC class II gene upregulation has been associated with other chronic pain conditions including pancreatitis, postherpetic neuralgia, inguinal hernia repair, complex regional pain syndrome, lumbar disc herniation, rheumatoid arthritis, chronic inflammatory response syndrome, temporomandibular disorders, and LBP. Furthermore, preclinical studies have shown a role for MHC complex in a postherpetic pain model in mice^43^ and in neuropathic pain in rats,^16^ where a contrasting effect was found in female rats.^17^

Of the top 10 hypomethylated CpGs in men, 7 mapped to the gene ZNF718. Little is known about this protein; zinc finger proteins are involved in transcriptional regulation and it interacts with pain mediators TNF and NGF.^49^ ZNF718 has been associated with DNA methylation for sex hormone-binding globulin and bioavailable testosterone in males in childhood.^2^ Another study found differential methylation of ZNF718 in whole blood of severe asthmatics compared with healthy controls.^50^ Further studies are needed to elucidate the role of ZNF718 in LBP in men.

A major limitation of this study is the small sample size for the discovery cohort*,* although this is partially mitigated by the inclusion of an independent validation cohort*.* Despite this limitation, several interesting observations are reported that will guide future, larger scale studies. Increased sample size will allow us to examine the generalisability of these findings and the interaction of epigenetic reprogramming with risk factors for LBP (eg, early life adversity) and will enable deeper exploration of phenotypic traits (eg, disability severity, anxiety, and depression). Epigenetics may also be important in racial disparities in chronic pain.^3^

Epigenetic programming is cell type specific, and this study was performed in peripheral T cells. We identified genes with clear function in immune cells (eg, MHC genes and cytokines) that might contribute to chronic pain pathophysiology. Nevertheless, several differentially methylated genes have roles in the central nervous system. It is unclear whether differential methylation in these genes reflects concurrent changes in the central nervous system in humans, but we previously observed an overlap between differentially methylated profiles in T cells and prefrontal cortex in rats with peripheral nerve injury. An important question to be addressed in prospective studies is whether these differentially methylated sites precede or follow the appearance of chronic pain and whether they play a causal role or are a consequence of chronic pain. A related question is whether the epigenetic differences reflect earlier experiences that confer susceptibility and could therefore serve as predictors of risk to developing chronic pain.

In conclusion, chronic LBP is associated with sex-specific epigenome-wide DNA methylation signatures in circulating T cells. We provide proof-of-principle data indicating that the methylation level of a small number of CpGs sites can categorise the pain status (control or LBP) of each participant and that this polygenic methylation score is sex specific. Our study provides preliminary evidence and justification for larger studies to establish associations between chronic LBP, risk factors (eg, previous trauma, smoking), comorbidities (eg, anxiety, depression, and impaired sleep), and DNA methylation. Establishing a causal link between DNA methylation and LBP will open up new windows for the use of therapeutics involving epigenetic reprogramming and development of biomarkers for predicting risk of chronic pain.

## Disclosures

Authors S. Gregoire, D. Cheishvili, M. Szyf, and L.S. Stone are coinventors on a provisional patent protecting the methylation signatures described here for possible use as a biomarker for chronic pain. D. Cheishvilli and M. Szyf are associated with HKG Epitherapeutics that develops epigenetic biomarkers for clinical use. The remaining authors have no conflicts of interest to declare.

## Appendix A. Supplemental digital content

Supplemental digital content associated with this article can be found online at http://links.lww.com/PR9/A131.

## Supplementary Material

SUPPLEMENTARY MATERIAL

## References

[1] Alvarado S Tajerian M Suderman M Machnes Z Pierfelice S Millecamps M Stone LS Szyf M. An epigenetic hypothesis for the genomic memory of pain. Front Cell Neurosci 2015;9:88. 25852480 10.3389/fncel.2015.00088 PMC4371710

[2] Arathimos R Sharp GC Granell R Tilling K Relton CL. Associations of sex hormone-binding globulin and testosterone with genome-wide DNA methylation. BMC Genet 2018;19:113. 30547757 10.1186/s12863-018-0703-y PMC6295101

[3] Aroke EN Jackson P Overstreet DS Penn TM Rumble DD Kehrer CV Michl AN Hasan FN Sims AM Quinn T Long DL Goodin BR. Race, social status, and depressive symptoms: a moderated mediation analysis of chronic low back pain interference and severity. Clin J Pain 2020;36:658–66. 32487870 10.1097/AJP.0000000000000849 PMC7725357

[4] Aroke EN Overstreet DS Penn TM Crossman DK Jackson P Tollefsbol TO Quinn TL Yi N Goodin BR. Identification of DNA methylation associated enrichment pathways in adults with non-specific chronic low back pain. Mol Pain 2020;16:1744806920972889. 33169629 10.1177/1744806920972889 PMC7658508

[5] Balagué F Mannion AF Pellisé F Cedraschi C. Non-specific low back pain. Lancet 2012;379:482–91. 21982256 10.1016/S0140-6736(11)60610-7

[6] Bale TL Epperson CN. Sex differences and stress across the lifespan. Nat Neurosci 2015;18:1413–20. 26404716 10.1038/nn.4112 PMC4620712

[7] Bardou P Mariette J Escudie F Djemiel C Klopp C. jvenn: an interactive Venn diagram viewer. BMC Bioinformatics 2014;15:293. 25176396 10.1186/1471-2105-15-293 PMC4261873

[8] Bell JT Loomis AK Butcher LM Gao F Zhang B Hyde CL Sun J Wu H Ward K Harris J Scollen S Davies MN Schalkwyk LC Mill J Mu TC Williams FM Li N Deloukas P Beck S McMahon SB Wang J John SL Spector TD. Differential methylation of the TRPA1 promoter in pain sensitivity. Nat Commun 2014;5:2978. 24496475 10.1038/ncomms3978 PMC3926001

[9] Borghol N Suderman M McArdle W Racine A Hallett M Pembrey M Hertzman C Power C Szyf M. Associations with early-life socio-economic position in adult DNA methylation. Int J Epidemiol 2012;41:62–74. 22422449 10.1093/ije/dyr147 PMC3304522

[10] Bradley MS Burke EE Grenier C Amundsen CL Murphy SK Siddiqui NY. A genome-scale DNA methylation study in women with interstitial cystitis/bladder pain syndrome. Neurourol Urodyn 2018;37:1485–93. 29363787 10.1002/nau.23489 PMC5924587

[11] Cao-Lei L Massart R Suderman MJ Machnes Z Elgbeili G Laplante DP Szyf M King S. DNA methylation signatures triggered by prenatal maternal stress exposure to a natural disaster: project Ice Storm. PLoS One 2014;9:e107653. 25238154 10.1371/journal.pone.0107653 PMC4169571

[12] Cheishvili D Parashar S Mahmood N Arakelian A Kremer R Goltzman D Szyf M Rabbani SA. Identification of an epigenetic signature of osteoporosis in blood DNA of postmenopausal women. J Bone Miner Res 2018;33:1980–9. 29924424 10.1002/jbmr.3527

[13] Chidambaran V Zhang X Geisler K Stubbeman BL Chen X Weirauch MT Meller J Ji H. Enrichment of genomic pathways based on differential DNA methylation associated with chronic postsurgical pain and anxiety in children: a prospective, pilot study. J Pain 2019;20:771–85. 30639570 10.1016/j.jpain.2018.12.008 PMC6616015

[14] Chidambaran V Zhang X Martin LJ Ding L Weirauch MT Geisler K Stubbeman BL Sadhasivam S Ji H. DNA methylation at the mu-1 opioid receptor gene (OPRM1) promoter predicts preoperative, acute, and chronic postsurgical pain after spine fusion. Pharmgenomics Pers Med 2017;10:157–68. 28533693 10.2147/PGPM.S132691 PMC5432115

[15] Ciampi de Andrade D Maschietto M Galhardoni R Gouveia G Chile T Victorino Krepischi AC Dale CS Brunoni AR Parravano DC Cueva Moscoso AS Raicher I Kaziyama HHS Teixeira MJ Brentani HP. Epigenetics insights into chronic pain: DNA hypomethylation in fibromyalgia-a controlled pilot-study. PAIN 2017;158:1473–80. 28621701 10.1097/j.pain.0000000000000932

[16] Dominguez CA Lidman O Hao JX Diez M Tuncel J Olsson T Wiesenfeld-Hallin Z Piehl F Xu XJ. Genetic analysis of neuropathic pain-like behavior following peripheral nerve injury suggests a role of the major histocompatibility complex in development of allodynia. PAIN 2008;136:313–19. 17764842 10.1016/j.pain.2007.07.009

[17] Dominguez CA Lidman O Olsson T Wiesenfeld-Hallin Z Piehl F Xu XJ. Contrasting genetic effects of major histocompatibility complex on ischemic peripheral nerve and spinal cord injury in female rats. Neurosci Lett 2008;443:95–8. 18675884 10.1016/j.neulet.2008.07.063

[18] Dorsey SG Renn CL Griffioen M Lassiter CB Zhu S Huot-Creasy H McCracken C Mahurkar A Shetty AC Jackson-Cook CK Kim H Henderson WA Saligan L Gill J Colloca L Lyon DE Starkweather AR. Whole blood transcriptomic profiles can differentiate vulnerability to chronic low back pain. PLoS One 2019;14:e0216539. 31095601 10.1371/journal.pone.0216539 PMC6522025

[19] Edgar R Domrachev M Lash AE. Gene Expression Omnibus: NCBI gene expression and hybridization array data repository. Nucleic Acids Res 2002;30:207–10. 11752295 10.1093/nar/30.1.207 PMC99122

[20] Farrell C Doolin K NOL Jairaj C Roddy D Tozzi L Morris D Harkin A Frodl T Nemoda Z Szyf M Booij L O'Keane V. DNA methylation differences at the glucocorticoid receptor gene in depression are related to functional alterations in hypothalamic-pituitary-adrenal axis activity and to early life emotional abuse. Psychiatry Res 2018;265:341–8. 29793048 10.1016/j.psychres.2018.04.064

[21] Ferreira PH Beckenkamp P Maher CG Hopper JL Ferreira ML. Nature or nurture in low back pain? Results of a systematic review of studies based on twin samples. Eur J Pain 2013;17:957–71. 23335362 10.1002/j.1532-2149.2012.00277.x

[22] Global Burden of Disease Study C. Global, regional, and national incidence, prevalence, and years lived with disability for 301 acute and chronic diseases and injuries in 188 countries, 1990-2013: a systematic analysis for the Global Burden of Disease Study 2013. Lancet 2015;386:743–800. 26063472 10.1016/S0140-6736(15)60692-4 PMC4561509

[23] Gregoire S Jang SH Szyf M Stone LS. Prenatal maternal stress is associated with increased sensitivity to neuropathic pain and sex-specific changes in supraspinal mRNA expression of epigenetic- and stress-related genes in adulthood. Behav Brain Res 2020;380:112396. 31786273 10.1016/j.bbr.2019.112396

[24] Gregoire S Millecamps M Naso L Do Carmo S Cuello AC Szyf M Stone LS. Therapeutic benefits of the methyl donor S-adenosylmethionine on nerve injury-induced mechanical hypersensitivity and cognitive impairment in mice. PAIN 2017;158:802–10. 28030474 10.1097/j.pain.0000000000000811

[25] Houshdaran S Nezhat CR Vo KC Zelenko Z Irwin JC Giudice LC. Aberrant endometrial DNA methylome and associated gene expression in women with endometriosis. Biol Reprod 2016;95:93. 27535958 10.1095/biolreprod.116.140434 PMC5178151

[26] Huls A Czamara D. Methodological challenges in constructing DNA methylation risk scores. Epigenetics 2020;15:1–11. 31318318 10.1080/15592294.2019.1644879 PMC6961658

[27] Lacroix-Fralish ML Ledoux JB Mogil JS. The Pain Genes Database: an interactive web browser of pain-related transgenic knockout studies. PAIN 2007;131:3 e1–4. 17574758 10.1016/j.pain.2007.04.041

[28] Laumet G Ma J Robison AJ Kumari S Heijnen CJ Kavelaars A. T cells as an emerging target for chronic pain therapy. Front Mol Neurosci 2019;12:216. 31572125 10.3389/fnmol.2019.00216 PMC6749081

[29] Livshits G Malkin I Freidin MB Xia Y Gao F Wang J Spector TD MacGregor A Bell JT Williams FMK. Genome-wide methylation analysis of a large population sample shows neurological pathways involvement in chronic widespread musculoskeletal pain. PAIN 2017;158:1053–62. 28221285 10.1097/j.pain.0000000000000880 PMC5427989

[30] Massart R Dymov S Millecamps M Suderman M Gregoire S Koenigs K Alvarado S Tajerian M Stone LS Szyf M. Overlapping signatures of chronic pain in the DNA methylation landscape of prefrontal cortex and peripheral T cells. Sci Rep 2016;6:19615. 26817950 10.1038/srep19615 PMC4730199

[31] McCartney DL Hillary RF Stevenson AJ Ritchie SJ Walker RM Zhang Q Morris SW Bermingham ML Campbell A Murray AD Whalley HC Gale CR Porteous DJ Haley CS McRae AF Wray NR Visscher PM McIntosh AM Evans KL Deary IJ Marioni RE. Epigenetic prediction of complex traits and death. Genome Biol 2018;19:136. 30257690 10.1186/s13059-018-1514-1 PMC6158884

[32] Meloto CB Benavides R Lichtenwalter RN Wen X Tugarinov N Zorina-Lichtenwalter K Chabot-Dore AJ Piltonen MH Cattaneo S Verma V Klares R III Khoury S Parisien M Diatchenko L. Human pain genetics database: a resource dedicated to human pain genetics research. PAIN 2018;159:749–63. 29300278 10.1097/j.pain.0000000000001135

[33] Meucci RD Fassa AG Faria NM. Prevalence of chronic low back pain: systematic review. Rev Saude Publica 2015;49:1–10. 26487293 10.1590/S0034-8910.2015049005874 PMC4603263

[34] Millecamps M Laferriere A Ragavendran JV Stone LS Coderre TJ. Role of peripheral endothelin receptors in an animal model of complex regional pain syndrome type 1 (CRPS-I). PAIN 2010;151:174–83. 20675053 10.1016/j.pain.2010.07.003 PMC4474643

[35] Nemoda Z Massart R Suderman M Hallett M Li T Coote M Cody N Sun ZS Soares CN Turecki G Steiner M Szyf M. Maternal depression is associated with DNA methylation changes in cord blood T lymphocytes and adult hippocampi. Transl Psychiatry 2015;5:e545. 25849984 10.1038/tp.2015.32 PMC4462598

[36] Niederberger E Resch E Parnham MJ Geisslinger G. Drugging the pain epigenome. Nat Rev Neurol 2017;13:434–47. 28548108 10.1038/nrneurol.2017.68

[37] Page GM Lacasse A Quebec Back Pain C Beaudet N Choiniere M Deslauriers S Diatchenko L Dupuis L Gregoire S Hovey R Leclair E Leonard G Meloto CB Montagna F Parent A Rainville P Roy JS Roy M Ware MA Wideman TH Stone LS. The Quebec low back pain study: a protocol for an innovative 2-tier provincial cohort. Pain Rep 2020;5:e799. 32072095 10.1097/PR9.0000000000000799 PMC7004506

[38] Parisien M Samoshkin A Tansley SN Piltonen MH Martin LJ El-Hachem N Dagostino C Allegri M Mogil JS Khoutorsky A Diatchenko L. Genetic pathway analysis reveals a major role for extracellular matrix organization in inflammatory and neuropathic pain. PAIN 2019;160:932–44. 30763288 10.1097/j.pain.0000000000001471

[39] Polli A Godderis L Ghosh M Ickmans K Nijs J. Epigenetic and miRNA expression changes in people with pain: a systematic review. J Pain 2020;21:763–80. 31837447 10.1016/j.jpain.2019.12.002

[40] Provencal N Suderman MJ Guillemin C Massart R Ruggiero A Wang D Bennett AJ Pierre PJ Friedman DP Cote SM Hallett M Tremblay RE Suomi SJ Szyf M. The signature of maternal rearing in the methylome in rhesus macaque prefrontal cortex and T cells. J Neurosci 2012;32:15626–42. 23115197 10.1523/JNEUROSCI.1470-12.2012 PMC3490439

[41] Raudvere U Kolberg L Kuzmin I Arak T Adler P Peterson H Vilo Jg. Profiler: a web server for functional enrichment analysis and conversions of gene lists (2019 update). Nucleic Acids Res 2019;47:W191–8. 31066453 10.1093/nar/gkz369 PMC6602461

[42] Rosen SF Ham B Haichin M Walters IC Tohyama S Sotocinal SG Mogil JS. Increased pain sensitivity and decreased opioid analgesia in T-cell-deficient mice and implications for sex differences. PAIN 2019;160:358–66. 30335680 10.1097/j.pain.0000000000001420

[43] Sato-Takeda M Takasaki I Takeda K Sasaki A Andoh T Nojima H Shiraki K Kuraishi Y Hanaoka K Tokunaga K Yabe T. Major histocompatibility complex haplotype is associated with postherpetic pain in mice. Anesthesiology 2006;104:1063–9. 16645460 10.1097/00000542-200605000-00024

[44] Tajerian M Alvarado S Millecamps M Dashwood T Anderson KM Haglund L Ouellet J Szyf M Stone LS. DNA methylation of SPARC and chronic low back pain. Mol Pain 2011;7:65. 21867537 10.1186/1744-8069-7-65 PMC3182907

[45] Tajerian M Alvarado S Millecamps M Vachon P Crosby C Bushnell MC Szyf M Stone LS. Peripheral nerve injury is associated with chronic, reversible changes in global DNA methylation in the mouse prefrontal cortex. PLoS One 2013;8:e55259. 23383129 10.1371/journal.pone.0055259 PMC3557255

[46] Takenaka S Sukenaga N Ohmuraya M Matsuki Y Maeda L Takao Y Hirose M. Association between neuropathic pain characteristics and DNA methylation of transient receptor potential ankyrin 1 in human peripheral blood. Medicine (Baltimore) 2020;99:e19325. 32080151 10.1097/MD.0000000000019325 PMC7034692

[47] Tian Y Morris TJ Webster AP Yang Z Beck S Feber A Teschendorff AE. ChAMP: updated methylation analysis pipeline for Illumina BeadChips. Bioinformatics 2017;33:3982–4. 28961746 10.1093/bioinformatics/btx513 PMC5860089

[48] Ultsch A Kringel D Kalso E Mogil JS Lotsch J. A data science approach to candidate gene selection of pain regarded as a process of learning and neural plasticity. PAIN 2016;157:2747–57. 27548044 10.1097/j.pain.0000000000000694

[49] Vanderwall AG Milligan ED. Cytokines in pain: harnessing endogenous anti-inflammatory signaling for improved pain management. Front Immunol 2019;10:3009. 31921220 10.3389/fimmu.2019.03009 PMC6935995

[50] Wysocki K Conley Y Wenzel S. Epigenome variation in severe asthma. Biol Res Nurs 2015;17:263–9. 25288825 10.1177/1099800414553463 PMC4387102

