# Supplementary material for: Epigenetic signature of chronic low back pain in human T cells
Source: Pain Rep. 2021 Nov 3;6(4):e960. doi: 10.1097/PR9.0000000000000960 (PMC8568391; doi:10.1097/PR9.0000000000000960)
Supplement: SUPPLEMENTARY MATERIAL [file painreports-6-e960-s001.pdf]

Supplementary Figures:

Supplementary Figure 1:

Analysis comparing the highly clustered subset of healthy control women versus LBP women (n=4/group) * **(A)** Volcano plot of the differentially methylated positions in the subset of women with x and y axes displaying, respectively, the delta-beta values (effect size) and the log10 of P values for each CpGs site. CpGs more or less methylated in LBP and controls (delta-beta > 10% and P<0.05) are represented in red and blue, respectively. **(B)** Heatmap representation of the methylation profile of the differentially methylated CpGs between healthy controls and LBP participants. Representation of the **(C)** the genomic distribution and **(D)** the neighborhood context of the differentially methylated CpGs detected in LBP participants compared to controls.

* LBP = Low Back Pain, Not DM = Not Differentially Methylated, NS = No significant, W = Women

Supplementary Figure 2:

Correlation between average methylation values for cg21149944, cg22831726 and cg07420274 obtained with Illumina 850K array vs. pyrosequencing analysis in the *Discovery Cohort*.

Supplementary Figure 3:

Analysis of the clinical phenotype of the subset of highly clustered female LBP participants (LBP subset) revealed differences from the other female LBP participants.

* LBP = Low Back Pain, ODI = Oswestry Disability Index, HADS = Hospital anxiety and depression scale, PCS = Pain catastrophizing scale, EQ = EuroQol, SCL = Symptoms

Checklist. Data are expressed as mean ± standard error to the mean (SEM) and an unpaired *t*-test was used to compare groups. Unpaired two-tailed (Discovery cohort) or one-tailed (Validation cohort) *t* test, * = $p<0.05$, ** = $p<0.01$.

Supplementary Figure 4:

(A) Representation of gene ontology terms (top 10) and **(B)** KEGG pathways for differentially methylated positions (DMPs) in the subset of highly clustered female LBP participants compared to other 4 LBP participants.

Supplementary Figure 5:

(A&B) Representation of gene ontology terms (top 10) and **(C&D)** KEGG pathways in women and men for hypo and hyper differentially methylated positions (DMPs) respectively. Not there were no significant ontologies for males in the hyper-methylated dataset nor in the KEGG pathways.

Supplementary Figure 6:

Graphic representation (Pathview) of enriched pathways found in women with LBP compared to healthy controls.

Supplementary Tables:

Supplementary Table 1:

Pyrosequencing primers.

Supplementary Table 2: List of all differentially methylated CpGs sites (hypo- and hyper-methylated) between Healthy controls and Low Back Pain participants in both women and men ($p<0.05$ and ($\Delta\beta$) ≥ 10%).

Supplementary Table 3: List of all differentially methylated CpGs sites (hypo- and hyper-methylated) between the subset of highly clustered female LBP participants compared to other 4 LBP participants ($p<0.001$ and ($\Delta\beta$) ≥ 20%).

Supplementary Table 4:

Genomic characteristics of the differentially hypo- and hyper-methylated positions between healthy controls and LBP participants in women and men.

Supplementary Table 5:

Summary of GO-enrichment and KEGG pathways analysis of differentially hypo- and hyper-methylated CpGs, analyzed together or separately in both women and men.

Supplementary Table 6:

Summary of GO-enrichment and KEGG pathways analysis of differentially hypo- and hyper-methylated CpGs, analyzed together or separately in the subset of highly clustered female LBP participants compared to other 4 LBP participants.

A

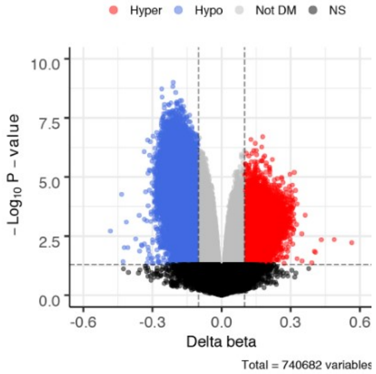

B

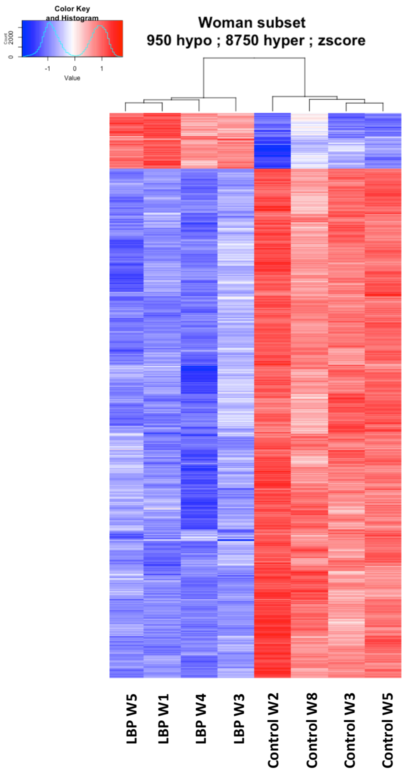

C

	Women Subset				
CpGs characteristics	Hypo-methylated CpGs sites		Hyper-methylated CpGs sites		P
	N	%	N	%	
**Methylation status**	8750	90.21	950	9.79	
**Genomic Distribution**					
TSS1500	593	6.78	93	9.79	**0.0011**
TSS200	124	1.42	71	7.47	**<0.0001**
5'UTR	650	7.43	122	12.84	**<0.0001**
1st exon	36	0.41	32	3.37	**<0.0001**
Body	3556	40.64	426	44.84	**0.0137**
3'UTR	148	1.69	18	1.89	0.5992
ExonBnd	56	0.64	3	0.32	0.2763
IGR	3587	40.99	185	19.47	**<0.0001**
**Neighborhood context**					
Island	12	0.14	153	16.11	**<0.0001**
Shelf	488	5.58	67	7.05	0.0659
Shore	509	5.82	178	18.74	**<0.0001**
Opensea	7741	88.47	552	58.11	**<0.0001**

D

Ilumina ID	logFC	P.Value	deltaBeta	gene
TOP 10-Hypermethylated				
cg15053404	-0.22	1.82375E-06	0.0005	
cg02183960	-0.21	3.7906E-06	0.0006	IL7R
cg08841318	-0.21	4.33915E-06	0.0006	RNF217
cg12451425	-0.21	4.94705E-06	0.0006	HOXB-AS1
cg06817490	-0.21	5.75005E-06	0.0007	
cg05095590	-0.32	6.36189E-06	0.0007	MAD1L1
cg24885723	-0.30	7.70744E-06	0.0007	CA6
cg21588347	-0.21	8.08859E-06	0.0008	TOP1
cg23651386	-0.26	1.03359E-05	0.0008	SAE1
cg16178058	-0.24	1.17003E-05	0.0009	ETS1
TOP 10-Hypomethylated				
cg04202945	0.21	1.00128E-09	-0.2099	FBN2
cg18274164	0.21	1.49076E-09	-0.2115	
cg08577426	0.26	1.88155E-09	-0.2598	C2
cg08947084	0.26	4.17586E-09	-0.2580	SNORD47
cg27229100	0.22	7.30838E-09	-0.2196	SNORD12
cg04687131	0.23	8.46042E-09	-0.2310	ATP9A
cg11978051	0.25	8.49758E-09	-0.2482	COL17A1
cg21554616	0.23	9.09763E-09	-0.2280	SAMD4A
cg02411312	0.25	1.19109E-08	-0.2486	WEE1
cg07034383	0.21	1.5268E-08	-0.2114	HN1

Supplementary Figure 1

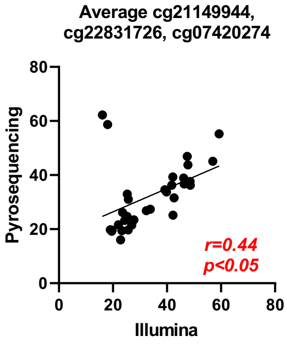

Supplementary Figure 2

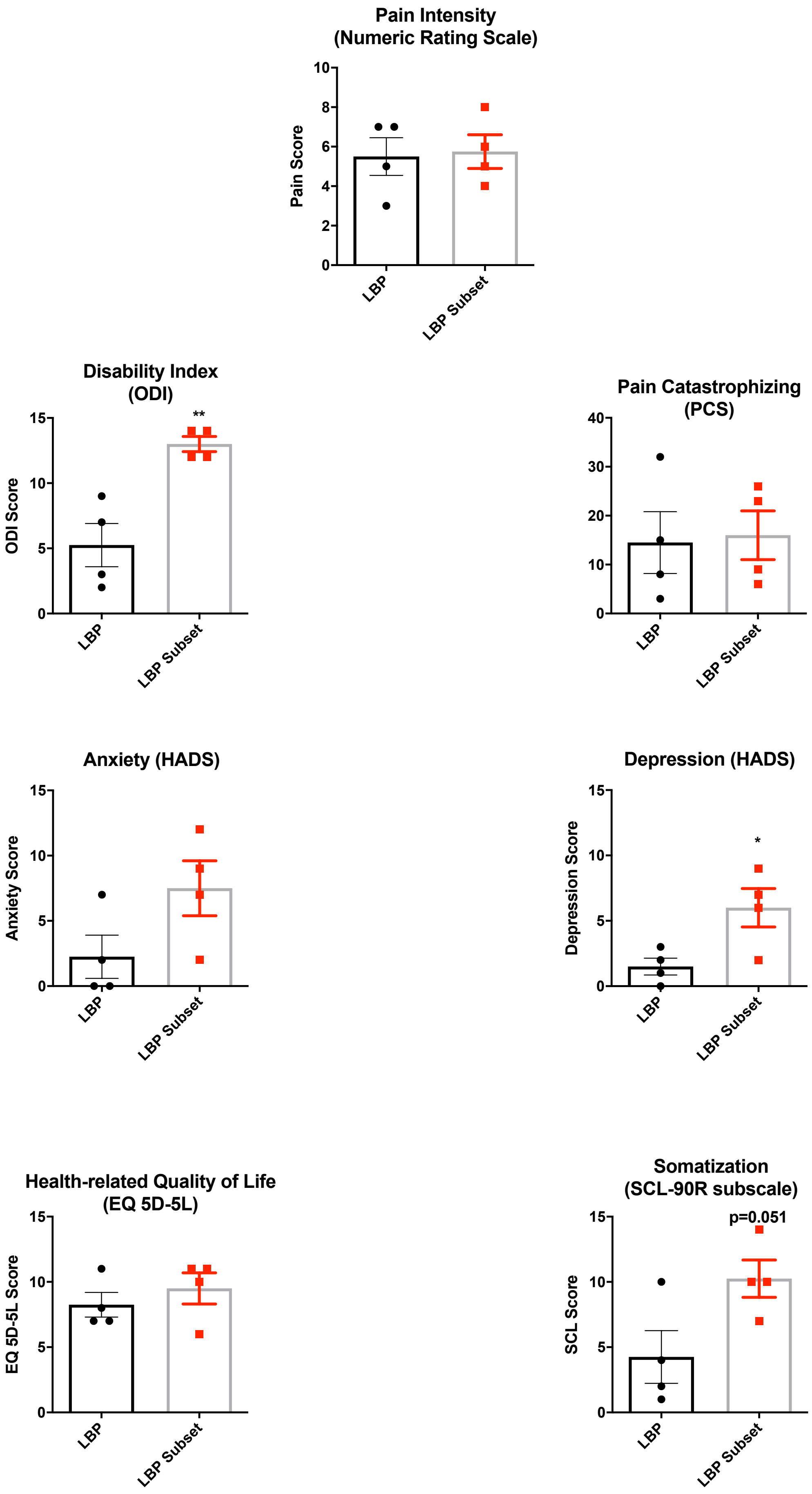

Supplementary Figure 3

A

GENE ONTOLOGY

Molecular Function

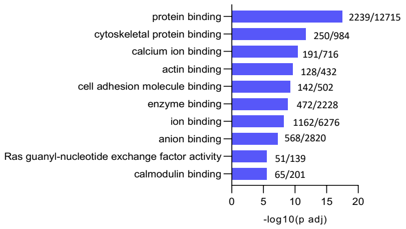

Biological Process

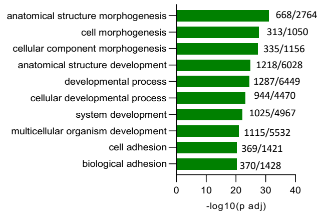

Cellular Component

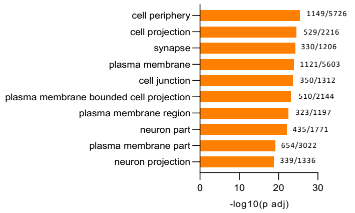

B

KEGG PATHWAY

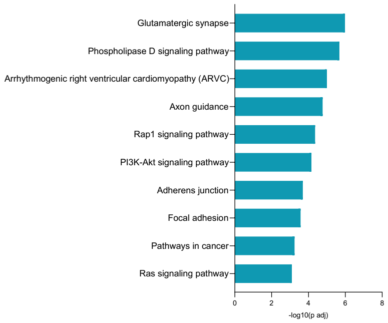

Name	Adj p value	Dysregulated genes	Dysregulated genes names
Glutamatergic synapse	1.04006E-06	45	ADCY9,GNAS,GRIN3A,GRIK1,PRKCA,PPP3CC,GNG2,ITPR2,PPP3CB,GRIK2,GRM1,CACNA1C,PLCB4,SLC17A6,PPP3CA,CACNA1D,PLCB1,ADCY2,SLC38A3,DLGAP1,GRIN2B,ADCY5,GRIN2A,GNG7,ADRBK2,SLC1A2,GNG12,GNB1,MAPK1,PLA2G4E,SLC1A3,GRM3,GNAI1,PRKCB,GRIA1,GRIN1,GRM5,SHANK1,HOMER2,PLD1,DLG4,GRM8,GRM4,PLA2G4A,GRIK4
Phospholipase D signaling pathway	2.0922E-06	53	ADCY9,GNAS,DGKA,DGKZ,AVPR1A,AGPAT5,PRKCA,CXCR2,GRM1,DGKG,PDGFD,FYN,PLCB4,RALGDS,SYK,RAPGEF4,AVPR1B,PLCB1,ADCY2,PIP5K1B,RALA,PIK3R5,ADCY5,INSR,LPAR1,DGKI,PDGFC,AGTR1,MRAS,RALB,EGFR,EGF,CYTH3,KITLG,AKT3,PDGFA,PLCG2,MAPK1,PLA2G4E,GRM3,GRM5,PLD1,PIK3R3,GRM8,CYTH1,RHEB,PIK3CG,GRM4,PLA2G4A,DGKB,CXCR1,PIK3R6,DNM3
Arrhythmogenic right ventricular cardiomyopathy (ARVC)	9.99403E-06	33	LEF1,ITGA2,TCF7,ITGA6,RYR2,SLC8A3,ITGB6,CACNA1C,ITGB5,CTNNA2,LAMA1,CACNB4,CACNA1D,ITGB3,SGCA,CACNA2D1,SGCG,CTNNA3,SLC8A1,CACNG4,ITGA9,TCF7L2,TCF7L1,CACNA2D4,PKP2,SGCD,DSG2,ITGA4,ATP2A2,ITGB1,CACNG2,ACTN2,CDH2
Axon guidance	1.71611E-05	59	ABLIM1,FES,LRRC4C,SSH1,PRKCA,EPHA5,ROBO2,PPP3CC,NFATC2,PPP3CB,SEMA3A,FYN,SEMA5A,PTK2,SEMA3C,PLXNA4,NTN4,PPP3CA,SEMA5B,SEMA3E,SLIT1,PARD3,BMP7,ABL1,SEMA3D,SMO,MET,NCK2,NGEF,PLXNA2,PLXNC1,CAMK2A,DPYSL5,EFNB2,SEMA6D,UNC5C,CXCL12,PLCG2,ENAH,PARD6G,EPHB2,MAPK1,SEMA4A,GNAI1,SLIT2,EFNA5,ROBO1,NRP1,PIK3R3,RGMA,TRPC6,SLIT3,SRGAP1,SRGAP3,ITGB1,ABLIM3,RGS3,UNC5D,SEMA6A
Rap1 signaling pathway	4.38133E-05	65	ADCY9,GNAS,SKAP1,PRKCA,RAP1GAP,RASGRP3,PDGFD,FGF9,PLCB4,RALGDS,MAGI2,RAPGEF4,MAGI1,PLCB1,ITGB3,ADCY2,FGF1,IGF1R,PARD3,RALA,GRIN2B,ITGAL,ADCY5,SIPA1L3,GRIN2A,INSR,LPAR1,ANGPT2,PDGFC,MET,CDH1,TEK,MRAS,RALB,EGFR,EGF,KITLG,AKT3,DOCK4,RAPGEF5,PDGFA,ANGPT4,ENAH,PARD6G,PRKCI,MAPK1,TLN2,GNAI1,PRKCB,CALML3,ANGPT1,EFNA5,GRIN1,PIK3R3,FPR1,THBS1,CRK,CSF1R,TIAM1,RAPGEF1,IGF1,FLT1,VAV3,ITGB1,VAV2
PI3K-Akt signaling pathway	6.9216E-05	96	IL7R,RPTOR,G6PC3,ITGA2,LAMA3,PPP2R5A,ITGA6,CDKN1B,PRKCA,NFKB1,BDNF,GNG2,NTRK1,PDGFD,C8ORF44-SGK3,ITGB6,PTK2,FGF9,LAMC1,PPP2R2B,NTRK2,ITGB5,LAMA1,SYK,MAGI2,MAGI1,RPS6,PRKAA2,ITGB3,TNXB,VWF,FGF1,PPP2R5C,IGF1R,PIK3AP1,PIK3R5,TNR,BCL2,INSR,LPAR1,GHR,ANGPT2,GNG7,PDGFC,IFNA8,MET,CREB5,TEK,EGFR,EGF,GNG12,ATF2,KITLG,YWHAQ,LAMB4,GNB1,AKT3,ITGA9,PDGFA,OSMR,ANGPT4,PPP2R2A,MAPK1,YWHAE,G6PC,PPP2R3A,TNN,SGK1,COL4A2,ANGPT1,EFNA5,CSF3R,PRLR,LAMB1,PIK3R3,HSP90AA1,THBS1,RHEB,TNC,PIK3CG,CDK6,ITGA4,FOXO3,CSF1R,FLT3,IGF1,IBSP,FLT1,TGFA,LAMA4,FASLG,THBS4,PIK3R6,ITGB1,PRL,RELN
Adherens junction	0.000202828	29	PTPRJ,LEF1,SMAD3,LMO7,YES1,TCF7,ACTN1,TGFBR2,CREBBP,WASF2,FYN,WASF3,CTNNA2,IGF1R,PARD3,WASL,SORBS1,INSR,MET,CTNNA3,CDH1,EGFR,TCF7L2,TCF7L1,MAPK1,TJP1,ACTN4,PTPRM,BAIAP2
Focal adhesion	0.000269264	60	BIRC2,ITGA2,ACTN1,DOCK1,LAMA3,ITGA6,PARVB,PRKCA,PDGFD,MAPK10,FYN,ITGB6,PTK2,LAMC1,ITGB5,LAMA1,ITGB3,TNXB,VWF,IGF1R,TNR,BCL2,PDGFC,MYLK,MET,EGFR,EGF,LAMB4,CAPN2,PXN,AKT3,ITGA9,PDGFA,MAPK1,TLN2,TNN,PRKCB,COL4A2,PARVA,PPP1CC,LAMB1,PIK3R3,THBS1,TNC,ARHGAP35,ITGA4,ZYX,CRK,ACTN4,RAPGEF1,IGF1,IBSP,FLT1,LAMA4,THBS4,VAV3,ITGB1,RELN,VAV2,MAPK8
Pathways in cancer	0.000569459	129	IL7R,ETS1,CBL,ADCY9,BIRC2,RARA,GNAS,LEF1,STAT3,SMAD3,IL6ST,ITGA2,TCF7,AXIN2,LAMA3,ITGA6,ARNT,CDKN1B,PRKCA,TGFBR2,CREBBP,GSTA4,NFKB1,CDKN2A,RUNX1,DAPK1,GNG2,MITF,RASGRP3,NTRK1,MAPK10,IFNG,PTK2,FGF9,LAMC1,PLCB4,IL5RA,RALGDS,GLI2,PTGS2,CTNNA2,LAMA1,RARB,PLCB1,ZBTB16,ADCY2,FGF1,IGF1R,GSTO2,RUNX1T1,RALA,EML4,PTGER3,ADCY5,STAT5B,ABL1,TGFB2,BCL2,LPAR1,GNG7,SMO,IFNA8,MET,ALK,PTGER2,AGTR1,STAT4,CAMK2A,CTNNA3,TRAF1,FAS,CDH1,RALB,ARHGEF11,EGFR,NCOA1,NOTCH4,EGF,LRP5,GNG12,KITLG,GLI1,LAMB4,GNB1,AKT3,TCF7L2,PDGFA,CXCL12,WNT7A,TCF7L1,PLCG2,HHIP,MAPK1,IL5,GNAI1,PRKCB,CALML3,COL4A2,CSF3R,PPARG,SLC2A1,PLD1,LAMB1,PIK3R3,HSP90AA1,HMOX1,RASGRP4,MSH3,CDK6,CRK,GSTM1,CSF1R,FLT3,GLI3,MECOM,MGST2,RASGRP1,RASSF1,IL23R,IGF1,TGFA,LAMA4,FASLG,MGST1,CCNA1,ITGB1,MAPK8,ESR1,EGLN1
Ras signaling pathway	0.000798918	66	ETS1,PRKCA,NFKB1,BDNF,GNG2,RGL1,RASGRP3,NTRK1,PDGFD,MAPK10,FGF9,NTRK2,RALGDS,EXOC2,FGF1,IGF1R,RALA,GRIN2B,GRIN2A,ABL1,KSR1,INSR,ANGPT2,GNG7,PDGFC,RASAL2,MET,PLA2G12B,TEK,MRAS,RALB,EGFR,EGF,GNG12,KITLG,RASGRF2,GNB1,AKT3,RAPGEF5,PDGFA,PLCG2,ANGPT4,TBK1,MAPK1,PLA2G4E,PRKCB,CALML3,ANGPT1,EFNA5,GRIN1,PLD1,PIK3R3,RASGRP4,ETS2,CSF1R,FLT3,TIAM1,RASGRP1,RASSF1,IGF1,FLT1,PLA2G4A,TGFA,FASLG,RASAL1,MAPK8

Supplementary Figure 4

A

GENE ONTOLOGY – HYPOMETHYLATED DMPs

WOMEN | **MEN**

Molecular Function | Biological Process | Cellular Component | Cellular Component

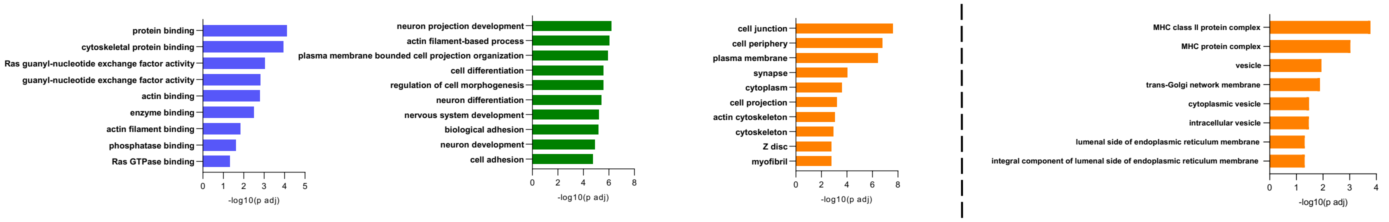

B

GENE ONTOLOGY – HYPERMETHYLATED DMPs

WOMEN

Molecular Function | Biological Process | Cellular Component

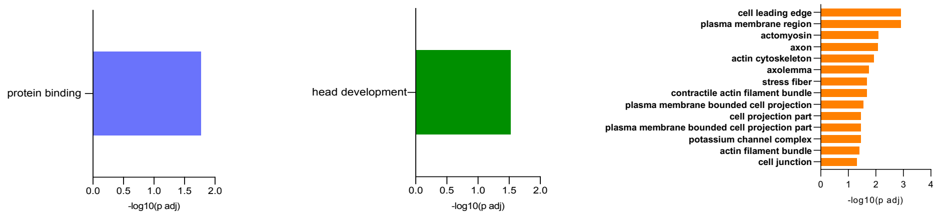

C

KEGG PATHWAY – HYPOMETHYLATED DMPs

KEGG PATHWAY – HYPERMETHYLATED DMPs

WOMEN

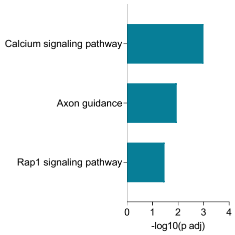

Name	Adj p value	Dysregulated genes	Dysregulated genes names
Calcium signaling pathway	0.0010	23	RYR3,PPIF,ASPH,AGTR1,LHCGR,PPP3CA,EGFR,MYLK,SLC8A1,PRKCB,CASQ2,CAMK1G,RYR2,P2RX1,EDNRB,GNAL,ATP2A2,GRM5,PPP3CB,PTGER3,PRKCG,CHRNA7,CACNA1C
Axon guidance	0.0113	20	SEMA6D,NCK2,PARD3,PARD6G,ABLIM3,FYN,NFATC2,EPHA5,SLIT3,PPP3CA,SLIT1,BMP7,EFNA5,PLXNC1,SMO,NRP1,NGEF,PLXNA4,SEMA6A,PPP3CB
Rap1 signaling pathway	0.0339	21	FARP2,RAPGEF5,PARD3,PARD6G,INSR,APBB1IP,DOCK4,CRK,ITGAL,GRIN2B,RAPGEF1,FGF1,EGFR,RAPGEF6,EFNA5,MAGI2,RASGRP3,PRKCB,ANGPT2,TLN2,PRKCG

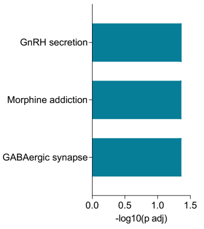

Name	Adj p value	Dysregulated genes	Dysregulated genes names
GnRH secretion	0.0429	6	KCNN3,GABBR2,ITPR1,HCN1,MAP2K1,PRKCA
Morphine addiction	0.0432	7	GABRA5,GABBR2,GABRA2,PDE8A,GNAI1,GNB1,PRKCA
GABAergic synapse	0.0432	7	GABRA5,GABBR2,GABRA2,GAD1, GNAI1,GNB1,PRKCA

Supplementary Figure 5

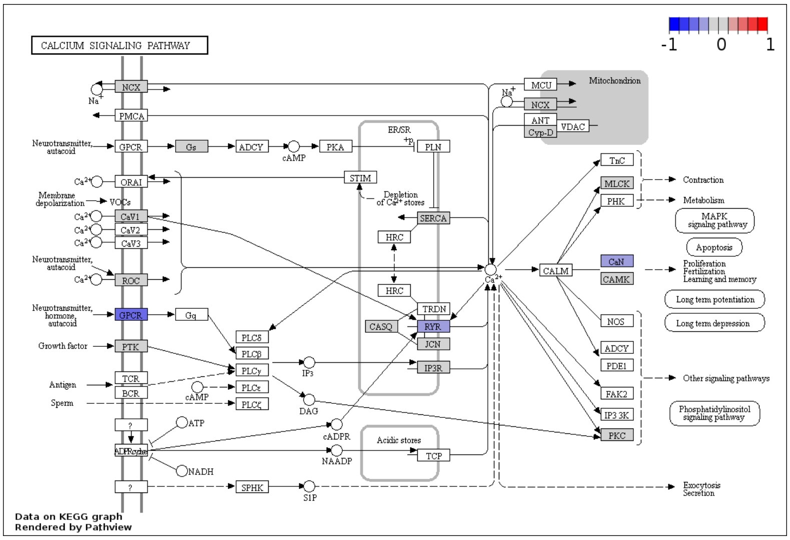

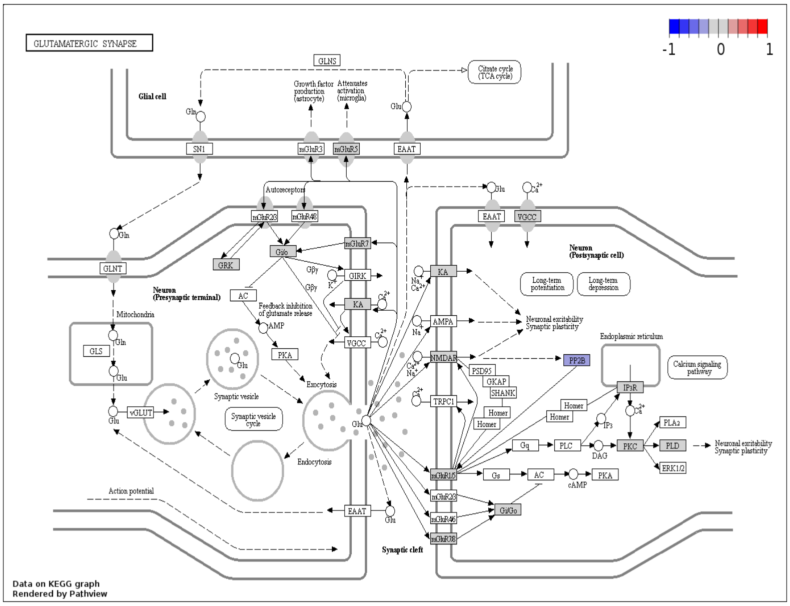

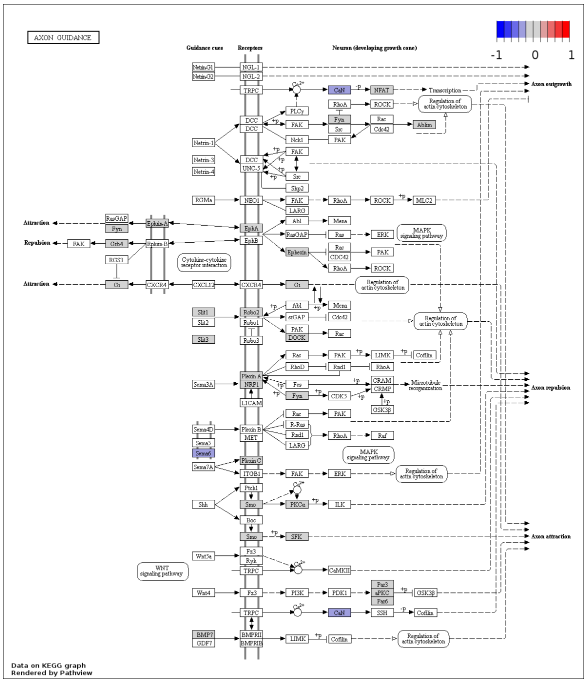

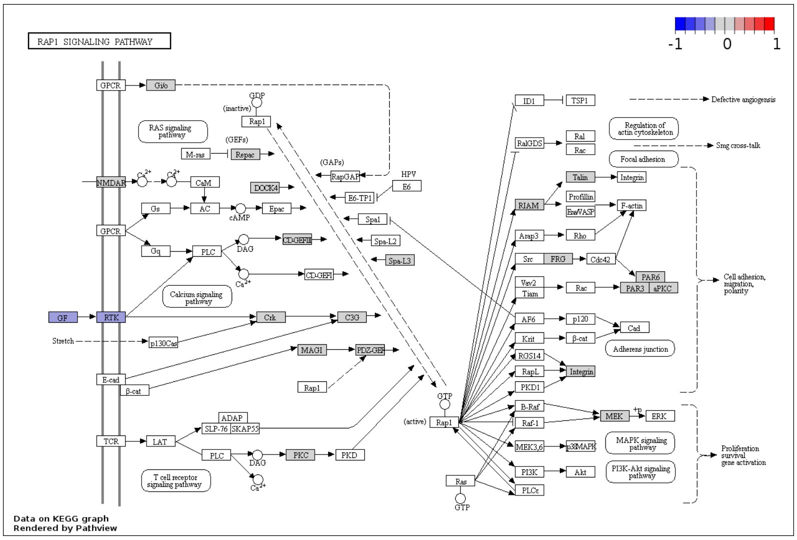

Supplementary Figure 6

SUPPLEMENTARY TABLE 1

Gene	Target ID	Forward primer	Reverse primer	Sequence primer (5' to 3')
ZNF718	cg21149944, cg22831726	ATTTAGTGTTGTGTATGGTTGTAAT	AATACCCCACCCTCCTCTCTACT	GGGAGATTTATAGTTTTATTTAAT
GAD1	cg07420274	GTTTAAGAAGGATAGTGTGGTTATAGG	AAAAAACTTTCCCATAATAATCCCCACTTC	GTGGTTATAGGGAGTG

SUPPLEMENTAL TABLE 2 - FEMALE HYPER

	logFC	AveExpr	t	P.Value	adj.P.Val	B	Normal_f_A	LBP_f_AVG	deltaBeta	CHR	MAPINFO	Strand	Type	gene	feature	cgi	feat.cgi	UCSC_Islan	SNP_ID	SNP_DISTANCE
cg0301360	-0.32593	0.451455	-2.73399	0.015115	0.224783	-4.2515	0.288492	0.614418	0.325926	1	2.37E+08	F	II	LGALS8	Body	opensea	Body-opens		rs2870452	0;11;22
cg0762977	-0.29828	0.37619	-2.66522	0.01737	0.225056	-4.38502	0.227052	0.525328	0.298276	10	13972210	R	II	FRMD4A	Body	opensea	Body-opens		rs5672810	47;37;2
cg0453118	-0.24954	0.480158	-3.7364	0.001912	0.224708	-2.21658	0.355389	0.604927	0.249538	12	10563981	R	II	KLRC4-KLRI	TSS1500	opensea	TSS1500-op		rs1167899	36
cg0804118	-0.24039	0.538699	-3.57254	0.002685	0.224708	-2.55584	0.418502	0.658895	0.240393	12	10564015	R	II	KLRC4-KLRI	TSS1500	opensea	TSS1500-op			
cg0750244	-0.12254	0.635331	-6.73463	5.9E-06	0.154235	3.688649	0.574064	0.696599	0.122536	19	57682795	F	II		IGR	shore	IGR-shore	chr19:5768		
cg1338861	-0.23676	0.567043	-2.71573	0.015685	0.224896	-4.28707	0.448664	0.685422	0.236758	2	2.39E+08	R	II	RAMP1	Body	opensea	Body-opens		rs1148629	34;33
cg0248473	-0.23338	0.4575	-2.7135	0.015756	0.224896	-4.2914	0.34081	0.57419	0.23338	6	56096198	F	II	COL21A1	5'UTR	opensea	5'UTR-open		rs1907766	18;28
cg0486300	-0.20316	0.707918	-3.31628	0.004569	0.224708	-3.08337	0.606337	0.809498	0.20316	1	59043208	F	II	TACSTD2	TSS200	island	TSS200-isla	chr1:59042		
cg1262806	-0.10266	0.54327	-6.42709	1.01E-05	0.161665	3.130623	0.49194	0.594601	0.102661	1	56453730	F	II		IGR	opensea	IGR-opense		rs1819363	6
cg2002179	-0.20017	0.292021	-3.27915	0.004935	0.224708	-3.15935	0.191936	0.392106	0.20017	17	181288	F	II	RPH3AL	5'UTR	shelf	5'UTR-shelf	chr17:185(	rs5368618	4;24;42;43
cg1934361	-0.12276	0.568623	-6.33472	1.2E-05	0.161665	2.960497	0.507242	0.630003	0.122761	22	28010528	F	I		IGR	island	IGR-island	chr22:2801	rs5363074	20;38
cg1608055	-0.19848	0.646134	-2.96619	0.009412	0.224708	-3.79268	0.546896	0.745372	0.198476	1	59043199	F	II	TACSTD2	TSS200	island	TSS200-isla	chr1:59042	rs5671867	0;3
cg0513789	-0.10577	0.401218	-6.24428	1.41E-05	0.161665	2.792843	0.348332	0.454104	0.105772	8	50164320	R	II		IGR	opensea	IGR-opense		rs1404909	40;2
cg1400521	-0.19491	0.419553	-6.69996	6.27E-06	0.154235	3.626389	0.322097	0.517008	0.194911	2	1.72E+08	F	I	GAD1	Body	island	Body-island	chr2:1716	rs5590821	19;26
cg1458008	-0.16608	0.658525	-5.99023	2.23E-05	0.186757	2.315995	0.575486	0.741564	0.166078	2	2.4E+08	F	II		IGR	opensea	IGR-opense		rs5514994	13;17
cg2542076	-0.19299	0.28644	-2.66715	0.017302	0.225034	-4.3813	0.189943	0.382937	0.192994	5	1.49E+08	R	II	ABLIM3	Body	opensea	Body-opens		rs1885730	24
cg2244084	-0.11832	0.819323	-5.96923	2.32E-05	0.186757	2.276192	0.760165	0.878481	0.118316	4	1.84E+08	F	II		IGR	shore	IGR-shore	chr4:1843	rs5314858	12;42
cg0288314	-0.18773	0.61487	-6.71897	6.06E-06	0.154235	3.660545	0.521006	0.708735	0.187729	12	9217769	F	II	LOC14457	TSS200	shore	TSS200-sho	chr12:921		
cg0929604	-0.18623	0.168839	-4.62258	0.000312	0.224708	-0.38554	0.075723	0.261956	0.186233	15	43477606	R	I	CCNDBP1	1stExon	island	1stExon-isl	chr15:434	rs5690223	49;20;11;6;2
cg1880516	-0.1853	0.69116	-3.92594	0.001292	0.224708	-1.82337	0.59851	0.78381	0.1853	19	36265700	R	I	SNX26	TSS1500	shore	TSS1500-sh	chr19:362(	rs807487;r	50;49;42
cg2019824	-0.18299	0.586682	-4.97117	0.000156	0.224708	0.322085	0.495188	0.678176	0.182987	6	29855110	R	II	HLA-H	TSS1500	shore	TSS1500-sh	chr6:2985	rs1611607	50;49;27
cg0715538	-0.18138	0.513422	-3.16545	0.006243	0.224708	-3.39102	0.422734	0.60411	0.181376	17	180856	R	II	RPH3AL	5'UTR	opensea	5'UTR-open		rs2018246	10;1
cg0935910	-0.17666	0.571613	-3.667	0.002208	0.224708	-2.36039	0.483283	0.659943	0.176659	1	1.55E+08	F	II	KCNN3	Body	opensea	Body-opens			
cg0921968	-0.17487	0.481411	-2.3932	0.029871	0.232433	-4.89991	0.393976	0.568846	0.17487	10	11789383	F	II	ECHDC3	Body	opensea	Body-opens		rs2012984	0;1;14;22;37;50
cg0925585	-0.17456	0.146688	-3.28661	0.004859	0.224708	-3.1441	0.059408	0.233968	0.174559	2	65607991	R	II	SPRED2	Body	opensea	Body-opens		rs2308100	1
cg2430901	-0.17108	0.749967	-3.46718	0.003342	0.224708	-2.77332	0.664424	0.835509	0.171085	21	44444757	F	II	PKNOX1	Body	opensea	Body-opens		rs5289529	0;1;18;19;20
cg2513464	-0.53552	0.529832	-5.33181	7.72E-05	0.220729	1.041641	0.262073	0.797592	0.535519	12	25454990	R	II		IGR	opensea	IGR-opense		rs3770902	12;1
cg0873857	-0.17076	0.41102	-3.09043	0.007289	0.224708	-3.54297	0.32564	0.4964	0.170761	1	1475265	R	I	C1orf70	Body	shore	Body-shore	chr1:1476(		
cg1068062	-0.11087	0.721394	-5.29413	8.3E-05	0.223139	0.967136	0.665959	0.77683	0.11087	5	1.39E+08	R	I		IGR	opensea	IGR-opense		rs1382188	43;23;22;5
cg0789989	-0.16896	0.579775	-2.94368	0.009857	0.224708	-3.83764	0.495296	0.664254	0.168958	10	61043529	F	II	FAM13C	Body	opensea	Body-opens		rs5572479	1;10;16;32
cg0436298	-0.13524	0.382358	-5.20894	9.79E-05	0.223139	0.798076	0.314736	0.449979	0.135243	2	1.71E+08	F	II		IGR	island	IGR-island	chr2:1706	rs5518436	37
cg1898002	-0.18213	0.610219	-5.17156	0.000105	0.223139	0.723632	0.519156	0.701282	0.182125	17	33104065	R	II		IGR	opensea	IGR-opense		rs5682132	35
cg1408752	-0.1674	0.743827	-3.72371	0.001963	0.224708	-2.24287	0.660127	0.827527	0.1674	1	38349487	R	II	INPP5B	Body	opensea	Body-opens		rs5616381	20;4
cg2038021	-0.16453	0.637354	-5.68925	3.91E-05	0.208563	1.740166	0.55509	0.719618	0.164529	12	9217688	F	II	LOC14457	TSS200	island	TSS200-isla	chr12:921		
cg1353855	-0.1634	0.846485	-2.74087	0.014906	0.224708	-4.23809	0.764784	0.928186	0.163402	14	57050624	F	II	TMEM260	Body	shelf	Body-shelf	chr14:570	rs1067952	1
cg0742027	-0.16284	0.498581	-6.62852	7.1E-06	0.154235	3.49756	0.41716	0.580002	0.162842	2	1.72E+08	F	II	GAD1	Body	shore	Body-shore	chr2:1716	rs5343961	48
cg1882229	-0.16216	0.575466	-4.3165	0.00058	0.224708	-1.01454	0.494385	0.656547	0.162162	11	1.09E+08	F	II	DDX10	Body	opensea	Body-opens		rs5374019	28
cg2282286	-0.16214	0.534169	-2.52913	0.02282	0.226555	-4.64546	0.453097	0.615241	0.162144	8	1.45E+08	F	II	PLEC1	Body	island	Body-island	chr8:1450(	rs3769539	0;41
cg1307736	-0.15997	0.440934	-3.13054	0.00671	0.224708	-3.46183	0.360948	0.52092	0.159972	18	34908626	F	II	BRUNOL4	Body	opensea	Body-opens		rs1838668	1;25
cg2611412	-0.15775	0.753343	-7.48611	1.66E-06	0.133374	4.998089	0.674469	0.832216	0.157747	12	9217669	F	I	LOC14457	TSS200	island	TSS200-isla	chr12:921		
cg1669914	-0.15711	0.532242	-2.89021	0.010997	0.224708	-3.94406	0.453685	0.610799	0.157114	1	59043255	R	II	TACSTD2	TSS200	island	TSS200-isla	chr1:59042		
cg0532888	-0.15703	0.203823	-3.0155	0.008505	0.224708	-3.69387	0.125308	0.282338	0.157031	11	30943623	F	II	DCDC5	Body	opensea	Body-opens		rs5757152	1
cg1640275	-0.15359	0.797666	-2.36884	0.031335	0.23376	-4.94485	0.720868	0.874463	0.153595	10	35311004	R	II	CUL2	Body	opensea	Body-opens		rs1873344	7;1
cg2203827	-0.11006	0.411247	-4.75028	0.000242	0.224708	-0.12508	0.356218	0.466276	0.110057	15	75382874	F	II		IGR	opensea	IGR-opense		rs1461053	51
cg1089968	-0.1115	0.55446	-4.72934	0.000252	0.224708	-0.16771	0.498712	0.610209	0.111497	5	1.59E+08	F	II		IGR	island	IGR-island	chr5:1585	rs5374335	2;9
cg2557180	-0.15291	0.722141	-3.14945	0.006453	0.224708	-3.4235	0.645685	0.798598	0.152913	3	69299492	F	II	FRMD4B	Body	opensea	Body-opens		rs5397297	1
cg2327228	-0.1516	0.443945	-2.76928	0.014071	0.224708	-4.18254	0.368146	0.519745	0.151599	10	61043477	F	II	FAM13C	Body	opensea	Body-opens			
cg1090950	-0.15116	0.589185	-2.72279	0.015462	0.224896	-4.27333	0.513606	0.664764	0.151157	17	38081995	F	II	ORMDL3	5'UTR	shore	5'UTR-shor	chr17:380	rs1886344	0;1;26;28;39
cg0333616	-0.15102	0.301073	-4.46495	0.000429	0.224708	-0.70874	0.225563	0.376583	0.15102	11	2930995	R	I	SLC22A18	Body	island	Body-island	chr11:293(	rs5274670	37;19;15;10
cg0361542	-0.14937	0.496266	-2.2904	0.036521	0.238803	-5.08808	0.421583	0.57095	0.149367	16	19777410	R	II	IQCK	Body	opensea	Body-opens		rs1909659	2
cg0877731	-0.10064	0.655417	-4.60056	0.000326	0.224708	-0.43058	0.605098	0.705736	0.100638	5	1.59E+08	R	I		IGR	island	IGR-island	chr5:1585	rs5504999	34;24
cg1805700	-0.14899	0.40363	-4.29588	0.000605	0.224708	-1.05711	0.329136	0.478124	0.148989	16	87309765	R	II	LOC10192	Body	opensea	Body-opens		rs1825039	17;10;8;7;3
cg1796634	-0.11594	0.228045	-4.56107	0.000353	0.224708	-0.51144	0.170074	0.286015	0.115941	7	1.02E+08	F	II		IGR	shelf	IGR-shelf	chr7:1020	rs5424251	1;14;16
cg1201763	-0.14881	0.761029	-4.12206	0.000863	0.224708	-1.41668	0.686623	0.835434	0.148811	7	1980892	F	II	MAD1L1	Body	shore	Body-shore	chr7:1978(	rs7686202	0;21
cg2503563	-0.11093	0.39795	-4.48067	0.000415	0.224708	-0.67643	0.342484	0.453415	0.110931	1	35586358	F	I		IGR	island	IGR-island	chr1:3558(	rs5398088	13;20;30
cg0180803	-0.14615	0.338329	-2.48384	0.024972	0.228286	-4.73091	0.265255	0.411403	0.146147	22	45809952	R	I	RIBC2	Body	island	Body-island	chr22:458(	rs5691365	36
cg1391880	-0.14545	0.596533	-3.54271	0.002857	0.224708	-2.61749	0.523807	0.669258	0.145451	1	2043761	R	II	PRKCZ	5'UTR	shelf	5'UTR-shelf	chr1:2046	rs5560603	45;2
cg0135185	-0.14554	0.433475	-4.44746	0.000444	0.224708	-0.7447	0.360703	0.506247	0.145544	22	39320511	F	II		IGR	shelf	IGR-shelf	chr22:393	rs5611344	8
cg1927489	-0.14524	0.343718	-2.63567	0.018435	0.225076	-4.44201	0.271099	0.416338	0.145239	7	1.54E+08	R	I	DPP6	TSS200	island	TSS200-isla	chr7:1535	rs5316338	51;45;33;30
cg1470629	-0.10116	0.756314	-4.42623	0.000464	0.224708	-0.78838	0.705733	0.806895	0.101162	2	43328266	R	I		IGR	island	IGR-island	chr2:4332	rs5371966	17;2
cg1060508	-0.1444	0.524756	-7.04003	3.49E-06	0.154235	4.230043	0.452556	0.596956	0.144401	12	9217763	F	II	LOC14457	TSS200	shore	TSS200-sho	chr12:921		

cg2317221	-0.12065	0.190578	-4.37861	0.000511	0.224708	-0.88644	0.130252	0.250903	0.12065	3	4393231	F	II		IGR	opensea	IGR-opense		rs5592476	1
cg0135394	-0.13771	0.712103	-4.34664	0.000545	0.224708	-0.95237	0.643247	0.780959	0.137712	3	1.92E+08	R	II		IGR	opensea	IGR-opense		rs1432926	36;26;24
cg1223578	-0.14214	0.249161	-2.70219	0.01612	0.224896	-4.31339	0.17809	0.320232	0.142142	3	1.23E+08	F	II	MYLK	Body	opensea	Body-opens		rs5370104	25;45
cg0182101	-0.14171	0.74671	-2.68525	0.016682	0.224899	-4.34625	0.675855	0.817565	0.141709	1	59043280	R	I	TACSTD2	TSS200	island	TSS200-isla	chr1:5904		
cg1729275	-0.14101	0.481656	-5.0005	0.000147	0.224708	0.381119	0.411149	0.552164	0.141015	19	49636594	R	II	PPFIA3	Body	island	Body-islanc	chr19:496	rs5524649	32
cg1119859	-0.1401	0.348254	-2.50969	0.023721	0.227121	-4.68221	0.278205	0.418303	0.140098	3	66548117	R	II	LRIG1	Body	shelf	Body-shelf	chr3:6655	rs1878435	21;17
cg1026238	-0.13992	0.303524	-3.47064	0.003318	0.224708	-2.76619	0.233567	0.373482	0.139915	6	29855435	R	II	HLA-H	Body	island	Body-islanc	chr6:2985	rs3128881	51;45;36;28;12
cg0498161	-0.14531	0.719188	-4.2796	0.000625	0.224708	-1.09074	0.646532	0.791845	0.145312	2	43328122	F	II		IGR	island	IGR-island	chr2:4332	rs5514137	33
cg0600151	-0.14716	0.308221	-4.25102	0.000663	0.224708	-1.1498	0.234642	0.381801	0.147159	13	23500838	R	II		IGR	island	IGR-island	chr13:235	rs5741515	37;17
cg0901263	-0.13968	0.320684	-3.77558	0.001763	0.224708	-2.13533	0.250842	0.390526	0.139684	16	90114173	R	I	LOC10013	TSS200	opensea	TSS200-ope		rs5677693	43
cg1847574	-0.10052	0.293718	-4.22108	0.000704	0.224708	-1.2117	0.243456	0.34398	0.100524	3	1.78E+08	F	II		IGR	opensea	IGR-opense		rs5766033	6
cg0628524	-0.13961	0.254389	-6.78013	5.45E-06	0.154235	3.770119	0.184585	0.324193	0.139607	14	1.06E+08	R	II	CRIP2	Body	island	Body-islanc	chr14:105		
cg1074999	-0.13949	0.445317	-2.22788	0.041218	0.24425	-5.20054	0.375572	0.515061	0.13949	2	25474327	F	I	DNMT3A	Body	shore	Body-shore	chr2:2547	rs934780;r	7;17
cg2268404	-0.1384	0.53591	-3.69757	0.002072	0.224708	-2.29706	0.466711	0.605108	0.138396	19	1904554	R	II	SCAMP4	TSS1500	shore	TSS1500-sh	chr19:190	rs1844210	16
cg2411852	-0.13759	0.557314	-2.46287	0.026033	0.229162	-4.77025	0.48852	0.626108	0.137588	13	47472330	F	I	HTR2A	TSS1500	opensea	TSS1500-op		rs5497097	13;30
cg2168951	-0.13752	0.352635	-3.06651	0.007657	0.224708	-3.59124	0.283876	0.421394	0.137518	21	47935083	F	II	DIP2A	Body	opensea	Body-opens		rs5448815	1;35
cg2089997	-0.1286	0.303018	-4.16077	0.000797	0.224708	-1.33652	0.238719	0.367317	0.128598	8	52913152	F	II		IGR	opensea	IGR-opense		rs1914176	1;34;41;46;48
cg0026582	-0.1004	0.244569	-4.15925	0.000799	0.224708	-1.33966	0.194369	0.29477	0.100401	6	82323837	R	II		IGR	opensea	IGR-opense			
cg1344069	-0.27534	0.231266	-4.12691	0.000854	0.224708	-1.40663	0.093594	0.368938	0.275343	1	1186357	F	II		IGR	shelf	IGR-shelf	chr1:1189	rs1485767	1;33;47
cg2140292	-0.13747	0.233175	-2.49404	0.024471	0.227781	-4.71172	0.164438	0.301913	0.137475	15	27112305	R	I	GABRA5	1stExon	island	1stExon-isl	chr15:271	rs5389060	37
cg1219383	-0.11044	0.506376	-4.11465	0.000876	0.224708	-1.43204	0.451159	0.561594	0.110435	17	30244370	F	I		IGR	island	IGR-island	chr17:302	rs5716466	22;24
cg0346608	-0.28942	0.733004	-4.10379	0.000896	0.224708	-1.45453	0.588293	0.877715	0.289422	5	1.39E+08	R	II		IGR	island	IGR-island	chr5:1390	rs5750726	25
cg2283063	-0.13349	0.218182	-4.08266	0.000936	0.224708	-1.49833	0.151436	0.284928	0.133492	4	37237877	R	II		IGR	opensea	IGR-opense		rs5380404	43;36;10
cg0591384	-0.10655	0.236384	-4.06869	0.000963	0.224708	-1.52729	0.183109	0.289658	0.106549	8	50139926	F	II		IGR	opensea	IGR-opense		rs5541709	1
cg0130138	-0.11463	0.360476	-4.06369	0.000973	0.224708	-1.53765	0.303163	0.417789	0.114626	18	15198067	R	I		IGR	island	IGR-island	chr18:151	rs5479163	22
cg0828036	-0.13728	0.351317	-2.61939	0.019049	0.225391	-4.47332	0.282677	0.419956	0.137278	14	71110536	F	II	TTC9	Body	shore	Body-shore	chr14:711	rs7406331	13;30;31;35;42
cg2717962	-0.13691	0.669052	-4.23955	0.000678	0.224708	-1.17351	0.600598	0.737506	0.136908	1	2.26E+08	F	I	LEFTY2	Body	island	Body-islanc	chr1:2261	rs5301844	27;33;40
cg0931419	-0.13531	0.438794	-2.30671	0.03538	0.237637	-5.0585	0.371141	0.506447	0.135306	19	22816508	R	II	ZNF492	TSS1500	shore	TSS1500-sh	chr19:228	rs5560885	12
cg1379721	-0.13503	0.424986	-3.37285	0.004064	0.224708	-2.96736	0.357473	0.492499	0.135026	7	71870614	F	II	CALN1	5'UTR	opensea	5'UTR-open		rs5639559	9;38;42;43
cg2511709	-0.13489	0.679633	-2.53671	0.022478	0.226384	-4.63109	0.612185	0.74708	0.134894	3	1.51E+08	R	II	MED12L	Body	opensea	Body-opens		rs5723240	45;35;13;2;1
cg1542335	-0.12284	0.314731	-3.97285	0.001173	0.224708	-1.72606	0.253312	0.37615	0.122839	2	25149977	R	I		IGR	opensea	IGR-opense		rs1855462	42;41
cg2135139	-0.1334	0.733803	-2.77692	0.013854	0.224708	-4.16756	0.667102	0.800503	0.133401	6	1.62E+08	F	I	AGPAT4	Body	opensea	Body-opens		rs5544091	1
cg0782490	-0.13999	0.639186	-3.95251	0.001223	0.224708	-1.76825	0.569189	0.709183	0.139993	4	1.84E+08	R	II		IGR	shore	IGR-shore	chr4:1843	rs5680286	12
cg0528318	-0.10109	0.825303	-3.94217	0.001249	0.224708	-1.7897	0.774758	0.875848	0.10109	6	79620031	F	II		IGR	shore	IGR-shore	chr6:7962	rs1877119	0;28;30
cg0509559	-0.13326	0.348789	-2.91563	0.01044	0.224708	-3.89353	0.282161	0.415417	0.133256	7	2139259	R	I	MAD1L1	Body	opensea	Body-opens		rs1895159	38
cg0302071	-0.13314	0.688516	-6.09589	1.84E-05	0.178987	2.515358	0.621945	0.755087	0.133142	17	180404	R	II	RPH3AL	5'UTR	opensea	5'UTR-open		rs5429897	47;45
cg0108931	-0.1331	0.38008	-8.71377	2.45E-07	0.083564	6.976756	0.313529	0.446631	0.133102	2	1.72E+08	F	I	GAD1	Body	island	Body-islanc	chr2:1716	rs5669427	23
cg2618868	-0.13269	0.253711	-2.38153	0.030564	0.233116	-4.92146	0.187365	0.320056	0.132691	15	50540269	R	II	HDC	Body	opensea	Body-opens		rs5370295	1
cg0088169	-0.1099	0.799943	-3.89771	0.00137	0.224708	-1.88196	0.744992	0.854895	0.109903	2	43328003	R	II		IGR	island	IGR-island	chr2:4332	rs5411003	50;23
cg1173167	-0.13248	0.606516	-4.9336	0.000168	0.224708	0.246365	0.540277	0.672756	0.132479	1	2995604	R	I	PRDM16	Body	shore	Body-shore	chr1:2997	rs5711008	42;30;25;21;15;9;1
cg1432619	-0.13179	0.32646	-2.5472	0.022012	0.225946	-4.61119	0.260567	0.392353	0.131786	9	1.17E+08	F	I	KIF12	5'UTR	island	5'UTR-islan	chr9:1168		
cg2431542	-0.11196	0.382949	-3.86654	0.001461	0.224708	-1.94663	0.326968	0.43893	0.111962	1	2.08E+08	F	II		IGR	shore	IGR-shore	chr1:2080	rs5495203	1;31;36
cg0984231	-0.13166	0.363541	-3.76289	0.00181	0.224708	-2.16164	0.297713	0.429369	0.131656	9	1.01E+08	F	II	GABBR2	Body	opensea	Body-opens		rs5464026	0;6;48
cg2250917	-0.13141	0.749808	-3.1395	0.006587	0.224708	-3.44367	0.684102	0.815514	0.131412	1	25234806	F	II	RUNX3	Body	island	Body-islanc	chr1:2523	rs1419031	4;38
cg1069876	-0.13133	0.271232	-3.86644	0.001461	0.224708	-1.94684	0.205568	0.336895	0.131326	16	1583463	R	II	TMEM204	5'UTR	shore	5'UTR-shor	chr16:158	rs1397654	21;20;1
cg1447383	-0.11288	0.650193	-3.83048	0.001574	0.224708	-2.02145	0.593755	0.706632	0.112877	9	1.31E+08	F	II		IGR	shelf	IGR-shelf	chr9:1314	rs5390953	25
cg1218996	-0.13094	0.451196	-3.10139	0.007126	0.224708	-3.52081	0.385725	0.516667	0.130941	20	31098130	F	II	LOC10192	Body	opensea	Body-opens		rs5426220	33
cg1357592	-0.13083	0.486094	-5.21366	9.7E-05	0.223139	0.807453	0.420678	0.551511	0.130833	12	9217583	R	I	LOC14457	TSS200	island	TSS200-isla	chr12:921		
cg2561426	-0.10069	0.672189	-3.81998	0.001608	0.224708	-2.04324	0.621845	0.722534	0.100688	2	2.24E+08	R	II		IGR	opensea	IGR-opense		rs5600670	2
cg2113792	-0.13055	0.397715	-5.10769	0.000119	0.223139	0.596111	0.332439	0.462991	0.130552	14	1.06E+08	F	II	CRIP2	Body	island	Body-islanc	chr14:105	rs1393120	5;25;45
cg2679499	-0.13046	0.575991	-2.99918	0.008795	0.224708	-3.72663	0.510759	0.641223	0.130464	3	11624030	F	II	VGLL4	TSS200	opensea	TSS200-ope		rs1928821	31;34;47
cg1550917	-0.13043	0.496274	-2.59114	0.02016	0.225654	-4.52744	0.431061	0.561487	0.130425	13	19919208	R	II	LOC10010	TSS200	island	TSS200-isla	chr13:199	rs5695056	35;25;1
cg0526739	-0.13014	0.3463	-4.47207	0.000423	0.224708	-0.69411	0.28123	0.411371	0.130141	20	32856898	F	I	ASIP	Body	island	Body-islanc	chr20:328		
cg0257072	-0.12963	0.504625	-2.90285	0.010716	0.224708	-3.91895	0.439811	0.569438	0.129627	9	71509406	R	II	PIP5K1B	Body	opensea	Body-opens			
cg1784291	-0.1296	0.660939	-5.45231	6.13E-05	0.219272	1.278834	0.596138	0.72574	0.129602	13	1.14E+08	F	I	ATP11A	3'UTR	island	3'UTR-islan	chr13:113	rs5389931	21;22;25;33
cg0873028	-0.12937	0.63438	-3.20364	0.005769	0.224708	-3.31339	0.569694	0.699065	0.129371	2	96922414	F	II	TMEM127	Body	opensea	Body-opens		rs5537026	17
cg2155409	-0.12929	0.716122	-3.41078	0.003756	0.224708	-2.88942	0.651479	0.780764	0.129285	16	87993346	R	I	BANP	TSS200	shore	TSS200-shc	chr16:879		
cg0006473	-0.12921	0.319584	-2.81894	0.012719	0.224708	-4.08498	0.254978	0.384189	0.129211	7	1.54E+08	R	I	DPP6	TSS200	island	TSS200-isla	chr7:1535	rs5316338	47;41;29;26
cg1591960	-0.21926	0.210989	-3.70404	0.002045	0.224708	-2.28365	0.101359	0.320618	0.219258	5	1.51E+08	F	II		IGR	opensea	IGR-opense		rs3729722	25;35;36
cg1591741	-0.12917	0.382681	-2.19035	0.044302	0.248079	-5.26729	0.318098	0.447265	0.129167	15	69010214	F	II	CORO2B	Body	opensea	Body-opens		rs5728831	0;34;38
cg2306004	-0.10668	0.496536	-3.69884	0.002067	0.224708	-2.29443	0.443194	0.549879	0.106685	12	1.31E+08	R	II		IGR	shelf	IGR-shelf	chr12:130	rs5427132	34
cg1178716	-0.12888	0.300878	-2.14945	0.047905	0.252488	-5.33935	0.236436	0.36532	0.128884	14	33407370	R	II	NPAS3	TSS1500	shelf	TSS1500-sh	chr14:334	rs5426679	29;23
cg0177786	-0.12811	0.359102	-3.69587	0.002079	0.224708	-2.30058	0.295049	0.423154	0.128106	4	56023843	R	I		IGR	island	IGR-island	chr4:5602	rs1401537	39;18
cg1569608	-0.12886	0.587698	-3.28951	0.00483	0.224708	-3.13816	0.523269	0.652127	0.128858	11	8931543	F	II	AKIP1	TSS1500	shore	TSS1500-sh	chr11:893	rs5431926	4;23

cg0767480	-0.2063	0.417705	-3.67947	0.002151	0.224708	-2.33455	0.314555	0.520855	0.2063	13	1.09E+08	R	II		IGR	opensea	IGR-opense		rs5355307	29;1
cg0611423	-0.12868	0.635903	-2.79496	0.013356	0.224708	-4.13216	0.571565	0.700241	0.128676	21	47960237	R	II	DIP2A	Body	opensea	Body-opens		rs5437551	32;2
cg1083306	-0.12808	0.619311	-3.01344	0.008541	0.224708	-3.69802	0.555272	0.683351	0.128079	12	1.12E+08	R	I	FAM109A	TSS1500	shore	TSS1500-sh	chr12:1118	rs5613937	49;16;6
cg0293344	-0.12687	0.571512	-3.66571	0.002214	0.224708	-2.36305	0.508077	0.634948	0.126871	2	10410713	R	II		IGR	opensea	IGR-opense		rs1911412	13
cg1667281	-0.12755	0.630103	-3.37503	0.004045	0.224708	-2.96289	0.566326	0.693879	0.127553	18	3606680	R	II	DLGAP1-AS	Body	opensea	Body-opens		rs5372748	13;2;1
cg1538919	-0.12752	0.617037	-2.55654	0.021605	0.225763	-4.59343	0.553277	0.680797	0.12752	3	32462380	F	II	CMTM7	Body	opensea	Body-opens		rs347160;r	13;42
cg0344291	-0.12743	0.464914	-2.47549	0.025389	0.228651	-4.74659	0.401197	0.528632	0.127434	20	44994454	R	II	SLC35C2	TSS1500	shore	TSS1500-sh	chr20:4499	rs3758712	7
cg0099187	-0.10248	0.351303	-3.64433	0.002314	0.224708	-2.40732	0.300061	0.402545	0.102485	6	1.61E+08	F	I		IGR	island	IGR-island	chr6:1611(	rs5278564	9
cg0008549	-0.10163	0.349797	-3.63133	0.002377	0.224708	-2.43424	0.298981	0.400612	0.101631	1	2.08E+08	R	II		IGR	shore	IGR-shore	chr1:20804	rs926628	40
cg1279815	-0.12738	0.241869	-3.85775	0.001487	0.224708	-1.96487	0.17818	0.305558	0.127379	1	19600719	F	I	AKR7L	TSS200	island	TSS200-isla	chr1:1960(	rs1859181	12;20;21;38
cg2316001	-0.12714	0.440754	-2.89223	0.010952	0.224708	-3.94003	0.377186	0.504322	0.127136	4	46391929	R	II	GABRA2	TSS1500	island	TSS1500-isl	chr4:4639:	rs5599773	41;13
cg2507007	-0.11258	0.708562	-3.58236	0.002631	0.224708	-2.53555	0.65227	0.764853	0.112584	15	27083993	R	II		IGR	island	IGR-island	chr15:2708	rs1155632	50;44;27
cg0541981	-0.14529	0.2649	-3.57592	0.002667	0.224708	-2.54884	0.192258	0.337543	0.145286	6	31276437	R	I		IGR	island	IGR-island	chr6:3127(	rs9264960	43;42;27;12;4
cg1886359	-0.1271	0.443622	-2.47635	0.025346	0.228558	-4.74498	0.380069	0.507174	0.127105	3	6902845	R	I	GRM7	1stExon	island	1stExon-isla	chr3:69028	rs5480698	33;19
cg0081337	-0.12674	0.541014	-3.19433	0.005882	0.224708	-3.33231	0.477646	0.604381	0.126735	1	1475209	R	I	C1orf70	Body	island	Body-island	chr1:14749	rs5689947	21
cg1334076	-0.1262	0.474364	-3.55953	0.002759	0.224708	-2.58273	0.411265	0.537463	0.126198	8	1.42E+08	F	I		IGR	opensea	IGR-opense		rs1422023	0;2;10;11;24
cg2431013	-0.12672	0.648583	-4.20635	0.000726	0.224708	-1.24218	0.585223	0.711942	0.126719	7	1.4E+08	F	II	SLC37A3	TSS1500	shore	TSS1500-sh	chr7:14009	rs4407796	26
cg2379504	-0.1267	0.416607	-5.33203	7.72E-05	0.220729	1.04208	0.353257	0.479957	0.1267	12	9217529	R	I	LOC14457:	TSS1500	island	TSS1500-isl	chr12:9217		
cg0934179	-0.12664	0.478885	-4.0196	0.001065	0.224708	-1.62908	0.415552	0.542204	0.126638	14	62036609	F	II	FLJ22447	TSS1500	opensea	TSS1500-op		rs5574028	1;14;36
cg2501024	-0.12662	0.692382	-3.00925	0.008615	0.224708	-3.70642	0.629069	0.755694	0.126625	21	37451839	R	II	LOC10013:	Body	opensea	Body-opens		rs7321061	51;50;47;38;10;2
cg0997886	-0.14846	0.382928	-3.53912	0.002878	0.224708	-2.62488	0.308698	0.457158	0.148459	4	56023921	F	I		IGR	island	IGR-island	chr4:5602:	rs5711209	36;43
cg1310834	-0.12631	0.578364	-3.77285	0.001773	0.224708	-2.14099	0.515211	0.641517	0.126306	17	11608711	R	II	DNAH9	Body	opensea	Body-opens			
cg0590328	-0.10978	0.693018	-3.52697	0.002952	0.224708	-2.64998	0.63813	0.747907	0.109777	2	1.3E+08	F	I		IGR	island	IGR-island	chr2:1303	rs5382672	3
cg0767180	-0.12942	0.265843	-3.52483	0.002965	0.224708	-2.65441	0.201132	0.330554	0.129422	5	74907694	R	I		IGR	island	IGR-island	chr5:7490	rs5558866	37;34
cg1276512	-0.11204	0.494323	-3.52374	0.002972	0.224708	-2.65664	0.438303	0.550342	0.112039	10	1.32E+08	F	I		IGR	opensea	IGR-opense		rs5503527	4;5;37
cg1834395	-0.12601	0.286977	-2.18633	0.044645	0.248546	-5.2744	0.223971	0.349984	0.126013	14	48144197	R	I	MDGA2	TSS200	island	TSS200-isla	chr14:481	rs5557021	27
cg2666629	-0.12599	0.687453	-3.04326	0.008033	0.224708	-3.63806	0.624457	0.750449	0.125992	13	1.14E+08	F	II	ATP11A	3'UTR	island	3'UTR-islan	chr13:113	rs3832910	38
cg1721093	-0.1258	0.482656	-3.06908	0.007617	0.224708	-3.58605	0.419757	0.545555	0.125798	1	59043173	R	I	TACSTD2	TSS200	island	TSS200-isla	chr1:5904	rs5273258	50;33
cg2251752	-0.1255	0.289516	-2.92136	0.010318	0.224708	-3.88213	0.226767	0.352266	0.125499	21	48056967	F	II	PRMT2	Body	shore	Body-shore	chr21:480	rs5741353	0;1;14
cg1657287	-0.12246	0.438089	-3.48154	0.003243	0.224708	-2.74371	0.376857	0.49932	0.122463	4	56024045	F	II		IGR	island	IGR-island	chr4:5602:	rs1771224	18;39
cg2445006	-0.12518	0.295599	-2.93394	0.010055	0.224708	-3.85706	0.233011	0.358187	0.125175	1	1.56E+08	R	II	SLC25A44	5'UTR	island	5'UTR-islan	chr1:1561(	rs5767060	41;26
cg2742362	-0.1248	0.459583	-2.98819	0.008996	0.224708	-3.74865	0.397181	0.521985	0.124804	3	69299355	R	II	FRMD4B	Body	opensea	Body-opens		rs5603839	12;8
cg1322162	-0.12437	0.261635	-2.66562	0.017356	0.225046	-4.38425	0.199451	0.323819	0.124368	22	47134912	F	II	CERK	TSS1500	opensea	TSS1500-op		rs1899010	24
cg1700156	-0.12393	0.34844	-4.67795	0.000279	0.224708	-0.27246	0.286474	0.410406	0.123932	1	2990490	F	I	PRDM16	Body	island	Body-island	chr1:2990(	rs5438112	13;14;18
cg1975743	-0.12386	0.573466	-4.05364	0.000993	0.224708	-1.55849	0.511539	0.635394	0.123856	7	71868412	F	II	CALN1	ExonBnd	opensea	ExonBnd-op		rs1454043	23;40;51
cg0002924	-0.12383	0.615475	-3.82422	0.001594	0.224708	-2.03443	0.553563	0.677388	0.123825	7	1.37E+08	F	II	DGKI	Body	opensea	Body-opens		rs1126399	1
cg0158409	-0.15101	0.555627	-3.39002	0.003922	0.224708	-2.9321	0.480124	0.63113	0.151006	21	45254457	R	II		IGR	opensea	IGR-opense		rs1926338	47;2
cg0567873	-0.11522	0.569759	-3.38763	0.003941	0.224708	-2.93701	0.512146	0.627371	0.115225	5	43010511	F	II		IGR	opensea	IGR-opense		rs1119135	19
cg1648094	-0.14686	0.293741	-3.37904	0.004012	0.224708	-2.95466	0.220312	0.367171	0.146859	5	10533585	F	II		IGR	opensea	IGR-opense		rs1482503	22;47
cg2137962	-0.12367	0.43181	-4.66909	0.000284	0.224708	-0.29054	0.369977	0.493643	0.123666	10	33538715	R	II	NRP1	Body	opensea	Body-opens		rs5338683	33
cg0259932	-0.1229	0.619265	-2.82664	0.012521	0.224708	-4.06982	0.557816	0.680714	0.122898	4	1.22E+08	F	II	TNIP3	TSS1500	opensea	TSS1500-op		rs1930318	14;18;21
cg1612751	-0.13169	0.732067	-3.37191	0.004072	0.224708	-2.96929	0.666221	0.797912	0.131692	10	29273678	F	II		IGR	opensea	IGR-opense		rs5363367	17
cg1234267	-0.12272	0.346528	-2.24314	0.040022	0.242641	-5.17324	0.285166	0.407889	0.122723	10	60411127	F	II	BICC1	Body	opensea	Body-opens		rs1176508	4;17;46
cg2212378	-0.159	0.359987	-3.35374	0.004228	0.224708	-3.00659	0.280487	0.439487	0.159	8	1.44E+08	F	II		IGR	opensea	IGR-opense		rs5746854	38
cg0161893	-0.12267	0.455398	-3.15506	0.006379	0.224708	-3.41211	0.394063	0.516732	0.122668	3	72870430	F	II	SHQ1	Body	opensea	Body-opens		rs7754433	0;1;24;26;34;48
cg2014157	-0.1226	0.41623	-2.50634	0.02388	0.227295	-4.68854	0.354928	0.477532	0.122605	12	12225262	R	I	BCL2L14	5'UTR	opensea	5'UTR-open		rs5577052	34;33;30;1
cg1952526	-0.11414	0.638329	-3.34218	0.004331	0.224708	-3.03031	0.581258	0.6954	0.114142	11	91864865	F	II		IGR	opensea	IGR-opense		rs1566061	25;30
cg1707940	-0.12209	0.62354	-3.14785	0.006475	0.224708	-3.42674	0.562495	0.684584	0.122089	3	1.43E+08	F	II	SLC9A9	Body	opensea	Body-opens		rs1824680	0;21;37;47
cg1561808	-0.33007	0.423645	-3.33294	0.004414	0.224708	-3.04923	0.258611	0.588679	0.330068	14	1.07E+08	R	II		IGR	opensea	IGR-opense		rs5543963	51;36;14;12;8;7;2;1
cg1928675	-0.12527	0.390131	-3.32911	0.004449	0.224708	-3.05708	0.327496	0.452766	0.125271	1	2.29E+08	R	II		IGR	opensea	IGR-opense		rs5754440	1
cg1344750	-0.11631	0.346132	-3.32414	0.004495	0.224708	-3.06727	0.287976	0.404288	0.116312	9	469777	F	I		IGR	shore	IGR-shore	chr9:4701(	rs1492647	3;13;21;24;28;34
cg1597151	-0.12183	0.645365	-2.30643	0.035399	0.237641	-5.059	0.584452	0.706278	0.121826	11	57159174	R	II	PRG2	TSS1500	opensea	TSS1500-op		rs5493842	8
cg0549100	-0.135	0.299407	-3.29285	0.004797	0.224708	-3.13133	0.231905	0.36691	0.135005	4	62066539	F	I		IGR	island	IGR-island	chr4:6206	rs1386678	21;23
cg1770058	-0.12125	0.667415	-2.865	0.011579	0.224708	-3.99402	0.606788	0.728042	0.121254	14	1.06E+08	R	II	CRIP2	Body	shore	Body-shore	chr14:1059	rs5876809	45;35;8
cg0627929	-0.12124	0.380479	-2.34464	0.032856	0.23511	-4.98928	0.319856	0.441101	0.121244	10	601816	F	II	DIP2C	Body	opensea	Body-opens		rs5692921	0;16;18;44;45;48
cg2442887	-0.10166	0.305492	-3.28506	0.004875	0.224708	-3.14727	0.254663	0.35632	0.101657	6	21664652	F	I		IGR	island	IGR-island	chr6:2166	rs5745930	11;40
cg2447656	-0.12124	0.406596	-3.78822	0.001717	0.224708	-2.10911	0.345974	0.467218	0.121244	13	95954382	F	II	ABCC4	TSS1500	shore	TSS1500-sh	chr13:959		
cg2065507	-0.10379	0.22568	-3.28435	0.004882	0.224708	-3.14871	0.173784	0.277577	0.103793	19	40315011	R	I		IGR	island	IGR-island	chr19:403:	rs2010953	41;29
cg2159496	-0.12123	0.259572	-2.20387	0.043167	0.246657	-5.24331	0.198956	0.320188	0.121232	3	3152916	F	II	IL5RA	TSS1500	opensea	TSS1500-op		rs1787849	14;27
cg2190079	-0.12121	0.648175	-2.8198	0.012697	0.224708	-4.0833	0.587568	0.708781	0.121213	8	1.45E+08	F	I	PLEC1	Body	shore	Body-shore	chr8:1450(	rs1892335	0;1;25;28
cg2556947	-0.11094	0.578358	-3.27592	0.004968	0.224708	-3.16595	0.522891	0.633826	0.110936	8	41108491	F	II		IGR	opensea	IGR-opense		rs5555040	1
cg0093061	-0.12087	0.591394	-3.93694	0.001263	0.224708	-1.80055	0.530959	0.651828	0.120869	18	74088820	R	II	ZNF516	Body	shelf	Body-shelf	chr18:7409	rs5670754	47;46
cg1117363	-0.28883	0.313195	-3.26636	0.005067	0.224708	-3.18547	0.16878	0.45761	0.288831	10	65632259	R	II		IGR	opensea	IGR-opense		rs1418313	38;30;17;14;9
cg0006190	-0.12073	0.665485	-3.3524	0.00424	0.224708	-3.00934	0.605121	0.725849	0.120728	11	8931485	F	II	AKIP1	TSS1500	shore	TSS1500-sh	chr11:893:	rs5718732	0;25;38;39

cg2622001	-0.10397	0.167143	-3.26443	0.005087	0.224708	-3.18942	0.115158	0.219128	0.10397	8	76320150	R	I		IGR	island	IGR-island	chr8:7631	rs5527491	49;45;42;35
cg0354616	-0.12068	0.390168	-2.33786	0.033294	0.235486	-5.0017	0.329826	0.450511	0.120684	6	35654363	R	II	FKBP5	5'UTR	shore	5'UTR-shor	chr6:3565		
cg0525649	-0.1205	0.451034	-3.55056	0.002811	0.224708	-2.60127	0.390784	0.511284	0.1205	2	74643745	F	II	C2orf81	Body	shore	Body-shore	chr2:7464	rs5752104	25
cg0710491	-0.12042	0.454563	-4.189	0.000752	0.224708	-1.27808	0.394353	0.514772	0.120419	11	46958073	R	I	C11orf49	TSS200	shore	TSS200-sho	chr11:469	rs5739559	22
cg0447920	-0.10842	0.532699	-3.24929	0.005249	0.224708	-3.22034	0.478488	0.58691	0.108422	8	61823106	R	II		IGR	shore	IGR-shore	chr8:6182		
cg0879156	-0.12041	0.437463	-4.50157	0.000398	0.224708	-0.63349	0.377255	0.49767	0.120415	2	66736054	F	II	MEIS1	Body	opensea	Body-opens		rs2005358	34
cg1907653	-0.11839	0.557531	-3.23935	0.005359	0.224708	-3.24061	0.498338	0.616723	0.118385	18	75072036	R	II		IGR	opensea	IGR-opense		rs5659027	39;36;35;28;10;2
cg1317200	-0.12003	0.513543	-2.68455	0.016705	0.224901	-4.34761	0.453528	0.573557	0.120029	2	88390175	F	II	SMYD1	Body	opensea	Body-opens		rs5749572	1;47
cg1490514	-0.11991	0.472547	-2.76801	0.014107	0.224708	-4.18502	0.412591	0.532502	0.119911	15	70387094	R	II	TLE3	5'UTR	shore	5'UTR-shor	chr15:7038	rs5513355	11;6;5
cg1864425	-0.11991	0.630572	-5.93147	2.49E-05	0.190792	2.204495	0.570619	0.690526	0.119907	12	9217859	F	I	LOC14457	Body	shore	Body-shore	chr12:921		
cg2488572	-0.11978	0.629596	-2.87856	0.011262	0.224708	-3.96716	0.569705	0.689487	0.119783	1	9030439	F	II	CA6	Body	opensea	Body-opens		rs5342159	14
cg1347822	-0.11965	0.232286	-2.83937	0.0122	0.224708	-4.0447	0.17246	0.292111	0.119651	18	70209432	R	I	CBLN2	5'UTR	island	5'UTR-islan	chr18:702	rs5719552	38;12
cg1140863	-0.11968	0.431794	-3.22788	0.005487	0.224708	-3.26399	0.371952	0.491636	0.119685	1	4856487	R	II		IGR	opensea	IGR-opense		rs5455687	18;10;7
cg2301988	-0.10284	0.684294	-3.22691	0.005498	0.224708	-3.26599	0.632876	0.735713	0.102837	1	1.47E+08	F	II		IGR	island	IGR-island	chr1:1465	rs5875975	12;33;46;49
cg2093329	-0.14066	0.601485	-3.21728	0.005609	0.224708	-3.28561	0.531157	0.671813	0.140656	11	1.29E+08	R	II		IGR	island	IGR-island	chr11:128	rs5274549	23
cg1206742	-0.13015	0.481315	-3.2112	0.00568	0.224708	-3.29798	0.416239	0.546391	0.130152	1	2.03E+08	R	II		IGR	shelf	IGR-shelf	chr1:2032	rs1921741	51;41;5
cg0177801	-0.12437	0.676898	-3.20902	0.005706	0.224708	-3.30242	0.614714	0.739081	0.124367	1	1.93E+08	R	II		IGR	opensea	IGR-opense			
cg0213271	-0.11946	0.421236	-3.28446	0.004881	0.224708	-3.1485	0.361505	0.480966	0.119461	17	46656690	F	I	HOXB4	TSS1500	shore	TSS1500-sh	chr17:466	rs5514207	6
cg2521871	-0.11928	0.196086	-3.04817	0.007952	0.224708	-3.62819	0.136449	0.255724	0.119275	20	22565228	R	I	FOXA2	5'UTR	island	5'UTR-islan	chr20:225		
cg0175975	-0.11926	0.877483	-2.35532	0.032176	0.234492	-4.96971	0.817851	0.937115	0.119264	11	66579670	R	II	C11orf80	Body	opensea	Body-opens			
cg0106894	-0.11101	0.201936	-3.19226	0.005907	0.224708	-3.33653	0.146431	0.257442	0.111011	20	9049273	F	I		IGR	island	IGR-island	chr20:904		
cg0681837	-0.11891	0.576859	-3.83022	0.001574	0.224708	-2.02198	0.517404	0.636315	0.118911	8	56793504	R	II	LYN	5'UTR	shore	5'UTR-shor	chr8:5679	rs6474026	13
cg1153313	-0.14462	0.596468	-3.18103	0.006046	0.224708	-3.35937	0.524156	0.668779	0.144622	12	1.05E+08	F	II		IGR	opensea	IGR-opense		rs7650844	0
cg0476044	-0.11891	0.393542	-2.19042	0.044296	0.248068	-5.26716	0.334089	0.452994	0.118905	21	46890849	R	II	COL18A1	Body	island	Body-islanc	chr21:468	rs1411442	21
cg0025588	-0.11889	0.327787	-4.3885	0.000501	0.224708	-0.86608	0.268341	0.387232	0.118892	2	1.29E+08	F	II	WDR33	TSS1500	shore	TSS1500-sh	chr2:1285	rs1155108	19
cg0664508	-0.11879	0.641987	-2.90491	0.010671	0.224708	-3.91484	0.582593	0.70138	0.118787	15	85553820	F	II	PDE8A	5'UTR	opensea	5'UTR-open		rs6811599	10
cg0188405	-0.10873	0.372507	-3.16164	0.006293	0.224708	-3.39875	0.318142	0.426872	0.10873	2	25150051	F	II		IGR	opensea	IGR-opense		rs5346995	1
cg1394228	-0.11863	0.521574	-2.68971	0.016532	0.224896	-4.3376	0.462259	0.580889	0.11863	10	1.13E+08	R	II	RBM20	Body	opensea	Body-opens		rs1442822	35;27
cg1123194	-0.11863	0.319803	-2.18244	0.044979	0.248899	-5.28128	0.260489	0.379118	0.118629	8	63161616	R	I	NKAIN3	1stExon	island	1stExon-isl	chr8:6316	rs5353119	28;6
cg0884131	-0.11846	0.155284	-3.62731	0.002397	0.224708	-2.44256	0.096054	0.214514	0.11846	6	1.25E+08	F	I	RNF217	1stExon	island	1stExon-isl	chr6:1252		
cg1191983	-0.10064	0.649586	-3.15366	0.006397	0.224708	-3.41496	0.599268	0.699904	0.100636	8	57350735	R	II		IGR	shore	IGR-shore	chr8:5735	rs5461053	15;11
cg0619495	-0.11836	0.544374	-2.38851	0.030147	0.232737	-4.90857	0.485193	0.603555	0.118362	16	27244433	F	II	NSMCE1	Body	opensea	Body-opens		rs2000325	0;1;29
cg0773632	-0.11834	0.776206	-5.5114	5.47E-05	0.219272	1.394535	0.717034	0.835378	0.118344	4	52772969	R	II	DCUN1D4	Body	opensea	Body-opens		rs1817708	50;1
cg1464892	-0.11823	0.713025	-3.04836	0.007949	0.224708	-3.6278	0.653908	0.772141	0.118233	4	1.7E+08	F	II	PALLD	5'UTR	opensea	5'UTR-open		rs5382954	1;11;42
cg0351346	-0.18258	0.644011	-3.13553	0.006641	0.224708	-3.45171	0.55272	0.735303	0.182584	1	58898672	F	II		IGR	opensea	IGR-opense		rs5765044	0;1;17;22;41
cg1620195	-0.11797	0.524504	-2.61875	0.019073	0.225391	-4.47455	0.465517	0.58349	0.117973	4	1.41E+08	F	II	MAML3	Body	opensea	Body-opens		rs5290159	0
cg2436884	-0.11779	0.383972	-2.13811	0.048952	0.253847	-5.35922	0.325078	0.442866	0.117788	19	58545160	F	I	ZSCAN1	TSS1500	island	TSS1500-isl	chr19:585	rs5561099	15
cg1277689	-0.11755	0.279811	-2.14693	0.048137	0.252876	-5.34378	0.221037	0.338586	0.117549	9	1.15E+08	F	II	MIR3134	Body	opensea	Body-opens			
cg1624081	-0.16512	0.391754	-3.11444	0.006937	0.224708	-3.49441	0.309196	0.474313	0.165117	2	65861662	F	II		IGR	opensea	IGR-opense		rs5340833	51
cg2171399	-0.11461	0.427962	-3.10419	0.007085	0.224708	-3.51516	0.370656	0.485268	0.114613	8	21718256	F	II		IGR	opensea	IGR-opense			
cg0088473	-0.11738	0.268788	-2.61339	0.01928	0.225391	-4.48484	0.210099	0.327478	0.117379	9	85678133	F	I	RASEF	TSS200	island	TSS200-isla	chr9:8567	rs1505397	18;47
cg2221339	-0.11734	0.319326	-2.31502	0.034812	0.237078	-5.04338	0.260657	0.377996	0.11734	1	2.01E+08	F	I	C1orf106	TSS200	island	TSS200-isla	chr1:2008	rs5712494	16;38
cg2098575	-0.11695	0.25862	-2.66253	0.017464	0.225076	-4.39023	0.200142	0.317097	0.116955	3	24536474	R	I	THRB	TSS200	island	TSS200-isla	chr3:2453		
cg1097362	-0.11672	0.69928	-2.3718	0.031153	0.233679	-4.93941	0.640923	0.757638	0.116715	2	86423274	R	II	IMMT	TSS1500	shore	TSS1500-sh	chr2:8642	rs5405563	40
cg1221874	-0.10842	0.680235	-3.08613	0.007354	0.224708	-3.55165	0.626023	0.734447	0.108424	21	37451666	R	II		IGR	opensea	IGR-opense		rs1865503	51;18;1
cg2042671	-0.11638	0.427998	-3.66141	0.002233	0.224708	-2.37195	0.369808	0.486188	0.11638	22	50248907	F	II	ZBED4	5'UTR	shore	5'UTR-shor	chr22:502		
cg0163454	-0.11633	0.310935	-2.99886	0.008801	0.224708	-3.72727	0.252768	0.369102	0.116333	14	71128147	R	II	TTC9	Body	opensea	Body-opens		rs1899764	2
cg2303267	-0.11623	0.714442	-3.1237	0.006806	0.224708	-3.47568	0.656328	0.772555	0.116226	17	18671452	R	II	FBXW10	Body	opensea	Body-opens		rs1875588	50;32;22;4
cg0738577	-0.10529	0.551991	-3.0766	0.0075	0.224708	-3.57088	0.499344	0.604638	0.105294	3	72320634	F	II		IGR	opensea	IGR-opense		rs1437369	1
cg0579584	-0.11596	0.476252	-4.79771	0.00022	0.224708	-0.02869	0.41827	0.534233	0.115963	3	4794082	F	II	ITPR1	Body	opensea	Body-opens		rs1866997	17;46
cg1694222	-0.11563	0.294863	-2.84888	0.011966	0.224708	-4.02591	0.237049	0.352677	0.115628	4	6050460	F	I	JAKMIP1	Body	opensea	Body-opens		rs5435200	14;25;37
cg0470751	-0.12044	0.344342	-3.06577	0.007669	0.224708	-3.59273	0.284122	0.404563	0.120442	10	21799314	F	I		IGR	island	IGR-island	chr10:217	rs1115909	43
cg0232048	-0.11559	0.297609	-2.37063	0.031225	0.233736	-4.94155	0.239814	0.355404	0.11559	13	1.1E+08	R	I	IRS2	5'UTR	island	5'UTR-islan	chr13:110	rs1864147	6
cg0203544	-0.11527	0.514364	-2.33509	0.033475	0.23565	-5.00676	0.456729	0.572	0.115272	7	40027398	F	II	CDK13	Body	opensea	Body-opens		rs1490287	0;47
cg1697772	-0.11522	0.604932	-3.0628	0.007716	0.224708	-3.59871	0.547324	0.66254	0.115216	2	85105984	R	II	TRABD2A	Body	shore	Body-shore	chr2:8510		
cg0008466	-0.11515	0.432454	-2.33922	0.033206	0.235369	-4.9992	0.374878	0.490029	0.115151	3	1.89E+08	F	I	TPRG1-AS1	TSS200	island	TSS200-isla	chr3:1886	rs1496605	17;21;30;48;49
cg2633390	-0.11512	0.407175	-2.44378	0.027035	0.22984	-4.80595	0.349614	0.464736	0.115122	8	17354701	F	I	SLC7A2	1stExon	island	1stExon-isl	chr8:1735	rs5514557	34;43
cg1955158	-0.11496	0.155519	-2.93797	0.009973	0.224708	-3.84902	0.098039	0.212999	0.11496	20	62579792	R	I	UCKL1	Body	shelf	Body-shelf	chr20:625	rs5408851	41;37;36;12;1
cg2515095	-0.11496	0.373907	-4.65485	0.000292	0.224708	-0.31959	0.316429	0.431386	0.114957	4	41540229	R	II	LIMCH1	Body	opensea	Body-opens		rs8017685	46;43;22
cg1827384	-0.11491	0.30752	-3.26152	0.005118	0.224708	-3.19536	0.250065	0.364975	0.11491	5	45695643	R	I	HCN1	Body	island	Body-islanc	chr5:4569	rs1138600	32
cg0770023	-0.12256	0.380832	-3.03603	0.008153	0.224708	-3.65262	0.319553	0.442111	0.122558	4	39171231	R	II		IGR	shore	IGR-shore	chr4:3917		
cg0803523	-0.10249	0.132217	-3.02944	0.008265	0.224708	-3.66586	0.080975	0.18346	0.102485	1	62107430	R	II		IGR	opensea	IGR-opense			
cg0121643	-0.10181	0.188371	-3.02669	0.008312	0.224708	-3.6714	0.137465	0.239276	0.101811	15	77376235	F	I		IGR	opensea	IGR-opense		rs5275803	0;30
cg0606298	-0.10333	0.239228	-3.02211	0.00839	0.224708	-3.68061	0.187563	0.290892	0.103328	3	22413960	R	I		IGR	island	IGR-island	chr3:2241		

cg0868262	-0.11487	0.403757	-3.07725	0.00749	0.224708	-3.56958	0.346323	0.461192	0.114869	8	1.28E+08	F	II	LOC72767	Body	opensea	Body-opens		rs1837544	1;24;32;35
cg2737910	-0.10095	0.401581	-3.01671	0.008484	0.224708	-3.69145	0.351105	0.452057	0.100952	5	74907287	R	II		IGR	island	IGR-island	chr5:7490	rs5765296	38;29;25;21;15
cg1221270	-0.11466	0.267724	-2.59684	0.019931	0.225549	-4.51655	0.210393	0.325055	0.114661	19	42817984	R	II	TMEM145	Body	island	Body-island	chr19:428	rs1146286	37
cg0245746	-0.11456	0.542654	-3.03918	0.008101	0.224708	-3.64627	0.485372	0.599936	0.114565	8	9913680	R	II	MSRA	Body	shore	Body-shore	chr8:9911	rs1400165	22
cg1777628	-0.11443	0.669184	-2.96138	0.009505	0.224708	-3.80228	0.611971	0.726397	0.114426	1	1.54E+08	F	II	S100A13	5'UTR	opensea	5'UTR-open		rs5489756	6;31;46
cg1346064	-0.11433	0.280301	-2.27303	0.037773	0.240072	-5.11949	0.223138	0.337465	0.114327	1	2.1E+08	F	I	G0S2	1stExon	island	1stExon-isl	chr1:2098	rs1154271	8;17
cg0823916	-0.10861	0.493101	-3.00724	0.008651	0.224708	-3.71045	0.438797	0.547406	0.108609	15	1E+08	R	II		IGR	opensea	IGR-opense		rs5587818	25;11
cg0550682	-0.11431	0.697317	-2.56948	0.021053	0.225654	-4.56879	0.640161	0.754472	0.114312	13	47472349	F	II	HTR2A	TSS1500	opensea	TSS1500-op		rs1914231	11;35;36
cg0917936	-0.13033	0.624374	-3.0052	0.008687	0.224708	-3.71454	0.559209	0.689539	0.13033	22	39407384	F	II		IGR	opensea	IGR-opense		rs6001376	15;26;29;31;42
cg0378166	-0.11425	0.291754	-3.04395	0.008022	0.224708	-3.63669	0.234629	0.34888	0.114251	11	62255221	R	II	AHNAK	Body	opensea	Body-opens			
cg2574443	-0.11399	0.213441	-4.18279	0.000762	0.224708	-1.29092	0.156448	0.270434	0.113986	16	83760920	F	II	CDH13	Body	opensea	Body-opens		rs1891770	11;18;33;39
cg2002459	-0.11394	0.419027	-5.90064	2.64E-05	0.193904	2.14582	0.362059	0.475995	0.113937	11	57136075	F	II	P2RX3	Body	opensea	Body-opens		rs5723652	10;26
cg0331691	-0.11388	0.373042	-3.96558	0.001191	0.224708	-1.74113	0.316102	0.429983	0.113881	3	1.51E+08	F	II	P2RY14	5'UTR	opensea	5'UTR-open		rs1926100	20
cg0666513	-0.1136	0.75567	-3.20352	0.005771	0.224708	-3.31362	0.698869	0.812471	0.113602	16	81721842	R	II	CMIP	Body	opensea	Body-opens		rs5430912	25;9;6
cg0761878	-0.11349	0.676205	-3.75616	0.001835	0.224708	-2.1756	0.619462	0.732949	0.113487	2	2.4E+08	F	II	HDAC4	Body	opensea	Body-opens		rs5631688	5
cg1404069	-0.1134	0.695253	-2.81478	0.012827	0.224708	-4.09317	0.638553	0.751953	0.1134	7	1.17E+08	F	II	CTTNBP2	Body	opensea	Body-opens		rs3569093	19;44;45;46
cg0098703	-0.11054	0.261573	-2.97925	0.009163	0.224708	-3.76655	0.206303	0.316842	0.110539	5	1.41E+08	F	II		IGR	island	IGR-island	chr5:1408	rs5718368	8
cg0281289	-0.11334	0.167009	-3.37092	0.00408	0.224708	-2.97133	0.110337	0.223681	0.113343	8	13134146	F	II	DLC1	Body	island	Body-island	chr8:1313	rs5734852	1;27;40;49
cg0890944	-0.11829	0.56869	-2.97254	0.00929	0.224708	-3.77997	0.509545	0.627835	0.11829	15	25010154	F	II		IGR	opensea	IGR-opense		rs5594827	0;20;36
cg2434002	-0.11333	0.607557	-2.91394	0.010476	0.224708	-3.89689	0.550894	0.664221	0.113327	10	94304119	R	II	IDE	Body	opensea	Body-opens		rs1409972	6
cg2592052	-0.11321	0.479959	-2.79249	0.013423	0.224708	-4.13701	0.423353	0.536565	0.113212	17	42147463	R	II	G6PC3	TSS1500	shore	TSS1500-sh	chr17:421	rs1929657	14;11
cg1211488	-0.11315	0.571372	-2.51367	0.023534	0.22692	-4.6747	0.514798	0.627946	0.113148	8	1.08E+08	F	II	OXR1	Body	opensea	Body-opens		rs1457398	51
cg1511129	-0.1855	0.2553	-2.96271	0.009479	0.224708	-3.79964	0.162551	0.348049	0.185497	18	47847332	F	II		IGR	opensea	IGR-opense		rs5642781	0;32
cg2223644	-0.11293	0.202629	-2.57761	0.020714	0.225654	-4.55329	0.146163	0.259094	0.112931	9	13279528	F	I	MPDZ	1stExon	island	1stExon-isl	chr9:1327	rs7752040	11
cg0404085	-0.1129	0.738331	-2.329	0.033875	0.23606	-5.01789	0.681882	0.79478	0.112898	19	17201629	F	II	MYO9B	5'UTR	opensea	5'UTR-open		rs5592622	6
cg1075708	-0.11371	0.468592	-2.95887	0.009554	0.224708	-3.8073	0.411736	0.525447	0.113711	3	39336300	F	II		IGR	opensea	IGR-opense		rs5291422	1
cg1826682	-0.10032	0.434164	-2.95616	0.009608	0.224708	-3.81273	0.384003	0.484325	0.100322	17	46027572	F	II		IGR	opensea	IGR-opense			
cg1567882	-0.11287	0.424901	-3.85524	0.001495	0.224708	-1.97008	0.368465	0.481336	0.112871	18	77266975	F	II	NFATC1	Body	shelf	Body-shelf	chr18:772	rs5524270	1;7;43;47;50
cg2691714	-0.13508	0.253524	-2.94867	0.009756	0.224708	-3.82769	0.185986	0.321063	0.135078	1	91184770	R	II		IGR	shore	IGR-shore	chr1:9118	rs1143862	2;1
cg2545928	-0.11272	0.534081	-3.16052	0.006307	0.224708	-3.40102	0.477722	0.59044	0.112718	12	1.24E+08	R	II	ZNF664	5'UTR	opensea	5'UTR-open		rs5545240	45;43;1
cg1535252	-0.1127	0.562247	-3.65927	0.002243	0.224708	-2.37639	0.505897	0.618598	0.112701	22	39812626	R	II	TAB1	Body	opensea	Body-opens		rs5551565	48;2
cg2175452	-0.11249	0.614808	-4.30362	0.000595	0.224708	-1.04114	0.558562	0.671055	0.112494	6	1.49E+08	R	II	UST	Body	opensea	Body-opens			
cg2394480	-0.11223	0.288244	-2.25226	0.039323	0.241792	-5.15687	0.232128	0.344359	0.112231	20	11871384	F	I	BTBD3	TSS200	island	TSS200-isla	chr20:118		
cg0395049	-0.1122	0.633511	-3.55372	0.002792	0.224708	-2.59472	0.577409	0.689613	0.112205	11	1.27E+08	F	II	KIRREL3	Body	opensea	Body-opens		rs5459230	1
cg0854693	-0.11211	0.322916	-2.7772	0.013846	0.224708	-4.16701	0.26686	0.378973	0.112113	7	79763240	R	II	GNAI1	TSS1500	shore	TSS1500-sh	chr7:7976		
cg1799691	-0.11206	0.368842	-2.82918	0.012456	0.224708	-4.0648	0.312811	0.424874	0.112063	1	26024404	R	II	MAN1C1	Body	opensea	Body-opens		rs5575245	1
cg0863270	-0.13483	0.65658	-2.92836	0.010171	0.224708	-3.86819	0.589163	0.723997	0.134834	21	37451849	R	II		IGR	opensea	IGR-opense		rs5400640	48;20;12;1
cg1879483	-0.112	0.214209	-3.01468	0.008519	0.224708	-3.69551	0.158209	0.270209	0.112	1	2.15E+08	R	I	KCNK2	TSS1500	island	TSS1500-isl	chr1:2152	rs5336556	20
cg0769088	-0.14268	0.588751	-2.92479	0.010246	0.224708	-3.8753	0.517411	0.660091	0.142679	11	1.29E+08	R	II		IGR	island	IGR-island	chr11:128	rs5619231	28
cg0191161	-0.1336	0.310428	-2.9233	0.010277	0.224708	-3.87826	0.243627	0.377228	0.133601	3	1.96E+08	R	I		IGR	opensea	IGR-opense		rs1448881	44;40;31;14
cg0303584	-0.11175	0.549683	-2.58304	0.02049	0.225654	-4.54293	0.493807	0.605559	0.111752	6	91003200	R	II	BACH2	5'UTR	shore	5'UTR-shor	chr6:9100	rs5719522	47;26;10;1
cg2699238	-0.11175	0.688988	-3.93572	0.001266	0.224708	-1.80309	0.633113	0.744862	0.111749	3	71478152	R	II	FOXP1	5'UTR	opensea	5'UTR-open		rs7295939	18;1
cg2564315	-0.11159	0.567798	-2.23944	0.040309	0.243063	-5.17986	0.512003	0.623592	0.111589	6	1.59E+08	F	I	SYTL3	5'UTR	opensea	5'UTR-open		rs5600143	16;46
cg1073273	-0.11158	0.561734	-3.23179	0.005443	0.224708	-3.25604	0.505944	0.617523	0.111579	9	97951665	F	II	FANCC	Body	opensea	Body-opens		rs5699319	1;2;5;24
cg1348432	-0.13307	0.616349	-2.91629	0.010426	0.224708	-3.89221	0.549811	0.682886	0.133074	2	2.35E+08	R	II		IGR	opensea	IGR-opense		rs5644946	10;4;1
cg0763937	-0.11141	0.392421	-2.51397	0.02352	0.22692	-4.67414	0.336713	0.448128	0.111415	16	1584516	R	I	IFT140	Body	island	Body-island	chr16:158	rs1918718	41;26;2
cg0619004	-0.1114	0.578329	-3.5507	0.00281	0.224708	-2.60097	0.522629	0.634029	0.1114	16	83986382	R	II	OSGIN1	5'UTR	opensea	5'UTR-open		rs5447833	8
cg1641297	-0.11135	0.628151	-3.16468	0.006253	0.224708	-3.39259	0.572475	0.683827	0.111352	9	1.31E+08	R	II	FAM102A	Body	opensea	Body-opens		rs1460215	22
cg0893892	-0.11126	0.644198	-2.37351	0.031049	0.233542	-4.93626	0.588567	0.699829	0.111262	15	64110513	R	II	HERC1	5'UTR	opensea	5'UTR-open		rs5555566	13;1
cg1209722	-0.11116	0.217478	-2.24211	0.040101	0.242791	-5.17507	0.161899	0.273057	0.111158	8	16885000	R	I	EFHA2	1stExon	island	1stExon-isl	chr8:1688	rs5400761	23;13
cg1392088	-0.11111	0.174877	-2.84281	0.012115	0.224708	-4.03791	0.119324	0.23043	0.111106	21	22370438	R	I	NCAM2	TSS200	island	TSS200-isla	chr21:223	rs1115217	13
cg0874090	-0.11102	0.558607	-2.68781	0.016596	0.224896	-4.34129	0.503097	0.614118	0.111021	2	2.4E+08	R	II	HDAC4	Body	opensea	Body-opens		rs5525310	10
cg1379060	-0.11097	0.295341	-2.72873	0.015278	0.224795	-4.26177	0.239856	0.350826	0.11097	3	24536478	R	I	THRB	TSS200	island	TSS200-isla	chr3:2453		
cg0432912	-0.11088	0.508801	-4.87166	0.00019	0.224708	0.121199	0.453363	0.564239	0.110876	14	1.06E+08	F	I	CRIP2	Body	island	Body-island	chr14:105	rs5876740	39
cg2569724	-0.11083	0.560131	-2.64648	0.018038	0.225076	-4.42119	0.504715	0.615547	0.110832	3	17363960	F	II	TBC1D5	Body	opensea	Body-opens		rs5694529	0;12
cg0073947	-0.11076	0.580147	-2.31916	0.034532	0.236784	-5.03583	0.524768	0.635525	0.110757	14	69415588	F	II	ACTN1	Body	opensea	Body-opens		rs3722395	1;38
cg0513048	-0.15889	0.282486	-2.87103	0.011437	0.224708	-3.98208	0.203043	0.361929	0.158887	8	98271949	R	II		IGR	opensea	IGR-opense		rs5573853	31;7;2;1
cg0064100	-0.11049	0.498116	-2.30445	0.035536	0.237698	-5.06259	0.442871	0.553361	0.11049	3	18459838	R	II	SATB1	Body	opensea	Body-opens		rs1873370	1
cg0777530	-0.1008	0.242589	-2.86972	0.011468	0.224708	-3.98468	0.192191	0.292987	0.100795	3	12595852	F	II		IGR	shelf	IGR-shelf	chr3:1259	rs5402886	26
cg2417797	-0.11041	0.406319	-2.83233	0.012377	0.224708	-4.05859	0.351113	0.461526	0.110413	1	89127007	R	II	PKN2-AS1	Body	opensea	Body-opens		rs5410813	48;38
cg1409682	-0.11037	0.549563	-2.65011	0.017907	0.225076	-4.41419	0.494379	0.604747	0.110367	7	1.11E+08	R	II	LRRN3	1stExon	opensea	1stExon-op		rs5577189	38;6;2
cg2365138	-0.11015	0.322039	-2.92175	0.01031	0.224708	-3.88135	0.266962	0.377116	0.110154	19	47685890	F	II	SAE1	Body	opensea	Body-opens		rs5414603	44
cg1035169	-0.11011	0.23175	-3.08141	0.007426	0.224708	-3.56118	0.176696	0.286804	0.110107	11	24518557	F	I	LUZP2	5'UTR	island	5'UTR-islan	chr11:245	rs1441074	14
cg0727884	-0.14409	0.211508	-2.85518	0.011813	0.224708	-4.01346	0.139465	0.283551	0.144087	6	1.09E+08	F	II		IGR	opensea	IGR-opense		rs5584787	9;33;39

cg1191566	-0.10996	0.565064	-3.86918	0.001453	0.224708	-1.94115	0.510082	0.620045	0.109963	1	1.54E+08	R	II	S100A13	TSS200	opensea	TSS200-ope		rs1430884	1
cg1570077	-0.10979	0.442137	-3.47156	0.003311	0.224708	-2.76428	0.387245	0.49703	0.109785	4	1.09E+08	R	II	CYP2U1	TSS1500	shore	TSS1500-sh	chr4:1088	rs5487252	1
cg1398548	-0.101	0.208917	-2.84481	0.012066	0.224708	-4.03396	0.158419	0.259416	0.100997	1	2.4E+08	F	I		IGR	island	IGR-island	chr1:2395	rs5506474	3
cg0463564	-0.10974	0.450924	-2.61965	0.019038	0.225391	-4.47281	0.396054	0.505793	0.109739	16	12183938	R	I	SNX29	Body	opensea	Body-opens		rs5334388	8
cg0880284	-0.10982	0.619121	-2.84285	0.012114	0.224708	-4.03783	0.564211	0.67403	0.109819	12	89748726	F	II		IGR	shore	IGR-shore	chr12:8974		
cg2666882	-0.10967	0.380236	-2.86505	0.011577	0.224708	-3.99393	0.325403	0.435069	0.109666	6	292823	F	I	DUSP22	Body	island	Body-islanc	chr6:2919	rs815596;r	10;19;23;40
cg1576539	-0.1001	0.655264	-2.8402	0.01218	0.224708	-4.04305	0.605212	0.705316	0.100104	21	46409994	F	II		IGR	shore	IGR-shore	chr21:4641	rs1824241	0;13;38;41
cg1644005	-0.10965	0.363475	-3.87553	0.001434	0.224708	-1.92798	0.308652	0.418298	0.109646	20	32856846	F	I	ASIP	Body	island	Body-islanc	chr20:3285	rs5763371	15
cg2174015	-0.10952	0.216366	-2.17674	0.045473	0.249524	-5.29135	0.161608	0.271124	0.109516	17	181194	R	II	LOC10050	5'UTR	shelf	5'UTR-shelf	chr17:185		
cg0272545	-0.10943	0.520332	-2.78699	0.013574	0.224708	-4.14781	0.465617	0.575047	0.10943	22	50249174	F	II	ZBED4	5'UTR	shore	5'UTR-shore	chr22:5025	rs1860528	0;47
cg1040339	-0.10943	0.602644	-5.04316	0.000135	0.223139	0.466818	0.547931	0.657357	0.109426	15	63349192	R	II	TPM1	Body	opensea	Body-opens		rs2002572	2
cg2370836	-0.10943	0.358483	-2.37617	0.030887	0.233383	-4.93135	0.30377	0.413195	0.109425	7	1.46E+08	F	II	CNTNAP2	TSS200	island	TSS200-isla	chr7:1458	rs5280428	5
cg1456036	-0.14319	0.536063	-2.82715	0.012508	0.224708	-4.0688	0.464467	0.607658	0.143191	1	58898552	R	II		IGR	opensea	IGR-opensea		rs5683429	28;13
cg2546118	-0.10936	0.461225	-2.94094	0.009912	0.224708	-3.8431	0.406543	0.515907	0.109364	12	1.23E+08	F	II	MLXIP	Body	shore	Body-shore	chr12:1225	rs5315780	49
cg2130555	-0.1093	0.519241	-2.55976	0.021467	0.225654	-4.58731	0.464591	0.573892	0.109301	12	1.08E+08	R	II	BTBD11	Body	opensea	Body-opens		rs5696865	2;1
cg0849011	-0.10928	0.254593	-2.37266	0.031101	0.233625	-4.93781	0.199955	0.309232	0.109277	11	30038675	F	I	KCNA4	TSS200	island	TSS200-isla	chr11:3003	rs5386345	40;50
cg1452271	-0.10905	0.35172	-2.45268	0.026563	0.229527	-4.78931	0.297196	0.406243	0.109047	9	1.31E+08	F	I	SLC25A25	Body	opensea	Body-opens		rs5566265	2;18;24
cg0573275	-0.10729	0.294847	-2.81415	0.012844	0.224708	-4.09442	0.241201	0.348494	0.107293	7	6704045	F	I		IGR	island	IGR-island	chr7:6703		
cg0546437	-0.13128	0.210793	-2.81126	0.01292	0.224708	-4.1001	0.145151	0.276435	0.131284	10	1.12E+08	R	II		IGR	opensea	IGR-opensea		rs7335091	23;19;10;1
cg1401740	-0.12613	0.461491	-2.81053	0.012939	0.224708	-4.10155	0.398427	0.524555	0.126128	2	86225602	R	II		IGR	opensea	IGR-opensea		rs1416376	11
cg2403904	-0.11634	0.125843	-2.80193	0.013167	0.224708	-4.11846	0.067673	0.184013	0.116339	6	1.71E+08	F	II		IGR	island	IGR-island	chr6:1705	rs7761221	33;37
cg0832592	-0.10901	0.64195	-3.00605	0.008672	0.224708	-3.71284	0.587446	0.696454	0.109009	1	2.26E+08	F	II	LIN9	Body	opensea	Body-opens		rs1427713	1;50
cg1512421	-0.10899	0.341997	-2.14005	0.048771	0.253574	-5.35581	0.287503	0.396491	0.108989	4	1.73E+08	F	I	GALNTL6	TSS200	island	TSS200-isla	chr4:1727	rs5752688	6;47
cg0646342	-0.11718	0.882141	-2.794	0.013382	0.224708	-4.13405	0.82355	0.940731	0.11718	8	55471698	F	II		IGR	opensea	IGR-opensea		rs5755890	17;33;39
cg0135528	-0.10875	0.28295	-2.97266	0.009288	0.224708	-3.77974	0.228575	0.337325	0.10875	17	40264320	F	II	DHX58	5'UTR	opensea	5'UTR-open		rs1808727	0;25;50;51
cg1057024	-0.10374	0.359032	-2.791	0.013463	0.224708	-4.13993	0.30716	0.410904	0.103744	7	1.59E+08	R	I		IGR	island	IGR-island	chr7:1587	rs5532953	31;29;14;13;6;1
cg1290911	-0.1014	0.519096	-2.78956	0.013503	0.224708	-4.14277	0.468395	0.569797	0.101401	11	1.02E+08	R	II		IGR	opensea	IGR-opensea		rs7112218	46;35;24;9
cg2459186	-0.10866	0.620551	-2.93052	0.010126	0.224708	-3.86388	0.566223	0.674879	0.108656	15	66697173	F	II	MAP2K1	Body	opensea	Body-opens		rs5605854	10
cg0609229	-0.1086	0.588194	-2.25406	0.039186	0.241665	-5.15363	0.533897	0.642492	0.108596	3	71180818	F	II	FOXP1	TSS1500	opensea	TSS1500-op		rs5779440	39;51
cg0282420	-0.10474	0.234605	-2.78563	0.013611	0.224708	-4.15048	0.182235	0.286976	0.104741	18	6729810	R	I		IGR	island	IGR-island	chr18:6729		
cg1541929	-0.10859	0.641086	-2.25654	0.038999	0.24144	-5.14919	0.586793	0.695379	0.108585	19	56572945	R	II	NLRP5	3'UTR	opensea	3'UTR-open		rs3762166	47;7
cg1724391	-0.10858	0.627979	-2.72451	0.015409	0.224896	-4.26999	0.573689	0.682269	0.10858	21	47982351	R	II	DIP2A	Body	opensea	Body-opens		rs7777843	25;1
cg0579019	-0.10855	0.2893	-3.01754	0.00847	0.224708	-3.68978	0.235024	0.343576	0.108552	12	1.31E+08	R	I	FZD10-AS1	TSS1500	island	TSS1500-isl	chr12:130		
cg0975708	-0.1085	0.230464	-3.25722	0.005164	0.224708	-3.20416	0.176213	0.284715	0.108502	16	1585644	F	II	IFT140	Body	shore	Body-shore	chr16:1583	rs1414607	6
cg2181633	-0.10849	0.379234	-2.25226	0.039323	0.241792	-5.15687	0.324988	0.433481	0.108493	17	27044629	F	I	RAB34	Body	island	Body-islanc	chr17:2704	rs5419273	22;38
cg0740069	-0.10835	0.544751	-4.57767	0.000342	0.224708	-0.47745	0.490054	0.598927	0.108353	20	21599928	R	II	LOC10192	Body	opensea	Body-opens		rs1161275	36;24;1
cg0089166	-0.10825	0.330305	-2.79937	0.013236	0.224708	-4.12349	0.276181	0.38443	0.108249	20	44868936	R	II	CDH22	Body	opensea	Body-opens		rs1831786	24;13;7
cg0002708	-0.10822	0.233277	-2.78029	0.01376	0.224708	-4.16095	0.179167	0.287388	0.108221	18	5543801	F	I	EPB41L3	5'UTR	island	5'UTR-islan	chr18:5543	rs5335151	45
cg1398113	-0.10213	0.347789	-2.76908	0.014077	0.224708	-4.18293	0.296726	0.398852	0.102126	14	76734493	R	II		IGR	opensea	IGR-opensea			
cg1569471	-0.10816	0.458649	-2.99623	0.008849	0.224708	-3.73254	0.404569	0.51273	0.108161	11	1.18E+08	R	II	TMEM25	5'UTR	shore	5'UTR-shore	chr11:1184		
cg0387408	-0.10813	0.185133	-2.70156	0.016141	0.224896	-4.31462	0.13107	0.239196	0.108125	1	14925172	R	I	KAZN	TSS200	island	TSS200-isla	chr1:1492		
cg2145366	-0.10001	0.435758	-2.76454	0.014207	0.224708	-4.19181	0.385753	0.485763	0.10001	3	1.94E+08	R	II		IGR	opensea	IGR-opensea		rs7389011	44
cg1253426	-0.10805	0.699992	-2.50189	0.024093	0.227447	-4.69693	0.645968	0.754017	0.10805	1	26092973	F	II	MAN1C1	Body	opensea	Body-opens		rs1901110	41;49
cg1624832	-0.10786	0.266781	-2.17383	0.045727	0.249891	-5.29649	0.212849	0.320713	0.107864	4	1.88E+08	F	I	FAT1	5'UTR	island	5'UTR-islan	chr4:1876	rs1887875	20;36
cg0319259	-0.11829	0.303678	-2.75437	0.014503	0.224708	-4.2117	0.244533	0.362824	0.118291	19	12305854	F	I		IGR	island	IGR-island	chr19:123	rs5600366	5;9;15;22;25;32
cg0421747	-0.11242	0.308726	-2.75385	0.014519	0.224708	-4.21272	0.252514	0.364937	0.112423	12	6895978	F	II		IGR	opensea	IGR-opensea		rs1125748	7;21
cg0015553	-0.10312	0.236639	-2.75362	0.014525	0.224708	-4.21317	0.185078	0.288199	0.103122	12	1.33E+08	R	II		IGR	shelf	IGR-shelf	chr12:132	rs1124697	38;34;1
cg1082796	-0.11134	0.283971	-2.75047	0.014619	0.224708	-4.21933	0.2283	0.339641	0.111341	10	17548676	F	II		IGR	opensea	IGR-opensea		rs1845683	0;26
cg0627410	-0.11782	0.429376	-2.74885	0.014667	0.224708	-4.2225	0.370467	0.488285	0.117818	22	47798088	F	II		IGR	opensea	IGR-opensea		rs1849938	1;20;29
cg1667230	-0.10786	0.639842	-4.62096	0.000313	0.224708	-0.38886	0.585912	0.693773	0.10786	16	70780682	F	II	VAC14	Body	opensea	Body-opens		rs1482544	1;18
cg0046398	-0.10784	0.311523	-2.65113	0.01787	0.225076	-4.41224	0.257604	0.365442	0.107838	16	1583984	F	I	IFT140	Body	island	Body-islanc	chr16:1583	rs5564433	7
cg0944331	-0.10769	0.166802	-3.48505	0.00322	0.224708	-2.73647	0.112959	0.220645	0.107686	15	36871342	R	II	C15orf41	TSS1500	shore	TSS1500-sh	chr15:3687		
cg2398260	-0.10766	0.193562	-3.68018	0.002148	0.224708	-2.33308	0.139732	0.247392	0.107659	1	1823379	R	II	GNB1	TSS1500	shore	TSS1500-sh	chr1:1821	rs7571836	15;10
cg2569664	-0.10754	0.637039	-2.61881	0.019071	0.225391	-4.47442	0.583271	0.690807	0.107536	9	1.24E+08	F	II	CNTRL	Body	opensea	Body-opens		rs7500879	0;7
cg2642248	-0.10737	0.252622	-2.48306	0.025011	0.228314	-4.73237	0.198936	0.306308	0.107372	5	16935950	R	I	MYO10	1stExon	island	1stExon-isl	chr5:1693	rs5455530	38;29;18;2
cg0088520	-0.1129	0.389333	-2.73069	0.015217	0.224792	-4.25794	0.332885	0.445782	0.112897	15	70827566	R	II		IGR	opensea	IGR-opensea		rs5442891	38;19;1
cg2388136	-0.10737	0.553237	-2.27127	0.037902	0.240217	-5.12266	0.499554	0.606919	0.107366	13	47472343	F	II	HTR2A	TSS1500	opensea	TSS1500-op		rs5497097	0;17;41;42
cg1805917	-0.10735	0.31451	-4.80652	0.000216	0.224708	-0.01081	0.260837	0.368182	0.107346	10	1282755	F	II	ADARB2	Body	shore	Body-shore	chr10:128	rs5657412	0;1;3;4;23;23
cg2676970	-0.10734	0.478578	-2.92711	0.010197	0.224708	-3.87068	0.424906	0.532249	0.107344	8	1.41E+08	F	I	TRAPPC9	Body	opensea	Body-opens		rs1480574	11;12;43
cg1826511	-0.10732	0.499965	-2.46932	0.025702	0.228885	-4.75816	0.446307	0.553623	0.107316	7	2139243	F	II	MAD1L1	Body	opensea	Body-opens		rs7303844	31;43;48
cg0652857	-0.10725	0.273804	-3.09549	0.007213	0.224708	-3.53274	0.220178	0.327431	0.107253	1	2.07E+08	R	I	FAIM3	Body	island	Body-islanc	chr1:2070	rs5284563	11
cg0441517	-0.10722	0.249416	-2.44753	0.026835	0.229795	-4.79894	0.195805	0.303028	0.107222	2	1.77E+08	F	I	HOXD13	1stExon	island	1stExon-isl	chr2:1769	rs1399954	36
cg1060327	-0.10707	0.36287	-2.55798	0.021543	0.225702	-4.59069	0.309336	0.416405	0.107069	3	239665	F	I	CHL1	5'UTR	island	5'UTR-islan	chr3:2383	rs5710245	33
cg0262287	-0.10706	0.524109	-2.86522	0.011573	0.224708	-3.99359	0.470577	0.577641	0.107064	4	71836756	R	II	MOB1B	Body	opensea	Body-opens		rs5669747	2;1

cg2602487	-0.10696	0.457587	-2.69457	0.016371	0.224896	-4.32818	0.404108	0.511067	0.10696	18	54541128	F	II	WDR7	Body	opensea	Body-opens		rs5352012	9;12
cg2324402	-0.14716	0.197513	-2.71022	0.015861	0.224896	-4.29779	0.123934	0.271091	0.147157	8	1.44E+08	F	I		IGR	opensea	IGR-opense		rs1434202	29;30;46
cg0037821	-0.10419	0.463424	-2.70666	0.015975	0.224896	-4.30471	0.411328	0.515519	0.104191	8	1237075	F	II		IGR	opensea	IGR-opense		rs1121788	0;7;39;41;43;48
cg2266890	-0.10355	0.586223	-2.7041	0.016058	0.224896	-4.30968	0.534446	0.638	0.103554	11	1.28E+08	F	II		IGR	opensea	IGR-opense		rs5641782	16;36;48
cg1897267	-0.10693	0.769404	-4.2948	0.000606	0.224708	-1.05934	0.715938	0.82287	0.106932	22	40784025	R	II	SGSM3	5'UTR	opensea	5'UTR-open			
cg2637251	-0.1067	0.339099	-4.00431	0.001099	0.224708	-1.66078	0.285748	0.39245	0.106702	1	36039159	F	II	TFAP2E	5'UTR	island	5'UTR-islan	chr1:3603	rs3766540	4;17;32
cg2618008	-0.10583	0.318239	-2.69688	0.016294	0.224896	-4.32371	0.265323	0.371156	0.105833	14	76734550	R	II		IGR	opensea	IGR-opense		rs1122169	1;1
cg2090447	-0.10503	0.55486	-2.69652	0.016306	0.224896	-4.3244	0.502346	0.607375	0.105028	8	61823027	F	II		IGR	island	IGR-island	chr8:6182		
cg0908199	-0.1067	0.569497	-3.24578	0.005288	0.224708	-3.22749	0.516147	0.622847	0.1067	3	1.19E+08	F	II	CDGAP	TSS1500	shore	TSS1500-sh	chr3:1190	rs7570768	7;8
cg0654536	-0.10667	0.603816	-2.1412	0.048665	0.253405	-5.35381	0.550482	0.65715	0.106668	7	1.11E+08	F	II	LRRN3	5'UTR	opensea	5'UTR-open		rs5378047	1
cg2350564	-0.10666	0.55362	-3.53139	0.002925	0.224708	-2.64085	0.500288	0.606951	0.106663	6	31088434	R	II	CDSN	TSS1500	opensea	TSS1500-op		rs1866306	13
cg0840804	-0.11797	0.237362	-2.68868	0.016566	0.224896	-4.3396	0.178375	0.29635	0.117974	7	11381133	F	II		IGR	opensea	IGR-opense		rs1864482	9;49
cg2491500	-0.10662	0.460601	-2.66596	0.017344	0.225046	-4.3836	0.407291	0.513911	0.10662	15	43001029	F	II	STARD9	Body	opensea	Body-opens		rs5348960	17;22;46
cg1768167	-0.10656	0.280372	-3.274	0.004988	0.224708	-3.16988	0.227091	0.333653	0.106562	11	3039964	R	II	CARS	ExonBnd	opensea	ExonBnd-op		rs5378275	13;1
cg0224957	-0.11844	0.864308	-2.68704	0.016621	0.224896	-4.34279	0.805087	0.92353	0.118443	10	52434778	R	I		IGR	island	IGR-island	chr10:5243	rs1154002	48;43;22;21;12;2
cg1086159	-0.10655	0.539795	-5.3228	7.85E-05	0.220729	1.023846	0.486521	0.593068	0.106547	1	1.12E+08	F	II	C1orf162	5'UTR	opensea	5'UTR-open			
cg2011121	-0.10652	0.759891	-2.3288	0.033888	0.236083	-5.01825	0.706632	0.81315	0.106518	17	48585264	F	II	MYCBPAP	TSS1500	shore	TSS1500-sh	chr17:4858	rs5464236	8;16;39
cg0159116	-0.10651	0.261985	-2.88122	0.011201	0.224708	-3.96188	0.208731	0.315239	0.106508	10	1.3E+08	R	II	MKI67	Body	opensea	Body-opens		rs1008239	31;3
cg0005296	-0.11037	0.620828	-2.67751	0.016944	0.224901	-4.36125	0.565646	0.676011	0.110365	2	85135823	R	II		IGR	shelf	IGR-shelf	chr2:8513	rs5436227	42;6;1
cg0796284	-0.15565	0.50863	-2.67697	0.016963	0.224901	-4.36229	0.430802	0.586457	0.155655	7	1.53E+08	R	II		IGR	opensea	IGR-opense			
cg0966410	-0.11302	0.465811	-2.67658	0.016976	0.224901	-4.36305	0.409302	0.522321	0.113019	12	6855032	R	II		IGR	shelf	IGR-shelf	chr12:685	rs1467705	34
cg2191635	-0.11788	0.757645	-2.6735	0.017082	0.224901	-4.36902	0.698704	0.816587	0.117883	21	15077562	R	I		IGR	island	IGR-island	chr21:150		
cg0891711	-0.12852	0.335835	-2.67038	0.01719	0.224959	-4.37506	0.271573	0.400097	0.128524	10	1.15E+08	R	II		IGR	opensea	IGR-opense		rs1881803	33
cg2216517	-0.1065	0.437104	-2.6796	0.016873	0.224901	-4.35721	0.383856	0.490353	0.106496	1	1.11E+08	R	II	KCNA2	TSS1500	shore	TSS1500-sh	chr1:1111	rs5466940	27;25;14;2
cg1468953	-0.10647	0.287207	-2.53896	0.022377	0.226346	-4.62682	0.233971	0.340443	0.106472	10	94450866	R	II	HHEX	Body	shore	Body-shore	chr10:944	rs5348329	26
cg0732261	-0.10632	0.311042	-2.38845	0.030151	0.232737	-4.90869	0.257882	0.364202	0.10632	3	71503426	R	II	FOXP1	5'UTR	opensea	5'UTR-open		rs1045195	49;9
cg0832091	-0.1063	0.370651	-2.87089	0.01144	0.224708	-3.98236	0.317503	0.4238	0.106297	11	48065094	F	II	PTPRJ	Body	opensea	Body-opens		rs5355307	3;20;36;40;45
cg1021540	-0.11618	0.379056	-2.66521	0.01737	0.225056	-4.38503	0.320965	0.437147	0.116182	8	1.31E+08	R	II		IGR	opensea	IGR-opense		rs5274408	26;2
cg1975710	-0.10629	0.420424	-2.26272	0.038535	0.240988	-5.13806	0.367279	0.473569	0.10629	1	6605447	R	II	NOL9	Body	opensea	Body-opens		rs1827341	1
cg0001252	-0.10627	0.166296	-3.23672	0.005388	0.224708	-3.24597	0.113162	0.219431	0.106269	15	98503952	R	I	ARRDC4	1stExon	island	1stExon-isl	chr15:985	rs1151375	43;36;34;17;13;2
cg2649179	-0.10617	0.179881	-3.15789	0.006342	0.224708	-3.40637	0.126797	0.232965	0.106168	10	1.29E+08	R	I	DOCK1	TSS200	island	TSS200-isla	chr10:128		
cg1887629	-0.10614	0.352764	-2.78111	0.013737	0.224708	-4.15935	0.299696	0.405832	0.106136	11	1.19E+08	F	II	C2CD2L	Body	opensea	Body-opens		rs5429321	28;51
cg0778140	-0.11682	0.661429	-2.64516	0.018086	0.225076	-4.42373	0.603017	0.719842	0.116825	17	19773621	R	II		IGR	shore	IGR-shore	chr17:197		
cg1630662	-0.1041	0.124868	-2.64477	0.018101	0.225076	-4.42449	0.072821	0.176916	0.104096	8	1.19E+08	R	II		IGR	opensea	IGR-opense		rs5635503	2;1
cg1055574	-0.106	0.556169	-2.29658	0.036084	0.23834	-5.07688	0.503172	0.609167	0.105996	1	25946258	R	II	MAN1C1	Body	shore	Body-shore	chr1:2594	rs1419674	27
cg0908410	-0.10594	0.760617	-2.84398	0.012086	0.224708	-4.03558	0.707646	0.813589	0.105943	17	20904603	F	II	USP22	3'UTR	opensea	3'UTR-open		rs5474760	1;12;16;18
cg0176685	-0.10276	0.105994	-2.63044	0.01863	0.225119	-4.45208	0.054612	0.157376	0.102764	6	1.71E+08	F	II		IGR	island	IGR-island	chr6:1705	rs7761221	14;18;36;38;50
cg0419216	-0.10593	0.473879	-2.30729	0.03534	0.237559	-5.05744	0.420914	0.526844	0.10593	15	64806741	R	II	ZNF609	Body	opensea	Body-opens			
cg1445861	-0.10588	0.584554	-3.23243	0.005436	0.224708	-3.25473	0.531615	0.637493	0.105879	9	8858644	R	II	PTPRD	5'UTR	island	5'UTR-islan	chr9:8857	rs1421512	40;35
cg0173043	-0.12649	0.255086	-2.62729	0.018748	0.225209	-4.45813	0.191841	0.318331	0.126489	1	18857608	R	II		IGR	opensea	IGR-opense		rs5682718	29;27;12
cg1452540	-0.10586	0.451004	-3.10196	0.007118	0.224708	-3.51967	0.398076	0.503933	0.105856	15	63570327	F	II	APH1B	Body	shore	Body-shore	chr15:635	rs5539718	1;13
cg0714308	-0.10575	0.325905	-2.82988	0.012439	0.224708	-4.06342	0.273029	0.378781	0.105752	7	70597921	F	I	WBSCR17	1stExon	island	1stExon-isl	chr7:7059	rs2021303	5;14;42;49
cg1366773	-0.10569	0.131625	-6.25147	1.39E-05	0.161665	2.806196	0.078777	0.184472	0.105695	14	1.06E+08	F	II	CRIP2	Body	island	Body-islanc	chr14:105		
cg2162654	-0.10564	0.333487	-3.07938	0.007457	0.224708	-3.56526	0.280667	0.386308	0.105641	2	10720198	R	II	NOL10	Body	opensea	Body-opens		rs3728602	41
cg2765986	-0.10555	0.383581	-3.25356	0.005203	0.224708	-3.21162	0.330806	0.436355	0.105549	17	30882177	R	II	MYO1D	Body	opensea	Body-opens		rs1924263	24;9;1
cg1419970	-0.10555	0.551042	-2.93277	0.01008	0.224708	-3.85939	0.498269	0.603815	0.105546	6	90983735	R	II	BACH2	5'UTR	opensea	5'UTR-open		rs5638636	48;3;2;1
cg0774694	-0.1055	0.235685	-2.60666	0.019542	0.225391	-4.49773	0.182934	0.288436	0.105502	3	238615	F	I	CHL1	TSS200	island	TSS200-isla	chr3:2383	rs5698015	24
cg1443316	-0.11831	0.651863	-2.60974	0.019421	0.225391	-4.49183	0.59271	0.711016	0.118307	8	28911793	R	II		IGR	opensea	IGR-opense		rs5586872	2
cg0557233	-0.10626	0.671374	-2.6077	0.019501	0.225391	-4.49574	0.618243	0.724505	0.106262	7	55506671	R	II		IGR	opensea	IGR-opense		rs5383429	29;2
cg1999545	-0.10546	0.573089	-3.91745	0.001315	0.224708	-1.84098	0.520359	0.625819	0.10546	3	30647672	F	II	TGFBR2	TSS1500	shore	TSS1500-sh	chr3:3064	rs5388513	3;6;28;45
cg1603423	-0.16922	0.579842	-2.599	0.019844	0.225474	-4.51241	0.495234	0.664451	0.169217	6	14371975	F	II		IGR	opensea	IGR-opense		rs1507866	1;17;39
cg0226914	-0.15089	0.317481	-2.59788	0.019889	0.225549	-4.51456	0.242039	0.392924	0.150885	10	1.15E+08	F	II		IGR	opensea	IGR-opense			
cg2320475	-0.11875	0.402014	-2.59716	0.019918	0.225549	-4.51592	0.342637	0.461391	0.118754	21	18376383	F	II		IGR	opensea	IGR-opense			
cg2047812	-0.10536	0.184439	-2.86716	0.011528	0.224708	-3.98975	0.131757	0.237121	0.105363	14	27067372	F	I	NOVA1	TSS1500	shore	TSS1500-sh	chr14:270		
cg2438224	-0.17092	0.585071	-2.59472	0.020015	0.225563	-4.5206	0.499611	0.670531	0.17092	15	66947171	F	II		IGR	shore	IGR-shore	chr15:669	rs7401971	15;46
cg0901743	-0.10532	0.317574	-2.37844	0.03075	0.23333	-4.92716	0.264916	0.370232	0.105316	5	16179660	F	I	MARCH11	1stExon	island	1stExon-isl	chr5:1617	rs5543882	51
cg2646678	-0.11569	0.172264	-2.58318	0.020484	0.225654	-4.54265	0.114419	0.230109	0.11569	8	1.44E+08	F	II		IGR	opensea	IGR-opense			
cg1015271	-0.10531	0.460564	-2.23036	0.041021	0.244026	-5.19611	0.407911	0.513216	0.105305	20	44994449	F	II	SLC35C2	TSS1500	shore	TSS1500-sh	chr20:449	rs3758712	0
cg0708694	-0.139	0.393595	-2.57885	0.020662	0.225654	-4.55092	0.324094	0.463096	0.139001	6	1.09E+08	R	II		IGR	opensea	IGR-opense			
cg1818221	-0.10528	0.517114	-3.5064	0.00308	0.224708	-2.69243	0.464475	0.569752	0.105277	1	1.51E+08	F	II	FAM63A	5'UTR	shelf	5'UTR-shelf	chr1:1509	rs5877230	5;19
cg2257236	-0.10527	0.589111	-2.98271	0.009098	0.224708	-3.75963	0.536476	0.641746	0.105269	5	501938	R	I	SLC9A3	Body	island	Body-islanc	chr5:5018	rs1127981	47;36;29;17;14
cg1039112	-0.10513	0.251259	-3.04267	0.008043	0.224708	-3.63925	0.198694	0.303825	0.105131	21	43319594	R	II	C2CD2	Body	opensea	Body-opens		rs1711408	40
cg2313346	-0.1051	0.47945	-2.40737	0.029049	0.231636	-4.87368	0.426899	0.532002	0.105102	12	32292908	R	II	BICD1	Body	opensea	Body-opens		rs1895771	33;30;15
cg2377498	-0.10503	0.43267	-2.85125	0.011908	0.224708	-4.02123	0.380156	0.485184	0.105029	11	1.28E+08	F	I	ETS1	Body	island	Body-islanc	chr11:128	rs4396302	25;28

cg0615392	-0.10493	0.532953	-2.74748	0.014708	0.224708	-4.22518	0.480489	0.585417	0.104928	17	78755379	F	I	RPTOR	Body	opensea	Body-opens		rs5744280	17
cg1344362	-0.10481	0.495807	-3.64645	0.002304	0.224708	-2.40294	0.443401	0.548213	0.104811	1	59043070	F	I	TACSTD2	5'UTR	island	5'UTR-islan	chr1:5904	rs5413819	35;40
cg2241792	-0.10478	0.424227	-2.95238	0.009682	0.224708	-3.82028	0.37184	0.476615	0.104775	8	1.44E+08	R	II	TOP1MT	TSS1500	shore	TSS1500-sh	chr8:1444	rs5575007	49;48;39;33;31;1
cg1475222	-0.10472	0.874418	-2.96526	0.00943	0.224708	-3.79454	0.822058	0.926777	0.104719	20	34000481	F	II	UQCC	TSS1500	opensea	TSS1500-op		rs3742555	1;49
cg1489812	-0.10464	0.417527	-2.56957	0.02105	0.225654	-4.56863	0.365209	0.469846	0.104637	15	81587493	R	II	IL16	Body	opensea	Body-opens		rs5683418	23
cg2571968	-0.10445	0.582368	-2.89203	0.010956	0.224708	-3.94044	0.530145	0.634591	0.104446	3	1E+08	F	II	ADGRG7	TSS1500	opensea	TSS1500-op		rs5610058	23;41;43
cg2027637	-0.10439	0.19167	-2.45946	0.026209	0.229328	-4.77663	0.139475	0.243866	0.104391	3	99595026	F	I	C3orf26	Body	island	Body-islanc	chr3:9959		
cg2135833	-0.18233	0.665841	-2.54067	0.022301	0.226241	-4.62359	0.574678	0.757004	0.182326	17	6558440	F	II		IGR	island	IGR-island	chr17:655	rs5357655	31
cg0548503	-0.10434	0.309838	-2.96046	0.009523	0.224708	-3.80413	0.257667	0.362008	0.104341	6	1.12E+08	R	II	TRAF3IP2	TSS200	opensea	TSS200-ope		rs5685093	39;29;20;7;2
cg0834939	-0.10431	0.685979	-3.44152	0.003524	0.224708	-2.82617	0.633825	0.738132	0.104307	18	11857605	F	II	GNAL	1stExon	opensea	1stExon-op		rs7340958	29
cg1614312	-0.1042	0.570663	-2.44622	0.026905	0.229799	-4.80139	0.51856	0.622765	0.104205	13	30929119	F	II	LINC00426	Body	opensea	Body-opens		rs1151671	0;30
cg1504495	-0.10418	0.305326	-2.32126	0.034391	0.236667	-5.03201	0.253237	0.357414	0.104177	11	30038672	F	I	KCNA4	TSS200	island	TSS200-isla	chr11:300	rs5386345	43
cg1103383	-0.10412	0.235777	-2.32607	0.03407	0.236372	-5.02324	0.183716	0.287839	0.104123	3	77087860	F	I	ROBO2	TSS1500	shore	TSS1500-sh	chr3:7708	rs5517502	3;24
cg2410456	-0.10407	0.313769	-2.72892	0.015271	0.224793	-4.26139	0.261736	0.365803	0.104067	17	40932508	R	I	WNK4	TSS200	island	TSS200-isla	chr17:409	rs5388144	37;3
cg0705206	-0.10402	0.328659	-2.47447	0.025441	0.228719	-4.7485	0.27665	0.380668	0.104018	10	99255236	R	II	MMS19	Body	shelf	Body-shelf	chr10:992	rs2900126	29
cg0203816	-0.11762	0.139854	-2.52436	0.023038	0.226614	-4.65448	0.081044	0.198665	0.117621	22	39784481	R	I		IGR	island	IGR-island	chr22:397	rs5471784	19
cg1054514	-0.10393	0.382435	-2.48211	0.025058	0.228362	-4.73416	0.330467	0.434402	0.103934	19	42070902	F	I	CEACAM21	5'UTR	opensea	5'UTR-open		rs5530406	0;47
cg1042085	-0.15218	0.448225	-2.51741	0.02336	0.226902	-4.66763	0.372133	0.524317	0.152184	6	41207397	R	II		IGR	opensea	IGR-opense		rs5581566	37
cg0863389	-0.10297	0.546793	-2.5171	0.023374	0.226911	-4.66821	0.495307	0.598279	0.102973	13	1E+08	R	II		IGR	opensea	IGR-opense		rs5704046	3;2
cg0007619	-0.10886	0.243356	-2.5157	0.023439	0.22692	-4.67087	0.188928	0.297784	0.108857	5	1.41E+08	R	I		IGR	shore	IGR-shore	chr5:1408	rs251022;r	36;28;2
cg0536430	-0.10801	0.350856	-2.51567	0.023441	0.22692	-4.67093	0.29685	0.404862	0.108012	10	1.04E+08	R	II		IGR	island	IGR-island	chr10:103	rs5308336	8
cg0031221	-0.10393	0.853538	-4.44272	0.000449	0.224708	-0.75446	0.801573	0.905504	0.103931	5	96063813	R	II	CAST	Body	opensea	Body-opens		rs5559708	42;27;5;2
cg0524146	-0.10388	0.154666	-3.16216	0.006286	0.224708	-3.39769	0.102725	0.206606	0.103881	19	22816980	R	I	ZNF492	TSS200	shore	TSS200-shc	chr19:228	rs5658552	51
cg0021729	-0.10386	0.543071	-2.99615	0.00885	0.224708	-3.73271	0.491143	0.594999	0.103856	15	50200296	F	II	ATP8B4	Body	opensea	Body-opens		rs1907856	25;42;51
cg0333049	-0.10381	0.355401	-3.05064	0.007912	0.224708	-3.62322	0.303497	0.407304	0.103807	10	94452236	F	I	HHEX	Body	island	Body-islanc	chr10:944	rs5338245	1;7
cg1506001	-0.10379	0.366271	-2.26036	0.038711	0.241192	-5.14231	0.314377	0.418166	0.103789	19	58446783	F	II	ZNF418	TSS200	island	TSS200-isla	chr19:584	rs5753763	5;50
cg1389669	-0.10365	0.694432	-2.55231	0.021789	0.225834	-4.60148	0.642607	0.746257	0.10365	5	13770231	F	II	DNAH5	Body	opensea	Body-opens		rs1905399	1;7;10;14;17;19
cg0983797	-0.10363	0.574155	-2.50568	0.023911	0.22731	-4.68978	0.522339	0.625971	0.103632	7	1.11E+08	R	II	LRRN3	5'UTR	opensea	5'UTR-open		rs5538823	49
cg0990750	-0.1036	0.436537	-2.4154	0.028593	0.231218	-4.85877	0.384739	0.488336	0.103597	13	37248244	F	I	C13orf36	5'UTR	island	5'UTR-islan	chr13:372		
cg0886471	-0.10346	0.590625	-2.4833	0.024999	0.228308	-4.73192	0.538897	0.642353	0.103455	14	69436506	R	II	ACTN1	Body	opensea	Body-opens		rs1399096	51
cg1695737	-0.10133	0.359824	-2.49317	0.024514	0.227821	-4.71336	0.309161	0.410488	0.101327	8	42596672	R	II		IGR	opensea	IGR-opense		rs1898945	28;3;1
cg0629310	-0.10343	0.272348	-2.28655	0.036794	0.239116	-5.09504	0.220633	0.324063	0.10343	13	1.1E+08	F	II	IRS2	1stExon	island	1stExon-isl	chr13:110		
cg0875045	-0.17703	0.517313	-2.48883	0.024726	0.228039	-4.72153	0.428795	0.60583	0.177034	17	6558815	R	II		IGR	shore	IGR-shore	chr17:655	rs8078913	49
cg1487517	-0.10328	0.403533	-2.18639	0.04464	0.248546	-5.2743	0.351891	0.455175	0.103283	2	50574196	R	I	NRXN1	Body	island	Body-islanc	chr2:5057	rs1898733	15
cg0519107	-0.10327	0.252541	-2.45828	0.026271	0.22938	-4.77885	0.200908	0.304174	0.103266	4	66536186	R	I	EPHA5	TSS1500	island	TSS1500-isl	chr4:6653		
cg1053088	-0.10319	0.308492	-2.35371	0.032278	0.234548	-4.97266	0.256899	0.360085	0.103186	5	3596207	F	I	IRX1	1stExon	island	1stExon-isl	chr5:3594	rs5527259	28
cg2262022	-0.10314	0.355685	-2.62362	0.018887	0.225339	-4.46518	0.304112	0.407257	0.103145	7	1.54E+08	F	I	DPP6	TSS200	island	TSS200-isla	chr7:1535	rs5501179	5;6;51
cg0299613	-0.10312	0.410375	-5.54349	5.15E-05	0.216493	1.457176	0.358817	0.461933	0.103116	6	1.53E+08	R	II	SYNE1	TSS1500	shore	TSS1500-sh	chr6:1529		
cg0649596	-0.1031	0.487425	-2.94546	0.009821	0.224708	-3.83408	0.435873	0.538977	0.103104	7	1.54E+08	R	II	DPP6	1stExon	island	1stExon-isl	chr7:1535	rs5662032	21
cg1418003	-0.10303	0.295083	-4.3436	0.000549	0.224708	-0.95863	0.243569	0.346597	0.103028	9	1.23E+08	R	II	MEGF9	Body	shore	Body-shore	chr9:1234	rs5291646	46
cg1883214	-0.10299	0.484365	-2.62849	0.018703	0.225205	-4.45582	0.432869	0.535862	0.102994	3	1.02E+08	F	II	LOC15222	TSS200	opensea	TSS200-ope		rs1511578	0;1;14;23;24
cg1893272	-0.10296	0.229952	-2.71515	0.015703	0.224896	-4.28819	0.17847	0.281434	0.102964	10	1.29E+08	R	I	DOCK1	Body	island	Body-islanc	chr10:128	rs5353975	51
cg2128100	-0.10295	0.367107	-2.54288	0.022203	0.226158	-4.6194	0.315632	0.418583	0.102951	18	14748298	R	I	ANKRD30B	1stExon	shore	1stExon-sh	chr18:147	rs1152202	45;35;22;1
cg1031315	-0.12762	0.850876	-2.46202	0.026077	0.229196	-4.77185	0.787067	0.914685	0.127618	2	1.07E+08	F	II		IGR	shelf	IGR-shelf	chr2:1068	rs3721609	18;29
cg2626687	-0.10291	0.695671	-3.27632	0.004964	0.224708	-3.16514	0.644218	0.747124	0.102906	3	23957486	R	II	RPL15	TSS1500	shore	TSS1500-sh	chr3:2395	rs1885726	27;18
cg1159250	-0.1027	0.296847	-2.54069	0.0223	0.226241	-4.62355	0.245499	0.348195	0.102696	7	1.46E+08	F	I	CNTNAP2	TSS200	island	TSS200-isla	chr7:1458	rs5608750	13;20
cg0100018	-0.10266	0.662757	-2.98699	0.009018	0.224708	-3.75106	0.611425	0.71409	0.102664	9	1.34E+08	F	II	QRFP	1stExon	opensea	1stExon-op		rs1426949	23;41;44
cg0183267	-0.10264	0.635742	-2.73878	0.014969	0.224747	-4.24216	0.58442	0.687063	0.102643	12	1.23E+08	F	II	VPS37B	Body	opensea	Body-opens		rs5407435	49
cg2187740	-0.10264	0.542116	-2.55444	0.021696	0.225763	-4.59742	0.490796	0.593436	0.10264	1	6605464	F	II	NOL9	Body	opensea	Body-opens		rs5304333	14;33;45
cg0372669	-0.10995	0.649742	-2.44583	0.026926	0.229816	-4.80211	0.594769	0.704714	0.109945	19	58315178	R	II		IGR	opensea	IGR-opense			
cg2271576	-0.10262	0.607322	-2.52274	0.023113	0.22668	-4.65755	0.556009	0.658634	0.102625	1	10556294	R	II	PEX14	Body	opensea	Body-opens			
cg0124585	-0.10262	0.543344	-2.51274	0.023578	0.226934	-4.67645	0.492036	0.594652	0.102616	6	90894791	F	II	BACH2	5'UTR	opensea	5'UTR-open		rs5469890	1;9;40;49
cg2675442	-0.10262	0.182843	-3.35115	0.004251	0.224708	-3.0119	0.131535	0.23415	0.102615	3	1.13E+08	R	II	CD200R1	TSS200	opensea	TSS200-ope			
cg2280758	-0.11785	0.213932	-2.42798	0.027892	0.230634	-4.83538	0.155009	0.272855	0.117846	5	53564	R	I		IGR	island	IGR-island	chr5:5311	rs5510302	48;30
cg0394446	-0.10979	0.39281	-2.42653	0.027972	0.230684	-4.83809	0.337916	0.447704	0.109788	4	3765186	R	II		IGR	shore	IGR-shore	chr4:3767	rs5530060	37;30
cg0462411	-0.10257	0.425166	-2.64122	0.01823	0.225076	-4.43132	0.373882	0.47645	0.102568	20	13976093	R	I	MACROD2	TSS200	island	TSS200-isla	chr20:139	rs3701202	16
cg0066794	-0.11143	0.857421	-2.415	0.028616	0.231224	-4.85952	0.801707	0.913134	0.111427	13	1.01E+08	F	II		IGR	shore	IGR-shore	chr13:100	rs1489892	0
cg2707311	-0.10254	0.663975	-3.18534	0.005992	0.224708	-3.3506	0.612703	0.715246	0.102544	16	56228744	R	II	DKFZP434I	TSS1500	shelf	TSS1500-sh	chr16:562		
cg1891920	-0.10253	0.542161	-2.49265	0.024539	0.227869	-4.71434	0.490897	0.593424	0.102527	11	96012039	F	II	MAML2	Body	opensea	Body-opens			
cg0020827	-0.10253	0.648783	-2.96442	0.009446	0.224708	-3.79621	0.59752	0.700047	0.102527	7	4779342	R	II	FOXK1	Body	opensea	Body-opens		rs1840296	40;13;2;1
cg2245623	-0.11063	0.288541	-2.39116	0.029991	0.232589	-4.90368	0.233225	0.343856	0.11063	13	48718766	R	II		IGR	opensea	IGR-opense		rs5308410	28;22;20;1
cg1780641	-0.10245	0.463441	-2.20309	0.043232	0.246746	-5.24469	0.412217	0.514666	0.102449	3	18459963	F	II	SATB1	Body	opensea	Body-opens		rs1461314	38
cg1853962	-0.10239	0.573294	-3.57385	0.002678	0.224708	-2.55312	0.522099	0.624488	0.102389	17	10051407	F	II	GAS7	Body	opensea	Body-opens		rs7530779	0;45
cg0537402	-0.10234	0.693248	-2.78462	0.013639	0.224708	-4.15246	0.642076	0.74442	0.102345	19	55549746	F	II	GP6	TSS200	opensea	TSS200-ope		rs1654431	12;14

cg0339399	-0.19415	0.234961	-2.38738	0.030214	0.232799	-4.91066	0.137887	0.332034	0.194147	4	1580377	F	I		IGR	island	IGR-island	chr4:1580	rs5342840	31;32
cg1674560	-0.10224	0.632671	-3.3384	0.004365	0.224708	-3.03805	0.581551	0.683792	0.102241	2	2.02E+08	F	II	CASP10	TSS200	opensea	TSS200-ope		rs7142428	9;51
cg2040485	-0.10222	0.703342	-4.9111	0.000176	0.224708	0.200931	0.652231	0.754452	0.102221	9	99374660	F	II	CDC14B	Body	opensea	Body-opens		rs5682216	0;37
cg1401905	-0.10217	0.756692	-2.88828	0.011041	0.224708	-3.94788	0.705607	0.807776	0.102168	9	1.08E+08	F	II	ABCA1	TSS1500	island	TSS1500-isl	chr9:10768	rs5289522	14;39
cg0660208	-0.10215	0.329907	-2.35296	0.032325	0.234567	-4.97403	0.27883	0.380984	0.102155	16	1583883	R	I	IFT140	Body	island	Body-island	chr16:1583	rs5379185	51;10;8
cg0876793	-0.10213	0.56377	-2.37354	0.031047	0.233536	-4.93619	0.512703	0.614837	0.102134	7	25989524	F	II		IGR	shore	IGR-shore	chr7:2599		
cg2602301	-0.10207	0.266986	-2.28802	0.03669	0.239006	-5.09239	0.215952	0.31802	0.102069	21	31311859	F	I	GRIK1	1stExon	island	1stExon-isl	chr21:3131	rs1865063	0
cg1629876	-0.10207	0.608353	-2.53713	0.022459	0.226384	-4.6303	0.55732	0.659386	0.102065	22	44445889	R	II	PARVB	Body	opensea	Body-opens			
cg1167053	-0.10202	0.533736	-2.36597	0.031511	0.234003	-4.95013	0.482727	0.584745	0.102018	3	71421949	F	II	FOXP1	5'UTR	opensea	5'UTR-open		rs1893613	0;12
cg1081928	-0.10199	0.68926	-2.68739	0.01661	0.224896	-4.34211	0.638267	0.740253	0.101986	22	39810539	F	II	TAB1	Body	opensea	Body-opens		rs5721770	7;11;21;31
cg2272972	-0.10197	0.400875	-6.33631	1.19E-05	0.161665	2.963444	0.349892	0.451858	0.101966	1	3123854	F	II	PRDM16	Body	opensea	Body-opens		rs1932886	1;9;46
cg1114669	-0.10195	0.560307	-3.47381	0.003296	0.224708	-2.75964	0.50933	0.611284	0.101955	12	47219737	F	I	SLC38A4	1stExon	opensea	1stExon-op		rs5591118	1;2
cg0126107	-0.10195	0.259237	-2.48202	0.025062	0.228362	-4.73432	0.208261	0.310213	0.101952	4	1.88E+08	F	II	FAT1	TSS200	island	TSS200-isla	chr4:18764	rs2276929	9;46
cg1351882	-0.1019	0.55873	-2.78853	0.013531	0.224708	-4.14479	0.507778	0.609681	0.101903	1	8523537	F	II	RERE	Body	opensea	Body-opens		rs7404964	14
cg1574115	-0.11115	0.472525	-2.364	0.031633	0.234051	-4.95375	0.416948	0.528103	0.111154	1	1.11E+08	F	II		IGR	shelf	IGR-shelf	chr1:1106	rs5574573	14;21;24;35
cg2439297	-0.10188	0.5554	-4.20582	0.000727	0.224708	-1.24328	0.504459	0.606341	0.101882	4	74089169	F	II	ANKRD17	TSS1500	opensea	TSS1500-op		rs5762947	3;37
cg1038755	-0.10179	0.224987	-2.36117	0.031809	0.234211	-4.95895	0.174095	0.27588	0.101786	11	30038615	R	I	KCNA4	TSS200	island	TSS200-isla	chr11:3003		
cg1568569	-0.10178	0.285026	-2.92014	0.010344	0.224708	-3.88456	0.234137	0.335916	0.101779	7	70597351	F	I	WBSCR17	TSS200	island	TSS200-isla	chr7:7059	rs1023496	14;16
cg0072897	-0.10176	0.472875	-2.7852	0.013623	0.224708	-4.15133	0.421993	0.523757	0.101763	17	37036448	F	II	LASP1	Body	opensea	Body-opens		rs226213	40
cg0316052	-0.10171	0.438376	-3.26537	0.005078	0.224708	-3.18751	0.38752	0.489231	0.101711	17	80928410	R	I	B3GNTL1	Body	shore	Body-shore	chr17:809	rs7337286	16;12;3;1
cg0073566	-0.10391	0.300918	-2.3509	0.032456	0.234639	-4.97781	0.248962	0.352873	0.103911	3	74663796	F	I		IGR	island	IGR-island	chr3:7466	rs5459523	7;8;49
cg2197153	-0.10169	0.429659	-3.76049	0.001819	0.224708	-2.16662	0.378815	0.480504	0.10169	14	39734390	F	II	CTAGE5	TSS1500	shore	TSS1500-sh	chr14:397	rs5587431	1
cg1308842	-0.10165	0.54956	-2.50262	0.024057	0.227443	-4.69555	0.498732	0.600387	0.101654	11	940618	R	II	AP2A2	Body	shelf	Body-shelf	chr11:943	rs5643884	45;12;8;1
cg2385017	-0.10526	0.446222	-2.33971	0.033174	0.23536	-4.9983	0.393594	0.49885	0.105256	10	22542726	F	II		IGR	island	IGR-island	chr10:2254	rs1896988	34
cg0864199	-0.10162	0.390003	-2.63032	0.018635	0.225119	-4.45232	0.339194	0.440812	0.101618	1	54822503	F	II	SSBP3	Body	shore	Body-shore	chr1:5482		
cg2659997	-0.1016	0.610409	-2.43647	0.027429	0.230075	-4.81958	0.55961	0.661208	0.101598	9	1.28E+08	F	II	SCAI	Body	opensea	Body-opens		rs5598086	0;5;7;44
cg2591894	-0.1016	0.839985	-4.00908	0.001088	0.224708	-1.65088	0.789187	0.890784	0.101597	17	41365094	F	II	TMEM106A	Body	shore	Body-shore	chr17:413	rs1505008	1;37
cg2268756	-0.12074	0.778614	-2.33163	0.033701	0.235831	-5.01307	0.718246	0.838982	0.120736	19	36439299	F	II		IGR	shelf	IGR-shelf	chr19:364	rs1401404	0;19;30
cg1907857	-0.10159	0.289638	-2.35231	0.032366	0.234603	-4.97523	0.238844	0.340432	0.101588	5	17217877	F	I	BASP1	5'UTR	island	5'UTR-islan	chr5:1721	rs1476216	22
cg0974744	-0.10158	0.462437	-2.76237	0.01427	0.224708	-4.19605	0.411646	0.513228	0.101582	15	70387268	R	II	TLE3	Body	shore	Body-shore	chr15:703	rs5290753	46;45;42;37;36;23;11;10;8
cg0733011	-0.10158	0.52786	-2.34379	0.03291	0.235122	-4.99083	0.477071	0.578649	0.101578	3	11624023	F	II	VGLL4	Body	opensea	Body-opens		rs1928821	38;41
cg0982776	-0.23264	0.335559	-2.32238	0.034316	0.236584	-5.02996	0.219237	0.451882	0.232645	15	66947392	F	I		IGR	island	IGR-island	chr15:669		
cg2577101	-0.10157	0.351373	-2.74698	0.014723	0.224708	-4.22616	0.300586	0.40216	0.101574	7	25989735	F	II	MIR148A	TSS200	shore	TSS200-sho	chr7:2599		
cg1627354	-0.10795	0.678641	-2.31932	0.034521	0.236777	-5.03554	0.624667	0.732614	0.107947	10	1.23E+08	R	II		IGR	opensea	IGR-opense		rs5365527	1
cg2543194	-0.10157	0.67454	-2.37796	0.030779	0.23334	-4.92806	0.623755	0.725324	0.101569	4	40313893	R	II	LOC10106	Body	opensea	Body-opens		rs5279890	11
cg0577132	-0.10155	0.412036	-4.20677	0.000725	0.224708	-1.24131	0.361262	0.462811	0.101549	3	1.71E+08	R	II	PLD1	Body	opensea	Body-opens			
cg1372576	-0.10148	0.587899	-2.53225	0.022679	0.226517	-4.63955	0.537158	0.63864	0.101482	1	1.83E+08	R	II	NPL	5'UTR	opensea	5'UTR-open		rs7306535	38;16
cg0311797	-0.10148	0.174543	-2.53569	0.022524	0.226391	-4.63302	0.123805	0.225282	0.101477	18	11149435	F	I	FAM38B	TSS1500	island	TSS1500-isl	chr18:111	rs5724852	51
cg0758600	-0.10146	0.480827	-5.61381	4.51E-05	0.208563	1.594024	0.430096	0.531559	0.101463	19	49636535	F	I	PPFIA3	Body	island	Body-island	chr19:496	rs5524649	29
cg0460299	-0.10146	0.330453	-2.36433	0.031613	0.234049	-4.95315	0.279725	0.381182	0.101457	17	62009628	R	II	CD79B	1stExon	opensea	1stExon-op		rs1999304	23;2;1
cg1872340	-0.10145	0.534501	-2.40235	0.029338	0.231819	-4.88298	0.483774	0.585228	0.101454	11	1911547	R	I	LSP1	3'UTR	opensea	3'UTR-open		rs5656858	33
cg2055832	-0.10393	0.455145	-2.30584	0.03544	0.237641	-5.06007	0.403182	0.507108	0.103926	2	2.35E+08	R	I		IGR	opensea	IGR-opense		rs5308705	37;34;32;22;1
cg1809698	-0.1014	0.602709	-2.773	0.013965	0.224708	-4.17525	0.552008	0.65341	0.101402	3	11623873	R	II	VGLL4	Body	opensea	Body-opens		rs5475545	29;6;2
cg2268199	-0.10139	0.559575	-5.47992	5.81E-05	0.219272	1.332945	0.508879	0.610271	0.101393	2	65131547	R	II	LOC40095	Body	opensea	Body-opens		rs1302790	38;36;35;26;5
cg2570276	-0.10401	0.816721	-2.29907	0.03591	0.238096	-5.07235	0.764717	0.868725	0.104008	12	69605100	F	II		IGR	opensea	IGR-opense		rs3586899	0;12
cg1732871	-0.10137	0.541327	-2.76715	0.014132	0.224708	-4.1867	0.490641	0.592012	0.101372	7	56516243	R	II	LOC65022	TSS200	shore	TSS200-sho	chr7:5651	rs5433559	25;22;3
cg1423760	-0.10132	0.557143	-2.74874	0.01467	0.224708	-4.22271	0.506481	0.607805	0.101324	3	1.79E+08	F	II	MFN1	TSS1500	shore	TSS1500-sh	chr3:1790	rs1386525	8;27
cg0375333	-0.10123	0.207287	-2.728	0.0153	0.224807	-4.26318	0.156672	0.257903	0.101231	2	1.16E+08	R	I	DPP10	TSS1500	island	TSS1500-isl	chr2:1159		
cg2243531	-0.10122	0.286405	-2.31494	0.034817	0.237078	-5.04352	0.235793	0.337017	0.101224	17	64669739	F	II	PRKCA	Body	opensea	Body-opens		rs1502969	2
cg2426816	-0.10122	0.660891	-2.42893	0.02784	0.230593	-4.83363	0.610281	0.7115	0.101219	22	39747459	F	II	SYNGR1	Body	shore	Body-shore	chr22:397	rs1896786	0;23
cg1217116	-0.10122	0.592658	-2.87985	0.011232	0.224708	-3.96459	0.542049	0.643268	0.101218	9	1.27E+08	F	II	DENND1A	Body	opensea	Body-opens		rs5665526	21
cg2641550	-0.10119	0.520823	-3.4825	0.003237	0.224708	-2.74174	0.470226	0.57142	0.101194	21	46344680	F	II	ITGB2-AS1	Body	opensea	Body-opens		rs5394812	0;8;48
cg0049065	-0.10117	0.273364	-2.53171	0.022703	0.226534	-4.64057	0.22278	0.323949	0.101169	2	1.65E+08	R	I	FIGN	TSS1500	shore	TSS1500-sh	chr2:1645	rs5630044	7;6
cg2609983	-0.17858	0.387698	-2.25925	0.038795	0.241232	-5.14431	0.29841	0.476986	0.178576	15	66947568	R	I		IGR	island	IGR-island	chr15:669	rs5357947	27
cg1617034	-0.10113	0.667234	-4.04915	0.001002	0.224708	-1.56781	0.616671	0.717798	0.101127	19	2424006	R	II	TMPRSS9	Body	island	Body-island	chr19:242	rs5522450	23;2
cg0599741	-0.10111	0.349145	-3.70697	0.002032	0.224708	-2.27757	0.298591	0.399698	0.101107	2	2.4E+08	F	II	HDAC4	Body	shore	Body-shore	chr2:2401	rs1908550	23;46
cg0016935	-0.11488	0.518317	-2.25235	0.039316	0.241781	-5.15671	0.460879	0.575754	0.114876	20	54922827	R	II		IGR	shelf	IGR-shelf	chr20:549	rs5278543	21
cg0288186	-0.10107	0.481541	-2.61439	0.019241	0.225391	-4.48291	0.431007	0.532076	0.101069	17	999650	R	I	ABR	Body	opensea	Body-opens		rs2257532	25;24;2
cg0851026	-0.10106	0.190733	-2.36776	0.031401	0.233871	-4.94684	0.1402	0.241265	0.101064	13	1.1E+08	F	I	IRS2	1stExon	island	1stExon-isl	chr13:110	rs5328102	29
cg0453658	-0.10876	0.539018	-2.24541	0.039847	0.242456	-5.16917	0.484636	0.5934	0.108764	10	6919371	R	II		IGR	opensea	IGR-opense		rs5777894	42;29
cg1026119	-0.10106	0.375545	-3.22973	0.005466	0.224708	-3.26024	0.325016	0.426074	0.101058	11	1.25E+08	F	II	FEZ1	5'UTR	shore	5'UTR-shor	chr11:125	rs1478858	2;13;37
cg1304622	-0.10044	0.335268	-2.24288	0.040042	0.242678	-5.1737	0.285047	0.385489	0.100443	12	1.31E+08	F	II		IGR	opensea	IGR-opense		rs5440711	7;27
cg1665524	-0.10102	0.232746	-3.50448	0.003093	0.224708	-2.69639	0.182236	0.283256	0.101019	20	32857072	R	I	ASIP	3'UTR	island	3'UTR-islan	chr20:328	rs5653446	32;14
cg2284135	-0.10095	0.256631	-3.12682	0.006762	0.224708	-3.46936	0.206154	0.307108	0.100954	1	1.84E+08	F	II	RGL1	Body	opensea	Body-opens		rs1833821	48;51

cg1680442	-0.10091	0.659924	-2.69387	0.016394	0.224896	-4.32954	0.60947	0.710378	0.100908	3	71367137	F	II	FOXP1	5'UTR	opensea	5'UTR-open		rs5576592	0
cg0939670	-0.10088	0.227507	-2.76339	0.01424	0.224708	-4.19406	0.177066	0.277947	0.100881	1	2.03E+08	F	II	PPFIA4	Body	island	Body-islanc	chr1:2030	rs5347469	4;7
cg1124737	-0.21177	0.249097	-2.22943	0.041095	0.244107	-5.19778	0.143214	0.354981	0.211767	22	39784982	F	II		IGR	island	IGR-island	chr22:397	rs5640351	3;44
cg1542788	-0.11188	0.295459	-2.22924	0.04111	0.244112	-5.19811	0.23952	0.351398	0.111878	8	55379663	F	I		IGR	island	IGR-island	chr8:5537	rs5671180	35
cg2479517	-0.1008	0.788358	-4.75117	0.000241	0.224708	-0.12328	0.73796	0.838756	0.100796	10	1.09E+08	R	II	SORCS1	Body	opensea	Body-opens		rs5591673	51;31;1
cg1670366	-0.11962	0.585994	-2.2252	0.041432	0.244509	-5.20533	0.526184	0.645804	0.119619	6	41207506	F	II		IGR	opensea	IGR-opense			
cg0495730	-0.10775	0.545873	-2.20941	0.04271	0.246052	-5.23346	0.491998	0.599748	0.107749	12	92180808	F	II		IGR	opensea	IGR-opense		rs1484999	1;7
cg0389283	-0.10076	0.682347	-5.9793	2.28E-05	0.186757	2.295287	0.63197	0.732725	0.100755	2	2.39E+08	R	I	LRRFIP1	TSS1500	shore	TSS1500-sh	chr2:2385	rs5615905	44;14
cg0609853	-0.10074	0.404552	-2.20472	0.043096	0.246579	-5.2418	0.354182	0.454922	0.10074	10	76727919	F	II	MYST4	Body	opensea	Body-opens		rs5444744	0;1;11;22
cg2371234	-0.10071	0.291816	-2.24139	0.040157	0.242872	-5.17637	0.241461	0.342172	0.10071	10	60936794	F	I	PHYHIPL	Body	island	Body-islanc	chr10:609	rs3763659	16;37
cg0659883	-0.10175	0.211943	-2.19994	0.043494	0.24707	-5.25029	0.161069	0.262817	0.101749	3	2140699	R	I		IGR	island	IGR-island	chr3:2140	rs5705063	33
cg2439971	-0.15053	0.172752	-2.19617	0.043811	0.247395	-5.25699	0.097485	0.248019	0.150534	22	39784796	R	II		IGR	island	IGR-island	chr22:397		
cg1765030	-0.10063	0.593616	-2.54663	0.022037	0.225993	-4.61226	0.543301	0.64393	0.100629	3	13001450	F	II	IQSEC1	Body	opensea	Body-opens		rs5736424	45
cg1535231	-0.10063	0.50906	-3.6037	0.002517	0.224708	-2.49141	0.458746	0.559375	0.100629	2	1.61E+08	R	II	CD302	TSS1500	shore	TSS1500-sh	chr2:1606	rs1883371	18
cg1461310	-0.12645	0.711273	-2.18682	0.044603	0.248479	-5.27353	0.648049	0.774497	0.126448	9	1.08E+08	F	II		IGR	opensea	IGR-opense		rs7274032	0;1;8;18;38;42
cg2522328	-0.10574	0.411348	-2.18644	0.044636	0.248546	-5.27422	0.358477	0.464219	0.105742	2	1.28E+08	R	II		IGR	island	IGR-island	chr2:1284		
cg0243412	-0.1006	0.476259	-3.23745	0.00538	0.224708	-3.24449	0.425958	0.526561	0.100604	7	4901798	R	I	PAPOLB	TSS200	shore	TSS200-sho	chr7:4901	rs2007521	47;42
cg0690647	-0.1005	0.211203	-2.39012	0.030052	0.232664	-4.9056	0.160953	0.261452	0.100499	2	1.08E+08	R	I	ST6GAL2	TSS200	island	TSS200-isla	chr2:1075	rs5603966	42
cg1071786	-0.10048	0.554847	-2.83123	0.012405	0.224708	-4.06077	0.504606	0.605089	0.100484	1	2.06E+08	R	II	SLC41A1	5'UTR	shore	5'UTR-shor	chr1:2057		
cg2548115	-0.14404	0.495176	-2.17787	0.045374	0.249398	-5.28935	0.423156	0.567197	0.144041	15	66947564	R	II		IGR	island	IGR-island	chr15:669	rs5357947	23
cg0430350	-0.10048	0.598434	-3.69949	0.002064	0.224708	-2.29307	0.548193	0.648675	0.100482	19	35453690	F	II	ZNF792	Body	shore	Body-shore	chr19:354	rs5774584	3;50
cg0754052	-0.10038	0.203225	-2.73033	0.015228	0.224792	-4.25864	0.153034	0.253417	0.100383	1	14925170	R	I	KAZN	TSS200	island	TSS200-isla	chr1:1492		
cg0719976	-0.15405	0.684356	-2.16654	0.046368	0.250588	-5.30933	0.607333	0.76138	0.154046	1	5347468	R	II		IGR	opensea	IGR-opense		rs565916;r	44;31;29;13;1
cg0680633	-0.10028	0.700361	-3.676	0.002167	0.224708	-2.34174	0.65022	0.750502	0.100282	9	20508649	R	II	MLLT3	Body	opensea	Body-opens		rs1477608	29
cg0906065	-0.10023	0.620245	-4.81242	0.000213	0.224708	0.001166	0.570129	0.670361	0.100232	10	90985062	R	II	LIPA	Body	opensea	Body-opens		rs5334254	2
cg2410619	-0.10016	0.565867	-2.91659	0.010419	0.224708	-3.89161	0.515787	0.615946	0.100158	19	38422618	F	II	SIPA1L3	5'UTR	opensea	5'UTR-open		rs5294120	8;21;43
cg0305261	-0.10016	0.529104	-2.31669	0.034698	0.236945	-5.04033	0.479025	0.579183	0.100158	17	17410416	F	II	PEMT	Body	opensea	Body-opens		rs5394903	2;30;32;36
cg2660963	-0.10012	0.235186	-2.3043	0.035547	0.237702	-5.06287	0.185126	0.285246	0.10012	13	28366814	R	I	GSX1	5'UTR	island	5'UTR-islan	chr13:283		

SUPPLEMENTAL TABLE 2 - FEMALE HYPO

	logFC	AveExpr	t	P.Value	adj.P.Val	B	Normal_f_A	LBP_f_AVG	deltaBeta	CHR	MAPINFO	Strand	Type	gene	feature	cgi	feat.cgi	UCSC_Islan	SNP_ID	SNP_DISTANCE
cg1259227	0.153449	0.68634	7.54499	1.51E-06	0.133374	5.097468	0.763065	0.609615	-0.15345	12	3427813	R	II		IGR	opensea	IGR-opense		rs7299862	35;2
cg2665477	0.274619	0.385845	3.036448	0.008146	0.224708	-3.65178	0.523154	0.248535	-0.27462	12	740100	F	II	NINJ2	Body	opensea	Body-opens		rs5398488	15
cg1784632	0.101997	0.429045	6.061821	1.96E-05	0.180489	2.451235	0.480044	0.378047	-0.102	2	79168411	R	II		IGR	opensea	IGR-opense			
cg2575542	0.261975	0.445129	2.610006	0.019411	0.225391	-4.49132	0.576116	0.314141	-0.26197	19	13875111	R	I	MRI1	TSS1500	island	TSS1500-isl	chr19:1387	rs5404156	49;18
cg2007591	0.125867	0.614368	5.749773	3.49E-05	0.202189	1.856885	0.677301	0.551434	-0.12587	5	6837863	F	II		IGR	opensea	IGR-opense		rs1121560	0;10;51
cg0879814	0.259916	0.616842	2.368031	0.031384	0.233855	-4.94634	0.746801	0.486884	-0.25992	4	36305221	R	II	DTHD1	Body	opensea	Body-opens		rs3507478	1;1
cg1290159	0.119401	0.37881	5.585301	4.76E-05	0.20957	1.538619	0.438511	0.31911	-0.1194	11	44527562	R	II		IGR	opensea	IGR-opense		rs5543129	2
cg1647469	0.254341	0.40547	2.763572	0.014235	0.224708	-4.19371	0.532641	0.278299	-0.25434	19	13875014	R	II	MRI1	TSS1500	shore	TSS1500-sh	chr19:1387	rs1464733	34;12
cg0779992	0.163406	0.624492	5.472197	5.9E-05	0.219272	1.317823	0.706196	0.542789	-0.16341	14	1.07E+08	R	II		IGR	opensea	IGR-opense		rs1013380	12;5
cg2687422	0.143923	0.58207	5.47165	5.9E-05	0.219272	1.316751	0.654031	0.510108	-0.14392	2	1.06E+08	F	I		IGR	island	IGR-island	chr2:1058	rs5722912	1;14;25
cg2360399	0.25291	0.616304	2.43989	0.027244	0.229955	-4.8132	0.742759	0.489849	-0.25291	6	1.57E+08	R	II	ARID1B	Body	opensea	Body-opens		rs5727022	38;31;1;1
cg0508466	0.249867	0.586746	2.535263	0.022543	0.226447	-4.63384	0.711679	0.461812	-0.24987	3	1.26E+08	F	II	ALG1L	5'UTR	opensea	5'UTR-open		rs1133474	3;12;40
cg2336301	0.248088	0.609507	2.183169	0.044916	0.248813	-5.27999	0.733551	0.485463	-0.24809	8	42123384	R	II	LOC10192	Body	opensea	Body-opens		rs5278849	50;39;1
cg1946972	0.103858	0.485022	5.401465	6.75E-05	0.219272	1.178962	0.536951	0.433093	-0.10386	14	1.05E+08	F	II		IGR	opensea	IGR-opense		rs7845516	8;51
cg0648999	0.247248	0.472487	3.228632	0.005479	0.224708	-3.26247	0.596111	0.348863	-0.24725	19	47082996	R	II	PPP5D1	Body	opensea	Body-opens		rs5657773	38;11
cg1800834	0.240245	0.209726	3.561087	0.00275	0.224708	-2.57951	0.329849	0.089604	-0.24024	19	1096084	R	II	POLR2E	TSS1500	shore	TSS1500-sh	chr19:109	rs5659822	46;42;25;1
cg0935251	0.233892	0.33096	2.671751	0.017143	0.224959	-4.37239	0.447906	0.214013	-0.23389	19	21265421	R	II	ZNF714	5'UTR	island	5'UTR-islan	chr19:212	rs8101412	50
cg2696871	0.109648	0.654195	5.234195	9.32E-05	0.223139	0.848275	0.709019	0.599371	-0.10965	15	20152530	R	II		IGR	shelf	IGR-shelf	chr15:201	rs5605057	30;23;9;1
cg0411861	0.228936	0.439228	3.172372	0.006155	0.224708	-3.37696	0.553696	0.32476	-0.22894	4	62707027	R	II	LPHN3	Body	opensea	Body-opens		rs5334296	18;3
cg0821455	0.101169	0.734509	5.22188	9.55E-05	0.223139	0.823803	0.785093	0.683924	-0.10117	16	85250798	R	II		IGR	opensea	IGR-opense		rs1178703	43
cg2074390	0.10946	0.391394	5.200219	9.96E-05	0.223139	0.78072	0.446124	0.336664	-0.10946	13	34116177	R	II		IGR	shore	IGR-shore	chr13:341	rs5371615	48;29
cg2625972	0.11025	0.547597	5.078957	0.000126	0.223139	0.538596	0.602722	0.492472	-0.11025	20	50652947	R	II		IGR	opensea	IGR-opense		rs2426407	12
cg0632665	0.14059	0.769509	5.076967	0.000127	0.223139	0.534609	0.839804	0.699214	-0.14059	11	1.17E+08	F	II		IGR	opensea	IGR-opense		rs1229322	10;32
cg1491168	0.220278	0.369716	2.920366	0.010339	0.224708	-3.8841	0.479855	0.259578	-0.22028	12	739980	F	I	NINJ2	Body	opensea	Body-opens		rs5568616	27;29;38;43
cg1318084	0.106585	0.773784	5.071622	0.000128	0.223139	0.523899	0.827076	0.720491	-0.10659	2	2711168	F	II		IGR	opensea	IGR-opense		rs1897816	0;1;6;22
cg2175831	0.211346	0.512506	3.487244	0.003205	0.224708	-2.73195	0.618178	0.406833	-0.21135	14	1.03E+08	F	II	CDC42BPB	Body	opensea	Body-opens		rs3588035	21;24;47
cg1465584	0.100542	0.649159	4.987675	0.000151	0.224708	0.35532	0.69943	0.598888	-0.10054	2	2711189	F	II		IGR	opensea	IGR-opense		rs5651033	1;31
cg2685011	0.208852	0.48562	2.695073	0.016354	0.224896	-4.32721	0.590047	0.381194	-0.20885	6	1.7E+08	R	II	C6orf208	TSS1500	shore	TSS1500-sh	chr6:1701	rs5881841	5;1
cg0428208	0.2071	0.599095	2.189782	0.04435	0.248142	-5.2683	0.702645	0.495545	-0.2071	9	1.25E+08	R	II	LHX6	Body	island	Body-islanc	chr9:1249	rs1501862	2
cg1130024	0.147082	0.625142	4.874771	0.000189	0.224708	0.127491	0.698683	0.551601	-0.14708	9	1.37E+08	F	II		IGR	opensea	IGR-opense		rs1466424	1;3;5;15;42;46
cg2054042	0.206232	0.433906	3.71517	0.001998	0.224708	-2.26058	0.537022	0.33079	-0.20623	3	73045686	F	II	PPP4R2	TSS1500	shore	TSS1500-sh	chr3:7304		
cg0336328	0.206092	0.412641	2.426553	0.027971	0.230679	-4.83804	0.515687	0.309596	-0.20609	9	1.25E+08	R	II	LHX6	Body	island	Body-islanc	chr9:1249		
cg1488296	0.166896	0.227135	4.837345	0.000203	0.224708	0.051704	0.310583	0.143687	-0.1669	2	3699353	F	I		IGR	island	IGR-island	chr2:3699	rs5757995	51
cg1969278	0.200668	0.373064	2.624912	0.018838	0.225325	-4.46271	0.473398	0.27273	-0.20067	12	739953	F	I	LOC10004	TSS200	opensea	TSS200-ope		rs5356011	12
cg0199598	0.186472	0.718588	4.759201	0.000237	0.224708	-0.10694	0.811824	0.625352	-0.18647	6	1.47E+08	R	II	STXBP5-AS	Body	opensea	Body-opens		rs1529013	19
cg0557810	0.184613	0.350356	2.580399	0.020598	0.225654	-4.54797	0.442663	0.25805	-0.18461	12	739986	F	I	LOC10004	TSS200	opensea	TSS200-ope		rs5568616	21;23;32;37
cg1496664	0.174495	0.582432	2.820928	0.012668	0.224708	-4.08107	0.66968	0.495185	-0.1745	8	1.26E+08	F	II	MTSS1	Body	opensea	Body-opens		rs1009051	17
cg2113840	0.168975	0.349884	2.872313	0.011407	0.224708	-3.97954	0.434372	0.265397	-0.16897	5	1.32E+08	R	II	IRF1	TSS1500	shore	TSS1500-sh	chr5:1318	rs4152564	34;23;2
cg0741448	0.167795	0.633727	2.32145	0.034378	0.236654	-5.03166	0.717624	0.549829	-0.1678	6	31107186	R	II	PSORS1C1	Body	opensea	Body-opens		rs5399358	43;1
cg1993242	0.11169	0.486057	4.782301	0.000227	0.224708	-0.05999	0.541902	0.430212	-0.11169	1	2.22E+08	R	II		IGR	opensea	IGR-opense		rs5714418	28;27;26;1;1
cg1658974	0.166424	0.816291	2.427348	0.027927	0.230655	-4.83657	0.899503	0.73308	-0.16642	19	8367881	R	II	CD320	ExonBnd	opensea	ExonBnd-o		rs7965826	38;35;27;25;22;4;1
cg1778565	0.10897	0.582754	4.755101	0.000239	0.224708	-0.11528	0.637239	0.52827	-0.10897	20	809805	F	II		IGR	opensea	IGR-opense		rs5869109	7;17;31;32
cg0518696	0.13045	0.646545	4.753511	0.00024	0.224708	-0.11851	0.71177	0.58132	-0.13045	5	1.21E+08	F	II		IGR	opensea	IGR-opense			
cg2030051	0.165692	0.307064	4.329903	0.000564	0.224708	-0.98688	0.38991	0.224218	-0.16569	1	2120985	F	II	C1orf86	Body	shore	Body-shore	chr1:2121	rs1867511	11;19;28
cg0115049	0.165236	0.374128	3.646368	0.002304	0.224708	-2.4031	0.456746	0.29151	-0.16524	1	2120978	F	II	FAAP20	Body	shore	Body-shore	chr1:2121	rs1867511	18;26;35
cg2622722	0.16514	0.439325	3.398558	0.003853	0.224708	-2.91455	0.521895	0.356755	-0.16514	1	2121039	R	I	C1orf86	Body	island	Body-islanc	chr1:2121	rs1867511	45;37;28
cg0298100	0.164423	0.254796	3.43096	0.003602	0.224708	-2.8479	0.337007	0.172584	-0.16442	10	1.35E+08	R	II	GPR123	Body	shore	Body-shore	chr10:134	rs1906855	35;10
cg1456541	0.164223	0.480616	3.350766	0.004254	0.224708	-3.01269	0.562727	0.398504	-0.16422	22	29412439	R	II	ZNRF3	Body	opensea	Body-opens		rs3037486	24;22
cg0607714	0.102753	0.468748	4.701828	0.000266	0.224708	-0.22375	0.520124	0.417371	-0.10275	7	1.05E+08	R	II		IGR	opensea	IGR-opense		rs1141633	47;25;1
cg1934725	0.162221	0.704316	4.706994	0.000263	0.224708	-0.21323	0.785426	0.623205	-0.16222	8	11706360	F	II	CTSB	Body	opensea	Body-opens		rs1127696	0;1;8;37;40
cg0025591	0.161906	0.41355	2.877426	0.011288	0.224708	-3.96941	0.494503	0.332597	-0.16191	5	1.32E+08	F	II	IRF1	TSS1500	shore	TSS1500-sh	chr5:1318	rs5496419	1;15
cg1311153	0.161576	0.587717	4.007148	0.001093	0.224708	-1.6549	0.668505	0.506928	-0.16158	1	1886543	R	I	KIAA1751	3'UTR	island	3'UTR-islan	chr1:1886	rs2748976	44;26;15;13
cg0533776	0.161338	0.553102	3.876145	0.001432	0.224708	-1.9267	0.633771	0.472433	-0.16134	1	2121349	R	I	C1orf86	Body	island	Body-islanc	chr1:2121		
cg2317716	0.298125	0.583637	4.663767	0.000287	0.224708	-0.3014	0.7327	0.434575	-0.29813	1	1.16E+08	F	II		IGR	opensea	IGR-opense		rs5695769	17;22;49
cg2155710	0.160724	0.237786	3.763869	0.001806	0.224708	-2.15962	0.318148	0.157424	-0.16072	15	50410962	R	II	ATP8B4	5'UTR	opensea	5'UTR-open		rs5397142	47;31;21;9
cg1568298	0.158937	0.337608	3.670287	0.002193	0.224708	-2.35357	0.417077	0.25814	-0.15894	8	56904810	F	II	LYN	Body	opensea	Body-opens		rs5536603	1;42
cg0037983	0.113399	0.830712	4.607389	0.000322	0.224708	-0.41661	0.887411	0.774012	-0.1134	16	3137338	R	II		IGR	shore	IGR-shore	chr16:313	rs5446093	48;43;41;2
cg2033126	0.197499	0.584463	4.595374	0.00033	0.224708	-0.4412	0.683213	0.485714	-0.1975	13	73829188	R	II		IGR	opensea	IGR-opense		rs5566250	20
cg0402816	0.110523	0.602691	4.571971	0.000345	0.224708	-0.48912	0.657952	0.547429	-0.11052	12	76107548	F	II		IGR	opensea	IGR-opense		rs1894781	0;1;15;31
cg2390014	0.185977	0.540983	4.556328	0.000357	0.224708	-0.52117	0.633971	0.447994	-0.18598	9	22142053	R	II		IGR	opensea	IGR-opense		rs5579372	1
cg0586391	0.15452	0.575422	4.294241	0.000607	0.224708	-1.0605	0.652682	0.498162	-0.15452	4	1.55E+08	R	II	DCHS2	1stExon	opensea	1stExon-op		rs5432459	33;29;20;15;1

cg1396733	0.153978	0.735426	2.943206	0.009866	0.224708	-3.83858	0.812415	0.658437	-0.15398	8	17533669	F	II	MTUS1	Body	opensea	Body-opens		rs2301543	24;46
cg1987622	0.15391	0.395164	4.479491	0.000416	0.224708	-0.67885	0.472119	0.318209	-0.15391	7	12641129	F	II	SCIN	Body	opensea	Body-opens		rs1882068	8;16;23
cg2202288	0.181555	0.618169	4.520241	0.000383	0.224708	-0.59518	0.708947	0.527392	-0.18156	2	1.06E+08	R	II		IGR	island	IGR-island	chr2:1058	rs1851525	28;16
cg0551912	0.112496	0.606244	4.490627	0.000407	0.224708	-0.65597	0.662492	0.549996	-0.1125	7	1.15E+08	R	II		IGR	opensea	IGR-opense		rs7809915	32
cg0425207	0.152371	0.581908	2.286229	0.036817	0.239161	-5.09563	0.658093	0.505722	-0.15237	9	1.38E+08	F	II	COL5A1	Body	shelf	Body-shelf	chr9:1377	rs1131101	0;11;17;26;41
cg0100389	0.151963	0.78528	3.271473	0.005014	0.224708	-3.17504	0.861262	0.709298	-0.15196	8	17533903	F	II	MTUS1	Body	opensea	Body-opens		rs5300957	0;45;49;50
cg1205884	0.149169	0.63102	4.470058	0.000424	0.224708	-0.69824	0.705605	0.556436	-0.14917	20	57844021	R	II		IGR	opensea	IGR-opense			
cg2364490	0.110151	0.74888	4.464926	0.000429	0.224708	-0.70878	0.803956	0.693805	-0.11015	2	59616842	R	II		IGR	opensea	IGR-opense		rs5348907	47;38;29;28;20;15;7;5;1
cg0664302	0.151431	0.461606	2.546715	0.022034	0.225975	-4.61211	0.537322	0.385891	-0.15143	2	2.42E+08	F	I	FARP2	TSS1500	shore	TSS1500-sh	chr2:2422	rs5713563	12
cg1296842	0.149644	0.536911	4.724779	0.000254	0.224708	-0.17699	0.611733	0.462089	-0.14964	10	85898499	F	II	GHITM	TSS1500	shore	TSS1500-sh	chr10:858	rs5676181	0;18;20
cg2497117	0.12493	0.658946	4.453841	0.000439	0.224708	-0.73158	0.721411	0.596481	-0.12493	10	94155343	F	II		IGR	opensea	IGR-opense		rs1138647	11;17;47
cg1305262	0.10355	0.629571	4.445063	0.000446	0.224708	-0.74963	0.681346	0.577796	-0.10355	10	7071021	F	II		IGR	opensea	IGR-opense		rs1125495	9;27
cg1872712	0.146533	0.695501	4.441479	0.00045	0.224708	-0.757	0.768768	0.622235	-0.14653	17	406137	R	II		IGR	shore	IGR-shore	chr17:406	rs1407515	17
cg0718089	0.149577	0.80385	2.768526	0.014093	0.224708	-4.18401	0.878638	0.729061	-0.14958	6	32729130	F	II	HLA-DQB2	Body	shore	Body-shore	chr6:3272	rs1446337	51
cg2753272	0.149414	0.390259	3.94071	0.001253	0.224708	-1.79273	0.464967	0.315552	-0.14941	8	17534102	F	II	MTUS1	Body	opensea	Body-opens		rs1506595	1;23;48
cg0594523	0.11938	0.729574	4.415696	0.000474	0.224708	-0.81006	0.789264	0.669883	-0.11938	17	12425767	R	II		IGR	opensea	IGR-opense		rs1849381	26
cg0317171	0.103827	0.454101	4.397975	0.000491	0.224708	-0.84655	0.506014	0.402187	-0.10383	6	1.65E+08	F	II		IGR	opensea	IGR-opense			
cg0704743	0.116883	0.681771	4.397057	0.000492	0.224708	-0.84844	0.740212	0.623329	-0.11688	22	48346046	R	II		IGR	opensea	IGR-opense			
cg0274545	0.105517	0.562616	4.395387	0.000494	0.224708	-0.85188	0.615375	0.509858	-0.10552	15	96207497	R	II		IGR	opensea	IGR-opense		rs5371572	2
cg2338488	0.243597	0.453273	4.387775	0.000501	0.224708	-0.86756	0.575072	0.331475	-0.2436	1	1.16E+08	F	II		IGR	opensea	IGR-opense		rs1887178	3;20
cg1405816	0.108004	0.527864	4.386863	0.000502	0.224708	-0.86944	0.581866	0.473862	-0.108	22	48286722	F	II		IGR	opensea	IGR-opense		rs1806724	1;22
cg1863899	0.149144	0.648692	2.452215	0.026588	0.229555	-4.79019	0.723264	0.57412	-0.14914	15	47656820	R	II	SEMA6D	5'UTR	opensea	5'UTR-open		rs5320859	2;1
cg1583477	0.150836	0.515866	4.372522	0.000517	0.224708	-0.89899	0.591283	0.440448	-0.15084	17	43919576	R	II		IGR	opensea	IGR-opense			
cg0623831	0.148811	0.323067	2.392754	0.029897	0.232477	-4.90074	0.397473	0.248662	-0.14881	19	21265364	F	II	ZNF714	5'UTR	island	5'UTR-islan	chr19:212	rs5606097	0;9
cg2021804	0.147717	0.617091	4.360923	0.00053	0.224708	-0.9229	0.690949	0.543233	-0.14772	6	14369697	R	II		IGR	opensea	IGR-opense			
cg2201576	0.148387	0.546822	3.253257	0.005206	0.224708	-3.21224	0.621015	0.472628	-0.14839	6	34624127	F	II	C6orf106	Body	opensea	Body-opens		rs5715076	0;21;39;46
cg1770343	0.148205	0.612452	4.801945	0.000218	0.224708	-0.0201	0.686554	0.538349	-0.14821	7	22186517	R	II	RAPGEF5	ExonBnd	opensea	ExonBnd-op		rs5342141	1
cg0433349	0.148094	0.458962	3.909868	0.001336	0.224708	-1.85672	0.533009	0.384915	-0.14809	17	18763778	R	II	PRPSAP2	5'UTR	shore	5'UTR-shor	chr17:187		
cg0592887	0.147462	0.54085	2.291359	0.036452	0.238704	-5.08634	0.614581	0.467119	-0.14746	1	72302970	R	II	NEGR1	Body	opensea	Body-opens		rs1141429	1
cg0503750	0.146964	0.404178	2.22375	0.041547	0.244654	-5.20791	0.47766	0.330696	-0.14696	9	1.25E+08	R	II	LHX6	Body	island	Body-islanc	chr9:1249	rs5294799	22;9
cg2756513	0.16051	0.614254	4.313958	0.000583	0.224708	-1.01979	0.694509	0.533999	-0.16051	12	63696438	R	II		IGR	opensea	IGR-opense		rs5634446	20;14;2
cg2195015	0.146322	0.601367	2.719576	0.015563	0.224896	-4.27959	0.674528	0.528206	-0.14632	13	28555387	F	II	PRHOXNB	Body	shore	Body-shore	chr13:285		
cg0086161	0.101386	0.579866	4.299853	0.0006	0.224708	-1.04891	0.630559	0.529173	-0.10139	4	62065095	F	II		IGR	shore	IGR-shore	chr4:6206	rs1456863	30;41
cg1183959	0.14622	0.651265	4.794205	0.000221	0.224708	-0.03581	0.724375	0.578155	-0.14622	20	62833186	F	II	MYT1	Body	opensea	Body-opens		rs1866857	1
cg0577940	0.146039	0.487215	2.896459	0.010857	0.224708	-3.93164	0.560235	0.414196	-0.14604	7	1198841	F	II	ZFAND2A	5'UTR	shore	5'UTR-shor	chr7:1198	rs7851594	20;31;36;48
cg1678126	0.145665	0.429793	3.093927	0.007237	0.224708	-3.5359	0.502626	0.356961	-0.14566	2	1.01E+08	F	I	NMS	Body	opensea	Body-opens		rs5493870	0;22
cg2544918	0.110344	0.453908	4.289033	0.000613	0.224708	-1.07125	0.50908	0.398736	-0.11034	3	70115097	F	II		IGR	opensea	IGR-opense		rs5751266	31
cg1439150	0.100561	0.631507	4.270519	0.000637	0.224708	-1.1095	0.681788	0.581227	-0.10056	11	1.16E+08	R	II		IGR	opensea	IGR-opense		rs1386882	4
cg0330717	0.128559	0.71304	4.269175	0.000638	0.224708	-1.11228	0.77732	0.648761	-0.12856	3	1.96E+08	F	II		IGR	opensea	IGR-opense		rs6583331	23;34;40;41;50
cg2239822	0.111289	0.516578	4.263415	0.000646	0.224708	-1.12418	0.572222	0.460933	-0.11129	4	1.56E+08	F	II		IGR	shore	IGR-shore	chr4:1556		
cg0030391	0.145632	0.763986	3.103399	0.007097	0.224708	-3.51675	0.836802	0.69117	-0.14563	1	1.2E+08	R	II	REG4	3'UTR	opensea	3'UTR-open		rs5682227	44;1
cg2495178	0.145284	0.698119	2.647036	0.018018	0.225076	-4.42012	0.770761	0.625477	-0.14528	5	1.32E+08	R	II	SEPT8	TSS1500	shore	TSS1500-sh	chr5:1321	rs1159018	7;2
cg2023547	0.144855	0.865431	2.17653	0.045491	0.249561	-5.29172	0.937859	0.793004	-0.14486	13	1.14E+08	R	II	TMCO3	Body	shore	Body-shore	chr13:114	rs1065494	43;14;1
cg2645467	0.144597	0.630401	3.596596	0.002555	0.224708	-2.5061	0.7027	0.558103	-0.1446	9	37011472	F	II	PAX5	Body	opensea	Body-opens		rs944066;r	7;13;29
cg0456601	0.143564	0.545716	3.314578	0.004585	0.224708	-3.08686	0.617498	0.473934	-0.14356	5	1.68E+08	R	II	TENM2	Body	opensea	Body-opens		rs5559666	1
cg1234649	0.14264	0.518875	3.969712	0.00118	0.224708	-1.73256	0.590195	0.447556	-0.14264	5	1.11E+08	F	II	STARD4-AS	Body	opensea	Body-opens		rs5766634	11
cg0129524	0.142608	0.489411	2.380741	0.030611	0.23319	-4.92292	0.560715	0.418106	-0.14261	2	1.06E+08	R	II	NCK2	5'UTR	opensea	5'UTR-open		rs4851091	29;2
cg2570521	0.14244	0.652089	4.644096	0.000299	0.224708	-0.34157	0.723308	0.580869	-0.14244	5	1.19E+08	R	II	TNFAIP8	TSS1500	shore	TSS1500-sh	chr5:1186	rs1897500	46
cg1947734	0.102074	0.744685	4.216697	0.000711	0.224708	-1.22077	0.795722	0.693647	-0.10207	19	48699872	R	II		IGR	shore	IGR-shore	chr19:486		
cg1843678	0.142032	0.508243	3.011613	0.008573	0.224708	-3.70168	0.579258	0.437227	-0.14203	1	3704244	F	II	LRRC47	Body	shore	Body-shore	chr1:3703	rs5391909	0;14;26
cg2336318	0.244249	0.626568	4.206002	0.000726	0.224708	-1.24289	0.748692	0.504444	-0.24425	2	1.81E+08	R	II		IGR	opensea	IGR-opense		rs5647866	10
cg2623654	0.142809	0.591585	4.201394	0.000733	0.224708	-1.25243	0.66299	0.520181	-0.14281	8	1.44E+08	F	II		IGR	opensea	IGR-opense		rs5876270	12;14;14;16;47
cg0129281	0.179024	0.511233	4.201118	0.000734	0.224708	-1.253	0.600745	0.421721	-0.17902	3	1.84E+08	R	II		IGR	shore	IGR-shore	chr3:1842	rs5626114	27
cg1275574	0.121015	0.337131	4.200041	0.000735	0.224708	-1.25523	0.397638	0.276623	-0.12101	4	1.85E+08	R	II		IGR	shore	IGR-shore	chr4:1846	rs5600000	48;23;12
cg0846867	0.104205	0.425232	4.198989	0.000737	0.224708	-1.2574	0.477335	0.37313	-0.1042	15	61927784	F	II		IGR	opensea	IGR-opense		rs7459980	1
cg0605948	0.111496	0.613805	4.195952	0.000742	0.224708	-1.26369	0.669553	0.558057	-0.1115	10	46171106	F	II		IGR	shelf	IGR-shelf	chr10:461		
cg0109151	0.139734	0.241418	4.190566	0.00075	0.224708	-1.27483	0.311285	0.171552	-0.13973	4	1.85E+08	F	II		IGR	shore	IGR-shore	chr4:1846	rs1117468	11;31
cg1395651	0.124244	0.547814	4.190094	0.000751	0.224708	-1.27581	0.609936	0.485692	-0.12424	12	40511665	F	II		IGR	opensea	IGR-opense			
cg0888034	0.141754	0.587135	2.830596	0.012421	0.224708	-4.06201	0.658012	0.516258	-0.14175	22	49860548	F	II	C22orf34	Body	opensea	Body-opens		rs1880245	5
cg1391263	0.109205	0.529572	4.179724	0.000767	0.224708	-1.29727	0.584175	0.47497	-0.1092	9	1.36E+08	F	II		IGR	shore	IGR-shore	chr9:1360	rs5356506	46
cg0182806	0.105757	0.402657	4.178179	0.000769	0.224708	-1.30047	0.455536	0.349779	-0.10576	5	1.41E+08	F	II		IGR	shore	IGR-shore	chr5:1406	rs5501447	48;50
cg1340577	0.141534	0.4621	4.212672	0.000717	0.224708	-1.22909	0.532867	0.391333	-0.14153	22	32599648	F	II	RFPL2	TSS200	opensea	TSS200-ope		rs5673285	1;35
cg0526362	0.100063	0.571564	4.171017	0.00078	0.224708	-1.3153	0.621596	0.521533	-0.10006	2	76539134	F	II		IGR	opensea	IGR-opense			
cg1412218	0.130638	0.605788	4.1624	0.000794	0.224708	-1.33314	0.671107	0.54047	-0.13064	8	1.3E+08	F	II		IGR	opensea	IGR-opense		rs5596979	1;5;44

cg1663425	0.100344	0.438908	4.157921	0.000802	0.224708	-1.34241	0.489079	0.388736	-0.10034	6	1.48E+08	R	II		IGR	opensea	IGR-opense		rs5529425	26
cg1799683	0.141437	0.496222	3.651561	0.002279	0.224708	-2.39235	0.56694	0.425503	-0.14144	11	78616440	R	II	ODZ4	Body	shore	Body-shore	chr11:786	rs1909848	17;12
cg1871587	0.183223	0.551566	4.150056	0.000815	0.224708	-1.3587	0.643178	0.459954	-0.18322	2	2.43E+08	R	II		IGR	shelf	IGR-shelf	chr2:2426	rs5318817	10;2
cg2308076	0.141146	0.562093	3.83913	0.001546	0.224708	-2.0035	0.632666	0.49152	-0.14115	1	1.71E+08	R	II	FMO1	TSS1500	opensea	TSS1500-op		rs2064078	10
cg0632910	0.140671	0.593891	3.194014	0.005885	0.224708	-3.33297	0.664226	0.523555	-0.14067	8	20810381	R	II	LOC10246	TSS1500	opensea	TSS1500-op		rs1134091	1
cg1140151	0.105648	0.578283	4.137974	0.000835	0.224708	-1.38372	0.631107	0.525459	-0.10565	7	1.19E+08	F	II		IGR	opensea	IGR-opense		rs1510832	0;19
cg2080282	0.10487	0.474309	4.137094	0.000837	0.224708	-1.38554	0.526744	0.421874	-0.10487	6	30782080	R	II		IGR	opensea	IGR-opense		rs5306903	38;15;2
cg1785709	0.140535	0.638535	3.394915	0.003882	0.224708	-2.92204	0.708803	0.568268	-0.14054	6	30907280	F	II	DPCR1	TSS1500	opensea	TSS1500-op			
cg0041617	0.118543	0.687804	4.13022	0.000849	0.224708	-1.39978	0.747076	0.628532	-0.11854	17	406140	R	II		IGR	shore	IGR-shore	chr17:406	rs1407515	20
cg1983196	0.130939	0.725148	4.126933	0.000854	0.224708	-1.40659	0.790618	0.659679	-0.13094	2	317929	R	II		IGR	shelf	IGR-shelf	chr2:3148	rs7792054	20
cg1508613	0.108491	0.39758	4.121612	0.000864	0.224708	-1.41761	0.451826	0.343335	-0.10849	8	1.13E+08	R	II		IGR	opensea	IGR-opense		rs5628999	49;46;43;30;9
cg0343116	0.139683	0.566227	3.344294	0.004312	0.224708	-3.02596	0.636068	0.496385	-0.13968	3	1.22E+08	F	II	CD86	5'UTR	opensea	5'UTR-open		rs1407765	0;6;14;15;29;35
cg1805437	0.139507	0.441442	3.626504	0.002401	0.224708	-2.44422	0.511195	0.371688	-0.13951	10	81194201	F	II	ZCCHC24	Body	opensea	Body-open		rs5744125	4;18;35;42;44
cg0867790	0.148534	0.727985	4.099788	0.000903	0.224708	-1.46283	0.802252	0.653718	-0.14853	2	2.38E+08	R	II		IGR	opensea	IGR-opense		rs999033;r	30;18;13;11;2
cg1635485	0.120512	0.663489	4.099651	0.000903	0.224708	-1.46311	0.723744	0.603233	-0.12051	12	19218990	F	II		IGR	opensea	IGR-opense		rs5481738	12
cg0453100	0.119158	0.623793	4.099642	0.000903	0.224708	-1.46313	0.683372	0.564214	-0.11916	2	1.28E+08	F	II		IGR	opensea	IGR-opense			
cg0220010	0.104445	0.407157	4.080114	0.00094	0.224708	-1.5036	0.459379	0.354934	-0.10444	10	1.23E+08	F	II		IGR	opensea	IGR-opense		rs5403855	1;8;10;29;36
cg0788982	0.102864	0.710653	4.07936	0.000942	0.224708	-1.50517	0.762085	0.659221	-0.10286	10	93443413	F	II		IGR	opensea	IGR-opense		rs5283471	0;3;36
cg2735389	0.139166	0.323489	3.087084	0.007339	0.224708	-3.54972	0.393072	0.253905	-0.13917	3	1.95E+08	F	I	MUC4	Body	island	Body-islan	chr3:1954	rs1995527	8;27
cg0673730	0.13904	0.684258	2.649874	0.017915	0.225076	-4.41465	0.753778	0.614738	-0.13904	4	1.85E+08	R	II	ENPP6	Body	shelf	Body-shelf	chr4:1850	rs5768557	6
cg2042238	0.118408	0.342609	4.067359	0.000965	0.224708	-1.53004	0.401813	0.283405	-0.11841	1	2.01E+08	R	II		IGR	opensea	IGR-opense		rs2809341	48;20
cg1188541	0.138819	0.494323	3.066947	0.00765	0.224708	-3.59035	0.563732	0.424913	-0.13882	21	30715207	R	II	BACH1	3'UTR	opensea	3'UTR-open		rs5398539	13
cg0677718	0.137825	0.605132	2.146216	0.048202	0.252931	-5.34502	0.674045	0.53622	-0.13782	4	1.06E+08	R	II	PPA2	Body	opensea	Body-open		rs1488741	31;2;1
cg1357146	0.137738	0.631068	2.255481	0.039079	0.241564	-5.15108	0.699937	0.562199	-0.13774	9	1.25E+08	F	I	LHX6	Body	island	Body-islan	chr9:1249	rs1120438	44
cg2436412	0.13757	0.577934	2.843797	0.012091	0.224708	-4.03595	0.646719	0.509149	-0.13757	15	33757827	F	II	RYR3	Body	opensea	Body-open		rs5452455	0;4;13;40
cg1193862	0.165802	0.614098	4.056361	0.000988	0.224708	-1.55285	0.696999	0.531198	-0.1658	1	2.44E+08	F	II		IGR	opensea	IGR-opense		rs5770717	36
cg0366689	0.137296	0.60917	3.967455	0.001186	0.224708	-1.73724	0.677818	0.540522	-0.1373	11	76847277	R	II	MYO7A	Body	shore	Body-shore	chr11:768	rs1841666	37
cg0900931	0.13708	0.651197	3.728641	0.001943	0.224708	-2.23266	0.719737	0.582657	-0.13708	9	6566991	F	II	GLDC	Body	opensea	Body-open			
cg1170604	0.100411	0.69667	4.051859	0.000997	0.224708	-1.56218	0.746875	0.646465	-0.10041	13	1.1E+08	R	II		IGR	opensea	IGR-opense		rs2182920	37;11;2
cg0970995	0.136414	0.374021	4.863546	0.000193	0.224708	0.104774	0.442228	0.305814	-0.13641	12	54017699	F	II	ATF7	5'UTR	shelf	5'UTR-shelf	chr12:540	rs1854655	0
cg2497143	0.100524	0.686901	4.050181	0.001	0.224708	-1.56566	0.737163	0.636639	-0.10052	18	22094439	F	II		IGR	opensea	IGR-opense		rs5723839	15;39
cg2088809	0.113366	0.732772	4.047734	0.001005	0.224708	-1.57073	0.789456	0.676089	-0.11337	7	1.55E+08	F	II		IGR	opensea	IGR-opense		rs1866531	0;38
cg1669256	0.117237	0.601861	4.038496	0.001025	0.224708	-1.58989	0.66048	0.543243	-0.11724	2	39135866	R	II		IGR	opensea	IGR-opense		rs1463480	20
cg2731357	0.107165	0.728968	4.034403	0.001033	0.224708	-1.59838	0.782551	0.675386	-0.10716	7	1.57E+08	R	II		IGR	shore	IGR-shore	chr7:1572	rs5556188	51;29;27;16
cg1840833	0.111857	0.494547	4.032966	0.001036	0.224708	-1.60136	0.550475	0.438618	-0.11186	18	3305544	R	II		IGR	opensea	IGR-opense		rs5344272	42
cg2144677	0.111647	0.557174	4.032234	0.001038	0.224708	-1.60287	0.612998	0.501351	-0.11165	2	2.23E+08	R	II		IGR	opensea	IGR-opense		rs5305531	2
cg0565892	0.134213	0.536845	4.03131	0.00104	0.224708	-1.60479	0.603952	0.469739	-0.13421	7	13191068	R	II		IGR	opensea	IGR-opense		rs5354259	20
cg0566644	0.13636	0.714198	3.130507	0.006711	0.224708	-3.46189	0.782378	0.646018	-0.13636	22	26023816	F	II	ADRBK2	Body	opensea	Body-open		rs1174756	0;41;49
cg1822494	0.135617	0.449982	4.835554	0.000204	0.224708	0.048074	0.51779	0.382173	-0.13562	1	1225781	R	II	SCNN1D	Body	shelf	Body-shelf	chr1:1228	rs1131974	30;29;28;15;2;1
cg1529342	0.135374	0.563475	5.443699	6.23E-05	0.219272	1.261947	0.631162	0.495788	-0.13537	2	37897716	F	II	CDC42EP3	5'UTR	shore	5'UTR-shor	chr2:3789	rs1133621	0;34
cg2045833	0.135222	0.513065	4.682508	0.000277	0.224708	-0.26315	0.580676	0.445454	-0.13522	13	32502384	R	II	EEF1DP3	Body	opensea	Body-open			
cg1726612	0.135128	0.584152	4.143501	0.000826	0.224708	-1.37227	0.651716	0.516588	-0.13513	21	30476810	R	II	MAP3K7CL	5'UTR	opensea	5'UTR-open		rs1870250	6;2
cg0844007	0.118569	0.519811	4.01234	0.001081	0.224708	-1.64413	0.579096	0.460526	-0.11857	2	2.42E+08	R	II		IGR	shore	IGR-shore	chr2:2418	rs1454877	1
cg1980578	0.104985	0.71858	4.011706	0.001083	0.224708	-1.64545	0.771073	0.666088	-0.10499	17	70472110	R	II		IGR	opensea	IGR-opense		rs5739337	48
cg0302528	0.111937	0.634696	4.011444	0.001083	0.224708	-1.64599	0.690665	0.578728	-0.11194	8	19752474	R	II		IGR	opensea	IGR-opense		rs1476263	1
cg0425867	0.110498	0.623173	4.008537	0.00109	0.224708	-1.65202	0.678422	0.567924	-0.1105	1	94240208	F	II		IGR	opensea	IGR-opense		rs5343663	3;26;32
cg2759689	0.134911	0.532152	3.691639	0.002098	0.224708	-2.30934	0.599608	0.464697	-0.13491	16	49621173	F	II	ZNF423	Body	opensea	Body-open		rs5494576	0;7
cg2688694	0.134125	0.584055	2.721888	0.015491	0.224896	-4.27509	0.651117	0.516992	-0.13413	14	90399629	F	II	EFCAB11	Body	opensea	Body-open		rs1173870	0;1;9
cg0903593	0.134116	0.643833	2.496027	0.024375	0.227653	-4.70798	0.710891	0.576775	-0.13412	12	1.29E+08	R	II	SLC15A4	Body	shore	Body-shore	chr12:129	rs5426064	18;14
cg0916364	0.134091	0.576456	4.107827	0.000888	0.224708	-1.44617	0.643501	0.509411	-0.13409	9	6566510	R	II	GLDC	Body	opensea	Body-open		rs5738601	48;30;22
cg0597327	0.117303	0.662401	4.000228	0.001108	0.224708	-1.66925	0.721052	0.60375	-0.1173	22	32860714	F	II		IGR	opensea	IGR-opense			
cg2012459	0.10467	0.499302	3.999482	0.00111	0.224708	-1.6708	0.551637	0.446967	-0.10467	14	35825453	R	II		IGR	opensea	IGR-opense		rs8005252	19;18;2;1
cg1785835	0.134035	0.564209	4.079342	0.000942	0.224708	-1.5052	0.631227	0.497192	-0.13403	3	1.78E+08	R	II	KCNMB2-A	Body	opensea	Body-open		rs5304138	40;25;13;2
cg0225064	0.13392	0.547292	2.993881	0.008892	0.224708	-3.73724	0.614252	0.480332	-0.13392	15	45020024	R	II	LOC10041	TSS1500	opensea	TSS1500-op		rs1381315	40;1
cg0016345	0.133892	0.560194	3.963655	0.001195	0.224708	-1.74512	0.62714	0.493248	-0.13389	20	10411528	R	II	MKKS	5'UTR	shelf	5'UTR-shelf	chr20:104		
cg1783618	0.102677	0.605361	3.984544	0.001145	0.224708	-1.70179	0.6567	0.554023	-0.10268	21	36570494	F	II		IGR	opensea	IGR-opense		rs5651723	50;51
cg0929501	0.133759	0.574825	3.650867	0.002283	0.224708	-2.39379	0.641704	0.507945	-0.13376	8	99503021	F	II	STK3	Body	opensea	Body-open		rs1828935	0;30;31
cg0893710	0.135429	0.676492	3.973463	0.001171	0.224708	-1.72478	0.744207	0.608778	-0.13543	16	3136857	R	II		IGR	shelf	IGR-shelf	chr16:313	rs5773290	45;2
cg0048206	0.127083	0.609905	3.971589	0.001176	0.224708	-1.72866	0.673447	0.546364	-0.12708	1	61478983	F	II		IGR	opensea	IGR-opense		rs1486563	23;29
cg0248832	0.109456	0.533992	3.971003	0.001177	0.224708	-1.72988	0.58872	0.479264	-0.10946	19	5841442	R	II		IGR	shelf	IGR-shelf	chr19:584		
cg0234365	0.133623	0.527394	3.554404	0.002788	0.224708	-2.59332	0.594205	0.460582	-0.13362	21	30476730	R	II	MAP3K7CL	5'UTR	opensea	5'UTR-open			
cg0662591	0.133338	0.505576	3.124204	0.006798	0.224708	-3.47465	0.572246	0.438907	-0.13334	4	1.2E+08	F	II	SEC24D	Body	opensea	Body-open			
cg0738337	0.132961	0.456305	3.391558	0.003909	0.224708	-2.92894	0.522785	0.389824	-0.13296	7	40766405	R	II	SUGCT	Body	opensea	Body-open		rs1875599	1
cg1518630	0.113514	0.548872	3.965984	0.00119	0.224708	-1.74029	0.605629	0.492115	-0.11351	13	55110248	R	II		IGR	opensea	IGR-opense		rs5697292	42;31;26;1

cg2109415	0.132951	0.514339	3.255701	0.00518	0.224708	-3.20725	0.580815	0.447864	-0.13295	7	17345221	F	II	AHR	Body	opensea	Body-opens		rs1154274	0;39;41
cg1079076	0.102299	0.639782	3.96078	0.001202	0.224708	-1.75109	0.690932	0.588632	-0.1023	17	47562801	R	II		IGR	opensea	IGR-opense		rs1506627	47;27;2;1
cg1550090	0.132409	0.505744	3.435158	0.003571	0.224708	-2.83926	0.571948	0.43954	-0.13241	6	1.12E+08	R	II	LAMA4	Body	opensea	Body-opens		rs2005416	49;40;36;24;7;2
cg2221033	0.131893	0.342768	2.540733	0.022298	0.226234	-4.62346	0.408714	0.276822	-0.13189	11	2322808	R	I	C11orf21	Body	opensea	Body-opens		rs1422473	28;7
cg1327889	0.112581	0.516314	3.956353	0.001213	0.224708	-1.76027	0.572604	0.460023	-0.11258	13	84451164	R	II		IGR	shelf	IGR-shelf	chr13:8445	rs5749292	31;1
cg2532668	0.131433	0.580674	3.254706	0.005191	0.224708	-3.20928	0.64639	0.514957	-0.13143	5	1.68E+08	F	II	TENM2	Body	opensea	Body-opens		rs1930661	15
cg0053658	0.103267	0.59377	3.943702	0.001245	0.224708	-1.78652	0.645404	0.542136	-0.10327	12	1.08E+08	F	II		IGR	opensea	IGR-opense		rs5545677	0
cg1593156	0.131408	0.563223	3.498657	0.00313	0.224708	-2.70841	0.628927	0.497519	-0.13141	9	71985131	F	II	FAM189A2	Body	opensea	Body-opens		rs1113834	20;38
cg0234164	0.13133	0.81184	3.613533	0.002467	0.224708	-2.47106	0.877505	0.746175	-0.13133	17	71756141	F	II	C17orf54	Body	opensea	Body-opens		rs7137349	15;17
cg1337635	0.131254	0.480226	3.375575	0.004041	0.224708	-2.96177	0.545853	0.414599	-0.13125	6	87892453	F	II	ZNF292	Body	opensea	Body-opens		rs1178603	7;21;32
cg0255615	0.111694	0.503657	3.933279	0.001273	0.224708	-1.80815	0.559504	0.447811	-0.11169	3	1.14E+08	R	II		IGR	opensea	IGR-opense		rs3741836	7
cg1862266	0.116989	0.732549	3.93281	0.001274	0.224708	-1.80912	0.791043	0.674054	-0.11699	1	2.24E+08	R	II		IGR	opensea	IGR-opense			
cg0583511	0.130828	0.434969	3.191525	0.005916	0.224708	-3.33803	0.500383	0.369555	-0.13083	7	48294858	F	II	ABCA13	Body	opensea	Body-opens			
cg1427204	0.104163	0.774375	3.928216	0.001286	0.224708	-1.81865	0.826456	0.722293	-0.10416	12	92453691	F	II		IGR	opensea	IGR-opense			
cg0527136	0.13158	0.692634	3.927851	0.001287	0.224708	-1.81941	0.758424	0.626844	-0.13158	10	27259297	R	II		IGR	opensea	IGR-opense			
cg0719037	0.143667	0.556715	3.925294	0.001294	0.224708	-1.82472	0.628549	0.484882	-0.14367	9	1.38E+08	R	II		IGR	opensea	IGR-opense		rs2006720	0
cg2731159	0.188166	0.755849	3.9213	0.001304	0.224708	-1.833	0.849932	0.661766	-0.18817	4	1521984	R	II		IGR	island	IGR-island	chr4:1521	rs1394081	39;34;29;15
cg0733609	0.12893	0.457166	3.921249	0.001305	0.224708	-1.83311	0.521631	0.392701	-0.12893	2	1.4E+08	R	II		IGR	opensea	IGR-opense		rs5690980	47
cg1981640	0.130617	0.693982	3.126773	0.006763	0.224708	-3.46945	0.75929	0.628673	-0.13062	22	45993430	F	II	FBLN1	Body	opensea	Body-opens		rs5328689	16
cg0244969	0.103641	0.648304	3.91912	0.00131	0.224708	-1.83753	0.700124	0.596483	-0.10364	1	1.96E+08	R	II		IGR	opensea	IGR-opense		rs5413744	43;40;1
cg2679460	0.123853	0.511319	3.917677	0.001314	0.224708	-1.84052	0.573245	0.449392	-0.12385	10	89942354	R	II		IGR	opensea	IGR-opense		rs5509443	28
cg0008981	0.104051	0.61611	3.915166	0.001321	0.224708	-1.84573	0.668136	0.564085	-0.10405	3	72641008	R	II		IGR	opensea	IGR-opense		rs5618881	16;2;1
cg2469376	0.128842	0.24704	3.915036	0.001321	0.224708	-1.846	0.31146	0.182619	-0.12884	1	1.68E+08	F	II		IGR	opensea	IGR-opense		rs1387513	3;13;48
cg1361382	0.130486	0.784217	2.633365	0.018521	0.225084	-4.44645	0.849459	0.718974	-0.13049	22	45897722	F	II	FBLN1	TSS1500	shore	TSS1500-sh	chr22:4589	rs1124286	0;28
cg2234506	0.106119	0.778598	3.912395	0.001329	0.224708	-1.85148	0.831658	0.725539	-0.10612	5	6795634	R	II		IGR	opensea	IGR-opense		rs5626137	29;8
cg0023972	0.115292	0.369106	3.910891	0.001333	0.224708	-1.8546	0.426752	0.31146	-0.11529	12	1.09E+08	F	II		IGR	opensea	IGR-opense		rs1901122	1;25;35
cg2349277	0.13045	0.70838	3.528859	0.00294	0.224708	-2.64608	0.773605	0.643155	-0.13045	6	36928572	F	II	PI16	Body	shore	Body-shore	chr6:3692	rs1875644	32
cg1513125	0.130285	0.531777	2.77412	0.013933	0.224708	-4.17305	0.59692	0.466635	-0.13029	20	31805182	R	II	C20orf71	5'UTR	opensea	5'UTR-open		rs5351642	43;9;2;1;1
cg2571614	0.104719	0.531592	3.891192	0.001388	0.224708	-1.89548	0.583951	0.479233	-0.10472	3	33763505	R	II		IGR	shelf	IGR-shelf	chr3:3375	rs5389048	46;1
cg0924328	0.130258	0.229966	3.967466	0.001186	0.224708	-1.73722	0.295095	0.164837	-0.13026	9	6015084	R	II	RANBP6	Body	opensea	Body-opens			
cg1707392	0.129835	0.500535	3.230916	0.005453	0.224708	-3.25782	0.565453	0.435618	-0.12984	10	81106651	F	II	PPIF	TSS1500	shore	TSS1500-sh	chr10:811	rs1693709	42
cg1108453	0.107805	0.582822	3.882996	0.001412	0.224708	-1.91248	0.636724	0.528919	-0.1078	6	1.32E+08	R	II		IGR	shelf	IGR-shelf	chr6:1322		
cg2758679	0.438928	0.508526	3.879298	0.001423	0.224708	-1.92016	0.72799	0.289062	-0.43893	5	13664584	R	II		IGR	opensea	IGR-opense		rs5705085	13;3;2;2;1
cg2128108	0.129775	0.52239	3.373302	0.00406	0.224708	-2.96644	0.587278	0.457503	-0.12978	5	55067187	R	II	DDX4	Body	opensea	Body-opens		rs5311283	28
cg0679159	0.129655	0.446112	3.515512	0.003023	0.224708	-2.67363	0.510939	0.381284	-0.12966	6	32823292	R	II	PSMB9	Body	shore	Body-shore	chr6:3282	rs3763346	37;1
cg1481659	0.129434	0.494231	3.823885	0.001595	0.224708	-2.03513	0.558948	0.429514	-0.12943	3	1.42E+08	F	II	ATP1B3	Body	opensea	Body-opens		rs7819298	9;36;37;43
cg0433552	0.129067	0.667716	3.101658	0.007122	0.224708	-3.52027	0.732249	0.603182	-0.12907	10	53790078	R	II	PRKG1	Body	opensea	Body-opens		rs1073389	46
cg1354650	0.128767	0.53406	3.191387	0.005917	0.224708	-3.33831	0.598443	0.469676	-0.12877	14	78864773	R	II	NRXN3	Body	opensea	Body-opens		rs5640816	2;1
cg1132967	0.111548	0.517063	3.855729	0.001494	0.224708	-1.96906	0.572838	0.461289	-0.11155	5	2165422	R	II		IGR	opensea	IGR-opense		rs5373332	30
cg0937751	0.128476	0.548079	4.890807	0.000183	0.224708	0.159925	0.612316	0.483841	-0.12848	17	11786949	R	II	DNAH9	5'UTR	opensea	5'UTR-open		rs1918127	45;26;10;2;1
cg1879578	0.128376	0.609648	4.806157	0.000216	0.224708	-0.01155	0.673835	0.54546	-0.12838	12	41087584	R	II	CNTN1	5'UTR	shore	5'UTR-shore	chr12:4108		
cg0797309	0.128364	0.474081	2.850773	0.01192	0.224708	-4.02217	0.538263	0.409899	-0.12836	16	451040	R	I	DECR2	TSS1500	island	TSS1500-isl	chr16:4508	rs1123893	47;16
cg0613362	0.127926	0.560737	3.265391	0.005077	0.224708	-3.18746	0.6247	0.496774	-0.12793	4	1.01E+08	R	II	EMCN	Body	opensea	Body-opens		rs3611005	50;2;1
cg0934018	0.145493	0.546092	3.832874	0.001566	0.224708	-2.01648	0.618838	0.473345	-0.14549	5	1.51E+08	R	II		IGR	opensea	IGR-opense		rs7963111	24;7;3
cg2136987	0.175341	0.685986	3.829896	0.001576	0.224708	-2.02266	0.773656	0.598316	-0.17534	5	1.51E+08	F	II		IGR	opensea	IGR-opense		rs1997731	0;10
cg0249618	0.127833	0.711766	5.431063	6.38E-05	0.219272	1.237141	0.775683	0.64785	-0.12783	8	1.34E+08	R	II	TG	Body	opensea	Body-opens		rs1494385	2
cg0753924	0.103778	0.716647	3.823986	0.001595	0.224708	-2.03492	0.768536	0.664757	-0.10378	7	93919388	F	II		IGR	opensea	IGR-opense		rs5531540	7;9;16;36
cg0504539	0.127683	0.496762	4.547177	0.000363	0.224708	-0.53993	0.560603	0.43292	-0.12768	18	6249208	R	II	L3MBTL4	Body	opensea	Body-opens		rs5448058	50;26;14
cg0439339	0.102994	0.507724	3.820772	0.001606	0.224708	-2.04159	0.559221	0.456227	-0.10299	11	1.12E+08	R	II		IGR	opensea	IGR-opense		rs5663130	1
cg1424870	0.127455	0.663672	2.773356	0.013955	0.224708	-4.17455	0.727399	0.599945	-0.12745	5	1.51E+08	R	II	CTB-12O2.1	Body	opensea	Body-opens		rs5434732	11;2
cg0218455	0.127296	0.163954	2.575829	0.020788	0.225654	-4.55669	0.227602	0.100307	-0.1273	16	68104456	F	II	DUS2	Body	opensea	Body-opens		rs5655812	6;33
cg2665875	0.127022	0.479327	4.056733	0.000987	0.224708	-1.55207	0.542839	0.415816	-0.12702	14	77666908	F	II	TMEM63C	5'UTR	opensea	5'UTR-open		rs1710545	29
cg0965895	0.126971	0.322247	4.482504	0.000414	0.224708	-0.67266	0.385733	0.258762	-0.12697	8	56904259	R	II	LYN	Body	opensea	Body-opens		rs2667977	17;14;2
cg1290696	0.102064	0.405297	3.808052	0.001648	0.224708	-2.06798	0.456329	0.354265	-0.10206	3	3807867	R	II		IGR	opensea	IGR-opense		rs5648868	1
cg2122365	0.126862	0.420746	3.095326	0.007216	0.224708	-3.53307	0.484177	0.357315	-0.12686	10	15873568	R	II	FAM188A	Body	opensea	Body-opens		rs1153725	24
cg0132928	0.111201	0.390584	3.800257	0.001675	0.224708	-2.08415	0.446184	0.334983	-0.1112	5	31657134	R	II		IGR	opensea	IGR-opense		rs5723343	43;38
cg0047851	0.126661	0.504829	3.488453	0.003197	0.224708	-2.72946	0.56816	0.441499	-0.12666	2	1.61E+08	R	II	ITGB6	Body	opensea	Body-opens		rs5569174	7;1
cg0959462	0.126661	0.423343	4.227663	0.000695	0.224708	-1.19809	0.486674	0.360013	-0.12666	5	1.19E+08	R	II	TNFAIP8	TSS1500	shore	TSS1500-sh	chr5:1186	rs7610611	27;21
cg1499231	0.103764	0.337581	3.796642	0.001688	0.224708	-2.09164	0.389462	0.285699	-0.10376	7	1.55E+08	R	II		IGR	opensea	IGR-opense			
cg0796772	0.100968	0.69164	3.795706	0.001691	0.224708	-2.09359	0.742124	0.641156	-0.10097	14	21199232	F	II		IGR	opensea	IGR-opense		rs5704454	6;22;45
cg0274071	0.126538	0.485289	3.681949	0.00214	0.224708	-2.32941	0.548558	0.42202	-0.12654	5	1.51E+08	F	II	SLC36A1	TSS1500	opensea	TSS1500-op		rs1390628	0;44;47
cg1660483	0.126514	0.363038	3.201839	0.005791	0.224708	-3.31704	0.426295	0.299781	-0.12651	7	1.08E+08	F	II	NRCAM	5'UTR	opensea	5'UTR-open		rs5572698	0;23;28;45
cg2119057	0.117275	0.396264	3.789778	0.001712	0.224708	-2.10588	0.454901	0.337626	-0.11728	13	74818020	F	II		IGR	opensea	IGR-opense		rs5415324	15
cg2702628	0.103962	0.528144	3.785659	0.001727	0.224708	-2.11442	0.580125	0.476163	-0.10396	2	18248362	F	II		IGR	opensea	IGR-opense		rs1163842	0;14;21;25

cg0054177	0.126487	0.799895	2.257169	0.038951	0.241391	-5.14805	0.863138	0.736651	-0.12649	2	3652840	R	II	COLEC11	TSS1500	opensea	TSS1500-op		rs5403612	12
cg0095758	0.126327	0.523546	3.160388	0.006309	0.224708	-3.4013	0.586709	0.460383	-0.12633	15	86189830	R	II	AKAP13	Body	opensea	Body-opens		rs5378322	51;27
cg1967409	0.126286	0.640498	3.056463	0.007818	0.224708	-3.61148	0.703641	0.577355	-0.12629	15	90643766	F	I	IDH2	Body	shore	Body-shore	chr15:9064	rs1431235	16;18
cg2742285	0.138646	0.738337	3.773527	0.00177	0.224708	-2.13959	0.80766	0.669015	-0.13865	2	1.06E+08	R	II		IGR	island	IGR-island	chr2:1058	rs5370985	48;32;15
cg2119995	0.126201	0.7515	5.008411	0.000145	0.224708	0.397027	0.814601	0.688399	-0.1262	1	2.23E+08	R	II	MIA3	Body	shore	Body-shore	chr1:2227		
cg0814986	0.109934	0.551176	3.768082	0.001791	0.224708	-2.15088	0.606144	0.496209	-0.10993	21	24264513	F	II		IGR	opensea	IGR-opense			
cg0132369	0.126084	0.400033	3.258128	0.005154	0.224708	-3.2023	0.463075	0.336991	-0.12608	3	52705086	R	II	PBRM1	Body	opensea	Body-opens		rs1144923	2
cg0492268	0.100934	0.424667	3.761924	0.001814	0.224708	-2.16365	0.475134	0.3742	-0.10093	3	1.89E+08	F	II		IGR	opensea	IGR-opense		rs9815495	24;28
cg1290638	0.125821	0.442984	3.556752	0.002775	0.224708	-2.58847	0.505894	0.380073	-0.12582	22	32599516	F	I	RFPL2	TSS200	opensea	TSS200-ope		rs5423692	17;24
cg1381544	0.125812	0.540612	3.537674	0.002887	0.224708	-2.62788	0.603518	0.477706	-0.12581	11	88028475	R	II	CTSC	Body	opensea	Body-opens		rs5606178	21;18;8
cg1861028	0.125716	0.505258	5.28145	8.51E-05	0.223139	0.942019	0.568116	0.4424	-0.12572	18	36994604	F	II	LINC00669	Body	opensea	Body-opens		rs5660288	0
cg0979936	0.105701	0.677088	3.75466	0.001841	0.224708	-2.17871	0.729938	0.624237	-0.1057	20	51268046	F	II		IGR	opensea	IGR-opense		rs5594264	0;36;39
cg0023935	0.125406	0.508936	2.879392	0.011243	0.224708	-3.96551	0.571639	0.446233	-0.12541	16	3115133	F	I	IL32	TSS1500	opensea	TSS1500-op		rs1131244	0;28;40;41
cg2382102	0.12532	0.486382	3.132309	0.006686	0.224708	-3.45824	0.549042	0.423722	-0.12532	13	99962835	F	II	UBAC2	Body	opensea	Body-opens			
cg0601916	0.119867	0.602858	3.745976	0.001874	0.224708	-2.19672	0.662791	0.542924	-0.11987	6	1.56E+08	R	II		IGR	opensea	IGR-opense		rs1138755	49;24
cg0107608	0.124979	0.515442	3.150774	0.006436	0.224708	-3.42081	0.577932	0.452952	-0.12498	3	1.78E+08	F	II	KCNMB2	5'UTR	opensea	5'UTR-open		rs5316963	0;29
cg2427744	0.11332	0.574709	3.742266	0.001889	0.224708	-2.20441	0.631369	0.518049	-0.11332	14	52796897	F	II		IGR	opensea	IGR-opense		rs1834354	27;43
cg1647310	0.110363	0.538788	3.739072	0.001901	0.224708	-2.21103	0.593969	0.483606	-0.11036	5	1.31E+08	F	II		IGR	opensea	IGR-opense		rs5610944	1;23;30
cg0717553	0.127795	0.475083	3.738724	0.001903	0.224708	-2.21175	0.53898	0.411185	-0.1278	16	15991691	F	II		IGR	opensea	IGR-opense		rs5528229	0
cg0192134	0.124611	0.763622	2.561069	0.021411	0.225654	-4.58482	0.825928	0.701317	-0.12461	1	1.6E+08	R	II	FCRL6	Body	opensea	Body-opens		rs5448720	29;2;1
cg1655686	0.124479	0.776837	3.829168	0.001578	0.224708	-2.02417	0.839076	0.714597	-0.12448	20	55029866	F	II	CASS4	Body	opensea	Body-opens		rs5297707	26
cg0715138	0.123623	0.516234	3.731447	0.001932	0.224708	-2.22684	0.578045	0.454422	-0.12362	5	1.27E+08	F	II		IGR	opensea	IGR-opense		rs5715783	40
cg0392093	0.124158	0.501421	3.025307	0.008335	0.224708	-3.67417	0.5635	0.439342	-0.12416	2	55538851	F	II	CCDC88A	Body	opensea	Body-opens			
cg1968280	0.124101	0.660128	3.210673	0.005686	0.224708	-3.29906	0.722178	0.598077	-0.1241	8	1.03E+08	F	II	NCALD	5'UTR	opensea	5'UTR-open		rs5481098	13
cg1190092	0.144183	0.399392	3.727315	0.001948	0.224708	-2.2354	0.471483	0.327301	-0.14418	3	1.84E+08	F	II		IGR	shore	IGR-shore	chr3:1842	rs5332715	36;44
cg2359419	0.100768	0.452704	3.724251	0.001961	0.224708	-2.24175	0.503088	0.40232	-0.10077	2	45121446	F	II		IGR	opensea	IGR-opense		rs5745534	0;1;20;33
cg2137702	0.124007	0.7207	2.648268	0.017973	0.225076	-4.41775	0.782703	0.658696	-0.12401	22	49915303	F	II	C22orf34	Body	opensea	Body-opens		rs5381209	35
cg2331631	0.123996	0.521651	3.10725	0.007041	0.224708	-3.50896	0.583649	0.459653	-0.124	12	21606508	R	II	PYROXD1	Body	opensea	Body-opens			
cg2308006	0.114234	0.670009	3.722233	0.001969	0.224708	-2.24594	0.727125	0.612892	-0.11423	5	61028303	F	II		IGR	opensea	IGR-opense		rs7875043	30
cg2374342	0.123969	0.712263	3.639206	0.002339	0.224708	-2.41793	0.774248	0.650278	-0.12397	8	13170741	R	II	DLC1	Body	opensea	Body-opens		rs5633304	26
cg2153399	0.123797	0.406091	2.556167	0.021621	0.225763	-4.59414	0.46799	0.344193	-0.1238	12	1.29E+08	F	I	SLC15A4	Body	opensea	Body-opens		rs1461845	0;46
cg1736572	0.12346	0.734996	5.226036	9.47E-05	0.223139	0.832063	0.796727	0.673266	-0.12346	2	2.43E+08	R	II	D2HGDH	Body	opensea	Body-opens		rs1430110	49;31
cg1975577	0.114876	0.47131	3.714675	0.002	0.224708	-2.2616	0.528748	0.413872	-0.11488	6	29067386	R	II		IGR	opensea	IGR-opense		rs1813841	42;7
cg1045613	0.123361	0.575104	2.842663	0.012119	0.224708	-4.03819	0.636784	0.513423	-0.12336	9	76985951	F	II	LOC10192	TSS1500	opensea	TSS1500-op		rs5668494	0;1;5;13
cg2295218	0.123323	0.550647	3.780399	0.001745	0.224708	-2.12533	0.612308	0.488985	-0.12332	11	9091264	F	II	SCUBE2	Body	opensea	Body-opens		rs5540114	42
cg1295889	0.123083	0.625714	2.638756	0.018321	0.225076	-4.43607	0.687255	0.564172	-0.12308	2	59444755	F	II	LOC10192	TSS200	opensea	TSS200-ope		rs5606858	0;1;8;15;20;39
cg0170399	0.122949	0.787593	2.392324	0.029922	0.232532	-4.90153	0.849068	0.726119	-0.12295	14	22103310	F	II	OR10G2	TSS1500	opensea	TSS1500-op		rs2858658	0;49
cg2472936	0.104088	0.373142	3.707061	0.002032	0.224708	-2.27738	0.425186	0.321098	-0.10409	16	87194677	F	II		IGR	opensea	IGR-opense		rs5692535	0;5
cg1533221	0.122931	0.367192	2.648074	0.01798	0.225076	-4.41812	0.428658	0.305727	-0.12293	13	28555064	F	II	PRHOXNB	Body	island	Body-island	chr13:285	rs5762726	11;50
cg1884235	0.101866	0.456756	3.703095	0.002049	0.224708	-2.2856	0.507688	0.405823	-0.10187	13	97794052	F	II		IGR	opensea	IGR-opense		rs5468635	17
cg1732911	0.122825	0.59925	2.784452	0.013644	0.224708	-4.15279	0.660662	0.537837	-0.12282	20	62690171	F	II	TCEA2	5'UTR	shore	5'UTR-shore	chr20:626	rs1418279	0;4;33;51
cg1019310	0.132673	0.518197	3.693855	0.002088	0.224708	-2.30475	0.584534	0.451861	-0.13267	7	75924270	R	II		IGR	opensea	IGR-opense		rs5313143	47;28;25;21;20;3
cg0159357	0.122821	0.467335	3.285381	0.004871	0.224708	-3.14661	0.528745	0.405925	-0.12282	17	60818877	R	II	MARCH10	Body	opensea	Body-opens			
cg0207322	0.122718	0.62739	3.562086	0.002744	0.224708	-2.57745	0.688749	0.566032	-0.12272	1	3458304	R	II	MEGF6	Body	shore	Body-shore	chr1:3459	rs5357245	49;41;1
cg0229647	0.122683	0.522292	4.062105	0.000976	0.224708	-1.54094	0.583634	0.460951	-0.12268	8	1.04E+08	R	II	AZIN1-AS1	Body	opensea	Body-opens		rs5525803	32;10
cg0320315	0.122633	0.826888	2.66068	0.01753	0.225076	-4.39379	0.888205	0.765572	-0.12263	7	1.33E+08	F	II	EXOC4	Body	opensea	Body-opens		rs5524197	0;18;19
cg0906943	0.122583	0.524208	3.045256	0.008	0.224708	-3.63405	0.5855	0.462917	-0.12258	5	80584068	F	II	CKMT2-AS1	Body	opensea	Body-opens		rs5318956	5;42
cg0545138	0.122583	0.435381	4.459044	0.000434	0.224708	-0.72088	0.496672	0.37409	-0.12258	3	1.98E+08	R	II	LRCH3	Body	shore	Body-shore	chr3:1975		
cg0795386	0.122434	0.436133	5.823003	3.04E-05	0.197233	1.997493	0.49735	0.374916	-0.12243	14	20483579	F	II	OR4K14	TSS1500	opensea	TSS1500-op		rs7450995	1;9;48;51
cg1800332	0.122398	0.526877	4.225594	0.000698	0.224708	-1.20237	0.588075	0.465678	-0.1224	12	32138012	F	II	KIAA1551	Body	opensea	Body-opens		rs7694315	15;31;46
cg1415343	0.122369	0.413951	3.753117	0.001847	0.224708	-2.18191	0.475136	0.352766	-0.12237	5	1.19E+08	R	I	TNFAIP8	TSS1500	shore	TSS1500-sh	chr5:1186	rs7610611	46;40
cg1400827	0.12212	0.580268	3.377461	0.004025	0.224708	-2.9579	0.641328	0.519208	-0.12212	12	68845785	R	II	LOC10050	TSS1500	opensea	TSS1500-op		rs5639455	12
cg1692589	0.109687	0.591243	3.682187	0.002139	0.224708	-2.32892	0.646086	0.536399	-0.10969	13	1.03E+08	R	II		IGR	opensea	IGR-opense		rs5711842	1
cg2095731	0.121981	0.480526	3.364618	0.004134	0.224708	-2.98427	0.541517	0.419536	-0.12198	2	38976409	F	II	SFRS7	Body	shore	Body-shore	chr2:3897		
cg1961863	0.351387	0.390421	3.681716	0.002141	0.224708	-2.3299	0.566114	0.214727	-0.35139	17	69085963	R	II		IGR	opensea	IGR-opense		rs2012264	2;1
cg0346532	0.121932	0.353462	3.799707	0.001677	0.224708	-2.08529	0.414428	0.292496	-0.12193	6	32823078	R	II	PSMB9	Body	shore	Body-shore	chr6:3282		
cg1778568	0.121823	0.442028	3.344118	0.004313	0.224708	-3.02632	0.50294	0.381116	-0.12182	18	21322159	F	II	LAMA3	Body	opensea	Body-opens		rs5669774	26
cg0023417	0.121691	0.599052	3.382042	0.003987	0.224708	-2.94849	0.659897	0.538206	-0.12169	5	1.57E+08	R	II	ITK	Body	opensea	Body-opens		rs1396556	7;1
cg0172007	0.121514	0.496275	4.177524	0.00077	0.224708	-1.30183	0.557032	0.435518	-0.12151	12	31835278	R	II	AMN1	Body	opensea	Body-opens			
cg0613436	0.121407	0.635203	3.337169	0.004376	0.224708	-3.04057	0.695906	0.574499	-0.12141	3	1.25E+08	F	II	SLC12A8	Body	opensea	Body-opens		rs5506197	1;5;13;48
cg1498181	0.114482	0.592036	3.668772	0.0022	0.224708	-2.35671	0.649277	0.534795	-0.11448	7	96597711	F	II		IGR	opensea	IGR-opense		rs5549519	22
cg1700564	0.121372	0.564159	3.708538	0.002026	0.224708	-2.27432	0.624845	0.503473	-0.12137	7	23304848	R	II	GPNMB	Body	opensea	Body-opens			
cg0313489	0.101095	0.637794	3.665789	0.002213	0.224708	-2.36289	0.688341	0.587246	-0.10109	7	66343288	R	II		IGR	opensea	IGR-opense		rs5752921	1
cg2256827	0.121227	0.437832	2.784727	0.013636	0.224708	-4.15225	0.498446	0.377219	-0.12123	3	69811867	R	II	MITF	TSS1500	opensea	TSS1500-op		rs2014262	37

cg2685595	0.121197	0.534738	4.337639	0.000555	0.224708	-0.97092	0.595337	0.47414	-0.1212	8	93020657	F	II	RUNX1T1	Body	opensea	Body-opens		rs5326829	14
cg2581860	0.107958	0.553176	3.658974	0.002245	0.224708	-2.377	0.607155	0.499197	-0.10796	2	1.01E+08	R	II		IGR	opensea	IGR-opense		rs1379590	51
cg0941579	0.116012	0.483807	3.657801	0.00225	0.224708	-2.37943	0.541813	0.425801	-0.11601	3	23800187	F	II		IGR	opensea	IGR-opense		rs5727585	4;17
cg1888038	0.121126	0.66014	4.862354	0.000193	0.224708	0.102362	0.720703	0.599577	-0.12113	6	32365113	R	II	BTNL2	Body	opensea	Body-opens		rs1178304	40;29;24;22;15;2;1
cg2314547	0.121074	0.371817	3.127715	0.006749	0.224708	-3.46754	0.432354	0.31128	-0.12107	3	66522924	F	II	LRIG1	Body	opensea	Body-opens		rs5344157	1;23
cg2540564	0.121022	0.580172	3.756669	0.001833	0.224708	-2.17454	0.640683	0.519662	-0.12102	2	2.18E+08	F	II	DIRC3	Body	opensea	Body-opens		rs5431954	11;22
cg1587459	0.103145	0.515548	3.649092	0.002291	0.224708	-2.39746	0.56712	0.463975	-0.10314	16	29032168	R	II		IGR	opensea	IGR-opense		rs1454786	1
cg1458518	0.121019	0.400551	3.349867	0.004262	0.224708	-3.01453	0.46106	0.340041	-0.12102	12	1.05E+08	F	II	CHST11	Body	opensea	Body-opens		rs5773223	1;27;35
cg0418843	0.120959	0.515451	4.005895	0.001096	0.224708	-1.6575	0.57593	0.454971	-0.12096	20	24500288	F	II	SYNDIG1	5'UTR	opensea	5'UTR-open		rs5491396	1
cg1295081	0.120898	0.206112	3.648305	0.002295	0.224708	-2.39909	0.266561	0.145662	-0.1209	7	1198977	F	I	ZFAND2A	5'UTR	island	5'UTR-islan	chr7:1198	rs5967624	8;31
cg1322110	0.120834	0.803929	3.849899	0.001512	0.224708	-1.98116	0.864346	0.743511	-0.12083	12	52846088	R	II	KRT6B	TSS200	opensea	TSS200-ope		rs1451551	50;46;2
cg2670300	0.120798	0.52028	2.942515	0.00988	0.224708	-3.83996	0.580679	0.459881	-0.1208	15	55834750	F	II	PYGO1	3'UTR	opensea	3'UTR-open		rs1849950	11;18;19
cg1203661	0.120759	0.470584	2.990717	0.00895	0.224708	-3.74359	0.530964	0.410205	-0.12076	4	80929171	R	II	ANTXR2	Body	opensea	Body-opens		rs5659504	28;2;1
cg2590242	0.120688	0.579656	3.689538	0.002107	0.224708	-2.31369	0.64	0.519312	-0.12069	12	94773488	R	II	CEP83	Body	opensea	Body-opens			
cg0458593	0.12064	0.641353	4.227151	0.000696	0.224708	-1.19915	0.701673	0.581033	-0.12064	14	1.02E+08	R	II	MIR376B	TSS1500	opensea	TSS1500-op		rs7270057	1
cg0871134	0.120619	0.481012	3.347386	0.004284	0.224708	-3.01962	0.541322	0.420703	-0.12062	9	1.24E+08	F	II	TRAF1	Body	opensea	Body-opens		rs1850025	1;34
cg1422675	0.120415	0.612572	5.498698	5.61E-05	0.219272	1.369695	0.672779	0.552364	-0.12042	5	1.08E+08	F	II	FBXL17	Body	opensea	Body-opens		rs5742852	0;2;18;19
cg2489933	0.120403	0.455581	3.217401	0.005608	0.224708	-3.28536	0.515783	0.39538	-0.1204	3	1.68E+08	F	II	GOLIM4	Body	opensea	Body-opens		rs5725839	31
cg1665179	0.115482	0.44306	3.617238	0.002448	0.224708	-2.4634	0.500801	0.385319	-0.11548	18	715830	F	II		IGR	shelf	IGR-shelf	chr18:712	rs5305979	0
cg0174218	0.12033	0.53084	3.186691	0.005975	0.224708	-3.34786	0.591005	0.470675	-0.12033	9	6566568	F	II	GLDC	Body	opensea	Body-opens		rs1821660	7;34;38;48
cg1941280	0.102095	0.605677	3.614433	0.002462	0.224708	-2.4692	0.656725	0.55463	-0.10209	1	69916760	F	II		IGR	opensea	IGR-opense		rs1503112	22;23;51
cg1822589	0.113858	0.433692	3.613754	0.002465	0.224708	-2.4706	0.490621	0.376763	-0.11386	6	80087320	F	II		IGR	opensea	IGR-opense		rs1410107	1;41
cg1154971	0.120275	0.52597	2.653727	0.017777	0.225076	-4.40722	0.586107	0.465832	-0.12028	2	55001373	F	II	EML6	Body	opensea	Body-opens		rs5554022	0;32
cg0216474	0.127796	0.396393	3.613128	0.002469	0.224708	-2.4719	0.460291	0.332495	-0.1278	6	76206048	F	II		IGR	opensea	IGR-opense		rs1172187	1
cg1316611	0.125656	0.458589	3.612198	0.002473	0.224708	-2.47382	0.521417	0.395761	-0.12566	3	1.14E+08	F	II		IGR	opensea	IGR-opense		rs5403381	12
cg0413740	0.104059	0.390448	3.610668	0.002481	0.224708	-2.47699	0.442477	0.338419	-0.10406	14	62414451	R	II		IGR	opensea	IGR-opense		rs3750408	16
cg0278141	0.108035	0.467811	3.607915	0.002495	0.224708	-2.48269	0.521829	0.413794	-0.10804	6	27302893	F	II		IGR	opensea	IGR-opense		rs5355633	0;26
cg0115452	0.120199	0.679478	2.753247	0.014537	0.224708	-4.21391	0.739577	0.619379	-0.1202	6	1.52E+08	F	II	AKAP12	Body	shore	Body-shore	chr6:1516	rs1422944	0;23
cg1387843	0.102113	0.681072	3.606134	0.002505	0.224708	-2.48637	0.732128	0.630015	-0.10211	12	66409494	F	II		IGR	opensea	IGR-opense		rs5509891	0;23;37
cg0466162	0.10317	0.645958	3.605529	0.002508	0.224708	-2.48762	0.697543	0.594373	-0.10317	12	28174838	R	II		IGR	opensea	IGR-opense		rs5546425	40;23
cg1931836	0.188241	0.642707	3.602957	0.002521	0.224708	-2.49294	0.736827	0.548587	-0.18824	6	55774999	R	II		IGR	opensea	IGR-opense		rs5306838	19
cg0865866	0.120045	0.688609	3.417919	0.003701	0.224708	-2.87474	0.748631	0.628586	-0.12004	12	1.09E+08	F	II	SVOP	Body	opensea	Body-opens		rs5665863	19;45
cg0024209	0.114414	0.517534	3.600466	0.002534	0.224708	-2.49809	0.574741	0.460327	-0.11441	1	2.36E+08	F	II		IGR	opensea	IGR-opense		rs5608731	0;21;26
cg1448406	0.125739	0.56639	3.600164	0.002536	0.224708	-2.49872	0.629259	0.50352	-0.12574	13	1.07E+08	F	II		IGR	opensea	IGR-opense			
cg1753736	0.115014	0.584081	3.600126	0.002536	0.224708	-2.4988	0.641588	0.526574	-0.11501	4	75260310	F	II		IGR	opensea	IGR-opense		rs5771241	1;2;22;27
cg0530060	0.133225	0.630232	3.59934	0.00254	0.224708	-2.50042	0.696844	0.56362	-0.13322	12	52235472	R	II		IGR	opensea	IGR-opense		rs1258068	14;1
cg1527215	0.120026	0.474538	3.051983	0.00789	0.224708	-3.62051	0.534551	0.414525	-0.12003	4	38525916	F	II	LINC01258	TSS1500	opensea	TSS1500-op		rs7313995	38
cg0136902	0.119995	0.468382	3.248575	0.005257	0.224708	-3.2218	0.52838	0.408385	-0.12	10	11133829	R	II	CELF2	Body	opensea	Body-opens		rs1147858	37;8
cg1788004	0.119328	0.671245	3.597517	0.00255	0.224708	-2.50419	0.730909	0.611581	-0.11933	12	1.11E+08	R	II		IGR	opensea	IGR-opense		rs1413333	36;31;28
cg0726797	0.119916	0.461702	3.188491	0.005953	0.224708	-3.3442	0.52166	0.401744	-0.11992	8	62539945	F	II	ASPH	Body	opensea	Body-opens		rs1920400	0;24;27;51
cg0701959	0.119877	0.457177	3.25064	0.005235	0.224708	-3.21758	0.517115	0.397239	-0.11988	17	34207481	R	II	CCL5	TSS200	opensea	TSS200-ope		rs5359131	1
cg0449163	0.119785	0.533148	3.270007	0.005029	0.224708	-3.17803	0.593041	0.473255	-0.11979	2	1.74E+08	F	II	ZAK	Body	opensea	Body-opens		rs1116036	0;21
cg2437621	0.119674	0.49944	3.035146	0.008168	0.224708	-3.65439	0.559277	0.439603	-0.11967	19	38918135	R	II	RASGRP4	TSS1500	opensea	TSS1500-op		rs5374606	22;8;2
cg0205252	0.113698	0.279964	3.59211	0.002579	0.224708	-2.51538	0.336813	0.223115	-0.1137	18	61662355	F	II		IGR	opensea	IGR-opense		rs5280166	0;1;14
cg1828745	0.109493	0.451511	3.590317	0.002588	0.224708	-2.51908	0.506258	0.396765	-0.10949	6	1.22E+08	R	II		IGR	opensea	IGR-opense		rs5434158	19;12;2
cg1017446	0.124973	0.658007	3.587024	0.002606	0.224708	-2.52589	0.720493	0.59552	-0.12497	8	11739341	F	II		IGR	opensea	IGR-opense		rs1831214	21;29;47
cg0013949	0.119635	0.72023	4.00045	0.001108	0.224708	-1.66879	0.780048	0.660413	-0.11963	3	52731124	F	II	GLT8D1	Body	opensea	Body-opens			
cg1333384	0.146129	0.604235	3.583105	0.002627	0.224708	-2.534	0.677299	0.53117	-0.14613	13	1.1E+08	R	II		IGR	opensea	IGR-opense		rs7764741	31
cg2321105	0.119629	0.453943	3.109822	0.007003	0.224708	-3.50376	0.513758	0.394128	-0.11963	3	1.68E+08	F	II	EGFEM1P	Body	opensea	Body-opens		rs5340285	0;26;34;41
cg1403406	0.112506	0.512391	3.580677	0.00264	0.224708	-2.53902	0.568644	0.456138	-0.11251	22	39282561	R	II		IGR	opensea	IGR-opense		rs5630978	9
cg1812492	0.103991	0.359764	3.577177	0.00266	0.224708	-2.54625	0.411759	0.307768	-0.10399	5	73294360	F	II		IGR	opensea	IGR-opense			
cg2208246	0.119413	0.605568	3.131002	0.006704	0.224708	-3.46089	0.665274	0.545862	-0.11941	14	24540415	R	II	CPNE6	TSS1500	opensea	TSS1500-op		rs5368055	49;17;7;2
cg0750432	0.119396	0.566217	3.594219	0.002567	0.224708	-2.51101	0.625915	0.506519	-0.1194	2	98860544	F	II	VWA3B	Body	opensea	Body-opens		rs7773337	18;29
cg1663493	0.119383	0.559625	3.041793	0.008057	0.224708	-3.64102	0.619316	0.499933	-0.11938	10	35033892	F	II	PARD3	Body	opensea	Body-opens		rs1881855	1
cg1005317	0.119334	0.526166	3.250818	0.005233	0.224708	-3.21722	0.585833	0.466499	-0.11933	20	48924790	R	II	LINC01270	Body	opensea	Body-opens		rs5403083	48
cg0936781	0.119271	0.595166	3.013842	0.008534	0.224708	-3.6972	0.654802	0.535531	-0.11927	21	30463648	R	II	MAP3K7CL	TSS1500	opensea	TSS1500-op		rs8131128	25;17;1
cg0406541	0.119213	0.55413	3.930209	0.001281	0.224708	-1.81452	0.613737	0.494524	-0.11921	3	62799139	R	II	CADPS	Body	opensea	Body-opens		rs5713159	45;31;19;7;2
cg1583730	0.140129	0.589742	3.570344	0.002698	0.224708	-2.56038	0.659807	0.519678	-0.14013	6	32427751	F	II		IGR	opensea	IGR-opense		rs3771676	1;7;38
cg2672640	0.119197	0.756618	3.722539	0.001968	0.224708	-2.2453	0.816217	0.69702	-0.1192	2	46948107	R	II	SOCS5	5'UTR	opensea	5'UTR-open		rs1189315	34;1
cg1053671	0.119106	0.531696	3.201755	0.005792	0.224708	-3.31722	0.591249	0.472143	-0.11911	2	86089041	R	II	ST3GAL5	Body	opensea	Body-opens		rs1436850	1
cg0993190	0.119052	0.491956	3.067551	0.007641	0.224708	-3.58913	0.551482	0.43243	-0.11905	6	74160206	F	II	C6orf150	Body	shore	Body-shore	chr6:7416	rs5778603	46
cg0219385	0.119022	0.497294	3.568925	0.002706	0.224708	-2.56331	0.556805	0.437783	-0.11902	18	77960683	R	II	PARD6G	Body	opensea	Body-opens		rs1500228	46
cg0288308	0.103795	0.508035	3.566155	0.002721	0.224708	-2.56904	0.559932	0.456137	-0.10379	10	1.3E+08	R	II		IGR	opensea	IGR-opense		rs1436066	1
cg1407444	0.118952	0.798169	2.559666	0.021471	0.225654	-4.58749	0.857645	0.738692	-0.11895	5	1.49E+08	F	II	ABLIM3	Body	opensea	Body-opens		rs1429891	0;1;37

cg2082131	0.118789	0.490718	3.632303	0.002372	0.224708	-2.43222	0.550112	0.431324	-0.11879	2	10950732	F	II	PDIA6	Body	shore	Body-shore	chr2:10952	rs1136054	10
cg1776538	0.118781	0.58248	3.18025	0.006055	0.224708	-3.36095	0.64187	0.52309	-0.11878	12	1.17E+08	F	II	FBXW8	Body	opensea	Body-opens		rs1172971	8
cg0019358	0.118683	0.483295	2.85877	0.011727	0.224708	-4.00635	0.542636	0.423953	-0.11868	1	77879926	F	II	AK5	Body	opensea	Body-opens		rs7861829	0;1;50
cg0644219	0.118619	0.365143	3.959921	0.001205	0.224708	-1.75287	0.424452	0.305833	-0.11862	1	92414344	F	II	BRDT	TSS1500	shore	TSS1500-sh	chr1:92414		
cg0500709	0.11861	0.592339	3.338881	0.00436	0.224708	-3.03706	0.651644	0.533034	-0.11861	16	77322779	R	II	ADAMTS18	Body	opensea	Body-opens		rs1164331	24;1
cg1870074	0.118583	0.3955	3.105124	0.007071	0.224708	-3.51326	0.454792	0.336208	-0.11858	12	1.13E+08	R	II	NAA25	Body	opensea	Body-opens			
cg1069440	0.11856	0.53022	3.91923	0.00131	0.224708	-1.8373	0.5895	0.47094	-0.11856	7	1.44E+08	F	II	OR2F1	TSS1500	opensea	TSS1500-op		rs1811382	1;23;51
cg0364500	0.118551	0.500596	3.011614	0.008573	0.224708	-3.70168	0.559871	0.44132	-0.11855	7	1.02E+08	F	II	FBXL13	Body	opensea	Body-opens		rs5554587	35;46
cg1182179	0.107919	0.393298	3.555977	0.002779	0.224708	-2.59007	0.447257	0.339339	-0.10792	22	47611604	R	II		IGR	shelf	IGR-shelf	chr22:4760	rs1923492	21;2;1
cg1743667	0.118484	0.532674	3.257154	0.005165	0.224708	-3.20428	0.591916	0.473432	-0.11848	4	1.06E+08	F	II	ARHGEF38	Body	opensea	Body-opens		rs1405735	8;49;50
cg2760128	0.118396	0.580377	2.310411	0.035126	0.237348	-5.05176	0.639575	0.521178	-0.1184	2	39492724	F	II	MAP4K3	Body	opensea	Body-opens		rs5345208	7
cg0679569	0.11831	0.663268	2.459423	0.026211	0.229328	-4.7767	0.722423	0.604113	-0.11831	16	50648887	R	II	NKD1	Body	opensea	Body-opens		rs5569132	39;1
cg0038025	0.118277	0.783425	2.48378	0.024975	0.228296	-4.73102	0.842564	0.724287	-0.11828	7	48465916	R	II	ABCA13	Body	opensea	Body-opens		rs7561903	33
cg1625865	0.1182	0.504943	2.816914	0.012772	0.224708	-4.08898	0.564043	0.445843	-0.1182	3	29338542	F	II	RBMS3	Body	opensea	Body-opens		rs8023379	0;27;51
cg1492584	0.127961	0.448771	3.550885	0.002809	0.224708	-2.60059	0.512751	0.384791	-0.12796	21	35790214	F	II		IGR	opensea	IGR-opense		rs5487386	6;14;32;34
cg0983683	0.118141	0.561284	3.571669	0.00269	0.224708	-2.55764	0.620354	0.502213	-0.11814	18	77723602	R	II	HSBP1L1	TSS1500	shore	TSS1500-sh	chr18:7772	rs5501489	21;4
cg0979028	0.118129	0.668254	3.136583	0.006627	0.224708	-3.44958	0.727319	0.60919	-0.11813	7	1.24E+08	R	II	LOC101928	Body	opensea	Body-opens		rs7843655	24;1
cg0267253	0.118083	0.579191	3.343797	0.004316	0.224708	-3.02698	0.638233	0.520149	-0.11808	11	1.21E+08	F	II	GRIK4	Body	opensea	Body-opens		rs1402963	0;6
cg0501795	0.118026	0.564838	3.104168	0.007085	0.224708	-3.5152	0.623851	0.505825	-0.11803	3	1.51E+08	F	II	GPR87	5'UTR	opensea	5'UTR-open		rs1192614	31;36
cg1723928	0.104934	0.710805	3.546917	0.002832	0.224708	-2.60879	0.763272	0.658338	-0.10493	17	29911092	R	II		IGR	opensea	IGR-opense		rs1882077	1
cg1268281	0.117997	0.45417	2.891762	0.010962	0.224708	-3.94097	0.513169	0.395172	-0.118	2	1.69E+08	R	II	STK39	Body	opensea	Body-opens		rs1861657	42
cg2380665	0.117954	0.48803	3.636285	0.002353	0.224708	-2.42398	0.547008	0.429053	-0.11795	7	1.11E+08	R	II	IMMP2L	Body	opensea	Body-opens		rs1150644	46
cg2399336	0.117826	0.452816	3.356506	0.004204	0.224708	-3.00091	0.511729	0.393903	-0.11783	14	50086035	R	II	MGAT2	TSS1500	shore	TSS1500-sh	chr14:5008	rs1482780	47
cg1100313	0.117699	0.457593	3.141425	0.006561	0.224708	-3.43976	0.516442	0.398743	-0.1177	1	1.59E+08	R	II	AIM2	5'UTR	opensea	5'UTR-open		rs4126445	15;10;2;1
cg2253472	0.106899	0.527086	3.539665	0.002875	0.224708	-2.62377	0.580535	0.473636	-0.1069	13	78358711	F	II		IGR	opensea	IGR-opense		rs1870320	1;37
cg1923518	0.117613	0.4156	3.288995	0.004835	0.224708	-3.13922	0.474407	0.356793	-0.11761	3	1.56E+08	R	II	KCNAB1-AS	TSS200	opensea	TSS200-ope			
cg0245852	0.103838	0.630432	3.537776	0.002886	0.224708	-2.62767	0.682351	0.578513	-0.10384	14	24166813	R	II		IGR	opensea	IGR-opense		rs6071922	50;35;1
cg1237640	0.117523	0.452583	3.240639	0.005344	0.224708	-3.23799	0.511344	0.393821	-0.11752	14	21236306	R	II	EDDM3B	TSS1500	opensea	TSS1500-op		rs1144170	33;19;1
cg1114618	0.110082	0.663478	3.537552	0.002888	0.224708	-2.62813	0.718519	0.608437	-0.11008	15	39593169	F	II		IGR	opensea	IGR-opense		rs3708730	1;26
cg1976647	0.117488	0.368341	3.00675	0.00866	0.224708	-3.71144	0.427085	0.309597	-0.11749	11	2322802	R	II	C11orf21	Body	opensea	Body-opens		rs1422473	22;1
cg0448010	0.117477	0.775002	2.627521	0.01874	0.225209	-4.45769	0.83374	0.716263	-0.11748	5	72934606	F	II	RGNEF	5'UTR	opensea	5'UTR-open		rs5649205	17;28;32;46
cg1405869	0.104953	0.577016	3.536365	0.002895	0.224708	-2.63058	0.629492	0.524539	-0.10495	13	63200547	R	II		IGR	opensea	IGR-opense		rs5633557	22
cg2445215	0.117453	0.503768	3.203825	0.005767	0.224708	-3.313	0.562495	0.445041	-0.11745	2	12433867	R	II	LOC100506	Body	opensea	Body-opens		rs5563541	1
cg0322432	0.117447	0.531127	3.016576	0.008486	0.224708	-3.69171	0.589851	0.472404	-0.11745	3	1.57E+08	R	II	CCNL1	TSS1500	shore	TSS1500-sh	chr3:15687	rs5757058	46
cg0747531	0.117398	0.664825	2.890233	0.010997	0.224708	-3.944	0.723524	0.606126	-0.1174	5	54717280	R	II	SKIV2L2	Body	opensea	Body-opens		rs3716455	44
cg2249596	0.116912	0.645868	3.531041	0.002927	0.224708	-2.64157	0.704324	0.587411	-0.11691	2	5622155	R	II		IGR	opensea	IGR-opense		rs5974547	20;1
cg0074614	0.120102	0.604945	3.529322	0.002937	0.224708	-2.64512	0.664996	0.544893	-0.1201	20	39551744	F	II		IGR	opensea	IGR-opense		rs1864539	47
cg2759388	0.117357	0.736994	2.66792	0.017275	0.225017	-4.3798	0.795672	0.678315	-0.11736	4	84194117	R	II	COQ2	Body	opensea	Body-opens		rs5528840	4
cg1974334	0.10527	0.547638	3.527846	0.002946	0.224708	-2.64817	0.600272	0.495003	-0.10527	2	317938	R	II		IGR	shelf	IGR-shelf	chr2:31482	rs7792054	29;4;1
cg0854672	0.117311	0.523845	3.548667	0.002822	0.224708	-2.60517	0.5825	0.465189	-0.11731	10	67705174	F	II	CTNNA3	Body	opensea	Body-opens		rs5763577	1;33
cg0141092	0.117261	0.664043	3.364822	0.004132	0.224708	-2.98385	0.722673	0.605413	-0.11726	2	1.92E+08	R	II	MYO1B	Body	opensea	Body-opens		rs1488319	51;31
cg2753727	0.117238	0.394641	3.349786	0.004263	0.224708	-3.0147	0.45326	0.336021	-0.11724	3	1.48E+08	R	II	AGTR1	5'UTR	opensea	5'UTR-open		rs5446051	50;35
cg1025633	0.117213	0.65397	3.496339	0.003145	0.224708	-2.71319	0.712576	0.595363	-0.11721	17	76801775	F	II	USP36	Body	shelf	Body-shelf	chr17:7679	rs1127790	9;24;33
cg0842867	0.11718	0.414071	3.671821	0.002186	0.224708	-2.35039	0.472661	0.355481	-0.11718	3	1.77E+08	F	II	LINC00578	Body	opensea	Body-opens		rs1506197	9;32
cg0427662	0.117046	0.378392	2.876872	0.011301	0.224708	-3.97051	0.436915	0.319869	-0.11705	17	57918500	F	II	MIR21	TSS200	opensea	TSS200-ope		rs5586536	0;9;25;28;28;33
cg0625562	0.106371	0.676299	3.513719	0.003034	0.224708	-2.67733	0.729484	0.623113	-0.10637	5	1.7E+08	F	II		IGR	opensea	IGR-opense		rs1465923	24;43;48
cg1460992	0.101683	0.469301	3.5131	0.003038	0.224708	-2.67861	0.520142	0.418459	-0.10168	1	2.2E+08	R	II		IGR	opensea	IGR-opense			
cg0743670	0.117004	0.621476	3.129553	0.006724	0.224708	-3.46382	0.679978	0.562974	-0.117	10	88717623	R	II	MMRN2	TSS200	opensea	TSS200-ope		rs1148980	1
cg1096005	0.112931	0.235995	3.506177	0.003082	0.224708	-2.6929	0.29246	0.179529	-0.11293	6	28664226	R	II		IGR	opensea	IGR-opense		rs1689421	15
cg2127873	0.119227	0.61689	3.502982	0.003102	0.224708	-2.69949	0.676503	0.557276	-0.11923	4	38797651	F	II		IGR	opensea	IGR-opense		rs1479840	0;12;35
cg1746443	0.116972	0.602111	3.938591	0.001259	0.224708	-1.79713	0.660597	0.543625	-0.11697	1	1.85E+08	R	II	FAM129A	Body	opensea	Body-opens		rs4651242	41;27;22
cg0316539	0.116939	0.421855	2.988481	0.008991	0.224708	-3.74807	0.480324	0.363385	-0.11694	12	1.2E+08	R	II	CIT	Body	opensea	Body-opens		rs5832550	31;4;1
cg1782217	0.126423	0.539151	3.496976	0.003141	0.224708	-2.71188	0.602363	0.47594	-0.12642	2	1.45E+08	F	II		IGR	opensea	IGR-opense		rs5737327	0
cg0179424	0.116895	0.502179	3.484195	0.003226	0.224708	-2.73824	0.560627	0.443731	-0.1169	8	1.46E+08	R	II	KIAA1688	TSS1500	opensea	TSS1500-op			
cg0741815	0.116876	0.430014	2.947834	0.009773	0.224708	-3.82935	0.488452	0.371576	-0.11688	19	13056052	R	II	RAD23A	TSS1500	shore	TSS1500-sh	chr19:1305		
cg2469968	0.118372	0.61342	3.491043	0.00318	0.224708	-2.72412	0.672606	0.554234	-0.11837	1	95268325	F	II		IGR	opensea	IGR-opense			
cg1610107	0.116791	0.524272	3.728394	0.001944	0.224708	-2.23317	0.582667	0.465877	-0.11679	10	1.3E+08	F	II	CLRN3	3'UTR	opensea	3'UTR-open		rs2296785	27;40
cg0805963	0.116657	0.403878	2.825057	0.012562	0.224708	-4.07293	0.462206	0.345549	-0.11666	8	6274876	R	II	MCPH1	Body	opensea	Body-opens		rs5367941	38
cg0151816	0.107236	0.655261	3.487994	0.0032	0.224708	-2.7304	0.708879	0.601643	-0.10724	6	92828798	F	II		IGR	opensea	IGR-opense		rs1174980	1;3;44
cg1975451	0.116549	0.562559	3.472231	0.003307	0.224708	-2.7629	0.620833	0.504284	-0.11655	2	2.25E+08	F	II	WDFY1	ExonBnd	opensea	ExonBnd-op		rs1407775	0;2;6
cg1912124	0.116545	0.660466	3.567931	0.002711	0.224708	-2.56537	0.718739	0.602194	-0.11654	1	89266282	R	II	PKN2	Body	opensea	Body-opens		rs5700623	48;33
cg1366429	0.114154	0.483352	3.484723	0.003222	0.224708	-2.73715	0.54043	0.426275	-0.11415	11	9587813	F	II		IGR	opensea	IGR-opense		rs1502162	0
cg1264556	0.116369	0.443233	3.220473	0.005572	0.224708	-3.2791	0.501418	0.385049	-0.11637	1	51339561	R	II	FAF1	Body	opensea	Body-opens		rs1921246	37;2
cg1749995	0.126693	0.481648	3.483926	0.003227	0.224708	-2.73879	0.544994	0.418301	-0.12669	12	40562179	F	II		IGR	opensea	IGR-opense		rs5725517	0;15;39

cg1558030	0.116335	0.52214	2.128001	0.049902	0.255014	-5.37686	0.580307	0.463972	-0.11633	1	46814106	R	II	NSUN4	Body	opensea	Body-opens		rs6657520	16
cg1372761	0.116313	0.588405	3.323765	0.004499	0.224708	-3.06804	0.646562	0.530249	-0.11631	19	7145317	F	II	INSR	Body	shelf	Body-shelf	chr19:714		
cg1803155	0.116268	0.439998	3.29494	0.004776	0.224708	-3.12706	0.498132	0.381864	-0.11627	11	1.21E+08	R	II	GRIK4	Body	opensea	Body-opens		rs5408820	37;25
cg1387375	0.123273	0.444845	3.47168	0.00331	0.224708	-2.76404	0.506482	0.383209	-0.12327	2	317826	R	II		IGR	shelf	IGR-shelf	chr2:3148	rs5515383	33;13
cg0265823	0.116122	0.442684	3.073358	0.00755	0.224708	-3.57742	0.500745	0.384623	-0.11612	2	2.25E+08	R	II	CUL3	TSS1500	opensea	TSS1500-op			
cg1555935	0.116109	0.551675	2.426717	0.027962	0.230664	-4.83774	0.609729	0.49362	-0.11611	18	74785799	R	II	MBP	Body	opensea	Body-opens		rs5493903	23;18;13;2
cg2474550	0.116104	0.506427	2.980528	0.009139	0.224708	-3.76399	0.564479	0.448374	-0.1161	4	88728805	R	II	IBSP	Body	opensea	Body-opens		rs5392891	47;21;14;1
cg0966597	0.116084	0.550169	3.153867	0.006395	0.224708	-3.41453	0.608211	0.492127	-0.11608	2	1.73E+08	F	II	SLC25A12	Body	opensea	Body-opens		rs5507590	0
cg2741532	0.141973	0.763228	3.465732	0.003352	0.224708	-2.7763	0.834214	0.692241	-0.14197	22	17901078	R	II		IGR	shelf	IGR-shelf	chr22:178	rs1440106	18;4;1
cg1113124	0.106324	0.615155	3.464415	0.003361	0.224708	-2.77901	0.668317	0.561993	-0.10632	20	1396853	F	II		IGR	opensea	IGR-opense		rs1483980	0;21;34
cg1907149	0.109747	0.565333	3.462494	0.003374	0.224708	-2.78297	0.620207	0.51046	-0.10975	1	63604560	F	II		IGR	opensea	IGR-opense		rs1118257	1
cg1807393	0.116068	0.585454	3.271881	0.00501	0.224708	-3.1742	0.643487	0.52742	-0.11607	4	1.01E+08	R	II	DAPP1	3'UTR	opensea	3'UTR-open			
cg1167887	0.120138	0.59193	3.461347	0.003382	0.224708	-2.78533	0.651999	0.531861	-0.12014	12	26340149	F	II		IGR	opensea	IGR-opense		rs5736756	0;14;19;20
cg2572919	0.101604	0.48406	3.460689	0.003387	0.224708	-2.78669	0.534862	0.433258	-0.1016	1	17520216	R	II		IGR	opensea	IGR-opense			
cg1060127	0.100489	0.484787	3.460355	0.003389	0.224708	-2.78738	0.535032	0.434543	-0.10049	1	1.1E+08	R	II		IGR	opensea	IGR-opense		rs1869738	46
cg1749653	0.101546	0.580047	3.460327	0.003389	0.224708	-2.78743	0.63082	0.529274	-0.10155	3	59712199	R	II		IGR	opensea	IGR-opense		rs1456800	11;4
cg2480926	0.115893	0.45934	3.545446	0.002841	0.224708	-2.61183	0.517287	0.401394	-0.11589	17	74260692	F	II	FAM100B	TSS1500	shore	TSS1500-sh	chr17:742	rs5638810	50
cg0141512	0.104509	0.563809	3.456138	0.003419	0.224708	-2.79606	0.616063	0.511554	-0.10451	2	85929746	R	II		IGR	opensea	IGR-opense		rs1829442	43;1
cg0671774	0.112762	0.553831	3.455396	0.003424	0.224708	-2.79759	0.610212	0.49745	-0.11276	8	23626859	F	II		IGR	opensea	IGR-opense		rs5431190	0;1;4;46
cg2202644	0.115786	0.64401	2.699524	0.016207	0.224896	-4.31857	0.701903	0.586117	-0.11579	6	17442621	R	II	CAP2	Body	opensea	Body-opens		rs5330808	26;19
cg0859126	0.115756	0.480498	2.757732	0.014405	0.224708	-4.20514	0.538376	0.42262	-0.11576	17	56082867	F	I	SFRS1	3'UTR	shore	3'UTR-shore	chr17:560	rs1420721	41
cg2373441	0.132174	0.567622	3.45046	0.003459	0.224708	-2.80776	0.63371	0.501535	-0.13217	13	61221316	R	II		IGR	opensea	IGR-opense		rs1423965	38;10;3
cg2251422	0.115743	0.652221	3.471427	0.003312	0.224708	-2.76456	0.710092	0.594349	-0.11574	1	94118054	R	II	BCAR3	Body	opensea	Body-opens		rs1851198	18;2
cg0178509	0.115731	0.601335	3.098783	0.007165	0.224708	-3.52608	0.6592	0.543469	-0.11573	8	22756144	F	II	PEBP4	Body	opensea	Body-opens		rs1415635	1;45;48;50
cg1074314	0.115725	0.622941	3.439029	0.003543	0.224708	-2.8313	0.680804	0.565078	-0.11573	13	94446931	F	II	GPC6	Body	opensea	Body-opens		rs5623907	1;8;35
cg1265858	0.115705	0.357857	5.320879	7.88E-05	0.220729	1.020044	0.415709	0.300004	-0.11571	10	26736531	F	II	APBB1IP	5'UTR	opensea	5'UTR-open			
cg0278107	0.115631	0.428946	3.574725	0.002673	0.224708	-2.55132	0.486761	0.37113	-0.11563	13	1.01E+08	F	I	GGACT	TSS200	island	TSS200-isla	chr13:101		
cg1083838	0.115596	0.627221	3.1988	0.005827	0.224708	-3.32323	0.685019	0.569423	-0.1156	10	1156113	R	I	WDR37	Body	opensea	Body-opens		rs5400188	35;6
cg1362024	0.115441	0.44105	3.018349	0.008455	0.224708	-3.68815	0.49877	0.38333	-0.11544	9	1.26E+08	F	II	DENND1A	Body	opensea	Body-opens		rs5630428	48
cg1391671	0.115429	0.769174	2.363534	0.031662	0.234067	-4.95461	0.826888	0.711459	-0.11543	2	1.29E+08	F	II	WDR33	5'UTR	shelf	5'UTR-shelf	chr2:1285	rs6430953	25;42
cg1270236	0.115374	0.554227	3.109767	0.007004	0.224708	-3.50387	0.611914	0.49654	-0.11537	1	86035283	R	II	DDAH1	5'UTR	opensea	5'UTR-open		rs5689986	29;15;1
cg1604352	0.123887	0.688888	3.443092	0.003513	0.224708	-2.82293	0.750832	0.626945	-0.12389	6	47155663	F	II		IGR	opensea	IGR-opense		rs5341999	1;10;11;23;33
cg0954940	0.115374	0.764056	3.077863	0.00748	0.224708	-3.56833	0.821742	0.706369	-0.11537	8	10331768	R	II	LINCR-000	TSS1500	opensea	TSS1500-op		rs1811592	26;16;2;1
cg2012665	0.115167	0.500018	3.270707	0.005022	0.224708	-3.1766	0.557601	0.442434	-0.11517	17	76801712	F	II	USP36	Body	shelf	Body-shelf	chr17:767	rs5510390	1;40
cg2478404	0.109244	0.410037	3.440119	0.003534	0.224708	-2.82905	0.464659	0.355415	-0.10924	3	33034475	F	II		IGR	opensea	IGR-opense		rs5465351	1;4;25;36;49
cg1626593	0.115161	0.45097	3.193281	0.005894	0.224708	-3.33446	0.50855	0.393389	-0.11516	10	1.12E+08	R	II	RBM20	Body	opensea	Body-opens		rs5287368	41
cg1820491	0.115133	0.479797	2.787121	0.01357	0.224708	-4.14755	0.537364	0.422231	-0.11513	10	1.06E+08	F	II	CFAP43	Body	opensea	Body-opens		rs1163266	0
cg0411410	0.110855	0.432699	3.43891	0.003543	0.224708	-2.83154	0.488126	0.377272	-0.11085	3	13339321	F	II		IGR	opensea	IGR-opense		rs6058874	0;4
cg0238449	0.115132	0.558944	3.345507	0.004301	0.224708	-3.02348	0.61651	0.501378	-0.11513	1	1.62E+08	R	II	NOS1AP	Body	opensea	Body-opens			
cg2518976	0.115106	0.549365	3.388084	0.003937	0.224708	-2.93607	0.606918	0.491812	-0.11511	6	1.12E+08	R	II	FYN	5'UTR	opensea	5'UTR-open		rs1916832	41;17;14;11
cg1810102	0.115102	0.802806	2.925624	0.010228	0.224708	-3.87363	0.860357	0.745255	-0.1151	4	71226205	F	II	SMR3A	TSS1500	opensea	TSS1500-op		rs1000779	0
cg1868999	0.115032	0.543049	4.153719	0.000809	0.224708	-1.35111	0.600565	0.485533	-0.11503	5	55164885	R	II	IL31RA	5'UTR	opensea	5'UTR-open		rs5300594	23;22;5;1
cg1674421	0.118082	0.544534	3.434112	0.003579	0.224708	-2.84142	0.603575	0.485493	-0.11808	14	49872206	F	II		IGR	opensea	IGR-opense		rs1500094	1;8
cg2045008	0.115029	0.504781	3.207789	0.00572	0.224708	-3.30493	0.562296	0.447266	-0.11503	6	75857507	R	II	COL12A1	ExonBnd	opensea	ExonBnd-op		rs1135498	36;35;13
cg0642230	0.114956	0.537219	3.184413	0.006003	0.224708	-3.35249	0.594697	0.479741	-0.11496	10	1352781	R	II	ADARB2	Body	opensea	Body-opens		rs1131878	48;35
cg1258918	0.110519	0.463441	3.429676	0.003612	0.224708	-2.85055	0.5187	0.408181	-0.11052	10	1226886	F	II		IGR	shelf	IGR-shelf	chr10:123	rs5778733	0;1;9;14;19;41;50
cg2414031	0.130323	0.546922	3.428979	0.003617	0.224708	-2.85198	0.612084	0.481761	-0.13032	1	1.85E+08	R	II		IGR	opensea	IGR-opense		rs1503022	17;1
cg0785156	0.124454	0.523304	3.427802	0.003626	0.224708	-2.8544	0.585531	0.461077	-0.12445	18	48695911	R	II		IGR	opensea	IGR-opense			
cg2510667	0.114951	0.627703	2.997615	0.008824	0.224708	-3.72976	0.685178	0.570227	-0.11495	21	17452671	R	II	MIR99AHG	Body	opensea	Body-opens			
cg1614517	0.103444	0.446009	3.426589	0.003635	0.224708	-2.8569	0.497731	0.394287	-0.10344	21	25596030	F	II		IGR	opensea	IGR-opense		rs1500489	0;9;28;39
cg0167462	0.114919	0.453617	3.413665	0.003734	0.224708	-2.88349	0.511077	0.396158	-0.11492	7	1.06E+08	R	II	NAMPT	Body	opensea	Body-opens		rs5367027	21
cg0120872	0.113098	0.444211	3.424692	0.003649	0.224708	-2.8608	0.50076	0.387662	-0.1131	5	6829916	F	II		IGR	opensea	IGR-opense			
cg2130398	0.110274	0.423508	3.424104	0.003654	0.224708	-2.86201	0.478645	0.36837	-0.11027	2	1.06E+08	R	II		IGR	opensea	IGR-opense		rs1016902	39;24;5;1
cg1021786	0.114909	0.417666	3.109403	0.007009	0.224708	-3.50461	0.47512	0.360211	-0.11491	10	99188343	R	II	PGAM1	Body	shelf	Body-shelf	chr10:991	rs1475377	45;34
cg0073439	0.114813	0.610166	3.532971	0.002915	0.224708	-2.63759	0.667572	0.552759	-0.11481	10	4122651	R	II	LOC10192	Body	opensea	Body-opens		rs1166529	22;5;2
cg0311210	0.100165	0.423121	3.419117	0.003692	0.224708	-2.87227	0.473204	0.373039	-0.10017	3	55469470	F	II		IGR	opensea	IGR-opense		rs1862543	0;41;44
cg1528653	0.114786	0.570294	3.296514	0.00476	0.224708	-3.12384	0.627688	0.512901	-0.11479	16	19823027	F	II	IQCK	Body	opensea	Body-opens		rs1470942	1;33
cg0629748	0.105685	0.709218	3.418842	0.003694	0.224708	-2.87284	0.762061	0.656376	-0.10569	16	73240390	R	II		IGR	opensea	IGR-opense		rs5575901	4;2;1
cg2417153	0.109292	0.509019	3.418574	0.003696	0.224708	-2.87339	0.563665	0.454374	-0.10929	8	55361122	F	II		IGR	opensea	IGR-opense			
cg0903796	0.158992	0.307989	3.418379	0.003698	0.224708	-2.87379	0.387485	0.228493	-0.15899	2	37828140	R	II		IGR	opensea	IGR-opense		rs1156806	20;18
cg0724389	0.11476	0.474268	2.630873	0.018614	0.225119	-4.45124	0.531648	0.416888	-0.11476	3	18195180	F	II	LOC33986	Body	opensea	Body-opens		rs5592653	0;3
cg0282524	0.10092	0.442077	3.417683	0.003703	0.224708	-2.87522	0.492537	0.391617	-0.10092	2	1.97E+08	R	II		IGR	opensea	IGR-opense		rs5754973	22;1
cg1067954	0.117677	0.549997	3.417254	0.003706	0.224708	-2.87611	0.608836	0.491159	-0.11768	1	21532794	R	II		IGR	opensea	IGR-opense		rs5604575	1
cg2696124	0.114614	0.458974	2.889902	0.011004	0.224708	-3.94466	0.516281	0.401667	-0.11461	6	90813309	F	II	BACH2	5'UTR	opensea	5'UTR-open		rs5570660	15;20;42

cg0287836	0.105336	0.741099	3.414929	0.003724	0.224708	-2.88089	0.793767	0.688431	-0.10534	6	272080	R	II		IGR	opensea	IGR-opense		rs3690126	35;1
cg0010212	0.114595	0.583376	4.030504	0.001041	0.224708	-1.60646	0.640673	0.526079	-0.11459	8	1.31E+08	F	II	FAM49B	Body	opensea	Body-opens		rs5756457	1;19
cg2266935	0.106231	0.623476	3.414848	0.003725	0.224708	-2.88106	0.676591	0.57036	-0.10623	5	1.51E+08	R	II		IGR	opensea	IGR-opense		rs1386673	5
cg0370596	0.114468	0.607835	2.71762	0.015625	0.224896	-4.28339	0.665069	0.550601	-0.11447	7	1.12E+08	F	II	DOCK4	Body	opensea	Body-opens		rs1129577	0
cg2030434	0.114461	0.611658	3.291348	0.004812	0.224708	-3.1344	0.668889	0.554428	-0.11446	10	13739925	R	II	FRMD4A	Body	opensea	Body-opens		rs1396537	49;16
cg2287587	0.114461	0.498006	3.206723	0.005733	0.224708	-3.3071	0.555237	0.440775	-0.11446	4	72978321	F	II	NPFFR2	5'UTR	opensea	5'UTR-open		rs7639025	1;17;33;44
cg1776979	0.114432	0.383436	3.557988	0.002768	0.224708	-2.58591	0.440652	0.32622	-0.11443	4	89976368	R	II	FAM13A	Body	opensea	Body-opens		rs7655442	46;17
cg1645882	0.114406	0.284922	2.484594	0.024935	0.228258	-4.72949	0.342125	0.227719	-0.11441	13	44261002	R	II	ENOX1	5'UTR	opensea	5'UTR-open		rs7972945	1
cg0238763	0.11609	0.363726	3.410811	0.003756	0.224708	-2.88936	0.421771	0.305681	-0.11609	8	50084756	R	II		IGR	opensea	IGR-opense		rs5564741	13;1
cg1475850	0.121557	0.527896	3.410722	0.003757	0.224708	-2.88954	0.588675	0.467118	-0.12156	6	1.44E+08	R	II		IGR	opensea	IGR-opense		rs3702481	43;1
cg0841521	0.117436	0.514035	3.403887	0.00381	0.224708	-2.9036	0.572752	0.455317	-0.11744	6	19906065	R	II		IGR	opensea	IGR-opense			
cg0238494	0.112894	0.415988	3.400596	0.003836	0.224708	-2.91036	0.472435	0.359541	-0.11289	3	1.96E+08	R	II		IGR	opensea	IGR-opense			
cg1289553	0.114371	0.671177	4.455442	0.000437	0.224708	-0.72828	0.728362	0.613991	-0.11437	16	58063504	F	II	MMP15	Body	shelf	Body-shelf	chr16:580	rs5505787	3;9
cg1425803	0.114315	0.619346	2.553869	0.021721	0.225763	-4.59851	0.676504	0.562189	-0.11432	9	8711763	R	II	PTPRD	Body	shore	Body-shore	chr9:8713	rs1925176	49;43;4;1
cg1561753	0.11429	0.455048	4.107249	0.000889	0.224708	-1.44737	0.512193	0.397902	-0.11429	1	1.99E+08	F	II	PTPRC	TSS1500	opensea	TSS1500-op		rs1214128	0
cg0725253	0.102915	0.687335	3.394318	0.003887	0.224708	-2.92327	0.738793	0.635878	-0.10292	12	59845805	R	II		IGR	opensea	IGR-opense			
cg2172108	0.114254	0.520806	3.601306	0.00253	0.224708	-2.49636	0.577933	0.463679	-0.11425	9	37667895	R	II	FRMPD1	5'UTR	opensea	5'UTR-open		rs5434572	32;17
cg0836560	0.119448	0.356005	3.393842	0.003891	0.224708	-2.92424	0.415729	0.296281	-0.11945	19	33726655	R	II		IGR	island	IGR-island	chr19:337		
cg1462898	0.114236	0.586163	3.207129	0.005728	0.224708	-3.30628	0.643281	0.529045	-0.11424	2	28717630	R	II	PLB1	TSS1500	opensea	TSS1500-op			
cg1340841	0.118998	0.605617	3.391788	0.003907	0.224708	-2.92846	0.665116	0.546118	-0.119	1	1.53E+08	F	II		IGR	opensea	IGR-opense			
cg1502960	0.114235	0.711826	3.327635	0.004463	0.224708	-3.06011	0.768943	0.654708	-0.11423	7	47377238	F	II	TNS3	Body	opensea	Body-opens		rs5558151	0;23;44
cg1891709	0.114205	0.471299	2.914287	0.010468	0.224708	-3.8962	0.528402	0.414197	-0.11421	2	2.02E+08	F	II	CFLAR	TSS1500	opensea	TSS1500-op			
cg0912199	0.10043	0.682614	3.389642	0.003925	0.224708	-2.93287	0.732829	0.632399	-0.10043	1	1.81E+08	R	II		IGR	opensea	IGR-opense		rs5771152	39;35;2
cg1405720	0.11416	0.610817	3.905494	0.001348	0.224708	-1.8658	0.667897	0.553737	-0.11416	2	39659093	F	II	MAP4K3	Body	opensea	Body-opens		rs7950591	1;15;24;45
cg2542698	0.102433	0.503591	3.388582	0.003933	0.224708	-2.93505	0.554807	0.452374	-0.10243	18	61683198	F	II		IGR	opensea	IGR-opense		rs1511701	28;46
cg1544481	0.114138	0.702868	2.601384	0.01975	0.225445	-4.50784	0.759937	0.6458	-0.11414	17	75721911	R	II	LOC10013	Body	opensea	Body-opens		rs8080638	51;21;1
cg1842961	0.11446	0.732146	3.387914	0.003939	0.224708	-2.93643	0.789376	0.674916	-0.11446	3	1.44E+08	R	II		IGR	opensea	IGR-opense		rs5587367	37;21;17;16;2
cg0724078	0.102082	0.752477	3.387634	0.003941	0.224708	-2.937	0.803518	0.701436	-0.10208	5	1.5E+08	F	II		IGR	opensea	IGR-opense		rs1480925	1;8;15;34;42;43;47;47;48;49
cg1208130	0.107931	0.525413	3.386702	0.003949	0.224708	-2.93892	0.579378	0.471447	-0.10793	2	38346853	F	II		IGR	opensea	IGR-opense		rs5649776	0;20;49
cg1965882	0.114204	0.601432	3.386456	0.003951	0.224708	-2.93942	0.658534	0.54433	-0.1142	3	1.88E+08	R	II		IGR	opensea	IGR-opense			
cg0930447	0.114112	0.582658	3.23221	0.005438	0.224708	-3.25518	0.639714	0.525602	-0.11411	18	76916901	R	II	ATP9B	Body	opensea	Body-opens		rs1145147	1
cg1009312	0.114112	0.569198	3.425837	0.003641	0.224708	-2.85845	0.626254	0.512142	-0.11411	17	1335117	F	II	CRK	Body	opensea	Body-opens			
cg1738166	0.107601	0.556914	3.382613	0.003982	0.224708	-2.94732	0.610714	0.503114	-0.1076	4	1.13E+08	R	II		IGR	opensea	IGR-opense		rs1432030	1
cg2530440	0.114067	0.550622	3.235545	0.005401	0.224708	-3.24838	0.607655	0.493589	-0.11407	17	32485152	R	II	ACCN1	TSS1500	shore	TSS1500-sh	chr17:324	rs1862167	41;1
cg1747448	0.118458	0.473768	3.380733	0.003998	0.224708	-2.95118	0.532997	0.414539	-0.11846	12	20047827	F	II		IGR	opensea	IGR-opense		rs5284874	44
cg2748343	0.114035	0.538292	4.67754	0.000279	0.224708	-0.27329	0.59531	0.481275	-0.11404	1	2.48E+08	F	II	OR2L5	TSS1500	opensea	TSS1500-op		rs1419421	0;20;40
cg0550161	0.114033	0.492506	3.018402	0.008455	0.224708	-3.68805	0.549522	0.435489	-0.11403	4	1.85E+08	R	II	IRF2	Body	opensea	Body-opens		rs6233775	34;13
cg0570272	0.11402	0.466441	3.687477	0.002116	0.224708	-2.31796	0.523451	0.409431	-0.11402	4	1.12E+08	R	II	PITX2	TSS1500	shelf	TSS1500-sh	chr4:1115	rs5411311	29;1
cg1807249	0.114017	0.612892	3.758669	0.001826	0.224708	-2.1704	0.6699	0.555883	-0.11402	12	40944308	F	II	MUC19	Body	opensea	Body-opens			
cg1194257	0.113946	0.603093	4.06887	0.000962	0.224708	-1.52691	0.660066	0.54612	-0.11395	2	48952008	R	II	LHCGR	Body	opensea	Body-opens		rs4366951	39;21;20;1
cg1409865	0.113935	0.451007	3.839428	0.001545	0.224708	-2.00288	0.507974	0.39404	-0.11393	1	1.12E+08	F	II	KCND3-AS1	TSS1500	opensea	TSS1500-op		rs5345913	1;35;36;43;46
cg2373248	0.10634	0.586844	3.373896	0.004055	0.224708	-2.96522	0.640013	0.533674	-0.10634	3	12930997	R	II		IGR	opensea	IGR-opense		rs1145087	1
cg1823151	0.113815	0.610492	2.444631	0.02699	0.22984	-4.80435	0.6674	0.553585	-0.11381	8	1.41E+08	F	II	TRAPPC9	Body	opensea	Body-opens		rs5374153	29;39
cg2054701	0.113793	0.506292	2.856364	0.011785	0.224708	-4.01111	0.563189	0.449396	-0.11379	12	1.11E+08	R	II	PPP1CC	Body	opensea	Body-opens		rs5500907	44;32;25;20
cg1453356	0.11379	0.447718	3.013068	0.008548	0.224708	-3.69876	0.504613	0.390822	-0.11379	3	52705872	R	II	PBRM1	Body	opensea	Body-opens		rs5497009	33;22
cg2524096	0.113767	0.539397	3.684893	0.002127	0.224708	-2.32332	0.59628	0.482513	-0.11377	4	79339974	R	II	FRAS1	Body	opensea	Body-opens		rs1449887	48;45;26
cg0621425	0.113726	0.658859	2.873626	0.011376	0.224708	-3.97694	0.715722	0.601996	-0.11373	13	73438525	F	II	PIBF1	Body	opensea	Body-opens		rs7853179	0;13
cg1148027	0.113716	0.429331	2.355286	0.032178	0.234492	-4.96976	0.486189	0.372473	-0.11372	17	83580	R	II	RPH3AL	Body	shore	Body-shore	chr17:850	rs1157297	8;1
cg1934716	0.104741	0.794549	3.364138	0.004138	0.224708	-2.98525	0.84692	0.742178	-0.10474	2	65763752	R	II		IGR	opensea	IGR-opense		rs1437608	42;11;2;1
cg0591920	0.100258	0.467158	3.364063	0.004138	0.224708	-2.98541	0.517287	0.417029	-0.10026	4	5914761	R	II		IGR	opensea	IGR-opense		rs5412842	45
cg0112413	0.113704	0.431233	3.025238	0.008336	0.224708	-3.67431	0.488085	0.374381	-0.1137	22	32599511	F	II	RFPL2	TSS200	opensea	TSS200-ope		rs5746053	0;22;29
cg0727201	0.113667	0.706381	3.445037	0.003499	0.224708	-2.81893	0.763215	0.649548	-0.11367	6	11767836	F	II	ADTRP	Body	opensea	Body-opens		rs1465744	0;1;18;40
cg0888183	0.113635	0.59445	3.573673	0.002679	0.224708	-2.5535	0.651268	0.537633	-0.11363	12	5348711	R	II	LOC10192	Body	opensea	Body-opens		rs3713237	32
cg2598271	0.11356	0.630831	4.316607	0.00058	0.224708	-1.01432	0.687612	0.574051	-0.11356	18	11027789	R	II	PIEZO2	Body	opensea	Body-opens		rs3735900	26
cg0446232	0.102902	0.598015	3.356458	0.004204	0.224708	-3.00101	0.649466	0.546564	-0.1029	14	91697876	R	II		IGR	shelf	IGR-shelf	chr14:917	rs1422905	38;6;5
cg2482723	0.107628	0.617928	3.356248	0.004206	0.224708	-3.00144	0.671742	0.564114	-0.10763	11	1.16E+08	F	II		IGR	shelf	IGR-shelf	chr11:115	rs1166368	0
cg1416439	0.113334	0.490336	3.290927	0.004816	0.224708	-3.13527	0.547003	0.433669	-0.11333	17	28707127	R	II	CPD	TSS1500	shore	TSS1500-sh	chr17:287	rs5644120	7
cg0546240	0.11131	0.687069	3.351847	0.004245	0.224708	-3.01047	0.742724	0.631414	-0.11131	11	59266110	R	II		IGR	opensea	IGR-opense		rs5323318	39
cg0511474	0.191201	0.70471	3.351802	0.004245	0.224708	-3.01057	0.80031	0.609109	-0.1912	2	2.43E+08	F	II		IGR	shelf	IGR-shelf	chr2:2426	rs5594630	0
cg2277189	0.113298	0.501202	2.90952	0.010571	0.224708	-3.90569	0.557851	0.444553	-0.1133	8	17613996	R	II	MTUS1	5'UTR	opensea	5'UTR-open		rs5499932	49;2;1
cg1102082	0.10591	0.579705	3.350565	0.004256	0.224708	-3.0131	0.63266	0.526749	-0.10591	15	79128547	R	II		IGR	shelf	IGR-shelf	chr15:791	rs4539564	50;28;2
cg1998339	0.132133	0.475997	3.350497	0.004256	0.224708	-3.01324	0.542063	0.40993	-0.13213	13	29115045	R	II		IGR	opensea	IGR-opense		rs5451385	38;2
cg0838841	0.113294	0.542914	3.191309	0.005918	0.224708	-3.33847	0.599561	0.486267	-0.11329	2	2.26E+08	R	II	DOCK10	Body	opensea	Body-opens		rs5635179	7
cg2529610	0.113282	0.48452	2.969513	0.009348	0.224708	-3.78603	0.541161	0.427879	-0.11328	2	1.45E+08	R	II	ZEB2	Body	opensea	Body-opens		rs5374792	46;43;21

cg1617725	0.11321	0.646378	2.91151	0.010528	0.224708	-3.90173	0.702983	0.589774	-0.11321	6	1.2E+08	F	II	MAN1A1	Body	opensea	Body-opens		rs5548515	44
cg0946539	0.113194	0.676586	3.506287	0.003081	0.224708	-2.69267	0.733182	0.619989	-0.11319	4	1.41E+08	R	II	MAML3	Body	opensea	Body-opens		rs5616773	38;2
cg0525422	0.113183	0.494037	2.850599	0.011924	0.224708	-4.02251	0.550628	0.437445	-0.11318	10	61050062	R	II	FAM13C	Body	opensea	Body-opens		rs5729288	25;5
cg1017094	0.113145	0.533314	4.821674	0.00021	0.224708	0.019932	0.589886	0.476742	-0.11314	12	57599717	F	II	LRP1	Body	shelf	Body-shelf	chr12:5759	rs1460269	0;18
cg2581716	0.113112	0.566204	2.154655	0.047433	0.251904	-5.33022	0.62276	0.509648	-0.11311	18	72167213	R	II	CNDP2	1stExon	shelf	1stExon-sh	chr18:7216	rs1918690	31;17;2
cg0082985	0.11311	0.472292	2.613164	0.019288	0.225391	-4.48526	0.528847	0.415737	-0.11311	2	1.79E+08	F	II	OSBPL6	5'UTR	opensea	5'UTR-open		rs5694126	0;1
cg0667966	0.119534	0.585416	3.345378	0.004302	0.224708	-3.02374	0.645183	0.525649	-0.11953	8	94966773	F	II		IGR	opensea	IGR-opense		rs1837980	0;16
cg1810095	0.131191	0.56968	3.344699	0.004308	0.224708	-3.02513	0.635275	0.504084	-0.13119	2	2.14E+08	F	II		IGR	opensea	IGR-opense		rs6218803	18;24;25
cg1355382	0.112252	0.523963	3.34459	0.004309	0.224708	-3.02536	0.580089	0.467837	-0.11225	4	1.22E+08	F	II		IGR	opensea	IGR-opense		rs5728741	41
cg0605737	0.101447	0.334868	3.344362	0.004311	0.224708	-3.02582	0.385591	0.284145	-0.10145	1	89507082	R	II		IGR	opensea	IGR-opense		rs1508799	32
cg2573408	0.113091	0.426475	2.767731	0.014115	0.224708	-4.18557	0.483021	0.36993	-0.11309	19	46969919	R	II	PNMAL1	3'UTR	opensea	3'UTR-open		rs5658754	36
cg0870784	0.113082	0.436827	3.414778	0.003725	0.224708	-2.8812	0.493368	0.380286	-0.11308	10	13061436	R	II	CCDC3	5'UTR	opensea	5'UTR-open		rs5510475	8
cg2475649	0.11306	0.639074	3.598017	0.002547	0.224708	-2.50316	0.695604	0.582544	-0.11306	18	21754024	F	II	OSBPL1A	Body	opensea	Body-opens		rs1841844	1;7;34;36
cg1076455	0.113021	0.532877	3.780045	0.001747	0.224708	-2.12607	0.589388	0.476367	-0.11302	11	12419531	R	II	PARVA	Body	opensea	Body-opens		rs1818671	50;44
cg2041401	0.112988	0.703936	2.780266	0.013761	0.224708	-4.161	0.76043	0.647442	-0.11299	4	1.41E+08	R	II	MAML3	Body	opensea	Body-opens		rs5379888	34;5;1
cg1868768	0.112986	0.349938	3.748322	0.001865	0.224708	-2.19185	0.406431	0.293445	-0.11299	6	4801802	R	II	CDYL	Body	opensea	Body-opens			
cg1460121	0.111284	0.580597	3.339323	0.004356	0.224708	-3.03616	0.636239	0.524955	-0.11128	7	1.56E+08	R	I		IGR	shelf	IGR-shelf	chr7:1556	rs3734060	35;18
cg1870459	0.112957	0.505954	2.690626	0.016502	0.224896	-4.33584	0.562433	0.449475	-0.11296	4	1.54E+08	F	II	TRIM2	5'UTR	opensea	5'UTR-open		rs1140632	1;18
cg1579558	0.112922	0.437227	3.00772	0.008642	0.224708	-3.70949	0.493688	0.380766	-0.11292	2	2.43E+08	F	II	DTYMK	TSS1500	shore	TSS1500-sh	chr2:2426	rs1486185	38;49
cg0441044	0.112913	0.49394	2.843652	0.012094	0.224708	-4.03624	0.550396	0.437483	-0.11291	6	1.44E+08	R	II	C6orf94	Body	opensea	Body-opens		rs5456474	38;2;1
cg2278647	0.119714	0.388816	3.333185	0.004412	0.224708	-3.04874	0.448673	0.328959	-0.11971	4	26198975	F	II		IGR	opensea	IGR-opense		rs5473382	0
cg1111353	0.120665	0.582314	3.331725	0.004425	0.224708	-3.05173	0.642646	0.521981	-0.12066	11	1.16E+08	R	II		IGR	opensea	IGR-opense		rs1262591	43;42
cg1852223	0.203647	0.378308	3.331074	0.004431	0.224708	-3.05307	0.480132	0.276484	-0.20365	3	1.97E+08	R	II		IGR	opensea	IGR-opense			
cg0935305	0.112871	0.428339	3.190472	0.005929	0.224708	-3.34017	0.484775	0.371903	-0.11287	2	1.67E+08	R	II	SCN1A	3'UTR	opensea	3'UTR-open			
cg0015579	0.112822	0.603315	2.731011	0.015207	0.224792	-4.25731	0.659725	0.546904	-0.11282	2	1.73E+08	R	II	METAP1D	Body	opensea	Body-opens		rs9973784	29
cg2736557	0.114641	0.547333	3.329036	0.00445	0.224708	-3.05724	0.604654	0.490013	-0.11464	2	1.47E+08	F	II		IGR	opensea	IGR-opense		rs5407420	0;18;51
cg1036590	0.112749	0.535285	3.940615	0.001253	0.224708	-1.79293	0.591659	0.47891	-0.11275	11	78568009	R	II	ODZ4	Body	opensea	Body-opens		rs5317268	46;2
cg2587349	0.106116	0.575683	3.326758	0.004471	0.224708	-3.06191	0.628741	0.522624	-0.10612	20	17973930	F	II		IGR	opensea	IGR-opense		rs1407710	42;51
cg2475965	0.112637	0.507155	3.330782	0.004434	0.224708	-3.05366	0.563474	0.450837	-0.11264	17	76801065	R	II	USP36	Body	shelf	Body-shelf	chr17:7679	rs7476313	41;13;1
cg1627899	0.101213	0.591956	3.322123	0.004514	0.224708	-3.07141	0.642562	0.541349	-0.10121	6	13715593	F	II		IGR	shelf	IGR-shelf	chr6:1371		
cg0605566	0.112536	0.163204	2.348009	0.03264	0.234795	-4.98311	0.219472	0.106936	-0.11254	8	4399574	R	II	CSMD1	Body	opensea	Body-opens		rs5377435	21;11
cg2584078	0.11248	0.683125	3.280366	0.004922	0.224708	-3.15686	0.739365	0.626885	-0.11248	20	50156843	F	II	NFATC2	Body	shelf	Body-shelf	chr20:5015	rs5475672	1;30;31
cg0435315	0.112461	0.491427	3.468399	0.003333	0.224708	-2.7708	0.547658	0.435197	-0.11246	15	77722632	F	II	HMG20A	5'UTR	opensea	5'UTR-open		rs1892357	1
cg0634612	0.112448	0.463686	2.950063	0.009728	0.224708	-3.8249	0.51991	0.407463	-0.11245	19	50804360	F	II	MYH14	Body	opensea	Body-opens		rs5651906	0;38;39;46
cg0161707	0.112375	0.48151	3.811313	0.001637	0.224708	-2.06121	0.537697	0.425322	-0.11238	7	964629	R	II	ADAP1	Body	shore	Body-shore	chr7:9659	rs1433319	35;1
cg1447836	0.103714	0.546018	3.314879	0.004583	0.224708	-3.08624	0.597876	0.494161	-0.10371	9	99209707	F	II		IGR	shelf	IGR-shelf	chr9:9921	rs5481274	0
cg1665803	0.112322	0.473858	3.039436	0.008096	0.224708	-3.64576	0.530019	0.417697	-0.11232	10	22919045	F	II	PIP4K2A	Body	opensea	Body-opens		rs1878955	0;40;42;44
cg2263700	0.10517	0.457769	3.312907	0.004601	0.224708	-3.09028	0.510354	0.405184	-0.10517	2	706036	R	II		IGR	opensea	IGR-opense			
cg2346182	0.112244	0.594779	3.013309	0.008544	0.224708	-3.69827	0.650901	0.538658	-0.11224	7	1.35E+08	R	II	CALD1	Body	opensea	Body-opens			
cg1748639	0.112193	0.585691	2.826059	0.012536	0.224708	-4.07096	0.641788	0.529595	-0.11219	1	1.72E+08	F	II	C1orf105	Body	opensea	Body-opens		rs5404290	0;34
cg0845203	0.112163	0.512756	2.989048	0.00898	0.224708	-3.74693	0.568837	0.456674	-0.11216	22	28426107	F	II	TTC28	Body	opensea	Body-opens		rs5692076	1;21
cg0530422	0.10943	0.481245	3.310211	0.004627	0.224708	-3.0958	0.53596	0.42653	-0.10943	5	4234168	F	II		IGR	opensea	IGR-opense		rs5679892	1
cg0146418	0.11216	0.481936	2.736999	0.015024	0.22478	-4.24563	0.538017	0.425856	-0.11216	10	52191661	F	II	SGMS1	5'UTR	opensea	5'UTR-open		rs5672233	0
cg0834360	0.112159	0.508947	3.096267	0.007202	0.224708	-3.53117	0.565027	0.452867	-0.11216	10	43894095	F	II	HNRNPF	5'UTR	shore	5'UTR-shor	chr10:4389	rs5523722	1;13;17;37
cg0728549	0.117113	0.522151	3.308433	0.004644	0.224708	-3.09944	0.580708	0.463595	-0.11711	1	32071681	F	II		IGR	opensea	IGR-opense		rs5399555	24;49
cg2463970	0.112156	0.544173	2.842657	0.012119	0.224708	-4.03821	0.600251	0.488095	-0.11216	1	64986155	R	II	CACHD1	Body	opensea	Body-opens		rs5298374	13
cg2628520	0.120793	0.647089	3.307694	0.004651	0.224708	-3.10095	0.707486	0.586693	-0.12079	20	40342240	F	II		IGR	opensea	IGR-opense		rs7598542	1;22
cg0830404	0.111966	0.632054	3.418901	0.003694	0.224708	-2.87272	0.688037	0.576071	-0.11197	20	237253	F	II	DEFB132	TSS1500	opensea	TSS1500-op		rs5729680	36;44;48
cg0425883	0.111958	0.533638	3.057418	0.007802	0.224708	-3.60956	0.589617	0.477659	-0.11196	2	1.03E+08	F	II	IL1R1	5'UTR	opensea	5'UTR-open		rs7462859	33;42;50
cg0759554	0.103867	0.513532	3.305506	0.004672	0.224708	-3.10543	0.565465	0.461598	-0.10387	13	77919764	F	II		IGR	opensea	IGR-opense		rs5666234	28
cg1095437	0.11189	0.645778	2.495614	0.024395	0.227653	-4.70875	0.701724	0.589833	-0.11189	4	66188645	F	II	EPHA5	3'UTR	opensea	3'UTR-open		rs1015532	0;27
cg2136222	0.11189	0.622853	3.448475	0.003474	0.224708	-2.81185	0.678798	0.566908	-0.11189	8	1864982	R	II	ARHGEF10	Body	opensea	Body-opens		rs1857418	50;47;2
cg0241880	0.111841	0.460672	3.017855	0.008464	0.224708	-3.68914	0.516593	0.404752	-0.11184	12	94766510	F	II	CEP83	Body	opensea	Body-opens		rs5698437	0
cg1349476	0.111835	0.574113	3.049539	0.00793	0.224708	-3.62543	0.63003	0.518195	-0.11184	1	2.44E+08	F	II	MIR4677	TSS1500	opensea	TSS1500-op		rs1924570	0;1
cg2260951	0.1109	0.485029	3.29823	0.004743	0.224708	-3.12032	0.540478	0.429579	-0.1109	7	28891164	R	II		IGR	shelf	IGR-shelf	chr7:2889	rs763811;r	44;33;19;17;2
cg0581313	0.111819	0.650973	3.421194	0.003676	0.224708	-2.868	0.706883	0.595064	-0.11182	12	1.19E+08	F	II	SRRM4	Body	opensea	Body-opens		rs1507520	5;34
cg1947937	0.111813	0.658903	3.459854	0.003393	0.224708	-2.78841	0.71481	0.602997	-0.11181	10	99734154	F	II	CRTAC1	Body	opensea	Body-opens		rs1405161	8;37
cg0678409	0.11145	0.557618	3.296181	0.004764	0.224708	-3.12452	0.613344	0.501893	-0.11145	5	40151155	F	II		IGR	opensea	IGR-opense		rs7162384	30
cg1177427	0.111718	0.573872	3.778773	0.001751	0.224708	-2.12871	0.62973	0.518013	-0.11172	17	49110507	R	II	SPAG9	Body	opensea	Body-opens		rs5492084	2
cg0101984	0.111714	0.504965	3.142454	0.006547	0.224708	-3.43768	0.560822	0.449108	-0.11171	11	1.19E+08	F	II	ABCG4	Body	shelf	Body-shelf	chr11:119	rs5737133	0;32;45
cg0867146	0.105976	0.453083	3.292859	0.004797	0.224708	-3.13131	0.506071	0.400095	-0.10598	12	51912686	R	II		IGR	opensea	IGR-opense		rs5409346	41;37;9;6
cg0895051	0.125533	0.571305	3.292656	0.004799	0.224708	-3.13173	0.634072	0.508538	-0.12553	2	1.35E+08	R	II		IGR	opensea	IGR-opense		rs5540531	38;32;3
cg0085853	0.103232	0.684216	3.291436	0.004811	0.224708	-3.13422	0.735832	0.632599	-0.10323	13	73743021	F	II		IGR	opensea	IGR-opense		rs1445758	0
cg2089284	0.111688	0.674381	2.502688	0.024054	0.227443	-4.69542	0.730225	0.618537	-0.11169	3	1.57E+08	R	II	CCNL1	TSS1500	shore	TSS1500-sh	chr3:1568	rs5757058	32

cg1167460	0.111646	0.648127	2.289533	0.036582	0.23891	-5.08965	0.70395	0.592304	-0.11165	22	49910987	F	II	C22orf34	Body	opensea	Body-opens		rs5440797	29;31
cg2658237	0.111599	0.483532	3.145195	0.00651	0.224708	-3.43212	0.539332	0.427733	-0.1116	4	4639639	R	II	STX18-AS1	Body	opensea	Body-opens		rs5517322	29;28;23;1
cg1513811	0.111554	0.432197	3.285499	0.00487	0.224708	-3.14637	0.487974	0.37642	-0.11155	6	1.5E+08	F	II	PPP1R14C	Body	opensea	Body-opens			
cg1161645	0.111534	0.37473	3.131132	0.006702	0.224708	-3.46062	0.430496	0.318963	-0.11153	4	38525723	R	II	LINC01258	TSS1500	opensea	TSS1500-op		rs1832261	33
cg0351287	0.111498	0.597292	3.414885	0.003724	0.224708	-2.88098	0.653041	0.541542	-0.1115	3	1.2E+08	R	II	GPR156	Body	opensea	Body-opens		rs5631941	25
cg0875243	0.111488	0.423265	2.956449	0.009602	0.224708	-3.81215	0.479009	0.367521	-0.11149	12	1.11E+08	R	II	PPTC7	Body	shelf	Body-shelf	chr12:111	rs1891183	11;2
cg1416871	0.111443	0.559753	3.150519	0.006439	0.224708	-3.42132	0.615474	0.504031	-0.11144	10	60579479	R	II	BICC1	Body	opensea	Body-opens		rs7902375	24
cg0427048	0.111846	0.344695	3.283809	0.004887	0.224708	-3.14982	0.400617	0.288772	-0.11185	11	85827505	F	II		IGR	opensea	IGR-opense		rs1497039	1;16;19;32
cg2741724	0.115647	0.55759	3.283694	0.004888	0.224708	-3.15006	0.615414	0.499767	-0.11565	7	20323695	F	II		IGR	opensea	IGR-opense		rs2529019	10
cg0547959	0.119897	0.617956	3.28288	0.004897	0.224708	-3.15172	0.677904	0.558007	-0.1199	11	1.35E+08	R	II		IGR	opensea	IGR-opense		rs5655423	40;22;16;2
cg1016849	0.114131	0.711136	3.281773	0.004908	0.224708	-3.15398	0.768201	0.65407	-0.11413	3	1.88E+08	R	II		IGR	opensea	IGR-opense		rs5583051	51;24;14
cg2410804	0.123082	0.665934	3.281763	0.004908	0.224708	-3.15401	0.727475	0.604393	-0.12308	12	63700576	F	II		IGR	opensea	IGR-opense		rs3689994	0;23
cg1733049	0.111405	0.565858	2.923948	0.010263	0.224708	-3.87697	0.621561	0.510155	-0.11141	13	67550124	F	II	PCDH9-AS3	TSS1500	opensea	TSS1500-op		rs5415131	32;46
cg1212670	0.111405	0.606557	2.791229	0.013457	0.224708	-4.13949	0.66226	0.550855	-0.11141	1	1.57E+08	F	II	C1orf92	TSS1500	shore	TSS1500-sh	chr1:1568	rs5416842	7;41
cg1749257	0.103045	0.476361	3.280126	0.004925	0.224708	-3.15735	0.527884	0.424839	-0.10304	6	1.59E+08	R	II		IGR	opensea	IGR-opense		rs5410837	2
cg1426334	0.111392	0.465364	2.802032	0.013165	0.224708	-4.11826	0.52106	0.409668	-0.11139	7	1.51E+08	F	II	RHEB	Body	opensea	Body-opens		rs1150018	17;18
cg2378620	0.101543	0.459841	3.278799	0.004938	0.224708	-3.16006	0.510613	0.40907	-0.10154	10	77057425	R	II		IGR	shelf	IGR-shelf	chr10:770		
cg0388003	0.111374	0.635165	3.040104	0.008085	0.224708	-3.64442	0.690852	0.579477	-0.11137	16	55866757	F	II	CES1	Body	island	Body-island	chr16:558	rs5735936	0;35;39;41;42;50
cg1611698	0.111346	0.581638	3.309678	0.004632	0.224708	-3.09689	0.637311	0.525965	-0.11135	6	97395162	R	II	KLHL32	5'UTR	opensea	5'UTR-open		rs5820473	20;2
cg0813634	0.111318	0.796172	2.191821	0.044177	0.247847	-5.26469	0.851831	0.740513	-0.11132	9	1.39E+08	F	II	SDCCAG3	Body	shore	Body-shore	chr9:1393	rs5300374	3;17;24
cg2494268	0.115332	0.691082	3.275861	0.004968	0.224708	-3.16607	0.748748	0.633416	-0.11533	13	77518644	R	II		IGR	opensea	IGR-opense			
cg2645366	0.106536	0.53973	3.273006	0.004998	0.224708	-3.1719	0.592998	0.486462	-0.10654	10	90411391	R	II		IGR	opensea	IGR-opense		rs1384306	2
cg0900561	0.111317	0.467785	3.025889	0.008325	0.224708	-3.67301	0.523444	0.412126	-0.11132	1	1.86E+08	R	II	HMCN1	Body	opensea	Body-opens		rs5664036	1
cg0151222	0.110806	0.72477	3.271762	0.005011	0.224708	-3.17445	0.780173	0.669367	-0.11081	12	1.22E+08	F	II		IGR	opensea	IGR-opense		rs1910062	1;18
cg0743197	0.111283	0.535297	4.133579	0.000843	0.224708	-1.39282	0.590939	0.479656	-0.11128	14	52142383	F	II	FRMD6	5'UTR	opensea	5'UTR-open			
cg2607995	0.111251	0.554555	2.144718	0.04834	0.253098	-5.34764	0.610181	0.49893	-0.11125	12	1.29E+08	F	II	SLC15A4	Body	opensea	Body-opens		rs5322940	6;9;10
cg2035837	0.100507	0.583734	3.270207	0.005027	0.224708	-3.17762	0.633987	0.53348	-0.10051	2	1.47E+08	F	II		IGR	opensea	IGR-opense			
cg2403312	0.111202	0.201329	4.018756	0.001067	0.224708	-1.63083	0.25693	0.145728	-0.1112	16	30485383	R	II	ITGAL	Body	island	Body-island	chr16:304	rs3755904	2
cg1751534	0.111155	0.542431	2.759377	0.014357	0.224708	-4.20192	0.598008	0.486854	-0.11115	1	1.59E+08	R	II	AIM2	TSS1500	opensea	TSS1500-op		rs5652591	46;45;10;3
cg2745452	0.111146	0.578416	3.182894	0.006022	0.224708	-3.35558	0.633989	0.522843	-0.11115	1	2.47E+08	F	II	SMYD3	TSS200	opensea	TSS200-ope		rs1829293	23;30
cg2474346	0.101247	0.611832	3.268825	0.005041	0.224708	-3.18045	0.662456	0.561208	-0.10125	13	65669420	R	II		IGR	opensea	IGR-opense		rs5744790	29
cg0372967	0.11113	0.445433	2.641388	0.018224	0.225076	-4.431	0.500998	0.389868	-0.11113	5	71855774	F	II	LOC10250	Body	opensea	Body-opens			
cg0447469	0.103274	0.576428	3.266368	0.005067	0.224708	-3.18547	0.628065	0.524791	-0.10327	17	55158407	R	II		IGR	shelf	IGR-shelf	chr17:551	rs1401916	2
cg2259909	0.111103	0.609131	3.250032	0.005241	0.224708	-3.21882	0.664683	0.553579	-0.1111	7	1.16E+08	R	II	LINC01510	Body	opensea	Body-opens		rs5319736	39;1
cg2501344	0.123756	0.708676	3.265029	0.005081	0.224708	-3.1882	0.770554	0.646798	-0.12376	11	1.07E+08	F	II		IGR	shelf	IGR-shelf	chr11:107		
cg1398071	0.111084	0.634447	3.010531	0.008592	0.224708	-3.70385	0.689989	0.578905	-0.11108	2	2.18E+08	F	II	TNP1	TSS1500	opensea	TSS1500-op		rs5327672	8;17;28
cg2358408	0.111071	0.686203	4.379948	0.00051	0.224708	-0.88369	0.741739	0.630668	-0.11107	13	33626629	R	II	KL	Body	opensea	Body-opens		rs1497347	37;12;1
cg0763573	0.23137	0.801239	3.256766	0.005169	0.224708	-3.20508	0.916924	0.685554	-0.23137	2	2.43E+08	R	II		IGR	shelf	IGR-shelf	chr2:2426	rs5318817	50;42;21
cg0596822	0.105065	0.679342	3.256408	0.005173	0.224708	-3.20581	0.731874	0.626809	-0.10506	11	5770442	F	II		IGR	opensea	IGR-opense		rs5278813	25;26;27
cg0999405	0.111069	0.532565	3.773483	0.001771	0.224708	-2.13968	0.588099	0.47703	-0.11107	10	14598259	F	II	FAM107B	5'UTR	opensea	5'UTR-open			
cg0170833	0.110979	0.556331	3.159151	0.006325	0.224708	-3.40381	0.611821	0.500842	-0.11098	20	20484969	R	II	RALGAPA2	Body	opensea	Body-opens		rs5746230	48;36;1
cg1790226	0.112223	0.482968	3.253317	0.005206	0.224708	-3.21212	0.53908	0.426857	-0.11222	16	77490838	F	II		IGR	opensea	IGR-opense		rs3775960	5;6;11;41;51
cg0939330	0.110978	0.460446	2.840099	0.012182	0.224708	-4.04326	0.515935	0.404957	-0.11098	2	2.01E+08	R	II	TYW5	Body	opensea	Body-opens		rs1808259	50;2
cg0829168	0.102847	0.492644	3.252172	0.005218	0.224708	-3.21445	0.544068	0.441221	-0.10285	22	49749753	F	II		IGR	opensea	IGR-opense		rs5325898	37
cg0182970	0.117054	0.560561	3.251787	0.005222	0.224708	-3.21524	0.619089	0.502034	-0.11705	22	32047972	R	II		IGR	opensea	IGR-opense		rs6223917	33;4
cg1175076	0.118815	0.543415	3.251744	0.005223	0.224708	-3.21533	0.602823	0.484007	-0.11882	7	12521687	F	II		IGR	opensea	IGR-opense		rs1467679	1;35;41
cg0488514	0.110863	0.63503	3.105155	0.007071	0.224708	-3.5132	0.690461	0.579599	-0.11086	9	1.1E+08	R	II	MIR548Q	Body	opensea	Body-opens		rs5409613	2
cg1055686	0.110855	0.566796	3.178584	0.006076	0.224708	-3.36434	0.622224	0.511369	-0.11085	6	16413526	R	II	ATXN1	5'UTR	opensea	5'UTR-open		rs5700094	1
cg0575869	0.110845	0.418092	2.886906	0.011072	0.224708	-3.95061	0.473514	0.36267	-0.11084	6	4790999	F	II	CDYL	Body	opensea	Body-opens		rs1876937	12
cg0792195	0.110834	0.676502	3.721012	0.001974	0.224708	-2.24847	0.731919	0.621086	-0.11083	12	14107199	F	II	GRIN2B	5'UTR	opensea	5'UTR-open		rs5560964	19
cg2497680	0.110799	0.591724	3.051165	0.007903	0.224708	-3.62215	0.647124	0.536325	-0.1108	1	1.6E+08	F	II	CRP	TSS1500	opensea	TSS1500-op		rs5610226	6
cg1312114	0.110752	0.558512	2.472832	0.025524	0.228747	-4.75158	0.613888	0.503136	-0.11075	15	33218656	R	II	FMN1	Body	opensea	Body-opens		rs5462501	39
cg0200871	0.110684	0.592789	3.306383	0.004664	0.224708	-3.10364	0.648131	0.537447	-0.11068	6	1.33E+08	F	II	LINC00326	Body	opensea	Body-opens		rs1929208	0;1;48
cg2005885	0.110668	0.570946	3.30135	0.004713	0.224708	-3.11394	0.626279	0.515612	-0.11067	2	2.25E+08	F	II	AP1S3	Body	opensea	Body-opens		rs3738281	1;19
cg1818895	0.100941	0.459874	3.246554	0.005279	0.224708	-3.22592	0.510344	0.409404	-0.10094	16	65613388	F	II		IGR	opensea	IGR-opense		rs5771782	50
cg0820054	0.110643	0.608911	3.439485	0.003539	0.224708	-2.83036	0.664232	0.55359	-0.11064	1	2121449	F	II	C1orf86	Body	island	Body-island	chr1:2121	rs5537926	7;35
cg1126407	0.120658	0.670207	3.242623	0.005322	0.224708	-3.23394	0.730536	0.609878	-0.12066	14	71355741	R	II		IGR	opensea	IGR-opense		rs5363285	16;1
cg0445242	0.110615	0.583084	3.057928	0.007794	0.224708	-3.60853	0.638392	0.527777	-0.11061	6	1.51E+08	F	II	MTHFD1L	Body	opensea	Body-opens			
cg0898578	0.110613	0.488675	3.79481	0.001694	0.224708	-2.09544	0.543982	0.433369	-0.11061	6	39196340	F	I	KCNK5	Body	shore	Body-shore	chr6:3919	rs1118568	15;28
cg1688693	0.102236	0.632187	3.240053	0.005351	0.224708	-3.23918	0.683305	0.581069	-0.10224	2	2.4E+08	F	II		IGR	opensea	IGR-opense		rs5653095	0;1;11
cg1957438	0.127396	0.446407	3.23925	0.00536	0.224708	-3.24082	0.510104	0.382709	-0.1274	4	10075174	R	II		IGR	opensea	IGR-opense		rs1926784	1
cg1278796	0.100123	0.489437	3.239061	0.005362	0.224708	-3.24121	0.539498	0.439375	-0.10012	4	1.5E+08	R	II		IGR	opensea	IGR-opense		rs7295998	2
cg0198081	0.100432	0.495372	3.238734	0.005365	0.224708	-3.24187	0.545589	0.445156	-0.10043	4	1.13E+08	F	II		IGR	shore	IGR-shore	chr4:1132	rs1406720	27
cg1063624	0.110608	0.312564	2.395598	0.02973	0.232293	-4.89548	0.367868	0.25726	-0.11061	1	1.59E+08	F	II	AIM2	TSS1500	opensea	TSS1500-op		rs1845798	6;36

cg0668165	0.101996	0.571365	3.237839	0.005375	0.224708	-3.2437	0.622363	0.520366	-0.102	20	22777067	F	II		IGR	opensea	IGR-opense		rs5396566	1;6;18;42
cg0571077	0.110512	0.650096	3.445128	0.003498	0.224708	-2.81874	0.705352	0.59484	-0.11051	2	65089821	F	II	LOC10192	Body	shelf	Body-shelf	chr2:6509	rs5470309	5;20
cg0796525	0.110475	0.525571	3.221409	0.005561	0.224708	-3.27719	0.580808	0.470333	-0.11047	15	38342973	R	II	LOC10192	Body	opensea	Body-opens			
cg2311904	0.110459	0.588537	3.564543	0.00273	0.224708	-2.57237	0.643767	0.533308	-0.11046	10	73572537	F	II	CDH23	Body	opensea	Body-opens		rs1408849	1;2
cg1836228	0.110427	0.558835	2.508393	0.023783	0.227223	-4.68466	0.614049	0.503621	-0.11043	7	1.57E+08	R	I	DNAJB6	Body	island	Body-island	chr7:1572(		
cg1731254	0.114194	0.442828	3.23376	0.005421	0.224708	-3.25202	0.499924	0.385731	-0.11419	7	24561256	F	II		IGR	opensea	IGR-opense		rs1844744	5;23;28
cg2689901	0.101566	0.451694	3.232817	0.005431	0.224708	-3.25394	0.502478	0.400911	-0.10157	5	86042549	R	II		IGR	opensea	IGR-opense		rs5764025	37;4
cg2442625	0.110425	0.602989	2.55342	0.02174	0.225763	-4.59937	0.658201	0.547776	-0.11043	12	94703450	R	II	CEP83	Body	opensea	Body-opens		rs7336859	42
cg1840232	0.110407	0.582412	2.477077	0.02531	0.228534	-4.74361	0.637616	0.527209	-0.11041	12	1.29E+08	R	II	SLC15A4	Body	opensea	Body-opens		rs1461845	23
cg1131562	0.110345	0.481913	3.093388	0.007245	0.224708	-3.53698	0.537085	0.426741	-0.11034	17	57829517	R	II	VMP1	Body	opensea	Body-opens		rs5682089	49;34;15;1
cg1848967	0.110227	0.434658	3.890009	0.001391	0.224708	-1.89793	0.489772	0.379545	-0.11023	10	1408561	F	II	ADARB2	Body	shelf	Body-shelf	chr10:140	rs1140921	0;20;23;26
cg2171391	0.104918	0.500591	3.230657	0.005456	0.224708	-3.25834	0.55305	0.448132	-0.10492	5	1.04E+08	F	II		IGR	opensea	IGR-opense		rs1472804	35;42
cg0722341	0.105591	0.523034	3.230579	0.005457	0.224708	-3.2585	0.57583	0.470239	-0.10559	2	28668934	F	II		IGR	opensea	IGR-opense		rs6752004	15
cg2618953	0.105703	0.469752	3.229896	0.005464	0.224708	-3.25989	0.522604	0.416901	-0.1057	18	56791106	F	II		IGR	opensea	IGR-opense			
cg2687819	0.112138	0.684551	3.229641	0.005467	0.224708	-3.26042	0.74062	0.628482	-0.11214	5	1794420	R	II		IGR	opensea	IGR-opense		rs5319988	44
cg0357819	0.110153	0.490685	2.765927	0.014167	0.224708	-4.1891	0.545761	0.435608	-0.11015	2	63671845	F	II	C2orf86	Body	opensea	Body-opens		rs1121230	0;41
cg2037253	0.102272	0.630423	3.228304	0.005482	0.224708	-3.26314	0.68156	0.579287	-0.10227	1	1.55E+08	R	II		IGR	opensea	IGR-opense		rs5504320	45
cg1353051	0.100047	0.583884	3.228224	0.005483	0.224708	-3.2633	0.633908	0.53386	-0.10005	1	30951615	R	II		IGR	opensea	IGR-opense		rs6703542	42;39;14
cg1675590	0.103322	0.526978	3.227249	0.005494	0.224708	-3.26529	0.578638	0.475317	-0.10332	10	44567089	F	II		IGR	opensea	IGR-opense		rs5622879	0
cg2174558	0.11014	0.560804	3.286229	0.004863	0.224708	-3.14487	0.615873	0.505734	-0.11014	12	12518875	R	II	LOH12CR1	Body	opensea	Body-opens		rs5711745	16;7;1
cg1140446	0.118442	0.426391	3.226051	0.005508	0.224708	-3.26773	0.485612	0.36717	-0.11844	2	1.06E+08	R	II		IGR	opensea	IGR-opense			
cg1419365	0.110124	0.591353	3.692467	0.002094	0.224708	-2.30762	0.646415	0.536291	-0.11012	9	94178868	R	II	NFIL3	5'UTR	opensea	5'UTR-open			
cg0832329	0.103092	0.523448	3.224326	0.005528	0.224708	-3.27125	0.574994	0.471902	-0.10309	13	80563028	R	II		IGR	opensea	IGR-opense		rs5279103	12;1
cg1051172	0.108002	0.537156	3.223875	0.005533	0.224708	-3.27217	0.591158	0.483155	-0.108	14	24813353	F	II		IGR	opensea	IGR-opense			
cg2559802	0.119195	0.561842	3.223588	0.005536	0.224708	-3.27275	0.62144	0.502245	-0.1192	2	877141	F	II		IGR	opensea	IGR-opense		rs7315303	0;1
cg1026960	0.11012	0.488129	3.248593	0.005257	0.224708	-3.22176	0.543189	0.433069	-0.11012	4	1.2E+08	R	II	USP53	5'UTR	opensea	5'UTR-open		rs1469883	1
cg1590905	0.110057	0.524207	3.017376	0.008472	0.224708	-3.69011	0.579236	0.469179	-0.11006	12	71837175	R	II	LGR5	Body	shelf	Body-shelf	chr12:718	rs5607118	34;1
cg0505945	0.115409	0.465414	3.222426	0.00555	0.224708	-3.27512	0.523118	0.407709	-0.11541	11	13011156	F	II		IGR	opensea	IGR-opense			
cg1230198	0.110046	0.501675	3.002628	0.008733	0.224708	-3.71971	0.556698	0.446652	-0.11005	2	1E+08	R	II	AFF3	Body	opensea	Body-opens		rs1479200	47
cg1176927	0.11004	0.642794	2.704471	0.016046	0.224896	-4.30896	0.697814	0.587774	-0.11004	3	1.95E+08	F	II	XXYLT1	Body	opensea	Body-opens		rs5359511	1;45
cg1371440	0.110029	0.449709	3.234311	0.005415	0.224708	-3.25089	0.504724	0.394695	-0.11003	9	1.35E+08	F	II	RAPGEF1	Body	opensea	Body-opens		rs5474923	1;18
cg0082663	0.110028	0.511277	2.403072	0.029296	0.231808	-4.88164	0.566291	0.456263	-0.11003	19	46969832	F	II	PNMAL1	3'UTR	opensea	3'UTR-open		rs5392116	0;15
cg0464507	0.109391	0.449619	3.220266	0.005574	0.224708	-3.27952	0.504314	0.394923	-0.10939	11	34393106	F	II		IGR	opensea	IGR-opense		rs5693732	0;4;43
cg0554789	0.163382	0.461631	3.220121	0.005576	0.224708	-3.27982	0.543321	0.37994	-0.16338	3	1.97E+08	R	II		IGR	opensea	IGR-opense		rs5712945	23;15
cg1220181	0.110021	0.595131	2.312303	0.034997	0.237181	-5.04832	0.650141	0.54012	-0.11002	2	39493370	R	II	MAP4K3	Body	opensea	Body-opens		rs1750554	51;17;9
cg1166928	0.11002	0.318017	2.492149	0.024564	0.227897	-4.71528	0.373027	0.263007	-0.11002	5	37721782	R	I	WDR70	Body	opensea	Body-opens		rs5643034	25;2
cg1214826	0.126653	0.586795	3.218571	0.005594	0.224708	-3.28298	0.650122	0.523469	-0.12665	2	1.21E+08	F	II		IGR	shelf	IGR-shelf	chr2:1214	rs875825	20
cg2538101	0.110017	0.394115	3.389209	0.003928	0.224708	-2.93376	0.449124	0.339107	-0.11002	3	3151795	R	II	IL5RA	5'UTR	opensea	5'UTR-open		rs2290608	38
cg2590022	0.109994	0.701466	3.298935	0.004737	0.224708	-3.11888	0.756463	0.646469	-0.10999	4	1.23E+08	F	II	KIAA1109	Body	opensea	Body-opens		rs5416179	32
cg0794895	0.10309	0.547899	3.21599	0.005624	0.224708	-3.28823	0.599444	0.496354	-0.10309	6	45649741	R	II		IGR	opensea	IGR-opense		rs1432177	2
cg1784609	0.109972	0.46244	3.366513	0.004117	0.224708	-2.98038	0.517426	0.407454	-0.10997	3	1.57E+08	F	II	LINC00880	Body	opensea	Body-opens		rs5338856	1;14;23;39
cg1665226	0.11808	0.574044	3.211915	0.005672	0.224708	-3.29653	0.633084	0.515004	-0.11808	4	97948716	F	II		IGR	opensea	IGR-opense		rs5625731	0;24
cg1432935	0.109965	0.527824	3.514474	0.003029	0.224708	-2.67577	0.582807	0.472842	-0.10996	11	62573880	F	II	NXF1	TSS1500	shore	TSS1500-sh	chr11:625	rs5425435	9;33
cg0719877	0.10991	0.494556	2.520241	0.023228	0.226763	-4.66228	0.549511	0.439602	-0.10991	7	92439762	R	II	CDK6	Body	opensea	Body-opens		rs1878481	40;32;28;1
cg0647328	0.109873	0.448831	3.067991	0.007634	0.224708	-3.58825	0.503767	0.393894	-0.10987	6	32820102	R	II	TAP1	Body	shore	Body-shore	chr6:3282(		
cg2621968	0.10419	0.465658	3.209719	0.005697	0.224708	-3.301	0.517752	0.413563	-0.10419	2	58568596	F	II		IGR	opensea	IGR-opense		rs1812388	8;15;29
cg2353245	0.109845	0.602103	2.775911	0.013883	0.224708	-4.16954	0.657025	0.54718	-0.10985	14	75910026	F	II	JDP2	Body	opensea	Body-opens		rs1394330	0
cg0404563	0.104759	0.563014	3.207504	0.005723	0.224708	-3.30551	0.615393	0.510634	-0.10476	2	1.96E+08	F	II		IGR	opensea	IGR-opense		rs5421717	4;20;50
cg1212026	0.109815	0.459385	2.594563	0.020022	0.225563	-4.5209	0.514292	0.404478	-0.10981	5	1.26E+08	F	II	ALDH7A1	Body	opensea	Body-opens		rs1437451	0;29;32
cg0720663	0.109803	0.584586	3.447606	0.00348	0.224708	-2.81364	0.639487	0.529684	-0.1098	5	1.42E+08	R	II	FGF1	5'UTR	opensea	5'UTR-open		rs1407738	15;6;1
cg0055461	0.103058	0.504164	3.204435	0.00576	0.224708	-3.31176	0.555693	0.452635	-0.10306	16	79747651	R	II		IGR	opensea	IGR-opense		rs5353848	48;32;1
cg2630569	0.109797	0.608189	3.86484	0.001466	0.224708	-1.95016	0.663088	0.553291	-0.1098	12	442192	R	II	KDM5A	Body	opensea	Body-opens		rs1154946	29;1
cg1756034	0.109774	0.474431	3.212402	0.005666	0.224708	-3.29554	0.529318	0.419544	-0.10977	17	684490	F	II	RNMTL1	TSS1500	shore	TSS1500-sh	chr17:685	rs7397552	7;31;44
cg0005222	0.109754	0.632093	3.568966	0.002705	0.224708	-2.56323	0.686969	0.577216	-0.10975	12	15783053	F	II	EPS8	Body	opensea	Body-opens		rs5569469	0;31;48
cg1423265	0.109664	0.52869	3.168259	0.006207	0.224708	-3.38531	0.583522	0.473858	-0.10966	14	45564553	F	II	PRPF39	Body	opensea	Body-opens		rs5370135	1;27;31
cg0454131	0.109584	0.43952	3.554613	0.002787	0.224708	-2.59289	0.494312	0.384728	-0.10958	15	33897878	F	II	RYR3	Body	opensea	Body-opens		rs5532466	7;43
cg1896824	0.109514	0.457976	3.540488	0.00287	0.224708	-2.62207	0.512733	0.40322	-0.10951	4	1.24E+08	F	II	IL21-AS1	Body	opensea	Body-opens		rs1143013	9
cg1720706	0.109503	0.64449	2.793208	0.013403	0.224708	-4.1356	0.699241	0.589738	-0.1095	5	95624196	F	II	LOC10192	Body	opensea	Body-opens		rs5425205	0;51
cg1304189	0.100854	0.735801	3.201689	0.005793	0.224708	-3.31735	0.786228	0.685374	-0.10085	22	47609321	F	II		IGR	island	IGR-island	chr22:476(	rs1454939	0;13
cg0592415	0.10529	0.561304	3.201636	0.005793	0.224708	-3.31746	0.613949	0.508659	-0.10529	8	1.33E+08	F	II		IGR	opensea	IGR-opense			
cg1963310	0.109493	0.485039	3.547172	0.002831	0.224708	-2.60826	0.539785	0.430292	-0.10949	13	50028789	R	II	SETDB2	Body	opensea	Body-opens		rs1853041	1
cg2522382	0.109487	0.466476	3.736624	0.001911	0.224708	-2.21611	0.521219	0.411732	-0.10949	13	33593349	F	II	KL	Body	shore	Body-shore	chr13:335	rs1919689	1;4
cg1093780	0.108684	0.379657	3.198415	0.005832	0.224708	-3.32401	0.433999	0.325315	-0.10868	4	26177098	F	I		IGR	opensea	IGR-opense		rs6847292	12
cg0018217	0.109484	0.572991	2.848197	0.011983	0.224708	-4.02726	0.627733	0.518248	-0.10948	9	21852933	R	II	MTAP	Body	opensea	Body-opens			

cg0093262	0.109478	0.656527	3.859664	0.001482	0.224708	-1.9609	0.711266	0.601788	-0.10948	14	67975805	F	II	TMEM229B	5'UTR	opensea	5'UTR-open		rs1147882	0;1;17
cg1018477	0.101514	0.476671	3.195606	0.005866	0.224708	-3.32973	0.527428	0.425914	-0.10151	17	62102410	R	II		IGR	opensea	IGR-opense		rs5323188	35;33;26;10;8
cg0519923	0.10865	0.361547	3.195065	0.005873	0.224708	-3.33083	0.415872	0.307222	-0.10865	12	43548942	F	II		IGR	opensea	IGR-opense		rs5299120	23;34
cg1458429	0.109465	0.357678	2.545272	0.022097	0.226019	-4.61485	0.41241	0.302946	-0.10946	11	1.22E+08	R	II	MIR100HG	Body	opensea	Body-opens			
cg1145849	0.109459	0.600297	3.884582	0.001407	0.224708	-1.90919	0.655027	0.545568	-0.10946	5	1.68E+08	F	II	SLIT3	Body	opensea	Body-opens		rs5539306	0;7;19;46
cg0236772	0.109448	0.621812	4.29299	0.000608	0.224708	-1.06308	0.676536	0.567088	-0.10945	1	46378857	F	II	MAST2	Body	opensea	Body-opens		rs5390530	2;49
cg2185241	0.10944	0.545509	2.794669	0.013363	0.224708	-4.13273	0.600229	0.49079	-0.10944	2	2.04E+08	F	II	ICA1L	Body	opensea	Body-opens		rs5511272	1;30
cg0616053	0.109413	0.725199	2.889854	0.011005	0.224708	-3.94476	0.779906	0.670493	-0.10941	1	2.45E+08	R	II	ADSS	Body	opensea	Body-opens			
cg2266034	0.109411	0.514796	3.204309	0.005761	0.224708	-3.31202	0.569501	0.46009	-0.10941	20	48322124	R	II	B4GALT5	Body	opensea	Body-opens		rs1509631	38;20;16;11;2;1
cg0036087	0.109391	0.658759	4.024428	0.001055	0.224708	-1.61906	0.713454	0.604063	-0.10939	5	1.74E+08	R	II	FLJ16171	Body	opensea	Body-opens		rs5659952	7;1
cg1912796	0.109366	0.558099	2.886795	0.011074	0.224708	-3.95083	0.612782	0.503416	-0.10937	4	40827206	R	II	APBB2	Body	opensea	Body-opens		rs5492178	30;19;2
cg0715542	0.10569	0.492646	3.189978	0.005935	0.224708	-3.34118	0.54549	0.439801	-0.10569	1	12114402	R	II		IGR	opensea	IGR-opense			
cg1231117	0.109332	0.547334	2.908905	0.010584	0.224708	-3.90691	0.602	0.492668	-0.10933	4	1.19E+08	F	II	NDST3	Body	opensea	Body-opens		rs1384381	28
cg1563888	0.109242	0.688999	4.721155	0.000256	0.224708	-0.18437	0.743619	0.634378	-0.10924	8	17212177	F	II	MTMR7	Body	opensea	Body-opens		rs7594166	13;31;32;36;49
cg1556652	0.109221	0.494079	3.320044	0.004534	0.224708	-3.07567	0.548689	0.439468	-0.10922	6	38068342	R	II	ZFAND3	Body	opensea	Body-opens		rs3738140	3
cg1316349	0.109216	0.639349	2.72687	0.015335	0.224819	-4.26538	0.693957	0.584741	-0.10922	1	2.16E+08	R	II	KCTD3	Body	opensea	Body-opens		rs5289969	10;1
cg1548622	0.10919	0.429803	3.914873	0.001322	0.224708	-1.84634	0.484398	0.375208	-0.10919	8	1.02E+08	F	II	ANKRD46	Body	opensea	Body-opens		rs5345086	30
cg0325222	0.106429	0.529166	3.186464	0.005978	0.224708	-3.34832	0.582381	0.475952	-0.10643	9	22214533	R	II		IGR	opensea	IGR-opense		rs5750912	1
cg0481141	0.10915	0.379342	2.765531	0.014179	0.224708	-4.18987	0.433917	0.324767	-0.10915	1	1.74E+08	R	II	SNORD80	TSS1500	shelf	TSS1500-sh	chr1:1738	rs5374956	24;7;2
cg0632875	0.101422	0.580447	3.184737	0.005999	0.224708	-3.35183	0.631158	0.529736	-0.10142	7	1.57E+08	R	II		IGR	shore	IGR-shore	chr7:1573	rs5724925	51;23;8;2
cg1422072	0.10913	0.714492	4.029583	0.001043	0.224708	-1.60837	0.769057	0.659927	-0.10913	4	1.02E+08	R	II	PPP3CA	Body	opensea	Body-opens			
cg0577212	0.109119	0.466823	3.021025	0.008409	0.224708	-3.68278	0.521382	0.412263	-0.10912	16	31539169	R	II	AHSP	TSS200	opensea	TSS200-ope		rs7749354	15;2;1
cg0321021	0.109096	0.58188	4.43695	0.000454	0.224708	-0.76632	0.636428	0.527332	-0.1091	5	1.19E+08	R	I	TNFAIP8	Body	shore	Body-shore	chr5:1186	rs1134299	36
cg1470355	0.107362	0.484541	3.183264	0.006018	0.224708	-3.35483	0.538222	0.43086	-0.10736	1	48670405	F	II		IGR	opensea	IGR-opense		rs1390769	1;4;10;48;49
cg1582209	0.106241	0.600589	3.183198	0.006018	0.224708	-3.35496	0.65371	0.547469	-0.10624	21	26561322	R	II		IGR	opensea	IGR-opense		rs5343791	48;12
cg2396735	0.10909	0.567579	3.378279	0.004018	0.224708	-2.95622	0.622123	0.513034	-0.10909	5	1.06E+08	R	II	LOC10246	Body	opensea	Body-opens		rs1924286	36;14
cg2339701	0.108801	0.469584	3.182542	0.006027	0.224708	-3.35629	0.523984	0.415183	-0.1088	10	10571132	F	II		IGR	opensea	IGR-opense			
cg1720857	0.100609	0.64389	3.182433	0.006028	0.224708	-3.35652	0.694194	0.593586	-0.10061	9	1.11E+08	F	II		IGR	opensea	IGR-opense		rs5649433	13;27
cg1244350	0.102189	0.497056	3.182238	0.00603	0.224708	-3.35691	0.54815	0.445961	-0.10219	2	1.14E+08	F	II		IGR	opensea	IGR-opense		rs5491095	9
cg0052437	0.100566	0.305625	3.180781	0.006049	0.224708	-3.35987	0.355908	0.255342	-0.10057	10	3894968	F	II		IGR	opensea	IGR-opense		rs1456447	0;1;24;39
cg0027566	0.109076	0.499979	2.860994	0.011674	0.224708	-4.00195	0.554516	0.445441	-0.10908	20	48317973	R	II	B4GALT5	Body	opensea	Body-opens		rs1414485	1
cg0223925	0.140476	0.606008	3.179817	0.006061	0.224708	-3.36183	0.676246	0.53577	-0.14048	8	8241752	F	II		IGR	shore	IGR-shore	chr8:8243	rs2976956	20
cg1443827	0.109069	0.488732	2.973516	0.009271	0.224708	-3.77803	0.543266	0.434198	-0.10907	5	1.43E+08	F	II	NR3C1	5'UTR	opensea	5'UTR-open			
cg0236070	0.109063	0.455339	3.321866	0.004517	0.224708	-3.07193	0.51007	0.401007	-0.10906	5	1.45E+08	R	II	SH3RF2	5'UTR	shore	5'UTR-shor	chr5:1453	rs5458217	0;4;8;19
cg1652994	0.109024	0.682165	3.15286	0.006408	0.224708	-3.41657	0.736677	0.627653	-0.10902	10	98896466	F	II	SLIT1	Body	opensea	Body-opens		rs1427190	0;45
cg2149585	0.115706	0.394102	3.177606	0.006088	0.224708	-3.36633	0.451956	0.336249	-0.11571	1	2.22E+08	R	II		IGR	opensea	IGR-opense		rs1886349	42;2;1
cg1735582	0.109023	0.434783	3.042672	0.008043	0.224708	-3.63925	0.489294	0.380271	-0.10902	14	72516174	R	II	RGS6	5'UTR	opensea	5'UTR-open		rs5482225	22;16;4;2
cg2102814	0.109011	0.465191	2.338115	0.033277	0.235474	-5.00123	0.519696	0.410685	-0.10901	17	79581711	F	II	NPLOC4	Body	opensea	Body-opens		rs5644910	42;43
cg0972886	0.108993	0.429319	2.150841	0.047779	0.252347	-5.33691	0.483815	0.374822	-0.10899	15	85951572	R	II	AKAP13	5'UTR	opensea	5'UTR-open		rs5523923	14
cg2697586	0.10899	0.4799	2.955325	0.009624	0.224708	-3.81439	0.534395	0.425404	-0.10899	1	57590662	F	II	DAB1	Body	opensea	Body-opens		rs5557882	1;26
cg1720687	0.135327	0.647202	3.174804	0.006124	0.224708	-3.37202	0.714866	0.579539	-0.13533	8	74983376	R	II		IGR	opensea	IGR-opense		rs1124376	51;40
cg2586738	0.108959	0.515989	2.780653	0.01375	0.224708	-4.16024	0.570469	0.46151	-0.10896	8	27936225	R	II	NUGGC	5'UTR	opensea	5'UTR-open		rs5599172	6;2;1
cg1349344	0.106993	0.610927	3.174018	0.006134	0.224708	-3.37362	0.664424	0.557431	-0.10699	3	5833716	F	II		IGR	opensea	IGR-opense		rs5601762	0;1
cg1773152	0.108956	0.625417	3.535715	0.002899	0.224708	-2.63192	0.679895	0.570939	-0.10896	3	27235572	R	II	NEK10	Body	opensea	Body-opens		rs1385442	51;15;6
cg0664565	0.108935	0.586181	2.941633	0.009898	0.224708	-3.84172	0.640648	0.531714	-0.10893	2	1.53E+08	F	II	CACNB4	Body	opensea	Body-opens		rs1488132	0;1;30;36;43;51
cg2189452	0.108929	0.607749	2.653969	0.017768	0.225076	-4.40675	0.662214	0.553285	-0.10893	1	1.85E+08	R	II	FAM129A	Body	opensea	Body-opens		rs5773163	40
cg1714394	0.108925	0.492319	3.059563	0.007768	0.224708	-3.60524	0.546782	0.437857	-0.10893	12	1.17E+08	R	II	FBXW8	Body	opensea	Body-opens		rs1458689	40;18;2
cg0475741	0.108884	0.553352	3.116785	0.006903	0.224708	-3.48967	0.607794	0.49891	-0.10888	13	76259545	F	II	LMO7	Body	opensea	Body-opens		rs7483109	21;30
cg1053374	0.108876	0.675268	3.596307	0.002556	0.224708	-2.5067	0.729706	0.62083	-0.10888	10	1400014	F	II	ADARB2	Body	opensea	Body-opens		rs1485166	22;49
cg1542579	0.106879	0.481935	3.168303	0.006207	0.224708	-3.38523	0.535375	0.428496	-0.10688	21	43820702	R	II		IGR	opensea	IGR-opense		rs5284330	49;32;1
cg2263182	0.108847	0.458093	2.797029	0.013299	0.224708	-4.12809	0.512517	0.40367	-0.10885	6	1.44E+08	R	II	ZC2HC1B	Body	opensea	Body-opens			
cg1788306	0.108825	0.645275	3.466991	0.003343	0.224708	-2.7737	0.699688	0.590863	-0.10883	15	57277634	R	II	TCF12	Body	opensea	Body-opens			
cg1159481	0.108801	0.512662	3.06261	0.007719	0.224708	-3.59909	0.567063	0.458262	-0.1088	11	1.15E+08	F	II	CADM1	Body	opensea	Body-opens		rs3774602	2;14;20;21;23
cg1795101	0.115165	0.448948	3.165989	0.006236	0.224708	-3.38993	0.506531	0.391365	-0.11517	11	59972522	F	II		IGR	opensea	IGR-opense		rs1907343	29
cg2329546	0.108799	0.513169	3.077003	0.007494	0.224708	-3.57007	0.567569	0.458769	-0.1088	7	87919037	F	II	STEAP4	5'UTR	opensea	5'UTR-open		rs5337284	0;30
cg2421127	0.108781	0.446708	3.987875	0.001137	0.224708	-1.69488	0.501098	0.392317	-0.10878	14	1.01E+08	F	II	WDR25	Body	opensea	Body-opens		rs5416025	30
cg0554047	0.112645	0.367677	3.16529	0.006245	0.224708	-3.39135	0.424	0.311355	-0.11265	3	1.97E+08	R	II		IGR	shore	IGR-shore	chr3:1971	rs5438732	1
cg0138533	0.108769	0.644898	2.155689	0.047339	0.251776	-5.32841	0.699282	0.590513	-0.10877	2	1.28E+08	F	II	MYO7B	Body	shore	Body-shore	chr2:1283	rs1142165	0;17
cg0275513	0.108764	0.583784	2.689929	0.016525	0.224896	-4.33719	0.638166	0.529402	-0.10876	4	1.84E+08	F	II	WWC2	Body	opensea	Body-opens		rs1045480	15;46
cg0571620	0.122404	0.478145	3.164019	0.006262	0.224708	-3.39393	0.539347	0.416944	-0.1224	4	1.75E+08	R	II		IGR	opensea	IGR-opense		rs5379179	50;48;8;2;1
cg1554257	0.109143	0.460568	3.162214	0.006285	0.224708	-3.39759	0.515139	0.405997	-0.10914	6	1.51E+08	R	II		IGR	opensea	IGR-opense			
cg0826803	0.121065	0.419123	3.162014	0.006288	0.224708	-3.39799	0.479656	0.358591	-0.12107	1	2.3E+08	R	II		IGR	opensea	IGR-opense			
cg1643564	0.108721	0.395622	3.470432	0.003319	0.224708	-2.76661	0.449982	0.341261	-0.10872	6	1.28E+08	F	II	THEMIS	5'UTR	opensea	5'UTR-open		rs5667728	19
cg2213009	0.108709	0.620735	3.342827	0.004325	0.224708	-3.02897	0.675089	0.566381	-0.10871	2	59098148	F	II	LINC01122	Body	opensea	Body-opens		rs7553148	0;6;26

cg0247252	0.108681	0.703505	2.868286	0.011501	0.224708	-3.98752	0.757846	0.649165	-0.10868	1	1E+08	F	II	PALMD	Body	opensea	Body-opens		rs1162021 47
cg0187172	0.108663	0.43957	4.232715	0.000688	0.224708	-1.18764	0.493901	0.385238	-0.10866	12	32120103	R	II	C12orf35	5'UTR	opensea	5'UTR-open		rs5630743 33;22;19;11
cg1600159	0.109414	0.577304	3.157854	0.006342	0.224708	-3.40644	0.632011	0.522597	-0.10941	10	3800109	R	II		IGR	opensea	IGR-opense		rs1895452 37;35;2;1
cg1287001	0.102166	0.627653	3.157131	0.006352	0.224708	-3.40791	0.678736	0.57657	-0.10217	10	3847895	F	II		IGR	opensea	IGR-opense		
cg2484473	0.105187	0.641623	3.156847	0.006355	0.224708	-3.40848	0.694216	0.58903	-0.10519	21	47075042	F	II		IGR	opensea	IGR-opense		rs1918137 0;46
cg0075719	0.108663	0.427339	2.7635	0.014237	0.224708	-4.19385	0.481671	0.373007	-0.10866	3	1.85E+08	F	II	IGF2BP2	Body	opensea	Body-opens		rs1811918 1
cg0355697	0.108648	0.550115	3.196077	0.00586	0.224708	-3.32877	0.604439	0.495791	-0.10865	13	24249366	F	II	TNFRSF19	3'UTR	opensea	3'UTR-open		rs5426259 0
cg0888230	0.104913	0.46032	3.15452	0.006386	0.224708	-3.41321	0.512776	0.407863	-0.10491	19	29489929	R	II		IGR	opensea	IGR-opense		rs1404326 46;24;2
cg2017532	0.108627	0.479531	2.770765	0.014029	0.224708	-4.17963	0.533844	0.425217	-0.10863	13	50858812	F	II	DLEU1	Body	opensea	Body-opens		rs5378112 0
cg0075782	0.108597	0.557229	2.509818	0.023715	0.227118	-4.68197	0.611528	0.502931	-0.1086	3	1.55E+08	R	II	PLCH1	Body	opensea	Body-opens		rs5756424 33
cg1040107	0.108572	0.436901	3.374242	0.004052	0.224708	-2.96451	0.491187	0.382615	-0.10857	1	66673501	R	II	PDE4B	Body	opensea	Body-opens		rs5399550 45;26;24;1
cg2579131	0.108543	0.550671	2.911119	0.010537	0.224708	-3.9025	0.604943	0.496399	-0.10854	14	1E+08	R	II	EML1	Body	opensea	Body-opens		rs1380450 2;1
cg2440142	0.108527	0.626934	4.311811	0.000585	0.224708	-1.02422	0.681197	0.57267	-0.10853	5	77014952	R	II	TBCA	Body	opensea	Body-opens		rs5481307 1
cg1812171	0.108504	0.492728	2.809669	0.012962	0.224708	-4.10324	0.54698	0.438476	-0.1085	2	17738759	F	II	VSNL1	5'UTR	opensea	5'UTR-open		rs5559629 0;8;15;32
cg0979825	0.108488	0.522226	2.923427	0.010274	0.224708	-3.87801	0.57647	0.467982	-0.10849	8	59763565	R	II	TOX	Body	opensea	Body-opens		rs5524859 30
cg0612731	0.108463	0.483689	3.006494	0.008664	0.224708	-3.71195	0.537921	0.429458	-0.10846	11	9599460	R	II	WEE1	Body	shelf	Body-shelf	chr11:959	rs5500584 35;1
cg0745533	0.108429	0.776295	3.052917	0.007875	0.224708	-3.61863	0.830509	0.72208	-0.10843	1	62219881	R	II	INADL	5'UTR	opensea	5'UTR-open		rs5532823 35;1
cg2521453	0.108425	0.76686	4.521241	0.000383	0.224708	-0.59312	0.821073	0.712648	-0.10842	1	1.86E+08	F	II	PDC	5'UTR	opensea	5'UTR-open		
cg2329127	0.108421	0.6084	3.14892	0.00646	0.224708	-3.42456	0.662611	0.55419	-0.10842	12	15866615	F	II	EPS8	5'UTR	opensea	5'UTR-open		rs5346277 50
cg2302587	0.108403	0.585164	3.436966	0.003558	0.224708	-2.83554	0.639366	0.530963	-0.1084	7	77487860	F	II	PHTF2	Body	opensea	Body-opens		rs5363026 41;41
cg2117895	0.108377	0.401394	2.414235	0.028659	0.231242	-4.86094	0.455583	0.347206	-0.10838	5	1.49E+08	R	II	ABLIM3	Body	opensea	Body-opens		rs7953774 48;44
cg0066987	0.108366	0.413636	4.013084	0.00108	0.224708	-1.64259	0.467819	0.359453	-0.10837	12	59195970	F	II	LOC10050	Body	opensea	Body-opens		rs5675286 8
cg2547879	0.108355	0.620851	3.177339	0.006092	0.224708	-3.36687	0.675028	0.566674	-0.10835	8	1.41E+08	R	II	TRAPPC9	Body	opensea	Body-opens		rs5703298 34;30;10
cg1237813	0.17006	0.372018	3.140933	0.006568	0.224708	-3.44076	0.457048	0.286988	-0.17006	3	1.97E+08	R	II		IGR	opensea	IGR-opense		rs1143720 48;13
cg0062543	0.118346	0.52307	3.140498	0.006574	0.224708	-3.44164	0.582243	0.463897	-0.11835	11	34706961	R	II		IGR	opensea	IGR-opense		rs5525057 48;42;1
cg1857398	0.104983	0.561013	3.139978	0.006581	0.224708	-3.4427	0.613505	0.508522	-0.10498	2	85908284	F	II		IGR	opensea	IGR-opense		rs1393303 0
cg1424183	0.100299	0.567947	3.139702	0.006584	0.224708	-3.44326	0.618097	0.517798	-0.1003	7	48124921	R	II		IGR	shelf	IGR-shelf	chr7:4812	rs5325277 1
cg2069646	0.108158	0.534119	3.139274	0.00659	0.224708	-3.44412	0.588198	0.48004	-0.10816	16	54277492	R	II		IGR	opensea	IGR-opense		
cg0536068	0.10434	0.416201	3.139217	0.006591	0.224708	-3.44424	0.468371	0.364031	-0.10434	14	1.06E+08	R	II		IGR	opensea	IGR-opense		rs1857465 2
cg2710004	0.106166	0.543427	3.138144	0.006606	0.224708	-3.44641	0.59651	0.490344	-0.10617	20	49311875	F	II		IGR	opensea	IGR-opense		rs5286891 0;1;2;26;26;29
cg0551627	0.103765	0.603881	3.138106	0.006606	0.224708	-3.44649	0.655763	0.551998	-0.10376	10	60237728	R	II		IGR	opensea	IGR-opense		rs5423898 11
cg0296844	0.108344	0.42784	2.837139	0.012256	0.224708	-4.0491	0.482012	0.373668	-0.10834	2	68972158	F	II	ARHGAP25	Body	opensea	Body-opens		rs5400394 0;1
cg0842826	0.108314	0.573128	4.241961	0.000675	0.224708	-1.16853	0.627285	0.51897	-0.10831	7	55238412	F	II	EGFR	3'UTR	opensea	3'UTR-open		rs5651051 6;44;47;49
cg1305295	0.108233	0.381177	5.668156	4.07E-05	0.208563	1.699371	0.435293	0.32706	-0.10823	1	37467416	F	II	GRIK3	Body	opensea	Body-opens		rs5567807 50
cg1284737	0.108226	0.458713	2.682815	0.016764	0.224901	-4.35098	0.512826	0.4046	-0.10823	19	13056058	R	II	RAD23A	TSS1500	shore	TSS1500-sh	chr19:130	
cg2425654	0.114157	0.50757	3.133254	0.006673	0.224708	-3.45632	0.564649	0.450492	-0.11416	12	1.02E+08	R	II		IGR	opensea	IGR-opense		rs1122641 49;21;2
cg1714101	0.100683	0.644961	3.132662	0.006681	0.224708	-3.45752	0.695303	0.594619	-0.10068	1	1.69E+08	F	II		IGR	opensea	IGR-opense		rs5660502 28;45
cg1873597	0.108183	0.47872	3.276565	0.004961	0.224708	-3.16463	0.532811	0.424628	-0.10818	1	2.08E+08	R	II	CD46	Body	shelf	Body-shelf	chr1:2079	rs1178757 26;1
cg0560415	0.101077	0.381341	3.132088	0.006689	0.224708	-3.45869	0.431879	0.330802	-0.10108	2	1.78E+08	R	II		IGR	opensea	IGR-opense		rs5407355 24;2;1
cg0437525	0.108159	0.392243	3.082158	0.007414	0.224708	-3.55966	0.446322	0.338164	-0.10816	5	66241308	F	II	MAST4	Body	opensea	Body-opens		rs1150686 0;23;42
cg0623543	0.108156	0.450129	3.814984	0.001625	0.224708	-2.05359	0.504207	0.396051	-0.10816	16	30485966	F	II	ITGAL	Body	shore	Body-shore	chr16:304	rs5437776 3;47
cg1808751	0.108103	0.553738	2.87769	0.011282	0.224708	-3.96888	0.60779	0.499687	-0.1081	1	2.42E+08	F	II	KMO	Body	opensea	Body-opens		rs7709607 1;36
cg0608900	0.11858	0.528499	3.130108	0.006716	0.224708	-3.4627	0.587789	0.469209	-0.11858	15	50427638	F	II		IGR	opensea	IGR-opense		rs1749960 8;39
cg0141639	0.108101	0.437614	3.184505	0.006002	0.224708	-3.3523	0.491664	0.383564	-0.1081	20	55806433	R	II	BMP7	Body	opensea	Body-opens		rs5555885 36;14
cg2119172	0.112951	0.585407	3.12896	0.006732	0.224708	-3.46502	0.641882	0.528931	-0.11295	20	46086203	F	II		IGR	opensea	IGR-opense		rs1180112 1;50
cg1232214	0.117223	0.645164	3.128949	0.006732	0.224708	-3.46505	0.703775	0.586552	-0.11722	3	29197502	R	II		IGR	opensea	IGR-opense		rs4680717 37;2
cg2358205	0.108085	0.645074	3.281007	0.004916	0.224708	-3.15555	0.699117	0.591032	-0.10808	3	3152201	R	II	IL5RA	TSS200	opensea	TSS200-ope		rs1132542 28;17;12;9
cg2694621	0.100561	0.720573	3.128278	0.006742	0.224708	-3.4664	0.770853	0.670292	-0.10056	2	1.21E+08	R	II		IGR	opensea	IGR-opense		rs1807187 44;34;1
cg1754975	0.108083	0.669544	3.488257	0.003199	0.224708	-2.72986	0.723585	0.615502	-0.10808	14	33981965	R	II	NPAS3	Body	opensea	Body-opens		rs8012701 37;1
cg0847861	0.108051	0.546812	2.975537	0.009233	0.224708	-3.77398	0.600838	0.492787	-0.10805	10	48372128	R	II	ZNF488	3'UTR	shore	3'UTR-shore	chr10:483	rs5355257 2
cg1486616	0.10154	0.507477	3.127371	0.006754	0.224708	-3.46824	0.558247	0.456707	-0.10154	8	1.3E+08	R	II		IGR	opensea	IGR-opense		rs1863095 48
cg1124864	0.108046	0.546275	2.855712	0.0118	0.224708	-4.0124	0.600298	0.492252	-0.10805	12	50645711	F	II	LIMA1	5'UTR	opensea	5'UTR-open		rs5773929 0
cg0173693	0.108045	0.54808	3.391165	0.003912	0.224708	-2.92974	0.602102	0.494057	-0.10804	2	1.06E+08	F	II	FHL2	TSS200	opensea	TSS200-ope		rs1122585 4;12;13
cg1815668	0.107558	0.388269	3.125623	0.006779	0.224708	-3.47178	0.442048	0.33449	-0.10756	6	53097064	R	II		IGR	opensea	IGR-opense		rs1490449 16;4;1
cg2733558	0.108045	0.506107	2.759143	0.014364	0.224708	-4.20238	0.560129	0.452085	-0.10804	5	95616198	F	II	LOC10192	Body	opensea	Body-opens		
cg1155498	0.107939	0.652956	3.292908	0.004796	0.224708	-3.13121	0.706926	0.598987	-0.10794	14	32937945	R	II	AKAP6	Body	opensea	Body-opens		rs1163562 40;34;11
cg0089229	0.119975	0.466058	3.121713	0.006834	0.224708	-3.4797	0.526046	0.406071	-0.11998	13	49323918	F	II		IGR	opensea	IGR-opense		rs5729498 32
cg0487384	0.100931	0.486906	3.120992	0.006844	0.224708	-3.48116	0.537371	0.43644	-0.10093	8	1.01E+08	F	II		IGR	opensea	IGR-opense		rs5628726 0
cg1085327	0.102967	0.622351	3.120462	0.006851	0.224708	-3.48223	0.673834	0.570867	-0.10297	8	86436099	R	II		IGR	opensea	IGR-opense		rs1918059 29
cg0130600	0.111287	0.600563	3.119874	0.006859	0.224708	-3.48342	0.656206	0.544919	-0.11129	2	24802612	F	II		IGR	opensea	IGR-opense		rs5566489 0;17
cg1744819	0.107936	0.399824	3.055448	0.007834	0.224708	-3.61353	0.453793	0.345856	-0.10794	3	1.87E+08	R	II	ST6GAL1	5'UTR	opensea	5'UTR-open		rs1848526 29;25
cg2474607	0.107897	0.742764	3.801976	0.001669	0.224708	-2.08058	0.796713	0.688816	-0.1079	10	1286380	F	II	ADARB2	Body	shore	Body-shore	chr10:128	rs1886741 30;37;47
cg0401550	0.107865	0.765704	2.617872	0.019107	0.225391	-4.47623	0.819636	0.711771	-0.10787	13	1.13E+08	R	I	TUBGCP3	Body	island	Body-island	chr13:113	rs5531276 51;31;30;15;12
cg0327856	0.107857	0.628998	3.363814	0.004141	0.224708	-2.98592	0.682926	0.575069	-0.10786	5	5166415	F	II	ADAMTS16	Body	opensea	Body-opens		rs3462702 0;3

cg0680570	0.107843	0.549267	3.679044	0.002153	0.224708	-2.33543	0.603189	0.495346	-0.10784	16	81160871	F	II	PKD1L2	Body	opensea	Body-opens		rs3408037	11
cg0389256	0.107831	0.673978	3.219853	0.005579	0.224708	-3.28036	0.727894	0.620062	-0.10783	1	1.45E+08	R	II	PDE4DIP	Body	shore	Body-shore	chr1:1450		
cg1393153	0.103957	0.710959	3.11365	0.006948	0.224708	-3.49602	0.762938	0.658981	-0.10396	9	19791354	F	II		IGR	shelf	IGR-shelf	chr9:1978		
cg1258012	0.107728	0.583629	3.111895	0.006973	0.224708	-3.49957	0.637492	0.529765	-0.10773	4	1.53E+08	F	II		IGR	opensea	IGR-opense		rs5774945	1;35
cg0429653	0.107816	0.402065	3.20342	0.005772	0.224708	-3.31383	0.455973	0.348157	-0.10782	14	95778707	F	II	CLMN	Body	opensea	Body-opens		rs1928476	0;1;7;26
cg1102206	0.107783	0.512665	3.544507	0.002846	0.224708	-2.61376	0.566556	0.458773	-0.10778	15	83298969	F	II	CPEB1	Body	opensea	Body-opens			
cg2406791	0.107783	0.547785	2.994637	0.008878	0.224708	-3.73573	0.601676	0.493894	-0.10778	6	16729610	F	II	ATXN1	5'UTR	opensea	5'UTR-open			
cg2038076	0.107719	0.583278	2.655825	0.017702	0.225076	-4.40317	0.637138	0.529419	-0.10772	7	1.3E+08	F	II	MEST	TSS1500	shore	TSS1500-sh	chr7:1301	rs5505774	37
cg0673878	0.107641	0.418108	3.227215	0.005495	0.224708	-3.26536	0.471928	0.364287	-0.10764	20	19265058	R	II	LOC10013(	Body	opensea	Body-opens		rs3692381	49;42
cg0138037	0.107641	0.560474	2.876748	0.011304	0.224708	-3.97075	0.614294	0.506653	-0.10764	4	40765329	F	II	NSUN7	Body	opensea	Body-opens		rs5355926	6;7
cg1267218	0.124806	0.573661	3.108142	0.007028	0.224708	-3.50716	0.636064	0.511258	-0.12481	6	32427868	R	II		IGR	opensea	IGR-opense		rs2873223	29
cg2323177	0.11633	0.584695	3.107386	0.007039	0.224708	-3.50869	0.642861	0.52653	-0.11633	1	1.69E+08	R	II		IGR	opensea	IGR-opense		rs5602669	2;1
cg1956153	0.107611	0.487382	2.740271	0.014924	0.224708	-4.23925	0.541187	0.433576	-0.10761	5	1.31E+08	F	II	RAPGEF6	Body	opensea	Body-opens		rs1922406	0;12;27
cg2517992	0.107574	0.724168	3.238544	0.005367	0.224708	-3.24226	0.777955	0.670382	-0.10757	8	16959530	R	II	MICU3	Body	opensea	Body-opens		rs5549638	51;9;5
cg0634918	0.107566	0.676893	3.167667	0.006215	0.224708	-3.38652	0.730676	0.62311	-0.10757	1	1.78E+08	R	II	RASAL2	Body	opensea	Body-opens		rs1853940	40;36;31;26;4;1
cg0620828	0.141143	0.221139	3.106753	0.007048	0.224708	-3.50997	0.291711	0.150568	-0.14114	1	58858074	R	I		IGR	opensea	IGR-opense		rs849493	17
cg1961950	0.104987	0.429337	3.106402	0.007053	0.224708	-3.51068	0.48183	0.376843	-0.10499	11	85850481	F	II		IGR	opensea	IGR-opense		rs1504211	10;30;36;43
cg0932952	0.113834	0.555021	3.106296	0.007054	0.224708	-3.51089	0.611939	0.498104	-0.11383	1	1.82E+08	R	II		IGR	opensea	IGR-opense		rs1481664	41;40;25
cg1385683	0.107515	0.613043	3.037187	0.008134	0.224708	-3.65029	0.666801	0.559285	-0.10752	3	1.57E+08	F	II	LINC00886	Body	opensea	Body-opens		rs5392272	1
cg0537474	0.107435	0.660194	2.783379	0.013674	0.224708	-4.1549	0.713911	0.606476	-0.10743	10	13770043	F	II	FRMD4A	Body	opensea	Body-opens		rs6602676	22
cg1681640	0.10743	0.584203	4.052277	0.000996	0.224708	-1.56131	0.637918	0.530488	-0.10743	9	1.38E+08	R	II	FCN1	TSS200	opensea	TSS200-ope		rs2890997	4;1
cg1334271	0.107426	0.634261	3.168879	0.006199	0.224708	-3.38406	0.687974	0.580548	-0.10743	3	1.23E+08	F	II	MYLK	Body	opensea	Body-opens		rs1124707	3
cg1802326	0.107426	0.537631	3.306174	0.004666	0.224708	-3.10407	0.591344	0.483918	-0.10743	11	84403466	R	II	DLG2	Body	opensea	Body-opens		rs5309154	2
cg0200593	0.10741	0.636986	2.596264	0.019954	0.225563	-4.51764	0.690691	0.583281	-0.10741	13	74137411	F	II	LINC00392	TSS1500	opensea	TSS1500-op		rs5739170	18
cg2578870	0.100251	0.617866	3.104266	0.007084	0.224708	-3.515	0.667992	0.567741	-0.10025	17	40225722	F	II		IGR	shore	IGR-shore	chr17:402	rs5584147	1
cg2684911	0.107398	0.531022	3.222884	0.005544	0.224708	-3.27419	0.584721	0.477323	-0.1074	3	1.24E+08	F	II	KALRN	3'UTR	opensea	3'UTR-open		rs1906726	0;1;25
cg2327783	0.107393	0.591475	2.30059	0.035804	0.237939	-5.0696	0.645172	0.537779	-0.10739	1	3704460	F	II	LRRC47	Body	shore	Body-shore	chr1:3703(	rs1428549	0;4;19;36;42;50
cg1799699	0.111709	0.463318	3.10308	0.007101	0.224708	-3.5174	0.519172	0.407464	-0.11171	11	86651974	F	II		IGR	opensea	IGR-opense		rs5448632	1
cg2245283	0.172165	0.399315	3.102453	0.007111	0.224708	-3.51866	0.485397	0.313233	-0.17216	6	28664155	F	I		IGR	opensea	IGR-opense			
cg2531385	0.107371	0.480897	3.415082	0.003723	0.224708	-2.88057	0.534583	0.427211	-0.10737	5	1.07E+08	F	II	EFNA5	Body	opensea	Body-opens		rs7715798	1;37;40
cg1343743	0.107338	0.552638	3.705147	0.00204	0.224708	-2.28135	0.606307	0.498969	-0.10734	3	66354186	R	II	SLC25A26	Body	opensea	Body-opens		rs6224687	51;38;4
cg2352116	0.124635	0.522843	3.100756	0.007135	0.224708	-3.5221	0.58516	0.460525	-0.12463	14	77287324	F	II		IGR	opensea	IGR-opense		rs5457683	1;41
cg1129186	0.110519	0.497881	3.099847	0.007149	0.224708	-3.52393	0.55314	0.442621	-0.11052	2	1.74E+08	R	II		IGR	opensea	IGR-opense		rs7640536	31;28;11;1
cg0600117	0.107336	0.728196	3.435698	0.003567	0.224708	-2.83815	0.781864	0.674527	-0.10734	22	29616276	F	II	EMID1	Body	opensea	Body-opens		rs1465698	0;45
cg0848016	0.107332	0.496533	2.454253	0.026481	0.229518	-4.78638	0.550199	0.442868	-0.10733	15	41036515	F	II	RMDN3	Body	opensea	Body-opens			
cg0998867	0.103157	0.471961	3.098015	0.007176	0.224708	-3.52763	0.523539	0.420382	-0.10316	4	8351738	F	II		IGR	shelf	IGR-shelf	chr4:8347	rs7795991	15;33
cg0777779	0.107331	0.627625	3.394193	0.003888	0.224708	-2.92352	0.68129	0.573959	-0.10733	2	2.33E+08	F	II	ALPPL2	TSS1500	shore	TSS1500-sh	chr2:2332	rs5382976	15;41
cg0959046	0.112226	0.482	3.096215	0.007203	0.224708	-3.53127	0.538112	0.425887	-0.11223	22	39601450	F	II		IGR	opensea	IGR-opense		rs1920335	1;16;45
cg2597035	0.107304	0.474951	3.187199	0.005969	0.224708	-3.34683	0.528604	0.421299	-0.1073	15	59514661	F	II	MYO1E	Body	opensea	Body-opens		rs5762771	35;46
cg0250039	0.107262	0.522524	3.168297	0.006207	0.224708	-3.38524	0.576155	0.468894	-0.10726	6	1.39E+08	F	II	PBOV1	1stExon	opensea	1stExon-op		rs7377468	0;28;46
cg1343199	0.107166	0.678973	3.149583	0.006451	0.224708	-3.42322	0.732556	0.62539	-0.10717	4	76419913	F	II	RCHY1	5'UTR	opensea	5'UTR-open			
cg0545402	0.107157	0.560703	2.642319	0.01819	0.225076	-4.42921	0.614282	0.507125	-0.10716	1	1.53E+08	F	II	S100A12	TSS1500	opensea	TSS1500-op		rs5333639	0;45;48
cg2437633	0.107154	0.60126	3.092968	0.007251	0.224708	-3.53783	0.654837	0.547683	-0.10715	2	1.92E+08	F	II	MYO1B	Body	opensea	Body-opens		rs5363003	0;41
cg1380764	0.10636	0.610419	3.090693	0.007285	0.224708	-3.54243	0.663599	0.55724	-0.10636	20	38785218	R	II		IGR	opensea	IGR-opense		rs1844193	24
cg1974034	0.106258	0.573684	3.08919	0.007308	0.224708	-3.54546	0.626812	0.520555	-0.10626	8	20693391	R	II		IGR	opensea	IGR-opense		rs5566613	17;9
cg2411102	0.10715	0.387356	3.809764	0.001643	0.224708	-2.06442	0.440931	0.33378	-0.10715	6	32819921	R	II	TAP1	Body	shore	Body-shore	chr6:3282(	rs5554933	12;2
cg2651001	0.107143	0.575526	2.886143	0.011089	0.224708	-3.95212	0.629097	0.521954	-0.10714	3	1.87E+08	R	II	EIF4A2	Body	shelf	Body-shelf	chr3:1865(	rs5272957	50;43;14;1
cg2452567	0.100042	0.646028	3.086714	0.007345	0.224708	-3.55046	0.696049	0.596007	-0.10004	6	50576282	F	II		IGR	opensea	IGR-opense		rs1854122	0;11;39;40
cg0113502	0.107138	0.44397	3.308345	0.004645	0.224708	-3.09962	0.497539	0.390401	-0.10714	5	7872175	R	II	MTRR	Body	shelf	Body-shelf	chr5:7868	rs7821590	48;45;27;1
cg1936333	0.107097	0.49677	2.845933	0.012038	0.224708	-4.03173	0.550319	0.443222	-0.1071	8	77897727	F	II	PEX2	5'UTR	opensea	5'UTR-open			
cg1336836	0.107086	0.701557	2.370285	0.031246	0.233736	-4.94219	0.7551	0.648014	-0.10709	5	1.25E+08	F	II	LOC10192	Body	opensea	Body-opens			
cg2548125	0.107084	0.493909	2.873381	0.011382	0.224708	-3.97743	0.54745	0.440367	-0.10708	7	1.42E+08	F	II	TAS2R38	1stExon	opensea	1stExon-op		rs1411968	0;1;13;14
cg1974727	0.107068	0.483746	2.816213	0.01279	0.224708	-4.09036	0.53728	0.430212	-0.10707	6	28553390	R	II	SCAND3	Body	shore	Body-shore	chr6:2855	rs1865280	2
cg1590626	0.107063	0.426691	2.767539	0.014121	0.224708	-4.18594	0.480223	0.37316	-0.10706	3	1.87E+08	F	II	SNORA4	TSS200	shelf	TSS200-she	chr3:1865(	rs2002600	0;1;11;46
cg0361394	0.11637	0.447278	3.078859	0.007465	0.224708	-3.56632	0.505464	0.389093	-0.11637	5	86416413	F	II		IGR	opensea	IGR-opense		rs5349473	43
cg0258409	0.107054	0.537859	2.777101	0.013849	0.224708	-4.16721	0.591386	0.484332	-0.10705	17	6461275	F	II	PITPNM3	TSS1500	shore	TSS1500-sh	chr17:646(	rs1411379	5;8;37;44
cg1569791	0.120971	0.797189	3.077406	0.007487	0.224708	-3.56925	0.857674	0.736703	-0.12097	16	86060428	F	II		IGR	opensea	IGR-opense		rs1138638	0;1
cg2047500	0.10702	0.591649	3.000638	0.008769	0.224708	-3.7237	0.645159	0.538139	-0.10702	7	1.23E+08	R	II	WASL	Body	opensea	Body-opens			
cg1671935	0.106997	0.484461	3.091268	0.007276	0.224708	-3.54127	0.537959	0.430963	-0.107	1	92218200	F	II	TGFBR3	Body	opensea	Body-opens		rs1757348	45
cg2315639	0.106994	0.528819	3.110516	0.006993	0.224708	-3.50236	0.582316	0.475322	-0.10699	3	1.7E+08	F	II	SAMD7	Body	opensea	Body-opens		rs9870311	18;48
cg0384913	0.110896	0.256196	3.074682	0.00753	0.224708	-3.57475	0.311644	0.200748	-0.1109	8	1.04E+08	R	II		IGR	opensea	IGR-opense			
cg2362755	0.116917	0.474134	3.074659	0.00753	0.224708	-3.5748	0.532592	0.415676	-0.11692	2	2.27E+08	F	II		IGR	opensea	IGR-opense		rs5383838	15
cg1095753	0.114867	0.62839	3.074119	0.007538	0.224708	-3.57589	0.685823	0.570956	-0.11487	18	29147253	F	II		IGR	opensea	IGR-opense		rs1481839	1;20
cg0409488	0.106973	0.421465	3.666662	0.002209	0.224708	-2.36108	0.474952	0.367978	-0.10697	17	38023830	F	II	ZPBP2	TSS1500	shore	TSS1500-sh	chr17:380	rs5679148	25

cg0554552	0.102049	0.585542	3.07032	0.007598	0.224708	-3.58355	0.636566	0.534517	-0.10205	1	93283109	R	II		IGR	opensea	IGR-opense		rs5293888	2;1
cg0993090	0.116159	0.610527	3.068985	0.007618	0.224708	-3.58624	0.668607	0.552448	-0.11616	6	1.59E+08	R	II		IGR	opensea	IGR-opense		rs1155769	13;3
cg1102966	0.107362	0.527649	3.068632	0.007624	0.224708	-3.58695	0.58133	0.473968	-0.10736	11	1.32E+08	F	II		IGR	opensea	IGR-opense		rs5676464	0;1;32
cg0267144	0.102486	0.5449	3.068317	0.007629	0.224708	-3.58759	0.596143	0.493657	-0.10249	5	73614960	F	II		IGR	opensea	IGR-opense		rs7765994	40;42;48
cg2734056	0.106971	0.650578	3.984814	0.001144	0.224708	-1.70123	0.704063	0.597092	-0.10697	20	16452834	R	II	KIF16B	Body	opensea	Body-opens		rs1884030	16
cg0602410	0.106956	0.471245	3.000591	0.00877	0.224708	-3.72379	0.524723	0.417767	-0.10696	1	1.54E+08	F	II	SNAPIN	TSS1500	shore	TSS1500-sh	chr1:1536:		
cg2642996	0.106952	0.661158	2.932391	0.010087	0.224708	-3.86015	0.714634	0.607682	-0.10695	15	83867672	F	II	HDGFRP3	Body	opensea	Body-opens		rs5557750	24
cg0335865	0.106944	0.480351	3.002288	0.008739	0.224708	-3.72039	0.533823	0.426879	-0.10694	2	1.69E+08	F	II	LOC10561(	Body	opensea	Body-opens		rs1132007	18
cg0999221	0.114784	0.429052	3.065703	0.00767	0.224708	-3.59286	0.486445	0.37166	-0.11478	11	32353565	R	II		IGR	shore	IGR-shore	chr11:3235		
cg1691705	0.101627	0.552427	3.065331	0.007676	0.224708	-3.59361	0.603241	0.501614	-0.10163	2	59690787	R	II		IGR	opensea	IGR-opense		rs5651533	43;29
cg1393180	0.105214	0.479102	3.065305	0.007676	0.224708	-3.59366	0.531709	0.426495	-0.10521	12	71983445	F	II		IGR	opensea	IGR-opense		rs1180832	0
cg0575476	0.10691	0.673845	3.575858	0.002667	0.224708	-2.54898	0.727301	0.62039	-0.10691	22	18307227	R	II	MICAL3	Body	opensea	Body-opens		rs5314756	39
cg0311391	0.106881	0.737299	3.581101	0.002638	0.224708	-2.53814	0.790739	0.683858	-0.10688	22	18402483	F	II	MICAL3	5'UTR	opensea	5'UTR-open		rs5745514	0
cg0631187	0.103961	0.67176	3.062883	0.007715	0.224708	-3.59854	0.72374	0.619779	-0.10396	12	93427724	F	II		IGR	opensea	IGR-opense		rs1502121	12
cg0249336	0.106876	0.386826	2.903962	0.010692	0.224708	-3.91673	0.440263	0.333388	-0.10688	19	36204278	R	II	ZBTB32	5'UTR	shelf	5'UTR-shelf	chr19:362(	rs5393570	2
cg0205215	0.106873	0.479682	2.82044	0.01268	0.224708	-4.08203	0.533118	0.426246	-0.10687	8	26211126	F	II	PPP2R2A	Body	opensea	Body-opens			
cg0139778	0.132717	0.495072	3.061508	0.007737	0.224708	-3.60132	0.56143	0.428713	-0.13272	10	3894712	R	II		IGR	opensea	IGR-opense		rs5388411	1
cg1016737	0.157555	0.572041	3.061304	0.00774	0.224708	-3.60173	0.650818	0.493263	-0.15756	1	2.29E+08	R	I		IGR	island	IGR-island	chr1:2287	rs5386267	26;7;6;1
cg1363601	0.10019	0.495332	3.061115	0.007743	0.224708	-3.60211	0.545427	0.445237	-0.10019	9	84004731	F	II		IGR	opensea	IGR-opense		rs1113919	31;36;46;50
cg0674600	0.106871	0.794342	2.188565	0.044454	0.248276	-5.27045	0.847777	0.740906	-0.10687	8	1.31E+08	R	II	ASAP1	Body	opensea	Body-opens		rs5671878	18;3;1
cg0163006	0.106847	0.465425	2.946781	0.009794	0.224708	-3.83145	0.518848	0.412002	-0.10685	8	1.02E+08	R	II	SNX31	Body	opensea	Body-opens		rs7929575	41;13;10
cg0064031	0.106846	0.523285	3.052863	0.007876	0.224708	-3.61873	0.576708	0.469862	-0.10685	8	67835853	R	II	SNORD87	TSS1500	shore	TSS1500-sh	chr8:6783		
cg2479654	0.106842	0.338459	3.723053	0.001966	0.224708	-2.24424	0.39188	0.285038	-0.10684	10	1.09E+08	F	II	SORCS1	Body	opensea	Body-opens		rs1477672	36
cg2563243	0.106828	0.783976	5.278587	8.55E-05	0.223139	0.936345	0.83739	0.730562	-0.10683	11	18764080	R	II	PTPN5	Body	opensea	Body-opens		rs2007139	38;20;2;1
cg1775909	0.106826	0.532053	2.714992	0.015708	0.224896	-4.28851	0.585466	0.47864	-0.10683	6	56755566	R	II	DST	Body	opensea	Body-opens		rs7345970	32;21;1
cg1290623	0.106817	0.745084	2.374178	0.031008	0.233467	-4.93502	0.798493	0.691675	-0.10682	10	50122576	F	II	LRRC18	TSS1500	opensea	TSS1500-op		rs5538847	15;20;33
cg1342383	0.113817	0.456448	3.057468	0.007801	0.224708	-3.60946	0.513356	0.399539	-0.11382	10	4818881	F	II		IGR	opensea	IGR-opense		rs1408021	31
cg0535515	0.106795	0.707569	2.658443	0.017609	0.225076	-4.39811	0.760967	0.654172	-0.1068	1	2.07E+08	R	II	C4BPB	Body	opensea	Body-opens		rs1501273	49;1
cg2109709	0.106795	0.501656	3.1784	0.006078	0.224708	-3.36471	0.555053	0.448259	-0.10679	5	1.19E+08	R	II	TNFAIP8	Body	shelf	Body-shelf	chr5:1186	rs1896012	48;46;2
cg2143413	0.162663	0.392687	3.05688	0.007811	0.224708	-3.61064	0.474019	0.311356	-0.16266	3	1.97E+08	R	II		IGR	opensea	IGR-opense		rs1143720	46;11
cg0943944	0.10677	0.536463	3.221262	0.005563	0.224708	-3.27749	0.589848	0.483077	-0.10677	3	1.75E+08	R	II	NAALADL2	Body	opensea	Body-opens		rs5398480	51
cg0377090	0.106752	0.599828	3.19644	0.005856	0.224708	-3.32803	0.653204	0.546452	-0.10675	19	56443824	R	II	NLRP13	TSS200	opensea	TSS200-ope		rs2017260	34
cg2400540	0.101396	0.59982	3.056121	0.007823	0.224708	-3.61217	0.650518	0.549122	-0.1014	7	23060529	F	II		IGR	opensea	IGR-opense		rs1879437	1;17;49
cg1367173	0.116976	0.309006	3.056035	0.007824	0.224708	-3.61234	0.367494	0.250518	-0.11698	17	18836108	F	II		IGR	opensea	IGR-opense		rs1886823	14
cg2305344	0.109242	0.493549	3.055784	0.007828	0.224708	-3.61285	0.54817	0.438928	-0.10924	14	78471899	R	II		IGR	opensea	IGR-opense		rs1462769	40;28;27
cg2068419	0.106739	0.468421	2.955578	0.009619	0.224708	-3.81389	0.52179	0.415052	-0.10674	5	1.42E+08	F	II	FGF1	5'UTR	opensea	5'UTR-open		rs1880151	47;51
cg0707176	0.106693	0.725594	3.048783	0.007942	0.224708	-3.62695	0.77894	0.672247	-0.10669	11	92441315	F	II	FAT3	Body	opensea	Body-opens		rs5369830	1;33
cg0161364	0.106691	0.505906	2.846592	0.012022	0.224708	-4.03043	0.559251	0.45256	-0.10669	10	28271425	R	II	ARMC4	TSS1500	opensea	TSS1500-op		rs5310922	51;23;1
cg2111359	0.106671	0.530508	2.766897	0.014139	0.224708	-4.1872	0.583843	0.477172	-0.10667	14	31858067	R	II	HEATR5A	1stExon	opensea	1stExon-op		rs3887865	44;23;2
cg0445364	0.10667	0.542103	2.501638	0.024105	0.227447	-4.69741	0.595438	0.488768	-0.10667	2	40579303	F	II	SLC8A1	Body	opensea	Body-opens		rs1858235	13;17;21
cg0922131	0.106656	0.684735	2.665794	0.01735	0.225046	-4.38391	0.738063	0.631407	-0.10666	6	1.17E+08	R	II	TRAPPC3L	Body	opensea	Body-opens		rs5706721	34;7
cg1040863	0.106655	0.676223	3.321541	0.00452	0.224708	-3.0726	0.72955	0.622895	-0.10666	2	96869389	R	II	STARD7	Body	opensea	Body-opens		rs5636720	3;9;27;37;38
cg1179959	0.142737	0.459756	3.049964	0.007923	0.224708	-3.62457	0.531124	0.388387	-0.14274	12	68845869	F	II		IGR	opensea	IGR-opense		rs1903222	0
cg0675449	0.106627	0.473702	3.058368	0.007787	0.224708	-3.60764	0.527015	0.420389	-0.10663	10	52268375	R	II	SGMS1	5'UTR	opensea	5'UTR-open		rs5400935	39;22
cg0450792	0.106626	0.483688	2.785847	0.013605	0.224708	-4.15005	0.537001	0.430375	-0.10663	2	2.11E+08	R	II	UNC80	Body	opensea	Body-opens		rs5480498	20;2
cg0719208	0.102251	0.491654	3.047152	0.007969	0.224708	-3.63023	0.542779	0.440528	-0.10225	14	75981936	F	II		IGR	opensea	IGR-opense			
cg2369118	0.106613	0.493056	3.436316	0.003562	0.224708	-2.83688	0.546362	0.439749	-0.10661	22	29305360	R	II	ZNRF3	Body	opensea	Body-opens		rs5364290	1
cg0679435	0.113351	0.631459	3.044386	0.008014	0.224708	-3.6358	0.688134	0.574783	-0.11335	1	17014799	R	II		IGR	shelf	IGR-shelf	chr1:1701	rs5584377	2;1
cg1796966	0.105882	0.586759	3.043916	0.008022	0.224708	-3.63675	0.6397	0.533818	-0.10588	1	2.21E+08	R	II		IGR	opensea	IGR-opense		rs5754031	26;7;6
cg0274569	0.109559	0.581016	3.043656	0.008026	0.224708	-3.63727	0.635796	0.526237	-0.10956	9	93822926	R	II		IGR	opensea	IGR-opense			
cg2217613	0.106108	0.563716	3.043384	0.008031	0.224708	-3.63782	0.61677	0.510662	-0.10611	10	33442301	F	II		IGR	opensea	IGR-opense		rs1864286	23;39
cg0938537	0.106612	0.55727	3.383132	0.003978	0.224708	-2.94625	0.610576	0.503964	-0.10661	6	1.43E+08	R	II	LOC15391(	TSS1500	opensea	TSS1500-op		rs5433593	2
cg1671060	0.106089	0.552969	3.042631	0.008043	0.224708	-3.63933	0.606014	0.499925	-0.10609	15	33424752	R	II		IGR	opensea	IGR-opense		rs5397189	30
cg0857159	0.102588	0.506568	3.041843	0.008056	0.224708	-3.64092	0.557861	0.455274	-0.10259	6	64225663	F	II		IGR	opensea	IGR-opense			
cg0252498	0.106594	0.676022	3.066519	0.007657	0.224708	-3.59122	0.729319	0.622725	-0.10659	3	1.88E+08	F	I	LPP	5'UTR	opensea	5'UTR-open		rs5550028	0;20;37
cg1088122	0.106556	0.441953	2.367832	0.031397	0.233867	-4.9467	0.495231	0.388675	-0.10656	2	9984929	R	II	TAF1B	Body	shore	Body-shore	chr2:9983	rs1856685	51;30;14
cg0071382	0.106532	0.798168	2.980379	0.009142	0.224708	-3.76429	0.851434	0.744902	-0.10653	12	7633946	R	II	CD163	Body	opensea	Body-opens		rs7557616	1
cg1685118	0.103346	0.603054	3.041419	0.008063	0.224708	-3.64177	0.654727	0.551381	-0.10335	22	35415024	R	II		IGR	opensea	IGR-opense		rs916243	31
cg0248733	0.278444	0.413133	3.041418	0.008063	0.224708	-3.64178	0.552355	0.27391	-0.27844	1	1.47E+08	F	II		IGR	island	IGR-island	chr1:1465	rs5877106	16
cg0441955	0.106483	0.399634	2.708604	0.015913	0.224896	-4.30093	0.452875	0.346392	-0.10648	6	1.11E+08	R	II	RPF2	TSS1500	shore	TSS1500-sh	chr6:1113(	rs3431735	11
cg1671858	0.106416	0.563266	2.86441	0.011593	0.224708	-3.99519	0.616474	0.510057	-0.10642	12	1.33E+08	R	II	ANKLE2	Body	shore	Body-shore	chr12:133	rs5777973	50;16
cg0116279	0.106337	0.592858	3.081582	0.007423	0.224708	-3.56083	0.646026	0.53969	-0.10634	1	64293256	R	II	ROR1	Body	opensea	Body-opens		rs5512446	27
cg0205002	0.106313	0.565105	3.380614	0.003999	0.224708	-2.95142	0.618262	0.511948	-0.10631	7	43156037	R	II	HECW1	5'UTR	shelf	5'UTR-shelf	chr7:4315	rs5330254	34
cg1557429	0.108837	0.499261	3.038866	0.008106	0.224708	-3.64691	0.55368	0.444842	-0.10884	7	25590861	F	II		IGR	opensea	IGR-opense		rs1778270	29;47

cg2102646	0.10628	0.580677	3.537167	0.00289	0.224708	-2.62893	0.633817	0.527537	-0.10628	3	13539059	F	II	HDAC11	Body	opensea	Body-opens		rs3683436	8
cg1660282	0.101817	0.659384	3.037895	0.008122	0.224708	-3.64886	0.710293	0.608475	-0.10182	17	72081361	F	II		IGR	opensea	IGR-opense		rs1142388	1;9;29;41;51
cg0186130	0.106276	0.528354	2.959095	0.00955	0.224708	-3.80686	0.581492	0.475216	-0.10628	7	77827744	R	II	MAGI2	Body	opensea	Body-opens		rs5735621	28;21;7
cg1356445	0.106275	0.314215	4.056051	0.000988	0.224708	-1.55349	0.367352	0.261077	-0.10628	14	62004353	R	II	PRKCH	Body	opensea	Body-opens		rs5698333	1
cg1066239	0.109079	0.573886	3.036234	0.00815	0.224708	-3.6522	0.628425	0.519346	-0.10908	1	24046776	R	II		IGR	opensea	IGR-opense		rs1150834	14;2
cg1593690	0.105047	0.581252	3.03604	0.008153	0.224708	-3.6526	0.633776	0.528728	-0.10505	17	30024653	F	II		IGR	opensea	IGR-opense		rs1413264	31
cg2321664	0.106253	0.643071	2.915805	0.010436	0.224708	-3.89318	0.696197	0.589944	-0.10625	7	1.42E+08	R	II	MGAM	ExonBnd	opensea	ExonBnd-o			
cg2301470	0.103771	0.488997	3.034915	0.008172	0.224708	-3.65486	0.540882	0.437111	-0.10377	5	1.69E+08	F	II		IGR	opensea	IGR-opense			
cg1545269	0.127842	0.605279	3.034511	0.008179	0.224708	-3.65567	0.669201	0.541358	-0.12784	3	1.02E+08	R	II		IGR	opensea	IGR-opense		rs5704379	51;47;46;22;11
cg0366507	0.106248	0.469924	3.79061	0.001709	0.224708	-2.10416	0.523048	0.416799	-0.10625	5	1.19E+08	F	II	TNFAIP8	Body	shore	Body-shore	chr5:1186	rs5655353	26
cg2440171	0.106225	0.520647	3.038052	0.00812	0.224708	-3.64855	0.573759	0.467534	-0.10622	9	71532723	F	II	PIP5K1B	Body	opensea	Body-opens		rs1398280	1;16;31
cg2504587	0.104094	0.607681	3.031237	0.008234	0.224708	-3.66225	0.659728	0.555634	-0.10409	21	18188931	F	II		IGR	opensea	IGR-opense		rs5672681	4;7;43;45
cg1629503	0.106197	0.648407	2.54486	0.022115	0.226059	-4.61563	0.701505	0.595308	-0.1062	5	94784489	F	II	FAM81B	Body	opensea	Body-opens		rs5710572	12;50
cg1049125	0.107895	0.718349	3.029903	0.008257	0.224708	-3.66494	0.772296	0.664402	-0.10789	11	45699694	F	II		IGR	opensea	IGR-opense		rs1167588	0;38
cg0185850	0.353196	0.353056	3.029349	0.008266	0.224708	-3.66605	0.529654	0.176457	-0.3532	17	68202566	F	II		IGR	opensea	IGR-opense		rs1144290	1;20
cg0840856	0.133348	0.52558	3.02885	0.008275	0.224708	-3.66705	0.592255	0.458906	-0.13335	5	67067213	R	II		IGR	opensea	IGR-opense		rs5292816	45;2
cg1418413	0.100007	0.476624	3.026491	0.008315	0.224708	-3.6718	0.526628	0.426621	-0.10001	1	34966511	F	II		IGR	opensea	IGR-opense		rs5538281	0;14;27
cg2614402	0.106182	0.521944	2.659701	0.017564	0.225076	-4.39568	0.575035	0.468853	-0.10618	3	69047209	F	II	EOGT	Body	opensea	Body-opens		rs1473270	1;30
cg0202077	0.106164	0.525045	3.665079	0.002216	0.224708	-2.36436	0.578127	0.471963	-0.10616	5	1.39E+08	R	II	UBE2D2	5'UTR	opensea	5'UTR-open		rs5492604	1
cg2071294	0.106135	0.587911	2.922802	0.010288	0.224708	-3.87925	0.640979	0.534843	-0.10614	1	39888404	R	II	MACF1	ExonBnd	opensea	ExonBnd-o		rs2008493	42;39;38
cg1988188	0.101974	0.588361	3.024876	0.008343	0.224708	-3.67504	0.639348	0.537374	-0.10197	7	12362656	F	II		IGR	opensea	IGR-opense		rs5761410	6;7;12;19;31
cg1703169	0.10613	0.397367	3.001498	0.008754	0.224708	-3.72197	0.450432	0.344302	-0.10613	12	94602306	F	II	PLXNC1	Body	opensea	Body-opens		rs1925419	0;1;10;27
cg2640154	0.124016	0.541343	3.023609	0.008364	0.224708	-3.67759	0.603351	0.479335	-0.12402	6	91078974	F	II		IGR	opensea	IGR-opense		rs5483666	42;47
cg2119404	0.106129	0.47417	2.947929	0.009771	0.224708	-3.82916	0.527234	0.421105	-0.10613	3	11862088	R	II	TAMM41	Body	opensea	Body-opens			
cg1768394	0.106093	0.491899	3.084877	0.007374	0.224708	-3.55438	0.544946	0.438852	-0.10609	15	65763347	F	II	DPP8	Body	opensea	Body-opens		rs5557354	18;20;48
cg1685386	0.106092	0.340734	3.087314	0.007336	0.224708	-3.54925	0.39378	0.287688	-0.10609	6	32823116	R	II	PSMB9	Body	shore	Body-shore	chr6:3282		
cg0411210	0.106084	0.531166	2.666853	0.017313	0.225034	-4.38187	0.584208	0.478124	-0.10608	2	1.44E+08	R	II	KYNU	Body	opensea	Body-opens			
cg1253170	0.106078	0.56275	2.684061	0.016722	0.224901	-4.34856	0.615789	0.509711	-0.10608	5	33834545	F	II	ADAMTS12	Body	opensea	Body-opens		rs1415835	0;1;3
cg2073684	0.106053	0.4796	2.913207	0.010492	0.224708	-3.89835	0.532626	0.426573	-0.10605	2	73902031	F	II	ALMS1P	ExonBnd	opensea	ExonBnd-o		rs2010199	0;1
cg1990510	0.106045	0.606333	3.177057	0.006095	0.224708	-3.36744	0.659355	0.55331	-0.10604	20	57892254	F	II	EDN3	Body	opensea	Body-opens		rs5458164	0;9;28;46
cg0335173	0.100295	0.463985	3.016607	0.008486	0.224708	-3.69165	0.514133	0.413837	-0.1003	18	21717196	F	II		IGR	shore	IGR-shore	chr18:217	rs7949387	24
cg2373297	0.106044	0.625823	5.420183	6.51E-05	0.219272	1.215768	0.678845	0.572801	-0.10604	20	57414274	F	II	GNASAS	Body	shore	Body-shore	chr20:574	rs3761263	6;48
cg0715242	0.106037	0.479866	3.570062	0.002699	0.224708	-2.56096	0.532884	0.426848	-0.10604	2	33713297	R	II	RASGRP3	5'UTR	opensea	5'UTR-open		rs5339412	6;2;1
cg0832111	0.106007	0.507053	2.766569	0.014149	0.224708	-4.18784	0.560057	0.45405	-0.10601	5	77701852	F	II	SCAMP1	Body	opensea	Body-opens		rs1824511	0
cg1178633	0.106001	0.515915	3.13518	0.006646	0.224708	-3.45242	0.568915	0.462915	-0.106	18	56809717	F	II	SEC11C	Body	shelf	Body-shelf	chr18:568	rs1260467	26;35
cg1228090	0.106	0.602667	4.637374	0.000303	0.224708	-0.3553	0.655667	0.549667	-0.106	6	28501816	R	II	GPX5	Body	opensea	Body-opens		rs1379079	49;40
cg2656269	0.105997	0.476517	3.193958	0.005886	0.224708	-3.33308	0.529515	0.423518	-0.106	16	23850404	F	II	PRKCB	Body	shelf	Body-shelf	chr16:238	rs1930964	0
cg0893862	0.105987	0.509122	3.347187	0.004286	0.224708	-3.02003	0.562116	0.456128	-0.10599	18	42832579	R	II	SLC14A2	5'UTR	opensea	5'UTR-open			
cg2155418	0.105987	0.534937	2.839306	0.012202	0.224708	-4.04482	0.58793	0.481943	-0.10599	1	1.16E+08	R	II	CASQ2	Body	opensea	Body-opens			
cg1736601	0.105943	0.586411	4.230241	0.000691	0.224708	-1.19276	0.639382	0.533439	-0.10594	10	1.25E+08	F	II	IKZF5	Body	opensea	Body-opens		rs5416002	20;38
cg1023666	0.105932	0.580872	3.104594	0.007079	0.224708	-3.51433	0.633838	0.527907	-0.10593	3	1.88E+08	R	II	LPP	Body	opensea	Body-opens		rs5615344	34;2
cg2214428	0.106399	0.673411	3.008983	0.00862	0.224708	-3.70696	0.72661	0.620211	-0.1064	5	1.69E+08	R	II		IGR	opensea	IGR-opense		rs1904611	38;32;6;1
cg2070796	0.10592	0.508016	3.142118	0.006552	0.224708	-3.43836	0.560975	0.455056	-0.10592	12	1.31E+08	R	II	ADGRD1	Body	opensea	Body-opens		rs2021903	6;4
cg2179900	0.1059	0.686078	3.128677	0.006736	0.224708	-3.4656	0.739028	0.633128	-0.1059	1	65223175	F	II	RAVER2	Body	opensea	Body-opens			
cg0073265	0.105874	0.474678	4.922221	0.000172	0.224708	0.223389	0.527615	0.421741	-0.10587	2	1.01E+08	R	II	NMS	Body	opensea	Body-opens		rs1888129	51;1
cg1370643	0.105858	0.69235	2.786832	0.013578	0.224708	-4.14812	0.745279	0.639422	-0.10586	9	17612104	R	II	SH3GL2	Body	opensea	Body-opens		rs1430275	27;15
cg0556714	0.108066	0.702659	3.007635	0.008644	0.224708	-3.70966	0.756692	0.648626	-0.10807	22	50980910	F	II		IGR	shore	IGR-shore	chr22:509	rs1921307	0;7;15
cg1269626	0.105854	0.507999	2.958067	0.00957	0.224708	-3.80891	0.560926	0.455072	-0.10585	14	67446489	R	II	GPHN	Body	opensea	Body-opens		rs5423226	32;1
cg0163323	0.105848	0.504298	3.493862	0.003162	0.224708	-2.7183	0.557222	0.451374	-0.10585	2	2.17E+08	R	II	XRCC5	Body	opensea	Body-opens		rs5779289	12
cg0657207	0.105811	0.693368	2.459808	0.026191	0.229328	-4.77598	0.746274	0.640463	-0.10581	12	32487307	R	II	BICD1	Body	opensea	Body-opens			
cg1769413	0.116439	0.44639	3.00665	0.008661	0.224708	-3.71164	0.50461	0.388171	-0.11644	4	14858290	F	I		IGR	opensea	IGR-opense		rs3679657	36;41;49;51
cg0585460	0.105796	0.593139	2.854291	0.011835	0.224708	-4.01521	0.646037	0.540241	-0.1058	2	40351367	R	II	SLC8A1	Body	opensea	Body-opens		rs5483373	23;15;5;4;2;1
cg0648577	0.105781	0.549637	2.731464	0.015193	0.224792	-4.25643	0.602527	0.496746	-0.10578	1	1.85E+08	R	II	FAM129A	Body	opensea	Body-opens		rs1408609	16;15;10;9
cg1090000	0.105557	0.433242	3.00601	0.008673	0.224708	-3.71292	0.48602	0.380464	-0.10556	17	65243288	F	II		IGR	shore	IGR-shore	chr17:652		
cg1615890	0.126464	0.536507	3.005661	0.008679	0.224708	-3.71362	0.599739	0.473275	-0.12646	7	1.14E+08	R	II		IGR	opensea	IGR-opense		rs1416447	24;2
cg1258676	0.105742	0.69026	2.913365	0.010488	0.224708	-3.89804	0.743131	0.637389	-0.10574	15	60868447	R	II	RORA-AS1	Body	opensea	Body-opens		rs1385276	36;1
cg1958505	0.110222	0.500648	3.003663	0.008715	0.224708	-3.71763	0.555759	0.445537	-0.11022	7	85103687	R	II		IGR	opensea	IGR-opense		rs2091086	18;1
cg2083920	0.105737	0.630866	3.532976	0.002915	0.224708	-2.63758	0.683735	0.577998	-0.10574	2	86007629	R	II	ATOH8	Body	opensea	Body-opens		rs1382318	43;25;9;1
cg0539294	0.107458	0.558293	3.002678	0.008732	0.224708	-3.71961	0.612022	0.504564	-0.10746	13	77091333	R	II		IGR	opensea	IGR-opense		rs1905198	46;44;38;2
cg1713862	0.105734	0.696094	3.738612	0.001903	0.224708	-2.21198	0.748962	0.643227	-0.10573	7	22184820	F	II	RAPGEF5	ExonBnd	opensea	ExonBnd-o		rs3691574	15;46
cg0082960	0.102329	0.60815	3.002487	0.008736	0.224708	-3.71999	0.659314	0.556986	-0.10233	16	84678923	F	II		IGR	shelf	IGR-shelf	chr16:846	rs5322773	9;15;26
cg2414540	0.105714	0.528506	2.891663	0.010965	0.224708	-3.94117	0.581363	0.475649	-0.10571	1	1.59E+08	R	II	AIM2	TSS1500	opensea	TSS1500-o		rs5510582	24;17;8;1
cg2158004	0.10571	0.420378	3.127742	0.006749	0.224708	-3.46749	0.473233	0.367523	-0.10571	17	17929070	F	II	ATPAF2	Body	opensea	Body-opens		rs1134836	36
cg0298994	0.105702	0.626216	6.260368	1.37E-05	0.161665	2.822742	0.679067	0.573365	-0.1057	16	31539234	F	II	AHSP	5'UTR	opensea	5'UTR-open		rs5426024	2;5

cg2266603	0.105682	0.528617	2.810855	0.01293	0.224708	-4.10091	0.581458	0.475776	-0.10568	1	1.5E+08	F	II	VPS45	Body	opensea	Body-opens		rs1419694	0;27
cg0458915	0.107929	0.565908	3.000579	0.00877	0.224708	-3.72382	0.619873	0.511944	-0.10793	6	1.48E+08	F	II		IGR	opensea	IGR-opense		rs5291172	12;48
cg2299401	0.105678	0.605155	3.279625	0.00493	0.224708	-3.15838	0.657994	0.552317	-0.10568	11	10044327	F	II	SBF2	Body	opensea	Body-opens		rs5703060	0;1
cg1274612	0.105672	0.417771	2.864278	0.011596	0.224708	-3.99545	0.470608	0.364935	-0.10567	7	1.29E+08	R	II	SMO	Body	opensea	Body-opens		rs1157402	1
cg2017199	0.101293	0.510134	2.997217	0.008831	0.224708	-3.73056	0.560781	0.459488	-0.10129	8	67343066	R	II		IGR	shore	IGR-shore	chr8:6734	rs3524340	27;22
cg0753579	0.105663	0.50993	2.771304	0.014013	0.224708	-4.17857	0.562762	0.457099	-0.10566	3	1.15E+08	R	II	ZBTB20	Body	opensea	Body-opens		rs1060854	3
cg1181266	0.105658	0.673156	2.194086	0.043986	0.24764	-5.26067	0.725985	0.620327	-0.10566	1	62607877	R	II	INADL	Body	opensea	Body-opens		rs7938521	39;29;20;8;1
cg1073478	0.108507	0.481175	2.994172	0.008886	0.224708	-3.73666	0.535428	0.426921	-0.10851	8	19833012	F	II		IGR	opensea	IGR-opense		rs5616270	0;14;49
cg1623627	0.105654	0.438692	3.106795	0.007047	0.224708	-3.50988	0.49152	0.385865	-0.10565	6	87891939	F	II	ZNF292	Body	opensea	Body-opens		rs5755990	10;21;22
cg2434887	0.104154	0.380667	2.992591	0.008915	0.224708	-3.73983	0.432745	0.32859	-0.10415	6	1.49E+08	F	II		IGR	opensea	IGR-opense		rs1485131	0;1
cg1457513	0.105652	0.559444	3.552325	0.0028	0.224708	-2.59762	0.61227	0.506618	-0.10565	22	31933466	R	II	SFI1	Body	opensea	Body-opens		rs5365868	18
cg1335547	0.105601	0.482394	3.107323	0.007039	0.224708	-3.50882	0.535195	0.429594	-0.1056	6	17431988	R	II	CAP2	Body	opensea	Body-opens		rs1884508	34;2
cg2349849	0.100758	0.572292	2.990599	0.008952	0.224708	-3.74382	0.62267	0.521913	-0.10076	7	90095672	F	II		IGR	opensea	IGR-opense			
cg1913837	0.116503	0.449038	2.990236	0.008958	0.224708	-3.74455	0.50729	0.390787	-0.1165	2	1.49E+08	R	II		IGR	opensea	IGR-opense		rs5435285	7
cg0117780	0.1056	0.475679	3.080408	0.007441	0.224708	-3.5632	0.528479	0.422878	-0.1056	17	39042031	F	II	KRT20	TSS1500	opensea	TSS1500-op		rs1397193	32;41
cg2160397	0.105573	0.427513	4.529099	0.000377	0.224708	-0.577	0.480299	0.374727	-0.10557	1	71534275	F	II	MIR186	TSS1500	opensea	TSS1500-op		rs5699794	28;32;34
cg0864427	0.101385	0.484359	2.987438	0.00901	0.224708	-3.75016	0.535051	0.433666	-0.10139	2	2.27E+08	R	II		IGR	opensea	IGR-opense		rs5466826	30;25;1
cg0929206	0.105783	0.632071	2.987176	0.009015	0.224708	-3.75068	0.684963	0.57918	-0.10578	4	77801348	F	II		IGR	opensea	IGR-opense		rs1860110	0
cg0047894	0.126139	0.580745	2.987049	0.009017	0.224708	-3.75093	0.643815	0.517676	-0.12614	2	75854312	R	II		IGR	opensea	IGR-opense		rs5605754	8
cg1488599	0.105528	0.417535	2.915526	0.010442	0.224708	-3.89374	0.4703	0.364771	-0.10553	17	59828209	F	II	BRIP1	Body	opensea	Body-opens			
cg2092004	0.111158	0.613107	2.986327	0.009031	0.224708	-3.75238	0.668685	0.557528	-0.11116	2	41822300	F	II		IGR	opensea	IGR-opense		rs5449718	2;33;39;40
cg1768146	0.101279	0.693929	2.984769	0.00906	0.224708	-3.7555	0.744568	0.643289	-0.10128	4	10131207	R	II		IGR	opensea	IGR-opense		rs6228660	48;44;43;40;16;8
cg2456342	0.108605	0.600407	2.984064	0.009073	0.224708	-3.75691	0.654709	0.546104	-0.1086	6	1.38E+08	R	II		IGR	opensea	IGR-opense		rs5541432	1
cg2068996	0.105522	0.464838	2.409716	0.028915	0.231543	-4.86932	0.517599	0.412077	-0.10552	1	28390766	R	II	EYA3	Body	opensea	Body-opens		rs1430206	36;1
cg1701692	0.107611	0.676166	2.982305	0.009106	0.224708	-3.76044	0.729971	0.622361	-0.10761	5	1.41E+08	R	II		IGR	opensea	IGR-opense		rs5467619	5;1
cg1471321	0.103289	0.585916	2.981929	0.009113	0.224708	-3.76119	0.63756	0.534271	-0.10329	1	33924313	R	II		IGR	opensea	IGR-opense		rs5772192	10;2
cg0413794	0.10552	0.525075	2.709743	0.015876	0.224896	-4.29872	0.577835	0.472316	-0.10552	4	1.64E+08	R	II	NAF1	Body	shore	Body-shore	chr4:1640	rs5424135	46;36
cg0196633	0.105518	0.497771	2.96626	0.009411	0.224708	-3.79254	0.55053	0.445012	-0.10552	2	1.28E+08	F	II	MYO7B	Body	shelf	Body-shelf	chr2:1283	rs5305738	0;3;25
cg1042498	0.111096	0.621255	2.980493	0.00914	0.224708	-3.76406	0.676804	0.565707	-0.1111	1	29872399	R	II		IGR	opensea	IGR-opense		rs5611094	26;2;1
cg2169421	0.105518	0.588673	3.547801	0.002827	0.224708	-2.60696	0.641432	0.535914	-0.10552	8	48565455	F	II	SPIDR	Body	opensea	Body-opens		rs5326918	6;8
cg2157979	0.10546	0.650252	3.275919	0.004968	0.224708	-3.16595	0.702981	0.597522	-0.10546	8	18577100	R	II	PSD3	Body	opensea	Body-opens		rs7768848	39;2
cg1249390	0.105416	0.735551	3.211571	0.005676	0.224708	-3.29723	0.788259	0.682843	-0.10542	22	18342351	F	II	MICAL3	Body	opensea	Body-opens		rs5606308	32
cg2520695	0.100779	0.55183	2.979348	0.009161	0.224708	-3.76636	0.60222	0.501441	-0.10078	21	40219706	R	II		IGR	opensea	IGR-opense		rs8012854	1
cg0676391	0.103894	0.402413	2.979151	0.009165	0.224708	-3.76675	0.45436	0.350466	-0.10389	16	74260395	F	II		IGR	opensea	IGR-opense		rs5609067	4
cg0408314	0.105399	0.482061	3.111628	0.006977	0.224708	-3.50011	0.53476	0.429362	-0.1054	17	64028625	R	II	CEP112	Body	opensea	Body-opens		rs5435131	42;2
cg0416316	0.105364	0.425208	2.650183	0.017904	0.225076	-4.41405	0.47789	0.372526	-0.10536	21	26768888	F	II	LINC00158	Body	opensea	Body-opens		rs5444963	1;13
cg2338199	0.120865	0.50223	2.97714	0.009203	0.224708	-3.77077	0.562663	0.441797	-0.12087	1	88867081	R	II		IGR	opensea	IGR-opense		rs5368460	22
cg2228165	0.105364	0.642729	3.755608	0.001837	0.224708	-2.17674	0.695411	0.590048	-0.10536	17	62197507	F	II	ERN1	Body	opensea	Body-opens		rs1513344	0;34
cg1707316	0.109611	0.498748	2.976722	0.009211	0.224708	-3.77161	0.553553	0.443942	-0.10961	3	1.11E+08	F	II		IGR	opensea	IGR-opense		rs5601347	29;37
cg0912267	0.102675	0.472261	2.976292	0.009219	0.224708	-3.77247	0.523599	0.420923	-0.10268	2	46897833	F	II		IGR	opensea	IGR-opense		rs1883472	9;24;37;38
cg2719373	0.105352	0.501151	3.052781	0.007877	0.224708	-3.6189	0.553827	0.448475	-0.10535	7	90562815	F	II	CDK14	Body	opensea	Body-opens		rs5416637	1;39
cg1804540	0.105332	0.73324	2.536463	0.022489	0.226384	-4.63156	0.785906	0.680574	-0.10533	10	11564762	R	II	USP6NL	Body	opensea	Body-opens		rs2005087	32
cg2462877	0.101652	0.454051	2.974274	0.009257	0.224708	-3.77651	0.504877	0.403225	-0.10165	8	1.3E+08	F	II		IGR	opensea	IGR-opense		rs1448584	11;20;25;35
cg1585747	0.105309	0.556178	3.108848	0.007017	0.224708	-3.50573	0.608833	0.503523	-0.10531	7	1.05E+08	R	II	SRPK2	Body	shelf	Body-shelf	chr7:1049	rs5709144	26;25;24
cg2099411	0.105278	0.470403	3.500884	0.003116	0.224708	-2.70382	0.523043	0.417764	-0.10528	1	2.1E+08	R	II	CAMK1G	Body	opensea	Body-opens		rs1824074	23;1
cg2354861	0.105278	0.555165	3.116525	0.006907	0.224708	-3.4902	0.607804	0.502526	-0.10528	7	1.48E+08	R	II	CNTNAP2	Body	opensea	Body-opens		rs5618143	30
cg0092963	0.109262	0.533492	2.971958	0.009301	0.224708	-3.78114	0.588123	0.478861	-0.10926	1	1379667	R	II		IGR	opensea	IGR-opense		rs1386918	37
cg0908662	0.101287	0.650106	2.970439	0.00933	0.224708	-3.78418	0.700749	0.599462	-0.10129	18	29070510	R	II		IGR	opensea	IGR-opense			
cg0884853	0.105261	0.732188	3.839544	0.001544	0.224708	-2.00264	0.784818	0.679558	-0.10526	8	1.03E+08	F	II	NCALD	5'UTR	opensea	5'UTR-open		rs1887738	1;35
cg0888459	0.105256	0.565534	4.347484	0.000544	0.224708	-0.95062	0.618162	0.512906	-0.10526	11	62037191	F	II	SCGB2A2	TSS1500	opensea	TSS1500-op		rs5618837	0;1;30;36
cg0246939	0.105236	0.598212	2.938265	0.009967	0.224708	-3.84844	0.65083	0.545594	-0.10524	8	412261	R	II	FBXO25	Body	opensea	Body-opens		rs5674489	31
cg1678976	0.105226	0.39333	3.46156	0.003381	0.224708	-2.78489	0.445943	0.340717	-0.10523	14	89066213	F	II	ZC3H14	Body	opensea	Body-opens		rs7145665	48
cg0572875	0.121095	0.443281	2.965398	0.009427	0.224708	-3.79426	0.503828	0.382733	-0.1211	14	1.05E+08	F	II		IGR	opensea	IGR-opense		rs5773501	45
cg2447548	0.105223	0.578215	3.585298	0.002615	0.224708	-2.52946	0.630826	0.525603	-0.10522	8	1625395	R	II	DLGAP2	Body	opensea	Body-opens		rs7629852	47;46;30
cg0955115	0.104386	0.562739	2.963229	0.009469	0.224708	-3.7986	0.614932	0.510546	-0.10439	1	2.1E+08	F	II		IGR	opensea	IGR-opense		rs5762574	0;10;41
cg0032739	0.105196	0.424157	3.713906	0.002003	0.224708	-2.2632	0.476755	0.371559	-0.1052	16	48642773	R	II	MIR5095	Body	shore	Body-shore	chr16:486	rs5733662	50;36
cg0111221	0.104587	0.571637	2.960741	0.009518	0.224708	-3.80357	0.62393	0.519343	-0.10459	14	89285377	R	II		IGR	opensea	IGR-opense		rs5735944	20
cg2684088	0.105176	0.531871	3.315078	0.004581	0.224708	-3.08584	0.58446	0.479283	-0.10518	18	3412693	R	II	TGIF1	TSS200	opensea	TSS200-ope		rs5608698	38
cg0791446	0.105137	0.572367	3.973613	0.001171	0.224708	-1.72446	0.624935	0.519798	-0.10514	1	1.59E+08	F	II	PYHIN1	Body	opensea	Body-opens		rs1424249	0;18
cg1597620	0.105136	0.521589	3.268984	0.00504	0.224708	-3.18012	0.574157	0.469021	-0.10514	13	94806595	R	II	GPC6-AS1	Body	opensea	Body-opens		rs5757770	1
cg2234168	0.105123	0.629579	2.6864	0.016643	0.224899	-4.34403	0.68214	0.577017	-0.10512	15	61342360	R	II	RORA	Body	opensea	Body-opens			
cg0779957	0.105119	0.542497	2.82924	0.012455	0.224708	-4.06469	0.595057	0.489938	-0.10512	5	70309843	R	II	NAIP	Body	opensea	Body-opens		rs5691943	4
cg0612174	0.105084	0.663771	2.836455	0.012273	0.224708	-4.05045	0.716313	0.611229	-0.10508	10	34511567	F	II	PARD3	Body	opensea	Body-opens		rs7456207	42;51
cg1175227	0.105081	0.696271	3.234049	0.005418	0.224708	-3.25143	0.748811	0.64373	-0.10508	2	85921036	F	II	GNLY	TSS1500	opensea	TSS1500-op		rs5736511	0;36;48

cg0157197	0.101467	0.644137	2.956166	0.009607	0.224708	-3.81271	0.69487	0.593403	-0.10147	7	84122752	R	II		IGR	opensea	IGR-opense		rs1862025	49;2
cg0121434	0.110841	0.819453	2.955656	0.009618	0.224708	-3.81373	0.874874	0.764033	-0.11084	17	406501	F	II		IGR	island	IGR-island	chr17:406	rs2011125	47
cg0253679	0.105074	0.517524	2.670359	0.017191	0.224959	-4.37509	0.570061	0.464987	-0.10507	5	1.68E+08	R	II	TENM2	Body	opensea	Body-opens		rs5635933	17;1
cg0335825	0.105062	0.52142	2.838319	0.012227	0.224708	-4.04677	0.573951	0.468889	-0.10506	2	1.83E+08	R	II	PPP1R1C	Body	opensea	Body-opens		rs1146932	28;2
cg2582767	0.109905	0.52764	2.955248	0.009626	0.224708	-3.81455	0.582592	0.472687	-0.10991	17	15255513	F	II		IGR	opensea	IGR-opense		rs5677320	37;41
cg0202594	0.103159	0.519611	2.954933	0.009632	0.224708	-3.81518	0.571191	0.468032	-0.10316	4	80998921	F	II		IGR	opensea	IGR-opense		rs5325634	11;22;48
cg0893093	0.100832	0.612565	2.953999	0.00965	0.224708	-3.81704	0.662981	0.562149	-0.10083	10	64755207	F	II		IGR	opensea	IGR-opense		rs1446983	16;40
cg0240627	0.107856	0.602564	2.953418	0.009662	0.224708	-3.8182	0.656492	0.548636	-0.10786	12	52849755	R	II		IGR	opensea	IGR-opense		rs5376864	39;1
cg2497619	0.104151	0.449226	2.952551	0.009679	0.224708	-3.81993	0.501301	0.39715	-0.10415	4	1.01E+08	R	II		IGR	opensea	IGR-opense		rs5511034	2;1
cg1426491	0.10042	0.322759	2.951628	0.009697	0.224708	-3.82177	0.372969	0.272549	-0.10042	3	1.86E+08	F	II		IGR	opensea	IGR-opense		rs5418538	1;11
cg2597534	0.101735	0.663941	2.950149	0.009727	0.224708	-3.82473	0.714809	0.613074	-0.10173	2	2.05E+08	F	II		IGR	opensea	IGR-opense		rs1174893	5
cg1689043	0.105044	0.454927	3.031277	0.008234	0.224708	-3.66217	0.507449	0.402405	-0.10504	5	1.49E+08	R	II	AFAP1L1	Body	opensea	Body-opens		rs1444141	17;2
cg0460133	0.10504	0.521658	2.843921	0.012088	0.224708	-4.03571	0.574178	0.469137	-0.10504	12	26209446	R	II	RASSF8	Body	opensea	Body-opens		rs5428438	2;1
cg1955303	0.104101	0.548944	2.949266	0.009744	0.224708	-3.82649	0.600995	0.496893	-0.1041	4	26196680	F	II		IGR	opensea	IGR-opense		rs5390600	0;1;10;48
cg2299056	0.104995	0.511634	3.552647	0.002799	0.224708	-2.59695	0.564132	0.459137	-0.10499	9	1.2E+08	R	II	ASTN2	Body	opensea	Body-opens		rs1868995	1
cg1360235	0.104968	0.644451	2.469924	0.025671	0.228885	-4.75703	0.696935	0.591967	-0.10497	3	14885560	F	II	FGD5	Body	opensea	Body-opens		rs5469159	33
cg0558231	0.104937	0.514115	3.344116	0.004313	0.224708	-3.02633	0.566583	0.461646	-0.10494	4	5760808	F	II	EVC	Body	opensea	Body-opens		rs5682419	0;31
cg0678533	0.104937	0.611831	3.543731	0.002851	0.224708	-2.61537	0.664299	0.559362	-0.10494	5	1.6E+08	R	II	CCNJL	Body	opensea	Body-opens		rs5763863	2;1
cg2429475	0.105103	0.541692	2.946044	0.009809	0.224708	-3.83292	0.594243	0.48914	-0.1051	12	46028329	F	II		IGR	opensea	IGR-opense		rs3744955	1;19
cg2036166	0.104925	0.583917	3.350127	0.00426	0.224708	-3.014	0.636379	0.531454	-0.10492	12	1.19E+08	F	II	TAOK3	Body	opensea	Body-opens		rs5393535	7;13
cg2461998	0.101498	0.564541	2.945451	0.009821	0.224708	-3.8341	0.61529	0.513792	-0.1015	4	28094243	F	II		IGR	opensea	IGR-opense		rs5493168	0;21
cg0475943	0.10157	0.402987	2.944058	0.009849	0.224708	-3.83688	0.453772	0.352202	-0.10157	8	50242329	F	II		IGR	opensea	IGR-opense		rs1843899	0;27
cg1389533	0.104913	0.622336	3.287855	0.004846	0.224708	-3.14155	0.674793	0.56988	-0.10491	9	6418884	R	II	UHRF2	Body	opensea	Body-opens		rs1868079	47
cg1963364	0.104871	0.451153	2.422013	0.028223	0.230986	-4.84649	0.503589	0.398717	-0.10487	15	76428015	R	II	C15orf27	Body	opensea	Body-opens		rs885388;r	23;14;1
cg2166463	0.10344	0.52181	2.942625	0.009878	0.224708	-3.83974	0.573531	0.47009	-0.10344	3	70432664	R	I		IGR	opensea	IGR-opense		rs1820123	13;3
cg1201432	0.124056	0.463258	2.942548	0.00988	0.224708	-3.8399	0.525286	0.40123	-0.12406	11	69226530	F	II		IGR	opensea	IGR-opense		rs5276513	13;20
cg0372532	0.104815	0.414377	2.831337	0.012402	0.224708	-4.06055	0.466784	0.36197	-0.10481	20	50015815	F	II	NFATC2	Body	opensea	Body-opens		rs7545307	0;11;49
cg1513737	0.104813	0.521853	3.45139	0.003453	0.224708	-2.80584	0.57426	0.469446	-0.10481	12	23729510	F	II	SOX5	Body	opensea	Body-opens		rs1860090	0;1
cg0508452	0.109722	0.506583	2.941783	0.009895	0.224708	-3.84142	0.561444	0.451722	-0.10972	8	1.3E+08	R	II		IGR	opensea	IGR-opense		rs5546598	1
cg0625075	0.1048	0.5797	3.065226	0.007678	0.224708	-3.59382	0.6321	0.5273	-0.1048	15	49329117	R	II	SECISBP2L	Body	opensea	Body-opens		rs5559597	46
cg1989796	0.104111	0.564077	2.941629	0.009898	0.224708	-3.84173	0.616132	0.512022	-0.10411	20	56416164	F	II		IGR	opensea	IGR-opense		rs7329123	1;15
cg0189306	0.110864	0.552041	2.941049	0.00991	0.224708	-3.84289	0.607473	0.496609	-0.11086	7	90254267	F	II		IGR	opensea	IGR-opense		rs5766203	19
cg2044078	0.12285	0.610956	2.940571	0.00992	0.224708	-3.84384	0.672381	0.549531	-0.12285	6	1.09E+08	F	II		IGR	opensea	IGR-opense		rs5453437	7;42
cg0493642	0.10088	0.42449	2.940348	0.009924	0.224708	-3.84428	0.47493	0.37405	-0.10088	3	1.46E+08	R	II		IGR	opensea	IGR-opense		rs1685787	27;26;8
cg1091651	0.104764	0.633764	3.537216	0.00289	0.224708	-2.62883	0.686146	0.581382	-0.10476	10	1246336	R	II	ADARB2	Body	opensea	Body-opens		rs1722156	24
cg0086992	0.104757	0.480655	2.240579	0.04022	0.242939	-5.17782	0.533034	0.428277	-0.10476	1	67051101	R	II	SGIP1	Body	opensea	Body-opens			
cg0653055	0.104752	0.530699	2.763724	0.014231	0.224708	-4.19341	0.583075	0.478323	-0.10475	15	54304778	F	II	UNC13C	TSS1500	opensea	TSS1500-op		rs1861455	0
cg2112560	0.104701	0.42228	3.173263	0.006143	0.224708	-3.37515	0.47463	0.369929	-0.1047	6	1.11E+08	F	II	SLC16A10	Body	opensea	Body-opens			
cg1919746	0.11061	0.508017	2.936011	0.010013	0.224708	-3.85293	0.563322	0.452712	-0.11061	1	1.86E+08	F	II		IGR	opensea	IGR-opense		rs1175151	11
cg1597187	0.102659	0.418083	2.93595	0.010014	0.224708	-3.85306	0.469412	0.366753	-0.10266	2	2.27E+08	R	II		IGR	opensea	IGR-opense		rs1380049	20;2;1
cg0755306	0.113101	0.540834	2.935468	0.010024	0.224708	-3.85402	0.597385	0.484284	-0.1131	18	54118660	F	II		IGR	opensea	IGR-opense		rs5741838	29;47
cg1316063	0.151107	0.552668	2.934865	0.010036	0.224708	-3.85522	0.628222	0.477115	-0.15111	1	1.61E+08	F	II		IGR	opensea	IGR-opense		rs1883734	1;5;5;10
cg1051615	0.110544	0.571935	2.933966	0.010055	0.224708	-3.85701	0.627207	0.516663	-0.11054	4	45624041	F	II		IGR	opensea	IGR-opense		rs2865487	0;8;35
cg0431586	0.106199	0.396014	2.933246	0.01007	0.224708	-3.85845	0.449114	0.342915	-0.1062	13	47103926	F	II		IGR	opensea	IGR-opense		rs5452154	43
cg2588287	0.10468	0.71337	3.348746	0.004272	0.224708	-3.01683	0.76571	0.66103	-0.10468	22	17701547	R	II	CECR1	TSS1500	opensea	TSS1500-op		rs1896588	45;19
cg0854272	0.105642	0.502215	2.931748	0.010101	0.224708	-3.86143	0.555036	0.449394	-0.10564	8	75010420	R	II		IGR	opensea	IGR-opense		rs8024860	8
cg0492857	0.130016	0.371825	2.931246	0.010111	0.224708	-3.86243	0.436833	0.306816	-0.13002	12	63696410	F	II		IGR	opensea	IGR-opense		rs5634446	10;16;28;44
cg0004059	0.10467	0.548504	3.446305	0.003489	0.224708	-2.81632	0.60084	0.496169	-0.10467	5	1.45E+08	R	II	SH3RF2	5'UTR	shore	5'UTR-shor	chr5:1453	rs5458217	28;24;20;9
cg1043453	0.103015	0.568825	2.929078	0.010156	0.224708	-3.86675	0.620333	0.517318	-0.10301	8	1.04E+08	R	II		IGR	opensea	IGR-opense		rs5329185	29
cg2389809	0.102406	0.635704	2.927991	0.010179	0.224708	-3.86892	0.686907	0.584501	-0.10241	6	1.67E+08	R	II		IGR	opensea	IGR-opense		rs5578234	2
cg2476166	0.103332	0.485649	2.927235	0.010195	0.224708	-3.87042	0.537315	0.433983	-0.10333	2	1.09E+08	F	II		IGR	opensea	IGR-opense			
cg2070828	0.116673	0.567502	2.927143	0.010196	0.224708	-3.87061	0.625839	0.509166	-0.11667	5	1.27E+08	F	II		IGR	opensea	IGR-opense		rs1147652	1
cg0525937	0.104665	0.52678	2.889497	0.011013	0.224708	-3.94547	0.579113	0.474448	-0.10467	13	60308743	R	II	DIAPH3	Body	opensea	Body-opens		rs1180984	49;28;5;2
cg2175155	0.106475	0.452941	2.924034	0.010262	0.224708	-3.8768	0.506178	0.399704	-0.10647	21	32451135	F	II		IGR	opensea	IGR-opense		rs5522770	1;24;26;39
cg1126754	0.10466	0.644107	3.363927	0.00414	0.224708	-2.98568	0.696437	0.591777	-0.10466	7	27167828	F	II	HOXA3	TSS1500	shore	TSS1500-sh	chr7:2716	rs1859162	27
cg2206326	0.104653	0.56354	3.218093	0.005599	0.224708	-3.28395	0.615867	0.511214	-0.10465	2	44958986	R	II	CAMKMT	Body	opensea	Body-opens		rs1437746	14
cg1386103	0.104631	0.498357	2.77752	0.013838	0.224708	-4.16639	0.550673	0.446041	-0.10463	11	5385322	R	II	OR51B5	Body	opensea	Body-opens		rs1846079	49;32
cg0584896	0.104617	0.581484	3.558856	0.002763	0.224708	-2.58412	0.633792	0.529176	-0.10462	2	1.74E+08	F	II	MLK7-AS1	Body	opensea	Body-opens		rs1397590	0;1;14;15;18;37
cg1356201	0.100878	0.625172	2.922327	0.010298	0.224708	-3.8802	0.675611	0.574733	-0.10088	22	22754456	R	II		IGR	opensea	IGR-opense		rs1442580	2
cg0762003	0.109278	0.470154	2.922208	0.0103	0.224708	-3.88044	0.524793	0.415515	-0.10928	13	39771708	R	II		IGR	opensea	IGR-opense		rs5583166	24
cg0875051	0.107321	0.52271	2.921477	0.010315	0.224708	-3.88189	0.576371	0.46905	-0.10732	5	32537617	R	II		IGR	opensea	IGR-opense		rs1909435	29;27;13
cg1314519	0.104599	0.419395	2.732545	0.01516	0.224783	-4.25432	0.471694	0.367095	-0.1046	7	1.06E+08	F	II	NAMPT	TSS1500	shore	TSS1500-sh	chr7:1059	rs6133008	48
cg1511091	0.101768	0.68086	2.918404	0.010381	0.224708	-3.88801	0.731744	0.629976	-0.10177	7	7959339	F	II		IGR	opensea	IGR-opense			
cg2522615	0.104593	0.468249	3.571726	0.00269	0.224708	-2.55752	0.520545	0.415952	-0.10459	10	89554130	R	II	ATAD1	Body	opensea	Body-opens			

cg1164545	0.104583	0.709723	2.957616	0.009579	0.224708	-3.80981	0.762014	0.657432	-0.10458	3	52864694	F	II	ITIH4	5'UTR	opensea	5'UTR-open			
cg2488821	0.104564	0.542036	3.098164	0.007174	0.224708	-3.52733	0.594318	0.489754	-0.10456	11	1.29E+08	F	II	ARHGAP32	Body	opensea	Body-opens		rs1136691	43
cg1868018	0.104541	0.633585	3.311993	0.00461	0.224708	-3.09215	0.685855	0.581314	-0.10454	14	35629311	R	II	KIAA0391	Body	opensea	Body-opens		rs1907458	10
cg1680729	0.106489	0.494631	2.915417	0.010444	0.224708	-3.89395	0.547875	0.441386	-0.10649	16	14375926	F	II		IGR	shelf	IGR-shelf	chr16:1437	rs1850731	0;3
cg2652176	0.101195	0.606618	2.915082	0.010451	0.224708	-3.89462	0.657215	0.55602	-0.10119	11	59295398	F	II		IGR	opensea	IGR-opense			
cg1176439	0.104509	0.575926	3.233833	0.00542	0.224708	-3.25187	0.62818	0.523671	-0.10451	21	47864791	F	II	PCNT	Body	opensea	Body-opens		rs1512361	0;4;22;25;32;47
cg0698629	0.104502	0.523312	3.137202	0.006618	0.224708	-3.44832	0.575563	0.471061	-0.1045	8	1.31E+08	R	II	CCDC26	Body	opensea	Body-opens		rs1881361	46
cg0086820	0.10448	0.480046	2.783021	0.013684	0.224708	-4.1556	0.532286	0.427806	-0.10448	6	1.5E+08	F	II	TAB2	Body	shore	Body-shore	chr6:1496	rs1445708	34;51
cg0340756	0.110816	0.580637	2.91295	0.010497	0.224708	-3.89886	0.636045	0.525228	-0.11082	8	1682921	R	II		IGR	opensea	IGR-opense		rs5464896	50;47;37;35
cg1172772	0.102426	0.525448	2.912663	0.010503	0.224708	-3.89943	0.576661	0.474235	-0.10243	18	42742510	R	II		IGR	opensea	IGR-opense		rs1465537	13;2;1
cg1216261	0.104444	0.509078	2.821523	0.012652	0.224708	-4.0799	0.5613	0.456856	-0.10444	6	18147472	F	II	TPMT	Body	opensea	Body-opens			
cg0655523	0.104438	0.804834	2.807143	0.013028	0.224708	-4.10821	0.857053	0.752615	-0.10444	4	1.07E+08	R	II	ARHGEF38	Body	opensea	Body-opens		rs5446146	19
cg0405506	0.104415	0.54307	3.268364	0.005046	0.224708	-3.18139	0.595277	0.490863	-0.10441	3	25382277	F	II	RARB	TSS200	opensea	TSS200-ope		rs1459922	25
cg2006035	0.104399	0.453716	2.737781	0.015	0.224756	-4.24411	0.505915	0.401517	-0.1044	8	1.07E+08	F	II	ZFPM2-AS1	Body	opensea	Body-opens		rs1896580	4;26;26
cg0956311	0.113831	0.435879	2.908535	0.010592	0.224708	-3.90764	0.492794	0.378963	-0.11383	14	1E+08	F	II		IGR	opensea	IGR-opense		rs1897442	0;2
cg0280455	0.10439	0.838554	3.682326	0.002139	0.224708	-2.32863	0.890749	0.786358	-0.10439	11	7022088	R	II	ZNF214	Body	opensea	Body-opens		rs5684049	43;32;19;2;1
cg1343487	0.103067	0.555457	2.907122	0.010623	0.224708	-3.91045	0.606991	0.503924	-0.10307	1	1.01E+08	F	II		IGR	opensea	IGR-opense		rs1152743	22;38
cg2242101	0.104666	0.713253	2.906922	0.010628	0.224708	-3.91085	0.765586	0.66092	-0.10467	1	1.51E+08	R	II		IGR	opensea	IGR-opense		rs1379003	44
cg1983404	0.104373	0.549472	3.00646	0.008665	0.224708	-3.71202	0.601658	0.497286	-0.10437	3	1.6E+08	R	II	SCHIP1	Body	opensea	Body-opens		rs5479757	49;46;36;19;1
cg2434215	0.10437	0.538467	2.797638	0.013283	0.224708	-4.1269	0.590652	0.486282	-0.10437	8	1.25E+08	F	II	FER1L6	5'UTR	opensea	5'UTR-open		rs1446023	0;3;18
cg0130318	0.104365	0.793843	2.957136	0.009588	0.224708	-3.81077	0.846026	0.741661	-0.10436	16	8714793	F	II	C16orf68	TSS1500	shore	TSS1500-sh	chr16:871	rs1496868	0;27;34;42;45
cg1115030	0.107764	0.410842	2.903988	0.010692	0.224708	-3.91668	0.464724	0.35696	-0.10776	1	1.98E+08	F	II		IGR	opensea	IGR-opense		rs5311340	0
cg0130256	0.104334	0.571677	3.309031	0.004638	0.224708	-3.09822	0.623844	0.51951	-0.10433	12	1.31E+08	F	II	RIMBP2	5'UTR	opensea	5'UTR-open		rs5314280	0;10;16;40
cg0424368	0.102086	0.635584	2.903601	0.0107	0.224708	-3.91745	0.686627	0.584541	-0.10209	6	86395706	F	II		IGR	opensea	IGR-opense		rs5544356	0;1;7;25;39
cg2736599	0.100076	0.510086	2.901386	0.010749	0.224708	-3.92186	0.560124	0.460048	-0.10008	5	1.23E+08	F	II		IGR	opensea	IGR-opense			
cg1692030	0.109947	0.583215	2.901212	0.010752	0.224708	-3.9222	0.638189	0.528242	-0.10995	4	74548513	R	II		IGR	opensea	IGR-opense		rs5495018	51;41;41;37;2;1
cg1648806	0.105536	0.545612	2.900671	0.010764	0.224708	-3.92328	0.59838	0.492844	-0.10554	1	2.05E+08	R	II		IGR	shore	IGR-shore	chr1:2046	rs5549247	32;27
cg0474987	0.10433	0.571388	2.722668	0.015466	0.224896	-4.27357	0.623553	0.519223	-0.10433	8	71039880	R	II	NCOA2	Body	opensea	Body-opens		rs1146388	16;1
cg0547735	0.104284	0.47836	3.076647	0.007499	0.224708	-3.57079	0.530503	0.426218	-0.10428	3	1.32E+08	R	II	CPNE4	Body	opensea	Body-opens			
cg2751207	0.111081	0.30635	2.898031	0.010823	0.224708	-3.92852	0.36189	0.25081	-0.11108	4	1.13E+08	R	II		IGR	shelf	IGR-shelf	chr4:1132	rs4834256	2
cg0276782	0.100862	0.545123	2.898018	0.010823	0.224708	-3.92855	0.595554	0.494692	-0.10086	4	90022810	F	II		IGR	opensea	IGR-opense			
cg1165921	0.100656	0.345415	2.897464	0.010835	0.224708	-3.92965	0.395743	0.295086	-0.10066	16	71642351	F	II		IGR	opensea	IGR-opense		rs5538458	11;14;30;33
cg1035690	0.113664	0.719802	2.897421	0.010836	0.224708	-3.92973	0.776634	0.66297	-0.11366	22	49881777	F	II		IGR	shore	IGR-shore	chr22:498	rs5550509	33;44
cg1043256	0.104253	0.561213	2.595884	0.019969	0.225563	-4.51837	0.61334	0.509087	-0.10425	17	46709858	F	II	MIR196A1	Body	shore	Body-shore	chr17:467	rs1864495	1;17;41
cg1331954	0.104251	0.413929	3.024809	0.008344	0.224708	-3.67517	0.466054	0.361803	-0.10425	2	71580473	F	II	ZNF638	Body	opensea	Body-opens		rs5751349	1
cg2756978	0.105127	0.510021	2.893332	0.010927	0.224708	-3.93785	0.562584	0.457458	-0.10513	6	27078427	R	II		IGR	opensea	IGR-opense		rs1394768	18
cg1844236	0.104244	0.36258	2.829345	0.012452	0.224708	-4.06448	0.414702	0.310458	-0.10424	7	44677772	F	II	OGDH	Body	opensea	Body-opens		rs3699458	0;1;7
cg0032942	0.106683	0.497618	2.891869	0.01096	0.224708	-3.94076	0.550959	0.444276	-0.10668	3	38193513	F	II		IGR	opensea	IGR-opense		rs5611060	18
cg2602945	0.104204	0.489343	3.126917	0.00676	0.224708	-3.46916	0.541445	0.437241	-0.1042	7	47818048	F	II	PKD1L1	Body	opensea	Body-opens		rs1436480	0;1;49;51
cg2709656	0.104168	0.567451	3.023454	0.008367	0.224708	-3.6779	0.619535	0.515367	-0.10417	1	33419301	R	II	RNF19B	Body	opensea	Body-opens		rs5327542	2;1
cg0934587	0.102596	0.643147	2.891502	0.010968	0.224708	-3.94149	0.694445	0.591849	-0.1026	1	1.7E+08	F	II		IGR	opensea	IGR-opense			
cg1448540	0.102903	0.468425	2.891338	0.010972	0.224708	-3.94181	0.519877	0.416974	-0.1029	2	45063342	R	II		IGR	opensea	IGR-opense		rs1137184	51;50;34;16;2
cg1054341	0.101422	0.52283	2.891324	0.010972	0.224708	-3.94184	0.573541	0.472119	-0.10142	3	37489393	R	II		IGR	opensea	IGR-opense		rs7628974	50;45;2
cg1399762	0.104154	0.589228	3.299025	0.004736	0.224708	-3.1187	0.641305	0.537151	-0.10415	15	51641258	F	II	GLDN	Body	opensea	Body-opens		rs1417915	1;23;25
cg2607907	0.104165	0.503096	2.890993	0.01098	0.224708	-3.9425	0.555179	0.451014	-0.10416	18	41361082	F	II		IGR	opensea	IGR-opense		rs5333896	22;51
cg2027564	0.112852	0.418118	2.890952	0.01098	0.224708	-3.94258	0.474544	0.361692	-0.11285	10	10911683	R	II		IGR	opensea	IGR-opense		rs1382590	2
cg0933585	0.105385	0.486857	2.890238	0.010997	0.224708	-3.944	0.53955	0.434164	-0.10539	3	5307317	R	II		IGR	opensea	IGR-opense		rs5388905	1
cg2611327	0.104141	0.524958	3.105665	0.007064	0.224708	-3.51217	0.577028	0.472887	-0.10414	1	54484669	R	II	LDLRAD1	TSS1500	opensea	TSS1500-op		rs2000641	51;49;2;1
cg2386661	0.104126	0.779724	2.411213	0.02883	0.231424	-4.86655	0.831787	0.727661	-0.10413	7	1.31E+08	R	II	MKLN1	Body	opensea	Body-opens		rs5612420	14;1
cg1752446	0.104124	0.443636	3.007859	0.00864	0.224708	-3.70921	0.495698	0.391575	-0.10412	3	46411369	R	II	CCR5	TSS1500	opensea	TSS1500-op		rs4138484	2;1
cg0120460	0.103163	0.443288	2.88954	0.011012	0.224708	-3.94538	0.49487	0.391707	-0.10316	16	80619852	R	II		IGR	opensea	IGR-opense		rs5339601	27;21;14
cg1922722	0.104121	0.495187	3.347806	0.00428	0.224708	-3.01876	0.547247	0.443126	-0.10412	1	67660601	F	II	IL23R	Body	opensea	Body-opens		rs5383818	0
cg1386880	0.11017	0.660564	2.8891	0.011022	0.224708	-3.94625	0.71565	0.605479	-0.11017	9	87917961	F	II		IGR	opensea	IGR-opense		rs5392606	0;6;15;30
cg1192373	0.104078	0.473722	2.956599	0.009599	0.224708	-3.81185	0.52576	0.421683	-0.10408	3	1.94E+08	F	II	LSG1	Body	opensea	Body-opens		rs5558417	36;37
cg2648973	0.110589	0.598311	2.888353	0.011039	0.224708	-3.94774	0.653606	0.543016	-0.11059	10	3930603	R	II		IGR	opensea	IGR-opense		rs5466613	45;34;28
cg0082630	0.104076	0.470456	2.906792	0.01063	0.224708	-3.91111	0.522494	0.418418	-0.10408	18	12998806	R	II	CEP192	5'UTR	opensea	5'UTR-open		rs5427516	47;46;20;1
cg1126746	0.114653	0.598341	2.886869	0.011073	0.224708	-3.95068	0.655668	0.541015	-0.11465	7	1.38E+08	F	II		IGR	opensea	IGR-opense			
cg0767569	0.104072	0.617051	3.413108	0.003738	0.224708	-2.88463	0.669087	0.565015	-0.10407	5	1.19E+08	R	II	TNFAIP8	TSS1500	shore	TSS1500-sh	chr5:1186	rs5704730	49;6
cg1753908	0.104054	0.496468	2.834044	0.012334	0.224708	-4.05521	0.548495	0.444442	-0.10405	1	42265746	F	II	HIVEP3	5'UTR	opensea	5'UTR-open		rs1450071	32;42
cg0981006	0.107749	0.662555	2.88633	0.011085	0.224708	-3.95175	0.716429	0.608681	-0.10775	20	57357380	F	II		IGR	opensea	IGR-opense		rs7482697	0;15
cg0808706	0.104017	0.574019	2.619115	0.019059	0.225391	-4.47384	0.626027	0.52201	-0.10402	16	87795808	F	II	KLHDC4	Body	shelf	Body-shelf	chr16:877	rs1865701	1;27;33;41;43
cg1624718	0.104009	0.492314	3.203931	0.005766	0.224708	-3.31279	0.544318	0.440309	-0.10401	17	63822421	R	II	CCDC46	Body	opensea	Body-opens		rs1999385	45;39;29;1
cg2709776	0.10845	0.515849	2.885106	0.011113	0.224708	-3.95418	0.570074	0.461624	-0.10845	14	21923087	R	II		IGR	shore	IGR-shore	chr14:219	rs7912057	9
cg2066343	0.104006	0.465348	2.805019	0.013085	0.224708	-4.11239	0.51735	0.413345	-0.10401	12	5696809	F	II	ANO2	Body	opensea	Body-opens		rs5295548	40;48

cg0189178	0.101744	0.674645	2.881171	0.011202	0.224708	-3.96198	0.725517	0.623773	-0.10174	3	1.78E+08	R	II		IGR	opensea	IGR-opense		rs5278634	27;1
cg0279774	0.114524	0.62591	2.880362	0.011221	0.224708	-3.96359	0.683172	0.568648	-0.11452	4	96811007	F	II		IGR	opensea	IGR-opense		rs1118303	0;15
cg1332191	0.103973	0.569706	3.521644	0.002984	0.224708	-2.66098	0.621692	0.517719	-0.10397	16	80779008	F	II	CDYL2	Body	opensea	Body-opens		rs5374096	36
cg2546931	0.103967	0.611448	3.335234	0.004393	0.224708	-3.04454	0.663432	0.559465	-0.10397	1	48704831	R	II	SLC5A9	Body	opensea	Body-opens		rs7906706	40;24
cg2018704	0.108814	0.513274	2.878597	0.011261	0.224708	-3.96709	0.567681	0.458867	-0.10881	3	28982939	R	II		IGR	opensea	IGR-opense			
cg0994242	0.103947	0.652042	3.422761	0.003664	0.224708	-2.86478	0.704015	0.600068	-0.10395	17	74913419	F	II	MGAT5B	Body	opensea	Body-opens		rs1171965	0;28
cg2363851	0.10394	0.511016	2.844685	0.012069	0.224708	-4.0342	0.562986	0.459046	-0.10394	6	39080067	F	II	C6orf64	Body	shelf	Body-shelf	chr6:39082	rs1415820	15;32;49
cg2740564	0.103936	0.461262	2.487482	0.024792	0.228087	-4.72406	0.51323	0.409294	-0.10394	5	42547112	F	II	GHR	TSS1500	opensea	TSS1500-op			
cg0624809	0.103925	0.479613	2.657312	0.017649	0.225076	-4.4003	0.531575	0.42765	-0.10392	18	6004245	F	II	L3MBTL4	Body	opensea	Body-opens		rs5430257	0
cg0570946	0.103904	0.608765	3.030751	0.008242	0.224708	-3.66323	0.660717	0.556813	-0.1039	18	21794764	F	II	OSBPL1A	Body	opensea	Body-opens		rs1424704	1;15
cg0258278	0.103865	0.558847	2.600573	0.019782	0.225467	-4.50939	0.61078	0.506915	-0.10387	9	78291792	F	II	MIR548H3	Body	opensea	Body-opens		rs5726178	0;13;51
cg0324883	0.103842	0.564828	3.519353	0.002999	0.224708	-2.66571	0.616749	0.512907	-0.10384	12	55864950	F	II	OR6C70	TSS1500	opensea	TSS1500-op		rs5517369	0;25
cg0862801	0.100101	0.595479	2.874961	0.011345	0.224708	-3.97429	0.645529	0.545428	-0.1001	5	1.76E+08	R	II		IGR	opensea	IGR-opense			
cg0079539	0.103351	0.585824	2.874785	0.011349	0.224708	-3.97464	0.6375	0.534149	-0.10335	14	87113901	F	II		IGR	opensea	IGR-opense		rs5332678	8
cg1865775	0.103839	0.52056	2.414201	0.028661	0.231242	-4.861	0.572479	0.46864	-0.10384	17	9834056	F	II	GAS7	Body	opensea	Body-opens		rs5534885	1;7;42;51
cg1441216	0.10383	0.613989	2.755157	0.01448	0.224708	-4.21017	0.665904	0.562074	-0.10383	4	81957210	R	II	BMP3	Body	opensea	Body-opens		rs1003156	26;1
cg2224770	0.103805	0.637792	2.871218	0.011432	0.224708	-3.98171	0.689694	0.585889	-0.10381	13	39953175	F	II	LHFP	Body	opensea	Body-opens		rs1492705	3;20;26;39;47
cg0379385	0.109531	0.522767	2.873607	0.011377	0.224708	-3.97698	0.577533	0.468002	-0.10953	13	76867659	F	II		IGR	opensea	IGR-opense		rs5769345	5;8;37
cg2259067	0.103805	0.620375	3.718629	0.001984	0.224708	-2.25341	0.672277	0.568473	-0.1038	5	1.45E+08	F	II	SH3RF2	Body	opensea	Body-opens		rs5577051	0;5;18
cg0087016	0.103799	0.407433	3.712577	0.002009	0.224708	-2.26595	0.459332	0.355533	-0.1038	12	12584171	F	II	LOH12CR1	Body	opensea	Body-opens		rs1923269	0;20;31
cg2539346	0.103799	0.660462	2.655878	0.0177	0.225076	-4.40307	0.712361	0.608562	-0.1038	12	1.04E+08	R	II	C12orf42	Body	opensea	Body-opens		rs5731392	45;1
cg2276405	0.104574	0.630509	2.872062	0.011413	0.224708	-3.98004	0.682796	0.578222	-0.10457	16	56684029	R	II		IGR	opensea	IGR-opense		rs7197489	38;14;2
cg2345122	0.103795	0.53801	2.580156	0.020608	0.225654	-4.54843	0.589908	0.486113	-0.10379	8	1.3E+08	R	II	LINC00824	Body	opensea	Body-opens		rs5311545	1
cg0565908	0.117185	0.538609	2.870872	0.011441	0.224708	-3.9824	0.597201	0.480016	-0.11719	7	1.14E+08	F	II		IGR	opensea	IGR-opense		rs5780346	1;28
cg2151217	0.100914	0.470258	2.870387	0.011452	0.224708	-3.98336	0.520715	0.419801	-0.10091	2	85971548	F	II		IGR	opensea	IGR-opense		rs1885887	1
cg0880370	0.103781	0.556213	2.638584	0.018327	0.225076	-4.43641	0.608104	0.504323	-0.10378	16	57273293	F	II	RSPRY1	3'UTR	opensea	3'UTR-open		rs1417706	2;41
cg2181199	0.102337	0.621159	2.869583	0.011471	0.224708	-3.98495	0.672328	0.569991	-0.10234	4	1.47E+08	R	II		IGR	opensea	IGR-opense		rs5466790	45;1
cg0456508	0.111721	0.453645	2.868716	0.011491	0.224708	-3.98667	0.509505	0.397784	-0.11172	18	19184921	F	II		IGR	shelf	IGR-shelf	chr18:1918		
cg1809126	0.103776	0.608263	2.844175	0.012081	0.224708	-4.03521	0.660151	0.556375	-0.10378	17	63822838	R	II	CCDC46	TSS200	opensea	TSS200-ope		rs5713764	14;12;10;9;2;1
cg0942073	0.101347	0.614652	2.867982	0.011508	0.224708	-3.98812	0.665325	0.563978	-0.10135	7	25705835	R	II		IGR	opensea	IGR-opense		rs1899908	36;7;1
cg2632726	0.103758	0.634122	2.894034	0.010911	0.224708	-3.93646	0.686001	0.582243	-0.10376	15	48710196	F	II	FBN1	Body	opensea	Body-opens		rs1430775	0;3;14;16
cg1744648	0.105876	0.467342	2.865343	0.01157	0.224708	-3.99335	0.52028	0.414404	-0.10588	6	6772111	F	II		IGR	opensea	IGR-opense			
cg0012261	0.127734	0.764187	2.865302	0.011571	0.224708	-3.99343	0.828054	0.700321	-0.12773	7	1562658	R	II		IGR	opensea	IGR-opense		rs5553967	41;29;14;12
cg1626680	0.106964	0.483338	2.865037	0.011578	0.224708	-3.99395	0.53682	0.429856	-0.10696	20	30943371	R	II		IGR	shelf	IGR-shelf	chr20:3094		
cg0148241	0.106417	0.521001	2.864785	0.011584	0.224708	-3.99445	0.57421	0.467793	-0.10642	1	2.26E+08	F	II		IGR	opensea	IGR-opense		rs5579066	7;27
cg2523248	0.103758	0.638935	3.626207	0.002403	0.224708	-2.44484	0.690813	0.587056	-0.10376	21	41754473	R	II	DSCAM-AS1	TSS1500	opensea	TSS1500-op		rs6222380	14;11;2
cg1137518	0.103742	0.444956	2.837107	0.012257	0.224708	-4.04916	0.496827	0.393085	-0.10374	1	2.37E+08	R	II	RYR2	Body	opensea	Body-opens		rs1463152	51;1
cg0539749	0.10374	0.624565	3.394985	0.003881	0.224708	-2.92189	0.676435	0.572695	-0.10374	13	33105476	R	II	N4BP2L2	Body	opensea	Body-opens		rs1497398	31;18;13;8
cg0487683	0.115368	0.535625	2.863343	0.011618	0.224708	-3.99731	0.593309	0.477941	-0.11537	5	33273644	F	II		IGR	opensea	IGR-opense		rs2331189	11;24;33;47
cg2104930	0.116958	0.497978	2.861621	0.011659	0.224708	-4.00071	0.556457	0.439499	-0.11696	17	53702388	F	II		IGR	opensea	IGR-opense		rs5651161	0;30
cg0425989	0.116318	0.541624	2.861539	0.011661	0.224708	-4.00087	0.599783	0.483465	-0.11632	8	1.3E+08	R	II		IGR	opensea	IGR-opense		rs1095647	12;2;1
cg2515918	0.103735	0.537225	3.185557	0.005989	0.224708	-3.35016	0.589092	0.485357	-0.10373	2	2.31E+08	R	II	TRIP12	Body	opensea	Body-opens			
cg1487909	0.103716	0.548784	2.506772	0.02386	0.227295	-4.68772	0.600642	0.496926	-0.10372	1	1.85E+08	F	II	FAM129A	Body	opensea	Body-opens		rs5294297	1;4;11;24;44
cg0451843	0.103707	0.542734	2.886639	0.011078	0.224708	-3.95114	0.594587	0.49088	-0.10371	7	55981411	F	II	ZNF713	Body	opensea	Body-opens		rs5499886	27
cg0043240	0.106182	0.511776	2.86049	0.011686	0.224708	-4.00295	0.564867	0.458685	-0.10618	5	1.81E+08	F	II		IGR	opensea	IGR-opense		rs1808494	37
cg1236700	0.107979	0.411758	2.860423	0.011687	0.224708	-4.00308	0.465748	0.357768	-0.10798	18	55673626	F	II		IGR	opensea	IGR-opense		rs1821329	21
cg1597360	0.105682	0.497086	2.859871	0.0117	0.224708	-4.00418	0.549927	0.444245	-0.10568	5	96159726	F	II		IGR	opensea	IGR-opense		rs5397084	0;48
cg2708356	0.103685	0.519	2.878254	0.011269	0.224708	-3.96777	0.570842	0.467158	-0.10368	18	34522301	F	II	KIAA1328	Body	opensea	Body-opens		rs5519001	42
cg1866188	0.108964	0.548141	2.858437	0.011735	0.224708	-4.00701	0.602623	0.493659	-0.10896	4	833876	R	II		IGR	opensea	IGR-opense		rs5406829	24;1
cg1890455	0.103681	0.62812	2.805239	0.013079	0.224708	-4.11196	0.679961	0.57628	-0.10368	10	93624759	F	II	TNKS2	3'UTR	opensea	3'UTR-open		rs5768482	11
cg1113246	0.103672	0.57846	2.987014	0.009018	0.224708	-3.75101	0.630296	0.526624	-0.10367	8	1.42E+08	R	II	PTP4A3	5'UTR	shelf	5'UTR-shelf	chr8:14242	rs1832582	44;26
cg0702868	0.103653	0.608658	3.058486	0.007785	0.224708	-3.60741	0.660485	0.556832	-0.10365	6	1.14E+08	F	II	LOC10192	Body	opensea	Body-opens		rs1427248	26;47
cg0573078	0.106454	0.601277	2.853887	0.011844	0.224708	-4.01601	0.654504	0.54805	-0.10645	7	17193341	R	II		IGR	opensea	IGR-opense		rs1382460	5;2
cg0436921	0.100196	0.575168	2.85373	0.011848	0.224708	-4.01632	0.625266	0.52507	-0.1002	5	1.39E+08	R	II		IGR	opensea	IGR-opense		rs5686674	22;20
cg1322835	0.103626	0.519019	3.450407	0.00346	0.224708	-2.80787	0.570832	0.467206	-0.10363	20	53223230	F	II	DOK5	Body	opensea	Body-opens		rs5711444	0;1;17;22;36
cg1257266	0.103564	0.643001	3.006683	0.008661	0.224708	-3.71157	0.694783	0.591219	-0.10356	2	72788795	R	II	EXOC6B	Body	opensea	Body-opens		rs5449612	1
cg2453800	0.103563	0.581647	2.990654	0.008951	0.224708	-3.74371	0.633429	0.529866	-0.10356	1	17558947	R	II	PADI1	Body	opensea	Body-opens		rs5626957	39;30;29;2
cg0250191	0.103553	0.578837	2.80556	0.01307	0.224708	-4.11132	0.630613	0.52706	-0.10355	2	1.09E+08	R	II	LIMS1	Body	opensea	Body-opens		rs5487182	38;1
cg1173578	0.143851	0.600649	2.849298	0.011956	0.224708	-4.02509	0.672574	0.528723	-0.14385	3	1.27E+08	F	II		IGR	opensea	IGR-opense		rs5321871	26
cg1653280	0.101959	0.530867	2.8492	0.011958	0.224708	-4.02528	0.581847	0.479887	-0.10196	1	1.94E+08	R	II		IGR	opensea	IGR-opense		rs5756363	34;10
cg0858731	0.105158	0.550071	2.848246	0.011981	0.224708	-4.02716	0.60265	0.497492	-0.10516	8	1.3E+08	R	II		IGR	opensea	IGR-opense		rs5328739	49;5
cg2108022	0.103549	0.570944	3.343791	0.004316	0.224708	-3.02699	0.622719	0.51917	-0.10355	11	95933005	R	II	MAML2	Body	opensea	Body-opens		rs1898167	20
cg0396225	0.103513	0.519621	2.793462	0.013396	0.224708	-4.1351	0.571377	0.467864	-0.10351	13	96211557	R	II	CLDN10	Body	opensea	Body-opens		rs5545256	24
cg1583814	0.114602	0.437617	2.847345	0.012003	0.224708	-4.02894	0.494918	0.380316	-0.1146	10	28700847	R	II		IGR	opensea	IGR-opense			

cg1551234	0.103501	0.614967	2.819626	0.012701	0.224708	-4.08364	0.666717	0.563217	-0.1035	8	1.31E+08	R	II	CCDC26	Body	opensea	Body-opens		rs1895508	21
cg0434972	0.103499	0.640926	3.414409	0.003728	0.224708	-2.88196	0.692675	0.589176	-0.1035	19	51457389	F	II	KLK5	TSS1500	opensea	TSS1500-op		rs5390481	1;23;32;33
cg0176771	0.119783	0.467435	2.844859	0.012065	0.224708	-4.03386	0.527327	0.407544	-0.11978	6	1.41E+08	F	II		IGR	opensea	IGR-opense			
cg2496545	0.103488	0.545314	3.414281	0.003729	0.224708	-2.88222	0.597058	0.49357	-0.10349	2	7578495	F	II	LOC10050(	Body	opensea	Body-opens		rs5498317	46
cg0715399	0.103487	0.48607	2.970332	0.009332	0.224708	-3.7844	0.537813	0.434326	-0.10349	5	1.72E+08	R	II	SH3PXD2B	Body	opensea	Body-opens		rs5689607	38;25;8
cg1568569	0.103484	0.549348	3.143968	0.006527	0.224708	-3.43461	0.60109	0.497606	-0.10348	17	18247645	R	II	SHMT1	Body	opensea	Body-opens		rs1872239	33;15;1
cg1573538	0.103471	0.673581	3.062927	0.007714	0.224708	-3.59846	0.725316	0.621845	-0.10347	8	1.25E+08	R	II	FER1L6	Body	opensea	Body-opens		rs5575712	44;27;2
cg1299544	0.10346	0.654593	3.242233	0.005327	0.224708	-3.23474	0.706323	0.602863	-0.10346	6	1.23E+08	R	II	PKIB	Body	opensea	Body-opens		rs1837973	51;45;23
cg1791696	0.114559	0.350646	2.843236	0.012105	0.224708	-4.03706	0.407925	0.293366	-0.11456	15	79447300	F	II		IGR	opensea	IGR-opense		rs5404727	33;49
cg0793717	0.11355	0.476	2.843018	0.01211	0.224708	-4.03749	0.532775	0.419225	-0.11355	1	2.19E+08	R	II		IGR	opensea	IGR-opense		rs5416872	1
cg0295698	0.103452	0.681531	3.076321	0.007504	0.224708	-3.57144	0.733257	0.629805	-0.10345	1	74929125	F	II	TNNI3K	ExonBnd	opensea	ExonBnd-o|		rs1458406	1;6;7;21;26;41;45
cg0751281	0.103444	0.49481	3.563024	0.002739	0.224708	-2.57551	0.546532	0.443088	-0.10344	19	11203661	R	II	LDLR	Body	shore	Body-shore	chr19:112(	rs5511773	41;28;2;1
cg0577586	0.1121	0.380524	2.841746	0.012141	0.224708	-4.04	0.436574	0.324474	-0.1121	10	31026101	F	II		IGR	opensea	IGR-opense		rs1889843	0;1;29;49
cg0210114	0.105745	0.790043	2.841089	0.012158	0.224708	-4.0413	0.842916	0.737171	-0.10574	2	1.75E+08	R	II		IGR	opensea	IGR-opense		rs1449245	1
cg2713019	0.10343	0.566225	2.775736	0.013888	0.224708	-4.16988	0.61794	0.51451	-0.10343	8	29028955	F	II	KIF13B	Body	opensea	Body-opens		rs1827352	22;36
cg2589471	0.103394	0.677557	2.983936	0.009075	0.224708	-3.75717	0.729254	0.625859	-0.10339	4	75133599	F	II	MTHFD2L	Body	opensea	Body-opens		rs1685081	0
cg1329982	0.103388	0.418389	3.686778	0.002119	0.224708	-2.31941	0.470083	0.366695	-0.10339	1	92414737	R	II	BRDT	TSS200	island	TSS200-isla	chr1:92414		
cg0033265	0.103356	0.580684	3.173111	0.006145	0.224708	-3.37546	0.632362	0.529006	-0.10336	16	78132184	F	II	WWOX	TSS1500	shore	TSS1500-sh	chr16:781:	rs1466084	20
cg1553199	0.103353	0.64481	2.818285	0.012736	0.224708	-4.08628	0.696486	0.593134	-0.10335	12	94602562	R	II	PLXNC1	Body	opensea	Body-opens		rs5280029	22;15;13
cg1369789	0.103003	0.516054	2.837824	0.012239	0.224708	-4.04775	0.567555	0.464552	-0.103	17	73312128	F	II		IGR	opensea	IGR-opense		rs1141066	44
cg0986915	0.103346	0.628332	2.876061	0.01132	0.224708	-3.97212	0.680005	0.576659	-0.10335	8	6405472	R	II	ANGPT2	Body	opensea	Body-opens		rs7607168	51;49;2
cg1071857	0.103339	0.682648	3.380553	0.003999	0.224708	-2.95155	0.734318	0.630978	-0.10334	2	74859870	R	II	M1AP	Body	opensea	Body-opens		rs7805888	44;1
cg0758107	0.107328	0.531572	2.83703	0.012259	0.224708	-4.04932	0.585236	0.477908	-0.10733	6	40291048	F	II		IGR	opensea	IGR-opense		rs6032991	0;26
cg1946334	0.117488	0.725803	2.836708	0.012267	0.224708	-4.04995	0.784547	0.667059	-0.11749	13	27316791	F	II		IGR	opensea	IGR-opense		rs2182879	9
cg0158418	0.10332	0.529596	2.708977	0.015901	0.224896	-4.30021	0.581256	0.477936	-0.10332	18	24136594	F	II	KCTD1	Body	opensea	Body-opens		rs2006677	23;36;48
cg0423459	0.106103	0.5354	2.835781	0.01229	0.224708	-4.05178	0.588452	0.482349	-0.1061	5	3851581	R	II		IGR	shelf	IGR-shelf	chr5:3854!	rs5374055	6
cg1082900	0.103304	0.552091	3.484916	0.003221	0.224708	-2.73675	0.603743	0.500439	-0.1033	14	60179825	F	II	RTN1	Body	opensea	Body-opens		rs5530072	29
cg2753192	0.104264	0.432715	2.834674	0.012318	0.224708	-4.05397	0.484847	0.380583	-0.10426	13	98598462	R	II		IGR	opensea	IGR-opense		rs5741080	1
cg0877181	0.103286	0.632593	3.191916	0.005911	0.224708	-3.33723	0.684236	0.58095	-0.10329	14	37740094	R	II	MIPOL1	Body	opensea	Body-opens		rs1830127	2
cg0815317	0.103282	0.550313	2.754003	0.014514	0.224708	-4.21243	0.601954	0.498672	-0.10328	11	1.27E+08	F	II	KIRREL3	Body	opensea	Body-opens		rs5649188	0;30;45;51
cg0426365	0.106958	0.476174	2.833133	0.012357	0.224708	-4.05701	0.529653	0.422695	-0.10696	19	30521287	R	II		IGR	opensea	IGR-opense		rs7805828	44
cg0742232	0.10327	0.579206	2.874603	0.011354	0.224708	-3.975	0.630841	0.527571	-0.10327	1	1.87E+08	F	II	PTGS2	3'UTR	opensea	3'UTR-open		rs1848452	0;1
cg0543255	0.103216	0.499494	2.872424	0.011404	0.224708	-3.97932	0.551102	0.447886	-0.10322	17	62632136	R	II	SMURF2	Body	opensea	Body-opens		rs5554456	29;7;2
cg0590491	0.101059	0.671321	2.832581	0.01237	0.224708	-4.0581	0.72185	0.620791	-0.10106	6	1.56E+08	R	II		IGR	opensea	IGR-opense		rs5567461	2
cg1179840	0.103213	0.565817	2.980039	0.009148	0.224708	-3.76497	0.617423	0.51421	-0.10321	21	40145361	R	II	NCRNA001	Body	opensea	Body-opens		rs5352195	15
cg2199854	0.103202	0.700067	2.99421	0.008886	0.224708	-3.73659	0.751668	0.648466	-0.1032	10	33605101	F	II	NRP1	Body	opensea	Body-opens		rs1128471	1
cg0625205	0.103195	0.592694	3.515951	0.00302	0.224708	-2.67273	0.644292	0.541097	-0.10319	11	68923361	F	I	LOC338694	Body	shore	Body-shore	chr11:689:	rs5729407	26;28;31;43
cg2130847	0.10792	0.39439	2.831198	0.012405	0.224708	-4.06082	0.448349	0.34043	-0.10792	1	12911678	R	II		IGR	opensea	IGR-opense		rs1143835	45;44;35;26;2;1
cg2564555	0.103185	0.462917	2.90042	0.01077	0.224708	-3.92377	0.51451	0.411324	-0.10319	6	17470599	F	II	CAP2	Body	opensea	Body-opens		rs5680000	5;38
cg2578389	0.103185	0.426442	3.142158	0.006551	0.224708	-3.43828	0.478035	0.37485	-0.10319	10	85898649	F	II	GHITM	TSS1500	shore	TSS1500-sh	chr10:858!	rs1452945	30
cg1222168	0.103178	0.548994	2.908146	0.010601	0.224708	-3.90842	0.600584	0.497405	-0.10318	2	98827983	R	II	VWA3B	Body	opensea	Body-opens			
cg2358697	0.100337	0.490646	2.828915	0.012463	0.224708	-4.06533	0.540814	0.440478	-0.10034	2	1.24E+08	F	II		IGR	opensea	IGR-opense		rs1853358	1;12
cg1529259	0.100551	0.392266	2.828613	0.012471	0.224708	-4.06592	0.442541	0.34199	-0.10055	2	36374912	F	II		IGR	opensea	IGR-opense		rs5597701	1
cg1366330	0.103172	0.5934	3.170498	0.006179	0.224708	-3.38077	0.644986	0.541814	-0.10317	5	35046992	F	II	AGXT2	Body	opensea	Body-opens		rs5414041	38
cg1720284	0.101042	0.580903	2.825995	0.012538	0.224708	-4.07108	0.631424	0.530382	-0.10104	13	43294719	F	II		IGR	opensea	IGR-opense		rs1501921	19
cg0859073	0.10317	0.614548	2.819434	0.012706	0.224708	-4.08401	0.666133	0.562963	-0.10317	4	77624601	F	II	SHROOM3	Body	opensea	Body-opens			
cg2166208	0.10317	0.527374	2.753881	0.014518	0.224708	-4.21267	0.578959	0.475789	-0.10317	10	228801	R	II	ZMYND11	Body	opensea	Body-opens		rs5660542	19
cg1854628	0.103161	0.575646	3.696273	0.002078	0.224708	-2.29974	0.627227	0.524065	-0.10316	2	2.34E+08	R	II	NGEF	Body	opensea	Body-opens		rs1878709	14
cg1277418	0.107856	0.411866	2.823618	0.012598	0.224708	-4.07577	0.465794	0.357938	-0.10786	1	1.92E+08	F	II		IGR	opensea	IGR-opense		rs1124379	44;47
cg1721053	0.10315	0.573718	2.784456	0.013644	0.224708	-4.15278	0.625293	0.522143	-0.10315	3	1.83E+08	F	II	MCF2L2	Body	opensea	Body-opens		rs5675522	43
cg2497191	0.103141	0.660368	3.224899	0.005521	0.224708	-3.27008	0.711939	0.608797	-0.10314	15	66404837	F	II	MEGF11	Body	opensea	Body-opens		rs5580055	4;24
cg0714200	0.15496	0.603591	2.821183	0.012661	0.224708	-4.08057	0.681071	0.52611	-0.15496	17	6656315	F	II		IGR	opensea	IGR-opense		rs1503499	9
cg0979330	0.103097	0.516904	2.702692	0.016104	0.224896	-4.31242	0.568453	0.465355	-0.1031	1	2.13E+08	R	II	ATF3	5'UTR	opensea	5'UTR-open		rs1435368	19;2
cg0884791	0.103093	0.46416	2.729987	0.015239	0.224792	-4.25931	0.515707	0.412613	-0.10309	1	25105247	F	II	CLIC4	Body	opensea	Body-opens		rs7553761	9;10;24;29
cg0377503	0.103089	0.424812	2.578522	0.020676	0.225654	-4.55155	0.476357	0.373268	-0.10309	2	88874141	R	II	LOC101928	Body	opensea	Body-opens		rs5445031	50;28;1
cg0507971	0.103059	0.842346	2.423373	0.028147	0.23092	-4.84396	0.893875	0.790816	-0.10306	7	1.32E+08	F	II	PLXNA4	Body	opensea	Body-opens		rs1164385	0;38;44
cg0613664	0.102963	0.540869	2.576402	0.020764	0.225654	-4.5556	0.59235	0.489387	-0.10296	7	1.49E+08	F	II	ZNF282	Body	opensea	Body-opens			
cg2478713	0.109145	0.602037	2.818836	0.012722	0.224708	-4.08519	0.65661	0.547465	-0.10914	5	1.47E+08	R	II		IGR	opensea	IGR-opense			
cg1900330	0.102962	0.411336	2.91807	0.010388	0.224708	-3.88867	0.462817	0.359855	-0.10296	11	1.15E+08	R	II	CADM1	Body	opensea	Body-opens		rs7357214	21;19;1
cg0140004	0.102932	0.49524	3.177876	0.006085	0.224708	-3.36578	0.546706	0.443774	-0.10293	15	61091220	R	II	RORA	Body	opensea	Body-opens			
cg0388999	0.102896	0.537731	3.013115	0.008547	0.224708	-3.69866	0.589179	0.486284	-0.1029	1	1.58E+08	F	II	FCRL4	Body	opensea	Body-opens		rs5325413	0;2;16;24;34
cg0212232	0.241536	0.470285	2.81657	0.012781	0.224708	-4.08965	0.591053	0.349517	-0.24154	13	50194322	F	II		IGR	opensea	IGR-opense		rs1419685	21;31
cg0229028	0.102881	0.606743	2.663166	0.017442	0.225076	-4.38899	0.658183	0.555302	-0.10288	7	26479853	R	II	LOC441204	Body	opensea	Body-opens		rs5646655	8
cg0938403	0.111907	0.585976	2.814847	0.012826	0.224708	-4.09305	0.641929	0.530022	-0.11191	8	1.04E+08	F	II		IGR	opensea	IGR-opense			

cg2636558	0.102871	0.638947	3.062071	0.007728	0.224708	-3.60018	0.690382	0.587512	-0.10287	1	1.72E+08	R	II	DNM3	Body	opensea	Body-opens		rs5575786	51;49
cg0534932	0.100059	0.654956	2.813509	0.012861	0.224708	-4.09568	0.704986	0.604926	-0.10006	9	1.11E+08	R	II		IGR	opensea	IGR-opense		rs1898441	45;1
cg0745409	0.102863	0.495641	3.442531	0.003517	0.224708	-2.82409	0.547073	0.44421	-0.10286	13	52435964	F	II	CCDC70	TSS200	opensea	TSS200-ope			
cg2662097	0.101348	0.513267	2.810507	0.012939	0.224708	-4.10159	0.563941	0.462593	-0.10135	4	1.52E+08	R	II		IGR	opensea	IGR-opense		rs5577611	1;14;50
cg1721729	0.102858	0.392607	2.570135	0.021026	0.225654	-4.56755	0.444036	0.341178	-0.10286	1	1.59E+08	F	II	AIM2	TSS1500	opensea	TSS1500-op		rs1162896	1;8;42
cg2429828	0.102853	0.526582	3.359125	0.004181	0.224708	-2.99554	0.578009	0.475155	-0.10285	6	1.25E+08	F	II	RNF217	5'UTR	opensea	5'UTR-open			
cg1346400	0.102849	0.64541	3.119798	0.006861	0.224708	-3.48357	0.696835	0.593985	-0.10285	17	38976709	F	II	TMEM99	5'UTR	opensea	5'UTR-open		rs5535599	1;7;15;20;38;45
cg0181928	0.100841	0.627016	2.807193	0.013027	0.224708	-4.10811	0.677436	0.576595	-0.10084	12	46006284	R	II		IGR	opensea	IGR-opense			
cg0300461	0.102848	0.50801	2.751816	0.014579	0.224708	-4.2167	0.559434	0.456586	-0.10285	2	2.32E+08	R	II	ARMC9	Body	opensea	Body-opens		rs1855918	47;1
cg1354054	0.104315	0.540543	2.807138	0.013029	0.224708	-4.10822	0.592701	0.488385	-0.10432	19	16424005	R	II		IGR	opensea	IGR-opense		rs1440038	48;28
cg1887592	0.102835	0.700473	3.165524	0.006242	0.224708	-3.39087	0.751891	0.649056	-0.10284	5	1.16E+08	R	II	SEMA6A	5'UTR	opensea	5'UTR-open		rs5441291	5;2
cg0324583	0.210239	0.400706	2.806917	0.013034	0.224708	-4.10865	0.505825	0.295586	-0.21024	17	6656617	F	II		IGR	opensea	IGR-opense		rs1871828	0;16;19
cg1355136	0.100246	0.598094	2.80673	0.013039	0.224708	-4.10902	0.648217	0.547971	-0.10025	5	57370189	R	II		IGR	opensea	IGR-opense		rs5446055	40
cg2691213	0.100684	0.49667	2.806417	0.013048	0.224708	-4.10964	0.547012	0.446328	-0.10068	7	1.3E+08	F	II		IGR	opensea	IGR-opense			
cg0637296	0.103472	0.58639	2.806282	0.013051	0.224708	-4.1099	0.638126	0.534653	-0.10347	1	43351860	R	II		IGR	shore	IGR-shore	chr1:43351	rs1922801	1
cg0106202	0.102834	0.390739	3.814759	0.001626	0.224708	-2.05406	0.442157	0.339322	-0.10283	1	1.62E+08	R	II	SH2D1B	TSS1500	opensea	TSS1500-op			
cg0045101	0.102821	0.464829	3.176102	0.006107	0.224708	-3.36938	0.516239	0.413418	-0.10282	18	6315761	R	II	L3MBTL4	5'UTR	opensea	5'UTR-open		rs1670131	19
cg2065417	0.10281	0.442587	4.786922	0.000225	0.224708	-0.0506	0.493992	0.391182	-0.10281	3	1.57E+08	R	II	CCNL1	Body	opensea	Body-opens		rs1133126	24;1
cg0985234	0.102801	0.555301	2.807043	0.013031	0.224708	-4.10841	0.606701	0.5039	-0.1028	6	1.12E+08	R	II	REV3L	Body	opensea	Body-opens		rs1860621	1
cg2330542	0.106958	0.445279	2.803274	0.013131	0.224708	-4.11582	0.498758	0.3918	-0.10696	11	1.1E+08	R	II		IGR	opensea	IGR-opense		rs5731021	51;32;31;2
cg0359373	0.102792	0.516807	3.446321	0.003489	0.224708	-2.81628	0.568203	0.465411	-0.10279	2	2.02E+08	R	II	AOX2P	Body	opensea	Body-opens		rs7398839	36;22;13;1
cg0583581	0.10573	0.581246	2.801598	0.013176	0.224708	-4.11912	0.634111	0.528382	-0.10573	1	1.54E+08	R	II		IGR	opensea	IGR-opense		rs5612743	35;9;6
cg2070750	0.101374	0.595856	2.799274	0.013239	0.224708	-4.12368	0.646543	0.545169	-0.10137	2	38663934	F	II		IGR	opensea	IGR-opense		rs925256	27
cg2034726	0.102789	0.717348	2.166127	0.046405	0.250615	-5.31006	0.768743	0.665953	-0.10279	17	3808157	F	II	P2RX1	ExonBnd	opensea	ExonBnd-op		rs5571870	0;1;22;25
cg2476898	0.102773	0.668879	3.021522	0.0084	0.224708	-3.68178	0.720265	0.617492	-0.10277	6	1.25E+08	F	II	NKAIN2	TSS1500	opensea	TSS1500-op		rs5735528	27;48
cg1390845	0.114759	0.562184	2.797464	0.013288	0.224708	-4.12724	0.619563	0.504804	-0.11476	13	73909590	R	II		IGR	opensea	IGR-opense		rs5331352	49;46;45;2
cg0875272	0.102762	0.620141	2.831897	0.012388	0.224708	-4.05944	0.671522	0.56876	-0.10276	21	36254259	R	II	RUNX1	Body	opensea	Body-opens		rs1848865	2;1
cg0653900	0.102748	0.468761	2.917172	0.010407	0.224708	-3.89046	0.520135	0.417387	-0.10275	2	2.08E+08	F	II	CPO	Body	opensea	Body-opens		rs1150623	0;29
cg0130319	0.102735	0.434877	3.14393	0.006527	0.224708	-3.43468	0.486245	0.38351	-0.10274	7	1.29E+08	R	II	AHCYL2	Body	opensea	Body-opens			
cg1218908	0.103285	0.501664	2.794501	0.013368	0.224708	-4.13306	0.553306	0.450022	-0.10328	4	1.85E+08	F	II		IGR	opensea	IGR-opense		rs1905910	0
cg0023317	0.107975	0.700539	2.793959	0.013383	0.224708	-4.13413	0.754527	0.646552	-0.10798	2	31499671	R	II		IGR	opensea	IGR-opense		rs1827928	15;1
cg1336400	0.102734	0.6138	2.653695	0.017778	0.225076	-4.40728	0.665167	0.562433	-0.10273	1	1.79E+08	F	II	RALGPS2	Body	opensea	Body-opens		rs5690147	18;39
cg0704579	0.102726	0.756215	3.051458	0.007899	0.224708	-3.62156	0.807578	0.704851	-0.10273	6	1.17E+08	F	II	DSE	5'UTR	opensea	5'UTR-open		rs1170718	16
cg1180086	0.102722	0.611541	2.816764	0.012776	0.224708	-4.08927	0.662902	0.56018	-0.10272	10	64981888	R	II	JMJD1C	Body	opensea	Body-opens		rs5571536	4
cg0741889	0.102708	0.447591	3.743916	0.001882	0.224708	-2.20099	0.498946	0.396237	-0.10271	6	52150456	R	II	MCM3	TSS1500	shore	TSS1500-sh	chr6:5214!	rs5337167	42;32
cg0582052	0.101293	0.623833	2.791596	0.013447	0.224708	-4.13877	0.67448	0.573187	-0.10129	10	82132926	R	II		IGR	opensea	IGR-opense		rs5884460	30
cg1683663	0.102705	0.341238	2.888792	0.011029	0.224708	-3.94686	0.39259	0.289886	-0.1027	17	57905956	F	II	VMP1	Body	opensea	Body-opens		rs5463910	19
cg1060029	0.100157	0.444436	2.788069	0.013544	0.224708	-4.14569	0.494514	0.394357	-0.10016	20	1789945	F	II		IGR	opensea	IGR-opense		rs5665149	38
cg0857742	0.102697	0.53892	2.780794	0.013746	0.224708	-4.15997	0.590268	0.487571	-0.1027	6	31876831	F	II	C2	5'UTR	opensea	5'UTR-open		rs1909883	28
cg0080369	0.102673	0.418339	2.702712	0.016103	0.224896	-4.31238	0.469675	0.367002	-0.10267	3	46411474	R	II	CCR5	TSS200	opensea	TSS200-ope		rs5509919	44
cg2288665	0.102644	0.470282	3.114582	0.006935	0.224708	-3.49413	0.521604	0.41896	-0.10264	6	498807	R	II	EXOC2	Body	opensea	Body-opens		rs5451429	1
cg1439379	0.10263	0.630426	2.786209	0.013595	0.224708	-4.14934	0.681741	0.579111	-0.10263	15	45379815	F	II		IGR	opensea	IGR-opense		rs1466085	40
cg2649853	0.102638	0.525444	2.943633	0.009858	0.224708	-3.83773	0.576763	0.474124	-0.10264	2	1.98E+08	R	II	HSPD1	Body	shore	Body-shore	chr2:1983(	rs1381707	28;27;14
cg1232119	0.102638	0.768334	3.247444	0.00527	0.224708	-3.2241	0.819653	0.717015	-0.10264	13	78550384	F	II	EDNRB	TSS1500	opensea	TSS1500-op		rs1866987	0;30
cg2236001	0.109365	0.383403	2.785434	0.013617	0.224708	-4.15086	0.438085	0.32872	-0.10937	2	1.05E+08	R	II		IGR	shore	IGR-shore	chr2:10527		
cg1713618	0.103821	0.541768	2.785365	0.013619	0.224708	-4.151	0.593678	0.489858	-0.10382	5	6030096	R	II		IGR	opensea	IGR-opense		rs1880286	30;2
cg0436148	0.102637	0.530707	3.059916	0.007762	0.224708	-3.60452	0.582026	0.479389	-0.10264	8	1.28E+08	F	II	CASC8	Body	opensea	Body-opens		rs5284540	46
cg2180802	0.102629	0.428929	2.807529	0.013018	0.224708	-4.10745	0.480244	0.377615	-0.10263	18	11871622	F	II	GNAL	Body	opensea	Body-opens		rs5325637	0
cg1346538	0.102622	0.542247	2.348752	0.032592	0.234761	-4.98175	0.593558	0.490936	-0.10262	1	47282666	F	II	CYP4B1	Body	opensea	Body-opens		rs1817041	0;1;18;37
cg1108893	0.102606	0.517092	3.105478	0.007066	0.224708	-3.51255	0.568394	0.465789	-0.10261	7	78629839	F	II	MAGI2	Body	opensea	Body-opens		rs1117729	31
cg2097580	0.102577	0.613854	3.297398	0.004752	0.224708	-3.12203	0.665143	0.562565	-0.10258	1	76254925	R	II	RABGGTB	Body	opensea	Body-opens		rs5334552	43
cg1269676	0.102559	0.411563	2.691316	0.016479	0.224896	-4.3345	0.462842	0.360283	-0.10256	18	60848167	F	II	BCL2	Body	opensea	Body-opens		rs5664592	4;17;28
cg1947099	0.102538	0.689007	3.45052	0.003459	0.224708	-2.80763	0.740276	0.637738	-0.10254	2	1.8E+08	R	II	SESTD1	5'UTR	opensea	5'UTR-open		rs5366778	28
cg0524182	0.102532	0.547869	2.743233	0.014835	0.224708	-4.23347	0.599135	0.496603	-0.10253	4	85648511	R	II	WDFY3	Body	opensea	Body-opens		rs5579855	48;36
cg2619848	0.103969	0.576795	2.781334	0.013731	0.224708	-4.15891	0.62878	0.524811	-0.10397	13	1.04E+08	F	II		IGR	opensea	IGR-opense		rs5367734	1
cg1339990	0.102528	0.579326	3.006697	0.00866	0.224708	-3.71154	0.630591	0.528062	-0.10253	15	50352288	F	II	ATP8B4	Body	opensea	Body-opens		rs5693275	28
cg2631598	0.102522	0.668922	5.074662	0.000127	0.223139	0.52999	0.720183	0.617661	-0.10252	1	65363870	F	II	JAK1	5'UTR	opensea	5'UTR-open		rs1400224	36
cg1179621	0.102512	0.562006	2.980447	0.00914	0.224708	-3.76416	0.613262	0.51075	-0.10251	3	1.95E+08	F	II	C3orf21	Body	opensea	Body-opens		rs1479254	37
cg0965721	0.100406	0.577653	2.780244	0.013761	0.224708	-4.16105	0.627856	0.52745	-0.10041	5	1.58E+08	F	II		IGR	opensea	IGR-opense		rs5437879	41
cg0220948	0.1011	0.580505	2.779342	0.013786	0.224708	-4.16281	0.631055	0.529955	-0.1011	4	1.86E+08	F	II		IGR	opensea	IGR-opense		rs4541559	34;39;44
cg0882674	0.102508	0.545663	2.778881	0.013799	0.224708	-4.16372	0.596917	0.494409	-0.10251	8	1.05E+08	F	II	DPYS	3'UTR	opensea	3'UTR-open		rs5701781	22
cg1353759	0.116911	0.577037	2.778588	0.013808	0.224708	-4.16429	0.635493	0.518582	-0.11691	12	26451968	R	II		IGR	opensea	IGR-opense		rs5385523	48;1
cg1073305	0.102485	0.529107	3.105296	0.007069	0.224708	-3.51292	0.580349	0.477865	-0.10248	6	34778879	F	II	UHRF1BP1	Body	opensea	Body-opens		rs5311480	0
cg0322499	0.117941	0.433452	2.777652	0.013834	0.224708	-4.16613	0.492422	0.374481	-0.11794	1	51750976	F	II		IGR	opensea	IGR-opense		rs5510240	11;47

cg0652033	0.102475	0.590279	2.972043	0.009299	0.224708	-3.78097	0.641517	0.539042	-0.10248	10	93171002	F	II	LOC10018	Body	shore	Body-shore	chr10:931	rs1119443	0
cg2008421	0.10246	0.697335	2.724476	0.01541	0.224896	-4.27005	0.748565	0.646105	-0.10246	17	36997449	R	II	C17orf98	1stExon	island	1stExon-isl	chr17:369	rs6175091	9
cg1803815	0.114841	0.516556	2.776521	0.013866	0.224708	-4.16835	0.573977	0.459136	-0.11484	8	1.04E+08	R	II		IGR	opensea	IGR-opense			
cg1396880	0.102449	0.643696	2.93682	0.009996	0.224708	-3.85132	0.694921	0.592471	-0.10245	5	58081039	R	II	RAB3C	Body	opensea	Body-opens		rs446155	11
cg0616023	0.102448	0.520961	4.060057	0.00098	0.224708	-1.54518	0.572185	0.469736	-0.10245	9	1.35E+08	F	II	RAPGEF1	Body	opensea	Body-opens		rs1168735	1;23;44
cg0918599	0.102447	0.56353	3.539287	0.002877	0.224708	-2.62455	0.614754	0.512307	-0.10245	12	22090231	R	II	ABCC9	TSS1500	shelf	TSS1500-sh	chr12:220	rs7509213	30;17
cg0247627	0.100884	0.602639	2.773827	0.013942	0.224708	-4.17363	0.653081	0.552197	-0.10088	1	1.56E+08	R	II		IGR	opensea	IGR-opense		rs1842924	50
cg1065205	0.102395	0.546753	2.997217	0.008831	0.224708	-3.73056	0.59795	0.495555	-0.10239	13	43649126	R	II	DNAJC15	Body	opensea	Body-opens		rs1430925	36;35;28;11
cg1191258	0.102374	0.557842	2.6952	0.01635	0.224896	-4.32696	0.609029	0.506655	-0.10237	1	2.03E+08	F	II	MGAT4EP	Body	opensea	Body-opens		rs1092048	13;18;35;42
cg0159468	0.102366	0.575773	3.125539	0.00678	0.224708	-3.47195	0.626956	0.52459	-0.10237	16	3115053	R	II	IL32	TSS1500	opensea	TSS1500-op		rs1882202	46;2
cg0982766	0.102365	0.577468	2.814136	0.012844	0.224708	-4.09445	0.628651	0.526285	-0.10237	3	1.12E+08	R	II	SLC35A5	5'UTR	shore	5'UTR-shor	chr3:1122	rs5474139	46;27;11;9
cg0260019	0.113429	0.528821	2.769036	0.014078	0.224708	-4.18301	0.585536	0.472107	-0.11343	8	1.36E+08	R	II		IGR	opensea	IGR-opense		rs1861060	16;2
cg0013473	0.101499	0.612933	2.768946	0.014081	0.224708	-4.18319	0.663682	0.562183	-0.1015	1	1.2E+08	R	II		IGR	opensea	IGR-opense		rs5876723	37
cg2437971	0.100234	0.579574	2.768842	0.014084	0.224708	-4.18339	0.629691	0.529457	-0.10023	6	1.41E+08	F	II		IGR	opensea	IGR-opense		rs5541648	1;8;9;10;45
cg1212624	0.102358	0.614706	3.100915	0.007133	0.224708	-3.52177	0.665885	0.563527	-0.10236	14	59742285	F	II	DAAM1	Body	opensea	Body-opens		rs5550038	1;19;44
cg0783036	0.101532	0.503019	2.767966	0.014109	0.224708	-4.18511	0.553786	0.452253	-0.10153	3	1.54E+08	R	II		IGR	opensea	IGR-opense		rs5384325	21;2
cg2365209	0.102356	0.535251	3.670099	0.002194	0.224708	-2.35396	0.586429	0.484073	-0.10236	11	75347560	R	II	MAP6	Body	opensea	Body-opens			
cg0268500	0.102326	0.70401	2.759628	0.01435	0.224708	-4.20143	0.755173	0.652847	-0.10233	3	31903434	R	II	OSBPL10	Body	opensea	Body-opens		rs5659464	49;29
cg1034232	0.102308	0.468978	2.905889	0.01065	0.224708	-3.9129	0.520132	0.417824	-0.10231	14	52171238	F	II	FRMD6	Body	opensea	Body-opens		rs5523043	0;1;28;45;50
cg0037648	0.102299	0.736344	3.329186	0.004449	0.224708	-3.05694	0.787493	0.685194	-0.1023	21	46892832	F	II	COL18A1	Body	shore	Body-shore	chr21:468	rs5715049	3;40
cg0936726	0.102296	0.582098	2.832879	0.012363	0.224708	-4.05751	0.633247	0.53095	-0.1023	6	6643814	F	II	LY86	Body	opensea	Body-opens		rs1903988	41
cg2349032	0.101824	0.657556	2.766574	0.014149	0.224708	-4.18783	0.708469	0.606644	-0.10182	5	57014231	F	II		IGR	opensea	IGR-opense			
cg0290254	0.102296	0.533489	3.250043	0.005241	0.224708	-3.2188	0.584637	0.482341	-0.1023	16	50136493	R	II	HEATR3	Body	opensea	Body-opens		rs5563205	46;1
cg2625074	0.106165	0.538597	2.766421	0.014153	0.224708	-4.18813	0.591679	0.485514	-0.10617	6	1.23E+08	R	II		IGR	opensea	IGR-opense		rs7476909	1
cg0467990	0.102279	0.289431	3.551547	0.002805	0.224708	-2.59922	0.340571	0.238292	-0.10228	22	19752869	F	II	TBX1	Body	shore	Body-shore	chr22:197	rs5315765	21;28;30
cg0425806	0.101424	0.526265	2.765756	0.014172	0.224708	-4.18943	0.576977	0.475553	-0.10142	4	1.75E+08	F	II		IGR	opensea	IGR-opense			
cg1656617	0.102252	0.426932	3.561982	0.002745	0.224708	-2.57766	0.478058	0.375806	-0.10225	1	86315952	R	II	COL24A1	Body	opensea	Body-opens		rs5557481	50;14;1
cg1410981	0.102252	0.53066	3.119635	0.006863	0.224708	-3.48391	0.581786	0.479534	-0.10225	3	69963596	F	II	MITF	Body	opensea	Body-opens		rs5379503	0
cg0737893	0.102247	0.546483	4.249281	0.000665	0.224708	-1.15339	0.597606	0.495359	-0.10225	3	1.12E+08	F	II	TAGLN3	Body	opensea	Body-opens		rs7323207	43
cg1382844	0.10224	0.719994	3.630011	0.002384	0.224708	-2.43696	0.771114	0.668874	-0.10224	12	10459972	R	II	KLRD1	TSS1500	opensea	TSS1500-op		rs5530452	50;35
cg2461313	0.113469	0.467052	2.763257	0.014244	0.224708	-4.19433	0.523786	0.410318	-0.11347	8	1.29E+08	R	II		IGR	opensea	IGR-opense			
cg0870181	0.10015	0.643926	2.762062	0.014279	0.224708	-4.19666	0.694001	0.593851	-0.10015	8	95278142	R	II		IGR	shelf	IGR-shelf	chr8:9527	rs1457100	32
cg2694796	0.10223	0.623363	2.78614	0.013597	0.224708	-4.14948	0.674478	0.572247	-0.10223	17	54334619	F	II	ANKFN1	Body	opensea	Body-opens		rs1927016	0;8;50
cg0576650	0.102227	0.638471	3.059142	0.007774	0.224708	-3.60609	0.689585	0.587358	-0.10223	2	2.08E+08	F	II	DYTN	Body	opensea	Body-opens		rs5351007	1;39
cg1960210	0.108771	0.60847	2.760841	0.014314	0.224708	-4.19905	0.662855	0.554084	-0.10877	4	90800500	F	II		IGR	opensea	IGR-opense		rs1893177	27
cg2690455	0.102226	0.632835	2.891035	0.010979	0.224708	-3.94241	0.683948	0.581722	-0.10223	2	10587170	F	II	ODC1	5'UTR	shore	5'UTR-shor	chr2:1058		
cg1455272	0.102222	0.621505	2.967688	0.009383	0.224708	-3.78968	0.672616	0.570394	-0.10222	3	1.51E+08	R	II	IGSF10	TSS200	opensea	TSS200-ope		rs5651631	3
cg0007052	0.102189	0.531526	2.747123	0.014718	0.224708	-4.22587	0.582621	0.480432	-0.10219	13	43986655	R	II	ENOX1	Body	opensea	Body-opens		rs5556257	44;1
cg2089185	0.10218	0.631948	3.187798	0.005962	0.224708	-3.34561	0.683037	0.580858	-0.10218	10	1.19E+08	R	II	SHTN1	Body	opensea	Body-opens		rs5321389	47;15;2
cg1642618	0.106555	0.523841	2.759114	0.014365	0.224708	-4.20243	0.577119	0.470564	-0.10656	4	1.79E+08	F	II		IGR	opensea	IGR-opense		rs5545237	1;48
cg1917777	0.104131	0.556596	2.758675	0.014377	0.224708	-4.20329	0.608661	0.504531	-0.10413	4	1.42E+08	R	II		IGR	opensea	IGR-opense		rs1137738	1
cg1155650	0.102178	0.484784	3.57335	0.002681	0.224708	-2.55416	0.535873	0.433695	-0.10218	2	2.06E+08	R	II	PARD3B	Body	opensea	Body-opens		rs1494896	2
cg1188980	0.102163	0.508693	2.8743	0.011361	0.224708	-3.9756	0.559775	0.457612	-0.10216	15	86253551	F	II	AKAP13	Body	opensea	Body-opens		rs5277685	1
cg1244092	0.102156	0.818708	3.353689	0.004228	0.224708	-3.00669	0.869786	0.76763	-0.10216	7	1.58E+08	R	II	PTPRN2	Body	opensea	Body-opens		rs2860248	28;12
cg0514799	0.102148	0.503964	3.184272	0.006005	0.224708	-3.35278	0.555038	0.45289	-0.10215	5	72982878	F	II	ARHGEF28	Body	opensea	Body-opens		rs1925181	40
cg0489321	0.102139	0.624488	3.041518	0.008062	0.224708	-3.64157	0.675558	0.573419	-0.10214	9	14310315	R	II	NFIB	Body	shelf	Body-shelf	chr9:1431		
cg1623098	0.10213	0.374773	4.710967	0.000261	0.224708	-0.20513	0.425838	0.323708	-0.10213	14	52336657	F	II	GNG2	5'UTR	opensea	5'UTR-open		rs1431103	0;25;26;47
cg0267283	0.102106	0.513691	2.492018	0.02457	0.227903	-4.71553	0.564744	0.462638	-0.10211	8	6286451	R	II	MCPH1	Body	opensea	Body-opens		rs7935276	36;22;20;16
cg0738930	0.104964	0.504509	2.748275	0.014684	0.224708	-4.22362	0.556991	0.452027	-0.10496	2	2.32E+08	F	II		IGR	opensea	IGR-opense		rs3702063	8;36;41
cg1199382	0.102105	0.347191	2.834446	0.012323	0.224708	-4.05442	0.398244	0.296139	-0.1021	4	38525620	F	II	LINC01258	TSS1500	opensea	TSS1500-op		rs1387812	1
cg1229911	0.101971	0.591914	2.743876	0.014816	0.224708	-4.23221	0.6429	0.540929	-0.10197	3	72282244	R	II		IGR	opensea	IGR-opense		rs3595045	34;7;4;2
cg0645747	0.1021	0.41407	2.603521	0.019665	0.225391	-4.50375	0.46512	0.363021	-0.1021	4	1.52E+08	F	II	SH3D19	TSS1500	opensea	TSS1500-op		rs1929553	37
cg0757275	0.100487	0.533115	2.743212	0.014836	0.224708	-4.23351	0.583359	0.482872	-0.10049	2	1.58E+08	R	II		IGR	opensea	IGR-opense		rs5331996	1
cg1757391	0.102097	0.702674	2.766975	0.014137	0.224708	-4.18705	0.753723	0.651626	-0.1021	3	1.43E+08	F	II	SLC9A9	Body	opensea	Body-opens		rs1430244	0;12
cg1821131	0.102063	0.487078	3.310677	0.004623	0.224708	-3.09485	0.538109	0.436046	-0.10206	8	98437059	F	II	LOC10192	Body	opensea	Body-opens		rs3685368	0;21;27;36;37
cg0329148	0.102047	0.48884	3.862552	0.001473	0.224708	-1.9549	0.539864	0.437817	-0.10205	11	5879372	F	II	OR52E8	TSS1500	opensea	TSS1500-op		rs5592706	24
cg1883586	0.102046	0.449273	2.833083	0.012358	0.224708	-4.05711	0.500296	0.39825	-0.10205	14	52020726	F	II	FRMD6	5'UTR	opensea	5'UTR-open		rs1126210	2;30;32;37
cg0757520	0.102037	0.461855	2.850693	0.011922	0.224708	-4.02233	0.512874	0.410836	-0.10204	16	75046592	R	II	ZNRF1	Body	opensea	Body-opens		rs5499192	12;11;2;1
cg0624651	0.104291	0.440162	2.738193	0.014987	0.224747	-4.24331	0.492308	0.388017	-0.10429	8	1.02E+08	R	II		IGR	opensea	IGR-opense		rs5723783	48;42;39
cg0149367	0.102021	0.554089	3.561128	0.00275	0.224708	-2.57943	0.605099	0.503078	-0.10202	6	32784825	R	II	HLA-DOB	TSS200	opensea	TSS200-ope		rs2071469	44
cg1838544	0.102021	0.419411	2.884916	0.011117	0.224708	-3.95456	0.470422	0.368401	-0.10202	10	1.06E+08	R	II	GSTO2	TSS1500	shore	TSS1500-sh	chr10:106	rs5625682	24;15;13
cg0773984	0.102019	0.600512	3.9929	0.001125	0.224708	-1.68446	0.651522	0.549503	-0.10202	6	1.6E+08	R	II	FNDC1	Body	shelf	Body-shelf	chr6:1596	rs5304354	29
cg1063154	0.103447	0.5887	2.735869	0.015058	0.22478	-4.24784	0.640424	0.536977	-0.10345	4	1.15E+08	F	II		IGR	shelf	IGR-shelf	chr4:1149	rs5359994	42
cg2085848	0.101007	0.49575	2.73272	0.015154	0.224783	-4.25398	0.546254	0.445247	-0.10101	15	52369365	F	II		IGR	opensea	IGR-opense		rs5521638	1

cg1805371	0.102017	0.585485	3.31225	0.004608	0.224708	-3.09163	0.636493	0.534476	-0.10202	13	99049874	F	II	FARP1	Body	opensea	Body-opens		rs7652923	1;30;33;40
cg1174325	0.102006	0.504097	2.832005	0.012385	0.224708	-4.05923	0.5551	0.453094	-0.10201	4	14784843	F	II	LINC00504	Body	opensea	Body-opens		rs5578152	0;40;50
cg2600268	0.126224	0.555721	2.731164	0.015202	0.224792	-4.25701	0.618833	0.492609	-0.12622	9	85835742	F	II		IGR	opensea	IGR-opense			
cg0287964	0.102005	0.623916	2.541005	0.022286	0.226234	-4.62295	0.674919	0.572914	-0.10201	2	2.34E+08	F	II	KCNJ13	5'UTR	opensea	5'UTR-open			
cg1112934	0.101998	0.543753	3.36622	0.00412	0.224708	-2.98098	0.594752	0.492754	-0.102	11	19870761	F	II	NAV2	Body	opensea	Body-opens			
cg0067295	0.101991	0.566047	2.799096	0.013244	0.224708	-4.12403	0.617043	0.515052	-0.10199	4	1.7E+08	F	II	SH3RF1	Body	opensea	Body-opens		rs1920605	0;36;43;49
cg2570516	0.137084	0.336149	2.727905	0.015303	0.224807	-4.26337	0.404691	0.267608	-0.13708	8	1.06E+08	F	II		IGR	opensea	IGR-opense		rs5585244	1;28;39
cg2619642	0.101989	0.527341	3.383552	0.003975	0.224708	-2.94539	0.578335	0.476346	-0.10199	14	58615960	R	II	C14orf37	5'UTR	shelf	5'UTR-shelf	chr14:5861	rs5683508	2
cg2292642	0.101946	0.556925	3.525761	0.002959	0.224708	-2.65248	0.607898	0.505952	-0.10195	12	70721074	F	II	CNOT2	Body	opensea	Body-opens		rs5307862	18;37
cg2348559	0.100166	0.619795	2.726254	0.015354	0.224856	-4.26658	0.669878	0.569712	-0.10017	7	25601773	F	II		IGR	opensea	IGR-opense		rs3718350	1;39
cg0207907	0.107473	0.479255	2.726145	0.015358	0.224856	-4.2668	0.532992	0.425518	-0.10747	1	2.08E+08	R	II		IGR	opensea	IGR-opense		rs5520171	9;8
cg2259389	0.100597	0.572545	2.725578	0.015375	0.224886	-4.2679	0.622843	0.522246	-0.1006	20	1781910	F	II		IGR	shore	IGR-shore	chr20:178	rs5504520	0;2;34;35;48
cg0540883	0.101946	0.563435	3.011715	0.008572	0.224708	-3.70147	0.614408	0.512462	-0.10195	5	1.77E+08	F	II	LMAN2	Body	shelf	Body-shelf	chr5:1767	rs7545838	47
cg1568382	0.111912	0.540932	2.723386	0.015444	0.224896	-4.27217	0.596888	0.484976	-0.11191	6	37111556	R	II		IGR	opensea	IGR-opense		rs1137070	1
cg1395975	0.10193	0.629389	2.64197	0.018203	0.225076	-4.42988	0.680354	0.578424	-0.10193	2	2.07E+08	F	II	ADAM23	Body	opensea	Body-opens		rs1112685	31
cg1357148	0.101929	0.496443	2.942122	0.009888	0.224708	-3.84075	0.547407	0.445478	-0.10193	12	1.11E+08	F	II	ATP2A2	Body	opensea	Body-opens			
cg0567437	0.101915	0.399035	3.165864	0.006238	0.224708	-3.39018	0.449992	0.348077	-0.10191	20	43275684	R	II	ADA	Body	opensea	Body-opens			
cg2472795	0.101912	0.468385	2.454141	0.026487	0.229519	-4.78659	0.519341	0.417429	-0.10191	1	2.08E+08	F	II	CR2	Body	opensea	Body-opens		rs5761839	0;11;28
cg1440314	0.137382	0.609265	2.721119	0.015515	0.224896	-4.27658	0.677956	0.540574	-0.13738	10	70974027	R	II		IGR	opensea	IGR-opense			
cg1433946	0.108644	0.357227	2.720818	0.015524	0.224896	-4.27717	0.411549	0.302905	-0.10864	13	27927490	R	II		IGR	opensea	IGR-opense		rs5274154	24
cg0815404	0.101909	0.479322	2.947332	0.009783	0.224708	-3.83035	0.530277	0.428367	-0.10191	7	1.05E+08	F	II	ATXN7L1	Body	opensea	Body-opens		rs5725654	41
cg0909719	0.101903	0.419205	2.864295	0.011595	0.224708	-3.99542	0.470156	0.368253	-0.1019	22	26145960	R	II	MYO18B	5'UTR	opensea	5'UTR-open		rs5439446	2
cg1373868	0.108464	0.51707	2.71945	0.015567	0.224896	-4.27983	0.571302	0.462838	-0.10846	3	1.86E+08	R	II		IGR	opensea	IGR-opense		rs1918843	20;17
cg0514181	0.101894	0.585054	2.712506	0.015788	0.224896	-4.29335	0.636001	0.534107	-0.10189	1	2.48E+08	F	II	OR13G1	TSS1500	opensea	TSS1500-op		rs1809210	22
cg1022538	0.10189	0.436013	3.151411	0.006427	0.224708	-3.41951	0.486958	0.385068	-0.10189	6	1.09E+08	F	II	ARMC2	5'UTR	opensea	5'UTR-open		rs5521452	2;4
cg0423410	0.106291	0.463892	2.716367	0.015665	0.224896	-4.28583	0.517037	0.410747	-0.10629	10	6051242	R	II		IGR	opensea	IGR-opense		rs4129467	32;19;2
cg1426030	0.10188	0.638852	3.615205	0.002458	0.224708	-2.4676	0.689792	0.587912	-0.10188	18	61256157	F	II	SERPINB13	Body	opensea	Body-opens		rs1435459	0;47
cg1479259	0.108932	0.569176	2.714838	0.015713	0.224896	-4.28881	0.623642	0.51471	-0.10893	22	50980925	F	II		IGR	shore	IGR-shore	chr22:5098	rs1390045	0
cg0166327	0.103239	0.457621	2.714641	0.015719	0.224896	-4.28919	0.50924	0.406001	-0.10324	1	1.09E+08	R	II		IGR	opensea	IGR-opense		rs5517594	1
cg0411438	0.108469	0.596248	2.713321	0.015761	0.224896	-4.29176	0.650483	0.542014	-0.10847	1	2.29E+08	F	II		IGR	opensea	IGR-opense		rs5284557	1;41
cg0142010	0.101879	0.508366	2.741773	0.014879	0.224708	-4.23632	0.559306	0.457427	-0.10188	5	94237541	R	II	MCTP1	Body	opensea	Body-opens		rs1440062	13;2
cg1850882	0.101872	0.463866	2.557385	0.021569	0.225744	-4.59183	0.514802	0.41293	-0.10187	4	76440715	F	II	RCHY1	TSS1500	shore	TSS1500-sh	chr4:7643	rs1434152	13;19
cg1467966	0.124336	0.692003	2.712173	0.015798	0.224896	-4.29399	0.754171	0.629835	-0.12434	8	1.02E+08	F	II		IGR	opensea	IGR-opense		rs5491057	0;1
cg1536705	0.101867	0.483836	2.960572	0.009521	0.224708	-3.80391	0.53477	0.432903	-0.10187	7	92271711	R	II	CDK6	Body	opensea	Body-opens		rs5616818	33;26
cg2678396	0.109103	0.480988	2.710715	0.015845	0.224896	-4.29683	0.535539	0.426436	-0.1091	6	1.33E+08	R	II		IGR	opensea	IGR-opense		rs5438982	37;8;3
cg0012623	0.117702	0.23888	2.71067	0.015846	0.224896	-4.29691	0.297731	0.18003	-0.1177	1	58857835	F	II		IGR	opensea	IGR-opense		rs1901122	5;14;21
cg2305192	0.101866	0.519675	4.188085	0.000754	0.224708	-1.27997	0.570608	0.468742	-0.10187	3	1.05E+08	F	II	CBLB	Body	opensea	Body-opens		rs5554347	25;39
cg0508032	0.101845	0.50864	3.171533	0.006165	0.224708	-3.37866	0.559562	0.457717	-0.10184	14	64330822	F	II	SYNE2	5'UTR	opensea	5'UTR-open			
cg0148083	0.101834	0.469616	2.664925	0.01738	0.225076	-4.38559	0.520533	0.418699	-0.10183	3	1.14E+08	F	II	ZBTB20	Body	opensea	Body-opens			
cg2453311	0.119487	0.590548	2.70872	0.015909	0.224896	-4.30071	0.650292	0.530805	-0.11949	2	54622005	F	II		IGR	opensea	IGR-opense			
cg1379684	0.101827	0.55703	2.964051	0.009453	0.224708	-3.79695	0.607944	0.506117	-0.10183	15	76303634	F	II	NRG4	5'UTR	opensea	5'UTR-open		rs5412571	31
cg0369301	0.101771	0.565415	2.706123	0.015993	0.224896	-4.30575	0.6163	0.514529	-0.10177	10	95715050	F	II		IGR	opensea	IGR-opense		rs3727602	1
cg1303504	0.101144	0.540395	2.705183	0.016023	0.224896	-4.30758	0.590967	0.489823	-0.10114	1	2.21E+08	R	II		IGR	opensea	IGR-opense			
cg2555739	0.101811	0.566637	3.473331	0.003299	0.224708	-2.76064	0.617542	0.515731	-0.10181	10	62296386	R	II	ANK3	Body	opensea	Body-opens		rs5646519	48;15
cg0842356	0.101782	0.454546	2.691136	0.016485	0.224896	-4.33485	0.505437	0.403655	-0.10178	9	20355400	F	II	MLLT3	Body	opensea	Body-opens		rs5312661	25;48
cg1355908	0.106109	0.545154	2.703658	0.016073	0.224896	-4.31054	0.598208	0.492099	-0.10611	15	94441198	F	II		IGR	opensea	IGR-opense		rs5305341	0
cg1541008	0.101776	0.572693	3.665646	0.002214	0.224708	-2.36318	0.623582	0.521805	-0.10178	10	1.19E+08	R	II	PDZD8	Body	opensea	Body-opens		rs1480882	2;1
cg2074091	0.101763	0.391087	2.847944	0.011989	0.224708	-4.02776	0.441969	0.340205	-0.10176	3	1.25E+08	F	II	SLC12A8	Body	opensea	Body-opens		rs1870849	1;4;16;45
cg2563038	0.101733	0.502887	4.366379	0.000524	0.224708	-0.91166	0.553754	0.452021	-0.10173	1	1.57E+08	R	II	SH2D2A	Body	shore	Body-shore	chr1:1567	rs1415450	39;6
cg2481404	0.100746	0.50742	2.701787	0.016133	0.224896	-4.31418	0.557793	0.457047	-0.10075	1	44550872	F	II		IGR	opensea	IGR-opense		rs1399423	0;46
cg1566614	0.106017	0.463769	2.700951	0.016161	0.224896	-4.3158	0.516778	0.410761	-0.10602	3	1.3E+08	F	II		IGR	shelf	IGR-shelf	chr3:1300	rs5399514	21
cg1649647	0.101725	0.571059	3.156511	0.00636	0.224708	-3.40917	0.621921	0.520197	-0.10172	2	1.15E+08	F	II	LOC10106	Body	opensea	Body-opens		rs5752980	1
cg1919237	0.101685	0.696807	3.868838	0.001454	0.224708	-1.94186	0.747649	0.645964	-0.10169	11	62209356	F	II	AHNAK	Body	shelf	Body-shelf	chr11:622		
cg0443616	0.10173	0.711843	2.697795	0.016264	0.224896	-4.32193	0.762708	0.660979	-0.10173	9	33220728	F	II		IGR	opensea	IGR-opense		rs1162150	0;15
cg0426768	0.100728	0.576797	2.697147	0.016286	0.224896	-4.32319	0.627161	0.526433	-0.10073	6	1.06E+08	F	II		IGR	opensea	IGR-opense		rs1866413	0;24
cg2057825	0.100279	0.56379	2.695843	0.016329	0.224896	-4.32572	0.61393	0.51365	-0.10028	10	97732488	F	II		IGR	opensea	IGR-opense		rs5757202	34
cg1136171	0.10901	0.622472	2.695325	0.016346	0.224896	-4.32672	0.676977	0.567967	-0.10901	13	1E+08	R	II		IGR	opensea	IGR-opense		rs1454540	47;1
cg0693517	0.101669	0.585915	4.14462	0.000824	0.224708	-1.36995	0.63675	0.535081	-0.10167	2	1.15E+08	R	II	ACTR3	5'UTR	opensea	5'UTR-open		rs5475406	39;35;1
cg2070167	0.10166	0.413899	3.298872	0.004737	0.224708	-3.11901	0.464729	0.363069	-0.10166	2	64782942	F	II	AFTPH	Body	opensea	Body-opens		rs5682482	9;20
cg2525943	0.100159	0.529429	2.694838	0.016362	0.224896	-4.32767	0.579508	0.47935	-0.10016	11	23100280	R	II		IGR	opensea	IGR-opense		rs1168649	18;8;2
cg0554349	0.106148	0.595987	2.693585	0.016403	0.224896	-4.3301	0.649061	0.542913	-0.10615	2	1.51E+08	R	II		IGR	opensea	IGR-opense			
cg1801001	0.101658	0.716109	3.709507	0.002021	0.224708	-2.27231	0.766938	0.665279	-0.10166	15	98507208	F	II	ARRDC4	Body	shelf	Body-shelf	chr15:985	rs5536029	3;31;40
cg0457031	0.10164	0.68559	2.54215	0.022235	0.226195	-4.62077	0.73641	0.634769	-0.10164	6	24776902	R	II	GMNN	5'UTR	shore	5'UTR-shor	chr6:2477		
cg0212342	0.101631	0.415589	2.407768	0.029026	0.231628	-4.87294	0.466404	0.364773	-0.10163	8	74433128	R	II	STAU2	Body	opensea	Body-opens		rs5459806	1

cg1348046	0.101622	0.519767	2.634059	0.018495	0.225084	-4.44512	0.570577	0.468956	-0.10162	4	16795757	F	II	LDB2	Body	opensea	Body-open		rs5746426	0;5;11
cg0742707	0.110728	0.488767	2.688173	0.016584	0.224896	-4.34059	0.544131	0.433403	-0.11073	3	1.89E+08	F	II		IGR	opensea	IGR-opense		rs5750090	10
cg2408217	0.101608	0.557643	2.949932	0.009731	0.224708	-3.82516	0.608447	0.506839	-0.10161	3	1.87E+08	F	II	SNORA63	TSS1500	shore	TSS1500-sh	chr3:1865	rs5352836	1;2
cg1489925	0.117379	0.527968	2.685959	0.016658	0.224899	-4.34488	0.586658	0.469278	-0.11738	2	1.14E+08	R	II		IGR	opensea	IGR-opense		rs5771367	31;26;25;1
cg1393150	0.101604	0.592071	3.039641	0.008093	0.224708	-3.64535	0.642873	0.541269	-0.1016	2	1.7E+08	F	II	ABCB11	ExonBnd	opensea	ExonBnd-o		rs1815336	0
cg1168837	0.101601	0.709341	2.923318	0.010277	0.224708	-3.87823	0.760141	0.65854	-0.1016	1	49238689	F	II	BEND5	Body	shelf	Body-shelf	chr1:4924	rs1509334	1;7;10;16;18;19;27
cg0792043	0.106179	0.445764	2.681718	0.016801	0.224901	-4.3531	0.498853	0.392675	-0.10618	11	1.03E+08	F	II		IGR	opensea	IGR-opense		rs5307623	1;26;47
cg1536882	0.101601	0.517642	2.479954	0.025166	0.228429	-4.73821	0.568443	0.466842	-0.1016	10	31293955	F	II	ZNF438	Body	opensea	Body-open		rs1424217	45
cg0682353	0.165794	0.551751	2.680314	0.016849	0.224901	-4.35582	0.634648	0.468853	-0.16579	4	38719348	F	II		IGR	opensea	IGR-opense		rs1136911	0
cg1217668	0.101479	0.60139	2.679944	0.016861	0.224901	-4.35654	0.652129	0.55065	-0.10148	20	23018803	R	II		IGR	shore	IGR-shore	chr20:230	rs5463990	48
cg2151562	0.107048	0.530188	2.679917	0.016862	0.224901	-4.35659	0.583712	0.476664	-0.10705	3	1.52E+08	F	II		IGR	opensea	IGR-opense		rs7665697	34;42
cg0801726	0.101581	0.454104	2.411485	0.028815	0.231424	-4.86604	0.504894	0.403313	-0.10158	17	28500882	F	II	NSRP1	Body	opensea	Body-open		rs5602489	7
cg2733492	0.105101	0.720894	2.67891	0.016897	0.224901	-4.35854	0.773445	0.668344	-0.1051	9	1.02E+08	F	II		IGR	opensea	IGR-opense		rs5570898	0;4;10
cg0151664	0.105613	0.545918	2.678896	0.016897	0.224901	-4.35857	0.598724	0.493112	-0.10561	1	28471495	F	II		IGR	opensea	IGR-opense			
cg0060207	0.106763	0.65061	2.678805	0.0169	0.224901	-4.35874	0.703992	0.597229	-0.10676	5	33245479	F	II		IGR	opensea	IGR-opense		rs5540954	0;1
cg1685850	0.101579	0.636048	3.269302	0.005036	0.224708	-3.17947	0.686838	0.585259	-0.10158	11	88347154	F	II	GRM5	Body	opensea	Body-open		rs5370141	0;15;23;44
cg0571473	0.102778	0.451572	2.677644	0.01694	0.224901	-4.36099	0.502961	0.400183	-0.10278	1	32409096	F	II		IGR	shore	IGR-shore	chr1:3241	rs5467830	1
cg0349512	0.104192	0.666703	2.674768	0.017038	0.224901	-4.36656	0.718799	0.614607	-0.10419	9	1.38E+08	F	II		IGR	opensea	IGR-opense		rs5771753	0;1;14;19;25
cg1676068	0.103669	0.647837	2.672104	0.01713	0.224959	-4.37171	0.699672	0.596003	-0.10367	6	1.35E+08	F	II		IGR	opensea	IGR-opense			
cg2494672	0.101556	0.552171	2.834743	0.012316	0.224708	-4.05383	0.602949	0.501393	-0.10156	18	32398024	R	II	DTNA	Body	opensea	Body-open		rs1493362	50;15
cg2406231	0.101494	0.584089	2.977698	0.009192	0.224708	-3.76966	0.634836	0.533342	-0.10149	4	41610944	R	II	LIMCH1	Body	opensea	Body-open			
cg1077992	0.101489	0.523366	2.721805	0.015493	0.224896	-4.27525	0.574111	0.472622	-0.10149	3	1.67E+08	R	II	WDR49	Body	opensea	Body-open			
cg1602503	0.106351	0.642524	2.669213	0.01723	0.225005	-4.3773	0.6957	0.589349	-0.10635	1	2.07E+08	R	II		IGR	opensea	IGR-opense		rs5453057	10;9
cg1182858	0.101463	0.718259	3.597736	0.002549	0.224708	-2.50374	0.76899	0.667528	-0.10146	1	2E+08	R	II	NR5A2	5'UTR	opensea	5'UTR-open		rs1419838	2
cg1924378	0.101457	0.636797	3.232384	0.005436	0.224708	-3.25482	0.687525	0.586068	-0.10146	3	23567565	F	II	UBE2E2	Body	opensea	Body-open		rs5602612	1;20
cg2675391	0.101449	0.51185	3.27604	0.004967	0.224708	-3.1657	0.562574	0.461126	-0.10145	14	24540526	R	II	CPNE6	TSS200	opensea	TSS200-ope			
cg1705982	0.101441	0.522537	2.904882	0.010672	0.224708	-3.91491	0.573257	0.471816	-0.10144	15	39933961	R	II	FSIP1	Body	opensea	Body-open		rs2898686	50
cg2513493	0.103884	0.364528	2.663357	0.017435	0.225076	-4.38862	0.41647	0.312585	-0.10388	3	1.59E+08	R	II		IGR	opensea	IGR-opense			
cg1656616	0.10144	0.632661	2.795992	0.013327	0.224708	-4.13013	0.683381	0.581941	-0.10144	4	1.54E+08	R	II	FHDC1	Body	shelf	Body-shelf	chr4:1538	rs5719589	8
cg0361979	0.149856	0.530012	2.661832	0.017489	0.225076	-4.39157	0.604941	0.455084	-0.14986	2	2.35E+08	R	II		IGR	opensea	IGR-opense		rs4616477	24;1
cg2692858	0.102465	0.599816	2.661645	0.017495	0.225076	-4.39193	0.651048	0.548583	-0.10246	7	25591490	F	II		IGR	opensea	IGR-opense			
cg1543818	0.101435	0.398588	3.145375	0.006508	0.224708	-3.43176	0.449306	0.347871	-0.10143	3	1.24E+08	F	II	KALRN	Body	opensea	Body-open			
cg0475802	0.102205	0.48671	2.659819	0.01756	0.225076	-4.39546	0.537812	0.435607	-0.10221	2	1.52E+08	R	II		IGR	opensea	IGR-opense		rs5318671	49;24;2
cg1570487	0.101432	0.484208	2.820186	0.012687	0.224708	-4.08253	0.534924	0.433492	-0.10143	2	1.03E+08	R	II	SLC9A2	Body	shelf	Body-shelf	chr2:1032	rs1833148	16;1
cg0224285	0.101411	0.569011	2.736243	0.015047	0.22478	-4.24711	0.619716	0.518305	-0.10141	10	13768464	F	II	FRMD4A	Body	opensea	Body-open			
cg0546244	0.102601	0.453091	2.657968	0.017626	0.225076	-4.39903	0.504391	0.40179	-0.1026	16	77574072	F	II		IGR	opensea	IGR-opense		rs1919234	7
cg0161491	0.101404	0.605997	3.223353	0.005539	0.224708	-3.27323	0.656699	0.555296	-0.1014	1	2.02E+08	R	II	LGR6	Body	opensea	Body-open			
cg2652874	0.10138	0.505886	3.041764	0.008058	0.224708	-3.64108	0.556576	0.455196	-0.10138	10	75240045	R	II	PPP3CB	5'UTR	opensea	5'UTR-open		rs5349954	44;38
cg1713688	0.101361	0.509264	3.315913	0.004573	0.224708	-3.08413	0.559944	0.458583	-0.10136	9	682998	F	II	KANK1	Body	opensea	Body-open		rs1850826	23;26
cg2698671	0.101353	0.543463	2.782044	0.013711	0.224708	-4.15752	0.59414	0.492786	-0.10135	3	1.22E+08	R	II	ILDR1	Body	opensea	Body-open		rs5571512	2
cg2217504	0.101346	0.438235	2.456604	0.026358	0.229509	-4.78198	0.488908	0.387562	-0.10135	7	37779764	R	II	GPR141	TSS1500	opensea	TSS1500-o		rs5422985	42;14;12;2
cg0374820	0.101345	0.55205	3.174338	0.00613	0.224708	-3.37297	0.602722	0.501377	-0.10134	1	35811540	R	II	ZMYM4	Body	opensea	Body-open		rs1115049	25;17;13
cg2747935	0.102095	0.553268	2.652567	0.017818	0.225076	-4.40946	0.604315	0.50222	-0.1021	8	19969536	F	II		IGR	opensea	IGR-opense		rs5762644	5
cg2179041	0.114367	0.470794	2.652474	0.017822	0.225076	-4.40963	0.527978	0.413611	-0.11437	5	12862067	F	II		IGR	opensea	IGR-opense		rs1412505	1
cg1270932	0.105861	0.593396	2.652113	0.017835	0.225076	-4.41033	0.646327	0.540466	-0.10586	13	49465897	R	II		IGR	opensea	IGR-opense		rs3469983	51;27;1
cg2436679	0.101536	0.513052	2.650693	0.017886	0.225076	-4.41307	0.563819	0.462126	-0.10154	17	55884705	F	II		IGR	opensea	IGR-opense		rs5328610	10;40;49
cg1316645	0.101324	0.706533	2.708997	0.0159	0.224896	-4.30017	0.757195	0.655871	-0.10132	20	46369046	F	II	SULF2	Body	opensea	Body-open		rs5300245	1;49
cg2125094	0.101313	0.530661	2.7284	0.015288	0.224795	-4.2624	0.581318	0.480005	-0.10131	3	24181646	F	II	THRB	Body	opensea	Body-open		rs5681678	26
cg1065588	0.10131	0.46287	3.426813	0.003633	0.224708	-2.85644	0.513525	0.412215	-0.10131	20	5824213	F	II	C20orf196	Body	opensea	Body-open			
cg0340877	0.1013	0.542636	3.060617	0.007751	0.224708	-3.60311	0.593286	0.491986	-0.1013	2	1.44E+08	R	II	ARHGAP15	Body	opensea	Body-open		rs5766398	15;13
cg0145984	0.101298	0.351753	2.408003	0.029013	0.231604	-4.8725	0.402402	0.301104	-0.1013	3	69255721	R	II	FRMD4B	Body	opensea	Body-open		rs5495465	51;26;17
cg1838765	0.101291	0.783297	4.322107	0.000573	0.224708	-1.00297	0.833943	0.732651	-0.10129	10	1276341	F	II	ADARB2	Body	opensea	Body-open		rs1137448	11
cg2093723	0.100736	0.668101	2.644352	0.018116	0.225076	-4.42529	0.718469	0.617733	-0.10074	20	725968	F	II		IGR	opensea	IGR-opense		rs5404883	1;6;13;23;30
cg1452657	0.101289	0.653315	2.722935	0.015458	0.224896	-4.27305	0.703959	0.60267	-0.10129	7	1.39E+08	F	II	HIPK2	Body	opensea	Body-open		rs5480981	27
cg1551248	0.101289	0.679345	3.210671	0.005686	0.224708	-3.29906	0.72999	0.628701	-0.10129	2	1.11E+08	R	II	NPHP1	Body	opensea	Body-open		rs5320925	2;1
cg1899876	0.101269	0.454939	2.670522	0.017185	0.224959	-4.37477	0.505573	0.404304	-0.10127	7	75224305	R	II	HIP1	Body	opensea	Body-open		rs3742669	35;1
cg1141199	0.102921	0.487701	2.640009	0.018275	0.225076	-4.43366	0.539162	0.43624	-0.10292	10	90878257	F	II		IGR	opensea	IGR-opense		rs5370986	46
cg0794972	0.110255	0.407671	2.638758	0.018321	0.225076	-4.43607	0.462798	0.352544	-0.11025	17	32576670	F	II		IGR	opensea	IGR-opense		rs5612776	41
cg0722090	0.101269	0.57596	2.636536	0.018403	0.225076	-4.44035	0.626595	0.525326	-0.10127	3	46973284	F	II	CCDC12	Body	opensea	Body-open		rs5392739	21;36
cg1571468	0.101258	0.64568	3.231111	0.005451	0.224708	-3.25742	0.696309	0.595051	-0.10126	17	39217766	R	II	LOC73075	TSS1500	opensea	TSS1500-o			
cg2053821	0.101673	0.589968	2.638236	0.01834	0.225076	-4.43708	0.640804	0.539132	-0.10167	4	58027287	R	II		IGR	shelf	IGR-shelf	chr4:5802	rs1493992	47;46;35;2
cg1223222	0.101243	0.57502	2.386694	0.030255	0.232825	-4.91193	0.625641	0.524398	-0.10124	13	98825268	F	II	FARP1	5'UTR	shelf	5'UTR-shelf	chr13:988	rs7410869	0;1;2;51
cg2451142	0.109159	0.499962	2.634139	0.018492	0.225084	-4.44496	0.554541	0.445382	-0.10916	7	1.11E+08	R	II		IGR	opensea	IGR-opense		rs5753398	12
cg0034079	0.101243	0.582325	2.82597	0.012538	0.224708	-4.07113	0.632947	0.531704	-0.10124	2	2.16E+08	F	II	LINC00607	Body	opensea	Body-open			

cg0806734	0.101231	0.513597	2.467223	0.025809	0.228941	-4.76209	0.564212	0.462981	-0.10123	12	1.02E+08	F	II	GNPTAB	Body	opensea	Body-opens		rs3755020	0
cg1323720	0.113269	0.481125	2.633273	0.018524	0.225084	-4.44663	0.53776	0.424491	-0.11327	13	98192250	F	II		IGR	opensea	IGR-opense		rs5746394	1
cg0112073	0.110685	0.460548	2.633086	0.018531	0.225084	-4.44699	0.515891	0.405206	-0.11068	19	19080651	R	II		IGR	opensea	IGR-opense		rs1464859	41;27
cg1458016	0.101075	0.586943	2.632665	0.018547	0.225084	-4.4478	0.63748	0.536405	-0.10108	12	26286940	R	II		IGR	opensea	IGR-opense			
cg1772759	0.101252	0.439248	2.630914	0.018612	0.225119	-4.45117	0.489874	0.388622	-0.10125	5	1.18E+08	F	II		IGR	opensea	IGR-opense			
cg0849143	0.101231	0.522569	2.739985	0.014933	0.224708	-4.23981	0.573184	0.471953	-0.10123	1	78442533	R	II	FUBP1	Body	shore	Body-shore	chr1:7844		
cg0589080	0.100081	0.360134	2.630396	0.018632	0.225119	-4.45216	0.410174	0.310093	-0.10008	2	20716912	R	II		IGR	opensea	IGR-opense		rs5380727	21;6;4
cg0877964	0.29278	0.563747	2.627868	0.018727	0.225209	-4.45702	0.710137	0.417357	-0.29278	13	50194554	R	II		IGR	opensea	IGR-opense			
cg2592281	0.101224	0.584984	2.823776	0.012594	0.224708	-4.07546	0.635596	0.534373	-0.10122	7	1.3E+08	F	II	TMEM209	Body	opensea	Body-opens		rs1496216	10
cg0572974	0.115254	0.648773	2.626982	0.01876	0.225209	-4.45873	0.7064	0.591146	-0.11525	22	49937644	R	II		IGR	shelf	IGR-shelf	chr22:499		
cg0976898	0.159952	0.721465	2.62681	0.018766	0.225231	-4.45906	0.801441	0.641488	-0.15995	4	1.84E+08	R	II		IGR	opensea	IGR-opense		rs1892364	22
cg2132162	0.100299	0.504496	2.626299	0.018786	0.225286	-4.46004	0.554645	0.454346	-0.1003	15	68325193	F	II		IGR	opensea	IGR-opense		rs5573970	1;31;33
cg2110886	0.101218	0.665705	3.368052	0.004104	0.224708	-2.97722	0.716313	0.615096	-0.10122	7	28852950	F	II	CREB5	Body	opensea	Body-opens		rs1892503	0;39
cg0216827	0.266366	0.550099	2.620749	0.018996	0.225391	-4.4707	0.683282	0.416916	-0.26637	8	50110978	F	II		IGR	opensea	IGR-opense		rs5757957	0;1
cg0067293	0.101195	0.643677	3.656236	0.002257	0.224708	-2.38267	0.694275	0.59308	-0.1012	8	1.31E+08	F	II	CCDC26	Body	opensea	Body-opens		rs5291254	22;39
cg2420790	0.101192	0.812313	2.231246	0.040951	0.243912	-5.19453	0.862909	0.761717	-0.10119	11	74205489	F	II	LIPT2	TSS1500	shore	TSS1500-sh	chr11:742(	rs1783201	8;9;27
cg1505818	0.101188	0.476612	2.677683	0.016939	0.224901	-4.36091	0.527207	0.426018	-0.10119	2	60715593	F	II	BCL11A	Body	opensea	Body-opens		rs5776203	4
cg1231914	0.168948	0.677827	2.616941	0.019142	0.225391	-4.47801	0.762301	0.593353	-0.16895	10	1.35E+08	F	II		IGR	shelf	IGR-shelf	chr10:134	rs1453979	30
cg1022614	0.102205	0.433836	2.6166	0.019156	0.225391	-4.47867	0.484939	0.382734	-0.10221	12	76036209	R	II		IGR	opensea	IGR-opense			
cg0022658	0.103506	0.506939	2.613428	0.019278	0.225391	-4.48476	0.558692	0.455186	-0.10351	5	89000691	F	II		IGR	opensea	IGR-opense		rs5614567	1
cg1263259	0.101177	0.613758	4.70377	0.000265	0.224708	-0.2198	0.664347	0.56317	-0.10118	2	88899965	R	II	EIF2AK3	Body	opensea	Body-opens			
cg1154815	0.10116	0.585349	3.441918	0.003521	0.224708	-2.82535	0.635929	0.534769	-0.10116	2	2.02E+08	R	II	AOX2P	Body	opensea	Body-opens		rs1492170	50;13;4;1
cg0323036	0.109724	0.527889	2.607784	0.019498	0.225391	-4.49558	0.582751	0.473027	-0.10972	18	55681687	R	II		IGR	opensea	IGR-opense		rs1848742	45;41;35
cg0241035	0.10113	0.737743	2.743105	0.014839	0.224708	-4.23372	0.788308	0.687178	-0.10113	9	5438883	R	II	PLGRKT	TSS1500	shore	TSS1500-sh	chr9:5437	rs702277;r	33;30;28;19;4
cg0669018	0.101121	0.685581	2.937666	0.009979	0.224708	-3.84963	0.736142	0.63502	-0.10112	18	77960781	F	II	PARD6G	Body	opensea	Body-opens		rs1130568	27
cg0871230	0.157009	0.644967	2.604685	0.019619	0.225391	-4.50152	0.723471	0.566462	-0.15701	20	42417112	R	II		IGR	opensea	IGR-opense		rs5416046	44;6
cg2592925	0.101095	0.512154	2.791818	0.013441	0.224708	-4.13833	0.562702	0.461606	-0.1011	4	36211731	F	II	ARAP2	Body	opensea	Body-opens		rs3679273	0;1;36
cg1913932	0.101925	0.539212	2.601536	0.019744	0.225445	-4.50755	0.590175	0.48825	-0.10193	2	2.18E+08	R	II		IGR	opensea	IGR-opense			
cg1672955	0.10107	0.522926	2.805676	0.013067	0.224708	-4.11109	0.573461	0.47239	-0.10107	1	2.07E+08	R	II	FCAMR	5'UTR	opensea	5'UTR-open		rs1510587	31;21;2
cg0567350	0.101529	0.555746	2.600909	0.019769	0.225463	-4.50875	0.606511	0.504982	-0.10153	14	97176849	F	II		IGR	opensea	IGR-opense			
cg0767689	0.101063	0.501786	2.756433	0.014443	0.224708	-4.20768	0.552318	0.451255	-0.10106	19	5213590	R	II	PTPRS	Body	shore	Body-shore	chr19:521	rs5538257	43
cg0129419	0.101023	0.456659	3.692509	0.002094	0.224708	-2.30754	0.50717	0.406147	-0.10102	1	71368022	F	II	PTGER3	3'UTR	opensea	3'UTR-open		rs1161229	45
cg1518416	0.101019	0.517374	2.591344	0.020151	0.225654	-4.52706	0.567884	0.466865	-0.10102	18	13019478	R	II	CEP192	Body	opensea	Body-opens		rs1865096	47
cg1388508	0.101017	0.525779	3.393584	0.003893	0.224708	-2.92477	0.576288	0.475271	-0.10102	22	42823184	F	II	NFAM1	Body	opensea	Body-opens			
cg0816676	0.10101	0.483445	4.050361	0.001	0.224708	-1.56529	0.53395	0.432939	-0.10101	1	1.53E+08	R	II	LCE2A	5'UTR	opensea	5'UTR-open			
cg0808431	0.10107	0.689662	2.593252	0.020074	0.225603	-4.52341	0.740197	0.639128	-0.10107	8	1.27E+08	R	II		IGR	opensea	IGR-opense		rs5338644	48;11;4
cg1282317	0.101003	0.602835	3.250995	0.005231	0.224708	-3.21686	0.653337	0.552334	-0.101	16	78930322	R	II	WWOX	Body	opensea	Body-opens		rs5395586	37;32;26;17
cg0721548	0.109002	0.513857	2.59166	0.020139	0.225654	-4.52645	0.568358	0.459356	-0.109	1	1.52E+08	R	II		IGR	opensea	IGR-opense		rs5735933	26;14
cg2191352	0.101001	0.609697	4.691867	0.000272	0.224708	-0.24406	0.660198	0.559197	-0.101	1	1.85E+08	R	II	IVNS1ABP	5'UTR	shelf	5'UTR-shelf	chr1:1852	rs1808368	2;1
cg1930925	0.141452	0.362842	2.582546	0.02051	0.225654	-4.54387	0.433568	0.292116	-0.14145	2	9795717	R	II		IGR	opensea	IGR-opense		rs1451042	40;38;32;31;8
cg2157010	0.101708	0.476279	2.580793	0.020582	0.225654	-4.54722	0.527133	0.425425	-0.10171	1	1.79E+08	F	II		IGR	opensea	IGR-opense		rs1407135	0;2;18
cg0780769	0.100993	0.462343	3.675699	0.002168	0.224708	-2.34236	0.51284	0.411846	-0.10099	9	75406192	R	II	TMC1	Body	opensea	Body-opens		rs5571643	3
cg1735187	0.100985	0.431984	3.592304	0.002578	0.224708	-2.51498	0.482477	0.381491	-0.10099	1	2.05E+08	F	II	TMCC2	Body	opensea	Body-opens		rs5688866	49
cg1036203	0.100984	0.482799	4.291243	0.00061	0.224708	-1.06669	0.533291	0.432307	-0.10098	10	1.17E+08	R	II	FAM160B1	Body	opensea	Body-opens			
cg1079838	0.100973	0.485105	3.007733	0.008642	0.224708	-3.70947	0.535592	0.434619	-0.10097	17	10325221	R	II	MYH8	5'UTR	opensea	5'UTR-open			
cg0328892	0.107613	0.680045	2.577696	0.02071	0.225654	-4.55313	0.733851	0.626239	-0.10761	2	1.19E+08	R	II		IGR	shore	IGR-shore	chr2:1186	rs7519341	40;5
cg1199606	0.100971	0.693424	3.203828	0.005767	0.224708	-3.313	0.743909	0.642938	-0.10097	9	93635561	R	II	SYK	Body	opensea	Body-opens		rs3749133	1
cg1940605	0.100954	0.707149	3.040216	0.008083	0.224708	-3.64419	0.757626	0.656672	-0.10095	6	42204675	R	II	TRERF1	Body	opensea	Body-opens		rs9462785	31;2;1
cg0519850	0.100953	0.424307	3.177119	0.006095	0.224708	-3.36732	0.474784	0.373831	-0.10095	5	1.39E+08	R	II	SPATA24	Body	opensea	Body-opens		rs7891733	37;25;1
cg0562520	0.103701	0.674699	2.572632	0.020921	0.225654	-4.56279	0.72655	0.622849	-0.1037	6	80191740	R	II		IGR	opensea	IGR-opense		rs1499801	31;19;4;1;1
cg1202896	0.108199	0.432136	2.570231	0.021022	0.225654	-4.56737	0.486236	0.378036	-0.1082	11	34393189	R	II		IGR	opensea	IGR-opense		rs3977761	42;1
cg1359041	0.100946	0.498401	2.761328	0.0143	0.224708	-4.1981	0.548874	0.447928	-0.10095	20	43209782	R	II	PKIG	TSS1500	opensea	TSS1500-op		rs5624246	40;1
cg2485386	0.207165	0.592556	2.568854	0.02108	0.225654	-4.56999	0.696138	0.488973	-0.20716	1	1.47E+08	F	II		IGR	shore	IGR-shore	chr1:1465	rs5625739	18;36
cg1007086	0.13694	0.702785	2.568366	0.0211	0.225654	-4.57092	0.771255	0.634314	-0.13694	15	57664490	R	II		IGR	shelf	IGR-shelf	chr15:576	rs5737334	51;2;1
cg1560868	0.100931	0.715742	3.683611	0.002133	0.224708	-2.32597	0.766207	0.665277	-0.10093	1	2.04E+08	R	II	SNRPE	TSS1500	shore	TSS1500-sh	chr1:2038	rs5483193	45;25
cg2534275	0.107942	0.548983	2.567382	0.021142	0.225654	-4.57279	0.602954	0.495012	-0.10794	16	11473669	F	II		IGR	opensea	IGR-opense		rs5380570	0;13;15;38;39
cg0098006	0.117024	0.399824	2.564925	0.021246	0.225654	-4.57747	0.458336	0.341312	-0.11702	1	14849511	F	II		IGR	opensea	IGR-opense		rs1152782	0;39;48
cg1607080	0.100717	0.64759	2.562008	0.02137	0.225654	-4.58303	0.697949	0.597232	-0.10072	17	40196866	F	II		IGR	opensea	IGR-opense			
cg1334023	0.100226	0.594892	2.56125	0.021403	0.225654	-4.58447	0.645005	0.544779	-0.10023	8	81549775	R	II		IGR	opensea	IGR-opense		rs5395290	2
cg1107944	0.100904	0.513582	3.15798	0.00634	0.224708	-3.40618	0.564034	0.46313	-0.1009	8	1.31E+08	F	II	ASAP1	5'UTR	opensea	5'UTR-open		rs5293081	15;30;37;40
cg2152077	0.100059	0.730212	2.560697	0.021427	0.225654	-4.58552	0.780242	0.680183	-0.10006	12	58736428	R	II		IGR	opensea	IGR-opense		rs1398809	40;35;1
cg0445819	0.100901	0.582994	2.759273	0.01436	0.224708	-4.20212	0.633444	0.532543	-0.1009	1	2.2E+08	R	II	RAB3GAP2	Body	opensea	Body-opens		rs1399811	12
cg1897604	0.105701	0.488389	2.557553	0.021562	0.225744	-4.59151	0.54124	0.435538	-0.1057	13	24520049	F	I		IGR	opensea	IGR-opense		rs5713123	6;22;48
cg0457686	0.100885	0.583275	2.89315	0.010931	0.224708	-3.93821	0.633717	0.532832	-0.10088	2	1.45E+08	R	II	GTDC1	5'UTR	opensea	5'UTR-open		rs5588044	18;4;2;1

cg0154910	0.126359	0.741533	2.556397	0.021612	0.225763	-4.59371	0.804713	0.678354	-0.12636	5	10788719	R	II		IGR	opensea	IGR-opense		rs1142217	1
cg0787921	0.100884	0.593696	3.008836	0.008622	0.224708	-3.70725	0.644138	0.543254	-0.10088	14	63749933	F	II	RHOJ	ExonBnd	opensea	ExonBnd-o		rs5396262	16;21;22
cg2731813	0.101982	0.829595	2.555576	0.021647	0.225763	-4.59527	0.880586	0.778604	-0.10198	4	1.87E+08	F	II		IGR	opensea	IGR-opense		rs2603733	40;41
cg1816074	0.100879	0.647887	3.003215	0.008723	0.224708	-3.71853	0.698327	0.597447	-0.10088	3	1.51E+08	F	II	MED12L	Body	opensea	Body-opens		rs3718257	1;20;23;49
cg2714150	0.101959	0.393138	2.553497	0.021737	0.225763	-4.59922	0.444117	0.342159	-0.10196	10	5886111	R	II		IGR	shelf	IGR-shelf	chr10:588		
cg1807107	0.100877	0.51104	2.980788	0.009134	0.224708	-3.76347	0.561479	0.460602	-0.10088	16	78930307	R	II	WWOX	Body	opensea	Body-opens		rs5293225	45;44;37;22;17;11;2
cg0787438	0.103698	0.557075	2.547794	0.021986	0.225908	-4.61006	0.608924	0.505227	-0.1037	5	1.23E+08	F	II		IGR	opensea	IGR-opense		rs5399479	17
cg1973876	0.100864	0.636595	2.60676	0.019538	0.225391	-4.49754	0.687027	0.586163	-0.10086	21	47268725	R	II	PCBP3	TSS1500	opensea	TSS1500-o			
cg0342850	0.100023	0.613429	2.54595	0.022067	0.226008	-4.61356	0.663441	0.563418	-0.10002	1	2.33E+08	F	II		IGR	opensea	IGR-opense		rs1819431	0;19
cg1749759	0.100858	0.524466	2.620212	0.019017	0.225391	-4.47173	0.574895	0.474038	-0.10086	3	1.55E+08	F	II	LOC10050	TSS200	opensea	TSS200-ope		rs5758219	0
cg2582278	0.110859	0.802376	2.545142	0.022103	0.226027	-4.61509	0.857806	0.746947	-0.11086	12	1.33E+08	R	II		IGR	island	IGR-island	chr12:133	rs5499242	8;1
cg1081194	0.100848	0.601766	3.686173	0.002122	0.224708	-2.32067	0.65219	0.551342	-0.10085	1	1.57E+08	R	II	SH2D2A	Body	shore	Body-shore	chr1:1567	rs3773306	50;2
cg0332694	0.100825	0.619125	2.978876	0.00917	0.224708	-3.7673	0.669538	0.568712	-0.10083	9	1E+08	F	II	LOC28635	TSS1500	opensea	TSS1500-o		rs5340745	1;5;45
cg0242385	0.104406	0.402841	2.541044	0.022284	0.226234	-4.62287	0.455044	0.350637	-0.10441	10	33795998	F	II		IGR	opensea	IGR-opense		rs1921992	0;42
cg2134803	0.100822	0.578623	2.536372	0.022493	0.226384	-4.63173	0.629034	0.528212	-0.10082	5	1.29E+08	F	II	ADAMTS19	Body	opensea	Body-opens		rs1494714	0;21
cg0753166	0.100813	0.263162	3.797623	0.001684	0.224708	-2.08961	0.313569	0.212755	-0.10081	8	47751494	R	II	BEYLA	TSS1500	opensea	TSS1500-o		rs1451385	48;32;12;2
cg1131128	0.100802	0.677059	3.434103	0.003579	0.224708	-2.84144	0.72746	0.626658	-0.1008	12	63088234	F	II	PPM1H	Body	opensea	Body-opens		rs5726652	0
cg1409115	0.100783	0.546666	2.865751	0.011561	0.224708	-3.99254	0.597058	0.496275	-0.10078	4	79358764	F	II	FRAS1	Body	opensea	Body-opens		rs5445028	33;44
cg0815381	0.100766	0.402795	2.899864	0.010782	0.224708	-3.92488	0.453178	0.352413	-0.10077	20	50021369	F	II	NFATC2	Body	opensea	Body-opens		rs1932450	8;10;13;28;36
cg1735734	0.104518	0.414499	2.532149	0.022683	0.226517	-4.63974	0.466758	0.362239	-0.10452	2	58603854	F	II		IGR	opensea	IGR-opense		rs5549182	0;1;29
cg1862896	0.106621	0.512684	2.53161	0.022708	0.226534	-4.64076	0.565994	0.459373	-0.10662	12	43719927	R	II		IGR	opensea	IGR-opense		rs1482674	51
cg0537329	0.102231	0.569564	2.526826	0.022926	0.226596	-4.64982	0.62068	0.518449	-0.10223	1	1.9E+08	F	II		IGR	opensea	IGR-opense		rs7585728	0;14;18
cg2452639	0.100742	0.733124	2.592204	0.020117	0.225654	-4.52541	0.783495	0.682753	-0.10074	15	63128725	F	II	TLN2	Body	opensea	Body-opens		rs1140471	0;11;13;17;40;47
cg1326640	0.141269	0.424867	2.523664	0.02307	0.226614	-4.6558	0.495502	0.354233	-0.14127	3	89627094	R	II		IGR	opensea	IGR-opense		rs5682984	51;27;1
cg1015870	0.118171	0.541726	2.520509	0.023216	0.226759	-4.66177	0.600811	0.48264	-0.11817	5	1.16E+08	F	II		IGR	opensea	IGR-opense		rs5320644	1;44
cg0872787	0.10072	0.516197	2.961507	0.009503	0.224708	-3.80204	0.566557	0.465837	-0.10072	17	6427324	R	II	PITPNM3	Body	opensea	Body-opens		rs5352479	47;29
cg2126206	0.100708	0.4772	3.956694	0.001213	0.224708	-1.75957	0.527554	0.426846	-0.10071	20	1130995	F	II	PSMF1	Body	opensea	Body-opens		rs1381294	1;34
cg0663547	0.114183	0.661967	2.510468	0.023685	0.22709	-4.68075	0.719059	0.604875	-0.11418	5	1.24E+08	F	II		IGR	opensea	IGR-opense		rs1147127	0;1;20
cg0248820	0.100701	0.468865	2.458901	0.026238	0.229342	-4.77768	0.519216	0.418515	-0.1007	7	1.32E+08	F	II	PLXNA4	Body	opensea	Body-opens		rs7530553	0;19;38
cg0365105	0.222807	0.670048	2.509038	0.023752	0.22717	-4.68345	0.781451	0.558644	-0.22281	13	50194643	F	II		IGR	opensea	IGR-opense		rs5587308	44
cg0068711	0.100538	0.543734	2.508787	0.023764	0.22719	-4.68392	0.594002	0.493465	-0.10054	15	76129654	F	II		IGR	opensea	IGR-opense		rs1458165	1;3;23;24
cg2568239	0.100677	0.689035	3.947965	0.001235	0.224708	-1.77768	0.739374	0.638697	-0.10068	15	25137649	R	II	SNRPN	5'UTR	opensea	5'UTR-open		rs1155111	14;1
cg0238931	0.100946	0.489343	2.507312	0.023834	0.227252	-4.6867	0.539816	0.43887	-0.10095	12	76577488	F	II		IGR	opensea	IGR-opense		rs3697068	1
cg0850513	0.100664	0.572454	3.201002	0.005801	0.224708	-3.31875	0.622786	0.522122	-0.10066	1	2.05E+08	F	II	LEMD1	1stExon	opensea	1stExon-op			
cg0005560	0.124068	0.606674	2.503511	0.024015	0.227384	-4.69387	0.668708	0.54464	-0.12407	1	48508839	R	II		IGR	opensea	IGR-opense		rs5578441	33
cg1559208	0.100662	0.511728	2.8856	0.011101	0.224708	-3.9532	0.562059	0.461397	-0.10066	16	49683803	R	II	ZNF423	Body	opensea	Body-opens		rs7593493	37;25
cg0118993	0.100661	0.615387	2.579198	0.020648	0.225755	-4.55026	0.665717	0.565056	-0.10066	13	43385958	R	II	LINC01050	ExonBnd	opensea	ExonBnd-o		rs1919289	45;24;1
cg0136493	0.101963	0.471665	2.501241	0.024124	0.227447	-4.69815	0.522647	0.420684	-0.10196	16	73185687	R	II		IGR	opensea	IGR-opense		rs5382053	39;35;1
cg2593923	0.100657	0.463549	3.164288	0.006258	0.224708	-3.39338	0.513877	0.41322	-0.10066	18	6967140	R	II	LAMA1	Body	opensea	Body-opens			
cg0807655	0.100651	0.596584	2.822924	0.012616	0.224708	-4.07714	0.64691	0.546258	-0.10065	3	1.75E+08	F	II	NAALADL2-	Body	opensea	Body-opens		rs5411959	0
cg0802317	0.130529	0.661275	2.495213	0.024414	0.227653	-4.70951	0.726539	0.59601	-0.13053	19	32217882	R	II		IGR	opensea	IGR-opense		rs5532685	49;33;17
cg1590897	0.106833	0.606748	2.493396	0.024503	0.227809	-4.71293	0.660165	0.553332	-0.10683	1	2.1E+08	F	II		IGR	shelf	IGR-shelf	chr1:2099	rs1831958	50
cg2464725	0.10064	0.578538	3.443314	0.003511	0.224708	-2.82247	0.628858	0.528218	-0.10064	7	94997769	F	II	PON3	Body	opensea	Body-opens		rs5822130	48
cg1212603	0.100639	0.604473	2.472264	0.025552	0.228755	-4.75264	0.654793	0.554154	-0.10064	20	33658033	F	II	TRPC4AP	Body	opensea	Body-opens		rs1841684	14;31
cg0395267	0.108226	0.510107	2.487554	0.024789	0.228087	-4.72393	0.56422	0.455994	-0.10823	21	45126620	F	II		IGR	opensea	IGR-opense		rs5581136	0;9;31;32
cg0284191	0.100633	0.445936	2.568292	0.021104	0.225654	-4.57106	0.496253	0.39562	-0.10063	6	1.53E+08	R	II	SYNE1	5'UTR	shore	5'UTR-shor	chr6:1529	rs5615733	22;19;2;1
cg2250277	0.100626	0.585449	2.778513	0.01381	0.224708	-4.16444	0.635762	0.535136	-0.10063	14	24540516	R	II	CPNE6	TSS200	opensea	TSS200-ope			
cg0641939	0.100611	0.618873	2.593865	0.02005	0.225572	-4.52223	0.669179	0.568568	-0.10061	11	59952352	F	II	MS4A6A	TSS1500	opensea	TSS1500-o		rs1835661	1;15;16;25;35;38
cg1609800	0.100604	0.72708	2.726723	0.01534	0.224831	-4.26567	0.777382	0.676778	-0.1006	9	5425397	R	II	PLGRKT	Body	opensea	Body-opens		rs1496049	47;39;29;17;14;1
cg0022217	0.100598	0.50402	2.861524	0.011661	0.224708	-4.00091	0.554319	0.453721	-0.1006	7	55584235	R	II	VOPP1	Body	opensea	Body-opens		rs1884362	26
cg0894579	0.101324	0.51828	2.477848	0.025271	0.228521	-4.74216	0.568942	0.467618	-0.10132	13	27571183	R	II		IGR	opensea	IGR-opense		rs5447545	2
cg0126496	0.100595	0.486771	2.60764	0.019503	0.225391	-4.49586	0.537069	0.436474	-0.10059	11	1.24E+08	F	II	OR10G4	TSS1500	opensea	TSS1500-o		rs1410213	1;20;26;31;38
cg2068792	0.102028	0.47973	2.476113	0.025358	0.228595	-4.74542	0.530744	0.428715	-0.10203	8	88950581	R	II		IGR	opensea	IGR-opense		rs5694518	1
cg2124141	0.115261	0.532895	2.474533	0.025438	0.228719	-4.74839	0.590526	0.475265	-0.11526	2	43267816	F	II		IGR	opensea	IGR-opense		rs5308796	9;20;42
cg2215286	0.101039	0.564608	2.473782	0.025476	0.228719	-4.7498	0.615128	0.514089	-0.10104	22	24596179	R	II		IGR	opensea	IGR-opense			
cg1776812	0.100592	0.54866	2.519242	0.023275	0.226774	-4.66417	0.598956	0.498364	-0.10059	6	56663406	R	II	DST	Body	opensea	Body-opens		rs1923125	36;30;14;1
cg1084156	0.121482	0.234001	2.472464	0.025542	0.228755	-4.75227	0.294742	0.17326	-0.12148	9	83280018	R	II		IGR	opensea	IGR-opense		rs1137906	48;42;2;1
cg1110058	0.100586	0.617867	3.056744	0.007813	0.224708	-3.61092	0.66816	0.567575	-0.10059	6	75910011	R	II	COL12A1	Body	shore	Body-shore	chr6:7591	rs601846;r	50;42;15;2
cg0982740	0.101301	0.455575	2.472073	0.025562	0.228778	-4.753	0.506225	0.404924	-0.1013	3	1.95E+08	F	II		IGR	opensea	IGR-opense		rs7788498	17;48
cg0232395	0.100582	0.538828	3.115809	0.006917	0.224708	-3.49165	0.589119	0.488537	-0.10058	8	1.31E+08	R	II	FAM49B	5'UTR	opensea	5'UTR-open		rs7455088	2;1
cg1973177	0.100567	0.498155	3.014498	0.008523	0.224708	-3.69589	0.548438	0.447871	-0.10057	11	43942574	R	II	ALKBH3-AS	TSS200	opensea	TSS200-ope		rs3497181	45
cg2701014	0.138697	0.3374	2.464886	0.025929	0.229113	-4.76647	0.406749	0.268052	-0.1387	22	43259569	F	II		IGR	opensea	IGR-opense		rs1164783	12;27;46
cg0355382	0.100778	0.579405	2.463733	0.025988	0.229144	-4.76864	0.629794	0.529016	-0.10078	3	1.74E+08	F	II		IGR	shelf	IGR-shelf	chr3:1740	rs7964296	0
cg2229688	0.100565	0.423614	2.646922	0.018022	0.225076	-4.42034	0.473897	0.373332	-0.10057	3	1.21E+08	R	II	ARGFX	5'UTR	opensea	5'UTR-open		rs5382121	49;47;22;1

cg0122137	0.100555	0.443518	2.48271	0.025028	0.228322	-4.73303	0.493795	0.39324	-0.10056	17	1263732	F	II	YWHAE	Body	opensea	Body-opens		rs7863643	27
cg1030706	0.10052	0.507334	3.164898	0.00625	0.224708	-3.39214	0.557594	0.457074	-0.10052	10	64144210	R	II	ZNF365	Body	opensea	Body-opens		rs7075904	30;2
cg2729446	0.100512	0.558112	2.767295	0.014128	0.224708	-4.18642	0.608368	0.507856	-0.10051	3	29738423	R	II	RBMS3	Body	opensea	Body-opens			
cg2268619	0.110159	0.715943	2.456184	0.02638	0.229509	-4.78276	0.771023	0.660863	-0.11016	18	68088916	R	II		IGR	opensea	IGR-opense		rs1852640	1
cg1268735	0.10051	0.572498	2.704108	0.016058	0.224896	-4.30967	0.622753	0.522243	-0.10051	3	6937063	R	II	GRM7-AS2	TSS1500	opensea	TSS1500-op		rs5580675	21
cg0874315	0.100493	0.694856	2.929575	0.010146	0.224708	-3.86576	0.745102	0.644609	-0.10049	22	36346442	F	II	RBFOX2	Body	opensea	Body-opens			
cg0555504	0.100439	0.626038	2.453743	0.026507	0.229527	-4.78733	0.676257	0.575819	-0.10044	1	68000532	R	II		IGR	opensea	IGR-opense		rs1379622	46;24;2
cg0369878	0.100491	0.306194	4.689555	0.000273	0.224708	-0.24878	0.35644	0.255949	-0.10049	1	35135423	F	II	MIR552	TSS200	opensea	TSS200-ope		rs5670512	0;14;23
cg1919298	0.174679	0.601854	2.452023	0.026598	0.229569	-4.79055	0.689194	0.514514	-0.17468	15	57664704	R	II		IGR	shelf	IGR-shelf	chr15:576(	rs5378901	40;2
cg1705267	0.1509	0.64989	2.450891	0.026657	0.229647	-4.79266	0.725339	0.57444	-0.1509	2	3827356	R	II		IGR	opensea	IGR-opense		rs5371643	2
cg0859559	0.100215	0.470116	2.447192	0.026853	0.229795	-4.79957	0.520223	0.420008	-0.10021	7	16780162	F	II		IGR	opensea	IGR-opense		rs1861773	0;45
cg2673695	0.100479	0.653403	2.678967	0.016895	0.224901	-4.35843	0.703642	0.603164	-0.10048	11	34645679	R	II	EHF	5'UTR	opensea	5'UTR-open		rs1729217	45;32;14
cg2010709	0.130699	0.898343	2.442943	0.02708	0.22984	-4.8075	0.963692	0.832994	-0.1307	1	1.73E+08	F	II		IGR	opensea	IGR-opense		rs7973936	0
cg2009824	0.100473	0.522463	4.066552	0.000967	0.224708	-1.53172	0.5727	0.472227	-0.10047	13	74702420	R	II	KLF12	5'UTR	opensea	5'UTR-open		rs5403055	1
cg0282965	0.106069	0.617275	2.439107	0.027286	0.229999	-4.81466	0.670309	0.56424	-0.10607	1	2.36E+08	R	II		IGR	opensea	IGR-opense		rs1158685	1
cg2358011	0.101195	0.48149	2.437373	0.02738	0.230067	-4.81789	0.532088	0.430893	-0.1012	12	68511699	F	II		IGR	opensea	IGR-opense		rs1460827	0
cg0713665	0.117984	0.559276	2.434067	0.027559	0.230189	-4.82405	0.618269	0.500284	-0.11798	15	39627235	F	II		IGR	opensea	IGR-opense		rs1135652	0;1
cg2191751	0.104571	0.473008	2.43342	0.027594	0.230264	-4.82526	0.525294	0.420723	-0.10457	11	85887699	F	II		IGR	opensea	IGR-opense			
cg1432062	0.100461	0.567602	4.422523	0.000467	0.224708	-0.79601	0.617833	0.517372	-0.10046	19	54384186	F	II	PRKCG	TSS1500	shore	TSS1500-sh	chr19:5438		
cg1905227	0.10046	0.770988	4.260666	0.00065	0.224708	-1.12986	0.821218	0.720758	-0.10046	2	3704530	R	I	ALLC	TSS1500	opensea	TSS1500-op		rs1468647	31;1
cg0468108	0.100456	0.530952	2.861233	0.011668	0.224708	-4.00148	0.58118	0.480724	-0.10046	18	52918079	F	II	TCF4	Body	opensea	Body-opens		rs5684479	0
cg0749443	0.100447	0.446218	4.002101	0.001104	0.224708	-1.66537	0.496441	0.395995	-0.10045	10	52287340	F	II	SGMS1	5'UTR	opensea	5'UTR-open		rs5715778	0
cg1334094	0.100429	0.411567	3.448948	0.00347	0.224708	-2.81087	0.461781	0.361352	-0.10043	12	40229749	F	II	SLC2A13	Body	opensea	Body-opens		rs5408403	4
cg1325220	0.100428	0.622388	3.156188	0.006364	0.224708	-3.40982	0.672602	0.572174	-0.10043	8	16970768	R	II	EFHA2	Body	opensea	Body-opens		rs5663466	33;7
cg1981380	0.100417	0.714054	2.703319	0.016084	0.224896	-4.3112	0.764263	0.663846	-0.10042	2	2.19E+08	F	II	DIRC3	Body	opensea	Body-opens		rs1869073	0;8
cg2282782	0.100367	0.513163	3.243625	0.005311	0.224708	-3.2319	0.563346	0.462979	-0.10037	2	1.98E+08	F	II	HSPE1	Body	shelf	Body-shelf	chr2:1983(	rs5560777	0;15;18;25
cg0385095	0.100365	0.531174	2.853341	0.011858	0.224708	-4.01709	0.581357	0.480991	-0.10037	20	16713359	F	II	SNRPB2	Body	shelf	Body-shelf	chr20:167:	rs5377098	29;47
cg1557362	0.100353	0.5768	2.720373	0.015538	0.224896	-4.27804	0.626977	0.526624	-0.10035	8	57871479	F	II	IMPAD1	3'UTR	opensea	3'UTR-open		rs1132231	1;28
cg1549354	0.100343	0.705918	3.547206	0.00283	0.224708	-2.60819	0.756089	0.655746	-0.10034	4	1.85E+08	R	II	LOC10272:	Body	opensea	Body-opens		rs5431581	42;15;1
cg0111875	0.100331	0.681314	3.221276	0.005563	0.224708	-3.27746	0.73148	0.631149	-0.10033	3	31771462	F	II	OSBPL10	Body	opensea	Body-opens		rs1414950	16
cg1248612	0.100351	0.485235	2.407314	0.029052	0.231638	-4.87378	0.53541	0.435059	-0.10035	8	1.29E+08	F	II		IGR	opensea	IGR-opense		rs7957746	1
cg0530787	0.114737	0.847473	2.40416	0.029233	0.231766	-4.87962	0.904842	0.790104	-0.11474	1	6059070	F	I		IGR	opensea	IGR-opense		rs1830352	51
cg1359723	0.100323	0.696019	3.220081	0.005577	0.224708	-3.2799	0.74618	0.645858	-0.10032	12	10458222	F	II	KLRD1	5'UTR	opensea	5'UTR-open		rs5387291	0
cg0730179	0.100314	0.391	2.712896	0.015775	0.224896	-4.29259	0.441157	0.340843	-0.10031	3	1.68E+08	F	II	LINC01330	Body	opensea	Body-opens		rs1491570	0;1;51
cg0822459	0.108862	0.557247	2.394265	0.029808	0.23241	-4.89794	0.611678	0.502816	-0.10886	16	27074124	R	II		IGR	opensea	IGR-opense		rs5380550	39;34;31;29;27;18;14
cg1709684	0.116461	0.666587	2.392857	0.029891	0.232466	-4.90054	0.724817	0.608356	-0.11646	17	41487428	F	II		IGR	opensea	IGR-opense		rs1425243	1;11;23;29;46
cg1725378	0.100311	0.646783	2.741535	0.014886	0.224574	-4.23678	0.696938	0.596628	-0.10031	8	67587258	F	II	C8orf44	TSS1500	opensea	TSS1500-op		rs5315788	0;1;3;9
cg1354600	0.10031	0.561478	2.839951	0.012186	0.224708	-4.04355	0.611633	0.511324	-0.10031	1	1.93E+08	F	II	LINC01031	Body	opensea	Body-opens			
cg2152610	0.1003	0.496658	2.83814	0.012231	0.224708	-4.04712	0.546808	0.446508	-0.1003	15	32392647	R	II	CHRNA7	Body	opensea	Body-opens			
cg1606165	0.100294	0.504426	2.761825	0.014286	0.224708	-4.19713	0.554573	0.454279	-0.10029	11	1.15E+08	R	II	CADM1	Body	opensea	Body-opens		rs7357213	51;34;11;1
cg0861205	0.100104	0.558995	2.379156	0.030707	0.233304	-4.92584	0.609047	0.508944	-0.1001	7	35595790	R	II		IGR	opensea	IGR-opense		rs5523128	45;16;11;5
cg0138587	0.101894	0.496215	2.377341	0.030816	0.233364	-4.92919	0.547162	0.445268	-0.10189	5	1.24E+08	F	II		IGR	opensea	IGR-opense		rs7378434	8
cg0329333	0.100286	0.609027	3.160588	0.006306	0.224708	-3.40089	0.65917	0.558884	-0.10029	2	42941234	R	II	MTA3	Body	opensea	Body-opens		rs1153952	46;33;31;23
cg1293355	0.111585	0.724231	2.372851	0.031089	0.233614	-4.93746	0.780023	0.668438	-0.11158	8	24052851	R	II		IGR	opensea	IGR-opense		rs5430231	44;1
cg1229485	0.100285	0.688546	2.525165	0.023002	0.226614	-4.65296	0.738689	0.638404	-0.10028	15	60818560	F	II	RORA-AS1	Body	opensea	Body-opens			
cg1793680	0.100274	0.57259	3.004096	0.008707	0.224708	-3.71676	0.622727	0.522453	-0.10027	14	52334855	F	II	GNG2	5'UTR	opensea	5'UTR-open		rs5736511	1;5
cg2204645	0.100273	0.534722	2.973986	0.009262	0.224708	-3.77709	0.584858	0.484585	-0.10027	10	99052786	R	II	ARHGAP19	TSS1500	shore	TSS1500-sh	chr10:990!	rs1401327	20;19;18;13
cg1825242	0.10027	0.621848	3.081057	0.007431	0.224708	-3.56189	0.671983	0.571713	-0.10027	3	13862475	F	II	WNT7A	Body	shore	Body-shore	chr3:1386(	rs5539726	0;20;43;49
cg0606759	0.100249	0.613652	2.975862	0.009227	0.224708	-3.77333	0.663776	0.563527	-0.10025	4	1.77E+08	R	II	GPM6A	5'UTR	shelf	5'UTR-shelf	chr4:1769:	rs5297090	2
cg0510677	0.100229	0.529808	2.712072	0.015801	0.224896	-4.29419	0.579922	0.479693	-0.10023	8	97598420	R	II	SDC2	Body	opensea	Body-opens		rs3721959	2
cg1680170	0.100225	0.426023	2.699002	0.016225	0.224896	-4.31958	0.476135	0.37591	-0.10022	5	64110063	R	II	CWC27	Body	opensea	Body-opens		rs1483034	23
cg1094096	0.127666	0.496816	2.345718	0.032786	0.234969	-4.98731	0.560649	0.432983	-0.12767	6	80176276	F	II		IGR	opensea	IGR-opense		rs1833317	1;13;29
cg2670203	0.211012	0.614177	2.34424	0.032881	0.235116	-4.99001	0.719683	0.508671	-0.21101	16	66170991	R	II		IGR	opensea	IGR-opense		rs1168228	49;41;2;1
cg1441368	0.133433	0.864206	2.339916	0.033161	0.23536	-4.99793	0.930923	0.79749	-0.13343	7	2539342	F	II		IGR	opensea	IGR-opense		rs5426617	1;3;14;28;29;47
cg1250394	0.101174	0.383853	2.339033	0.033218	0.2354	-4.99954	0.43444	0.333266	-0.10117	9	1.2E+08	F	II		IGR	opensea	IGR-opense		rs1252039	33;44
cg1156488	0.100224	0.429094	2.786811	0.013579	0.224708	-4.14816	0.479207	0.378982	-0.10022	9	71040282	R	II	PGM5	Body	opensea	Body-opens			
cg1638940	0.109119	0.642178	2.333288	0.033593	0.23574	-5.01005	0.696737	0.587618	-0.10912	1	2.44E+08	R	II		IGR	opensea	IGR-opense		rs5387943	1
cg0887603	0.109494	0.630283	2.328215	0.033927	0.236137	-5.01932	0.68503	0.575536	-0.10949	1	14342668	F	II		IGR	opensea	IGR-opense		rs1922173	0;10;27;36;50
cg1416913	0.107227	0.429487	2.326166	0.034063	0.236365	-5.02306	0.483101	0.375873	-0.10723	6	14749088	R	II		IGR	opensea	IGR-opense		rs7745856	21;2
cg0931449	0.100224	0.49088	3.483312	0.003232	0.224708	-2.74006	0.540992	0.440768	-0.10022	7	90454617	F	II	CDK14	Body	opensea	Body-opens		rs5328641	0;28
cg1964310	0.113755	0.542036	2.319841	0.034486	0.236742	-5.03459	0.598914	0.485158	-0.11376	6	1.61E+08	R	II		IGR	opensea	IGR-opense		rs5481757	7;1
cg2371951	0.132174	0.44079	2.316364	0.034721	0.236957	-5.04092	0.506877	0.374703	-0.13217	2	1.03E+08	R	II		IGR	shelf	IGR-shelf	chr2:1028(	rs5478316	48;29;17;13;2;1
cg2257639	0.100218	0.579687	2.876038	0.01132	0.224708	-3.97216	0.629796	0.529578	-0.10022	13	24042599	F	II	LINC00327	TSS1500	shore	TSS1500-sh	chr13:240		
cg1339147	0.100848	0.593496	2.311477	0.035053	0.237251	-5.04982	0.64392	0.543072	-0.10085	4	1.84E+08	R	II		IGR	opensea	IGR-opense		rs2309866	42;35;7;1

cg1945934	0.100212	0.56016	3.641873	0.002326	0.224708	-2.41241	0.610267	0.510054	-0.10021	12	2513226	R	II	CACNA1C	Body	opensea	Body-opens		rs1880294	2
cg1515424	0.123426	0.266488	2.302841	0.035647	0.237795	-5.06551	0.328201	0.204775	-0.12343	17	6656507	F	II		IGR	opensea	IGR-opense		rs7213948	8;9;33
cg2018127	0.100197	0.602333	3.186513	0.005977	0.224708	-3.34822	0.652432	0.552235	-0.1002	7	20147323	F	II	LOC10192	Body	opensea	Body-opens		rs5633761	14;18
cg2680832	0.115146	0.70119	2.295053	0.036192	0.23844	-5.07965	0.758763	0.643617	-0.11515	14	70321034	R	II		IGR	shelf	IGR-shelf	chr14:703	rs5607009	47;15;1
cg2343324	0.10018	0.485186	2.880145	0.011226	0.224708	-3.96402	0.535276	0.435096	-0.10018	2	2.17E+08	R	II	XRCC5	Body	shelf	Body-shelf	chr2:2169	rs5617097	37;35
cg1621849	0.102653	0.517023	2.291105	0.03647	0.238731	-5.0868	0.568349	0.465697	-0.10265	11	34426751	F	II		IGR	opensea	IGR-opense		rs1484368	1;12;26;28;35;46;51
cg0263872	0.100179	0.498309	2.936172	0.01001	0.224708	-3.85261	0.548398	0.44822	-0.10018	10	52154700	R	II	SGMS1	5'UTR	opensea	5'UTR-open		rs715675	49
cg1238085	0.100175	0.695401	2.681063	0.016823	0.224901	-4.35437	0.745488	0.645313	-0.10018	17	45907760	F	II	LRRC46	TSS1500	shore	TSS1500-sh	chr17:459		
cg1861800	0.100463	0.656826	2.283579	0.037007	0.239397	-5.10042	0.707058	0.606595	-0.10046	5	1.23E+08	R	II		IGR	opensea	IGR-opense		rs1851255	38
cg0713690	0.105108	0.4978	2.276081	0.03755	0.23989	-5.11397	0.550354	0.445246	-0.10511	11	71278894	F	II		IGR	opensea	IGR-opense		rs5454114	12;21
cg0409211	0.106104	0.691665	2.272002	0.037848	0.240198	-5.12133	0.744716	0.638613	-0.1061	11	58728533	F	II		IGR	shelf	IGR-shelf	chr11:587	rs1510819	0;8
cg0141840	0.100171	0.67513	3.60687	0.002501	0.224708	-2.48485	0.725216	0.625045	-0.10017	2	1.79E+08	R	II	PDE11A	Body	opensea	Body-opens		rs1436984	37;35;17;1
cg1905532	0.100154	0.445159	2.94564	0.009817	0.224708	-3.83373	0.495236	0.395082	-0.10015	17	39341434	F	II	KRTAP4-1	TSS1500	opensea	TSS1500-op		rs1244931	39
cg1525482	0.102947	0.676724	2.247566	0.039681	0.242277	-5.1653	0.728197	0.62525	-0.10295	7	564137	R	II		IGR	shore	IGR-shore	chr7:5566	rs9801466	43;40;39;1
cg1457281	0.100149	0.573677	2.792662	0.013418	0.224708	-4.13667	0.623751	0.523602	-0.10015	12	23713608	R	II	SOX5	Body	opensea	Body-opens		rs1151929	33;32;18;6
cg1894678	0.131888	0.710736	2.23815	0.04041	0.243205	-5.18218	0.77668	0.644792	-0.13189	12	1.33E+08	F	II		IGR	shore	IGR-shore	chr12:133	rs5487764	1;36;37;40
cg1081722	0.141147	0.871942	2.233489	0.040775	0.243666	-5.19052	0.942515	0.801368	-0.14115	8	1.45E+08	F	II		IGR	shore	IGR-shore	chr8:1449	rs1454361	0;8;12;26;38
cg1382045	0.100144	0.739846	2.716413	0.015663	0.224896	-4.28574	0.789918	0.689774	-0.10014	22	49970140	F	II	C22orf34	Body	opensea	Body-opens		rs1412495	51
cg0216388	0.107246	0.454836	2.226593	0.04132	0.244388	-5.20284	0.50846	0.401213	-0.10725	6	27729346	R	I		IGR	opensea	IGR-opense		rs5435538	45;28;16
cg1206492	0.100133	0.752165	3.621753	0.002425	0.224708	-2.45405	0.802232	0.702099	-0.10013	10	82115447	F	II	DYDC2	TSS1500	shore	TSS1500-sh	chr10:821		
cg1750760	0.102164	0.601518	2.215662	0.042199	0.245371	-5.22234	0.6526	0.550436	-0.10216	1	68113012	F	II		IGR	opensea	IGR-opense		rs1157257	0;5;13;15;38;46
cg0838162	0.108409	0.431924	2.214625	0.042284	0.245513	-5.22418	0.486128	0.377719	-0.10841	13	24062873	R	II		IGR	opensea	IGR-opense		rs7344900	41;40;21
cg0004479	0.108813	0.323207	2.19708	0.043734	0.247331	-5.25536	0.377613	0.2688	-0.10881	1	1.47E+08	R	I		IGR	island	IGR-island	chr1:1465	rs1833455	37;15;1
cg0676195	0.100107	0.643671	3.032117	0.008219	0.224708	-3.66048	0.693725	0.593618	-0.10011	2	47453931	R	II	LOC10192	Body	opensea	Body-opens		rs5328998	20;1
cg2653322	0.100141	0.537706	2.193544	0.044032	0.247667	-5.26163	0.587776	0.487635	-0.10014	3	1.02E+08	R	II		IGR	opensea	IGR-opense		rs4336093	17
cg0633262	0.100102	0.683925	2.574738	0.020833	0.225654	-4.55877	0.733976	0.633874	-0.1001	4	40518663	F	II	RBM47	TSS1500	opensea	TSS1500-op		rs1129080	6;44
cg1629641	0.100084	0.44907	2.782294	0.013704	0.224708	-4.15702	0.499113	0.399028	-0.10008	8	1.26E+08	F	II	NSMCE2	Body	opensea	Body-opens		rs7973453	0;1;22;29
cg0425559	0.112392	0.69877	2.189304	0.044391	0.248182	-5.26914	0.754966	0.642574	-0.11239	15	27926475	R	II		IGR	opensea	IGR-opense		rs5671078	24;2
cg1152502	0.100072	0.496825	3.137242	0.006618	0.224708	-3.44824	0.54686	0.446789	-0.10007	20	17674233	F	II	BANF2	TSS200	opensea	TSS200-ope		rs5750991	0;28;41
cg0403438	0.109044	0.620493	2.185727	0.044696	0.248582	-5.27547	0.675015	0.565971	-0.10904	2	2.36E+08	F	II		IGR	opensea	IGR-opense		rs5602524	3;14;30;48
cg0963481	0.100071	0.589097	2.977094	0.009204	0.224708	-3.77087	0.639133	0.539061	-0.10007	10	1.29E+08	R	II	DOCK1	Body	opensea	Body-opens		rs5451282	41;22;1
cg2489881	0.136633	0.287213	2.179561	0.045227	0.249201	-5.28637	0.355529	0.218896	-0.13663	10	98504248	R	II		IGR	opensea	IGR-opense		rs5389246	29;17;9
cg2286841	0.100068	0.496286	3.057258	0.007805	0.224708	-3.60988	0.54632	0.446253	-0.10007	4	15987764	F	II	PROM1	Body	opensea	Body-opens		rs7688123	43
cg2304713	0.102121	0.451251	2.169832	0.046077	0.250246	-5.30353	0.502312	0.40019	-0.10212	3	24089778	R	II		IGR	opensea	IGR-opense		rs1497249	34;29;18
cg2340240	0.100062	0.538171	3.64024	0.002334	0.224708	-2.41579	0.588202	0.48814	-0.10006	15	86651355	R	II	LINC01584	Body	opensea	Body-opens		rs5322039	41;16
cg2715759	0.116566	0.757208	2.163309	0.046655	0.251007	-5.31502	0.815491	0.698925	-0.11657	10	70974085	F	II		IGR	opensea	IGR-opense		rs1460820	0;1;25
cg1800797	0.100058	0.464132	2.870275	0.011455	0.224708	-3.98358	0.514161	0.414103	-0.10006	19	38918129	F	II	RASGRP4	TSS1500	opensea	TSS1500-op		rs1401830	0;6;26;28;34
cg0118875	0.100054	0.53237	2.807647	0.013015	0.224708	-4.10722	0.582397	0.482343	-0.10005	20	47897068	R	II	C20orf199	Body	shore	Body-shore	chr20:478		
cg0586476	0.100049	0.713181	2.782561	0.013696	0.224708	-4.1565	0.763206	0.663156	-0.10005	19	49994115	F	II	RPL13A	Body	shelf	Body-shelf	chr19:499	rs1495076	0;1;23
cg2340567	0.100016	0.5266	3.08155	0.007424	0.224708	-3.56089	0.576608	0.476592	-0.10002	20	14690830	F	II	MACROD2	Body	opensea	Body-opens		rs5441572	18;22;34
cg1984494	0.100016	0.667005	3.285311	0.004872	0.224708	-3.14675	0.717013	0.616997	-0.10002	2	2.01E+08	R	II	TYW5	Body	opensea	Body-opens			
cg0681348	0.100002	0.619946	2.769134	0.014075	0.224708	-4.18282	0.669947	0.569945	-0.1	20	20499757	R	II	RALGAPA2	Body	opensea	Body-opens		rs5620859	46;7;1

SUPPLEMENTAL TABLE 2 - MALE HYPER

	logFC	AveExpr	t	P.Value	adj.P.Val	B	Normal_m_	LBP_m_AV	deltaBeta	CHR	MAPINFO	Strand	Type	gene	feature	cgi	feat.cgi	UCSC_Islan	SNP_ID	SNP_DISTANCE
cg2609465	-0.27021	0.76799	-7.80197	1.04E-06	0.219728	5.531949	0.632885	0.903096	0.27021	2	1802045	F	II	MYT1L	Body	island	Body-islanc	chr2:18016		
cg2186235	-0.24367	0.70329	-7.42036	1.92E-06	0.236012	4.896731	0.581455	0.825126	0.243671	2	1801628	R	II	MYT1L	Body	island	Body-islanc	chr2:18016		
cg2455064	-0.22595	0.324118	-6.00285	2.24E-05	0.500061	2.364543	0.211144	0.437093	0.225949	17	30846204	R	I	MYO1D	Body	island	Body-islanc	chr17:3084	rs3734564	30
cg1182153	-0.24933	0.655578	-4.16524	0.0008	0.840963	-1.28616	0.530912	0.780243	0.249331	12	1.32E+08	R	II	ADGRD1	Body	shelf	Body-shelf	chr12:1315	rs5314475	30;15;2;1
cg2618467	-0.20318	0.683798	-4.14433	0.000835	0.840963	-1.3293	0.582209	0.785387	0.203177	12	1.32E+08	R	II	ADGRD1	Body	shelf	Body-shelf	chr12:1315	rs1439058	40;39
cg2577241	-0.21462	0.632122	-4.05707	0.000998	0.840963	-1.50948	0.524811	0.739433	0.214622	12	1.32E+08	R	II	GPR133	Body	shelf	Body-shelf	chr12:1315	rs5406165	51;25;24;9;4
cg0808954	-0.2551	0.415005	-3.96754	0.001199	0.840963	-1.69451	0.287453	0.542558	0.255105	4	1607111	R	II		IGR	opensea	IGR-opense		rs1901245	20
cg1356292	-0.21294	0.612125	-3.91707	0.00133	0.840963	-1.79884	0.505652	0.718597	0.212944	10	7294271	R	II	SFMBT2	Body	opensea	Body-opens			
cg2720775	-0.27236	0.376835	-3.81065	0.001656	0.840963	-2.01889	0.240653	0.513018	0.272365	4	1607291	R	I		IGR	opensea	IGR-opense		rs5720609	39;38;37
cg1632279	-0.21741	0.544374	-3.03254	0.008259	0.840963	-3.61123	0.435671	0.653077	0.217406	1	1.2E+08	F	II	ZNF697	3'UTR	island	3'UTR-islan	chr1:12016	rs2000748	0;1
cg0289131	-0.24202	0.615523	-2.9715	0.00936	0.840963	-3.73334	0.494513	0.736533	0.24202	5	1.8E+08	F	II	GFPT2	Body	island	Body-islanc	chr5:17974	rs1142280	11
cg2067340	-0.22808	0.628862	-2.94568	0.009868	0.840963	-3.7848	0.514821	0.742903	0.228082	10	31040939	R	II		IGR	opensea	IGR-opense		rs5273292	42;1
cg1681468	-0.32424	0.420125	-2.88217	0.011235	0.840963	-3.91087	0.258007	0.582243	0.324236	8	91681699	F	II		IGR	opensea	IGR-opense		rs5893130	1;1
cg2670892	-0.28184	0.522801	-2.80607	0.013117	0.840963	-4.06082	0.381881	0.663721	0.28184	10	13826317	R	II	FRMD4A	Body	opensea	Body-opens		rs5739272	2;1
cg2546085	-0.21144	0.374568	-2.74317	0.014901	0.840963	-4.18382	0.26885	0.480286	0.211436	1	28764551	R	II	PHACTR4	TSS200	opensea	TSS200-ope		rs3571614	1
cg1553264	-0.21135	0.471942	-2.70473	0.016105	0.840963	-4.25852	0.366266	0.577617	0.211351	12	1.3E+08	R	I		IGR	opensea	IGR-opense		rs5299542	40;37;21;1
cg1903477	-0.20396	0.549801	-2.69362	0.01647	0.840963	-4.28004	0.447822	0.651779	0.203957	8	8641253	R	II		IGR	opensea	IGR-opense		rs5659688	51;47;28;11;1
cg2596039	-0.21215	0.312479	-2.24203	0.040208	0.840963	-5.12365	0.206404	0.418554	0.21215	8	9106559	F	II		IGR	opensea	IGR-opense		rs1115425	10;24

SUPPLEMENTAL TABLE 2 - MALE HYPO

	logFC	AveExpr	t	P.Value	adj.P.Val	B	Normal_m_	LBP_m_AV(	deltaBeta	CHR	MAPINFO	Strand	Type	gene	feature	cgi	feat.cgi	UCSC_Islan	SNP_ID	SNP_DISTANCE
cg1271720	0.332595	0.285098	7.073406	3.42E-06	0.279231	4.302223	0.451396	0.118801	-0.33259	4	124342	R	II	ZNF718	Body	island	Body-islanc	chr4:1243:	rs5559851	26;21;14;7
cg2114994	0.343547	0.295445	7.054797	3.53E-06	0.279231	4.269873	0.467219	0.123672	-0.34355	4	124344	R	II	ZNF718	TSS200	island	TSS200-isla	chr4:1243:	rs5559851	28;23;16;9
cg2032049	0.239867	0.211547	7.012273	3.79E-06	0.279231	4.195777	0.331481	0.091614	-0.23987	4	124232	F	II	ZNF718	Body	shore	Body-shore	chr4:1243:	rs5337524	3;27;47;50;51
cg1019730	0.27688	0.226587	6.777837	5.66E-06	0.347198	3.782847	0.365027	0.088147	-0.27688	4	125112	F	I	ZNF718	Body	shore	Body-shore	chr4:1243:	rs5590448	6;10;13;49
cg2283172	0.292878	0.251436	6.636711	7.22E-06	0.358226	3.530648	0.397875	0.104997	-0.29288	4	124315	R	II	ZNF718	TSS200	shore	TSS200-shc	chr4:1243:	rs5767596	38;35;34
cg0538828	0.310437	0.31724	6.402704	1.09E-05	0.382016	3.106474	0.472458	0.162021	-0.31044	4	125504	R	II	ZNF718	Body	shore	Body-shore	chr4:1243:	rs1892554	46;27;16
cg1981940	0.246623	0.221757	6.35584	1.18E-05	0.382016	3.02063	0.345068	0.098445	-0.24662	4	124285	F	II	ZNF718	TSS200	shore	TSS200-shc	chr4:1243:	rs5559851	33;38;45
cg2539349	0.154538	0.625501	5.780848	3.37E-05	0.507197	1.943455	0.70277	0.548232	-0.15454	17	17109936	F	II	PLD6	TSS1500	island	TSS1500-isl	chr17:171(	rs5396205	6;10
cg2473915	0.123278	0.589749	5.667263	4.17E-05	0.58678	1.725562	0.651388	0.52811	-0.12328	14	87427652	F	II		IGR	opensea	IGR-opense		rs1508268	1;29;34
cg2029629	0.144062	0.731636	5.187328	0.000104	0.774794	0.787479	0.803667	0.659605	-0.14406	2	2.1E+08	R	II		IGR	island	IGR-island	chr2:2100:	rs7679877	46
cg0049622	0.144381	0.593219	5.12881	0.000117	0.788345	0.671286	0.665409	0.521028	-0.14438	4	1.87E+08	R	II	F11-AS1	Body	shore	Body-shore	chr4:1873!	rs5308650	6
cg0903593	0.218864	0.63687	5.077276	0.000129	0.796248	0.568654	0.746302	0.527438	-0.21886	12	1.29E+08	R	II	SLC15A4	Body	shore	Body-shore	chr12:129:	rs5426064	18;14
cg2539766	0.21181	0.259722	4.77125	0.000236	0.817445	-0.04632	0.365627	0.153817	-0.21181	4	124622	F	II	ZNF718	Body	island	Body-islanc	chr4:1243:	rs1160090	16;51
cg1315086	0.115167	0.262788	4.723083	0.000259	0.835809	-0.1439	0.320371	0.205205	-0.11517	14	22951163	R	II		IGR	opensea	IGR-opense			
cg0225000	0.140158	0.677079	4.720069	0.000261	0.835809	-0.15002	0.747157	0.607	-0.14016	6	84224986	F	II	PRSS35	5'UTR	opensea	5'UTR-oper		rs579599	13
cg1375337	0.100113	0.731204	4.705956	0.000268	0.835809	-0.17865	0.78126	0.681147	-0.10011	3	1.94E+08	R	II		IGR	opensea	IGR-opense		rs5646004	23;16
cg0143735	0.125831	0.334092	4.558954	0.00036	0.840963	-0.47786	0.397007	0.271176	-0.12583	2	1782402	F	II		IGR	opensea	IGR-opense			
cg1977837	0.129645	0.867089	4.524222	0.000386	0.840963	-0.54879	0.931911	0.802267	-0.12964	12	297831	F	I		IGR	shore	IGR-shore	chr12:298:		
cg0048115	0.128529	0.727171	4.470645	0.00043	0.840963	-0.65837	0.791435	0.662906	-0.12853	12	96353086	F	II	AMDHD1	Body	shelf	Body-shelf	chr12:963!	rs7977244	18;20;30
cg2306382	0.143324	0.396898	4.433081	0.000464	0.840963	-0.73531	0.468559	0.325236	-0.14332	12	298151	R	II		IGR	shore	IGR-shore	chr12:298:		
cg1364986	0.121186	0.711386	4.414821	0.000481	0.840963	-0.77275	0.771979	0.650793	-0.12119	9	1.01E+08	R	II	GABBR2	Body	opensea	Body-open:		rs7714936	50;30;2
cg0081746	0.473372	0.56446	4.408031	0.000488	0.840963	-0.78667	0.801146	0.327774	-0.47337	10	1.12E+08	R	II	XPNPEP1	Body	opensea	Body-open:		rs5695761	33;20
cg1652477	0.167897	0.458568	4.389306	0.000507	0.840963	-0.82509	0.542516	0.374619	-0.1679	7	52568754	F	II		IGR	opensea	IGR-opense		rs5317215	24;41;44
cg1893909	0.102524	0.223649	4.371812	0.000525	0.840963	-0.86099	0.274911	0.172387	-0.10252	16	62673412	R	II		IGR	opensea	IGR-opense		rs5703895	10
cg1909337	0.119658	0.614511	4.344966	0.000555	0.840963	-0.91613	0.67434	0.554682	-0.11966	17	17110180	F	I	PLD6	TSS1500	island	TSS1500-isl	chr17:171(	rs5547423	29;33;36
cg1241203	0.108506	0.271043	4.246089	0.000678	0.840963	-1.11953	0.325296	0.21679	-0.10851	1	1.91E+08	R	II	LOC44070:	TSS200	opensea	TSS200-ope		rs5311813	45
cg2391484	0.153824	0.401077	4.22683	0.000705	0.840963	-1.1592	0.477989	0.324165	-0.15382	1	9327170	F	II	H6PD	3'UTR	shelf	3'UTR-shelf	chr1:9324:	rs5465987	0;7;10;31
cg0669362	0.161203	0.279084	4.20649	0.000735	0.840963	-1.20112	0.359685	0.198482	-0.1612	6	1.49E+08	F	II	UST	Body	opensea	Body-open:		rs1403307	12;28
cg2628017	0.128387	0.593832	4.204618	0.000738	0.840963	-1.20498	0.658026	0.529639	-0.12839	10	1.34E+08	F	II		IGR	opensea	IGR-opense		rs1932695	7;14;19;33;36;49;50
cg0199598	0.167211	0.749133	4.188573	0.000762	0.840963	-1.23805	0.832738	0.665527	-0.16721	6	1.47E+08	R	II	STXBP5-AS:	Body	opensea	Body-open:		rs1529013	19
cg0580792	0.102939	0.601847	4.158834	0.00081	0.840963	-1.29939	0.653316	0.550377	-0.10294	9	1.23E+08	F	II		IGR	opensea	IGR-opense		rs5490284	1;21;31;49
cg2572720	0.103856	0.746069	4.153478	0.000819	0.840963	-1.31044	0.797998	0.694141	-0.10386	19	45055395	F	II	CEACAM22	Body	opensea	Body-open:		rs5274406	1;3
cg2536233	0.106208	0.150398	4.1405	0.000841	0.840963	-1.33721	0.203501	0.097294	-0.10621	2	85811413	R	II	VAMP5	TSS200	island	TSS200-isla	chr2:8581:	rs6753051	24
cg1018438	0.109528	0.274517	4.118568	0.00088	0.840963	-1.38248	0.329281	0.219754	-0.10953	4	78289079	R	II		IGR	opensea	IGR-opense		rs5434449	49;29;28;8;1
cg2084829	0.135945	0.254663	4.106264	0.000902	0.840963	-1.40788	0.322636	0.186691	-0.13594	7	1E+08	R	I	ZAN	Body	opensea	Body-open:		rs1819105	47;10
cg2158551	0.14117	0.202835	4.00471	0.001111	0.840963	-1.61767	0.27342	0.132249	-0.14117	11	1.22E+08	R	II	LOC39995!	Body	opensea	Body-open:			
cg1166346	0.109971	0.557035	4.001601	0.001118	0.840963	-1.62409	0.612021	0.50205	-0.10997	2	1.37E+08	R	II		IGR	opensea	IGR-opense		rs7261703	31;17;2
cg0827417	0.106213	0.788715	3.967505	0.001199	0.840963	-1.69457	0.841822	0.735609	-0.10621	4	1625012	F	II		IGR	shore	IGR-shore	chr4:1625:	rs1125353	0;8;23;24;45;49
cg1703715	0.104107	0.507604	3.886514	0.001416	0.840963	-1.86204	0.559658	0.455551	-0.10411	10	81106469	R	II	PPIF	TSS1500	shore	TSS1500-sh	chr10:811(		
cg2115000	0.113483	0.607918	3.878268	0.001441	0.840963	-1.87909	0.66466	0.551177	-0.11348	16	88323811	F	II		IGR	opensea	IGR-opense		rs5865777	18;26;31;32;35
cg1321789	0.239663	0.47741	3.840053	0.001559	0.840963	-1.95811	0.597242	0.357579	-0.23966	3	1.84E+08	R	II		IGR	opensea	IGR-opense		rs2582093	39
cg2457885	0.129466	0.451089	3.837315	0.001568	0.840963	-1.96377	0.515822	0.386356	-0.12947	17	17110207	F	I	PLD6	TSS1500	island	TSS1500-isl	chr17:171(	rs5547423	2;6;9;44;46;48
cg1478991	0.150892	0.721575	3.813025	0.001648	0.840963	-2.01399	0.797021	0.646129	-0.15089	21	47582049	R	II	C21orf56	Body	island	Body-islanc	chr21:475!		
cg1845404	0.148693	0.749753	3.810035	0.001658	0.840963	-2.02018	0.8241	0.675406	-0.14869	7	3644951	F	II	SDK1	Body	opensea	Body-open:		rs5653575	0;1;31;37;46
cg0747531	0.127871	0.632901	3.731666	0.001949	0.840963	-2.18216	0.696837	0.568966	-0.12787	5	54717280	R	II	SKIV2L2	Body	opensea	Body-open:		rs3716455	44
cg1307736	0.247132	0.445576	3.708512	0.002045	0.840963	-2.22999	0.569142	0.32201	-0.24713	18	34908626	F	II	BRUNOL4	Body	opensea	Body-open:		rs1838668	1;25
cg1307637	0.118976	0.562896	3.703475	0.002066	0.840963	-2.2404	0.622384	0.503408	-0.11898	8	1.03E+08	F	II	NCALD	5'UTR	opensea	5'UTR-oper		rs6999047	12
cg2701473	0.108669	0.405599	3.683016	0.002156	0.840963	-2.28265	0.459934	0.351264	-0.10867	10	19777735	F	II	MALRD1	Body	opensea	Body-open:		rs1505908	49
cg2196492	0.102927	0.58357	3.668202	0.002223	0.840963	-2.31324	0.635034	0.532106	-0.10293	5	1.54E+08	R	II		IGR	island	IGR-island	chr5:1538:	rs5877028	20
cg2525096	0.101097	0.261832	3.664638	0.002239	0.840963	-2.3206	0.312381	0.211283	-0.1011	6	11318770	R	II	NEDD9	5'UTR	opensea	5'UTR-oper		rs5715238	45;43;18;7
cg1060816	0.127794	0.538328	3.647264	0.002321	0.840963	-2.35646	0.602225	0.47443	-0.12779	9	37964630	F	II	SHB	Body	opensea	Body-open:		rs5581372	15;49
cg1564805	0.182244	0.523018	3.646272	0.002326	0.840963	-2.3585	0.61414	0.431896	-0.18224	21	23123750	F	II	LINC01425	Body	opensea	Body-open:		rs1108887	26;51
cg0918322	0.177314	0.738638	3.645501	0.002329	0.840963	-2.36009	0.827295	0.649981	-0.17731	3	1.02E+08	F	II		IGR	opensea	IGR-opense		rs5455244	5;17
cg1012052	0.190978	0.422757	3.64203	0.002346	0.840963	-2.36726	0.518246	0.327268	-0.19098	3	69891885	F	II	MITF	Body	opensea	Body-open:		rs5382921	0;16;17;36;39;40;42
cg2617200	0.122872	0.181208	3.627493	0.002418	0.840963	-2.39725	0.242644	0.119772	-0.12287	8	22941858	R	I	LOC25489(	TSS200	opensea	TSS200-ope		rs7834950	42;27
cg1229655	0.150448	0.671178	3.606029	0.002527	0.840963	-2.44152	0.746402	0.595954	-0.15045	6	32728862	R	II	HLA-DQB2	Body	shore	Body-shore	chr6:3272!	rs5515555	49
cg2293380	0.324371	0.408813	3.56697	0.00274	0.840963	-2.52204	0.570998	0.246628	-0.32437	6	32605704	R	II	HLA-DQA1	Body	opensea	Body-open:		rs9272465	51;17;10;7;1
cg2191854	0.153695	0.558219	3.544347	0.002871	0.840963	-2.56864	0.635067	0.481372	-0.15369	8	1.46E+08	F	II	ZNF251	Body	shore	Body-shore	chr8:1459!	rs5557051	4
cg0300418	0.102526	0.729573	3.535771	0.002923	0.840963	-2.58629	0.780836	0.67831	-0.10253	21	40341619	R	II		IGR	opensea	IGR-opense		rs2836791	19
cg1023980	0.105325	0.839072	3.519515	0.003023	0.840963	-2.61975	0.891735	0.78641	-0.10533	12	48498370	R	II	PFKM	TSS1500	shore	TSS1500-sh	chr12:484!	rs1116840	45;14
cg1775057	0.108177	0.631079	3.502709	0.00313	0.840963	-2.65433	0.685167	0.57699	-0.10818	10	2544120	F	II		IGR	island	IGR-island	chr10:254:	rs1127751	2;18;37
cg0037821	0.151631	0.443622	3.498858	0.003155	0.840963	-2.66225	0.519437	0.367806	-0.15163	8	1237075	F	II		IGR	opensea	IGR-opense		rs1121788	0;7;39;41;43;48
cg2136723	0.120333	0.408446	3.473634	0.003324	0.840963	-2.7141	0.468613	0.34828	-0.12033	15	75166300	F	II	SCAMP2	TSS1500	shore	TSS1500-sh	chr15:751(	rs1291125	35;49
cg2701014	0.195453	0.268618	3.470816	0.003343	0.840963	-2.7199	0.366345	0.170892	-0.19545	22	43259569	F	II		IGR	opensea	IGR-opense		rs1164783	12;27;46

cg2565150	0.102655	0.171959	3.468591	0.003359	0.840963	-2.72447	0.223286	0.120631	-0.10266	2	85812023	F	II	VAMP5	Body	shore	Body-shore	chr2:8581	rs3683016	0;37;51
cg1675033	0.145811	0.463874	3.391263	0.003941	0.840963	-2.88313	0.53678	0.390969	-0.14581	6	1.31E+08	F	II	SAMD3	Body	opensea	Body-opens		rs1807621	0;44
cg0330717	0.132953	0.700238	3.387441	0.003972	0.840963	-2.89096	0.766715	0.633762	-0.13295	3	1.96E+08	F	II		IGR	opensea	IGR-opense		rs6583331	23;34;40;41;50
cg1347380	0.154481	0.375618	3.385487	0.003989	0.840963	-2.89497	0.452859	0.298378	-0.15448	10	1.03E+08	F	II		IGR	shelf	IGR-shelf	chr10:103(		
cg1561808	0.321262	0.439402	3.376597	0.004063	0.840963	-2.91317	0.600033	0.278771	-0.32126	14	1.07E+08	R	II		IGR	opensea	IGR-opense		rs5543963	51;36;14;12;8;7;2;1
cg0520081	0.192507	0.672328	3.368321	0.004133	0.840963	-2.93012	0.768581	0.576074	-0.19251	21	47581042	F	II		IGR	island	IGR-island	chr21:4758	rs7566080	32;38
cg0002892	0.108676	0.601494	3.358465	0.004218	0.840963	-2.95029	0.655832	0.547156	-0.10868	12	52908478	F	II	KRT5	3'UTR	opensea	3'UTR-open		rs5660322	45
cg0529124	0.109279	0.78192	3.352685	0.004268	0.840963	-2.96211	0.836559	0.72728	-0.10928	2	1.43E+08	R	II	LRP1B	Body	opensea	Body-opens		rs1158884	12;1
cg0229116	0.15443	0.404082	3.317159	0.004594	0.840963	-3.03473	0.481297	0.326867	-0.15443	14	59296302	R	II		IGR	opensea	IGR-opense		rs1160490	13;12
cg1919640	0.139783	0.339812	3.304609	0.004714	0.840963	-3.06035	0.409704	0.269921	-0.13978	6	1.11E+08	R	I	DDO	Body	island	Body-islanc	chr6:1107		
cg2391191	0.124449	0.649417	3.288283	0.004876	0.840963	-3.09366	0.711641	0.587192	-0.12445	2	68095136	R	I		IGR	opensea	IGR-opense		rs1910370	41;2
cg0906292	0.22752	0.320499	3.283981	0.00492	0.840963	-3.10243	0.434259	0.206739	-0.22752	5	1.31E+08	F	II		IGR	opensea	IGR-opense		rs5299444	0;6;27;39
cg2614828	0.133532	0.237331	3.276282	0.004999	0.840963	-3.11812	0.304097	0.170565	-0.13353	8	22941854	R	II	LOC25489	TSS200	opensea	TSS200-ope		rs7834950	38;23
cg1722158	0.107033	0.538006	3.270822	0.005055	0.840963	-3.12925	0.591523	0.48449	-0.10703	15	56033606	F	II	PRTG	Body	shore	Body-shore	chr15:560		
cg0057258	0.100931	0.210324	3.270426	0.005059	0.840963	-3.13006	0.260789	0.159858	-0.10093	17	17109740	F	II	PLD6	TSS200	shore	TSS200-shc	chr17:171(	rs3721957	2;28;38;40
cg2762002	0.111978	0.405048	3.24583	0.005323	0.840963	-3.18014	0.461037	0.349059	-0.11198	14	22924469	R	II		IGR	opensea	IGR-opense		rs1387916	46;40;20
cg0403438	0.127857	0.53802	3.244933	0.005333	0.840963	-3.18196	0.601949	0.474092	-0.12786	2	2.36E+08	F	II		IGR	opensea	IGR-opense		rs5602524	3;14;30;48
cg1707392	0.124044	0.473519	3.225073	0.005556	0.840963	-3.22235	0.535541	0.411498	-0.12404	10	81106651	F	II	PPIF	TSS1500	shore	TSS1500-sh	chr10:811(	rs1693709	42
cg1312627	0.120511	0.703253	3.21172	0.005711	0.840963	-3.24948	0.763508	0.642997	-0.12051	21	47581558	F	I	C21orf56	Body	island	Body-islanc	chr21:4758	rs1485430	17;19
cg0571654	0.141141	0.760715	3.205604	0.005784	0.840963	-3.2619	0.831286	0.690144	-0.14114	17	41728055	F	II	MEOX1	Body	opensea	Body-opens			
cg2645467	0.137574	0.624412	3.19832	0.005871	0.840963	-3.27668	0.693199	0.555625	-0.13757	9	37011472	F	II	PAX5	Body	opensea	Body-opens		rs944066;r	7;13;29
cg1511378	0.126475	0.587472	3.188964	0.005986	0.840963	-3.29566	0.65071	0.524234	-0.12648	6	53821396	F	II	LOC10192	Body	opensea	Body-opens		rs5369808	0;19
cg2355282	0.109044	0.811643	3.174807	0.006163	0.840963	-3.32436	0.866165	0.757121	-0.10904	17	64272071	R	II		IGR	opensea	IGR-opense		rs1504503	46;27;2;1
cg1175497	0.11682	0.335215	3.16635	0.006271	0.840963	-3.34149	0.393625	0.276805	-0.11682	14	22927264	F	II		IGR	opensea	IGR-opense			
cg1366647	0.112269	0.826394	3.165967	0.006276	0.840963	-3.34227	0.882528	0.770259	-0.11227	1	44249486	F	II	ST3GAL3	Body	opensea	Body-opens		rs1846966	0;8;19;38
cg0122841	0.117385	0.715162	3.163525	0.006308	0.840963	-3.34721	0.773855	0.656469	-0.11739	5	493893	R	II	SLC9A3	Body	shore	Body-shore	chr5:4948(	rs5740309	47;15;6;5
cg1314462	0.102299	0.74885	3.152962	0.006447	0.840963	-3.36859	0.799999	0.6977	-0.1023	3	1.49E+08	F	II		IGR	opensea	IGR-opense		rs1413352	0;33;36;37;38;39
cg1511144	0.106494	0.231299	3.146972	0.006527	0.840963	-3.38071	0.284546	0.178052	-0.10649	8	22941856	R	II	LOC25489	TSS200	opensea	TSS200-ope		rs7834950	40;25
cg2735389	0.121647	0.410328	3.141953	0.006595	0.840963	-3.39086	0.471152	0.349505	-0.12165	3	1.95E+08	F	I	MUC4	Body	island	Body-islanc	chr3:1954	rs1995527	8;27
cg1634042	0.114148	0.525219	3.131557	0.006738	0.840963	-3.41188	0.582293	0.468145	-0.11415	17	17110120	F	I	PLD6	TSS1500	island	TSS1500-isl	chr17:171(	rs5363954	9
cg0577338	0.10408	0.348565	3.123447	0.006851	0.840963	-3.42826	0.400605	0.296525	-0.10408	14	22946935	R	II		IGR	opensea	IGR-opense		rs1830613	27
cg0847733	0.277487	0.511367	3.122248	0.006868	0.840963	-3.43068	0.65011	0.372624	-0.27749	1	1.54E+08	R	II	S100A14	TSS1500	opensea	TSS1500-op		rs5701249	11;8
cg1468012	0.115939	0.392731	3.102376	0.007155	0.840963	-3.47078	0.4507	0.334761	-0.11594	7	47185016	R	II		IGR	opensea	IGR-opense		rs5459018	3;2
cg0511037	0.107247	0.467228	3.089083	0.007353	0.840963	-3.49757	0.520851	0.413604	-0.10725	18	19862697	R	II		IGR	opensea	IGR-opense		rs5432047	33;19;2
cg2396307	0.100945	0.34605	3.06185	0.007776	0.840963	-3.55237	0.396523	0.295578	-0.10095	6	2901712	F	II	SERPINB9	5'UTR	shore	5'UTR-shor	chr6:2903	rs1467323	0;1;19
cg1506377	0.123266	0.379599	3.055322	0.007881	0.840963	-3.56549	0.441232	0.317966	-0.12327	10	86093621	R	II	CCSER2	5'UTR	opensea	5'UTR-open		rs1814435	2
cg2028281	0.105572	0.353638	3.047319	0.008012	0.840963	-3.58157	0.406424	0.300852	-0.10557	1	43423072	F	II	SLC2A1	Body	shore	Body-shore	chr1:4342	rs5520179	5
cg2043385	0.116772	0.501203	3.041753	0.008104	0.840963	-3.59274	0.559589	0.442818	-0.11677	1	2.48E+08	R	II	OR2L13	5'UTR	shore	5'UTR-shor	chr1:2481(	rs5471019	25;22
cg1921470	0.20076	0.189285	3.036072	0.008199	0.840963	-3.60414	0.289665	0.088905	-0.20076	7	3157722	F	II		IGR	opensea	IGR-opense			
cg2539410	0.122454	0.878018	3.034263	0.00823	0.840963	-3.60777	0.939245	0.816791	-0.12245	19	45563250	R	II	SFRS16	Body	shelf	Body-shelf	chr19:455(	rs1123949	10;6
cg1061631	0.133983	0.304023	3.015111	0.00856	0.840963	-3.64616	0.371014	0.237031	-0.13398	10	15468812	R	II		IGR	opensea	IGR-opense		rs1507265	12
cg1599169	0.100591	0.368408	3.006481	0.008713	0.840963	-3.66344	0.418703	0.318112	-0.10059	5	1.52E+08	R	II		IGR	opensea	IGR-opense		rs5540272	33;27;14;2;1
cg1537788	0.142063	0.452519	2.998429	0.008858	0.840963	-3.67955	0.52355	0.381487	-0.14206	21	42440661	R	II		IGR	opensea	IGR-opense		rs1172538	31
cg1478790	0.100552	0.724674	2.993074	0.008955	0.840963	-3.69026	0.77495	0.674398	-0.10055	22	43387669	F	II	PACSIN2	5'UTR	opensea	5'UTR-open		rs5404029	0;26;42;44
cg0730093	0.109626	0.731446	2.98105	0.009179	0.840963	-3.71428	0.786259	0.676633	-0.10963	9	1.11E+08	R	II		IGR	opensea	IGR-opense		rs1905715	32;2;1
cg1612970	0.110832	0.674268	2.969029	0.009408	0.840963	-3.73828	0.729685	0.618852	-0.11083	1	2.47E+08	F	II		IGR	opensea	IGR-opense		rs3689746	28;35;46
cg2536926	0.108705	0.705396	2.967085	0.009445	0.840963	-3.74216	0.759748	0.651043	-0.1087	1	46649132	R	II	TSPAN1	Body	opensea	Body-opens		rs5497439	20
cg1232119	0.120082	0.746903	2.950304	0.009775	0.840963	-3.7756	0.806944	0.686862	-0.12008	13	78550384	F	II	EDNRB	TSS1500	opensea	TSS1500-op		rs1866987	0;30
cg1888776	0.116854	0.350542	2.931126	0.010166	0.840963	-3.81376	0.408969	0.292115	-0.11685	14	22945181	F	II		IGR	opensea	IGR-opense		rs7161733	11;31
cg0225494	0.112972	0.455988	2.921449	0.010369	0.840963	-3.83299	0.512474	0.399502	-0.11297	21	40361754	F	II		IGR	opensea	IGR-opense		rs5682595	1;8;13;18;38
cg1388347	0.101993	0.695734	2.90502	0.010723	0.840963	-3.8656	0.746731	0.644738	-0.10199	9	574312	F	II	KANK1	5'UTR	opensea	5'UTR-open		rs5369874	20;44
cg0454820	0.111201	0.237843	2.871867	0.011474	0.840963	-3.93124	0.293444	0.182243	-0.1112	12	9162872	R	II	KLRG1	3'UTR	opensea	3'UTR-open			
cg0146060	0.141596	0.638833	2.869365	0.011532	0.840963	-3.93618	0.709631	0.568034	-0.1416	7	1.03E+08	R	II	LRRC17	3'UTR	opensea	3'UTR-open		rs1324249	19
cg1711904	0.122175	0.589527	2.860047	0.011753	0.840963	-3.95458	0.650615	0.52844	-0.12218	13	1.07E+08	F	II		IGR	opensea	IGR-opense		rs5748890	0;25
cg1802006	0.137974	0.390448	2.859846	0.011758	0.840963	-3.95498	0.459435	0.321461	-0.13797	13	1.15E+08	F	I	RASA3	Body	opensea	Body-opens		rs5388431	0;1;18;36
cg1199842	0.100956	0.768288	2.859633	0.011763	0.840963	-3.9554	0.818766	0.71781	-0.10096	9	72198906	R	II	APBA1	5'UTR	opensea	5'UTR-open		rs1896161	27;9
cg1848975	0.169287	0.596051	2.851967	0.011948	0.840963	-3.97053	0.680695	0.511408	-0.16929	3	1.84E+08	R	II	MAP6D1	3'UTR	opensea	3'UTR-open		rs1417264	43
cg0596822	0.145226	0.663768	2.847791	0.01205	0.840963	-3.97876	0.73638	0.591155	-0.14523	11	5770442	F	II		IGR	opensea	IGR-opense		rs5278813	25;26;27
cg0427295	0.172277	0.413323	2.842537	0.01218	0.840963	-3.98912	0.499462	0.327185	-0.17228	11	63719761	R	II	NAA40	Body	opensea	Body-opens		rs1435758	49;47;42;2;1
cg2196230	0.102151	0.317235	2.840866	0.012221	0.840963	-3.99241	0.368311	0.26616	-0.10215	17	33760819	R	II	SLFN12	TSS1500	opensea	TSS1500-op		rs1151271	5
cg0047227	0.198009	0.532897	2.809214	0.013034	0.840963	-4.05466	0.631902	0.433893	-0.19801	11	2327755	R	I	TSPAN32	Body	opensea	Body-opens		rs2237858	51;50;34;30;26;3;2
cg0653661	0.160682	0.425107	2.809087	0.013037	0.840963	-4.05491	0.505448	0.344766	-0.16068	5	1.35E+08	F	I	MIR886	TSS200	island	TSS200-isla	chr5:1354	rs5773028	7;13
cg1496664	0.126594	0.614754	2.785578	0.013674	0.840963	-4.101	0.678051	0.551456	-0.12659	8	1.26E+08	F	II	MTSS1	Body	opensea	Body-opens		rs1009051	17
cg0869157	0.10034	0.470092	2.779073	0.013856	0.840963	-4.11373	0.520262	0.419922	-0.10034	5	14668489	R	II	FAM105B	Body	shelf	Body-shelf	chr5:1466	rs1491340	50;49
cg1162271	0.102464	0.303819	2.776472	0.013929	0.840963	-4.11882	0.355052	0.252587	-0.10246	17	56655260	R	II	TEX14	Body	opensea	Body-opens		rs1886965	41;31;1
cg0312690	0.106664	0.476533	2.757427	0.014477	0.840963	-4.15603	0.529865	0.423201	-0.10666	9	1.4E+08	R	II		IGR	opensea	IGR-opense			

cg0060035	0.108535	0.29451	2.740448	0.014984	0.840963	-4.18913	0.348778	0.240242	-0.10854	14	22942833	R	II		IGR	opensea	IGR-opense		rs5775700	42;37;34;17
cg2000414	0.123652	0.530981	2.729858	0.015308	0.840963	-4.20974	0.592807	0.469155	-0.12365	2	65718931	F	II		IGR	opensea	IGR-opense		rs1852897	21;22;45
cg1956640	0.124378	0.28353	2.728436	0.015352	0.840963	-4.2125	0.345719	0.221341	-0.12438	17	33759965	F	II	SLFN12	TSS1500	opensea	TSS1500-op			
cg1707886	0.111729	0.582854	2.726364	0.015416	0.840963	-4.21653	0.638718	0.52699	-0.11173	2	1.26E+08	R	II	CNTNAP5	Body	opensea	Body-opens		rs7924920	11;2
cg2181305	0.110818	0.658136	2.725242	0.015451	0.840963	-4.21871	0.713545	0.602727	-0.11082	19	56060880	R	II		IGR	shore	IGR-shore	chr19:5606		
cg2354990	0.10776	0.476464	2.724039	0.015489	0.840963	-4.22105	0.530344	0.422584	-0.10776	7	5184155	F	I		IGR	island	IGR-island	chr7:5183	rs1812386	4;42
cg2349032	0.104059	0.662117	2.722558	0.015536	0.840963	-4.22393	0.714147	0.610088	-0.10406	5	57014231	F	II		IGR	opensea	IGR-opense			
cg0715850	0.1941	0.429744	2.720227	0.015609	0.840963	-4.22846	0.526794	0.332694	-0.1941	5	1.35E+08	R	II		IGR	shore	IGR-shore	chr5:1354	rs5652797	47;26;2
cg2168698	0.114749	0.711691	2.718533	0.015662	0.840963	-4.23175	0.769066	0.654317	-0.11475	16	75252973	R	II	CTRB1	Body	opensea	Body-opens		rs2012454	18
cg1176657	0.111198	0.685921	2.711484	0.015887	0.840963	-4.24543	0.74152	0.630321	-0.1112	21	47581405	R	I	C21orf56	Body	island	Body-islanc	chr21:4758	rs2002575	18
cg0322009	0.107638	0.373502	2.70803	0.015998	0.840963	-4.25213	0.427321	0.319683	-0.10764	14	22928550	R	II		IGR	opensea	IGR-opense		rs1152784	10;9;1
cg1866187	0.102355	0.427293	2.702997	0.016161	0.840963	-4.26189	0.47847	0.376115	-0.10236	5	1.31E+08	F	II		IGR	opensea	IGR-opense		rs7864135	1;18
cg0666225	0.109045	0.536388	2.696481	0.016375	0.840963	-4.27451	0.590911	0.481866	-0.10905	1	16847157	F	II		IGR	shore	IGR-shore	chr1:1684	rs5385545	1;11;15;25;28;33
cg2159875	0.11974	0.842186	2.682407	0.016846	0.840963	-4.30174	0.902057	0.782316	-0.11974	1	1.6E+08	F	II		IGR	opensea	IGR-opense		rs5710350	17;39
cg2527780	0.124258	0.38966	2.678501	0.01698	0.840963	-4.30929	0.451789	0.32753	-0.12426	2	1.28E+08	F	II		IGR	shore	IGR-shore	chr2:1284	rs1870326	20;23
cg1239358	0.109576	0.787235	2.675267	0.017091	0.840963	-4.31554	0.842023	0.732447	-0.10958	7	45027643	F	II	C7orf40	TSS1500	shore	TSS1500-sh	chr7:4502	rs5775359	3;26
cg0325165	0.126024	0.31118	2.666294	0.017402	0.840963	-4.33286	0.374192	0.248168	-0.12602	17	33759512	F	II	SLFN12	1stExon	opensea	1stExon-op		rs5624302	12;38
cg2309983	0.120192	0.386123	2.666188	0.017406	0.840963	-4.33306	0.446219	0.326027	-0.12019	8	2585976	F	I		IGR	opensea	IGR-opense		rs5362757	2;5;26;27
cg1379721	0.131716	0.422539	2.656799	0.017738	0.840963	-4.35116	0.488397	0.356681	-0.13172	7	71870614	F	II	CALN1	5'UTR	opensea	5'UTR-open		rs5639559	9;38;42;43
cg2597585	0.10257	0.684495	2.652867	0.017878	0.840963	-4.35873	0.73578	0.633211	-0.10257	3	19930780	F	II	EFHB	Body	opensea	Body-opens		rs1876693	1
cg1879765	0.154923	0.388639	2.645953	0.018129	0.840963	-4.37204	0.466101	0.311177	-0.15492	5	1.35E+08	F	II	MIR886	TSS1500	shore	TSS1500-sh	chr5:1354		
cg1078883	0.111999	0.436417	2.645294	0.018153	0.840963	-4.3733	0.492416	0.380417	-0.112	5	1.33E+08	F	II		IGR	opensea	IGR-opense		rs5293137	2;27;31;39
cg0012499	0.164153	0.303741	2.637651	0.018434	0.840963	-4.38799	0.385818	0.221665	-0.16415	5	1.35E+08	F	I	MIR886	TSS200	island	TSS200-isla	chr5:1354	rs5598580	38;44
cg1715686	0.1088	0.47765	2.634995	0.018532	0.840963	-4.3931	0.53205	0.42325	-0.1088	1	18806982	F	II	KLHDC7A	TSS1500	shore	TSS1500-sh	chr1:1880		
cg0451520	0.10856	0.276893	2.623872	0.01895	0.840963	-4.41444	0.331173	0.222613	-0.10856	5	1.35E+08	F	II		IGR	shore	IGR-shore	chr5:1354	rs5493217	15
cg1557223	0.109097	0.520771	2.623002	0.018984	0.840963	-4.41611	0.57532	0.466223	-0.1091	7	5183992	F	II		IGR	island	IGR-island	chr7:5183	rs5510959	12;39
cg0111927	0.123765	0.525214	2.618542	0.019154	0.840963	-4.42466	0.587096	0.463331	-0.12376	6	1.11E+08	F	II	DDO	Body	island	Body-islanc	chr6:1107	rs1496133	23;44;49
cg1781863	0.128776	0.666872	2.611527	0.019425	0.840963	-4.43809	0.73126	0.602485	-0.12878	21	40361611	F	II		IGR	opensea	IGR-opense		rs5642788	1;30
cg2050727	0.183096	0.309043	2.60922	0.019515	0.840963	-4.44251	0.400591	0.217495	-0.1831	1	2.48E+08	F	II	OR2L13	1stExon	island	1stExon-isl	chr1:2481	rs1118340	26;31;47
cg2689694	0.158689	0.385261	2.606492	0.019622	0.840963	-4.44773	0.464605	0.305916	-0.15869	5	1.35E+08	F	I	MIR886	TSS200	island	TSS200-isla	chr5:1354	rs5598580	45;51
cg0300687	0.100005	0.38105	2.60423	0.019711	0.840963	-4.45205	0.431053	0.331047	-0.10001	14	22945582	F	II		IGR	opensea	IGR-opense			
cg0356583	0.117472	0.690706	2.601344	0.019826	0.840963	-4.45757	0.749442	0.63197	-0.11747	13	36466519	R	II	MIR548F5	Body	opensea	Body-opens		rs3691628	51;1
cg0049271	0.104355	0.31894	2.599839	0.019886	0.840963	-4.46044	0.371117	0.266763	-0.10435	17	33760278	F	I	SLFN12	TSS1500	opensea	TSS1500-op		rs1386353	18;23;24;30;34;43;47
cg1804944	0.10917	0.527891	2.593697	0.020132	0.840963	-4.47217	0.582476	0.473306	-0.10917	22	23245790	R	II		IGR	opensea	IGR-opense		rs1442090	41;31;18;1
cg0851354	0.141923	0.561922	2.586855	0.020409	0.840963	-4.48523	0.632884	0.490961	-0.14192	5	1.5E+08	F	II	LOC13446	Body	shelf	Body-shelf	chr5:1503	rs5708450	42
cg0894417	0.17924	0.292824	2.586812	0.020411	0.840963	-4.48531	0.382444	0.203204	-0.17924	1	2.48E+08	F	II	OR2L13	1stExon	island	1stExon-isl	chr1:2481	rs1118340	12;17;33;49
cg1485656	0.124867	0.678149	2.584862	0.02049	0.840963	-4.48903	0.740582	0.615715	-0.12487	1	19111089	R	II		IGR	shore	IGR-shore	chr1:1911	rs7405662	13
cg2690525	0.112215	0.315199	2.583042	0.020565	0.840963	-4.4925	0.371306	0.259092	-0.11221	19	15923097	R	II		IGR	opensea	IGR-opense		rs6718753	19
cg0392678	0.12327	0.369986	2.578732	0.020743	0.840963	-4.50071	0.431621	0.308351	-0.12327	1	1.6E+08	F	II	TAGLN2	Body	shore	Body-shore	chr1:1598	rs1445078	15
cg1177470	0.121377	0.539741	2.57487	0.020904	0.840963	-4.50807	0.600429	0.479052	-0.12138	16	1561735	F	II	IFT140	Body	shore	Body-shore	chr16:155	rs1470096	1;22
cg2534068	0.193039	0.403972	2.57483	0.020906	0.840963	-4.50814	0.500492	0.307453	-0.19304	5	1.35E+08	F	I	MIR886	TSS200	island	TSS200-isla	chr5:1354		
cg1983196	0.13632	0.686588	2.571577	0.021042	0.840963	-4.51433	0.754748	0.618428	-0.13632	2	317929	R	II		IGR	shelf	IGR-shelf	chr2:3148	rs7792054	20
cg2311877	0.107108	0.406514	2.569147	0.021144	0.840963	-4.51896	0.460068	0.35296	-0.10711	4	89152696	R	II	ABCG2	TSS1500	opensea	TSS1500-op		rs5506870	50;49;48
cg0647888	0.154183	0.340879	2.568902	0.021154	0.840963	-4.51942	0.417971	0.263788	-0.15418	5	1.35E+08	R	II		IGR	shore	IGR-shore	chr5:1354	rs5643603	46
cg1792791	0.106031	0.772841	2.558938	0.021579	0.840963	-4.53836	0.825857	0.719825	-0.10603	3	56953498	F	II	ARHGEF3	Body	opensea	Body-opens		rs1406070	0;35
cg2007389	0.118319	0.403605	2.557411	0.021645	0.840963	-4.54126	0.462764	0.344445	-0.11832	14	22945457	F	II		IGR	opensea	IGR-opense			
cg0008320	0.116576	0.669875	2.555717	0.021719	0.840963	-4.54447	0.728163	0.611587	-0.11658	6	1.11E+08	R	II	DDO	Body	island	Body-islanc	chr6:1107	rs5471762	51;25
cg0342280	0.107327	0.766325	2.550944	0.021926	0.840963	-4.55353	0.819989	0.712662	-0.10733	20	55292557	R	II		IGR	opensea	IGR-opense		rs1698023	40;16
cg0072306	0.103516	0.370541	2.548095	0.022051	0.840963	-4.55893	0.422299	0.318783	-0.10352	14	22938770	R	II		IGR	opensea	IGR-opense		rs5587059	43;38;1
cg1913932	0.11228	0.50087	2.546239	0.022133	0.840963	-4.56245	0.55701	0.44473	-0.11228	2	2.18E+08	R	II		IGR	opensea	IGR-opense			
cg2158741	0.132693	0.4154	2.545594	0.022161	0.840963	-4.56367	0.481746	0.349054	-0.13269	3	17194063	F	II		IGR	opensea	IGR-opense		rs1306234	8
cg0078594	0.145854	0.264163	2.545583	0.022162	0.840963	-4.56369	0.33709	0.191236	-0.14585	1	2.48E+08	F	II	OR2L13	1stExon	island	1stExon-isl	chr1:2481	rs1118340	35;40
cg2092035	0.120816	0.236594	2.537518	0.022521	0.840963	-4.57897	0.297002	0.176186	-0.12082	4	1.17E+08	R	II		IGR	opensea	IGR-opense		rs1399886	43;40;30;9;1;1
cg2030895	0.173494	0.746228	2.526696	0.023011	0.840963	-4.59943	0.832975	0.659481	-0.17349	11	42186623	R	II		IGR	opensea	IGR-opense		rs5735513	1
cg0343277	0.109889	0.584204	2.524906	0.023093	0.840963	-4.60281	0.639149	0.529259	-0.10989	1	21033761	F	II	KIF17	Body	shelf	Body-shelf	chr1:2103	rs875016;r	18;24;25;38
cg2368247	0.10981	0.428873	2.511054	0.023738	0.840963	-4.62895	0.483778	0.373968	-0.10981	14	22924109	R	II		IGR	opensea	IGR-opense		rs5717728	51;23
cg1333384	0.144032	0.576742	2.508495	0.023859	0.840963	-4.63377	0.648758	0.504727	-0.14403	13	1.1E+08	R	II		IGR	opensea	IGR-opense		rs7764741	31
cg2087723	0.154135	0.623513	2.503151	0.024114	0.840963	-4.64383	0.70058	0.546445	-0.15413	8	6876684	F	II	DEFA3	TSS1500	opensea	TSS1500-op		rs7150922	0;11;15;20;22;32;38;44;46
cg2287853	0.134717	0.378919	2.501448	0.024195	0.840963	-4.64703	0.446278	0.311561	-0.13472	17	78431627	F	II		IGR	shelf	IGR-shelf	chr17:7842	rs5632159	31;51
cg0939816	0.108707	0.678746	2.496759	0.024421	0.840963	-4.65585	0.733099	0.624392	-0.10871	21	40291207	R	II	LOC40086	Body	opensea	Body-opens		rs2836753	22;1
cg0374837	0.167992	0.284855	2.482352	0.025129	0.840963	-4.68291	0.368851	0.200859	-0.16799	1	2.48E+08	F	I	OR2L13	1stExon	island	1stExon-isl	chr1:2481	rs1118340	41;46
cg2250002	0.120748	0.262938	2.479166	0.025288	0.840963	-4.68888	0.323312	0.202564	-0.12075	14	22935574	R	II		IGR	opensea	IGR-opense		rs5662211	40
cg1461310	0.172981	0.724812	2.476694	0.025412	0.840963	-4.69351	0.811302	0.638321	-0.17298	9	1.08E+08	F	II		IGR	opensea	IGR-opense		rs7274032	0;1;8;18;38;42
cg1107989	0.110925	0.217577	2.474466	0.025525	0.840963	-4.69769	0.27304	0.162115	-0.11092	14	63671314	F	II	RHOJ	5'UTR	opensea	5'UTR-open		rs1454903	0;12;35
cg2153463	0.154966	0.793425	2.459836	0.026274	0.840963	-4.72505	0.870908	0.715942	-0.15497	21	42742432	R	II	MX2	5'UTR	opensea	5'UTR-open		rs1872874	18
cg2436412	0.122455	0.623898	2.456638	0.026441	0.840963	-4.73103	0.685126	0.56267	-0.12246	15	33757827	F	II	RYR3	Body	opensea	Body-opens		rs5452455	0;4;13;40

cg1757327	0.11993	0.644284	2.444178	0.027099	0.840963	-4.75426	0.704249	0.584319	-0.11993	22	42950376	R	II	SERHL2	Body	shore	Body-shore	chr22:4294	rs5411937	35;17
cg1160815	0.169514	0.313532	2.427118	0.028027	0.840963	-4.786	0.398289	0.228774	-0.16951	5	1.35E+08	R	I		IGR	shore	IGR-shore	chr5:1354	rs3757214	1
cg0874596	0.135563	0.236255	2.425779	0.028101	0.840963	-4.78849	0.304036	0.168474	-0.13556	5	1.35E+08	F	II	MIR886	TSS1500	shore	TSS1500-sh	chr5:1354	rs7158930	7;18
cg0269150	0.151943	0.363178	2.420326	0.028404	0.840963	-4.79861	0.43915	0.287206	-0.15194	3	8685827	R	II	C3orf32	5'UTR	opensea	5'UTR-open		rs5554027	47;28
cg0217181	0.105887	0.514028	2.416209	0.028635	0.840963	-4.80624	0.566971	0.461084	-0.10589	17	1645925	R	II	SERPINF2	TSS1500	opensea	TSS1500-op		rs7730532	35;13
cg2219923	0.103814	0.547047	2.409885	0.028994	0.840963	-4.81796	0.598954	0.49514	-0.10381	11	613857	F	II	IRF7	Body	shore	Body-shore	chr11:614	rs5449134	0;2;16;22;27;41;44;47;51
cg1604237	0.102096	0.645478	2.408184	0.029091	0.840963	-4.82111	0.696526	0.59443	-0.1021	2	2.27E+08	R	II		IGR	opensea	IGR-opense		rs5485805	42;19
cg1105915	0.108202	0.436518	2.405706	0.029233	0.840963	-4.82569	0.490618	0.382417	-0.1082	2	1.28E+08	F	II		IGR	island	IGR-island	chr2:1284	rs3731693	20
cg0560739	0.129856	0.709927	2.402194	0.029436	0.840963	-4.83219	0.774855	0.644999	-0.12986	21	47445196	F	II		IGR	opensea	IGR-opense		rs9984302	14;31
cg0117581	0.12347	0.198968	2.398798	0.029633	0.840963	-4.83847	0.260703	0.137233	-0.12347	12	4919001	F	II	KCNA6	5'UTR	island	5'UTR-islan	chr12:491		
cg1981380	0.100072	0.672405	2.387308	0.030309	0.840963	-4.85967	0.722441	0.622369	-0.10007	2	2.19E+08	F	II	DIRC3	Body	opensea	Body-opens		rs1869073	0;8
cg0541981	0.109416	0.231916	2.386332	0.030367	0.840963	-4.86147	0.286624	0.177208	-0.10942	6	31276437	R	I		IGR	island	IGR-island	chr6:3127	rs9264960	43;42;27;12;4
cg2685870	0.171593	0.252809	2.38575	0.030401	0.840963	-4.86254	0.338605	0.167013	-0.17159	12	4918848	R	I	KCNA6	5'UTR	island	5'UTR-islan	chr12:491	rs1137637	47
cg1164336	0.135416	0.396148	2.379923	0.030751	0.840963	-4.87328	0.463856	0.32844	-0.13542	14	22923261	R	II		IGR	opensea	IGR-opense		rs5486425	38;2;1
cg1445094	0.185289	0.219573	2.361829	0.03186	0.840963	-4.90653	0.312217	0.126928	-0.18529	16	3152320	R	II		IGR	shelf	IGR-shelf	chr16:315	rs7400558	49;33;30;27;16
cg1867864	0.167985	0.290794	2.35844	0.032072	0.840963	-4.91275	0.374787	0.206802	-0.16799	5	1.35E+08	R	II	MIR886	TSS200	island	TSS200-isla	chr5:1354	rs5548486	26;15;12
cg2283805	0.130193	0.340432	2.331234	0.033822	0.840963	-4.96249	0.405528	0.275335	-0.13019	19	872690	F	II	MED16	Body	shore	Body-shore	chr19:870	rs1913636	33;35;37
cg1858620	0.111105	0.424711	2.33016	0.033893	0.840963	-4.96445	0.480263	0.369158	-0.1111	14	22924450	F	II		IGR	opensea	IGR-opense		rs1427944	1;41
cg1987051	0.183206	0.329638	2.327779	0.03405	0.840963	-4.96879	0.421241	0.238035	-0.18321	12	4919081	R	I	KCNA6	5'UTR	island	5'UTR-islan	chr12:491	rs1281218	26
cg1045613	0.103836	0.538174	2.325646	0.034192	0.840963	-4.97268	0.590092	0.486256	-0.10384	9	76985951	F	II	LOC10192	TSS1500	opensea	TSS1500-op		rs5668494	0;1;5;13
cg1392085	0.155615	0.765318	2.321946	0.034439	0.840963	-4.97941	0.843125	0.68751	-0.15561	2	1.6E+08	F	II	TANC1	5'UTR	opensea	5'UTR-open		rs5422787	0;13
cg2172807	0.103692	0.608672	2.321098	0.034496	0.840963	-4.98096	0.660518	0.556827	-0.10369	5	9835108	F	II	LOC28569	Body	opensea	Body-opens		rs5506947	45;49
cg1387375	0.101711	0.38279	2.315835	0.034852	0.840963	-4.99053	0.433646	0.331934	-0.10171	2	317826	R	II		IGR	shelf	IGR-shelf	chr2:3148	rs5515383	33;13
cg0173578	0.107007	0.258076	2.282552	0.037179	0.840963	-5.05082	0.31158	0.204572	-0.10701	22	27520354	F	II		IGR	opensea	IGR-opense		rs5607008	49;51
cg2487559	0.107686	0.856755	2.27912	0.037427	0.840963	-5.05701	0.910598	0.802912	-0.10769	21	45153009	F	II	PDXK	Body	shelf	Body-shelf	chr21:451	rs5731164	39
cg0216827	0.195712	0.609209	2.276773	0.037597	0.840963	-5.06124	0.707065	0.511353	-0.19571	8	50110978	F	II		IGR	opensea	IGR-opense		rs5757957	0;1
cg1956153	0.101489	0.463074	2.268107	0.038233	0.840963	-5.07685	0.513818	0.41233	-0.10149	5	1.31E+08	F	II	RAPGEF6	Body	opensea	Body-opens		rs1922406	0;12;27
cg1067166	0.1437	0.275014	2.264446	0.038505	0.840963	-5.08344	0.346865	0.203164	-0.1437	12	4919230	R	I	KCNA6	1stExon	island	1stExon-isl	chr12:491	rs5776153	21
cg1548959	0.16334	0.164339	2.25407	0.039285	0.840963	-5.10207	0.246009	0.08267	-0.16334	17	76010131	F	II	TNRC6C	5'UTR	opensea	5'UTR-open		rs1810716	23;40
cg2532673	0.10782	0.44924	2.240538	0.040324	0.840963	-5.12631	0.50315	0.395331	-0.10782	4	2714321	R	II	FAM193A	Body	opensea	Body-opens		rs1126529	17;1
cg2263552	0.148929	0.601172	2.231351	0.041044	0.840963	-5.14273	0.675637	0.526708	-0.14893	6	32492390	R	II	HLA-DRB5	Body	shelf	Body-shelf	chr6:3248	rs1152803	38
cg0448192	0.181656	0.333455	2.217225	0.042174	0.840963	-5.1679	0.424283	0.242627	-0.18166	5	1.35E+08	R	II	MIR886	Body	island	Body-island	chr5:1354	rs5387104	38;18;2
cg1640661	0.11078	0.639969	2.212448	0.042563	0.840963	-5.17639	0.695359	0.584579	-0.11078	11	300404	F	II	IFITM5	TSS1500	shore	TSS1500-sh	chr11:299	rs5605609	21;30;31
cg0310346	0.103507	0.320817	2.210497	0.042723	0.840963	-5.17986	0.372571	0.269064	-0.10351	14	22938728	F	II		IGR	opensea	IGR-opense		rs5587059	1;6;43
cg2432521	0.135036	0.704268	2.202069	0.04342	0.840963	-5.19481	0.771785	0.63675	-0.13504	18	3258800	F	II		IGR	shelf	IGR-shelf	chr18:326	rs5428286	23;50
cg2145516	0.109216	0.568926	2.201595	0.043459	0.840963	-5.19565	0.623534	0.514319	-0.10922	2	50763653	F	II	NRXN1	Body	opensea	Body-opens		rs1413706	7;35;50
cg0477755	0.140841	0.840293	2.19695	0.043848	0.840963	-5.20388	0.910714	0.769872	-0.14084	6	32628953	R	II	HLA-DQB1	Body	shelf	Body-shelf	chr6:3263	rs3513991	49;48;42;41;40;39;38;34;33;16;14;11;2
cg0688874	0.177023	0.532172	2.192226	0.044246	0.840963	-5.21224	0.620684	0.443661	-0.17702	10	1.06E+08	F	I	SH3PXD2A	Body	opensea	Body-opens		rs1163638	7;8;17;35
cg2691383	0.152747	0.167968	2.189499	0.044478	0.840963	-5.21707	0.244341	0.091594	-0.15275	16	86542923	F	II	FOXF1	TSS1500	island	TSS1500-isl	chr16:865	rs7229591	4
cg1872057	0.119297	0.506566	2.162624	0.046822	0.840963	-5.26442	0.566215	0.446918	-0.1193	15	29278600	R	II	APBA2	5'UTR	opensea	5'UTR-open			
cg1801630	0.10527	0.608827	2.145038	0.048417	0.840963	-5.29523	0.661462	0.556193	-0.10527	15	31287286	F	I		IGR	shelf	IGR-shelf	chr15:312	rs7271044	8;13
cg0713690	0.104634	0.442279	2.131543	0.049675	0.840963	-5.31879	0.494596	0.389962	-0.10463	11	71278894	F	II		IGR	opensea	IGR-opense		rs5454114	12;21
cg0209468	0.111775	0.293799	2.130222	0.049799	0.840963	-5.32109	0.349687	0.237911	-0.11178	5	1.14E+08	F	II		IGR	opensea	IGR-opense		rs1895585	0
cg2475965	0.104277	0.455356	2.128948	0.04992	0.840963	-5.32331	0.507494	0.403217	-0.10428	17	76801065	R	II	USP36	Body	shelf	Body-shelf	chr17:767	rs7476313	41;13;1

SUPPLEMENTAL TABLE 3 - FEMALE SUBSET - HYPER

	logFC	AveExpr	t	P.Value	adj.P.Val	B	LBP_f_AVG	Normal_f_A	deltaBeta	CHR	MAPINFO	Strand	Type	gene	feature	cgi	feat.cgi	UCSC_Islan	SNP_ID	SNP_DISTANCE
cg15053404	-0.22031	0.630566	-12.9801	1.82E-06	0.000477	5.836794	0.740722	0.52041	0.220312	10	1.13E+08	F	II		IGR	opensea	IGR-opense		rs3681419	13
cg02183960	-0.20834	0.290706	-11.7405	3.79E-06	0.000591	5.113624	0.394875	0.186537	0.208337	5	35859088	R	II	IL7R	Body	opensea	Body-opens		rs1874228	9;2;1
cg08841318	-0.20836	0.175851	-11.5237	4.34E-06	0.000618	4.978337	0.28003	0.071672	0.208358	6	1.25E+08	F	I	RNF217	1stExon	island	1stExon-isl	chr6:12528		
cg12451425	-0.20824	0.413949	-11.3168	4.95E-06	0.000642	4.846629	0.518068	0.309831	0.208238	17	46625911	F	II	HOXB-AS1	Body	shore	Body-shore	chr17:4662		
cg06817490	-0.2099	0.337331	-11.0836	5.75E-06	0.000675	4.694979	0.442281	0.232382	0.209899	17	55888548	R	I		IGR	opensea	IGR-opense		rs7218692	22;21;13;11
cg05095590	-0.31524	0.370075	-10.9293	6.36E-06	0.000697	4.592715	0.527693	0.212456	0.315236	7	2139259	R	I	MAD1L1	Body	opensea	Body-opens		rs1895159	38
cg24885723	-0.29654	0.637775	-10.6417	7.71E-06	0.000748	4.397998	0.786043	0.489507	0.296536	1	9030439	F	II	CA6	Body	opensea	Body-opens		rs5342159	14
cg21588347	-0.20944	0.328914	-10.5704	8.09E-06	0.000762	4.348874	0.433635	0.224193	0.209442	20	39667871	R	II	TOP1	Body	opensea	Body-opens		rs5522316	43;27;1
cg23651386	-0.26047	0.347788	-10.2148	1.03E-05	0.000839	4.098555	0.478023	0.217553	0.26047	19	47685890	F	II	SAE1	Body	opensea	Body-opens		rs5414603	44
cg16178058	-0.24214	0.267346	-10.039	1.17E-05	0.00088	3.971464	0.388413	0.146278	0.242135	11	1.28E+08	F	I	ETS1	Body	opensea	Body-opens		rs7926975	15;18;35;46
cg14199707	-0.26159	0.564756	-10.0133	1.19E-05	0.000886	3.952667	0.695551	0.433961	0.261589	6	90983735	R	II	BACH2	5'UTR	opensea	5'UTR-oper		rs5638636	48;3;2;1
cg09988805	-0.22929	0.314874	-9.84537	1.34E-05	0.000932	3.828842	0.429522	0.200227	0.229294	2	43278552	F	II		IGR	opensea	IGR-opense		rs5503594	0;12;48
cg04217471	-0.26675	0.320452	-9.51947	1.71E-05	0.001034	3.582282	0.453826	0.187079	0.266747	12	6895978	F	II		IGR	opensea	IGR-opense		rs1125748	7;21
cg18722847	-0.2031	0.239742	-9.26622	2.07E-05	0.001125	3.384839	0.341294	0.13819	0.203104	3	44754129	F	I	ZNF502	TSS200	island	TSS200-isla	chr3:44754	rs1150324	12;26;48
cg19882512	-0.22738	0.388949	-9.25015	2.09E-05	0.001132	3.372134	0.502638	0.27526	0.227378	5	1.33E+08	F	II		IGR	shelf	IGR-shelf	chr5:1334	rs7656169	35
cg22438763	-0.23376	0.497179	-9.21673	2.15E-05	0.001143	3.345642	0.61406	0.380299	0.233761	1	47900256	R	I	FOXD2	TSS1500	island	TSS1500-isl	chr1:4789		
cg12201505	-0.2166	0.477686	-9.20077	2.17E-05	0.001148	3.332952	0.585988	0.369384	0.216604	4	1.24E+08	F	II	SPATA5	Body	opensea	Body-opens		rs5303529	0
cg01765967	-0.30709	0.484585	-9.17933	2.21E-05	0.001156	3.315875	0.638128	0.331042	0.307086	17	78637626	R	II	RPTOR	Body	opensea	Body-opens		rs5438319	15;6
cg27009448	-0.24936	0.516874	-9.16965	2.22E-05	0.00116	3.308157	0.641553	0.392194	0.24936	10	1.2E+08	R	II	C10orf46	Body	opensea	Body-opens			
cg24915004	-0.25317	0.493868	-9.07539	2.39E-05	0.001196	3.232548	0.620452	0.367285	0.253167	15	43001029	F	II	STARD9	Body	opensea	Body-opens		rs5348960	17;22;46
cg12189962	-0.29436	0.47329	-9.03614	2.47E-05	0.001211	3.200842	0.620468	0.326113	0.294355	20	31098130	F	II	LOC10192	Body	opensea	Body-opens		rs5426220	33
cg13344434	-0.20711	0.290081	-8.90932	2.72E-05	0.001264	3.097493	0.393637	0.186525	0.207112	6	35570573	R	II	FKBP5	Body	opensea	Body-opens		rs1847338	10
cg05057720	-0.21638	0.196016	-8.88089	2.79E-05	0.001277	3.074133	0.304208	0.087825	0.216383	14	38724675	R	I	CLEC14A	1stExon	island	1stExon-isl	chr14:387	rs1146228	24;21
cg23774988	-0.24323	0.435383	-8.84923	2.86E-05	0.001288	3.048032	0.556999	0.313767	0.243232	11	1.28E+08	F	I	ETS1	Body	island	Body-islanc	chr11:128	rs4396302	25;28
cg15352525	-0.23034	0.578288	-8.80122	2.97E-05	0.001309	3.008277	0.69346	0.463116	0.230344	22	39812626	R	II	TAB1	Body	opensea	Body-opens		rs5551565	48;2
cg16230121	-0.23315	0.323046	-8.72115	3.16E-05	0.001344	2.941532	0.439619	0.206473	0.233145	11	6435089	F	II	APBB1	5'UTR	opensea	5'UTR-oper		rs1884733	14
cg24398381	-0.27811	0.406117	-8.69759	3.22E-05	0.001356	2.921781	0.545171	0.267063	0.278108	18	77276814	R	II	NFATC1	Body	shelf	Body-shelf	chr18:772	rs5442072	42;38;31;13;2
cg04976117	-0.20715	0.362568	-8.62651	3.41E-05	0.001389	2.861884	0.466145	0.258991	0.207154	5	1.11E+08	R	II	CAMK4	Body	opensea	Body-opens		rs5363789	46;45;35;21;20;1
cg12121660	-0.20451	0.248045	-8.6221	3.43E-05	0.001391	2.858153	0.350301	0.145788	0.204513	17	46621709	F	I	HOXB2	Body	shore	Body-shore	chr17:4662		
cg06410591	-0.2181	0.3313	-8.6005	3.49E-05	0.001402	2.839856	0.440351	0.222248	0.218102	19	6741181	R	I	TRIP10	Body	island	Body-islanc	chr19:674	rs5464567	19
cg04113075	-0.20751	0.312116	-8.55132	3.63E-05	0.001428	2.798024	0.415871	0.208361	0.20751	6	1.47E+08	R	II	RAB32	Body	shore	Body-shore	chr6:1468	rs4128586	24
cg06011086	-0.24211	0.452597	-8.55015	3.63E-05	0.001428	2.797034	0.573653	0.331541	0.242111	8	61823275	F	II		IGR	shore	IGR-shore	chr8:6182	rs5524829	10
cg08280368	-0.29805	0.369234	-8.54849	3.64E-05	0.001429	2.795614	0.518258	0.220209	0.298049	14	71110536	F	II	TTC9	Body	shore	Body-shore	chr14:711	rs7406331	13;30;31;35;42
cg02295831	-0.22191	0.4582	-8.52857	3.7E-05	0.001441	2.778605	0.569156	0.347244	0.221912	3	16416167	F	II	RFTN1	Body	opensea	Body-opens		rs5752060	22;31;45
cg12633154	-0.32084	0.567807	-8.5275	3.7E-05	0.001441	2.77769	0.728228	0.407386	0.320842	6	30039435	R	I	RNF39	Body	island	Body-islanc	chr6:3003	rs2301752	19;18
cg18208842	-0.20395	0.567696	-8.43354	4E-05	0.001494	2.69694	0.669671	0.465722	0.203949	2	287395	F	I	FAM150B	Body	island	Body-islanc	chr2:2873	rs5613771	9
cg26768584	-0.24812	0.308676	-8.40855	4.08E-05	0.001509	2.675323	0.432736	0.184616	0.248119	3	18480242	R	II	SATB1	5'UTR	opensea	5'UTR-oper		rs5391052	20
cg10864951	-0.25664	0.308301	-8.35395	4.27E-05	0.001541	2.627888	0.436621	0.179981	0.25664	7	1159070	F	II	C7orf50	Body	shore	Body-shore	chr7:1155	rs5333103	0;11;13;49
cg21780393	-0.22353	0.337526	-8.32498	4.37E-05	0.001559	2.602606	0.449288	0.225763	0.223525	17	46618919	R	II		IGR	shore	IGR-shore	chr17:466		
cg00885208	-0.28072	0.419115	-8.29921	4.47E-05	0.001576	2.580046	0.559474	0.278757	0.280717	15	70827566	R	II		IGR	opensea	IGR-opense		rs5442891	38;19;1
cg12682972	-0.23862	0.397141	-8.28099	4.54E-05	0.001587	2.564057	0.516451	0.277831	0.23862	2	1.02E+08	F	II	MAP4K4	Body	opensea	Body-opens		rs3716943	4;48
cg16240816	-0.30381	0.409172	-8.27457	4.56E-05	0.001591	2.558418	0.561077	0.257267	0.303811	2	65861662	F	II		IGR	opensea	IGR-opense		rs5340833	51
cg27331241	-0.21029	0.199647	-8.26714	4.59E-05	0.001597	2.551887	0.304792	0.094501	0.210292	7	751830	F	I	PRKAR1B	5'UTR	island	5'UTR-islan	chr7:7517	rs5525994	13
cg24563570	-0.21414	0.246363	-8.19655	4.87E-05	0.001643	2.489546	0.353432	0.139294	0.214138	10	60936801	F	I	PHYHIPL	Body	island	Body-islanc	chr10:609	rs3763659	9;30
cg15297642	-0.23944	0.36435	-8.19378	4.88E-05	0.001645	2.487085	0.484072	0.244628	0.239444	18	13369816	R	II	LDLRAD4	5'UTR	opensea	5'UTR-oper			
cg10757080	-0.26521	0.48726	-8.17782	4.95E-05	0.001655	2.47292	0.619866	0.354655	0.265211	3	39336300	F	II		IGR	opensea	IGR-opense		rs5291422	1
cg22190381	-0.20306	0.459809	-8.15861	5.03E-05	0.001668	2.455833	0.561337	0.358281	0.203056	10	1.16E+08	R	II	ABLIM1	Body	opensea	Body-opens		rs5599614	50;2;1
cg25979005	-0.21015	0.413574	-8.13946	5.11E-05	0.001682	2.438766	0.51865	0.308498	0.210152	8	99794263	F	II	STK3	Body	opensea	Body-opens		rs5495433	47
cg09644974	-0.20842	0.406427	-8.13141	5.15E-05	0.001687	2.43158	0.510638	0.302216	0.208422	2	1.77E+08	R	II		IGR	shelf	IGR-shelf	chr2:1773		
cg06943912	-0.2341	0.447835	-8.10008	5.28E-05	0.001713	2.403555	0.564885	0.330785	0.234099	19	10715612	F	I	SLC44A2	Body	shelf	Body-shelf	chr19:107	rs5602263	0;1;3;44
cg03117976	-0.21817	0.193477	-8.07847	5.38E-05	0.001728	2.384168	0.302562	0.084393	0.21817	18	11149435	F	I	FAM38B	TSS1500	island	TSS1500-isl	chr18:111	rs5724852	51
cg26053175	-0.25933	0.391151	-8.07343	5.41E-05	0.001731	2.379638	0.520814	0.261488	0.259327	19	51858098	R	II	ETFB	TSS200	opensea	TSS200-ope		rs5428185	5
cg16709841	-0.23916	0.243853	-8.00848	5.72E-05	0.00178	2.321046	0.363433	0.124272	0.239161	12	11708901	R	II	LINC01252	Body	opensea	Body-opens		rs3746755	40;1
cg23870749	-0.21957	0.264904	-8.0073	5.72E-05	0.001781	2.319981	0.374689	0.15512	0.219569	1	2.27E+08	R	II	PSEN2	Body	opensea	Body-opens			
cg16971827	-0.24506	0.379995	-8.00706	5.72E-05	0.001781	2.319766	0.502523	0.257466	0.245057	11	1.19E+08	R	II	CBL	3'UTR	shelf	3'UTR-shelf	chr11:119	rs5614203	2
cg19196555	-0.20904	0.576082	-7.97835	5.87E-05	0.001802	2.293732	0.680602	0.471562	0.209039	4	57524758	R	II	HOPX	5'UTR	shelf	5'UTR-shelf	chr4:5752		
cg03013609	-0.72502	0.40937	-7.96664	5.93E-05	0.001812	2.283085	0.77188	0.046861	0.725019	1	2.37E+08	F	II	LGALS8	Body	opensea	Body-opens		rs2870452	0;11;22
cg22556683	-0.2097	0.32066	-7.93424	6.09E-05	0.001838	2.25356	0.425512	0.215808	0.209704	15	91427738	R	I	FES	TSS1500	island	TSS1500-isl	chr15:914	rs1386825	48
cg07384080	-0.22631	0.31251	-7.92256	6.16E-05	0.001847	2.242891	0.425663	0.199356	0.226307	1	1.56E+08	R	II		IGR	opensea	IGR-opense		rs6701295	27
cg05961294	-0.21895	0.463464	-7.91191	6.21E-05	0.001856	2.233157	0.572937	0.35399	0.218947	2	45028264	R	II		IGR	shore	IGR-shore	chr2:4502	rs1148572	7
cg23038277	-0.23015	0.426216	-7.91015	6.22E-05	0.001857	2.23154	0.541292	0.31114	0.230152	1	2.27E+08	R	I	ITPKB	Body	shelf	Body-shelf	chr1:2268	rs1149166	49;39;34;1
cg04192168	-0.29028	0.501771	-7.85974	6.5E-05	0.001896	2.185276	0.646912	0.356629	0.290283	15	64806741	R	II	ZNF609	Body	opensea	Body-opens			
cg26024874	-0.26288	0.472117	-7.85673	6.52E-05	0.001899	2.182502	0.603559	0.340674	0.262884	18	54541128	F	II	WDR7	Body	opensea	Body-opens		rs5352012	9;12
cg12909116	-0.24949	0.544182	-7.85121	6.55E-05	0.001903	2.177417	0.668925	0.419439	0.249486	11	1.02E+08	R	II		IGR	opensea	IGR-opense		rs7112218	46;35;24;9
cg06991787	-0.27061	0.610165	-7.82928	6.68E-05	0.001922	2.15719	0.745469	0.47486	0.270609	16	4094902	F	I	ADCY9	Body	opensea	Body-opens		rs5587014	22;23;31;51
cg04506765	-0.20974	0.2282	-7.81881	6.74E-05	0.00193	2.147507	0.333073	0.123328	0.209744	16	89601126	F	II	SPG7	Body	shelf	Body-shelf	chr16:896	rs5534166	5
cg09747445	-0.26338	0.478596	-7.81735	6.75E-05	0.001931	2.146165	0.610288	0.346903	0.263384	15	70387268	R	II	TLE3	Body	shore	Body-shore	chr15:703	rs5290753	46;45;42;37;36;23;11;10;8
cg26769700	-0.25094	0.481155	-7.80111	6.84E-05	0.001944	2.13113	0.606625	0.355686	0.250939	8	1.41E+08	F	I	TRAPPC9	Body	opensea	Body-opens		rs1480574	11;12;43
cg08899626	-0.22255	0.551364	-7.79594	6.88E-05	0.001949	2.126335	0.662636	0.440091	0.222545	4	16900199	R	II	LDB2	1stExon	opensea	1stExon-op		rs5307465	27
cg16352724	-0.29447	0.372014	-7.79041	6.91E-05	0.001953	2.121205	0.51925	0.224779	0.294471	6	1.43E+08	R	II	HIVEP2	5'UTR	opensea	5'UTR-oper		rs1170489	21
cg03448915	-0.22578	0.436192	-7.75833	7.11E-05	0.001981	2.091394	0.549081	0.323302	0.225779	16	66583078	F	I	TK2	Body	shore	Body-shore	chr16:665	rs5738853	18;21;27;42
cg21592975	-0.30658	0.588891	-7.75271	7.14E-05	0.001985	2.086161	0.742181	0.435601	0.30658	21	48053829	F	II		IGR	shore	IGR-shore	chr21:480	rs1472191	0;1;47
cg21093166	-0.20135	0.27827	-7.736	7.25E-05	0.001999	2.070583	0.378946	0.177595	0.201352	5	2752145	F	I	IRX2	TSS1500	island	TSS1500-isl	chr5:2748	rs5512808	4;6;48
cg12258042	-0.20536	0.288797	-7.73482	7.26E-05	0.002	2.06948	0.391476	0.186119	0.205357	8	4852036	R	I	CSMD1	5'UTR	island	5'UTR-islan	chr8:4848	rs5528899	44;15;6
cg24177970	-0.27278	0.418456	-7.72882	7.3E-05	0.002004	2.063878	0.554849	0.282064	0.272785	1	89127007	R	II	PKN2-AS1	Body	opensea	Body-opens		rs5410813	48;38
cg26176214	-0.20033	0.261037	-7.72836	7.3E-05	0.002005	2.063447	0.361201	0.160873	0.200328	2	2.4E+08	R	II	HDAC4	Body	opensea	Body-opens		rs5535244	10
cg00039170	-0.2282	0.311059	-7.71903	7.36E-05	0.002013	2.054723	0.425158	0.196959	0.228199	3	18480381	R	II	SATB1	TSS200	opensea	TSS200-ope		rs1143129	49;39;31
cg03018771	-0.20181	0.414741	-7.71122	7.41E-05	0.002022	2.047421	0.515648	0.313835	0.201813	1	27940619	R	II	FGR	Body	shore	Body-shore	chr1:2794	rs1863766	11;4;1
cg18282017	-0.25581	0.538233	-7.70296	7.46E-05	0.002029	2.039684	0.66614	0.410326	0.255814	2	2.08E+08	F	II	LOC10192	Body	opensea	Body-opens		rs5578146	33
cg21226442	-0.23509	0.537302	-7.69861	7.49E-05	0.002032	2.035605	0.654846	0.419758	0.235089	12	27088580	R	II	C12orf11	Body	shelf	Body-shelf	chr12:270	rs5462160	48;11
cg02208313	-0.22677	0.386015	-7.68756	7.57E-05	0.002042	2.025244	0.499399	0.272632	0.226767	17	80085332	F	II	CCDC57	Body	island	Body-islanc	chr17:800	rs5358530	1;9
cg16842449	-0.26166	0.499395	-7.65182	7.81E-05	0.002075	1.991636	0.630222	0.368567	0.261655	1	31233894	R	II		IGR	opensea	IGR-opense		rs1872358	14;13
cg20904475	-0.24468	0.566802	-7.64758	7.84E-05	0.00208	1.987632	0.689142	0.444463	0.244679	8	61823027	F	II		IGR	island	IGR-island	chr8:6182		
cg08905080	-0.22706	0.238634	-7.64566	7.86E-05	0.002081	1.985825	0.352166	0.125102	0.227064	16	89600764	R	II	SPG7	Body	shelf	Body-shelf	chr16:896	rs463930;r	48;37;34;1
cg08449791	-0.20524	0.487242	-7.64543	7.86E-05	0.002082	1.98561	0.589863	0.384622	0.205241	2	8465454	R	II	LINC00299	Body	opensea	Body-opens		rs7278106	50;14
cg02343604	-0.23102	0.299247	-7.60684	8.13E-05	0.002117	1.949141	0.414757	0.183738	0.231019	1	23884703	F	II	ID3	3'UTR	shore	3'UTR-shor	chr1:2388		
cg04479203	-0.23471	0.538644	-7.60287	8.16E-05	0.002122	1.945383	0.655999	0.421289	0.23471	8	61823106	R	II		IGR	shore	IGR-shore	chr8:6182		
cg05966255	-0.27435	0.510441	-7.59931	8.19E-05	0.002125	1.942009	0.647617	0.373266	0.274351	17	38758057	R	II		IGR	opensea	IGR-opense		rs5694599	1
cg01447281	-0.20008	0.240144	-7.57628	8.36E-05	0.002147	1.92015	0.340186	0.140102	0.200084	1	8482689	F	II	RERE	5'UTR	shore	5'UTR-shor	chr1:8483	rs1135996	34
cg21404980	-0.20683	0.254599	-7.57139	8.4E-05	0.002152	1.915502	0.358015	0.151184	0.206831	8	1.45E+08	F	II	ZC3H3	Body	shelf	Body-shelf	chr8:1446	rs5706929	5;8;9;37
cg14728071	-0.2067	0.197952	-7.54283	8.62E-05	0.002181	1.888295	0.301304	0.0946	0.206704	10	22030034	F	II	MLLT10	3'UTR	opensea	3'UTR-oper		rs1379468	0;3;33
cg11670533	-0.27971	0.551022	-7.53245	8.7E-05	0.002191	1.878384	0.690875	0.411169	0.279706	3	71421949	F	II	FOXP1	5'UTR	opensea	5'UTR-oper		rs1893613	0;12
cg03546163	-0.31854	0.403934	-7.53126	8.71E-05	0.002192	1.877256	0.563203	0.244665	0.318538	6	35654363	R	II	FKBP5	5'UTR	shore	5'UTR-shor	chr6:3565		
cg07086945	-0.2499	0.353281	-7.51582	8.83E-05	0.002208	1.862488	0.478231	0.22833	0.249901	6	1.09E+08	R	II		IGR	opensea	IGR-opense			
cg04297912	-0.28497	0.358143	-7.51564	8.83E-05	0.002208	1.862321	0.500628	0.215658	0.28497	11	1.02E+08	R	II	BIRC2	Body	opensea	Body-opens		rs5388000	41;10
cg25461186	-0.25941	0.477053	-7.51205	8.86E-05	0.002212	1.858879	0.606756	0.34735	0.259407	12	1.23E+08	F	II	MLXIP	Body	shore	Body-shore	chr12:122	rs5315780	49
cg25519284	-0.21686	0.291189	-7.49496	9E-05	0.002229	1.842501	0.399621	0.182757	0.216864	8	15397671	F	I	TUSC3	5'UTR	island	5'UTR-islan	chr8:1539	rs5562512	4;49
cg07278844	-0.25825	0.190133	-7.46598	9.24E-05	0.002262	1.814656	0.319256	0.061011	0.258245	6	1.09E+08	F	II		IGR	opensea	IGR-opense		rs5584787	9;33;39
cg09081994	-0.23659	0.577588	-7.46522	9.24E-05	0.002262	1.813933	0.695882	0.459295	0.236587	3	1.19E+08	F	II	CDGAP	TSS1500	shore	TSS1500-sh	chr3:1190	rs7570768	7;8
cg26708319	-0.2037	0.303683	-7.44903	9.38E-05	0.00228	1.798331	0.405534	0.201832	0.203701	4	1.12E+08	R	I	PITX2	Body	shore	Body-shore	chr4:1115	rs5578411	24
cg10555744	-0.30113	0.566389	-7.44695	9.4E-05	0.002282	1.796324	0.716954	0.415824	0.301129	1	25946258	R	II	MAN1C1	Body	shore	Body-shore	chr1:2594	rs1419674	27
cg23429235	-0.20709	0.306091	-7.41076	9.71E-05	0.002322	1.761352	0.409634	0.202549	0.207085	13	22103469	F	II	MICU2	Body	opensea	Body-opens		rs5740213	1
cg22855968	-0.23789	0.324173	-7.40985	9.72E-05	0.002322	1.760472	0.443119	0.205226	0.237893	6	1.35E+08	R	II		IGR	opensea	IGR-opense		rs3740639	37;22;2
cg03599009	-0.24119	0.325747	-7.40653	9.75E-05	0.002326	1.757253	0.446343	0.205152	0.241191	17	38501491	F	I	RARA	Body	island	Body-islanc	chr17:385	rs5400456	28
cg03275049	-0.30321	0.672588	-7.39563	9.85E-05	0.002339	1.74668	0.824193	0.520984	0.303209	11	1.28E+08	R	II		IGR	opensea	IGR-opense		rs5670371	48;7
cg18395917	-0.23093	0.335067	-7.38585	9.94E-05	0.00235	1.737189	0.450532	0.219602	0.23093	11	76033367	R	I		IGR	island	IGR-island	chr11:760	rs1426680	41;26
cg26099045	-0.22188	0.398859	-7.35295	0.000102	0.002385	1.705182	0.5098	0.287919	0.221881	2	64291800	F	II		IGR	opensea	IGR-opense		rs1890266	45
cg08320911	-0.24353	0.390929	-7.34901	0.000103	0.002389	1.701335	0.512694	0.269164	0.24353	11	48065094	F	II	PTPRJ	Body	opensea	Body-opens		rs5355307	3;20;36;40;45
cg02747184	-0.30964	0.519272	-7.34209	0.000103	0.002396	1.694583	0.674092	0.364453	0.309639	3	71422467	R	II	FOXP1	5'UTR	opensea	5'UTR-oper		rs1437129	43;14;1
cg22913471	-0.25956	0.343197	-7.33739	0.000104	0.002402	1.689991	0.472979	0.213415	0.259564	10	6635326	R	II		IGR	opensea	IGR-opense		rs1926465	16;15
cg08210036	-0.22701	0.252367	-7.33683	0.000104	0.002402	1.68945	0.36587	0.138864	0.227006	17	1775372	F	II	RPA1	Body	opensea	Body-opens			
cg13860006	-0.21571	0.288315	-7.32807	0.000105	0.002412	1.680888	0.396169	0.180461	0.215709	19	6741178	R	I	TRIP10	Body	island	Body-islanc	chr19:674	rs2012897	49;16
cg24617313	-0.20309	0.618549	-7.31683	0.000106	0.002426	1.669892	0.720092	0.517005	0.203087	20	57427146	R	I	GNAS	TSS1500	shore	TSS1500-sh	chr20:574	rs5401995	30;16;12
cg11115176	-0.2132	0.405826	-7.31602	0.000106	0.002426	1.6691	0.512425	0.299227	0.213198	10	1.16E+08	F	II	ABLIM1	Body	opensea	Body-opens		rs1827965	17
cg25011475	-0.21546	0.411911	-7.3142	0.000106	0.002428	1.667318	0.51964	0.304182	0.215458	10	88517608	F	I	BMPR1A	5'UTR	shore	5'UTR-shor	chr10:885	rs3749762	35
cg05678733	-0.21648	0.584522	-7.31006	0.000107	0.002431	1.663264	0.69276	0.476284	0.216476	5	43010511	F	II		IGR	opensea	IGR-opense		rs1119135	19
cg12476968	-0.20597	0.270774	-7.30956	0.000107	0.002431	1.662768	0.373758	0.167791	0.205966	15	34610829	R	II	SLC12A6	Body	opensea	Body-opens		rs1507518	45;35;28;8;5

cg20338754	-0.23121	0.288386	-7.30809	0.000107	0.002433	1.661335	0.40399	0.172781	0.231209	18	29677086	F	II	RNF138	Body	shelf	Body-shelf	chr18:2967		
cg02625086	-0.24751	0.341757	-7.29742	0.000108	0.002444	1.650874	0.46551	0.218005	0.247506	4	1.09E+08	F	II	LEF1	Body	shore	Body-shore	chr4:10908	rs5679971	0;1;45
cg17104283	-0.23399	0.349543	-7.29283	0.000108	0.002448	1.646361	0.466537	0.232549	0.233988	14	61946103	R	II	PRKCH	Body	opensea	Body-opens			
cg10642599	-0.23502	0.351588	-7.28832	0.000109	0.002453	1.641936	0.469096	0.234081	0.235016	10	74014931	F	I		IGR	opensea	IGR-opensea		rs5594281	8;9;41;44
cg23712342	-0.25163	0.315658	-7.28789	0.000109	0.002454	1.641513	0.441472	0.189844	0.251628	10	60936794	F	I	PHYHIPL	Body	island	Body-island	chr10:6093	rs3763659	16;37
cg19839221	-0.23684	0.378546	-7.28651	0.000109	0.002455	1.64016	0.496967	0.260125	0.236843	17	7787027	R	II	CHD3	TSS1500	shore	TSS1500-sh	chr17:7788		
cg21344746	-0.22207	0.451671	-7.26242	0.000111	0.002483	1.61646	0.562706	0.340636	0.22207	10	80831230	R	I	ZMIZ1	5'UTR	shore	5'UTR-shor	chr10:8083	rs1415845	48;34
cg11264678	-0.22107	0.439572	-7.2575	0.000112	0.002488	1.611614	0.550107	0.329036	0.221071	17	40466210	F	II	STAT3	3'UTR	shore	3'UTR-shor	chr17:4046	rs7545484	36
cg01106909	-0.2323	0.358256	-7.25628	0.000112	0.00249	1.610413	0.474404	0.242108	0.232296	11	1778627	R	II	HCCA2	Body	shelf	Body-shelf	chr11:1778	rs2015511	25;19;10;7;1
cg16125894	-0.25397	0.359089	-7.25117	0.000113	0.002496	1.605374	0.486072	0.232106	0.253967	1	1.81E+08	R	II		IGR	opensea	IGR-opensea		rs5658636	19;2
cg23896695	-0.28528	0.402451	-7.24359	0.000113	0.002505	1.59789	0.545089	0.259813	0.285276	8	1.29E+08	R	II	PVT1	Body	opensea	Body-opens			
cg26430287	-0.20083	0.400942	-7.23667	0.000114	0.002515	1.591057	0.501357	0.300527	0.20083	15	67439567	R	II	SMAD3	Body	opensea	Body-opens		rs5728921	9
cg25920520	-0.26088	0.513053	-7.22772	0.000115	0.002527	1.582208	0.643492	0.382614	0.260878	17	42147463	R	II	G6PC3	TSS1500	shore	TSS1500-sh	chr17:4214	rs1929657	14;11
cg09755397	-0.3078	0.517553	-7.2168	0.000116	0.002542	1.571401	0.671452	0.363653	0.307799	4	1.02E+08	R	II		IGR	opensea	IGR-opensea		rs5525733	6
cg26958839	-0.22722	0.461992	-7.18692	0.00012	0.002578	1.541767	0.575604	0.34838	0.227225	17	78637686	F	II	RPTOR	Body	opensea	Body-opens		rs5603864	14;27
cg01355089	-0.21605	0.304594	-7.18173	0.00012	0.002586	1.536605	0.412617	0.196571	0.216046	1	93495018	F	II		IGR	opensea	IGR-opensea		rs5750336	1;10;31;38;39
cg00686154	-0.21429	0.670012	-7.16864	0.000122	0.002603	1.523577	0.777156	0.562867	0.214288	14	68844858	R	II	RAD51B	Body	opensea	Body-opens		rs7405930	33
cg16375820	-0.2463	0.607084	-7.16682	0.000122	0.002606	1.521766	0.730237	0.483932	0.246305	5	55289001	F	II	IL6ST	5'UTR	shore	5'UTR-shor	chr5:55290	rs3774892	2;28;42
cg08995061	-0.25912	0.4465	-7.15095	0.000124	0.002626	1.505943	0.576061	0.316939	0.259121	2	1.74E+08	F	II	ZAK	Body	opensea	Body-opens		rs1841978	1;47
cg17494034	-0.22199	0.377633	-7.15032	0.000124	0.002626	1.505317	0.48863	0.266637	0.221992	1	12513497	F	II	VPS13D	Body	opensea	Body-opens		rs1817053	1
cg04635642	-0.26667	0.459053	-7.14045	0.000125	0.00264	1.49546	0.592388	0.325718	0.26667	16	12183938	R	I	SNX29	Body	opensea	Body-opens		rs5334388	8
cg07013955	-0.23785	0.346557	-7.1303	0.000126	0.002654	1.485316	0.46548	0.227635	0.237845	16	1595532	R	II	IFT140	Body	shelf	Body-shelf	chr16:1595	rs5735079	29;18;1
cg26797786	-0.27405	0.446485	-7.12814	0.000126	0.002656	1.483156	0.583511	0.309458	0.274053	3	18460713	R	II	SATB1	Body	opensea	Body-opens		rs5335991	13
cg00366435	-0.2222	0.328094	-7.12453	0.000127	0.002663	1.479551	0.439194	0.216994	0.2222	12	6896105	F	II		IGR	opensea	IGR-opensea		rs5296803	9
cg18057002	-0.27478	0.420573	-7.11778	0.000128	0.002672	1.472793	0.557961	0.283186	0.274776	16	87309765	R	II	LOC101928	Body	opensea	Body-opens		rs1825039	17;10;8;7;3
cg15506609	-0.20426	0.284082	-7.11735	0.000128	0.002672	1.472361	0.386214	0.18195	0.204265	7	27206073	R	I	HOXA9	TSS1500	island	TSS1500-isl	chr7:27207	rs5675550	44;35;25;10
cg10407807	-0.28513	0.449944	-7.10828	0.000129	0.002683	1.463269	0.592508	0.30738	0.285128	3	18481064	R	II	SATB1	TSS1500	opensea	TSS1500-op		rs5486008	6
cg14942588	-0.24647	0.346759	-7.10487	0.000129	0.002688	1.459852	0.469997	0.223522	0.246475	10	15183424	F	II	NMT2	ExonBnd	opensea	ExonBnd-op		rs7519120	39
cg09278089	-0.2653	0.531699	-7.09314	0.000131	0.002703	1.448071	0.664348	0.399049	0.265299	15	26092373	F	II	ATP10A	Body	opensea	Body-opens		rs3402081	0;1
cg19308612	-0.29349	0.34318	-7.09303	0.000131	0.002703	1.447959	0.489926	0.196434	0.293492	17	1775124	F	II	RPA1	Body	opensea	Body-opens		rs5517176	1
cg19130626	-0.2331	0.42681	-7.09218	0.000131	0.002704	1.447113	0.543359	0.310261	0.233098	12	11708829	F	II	LINC01252	Body	opensea	Body-opens		rs5644372	1;34
cg25596091	-0.23394	0.50497	-7.08485	0.000132	0.002715	1.439742	0.621942	0.387998	0.233944	8	59918888	F	II	TOX	Body	opensea	Body-opens		rs5704277	1
cg09970428	-0.23958	0.331029	-7.05257	0.000136	0.002762	1.40723	0.450819	0.211238	0.239581	6	1.19E+08	R	II		IGR	opensea	IGR-opensea			
cg21794396	-0.26754	0.462043	-7.04174	0.000137	0.002776	1.396292	0.595813	0.328272	0.267542	16	68315811	R	II	SLC7A6	Body	opensea	Body-opens			
cg06194950	-0.32817	0.5541	-7.04021	0.000137	0.002778	1.394743	0.718185	0.390015	0.32817	16	27244433	F	II	NSMCE1	Body	opensea	Body-opens		rs2000325	0;1;29
cg20817580	-0.2402	0.31074	-7.02993	0.000139	0.002795	1.384346	0.430842	0.190638	0.240204	17	37999368	F	II	IKZF3	Body	opensea	Body-opens			
cg24018814	-0.24597	0.290124	-7.02935	0.000139	0.002795	1.383758	0.41311	0.167138	0.245971	12	1.33E+08	F	II	GOLGA3	3'UTR	shore	3'UTR-shor	chr12:1333		
cg08209849	-0.22721	0.278923	-7.02857	0.000139	0.002797	1.382968	0.392527	0.165319	0.227208	1	64117325	R	II	PGM1	ExonBnd	opensea	ExonBnd-op		rs1858809	42;41;37;8;1
cg06946543	-0.22394	0.24399	-7.02259	0.00014	0.002805	1.376916	0.355958	0.132021	0.223937	13	76377366	F	II	LMO7	5'UTR	opensea	5'UTR-oper		rs5313187	29
cg16489771	-0.23317	0.453266	-7.01698	0.00014	0.002815	1.371238	0.569852	0.336681	0.233171	3	18480409	R	II	SATB1	TSS200	opensea	TSS200-ope		rs7494274	17
cg09151424	-0.24629	0.573634	-7.01155	0.000141	0.002822	1.365731	0.69678	0.450488	0.246291	3	71353997	F	II	FOXP1	TSS200	opensea	TSS200-ope		rs8015258	3
cg00928596	-0.26371	0.321324	-7.01114	0.000141	0.002823	1.365313	0.453178	0.18947	0.263707	16	14403022	F	I	MIR365-1	TSS200	opensea	TSS200-ope		rs5584491	0
cg25771013	-0.23251	0.355119	-7.00636	0.000142	0.002829	1.360465	0.471375	0.238864	0.232511	7	25989735	F	II	MIR148A	TSS200	shore	TSS200-sho	chr7:25990		
cg01534871	-0.22758	0.294682	-6.98659	0.000144	0.002858	1.340383	0.408472	0.180891	0.227581	1	19926850	F	II	C1orf151	Body	shelf	Body-shelf	chr1:19927	rs5690480	19;33
cg21395123	-0.20141	0.351485	-6.96621	0.000147	0.002888	1.319633	0.452189	0.250781	0.201408	4	89978388	R	I	FAM13A	TSS200	opensea	TSS200-ope		rs5417527	33;9;1
cg03722970	-0.22356	0.388985	-6.96362	0.000148	0.002892	1.316988	0.500766	0.277205	0.223562	5	40507277	R	II		IGR	opensea	IGR-opensea		rs5537411	45;36
cg24055239	-0.21027	0.255664	-6.95809	0.000148	0.002899	1.311354	0.3608	0.150527	0.210273	20	31099867	F	II	LOC10192	Body	opensea	Body-opens		rs5768951	12
cg05682528	-0.20702	0.291902	-6.95618	0.000149	0.002902	1.309403	0.395413	0.188391	0.207021	6	1.11E+08	F	II	SLC16A10	Body	opensea	Body-opens		rs5747659	1
cg15499265	-0.23249	0.651627	-6.95171	0.000149	0.00291	1.304844	0.767872	0.535383	0.232488	9	1.17E+08	R	II	AKNA	TSS1500	shelf	TSS1500-sh	chr9:11716	rs5341133	34;20
cg16824480	-0.21815	0.4295	-6.95148	0.000149	0.002911	1.304602	0.538573	0.320427	0.218146	17	74189931	R	II	RNF157	Body	opensea	Body-opens		rs7651864	28;2
cg25271243	-0.25989	0.371692	-6.94835	0.00015	0.002916	1.30141	0.501637	0.241747	0.25989	6	1.55E+08	F	II	OPRM1	Body	opensea	Body-opens		rs5330926	0;13;19;33
cg18981809	-0.21192	0.378801	-6.93565	0.000152	0.002934	1.288424	0.484759	0.272842	0.211917	5	1.78E+08	F	II	COL23A1	Body	opensea	Body-opens			
cg08038033	-0.26416	0.517233	-6.92019	0.000154	0.002956	1.272594	0.649312	0.385154	0.264158	3	71354056	R	II	FOXP1	5'UTR	opensea	5'UTR-oper			
cg13791460	-0.22329	0.499821	-6.91598	0.000155	0.002962	1.26828	0.611464	0.388178	0.223287	2	2.33E+08	R	II	DIS3L2	Body	opensea	Body-opens			
cg08497913	-0.21312	0.385547	-6.91408	0.000155	0.002965	1.266337	0.492104	0.278989	0.213115	12	1.32E+08	R	II	EP400	5'UTR	opensea	5'UTR-oper		rs5438797	1
cg17537714	-0.20871	0.720839	-6.90158	0.000157	0.002984	1.253502	0.825193	0.616486	0.208708	11	88104155	R	II		IGR	opensea	IGR-opensea		rs1130859	5;1
cg00010187	-0.22742	0.373245	-6.89497	0.000158	0.002992	1.246715	0.486954	0.259535	0.227418	10	43922867	R	II		IGR	opensea	IGR-opensea		rs1399963	29
cg23560640	-0.26858	0.496767	-6.88418	0.000159	0.003008	1.235617	0.631059	0.362475	0.268584	2	1.09E+08	F	II	CCDC138	Body	opensea	Body-opens		rs5736714	1;50
cg06945627	-0.2441	0.628241	-6.88116	0.00016	0.003013	1.232502	0.750291	0.50619	0.244101	5	35861023	F	II	IL7R	Body	opensea	Body-opens		rs1384825	0;1;45
cg14096828	-0.28376	0.562077	-6.87493	0.000161	0.003023	1.226087	0.703957	0.420197	0.28376	7	1.11E+08	R	II	LRRN3	1stExon	opensea	1stExon-op		rs5577189	38;6;2
cg03611307	-0.23966	0.414136	-6.86311	0.000163	0.003042	1.213902	0.533965	0.294307	0.239658	8	1.29E+08	R	II	MIR1205	TSS1500	opensea	TSS1500-o		rs7436509	8;39
cg08802841	-0.26432	0.650272	-6.85376	0.000164	0.003056	1.204257	0.782434	0.518111	0.264323	12	89748726	F	II		IGR	shore	IGR-shore	chr12:8974		
cg18193418	-0.22702	0.490788	-6.84639	0.000165	0.003069	1.196638	0.604297	0.377279	0.227018	10	72609729	R	II	SGPL1	Body	opensea	Body-opens		rs1400521	44;8;1
cg01832672	-0.26397	0.648192	-6.84628	0.000165	0.003069	1.196523	0.780178	0.516207	0.263971	12	1.23E+08	F	II	VPS37B	Body	opensea	Body-opens		rs5407435	49
cg11816048	-0.23743	0.350864	-6.84479	0.000166	0.003072	1.194989	0.469581	0.232148	0.237434	1	54750911	F	II	SSBP3	Body	opensea	Body-opens		rs1889195	1
cg15944766	-0.25333	0.381725	-6.84219	0.000166	0.003076	1.192296	0.508388	0.255061	0.253327	8	29210790	F	I		IGR	island	IGR-island	chr8:29210	rs5570301	14
cg04957307	-0.31223	0.558134	-6.83959	0.000167	0.00308	1.189611	0.714246	0.402021	0.312225	12	92180808	F	II		IGR	opensea	IGR-opensea		rs1484999	1;7
cg05931439	-0.22302	0.488311	-6.82919	0.000168	0.003096	1.178844	0.599821	0.3768	0.223021	14	23305957	R	I	MMP14	5'UTR	island	5'UTR-islan	chr14:2330	rs5623469	15;3;2
cg00641002	-0.30018	0.519653	-6.82529	0.000169	0.003102	1.174802	0.669742	0.369564	0.300178	3	18459838	R	II	SATB1	Body	opensea	Body-opens		rs1873370	1
cg01818220	-0.21087	0.284945	-6.82111	0.00017	0.00311	1.170468	0.390382	0.179507	0.210875	9	23821773	F	I	ELAVL2	1stExon	island	1stExon-isl	chr9:23820	rs5549186	3;25
cg09673015	-0.23265	0.398201	-6.81518	0.000171	0.003121	1.164318	0.514527	0.281876	0.232651	5	55302188	F	II		IGR	opensea	IGR-opensea			
cg24899545	-0.24381	0.405306	-6.79829	0.000173	0.003148	1.146782	0.527213	0.283399	0.243814	6	7112846	F	II	RREB1	5'UTR	shelf	5'UTR-shelf	chr6:71070	rs1917294	34
cg18273840	-0.22476	0.317858	-6.78542	0.000176	0.00317	1.13339	0.430238	0.205478	0.22476	5	45695643	R	I	HCN1	Body	island	Body-island	chr5:45695	rs1138600	32
cg14755660	-0.2581	0.305527	-6.7655	0.000179	0.003203	1.112627	0.434576	0.176478	0.258098	12	12635510	F	II	DUSP16	Body	opensea	Body-opens		rs5605189	0;39
cg10771443	-0.30214	0.475018	-6.76488	0.000179	0.003204	1.111981	0.62609	0.323946	0.302144	2	7018855	R	II	RSAD2	Body	opensea	Body-opens		rs1918724	1
cg00179026	-0.20322	0.312777	-6.75927	0.00018	0.003214	1.106127	0.414386	0.211167	0.203219	6	1.56E+08	F	II		IGR	opensea	IGR-opensea		rs5423218	30
cg16734384	-0.25338	0.515523	-6.75349	0.000181	0.003224	1.100091	0.642212	0.388834	0.253378	16	28186240	R	II	XPO6	Body	opensea	Body-opens		rs1415334	13;1
cg24491493	-0.21288	0.446226	-6.75036	0.000182	0.003229	1.096815	0.552668	0.339784	0.212884	6	1.59E+08	F	II	SYTL3	5'UTR	opensea	5'UTR-oper		rs5432134	0;1;20;49
cg22606224	-0.23803	0.400687	-6.74406	0.000183	0.00324	1.090235	0.519703	0.28167	0.238033	19	12266489	R	I	ZNF625	5'UTR	shore	5'UTR-shor	chr19:1226	rs1491627	37
cg16977720	-0.26831	0.614543	-6.73933	0.000184	0.00325	1.085284	0.748697	0.480389	0.268308	2	85105984	R	II	TRABD2A	Body	shore	Body-shore	chr2:85107		
cg04741118	-0.23601	0.366226	-6.7385	0.000184	0.003251	1.084412	0.484232	0.248221	0.236011	12	96720813	F	II	CDK17	Body	opensea	Body-opens		rs1379329	41
cg12189559	-0.24443	0.324811	-6.73313	0.000185	0.003261	1.078785	0.447025	0.202597	0.244427	4	1.54E+08	R	II		IGR	opensea	IGR-opensea		rs5464792	47;40;25;10
cg15122332	-0.24511	0.525477	-6.7323	0.000185	0.003262	1.077918	0.648031	0.402923	0.245108	18	809988	R	II	YES1	5'UTR	shore	5'UTR-shor	chr18:8110	rs7665027	35;30;9;2
cg12542255	-0.21894	0.661911	-6.72712	0.000186	0.003271	1.072491	0.771379	0.552442	0.218937	19	45976195	F	I	FOSB	Body	island	Body-island	chr19:4597	rs2005570	5;6;29
cg13401893	-0.29835	0.552703	-6.71948	0.000187	0.003284	1.064476	0.701877	0.403529	0.298348	6	30039432	R	I	RNF39	Body	island	Body-island	chr6:30038	rs2301752	16;15
cg16201957	-0.29926	0.529547	-6.70953	0.000189	0.003302	1.054038	0.679175	0.379918	0.299257	4	1.41E+08	F	II	MAML3	Body	opensea	Body-opens		rs5290159	0
cg10717869	-0.23551	0.573616	-6.70268	0.00019	0.003314	1.046837	0.69137	0.455862	0.235508	1	2.06E+08	R	II	SLC41A1	5'UTR	shore	5'UTR-shor	chr1:20578		
cg22535225	-0.24943	0.637169	-6.70215	0.000191	0.003315	1.046284	0.761885	0.512452	0.249434	9	1.04E+08	R	II	GRIN3A	3'UTR	opensea	3'UTR-oper		rs5368639	39
cg19238804	-0.22037	0.37941	-6.69896	0.000191	0.00332	1.042931	0.489594	0.269226	0.220368	17	57440226	R	II	YPEL2	Body	opensea	Body-opens		rs5653694	15
cg23073126	-0.20909	0.377444	-6.69826	0.000191	0.003321	1.042188	0.481989	0.272898	0.209091	5	52286130	F	II	ITGA2	Body	shore	Body-shore	chr5:52284		
cg01057656	-0.22884	0.387405	-6.68587	0.000194	0.003345	1.029153	0.501825	0.272984	0.228841	19	45281140	F	I	CBLC	5'UTR	island	5'UTR-islan	chr19:4528	rs5360793	13;29
cg15040252	-0.21596	0.339723	-6.68448	0.000194	0.003347	1.027683	0.447703	0.231743	0.215959	14	39656447	R	II	SEC23A	5'UTR	opensea	5'UTR-oper		rs5485990	38
cg13172001	-0.30684	0.532596	-6.67683	0.000195	0.00336	1.01963	0.686015	0.379177	0.306838	2	88390175	F	II	SMYD1	Body	opensea	Body-opens		rs5749572	1;47
cg02701505	-0.22821	0.322072	-6.67301	0.000196	0.003366	1.015596	0.436175	0.207968	0.228207	12	11709051	R	II	LINC01252	Body	opensea	Body-opens		rs1465336	2
cg19181419	-0.20816	0.455937	-6.67279	0.000196	0.003366	1.015366	0.560015	0.351859	0.208156	5	1.33E+08	F	II		IGR	opensea	IGR-opensea		rs3676897	0;6;19;33;47
cg10688481	-0.29743	0.498808	-6.67187	0.000196	0.003368	1.014396	0.647524	0.350092	0.297432	19	51858229	F	II	ETFB	TSS200	opensea	TSS200-ope		rs5729793	2;16;40
cg07110949	-0.22653	0.560736	-6.67109	0.000197	0.003369	1.013573	0.673999	0.447473	0.226526	4	1309878	F	I	MAEA	Body	shore	Body-shore	chr4:13097	rs6815464	23;30
cg05230130	-0.21368	0.506195	-6.66471	0.000198	0.003381	1.006834	0.613033	0.399357	0.213675	2	1.98E+08	R	II	ANKRD44	TSS1500	shore	TSS1500-sh	chr2:19817	rs5466399	30;1
cg04707519	-0.23134	0.364053	-6.66364	0.000198	0.003383	1.005703	0.479725	0.24838	0.231344	10	21799314	F	I		IGR	island	IGR-island	chr10:2179	rs1115909	43
cg00172603	-0.24027	0.400777	-6.66155	0.000198	0.003386	1.0035	0.520915	0.28064	0.240275	1	54822031	F	I	SSBP3	Body	island	Body-island	chr1:5482	rs1904627	30;33;37
cg01194310	-0.22813	0.407679	-6.66141	0.000198	0.003386	1.003348	0.521746	0.293612	0.228134	5	1.33E+08	R	II	TCF7	Body	opensea	Body-opens			
cg03864397	-0.25077	0.440762	-6.66126	0.000198	0.003386	1.003189	0.566147	0.315377	0.25077	2	2.02E+08	F	II	CASP10	5'UTR	opensea	5'UTR-oper		rs1433052	1;30;50
cg10351691	-0.21379	0.243729	-6.64353	0.000202	0.003418	0.984447	0.350626	0.136832	0.213794	11	24518557	F	I	LUZP2	5'UTR	island	5'UTR-islan	chr11:2451	rs1441074	14
cg13287909	-0.2382	0.44755	-6.63946	0.000203	0.003425	0.980135	0.56665	0.328451	0.238199	5	52286121	R	II	ITGA2	Body	shore	Body-shore	chr5:52284	rs1427264	19;13;10;2
cg27435333	-0.26431	0.502786	-6.63752	0.000203	0.003429	0.978084	0.634942	0.37063	0.264312	2	88357893	R	II		IGR	shelf	IGR-shelf	chr2:88358	rs1497410	6;2
cg07052063	-0.25722	0.347256	-6.63144	0.000204	0.00344	0.971638	0.475867	0.218644	0.257223	10	99255236	R	II	MMS19	Body	shelf	Body-shelf	chr10:9925	rs2900126	29
cg12067421	-0.26745	0.507919	-6.63082	0.000205	0.003441	0.970981	0.641643	0.374194	0.267449	1	2.03E+08	R	II		IGR	shelf	IGR-shelf	chr1:20324	rs1921741	51;41;5
cg25571973	-0.20287	0.674848	-6.61826	0.000207	0.003465	0.957656	0.776282	0.573414	0.202868	12	52347158	F	II	ACVR1B	TSS200	shore	TSS200-sho	chr12:5234		
cg14875171	-0.26488	0.41403	-6.61728	0.000207	0.003468	0.956618	0.546472	0.281588	0.264884	2	50574196	R	I	NRXN1	Body	island	Body-island	chr2:50574	rs1898733	15
cg18853591	-0.20352	0.356791	-6.61279	0.000208	0.003477	0.951853	0.45855	0.255032	0.203519	3	1.12E+08	F	II		IGR	opensea	IGR-opensea		rs1497487	0;1
cg15296664	-0.25455	0.658154	-6.60751	0.000209	0.003489	0.946242	0.785429	0.530879	0.25455	12	89748773	R	II		IGR	shore	IGR-shore	chr12:8974		
cg12060422	-0.27909	0.533622	-6.60069	0.000211	0.003501	0.938989	0.673165	0.39408	0.279085	10	1.03E+08	R	I	KAZALD1	1stExon	island	1stExon-isl	chr10:1028	rs1468453	42;16
cg13484324	-0.30826	0.630861	-6.60031	0.000211	0.003502	0.93858	0.78499	0.476733	0.308257	2	2.35E+08	R	II		IGR	opensea	IGR-opensea		rs5644946	10;4;1
cg01591526	-0.29317	0.595205	-6.5995	0.000211	0.003504	0.937725	0.741788	0.448622	0.293165	9	20373435	F	II	MLLT3	Body	opensea	Body-opens		rs5328726	0
cg09279736	-0.21702	0.473099	-6.59274	0.000212	0.003517	0.930529	0.58161	0.364588	0.217023	6	30039403	R	I	RNF39	Body	island	Body-island	chr6:30038	rs1175338	41;32
cg08913413	-0.20189	0.377479	-6.58982	0.000213	0.003523	0.927421	0.478424	0.276534	0.20189	2	16820369	F	II	FAM49A	5'UTR	opensea	5'UTR-oper		rs5277976	9;16;45;50
cg06916001	-0.24617	0.27575	-6.58529	0.000214	0.003532	0.922588	0.398834	0.152666	0.246168	16	14403004	F	I	MIR365-1	TSS200	opensea	TSS200-ope		rs5397252	12;18
cg05082111	-0.24179	0.321038	-6.57777	0.000216	0.003547	0.914575	0.441933	0.200143	0.24179	9	1.13E+08	F	II	AKAP2	5'UTR	opensea	5'UTR-oper			
cg06122405	-0.20872	0.65997	-6.57506	0.000216	0.003552	0.91168	0.764329	0.55561	0.208719	12	12557018	R	II	LOH12CR1	Body	opensea	Body-opens		rs1466548	1

cg21713991	-0.26227	0.449562	-6.57446	0.000216	0.003553	0.911042	0.580697	0.318428	0.262269	8	21718256	F	II		IGR	opensea	IGR-opense			
cg04116354	-0.25761	0.542616	-6.57307	0.000217	0.003556	0.909559	0.671422	0.413811	0.257612	1	26003643	R	II	MAN1C1	Body	opensea	Body-opens		rs5664331	45;10
cg07381806	-0.22987	0.419315	-6.5707	0.000217	0.003561	0.90703	0.534252	0.304378	0.229874	19	2094327	F	I	MOBKL2A	5'UTR	shore	5'UTR-shor	chr19:209	rs5515019	12;42
cg20625523	-0.20202	0.366662	-6.56828	0.000218	0.003567	0.904438	0.46767	0.265654	0.202015	2	64893849	R	II		IGR	opensea	IGR-opense		rs5688170	35;1
cg00728973	-0.23061	0.49463	-6.56459	0.000219	0.003575	0.900502	0.609937	0.379324	0.230613	17	37036448	F	II	LASP1	Body	opensea	Body-opens		rs226213	40
cg09277376	-0.27002	0.509997	-6.5603	0.000219	0.003582	0.895918	0.645007	0.374986	0.270022	1	47899676	F	II	MGC12982	Body	island	Body-islanc	chr1:4789	rs5296724	0;14
cg25027273	-0.2577	0.509086	-6.5551	0.000221	0.003594	0.890353	0.637936	0.380235	0.257701	15	1.02E+08	F	II	CHSY1	Body	opensea	Body-opens		rs3678788	1;2;3;14;26
cg11262921	-0.24955	0.512486	-6.55471	0.000221	0.003595	0.889942	0.637263	0.387709	0.249554	10	32264401	R	II		IGR	opensea	IGR-opense		rs5643623	10
cg21600258	-0.20906	0.337643	-6.55414	0.000221	0.003596	0.889328	0.442173	0.233113	0.20906	4	1.09E+08	R	II	LEF1	TSS1500	shore	TSS1500-sh	chr4:1090		
cg13271663	-0.26064	0.520264	-6.55048	0.000222	0.003603	0.885413	0.650586	0.389943	0.260644	15	70387303	R	II	TLE3	TSS200	shore	TSS200-sho	chr15:703	rs5405523	46;45;43;8
cg13478228	-0.21907	0.246328	-6.54694	0.000222	0.00361	0.881628	0.355861	0.136795	0.219066	18	70209432	R	I	CBLN2	5'UTR	island	5'UTR-islan	chr18:702	rs5719552	38;12
cg27468610	-0.23407	0.395908	-6.53928	0.000224	0.003626	0.873421	0.512945	0.278872	0.234073	7	1.52E+08	F	II	KMT2C	Body	opensea	Body-opens		rs1399824	0;8;20;43
cg18677148	-0.20908	0.344495	-6.53663	0.000225	0.003631	0.870579	0.449034	0.239957	0.209078	17	57712280	R	II	CLTC	Body	opensea	Body-opens		rs1166967	1
cg13303654	-0.26438	0.389143	-6.53497	0.000225	0.003635	0.868803	0.521334	0.256953	0.264381	10	31610754	F	II	ZEB1	Body	shore	Body-shore	chr10:316		
cg14107762	-0.27768	0.334272	-6.53448	0.000225	0.003636	0.868275	0.473111	0.195433	0.277677	9	1.18E+08	R	II	TNFSF8	Body	opensea	Body-opens		rs7915208	23;16
cg16053585	-0.23251	0.547852	-6.53417	0.000225	0.003637	0.867943	0.664107	0.431598	0.232509	2	86266870	F	II	POLR1A	Body	opensea	Body-opens		rs5775407	0;12;20
cg24213414	-0.24213	0.479913	-6.53102	0.000226	0.003643	0.864568	0.600975	0.35885	0.242125	12	92554979	F	II		IGR	opensea	IGR-opense		rs7728907	1;37
cg17413540	-0.24815	0.45223	-6.52782	0.000227	0.00365	0.861139	0.576304	0.328155	0.24815	7	40027286	R	II	CDK13	Body	opensea	Body-opens		rs1512246	46;42
cg00739471	-0.29539	0.591962	-6.51629	0.000229	0.003672	0.848751	0.739658	0.444266	0.295393	14	69415588	F	II	ACTN1	Body	opensea	Body-opens		rs3722395	1;38
cg15896892	-0.2381	0.335423	-6.51528	0.00023	0.003673	0.847675	0.454472	0.216374	0.238098	1	2.44E+08	F	II	ZNF238	TSS1500	shore	TSS1500-sh	chr1:2442	rs5642186	49
cg12552289	-0.20203	0.231801	-6.51351	0.00023	0.003677	0.845764	0.332816	0.130786	0.202031	11	1.33E+08	R	I	OPCML	TSS1500	island	TSS1500-isl	chr11:132	rs5702251	45;40;31;17
cg14384158	-0.21155	0.369086	-6.51228	0.00023	0.00368	0.844443	0.474863	0.263309	0.211554	9	1.4E+08	F	I	NELF	Body	shore	Body-shore	chr9:1403		
cg10925937	-0.20388	0.58739	-6.51078	0.000231	0.003683	0.842831	0.689332	0.485448	0.203883	4	1.06E+08	F	II		IGR	opensea	IGR-opense		rs1867280	1;8
cg22010886	-0.26694	0.475726	-6.50942	0.000231	0.003686	0.841376	0.609198	0.342254	0.266944	4	1.09E+08	F	II	LEF1	Body	opensea	Body-opens		rs5426138	0;1
cg24401737	-0.20591	0.494564	-6.50462	0.000232	0.003697	0.836209	0.597521	0.391607	0.205914	2	2.02E+08	F	II	CASP10	TSS200	opensea	TSS200-ope		rs5737536	37;48
cg14522718	-0.26529	0.359408	-6.50117	0.000233	0.003704	0.832491	0.492055	0.226761	0.265295	9	1.31E+08	F	I	SLC25A25	Body	opensea	Body-opens		rs5566265	2;18;24
cg07042743	-0.20422	0.438918	-6.49677	0.000234	0.003714	0.827758	0.541029	0.336807	0.204223	7	36693489	R	II	AOAH	Body	opensea	Body-opens		rs5434580	42;13
cg25984344	-0.22383	0.280864	-6.48942	0.000236	0.003729	0.819836	0.392781	0.168947	0.223834	10	1.29E+08	F	I	DOCK1	Body	island	Body-islanc	chr10:128	rs5618579	21
cg25884427	-0.26685	0.468515	-6.48817	0.000236	0.003732	0.818489	0.601942	0.335088	0.266854	1	54751035	F	II	SSBP3	Body	opensea	Body-opens		rs5280997	45
cg14286456	-0.22601	0.462214	-6.48791	0.000236	0.003732	0.81821	0.575217	0.34921	0.226007	1	8570225	F	II	RERE	Body	opensea	Body-opens		rs5723928	15;35;45
cg01618938	-0.27878	0.462227	-6.47583	0.000239	0.00376	0.805179	0.601618	0.322835	0.278782	3	72870430	F	II	SHQ1	Body	opensea	Body-opens		rs7754433	0;1;24;26;34;48
cg21626540	-0.25006	0.354012	-6.47569	0.000239	0.00376	0.805027	0.47904	0.228983	0.250058	2	10720198	R	II	NOL10	Body	opensea	Body-opens		rs3728602	41
cg09837977	-0.27486	0.588991	-6.47271	0.00024	0.003767	0.801812	0.726421	0.451561	0.27486	7	1.11E+08	R	II	LRRN3	5'UTR	opensea	5'UTR-open		rs5538823	49
cg05163268	-0.228	0.269309	-6.47185	0.00024	0.003768	0.800887	0.383308	0.15531	0.227998	5	1.8E+08	F	II		IGR	opensea	IGR-opense		rs1856850	0;2;22;37;42;48
cg09223486	-0.20565	0.495857	-6.46961	0.00024	0.003774	0.798463	0.598682	0.393031	0.205651	12	45763187	R	II	ANO6	Body	opensea	Body-opens		rs5354050	27
cg05949181	-0.25195	0.38576	-6.4694	0.000241	0.003774	0.798237	0.511736	0.259784	0.251951	22	39154591	R	II		IGR	shelf	IGR-shelf	chr22:391	rs5329882	33;17;8
cg27126310	-0.22418	0.465062	-6.46229	0.000242	0.003789	0.790558	0.577152	0.352971	0.224181	11	1.17E+08	R	II	SIK3	Body	opensea	Body-opens		rs1912984	46;28
cg06545367	-0.30427	0.610442	-6.44919	0.000246	0.003819	0.776376	0.762577	0.458306	0.304271	7	1.11E+08	F	II	LRRN3	5'UTR	opensea	5'UTR-open		rs5378047	1
cg17996919	-0.27722	0.379411	-6.44748	0.000246	0.003822	0.774523	0.518022	0.240799	0.277223	1	26024404	R	II	MAN1C1	Body	opensea	Body-opens		rs5575245	1
cg01362243	-0.21405	0.530231	-6.44586	0.000246	0.003825	0.772769	0.637255	0.423207	0.214048	2	45160389	R	II		IGR	shore	IGR-shore	chr2:4516	rs1503409	49;45
cg13451886	-0.2309	0.282784	-6.43872	0.000248	0.003842	0.765032	0.398232	0.167335	0.230897	17	33568791	R	II	SLFN5	TSS1500	shore	TSS1500-sh	chr17:335		
cg05485039	-0.24022	0.328452	-6.43833	0.000248	0.003843	0.764605	0.448561	0.208344	0.240217	6	1.12E+08	R	II	TRAF3IP2	TSS200	opensea	TSS200-ope		rs5685093	39;29;20;7;2
cg08360820	-0.24295	0.463945	-6.43526	0.000249	0.003849	0.761285	0.585421	0.342469	0.242953	10	11652640	F	I	USP6NL	5'UTR	shore	5'UTR-shor	chr10:116	rs5530911	3;22;26
cg13691247	-0.21371	0.221647	-6.43098	0.00025	0.003858	0.756631	0.328501	0.114792	0.213709	12	54071090	F	I	ATP5G2	TSS1500	island	TSS1500-isl	chr12:540	rs5431965	18;44;46
cg19679865	-0.26737	0.459443	-6.42299	0.000252	0.003875	0.747965	0.593127	0.325759	0.267368	1	8467904	F	II	RERE	5'UTR	opensea	5'UTR-open		rs1836582	18
cg27379109	-0.21106	0.426422	-6.42215	0.000252	0.003877	0.747052	0.531954	0.32089	0.211063	5	74907287	R	II		IGR	island	IGR-island	chr5:7490	rs5765296	38;29;25;21;15
cg15582951	-0.25664	0.365706	-6.4198	0.000253	0.003883	0.744496	0.494024	0.237389	0.256635	8	81411915	R	II	ZBTB10	Body	opensea	Body-opens			
cg14791398	-0.28027	0.489842	-6.41828	0.000253	0.003887	0.742843	0.629978	0.349706	0.280271	3	18462444	R	II	SATB1	Body	shelf	Body-shelf	chr3:1846	rs1435100	6
cg17210263	-0.20029	0.562878	-6.4182	0.000253	0.003887	0.74276	0.663021	0.462734	0.200287	1	2.03E+08	R	II		IGR	shore	IGR-shore	chr1:2032	rs5334492	1;31
cg27206867	-0.21508	0.411502	-6.41394	0.000255	0.003896	0.738121	0.519044	0.30396	0.215084	4	10119810	F	II	WDR1	TSS1500	shore	TSS1500-sh	chr4:1011	rs5477904	5;6;8;11;13;20;21;22;32;46;48
cg01763410	-0.21506	0.345724	-6.41083	0.000255	0.003904	0.734738	0.453251	0.238196	0.215055	18	31020209	R	II	CCDC178	TSS200	shore	TSS200-sho	chr18:310		
cg26921093	-0.25562	0.476244	-6.41021	0.000255	0.003905	0.734073	0.604053	0.348435	0.255618	17	63534688	R	II	AXIN2	Body	shore	Body-shore	chr17:635		
cg18266828	-0.22511	0.456635	-6.4102	0.000255	0.003905	0.734062	0.569191	0.344079	0.225112	17	46027572	F	II		IGR	opensea	IGR-opense			
cg03893901	-0.20354	0.484155	-6.40301	0.000257	0.003922	0.726231	0.585927	0.382384	0.203543	6	1.06E+08	R	II		IGR	opensea	IGR-opense		rs1809896	48;2
cg16240368	-0.20766	0.450979	-6.40238	0.000258	0.003923	0.72555	0.554811	0.347148	0.207663	1	1.19E+08	F	II	SPAG17	TSS1500	island	TSS1500-isl	chr1:1187	rs5306375	18
cg10215401	-0.27523	0.398076	-6.39965	0.000258	0.00393	0.722577	0.535692	0.26046	0.275232	8	1.31E+08	R	II		IGR	opensea	IGR-opense		rs5274408	26;2
cg03752513	-0.20201	0.2847	-6.39545	0.000259	0.003939	0.717999	0.385707	0.183694	0.202013	20	31098182	F	II	LOC10192	Body	opensea	Body-opens		rs5614551	22
cg00217293	-0.2066	0.514225	-6.39317	0.00026	0.003945	0.715513	0.617523	0.410927	0.206595	15	50200296	F	II	ATP8B4	Body	opensea	Body-opens		rs1907856	25;42;51
cg23824902	-0.27011	0.498565	-6.38876	0.000261	0.003955	0.710708	0.633621	0.363509	0.270112	1	9619882	F	II	SLC25A33	Body	opensea	Body-opens		rs5605887	25
cg25532922	-0.25591	0.620233	-6.38709	0.000262	0.003958	0.708876	0.74819	0.492276	0.255914	2	30453223	R	II	LBH	TSS1500	shore	TSS1500-sh	chr2:3045	rs1811024	13;5;2
cg11301253	-0.20128	0.304032	-6.38479	0.000262	0.003964	0.706375	0.404671	0.203394	0.201277	1	1.73E+08	F	II	TNFSF4	TSS1500	opensea	TSS1500-op		rs5520372	29;43;46
cg19695597	-0.23457	0.595637	-6.37835	0.000264	0.003978	0.699347	0.712922	0.478352	0.234569	14	62036992	F	II	FLJ22447	TSS1500	opensea	TSS1500-op			
cg02067461	-0.20171	0.401841	-6.37774	0.000264	0.003979	0.698681	0.502697	0.300986	0.201711	2	1.35E+08	F	II	MGAT5	Body	opensea	Body-opens		rs5719157	1;6
cg11651237	-0.22757	0.247552	-6.37198	0.000266	0.003993	0.692386	0.361335	0.133769	0.227566	15	27216529	F	I	GABRG3	5'UTR	island	5'UTR-islan	chr15:272	rs2839952	35
cg06897548	-0.22554	0.556589	-6.36629	0.000267	0.004005	0.686163	0.66936	0.443818	0.225542	22	39810706	F	II	TAB1	Body	opensea	Body-opens		rs5761073	47
cg07389228	-0.23521	0.295641	-6.35874	0.000269	0.004024	0.677907	0.413248	0.178035	0.235213	17	73685651	F	II	SAP30BP	Body	opensea	Body-opens		rs5428254	1;8;21;23
cg12696536	-0.23143	0.637012	-6.35055	0.000272	0.004043	0.66894	0.752726	0.521298	0.231428	2	10446128	R	II	HPCAL1	5'UTR	shore	5'UTR-shor	chr2:1044	rs5523380	15
cg22856324	-0.20367	0.239368	-6.341	0.000274	0.004066	0.658468	0.341205	0.137532	0.203673	2	72371255	F	I	CYP26B1	Body	island	Body-islanc	chr2:7237		
cg07071250	-0.20599	0.397397	-6.33649	0.000276	0.004076	0.65352	0.50039	0.294405	0.205985	18	21406132	R	II	LAMA3	Body	opensea	Body-opens		rs5442071	44;32
cg18794839	-0.21127	0.228504	-6.33162	0.000277	0.004088	0.648172	0.334139	0.12287	0.211269	1	2.15E+08	R	I	KCNK2	TSS1500	island	TSS1500-isl	chr1:2152	rs5336556	20
cg12910977	-0.27796	0.506068	-6.3237	0.000279	0.004105	0.639476	0.64505	0.367086	0.277964	5	1.38E+08	R	II	LRRTM2	3'UTR	opensea	3'UTR-open		rs5634303	27;14;10;1
cg24402372	-0.21589	0.363482	-6.31038	0.000283	0.004138	0.624821	0.471426	0.255537	0.215888	12	52997366	F	II		IGR	shelf	IGR-shelf	chr12:529		
cg12273325	-0.2263	0.351689	-6.31013	0.000283	0.004139	0.624544	0.464841	0.238536	0.226305	22	31689831	F	II	PIK3IP1	TSS1500	shelf	TSS1500-sh	chr22:316		
cg13643593	-0.24555	0.447882	-6.30305	0.000285	0.004156	0.616744	0.570658	0.325106	0.245552	4	1.54E+08	R	II	KIAA0922	Body	opensea	Body-opens		rs5767909	13
cg05728215	-0.21755	0.619732	-6.28751	0.00029	0.004195	0.599612	0.728505	0.510958	0.217547	9	1.31E+08	R	II		IGR	opensea	IGR-opense		rs5674422	18;1
cg12369481	-0.29658	0.561145	-6.28217	0.000291	0.004208	0.593713	0.709436	0.412854	0.296583	3	18459998	R	II	SATB1	Body	opensea	Body-opens			
cg25768456	-0.28953	0.35224	-6.28118	0.000292	0.00421	0.592626	0.497003	0.207478	0.289526	17	1775045	R	II	RPA1	Body	opensea	Body-opens		rs1729197	2
cg19996254	-0.24468	0.330102	-6.28083	0.000292	0.004211	0.592232	0.452441	0.207763	0.244678	10	1.16E+08	R	II	ABLIM1	5'UTR	opensea	5'UTR-open		rs5446480	19;12;2
cg24403567	-0.29297	0.380199	-6.28015	0.000292	0.004212	0.591483	0.526686	0.233712	0.292974	1	2.13E+08	F	II	NSL1	Body	opensea	Body-opens		rs5615185	3;49
cg25943763	-0.24555	0.395809	-6.27565	0.000293	0.004223	0.586507	0.518583	0.273035	0.245548	1	2.27E+08	F	II	ITPKB	Body	opensea	Body-opens			
cg00064733	-0.2686	0.328026	-6.27235	0.000294	0.004232	0.582863	0.462327	0.193725	0.268602	7	1.54E+08	R	I	DPP6	TSS200	island	TSS200-isla	chr7:1535	rs5316338	47;41;29;26
cg23668184	-0.23136	0.317618	-6.2721	0.000294	0.004233	0.582588	0.433299	0.201938	0.231361	2	5836713	F	I	SOX11	3'UTR	island	3'UTR-islan	chr2:5836	rs1926295	16
cg11400153	-0.20923	0.589894	-6.27129	0.000295	0.004235	0.581691	0.694511	0.485278	0.209233	4	1.54E+08	R	II	KIAA0922	Body	opensea	Body-opens		rs5458161	42;29;1
cg18353747	-0.23185	0.305127	-6.26742	0.000296	0.004245	0.577408	0.421055	0.1892	0.231855	13	30963558	R	II		IGR	opensea	IGR-opense		rs1929476	41;7;1
cg11815910	-0.21124	0.404607	-6.26397	0.000297	0.004253	0.573595	0.510228	0.298985	0.211243	6	1.67E+08	R	II	RNASET2	Body	shelf	Body-shelf	chr6:1673	rs5583974	35
cg23830082	-0.21136	0.446956	-6.25748	0.000299	0.00427	0.566406	0.552638	0.341275	0.211362	3	43196848	R	II		IGR	opensea	IGR-opense		rs4589902	49;46;27;1
cg11271054	-0.24158	0.521559	-6.25282	0.0003	0.004282	0.561241	0.64235	0.400767	0.241583	2	1.01E+08	R	II		IGR	opensea	IGR-opense		rs5599493	5;2
cg10261191	-0.20343	0.388053	-6.25244	0.000301	0.004283	0.560825	0.489768	0.286339	0.203429	11	1.25E+08	F	II	FEZ1	5'UTR	shore	5'UTR-shor	chr11:125	rs1478858	2;13;37
cg09451215	-0.20788	0.479454	-6.25164	0.000301	0.004285	0.559939	0.583396	0.375512	0.207884	6	82459960	F	II	FAM46A	Body	shore	Body-shore	chr6:8246	rs6175249	1
cg10959353	-0.20238	0.25144	-6.25028	0.000301	0.004288	0.558432	0.352631	0.150249	0.202383	2	2.29E+08	F	I	SPHKAP	TSS200	island	TSS200-isla	chr2:2290	rs5500997	8;32
cg02571465	-0.26647	0.460952	-6.25017	0.000301	0.004288	0.558302	0.594185	0.327718	0.266468	12	1213414	R	II	ERC1	Body	opensea	Body-opens		rs5749233	15;12;4
cg04836949	-0.21397	0.293641	-6.24929	0.000302	0.00429	0.557326	0.400624	0.186658	0.213965	13	46699875	R	II		IGR	opensea	IGR-opense		rs1147382	34
cg06525651	-0.20356	0.223674	-6.24632	0.000302	0.004297	0.554033	0.325455	0.121892	0.203563	10	1.29E+08	R	I	FAM196A	1stExon	island	1stExon-isl	chr10:128	rs5579427	48
cg08639389	-0.26179	0.53023	-6.24306	0.000304	0.004305	0.550425	0.661125	0.399335	0.26179	4	26995889	R	II	STIM2	Body	opensea	Body-opens		rs1401409	47;2
cg23133460	-0.2608	0.476272	-6.23808	0.000305	0.004319	0.544894	0.606672	0.345872	0.260801	12	32292908	R	II	BICD1	Body	opensea	Body-opens		rs1895771	33;30;15
cg17173975	-0.24026	0.415605	-6.23593	0.000306	0.004324	0.5425	0.535738	0.295473	0.240265	12	32292997	R	II	BICD1	Body	opensea	Body-opens		rs5743412	29;28
cg17806411	-0.27851	0.485183	-6.23255	0.000307	0.004334	0.538753	0.624439	0.345927	0.278512	3	18459963	F	II	SATB1	Body	opensea	Body-opens		rs1461314	38
cg03323696	-0.23037	0.388334	-6.22999	0.000308	0.004341	0.53591	0.503519	0.273149	0.23037	5	59189120	R	I	PDE4D	Body	island	Body-islanc	chr5:5918		
cg20587236	-0.26155	0.483463	-6.2294	0.000308	0.004342	0.535245	0.614237	0.352688	0.261549	12	1.1E+08	F	II	KCTD10	Body	opensea	Body-opens		rs5584205	0;21
cg03070297	-0.20955	0.212637	-6.22731	0.000309	0.004348	0.532932	0.317413	0.107861	0.209552	10	1.29E+08	F	I	DOCK1	Body	island	Body-islanc	chr10:128	rs5618579	26
cg10262387	-0.24604	0.306507	-6.22673	0.000309	0.004349	0.532283	0.429528	0.183486	0.246042	6	29855435	R	II	HLA-H	Body	island	Body-islanc	chr6:2985	rs3128881	51;45;36;28;12
cg06915826	-0.23494	0.267402	-6.22554	0.000309	0.004352	0.53096	0.384871	0.149934	0.234936	12	56329903	R	II	DGKA	5'UTR	shore	5'UTR-shor	chr12:563	rs5768502	17;15
cg04602992	-0.23887	0.329406	-6.221	0.000311	0.004363	0.525906	0.448842	0.209969	0.238873	17	62009628	R	II	CD79B	1stExon	opensea	1stExon-op		rs1999304	23;2;1
cg02125896	-0.20402	0.273441	-6.22072	0.000311	0.004363	0.525595	0.37545	0.171432	0.204018	12	6895774	R	II		IGR	opensea	IGR-opense		rs1431712	42;4
cg10182317	-0.20129	0.312516	-6.22026	0.000311	0.004364	0.525091	0.413159	0.211874	0.201286	6	1.23E+08	F	I	CLVS2	TSS200	island	TSS200-isla	chr6:1233	rs5591707	23
cg03217953	-0.24634	0.605137	-6.21193	0.000314	0.004385	0.515811	0.728309	0.481965	0.246345	20	52370216	F	II		IGR	opensea	IGR-opense		rs5724363	22
cg14905144	-0.30042	0.492519	-6.21121	0.000314	0.004386	0.515016	0.642731	0.342308	0.300423	15	70387094	R	II	TLE3	5'UTR	shore	5'UTR-shor	chr15:703	rs5513355	11;6;5
cg05715564	-0.2731	0.453888	-6.2107	0.000314	0.004387	0.514449	0.590436	0.317339	0.273096	9	1.31E+08	F	II	FAM102A	Body	opensea	Body-opens		rs5566514	0;12
cg26080847	-0.2088	0.244436	-6.21065	0.000314	0.004387	0.514385	0.348836	0.140037	0.208799	4	86396536	F	II	ARHGAP24	1stExon	opensea	1stExon-op		rs5723473	10;17;34;37
cg18808261	-0.25719	0.467277	-6.21053	0.000314	0.004387	0.514255	0.595871	0.338684	0.257187	3	18464935	R	II	SATB1	5'UTR	shore	5'UTR-shor	chr3:1846	rs5763052	21
cg24084780	-0.27708	0.314882	-6.21028	0.000314	0.004388	0.513974	0.453423	0.176342	0.277081	2	1.76E+08	R	II	CHN1	Body	opensea	Body-opens			
cg05491001	-0.25098	0.314324	-6.2064	0.000315	0.004397	0.50965	0.439813	0.188836	0.250977	4	62066539	F	I		IGR	island	IGR-island	chr4:6206	rs1386678	21;23
cg00084669	-0.27438	0.446921	-6.20463	0.000316	0.004401	0.507686	0.584111	0.309731	0.274381	3	1.89E+08	F	I	TPRG1-AS1	TSS200	island	TSS200-isla	chr3:1886	rs1496605	17;21;30;48;49
cg09086987	-0.26165	0.662328	-6.19868	0.000318	0.004417	0.501045	0.793152	0.531504	0.261648	3	18470432	F	II	SATB1	5'UTR	shelf	5'UTR-shelf	chr3:1846		
cg09847335	-0.21662	0.552263	-6.19811	0.000318	0.004419	0.500413	0.660571	0.443954	0.216617	17	62624233	R	II	SMURF2	Body	opensea	Body-opens		rs5721084	7
cg19798735	-0.25294	0.531965	-6.19789	0.000318	0.004419	0.500164	0.658435	0.405496	0.25294	7	1.11E+08	F	II	IMMP2L	Body	opensea	Body-opens		rs5512629	0;36;40
cg04408266	-0.20093	0.317409	-6.18841	0.000321	0.004444	0.489584	0.417872	0.216946	0.200925	1	2.12E+08	R	II	PPP2R5A	TSS1500	shore	TSS1500-sh	chr1:2124		
cg10181798	-0.24322	0.563019	-6.18362	0.000323	0.004456	0.484237	0.684629	0.441408	0.243221	14	64670300	R	II	SYNE2	Body	opensea	Body-opens		rs5808985	46;33;27
cg01851450	-0.2057	0.285438	-6.18116	0.000324	0.004462	0.481481	0.388287	0.182588	0.205699	6	1.47E+08	R	II	RAB32	TSS1500	shore	TSS1500-sh	chr6:1468		

cg26491791	-0.20293	0.199136	-6.17119	0.000327	0.00449	0.470331	0.300601	0.097671	0.20293	10	1.29E+08	R	I	DOCK1	TSS200	island	TSS200-isla	chr10:128		
cg24729879	-0.25028	0.474983	-6.16835	0.000328	0.004499	0.46715	0.600122	0.349845	0.250277	5	55288892	F	II	IL6ST	5'UTR	shore	5'UTR-shore	chr5:5529	rs7162410	7;14;19;21;33;51
cg05262463	-0.28427	0.469232	-6.1683	0.000328	0.004499	0.467103	0.611369	0.327095	0.284273	19	50056081	R	II		IGR	shelf	IGR-shelf	chr19:5005	rs5407943	50;41
cg24453118	-0.25822	0.457905	-6.16379	0.00033	0.00451	0.462043	0.587015	0.328796	0.258219	13	47229927	F	II	LRCH1	Body	opensea	Body-open		rs5586083	39;42
cg07364280	-0.21447	0.533395	-6.1637	0.00033	0.00451	0.461951	0.64063	0.426159	0.214471	15	99792732	F	II	TTC23	TSS1500	shore	TSS1500-sh	chr15:9979	rs1498677	11;18;23
cg16672818	-0.23534	0.648257	-6.15561	0.000333	0.004533	0.452878	0.765926	0.530588	0.235338	18	3606680	R	II	DLGAP1-AS	Body	opensea	Body-open		rs5372748	13;2;1
cg16910830	-0.21792	0.248343	-6.15109	0.000334	0.004544	0.447816	0.357302	0.139383	0.217919	7	1.46E+08	F	II	CNTNAP2	TSS200	island	TSS200-isla	chr7:1458	rs5280428	0
cg09397246	-0.20026	0.503607	-6.14848	0.000335	0.004551	0.444882	0.603739	0.403475	0.200264	15	91427361	F	I	FES	TSS1500	shore	TSS1500-sh	chr15:914		
cg12282552	-0.20678	0.316811	-6.14111	0.000338	0.004569	0.43661	0.420201	0.213422	0.20678	11	65321806	R	II	LTBP3	Body	island	Body-island	chr11:653		
cg01294808	-0.21547	0.446171	-6.13681	0.000339	0.004582	0.43178	0.553909	0.338434	0.215475	5	3599686	R	I	IRX1	Body	island	Body-island	chr5:3594	rs5522130	39;36;28;27;8
cg08864710	-0.26415	0.606346	-6.1357	0.00034	0.004587	0.430534	0.73842	0.474272	0.264148	14	69436506	R	II	ACTN1	Body	opensea	Body-open		rs1399096	51
cg07861448	-0.22854	0.421545	-6.13281	0.000341	0.004593	0.427282	0.535816	0.307273	0.228542	8	72757787	R	I	MSC	TSS1500	shore	TSS1500-sh	chr8:7275	rs5460552	40;13
cg05512157	-0.2794	0.374956	-6.13085	0.000341	0.004598	0.425077	0.514656	0.235256	0.2794	12	50901878	R	II	DIP2B	Body	shelf	Body-shelf	chr12:5090	rs5336185	41;2
cg12296772	-0.22933	0.334849	-6.12709	0.000343	0.004608	0.420852	0.449514	0.220183	0.229331	8	17271067	F	I	MTMR7	TSS200	island	TSS200-isla	chr8:1727	rs1407732	13;23;26;39;43;44;47;48
cg14046477	-0.20489	0.263973	-6.124	0.000344	0.004617	0.41738	0.366419	0.161528	0.204891	9	1.28E+08	R	I	NR6A1	Body	shore	Body-shore	chr9:1275	rs5424783	42;37;26;3
cg14603345	-0.20512	0.325102	-6.1192	0.000346	0.004631	0.411979	0.427662	0.222543	0.20512	20	11871375	F	I	BTBD3	TSS200	island	TSS200-isla	chr20:1187		
cg01264444	-0.23644	0.415948	-6.11834	0.000346	0.004633	0.411005	0.534166	0.29773	0.236436	7	1.5E+08	R	II	ZNF862	Body	opensea	Body-open		rs7727925	16;2
cg00627559	-0.22746	0.412511	-6.11717	0.000346	0.004636	0.409689	0.526239	0.298783	0.227456	3	1.02E+08	R	II		IGR	opensea	IGR-opensea		rs5591903	47
cg07013069	-0.20116	0.671219	-6.11225	0.000348	0.004649	0.404145	0.7718	0.570638	0.201162	12	1.17E+08	F	II	MED13L	Body	opensea	Body-open		rs5768726	35;50
cg12122293	-0.20868	0.45745	-6.10649	0.00035	0.004664	0.39765	0.561787	0.353112	0.208675	19	9920428	F	II		IGR	opensea	IGR-opensea		rs5637398	0
cg19501902	-0.24695	0.575547	-6.10065	0.000352	0.004682	0.391067	0.699024	0.452071	0.246953	3	1.11E+08	F	II	CD96	Body	opensea	Body-open			
cg17939889	-0.25525	0.431935	-6.09548	0.000354	0.004695	0.38523	0.559558	0.304312	0.255246	2	2.4E+08	F	I	HDAC4	Body	opensea	Body-open		rs1896678	26;27
cg00025518	-0.20241	0.298876	-6.09542	0.000354	0.004695	0.385158	0.400084	0.197669	0.202415	4	1.11E+08	R	II	CCDC109B	Body	opensea	Body-open		rs7984893	1
cg05678584	-0.20204	0.460458	-6.09449	0.000355	0.004698	0.384105	0.56148	0.359435	0.202044	20	13975883	F	I	MACROD2	TSS1500	island	TSS1500-isl	chr20:139		
cg11613902	-0.21595	0.489325	-6.08711	0.000358	0.00472	0.375771	0.597301	0.38135	0.21595	10	98156120	R	II	TLL2	Body	opensea	Body-open		rs5711910	49;31;7
cg07840532	-0.20687	0.342517	-6.07942	0.00036	0.004741	0.36708	0.445952	0.239083	0.206869	5	16618061	R	II	FAM134B	TSS1500	shore	TSS1500-sh	chr5:1661	rs5758605	7;2
cg15570017	-0.2025	0.498113	-6.07261	0.000363	0.004763	0.359372	0.599364	0.396861	0.202503	4	26275472	R	II		IGR	opensea	IGR-opensea		rs1687823	27;2
cg01624871	-0.2175	0.528462	-6.07064	0.000364	0.004769	0.357137	0.637211	0.419713	0.217498	11	6426894	R	II	APBB1	TSS1500	opensea	TSS1500-o			
cg27651321	-0.22401	0.444583	-6.06281	0.000367	0.004791	0.348272	0.556588	0.332578	0.22401	1	2.27E+08	F	II	ITPKB-IT1	Body	opensea	Body-open		rs1446679	1
cg19286755	-0.25981	0.406541	-6.06228	0.000367	0.004792	0.347662	0.536448	0.276634	0.259814	1	2.29E+08	R	II		IGR	opensea	IGR-opensea		rs5754440	1
cg02155398	-0.23724	0.248487	-6.05689	0.000369	0.004807	0.341561	0.36711	0.129865	0.237245	2	45160490	F	I		IGR	shore	IGR-shore	chr2:4516	rs5511215	1;22;30
cg21277869	-0.2088	0.378615	-6.05667	0.000369	0.004808	0.341305	0.483015	0.274216	0.208799	18	77163586	R	II	NFATC1	5'UTR	shelf	5'UTR-shelf	chr18:771	rs1164102	40;26;5
cg07127456	-0.22913	0.422012	-6.05289	0.000371	0.004819	0.337015	0.536576	0.307449	0.229127	16	66583081	F	II	TK2	Body	shore	Body-shore	chr16:665	rs5738853	15;18;24;39
cg02035448	-0.31281	0.531661	-6.0486	0.000372	0.004833	0.332145	0.688064	0.375259	0.312805	7	40027398	F	II	CDK13	Body	opensea	Body-open		rs1490287	0;47
cg13725767	-0.25701	0.601733	-6.04793	0.000373	0.004835	0.33139	0.730235	0.47323	0.257005	1	1.83E+08	R	II	NPL	5'UTR	opensea	5'UTR-ope		rs7306535	38;16
cg11909467	-0.23295	0.482597	-6.0476	0.000373	0.004836	0.331014	0.599074	0.36612	0.232954	8	1.33E+08	F	II		IGR	shelf	IGR-shelf	chr8:1329	rs7772168	32
cg09655719	-0.24919	0.612653	-6.04342	0.000374	0.004849	0.326265	0.737248	0.488059	0.249189	1	12513560	R	II	VPS13D	Body	opensea	Body-open		rs5554648	2
cg09664107	-0.26997	0.487053	-6.0432	0.000375	0.00485	0.326009	0.622037	0.35207	0.269968	12	6855032	R	II		IGR	shelf	IGR-shelf	chr12:685	rs1467705	34
cg14783703	-0.25179	0.517989	-6.03994	0.000376	0.00486	0.322311	0.643883	0.392096	0.251786	10	1.13E+08	R	II	RBM20	Body	opensea	Body-open		rs5372397	40;36;35;10;1
cg14824901	-0.21543	0.290926	-6.03598	0.000377	0.004872	0.317811	0.398643	0.183209	0.215434	1	2.1E+08	F	I	G0S2	TSS200	island	TSS200-isla	chr1:2098	rs5294138	15;38
cg12171165	-0.25476	0.597442	-6.03544	0.000378	0.004874	0.317191	0.724823	0.470061	0.254762	9	1.27E+08	F	II	DENND1A	Body	opensea	Body-open		rs5665526	21
cg10603275	-0.24496	0.374828	-6.03365	0.000378	0.004879	0.315157	0.49731	0.252346	0.244965	3	239665	F	I	CHL1	5'UTR	island	5'UTR-islan	chr3:2383	rs5710245	33
cg14020052	-0.25055	0.532287	-6.03186	0.000379	0.004885	0.313126	0.65756	0.407013	0.250547	2	2.4E+08	F	I	HDAC4	Body	opensea	Body-open		rs1896678	17;18
cg14357906	-0.20834	0.326122	-6.02632	0.000381	0.004903	0.306819	0.430291	0.221953	0.208338	2	68878598	R	II	PROKR1	Body	shelf	Body-shelf	chr2:6888	rs5669571	38;2
cg00122659	-0.25795	0.334182	-6.02372	0.000382	0.00491	0.303855	0.463155	0.205209	0.257946	1	1.8E+08	R	II		IGR	opensea	IGR-opensea			
cg13706362	-0.22631	0.477411	-6.02201	0.000383	0.004916	0.301914	0.590568	0.364255	0.226313	8	38178467	R	II	WHSC1L1	Body	opensea	Body-open		rs1491264	26;3
cg09183316	-0.21567	0.297087	-6.02191	0.000383	0.004916	0.301793	0.404924	0.189251	0.215672	12	58238232	F	II	CTDSP2	Body	shore	Body-shore	chr12:582	rs5437331	0;1;23;30
cg02794358	-0.20386	0.241527	-6.02029	0.000384	0.004921	0.299946	0.343456	0.139598	0.203858	2	54816818	R	II	SPTBN1	Body	opensea	Body-open		rs1431514	5
cg26155439	-0.21449	0.441734	-6.02009	0.000384	0.004922	0.299718	0.54898	0.334487	0.214493	19	47117785	F	II		IGR	opensea	IGR-opensea			
cg01145738	-0.21326	0.409446	-6.01526	0.000386	0.004938	0.294223	0.516077	0.302816	0.213261	11	1.1E+08	R	II	ZC3H12C	Body	shore	Body-shore	chr11:109	rs5272517	30;3
cg08936992	-0.22876	0.393643	-6.01363	0.000387	0.004944	0.292364	0.508025	0.27926	0.228765	20	31098005	F	II	LOC10192	Body	opensea	Body-open		rs1460195	1
cg02186614	-0.25974	0.271688	-6.01292	0.000387	0.004946	0.29155	0.401559	0.141817	0.259742	2	2.05E+08	F	II	CD28	Body	opensea	Body-open			
cg18681853	-0.21768	0.433949	-6.01285	0.000387	0.004946	0.291465	0.54279	0.325107	0.217684	2	2.25E+08	R	II	SERPINE2	5'UTR	opensea	5'UTR-ope		rs5550956	2
cg01628862	-0.24086	0.418077	-6.00677	0.000389	0.004967	0.284533	0.538509	0.297645	0.240864	4	89978381	R	II	FAM13A	TSS200	opensea	TSS200-ope		rs5417527	26;2
cg00786084	-0.20846	0.509622	-5.99996	0.000392	0.004988	0.276766	0.613852	0.405392	0.20846	3	45942460	F	II	CCR9	Body	opensea	Body-open		rs5619230	1;11
cg08440987	-0.24806	0.594502	-5.99935	0.000393	0.00499	0.276061	0.718532	0.470471	0.248062	11	1.28E+08	R	II		IGR	opensea	IGR-opensea		rs5448668	1;2;46
cg13961295	-0.25164	0.44875	-5.99868	0.000393	0.004992	0.275302	0.574569	0.322932	0.251636	2	23755963	R	II	KLHL29	5'UTR	opensea	5'UTR-ope		rs3477425	49;48;28;1
cg25310233	-0.27747	0.425361	-5.99814	0.000393	0.004993	0.274687	0.564094	0.286629	0.277465	1	31234437	F	II		IGR	opensea	IGR-opensea		rs5427421	0;1
cg02599325	-0.29251	0.638434	-5.99537	0.000394	0.005001	0.27152	0.784687	0.492181	0.292506	4	1.22E+08	F	II	TNIP3	TSS1500	opensea	TSS1500-o		rs1930318	14;18;21
cg00735667	-0.23759	0.316592	-5.9905	0.000396	0.005016	0.265952	0.435387	0.197798	0.237589	3	74663796	F	I		IGR	island	IGR-island	chr3:7466	rs5459523	7;8;49
cg26417310	-0.2254	0.354375	-5.9885	0.000397	0.005022	0.26366	0.467073	0.241677	0.225396	10	1.2E+08	R	II	CACUL1	Body	opensea	Body-open		rs5359287	44;33;18;13;6;2
cg20454887	-0.21271	0.463208	-5.98435	0.000399	0.005036	0.258916	0.569561	0.356854	0.212707	2	54935920	F	II		IGR	opensea	IGR-opensea		rs5355854	45
cg06101761	-0.21645	0.351545	-5.98185	0.0004	0.005044	0.256053	0.459769	0.243321	0.216448	6	5091721	R	II	LOC10012	Body	opensea	Body-open			
cg07143083	-0.21938	0.333826	-5.98176	0.0004	0.005044	0.25595	0.443519	0.224134	0.219385	7	70597921	F	I	WBSCR17	1stExon	island	1stExon-isl	chr7:7059	rs2021303	5;14;42;49
cg23495279	-0.20319	0.301316	-5.97889	0.000401	0.005054	0.252674	0.40291	0.199722	0.203188	8	4849827	F	I	CSMD1	Body	island	Body-island	chr8:4848	rs5624862	3;33
cg27594664	-0.22635	0.376661	-5.97806	0.000402	0.005057	0.251718	0.489836	0.263486	0.226349	8	1.28E+08	F	II	CASC19	Body	opensea	Body-open		rs1413371	34
cg02125365	-0.23075	0.450243	-5.97339	0.000404	0.005073	0.246366	0.565619	0.334868	0.230751	5	78813058	R	II		IGR	shelf	IGR-shelf	chr5:7880		
cg09741474	-0.2648	0.664348	-5.9653	0.000407	0.005099	0.237102	0.796749	0.531947	0.264802	11	6426183	F	II	APBB1	5'UTR	opensea	5'UTR-ope		rs5334424	0;23;50
cg04875456	-0.23992	0.624694	-5.96151	0.000409	0.005112	0.232753	0.744651	0.504736	0.239915	2	2.08E+08	R	II		IGR	opensea	IGR-opensea		rs5334485	14
cg16804428	-0.25391	0.662493	-5.95808	0.00041	0.005123	0.228812	0.78945	0.535535	0.253915	3	71367137	F	II	FOXP1	5'UTR	opensea	5'UTR-ope		rs5576592	0
cg23676767	-0.23388	0.333309	-5.95762	0.00041	0.005124	0.228284	0.450249	0.21637	0.233879	1	2.27E+08	R	II	ITPKB	Body	opensea	Body-open		rs7477428	7;2
cg08992499	-0.24956	0.611586	-5.95721	0.000411	0.005126	0.227815	0.736365	0.486806	0.249559	3	63956514	R	II	ATXN7	Body	opensea	Body-open		rs5339596	41;35;4
cg14945287	-0.26177	0.599205	-5.95463	0.000412	0.005134	0.224858	0.730091	0.46832	0.26177	14	74398293	R	II	ZNF410	3'UTR	opensea	3'UTR-ope		rs5461220	31
cg06930067	-0.21915	0.55813	-5.95143	0.000413	0.005144	0.221177	0.667707	0.448552	0.219155	14	68961273	R	II	RAD51B	Body	opensea	Body-open		rs1441378	45;23
cg05859076	-0.2541	0.4456	-5.95033	0.000414	0.005148	0.219917	0.572652	0.318549	0.254103	3	1.29E+08	F	II	RAB43	Body	shore	Body-shore	chr3:1288	rs1470665	1
cg23944804	-0.26775	0.31019	-5.94977	0.000414	0.00515	0.219269	0.444066	0.176314	0.267752	20	11871384	F	I	BTBD3	TSS200	island	TSS200-isla	chr20:1187		
cg19254152	-0.22809	0.465721	-5.94964	0.000414	0.00515	0.219125	0.579767	0.351675	0.228092	7	1083473	F	II	C7orf50	Body	shore	Body-shore	chr7:1084	rs5590196	48
cg06249604	-0.36276	0.427745	-5.94829	0.000415	0.005155	0.21757	0.609127	0.246362	0.362765	6	30039206	F	I	RNF39	Body	island	Body-island	chr6:3003	rs5289048	5;34;48
cg23729763	-0.25724	0.419952	-5.94703	0.000415	0.005159	0.216122	0.548571	0.291332	0.25724	11	1.3E+08	F	II	APLP2	Body	shore	Body-shore	chr11:129	rs5372480	9;19;28;35
cg17714025	-0.20272	0.282394	-5.94559	0.000416	0.005166	0.214	0.383755	0.181033	0.202721	11	30038619	R	I	KCNA4	TSS200	island	TSS200-isla	chr11:300		
cg17579667	-0.20827	0.290169	-5.94108	0.000418	0.00518	0.209289	0.394304	0.186034	0.20827	11	40315404	F	I	LRRC4C	5'UTR	opensea	5'UTR-ope			
cg21977218	-0.24333	0.479414	-5.93974	0.000421	0.005201	0.2013	0.60108	0.357748	0.243332	5	65443428	R	II	SFRS12	5'UTR	shelf	5'UTR-shelf	chr5:6543		
cg27005749	-0.22555	0.375456	-5.93099	0.000422	0.005212	0.197677	0.488232	0.262681	0.225551	8	1.42E+08	F	I	EIF2C2	Body	opensea	Body-open		rs5411174	8;12;14;19;20;51
cg25651219	-0.20887	0.551102	-5.92697	0.000424	0.005226	0.193048	0.655539	0.446665	0.208874	10	73895221	F	II	ASCC1	Body	opensea	Body-open		rs5700905	0;21
cg09770548	-0.26534	0.477914	-5.92649	0.000424	0.005227	0.192488	0.610585	0.345243	0.265342	14	1.05E+08	F	II	INF2	5'UTR	shore	5'UTR-shore	chr14:105		
cg10545142	-0.25188	0.3952	-5.92497	0.000425	0.005232	0.19074	0.521142	0.269257	0.251885	19	42070902	F	I	CEACAM21	5'UTR	opensea	5'UTR-ope		rs5530406	0;47
cg05805445	-0.21044	0.555397	-5.92185	0.000427	0.005241	0.187146	0.660614	0.450179	0.210435	4	1.24E+08	R	II	SPATA5	Body	opensea	Body-open		rs5599939	28;27
cg10296205	-0.22586	0.643773	-5.91762	0.000429	0.005256	0.182274	0.756701	0.530846	0.225855	6	1.59E+08	R	II	SYTL3	5'UTR	opensea	5'UTR-ope		rs7546550	36
cg23425059	-0.24324	0.467707	-5.9152	0.00043	0.005264	0.179482	0.589326	0.346088	0.243238	2	69727210	R	II	AAK1	Body	opensea	Body-open		rs1449936	24;10
cg09169291	-0.21668	0.410461	-5.91499	0.00043	0.005265	0.179242	0.518798	0.302123	0.216675	3	1.87E+08	F	II	ST6GAL1	5'UTR	opensea	5'UTR-ope		rs5301837	11
cg07517688	-0.20241	0.449902	-5.91247	0.000431	0.005273	0.176325	0.551108	0.348696	0.202411	14	70118078	F	II	SUSD6	5'UTR	opensea	5'UTR-ope		rs5596262	1;5;9
cg15890754	-0.20124	0.452518	-5.90994	0.000432	0.005282	0.173411	0.553136	0.351901	0.201235	2	1.73E+08	F	II	ITGA6	Body	shore	Body-shore	chr2:1732	rs5332535	28
cg06092290	-0.3054	0.594652	-5.89978	0.000437	0.005317	0.161678	0.747352	0.441952	0.3054	3	71180818	F	II	FOXP1	TSS1500	opensea	TSS1500-o		rs5779440	39;51
cg12204732	-0.22467	0.273614	-5.89916	0.000437	0.005319	0.160961	0.385951	0.161278	0.224673	10	1.29E+08	F	I	DOCK1	Body	island	Body-island	chr10:128	rs5618579	24
cg14257645	-0.20201	0.329704	-5.89734	0.000438	0.005325	0.158856	0.430711	0.228697	0.202014	9	1.33E+08	F	II	FNBP1	Body	opensea	Body-open		rs2296792	50
cg19645467	-0.20225	0.691002	-5.8892	0.000442	0.005352	0.149447	0.792129	0.589875	0.202254	12	1.22E+08	F	II	RNF34	Body	opensea	Body-open		rs1895460	42
cg23601905	-0.2171	0.306851	-5.88902	0.000442	0.005352	0.149232	0.415401	0.198301	0.2171	17	17661060	F	II	RAI1	5'UTR	opensea	5'UTR-ope		rs5729563	3;28;29
cg03129384	-0.22309	0.27356	-5.88706	0.000443	0.005359	0.14697	0.385106	0.162015	0.223092	10	1.29E+08	F	II	FAM196A	TSS1500	island	TSS1500-isl	chr10:128	rs5289970	20
cg15009294	-0.22963	0.547416	-5.88312	0.000445	0.005373	0.142403	0.662229	0.432603	0.229626	1	2.15E+08	F	II	CENPF	Body	opensea	Body-open			
cg05052463	-0.24799	0.537132	-5.88277	0.000445	0.005375	0.141998	0.661128	0.413137	0.247992	4	1.53E+08	R	II	FBXW7	Body	opensea	Body-open		rs5757336	30;4
cg07068761	-0.26561	0.613474	-5.87633	0.000448	0.005397	0.134543	0.746279	0.480669	0.26561	14	77505182	F	II		IGR	opensea	IGR-opensea		rs5633641	8;38;50
cg16298768	-0.2553	0.609055	-5.86825	0.000452	0.005425	0.125174	0.736704	0.481406	0.255298	22	44445889	R	II	PARVB	Body	opensea	Body-open			
cg27216937	-0.20578	0.757603	-5.86702	0.000453	0.00543	0.123749	0.860496	0.654711	0.205785	11	6425978	F	II	APBB1	Body	opensea	Body-open		rs5412162	47
cg04624110	-0.22954	0.427812	-5.86675	0.000453	0.005431	0.123426	0.542585	0.31304	0.229545	20	13976093	R	I	MACROD2	TSS200	island	TSS200-isla	chr20:139	rs3701202	16
cg05343227	-0.24801	0.466063	-5.8586	0.000457	0.00546	0.113967	0.590065	0.34206	0.248006	4	1.54E+08	R	II	KIAA0922	Body	opensea	Body-open		rs1900956	51;38;33;10;2
cg15564131	-0.24269	0.489203	-5.8581	0.000457	0.005461	0.113392	0.610546	0.367861	0.242686	3	18476925	R	II	SATB1	5'UTR	opensea	5'UTR-ope		rs5778360	6;1
cg19119032	-0.23002	0.281265	-5.85657	0.000458	0.005467	0.111615	0.396275	0.166255	0.230021	18	63418404	R	II	CDH7	5'UTR	island	5'UTR-islan	chr18:634		
cg13185413	-0.27615	0.322538	-5.8565	0.000458	0.005467	0.111533	0.460614	0.184463	0.276151	6	30039202	F	I	RNF39	Body	island	Body-island	chr6:3003	rs5289048	9;38
cg06062984	-0.21441	0.260606	-5.85627	0.000458	0.005468	0.111262	0.367813	0.1534	0.214413	3	22413960	R	I		IGR	island	IGR-island	chr3:2241		
cg02457461	-0.26146	0.544541	-5.85375	0.000459	0.005478	0.108342	0.675271	0.413812	0.261459	8	9913680	R	II	MSRA	Body	shore	Body-shore	chr8:9911	rs1400165	22
cg07721852	-0.20574	0.321175	-5.84536	0.000463	0.005506	0.098585	0.424046	0.218304	0.205742	11	1.19E+08	F	II		IGR	opensea	IGR-opensea		rs1812610	38;51
cg15998779	-0.22175	0.361646	-5.83618	0.000468	0.005539	0.087898	0.472519	0.250773	0.221746	3	1.6E+08	F	II		IGR	island	IGR-island	chr3:1597	rs5758817	50
cg16126286	-0.20239	0.268993	-5.83561	0.000468	0.005541	0.087239	0.370188	0.167798	0.20239	1	2.44E+08	R	I	SDCCAG8	Body	shore	Body-shore	chr1:2436	rs5620408	45
cg01768001	-0.26563	0.571495	-5.83266	0.00047	0.005553	0.0838	0.704311	0.43868	0.265631	1	24831147	F	II	RCAN3	5'UTR	shore	5'UTR-shore	chr1:2482		
cg26376706	-0.23	0.460256	-5.82992	0.000471	0.005563	0.080612	0.575258	0.345254	0.230004	2	2.31E+08	F	II		IGR	opensea	IGR-opensea		rs1905405	23
cg09107438	-0.20506	0.247213	-5.82671	0.000473	0.005574	0.076869	0.349744	0.144682	0.205062	12	56819060	R	II	TIMELESS	Body	opensea	Body-open		rs7247899	21
cg08313420	-0.26056	0.354244	-5.82355	0.000473	0.005575	0.076443	0.484526	0.223962	0.260564	7	6476110	F	II	DAGLB	Body	opensea	Body-open		rs5653868	27;51
cg03440588	-0.20419	0.498356	-5.82199	0.000475	0.005592	0.071363	0.600453	0.39626	0.204193	1	47900320	F	I	FOXD2	TSS1500	island	TSS1500-isl	chr1:4789	rs1511999	0;13
cg17038201	-0.25989	0.495003	-5.82184	0.000476	0.005592	0.071193	0.624947	0.365059	0.259888	9	1.29E+08	R	II	MVB12B	Body	opensea	Body-open			

cg16668359	-0.20323	0.301676	-5.81973	0.000477	0.0056	0.068724	0.40329	0.200063	0.203227	2	45159434	F	I		IGR	shore	IGR-shore	chr2:4515		
cg08191899	-0.24287	0.342352	-5.81734	0.000478	0.005609	0.065934	0.46379	0.220915	0.242875	21	35501530	R	II	MRPS6	Body	opensea	Body-opens		rs5337098	47;1
cg14413263	-0.22299	0.251289	-5.81277	0.00048	0.005626	0.060604	0.362784	0.139794	0.22299	5	1.28E+08	R	I	FBN2	TSS200	island	TSS200-isla	chr5:1278	rs5495461	36;35;24;22
cg04747245	-0.24448	0.440877	-5.81238	0.00048	0.005628	0.060147	0.563118	0.318637	0.244482	7	30197130	F	II	MTURN	Body	opensea	Body-opens		rs6173593	1;4;41
cg20023898	-0.20368	0.464712	-5.80914	0.000482	0.005639	0.056365	0.566553	0.362871	0.203682	16	67048469	F	II		IGR	island	IGR-island	chr16:670	rs5507543	18;26
cg06001519	-0.24998	0.323443	-5.80472	0.000485	0.005656	0.051197	0.448432	0.198454	0.249979	13	23500838	R	II		IGR	island	IGR-island	chr13:235	rs5741515	37;17
cg10173006	-0.2568	0.566838	-5.80382	0.000485	0.005659	0.05015	0.695237	0.438439	0.256799	1	1.18E+08	R	II		IGR	opensea	IGR-opense		rs5716484	21
cg05351767	-0.21052	0.314547	-5.80196	0.000486	0.005665	0.04797	0.419808	0.209287	0.210521	15	52527480	R	II	MYO5C	Body	opensea	Body-opens		rs7597529	14;1
cg03668323	-0.21283	0.347086	-5.79735	0.000488	0.005683	0.04259	0.453499	0.240673	0.212826	10	1.16E+08	F	II	ABLIM1	5'UTR	opensea	5'UTR-open		rs5744907	31
cg06818377	-0.23312	0.571771	-5.79381	0.00049	0.005697	0.038437	0.68833	0.455212	0.233118	8	56793504	R	II	LYN	5'UTR	shore	5'UTR-shor	chr8:5679	rs6474026	13
cg05572334	-0.24952	0.676674	-5.78131	0.000497	0.005742	0.023806	0.801432	0.551915	0.249516	7	55506671	R	II		IGR	opensea	IGR-opense		rs5383429	29;2
cg16078649	-0.28771	0.406825	-5.7799	0.000498	0.005748	0.022146	0.55068	0.262971	0.287709	6	30039466	R	I	RNF39	Body	island	Body-islanc	chr6:3003	rs2301752	50;49
cg15843262	-0.22834	0.239285	-5.77748	0.000499	0.005756	0.019309	0.353455	0.125115	0.22834	2	25473895	F	I	DNMT3A	Body	shore	Body-shore	chr2:2547	rs1153575	43;46
cg26817092	-0.20367	0.472425	-5.776	0.0005	0.005762	0.017576	0.574262	0.370587	0.203675	5	40672805	R	II		IGR	opensea	IGR-opense			
cg11512009	-0.22739	0.503571	-5.77466	0.000501	0.005768	0.016009	0.617267	0.389875	0.227392	17	38220694	F	II	THRA	5'UTR	shore	5'UTR-shor	chr17:382	rs1854091	1;14
cg02881866	-0.23306	0.488786	-5.77349	0.000501	0.005773	0.014633	0.605315	0.372258	0.233057	17	999650	R	I	ABR	Body	opensea	Body-opens		rs2257532	25;24;2
cg17048073	-0.27618	0.450902	-5.77205	0.000502	0.005778	0.012947	0.588992	0.312812	0.27618	8	23102091	F	II	CHMP7	5'UTR	shore	5'UTR-shor	chr8:2310	rs5714268	10;17
cg09463948	-0.23187	0.578872	-5.76934	0.000504	0.005788	0.009766	0.694808	0.462936	0.231873	9	1.33E+08	R	II	USP20	5'UTR	opensea	5'UTR-open		rs1823931	26;10
cg11874016	-0.24107	0.617535	-5.76918	0.000504	0.005789	0.009581	0.73807	0.497	0.24107	13	98112879	R	II	RAP2A	Body	opensea	Body-opens		rs1931078	35;13;7
cg16935794	-0.25021	0.512401	-5.76631	0.000505	0.005799	0.006212	0.637505	0.387296	0.250209	5	71525514	F	II	MRPS27	Body	opensea	Body-opens			
cg04664446	-0.24229	0.383427	-5.7626	0.000507	0.005813	0.001849	0.504574	0.26228	0.242295	1	1.51E+08	R	II	ARNT	Body	opensea	Body-opens		rs1405115	2
cg25354657	-0.22649	0.425172	-5.76147	0.000508	0.005817	0.000521	0.538419	0.311926	0.226494	11	1.3E+08	F	II	APLP2	Body	shelf	Body-shelf	chr11:129	rs1113778	16;18
cg21305555	-0.27079	0.54249	-5.76057	0.000509	0.005821	-0.00053	0.677886	0.407095	0.270791	12	1.08E+08	R	II	BTBD11	Body	opensea	Body-opens		rs5696865	2;1
cg11727282	-0.2094	0.370955	-5.75804	0.00051	0.00583	-0.0035	0.475654	0.266257	0.209397	9	23826368	F	I	ELAVL2	TSS1500	shelf	TSS1500-sh	chr9:2382	rs1169569	21
cg13977835	-0.21996	0.566027	-5.75576	0.000511	0.005839	-0.00618	0.676005	0.456049	0.219956	1	23883338	R	II		IGR	shore	IGR-shore	chr1:2388	rs5655141	30;2
cg23493133	-0.20568	0.228333	-5.75457	0.000512	0.005843	-0.00758	0.33117	0.125495	0.205675	19	50056114	F	II		IGR	shelf	IGR-shelf	chr19:500	rs1042194	9
cg00884731	-0.24375	0.286475	-5.75128	0.000514	0.005854	-0.01146	0.40835	0.1646	0.24375	9	85678133	F	I	RASEF	TSS200	island	TSS200-isla	chr9:8567	rs1505397	18;47
cg08633893	-0.26217	0.557729	-5.75066	0.000514	0.005858	-0.01218	0.688813	0.426644	0.262169	13	1E+08	R	II		IGR	opensea	IGR-opense		rs5704046	3;2
cg27419618	-0.29014	0.574654	-5.75011	0.000514	0.005859	-0.01282	0.719723	0.429585	0.290138	3	71180859	R	II	FOXP1	TSS1500	opensea	TSS1500-op		rs5779440	4
cg01785442	-0.2275	0.246718	-5.74811	0.000516	0.005867	-0.01518	0.360468	0.132968	0.2275	5	1.33E+08	R	II		IGR	opensea	IGR-opense		rs1904045	40;6
cg23095368	-0.21982	0.40709	-5.74562	0.000517	0.005876	-0.01811	0.517001	0.297178	0.219823	4	1.09E+08	R	II		IGR	opensea	IGR-opense			
cg15689796	-0.24811	0.505811	-5.74335	0.000518	0.005886	-0.02079	0.629867	0.381755	0.248111	14	93116574	F	II	RIN3	Body	opensea	Body-opens		rs1511067	33
cg22778135	-0.22349	0.443555	-5.73441	0.000523	0.005917	-0.03131	0.555301	0.331809	0.223492	17	46271818	F	II	SKAP1	Body	opensea	Body-opens		rs5516606	4;20;38;39
cg05732750	-0.22188	0.30129	-5.73263	0.000524	0.005924	-0.03341	0.412231	0.190348	0.221884	7	6704045	F	I		IGR	island	IGR-island	chr7:6703		
cg27515589	-0.23239	0.43888	-5.7281	0.000527	0.005944	-0.03875	0.555075	0.322685	0.23239	5	96259834	F	II		IGR	opensea	IGR-opense		rs5733643	4;11;12;49
cg25097419	-0.22693	0.306786	-5.72627	0.000528	0.005951	-0.04091	0.420249	0.193323	0.226926	5	79487997	F	II	SERINC5	Body	opensea	Body-opens		rs5562843	15;22;27
cg15354065	-0.21829	0.265644	-5.72397	0.00053	0.005959	-0.04363	0.374788	0.1565	0.218287	12	56327217	F	II	DGKA	5'UTR	shore	5'UTR-shor	chr12:563		
cg02714303	-0.20409	0.286158	-5.71757	0.000533	0.005983	-0.05118	0.388202	0.184114	0.204089	13	76334728	F	II	LMO7	TSS200	opensea	TSS200-ope		rs1828857	4;43
cg11179009	-0.21204	0.324797	-5.71625	0.000534	0.005989	-0.05274	0.430819	0.218776	0.212043	13	44880831	F	II		IGR	opensea	IGR-opense		rs1876204	45
cg22123784	-0.24134	0.367328	-5.71493	0.000535	0.005994	-0.0543	0.487998	0.246658	0.24134	8	1.44E+08	F	II		IGR	opensea	IGR-opense		rs5746854	38
cg25218716	-0.2306	0.22294	-5.71015	0.000538	0.006013	-0.05995	0.338241	0.107638	0.230604	20	22565228	R	I	FOXA2	5'UTR	island	5'UTR-islan	chr20:225		
cg21845390	-0.25613	0.42777	-5.70892	0.000538	0.006017	-0.0614	0.555836	0.299705	0.256131	13	40762260	F	II		IGR	opensea	IGR-opense		rs5358817	24;34
cg10665789	-0.20791	0.259916	-5.70834	0.000539	0.00602	-0.06209	0.363871	0.155962	0.207909	1	2.27E+08	F	II	ITPKB	Body	shelf	Body-shelf	chr1:2269	rs7547263	1;8
cg17250770	-0.22599	0.326422	-5.70204	0.000543	0.006046	-0.06954	0.439418	0.213427	0.225991	8	22500401	R	II	FLJ14107	TSS1500	opensea	TSS1500-op		rs5396728	50;43
cg04597393	-0.22043	0.548796	-5.70167	0.000543	0.006048	-0.06997	0.659012	0.438579	0.220433	8	1.42E+08	F	I		IGR	opensea	IGR-opense		rs5582902	17
cg24884214	-0.21551	0.483488	-5.70161	0.000543	0.006048	-0.07004	0.591245	0.375732	0.215513	2	2.35E+08	R	II		IGR	opensea	IGR-opense		rs5551668	9;6;3
cg06478504	-0.24522	0.432262	-5.7	0.000544	0.006054	-0.07195	0.554872	0.309653	0.24522	2	2.4E+08	F	I	HDAC4	Body	opensea	Body-opens		rs5449852	0;11;19
cg02148787	-0.20738	0.296517	-5.69905	0.000544	0.006058	-0.07307	0.400208	0.192827	0.207382	5	1.33E+08	R	II	TCF7	TSS1500	island	TSS1500-isl	chr5:1334	rs5321744	7;2
cg22821324	-0.2082	0.237807	-5.69804	0.000545	0.006062	-0.07427	0.341909	0.133706	0.208203	4	1.77E+08	R	II	GPM6A	5'UTR	island	5'UTR-islan	chr4:1769	rs1509373	50;33;24;12
cg00042489	-0.27441	0.487711	-5.68999	0.00055	0.006096	-0.0838	0.624916	0.350506	0.27441	1	2.27E+08	R	II	PSEN2	Body	opensea	Body-opens		rs1147786	15
cg00086577	-0.20215	0.519889	-5.68482	0.000553	0.006118	-0.08992	0.620963	0.418814	0.20215	6	32122894	F	II	PPT2	Body	shore	Body-shore	chr6:3212	rs1487888	1
cg07382454	-0.21671	0.278478	-5.68382	0.000554	0.006121	-0.09111	0.386834	0.170123	0.216711	19	19383976	R	I	TM6SF2	1stExon	island	1stExon-isl	chr19:193	rs1997296	20;9
cg10575067	-0.29663	0.580499	-5.6838	0.000554	0.006121	-0.09114	0.728812	0.432186	0.296625	19	39896215	R	II	ZFP36	TSS1500	shore	TSS1500-sh	chr19:398		
cg25557432	-0.20231	0.230906	-5.68269	0.000554	0.006125	-0.09246	0.332059	0.129753	0.202306	20	13976117	R	I	MACROD2	TSS200	island	TSS200-isla	chr20:139	rs3701202	40
cg11148130	-0.23241	0.363632	-5.68202	0.000555	0.006128	-0.09325	0.479839	0.247424	0.232415	3	73674068	F	II	PDZRN3	5'UTR	island	5'UTR-islan	chr3:7367	rs5781775	8;9;18;19;49
cg15047408	-0.2457	0.588094	-5.67852	0.000557	0.006142	-0.09739	0.710946	0.465241	0.245705	20	42427235	R	II		IGR	opensea	IGR-opense		rs1130644	9;6;4;2
cg26001902	-0.22107	0.252111	-5.67536	0.000559	0.006154	-0.10115	0.362644	0.141578	0.221066	2	1.09E+08	F	I	SLC5A7	TSS200	island	TSS200-isla	chr2:1086		
cg09375033	-0.21077	0.578478	-5.67487	0.000559	0.006156	-0.10173	0.683862	0.473094	0.210768	3	1.38E+08	F	II	DZIP1L	5'UTR	shore	5'UTR-shor	chr3:1378	rs3755067	40
cg01776373	-0.20133	0.31825	-5.67283	0.00056	0.006164	-0.10415	0.418914	0.217587	0.201327	11	46353961	F	II	DGKZ	TSS1500	shore	TSS1500-sh	chr11:463	rs5458661	45
cg13260278	-0.21299	0.542626	-5.67238	0.000561	0.006166	-0.10469	0.649119	0.436132	0.212987	10	1.21E+08	R	II	RGS10	Body	opensea	Body-opens		rs1397039	44;29
cg24490843	-0.23974	0.580821	-5.67097	0.000562	0.006172	-0.10636	0.70069	0.460953	0.239737	17	38752624	R	II		IGR	opensea	IGR-opense		rs1294446	41;2;1
cg19930116	-0.22706	0.505137	-5.66949	0.000562	0.006179	-0.10812	0.618667	0.391607	0.22706	14	50809588	F	II	CDKL1	Body	opensea	Body-opens		rs1157083	44;49
cg22336648	-0.26334	0.572259	-5.66816	0.000563	0.006184	-0.10969	0.703931	0.440587	0.263344	3	18480706	R	II	SATB1	TSS1500	opensea	TSS1500-op			
cg02439964	-0.25341	0.460493	-5.66638	0.000564	0.006191	-0.1118	0.587196	0.333789	0.253407	13	21276788	F	II	IL17D	TSS1500	shore	TSS1500-sh	chr13:212	rs1826859	6;21;27;47
cg16761216	-0.22512	0.498628	-5.66446	0.000566	0.006198	-0.11409	0.611189	0.386067	0.225121	4	38664394	F	II	KLF3	TSS1500	shore	TSS1500-sh	chr4:3866	rs5728778	8;39
cg14160787	-0.22429	0.466101	-5.66436	0.000566	0.006199	-0.1142	0.578245	0.353957	0.224288	21	43818241	F	II	TMPRSS3	TSS1500	opensea	TSS1500-op		rs1440926	0;14
cg11699576	-0.23955	0.436262	-5.66401	0.000566	0.006201	-0.11463	0.556036	0.316489	0.239547	14	99681881	F	II	BCL11B	Body	opensea	Body-opens		rs5276442	30
cg24069928	-0.23017	0.505099	-5.66324	0.000566	0.006204	-0.11554	0.620187	0.390012	0.230175	6	1.67E+08	F	II	RNASET2	Body	shelf	Body-shelf	chr6:1673	rs5397314	0
cg16792800	-0.20329	0.273953	-5.65898	0.000569	0.006222	-0.1206	0.375597	0.172309	0.203289	13	93879361	F	II	GPC6	1stExon	island	1stExon-isl	chr13:938	rs5622573	8;14;29;42
cg11690122	-0.22852	0.375005	-5.6588	0.000569	0.006222	-0.12082	0.489266	0.260745	0.228521	10	89825036	F	II		IGR	opensea	IGR-opense		rs5585300	0;1;12;16;19
cg20478129	-0.21658	0.202309	-5.65843	0.000569	0.006224	-0.12126	0.310598	0.09402	0.216578	14	27067372	F	I	NOVA1	TSS1500	shore	TSS1500-sh	chr14:270		
cg04346257	-0.21335	0.328543	-5.65828	0.00057	0.006224	-0.12143	0.435216	0.221871	0.213346	17	75430923	R	II	SEPT9	Body	opensea	Body-opens		rs5603254	2;1
cg18651870	-0.21911	0.38394	-5.65557	0.000571	0.006236	-0.12466	0.493496	0.274383	0.219113	18	70535005	R	I	NETO1	TSS200	island	TSS200-isla	chr18:705	rs5594633	20;10;1
cg22132509	-0.22272	0.551094	-5.65225	0.000573	0.006249	-0.12861	0.662454	0.439733	0.222721	3	1.6E+08	F	II	PPM1L	Body	shore	Body-shore	chr3:1604		
cg08577982	-0.22231	0.515588	-5.65136	0.000574	0.006253	-0.12967	0.626741	0.404435	0.222306	15	91164193	R	II	CRTC3	Body	opensea	Body-opens		rs7689413	48
cg20674738	-0.23349	0.286243	-5.65133	0.000574	0.006253	-0.1297	0.402987	0.1695	0.233487	1	26453892	R	II		IGR	opensea	IGR-opense		rs5754987	14
cg03343571	-0.27137	0.420383	-5.65084	0.000574	0.006255	-0.13028	0.556066	0.284699	0.271367	6	30039175	F	I	RNF39	Body	island	Body-islanc	chr6:3003	rs5289048	36
cg20506347	-0.23038	0.411301	-5.64944	0.000575	0.00626	-0.13195	0.526489	0.296114	0.230375	3	71355161	F	II	FOXP1	5'UTR	opensea	5'UTR-open		rs5614775	0;1
cg19620333	-0.21408	0.340585	-5.64861	0.000576	0.006264	-0.13294	0.447626	0.233545	0.214081	15	81596332	F	II	IL16	Body	opensea	Body-opens		rs5726740	0;2;10;28
cg18876294	-0.24379	0.37098	-5.64694	0.000577	0.00627	-0.13493	0.492877	0.249084	0.243793	11	1.19E+08	F	II	C2CD2L	Body	opensea	Body-opens		rs5429321	28;51
cg14997450	-0.21892	0.476517	-5.64669	0.000577	0.006271	-0.13522	0.585977	0.367057	0.21892	2	2.17E+08	F	II	PECR	TSS1500	shore	TSS1500-sh	chr2:2169	rs5602146	1
cg03735233	-0.20969	0.198686	-5.64498	0.000578	0.006279	-0.13727	0.303529	0.093843	0.209686	16	68318873	R	II	SLC7A6	Body	opensea	Body-opens		rs7453499	47;15
cg18919209	-0.26307	0.556032	-5.6413	0.00058	0.006295	-0.14165	0.687569	0.424495	0.263074	11	96012039	F	II	MAML2	Body	opensea	Body-opens			
cg06222774	-0.22031	0.400394	-5.64103	0.000581	0.006296	-0.14197	0.510549	0.29024	0.220309	8	66743912	F	II	PDE7A	Body	opensea	Body-opens		rs5523643	46
cg18343957	-0.28923	0.314609	-5.63911	0.000582	0.006305	-0.14426	0.459225	0.169993	0.289232	14	48144197	R	I	MDGA2	TSS200	island	TSS200-isla	chr14:481	rs5557021	27
cg04492228	-0.21342	0.257006	-5.63805	0.000583	0.00631	-0.14552	0.363716	0.150297	0.213419	10	8101513	R	II	GATA3	Body	shore	Body-shore	chr10:810	rs5407739	40;30;16
cg09676794	-0.22173	0.386719	-5.63752	0.000583	0.006314	-0.14616	0.497585	0.275853	0.221732	13	1E+08	R	II	CLYBL	Body	shore	Body-shore	chr13:100	rs7982636	13
cg07916304	-0.21543	0.401197	-5.63558	0.000584	0.006321	-0.14846	0.508912	0.293481	0.215431	2	25502340	R	II	DNMT3A	Body	shore	Body-shore	chr2:2549	rs5486788	48;1
cg25779483	-0.25325	0.548899	-5.63387	0.000585	0.006327	-0.1505	0.675526	0.422272	0.253253	4	89978300	F	II	FAM13A	1stExon	opensea	1stExon-op		rs5742794	7
cg16484042	-0.23227	0.419958	-5.63202	0.000586	0.006335	-0.15271	0.536092	0.303825	0.232267	16	14403405	R	II		IGR	opensea	IGR-opense		rs1823188	45;8
cg17775490	-0.22123	0.47382	-5.63104	0.000587	0.00634	-0.15388	0.584435	0.363206	0.22123	20	45179354	F	II	C20orf123	TSS200	opensea	TSS200-ope		rs1021181	24;29
cg13346003	-0.23007	0.316035	-5.62894	0.000588	0.006349	-0.15638	0.431072	0.200998	0.230074	6	1.26E+08	F	II	HDDC2	TSS1500	shore	TSS1500-sh	chr6:1256	rs5649209	1
cg26757820	-0.25663	0.28071	-5.62789	0.000589	0.006353	-0.15764	0.409023	0.152396	0.256627	10	76349278	R	II	ADK	Body	opensea	Body-opens		rs5752307	30;19
cg23357533	-0.2349	0.404181	-5.62789	0.000589	0.006353	-0.15764	0.521629	0.286732	0.234897	13	42044673	F	II	C13orf15	3'UTR	opensea	3'UTR-open		rs5398659	0;46
cg08693443	-0.23609	0.512969	-5.62677	0.00059	0.006357	-0.15897	0.631012	0.394927	0.236085	7	5537126	R	II	FBXL18	Body	shelf	Body-shelf	chr7:5539	rs1881757	11
cg11280255	-0.21505	0.390537	-5.62499	0.000591	0.006365	-0.1611	0.49806	0.283013	0.215047	10	94063299	F	II	MARCH5	Body	opensea	Body-opens		rs5328194	0;27
cg11420364	-0.22165	0.442834	-5.6214	0.000593	0.00638	-0.16539	0.553659	0.332008	0.22165	8	38822874	F	II	PLEKHA2	Body	opensea	Body-opens			
cg12776892	-0.31081	0.312992	-5.61898	0.000595	0.006392	-0.16828	0.468398	0.157586	0.310812	9	1.15E+08	F	II	MIR3134	Body	opensea	Body-opens			
cg12838263	-0.20488	0.391923	-5.61737	0.000596	0.0064	-0.1702	0.494362	0.289485	0.204876	14	1.04E+08	R	II	ZFYVE21	Body	shelf	Body-shelf	chr14:104		
cg23514529	-0.21276	0.276584	-5.61732	0.000596	0.0064	-0.17026	0.382966	0.170202	0.212765	17	37033946	F	II	LASP1	5'UTR	opensea	5'UTR-open		rs226216;r	50;51
cg21402921	-0.29626	0.259351	-5.61683	0.000596	0.006402	-0.17085	0.407482	0.111221	0.296261	15	27112305	R	I	GABRA5	1stExon	island	1stExon-isl	chr15:271	rs5389060	37
cg13713046	-0.22029	0.487392	-5.61204	0.0006	0.006424	-0.17657	0.59754	0.377245	0.220294	8	22500019	R	II	BIN3-IT1	TSS1500	opensea	TSS1500-op		rs1417865	45;15;6;1
cg27447382	-0.21443	0.321417	-5.61163	0.0006	0.006425	-0.17707	0.42863	0.214205	0.214425	3	23804563	F	II		IGR	opensea	IGR-opense			
cg07781401	-0.28948	0.664729	-5.61161	0.0006	0.006425	-0.17709	0.809471	0.519987	0.289485	17	19773621	R	II		IGR	shore	IGR-shore	chr17:197		
cg08938925	-0.29238	0.644461	-5.60963	0.000601	0.006434	-0.17946	0.790651	0.498271	0.29238	15	64110513	R	II	HERC1	5'UTR	opensea	5'UTR-open		rs5555566	13;1
cg24480695	-0.21157	0.244514	-5.60421	0.000605	0.006459	-0.18594	0.350299	0.138728	0.211571	11	18727628	R	I	IGSF22	Body	island	Body-islanc	chr11:187		
cg05897169	-0.27034	0.490616	-5.60168	0.000607	0.00647	-0.18896	0.625785	0.355447	0.270339	17	33568598	F	II	SLFN5	TSS1500	shore	TSS1500-sh	chr17:335		
cg01553004	-0.21179	0.269736	-5.59935	0.000608	0.006481	-0.19176	0.375632	0.16384	0.211791	14	77495672	R	II	C14orf4	TSS1500	shore	TSS1500-sh	chr14:774		
cg24631428	-0.20203	0.335632	-5.59803	0.000609	0.006486	-0.19334	0.436645	0.234618	0.202026	6	64281604	F	II	PTP4A1	TSS1500	shore	TSS1500-sh	chr6:6428	rs5631045	46
cg11747499	-0.25282	0.334927	-5.58547	0.000618	0.006542	-0.2084	0.461338	0.208515	0.252822	12	1.09E+08	F	II	SSH1	TSS1500	shore	TSS1500-sh	chr12:109	rs1406967	11
cg13246235	-0.238	0.516074	-5.58372	0.000619	0.00655	-0.21049	0.635075	0.397074	0.238001	6	12749978	F	I	PHACTR1	Body	island	Body-islanc	chr6:1274	rs5352632	12
cg00804794	-0.23656	0.634288	-5.57939	0.000622	0.00657	-0.21568	0.752566	0.51601	0.236556	6	1.58E+08	R	II		IGR	opensea	IGR-opense		rs3681141	1
cg12733607	-0.21793	0.428748	-5.57827	0.000623	0.006575	-0.21703	0.537714	0.319782	0.217932	10	21813501	R	II	C10orf140	5'UTR	shore	5'UTR-shor	chr10:218	rs5377790	5
cg26133769	-0.20386	0.32398	-5.57766	0.000623	0.006577	-0.21777	0.42591	0.22205	0.20386	4	30723855	R	I	PCDH7	1stExon	island	1stExon-isl	chr4:3072	rs5332347	18;6
cg22469897	-0.26393	0.363842	-5.57711	0.000624	0.00658	-0.21843	0.495807	0.231878	0.263929	19	51858276	F	II	ETFB	TSS200	opensea	TSS200-ope			
cg06375042	-0.20783	0.356129	-5.57617	0.000624	0.006584	-0.21955	0.460044	0.252214	0.20783	3	1.87E+08	R	II	ST6GAL1	5'UTR	opensea	5'UTR-open		rs5560562	46;43;11;1
cg10704887	-0.2161	0.391236	-5.57418	0.000626	0.006593	-0.22194	0.499285	0.283187	0.216098	2	2.35E+08	F	II		IGR	opensea	IGR-opense			
cg21164131	-0.21745	0.327876	-5.57084	0.000628	0.006607	-0.22596	0.436599	0.219153	0.217446	12	63544923	R	II	AVPR1A	1stExon	island	1stExon-isl	chr12:635	rs1865797	35
cg01207474	-0.22941	0.298321	-5.57025	0.000628	0.006609	-0.22666	0.413029	0.183614	0.229414	10	35736855	R	II	CCNY	5'UTR	opensea	5'UTR-open		rs5655081	4

cg18077971	-0.20286	0.298472	-5.56832	0.00063	0.006618	-0.22898	0.3999	0.197044	0.202856	2	2.23E+08	F	I	PAX3	TSS1500	shore	TSS1500-sh	chr2:2231	rs4550659	24;46
cg21327712	-0.23726	0.460461	-5.56812	0.00063	0.006619	-0.22923	0.579093	0.341829	0.237264	1	1.12E+08	F	II	DENND2D	TSS1500	shore	TSS1500-sh	chr1:1117	rs3543037	25
cg27157434	-0.24125	0.290722	-5.56332	0.000633	0.006641	-0.235	0.41135	0.170095	0.241255	1	1.18E+08	R	II	PTGFRN	3'UTR	opensea	3'UTR-oper			
cg10979132	-0.20581	0.383409	-5.55902	0.000636	0.00666	-0.24016	0.486313	0.280506	0.205807	22	17567287	F	II	IL17RA	Body	shore	Body-shore	chr22:175	rs1492164	35;49;51
cg07266910	-0.23367	0.441517	-5.55667	0.000638	0.006671	-0.24299	0.558353	0.324682	0.233671	3	1.79E+08	R	II	ZMAT3	Body	opensea	Body-open		rs2005581	22;1
cg23486701	-0.24054	0.462104	-5.55519	0.000639	0.006678	-0.24478	0.582372	0.341836	0.240536	2	54789491	R	II	SPTBN1	Body	shelf	Body-shelf	chr2:5478	rs5628160	1
cg16026241	-0.20718	0.555912	-5.55208	0.000641	0.006692	-0.24853	0.6595	0.452324	0.207176	15	38923883	F	II		IGR	opensea	IGR-opense			
cg10189885	-0.24416	0.435669	-5.55172	0.000642	0.006694	-0.24896	0.557749	0.313588	0.244161	2	1.98E+08	F	II	ANKRD44	TSS1500	shore	TSS1500-sh	chr2:1981	rs7398843	21
cg12774458	-0.22421	0.408069	-5.55145	0.000642	0.006695	-0.24928	0.520175	0.295964	0.224211	14	98445422	R	II	LINC01550	TSS1500	opensea	TSS1500-o		rs1450889	30
cg13763181	-0.2172	0.377235	-5.55066	0.000642	0.006698	-0.25022	0.485835	0.268636	0.217199	10	71087979	F	I	HK1	Body	opensea	Body-open		rs5463576	0;1;41
cg26023019	-0.22201	0.278711	-5.54282	0.000648	0.006736	-0.25967	0.389714	0.167707	0.222007	21	31311859	F	I	GRIK1	1stExon	island	1stExon-isl	chr21:313	rs1865063	0
cg21706867	-0.28165	0.47903	-5.54098	0.00065	0.006743	-0.2619	0.619855	0.338206	0.281648	5	54837161	F	II		IGR	opensea	IGR-opense		rs5768611	20
cg25351606	-0.2153	0.55213	-5.53737	0.000652	0.006758	-0.26624	0.65978	0.444481	0.2153	6	1.01E+08	F	I		IGR	island	IGR-island	chr6:1009	rs5503856	31;32;33;50
cg00732589	-0.20608	0.682444	-5.53444	0.000654	0.006772	-0.26979	0.785481	0.579406	0.206075	11	1.1E+08	R	II	FDX1	TSS1500	shore	TSS1500-sh	chr11:110	rs5287242	37;18;17;14;1
cg12513294	-0.22501	0.410857	-5.53388	0.000655	0.006774	-0.27045	0.523361	0.298352	0.225008	11	3889218	R	II	STIM1	Body	opensea	Body-open		rs1860415	2
cg23883696	-0.22139	0.338285	-5.53275	0.000656	0.00678	-0.27182	0.448982	0.227588	0.221394	18	70534298	R	I	NETO1	TSS1500	island	TSS1500-is	chr18:705	rs5481924	20;1
cg16217803	-0.21964	0.546818	-5.53221	0.000656	0.006782	-0.27248	0.656639	0.436998	0.219641	3	1.43E+08	F	II	CHST2	3'UTR	shore	3'UTR-shor	chr3:1428	rs3694602	1;21
cg00103299	-0.20596	0.358625	-5.53193	0.000656	0.006783	-0.27282	0.461604	0.255646	0.205958	15	93362247	R	II		IGR	opensea	IGR-opense		rs1838412	14;8
cg21705011	-0.25713	0.395962	-5.53188	0.000656	0.006784	-0.27287	0.524525	0.267399	0.257126	7	30196892	R	II	MTURN	Body	opensea	Body-open			
cg03330490	-0.21428	0.368434	-5.52937	0.000658	0.006795	-0.2759	0.475576	0.261292	0.214284	10	94452236	F	I	HHEX	Body	island	Body-island	chr10:944	rs5338245	1;7
cg21126583	-0.20799	0.24167	-5.52814	0.000659	0.0068	-0.27739	0.345664	0.137675	0.20799	7	1.46E+08	F	I	CNTNAP2	TSS200	island	TSS200-isla	chr7:1458	rs5608750	9;16
cg16957374	-0.25619	0.383113	-5.52618	0.00066	0.006809	-0.27976	0.511205	0.25502	0.256185	8	42596672	R	II		IGR	opensea	IGR-opense		rs1898945	28;3;1
cg21591274	-0.21217	0.320958	-5.52609	0.000661	0.006809	-0.27986	0.427042	0.214874	0.212169	4	74089071	F	II	ANKRD17	TSS1500	opensea	TSS1500-o			
cg02512559	-0.28851	0.338485	-5.52422	0.000662	0.006818	-0.28213	0.482741	0.194229	0.288512	16	75414554	F	II	CFDP1	Body	opensea	Body-open		rs5371444	0;5;17;39;50
cg24880056	-0.21565	0.284042	-5.52165	0.000664	0.006829	-0.28522	0.391868	0.176216	0.215652	18	31158234	R	I	ASXL3	TSS1500	opensea	TSS1500-o		rs1410221	3
cg18070458	-0.26756	0.468121	-5.52073	0.000665	0.006833	-0.28634	0.601901	0.334341	0.26756	11	1.21E+08	R	II		IGR	shelf	IGR-shelf	chr11:121	rs5780787	5;1
cg16745604	-0.20921	0.643494	-5.51994	0.000665	0.006837	-0.2873	0.748099	0.538888	0.20921	2	2.02E+08	F	II	CASP10	TSS200	opensea	TSS200-op		rs7142428	9;51
cg03736648	-0.2135	0.36314	-5.51743	0.000667	0.00685	-0.29033	0.46989	0.256389	0.213501	8	6575837	R	II	AGPAT5	Body	opensea	Body-open		rs5623307	45;41;34;26;1
cg18276442	-0.20181	0.279244	-5.51298	0.00067	0.006871	-0.29571	0.380147	0.178341	0.201806	8	81911441	F	II	PAG1	5'UTR	opensea	5'UTR-oper		rs1343912	0;5;24
cg05067754	-0.27441	0.364227	-5.51043	0.000672	0.006884	-0.2988	0.50143	0.227023	0.274406	2	1.99E+08	F	II	PLCL1	Body	opensea	Body-open		rs5772414	24;33;44
cg15124215	-0.26021	0.361884	-5.51042	0.000672	0.006884	-0.29881	0.491989	0.231778	0.260211	4	1.73E+08	F	I	GALNTL6	TSS200	island	TSS200-isla	chr4:1727	rs5752688	6;47
cg07780095	-0.22568	0.261826	-5.50992	0.000673	0.006885	-0.29942	0.374668	0.148985	0.225683	12	85674296	F	I	ALX1	Body	island	Body-island	chr12:856	rs3733334	0;34;48
cg17766305	-0.25427	0.620388	-5.50648	0.000675	0.006901	-0.30358	0.747524	0.493252	0.254272	10	90147030	F	II	RNLS	Body	opensea	Body-open		rs5416729	40
cg12944600	-0.24691	0.575923	-5.50148	0.000679	0.006926	-0.30964	0.699376	0.45247	0.246906	2	85147464	F	II		IGR	opensea	IGR-opense		rs1811449	1;12
cg12283746	-0.20278	0.208784	-5.4993	0.000681	0.006936	-0.31227	0.310174	0.107393	0.202781	15	75639596	R	II	NEIL1	TSS1500	island	TSS1500-is	chr15:756	rs5287542	39;14;2
cg23304747	-0.27312	0.503793	-5.49506	0.000684	0.006958	-0.31741	0.640356	0.367231	0.273125	12	1.24E+08	F	II		IGR	opensea	IGR-opense		rs5601154	0
cg13466988	-0.22142	0.548277	-5.494	0.000685	0.006963	-0.3187	0.658985	0.437569	0.221417	1	12538541	R	II	VPS13D	Body	opensea	Body-open		rs5461719	30;28;9
cg21031875	-0.23509	0.506173	-5.49034	0.000688	0.006981	-0.32313	0.623718	0.388629	0.23509	10	1.02E+08	R	II	DNMBP	Body	opensea	Body-open		rs7982225	44;15;1
cg01068944	-0.20218	0.217909	-5.48933	0.000689	0.006986	-0.32436	0.319001	0.116816	0.202185	20	9049273	F	I		IGR	island	IGR-island	chr20:904		
cg23089177	-0.21584	0.337039	-5.48891	0.000689	0.006988	-0.32487	0.444956	0.229121	0.215836	7	30196932	F	II	MTURN	Body	opensea	Body-open		rs5776761	1;33;35
cg05600342	-0.21722	0.356058	-5.48369	0.000693	0.00701	-0.3312	0.464668	0.247448	0.21722	11	250989	R	II	PSMD13	Body	opensea	Body-open		rs5532329	43;17;14;4
cg27363829	-0.24982	0.417005	-5.47925	0.000697	0.007031	-0.33659	0.541918	0.292093	0.249825	3	1.65E+08	F	I	SLITRK3	TSS200	opensea	TSS200-op			
cg01886311	-0.21867	0.484729	-5.47869	0.000697	0.007034	-0.33728	0.594065	0.375393	0.218672	12	47603650	F	II	PCED1B-AS	Body	opensea	Body-open		rs1503197	38;45
cg11749142	-0.21853	0.289571	-5.47763	0.000698	0.007039	-0.33857	0.398835	0.180308	0.218527	7	30197085	R	II	MTURN	Body	opensea	Body-open		rs2017161	41;13;2
cg01287377	-0.28739	0.383129	-5.47195	0.000702	0.007067	-0.34546	0.526834	0.239434	0.28739	3	60064969	R	II	FHIT	Body	opensea	Body-open		rs5276501	46;39;28;26;1
cg04415176	-0.23178	0.267928	-5.47135	0.000703	0.00707	-0.3462	0.383816	0.152039	0.231777	2	1.77E+08	F	I	HOXD13	1stExon	island	1stExon-isl	chr2:1769	rs1399954	36
cg05126388	-0.2095	0.749191	-5.46774	0.000706	0.007087	-0.35058	0.853944	0.644439	0.209505	16	49670144	R	II	ZNF423	Body	island	Body-island	chr16:496	rs1433937	43;32;5
cg24346629	-0.20413	0.219132	-5.46607	0.000707	0.007095	-0.35262	0.321199	0.117065	0.204134	3	74663689	F	I		IGR	island	IGR-island	chr3:7466		
cg11591310	-0.20558	0.237298	-5.46157	0.000711	0.007117	-0.35809	0.340086	0.13451	0.205576	12	12874295	F	II	CDKN1B	3'UTR	shelf	3'UTR-shelf	chr12:128	rs1929455	1
cg08750057	-0.21159	0.353571	-5.45727	0.000714	0.007138	-0.36333	0.459364	0.247777	0.211587	12	1.24E+08	R	II	C12orf65	5'UTR	shelf	5'UTR-shelf	chr12:123	rs5660275	13;6
cg21254135	-0.21774	0.456437	-5.45501	0.000716	0.007148	-0.36608	0.565308	0.347566	0.217742	7	76852292	R	II	CCDC146	Body	opensea	Body-open		rs1383743	39;36;16;1
cg16192197	-0.21149	0.415238	-5.45075	0.00072	0.007169	-0.37127	0.520981	0.309494	0.211487	6	1.12E+08	R	II		IGR	opensea	IGR-opense		rs5622603	17
cg11019064	-0.21244	0.43782	-5.4488	0.000721	0.007178	-0.37365	0.54404	0.331601	0.212439	14	71866033	R	II		IGR	opensea	IGR-opense		rs5754875	49
cg23250889	-0.23672	0.32118	-5.44803	0.000722	0.007182	-0.37458	0.439542	0.202819	0.236723	16	57300908	F	II	PLLP	Body	opensea	Body-open		rs5388076	0;29;34;49
cg06096336	-0.22186	0.43052	-5.44752	0.000722	0.007184	-0.3752	0.54145	0.31959	0.221859	2	2.32E+08	F	II	PSMD1	Body	opensea	Body-open		rs5290981	24;25
cg10138083	-0.22298	0.490536	-5.44559	0.000724	0.007193	-0.37756	0.602026	0.379045	0.222981	10	98156109	R	II	TLL2	Body	opensea	Body-open		rs5511022	46;38;20
cg06653045	-0.20311	0.198216	-5.44064	0.000728	0.00722	-0.3836	0.299772	0.096659	0.203112	17	68165064	F	I	KCNJ2	TSS1500	island	TSS1500-is	chr17:681		
cg15389373	-0.21419	0.482812	-5.43826	0.00073	0.007232	-0.38651	0.589906	0.375718	0.214188	13	1.01E+08	R	II	GGACT	5'UTR	shelf	5'UTR-shelf	chr13:101		
cg26524899	-0.21717	0.335266	-5.43783	0.00073	0.007234	-0.38703	0.443852	0.22668	0.217172	14	64932678	R	II	AKAP5	5'UTR	shore	5'UTR-shor	chr14:649		
cg02883147	-0.21095	0.599457	-5.43642	0.000731	0.007241	-0.38875	0.704931	0.493982	0.210948	12	9217769	F	II	LOC14457	TSS200	shore	TSS200-sho	chr12:921		
cg12789173	-0.2348	0.579166	-5.43612	0.000732	0.007242	-0.38911	0.696566	0.461766	0.2348	11	1.18E+08	F	II	AMICA1	Body	opensea	Body-open		rs5368504	5;28
cg25676030	-0.23117	0.414117	-5.4346	0.000733	0.00725	-0.39097	0.529703	0.298531	0.231171	3	4946693	R	II	BHLHE40-A	Body	opensea	Body-open		rs5766903	9;2;1
cg00461022	-0.21914	0.366256	-5.43301	0.000734	0.007258	-0.39291	0.475826	0.256685	0.219141	5	16618052	R	II	FAM134B	TSS1500	shore	TSS1500-sh	chr5:1661		
cg17705635	-0.23165	0.367727	-5.43263	0.000735	0.007259	-0.39338	0.483551	0.251903	0.231649	2	1.97E+08	F	II	HECW2	Body	opensea	Body-open		rs5294493	0;3;28;46
cg17984638	-0.24186	0.264421	-5.43174	0.000735	0.007264	-0.39446	0.385351	0.143491	0.24186	4	48136452	R	II	TXK	TSS200	opensea	TSS200-op		rs5288375	45;30;29;2
cg04478931	-0.20997	0.264941	-5.43165	0.000735	0.007264	-0.39458	0.369926	0.159956	0.20997	4	86396739	R	II	ARHGAP24	5'UTR	opensea	5'UTR-oper		rs5657956	8
cg07106927	-0.21458	0.607436	-5.43055	0.000736	0.007271	-0.39592	0.714727	0.500146	0.214581	1	1.61E+08	R	II	LY9	Body	shelf	Body-shelf	chr1:1607	rs5728646	17;1
cg01634544	-0.24398	0.323079	-5.42448	0.000741	0.007303	-0.40333	0.445071	0.201087	0.243984	14	71128147	R	II	TTC9	Body	opensea	Body-open		rs1899764	2
cg05276829	-0.24037	0.674339	-5.42433	0.000742	0.007303	-0.40352	0.794525	0.554153	0.240372	2	54861832	R	II	SPTBN1	Body	opensea	Body-open		rs1854514	49;40;9;2
cg00563824	-0.2009	0.390217	-5.42285	0.000743	0.00731	-0.40532	0.490665	0.289768	0.200897	1	2.09E+08	F	I		IGR	island	IGR-island	chr1:2094		
cg15843567	-0.23069	0.266399	-5.42254	0.000743	0.007312	-0.40571	0.381744	0.151055	0.230689	10	26223206	F	I	MYO3A	1stExon	island	1stExon-isl	chr10:262	rs7073224	24
cg11932301	-0.20191	0.202644	-5.42239	0.000743	0.007313	-0.40589	0.3036	0.101688	0.201912	10	76349339	F	II	ADK	Body	opensea	Body-open		rs5546731	0
cg06168539	-0.22349	0.422853	-5.42116	0.000744	0.007318	-0.40739	0.534596	0.31111	0.223485	12	53650032	F	II		IGR	shelf	IGR-shelf	chr12:536	rs1861474	0;1;22;42
cg07591278	-0.24414	0.49225	-5.42	0.000745	0.007325	-0.40881	0.614318	0.370182	0.244136	3	23851523	R	II	UBE2E1	TSS1500	shore	TSS1500-sh	chr3:2385	rs1932598	2
cg19095568	-0.25704	0.463692	-5.41912	0.000746	0.00733	-0.40989	0.592213	0.335171	0.257043	15	41062113	F	II	DNAJC17	Body	opensea	Body-open			
cg25156960	-0.20104	0.371188	-5.41786	0.000747	0.007337	-0.41143	0.471707	0.270669	0.201039	7	92645767	F	II		IGR	opensea	IGR-opense		rs1176625	13;30
cg00213487	-0.20138	0.494057	-5.41577	0.000749	0.007349	-0.41399	0.594749	0.393366	0.201383	15	75341903	F	II	PPCDC	3'UTR	opensea	3'UTR-oper		rs5488216	1
cg03388670	-0.22231	0.48717	-5.41308	0.000751	0.007363	-0.41728	0.598327	0.376013	0.222315	7	1.38E+08	F	II	SVOPL	ExonBnd	opensea	ExonBnd-o		rs1446381	0;1;45
cg13340765	-0.26402	0.478721	-5.41293	0.000751	0.007364	-0.41746	0.610731	0.346712	0.264019	8	1.42E+08	F	I		IGR	opensea	IGR-opense		rs1422023	0;2;10;11;24
cg20426710	-0.21917	0.442106	-5.41252	0.000752	0.007366	-0.41796	0.551692	0.332519	0.219172	22	50248907	F	II	ZBED4	5'UTR	shore	5'UTR-shor	chr22:502		
cg08239161	-0.24294	0.506456	-5.41074	0.000753	0.007375	-0.42014	0.627926	0.384985	0.242941	15	1E+08	R	II		IGR	opensea	IGR-opense		rs5587818	25;11
cg14898127	-0.25362	0.435478	-5.40939	0.000754	0.007382	-0.42179	0.562287	0.308668	0.253619	15	81587493	R	II	IL16	Body	opensea	Body-open		rs5683418	23
cg04710526	-0.21152	0.564392	-5.40861	0.000755	0.007386	-0.42275	0.670153	0.458632	0.211521	12	835793	F	II		IGR	opensea	IGR-opense		rs1142043	43
cg13240204	-0.30011	0.458279	-5.40674	0.000757	0.007396	-0.42505	0.608333	0.308224	0.300109	13	74276630	F	II	KLF12	Body	opensea	Body-open		rs1434573	0;31;50
cg03974193	-0.20813	0.393192	-5.40479	0.000758	0.007405	-0.42744	0.497257	0.289126	0.208131	2	1.02E+08	R	II	MAP4K4	Body	shore	Body-shore	chr2:1023		
cg19913496	-0.20487	0.359929	-5.40425	0.000759	0.007408	-0.42809	0.462363	0.257496	0.204867	1	1.17E+08	F	II		IGR	shore	IGR-shore	chr1:1166	rs5477613	32;36;42
cg03035849	-0.27567	0.565418	-5.40299	0.00076	0.007413	-0.42963	0.703252	0.427584	0.275668	6	91003200	R	II	BACH2	5'UTR	shore	5'UTR-shor	chr6:9100	rs5719522	47;26;10;1
cg14211915	-0.21248	0.259339	-5.40037	0.000762	0.007427	-0.43285	0.365577	0.153101	0.212475	9	94187714	F	II		IGR	shore	IGR-shore	chr9:9418	rs5561960	0;13
cg09962086	-0.27757	0.446976	-5.39786	0.000764	0.00744	-0.43593	0.585762	0.308189	0.277573	2	55260717	F	II	RTN4	Body	opensea	Body-open		rs5370346	8;13;14;15;41;44
cg00052964	-0.24476	0.648249	-5.39753	0.000765	0.007441	-0.43633	0.770628	0.525869	0.244758	2	85135823	R	II		IGR	shelf	IGR-shelf	chr2:8513	rs5436227	42;6;1
cg20563910	-0.20779	0.278094	-5.39666	0.000765	0.007446	-0.4374	0.38199	0.174198	0.207792	5	1.74E+08	R	I	MSX2	1stExon	island	1stExon-isl	chr5:1741		
cg25696648	-0.26496	0.636988	-5.39619	0.000766	0.007448	-0.43797	0.769467	0.50451	0.264956	9	1.24E+08	F	II	CNTRL	Body	opensea	Body-open		rs7500879	0;7
cg23057648	-0.20379	0.289822	-5.39523	0.000767	0.007453	-0.43915	0.391718	0.187925	0.203793	21	40984763	F	I	C21orf88	TSS200	opensea	TSS200-op			
cg23237433	-0.3141	0.43194	-5.39517	0.000767	0.007453	-0.43922	0.588993	0.274888	0.314105	13	24374391	F	II	MIPEP	Body	opensea	Body-open		rs1462936	0;1
cg20834627	-0.21129	0.458697	-5.39514	0.000767	0.007453	-0.43926	0.564343	0.35305	0.211293	1	2.27E+08	F	II	ITPKB	Body	opensea	Body-open		rs5757354	1;11
cg02886077	-0.21871	0.40054	-5.39398	0.000768	0.007458	-0.44069	0.509893	0.291187	0.218707	4	48779921	R	II	FRYL	5'UTR	shore	5'UTR-shor	chr4:4878	rs7315023	43
cg22435313	-0.24377	0.315481	-5.39334	0.000768	0.007461	-0.44147	0.437366	0.193595	0.243771	17	64669739	F	II	PRKCA	Body	opensea	Body-open		rs1502969	2
cg03727968	-0.22191	0.299554	-5.3893	0.000772	0.007484	-0.44643	0.410507	0.1886	0.221907	1	1.67E+08	F	II	CD247	Body	opensea	Body-open		rs5749722	1;20
cg19115729	-0.2814	0.652588	-5.38752	0.000774	0.007494	-0.44862	0.793287	0.511888	0.281399	11	1.08E+08	R	II	ATM	Body	opensea	Body-open		rs5401948	32
cg05191076	-0.22301	0.266643	-5.3861	0.000775	0.007501	-0.45035	0.378147	0.155139	0.223008	4	66536186	R	I	EPHA5	TSS1500	island	TSS1500-is	chr4:6653		
cg06108882	-0.23155	0.424879	-5.38362	0.000777	0.007515	-0.4534	0.540656	0.309102	0.231554	6	32835220	R	II		IGR	opensea	IGR-opense			
cg00884040	-0.21537	0.339013	-5.38133	0.000779	0.007526	-0.45621	0.4467	0.231326	0.215374	16	6069401	R	I	RBFOX1	TSS1500	island	TSS1500-is	chr16:606	rs5497682	20
cg00983956	-0.22361	0.279862	-5.37857	0.000782	0.007542	-0.45961	0.391665	0.16806	0.223605	19	58220669	R	II	ZNF154	TSS200	shore	TSS200-sho	chr19:582	rs1819334	41
cg20557159	-0.20856	0.650435	-5.3752	0.000785	0.00756	-0.46375	0.754714	0.546157	0.208557	17	75445261	R	II	SEPT9	Body	shelf	Body-shelf	chr17:754	rs5661808	35;34
cg13586889	-0.22005	0.605831	-5.37453	0.000785	0.007563	-0.46458	0.715857	0.495805	0.220053	2	1.73E+08	F	II	ITGA6	Body	shore	Body-shore	chr2:1732		
cg06036947	-0.20096	0.334187	-5.3718	0.000788	0.007579	-0.46793	0.434666	0.233707	0.200959	7	949834	F	II	ADAP1	Body	shore	Body-shore	chr7:9482	rs5444324	11;23;35
cg18448581	-0.23498	0.193651	-5.37159	0.000788	0.00758	-0.46819	0.31114	0.076162	0.234978	18	70534767	F	I	NETO1	1stExon	island	1stExon-isl	chr18:705		
cg25922624	-0.21145	0.371693	-5.36936	0.00079	0.007592	-0.47093	0.477416	0.265969	0.211447	4	1.54E+08	R	II	TRIM2	5'UTR	shore	5'UTR-shor	chr4:1540	rs1869046	35
cg02185248	-0.2355	0.441043	-5.36861	0.000791	0.007596	-0.47185	0.558795	0.323292	0.235503	17	78748494	R	II	RPTOR	Body	opensea	Body-open		rs1908559	50;39;38;31;24;23;1
cg07377422	-0.25467	0.542219	-5.36824	0.000791	0.007597	-0.4723	0.669555	0.414882	0.254673	19	46318633	F	I	RSPH6A	TSS200	island	TSS200-isla	chr19:463	rs1164641	0;27;47
cg16362955	-0.27573	0.466116	-5.36741	0.000792	0.007602	-0.47333	0.60398	0.328252	0.275728	16	57050428	R	II	NLRC5	TSS1500	opensea	TSS1500-o		rs1393354	25;20;2
cg26791805	-0.23286	0.464221	-5.36674	0.000792	0.007606	-0.47415	0.580652	0.34779	0.232862	14	99653857	F	II	BCL11B	Body	opensea	Body-open		rs5626344	43
cg26687842	-0.21209	0.743551	-5.36499	0.000794	0.007616	-0.47631	0.849595	0.637508	0.212087	13	41055491	F	II	LOC64698	TSS1500	opensea	TSS1500-o		rs1496252	38
cg00490655	-0.2148	0.286091	-5.36331	0.000795	0.007625	-0.47838	0.393492	0.17869	0.214802	2	1.65E+08	R	I	FIGN	TSS1500	shore	TSS1500-sh	chr2:1645	rs5630044	7;6
cg22524061	-0.22695	0.386491	-5.36306	0.000796	0.007627	-0.47869	0.499967	0.273015	0.226952	8	99959430	F	I	OSR2	5'UTR	shore	5'UTR-shor	chr8:9996	rs5674071	45
cg19216136	-0.22271	0.387463	-5.35973	0.000799	0.007644	-0.48279	0.498818	0.276108	0.22271	15	64796240	R	II	ZNF609	Body	opensea	Body-open			
cg25226014	-0.22976	0.230208	-5.35945	0.000799	0.007645	-0.48313	0.345087	0.115329	0.229758	3	45984942	F	II	CXCR6	TSS200	opensea	TSS200-op		rs5364511	24
cg10819289	-0.24448	0.690738	-5.35525	0.000803	0.007668	-0.4883	0.81298	0.568496	0.244484	22	39810539	F	II	TAB1	Body	opensea	Body-open		rs5721770	7;11;21;31
cg01552966	-0.225	0.480283	-5.35421	0.000804	0.007674	-0.48958	0.592786	0.367781	0.225004	2	25528330	R	II	DNMT3A	Body	opensea	Body-open			

cg16918625	-0.23206	0.38495	-5.3533	0.000805	0.007679	-0.49072	0.500979	0.26892	0.23206	3	30655974	F	II	TGFBR2	Body	opensea	Body-opens			
cg14175634	-0.28039	0.352434	-5.35272	0.000805	0.007682	-0.49142	0.492628	0.21224	0.280388	13	99647294	F	II	DOCK9	Body	opensea	Body-opens		rs1416935	1;27
cg02387618	-0.26017	0.434075	-5.35264	0.000805	0.007682	-0.49153	0.564161	0.303989	0.260172	5	1.33E+08	R	II	TCF7	Body	shelf	Body-shelf	chr5:1334		
cg14136214	-0.21119	0.565157	-5.35127	0.000807	0.00769	-0.49322	0.670751	0.459563	0.211188	9	1.25E+08	R	II	DAB2IP	3'UTR	opensea	3'UTR-oper		rs1881845	19;16
cg18145175	-0.24597	0.521275	-5.34965	0.000808	0.0077	-0.49521	0.644257	0.398292	0.245965	4	76621238	F	II		IGR	opensea	IGR-opense		rs5691231	1
cg13651908	-0.21962	0.303141	-5.34799	0.00081	0.00771	-0.49725	0.412949	0.193333	0.219616	2	2.05E+08	F	II	CD28	TSS1500	opensea	TSS1500-op		rs5774523	0;12;40
cg13499923	-0.29348	0.622657	-5.34641	0.000811	0.007718	-0.49921	0.769395	0.475919	0.293476	1	1.09E+08	R	II		IGR	opensea	IGR-opense		rs5683013	41
cg20301308	-0.2477	0.46825	-5.34352	0.000814	0.007732	-0.50277	0.592097	0.344402	0.247695	1	65534742	R	II		IGR	shore	IGR-shore	chr1:6553	rs1921076	11
cg00649330	-0.24392	0.437983	-5.34322	0.000814	0.007733	-0.50314	0.559941	0.316026	0.243915	11	1.28E+08	F	II	ETS1	Body	opensea	Body-opens			
cg21321167	-0.25117	0.455103	-5.34184	0.000815	0.00774	-0.50485	0.580687	0.329518	0.251169	5	89686304	F	II		IGR	opensea	IGR-opense		rs5357834	0;9;43;46
cg27208925	-0.21458	0.573619	-5.34178	0.000815	0.00774	-0.50492	0.680908	0.46633	0.214578	16	85937106	F	II	IRF8	Body	opensea	Body-opens		rs1930300	35
cg14512523	-0.24885	0.668264	-5.34128	0.000816	0.007743	-0.50554	0.792688	0.54384	0.248849	11	17230914	R	II		IGR	shore	IGR-shore	chr11:172	rs5518996	3
cg05531737	-0.22827	0.343288	-5.3399	0.000817	0.00775	-0.50724	0.457425	0.22915	0.228275	7	1094808	F	I	GPR146	5'UTR	shelf	5'UTR-shelf	chr7:1097	rs3724899	1;10;11;18;19;29;32
cg03442914	-0.26885	0.496915	-5.3389	0.000818	0.007756	-0.50848	0.631339	0.362491	0.268848	20	44994454	R	II	SLC35C2	TSS1500	shore	TSS1500-sh	chr20:449	rs3758712	7
cg24536818	-0.23263	0.278868	-5.33455	0.000822	0.007781	-0.51385	0.395184	0.162552	0.232632	12	55371892	F	II	KIAA0748	5'UTR	opensea	5'UTR-oper		rs1495607	0;1
cg01116137	-0.22594	0.699267	-5.33426	0.000823	0.007782	-0.51421	0.812239	0.586295	0.225945	20	25034263	F	II	ACSS1	Body	shelf	Body-shelf	chr20:250	rs7390607	8;29;31
cg11269842	-0.21102	0.475985	-5.33365	0.000823	0.007786	-0.51496	0.581493	0.370477	0.211016	6	24862223	R	II	FAM65B	Body	opensea	Body-opens		rs1831544	20;2
cg03853208	-0.22925	0.480798	-5.33277	0.000824	0.007791	-0.51605	0.595422	0.366174	0.229248	7	25989763	F	II	MIR148A	TSS200	shore	TSS200-sho	chr7:2599	rs1135741	36;45
cg21276413	-0.20139	0.206969	-5.33231	0.000824	0.007793	-0.51661	0.307663	0.106275	0.201388	15	27112287	R	I	GABRA5	1stExon	island	1stExon-isl	chr15:271	rs5761896	47;19
cg12467404	-0.26155	0.688199	-5.33161	0.000825	0.007797	-0.51749	0.818974	0.557424	0.261549	1	2.07E+08	R	II	CD55	Body	shore	Body-shore	chr1:2074	rs1123629	51;31;17
cg16193207	-0.29588	0.39304	-5.32772	0.000829	0.00782	-0.52229	0.540982	0.245098	0.295884	8	23107961	F	II	CHMP7	Body	shelf	Body-shelf	chr8:2310	rs1153730	38
cg14751182	-0.22932	0.362955	-5.32424	0.000832	0.007839	-0.52659	0.477615	0.248295	0.22932	22	41613712	R	II	L3MBTL2	Body	opensea	Body-opens		rs5719329	27;24;1
cg18832142	-0.25137	0.504787	-5.32333	0.000833	0.007845	-0.52772	0.630474	0.3791	0.251375	3	1.02E+08	F	II	LOC15222	TSS200	opensea	TSS200-ope		rs1511578	0;1;14;23;24
cg25431946	-0.25807	0.671476	-5.32243	0.000834	0.007849	-0.52883	0.800509	0.542443	0.258066	4	40313893	R	II	LOC10106	Body	opensea	Body-opens		rs5279890	11
cg06488150	-0.28569	0.421461	-5.32109	0.000835	0.007855	-0.53048	0.564306	0.278615	0.285691	7	6476003	F	II	DAGLB	Body	opensea	Body-opens		rs2009646	2;16;21;34
cg04398282	-0.26097	0.461976	-5.31927	0.000837	0.007864	-0.53274	0.592459	0.331493	0.260966	4	68424256	F	II	STAP1	TSS200	opensea	TSS200-ope			
cg26466508	-0.21631	0.578943	-5.31743	0.000839	0.007874	-0.53502	0.6871	0.470786	0.216314	6	76203597	R	II	FILIP1	TSS200	opensea	TSS200-ope		rs1475811	40;33;2;1
cg11378686	-0.20291	0.315943	-5.31583	0.00084	0.007884	-0.537	0.4174	0.214487	0.202913	6	43336278	R	II	ZNF318	Body	shore	Body-shore	chr6:4333	rs1131074	42;30
cg14433165	-0.298	0.670781	-5.31306	0.000843	0.0079	-0.54043	0.819781	0.521781	0.298	8	28911793	R	II		IGR	opensea	IGR-opense		rs5586872	2
cg22052645	-0.25844	0.425297	-5.31298	0.000843	0.0079	-0.54053	0.554515	0.29608	0.258435	2	85146702	F	II		IGR	opensea	IGR-opense		rs1391895	1;51
cg24317086	-0.20625	0.410687	-5.31295	0.000843	0.007901	-0.54057	0.513815	0.30756	0.206254	15	93423542	F	II		IGR	shelf	IGR-shelf	chr15:934	rs1181060	0
cg10009497	-0.24652	0.610422	-5.31159	0.000844	0.007909	-0.54225	0.73368	0.487165	0.246516	7	1095416	R	II	C7orf50	Body	shore	Body-shore	chr7:1097	rs1253300	8
cg00947782	-0.28438	0.30299	-5.30953	0.000846	0.007919	-0.54481	0.445178	0.160801	0.284376	6	30039142	F	I	RNF39	Body	island	Body-island	chr6:3003		
cg11033835	-0.22485	0.24532	-5.30807	0.000848	0.007926	-0.54661	0.357746	0.132894	0.224852	3	77087860	F	I	ROBO2	TSS1500	shore	TSS1500-sh	chr3:7708	rs5517502	3;24
cg23213230	-0.21595	0.384386	-5.30607	0.00085	0.007938	-0.54909	0.492363	0.276409	0.215954	13	50570091	F	II	TRIM13	TSS1500	shore	TSS1500-sh	chr13:505	rs1468697	1;6;19
cg07285386	-0.2114	0.57828	-5.306	0.00085	0.007938	-0.54918	0.68398	0.47258	0.2114	3	1.19E+08	F	II	B4GALT4	5'UTR	opensea	5'UTR-oper		rs5341841	0
cg10407744	-0.23115	0.634512	-5.30276	0.000853	0.007958	-0.55319	0.750086	0.518938	0.231148	9	97715349	F	II	C9orf3	Body	opensea	Body-opens		rs5440859	1
cg02717454	-0.2594	0.551912	-5.30104	0.000855	0.007968	-0.55533	0.681613	0.422212	0.259401	16	3928799	F	II	CREBBP	Body	shore	Body-shore	chr16:392	rs5442927	23
cg24096902	-0.22875	0.421827	-5.30085	0.000855	0.007969	-0.55556	0.536201	0.307452	0.228749	1	27754893	R	II	WASF2	Body	opensea	Body-opens		rs5322844	40
cg18115724	-0.21973	0.655212	-5.30052	0.000855	0.00797	-0.55597	0.765076	0.545348	0.219728	2	88390376	R	II	SMYD1	Body	opensea	Body-opens			
cg12097222	-0.25704	0.24203	-5.295	0.000861	0.008001	-0.56282	0.37055	0.11351	0.257039	8	16885000	R	I	EFHA2	1stExon	island	1stExon-isl	chr8:1688	rs5400761	23;13
cg09701062	-0.22176	0.35653	-5.29486	0.000861	0.008001	-0.56299	0.467412	0.245648	0.221764	5	1.33E+08	R	II		IGR	shelf	IGR-shelf	chr5:1334	rs244952;r	48;1
cg05193538	-0.23334	0.507927	-5.29467	0.000861	0.008002	-0.56323	0.624596	0.391257	0.233339	1	26097991	R	II	MAN1C1	Body	island	Body-island	chr1:2609	rs5772660	19;8
cg16533359	-0.26323	0.417184	-5.29326	0.000863	0.00801	-0.56498	0.548801	0.285567	0.263234	6	11710597	F	II		IGR	opensea	IGR-opense		rs5671785	0;1;29
cg07184013	-0.20708	0.331352	-5.2932	0.000863	0.00801	-0.56506	0.434894	0.22781	0.207084	7	25989618	R	II	MIR148A	TSS200	shore	TSS200-sho	chr7:2599	rs1856413	12;6
cg17801864	-0.23078	0.58739	-5.2928	0.000863	0.008013	-0.56555	0.702778	0.472003	0.230775	20	45179345	R	II	C20orf123	TSS200	opensea	TSS200-ope		rs5628499	11;10
cg09341797	-0.24502	0.515136	-5.28912	0.000867	0.008033	-0.57012	0.637647	0.392625	0.245022	14	62036609	F	II	FLJ22447	TSS1500	opensea	TSS1500-op		rs5574028	1;14;36
cg16108059	-0.23004	0.431845	-5.28746	0.000868	0.008042	-0.57219	0.546866	0.316824	0.230042	14	81421989	R	I	TSHR	5'UTR	shore	5'UTR-shor	chr14:814	rs2012527	26;8
cg24375364	-0.26407	0.571018	-5.28578	0.00087	0.008053	-0.57428	0.703054	0.438983	0.264071	7	2755000	R	II	AMZ1	3'UTR	shelf	3'UTR-shelf	chr7:2758	rs1434976	11;1
cg24727480	-0.22076	0.392546	-5.28499	0.000871	0.008058	-0.57525	0.502926	0.282166	0.22076	12	1.1E+08	R	II	KCTD10	Body	opensea	Body-opens		rs1418104	38;2;1
cg08594681	-0.22889	0.605904	-5.28242	0.000874	0.008075	-0.57845	0.72035	0.491458	0.228893	8	27468684	F	II	CLU	1stExon	shelf	1stExon-sh	chr8:2747		
cg12984893	-0.25227	0.464428	-5.28229	0.000874	0.008075	-0.57861	0.590563	0.338294	0.252269	10	1.34E+08	F	II	INPP5A	Body	shelf	Body-shelf	chr10:134	rs1810041	12
cg06493985	-0.2808	0.540921	-5.28225	0.000874	0.008076	-0.57865	0.681322	0.40052	0.280802	5	1.4E+08	R	II	PFDN1	Body	opensea	Body-opens		rs1821626	2
cg17547179	-0.26338	0.658117	-5.28181	0.000874	0.008078	-0.57921	0.789808	0.526427	0.263381	7	45068955	F	II	CCM2	Body	opensea	Body-opens		rs7618350	19
cg02497700	-0.23093	0.269513	-5.28038	0.000876	0.008085	-0.58099	0.384979	0.154048	0.230931	1	2.44E+08	R	II	ZNF238	TSS1500	shore	TSS1500-sh	chr1:2442	rs5691174	1
cg08860070	-0.22389	0.287467	-5.27742	0.000879	0.008103	-0.58467	0.399415	0.17552	0.223895	5	1.41E+08	R	I		IGR	shore	IGR-shore	chr5:1408	rs5332650	47;15
cg21208682	-0.20187	0.29843	-5.274	0.000882	0.008121	-0.58892	0.399363	0.197497	0.201867	8	1.42E+08	F	II	EIF2C2	Body	opensea	Body-opens		rs7558277	11
cg03105774	-0.22879	0.268992	-5.26968	0.000887	0.008145	-0.59429	0.383387	0.154597	0.22879	7	1.17E+08	F	II	ST7	Body	opensea	Body-opens		rs3716295	10;40
cg16587616	-0.20365	0.251429	-5.26868	0.000888	0.008151	-0.59554	0.353256	0.149602	0.203654	6	62996022	F	I	KHDRBS2	1stExon	island	1stExon-isl	chr6:6299	rs5445280	26;31
cg08903858	-0.27785	0.342672	-5.26858	0.000888	0.008151	-0.59566	0.481599	0.203745	0.277853	15	81595183	R	II	IL16	Body	opensea	Body-opens		rs1154742	19;13
cg09358400	-0.20914	0.373515	-5.26657	0.00089	0.008163	-0.59817	0.478083	0.268947	0.209136	15	89179232	R	II	ISG20	5'UTR	opensea	5'UTR-oper		rs1142684	35;1
cg22143698	-0.25277	0.534295	-5.26386	0.000893	0.008182	-0.60154	0.66068	0.407909	0.252771	5	10608058	R	II	ANKRD33B	Body	opensea	Body-opens		rs5358843	47;18
cg11080540	-0.23361	0.507756	-5.26118	0.000895	0.008198	-0.60488	0.624561	0.390951	0.23361	5	54897272	R	II		IGR	opensea	IGR-opense			
cg10037049	-0.20017	0.286865	-5.25674	0.0009	0.008225	-0.61041	0.386951	0.186778	0.200172	12	48276633	F	II	VDR	5'UTR	opensea	5'UTR-oper		rs5770566	1;43
cg01394102	-0.21647	0.365896	-5.25477	0.000902	0.008236	-0.61287	0.474131	0.257661	0.216471	4	2174605	F	II	POLN	Body	opensea	Body-opens		rs1381673	0;1;12;44
cg22668906	-0.25084	0.613562	-5.25473	0.000902	0.008237	-0.61292	0.73898	0.488143	0.250837	11	1.28E+08	F	II		IGR	opensea	IGR-opense		rs5641782	16;36;48
cg23603036	-0.20632	0.515829	-5.25109	0.000906	0.008257	-0.61746	0.618988	0.41267	0.206318	1	12675777	R	II	DHRS3	Body	shore	Body-shore	chr1:1267	rs3010915	43;2
cg16831926	-0.26821	0.551554	-5.2508	0.000906	0.008259	-0.61782	0.685658	0.417449	0.268208	4	1.09E+08	F	II	LEF1	Body	opensea	Body-opens		rs1878509	0;1;28
cg13719885	-0.24984	0.390608	-5.25014	0.000907	0.008262	-0.61865	0.515528	0.265689	0.249838	3	1.28E+08	R	II		IGR	island	IGR-island	chr3:1277		
cg06279296	-0.23578	0.400142	-5.24949	0.000908	0.008266	-0.61946	0.518033	0.282251	0.235782	10	601816	F	II	DIP2C	Body	opensea	Body-opens		rs5692921	0;16;18;44;45;48
cg03097013	-0.20579	0.351771	-5.24933	0.000908	0.008267	-0.61966	0.454667	0.248875	0.205792	20	60642264	F	II	TAF4	TSS1500	shore	TSS1500-sh	chr20:606	rs5596625	29;29;44
cg21771805	-0.24916	0.353516	-5.24876	0.000908	0.008271	-0.62036	0.478096	0.228936	0.24916	7	1.5E+08	F	II	GIMAP1	TSS1500	opensea	TSS1500-op		rs5595969	37;49
cg19694519	-0.23614	0.42692	-5.24788	0.000909	0.008276	-0.62147	0.544989	0.308851	0.236139	8	22389206	F	II	PPP3CC	Body	opensea	Body-opens		rs5746870	1
cg12943967	-0.23234	0.527631	-5.24759	0.00091	0.008277	-0.62183	0.643803	0.41146	0.232344	1	31501266	R	II	PUM1	Body	opensea	Body-opens		rs5773333	1
cg01543767	-0.21293	0.436033	-5.24505	0.000912	0.008294	-0.625	0.5425	0.329567	0.212933	4	1.1E+08	F	II	RPL34	TSS1500	opensea	TSS1500-op		rs5502116	0;1;32;35
cg20981615	-0.23765	0.249385	-5.24364	0.000914	0.008303	-0.62675	0.368209	0.13056	0.237649	4	48136280	R	II	TXK	TSS200	opensea	TSS200-ope			
cg04851848	-0.23611	0.454144	-5.24193	0.000916	0.008314	-0.6289	0.5722	0.336089	0.236112	5	16616019	F	II	LOC10192	TSS200	shore	TSS200-sho	chr5:1661	rs5308900	23;25;34
cg20661080	-0.23814	0.317176	-5.24158	0.000916	0.008316	-0.62933	0.436245	0.198106	0.238138	5	1.8E+08	F	II		IGR	opensea	IGR-opense		rs5669942	1;18
cg01557792	-0.21237	0.455055	-5.23689	0.000921	0.008345	-0.63519	0.561242	0.348868	0.212374	14	70162755	F	II	KIAA0247	Body	opensea	Body-opens			
cg15694715	-0.25034	0.46629	-5.23236	0.000926	0.008374	-0.64085	0.591458	0.341122	0.250337	11	1.18E+08	R	II	TMEM25	5'UTR	shore	5'UTR-shor	chr11:118		
cg07775309	-0.22327	0.255185	-5.23201	0.000926	0.008376	-0.64129	0.366817	0.143552	0.223266	3	12595852	F	II		IGR	shelf	IGR-shelf	chr3:1259	rs5402886	26
cg13518824	-0.24658	0.571839	-5.22974	0.000929	0.008389	-0.64413	0.695129	0.44855	0.246579	1	8523537	F	II	RERE	Body	opensea	Body-opens		rs7404964	14
cg09473510	-0.20499	0.257468	-5.22939	0.000929	0.008391	-0.64456	0.359961	0.154975	0.204986	4	1.87E+08	F	I	MTNR1A	TSS200	island	TSS200-isla	chr4:1874	rs5363418	33
cg06154060	-0.22295	0.351015	-5.22899	0.00093	0.008393	-0.64506	0.462488	0.239541	0.222947	10	98347412	R	II	TM9SF3	TSS1500	shore	TSS1500-sh	chr10:983	rs1391360	13
cg24104567	-0.2045	0.337644	-5.22873	0.00093	0.008394	-0.64539	0.439895	0.235394	0.204502	17	40932508	R	I	WNK4	TSS200	island	TSS200-isla	chr17:409	rs5388144	37;3
cg23723835	-0.25438	0.333727	-5.22841	0.00093	0.008396	-0.64579	0.460919	0.206535	0.254384	10	73869442	F	II	ASCC1	Body	opensea	Body-opens			
cg26534477	-0.23348	0.660681	-5.22818	0.000931	0.008398	-0.64608	0.777423	0.543939	0.233484	17	42147600	F	II	G6PC3	TSS1500	shore	TSS1500-sh	chr17:421		
cg11809038	-0.24317	0.514005	-5.22811	0.000931	0.008398	-0.64616	0.635588	0.392421	0.243167	13	21276845	R	II	IL17D	TSS1500	shore	TSS1500-sh	chr13:212	rs5600841	38;32;12
cg19711297	-0.24742	0.426273	-5.22758	0.000931	0.0084	-0.64683	0.549983	0.302564	0.24742	10	98347867	F	II	TM9SF3	TSS1500	shore	TSS1500-sh	chr10:983	rs5575056	1
cg19956136	-0.27639	0.540289	-5.22058	0.000939	0.00844	-0.6556	0.678486	0.402093	0.276392	14	93120784	R	II	RIN3	Body	opensea	Body-opens		rs5392867	19
cg02508461	-0.24953	0.453264	-5.22039	0.000939	0.008441	-0.65583	0.578032	0.328497	0.249535	1	2.35E+08	F	II		IGR	opensea	IGR-opense		rs5750042	6;15;18
cg20405891	-0.2045	0.448219	-5.21433	0.000946	0.008478	-0.66342	0.550467	0.345972	0.204495	11	1.34E+08	F	I	LOC28317	Body	opensea	Body-opens		rs1404208	1;8;41
cg08301869	-0.21968	0.442749	-5.21011	0.000951	0.008505	-0.6687	0.552588	0.33291	0.219677	4	6928241	R	II	TBC1D14	Body	shelf	Body-shelf	chr4:6925		
cg02495208	-0.21663	0.516758	-5.20983	0.000951	0.008506	-0.66905	0.625073	0.408443	0.21663	22	40341430	R	II	GRAP2	TSS1500	opensea	TSS1500-op		rs5370524	38;32;2
cg22054362	-0.22531	0.257955	-5.2075	0.000953	0.008522	-0.67198	0.370609	0.145301	0.225308	19	57050367	R	I	ZFP28	1stExon	island	1stExon-isl	chr19:570	rs5437945	44;30;9
cg09400963	-0.228	0.495464	-5.20716	0.000954	0.008524	-0.6724	0.609462	0.381465	0.227997	7	32555839	F	II	AVL9	Body	opensea	Body-opens		rs7893608	39
cg12623536	-0.23667	0.33037	-5.20692	0.000954	0.008525	-0.67271	0.448706	0.212034	0.236672	10	73848817	R	II	SPOCK2	TSS200	shore	TSS200-sho	chr10:738	rs5761333	37
cg05503210	-0.21342	0.320861	-5.20478	0.000956	0.00854	-0.67539	0.427572	0.214149	0.213423	16	56973760	R	II	HERPUD1	Body	opensea	Body-opens		rs5501837	18;15;2;1
cg09907509	-0.23282	0.440355	-5.2045	0.000957	0.008541	-0.67574	0.556767	0.323944	0.232823	13	37248244	F	I	C13orf36	5'UTR	island	5'UTR-islan	chr13:372		
cg00384221	-0.20688	0.49455	-5.20378	0.000958	0.008546	-0.67664	0.597992	0.391108	0.206884	6	52859375	R	II	GSTA4	5'UTR	shore	5'UTR-shor	chr6:5285	rs5499899	40
cg09082287	-0.21393	0.408454	-5.20209	0.00096	0.008556	-0.67877	0.515418	0.301489	0.213929	1	65730494	R	II	DNAJC6	5'UTR	shore	5'UTR-shor	chr1:6573		
cg18932726	-0.2061	0.245462	-5.20186	0.00096	0.008558	-0.67905	0.34851	0.142413	0.206096	10	1.29E+08	R	I	DOCK1	Body	island	Body-island	chr10:128	rs5353975	51
cg11546475	-0.25406	0.35847	-5.19964	0.000962	0.00857	-0.68184	0.485501	0.231439	0.254063	7	5733188	F	II	RNF216	Body	opensea	Body-opens			
cg01510609	-0.23421	0.261474	-5.1986	0.000963	0.008577	-0.68314	0.378579	0.144368	0.234211	2	3751134	F	I	DCDC2C	TSS200	island	TSS200-isla	chr2:3750	rs5370730	41;41
cg18096987	-0.23946	0.612863	-5.19704	0.000965	0.008588	-0.6851	0.732594	0.493132	0.239462	3	11623873	R	II	VGLL4	Body	opensea	Body-opens		rs5475545	29;6;2
cg10588617	-0.20353	0.288771	-5.1969	0.000965	0.008589	-0.68528	0.390538	0.187004	0.203534	2	43027940	F	II		IGR	opensea	IGR-opense		rs3728560	14;20
cg26415504	-0.21923	0.519702	-5.19638	0.000966	0.008592	-0.68592	0.629316	0.410089	0.219227	21	46344680	F	II	ITGB2-AS1	Body	opensea	Body-opens		rs5394812	0;8;48
cg07322611	-0.26613	0.326764	-5.19405	0.000969	0.008606	-0.68885	0.459829	0.193699	0.26613	3	71503426	R	II	FOXP1	5'UTR	opensea	5'UTR-oper		rs1045195	49;9
cg11921583	-0.22611	0.439656	-5.19343	0.000969	0.008609	-0.68963	0.552712	0.326599	0.226113	2	1.44E+08	R	II	ARHGAP15	5'UTR	opensea	5'UTR-oper		rs1813954	47;42
cg03348466	-0.20869	0.62999	-5.19304	0.00097	0.008612	-0.69012	0.734336	0.525644	0.208691	15	91104770	F	II	CRTC3	Body	opensea	Body-opens		rs5350502	29;43
cg04661346	-0.23921	0.574874	-5.19264	0.00097	0.008614	-0.69063	0.694479	0.455269	0.23921	12	27676221	R	II	PPFIBP1	TSS1500	opensea	TSS1500-op		rs5552136	35;20;5
cg06760077	-0.21265	0.504669	-5.18468	0.000979	0.008665	-0.70063	0.610995	0.398343	0.212652	2	1.19E+08	F	II		IGR	opensea	IGR-opense		rs5765211	32
cg16248329	-0.25081	0.280301	-5.18237	0.000982	0.008679	-0.70353	0.405709	0.154894	0.250815	4	1.88E+08	F	I	FAT1	5'UTR	island	5'UTR-islan	chr4:1876	rs1887875	20;36
cg09956615	-0.20143	0.346693	-5.18177	0.000983	0.008682	-0.70428	0.447407	0.24598	0.201427	7	2672989	R	II	TTYH3	Body	shore	Body-shore	chr7:2671	rs5684666	51;46;22;19
cg10441199	-0.24072	0.450587	-5.18149	0.000983	0.008684	-0.70464	0.570947	0.330228	0.240719	11	12155992	R	II	MICAL2	5'UTR	opensea	5'UTR-oper		rs9651616	13
cg01216438	-0.21045	0.221563	-5.1803	0.000984	0.008691	-0.70614	0.326787	0.11634	0.210447	15	77376235	F	I		IGR	opensea	IGR-opense		rs5275803	0;30
cg11654900	-0.2001	0.55138	-5.17906	0.000986	0.008699	-0.7077	0.65143	0.451329	0.2001	1	51444685	R	II		IGR	shore	IGR-shore	chr1:5144	rs1491134	21
cg12111855	-0.25665	0.563251	-5.17683	0.000988	0.008714	-0.71051	0.691576	0.434926	0.25665	1	1.18E+08	R	II		IGR	opensea	IGR-opense		rs1500133	2
cg10233311	-0.23387	0.467953	-5.1753	0.00099	0.008724	-0.71243	0.584888	0.351017	0.233871	3	1.02E+08	F	II		IGR	opensea	IGR-opense			

cg08654091	-0.22709	0.477246	-5.17529	0.00099	0.008724	-0.71244	0.590793	0.363699	0.227094	1	2.27E+08	R	II		IGR	opensea	IGR-opense		rs1912013	3
cg07483000	-0.23121	0.521325	-5.17411	0.000992	0.008731	-0.71392	0.63693	0.40572	0.23121	15	1E+08	F	II		IGR	opensea	IGR-opense		rs5632597	0;1;47
cg08730282	-0.28569	0.637443	-5.17373	0.000992	0.008734	-0.71441	0.780285	0.4946	0.285685	2	96922414	F	II	TMEM127	Body	opensea	Body-opens		rs5537026	17
cg20319945	-0.21539	0.479478	-5.17183	0.000994	0.008745	-0.71679	0.587175	0.371782	0.215393	12	1.33E+08	R	II	GOLGA3	Body	opensea	Body-opens		rs1176421	5;34
cg05634770	-0.27162	0.518956	-5.16919	0.000997	0.008762	-0.72012	0.654764	0.383148	0.271617	10	1.16E+08	R	II	ABLIM1	5'UTR	opensea	5'UTR-open		rs5744907	24
cg21887193	-0.21476	0.686657	-5.16877	0.000998	0.008764	-0.72065	0.794038	0.579275	0.214764	7	1.51E+08	R	I	AGAP3	Body	shore	Body-shore	chr7:1507	rs5286998	1;15;16
cg26760942	-0.23436	0.460877	-5.16695	0.001	0.008775	-0.72295	0.578059	0.343694	0.234364	10	1.16E+08	R	II	ABLIM1	5'UTR	opensea	5'UTR-open		rs1890148	51
cg14695332	-0.22047	0.590854	-5.16448	0.001003	0.00879	-0.72605	0.701086	0.480621	0.220465	1	2.27E+08	F	II	ITPKB	Body	opensea	Body-opens		rs1133414	0;37;49
cg02710015	-0.28152	0.440327	-5.16262	0.001005	0.008803	-0.7284	0.581087	0.299568	0.281518	12	55362424	R	II	KIAA0748	Body	opensea	Body-opens		rs1154614	10
cg01818341	-0.26375	0.612657	-5.15999	0.001008	0.008819	-0.73171	0.744533	0.480781	0.263753	15	67440222	R	II	SMAD3	5'UTR	opensea	5'UTR-open		rs5664778	10
cg15286847	-0.20742	0.318087	-5.1594	0.001009	0.008823	-0.73246	0.421797	0.214377	0.207419	16	84690433	R	II	KLHL36	Body	island	Body-island	chr16:846	rs1476559	30
cg22395761	-0.20942	0.208201	-5.15435	0.001015	0.008856	-0.73883	0.312913	0.103489	0.209424	12	6553937	R	II	CD27	TSS200	opensea	TSS200-ope			
cg12726213	-0.22067	0.427165	-5.15296	0.001017	0.008866	-0.74058	0.537502	0.316828	0.220673	8	87368021	R	II	WWP1	5'UTR	opensea	5'UTR-open		rs5413682	38;2;0
cg26707718	-0.20809	0.438386	-5.15071	0.001019	0.008878	-0.74342	0.542429	0.334343	0.208086	13	41064482	F	II		IGR	opensea	IGR-opense			
cg10749994	-0.32557	0.470738	-5.14329	0.001028	0.008927	-0.75279	0.633523	0.307953	0.32557	2	25474327	F	I	DNMT3A	Body	shore	Body-shore	chr2:2547	rs934780;r	7;17
cg06151145	-0.25729	0.549616	-5.14241	0.001029	0.008932	-0.75391	0.678263	0.42097	0.257293	4	48346434	F	II	SLAIN2	Body	shelf	Body-shelf	chr4:4834	rs5590993	24;36
cg12582959	-0.23484	0.555457	-5.14182	0.00103	0.008936	-0.75465	0.672875	0.438039	0.234836	19	56159199	R	II	CCDC106	5'UTR	shore	5'UTR-shor	chr19:561	rs5330639	45;40;10
cg13460643	-0.27272	0.30012	-5.14174	0.00103	0.008936	-0.75475	0.436482	0.163759	0.272723	1	2.1E+08	F	I	G0S2	1stExon	island	1stExon-isl	chr1:2098	rs1154271	8;17
cg04179740	-0.22914	0.316432	-5.13867	0.001034	0.008956	-0.75863	0.431004	0.20186	0.229145	10	73516760	R	II	CDH23	Body	opensea	Body-opens		rs5518048	36;1
cg22075820	-0.24099	0.495716	-5.13442	0.001039	0.008983	-0.764	0.616212	0.375219	0.240993	11	17300909	R	II	NUCB2	5'UTR	shelf	5'UTR-shelf	chr11:172	rs5490615	51;16;2;1
cg22165175	-0.2495	0.452399	-5.13031	0.001044	0.009009	-0.7692	0.577149	0.327648	0.249501	1	1.11E+08	R	II	KCNA2	TSS1500	shore	TSS1500-sh	chr1:1111	rs5466940	27;25;14;2
cg01492465	-0.20665	0.335363	-5.12988	0.001045	0.009013	-0.76974	0.438689	0.232037	0.206652	5	1.11E+08	R	II	CAMK4	Body	opensea	Body-opens		rs1879809	4;2
cg19987210	-0.20025	0.279654	-5.12466	0.001051	0.009044	-0.77635	0.379781	0.179527	0.200254	12	63544752	F	I	AVPR1A	1stExon	island	1stExon-isl	chr12:635	rs5762566	28
cg07385778	-0.21913	0.567769	-5.12277	0.001054	0.009054	-0.77875	0.677332	0.458205	0.219127	3	72320634	F	II		IGR	opensea	IGR-opense		rs1437369	1
cg02391509	-0.21886	0.429844	-5.12122	0.001056	0.009066	-0.78071	0.539276	0.320412	0.218864	3	27766382	F	II		IGR	shore	IGR-shore	chr3:2776	rs5648820	10
cg03738444	-0.23448	0.460221	-5.11885	0.001059	0.00908	-0.78371	0.577458	0.342983	0.234475	4	1.03E+08	R	II	NFKB1	Body	opensea	Body-opens		rs1394043	30;24;12
cg09757087	-0.21206	0.225882	-5.11786	0.00106	0.009087	-0.78496	0.331912	0.119851	0.212061	16	1585644	F	II	IFT140	Body	shore	Body-shore	chr16:158	rs1414607	6
cg08483099	-0.23498	0.424871	-5.1172	0.001061	0.009092	-0.7858	0.542361	0.307382	0.23498	1	2.4E+08	R	II	CHRM3-AS	Body	opensea	Body-opens		rs5662699	21;20;16
cg08641990	-0.26075	0.416958	-5.11564	0.001063	0.009102	-0.78777	0.547332	0.286585	0.260747	1	54822503	F	II	SSBP3	Body	shore	Body-shore	chr1:5482		
cg00462170	-0.26626	0.502618	-5.11547	0.001063	0.009103	-0.78799	0.63575	0.369486	0.266264	11	1.02E+08	R	II		IGR	opensea	IGR-opense			
cg03205581	-0.21038	0.608119	-5.11364	0.001065	0.009115	-0.79031	0.713309	0.50293	0.210379	14	99671848	F	II	BCL11B	Body	opensea	Body-opens		rs1868856	1
cg24223075	-0.2196	0.341026	-5.11285	0.001066	0.00912	-0.79131	0.450824	0.231228	0.219596	11	1.19E+08	F	II	CBL	Body	opensea	Body-opens		rs5697372	1;5;38
cg14785958	-0.22111	0.53847	-5.1127	0.001066	0.009121	-0.7915	0.649028	0.427913	0.221114	2	1.72E+08	R	II	TLK1	Body	opensea	Body-opens		rs5391045	2
cg06701914	-0.24385	0.340895	-5.11077	0.001069	0.009134	-0.79395	0.462818	0.218972	0.243847	18	74571505	R	II	ZNF236	Body	opensea	Body-opens		rs1908043	47;8;2
cg20763338	-0.22025	0.467509	-5.10956	0.00107	0.009142	-0.79549	0.577632	0.357385	0.220247	3	15246594	R	II	CAPN7	TSS1500	shore	TSS1500-sh	chr3:1524	rs1814085	38;34;21;14;9
cg22620221	-0.22375	0.352153	-5.10773	0.001073	0.009156	-0.79781	0.464029	0.240278	0.223751	7	1.54E+08	F	I	DPP6	TSS200	island	TSS200-isla	chr7:1535	rs5501179	5;6;51
cg26349275	-0.22264	0.417136	-5.1047	0.001076	0.009176	-0.80165	0.528454	0.305818	0.222636	9	21990277	F	II	CDKN2A	Body	shore	Body-shore	chr9:2198	rs1844325	40;46
cg15302376	-0.24344	0.492356	-5.10445	0.001077	0.009177	-0.80197	0.614078	0.370634	0.243444	2	25560263	R	II	DNMT3A	5'UTR	shelf	5'UTR-shelf	chr2:2556	rs5470844	51
cg23712018	-0.22463	0.265439	-5.10027	0.001082	0.009208	-0.80727	0.377752	0.153127	0.224625	6	30039132	F	I	RNF39	Body	island	Body-island	chr6:3003	rs9261293	6
cg23708361	-0.25214	0.36732	-5.09879	0.001084	0.009218	-0.80915	0.49339	0.24125	0.25214	7	1.46E+08	F	II	CNTNAP2	TSS200	island	TSS200-isla	chr7:1458	rs5280428	5
cg21202462	-0.26265	0.539621	-5.09813	0.001085	0.009223	-0.80999	0.670944	0.408297	0.262647	5	80830416	F	II	SSBP2	Body	opensea	Body-opens			
cg16412973	-0.24552	0.646138	-5.09806	0.001085	0.009223	-0.81009	0.768896	0.52338	0.245516	9	1.31E+08	R	II	FAM102A	Body	opensea	Body-opens		rs1460215	22
cg25595254	-0.22851	0.31075	-5.09772	0.001085	0.009226	-0.81051	0.425005	0.196495	0.22851	3	1.56E+08	F	II		IGR	opensea	IGR-opense		rs5699598	1;8;15;19;48
cg25570278	-0.25339	0.35326	-5.09767	0.001086	0.009226	-0.81058	0.479955	0.226565	0.25339	14	23306509	F	I	MMP14	Body	island	Body-island	chr14:233	rs1814005	36;37
cg11094543	-0.23779	0.264862	-5.09751	0.001086	0.009226	-0.81078	0.383755	0.145968	0.237787	2	2.42E+08	F	II	HDLBP	5'UTR	opensea	5'UTR-open		rs1126876	4;24
cg25661571	-0.20429	0.674426	-5.09429	0.00109	0.009248	-0.81487	0.776569	0.572283	0.204286	17	63543769	F	II	AXIN2	Body	opensea	Body-opens		rs5646069	7;14
cg04877165	-0.21743	0.474664	-5.09368	0.001091	0.009251	-0.81564	0.583378	0.365951	0.217427	2	1.74E+08	R	II	ZAK	Body	opensea	Body-opens			
cg07772781	-0.26662	0.577298	-5.09268	0.001092	0.009259	-0.81691	0.71061	0.443987	0.266622	3	1.02E+08	F	II		IGR	opensea	IGR-opense			
cg01245856	-0.25524	0.555643	-5.0914	0.001094	0.009268	-0.81854	0.683261	0.428025	0.255236	6	90894791	F	II	BACH2	5'UTR	opensea	5'UTR-open		rs5469890	1;9;40;49
cg02588451	-0.22487	0.596829	-5.08796	0.001098	0.009291	-0.82291	0.709262	0.484396	0.224867	1	2.27E+08	F	II	ITPKB-IT1	Body	opensea	Body-opens			
cg21145608	-0.20166	0.65408	-5.08656	0.0011	0.009302	-0.82469	0.754909	0.553251	0.201658	6	1.51E+08	R	II	PLEKHG1	5'UTR	opensea	5'UTR-open		rs7300218	16;39
cg04057147	-0.24099	0.377929	-5.08366	0.001104	0.009322	-0.82838	0.498426	0.257432	0.240994	5	79487759	F	II	SERINC5	Body	opensea	Body-opens			
cg15934804	-0.2034	0.297153	-5.08242	0.001105	0.00933	-0.82996	0.398852	0.195455	0.203396	7	2672400	R	II	TTYH3	Body	shore	Body-shore	chr7:2671	rs5350397	43;25
cg10240150	-0.21651	0.25764	-5.08061	0.001108	0.009344	-0.83227	0.365893	0.149387	0.216506	2	2.05E+08	F	II	CD28	TSS1500	opensea	TSS1500-op		rs1837972	6;25
cg12113316	-0.21348	0.376045	-5.07482	0.001115	0.009384	-0.83963	0.482785	0.269304	0.213481	8	37729089	R	II	RAB11FIP1	Body	opensea	Body-opens		rs2004810	45;26;25;19;3;1
cg15109018	-0.24857	0.323496	-5.07458	0.001116	0.009386	-0.83994	0.447779	0.199213	0.248566	12	85862615	R	II		IGR	opensea	IGR-opense		rs5305385	32
cg14353148	-0.2321	0.478231	-5.07172	0.00112	0.009406	-0.84358	0.594283	0.362179	0.232104	4	1.86E+08	R	II		IGR	opensea	IGR-opense		rs1401734	2
cg18265118	-0.26289	0.514437	-5.07084	0.001121	0.009412	-0.8447	0.645882	0.382992	0.26289	7	2139243	F	II	MAD1L1	Body	opensea	Body-opens		rs7303844	31;43;48
cg22589728	-0.22787	0.450873	-5.06957	0.001122	0.009421	-0.84632	0.56481	0.336936	0.227874	3	71439885	R	II	FOXP1	5'UTR	opensea	5'UTR-open			
cg06114320	-0.27176	0.26856	-5.06732	0.001125	0.009437	-0.84918	0.404441	0.132679	0.271763	2	10470282	R	II	HPCAL1	TSS200	opensea	TSS200-ope		rs1122597	47;2
cg07065759	-0.24338	0.492602	-5.0672	0.001126	0.009437	-0.84934	0.61429	0.370913	0.243377	2	1.98E+08	F	II	ANKRD44	Body	opensea	Body-opens		rs5743246	0;30
cg24405716	-0.23173	0.54601	-5.06637	0.001127	0.009443	-0.8504	0.661876	0.430144	0.231733	15	31280513	F	II	MTMR10	Body	shelf	Body-shelf	chr15:312	rs1421790	0;16;24
cg03906910	-0.22612	0.318118	-5.06432	0.00113	0.009457	-0.85302	0.431177	0.205059	0.226119	5	1.43E+08	R	II	NR3C1	1stExon	opensea	1stExon-op		rs3684340	16
cg12166476	-0.20742	0.343036	-5.0636	0.00113	0.009461	-0.85394	0.446745	0.239326	0.207419	13	76334866	F	II	LMO7	5'UTR	opensea	5'UTR-open		rs5473439	9;19;27
cg11173131	-0.22789	0.573364	-5.06272	0.001132	0.009467	-0.85505	0.687307	0.459422	0.227886	20	45179226	R	II	C20orf123	TSS200	opensea	TSS200-ope		rs5364540	32;31;2;1
cg15831212	-0.24415	0.603958	-5.06177	0.001133	0.009473	-0.85626	0.726033	0.481883	0.24415	5	1.4E+08	R	II	PFDN1	Body	opensea	Body-opens			
cg07324172	-0.26531	0.434541	-5.06166	0.001133	0.009474	-0.85641	0.567196	0.301886	0.265309	4	1.53E+08	R	II	FBXW7	5'UTR	opensea	5'UTR-open		rs2008076	35;34;2
cg18143792	-0.21939	0.392912	-5.06164	0.001133	0.009474	-0.85643	0.502607	0.283217	0.21939	1	26250290	F	II		IGR	shore	IGR-shore	chr1:2624	rs7266402	26
cg06806339	-0.20376	0.714539	-5.05997	0.001135	0.009486	-0.85855	0.816418	0.612661	0.203757	9	20508649	R	II	MLLT3	Body	opensea	Body-opens		rs1477608	29
cg15586429	-0.20741	0.302763	-5.05923	0.001136	0.009492	-0.8595	0.406467	0.199058	0.207408	2	1.5E+08	R	I	LYPD6	1stExon	island	1stExon-isl	chr2:1501	rs5592023	44;43
cg12974891	-0.24811	0.519278	-5.05807	0.001138	0.0095	-0.86098	0.643333	0.395222	0.248111	1	2.43E+08	R	II	CEP170	5'UTR	shore	5'UTR-shor	chr1:2434	rs6668649	18
cg23820828	-0.21657	0.450657	-5.05763	0.001139	0.009503	-0.86155	0.558944	0.34237	0.216574	8	99959602	R	II	OSR2	5'UTR	shore	5'UTR-shor	chr8:9996	rs5383325	46
cg18187859	-0.22557	0.598188	-5.05719	0.001139	0.009506	-0.86211	0.710971	0.485405	0.225565	21	43880997	R	II		IGR	opensea	IGR-opense		rs1000114	24;22;19;2
cg26530485	-0.24559	0.415839	-5.05654	0.00114	0.009509	-0.86293	0.538635	0.293043	0.245591	1	23496304	F	II	LUZP1	TSS1500	opensea	TSS1500-op		rs7809640	48
cg23488414	-0.20646	0.253929	-5.05606	0.001141	0.009513	-0.86355	0.357157	0.1507	0.206457	4	74088730	R	II	ANKRD17	Body	opensea	Body-opens		rs5673862	2
cg15685693	-0.20474	0.288971	-5.05594	0.001141	0.009513	-0.8637	0.39134	0.186602	0.204738	7	70597351	F	I	WBSCR17	TSS200	island	TSS200-isla	chr7:7059	rs1023496	14;16
cg11675876	-0.21239	0.41422	-5.0542	0.001143	0.009524	-0.86592	0.520415	0.308024	0.21239	3	1.89E+08	F	I	TPRG1-AS1	TSS200	island	TSS200-isla	chr3:1886	rs5657744	1;21;25;34
cg19274890	-0.29752	0.350424	-5.05368	0.001144	0.009528	-0.86658	0.499183	0.201664	0.29752	7	1.54E+08	R	I	DPP6	TSS200	island	TSS200-isla	chr7:1535	rs5316338	51;45;33;30
cg16006418	-0.20323	0.594586	-5.0531	0.001145	0.009532	-0.86732	0.696202	0.492971	0.203231	3	1.07E+08	F	II	BBX	5'UTR	opensea	5'UTR-open			
cg14473924	-0.2086	0.480395	-5.05144	0.001147	0.009544	-0.86944	0.584694	0.376095	0.208599	3	73674170	F	II	PDZRN3	TSS200	island	TSS200-isla	chr3:7367	rs1134716	12;18;35;38
cg22715764	-0.25815	0.621051	-5.0496	0.00115	0.009557	-0.87179	0.750128	0.491974	0.258154	1	10556294	R	II	PEX14	Body	opensea	Body-opens			
cg22866426	-0.21297	0.535293	-5.04801	0.001152	0.009569	-0.87382	0.641779	0.428807	0.212972	21	36263347	R	II	RUNX1	Body	island	Body-island	chr21:362	rs5511878	23;18
cg25330422	-0.28959	0.438018	-5.04541	0.001155	0.00959	-0.87715	0.582812	0.293225	0.289587	17	40467382	R	II	STAT3	3'UTR	shelf	3'UTR-shelf	chr17:404	rs1867985	23
cg08767938	-0.25698	0.577254	-5.04235	0.00116	0.009612	-0.88105	0.705743	0.448765	0.256979	7	25989524	F	II		IGR	shore	IGR-shore	chr7:2599		
cg07114422	-0.21006	0.494521	-5.04167	0.001161	0.009617	-0.88193	0.599551	0.389492	0.210059	1	2183438	R	II	SKI	Body	opensea	Body-opens		rs5415647	30
cg24039697	-0.21368	0.326906	-5.04072	0.001162	0.009623	-0.88314	0.433748	0.220065	0.213683	10	8094534	F	I	FLJ45983	Body	island	Body-island	chr10:809		
cg01778017	-0.26474	0.681809	-5.04004	0.001163	0.009628	-0.88401	0.814178	0.54944	0.264738	1	1.93E+08	R	II		IGR	opensea	IGR-opense			
cg21833076	-0.24956	0.345537	-5.0371	0.001167	0.00965	-0.88777	0.470315	0.220758	0.249557	11	1.05E+08	R	II		IGR	opensea	IGR-opense		rs5766006	0
cg10530883	-0.22631	0.315145	-5.03559	0.001169	0.009662	-0.88971	0.428298	0.201991	0.226307	5	3596207	F	I	IRX1	1stExon	island	1stExon-isl	chr5:3594	rs5527259	28
cg07917644	-0.23977	0.580928	-5.03365	0.001172	0.009677	-0.89218	0.700811	0.461045	0.239766	15	1.02E+08	F	II	CHSY1	Body	opensea	Body-opens		rs5692846	39
cg09890699	-0.21035	0.446275	-5.03293	0.001173	0.009682	-0.89311	0.551452	0.341099	0.210354	1	54821853	R	II	SSBP3	Body	shore	Body-shore	chr1:5482	rs1405956	37;16
cg22684041	-0.26535	0.548476	-5.03228	0.001174	0.009687	-0.8939	0.68115	0.415802	0.265348	19	1904554	R	II	SCAMP4	TSS1500	shore	TSS1500-sh	chr19:190	rs1844210	16
cg14525407	-0.21724	0.471681	-5.02953	0.001178	0.009707	-0.89745	0.580301	0.363061	0.217241	15	63570327	F	II	APH1B	Body	shore	Body-shore	chr15:635	rs5539718	1;13
cg16458838	-0.23904	0.669111	-5.02871	0.001179	0.009713	-0.8985	0.788633	0.54959	0.239043	15	57250375	F	II	TCF12	Body	opensea	Body-opens		rs5374860	15;23
cg26441817	-0.22249	0.526808	-5.02839	0.001179	0.009715	-0.89892	0.638051	0.415565	0.222486	15	31495225	R	II		IGR	opensea	IGR-opense			
cg02971209	-0.22324	0.498379	-5.02825	0.001179	0.009716	-0.89909	0.609998	0.38676	0.223238	8	8932770	R	II		IGR	opensea	IGR-opense		rs7319772	2;1
cg25476208	-0.24977	0.319771	-5.02516	0.001184	0.009737	-0.90305	0.444656	0.194886	0.24977	14	69434991	R	II	ACTN1	Body	opensea	Body-opens		rs5415837	40;39;11;8
cg05764155	-0.22458	0.454753	-5.02465	0.001184	0.009742	-0.9037	0.56704	0.342465	0.224576	1	1.12E+08	R	II	TMIGD3	Body	shore	Body-shore	chr1:1120	rs1425450	24
cg13974632	-0.21909	0.372411	-5.0237	0.001186	0.009748	-0.90492	0.481954	0.262868	0.219086	11	27740813	F	I	BDNF	Body	island	Body-island	chr11:277	rs1083521	25;31
cg14834362	-0.24257	0.498451	-5.0233	0.001186	0.009751	-0.90543	0.619737	0.377166	0.242571	2	1.98E+08	F	II	ANKRD44	Body	opensea	Body-opens		rs5731987	1
cg03461784	-0.22153	0.372359	-5.02208	0.001188	0.00976	-0.907	0.483127	0.261592	0.221535	14	98604556	R	II		IGR	opensea	IGR-opense			
cg08006309	-0.22136	0.443943	-5.02058	0.00119	0.00977	-0.90892	0.554622	0.333263	0.221359	16	1587810	R	II	IFT140	Body	island	Body-island	chr16:158	rs1443905	48;41;40;25;21
cg07793808	-0.21975	0.405201	-5.01792	0.001194	0.009791	-0.91232	0.515075	0.295328	0.219746	12	1.22E+08	R	II	KDM2B	TSS200	shore	TSS200-sho	chr12:122		
cg01682285	-0.26125	0.477802	-5.01634	0.001196	0.009802	-0.91435	0.608425	0.347179	0.261246	14	81422160	F	II	TSHR	1stExon	shore	1stExon-sh	chr14:814	rs1471379	9;18;34;44
cg19811761	-0.22637	0.312765	-5.01544	0.001198	0.00981	-0.91551	0.42595	0.19958	0.22637	19	57019022	F	I	ZNF471	TSS200	island	TSS200-isla	chr19:570	rs5474929	49
cg11082691	-0.20849	0.314947	-5.01508	0.001198	0.009812	-0.91597	0.41919	0.210704	0.208486	13	24183666	F	II	TNFRSF19	Body	opensea	Body-opens			
cg12963656	-0.22408	0.623548	-5.0141	0.0012	0.009819	-0.91723	0.73559	0.511507	0.224083	3	1.02E+08	F	II	LOC15222	TSS200	opensea	TSS200-ope		rs1854272	50
cg07487014	-0.23028	0.273897	-5.01104	0.001204	0.009843	-0.92115	0.389037	0.158758	0.230279	4	38491687	R	II	LINC01258	Body	opensea	Body-opens		rs5475432	26;11;7
cg15012027	-0.2247	0.328389	-5.01042	0.001205	0.009848	-0.92194	0.440737	0.216041	0.224696	2	99351550	R	II		IGR	shelf	IGR-shelf	chr2:9934		
cg22505924	-0.20843	0.298723	-5.00695	0.00121	0.009874	-0.92639	0.40294	0.194506	0.208434	2	64488157	F	II		IGR	opensea	IGR-opense		rs5762069	36;47
cg15429134	-0.2554	0.281093	-5.00229	0.001217	0.009906	-0.93237	0.408794	0.153393	0.255402	12	12619103	F	II	LOH12CR1	3'UTR	opensea	3'UTR-open		rs3803098	23;24;26;39
cg14992777	-0.21324	0.252341	-4.99895	0.001222	0.009927	-0.93667	0.358963	0.14572	0.213243	2	42879527	F	II	MTA3	Body	opensea	Body-opens		rs5275145	1;26
cg25104397	-0.25119	0.515581	-4.99789	0.001223	0.009935	-0.93803	0.641178	0.389985	0.251193	10	1.05E+08	R	II	C10orf26	5'UTR	opensea	5'UTR-open		rs5766124	29;28;27;4
cg13942283	-0.2592	0.539756	-4.99771	0.001223	0.009936	-0.93826	0.669354	0.410158	0.259196	10	1.13E+08	R	II	RBM20	Body	opensea	Body-opens		rs1442822	35;27
cg02725453	-0.20133	0.51931	-4.99613	0.001226	0.009949	-0.94029	0.619976	0.418645	0.201331	22	50249174	F	II	ZBED4	5'UTR	shore	5'UTR-shor	chr22:502	rs1860528	0;47
cg24368848	-0.30478	0.385789	-4.99499	0.001227	0.009958	-0.94176	0.538181	0.233397	0.304784	19	58545160	F	I	ZSCAN1	TSS1500	island	TSS1500-isl	chr19:585	rs5561099	15
cg12482300	-0.20776	0.248099	-4.99455	0.001228	0.009961	-0.94232	0.351979	0.144218	0.207761	1	2.27E+08	F	II	ITPKB	Body	opensea	Body-opens		rs1876040	12;27;38;47
cg01641371	-0.21301	0.2784	-4.99407	0.001229	0.009965	-0.94294	0.384904	0.171895	0.213009	17	26712092	R	II	SARM1	Body	island	Body-island	chr17:267	rs7847040	43;11
cg26280976	-0.28226	0.680787	-4.99117	0.001233	0.009987	-0.94664	0.821917	0.539657	0.28226	14	69660603	F	II		IGR	shore	IGR-shore	chr14:696	rs5405967	26;30;45

SUPPLEMENTAL TABLE 3 - FEMALE SUBSET - HYPO

	logFC	AveExpr	t	P.Value	adj.P.Val	B	LBP_f_AVG	Normal_f_A	deltaBeta	CHR	MAPINFO	Strand	Type	gene	feature	cgi	feat.cgi	UCSC_Islan	SNP_ID	SNP_DISTANCE
cg0420294	0.209857	0.508947	35.39074	1E-09	0.000315	11.74761	0.404019	0.613876	-0.20986	5	1.28E+08	R	II	FBN2	Body	opensea	Body-opens		rs1419626	7;4
cg1827416	0.211491	0.522894	33.57832	1.49E-09	0.000315	11.52651	0.417148	0.628639	-0.21149	8	1.05E+08	R	II		IGR	opensea	IGR-opense		rs1397863	38;27
cg0857742	0.259836	0.537106	32.561	1.88E-09	0.000315	11.39174	0.407188	0.667024	-0.25984	6	31876831	F	II	C2	5'UTR	opensea	5'UTR-oper		rs1909883	28
cg0894708	0.258033	0.583123	29.30234	4.18E-09	0.000315	10.89999	0.454106	0.712139	-0.25803	1	1.74E+08	R	II	SNORD47	TSS1500	shelf	TSS1500-sh	chr1:1738	rs5552291	27;1
cg2722910	0.219606	0.377589	27.20861	7.31E-09	0.000315	10.52718	0.267786	0.487393	-0.21961	20	47897260	R	II	SNORD12	Body	shelf	Body-shelf	chr20:4789	rs5460635	41;40;34;33
cg0468713	0.230976	0.53137	26.68574	8.46E-09	0.000315	10.426	0.415882	0.646859	-0.23098	20	50236499	F	II	ATP9A	Body	opensea	Body-opens		rs5596840	0;11;26
cg1197805	0.248211	0.505338	26.67024	8.5E-09	0.000315	10.42295	0.381233	0.629443	-0.24821	10	1.06E+08	F	II	COL17A1	TSS1500	opensea	TSS1500-op		rs5723495	5
cg2155461	0.227962	0.524228	26.43002	9.1E-09	0.000315	10.37526	0.410247	0.638209	-0.22796	14	55226755	F	II	SAMD4A	5'UTR	shore	5'UTR-shor	chr14:5522	rs5691047	23;27;51
cg0241131	0.248552	0.531953	25.50202	1.19E-08	0.000315	10.18377	0.407677	0.656229	-0.24855	11	9599530	R	II	WEE1	Body	shelf	Body-shelf	chr11:959	rs5722083	2
cg0703438	0.211441	0.474395	24.67509	1.53E-08	0.000315	10.00291	0.368675	0.580116	-0.21144	17	73149094	R	II	HN1	5'UTR	shore	5'UTR-shor	chr17:731	rs5488264	38
cg1171326	0.209985	0.502941	24.46769	1.63E-08	0.000315	9.955947	0.397948	0.607933	-0.20998	13	75940239	R	II	TBC1D4	Body	opensea	Body-opens		rs5772706	1
cg1222023	0.220385	0.469127	24.31051	1.71E-08	0.000315	9.919916	0.358935	0.57932	-0.22038	20	3364800	R	II	C20orf194	Body	opensea	Body-opens		rs8116213	39
cg1916297	0.235262	0.519432	24.26643	1.73E-08	0.000315	9.909742	0.401801	0.637063	-0.23526	7	38710069	F	II		IGR	opensea	IGR-opense		rs5568923	0
cg2305871	0.228046	0.639937	24.17748	1.78E-08	0.000315	9.889121	0.525914	0.75396	-0.22805	4	1779625	F	II		IGR	opensea	IGR-opense		rs5546610	0;1;12
cg0210186	0.215695	0.449262	24.02476	1.87E-08	0.000315	9.853422	0.341414	0.55711	-0.2157	11	74412729	F	II	CHRDL2	Body	opensea	Body-opens		rs3700805	1
cg1453852	0.206644	0.497267	24.0198	1.87E-08	0.000315	9.852258	0.393945	0.600589	-0.20664	4	23040711	F	II		IGR	opensea	IGR-opense		rs5336180	13
cg0881582	0.212884	0.495981	24.01856	1.87E-08	0.000315	9.851967	0.389539	0.602423	-0.21288	17	15169619	R	II	PMP22	TSS1500	shelf	TSS1500-sh	chr17:1516	rs5771876	2;1
cg0279598	0.250539	0.552601	23.72814	2.05E-08	0.000315	9.783016	0.427331	0.67787	-0.25054	10	81045119	F	II	ZMIZ1	Body	opensea	Body-opens		rs1487740	1;11;14;34;47
cg0893862	0.243642	0.515114	23.70481	2.06E-08	0.000315	9.777417	0.393293	0.636935	-0.24364	18	42832579	R	II	SLC14A2	5'UTR	opensea	5'UTR-oper			
cg1191067	0.226575	0.506969	23.6438	2.11E-08	0.000315	9.762735	0.393681	0.620257	-0.22658	2	27587064	F	II		IGR	opensea	IGR-opense		rs5358457	18;27
cg2698011	0.201592	0.629007	23.6396	2.11E-08	0.000315	9.761721	0.528211	0.729803	-0.20159	8	1.32E+08	F	II		IGR	opensea	IGR-opense		rs1900494	1;29;39
cg1627899	0.230398	0.581363	23.61572	2.12E-08	0.000315	9.755955	0.466164	0.696562	-0.2304	6	13715593	F	II		IGR	shelf	IGR-shelf	chr6:1371		
cg2429828	0.221865	0.530372	23.46349	2.23E-08	0.000315	9.71898	0.419439	0.641305	-0.22187	6	1.25E+08	F	II	RNF217	5'UTR	opensea	5'UTR-oper			
cg1124761	0.210434	0.474332	23.25405	2.39E-08	0.000315	9.667469	0.369116	0.579549	-0.21043	6	1.07E+08	R	II	LOC10042	Body	opensea	Body-opens			
cg0341152	0.224991	0.483614	23.15956	2.46E-08	0.000315	9.643984	0.371119	0.59611	-0.22499	17	66609980	R	II		IGR	opensea	IGR-opense		rs5415857	16;2
cg1647283	0.215438	0.487458	22.99689	2.59E-08	0.000315	9.603193	0.379739	0.595178	-0.21544	2	2.32E+08	F	II		IGR	opensea	IGR-opense		rs1167450	0;16
cg1616623	0.212171	0.498191	22.82403	2.75E-08	0.000315	9.559336	0.392105	0.604276	-0.21217	17	32480663	F	II	ACCN1	Body	shelf	Body-shelf	chr17:3248	rs5477043	1;17
cg1051172	0.251916	0.538298	22.8005	2.77E-08	0.000315	9.553326	0.41234	0.664256	-0.25192	14	24813353	F	II		IGR	opensea	IGR-opense			
cg0577212	0.254285	0.461583	22.54439	3.01E-08	0.000315	9.487264	0.33444	0.588726	-0.25429	16	31539169	R	II	AHSP	TSS200	opensea	TSS200-ope		rs7749354	15;2;1
cg0445242	0.255401	0.583744	22.35367	3.21E-08	0.000315	9.437293	0.456044	0.711445	-0.2554	6	1.51E+08	F	II	MTHFD1L	Body	opensea	Body-opens			
cg2368349	0.202524	0.455997	22.23102	3.35E-08	0.000315	9.4048	0.354735	0.557259	-0.20252	1	2.03E+08	R	II	CHIT1	Body	opensea	Body-opens		rs5432150	28;1
cg0932874	0.230687	0.550856	22.17108	3.41E-08	0.000315	9.388819	0.435512	0.666199	-0.23069	20	50031354	F	II	NFATC2	Body	opensea	Body-opens		rs5473995	26
cg0229387	0.2063	0.468343	21.91358	3.73E-08	0.000315	9.319389	0.365193	0.571493	-0.2063	3	1.24E+08	F	II	KALRN	Body	opensea	Body-opens		rs1114608	0;1;21;22
cg1381048	0.209232	0.480894	21.82236	3.85E-08	0.000315	9.294489	0.376278	0.58551	-0.20923	5	58440035	F	II	PDE4D	Body	opensea	Body-opens		rs5572610	0;20
cg0008472	0.214942	0.533913	21.78665	3.89E-08	0.000315	9.284698	0.426442	0.641384	-0.21494	8	94527855	R	II		IGR	opensea	IGR-opense		rs5651305	40
cg1055633	0.209495	0.505703	21.78346	3.9E-08	0.000315	9.283822	0.400956	0.610451	-0.20949	3	1.94E+08	R	II	LOC10050	Body	opensea	Body-opens		rs5371091	9
cg1067954	0.260315	0.557822	21.77766	3.91E-08	0.000315	9.282227	0.427664	0.687979	-0.26031	1	21532794	R	II		IGR	opensea	IGR-opense		rs5604575	1
cg1924378	0.229026	0.627274	21.75045	3.94E-08	0.000315	9.274745	0.512761	0.741787	-0.22903	3	23567565	F	II	UBE2E2	Body	opensea	Body-opens		rs5602612	1;20
cg1958801	0.248032	0.671793	21.74456	3.95E-08	0.000315	9.273124	0.547777	0.795809	-0.24803	2	9511523	F	II	ASAP2	Body	shelf	Body-shelf	chr2:9514	rs7465126	15;22;32
cg0754929	0.21681	0.612993	21.73664	3.96E-08	0.000315	9.270942	0.504588	0.721398	-0.21681	8	1.04E+08	R	II		IGR	opensea	IGR-opense		rs1473890	50;8;6
cg2179857	0.219801	0.671609	21.64299	4.09E-08	0.000315	9.245053	0.561709	0.78151	-0.2198	20	40091688	F	II	CHD6	Body	opensea	Body-opens		rs1818781	34
cg2026267	0.200669	0.554747	21.58815	4.17E-08	0.000315	9.229812	0.454412	0.655081	-0.20067	17	34891931	R	II	PIGW	5'UTR	shore	5'UTR-shor	chr17:348	rs5580998	36
cg0794770	0.213703	0.471998	21.58102	4.18E-08	0.000315	9.227826	0.365146	0.578849	-0.2137	19	58469897	R	II	C19orf18	3'UTR	opensea	3'UTR-oper		rs5488907	41;7;1
cg1107338	0.213073	0.524651	21.58089	4.18E-08	0.000315	9.22779	0.418114	0.631187	-0.21307	17	54305205	R	II	ANKFN1	Body	opensea	Body-opens		rs5615412	1
cg1658218	0.212367	0.528919	21.57334	4.19E-08	0.000315	9.225686	0.422735	0.635102	-0.21237	20	10935250	F	II		IGR	opensea	IGR-opense		rs1886133	0
cg1776812	0.254754	0.54012	21.56024	4.21E-08	0.000315	9.222033	0.412743	0.667497	-0.25475	6	56663406	R	II	DST	Body	opensea	Body-opens		rs1923125	36;30;14;1
cg0591831	0.211073	0.530815	21.52777	4.26E-08	0.000315	9.212961	0.425278	0.636351	-0.21107	2	1.83E+08	R	II	PDE1A	Body	opensea	Body-opens		rs1906086	4;2;1
cg2042731	0.236957	0.531015	21.43602	4.4E-08	0.000315	9.187217	0.412537	0.649494	-0.23696	6	1.35E+08	F	II		IGR	opensea	IGR-opense		rs5533134	9;27;28
cg1060315	0.208887	0.503558	21.42924	4.41E-08	0.000315	9.185309	0.399115	0.608002	-0.20889	10	13732625	R	II	FRMD4A	Body	opensea	Body-opens		rs1443379	42;2
cg0824537	0.213825	0.51366	21.41136	4.44E-08	0.000315	9.180269	0.406747	0.620572	-0.21383	17	25568385	F	II		IGR	opensea	IGR-opense		rs5603074	44;47
cg0504539	0.234093	0.513151	21.36752	4.5E-08	0.000315	9.167888	0.396105	0.630198	-0.23409	18	6249208	R	II	L3MBTL4	Body	opensea	Body-opens		rs5448058	50;26;14
cg1755753	0.210735	0.569451	21.34554	4.54E-08	0.000315	9.161663	0.464084	0.674819	-0.21073	9	90193634	R	II	DAPK1	Body	opensea	Body-opens		rs1433925	9
cg0743197	0.219246	0.533776	21.30754	4.6E-08	0.000315	9.150883	0.424153	0.643399	-0.21925	14	52142383	F	II	FRMD6	5'UTR	opensea	5'UTR-oper			
cg1222168	0.242662	0.537422	21.30454	4.61E-08	0.000315	9.15003	0.416091	0.658753	-0.24266	2	98827983	R	II	VWA3B	Body	opensea	Body-opens			
cg0441630	0.200799	0.599206	21.2619	4.67E-08	0.000315	9.137896	0.498807	0.699606	-0.2008	11	31143518	F	II		IGR	opensea	IGR-opense		rs1920425	1;10
cg2689856	0.239348	0.537021	21.26073	4.68E-08	0.000315	9.137562	0.417347	0.656695	-0.23935	17	10630621	F	II	TMEM220	Body	shelf	Body-shelf	chr17:1063	rs5403713	28;49;50
cg1793680	0.242053	0.562895	21.15926	4.85E-08	0.000315	9.108529	0.441869	0.683921	-0.24205	14	52334855	F	II	GNG2	5'UTR	opensea	5'UTR-oper		rs5736511	1;5
cg0711150	0.233171	0.518498	21.10673	4.94E-08	0.000315	9.093419	0.401912	0.635083	-0.23317	5	17352766	F	II		IGR	shore	IGR-shore	chr5:1735	rs5766065	1;32;39
cg1896824	0.235064	0.455857	21.01866	5.1E-08	0.000315	9.067952	0.338325	0.573388	-0.23506	4	1.24E+08	F	II	IL21-AS1	Body	opensea	Body-opens		rs1143013	9
cg2697586	0.262269	0.485331	20.91858	5.28E-08	0.000315	9.038816	0.354196	0.616465	-0.26227	1	57590662	F	II	DAB1	Body	opensea	Body-opens		rs5557882	1;26
cg2593948	0.211205	0.551482	20.85051	5.41E-08	0.000315	9.018879	0.44588	0.657084	-0.2112	1	69716492	R	II		IGR	opensea	IGR-opense		rs1833733	42;32;24;5;2;1
cg1746443	0.233736	0.597786	20.8469	5.42E-08	0.000315	9.017817	0.480918	0.714654	-0.23374	1	1.85E+08	R	II	FAM129A	Body	opensea	Body-opens		rs4651242	41;27;22
cg2080171	0.220645	0.406809	20.84433	5.42E-08	0.000315	9.017062	0.296487	0.517131	-0.22064	8	55278980	R	II		IGR	opensea	IGR-opense		rs5278555	37;29
cg0107200	0.221	0.486862	20.78057	5.55E-08	0.000315	8.998291	0.376362	0.597362	-0.221	6	1.36E+08	R	II	LINC00271	Body	opensea	Body-opens		rs5588935	1
cg0359373	0.223169	0.518038	20.76234	5.59E-08	0.000315	8.992907	0.406454	0.629622	-0.22317	2	2.02E+08	R	II	AOX2P	Body	opensea	Body-opens		rs7398839	36;22;13;1
cg0215026	0.201163	0.499079	20.7311	5.65E-08	0.000315	8.983664	0.398498	0.599661	-0.20116	11	1.25E+08	F	II	ROBO4	Body	opensea	Body-opens		rs5566130	0;3;21
cg0592619	0.251885	0.513047	20.70522	5.7E-08	0.000315	8.975992	0.387104	0.638989	-0.25189	15	57671053	F	II	CGNL1	5'UTR	shelf	5'UTR-shelf	chr15:576	rs5739496	1;4;12
cg0144104	0.217487	0.550788	20.68699	5.74E-08	0.000315	8.970579	0.442044	0.659532	-0.21749	16	19448742	F	II	TMC5	5'UTR	opensea	5'UTR-oper		rs1159719	43
cg2686447	0.222609	0.449209	20.65447	5.81E-08	0.000315	8.960906	0.337904	0.560514	-0.22261	5	39447279	F	II		IGR	opensea	IGR-opense		rs5333145	28;40;46
cg0446232	0.228149	0.597538	20.50141	6.14E-08	0.000315	8.915063	0.483464	0.711612	-0.22815	14	91697876	R	II		IGR	shelf	IGR-shelf	chr14:917	rs1422905	38;6;5
cg0468108	0.244063	0.528771	20.48786	6.17E-08	0.000315	8.91098	0.406739	0.650802	-0.24406	18	52918079	F	II	TCF4	Body	opensea	Body-opens		rs5684479	0
cg0273755	0.246365	0.564594	20.40578	6.36E-08	0.000315	8.886162	0.441412	0.687777	-0.24636	3	36690229	R	II		IGR	opensea	IGR-opense		rs5350842	29;15
cg1657603	0.253364	0.402697	20.40484	6.36E-08	0.000315	8.885879	0.276015	0.529379	-0.25336	1	2.35E+08	F	II		IGR	shore	IGR-shore	chr1:2352	rs1479996	1
cg1726353	0.229435	0.580681	20.36525	6.46E-08	0.000315	8.873854	0.465963	0.695398	-0.22943	8	1.46E+08	F	II	RPL8	Body	shore	Body-shore	chr8:1460	rs5711129	22;29;36
cg2497191	0.220383	0.662527	20.35779	6.48E-08	0.000315	8.871583	0.552335	0.772718	-0.22038	15	66404837	F	II	MEGF11	Body	opensea	Body-opens		rs5580055	4;24
cg2547679	0.21132	0.692693	20.35572	6.48E-08	0.000315	8.870954	0.587033	0.798354	-0.21132	8	1.17E+08	R	II	TRPS1	Body	opensea	Body-opens		rs5720543	43
cg0020511	0.207489	0.392729	20.29549	6.63E-08	0.000315	8.85258	0.288984	0.496473	-0.20749	7	86759403	F	II		IGR	opensea	IGR-opense		rs5512664	19
cg0317722	0.220553	0.673069	20.28153	6.66E-08	0.000315	8.848308	0.562792	0.783345	-0.22055	3	45117862	R	II		IGR	opensea	IGR-opense		rs5387119	21
cg2180951	0.217005	0.469511	20.26728	6.69E-08	0.000315	8.843947	0.361008	0.578014	-0.21701	14	50988575	R	II	MAP4K5	Body	opensea	Body-opens		rs5754857	48;38;30;2
cg2399336	0.261694	0.441304	20.24012	6.76E-08	0.000315	8.835617	0.310457	0.572151	-0.26169	14	50086035	R	II	MGAT2	TSS1500	shore	TSS1500-sh	chr14:500	rs1482780	47
cg2764641	0.204506	0.635994	20.21627	6.82E-08	0.000315	8.82829	0.533741	0.738247	-0.20451	10	77501817	F	II	C10orf11	Body	opensea	Body-opens		rs5582380	9;27
cg0680141	0.237241	0.458666	20.20941	6.84E-08	0.000315	8.82618	0.340046	0.577286	-0.23724	5	1.46E+08	F	II		IGR	shore	IGR-shore	chr5:1457	rs5438423	1
cg2588043	0.204706	0.409087	20.20228	6.86E-08	0.000315	8.823984	0.306734	0.511441	-0.20471	20	52756415	R	II		IGR	opensea	IGR-opense			
cg0912830	0.219003	0.450278	20.19426	6.88E-08	0.000315	8.821514	0.340776	0.559779	-0.219	2	82121644	F	II		IGR	opensea	IGR-opense			
cg1528408	0.220104	0.476743	20.18285	6.91E-08	0.000315	8.817999	0.366691	0.586795	-0.2201	10	77142527	R	II		IGR	opensea	IGR-opense		rs1932891	12
cg2529298	0.222072	0.542619	20.16036	6.97E-08	0.000315	8.811061	0.431583	0.653655	-0.22207	6	1.3E+08	F	II		IGR	opensea	IGR-opense		rs7762953	42
cg0314255	0.212815	0.458064	20.1235	7.06E-08	0.000315	8.799663	0.351657	0.564472	-0.21282	6	82958230	F	II	IBTK	TSS1500	shore	TSS1500-sh	chr6:8295	rs5657838	4;16
cg1984635	0.231112	0.531839	20.11734	7.08E-08	0.000315	8.797756	0.416283	0.647395	-0.23111	11	1.2E+08	R	II	PVRL1	Body	opensea	Body-opens		rs5327735	1
cg1670459	0.204215	0.377022	20.0808	7.17E-08	0.000315	8.786422	0.274914	0.47913	-0.20422	20	47896769	R	II	C20orf199	Body	shore	Body-shore	chr20:4789	rs1415615	49
cg0711014	0.238418	0.550494	20.07187	7.2E-08	0.000315	8.783645	0.431285	0.669703	-0.23842	5	1.42E+08	R	II	ARHGAP26	Body	opensea	Body-opens		rs5776527	36
cg1194181	0.208606	0.452856	20.04007	7.28E-08	0.000315	8.773751	0.348553	0.557159	-0.20861	3	1.33E+08	F	II	TMEM108	5'UTR	opensea	5'UTR-oper		rs5433467	0;1
cg0946920	0.23143	0.429414	19.96642	7.49E-08	0.000315	8.750746	0.313699	0.545129	-0.23143	4	1.6E+08	F	II		IGR	opensea	IGR-opense		rs5607921	30;47
cg0120143	0.20883	0.489597	19.96458	7.49E-08	0.000315	8.750169	0.385182	0.594012	-0.20883	6	1.06E+08	F	II		IGR	opensea	IGR-opense		rs5529253	42
cg1674247	0.204904	0.487924	19.92807	7.6E-08	0.000315	8.738717	0.385472	0.590376	-0.2049	9	13900570	R	II		IGR	opensea	IGR-opense		rs5435358	47;13;2
cg0543329	0.204595	0.437932	19.92456	7.61E-08	0.000315	8.737614	0.335634	0.540229	-0.20459	11	74814758	F	II		IGR	opensea	IGR-opense		rs5274595	2;5
cg0817733	0.255491	0.610804	19.88348	7.73E-08	0.000315	8.724688	0.483058	0.73855	-0.25549	3	98343882	F	II		IGR	opensea	IGR-opense			
cg1411437	0.221752	0.38474	19.87311	7.76E-08	0.000315	8.721418	0.273865	0.495616	-0.22175	19	45756845	R	II	MARK4	Body	shore	Body-shore	chr19:457		
cg1277675	0.20068	0.42828	19.85815	7.8E-08	0.000315	8.716698	0.32794	0.528621	-0.20068	17	45953055	R	II		IGR	shelf	IGR-shelf	chr17:459	rs5580288	28;24;19;2
cg0884183	0.20785	0.573492	19.83563	7.87E-08	0.000315	8.709582	0.469567	0.677417	-0.20785	15	39496814	R	II		IGR	opensea	IGR-opense		rs5411017	2;1
cg2262456	0.212036	0.41978	19.77664	8.04E-08	0.000315	8.690884	0.313761	0.525798	-0.21204	20	36271857	F	II		IGR	opensea	IGR-opense		rs5594301	0;23;27;31
cg0388999	0.239173	0.547599	19.77334	8.05E-08	0.000315	8.689834	0.428013	0.667186	-0.23917	1	1.58E+08	R	II	FCRL4	Body	opensea	Body-opens		rs5325413	0;2;16;24;34
cg1269206	0.24914	0.560722	19.6851	8.33E-08	0.000315	8.661709	0.436152	0.685292	-0.24914	4	1.58E+08	R	II		IGR	shelf	IGR-shelf	chr4:1578	rs1484839	33;16;12;2
cg0436921	0.249681	0.569415	19.64836	8.44E-08	0.000315	8.649942	0.444574	0.694256	-0.24968	5	1.39E+08	R	II		IGR	opensea	IGR-opense		rs5686674	22;20
cg1587459	0.21372	0.522691	19.62797	8.51E-08	0.000315	8.643396	0.415831	0.629551	-0.21372	16	29032168	R	II		IGR	opensea	IGR-opense		rs1454786	1
cg1536654	0.210893	0.505642	19.59791	8.61E-08	0.000316	8.633734	0.400196	0.611089	-0.21089	5	91888854	R	II		IGR	opensea	IGR-opense		rs5539390	1
cg2582450	0.203984	0.689048	19.5044	8.92E-08	0.000316	8.603527	0.587056	0.79104	-0.20398	3	42701690	R	II	ZBTB47	Body	shore	Body-shore	chr3:4270	rs5702300	39;30;2
cg1731254	0.250457	0.43745	19.50192	8.93E-08	0.000316	8.602722	0.312222	0.562679	-0.25046	7	24561256	F	II		IGR	opensea	IGR-opense		rs1844744	5;23;28
cg2286683	0.227955	0.526482	19.4412	9.14E-08	0.000316	8.582993	0.412505	0.64046	-0.22796	1	79151451	F	II		IGR	opensea	IGR-opense		rs5566962	47;49
cg2641674	0.21606	0.540833	19.42263	9.21E-08	0.000316	8.576942	0.432803	0.648863	-0.21606	1	34093069	F	II	CSMD2	Body	shelf	Body-shelf	chr1:3409	rs5568955	1
cg1017933	0.231499	0.481549	19.42097	9.21E-08	0.000316	8.5764	0.365799	0.597299	-0.2315	6	1.64E+08	F	II		IGR	opensea	IGR-opense		rs1464689	1;19
cg2632415	0.231049	0.550168	19.29179	9.68E-08	0.000321	8.534063	0.434644	0.665692	-0.23105	5	71490901	F	II	MAP1B	Body	opensea	Body-opens		rs5386090	0;6;18;47;51
cg2572919	0.223656	0.489307	19.26271	9.79E-08	0.000321	8.524477	0.37748	0.601135	-0.22366	1	17520216	R	II		IGR	opensea	IGR-opense			
cg0715399	0.24476	0.490579	19.23182	9.91E-08	0.000321	8.514267	0.3682	0.612959	-0.24476	5	1.72E+08	R	II	SH3PXD2B	Body	opensea	Body-opens		rs5689607	38;25;8
cg0582451	0.211831	0.487434	19.17214	1.01E-07	0.000321	8.494482	0.381519	0.59335	-0.21183	20	34482094	F	II	PHF20	Body	opensea	Body-opens		rs1126848	5;11
cg0007477	0.231969	0.516231	19.14115	1.03E-07	0.000321	8.484173	0.400246	0.632216	-0.23197	15	44120341	F	II	WDR76	Body	shore	Body-shore	chr15:441	rs5389829	1;7;12;13;44;51
cg2204033	0.260672	0.498809	19.13455	1.03E-07	0.000321	8.481973	0.368473	0.629145	-0.26067	13	28680729	F	II		IGR	opensea	IGR-opense		rs5363024	0;21
cg2325800	0.208987	0.565148	19.09683	1.04E-07	0.000321	8.469384	0.460654	0.669642	-0.20899	21	26881143	R	II		IGR	opensea	IGR-opense		rs1898105	39

cg2312263	0.220806	0.521513	19.09082	1.05E-07	0.000321	8.467376	0.411111	0.631916	-0.22081	7	73311043	R	II		IGR	opensea	IGR-opense		rs1889213	29;2;1
cg1468646	0.228226	0.651849	19.05202	1.06E-07	0.000321	8.454385	0.537736	0.765962	-0.22823	2	1.45E+08	R	II		IGR	opensea	IGR-opense		rs1928175	43
cg0369528	0.236065	0.5435	19.02905	1.07E-07	0.000321	8.446675	0.425468	0.661533	-0.23607	6	1.51E+08	R	II	PPP1R14C	Body	opensea	Body-opens		rs1889836	47;36;2
cg1186211	0.205745	0.505262	19.02878	1.07E-07	0.000321	8.446585	0.402389	0.608134	-0.20575	3	13579659	F	II		IGR	opensea	IGR-opense		rs5435268	37
cg1493729	0.212467	0.412849	18.99529	1.09E-07	0.000321	8.435321	0.306615	0.519082	-0.21247	1	1.78E+08	R	II	SEC16B	Body	opensea	Body-opens		rs1996610	45
cg2733980	0.20127	0.417283	18.96383	1.1E-07	0.000321	8.424715	0.316647	0.517918	-0.20127	10	72689218	R	II		IGR	opensea	IGR-opense		rs5717430	46;41;39;14
cg1686562	0.212972	0.423868	18.94245	1.11E-07	0.000321	8.417493	0.317382	0.530354	-0.21297	8	65758840	F	II		IGR	opensea	IGR-opense		rs5324719	0;25
cg2217691	0.202197	0.663721	18.88817	1.13E-07	0.000321	8.399105	0.562623	0.76482	-0.2022	11	11258914	R	II		IGR	opensea	IGR-opense		rs5756749	4
cg2382148	0.238868	0.623001	18.87649	1.14E-07	0.000321	8.395136	0.503568	0.742435	-0.23887	2	1.96E+08	F	II		IGR	opensea	IGR-opense		rs1160506	15
cg0348902	0.21411	0.512086	18.8565	1.15E-07	0.000321	8.388338	0.405031	0.619141	-0.21411	3	69819855	F	II	MITF	Body	opensea	Body-opens			
cg1512349	0.205456	0.496299	18.85393	1.15E-07	0.000321	8.387464	0.393571	0.599027	-0.20546	10	636241	R	II	DIP2C	Body	opensea	Body-opens		rs5615380	25;12;2
cg2257207	0.218652	0.458268	18.79816	1.18E-07	0.000321	8.368443	0.348942	0.567594	-0.21865	6	47074382	R	II		IGR	opensea	IGR-opense		rs1143630	38;31;11
cg1402406	0.213119	0.438942	18.79666	1.18E-07	0.000321	8.36793	0.332382	0.545502	-0.21312	7	1.16E+08	R	II		IGR	opensea	IGR-opense		rs5367369	49;37;30;1
cg0180551	0.216218	0.543072	18.79311	1.18E-07	0.000321	8.366715	0.434963	0.651181	-0.21622	11	1.03E+08	F	II	MMP12	TSS1500	opensea	TSS1500-op		rs1835895	0
cg0311612	0.221243	0.404709	18.76288	1.19E-07	0.000321	8.356368	0.294088	0.515331	-0.22124	1	2.31E+08	F	II		IGR	shelf	IGR-shelf	chr1:2312		
cg2725433	0.204105	0.488979	18.75465	1.2E-07	0.000321	8.353546	0.386927	0.591032	-0.20411	17	34192710	F	II	C17orf66	Body	opensea	Body-opens		rs1510993	40
cg0435190	0.218363	0.641584	18.743	1.2E-07	0.000321	8.349548	0.532403	0.750766	-0.21836	21	34441330	R	II	OLIG1	TSS1500	shore	TSS1500-sh	chr21:344	rs5614059	17
cg0179332	0.228594	0.434422	18.73179	1.21E-07	0.000321	8.345702	0.320125	0.548719	-0.22859	8	20030779	R	II	SLC18A1	Body	opensea	Body-opens		rs5531937	48;41;36;2;1
cg2167528	0.212202	0.55155	18.72482	1.21E-07	0.000321	8.343306	0.445448	0.657651	-0.2122	17	53392259	F	II	HLF	Body	opensea	Body-opens		rs7767993	17
cg2481946	0.207555	0.501534	18.71143	1.22E-07	0.000321	8.338701	0.397757	0.605312	-0.20755	1	28613551	R	II		IGR	opensea	IGR-opense		rs5580070	33;26;24;21
cg0554726	0.213227	0.611346	18.6946	1.22E-07	0.000321	8.332908	0.504733	0.71796	-0.21323	6	43611905	F	II	RSPH9	TSS1500	shore	TSS1500-sh	chr6:4361	rs5531703	43
cg1911996	0.217232	0.584892	18.6892	1.23E-07	0.000321	8.331045	0.476276	0.693508	-0.21723	1	2.37E+08	F	II	RYR2	Body	opensea	Body-opens		rs5493028	0;1;35;46;47
cg0915607	0.233461	0.500243	18.68493	1.23E-07	0.000321	8.329574	0.383513	0.616974	-0.23346	5	6491550	F	II	UBE2QL1	3'UTR	opensea	3'UTR-oper		rs5594770	10
cg0499485	0.20448	0.500025	18.66747	1.24E-07	0.000321	8.323549	0.397784	0.602265	-0.20448	12	26707553	R	II	ITPR2	Body	opensea	Body-opens			
cg0244800	0.200445	0.616806	18.65807	1.24E-07	0.000321	8.320305	0.516584	0.717028	-0.20044	9	4980116	F	II		IGR	opensea	IGR-opense		rs1477406	0;1;16
cg0269712	0.205623	0.407375	18.65541	1.24E-07	0.000321	8.319386	0.304563	0.510187	-0.20562	2	64628677	R	II		IGR	opensea	IGR-opense		rs3747222	2
cg0124900	0.244549	0.593314	18.65412	1.24E-07	0.000321	8.318939	0.47104	0.715589	-0.24455	6	1.45E+08	R	II	UTRN	Body	opensea	Body-opens		rs5722049	24;18
cg2593700	0.230017	0.503602	18.65272	1.25E-07	0.000321	8.318455	0.388594	0.618611	-0.23002	3	72309147	F	II		IGR	opensea	IGR-opense		rs5631104	0;17
cg0463974	0.233174	0.530217	18.64601	1.25E-07	0.000321	8.316138	0.41363	0.646804	-0.23317	3	63937019	F	II	ATXN7	Body	opensea	Body-opens		rs5665176	1
cg1334023	0.262519	0.583872	18.5907	1.28E-07	0.000322	8.296973	0.452613	0.715131	-0.26252	8	81549775	R	II		IGR	opensea	IGR-opense		rs5395290	2
cg0223407	0.232513	0.539299	18.57051	1.29E-07	0.000322	8.289954	0.423043	0.655556	-0.23251	5	67444023	R	II		IGR	opensea	IGR-opense		rs7312452	44;23
cg0357819	0.281307	0.495454	18.54594	1.3E-07	0.000322	8.281402	0.354801	0.636108	-0.28131	2	63671845	F	II	C2orf86	Body	opensea	Body-opens		rs1121230	0;41
cg1387684	0.208118	0.465058	18.52542	1.31E-07	0.000322	8.274247	0.360999	0.569117	-0.20812	3	84885990	R	II		IGR	opensea	IGR-opense		rs1483863	50;1
cg0196358	0.207714	0.487335	18.51264	1.32E-07	0.000322	8.269784	0.383477	0.591192	-0.20771	14	46389644	F	II		IGR	opensea	IGR-opense		rs1435248	17;24
cg0671774	0.237591	0.555273	18.49843	1.33E-07	0.000322	8.264821	0.436478	0.674069	-0.23759	8	23626859	F	II		IGR	opensea	IGR-opense		rs5431190	0;1;4;46
cg0075740	0.211744	0.501244	18.46002	1.35E-07	0.000323	8.251369	0.395372	0.607117	-0.21174	1	45050279	F	II	RNF220	Body	opensea	Body-opens		rs7407053	9;48
cg1653280	0.256283	0.523241	18.42268	1.37E-07	0.000323	8.238256	0.395099	0.651382	-0.25628	1	1.94E+08	R	II		IGR	opensea	IGR-opense		rs5756363	34;10
cg0653055	0.263095	0.525971	18.40892	1.37E-07	0.000323	8.233414	0.394424	0.657519	-0.2631	15	54304778	F	II	UNC13C	TSS1500	opensea	TSS1500-op		rs1861455	0
cg0434159	0.236313	0.572561	18.38756	1.39E-07	0.000324	8.225887	0.454405	0.690717	-0.23631	5	54357769	R	II		IGR	opensea	IGR-opense		rs1335719	36;31
cg2119473	0.214033	0.563871	18.38656	1.39E-07	0.000324	8.225532	0.456854	0.670887	-0.21403	20	48616106	F	II		IGR	opensea	IGR-opense		rs5590824	9;32
cg2335253	0.221479	0.444284	18.34896	1.41E-07	0.000325	8.212254	0.333544	0.555024	-0.22148	10	98136448	F	II	TLL2	ExonBnd	opensea	ExonBnd-op		rs1837512	26
cg1636809	0.21146	0.656139	18.30141	1.44E-07	0.000326	8.195405	0.550409	0.761868	-0.21146	7	7156638	F	II		IGR	opensea	IGR-opense		rs5737730	1
cg2104334	0.244703	0.552824	18.27734	1.45E-07	0.000326	8.186852	0.430473	0.675176	-0.2447	14	22247521	F	II		IGR	opensea	IGR-opense		rs1455863	1;4;6;40
cg2285719	0.20605	0.607308	18.2772	1.45E-07	0.000326	8.186803	0.504283	0.710333	-0.20605	4	1.42E+08	F	II		IGR	opensea	IGR-opense		rs5387319	1;48
cg1982035	0.208312	0.609983	18.27246	1.45E-07	0.000326	8.185116	0.505827	0.714138	-0.20831	11	68921264	F	II	LOC33869	Body	shelf	Body-shelf	chr11:689		
cg0126496	0.244578	0.47875	18.26696	1.46E-07	0.000326	8.183161	0.356461	0.601039	-0.24458	11	1.24E+08	F	II	OR10G4	TSS1500	opensea	TSS1500-op		rs1410213	1;20;26;31;38
cg0216970	0.21953	0.508541	18.22181	1.48E-07	0.000326	8.167062	0.398776	0.618306	-0.21953	8	1.08E+08	F	II	OXR1	Body	opensea	Body-opens		rs5388069	0;1;30
cg0533738	0.233244	0.519963	18.22138	1.48E-07	0.000326	8.166907	0.403341	0.636585	-0.23324	7	1.17E+08	F	II	ST7	Body	opensea	Body-opens			
cg0205947	0.229021	0.54884	18.20588	1.49E-07	0.000326	8.161368	0.434329	0.663351	-0.22902	1	68080498	F	II		IGR	opensea	IGR-opense		rs1116740	0;33;35
cg0770051	0.203021	0.561466	18.19065	1.5E-07	0.000326	8.15592	0.459955	0.662976	-0.20302	16	80603469	R	II	LINC01227	Body	opensea	Body-opens		rs5295520	51;48;43;1
cg1624718	0.22858	0.491218	18.16183	1.52E-07	0.000326	8.145589	0.376928	0.605508	-0.22858	17	63822421	R	II	CCDC46	Body	opensea	Body-opens		rs1999385	45;39;29;1
cg0129650	0.212912	0.606301	18.15956	1.52E-07	0.000326	8.144773	0.499845	0.712757	-0.21291	10	60627901	R	II		IGR	opensea	IGR-opense		rs7524255	29;22;16
cg2093931	0.2031	0.534038	18.15522	1.52E-07	0.000326	8.143217	0.432488	0.635587	-0.2031	8	30707701	R	II	TEX15	TSS1500	opensea	TSS1500-op		rs5422197	2
cg0417148	0.229933	0.593397	18.1497	1.53E-07	0.000326	8.141235	0.478431	0.708364	-0.22993	4	1.26E+08	F	II	FAT4	Body	opensea	Body-opens		rs5443897	0;15;32
cg1672955	0.247515	0.519716	18.12414	1.54E-07	0.000326	8.132047	0.395959	0.643474	-0.24752	1	2.07E+08	R	II	FCAMR	5'UTR	opensea	5'UTR-oper		rs1510587	31;21;2
cg2575734	0.210953	0.405043	18.10684	1.55E-07	0.000326	8.125816	0.299567	0.51052	-0.21095	5	1.71E+08	R	II		IGR	opensea	IGR-opense		rs1921510	20;38
cg2362101	0.20494	0.422973	18.08972	1.57E-07	0.000326	8.119642	0.320503	0.525443	-0.20494	7	1.35E+08	F	II	FAM180A	1stExon	opensea	1stExon-op		rs5441890	18;21;25;34
cg1123565	0.221728	0.460525	18.05507	1.59E-07	0.000326	8.107126	0.349661	0.571389	-0.22173	8	48525248	F	II	SPIDR	Body	opensea	Body-opens		rs1812416	0;1;42
cg1416439	0.25098	0.488072	18.02267	1.61E-07	0.000326	8.095387	0.362582	0.613563	-0.25098	17	28707127	R	II	CPD	TSS1500	shore	TSS1500-sh	chr17:287	rs5644120	7
cg0151071	0.225472	0.600152	18.00987	1.62E-07	0.000326	8.090744	0.487416	0.712888	-0.22547	15	66065837	F	II	DENND4A	5'UTR	opensea	5'UTR-oper		rs1874112	37
cg1738166	0.24447	0.56075	17.99946	1.63E-07	0.000326	8.086962	0.438515	0.682985	-0.24447	4	1.13E+08	R	II		IGR	opensea	IGR-opense		rs1432030	1
cg2652874	0.236491	0.501748	17.99766	1.63E-07	0.000326	8.086309	0.383503	0.619994	-0.23649	10	75240045	R	II	PPP3CB	5'UTR	opensea	5'UTR-oper		rs5349954	44;38
cg0111875	0.223429	0.681114	17.98609	1.63E-07	0.000326	8.082104	0.569399	0.792829	-0.22343	3	31771462	F	II	OSBPL10	Body	opensea	Body-opens		rs1414950	16
cg0514799	0.232838	0.507212	17.95093	1.66E-07	0.000326	8.069298	0.390792	0.623631	-0.23284	5	72982878	F	II	ARHGEF28	Body	opensea	Body-opens		rs1925181	40
cg1597360	0.264854	0.49665	17.94833	1.66E-07	0.000326	8.068349	0.364223	0.629077	-0.26485	5	96159726	F	II		IGR	opensea	IGR-opense		rs5397084	0;48
cg0470860	0.207374	0.405925	17.9253	1.68E-07	0.000326	8.05994	0.302238	0.509612	-0.20737	6	1.02E+08	R	II	GRIK2	Body	opensea	Body-opens		rs1135159	15;2;1
cg1964077	0.211666	0.435661	17.91071	1.69E-07	0.000326	8.054606	0.329828	0.541493	-0.21167	5	1.34E+08	R	II	C5orf66	5'UTR	opensea	5'UTR-oper		rs5580335	31
cg1253170	0.264609	0.548847	17.90927	1.69E-07	0.000326	8.05408	0.416542	0.681151	-0.26461	5	33834545	F	II	ADAMTS12	Body	opensea	Body-opens		rs1415835	0;1;3
cg2178416	0.236353	0.490417	17.86534	1.72E-07	0.000327	8.03798	0.372241	0.608593	-0.23635	14	65651398	R	II		IGR	opensea	IGR-opense		rs5378659	50
cg0361394	0.273522	0.450903	17.83621	1.74E-07	0.000327	8.027274	0.314142	0.587665	-0.27352	5	86416413	F	II		IGR	opensea	IGR-opense		rs5349473	43
cg1618538	0.230183	0.567378	17.82682	1.75E-07	0.000327	8.023816	0.452287	0.68247	-0.23018	13	29077350	F	II		IGR	opensea	IGR-opense		rs5431271	1
cg1015559	0.211743	0.568357	17.82251	1.75E-07	0.000327	8.022231	0.462486	0.674228	-0.21174	18	10423983	R	II		IGR	opensea	IGR-opense			
cg0822750	0.249642	0.592145	17.81166	1.76E-07	0.000327	8.018232	0.467324	0.716967	-0.24964	20	5743288	R	II	C20orf196	5'UTR	opensea	5'UTR-oper		rs1814599	17;2;1
cg2588209	0.264262	0.611212	17.79673	1.77E-07	0.000327	8.012727	0.479081	0.743343	-0.26426	20	62347673	F	II	ZGPAT	Body	opensea	Body-opens		rs5726000	1;42
cg1219263	0.204455	0.517315	17.76918	1.79E-07	0.000327	8.002546	0.415087	0.619542	-0.20445	10	1.13E+08	R	II	RBM20	Body	opensea	Body-opens		rs1887319	1;8;9;19
cg0534652	0.202019	0.689084	17.72968	1.82E-07	0.000327	7.987913	0.588075	0.790094	-0.20202	17	48542522	R	II	ACSF2	Body	shelf	Body-shelf	chr17:485	rs1844384	32;25
cg0769654	0.214817	0.546241	17.72888	1.82E-07	0.000327	7.987619	0.438833	0.653649	-0.21482	1	2.01E+08	R	II	PKP1	3'UTR	opensea	3'UTR-oper		rs1507578	1
cg0787921	0.246048	0.599209	17.7267	1.82E-07	0.000327	7.986809	0.476185	0.722233	-0.24605	14	63749933	F	II	RHOJ	ExonBnd	opensea	ExonBnd-op		rs5396262	16;21;22
cg1576412	0.223224	0.634484	17.71371	1.83E-07	0.000327	7.981985	0.522872	0.746097	-0.22322	4	1.07E+08	R	II	GIMD1	Body	opensea	Body-opens		rs1383325	2
cg0081838	0.210959	0.530047	17.70452	1.84E-07	0.000327	7.978569	0.424568	0.635527	-0.21096	5	33009006	R	II		IGR	opensea	IGR-opense		rs1851311	31;29;2
cg0558271	0.206066	0.543153	17.70379	1.84E-07	0.000327	7.9783	0.440121	0.646186	-0.20607	6	73043502	F	II	RIMS1	Body	opensea	Body-opens			
cg0051878	0.208446	0.594564	17.69204	1.85E-07	0.000327	7.97393	0.490341	0.698786	-0.20845	1	48912593	R	II	SPATA6	Body	opensea	Body-opens		rs7267934	47;32
cg0726007	0.249133	0.470708	17.68757	1.85E-07	0.000327	7.972264	0.346141	0.595274	-0.24913	19	45519636	F	II	RELB	Body	opensea	Body-opens		rs5424179	0
cg1038052	0.222001	0.5211	17.68476	1.85E-07	0.000327	7.971219	0.4101	0.632101	-0.222	10	1.08E+08	R	II	SORCS1	Body	opensea	Body-opens		rs1379283	51;37;27;11
cg1790500	0.210149	0.41964	17.67643	1.86E-07	0.000327	7.968117	0.314566	0.524714	-0.21015	7	83690400	R	II	SEMA3A	Body	opensea	Body-opens		rs5315737	18;2
cg1569329	0.217586	0.458799	17.67566	1.86E-07	0.000327	7.96783	0.350006	0.567592	-0.21759	12	7916371	F	II	LOC36003	TSS1500	opensea	TSS1500-op			
cg1354650	0.297591	0.528757	17.65684	1.88E-07	0.000327	7.960815	0.379962	0.677552	-0.29759	14	78864773	R	II	NRXN3	Body	opensea	Body-opens		rs5640816	2;1
cg1497441	0.213672	0.517331	17.62163	1.9E-07	0.000327	7.947657	0.410495	0.624167	-0.21367	3	24393915	F	II	THRB	5'UTR	opensea	5'UTR-oper			
cg1386835	0.230203	0.529253	17.61599	1.91E-07	0.000327	7.945545	0.414152	0.644354	-0.2302	9	1.03E+08	R	II		IGR	opensea	IGR-opense			
cg0846848	0.210434	0.416121	17.60798	1.91E-07	0.000327	7.942548	0.310905	0.521338	-0.21043	11	1.22E+08	F	II		IGR	opensea	IGR-opense		rs1823188	16;38
cg1126754	0.212508	0.628454	17.59221	1.93E-07	0.000327	7.936639	0.5222	0.734708	-0.21251	7	27167828	F	II	HOXA3	TSS1500	shore	TSS1500-sh	chr7:2716	rs1859162	27
cg2460606	0.207774	0.447831	17.54669	1.97E-07	0.000327	7.91954	0.343944	0.551718	-0.20777	17	44968863	F	II		IGR	opensea	IGR-opense		rs5666404	28;37;42;43;44
cg0900860	0.230016	0.518313	17.53022	1.98E-07	0.000327	7.913336	0.403305	0.633321	-0.23002	18	61814247	R	II	LINC00305	Body	opensea	Body-opens			
cg1993179	0.213028	0.587379	17.52771	1.98E-07	0.000327	7.912391	0.480865	0.693893	-0.21303	4	1.58E+08	F	II		IGR	opensea	IGR-opense		rs5324762	0;21
cg0311192	0.234087	0.515764	17.5265	1.98E-07	0.000327	7.911932	0.398721	0.632807	-0.23409	1	1.75E+08	F	II	RABGAP1L	Body	opensea	Body-opens		rs5559814	0
cg0652974	0.215701	0.521282	17.51967	1.99E-07	0.000327	7.90936	0.41432	0.629133	-0.2157	1	1.84E+08	R	II	RGL1	Body	opensea	Body-opens		rs5568437	42;34;2
cg1241986	0.20048	0.414226	17.51738	1.99E-07	0.000327	7.908494	0.313986	0.514466	-0.20048	5	31800978	F	II	PDZD2	Body	opensea	Body-opens		rs5755980	9;44
cg0588129	0.230321	0.486509	17.50619	2E-07	0.000327	7.904271	0.371348	0.601669	-0.23032	6	38322959	F	II	BTBD9	Body	opensea	Body-opens		rs1853913	0
cg2407122	0.240582	0.540094	17.50382	2E-07	0.000327	7.903377	0.419803	0.660385	-0.24058	1	29300664	F	II	EPB41	5'UTR	opensea	5'UTR-oper		rs5541785	10
cg0201891	0.210522	0.663783	17.50285	2E-07	0.000327	7.903012	0.558521	0.769044	-0.21052	6	1.24E+08	R	II		IGR	opensea	IGR-opense		rs1873534	49;14
cg0889672	0.228232	0.52364	17.44477	2.05E-07	0.000331	7.881027	0.409524	0.637756	-0.22823	11	19975201	R	II	NAV2	Body	opensea	Body-opens		rs2585777	10;2
cg0413161	0.224241	0.518256	17.44348	2.05E-07	0.000331	7.880538	0.406136	0.630376	-0.22424	3	46411447	R	II	CCR5	TSS200	opensea	TSS200-ope		rs5295473	41;17
cg0447285	0.202847	0.492959	17.39554	2.1E-07	0.000331	7.86232	0.391536	0.594382	-0.20285	12	59197803	F	II	LOC10050	Body	opensea	Body-opens		rs1436813	0
cg1669604	0.237587	0.507349	17.3934	2.1E-07	0.000331	7.861503	0.388555	0.626142	-0.23759	7	1.31E+08	R	II	LINC-PINT	Body	opensea	Body-opens			
cg1187671	0.208856	0.431205	17.36374	2.12E-07	0.000334	7.850192	0.326777	0.535633	-0.20886	11	32197287	R	II		IGR	opensea	IGR-opense		rs1845185	47;13
cg1512086	0.202472	0.377972	17.34829	2.14E-07	0.000335	7.84429	0.276736	0.479208	-0.20247	2	2.39E+08	F	II	RBM44	Body	opensea	Body-opens		rs5297306	14
cg1256927	0.201422	0.420729	17.34289	2.14E-07	0.000335	7.842228	0.320018	0.52144	-0.20142	4	1.07E+08	R	II		IGR	opensea	IGR-opense		rs1812003	0;26;34
cg1194507	0.225503	0.499007	17.31321	2.17E-07	0.000336	7.830864	0.386255	0.611759	-0.2255	6	56609630	R	II	RNU6-71P	Body	opensea	Body-opens		rs5426476	48;2
cg2410572	0.233038	0.612875	17.29259	2.19E-07	0.000336	7.822954	0.496356	0.729394	-0.23304	6	13007033	R	II	PHACTR1	Body	opensea	Body-opens			
cg2629692	0.225149	0.491619	17.26733	2.21E-07	0.000337	7.813248	0.379044	0.604193	-0.22515	6	1.25E+08	F	II	RNF217	Body	opensea	Body-opens		rs1504164	7;44
cg0456508	0.278699	0.464068	17.24782	2.23E-07	0.000337	7.805736	0.324718	0.603417	-0.2787	18	19184921	F	II		IGR	shelf	IGR-shelf	chr18:191		
cg1707548	0.211453	0.472152	17.23837	2.24E-07	0.000337	7.802093	0.366426	0.577879	-0.21145	11	68923332	F	II	LOC33869	Body	shore	Body-shore	chr11:689	rs1158705	17
cg0508452	0.269034	0.503883	17.23289	2.25E-07	0.000337	7.799981	0.369366	0.638399	-0.26903	8	1.3E+08	R	II		IGR	opensea	IGR-opense		rs5546598	1
cg2297339	0.22218	0.629901	17.23132	2.25E-07	0.000337	7.799378	0.518811	0.740991	-0.22218	21	35125405	F	II	ITSN1	Body	opensea	Body-opens		rs5497729	1;2;21;48
cg1581531	0.212289	0.519329	17.204	2.28E-07	0.000337	7.788826	0.413185	0.625474	-0.21229	14	60195269	R	II	RTN1	Body	shore	Body-shore	chr14:601	rs5424686	2
cg2540057	0.213135	0.495189	17.2006	2.28E-07	0.000337	7.787511	0.388622	0.601757	-0.21314	2	65804139	R	II		IGR	opensea	IGR-opense		rs5575694	35;1
cg2158530	0.217066	0.542574	17.18245	2.3E-07	0.000337	7.78049	0.434041	0.651107	-0.21707	3	1.33E+08	F	II	TF	Body	opensea	Body-opens		rs1892518	1
cg2216166	0.23561	0.412557	17.14619	2.33E-07	0.000337	7.766426	0.294752	0.530363	-0.23561	16	49709752	F	II	ZNF423	Body	opensea	Body-opens		rs5274302	38
cg1494629	0.253259	0.532763	17.14425	2.34E-07	0.000337	7.765673	0.406133	0.659392	-0.25326	18	74701280	F	II	MBP	Body	opensea	Body-opens		rs5632901	11;49

cg0434647	0.203123	0.45472	17.12357	2.36E-07	0.000337	7.757635	0.353159	0.556282	-0.20312	1	21958989	F	II	RAP1GAP	Body	opensea	Body-opens		rs1882229	19;21;26;28
cg1568874	0.20934	0.481512	17.12263	2.36E-07	0.000337	7.75727	0.376842	0.586182	-0.20934	10	78188497	R	II	C10orf11	Body	opensea	Body-opens			
cg0641082	0.261347	0.64365	17.11653	2.36E-07	0.000337	7.754896	0.512977	0.774324	-0.26135	18	74701492	R	II	MBP	Body	opensea	Body-opens			
cg0370971	0.205443	0.561079	17.1113	2.37E-07	0.000337	7.752859	0.458357	0.6638	-0.20544	3	1.34E+08	F	II		IGR	opensea	IGR-opense		rs7896191	1;25
cg2087293	0.21449	0.482443	17.11033	2.37E-07	0.000337	7.752481	0.375198	0.589688	-0.21449	21	40259397	F	II	LOC40086	Body	opensea	Body-opens		rs5583001	1;5
cg2077878	0.214648	0.640246	17.10179	2.38E-07	0.000337	7.749154	0.532922	0.74757	-0.21465	1	1.98E+08	F	II		IGR	opensea	IGR-opense			
cg2690455	0.245427	0.622829	17.0865	2.4E-07	0.000337	7.743191	0.500115	0.745543	-0.24543	2	10587170	F	II	ODC1	5'UTR	shore	5'UTR-shor	chr2:1058		
cg1842362	0.208248	0.535345	17.0803	2.4E-07	0.000337	7.740771	0.431221	0.639469	-0.20825	13	47994694	R	II		IGR	opensea	IGR-opense		rs7318036	40;7;2
cg0690521	0.210068	0.578566	17.06811	2.41E-07	0.000337	7.73601	0.473532	0.6836	-0.21007	2	1.28E+08	F	II	MYO7B	5'UTR	opensea	5'UTR-oper		rs1810168	0;5;19;41
cg0752962	0.210717	0.562993	17.05696	2.43E-07	0.000337	7.73165	0.457634	0.668352	-0.21072	8	1.31E+08	F	II	CCDC26	Body	opensea	Body-opens		rs1829455	0;2;19
cg1015420	0.223393	0.629529	17.04856	2.44E-07	0.000337	7.728365	0.517832	0.741226	-0.22339	11	39883276	R	II		IGR	opensea	IGR-opense		rs1148801	1
cg2764110	0.207479	0.585474	17.04679	2.44E-07	0.000337	7.727673	0.481734	0.689214	-0.20748	2	2.19E+08	R	II	CXCR2	TSS1500	opensea	TSS1500-op			
cg1488824	0.235138	0.437583	17.04615	2.44E-07	0.000337	7.727423	0.320014	0.555152	-0.23514	1	2.11E+08	R	II	KCNH1	Body	shore	Body-shore	chr1:2108	rs1167679	33;29;6;2;0
cg1388340	0.207063	0.393352	17.02983	2.46E-07	0.000337	7.721032	0.289821	0.496883	-0.20706	6	24974746	R	II	FAM65B	Body	opensea	Body-opens		rs5328979	25
cg1099000	0.246969	0.432649	17.02789	2.46E-07	0.000337	7.720269	0.309164	0.556133	-0.24697	17	65243288	F	II		IGR	shore	IGR-shore	chr17:652		
cg0080621	0.215653	0.449255	17.00673	2.48E-07	0.000337	7.711967	0.341429	0.557082	-0.21565	6	1.19E+08	F	II	SLC35F1	Body	opensea	Body-opens			
cg0491277	0.225307	0.656603	17.006	2.48E-07	0.000337	7.711681	0.54395	0.769256	-0.22531	10	78956948	F	II	KCNMA1	Body	opensea	Body-opens		rs1846892	0;1
cg0393199	0.250497	0.470631	17.0019	2.49E-07	0.000337	7.71007	0.345383	0.595879	-0.2505	4	88581953	F	II	DMP1	Body	opensea	Body-opens		rs7838077	26
cg1599751	0.240169	0.495571	16.99248	2.5E-07	0.000337	7.706369	0.375487	0.615655	-0.24017	1	81814874	R	II		IGR	opensea	IGR-opense			
cg1135877	0.238526	0.489725	16.9718	2.52E-07	0.000337	7.698235	0.370462	0.608988	-0.23853	6	1.14E+08	F	II	FLJ34503	Body	opensea	Body-opens		rs1388838	1;29;47
cg1274984	0.224917	0.440821	16.96977	2.52E-07	0.000337	7.697433	0.328363	0.553279	-0.22492	1	7315809	F	II	CAMTA1	Body	opensea	Body-opens		rs1458831	35;37
cg2541392	0.226091	0.517283	16.96683	2.52E-07	0.000337	7.696278	0.404237	0.630328	-0.22609	2	1.02E+08	F	II	TBC1D8	Body	opensea	Body-opens			
cg0669540	0.238738	0.659308	16.95823	2.53E-07	0.000337	7.692887	0.539939	0.778677	-0.23874	12	1.07E+08	F	II		IGR	opensea	IGR-opense		rs5272478	0;50
cg0717739	0.204664	0.588072	16.93555	2.56E-07	0.000338	7.68394	0.48574	0.690404	-0.20466	21	43100703	F	II	NCRNA001	Body	opensea	Body-opens		rs5617058	15
cg1370797	0.22281	0.617593	16.93503	2.56E-07	0.000338	7.683733	0.506188	0.728998	-0.22281	11	1.18E+08	F	II	TMPRSS4-A	Body	opensea	Body-opens		rs5299795	0;34
cg2733342	0.227527	0.541626	16.9301	2.56E-07	0.000338	7.681786	0.427863	0.65539	-0.22753	6	28552582	F	II	SCAND3	Body	shelf	Body-shelf	chr6:2855		
cg0450468	0.217561	0.472201	16.91115	2.59E-07	0.000338	7.674296	0.363421	0.580982	-0.21756	3	1.6E+08	F	II	SCHIP1	Body	opensea	Body-opens		rs3705470	0
cg0842608	0.254177	0.487306	16.91056	2.59E-07	0.000338	7.674062	0.360218	0.614394	-0.25418	1	43313732	R	II	ZNF691	5'UTR	shore	5'UTR-shor	chr1:4331	rs5665159	48;35;2
cg0877186	0.220294	0.550105	16.90869	2.59E-07	0.000338	7.67332	0.439958	0.660252	-0.22029	6	43264720	R	II	SLC22A7	TSS1500	opensea	TSS1500-op			
cg2381539	0.211566	0.429469	16.89687	2.6E-07	0.000338	7.668644	0.323686	0.535252	-0.21157	13	27827386	R	II	RPL21	5'UTR	shore	5'UTR-shor	chr13:278	rs1118004	48;21;2
cg1336981	0.221603	0.543231	16.88833	2.61E-07	0.000338	7.665258	0.43243	0.654033	-0.2216	1	55116866	F	II	C1orf175	Body	opensea	Body-opens		rs752467;r	25;33
cg2353059	0.218525	0.539414	16.8815	2.62E-07	0.000338	7.662552	0.430152	0.648677	-0.21852	15	48515109	R	II	SLC12A1	Body	opensea	Body-opens			
cg1489391	0.202892	0.503481	16.8804	2.62E-07	0.000338	7.662114	0.402035	0.604927	-0.20289	17	34394147	R	II	CCL18	Body	opensea	Body-opens			
cg2528667	0.21626	0.485942	16.87693	2.63E-07	0.000338	7.660739	0.377811	0.594072	-0.21626	1	2.46E+08	F	II		IGR	opensea	IGR-opense			
cg0983806	0.225489	0.593784	16.86672	2.64E-07	0.000338	7.656684	0.481039	0.706528	-0.22549	3	1.85E+08	R	II	IGF2BP2	Body	opensea	Body-opens		rs2121841	29;12;1
cg0905558	0.201004	0.473984	16.84619	2.66E-07	0.000338	7.648529	0.373482	0.574486	-0.201	15	31292849	R	II		IGR	opensea	IGR-opense		rs1164733	31;5;3;2;1
cg0106188	0.237406	0.547237	16.82766	2.68E-07	0.000338	7.641155	0.428534	0.66594	-0.23741	5	82822769	F	II	VCAN	Body	opensea	Body-opens		rs1448067	10;14
cg0465616	0.218717	0.425764	16.82541	2.69E-07	0.000338	7.640258	0.316405	0.535123	-0.21872	11	30453248	R	II	MPPED2	Body	opensea	Body-opens			
cg2301772	0.203687	0.523035	16.8006	2.72E-07	0.000339	7.630363	0.421191	0.624878	-0.20369	17	40110371	R	II	TTC25	Body	opensea	Body-opens		rs5410866	45
cg2548890	0.213613	0.517451	16.79876	2.72E-07	0.000339	7.629629	0.410645	0.624257	-0.21361	3	1.75E+08	F	II	NAALADL2	Body	opensea	Body-opens		rs1415000	5;40
cg2546931	0.22872	0.599307	16.79098	2.73E-07	0.000339	7.626521	0.484947	0.713667	-0.22872	1	48704831	R	II	SLC5A9	Body	opensea	Body-opens		rs7906706	40;24
cg0819190	0.221675	0.476679	16.789	2.73E-07	0.000339	7.625731	0.365841	0.587517	-0.22168	14	32769511	F	II		IGR	opensea	IGR-opense		rs5329718	0
cg1114169	0.321047	0.486606	16.78144	2.74E-07	0.000339	7.622712	0.326082	0.64713	-0.32105	1	1.62E+08	R	II		IGR	shelf	IGR-shelf	chr1:1616	rs5487993	30;13
cg1961257	0.23885	0.626432	16.75989	2.77E-07	0.000339	7.614089	0.507008	0.745857	-0.23885	1	2E+08	F	II		IGR	opensea	IGR-opense		rs5744837	0;1;33;50
cg0661097	0.204014	0.534354	16.75067	2.78E-07	0.000339	7.610397	0.432347	0.636361	-0.20401	2	1.14E+08	F	II		IGR	opensea	IGR-opense		rs6744051	46
cg2126988	0.241815	0.536625	16.75019	2.78E-07	0.000339	7.610202	0.415718	0.657533	-0.24181	13	90489341	F	II		IGR	opensea	IGR-opense		rs1486009	31
cg0365245	0.200801	0.548227	16.74798	2.78E-07	0.000339	7.609317	0.447827	0.648628	-0.2008	6	1670293	F	II	GMDS	Body	opensea	Body-opens		rs1713414	31;32;51
cg2651001	0.261707	0.579982	16.73278	2.8E-07	0.000339	7.603222	0.449128	0.710835	-0.26171	3	1.87E+08	R	II	EIF4A2	Body	shelf	Body-shelf	chr3:1865	rs5272957	50;43;14;1
cg0744033	0.215571	0.587061	16.71984	2.81E-07	0.000339	7.598024	0.479276	0.694847	-0.21557	14	1.07E+08	F	II		IGR	opensea	IGR-opense		rs1902660	0;16;39
cg1512074	0.20018	0.521694	16.71542	2.82E-07	0.000339	7.596248	0.421604	0.621785	-0.20018	2	9211406	F	II		IGR	opensea	IGR-opense		rs1446830	0
cg0060705	0.218725	0.583397	16.71208	2.82E-07	0.000339	7.594906	0.474035	0.69276	-0.21873	4	89733526	R	II	FAM13A	Body	opensea	Body-opens		rs1494121	1
cg0722501	0.201069	0.494824	16.70736	2.83E-07	0.000339	7.593011	0.394289	0.595359	-0.20107	17	74913153	F	II	MGAT5B	Body	opensea	Body-opens		rs8008971	0;1;51
cg2323867	0.227855	0.585287	16.70455	2.83E-07	0.000339	7.591878	0.471359	0.699215	-0.22786	2	1.7E+08	F	II	LRP2	Body	opensea	Body-opens		rs5465522	46
cg0425867	0.22099	0.628283	16.69179	2.85E-07	0.00034	7.586743	0.517788	0.738778	-0.22099	1	94240208	F	II		IGR	opensea	IGR-opense		rs5343663	3;26;32
cg2406547	0.238218	0.586696	16.67295	2.87E-07	0.000341	7.579153	0.467587	0.705806	-0.23822	6	1.46E+08	R	II	GRM1	Body	opensea	Body-opens			
cg2506620	0.226647	0.586908	16.67283	2.87E-07	0.000341	7.579103	0.473584	0.700232	-0.22665	6	56669999	F	II	DST	Body	opensea	Body-opens			
cg2001568	0.209375	0.395324	16.66922	2.88E-07	0.000341	7.577648	0.290637	0.500011	-0.20937	20	43835190	R	II	SEMG1	TSS1500	opensea	TSS1500-op		rs3751395	44;12
cg0136295	0.205554	0.40917	16.66452	2.88E-07	0.000341	7.575752	0.306393	0.511947	-0.20555	6	1.11E+08	R	II	METTL24	Body	opensea	Body-opens		rs7885796	2
cg2320754	0.205248	0.473933	16.6617	2.89E-07	0.000341	7.574616	0.371309	0.576557	-0.20525	2	66050436	F	II		IGR	opensea	IGR-opense			
cg0814071	0.227468	0.558584	16.6494	2.9E-07	0.000342	7.569648	0.44485	0.672318	-0.22747	3	1.27E+08	F	II		IGR	shelf	IGR-shelf	chr3:1271	rs5698110	1;10;17
cg2647658	0.216907	0.611914	16.64172	2.91E-07	0.000342	7.566546	0.503461	0.720368	-0.21691	1	2.33E+08	F	II		IGR	opensea	IGR-opense		rs5310591	36
cg1616041	0.227601	0.496085	16.62001	2.94E-07	0.000342	7.557763	0.382285	0.609886	-0.2276	8	1.29E+08	R	II		IGR	opensea	IGR-opense		rs5425783	1
cg1994907	0.21574	0.595029	16.61836	2.94E-07	0.000342	7.557095	0.487159	0.702898	-0.21574	3	1.86E+08	R	II	DGKG	Body	opensea	Body-opens		rs5422613	30;12
cg2069907	0.202673	0.502428	16.60974	2.96E-07	0.000342	7.553602	0.401092	0.603765	-0.20267	2	1.37E+08	R	II		IGR	opensea	IGR-opense		rs605887;r	50;35;2
cg2356324	0.211429	0.472571	16.60834	2.96E-07	0.000342	7.553033	0.366856	0.578285	-0.21143	7	15596973	F	II	AGMO	Body	opensea	Body-opens			
cg1242571	0.206118	0.483653	16.58454	2.99E-07	0.000343	7.543379	0.380594	0.586713	-0.20612	5	1.27E+08	F	II	MEGF10	Body	opensea	Body-opens		rs5585900	4;47
cg1448804	0.205644	0.61632	16.56879	3.01E-07	0.000344	7.536977	0.513498	0.719142	-0.20564	11	8788397	F	II	ST5	5'UTR	opensea	5'UTR-oper		rs5312581	37;45
cg1526979	0.231464	0.488314	16.56407	3.02E-07	0.000344	7.535058	0.372582	0.604046	-0.23146	5	68824022	F	II	OCLN	Body	opensea	Body-opens		rs5370652	0
cg0554849	0.241482	0.58895	16.5386	3.05E-07	0.000345	7.524688	0.468209	0.709691	-0.24148	7	81320956	F	II	LOC10012	TSS1500	opensea	TSS1500-op			
cg1324429	0.205859	0.479988	16.52737	3.07E-07	0.000345	7.520109	0.377058	0.582917	-0.20586	3	25832283	F	II	NGLY1	TSS1500	opensea	TSS1500-op		rs5612198	1;14;26
cg0929058	0.20714	0.403352	16.51231	3.09E-07	0.000345	7.513961	0.299782	0.506922	-0.20714	5	27821960	F	II		IGR	opensea	IGR-opense		rs5322509	38;40;44
cg2310117	0.208466	0.589551	16.50586	3.1E-07	0.000345	7.511326	0.485318	0.693784	-0.20847	1	1.59E+08	R	II	OR6N2	TSS1500	opensea	TSS1500-op		rs7454326	35;34
cg0755306	0.272134	0.551795	16.49424	3.11E-07	0.000345	7.506574	0.415728	0.687863	-0.27213	18	54118660	F	II		IGR	opensea	IGR-opense		rs5741838	29;47
cg2392356	0.20716	0.492891	16.48822	3.12E-07	0.000345	7.504114	0.38931	0.596471	-0.20716	10	35243037	R	II		IGR	opensea	IGR-opense			
cg0594238	0.212828	0.540994	16.4762	3.14E-07	0.000345	7.49919	0.43458	0.647408	-0.21283	5	60960537	F	II	LOC10192	Body	opensea	Body-opens		rs36032;rs	7;8;36
cg0191222	0.232164	0.590149	16.47325	3.14E-07	0.000345	7.497982	0.474067	0.706231	-0.23216	2	1.57E+08	R	II	LOC10192	Body	opensea	Body-opens		rs1134270	45;38
cg1168837	0.237982	0.700126	16.46653	3.15E-07	0.000345	7.495228	0.581135	0.819117	-0.23798	1	49238689	F	II	BEND5	Body	shelf	Body-shelf	chr1:4924	rs1509334	1;7;10;16;18;19;27
cg1505818	0.258392	0.483074	16.44608	3.18E-07	0.000345	7.486838	0.353878	0.61227	-0.25839	2	60715593	F	II	BCL11A	Body	opensea	Body-opens		rs5776203	4
cg1928209	0.239702	0.50605	16.43386	3.2E-07	0.000345	7.481817	0.386199	0.625901	-0.2397	18	61173138	R	II		IGR	opensea	IGR-opense			
cg2091237	0.223212	0.458553	16.43128	3.2E-07	0.000345	7.480757	0.346947	0.570159	-0.22321	6	1.22E+08	R	II		IGR	opensea	IGR-opense		rs7643694	50
cg2600268	0.309502	0.553386	16.42132	3.22E-07	0.000345	7.476662	0.398635	0.708137	-0.3095	9	85835742	F	II		IGR	opensea	IGR-opense			
cg0009810	0.211144	0.554462	16.41873	3.22E-07	0.000345	7.475594	0.44889	0.660034	-0.21114	2	2.3E+08	R	II	PID1	Body	opensea	Body-opens		rs5428015	2
cg0564067	0.252053	0.559991	16.40879	3.24E-07	0.000345	7.471503	0.433964	0.686018	-0.25205	12	68513016	R	II		IGR	opensea	IGR-opense		rs5353636	34;17
cg0098791	0.211275	0.447388	16.40158	3.25E-07	0.000345	7.468531	0.34175	0.553025	-0.21127	6	30115829	R	II	TRIM40	3'UTR	opensea	3'UTR-oper			
cg1721881	0.229634	0.562495	16.39926	3.25E-07	0.000345	7.467575	0.447678	0.677312	-0.22963	2	2.17E+08	F	II	LINC00607	Body	opensea	Body-opens		rs1446235	0;30
cg2708657	0.218748	0.632672	16.39048	3.26E-07	0.000345	7.463954	0.523298	0.742046	-0.21875	22	33093513	F	II	SYN3	Body	opensea	Body-opens		rs5753825	0;1;9
cg0140312	0.213655	0.598628	16.38824	3.27E-07	0.000345	7.463032	0.4918	0.705455	-0.21365	17	25566446	F	II		IGR	opensea	IGR-opense		rs5447275	28
cg2555513	0.207254	0.511298	16.37803	3.28E-07	0.000345	7.458818	0.407671	0.614925	-0.20725	9	3191504	R	II	LINC01231	Body	opensea	Body-opens		rs1181323	7;2
cg2183508	0.239901	0.601273	16.35736	3.31E-07	0.000345	7.450277	0.481322	0.721223	-0.2399	1	2.27E+08	F	II	CDC42BPA	Body	opensea	Body-opens		rs5333009	28
cg0810529	0.202935	0.588807	16.34832	3.33E-07	0.000345	7.446539	0.48734	0.690275	-0.20294	3	8694758	F	II	SSUH2	TSS1500	opensea	TSS1500-op		rs5497792	34
cg0715242	0.227058	0.479683	16.33756	3.34E-07	0.000345	7.442081	0.366153	0.593212	-0.22706	2	33713297	R	II	RASGRP3	5'UTR	opensea	5'UTR-oper		rs5339412	6;2;1
cg1120961	0.224757	0.454163	16.32835	3.36E-07	0.000345	7.438267	0.341784	0.566541	-0.22476	7	1.18E+08	F	II		IGR	opensea	IGR-opense		rs5410183	28
cg0356705	0.227258	0.550151	16.32418	3.36E-07	0.000345	7.436536	0.436522	0.66378	-0.22726	9	84108400	F	II		IGR	opensea	IGR-opense		rs5370476	1;15;37
cg1181881	0.255675	0.484341	16.32317	3.36E-07	0.000345	7.436118	0.356503	0.612179	-0.25568	1	1.52E+08	F	II	SNX27	Body	opensea	Body-opens		rs5544119	0;9;50
cg1768061	0.220441	0.508509	16.31704	3.37E-07	0.000345	7.433575	0.398288	0.618729	-0.22044	19	16191217	F	II	TPM4	Body	shelf	Body-shelf	chr19:161	rs5611485	4;7;11;31;41;50
cg1731702	0.217328	0.551391	16.31518	3.38E-07	0.000345	7.432805	0.442727	0.660055	-0.21733	2	86176514	R	II		IGR	opensea	IGR-opense		rs5535900	25;2
cg0426365	0.256355	0.480572	16.30015	3.4E-07	0.000345	7.426561	0.352394	0.608749	-0.25636	19	30521287	R	II		IGR	opensea	IGR-opense		rs7805828	44
cg1505675	0.233953	0.521544	16.29937	3.4E-07	0.000345	7.426238	0.404568	0.63852	-0.23395	3	3169441	R	II	TRNT1	5'UTR	shore	5'UTR-shor	chr3:3168	rs7923504	39;38;32;20;6
cg2151217	0.247529	0.466565	16.2909	3.41E-07	0.000345	7.422719	0.3428	0.590329	-0.24753	2	85971548	F	II		IGR	opensea	IGR-opense		rs1885887	1
cg2115698	0.237538	0.602607	16.27685	3.44E-07	0.000345	7.416873	0.483838	0.721376	-0.23754	1	1.57E+08	R	II	NTRK1	Body	opensea	Body-opens		rs1742586	49;41;39;2
cg1375818	0.220579	0.443985	16.27032	3.45E-07	0.000345	7.414155	0.333696	0.554275	-0.22058	2	1.02E+08	R	II		IGR	opensea	IGR-opense			
cg0953136	0.220163	0.439138	16.2496	3.48E-07	0.000345	7.405519	0.329056	0.549219	-0.22016	10	74436333	F	II		IGR	opensea	IGR-opense		rs5569122	1;17;29
cg0153080	0.225357	0.578758	16.23866	3.5E-07	0.000345	7.400952	0.46608	0.691437	-0.22536	22	30877800	R	II	SDC4P	TSS200	opensea	TSS200-ope		rs5627715	37;32
cg2284218	0.211613	0.539757	16.23597	3.5E-07	0.000345	7.399829	0.43395	0.645563	-0.21161	3	24401786	R	II	THRB	5'UTR	opensea	5'UTR-oper			
cg0896198	0.210609	0.580916	16.2297	3.51E-07	0.000345	7.397206	0.475612	0.686221	-0.21061	11	1.04E+08	R	II	PDGFD	Body	opensea	Body-opens		rs5614896	42
cg1862331	0.229682	0.52035	16.21823	3.53E-07	0.000345	7.392413	0.405508	0.635191	-0.22968	4	55711209	R	II		IGR	opensea	IGR-opense		rs5656072	51;1
cg0468648	0.205233	0.459994	16.216	3.53E-07	0.000345	7.391482	0.357377	0.56261	-0.20523	2	1.79E+08	R	II	PDE11A	Body	opensea	Body-opens		rs5558419	44;1
cg0623370	0.200707	0.462696	16.20865	3.54E-07	0.000345	7.388403	0.362343	0.56305	-0.20071	10	71768803	R	II		IGR	opensea	IGR-opense		rs5439855	28;22
cg1281236	0.205646	0.507345	16.20469	3.55E-07	0.000345	7.386745	0.404521	0.610168	-0.20565	5	1.02E+08	R	II	LINC00492	Body	opensea	Body-opens		rs5589742	30;2
cg2606220	0.21995	0.481249	16.20159	3.56E-07	0.000345	7.38545	0.371274	0.591224	-0.21995	3	1.51E+08	F	II	CLRN1	5'UTR	opensea	5'UTR-oper		rs5547214	22
cg1023505	0.214125	0.517494	16.19975	3.56E-07	0.000345	7.384679	0.410431	0.624556	-0.21413	3	1.34E+08	R	II	SLCO2A1	Body	opensea	Body-opens		rs5597786	44;11;2
cg0671739	0.212328	0.558153	16.18367	3.59E-07	0.000345	7.37794	0.451989	0.664317	-0.21233	7	1.22E+08	R	II	PTPRZ1	Body	opensea	Body-opens		rs7844136	34;33
cg0884791	0.255298	0.470992	16.18253	3.59E-07	0.000345	7.377461	0.343342	0.598641	-0.2553	1	25105247	F	II	CLIC4	Body	opensea	Body-opens		rs7553761	9;10;24;29
cg0807812	0.208037	0.654646	16.17866	3.59E-07	0.000345	7.375838	0.550627	0.758664	-0.20804	3	1.57E+08	F	II	VEPH1	Body	opensea	Body-opens		rs5603495	40
cg0861487	0.258659	0.386585	16.17412	3.6E-07	0.000346	7.37393	0.257256	0.515915	-0.25866	10	3824555	R	II	KLF6	Body	shore	Body-shore	chr10:382	rs1490189	21;2
cg0591280	0.205167	0.377454	16.17131	3.61E-07	0.000346	7.372752	0.27487	0.480038	-0.20517	9	37378390	F	II		IGR	opensea	IGR-opense		rs5759998	27
cg1797184	0.225011	0.679014	16.16667	3.61E-07	0.000346	7.370805	0.566508	0.791519	-0.22501	2	1.92E+08	F	II	MYO1B	5'UTR	opensea	5'UTR-oper		rs1114378	0;9
cg2760937	0.247198	0.493853	16.16314	3.62E-07	0.000346	7.369322	0.370254	0.617452	-0.2472	2	1.87E+08	R	II		IGR	opensea	IGR-opense		rs1446080	47;18
cg2300164	0.219569	0.594291	16.13038	3.67E-07	0.000348	7.355539	0.484506	0.704076	-0.21957	3	1.72E+08	R	II	TMEM212-	TSS1500	opensea	TSS1500-op		rs1501486	43;17

cg1005317	0.264075	0.538391	16.12601	3.68E-07	0.000348	7.353698	0.406354	0.670429	-0.26408	20	48924790	R	II	LINC01270	Body	opensea	Body-opens		rs5403083	48
cg1965921	0.208254	0.458125	16.12179	3.69E-07	0.000348	7.351919	0.353998	0.562252	-0.20825	12	21488180	R	II	SLCO1A2	5'UTR	opensea	5'UTR-oper			
cg1335547	0.235673	0.483451	16.11771	3.7E-07	0.000348	7.350201	0.365614	0.601287	-0.23567	6	17431988	R	II	CAP2	Body	opensea	Body-opens		rs1884508	34;2
cg2481404	0.241679	0.499786	16.11381	3.7E-07	0.000348	7.348554	0.378946	0.620625	-0.24168	1	44550872	F	II		IGR	opensea	IGR-opense		rs1399423	0;46
cg0981126	0.243096	0.518095	16.10574	3.72E-07	0.000348	7.345151	0.396547	0.639643	-0.2431	20	20477152	R	II	RALGAPA2	Body	opensea	Body-opens		rs5434792	34
cg1433204	0.220282	0.581573	16.07591	3.77E-07	0.000349	7.332542	0.471432	0.691714	-0.22028	6	1.53E+08	R	II		IGR	opensea	IGR-opense		rs1174476	46;14;2;1
cg1384445	0.204343	0.487407	16.07488	3.77E-07	0.000349	7.33211	0.385236	0.589579	-0.20434	17	41650882	F	II		IGR	opensea	IGR-opense			
cg2611327	0.234929	0.515831	16.0699	3.78E-07	0.000349	7.330002	0.398367	0.633295	-0.23493	1	54484669	R	II	LDLRAD1	TSS1500	opensea	TSS1500-op		rs2000641	51;49;2;1
cg0321923	0.21985	0.442889	16.05228	3.81E-07	0.000349	7.322536	0.332964	0.552814	-0.21985	14	88336254	R	II		IGR	opensea	IGR-opense		rs1924528	32
cg0069092	0.221374	0.506752	16.0501	3.81E-07	0.000349	7.321611	0.396065	0.617439	-0.22137	1	9118237	F	II	SLC2A5	Body	opensea	Body-opens		rs5354539	1;10;15
cg1133635	0.213357	0.582938	16.04549	3.82E-07	0.000349	7.319658	0.476259	0.689616	-0.21336	1	1.6E+08	R	II	NHLH1	5'UTR	shelf	5'UTR-shelf	chr1:1603		
cg2436679	0.253285	0.510911	16.03888	3.83E-07	0.000349	7.316853	0.384268	0.637553	-0.25328	17	55884705	F	II		IGR	opensea	IGR-opense		rs5328610	10;40;49
cg1156488	0.238091	0.425271	16.038	3.83E-07	0.000349	7.316481	0.306225	0.544317	-0.23809	9	71040282	R	II	PGM5	Body	opensea	Body-opens			
cg1063175	0.23231	0.628701	16.0361	3.84E-07	0.000349	7.315674	0.512545	0.744856	-0.23231	13	52985702	F	II		IGR	opensea	IGR-opense		rs1136012	21;43
cg1960840	0.201443	0.58603	16.02668	3.85E-07	0.000349	7.311674	0.485309	0.686752	-0.20144	6	49604722	R	II	RHAG	TSS200	opensea	TSS200-ope		rs5370302	34;24;19;1
cg2279975	0.249052	0.496756	16.02612	3.86E-07	0.000349	7.311434	0.37223	0.621282	-0.24905	7	29997642	R	II	SCRN1	Body	opensea	Body-opens		rs1487894	38;20;14
cg0034079	0.25175	0.58353	16.02523	3.86E-07	0.000349	7.311058	0.457655	0.709405	-0.25175	2	2.16E+08	F	II	LINC00607	Body	opensea	Body-opens			
cg2763605	0.208446	0.583997	16.02025	3.87E-07	0.000349	7.308942	0.479774	0.68822	-0.20845	11	57443308	R	II	ZDHHC5	Body	opensea	Body-opens		rs5466696	29;1
cg1165890	0.215891	0.544317	16.01366	3.88E-07	0.000349	7.306139	0.436372	0.652263	-0.21589	1	1.56E+08	R	II	ASH1L	5'UTR	opensea	5'UTR-oper		rs1902674	7;1
cg0033119	0.206157	0.585862	15.99776	3.91E-07	0.000349	7.299373	0.482784	0.68894	-0.20616	8	67638560	F	II	C8orf44-SG	5'UTR	opensea	5'UTR-oper		rs5377138	1;12
cg1748236	0.238288	0.50068	15.98754	3.93E-07	0.000349	7.295019	0.381535	0.619824	-0.23829	3	1.74E+08	R	II	NLGN1	Body	opensea	Body-opens		rs5563511	47;46;6;4;1
cg0746050	0.224656	0.554453	15.98029	3.94E-07	0.000349	7.29193	0.442125	0.666781	-0.22466	1	1.79E+08	F	II		IGR	opensea	IGR-opense		rs1410960	1;41
cg2202944	0.22092	0.509918	15.9735	3.95E-07	0.000349	7.289032	0.399458	0.620378	-0.22092	9	79136110	R	II		IGR	opensea	IGR-opense			
cg0888183	0.241532	0.587452	15.97296	3.95E-07	0.000349	7.288801	0.466685	0.708218	-0.24153	12	5348711	R	II	LOC10192	Body	opensea	Body-opens		rs3713237	32
cg1842961	0.242191	0.723646	15.97135	3.95E-07	0.000349	7.288114	0.602551	0.844741	-0.24219	3	1.44E+08	R	II		IGR	opensea	IGR-opense		rs5587367	37;21;17;16;2
cg1653226	0.217158	0.502305	15.96644	3.96E-07	0.000349	7.286022	0.393726	0.610885	-0.21716	12	53190908	R	II	KRT3	TSS1500	opensea	TSS1500-op		rs5690568	26;19;1
cg0300461	0.255434	0.514062	15.95727	3.98E-07	0.000349	7.282105	0.386345	0.641779	-0.25543	2	2.32E+08	R	II	ARMC9	Body	opensea	Body-opens		rs1855918	47;1
cg0049152	0.241296	0.54548	15.95681	3.98E-07	0.000349	7.281906	0.424832	0.666128	-0.2413	16	6913336	F	II	A2BP1	5'UTR	opensea	5'UTR-oper		rs1501042	8
cg0155761	0.225538	0.458018	15.95146	3.99E-07	0.000349	7.279621	0.345249	0.570787	-0.22554	9	1.37E+08	F	II		IGR	opensea	IGR-opense		rs5768841	0;8;20
cg1157708	0.20793	0.468398	15.94115	4.01E-07	0.000349	7.275215	0.364433	0.572363	-0.20793	2	38264725	F	II	RMDN2-AS	TSS1500	opensea	TSS1500-op		rs5401092	1;49
cg2607167	0.204934	0.488805	15.93638	4.02E-07	0.000349	7.273171	0.386338	0.591273	-0.20493	18	61139582	F	II		IGR	opensea	IGR-opense			
cg0248832	0.209498	0.515501	15.9329	4.03E-07	0.000349	7.271683	0.410753	0.62025	-0.2095	19	5841442	R	II		IGR	shelf	IGR-shelf	chr19:584		
cg1434907	0.21479	0.498515	15.9327	4.03E-07	0.000349	7.271598	0.39112	0.605911	-0.21479	9	78547359	F	II	PCSK5	Body	opensea	Body-opens		rs2017256	29
cg1476064	0.201611	0.512401	15.90405	4.08E-07	0.00035	7.25932	0.411595	0.613207	-0.20161	6	10965215	F	II	SYCP2L	3'UTR	opensea	3'UTR-oper		rs1120628	31;51
cg1919237	0.206348	0.691709	15.90045	4.09E-07	0.00035	7.257776	0.588535	0.794883	-0.20635	11	62209356	F	II	AHNAK	Body	shelf	Body-shelf	chr11:622		
cg0078979	0.243832	0.487206	15.8903	4.11E-07	0.00035	7.253418	0.36529	0.609122	-0.24383	2	1.79E+08	F	II	MIR548N	TSS1500	opensea	TSS1500-op		rs1447690	1;20
cg0190324	0.22761	0.461036	15.88043	4.13E-07	0.00035	7.249176	0.347231	0.574841	-0.22761	4	87373203	F	II	MAPK10	5'UTR	opensea	5'UTR-oper		rs5750245	1;33
cg1125825	0.238685	0.479766	15.88036	4.13E-07	0.00035	7.249145	0.360423	0.599108	-0.23869	9	1.41E+08	F	II	ARRDC1-AS	TSS1500	shore	TSS1500-sh	chr9:1405	rs1434819	0
cg1105491	0.209866	0.518845	15.87697	4.13E-07	0.00035	7.247689	0.413912	0.623778	-0.20987	6	1.12E+08	F	II	FYN	5'UTR	opensea	5'UTR-oper		rs5584912	10;16;31
cg1817949	0.230513	0.408627	15.84588	4.19E-07	0.000352	7.234301	0.293371	0.523884	-0.23051	4	1.48E+08	F	II		IGR	opensea	IGR-opense		rs1408676	22
cg1467521	0.235523	0.57777	15.83942	4.21E-07	0.000352	7.231515	0.460009	0.695531	-0.23552	5	98149610	F	II		IGR	opensea	IGR-opense		rs5285456	7;18
cg2293716	0.22325	0.524713	15.83093	4.22E-07	0.000353	7.227854	0.413088	0.636338	-0.22325	1	2.14E+08	F	II		IGR	opensea	IGR-opense		rs5441873	0;27;45
cg1038913	0.200876	0.613823	15.82794	4.23E-07	0.000353	7.226561	0.513385	0.714261	-0.20088	2	85998068	F	II	ATOH8	Body	opensea	Body-opens		rs5635670	32;35
cg1283102	0.24323	0.498637	15.82292	4.24E-07	0.000353	7.224395	0.377022	0.620252	-0.24323	1	1.61E+08	R	II		IGR	opensea	IGR-opense		rs5602811	1
cg0998808	0.232058	0.502799	15.81984	4.24E-07	0.000353	7.223064	0.38677	0.618828	-0.23206	2	1.3E+08	F	II		IGR	opensea	IGR-opense		rs5283931	31
cg0608900	0.274719	0.534403	15.81242	4.26E-07	0.000353	7.219856	0.397043	0.671762	-0.27472	15	50427638	F	II		IGR	opensea	IGR-opense		rs1749960	8;39
cg0258278	0.262265	0.560271	15.80184	4.28E-07	0.000353	7.21528	0.429139	0.691403	-0.26226	9	78291792	F	II	MIR548H3	Body	opensea	Body-opens		rs5726178	0;13;51
cg0186289	0.212446	0.485697	15.79924	4.29E-07	0.000353	7.214157	0.379474	0.59192	-0.21245	14	70548168	F	II	SLC8A3	Body	opensea	Body-opens			
cg1728094	0.243626	0.486066	15.79543	4.29E-07	0.000353	7.212505	0.364253	0.607879	-0.24363	5	96839154	R	II	LINC01340	TSS1500	opensea	TSS1500-op		rs3537739	8
cg0973045	0.204152	0.599323	15.79093	4.3E-07	0.000353	7.210557	0.497247	0.701399	-0.20415	3	1.07E+08	F	II	BBX	5'UTR	opensea	5'UTR-oper		rs5727485	2;33
cg1161566	0.222157	0.586083	15.78408	4.32E-07	0.000353	7.207591	0.475005	0.697162	-0.22216	3	1.13E+08	R	II	NAA50	Body	shelf	Body-shelf	chr3:1134		
cg1122813	0.229396	0.617326	15.78166	4.32E-07	0.000353	7.206541	0.502628	0.732024	-0.2294	2	63971489	R	II		IGR	opensea	IGR-opense		rs5687038	12;8
cg0247252	0.247773	0.687561	15.77691	4.33E-07	0.000353	7.204486	0.563675	0.811448	-0.24777	1	1E+08	F	II	PALMD	Body	opensea	Body-opens		rs1162021	47
cg0751507	0.216216	0.665922	15.76274	4.36E-07	0.000353	7.198336	0.557814	0.77403	-0.21622	12	42737622	R	II	PPHLN1	Body	opensea	Body-opens		rs1496070	28;12
cg1590626	0.268501	0.428351	15.76239	4.36E-07	0.000353	7.198184	0.294101	0.562602	-0.2685	3	1.87E+08	F	II	SNORA4	TSS200	shelf	TSS200-she	chr3:1865	rs2002600	0;1;11;46
cg1490857	0.223513	0.447007	15.75363	4.38E-07	0.000353	7.194383	0.33525	0.558764	-0.22351	19	45880430	R	II		IGR	opensea	IGR-opense		rs1858454	46;22
cg2057873	0.203929	0.57853	15.75033	4.39E-07	0.000353	7.192947	0.476566	0.680495	-0.20393	2	1.79E+08	F	II	OSBPL6	Body	opensea	Body-opens		rs5350668	0;23;27;34
cg0214572	0.214078	0.480739	15.74935	4.39E-07	0.000353	7.19252	0.3737	0.587778	-0.21408	4	1.14E+08	F	II	ANK2	Body	opensea	Body-opens		rs5506857	0;18
cg2202977	0.209443	0.593651	15.74849	4.39E-07	0.000353	7.192148	0.48893	0.698373	-0.20944	2	95534722	F	II		IGR	shelf	IGR-shelf	chr2:9553	rs6214693	5;24;39;41;43;47
cg2321895	0.2473	0.543996	15.74488	4.4E-07	0.000353	7.190578	0.420346	0.667645	-0.2473	14	59109856	R	II	DACT1	Body	shelf	Body-shelf	chr14:591	rs5782027	38;26;19
cg0232860	0.217773	0.477762	15.74341	4.4E-07	0.000353	7.189942	0.368876	0.586649	-0.21777	11	1.3E+08	R	II		IGR	shelf	IGR-shelf	chr11:129		
cg1225692	0.21342	0.563384	15.74214	4.4E-07	0.000353	7.189389	0.456673	0.670094	-0.21342	7	50310464	F	II		IGR	opensea	IGR-opense		rs1153596	0;12;46
cg0940036	0.24945	0.49466	15.73706	4.41E-07	0.000353	7.18718	0.369935	0.619385	-0.24945	2	98579411	F	II	TMEM131	Body	opensea	Body-opens		rs1389572	30
cg2391696	0.200556	0.527969	15.72771	4.43E-07	0.000353	7.183109	0.42769	0.628247	-0.20056	10	1.14E+08	F	II		IGR	opensea	IGR-opense		rs5541079	42;48
cg2131079	0.228703	0.477204	15.72411	4.44E-07	0.000353	7.181542	0.362852	0.591555	-0.2287	9	22210418	R	II		IGR	opensea	IGR-opense		rs5472777	25
cg2363014	0.201639	0.573216	15.7217	4.44E-07	0.000353	7.180495	0.472396	0.674035	-0.20164	9	36144545	R	II	GLIPR2	Body	opensea	Body-opens		rs1863759	13
cg1040863	0.233064	0.675447	15.70932	4.47E-07	0.000353	7.175099	0.558915	0.791979	-0.23306	2	96869389	F	II	STARD7	Body	opensea	Body-opens		rs5636720	3;9;27;37;38
cg0386774	0.232781	0.636376	15.70079	4.49E-07	0.000353	7.171379	0.519986	0.752767	-0.23278	14	62130280	F	II		IGR	opensea	IGR-opense		rs5617465	0;2;8;41
cg2717729	0.216167	0.641721	15.6906	4.51E-07	0.000353	7.166933	0.533637	0.749804	-0.21617	3	1.9E+08	R	II	CLDN1	Body	opensea	Body-opens		rs1928739	34
cg2622746	0.20901	0.508418	15.68813	4.52E-07	0.000353	7.165851	0.403914	0.612923	-0.20901	12	68553577	F	II	IFNG	TSS200	opensea	TSS200-ope			
cg0859073	0.249392	0.61333	15.68793	4.52E-07	0.000353	7.165767	0.488634	0.738026	-0.24939	4	77624601	F	II	SHROOM3	Body	opensea	Body-opens			
cg1121067	0.212471	0.54888	15.68521	4.52E-07	0.000353	7.164579	0.442644	0.655115	-0.21247	1	1.13E+08	R	II		IGR	opensea	IGR-opense		rs5551855	17;1
cg0198282	0.216703	0.537034	15.675	4.54E-07	0.000353	7.160114	0.428683	0.645386	-0.2167	11	9599270	R	II	WEE1	Body	shelf	Body-shelf	chr11:959	rs3727483	37;19;18;7;3;2
cg1585641	0.2105	0.472856	15.6684	4.56E-07	0.000353	7.157227	0.367606	0.578106	-0.2105	21	39760881	R	II	ERG	Body	opensea	Body-opens			
cg0179424	0.253006	0.498363	15.66665	4.56E-07	0.000353	7.156462	0.37186	0.624866	-0.25301	8	1.46E+08	R	II	KIAA1688	TSS1500	opensea	TSS1500-op			
cg0390251	0.204684	0.59609	15.6433	4.61E-07	0.000355	7.146238	0.493748	0.698432	-0.20468	1	2.39E+08	R	II		IGR	opensea	IGR-opense			
cg2063687	0.229971	0.567614	15.64153	4.62E-07	0.000355	7.145461	0.452628	0.682599	-0.22997	17	59883616	R	II	BRIP1	Body	opensea	Body-opens		rs1126027	2
cg2267202	0.207003	0.480671	15.63398	4.63E-07	0.000355	7.142152	0.37717	0.584173	-0.207	10	14062217	F	II	FRMD4A	Body	opensea	Body-opens		rs5337082	10;15;31;37
cg1506316	0.202992	0.545842	15.622	4.66E-07	0.000356	7.136892	0.444346	0.647338	-0.20299	8	27483086	R	II		IGR	opensea	IGR-opense		rs1896705	49;31
cg0265051	0.248772	0.538345	15.62076	4.66E-07	0.000356	7.136348	0.413959	0.662731	-0.24877	17	27048975	R	II	RPL23A	Body	shore	Body-shore	chr17:270	rs7552537	50;19;1
cg1417807	0.232427	0.548401	15.61374	4.68E-07	0.000357	7.133265	0.432187	0.664614	-0.23243	9	78777801	R	II	PCSK5	Body	opensea	Body-opens		rs5459156	4;1
cg1389044	0.200874	0.428173	15.59236	4.73E-07	0.000359	7.123864	0.327736	0.52861	-0.20087	15	1.02E+08	F	II	PCSK6	Body	opensea	Body-opens		rs5448296	1
cg1351919	0.20353	0.512644	15.58014	4.75E-07	0.00036	7.118479	0.41088	0.614409	-0.20353	14	1.01E+08	R	II		IGR	opensea	IGR-opense		rs1490269	40;22;2;1
cg1275311	0.216877	0.662888	15.57689	4.76E-07	0.00036	7.117049	0.55445	0.771326	-0.21688	3	18996624	R	II		IGR	opensea	IGR-opense		rs1122092	15
cg1965599	0.229463	0.49062	15.57222	4.77E-07	0.00036	7.114992	0.375888	0.605352	-0.22946	2	2.07E+08	R	II		IGR	opensea	IGR-opense		rs1176338	40;29;3;1
cg1797447	0.242412	0.497783	15.56283	4.79E-07	0.00036	7.110849	0.376577	0.618989	-0.24241	2	9490525	F	II	ASAP2	Body	opensea	Body-opens		rs1509861	1;21;26;47
cg2262959	0.267738	0.459411	15.55627	4.81E-07	0.00036	7.107951	0.325542	0.59328	-0.26774	6	16837449	R	II		IGR	opensea	IGR-opense		rs5735430	46;14;10
cg2176548	0.225766	0.529372	15.55379	4.81E-07	0.00036	7.10686	0.416489	0.642255	-0.22577	13	27195650	F	II	WASF3	5'UTR	opensea	5'UTR-oper		rs1919249	1;42;46
cg0140234	0.205244	0.604099	15.54887	4.82E-07	0.00036	7.104687	0.501477	0.706721	-0.20524	4	96472269	F	II		IGR	shore	IGR-shore	chr4:9646	rs1387741	0;1;46
cg0122367	0.216065	0.555405	15.54451	4.83E-07	0.00036	7.10276	0.447373	0.663438	-0.21606	11	15133828	F	II	INSC	TSS200	shelf	TSS200-she	chr11:151	rs5539107	10
cg2645366	0.239656	0.543454	15.54146	4.84E-07	0.00036	7.101412	0.423626	0.663282	-0.23966	10	90411391	R	II		IGR	opensea	IGR-opense		rs1384306	2
cg1537615	0.222472	0.454086	15.53749	4.85E-07	0.00036	7.099657	0.34285	0.565322	-0.22247	10	1.19E+08	R	II		IGR	shelf	IGR-shelf	chr10:118	rs1428123	2
cg1217751	0.230205	0.544511	15.53719	4.85E-07	0.00036	7.099523	0.429408	0.659613	-0.23021	6	1.11E+08	R	II		IGR	opensea	IGR-opense		rs5571034	9;37;49
cg1767661	0.206427	0.558435	15.53229	4.86E-07	0.00036	7.097355	0.455221	0.661648	-0.20643	10	80563421	F	II		IGR	opensea	IGR-opense		rs3724014	0;1;3;22
cg2679531	0.215946	0.469225	15.52886	4.87E-07	0.00036	7.095839	0.361252	0.577198	-0.21595	8	1.46E+08	F	II	C8orf77	Body	shelf	Body-shelf	chr8:1462	rs5696390	7;34
cg1085327	0.241611	0.613271	15.52813	4.87E-07	0.00036	7.095514	0.492465	0.734077	-0.24161	8	86436099	R	II		IGR	opensea	IGR-opense		rs1918059	29
cg1874069	0.223632	0.612458	15.52086	4.89E-07	0.00036	7.092296	0.500642	0.724274	-0.22363	14	1.05E+08	F	II		IGR	opensea	IGR-opense		rs7631297	7;21;36;39
cg2250576	0.225214	0.536042	15.50503	4.93E-07	0.000361	7.085282	0.423435	0.648649	-0.22521	13	1.13E+08	F	II	LOC10192	ExonBnd	opensea	ExonBnd-op			
cg0047005	0.21851	0.561311	15.49254	4.96E-07	0.000362	7.079741	0.452056	0.670566	-0.21851	15	72206554	R	II	MYO9A	Body	opensea	Body-opens		rs5309274	39;20
cg0675449	0.251021	0.482591	15.49041	4.96E-07	0.000362	7.078795	0.35708	0.608101	-0.25102	10	52268375	R	II	SGMS1	5'UTR	opensea	5'UTR-oper		rs5400935	39;22
cg0047851	0.231934	0.51617	15.48878	4.96E-07	0.000362	7.078072	0.400203	0.632137	-0.23193	2	1.61E+08	R	II	ITGB6	Body	opensea	Body-opens		rs5569174	7;1
cg0460926	0.236792	0.478233	15.48389	4.98E-07	0.000362	7.075904	0.359837	0.596629	-0.23679	10	81045085	R	II	ZMIZ1	Body	opensea	Body-opens			
cg0259672	0.221483	0.515218	15.46689	5.02E-07	0.000363	7.068345	0.404476	0.62596	-0.22148	11	76311399	R	II		IGR	opensea	IGR-opense		rs5577891	45
cg0791011	0.245048	0.423051	15.46557	5.02E-07	0.000363	7.06776	0.300527	0.545575	-0.24505	10	61062665	F	II	FAM13C	Body	opensea	Body-opens			
cg0194845	0.230885	0.566452	15.45837	5.04E-07	0.000363	7.064555	0.451009	0.681894	-0.23089	5	17253941	F	II	BASP1	5'UTR	opensea	5'UTR-oper			
cg2377253	0.249877	0.473005	15.45789	5.04E-07	0.000363	7.064342	0.348066	0.597943	-0.24988	16	89628650	R	II	RPL13	Body	shore	Body-shore	chr16:896	rs3751690	31;25;23;2
cg2147122	0.227317	0.615684	15.45546	5.04E-07	0.000363	7.063262	0.502026	0.729343	-0.22732	6	1.37E+08	R	II	MAP7	TSS200	opensea	TSS200-ope			
cg2339701	0.236761	0.486712	15.45347	5.05E-07	0.000363	7.062377	0.368331	0.605092	-0.23676	10	10571132	F	II		IGR	opensea	IGR-opense			
cg1082906	0.203667	0.451469	15.45038	5.06E-07	0.000363	7.060997	0.349636	0.553303	-0.20367	11	59069567	F	II		IGR	opensea	IGR-opense		rs5312620	0;1;20;42
cg2266956	0.231612	0.587587	15.44675	5.07E-07	0.000363	7.059382	0.471781	0.703393	-0.23161	7	93114291	F	II	MIR489	TSS1500	opensea	TSS1500-op		rs1883434	40;42
cg0227242	0.216816	0.483817	15.44175	5.08E-07	0.000363	7.057155	0.375409	0.592226	-0.21682	15	25756044	F	II		IGR	opensea	IGR-opense		rs5376673	36
cg0366935	0.208312	0.492021	15.42981	5.11E-07	0.000364	7.05183	0.387865	0.596177	-0.20831	3	71912936	F	II		IGR	opensea	IGR-opense		rs1447330	0;46;50
cg0443178	0.222667	0.469769	15.42187	5.13E-07	0.000364	7.048287	0.358435	0.581103	-0.22267	3	1.32E+08	F	II	CCRL1	TSS1500	opensea	TSS1500-op		rs5567934	0;44
cg1343743	0.226925	0.559856	15.40638	5.16E-07	0.000365	7.041367	0.446393	0.673319	-0.22693	3	66354186	R	II	SLC25A26	Body	opensea	Body-opens		rs6224687	51;38;4
cg0521480	0.228177	0.575865	15.39188	5.2E-07	0.000365	7.034882	0.461776	0.689953	-0.22818	12	2247203	R	II	CACNA1C	Body	opensea	Body-opens		rs5574706	28;26;24;23;1
cg1246415	0.225407	0.521283	15.38671	5.21E-07	0.000365	7.032569	0.40858	0.633987	-0.22541	3	70588826	F	II		IGR	opensea	IGR-opense		rs1864057	0
cg0060497	0.211387	0.416894	15.38299	5.22E-07	0.000365	7.030904	0.311201	0.522588	-0.21139	9	1.3E+08	F	II		IGR	shore	IGR-shore	chr9:1303	rs5324891	0
cg1562517	0.204676	0.448432	15.37172	5.25E-07	0.000365	7.025852	0.346094	0.55077	-0.20468	14	42167742	F	II	LRFN5	5'UTR	opensea	5'UTR-oper		rs1423565	30;50
cg2679460	0.244637	0.516972	15.36471	5.27E-07	0.000365	7.02271	0.394654	0.63929	-0.24464	10	89942354	R	II		IGR	opensea	IGR-opense		rs5509443	28

cg1208191	0.224489	0.668639	15.35908	5.28E-07	0.000365	7.020184	0.556394	0.780883	-0.22449	5	9370475	F	II	SEMA5A	Body	opensea	Body-opens		rs5715602	1;9;43;48
cg2713591	0.206719	0.515665	15.3573	5.29E-07	0.000365	7.019382	0.412306	0.619025	-0.20672	10	1.35E+08	R	II	SYCE1	Body	opensea	Body-opens		rs1085775	36;14
cg0882674	0.245058	0.538857	15.35647	5.29E-07	0.000365	7.01901	0.416328	0.661385	-0.24506	8	1.05E+08	F	II	DPYS	3'UTR	opensea	3'UTR-oper		rs5701781	22
cg1289958	0.236164	0.511723	15.35351	5.3E-07	0.000365	7.017684	0.393641	0.629805	-0.23616	3	19499616	F	II	KCNH8	Body	opensea	Body-opens		rs5488551	0;14
cg0147949	0.214381	0.570793	15.33643	5.34E-07	0.000365	7.010009	0.463603	0.677983	-0.21438	3	1.88E+08	F	II	LPP	5'UTR	opensea	5'UTR-oper		rs1836850	25
cg2219904	0.224259	0.551625	15.32681	5.37E-07	0.000365	7.005683	0.439495	0.663754	-0.22426	1	83346298	R	II		IGR	opensea	IGR-opense			
cg1828745	0.221953	0.463865	15.31097	5.41E-07	0.000365	6.998552	0.352889	0.574842	-0.22195	6	1.22E+08	R	II		IGR	opensea	IGR-opense		rs5434158	19;12;2
cg1180086	0.253645	0.605659	15.30879	5.41E-07	0.000365	6.997568	0.478836	0.732481	-0.25364	10	64981888	R	II	JMJD1C	Body	opensea	Body-opens		rs5571536	4
cg1477429	0.253302	0.537726	15.30347	5.43E-07	0.000365	6.995168	0.411075	0.664377	-0.2533	8	1.42E+08	F	II	PTK2	5'UTR	opensea	5'UTR-oper		rs3722781	1
cg0111738	0.203127	0.411777	15.30324	5.43E-07	0.000365	6.995068	0.310214	0.51334	-0.20313	20	56274096	F	II	PMEPA1	5'UTR	opensea	5'UTR-oper			
cg1216261	0.255486	0.519151	15.28944	5.46E-07	0.000366	6.988843	0.391408	0.646894	-0.25549	6	18147472	F	II	TPMT	Body	opensea	Body-opens			
cg2425121	0.203426	0.466265	15.2804	5.49E-07	0.000367	6.984761	0.364552	0.567978	-0.20343	11	10347429	F	II		IGR	opensea	IGR-opense			
cg1310549	0.235817	0.515185	15.27263	5.51E-07	0.000367	6.981248	0.397276	0.633094	-0.23582	11	73600155	R	II	PAAF1	5'UTR	opensea	5'UTR-oper		rs5586483	19
cg0684803	0.222665	0.53249	15.26932	5.52E-07	0.000367	6.979751	0.421157	0.643822	-0.22267	9	1.09E+08	F	II		IGR	opensea	IGR-opense		rs5345987	0;2;12;42
cg2589203	0.228071	0.532379	15.26089	5.54E-07	0.000368	6.975939	0.418343	0.646414	-0.22807	6	86395559	R	II		IGR	opensea	IGR-opense		rs5309111	21;17;4
cg0918599	0.214856	0.56482	15.25242	5.56E-07	0.000368	6.972107	0.457392	0.672248	-0.21486	12	22090231	R	II	ABCC9	TSS1500	shelf	TSS1500-sh	chr12:2209	rs7509213	30;17
cg1809126	0.207218	0.359533	15.24482	5.58E-07	0.000368	6.968666	0.255923	0.463142	-0.20722	15	66795595	R	II	SNORD18A	Body	opensea	Body-opens		rs1128650	46;40;12;1
cg1085960	0.255167	0.495585	15.24396	5.59E-07	0.000368	6.968274	0.368001	0.623168	-0.25517	8	30255368	F	II	RBPMS	Body	opensea	Body-opens		rs1119144	0;1;8;47
cg1600838	0.231196	0.543774	15.24229	5.59E-07	0.000368	6.967517	0.428176	0.659372	-0.2312	4	80224872	F	II	LINC01088	Body	opensea	Body-opens		rs5673475	8
cg0682294	0.22776	0.587175	15.23453	5.61E-07	0.000368	6.963998	0.473295	0.701055	-0.22776	3	1.51E+08	R	II	CLRN1	TSS200	opensea	TSS200-ope		rs5422689	32;28;13;4;2
cg0054467	0.211672	0.612148	15.2344	5.61E-07	0.000368	6.963941	0.506313	0.717984	-0.21167	11	39689583	F	II		IGR	opensea	IGR-opense		rs5388797	0;8;10;44
cg0326910	0.218784	0.534822	15.23293	5.62E-07	0.000368	6.963274	0.42543	0.644214	-0.21878	4	98315750	R	II	STPG2-AS1	Body	opensea	Body-opens			
cg0841521	0.234025	0.50623	15.23259	5.62E-07	0.000368	6.963119	0.389218	0.623242	-0.23402	6	19906065	R	II		IGR	opensea	IGR-opense			
cg0199592	0.219922	0.562749	15.23099	5.62E-07	0.000368	6.962395	0.452788	0.67271	-0.21992	1	24257839	F	II		IGR	opensea	IGR-opense		rs1121014	40
cg0237066	0.212078	0.664611	15.22096	5.65E-07	0.000368	6.957843	0.558572	0.77065	-0.21208	6	44934650	R	II	SUPT3H	Body	opensea	Body-opens		rs5416580	44;31;2
cg0300437	0.247586	0.570301	15.21299	5.67E-07	0.000368	6.954226	0.446508	0.694094	-0.24759	3	1.15E+08	R	II		IGR	opensea	IGR-opense		rs1144886	42;1
cg1904535	0.20505	0.468589	15.2102	5.68E-07	0.000368	6.952957	0.366064	0.571114	-0.20505	9	37954074	F	II	SHB	Body	opensea	Body-opens		rs1443766	0;32
cg0686718	0.203753	0.571205	15.20317	5.7E-07	0.000368	6.949762	0.469328	0.673081	-0.20375	12	1.14E+08	F	II	DTX1	Body	opensea	Body-opens		rs1917642	43
cg2365209	0.208407	0.532967	15.20189	5.7E-07	0.000368	6.949179	0.428764	0.637171	-0.20841	11	75347560	R	II	MAP6	Body	opensea	Body-opens			
cg1851615	0.215614	0.534527	15.20164	5.7E-07	0.000368	6.949066	0.42672	0.642335	-0.21561	3	1.49E+08	R	II	CPA3	3'UTR	opensea	3'UTR-oper		rs1837888	35;30
cg0681500	0.222142	0.638068	15.1923	5.73E-07	0.000368	6.944818	0.526997	0.749139	-0.22214	3	1.44E+08	R	II		IGR	opensea	IGR-opense		rs5768179	28
cg0460306	0.249116	0.539479	15.19197	5.73E-07	0.000368	6.944668	0.414921	0.664037	-0.24912	18	42290070	R	II	SETBP1	Body	opensea	Body-opens		rs5655318	10;9
cg0335865	0.257779	0.481465	15.19031	5.73E-07	0.000368	6.943914	0.352575	0.610354	-0.25778	2	1.69E+08	F	II	LOC10561(	Body	opensea	Body-opens		rs1132007	18
cg0558231	0.239447	0.509888	15.19004	5.73E-07	0.000368	6.94379	0.390165	0.629612	-0.23945	4	5760808	F	II	EVC	Body	opensea	Body-opens		rs5682419	0;31
cg1301792	0.235178	0.553788	15.18081	5.76E-07	0.000369	6.939589	0.436198	0.671377	-0.23518	7	1.17E+08	F	II		IGR	opensea	IGR-opense		rs7965377	9;19
cg1031553	0.223132	0.57145	15.17961	5.76E-07	0.000369	6.939042	0.459884	0.683016	-0.22313	13	43579592	F	II		IGR	opensea	IGR-opense			
cg1904310	0.210846	0.635173	15.16847	5.79E-07	0.00037	6.933965	0.52975	0.740596	-0.21085	6	1.17E+08	R	II	RFX6	Body	opensea	Body-opens		rs1900579	38
cg2452746	0.212381	0.607898	15.15909	5.82E-07	0.00037	6.929686	0.501708	0.714089	-0.21238	13	37244827	F	II		IGR	shelf	IGR-shelf	chr13:3724	rs5672775	38
cg0533997	0.200191	0.601786	15.15224	5.84E-07	0.000371	6.926561	0.501691	0.701882	-0.20019	2	64964564	F	II		IGR	opensea	IGR-opense		rs3728771	40
cg0948125	0.225225	0.560592	15.15186	5.84E-07	0.000371	6.926387	0.44798	0.673204	-0.22522	13	22260911	F	II	FGF9	Body	opensea	Body-opens		rs829219	32
cg0914635	0.235158	0.412628	15.14658	5.86E-07	0.000371	6.923976	0.295049	0.530207	-0.23516	11	8795619	F	II	LOC10272	Body	opensea	Body-opens			
cg1332255	0.20039	0.448089	15.14404	5.86E-07	0.000371	6.922815	0.347894	0.548284	-0.20039	11	74173518	R	II	KCNE3	5'UTR	opensea	5'UTR-oper		rs5553526	5
cg1601581	0.215611	0.648477	15.13464	5.89E-07	0.000371	6.918521	0.540672	0.756283	-0.21561	1	2.1E+08	F	II		IGR	opensea	IGR-opense		rs1509832	14
cg1232570	0.241853	0.502871	15.1311	5.9E-07	0.000371	6.916899	0.381945	0.623798	-0.24185	7	48338551	F	II	ABCA13	Body	opensea	Body-opens		rs1907537	0
cg0110216	0.208514	0.377943	15.13087	5.9E-07	0.000371	6.916796	0.273686	0.4822	-0.20851	5	1.04E+08	R	II		IGR	opensea	IGR-opense		rs5979629	22;16
cg0604837	0.234397	0.512226	15.12842	5.91E-07	0.000371	6.915673	0.395027	0.629424	-0.2344	13	74994693	F	II	LINC00381	Body	opensea	Body-opens		rs1810543	1;38
cg1099417	0.22623	0.525338	15.12734	5.91E-07	0.000371	6.915181	0.412223	0.638453	-0.22623	7	80412899	R	II	SEMA3C	Body	opensea	Body-opens			
cg2686382	0.210041	0.496997	15.12406	5.92E-07	0.000371	6.913677	0.391977	0.602018	-0.21004	16	3161760	F	II	ZNF205	TSS1500	opensea	TSS1500-op		rs1825385	10
cg2195220	0.221138	0.546079	15.1229	5.92E-07	0.000371	6.913149	0.43551	0.656649	-0.22114	2	2.31E+08	F	II	SP110	TSS1500	opensea	TSS1500-op			
cg0141193	0.20922	0.478591	15.11775	5.94E-07	0.000372	6.910791	0.373981	0.583201	-0.20922	5	1.51E+08	R	II	GM2A	Body	opensea	Body-opens		rs1911263	41;16
cg1719987	0.216129	0.610774	15.09585	6E-07	0.000374	6.900753	0.50271	0.718838	-0.21613	9	92505134	F	II		IGR	opensea	IGR-opense		rs7916587	18;30;41
cg1359920	0.204674	0.536938	15.08012	6.05E-07	0.000374	6.893533	0.434601	0.639275	-0.20467	14	1.01E+08	F	II		IGR	opensea	IGR-opense		rs5631399	21;40
cg1957438	0.288791	0.456478	15.07979	6.05E-07	0.000374	6.893384	0.312082	0.600873	-0.28879	4	10075174	R	II		IGR	opensea	IGR-opense		rs1926784	1
cg2164309	0.205592	0.510529	15.07974	6.05E-07	0.000374	6.893362	0.407733	0.613325	-0.20559	11	1.05E+08	F	II	CARD18	3'UTR	opensea	3'UTR-oper		rs1473012	4;9;45;50
cg0298340	0.212471	0.511477	15.0703	6.08E-07	0.000374	6.889021	0.405242	0.617713	-0.21247	11	46296193	R	II		IGR	shelf	IGR-shelf	chr11:4629	rs5307223	49;3;2
cg2565552	0.217844	0.602692	15.05745	6.12E-07	0.000375	6.883113	0.49377	0.711614	-0.21784	13	73942508	R	II		IGR	opensea	IGR-opense		rs1907202	50
cg2363875	0.209262	0.488808	15.05178	6.14E-07	0.000375	6.880501	0.384178	0.593439	-0.20926	5	1.48E+08	F	II		IGR	opensea	IGR-opense		rs5460078	28;45
cg1530606	0.223675	0.527944	15.05143	6.14E-07	0.000375	6.880343	0.416107	0.639782	-0.22367	22	39712956	F	II	RPL3	Body	shelf	Body-shelf	chr22:3971	rs3701033	0;25;34
cg0232395	0.23347	0.539823	15.0513	6.14E-07	0.000375	6.880282	0.423088	0.656558	-0.23347	8	1.31E+08	R	II	FAM49B	5'UTR	opensea	5'UTR-oper		rs7455088	2;1
cg0537218	0.205461	0.486677	15.04708	6.15E-07	0.000375	6.878338	0.383946	0.589407	-0.20546	3	1.25E+08	R	II		IGR	opensea	IGR-opense		rs5382653	21
cg0685009	0.204833	0.693982	15.0398	6.17E-07	0.000376	6.874983	0.591565	0.796398	-0.20483	1	1.83E+08	R	II	LAMC1	Body	opensea	Body-opens		rs5538018	38;33
cg1331835	0.238448	0.568465	15.0397	6.17E-07	0.000376	6.874938	0.449242	0.687689	-0.23845	7	6072034	R	II	ANKRD61	Body	opensea	Body-opens		rs5703818	45;6
cg2377773	0.218755	0.692503	15.03273	6.19E-07	0.000376	6.871726	0.583125	0.80188	-0.21875	1	1.61E+08	R	II	ITLN1	TSS1500	opensea	TSS1500-op		rs7948293	49;45;21
cg1237640	0.227927	0.469551	15.02769	6.21E-07	0.000376	6.869398	0.355587	0.583514	-0.22793	14	21236306	R	II	EDDM3B	TSS1500	opensea	TSS1500-op		rs1144170	33;19;1
cg1951555	0.223638	0.600734	15.02596	6.21E-07	0.000376	6.868599	0.488915	0.712553	-0.22364	8	1.1E+08	R	II		IGR	opensea	IGR-opense		rs1420475	2
cg2438227	0.214597	0.497888	15.0237	6.22E-07	0.000376	6.867558	0.390589	0.605186	-0.2146	15	82204026	R	II		IGR	opensea	IGR-opense		rs5419843	9
cg1918994	0.213382	0.691495	15.02144	6.23E-07	0.000376	6.866515	0.584804	0.798186	-0.21338	10	4512423	R	II		IGR	opensea	IGR-opense		rs5745815	31
cg1628445	0.213045	0.613835	15.0122	6.26E-07	0.000376	6.86225	0.507312	0.720357	-0.21305	1	1.65E+08	R	II	PBX1	Body	opensea	Body-opens		rs1386340	1
cg0977282	0.210098	0.647059	15.00555	6.28E-07	0.000377	6.859175	0.54201	0.752108	-0.2101	2	1.97E+08	R	II	HECW2	5'UTR	opensea	5'UTR-oper			
cg1665534	0.20845	0.444917	14.99855	6.3E-07	0.000377	6.85594	0.340693	0.549142	-0.20845	1	2.12E+08	R	II	RD3	5'UTR	opensea	5'UTR-oper		rs5583865	47;22;21
cg1735944	0.21889	0.567424	14.98101	6.35E-07	0.000379	6.847821	0.457979	0.676869	-0.21889	5	1.38E+08	F	II		IGR	opensea	IGR-opense		rs5589379	48
cg0464637	0.214581	0.478586	14.97578	6.37E-07	0.000379	6.845399	0.371295	0.585876	-0.21458	2	2.09E+08	F	II		IGR	opensea	IGR-opense			
cg0075782	0.274163	0.545957	14.9704	6.39E-07	0.000379	6.842907	0.408876	0.683039	-0.27416	3	1.55E+08	R	II	PLCH1	Body	opensea	Body-opens		rs5756424	33
cg1278806	0.200164	0.478041	14.96426	6.41E-07	0.000379	6.840061	0.377959	0.578123	-0.20016	20	10244011	F	II	SNAP25	5'UTR	opensea	5'UTR-oper			
cg0438103	0.215771	0.499976	14.96374	6.41E-07	0.000379	6.839818	0.39209	0.607861	-0.21577	11	1.18E+08	R	II	DSCAML1	Body	opensea	Body-opens		rs1844038	14;7
cg1614517	0.208804	0.460419	14.95823	6.42E-07	0.000379	6.837264	0.356017	0.564821	-0.2088	21	25596030	F	II		IGR	opensea	IGR-opense		rs1500489	0;9;28;39
cg2336111	0.200487	0.601384	14.95569	6.43E-07	0.000379	6.836085	0.501141	0.701628	-0.20049	7	1.57E+08	F	II	PTPRN2	Body	shelf	Body-shelf	chr7:1573(	rs1460666	12;40;49
cg1132119	0.223969	0.420679	14.95495	6.43E-07	0.000379	6.835741	0.308694	0.532664	-0.22397	3	37239890	F	II		IGR	opensea	IGR-opense		rs5445856	4;44
cg1873661	0.219892	0.564589	14.95477	6.44E-07	0.000379	6.835658	0.454643	0.674535	-0.21989	5	1.49E+08	F	II	PPARGC1B	Body	opensea	Body-opens		rs1138742	33;38
cg1323688	0.205551	0.488882	14.95009	6.45E-07	0.00038	6.833482	0.386106	0.591657	-0.20555	5	1.7E+08	F	II		IGR	opensea	IGR-opense		rs5377582	29
cg1022598	0.21482	0.660134	14.94278	6.47E-07	0.00038	6.83009	0.552724	0.767544	-0.21482	11	64443279	F	II	NRXN2	Body	opensea	Body-opens		rs5659344	3;4;5
cg2038891	0.202569	0.5246	14.94044	6.48E-07	0.00038	6.829003	0.423316	0.625885	-0.20257	3	1.34E+08	F	II	SLCO2A1	Body	opensea	Body-opens		rs1449385	0
cg1932573	0.200694	0.645351	14.91985	6.55E-07	0.00038	6.819426	0.545003	0.745698	-0.20069	7	1.42E+08	R	II		IGR	opensea	IGR-opense			
cg2563648	0.254614	0.482313	14.9166	6.56E-07	0.00038	6.81791	0.355006	0.609621	-0.25461	6	33241410	F	II	RPS18	Body	shore	Body-shore	chr6:3323	rs5345851	0;10
cg1585747	0.245128	0.54877	14.90803	6.59E-07	0.00038	6.813918	0.426206	0.671334	-0.24513	7	1.05E+08	R	II	SRPK2	Body	shelf	Body-shelf	chr7:1049	rs5709144	26;25;24
cg0186802	0.223759	0.52745	14.90642	6.59E-07	0.00038	6.813167	0.41557	0.639329	-0.22376	11	8798692	R	II	LOC10272	Body	opensea	Body-opens		rs1379531	38;2
cg1885677	0.236887	0.515603	14.89607	6.63E-07	0.00038	6.808341	0.39716	0.634046	-0.23689	3	15408790	R	II		IGR	opensea	IGR-opense		rs1496311	1
cg0494560	0.212536	0.55383	14.89437	6.63E-07	0.00038	6.807548	0.447562	0.660098	-0.21254	20	43118723	F	II	TTPAL	3'UTR	opensea	3'UTR-oper		rs5670893	44
cg2225231	0.244788	0.548919	14.88861	6.65E-07	0.00038	6.80486	0.426524	0.671313	-0.24479	9	1.21E+08	R	II		IGR	opensea	IGR-opense		rs5401597	35;22
cg1691705	0.236538	0.548913	14.8871	6.65E-07	0.00038	6.804159	0.430644	0.667182	-0.23654	2	59690787	R	II		IGR	opensea	IGR-opense		rs5651533	43;29
cg0064322	0.248067	0.550051	14.87987	6.68E-07	0.00038	6.80078	0.426017	0.674085	-0.24807	5	88866084	F	II		IGR	opensea	IGR-opense		rs7491094	41
cg2689427	0.205992	0.653673	14.87574	6.69E-07	0.00038	6.798853	0.550677	0.756669	-0.20599	13	21016241	F	II	CRYL1	Body	opensea	Body-opens		rs5458937	0;20;36;37;44
cg0306510	0.20183	0.422597	14.87069	6.71E-07	0.00038	6.79649	0.321682	0.523512	-0.20183	2	31497155	F	II		IGR	opensea	IGR-opense		rs1498246	0;24
cg1136794	0.227842	0.590544	14.86321	6.73E-07	0.00038	6.792996	0.476623	0.704465	-0.22784	9	90254311	F	II	DAPK1	Body	opensea	Body-opens		rs5631473	20;32
cg2543697	0.235076	0.566751	14.86277	6.74E-07	0.00038	6.792787	0.449213	0.684289	-0.23508	9	27461655	R	II	MOB3B	5'UTR	opensea	5'UTR-oper		rs1096794	14;5;1
cg2540629	0.210605	0.5524	14.86113	6.74E-07	0.00038	6.79202	0.447097	0.657702	-0.2106	14	61070289	R	II		IGR	opensea	IGR-opense		rs5654230	51;42;6
cg1211699	0.209393	0.529313	14.85549	6.76E-07	0.00038	6.789384	0.424617	0.63401	-0.20939	8	1.31E+08	F	II	GSDMC	Body	opensea	Body-opens			
cg1575870	0.216277	0.609088	14.85312	6.77E-07	0.00038	6.788272	0.50095	0.717227	-0.21628	12	25707667	R	II	IFLTD1	TSS1500	opensea	TSS1500-op		rs5559275	46;29;18
cg1778651	0.219166	0.547279	14.84984	6.78E-07	0.00038	6.786735	0.437696	0.656862	-0.21917	3	46410422	F	II	CCR5	TSS1500	opensea	TSS1500-op		rs5771138	11
cg0715542	0.243886	0.492413	14.84363	6.8E-07	0.00038	6.783828	0.37047	0.614356	-0.24389	1	12114402	R	II		IGR	opensea	IGR-opense			
cg0077998	0.223027	0.527842	14.84344	6.8E-07	0.00038	6.783738	0.416328	0.639355	-0.22303	22	39862809	F	II	MGAT3	5'UTR	opensea	5'UTR-oper		rs5719715	44
cg2700134	0.219383	0.516964	14.84126	6.81E-07	0.00038	6.782717	0.407272	0.626656	-0.21938	19	30855603	F	II		IGR	opensea	IGR-opense		rs5774801	0;11;22;24;48
cg2728896	0.229514	0.568123	14.83439	6.83E-07	0.00038	6.779496	0.453366	0.682879	-0.22951	21	34106765	R	II	C21orf66	3'UTR	opensea	3'UTR-oper			
cg0766389	0.213091	0.411965	14.83189	6.84E-07	0.00038	6.778323	0.30542	0.518511	-0.21309	4	1.22E+08	F	II	TNIP3	Body	opensea	Body-opens		rs5769055	1
cg0773984	0.213515	0.592765	14.82735	6.86E-07	0.00038	6.776194	0.486008	0.699523	-0.21351	6	1.6E+08	R	II	FNDC1	Body	shelf	Body-shelf	chr6:1596	rs5304354	29
cg1940579	0.22352	0.602341	14.82733	6.86E-07	0.00038	6.776184	0.490581	0.714101	-0.22352	2	1.62E+08	R	II		IGR	opensea	IGR-opense		rs1892078	32
cg0842105	0.235388	0.571678	14.82264	6.87E-07	0.00038	6.773986	0.453984	0.689372	-0.23539	3	1.49E+08	F	II	CPA3	Body	opensea	Body-opens		rs1150547	13;25
cg0549443	0.241619	0.528193	14.82239	6.87E-07	0.00038	6.773867	0.407384	0.649003	-0.24162	6	1.56E+08	R	II	NOX3	Body	opensea	Body-opens		rs5381981	41;9;2
cg2622453	0.226051	0.677593	14.82037	6.88E-07	0.00038	6.772919	0.564568	0.790619	-0.22605	3	1.56E+08	R	II	TIPARP	Body	opensea	Body-opens		rs1488327	9
cg0385766	0.217235	0.525339	14.82037	6.88E-07	0.00038	6.772918	0.416722	0.633957	-0.21723	5	1.72E+08	R	II	SH3PXD2B	Body	opensea	Body-opens		rs1852336	37;2
cg2346082	0.235282	0.363733	14.81803	6.89E-07	0.00038	6.771819	0.246092	0.481374	-0.23528	3	1.7E+08	R	II		IGR	opensea	IGR-opense		rs5580877	18;7;2
cg1555507	0.219849	0.634274	14.81196	6.91E-07	0.00038	6.768971	0.524349	0.744198	-0.21985	3	1.52E+08	R	II		IGR	opensea	IGR-opense		rs5471755	2
cg0813326	0.201165	0.555383	14.799	6.95E-07	0.000381	6.762878	0.4548	0.655965	-0.20116	17	78061119	F	II	CCDC40	Body	opensea	Body-opens		rs1115083	22;51
cg2273651	0.218728	0.578671	14.78539	7E-07	0.000381	6.756471	0.469307	0.688035	-0.21873	11	10089331	R	II	SBF2	Body	opensea	Body-opens		rs5755318	34;28;13
cg1399638	0.210835	0.488619	14.78435	7E-07	0.000381	6.755983	0.383201	0.594037	-0.21084	2	1.74E+08	R	II		IGR	opensea	IGR-opense		rs7640536	34;31;14;4;1
cg0418843	0.248537	0.517205	14.78035	7.02E-07	0.000381	6.754099	0.392936	0.641473	-0.24854	20	24500288	F	II	SYNDIG1	5'UTR	opensea	5'UTR-oper		rs5491396	1
cg0346062	0.216524	0.66995	14.78004	7.02E-07	0.000381	6.753955	0.561688	0.778212	-0.21652	11	1.2E+08	F	II	PVRL1	Body	opensea	Body-opens		rs5698581	0;1
cg1246900	0.229151	0.460815	14.77602	7.03E-07	0.000381	6.752058	0.34624	0.575391	-0.22915	7	1.3E+08	R	II		IGR	opensea	IGR-opense		rs5489640	12;1
cg1649650	0.233809	0.433395	14.75785	7.1E-07	0.000381	6.74349	0.316491	0.5503	-0.23381	16	9063534	F	II		IGR	opensea	IGR-opense		rs5356464	16

cg1457513	0.221978	0.568132	14.75667	7.1E-07	0.000381	6.742933	0.457143	0.679121	-0.22198	22	31933466	R	II	SFI1	Body	opensea	Body-opens		rs5365868	18
cg2253128	0.206549	0.549101	14.75335	7.11E-07	0.000381	6.741369	0.445826	0.652375	-0.20655	7	1.32E+08	R	II	PLXNA4	Body	opensea	Body-opens			
cg1334986	0.239067	0.623967	14.74805	7.13E-07	0.000381	6.738865	0.504433	0.743501	-0.23907	18	74781909	R	II	MBP	Body	opensea	Body-opens		rs5658853	21;18;6;1
cg0485565	0.217355	0.407212	14.74349	7.15E-07	0.000381	6.736709	0.298534	0.515889	-0.21736	3	1.34E+08	F	II	RAB6B	Body	opensea	Body-opens			
cg1664952	0.227419	0.427745	14.74295	7.15E-07	0.000381	6.736454	0.314035	0.541454	-0.22742	5	1.51E+08	R	II	SPARC	3'UTR	opensea	3'UTR-open		rs5651697	24
cg1901013	0.204266	0.512975	14.73856	7.17E-07	0.000381	6.734382	0.410842	0.615108	-0.20427	3	41695019	R	II	ULK4	Body	opensea	Body-opens		rs1474753	51;29
cg0280547	0.216944	0.628725	14.73452	7.18E-07	0.000381	6.732472	0.520253	0.737197	-0.21694	1	23787236	R	II	ASAP3	Body	opensea	Body-opens			
cg1735449	0.219529	0.47516	14.73261	7.19E-07	0.000381	6.731566	0.365395	0.584925	-0.21953	8	74037307	F	II		IGR	opensea	IGR-opense		rs5458792	37;42
cg1598515	0.212903	0.446877	14.73248	7.19E-07	0.000381	6.731507	0.340426	0.553329	-0.2129	6	1.32E+08	F	II		IGR	opensea	IGR-opense		rs5345485	0;23
cg0983683	0.248229	0.56521	14.72693	7.21E-07	0.000381	6.728878	0.441095	0.689325	-0.24823	18	77723602	R	II	HSBP1L1	TSS1500	shore	TSS1500-sh	chr18:777	rs5501489	21;4
cg2572082	0.215544	0.559029	14.72359	7.22E-07	0.000381	6.727298	0.451257	0.666801	-0.21554	1	2849682	F	II		IGR	shore	IGR-shore	chr1:2850	rs5593219	0;7;10;33
cg0968806	0.237381	0.646498	14.72299	7.22E-07	0.000381	6.727015	0.527808	0.765188	-0.23738	5	1.46E+08	R	II	PPP2R2B	5'UTR	opensea	5'UTR-open		rs5771630	50;2;1
cg1620620	0.222227	0.435851	14.71742	7.24E-07	0.000381	6.724378	0.324738	0.546964	-0.22223	3	14307158	F	II		IGR	opensea	IGR-opense		rs5690595	49
cg0776749	0.237685	0.607106	14.71395	7.26E-07	0.000381	6.722734	0.488263	0.725949	-0.23769	6	16698338	F	II	ATXN1	5'UTR	opensea	5'UTR-open		rs1876109	1;43
cg0122374	0.235628	0.491051	14.71277	7.26E-07	0.000381	6.722176	0.373237	0.608866	-0.23563	2	71309618	R	II		IGR	opensea	IGR-opense		rs1122254	45;34;19
cg0667097	0.229348	0.532153	14.71237	7.26E-07	0.000381	6.721988	0.417479	0.646827	-0.22935	9	1.24E+08	R	II		IGR	opensea	IGR-opense		rs1831295	27;1
cg1344283	0.240445	0.527635	14.71176	7.26E-07	0.000381	6.721698	0.407413	0.647858	-0.24044	2	4962504	F	II		IGR	opensea	IGR-opense		rs6721028	29;50
cg0404563	0.234833	0.553766	14.71154	7.26E-07	0.000381	6.721596	0.436349	0.671183	-0.23483	2	1.96E+08	F	II		IGR	opensea	IGR-opense		rs5421717	4;20;50
cg2703986	0.237863	0.554991	14.70845	7.28E-07	0.000381	6.72013	0.43606	0.673922	-0.23786	8	29357644	R	II		IGR	opensea	IGR-opense		rs1513267	2
cg0375923	0.20182	0.493003	14.70752	7.28E-07	0.000381	6.719691	0.392093	0.593914	-0.20182	15	68891950	F	II	CORO2B	Body	opensea	Body-opens		rs1508856	9
cg2715851	0.252857	0.508219	14.70537	7.29E-07	0.000381	6.718669	0.381791	0.634647	-0.25286	6	1.14E+08	F	II		IGR	opensea	IGR-opense		rs5336299	0
cg1184655	0.207138	0.557391	14.69748	7.32E-07	0.000381	6.714931	0.453822	0.66096	-0.20714	19	55788089	R	II	HSPBP1	Body	shelf	Body-shelf	chr19:557	rs5620439	36;23;10;6
cg1748400	0.206713	0.562007	14.69577	7.32E-07	0.000381	6.714118	0.45865	0.665364	-0.20671	2	75700435	R	II		IGR	opensea	IGR-opense		rs6760490	12;1
cg2492271	0.205014	0.445006	14.69362	7.33E-07	0.000381	6.713099	0.342499	0.547513	-0.20501	12	7954043	R	II		IGR	opensea	IGR-opense		rs7301419	48;10;6;2
cg0497161	0.232096	0.458487	14.69017	7.34E-07	0.000381	6.711457	0.342439	0.574535	-0.2321	3	61506940	R	II		IGR	opensea	IGR-opense		rs1915519	42;30;24
cg1636218	0.216544	0.641132	14.68685	7.35E-07	0.000381	6.709882	0.532861	0.749404	-0.21654	3	45390583	F	II		IGR	opensea	IGR-opense			
cg1310160	0.209739	0.385479	14.68664	7.36E-07	0.000381	6.709785	0.28061	0.490349	-0.20974	1	1.11E+08	F	II	SLC16A4	Body	opensea	Body-opens			
cg0854272	0.256446	0.50079	14.68591	7.36E-07	0.000381	6.709436	0.372567	0.629013	-0.25645	8	75010420	R	II		IGR	opensea	IGR-opense		rs8024860	8
cg0918933	0.202501	0.659072	14.67721	7.39E-07	0.000382	6.705304	0.557821	0.760322	-0.2025	19	8745107	R	II		IGR	opensea	IGR-opense		rs7811904	2
cg1453878	0.216753	0.651398	14.67714	7.39E-07	0.000382	6.705272	0.543022	0.759775	-0.21675	4	74402714	R	II		IGR	opensea	IGR-opense		rs1858112	32;19;17;7
cg0887746	0.213835	0.572417	14.66888	7.42E-07	0.000383	6.701344	0.465499	0.679334	-0.21384	1	17520718	R	II		IGR	opensea	IGR-opense		rs5617024	12;2
cg2253472	0.229507	0.529636	14.66043	7.45E-07	0.000384	6.697325	0.414883	0.644389	-0.22951	13	78358711	F	II		IGR	opensea	IGR-opense		rs1870320	1;37
cg2469296	0.207419	0.626293	14.65569	7.47E-07	0.000384	6.695071	0.522583	0.730002	-0.20742	5	55555411	R	II		IGR	opensea	IGR-opense		rs5511078	48;19
cg2309558	0.20338	0.43452	14.65191	7.48E-07	0.000384	6.693272	0.33283	0.53621	-0.20338	12	1.21E+08	R	II		IGR	shelf	IGR-shelf	chr12:120		
cg1327146	0.223895	0.556447	14.65003	7.49E-07	0.000384	6.692377	0.4445	0.668395	-0.2239	5	1.24E+08	R	II	LOC10192	Body	opensea	Body-opens		rs5319567	2
cg1468887	0.233864	0.518827	14.64919	7.5E-07	0.000384	6.691974	0.401895	0.635758	-0.23386	2	2.01E+08	R	II		IGR	opensea	IGR-opense		rs1144779	32
cg0022245	0.200493	0.536309	14.64829	7.5E-07	0.000384	6.691546	0.436062	0.636555	-0.20049	3	23632628	F	II	MIR548AC	Body	opensea	Body-opens			
cg1940346	0.209482	0.502207	14.63793	7.54E-07	0.000385	6.686611	0.397466	0.606948	-0.20948	12	1.23E+08	R	II	LRRC43	TSS1500	opensea	TSS1500-op		rs1492543	38;29;7;5
cg1611698	0.238539	0.594017	14.63095	7.56E-07	0.000385	6.683281	0.474747	0.713286	-0.23854	6	97395162	R	II	KLHL32	5'UTR	opensea	5'UTR-open		rs5820473	20;2
cg0889483	0.220852	0.520027	14.6276	7.58E-07	0.000385	6.681685	0.409601	0.630453	-0.22085	10	5119369	F	II	AKR1C3	Body	opensea	Body-opens		rs5489609	19
cg1775188	0.22716	0.434291	14.61923	7.61E-07	0.000386	6.677686	0.320711	0.547871	-0.22716	3	18141351	F	II	LOC33986	Body	opensea	Body-opens		rs5337594	32
cg1543175	0.20372	0.580443	14.61915	7.61E-07	0.000386	6.67765	0.478583	0.682303	-0.20372	17	48183636	F	II	PDK2	3'UTR	shore	3'UTR-shor	chr17:481	rs5524270	0;17;36
cg0163808	0.246173	0.538573	14.61499	7.63E-07	0.000386	6.675661	0.415486	0.661659	-0.24617	4	75185356	R	II		IGR	opensea	IGR-opense		rs2846231	34;2;1
cg1334094	0.219371	0.412869	14.61114	7.64E-07	0.000386	6.673824	0.303183	0.522554	-0.21937	12	40229749	F	II	SLC2A13	Body	opensea	Body-opens		rs5408403	4
cg2647751	0.2071	0.443898	14.59793	7.69E-07	0.000387	6.667506	0.340348	0.547448	-0.2071	1	47497280	F	II	CYP4X1	Body	opensea	Body-opens		rs1154168	12;31;50
cg1191066	0.209816	0.606226	14.59413	7.71E-07	0.000387	6.665689	0.501318	0.711134	-0.20982	20	61812321	F	II		IGR	shore	IGR-shore	chr20:618	rs5514920	1;36
cg0253485	0.245659	0.608663	14.57892	7.77E-07	0.000388	6.658406	0.485833	0.731492	-0.24566	12	66228466	F	II	HMGA2	Body	opensea	Body-opens		rs5365469	23
cg2086126	0.220141	0.53765	14.57399	7.79E-07	0.000388	6.656042	0.42758	0.647721	-0.22014	2	18944917	R	II		IGR	opensea	IGR-opense		rs5615764	51;1
cg1476736	0.235309	0.478328	14.56169	7.83E-07	0.000389	6.650143	0.360674	0.595982	-0.23531	18	46695469	R	II	DYM	Body	opensea	Body-opens		rs5758856	35;2
cg1264645	0.264758	0.593645	14.56155	7.83E-07	0.000389	6.650079	0.461266	0.726024	-0.26476	2	1.45E+08	R	II		IGR	opensea	IGR-opense			
cg0168566	0.208568	0.486633	14.55193	7.87E-07	0.000389	6.64546	0.382349	0.590917	-0.20857	15	68905043	R	II	CORO2B	Body	opensea	Body-opens		rs5608896	48;40;27
cg1270236	0.267043	0.553049	14.54979	7.88E-07	0.000389	6.644431	0.419528	0.686571	-0.26704	1	86035283	R	II	DDAH1	5'UTR	opensea	5'UTR-open		rs5689986	29;15;1
cg2246882	0.202724	0.447048	14.54896	7.88E-07	0.000389	6.644033	0.345686	0.54841	-0.20272	4	1.56E+08	R	II		IGR	opensea	IGR-opense		rs5532346	18;11;9
cg2708533	0.234613	0.648384	14.54343	7.91E-07	0.000389	6.641375	0.531077	0.76569	-0.23461	7	83784833	R	II	SEMA3A	Body	opensea	Body-opens		rs5575313	31;16;14
cg0209110	0.204798	0.531855	14.54202	7.91E-07	0.000389	6.640696	0.429456	0.634254	-0.2048	6	42123029	R	II	GUCA1A	TSS200	opensea	TSS200-ope			
cg2753945	0.224737	0.723727	14.5391	7.92E-07	0.000389	6.639293	0.611358	0.836095	-0.22474	9	88058136	F	II		IGR	opensea	IGR-opense		rs5754987	15
cg0648577	0.25943	0.560105	14.5369	7.93E-07	0.00039	6.638238	0.43039	0.68982	-0.25943	1	1.85E+08	R	II	FAM129A	Body	opensea	Body-opens		rs1408609	16;15;10;9
cg2093759	0.236031	0.590137	14.5298	7.96E-07	0.000391	6.634821	0.472121	0.708152	-0.23603	6	15876596	R	II		IGR	opensea	IGR-opense		rs1156600	24
cg0785451	0.215884	0.538818	14.5227	7.99E-07	0.000391	6.631402	0.430876	0.64676	-0.21588	4	42893831	R	II	GRXCR1	TSS1500	opensea	TSS1500-op		rs3697709	36;1
cg2110862	0.22254	0.51909	14.52171	7.99E-07	0.000391	6.630927	0.40782	0.63036	-0.22254	8	28022272	F	II	ELP3	Body	opensea	Body-opens		rs1840693	0;1
cg2480926	0.253624	0.453819	14.52139	8E-07	0.000391	6.630774	0.327006	0.580631	-0.25362	17	74260692	F	II	FAM100B	TSS1500	shore	TSS1500-sh	chr17:742	rs5638810	50
cg1063154	0.253797	0.570018	14.51737	8.01E-07	0.000391	6.628838	0.443119	0.696916	-0.2538	4	1.15E+08	F	II		IGR	shelf	IGR-shelf	chr4:1149	rs5359994	42
cg0543936	0.217523	0.526439	14.51378	8.03E-07	0.000391	6.627108	0.417677	0.6352	-0.21752	15	45028098	F	II	TRIM69	TSS1500	opensea	TSS1500-op			
cg0302972	0.226222	0.520265	14.5069	8.05E-07	0.000391	6.623791	0.410714	0.633376	-0.22622	16	18799331	R	II	RPS15A	Body	shore	Body-shore	chr16:188		
cg1348303	0.214808	0.445533	14.50429	8.07E-07	0.000391	6.622533	0.338129	0.552938	-0.21481	12	1.28E+08	R	II	LOC10192	TSS1500	opensea	TSS1500-op		rs1490893	11;5;2;1
cg1457344	0.213448	0.499581	14.50012	8.08E-07	0.000391	6.620524	0.392857	0.606305	-0.21345	7	27165187	F	II	HOXA3	5'UTR	shore	5'UTR-shor	chr7:2716	rs5283100	1;34
cg1215445	0.216817	0.532517	14.49015	8.12E-07	0.000392	6.615712	0.424109	0.640926	-0.21682	6	1.5E+08	R	II		IGR	opensea	IGR-opense		rs1849185	0
cg0141114	0.21977	0.502923	14.48601	8.14E-07	0.000392	6.61371	0.393038	0.612808	-0.21977	9	92770209	R	II		IGR	opensea	IGR-opense		rs1144121	5;2;1
cg2090297	0.200905	0.443342	14.48253	8.16E-07	0.000392	6.612032	0.34289	0.543795	-0.20091	8	11138403	F	II		IGR	shelf	IGR-shelf	chr8:1114	rs5385895	20;41;51
cg2203907	0.216564	0.662871	14.47603	8.18E-07	0.000393	6.608888	0.554589	0.771153	-0.21656	4	72635221	R	II	GC	Body	opensea	Body-opens		rs5622825	2
cg1367680	0.224474	0.539875	14.47	8.21E-07	0.000394	6.605974	0.427638	0.652112	-0.22447	9	89722432	R	II		IGR	opensea	IGR-opense		rs7590373	19;1
cg1959553	0.222324	0.459029	14.46167	8.24E-07	0.000394	6.601944	0.347867	0.570191	-0.22232	5	1.24E+08	R	II	ZNF608	Body	opensea	Body-opens		rs5668573	1
cg1747573	0.209576	0.533911	14.45889	8.25E-07	0.000394	6.600597	0.429123	0.638699	-0.20958	1	96830030	F	II		IGR	opensea	IGR-opense			
cg1778166	0.240852	0.590543	14.45767	8.26E-07	0.000394	6.600006	0.470117	0.710969	-0.24085	20	9334016	R	II	PLCB4	Body	opensea	Body-opens		rs1248029	38;16;15;1
cg2105004	0.23773	0.641414	14.44775	8.3E-07	0.000395	6.595202	0.522549	0.760279	-0.23773	3	17790877	F	II		IGR	opensea	IGR-opense		rs1446885	19;35
cg0709718	0.208248	0.461359	14.44178	8.33E-07	0.000395	6.592309	0.357235	0.565483	-0.20825	4	1.54E+08	R	II	FHDC1	3'UTR	shelf	3'UTR-shelf	chr4:1538	rs908527	13
cg0171591	0.200106	0.509096	14.43337	8.36E-07	0.000395	6.588233	0.409042	0.609149	-0.20011	17	4634022	R	II	MED11	TSS1500	shore	TSS1500-sh	chr17:463	rs1149520	2
cg1524257	0.213661	0.487429	14.43272	8.37E-07	0.000395	6.587914	0.380598	0.594259	-0.21366	2	1.37E+08	F	II		IGR	opensea	IGR-opense		rs1818321	1;7
cg1807393	0.261073	0.595373	14.43229	8.37E-07	0.000395	6.587709	0.464836	0.725909	-0.26107	4	1.01E+08	R	II	DAPP1	3'UTR	opensea	3'UTR-open			
cg0894575	0.22408	0.462106	14.43115	8.37E-07	0.000395	6.587153	0.350067	0.574146	-0.22408	6	54573250	R	II		IGR	opensea	IGR-opense		rs7966587	40
cg2749447	0.229607	0.451551	14.42618	8.39E-07	0.000395	6.584744	0.336748	0.566355	-0.22961	6	84172561	F	II		IGR	opensea	IGR-opense		rs1395408	8;36
cg0689336	0.209443	0.466149	14.42439	8.4E-07	0.000395	6.583874	0.361427	0.570871	-0.20944	20	47897124	F	II	C20orf199	Body	shore	Body-shore	chr20:478	rs5279959	22
cg0142016	0.219904	0.599539	14.41895	8.42E-07	0.000395	6.581232	0.489586	0.709491	-0.2199	15	54793071	F	II	UNC13C	Body	opensea	Body-opens		rs5712771	5
cg0113502	0.240583	0.440117	14.41805	8.43E-07	0.000395	6.580795	0.319825	0.560408	-0.24058	5	7872175	R	II	MTRR	Body	shelf	Body-shelf	chr5:7868	rs7821590	48;45;27;1
cg2286841	0.240809	0.494079	14.41466	8.44E-07	0.000395	6.579146	0.373675	0.614484	-0.24081	4	15987764	F	II	PROM1	Body	opensea	Body-opens		rs7688123	43
cg0182280	0.213424	0.521038	14.40957	8.46E-07	0.000395	6.576675	0.414326	0.62775	-0.21342	5	53615682	R	II	LINC01033	TSS1500	opensea	TSS1500-op		rs1146412	45;15;1
cg0145718	0.216623	0.578364	14.40871	8.47E-07	0.000395	6.576254	0.470052	0.686676	-0.21662	6	47252055	R	II	TNFRSF21	Body	opensea	Body-opens		rs1465783	12;2;1
cg1871197	0.21508	0.464658	14.40154	8.5E-07	0.000395	6.572768	0.357118	0.572199	-0.21508	3	1.51E+08	R	II		IGR	opensea	IGR-opense		rs5471840	12;2
cg1486825	0.23494	0.613682	14.40064	8.5E-07	0.000395	6.57233	0.496212	0.731152	-0.23494	6	82958285	F	II	IBTK	TSS1500	shore	TSS1500-sh	chr6:8295	rs5578034	15
cg2201576	0.335975	0.548551	14.39952	8.51E-07	0.000395	6.571784	0.380564	0.716539	-0.33598	6	34624127	F	II	C6orf106	Body	opensea	Body-opens		rs5715076	0;21;39;46
cg0145518	0.20081	0.660457	14.39951	8.51E-07	0.000395	6.571784	0.560052	0.760862	-0.20081	5	34917095	R	II	RAD1	TSS1500	shore	TSS1500-sh	chr5:3491		
cg1982384	0.227062	0.478465	14.39692	8.52E-07	0.000395	6.570522	0.364934	0.591996	-0.22706	6	1.39E+08	F	II	KIAA1244	Body	opensea	Body-opens		rs1877921	6;14
cg0616460	0.220833	0.515037	14.39672	8.52E-07	0.000395	6.570423	0.40462	0.625453	-0.22083	8	87086018	F	II		IGR	shelf	IGR-shelf	chr8:8708	rs1834219	0;9;12;24;46
cg0240440	0.229249	0.564529	14.3965	8.52E-07	0.000395	6.570319	0.449905	0.679154	-0.22925	9	5480172	R	II		IGR	opensea	IGR-opense			
cg2727824	0.216498	0.627648	14.39381	8.53E-07	0.000395	6.56901	0.519399	0.735897	-0.2165	2	2.17E+08	R	II		IGR	opensea	IGR-opense		rs3745788	42;1
cg0021188	0.223516	0.564377	14.39175	8.54E-07	0.000395	6.568004	0.452619	0.676135	-0.22352	3	1.76E+08	F	II		IGR	opensea	IGR-opense		rs5523777	0;5;10;17;40;47
cg2313071	0.215479	0.521957	14.39001	8.55E-07	0.000396	6.567159	0.414217	0.629696	-0.21548	9	78526426	R	II	PCSK5	Body	opensea	Body-opens		rs5771045	21;1
cg0920406	0.231964	0.54614	14.38505	8.57E-07	0.000396	6.564742	0.430158	0.662122	-0.23196	3	1.41E+08	R	II	SPSB4	Body	opensea	Body-opens		rs1885349	2
cg1173789	0.219197	0.705959	14.36595	8.66E-07	0.000397	6.555428	0.596361	0.815557	-0.2192	5	1.06E+08	R	II		IGR	opensea	IGR-opense		rs5662490	49;2;1
cg0088622	0.236853	0.536659	14.36544	8.66E-07	0.000397	6.555181	0.418233	0.655086	-0.23685	9	87374383	R	II	NTRK2	Body	opensea	Body-opens			
cg1341398	0.222349	0.551223	14.36387	8.67E-07	0.000397	6.554415	0.440049	0.662398	-0.22235	13	44868172	F	II		IGR	opensea	IGR-opense			
cg1941172	0.230732	0.484296	14.36347	8.67E-07	0.000397	6.554221	0.36893	0.599662	-0.23073	17	34207663	F	II	CCL5	TSS1500	opensea	TSS1500-op			
cg1124864	0.267126	0.545845	14.36198	8.67E-07	0.000397	6.553495	0.412282	0.679407	-0.26713	12	50645711	F	II	LIMA1	5'UTR	opensea	5'UTR-open		rs5773929	0
cg1528560	0.206334	0.612622	14.3521	8.72E-07	0.000398	6.548666	0.509455	0.715789	-0.20633	14	68466640	F	II	RAD51B	Body	opensea	Body-opens		rs1387527	4
cg1453724	0.249157	0.548762	14.3435	8.76E-07	0.000399	6.544466	0.424184	0.673341	-0.24916	9	1.27E+08	R	II	DENND1A	Body	opensea	Body-opens		rs3724937	38;1
cg0103195	0.206775	0.514593	14.34301	8.76E-07	0.000399	6.544224	0.411206	0.617981	-0.20677	6	31941361	F	II	STK19	Body	shore	Body-shore	chr6:3193	rs1196382	10;28;29;51
cg1593688	0.200932	0.543884	14.33931	8.78E-07	0.000399	6.542415	0.443418	0.64435	-0.20093	22	22390113	F	II		IGR	opensea	IGR-opense		rs1380607	1;18
cg1974034	0.241826	0.572718	14.3304	8.82E-07	0.0004	6.538057	0.451805	0.69363	-0.24183	8	20693391	R	II		IGR	opensea	IGR-opense		rs5566613	17;9
cg1590905	0.25481	0.527003	14.32911	8.82E-07	0.0004	6.537426	0.399598	0.654408	-0.25481	12	71837175	R	II	LGR5	Body	shelf	Body-shelf	chr12:718	rs5607118	34;1
cg1368502	0.218506	0.549711	14.32623	8.83E-07	0.0004	6.536016	0.440458	0.658964	-0.21851	1	79158388	F	II		IGR	opensea	IGR-opense		rs1839935	0;13
cg2257639	0.241699	0.574179	14.32149	8.86E-07	0.0004	6.533697	0.453329	0.695028	-0.2417	13	24042599	F	II	LINC00327	TSS1500	shore	TSS1500-sh	chr13:240		
cg0599901	0.214771	0.662775	14.32125	8.86E-07	0.0004	6.533575	0.55539	0.770161	-0.21477	3	63881712	R	II	ATXN7	5'UTR	opensea	5'UTR-open		rs5414952	39;10
cg1259363	0.211416	0.494888	14.321	8.86E-07	0.0004	6.533453	0.38918	0.600596	-0.21142	11	5143555	F	II	OR52A4	TSS1500	opensea	TSS1500-op		rs1124622	31
cg1049744	0.241312	0.49695	14.31979	8.86E-07	0.0004	6.532863	0.376294	0.617606	-0.24131	13	77427321	F	II		IGR	opensea	IGR-opense		rs5759555	0;1;17;23;46;47
cg0037625	0.206509	0.463254	14.31818	8.87E-07	0.0004	6.532074	0.359999	0.566508	-0.20651	13	23946885	R	II	SACS	5'UTR	shelf	5'UTR-shelf	chr13:239	rs1131096	34;22;2
cg0610555	0.205091	0.634361	14.31763	8.87E-07	0.0004	6.531804	0.531815	0.736907	-0.20509	6	22017093	R	II	FLJ22536	Body	opensea	Body-opens			
cg1393180	0.243675	0.472392	14.31306	8.89E-07	0.0004	6.529564	0.350555	0.594229	-0.24367	12	71983445	F	II		IGR	opensea	IGR-opense		rs1180832	0
cg2359419	0.214623	0.457397	14.30688	8.92E-07	0.0004	6.526535	0.350085	0.564708	-0.21462	2	45121446	F	II		IGR	opensea	IGR-opense		rs5745534	0;1;20;33
cg2057672	0.216707	0.650517	14.30472	8.93E-07	0.0004	6.525476	0.542164	0.758871	-0.21671	11	3637658	F	II		IGR	opensea	IGR-opense		rs5778313	0;24

cg0870287	0.257682	0.558469	14.30182	8.95E-07	0.0004	6.524052	0.429628	0.68731	-0.25768	10	1.01E+08	F	II		IGR	opensea	IGR-opense		rs1853060 1;32;42
cg0690631	0.225545	0.528713	14.29748	8.97E-07	0.0004	6.521922	0.41594	0.641486	-0.22555	6	74502300	R	II	CD109	Body	opensea	Body-opens		rs5651457 23;19;10;5;1
cg0146418	0.278602	0.493724	14.29744	8.97E-07	0.0004	6.521901	0.354423	0.633025	-0.2786	10	52191661	F	II	SGMS1	5'UTR	opensea	5'UTR-oper		rs5672233 0
cg1218908	0.253641	0.504656	14.29408	8.98E-07	0.0004	6.520254	0.377835	0.631477	-0.25364	4	1.85E+08	F	II		IGR	opensea	IGR-opense		rs1905910 0
cg0544729	0.251994	0.600165	14.2921	8.99E-07	0.0004	6.51928	0.474168	0.726162	-0.25199	5	1.58E+08	R	II	EBF1	Body	opensea	Body-opens		
cg2209337	0.221235	0.588192	14.29182	8.99E-07	0.0004	6.519142	0.477575	0.69881	-0.22123	2	1.74E+08	R	II	ZAK	Body	opensea	Body-opens		
cg1415440	0.205125	0.442048	14.28805	9.01E-07	0.0004	6.517293	0.339486	0.544611	-0.20512	18	52792171	R	II	LOC10192	TSS1500	opensea	TSS1500-op		rs5339661 16;2
cg1985948	0.23034	0.53457	14.28763	9.01E-07	0.0004	6.517088	0.4194	0.64974	-0.23034	21	39234433	R	II	KCNJ6	5'UTR	opensea	5'UTR-oper		rs1929960 37;13;12
cg0519545	0.23131	0.610396	14.28729	9.01E-07	0.0004	6.516919	0.494741	0.72605	-0.23131	12	69660384	F	II	CPSF6	3'UTR	opensea	3'UTR-oper		rs5389524 1;4
cg0320366	0.210381	0.573988	14.28469	9.03E-07	0.0004	6.51564	0.468798	0.679179	-0.21038	13	67946668	R	II	LINC00364	Body	opensea	Body-opens		rs1781590 28;27
cg2098820	0.224659	0.487153	14.28118	9.04E-07	0.0004	6.513916	0.374823	0.599482	-0.22466	11	22370099	F	II	SLC17A6	Body	opensea	Body-opens		rs1143591 0;9;28;47
cg2687819	0.2378	0.675287	14.28102	9.04E-07	0.0004	6.513837	0.556386	0.794187	-0.2378	5	1794420	R	II		IGR	opensea	IGR-opense		rs5319988 44
cg0533406	0.226735	0.523014	14.27652	9.06E-07	0.0004	6.511624	0.409646	0.636381	-0.22674	15	36715027	F	II		IGR	opensea	IGR-opense		rs5377240 22
cg1888576	0.209773	0.452161	14.2729	9.08E-07	0.0004	6.509847	0.347275	0.557048	-0.20977	14	1E+08	F	II	CCDC85C	Body	opensea	Body-opens		rs1419904 0;27
cg0715447	0.257871	0.462498	14.27208	9.08E-07	0.0004	6.509444	0.333562	0.591433	-0.25787	3	1.53E+08	R	II		IGR	opensea	IGR-opense		rs1808315 44;2;1
cg1268002	0.206759	0.55542	14.27141	9.09E-07	0.0004	6.509113	0.45204	0.658799	-0.20676	8	1.45E+08	R	II	SPATC1	Body	shelf	Body-shelf	chr8:1451(	rs5610169 49;17;14;2
cg0048844	0.2693	0.545778	14.26819	9.1E-07	0.0004	6.50753	0.411128	0.680428	-0.2693	1	74929641	R	II	FPGT-TNNI:	Body	opensea	Body-opens		rs7831695 32;26;24
cg0315439	0.224731	0.503214	14.26791	9.1E-07	0.0004	6.507391	0.390848	0.615579	-0.22473	5	16966088	R	II		IGR	opensea	IGR-opense		rs2019603 1
cg2093190	0.222973	0.583663	14.26669	9.11E-07	0.0004	6.506792	0.472177	0.69515	-0.22297	1	29591726	R	II	PTPRU	Body	opensea	Body-opens		rs5621702 13
cg1696069	0.226083	0.600839	14.26538	9.12E-07	0.0004	6.506148	0.487797	0.71388	-0.22608	2	2.08E+08	F	II	CPO	Body	opensea	Body-opens		rs5595102 1;37;43;49
cg0136902	0.259901	0.484634	14.24216	9.23E-07	0.000402	6.494707	0.354684	0.614585	-0.2599	10	11133829	R	II	CELF2	Body	opensea	Body-opens		rs1147858 37;8
cg1120388	0.227728	0.443754	14.23731	9.25E-07	0.000402	6.492316	0.329889	0.557618	-0.22773	2	65547670	F	II	SPRED2	Body	opensea	Body-opens		
cg0004059	0.234134	0.545656	14.23618	9.25E-07	0.000402	6.491758	0.428589	0.662722	-0.23413	5	1.45E+08	R	II	SH3RF2	5'UTR	shore	5'UTR-shor	chr5:1453:	rs5458217 28;24;20;9
cg0519685	0.205606	0.420418	14.23512	9.26E-07	0.000402	6.491236	0.317615	0.523221	-0.20561	15	57833021	R	II	CGNL1	Body	opensea	Body-opens		rs1440240 27;2
cg1132746	0.204508	0.538514	14.2299	9.29E-07	0.000402	6.488659	0.43626	0.640768	-0.20451	22	29628227	F	II	EMID1	ExonBnd	opensea	ExonBnd-o		rs1510036 1;33;37;47
cg1916733	0.231129	0.594951	14.2232	9.32E-07	0.000402	6.485351	0.479387	0.710516	-0.23113	8	62581684	F	II	ASPH	Body	opensea	Body-opens		rs1859688 15;18
cg2431807	0.205226	0.504595	14.22079	9.33E-07	0.000402	6.484157	0.401982	0.607209	-0.20523	17	18091662	R	II	ALKBH5	Body	shelf	Body-shelf	chr17:180(	rs7776975 7;1
cg2017005	0.204956	0.559209	14.21986	9.33E-07	0.000402	6.483697	0.456731	0.661687	-0.20496	9	94262767	F	II		IGR	opensea	IGR-opense		rs5640769 18;28
cg1659669	0.235117	0.623693	14.21141	9.37E-07	0.000403	6.479522	0.506134	0.741251	-0.23512	19	41260294	F	II	SNRPA	Body	shelf	Body-shelf	chr19:412!	rs5652021 1;6;19
cg2035837	0.206642	0.569472	14.20921	9.39E-07	0.000403	6.478437	0.466151	0.672793	-0.20664	2	1.47E+08	F	II		IGR	opensea	IGR-opense		
cg0567742	0.208667	0.410593	14.2066	9.4E-07	0.000403	6.477146	0.306259	0.514927	-0.20867	12	39767032	R	II	KIF21A	Body	opensea	Body-opens		rs1112785 39;21;18;6;5;2;1
cg2639999	0.225979	0.539117	14.20128	9.42E-07	0.000404	6.47451	0.426127	0.652106	-0.22598	1	1.99E+08	R	II	PTPRC	Body	opensea	Body-opens		
cg2485504	0.210705	0.564336	14.1967	9.45E-07	0.000404	6.472246	0.458983	0.669688	-0.21071	10	18938449	R	II	NSUN6	Body	shelf	Body-shelf	chr10:189(	rs3718488 21
cg0136760	0.205693	0.552526	14.19651	9.45E-07	0.000404	6.472152	0.44968	0.655372	-0.20569	16	66938874	F	II		IGR	opensea	IGR-opense		
cg2287587	0.261852	0.501171	14.1915	9.47E-07	0.000405	6.46967	0.370245	0.632097	-0.26185	4	72978321	F	II	NPFFR2	5'UTR	opensea	5'UTR-oper		rs7639025 1;17;33;44
cg2602945	0.232726	0.490015	14.19117	9.47E-07	0.000405	6.469508	0.373652	0.606379	-0.23273	7	47818048	F	II	PKD1L1	Body	opensea	Body-opens		rs1436480 0;1;49;51
cg0319951	0.243067	0.626955	14.18654	9.5E-07	0.000406	6.46721	0.505422	0.748489	-0.24307	5	71777883	R	II	ZNF366	5'UTR	opensea	5'UTR-oper		rs5334122 50;44;6;1
cg1512017	0.201385	0.442216	14.18049	9.53E-07	0.000406	6.464216	0.341523	0.542908	-0.20139	21	42091972	F	II	DSCAM	Body	opensea	Body-opens		rs1420568 1;32
cg2581152	0.217694	0.469519	14.17962	9.53E-07	0.000406	6.463783	0.360672	0.578366	-0.21769	2	74570564	R	II	SLC4A5	TSS200	opensea	TSS200-ope		rs5326647 24;1
cg2102395	0.253559	0.594755	14.17954	9.53E-07	0.000406	6.463743	0.467975	0.721534	-0.25356	5	59783035	R	II	PDE4D	5'UTR	opensea	5'UTR-oper		rs1497471 51
cg0920146	0.223347	0.61176	14.17499	9.55E-07	0.000406	6.461487	0.500087	0.723434	-0.22335	7	65613851	R	II	CRCP	Body	opensea	Body-opens		rs5313859 16;13
cg0219092	0.242968	0.593589	14.17421	9.56E-07	0.000406	6.461101	0.472105	0.715073	-0.24297	8	90427508	F	II		IGR	opensea	IGR-opense		rs5567773 0
cg0767594	0.211799	0.406771	14.17358	9.56E-07	0.000406	6.460788	0.300872	0.512671	-0.2118	15	62576064	F	II		IGR	opensea	IGR-opense		rs1484108 12;33;43
cg1475666	0.216435	0.441507	14.16797	9.59E-07	0.000407	6.458001	0.33329	0.549725	-0.21644	5	26488099	R	II		IGR	opensea	IGR-opense		rs5740078 48
cg1862383	0.228998	0.548147	14.16431	9.61E-07	0.000407	6.456187	0.433648	0.662646	-0.229	2	10261907	F	II	RRM2	TSS1500	shore	TSS1500-sh	chr2:1026:	rs7254277 34;40
cg1096011	0.217064	0.600594	14.16415	9.61E-07	0.000407	6.456109	0.492062	0.709127	-0.21706	3	1.15E+08	F	II	GAP43	Body	opensea	Body-opens		rs1483707 31
cg2620142	0.226299	0.607495	14.16195	9.62E-07	0.000407	6.455013	0.494346	0.720644	-0.2263	4	1.86E+08	R	II	LVCAT8	Body	opensea	Body-opens		rs1148782 31;22;2;1
cg2422848	0.242586	0.447583	14.15949	9.63E-07	0.000407	6.453794	0.32629	0.568877	-0.24259	8	1.11E+08	F	II		IGR	opensea	IGR-opense		rs5708242 1;15;27
cg0800101	0.203557	0.463371	14.15723	9.64E-07	0.000407	6.452669	0.361593	0.56515	-0.20356	11	1.3E+08	R	II		IGR	opensea	IGR-opense		rs1819657 24;19
cg2304990	0.242699	0.652476	14.15395	9.66E-07	0.000407	6.451041	0.531127	0.773826	-0.2427	3	58866128	R	II	C3orf67	Body	opensea	Body-opens		rs5641833 33;13;1
cg0864474	0.209801	0.501072	14.15344	9.66E-07	0.000407	6.450786	0.396171	0.605972	-0.2098	15	67211859	F	II		IGR	opensea	IGR-opense		rs3730233 27
cg1572623	0.204436	0.455235	14.15333	9.66E-07	0.000407	6.450732	0.353017	0.557453	-0.20444	17	4915387	R	II	KIF1C	Body	opensea	Body-opens		
cg1277177	0.208096	0.532302	14.15153	9.67E-07	0.000407	6.449838	0.428254	0.63635	-0.2081	18	59057418	F	II		IGR	opensea	IGR-opense		rs1924179 3;6;19;25
cg2169065	0.22339	0.536012	14.14717	9.69E-07	0.000407	6.447672	0.424317	0.647707	-0.22339	5	36369252	F	II		IGR	opensea	IGR-opense		rs1167224 0
cg1173707	0.20857	0.521579	14.14016	9.73E-07	0.000407	6.444183	0.417294	0.625864	-0.20857	13	75990822	R	II	TBC1D4	Body	opensea	Body-opens		rs1866204 34;29;1
cg0474761	0.20961	0.438266	14.12995	9.78E-07	0.000408	6.439101	0.333461	0.543071	-0.20961	8	6914838	F	II	DEFA5	TSS1500	opensea	TSS1500-op		rs5585978 34
cg2157010	0.259976	0.479515	14.12746	9.79E-07	0.000408	6.437865	0.349527	0.609503	-0.25998	1	1.79E+08	F	II		IGR	opensea	IGR-opense		rs1407135 0;2;18
cg2161012	0.213277	0.534748	14.12559	9.8E-07	0.000408	6.436932	0.428109	0.641387	-0.21328	20	50146088	R	II	NFATC2	5'UTR	opensea	5'UTR-oper		
cg2635504	0.21232	0.583399	14.12242	9.82E-07	0.000409	6.43535	0.477238	0.689559	-0.21232	2	32414912	F	II	SLC30A6	Body	opensea	Body-opens		rs5543297 1
cg0681920	0.204348	0.540289	14.11755	9.84E-07	0.000409	6.432926	0.438115	0.642463	-0.20435	3	1.25E+08	R	II	ITGB5	Body	opensea	Body-opens		rs1486930 0;15
cg0318150	0.220182	0.428124	14.11675	9.85E-07	0.000409	6.432527	0.318033	0.538215	-0.22018	6	7468364	F	II		IGR	opensea	IGR-opense		rs7758281 19
cg0667966	0.264073	0.596504	14.11488	9.86E-07	0.000409	6.431593	0.464468	0.728541	-0.26407	8	94966773	F	II		IGR	opensea	IGR-opense		rs1837980 0;16
cg1240788	0.226248	0.534573	14.11369	9.86E-07	0.000409	6.431	0.421449	0.647697	-0.22625	6	1.33E+08	F	II	MOXD1	Body	opensea	Body-opens		rs5304445 0;23;35
cg0288039	0.217342	0.40408	14.11032	9.88E-07	0.000409	6.429319	0.295409	0.512751	-0.21734	1	43313820	F	II	ZNF691	5'UTR	shore	5'UTR-shor	chr1:4331:	rs5375140 0;16
cg1761887	0.216698	0.4421	14.10935	9.89E-07	0.000409	6.428835	0.333751	0.550449	-0.2167	20	32235621	R	II	CBFA2T2	3'UTR	shelf	3'UTR-shelf	chr20:322:	rs5509248 2
cg1743667	0.266155	0.532956	14.1093	9.89E-07	0.000409	6.428812	0.399879	0.666034	-0.26615	4	1.06E+08	F	II	ARHGEF38	Body	opensea	Body-opens		rs1405735 8;49;50
cg2538101	0.227214	0.4033	14.10884	9.89E-07	0.000409	6.428581	0.289693	0.516907	-0.22721	3	3151795	R	II	IL5RA	5'UTR	opensea	5'UTR-oper		rs2290608 38
cg0625244	0.223693	0.433466	14.10648	9.9E-07	0.000409	6.427405	0.32162	0.545313	-0.22369	11	1.06E+08	F	II	AASDHPPT	Body	opensea	Body-opens		rs5608174 31
cg0524192	0.223506	0.445364	14.10022	9.93E-07	0.000409	6.424281	0.333611	0.557117	-0.22351	9	1.36E+08	R	II	RALGDS	Body	opensea	Body-opens		rs1806968 1;37
cg0845259	0.241889	0.596079	14.09734	9.95E-07	0.000409	6.422842	0.475134	0.717024	-0.24189	2	55529467	F	II	CCDC88A	Body	opensea	Body-opens		rs1931481 1;13
cg2611123	0.215196	0.420346	14.09503	9.96E-07	0.000409	6.421687	0.312748	0.527944	-0.2152	21	18500534	R	II		IGR	opensea	IGR-opense		rs1848198 45;42;41;6;2
cg0267144	0.238051	0.548451	14.08984	9.99E-07	0.000409	6.419096	0.429425	0.667476	-0.23805	5	73614960	F	II		IGR	opensea	IGR-opense		rs7765994 40;42;48
cg1963310	0.226668	0.478637	14.08964	9.99E-07	0.000409	6.418996	0.365303	0.591972	-0.22667	13	50028789	R	II	SETDB2	Body	opensea	Body-opens		rs1853041 1
cg0287046	0.200163	0.587204	14.07939	1E-06	0.00041	6.413871	0.487122	0.687285	-0.20016	9	37187506	F	II	ZCCHC7	Body	opensea	Body-opens		rs5749864 0;44
cg2560696	0.205526	0.655741	14.06911	1.01E-06	0.000411	6.40873	0.552978	0.758504	-0.20553	1	1.11E+08	F	II		IGR	opensea	IGR-opense		rs7411926 23
cg0662588	0.230259	0.609193	14.06585	1.01E-06	0.000411	6.407094	0.494063	0.724322	-0.23026	3	1.5E+08	F	II	RNF13	Body	opensea	Body-opens		rs5658027 1
cg0009331	0.24285	0.531466	14.06079	1.01E-06	0.000412	6.404563	0.410041	0.652891	-0.24285	13	27827907	R	II	RPL21	5'UTR	shore	5'UTR-shor	chr13:278:	rs1386590 37;21
cg2522382	0.226178	0.475135	14.05889	1.01E-06	0.000412	6.403611	0.362046	0.588224	-0.22618	13	33593349	F	II	KL	Body	shore	Body-shore	chr13:335!	rs1919689 1;4
cg2275613	0.214672	0.565872	14.05188	1.02E-06	0.000413	6.400096	0.458536	0.673208	-0.21467	1	2.46E+08	F	II	KIF26B	Body	opensea	Body-opens		rs5523809 28
cg2437633	0.249768	0.596345	14.04671	1.02E-06	0.000413	6.397504	0.471461	0.721229	-0.24977	2	1.92E+08	F	II	MYO1B	Body	opensea	Body-opens		rs5363003 0;41
cg2551709	0.225639	0.354923	14.04199	1.02E-06	0.000413	6.39514	0.242103	0.467742	-0.22564	14	61154039	F	II		IGR	opensea	IGR-opense		rs5487115 8;37
cg0250900	0.208666	0.477782	14.04198	1.02E-06	0.000413	6.395132	0.373449	0.582115	-0.20867	4	78226903	F	II		IGR	opensea	IGR-opense		rs5474008 1
cg1712558	0.215288	0.586592	14.03817	1.03E-06	0.000413	6.393221	0.478949	0.694236	-0.21529	10	1.23E+08	F	II		IGR	opensea	IGR-opense		rs1132105 1;15
cg0229456	0.23806	0.504904	14.03669	1.03E-06	0.000413	6.392477	0.385874	0.623934	-0.23806	18	55732481	R	II	NEDD4L	Body	opensea	Body-opens		rs5733739 11;2;1
cg2627755	0.211484	0.442604	14.02915	1.03E-06	0.000413	6.388691	0.336862	0.548346	-0.21148	14	54507223	F	II		IGR	opensea	IGR-opense		rs1143243 0;29
cg2573492	0.220161	0.550613	14.02557	1.03E-06	0.000413	6.386894	0.440532	0.660693	-0.22016	6	1.05E+08	R	II		IGR	shelf	IGR-shelf	chr6:10540	rs1505654 48
cg1491012	0.234265	0.45138	14.02462	1.03E-06	0.000413	6.386417	0.334248	0.568512	-0.23426	14	1.01E+08	R	II		IGR	opensea	IGR-opense		rs1512287 1
cg0095758	0.286366	0.525014	14.02067	1.04E-06	0.000413	6.38443	0.381832	0.668197	-0.28637	15	86189830	R	II	AKAP13	Body	opensea	Body-opens		rs5378322 51;27
cg1300219	0.202762	0.597099	14.01696	1.04E-06	0.000413	6.382566	0.495718	0.698481	-0.20276	13	97832207	R	II	LINC00456	TSS1500	opensea	TSS1500-op		rs5610977 40;16;2
cg1522475	0.226423	0.550848	14.01595	1.04E-06	0.000413	6.382059	0.437636	0.664059	-0.22642	13	91284474	F	II		IGR	opensea	IGR-opense		rs5276856 0;30
cg0394629	0.218054	0.437995	14.00963	1.04E-06	0.000413	6.378878	0.328968	0.547022	-0.21805	1	71140689	R	II		IGR	opensea	IGR-opense		rs5656799 17
cg2069646	0.221498	0.518814	14.00066	1.05E-06	0.000414	6.374366	0.408066	0.629563	-0.2215	16	54277492	R	II		IGR	opensea	IGR-opense		
cg2736599	0.241006	0.505612	13.99951	1.05E-06	0.000414	6.373782	0.385109	0.626115	-0.24101	5	1.23E+08	F	II		IGR	opensea	IGR-opense		
cg1757391	0.236063	0.682307	13.99763	1.05E-06	0.000414	6.372836	0.564275	0.800338	-0.23606	3	1.43E+08	F	II	SLC9A9	Body	opensea	Body-opens		rs1430244 0;12
cg1626354	0.231859	0.571252	13.99383	1.05E-06	0.000414	6.370921	0.455322	0.687181	-0.23186	8	1.16E+08	F	II	TRPS1	Body	opensea	Body-opens		rs5754789 0;44
cg2669815	0.207256	0.577133	13.99153	1.05E-06	0.000414	6.369763	0.473505	0.680761	-0.20726	3	73450135	R	II	PDZRN3	Body	opensea	Body-opens		rs3696897 45;29;6
cg1381802	0.226599	0.648718	13.98848	1.05E-06	0.000414	6.368228	0.535419	0.762018	-0.2266	8	18899193	F	II		IGR	opensea	IGR-opense		rs1395518 48
cg0854856	0.224827	0.530974	13.98486	1.06E-06	0.000414	6.366403	0.41856	0.643387	-0.22483	9	4100149	F	II	GLIS3	Body	opensea	Body-opens		rs5655915 40;44;49
cg0943466	0.200263	0.458023	13.97632	1.06E-06	0.000414	6.362093	0.357892	0.558154	-0.20026	15	67078890	F	II		IGR	opensea	IGR-opense		rs7537407 1
cg1700276	0.205378	0.426465	13.9726	1.06E-06	0.000414	6.360217	0.323776	0.529154	-0.20538	1	22579251	R	II		IGR	opensea	IGR-opense		rs7405999 2
cg0505633	0.20261	0.49908	13.97049	1.06E-06	0.000414	6.359153	0.397775	0.600386	-0.20261	8	35719518	R	II		IGR	opensea	IGR-opense		rs5360164 4;1
cg1154815	0.213111	0.582521	13.96903	1.06E-06	0.000414	6.358413	0.475965	0.689076	-0.21311	2	2.02E+08	R	II	AOX2P	Body	opensea	Body-opens		rs1492170 50;13;4;1
cg2743480	0.227304	0.561338	13.96729	1.06E-06	0.000414	6.357534	0.447686	0.67499	-0.2273	17	48880966	F	II		IGR	opensea	IGR-opense		rs5564592 28;39;46
cg2441544	0.215695	0.518766	13.96243	1.07E-06	0.000414	6.355084	0.410918	0.626613	-0.21569	6	80163855	F	II		IGR	opensea	IGR-opense		rs5308577 42;49
cg2715967	0.20793	0.503008	13.96121	1.07E-06	0.000414	6.354464	0.399042	0.606973	-0.20793	9	1.25E+08	R	II	DAB2IP	TSS1500	opensea	TSS1500-op		rs5711430 26
cg0395087	0.200912	0.517975	13.95839	1.07E-06	0.000415	6.35304	0.417519	0.618432	-0.20091	14	60045040	F	II	C14orf38	TSS1500	shore	TSS1500-sh	chr14:600	rs1435401 39
cg0562438	0.237199	0.535668	13.95311	1.07E-06	0.000415	6.350369	0.417068	0.654267	-0.2372	7	36068970	R	II		IGR	opensea	IGR-opense		rs5502660 49;49;48;39;14
cg1073305	0.243344	0.531905	13.9503	1.07E-06	0.000415	6.348951	0.410234	0.653577	-0.24334	6	34778879	F	II	UHRF1BP1	Body	opensea	Body-opens		rs5311480 0
cg1957621	0.214656	0.670249	13.94842	1.08E-06	0.000415	6.348	0.562921	0.777577	-0.21466	3	33402031	F	II	FBXL2	Body	opensea	Body-opens		
cg2009256	0.203036	0.654528	13.94601	1.08E-06	0.000415	6.346779	0.55301	0.756046	-0.20304	2	1.22E+08	R	II	GLI2	Body	opensea	Body-opens		rs1436221 40;1
cg1551248	0.228907	0.684617	13.94498	1.08E-06	0.000415	6.346259	0.570164	0.79907	-0.22891	2	1.11E+08	R	II	NPHP1	Body	opensea	Body-opens		rs5320925 2;1
cg0308879	0.230616	0.490577	13.94261	1.08E-06	0.000415	6.345062	0.375269	0.605885	-0.23062	1	2.2E+08	R	II		IGR	opensea	IGR-opense		rs5553475 19;1
cg0198081	0.228117	0.502375	13.94096	1.08E-06	0.000415	6.344227	0.388316	0.616433	-0.22812	4	1.13E+08	F	II		IGR	shore	IGR-shore	chr4:11320	rs1406720 27
cg1697338	0.227952	0.51834	13.93289	1.08E-06	0.000415	6.34014	0.404364	0.632317	-0.22795	1	1.55E+08	F	II	KCNN3	Body	opensea	Body-opens		
cg1325072	0.204198	0.596114	13.93154	1.09E-06	0.000415	6.339455	0.494015	0.698213	-0.2042	10	49490160	R	II		IGR	opensea	IGR-opense		rs5533160 1
cg0667186	0.226383	0.477071	13.92263	1.09E-06	0.000416	6.334938	0.36388	0.590263	-0.22638	3	99316428	R	II	MIR548G	Body	opensea	Body-opens		
cg0584843	0.236672	0.495095	13.91137	1.1E-06	0.000416	6.329228	0.376759	0.613431	-0.23667	4	1.54E+08	R	II		IGR	opensea	IGR-opense		
cg1445829	0.213766	0.518267	13.90825	1.1E-06	0.000416	6.327643	0.411384	0.62515	-0.21377	6	1.24E+08	R	II	NKAIN2	Body	opensea	Body-opens		
cg0487610	0.24269	0.505418	13.90027	1.1E-06	0.000417	6.323591	0.384073	0.626763	-0.24269	3	3551638	F	II		IGR	opensea	IGR-opense		rs5689076 0;28;34;40

cg1316611	0.266375	0.470414	13.89992	1.1E-06	0.000417	6.323414	0.337227	0.603602	-0.26637	3	1.14E+08	F	II		IGR	opensea	IGR-opense		rs5403381	12
cg2755241	0.260303	0.4552	13.89921	1.1E-06	0.000417	6.323057	0.325049	0.585352	-0.2603	16	79762853	R	II	MAFTRR	Body	opensea	Body-opens		rs5537948	49;41
cg1503290	0.220167	0.510675	13.89776	1.1E-06	0.000417	6.322316	0.400592	0.620758	-0.22017	5	1.71E+08	F	II	RANBP17	3'UTR	opensea	3'UTR-oper		rs5670221	0;38;39
cg1597620	0.227454	0.535075	13.89156	1.11E-06	0.000417	6.319167	0.421348	0.648802	-0.22745	13	94806595	R	II	GPC6-AS1	Body	opensea	Body-opens		rs5757770	1
cg0965274	0.209605	0.578958	13.88821	1.11E-06	0.000417	6.317461	0.474155	0.683761	-0.20961	16	67225437	F	II	E2F4	TSS1500	opensea	TSS1500-op		rs5731430	20
cg2355587	0.216437	0.5237	13.88212	1.11E-06	0.000418	6.314367	0.415482	0.631919	-0.21644	15	88990837	F	II		IGR	opensea	IGR-opense		rs5686454	22
cg1772837	0.223032	0.567453	13.87677	1.12E-06	0.000418	6.311646	0.455937	0.678969	-0.22303	21	27404776	F	II	APP	Body	opensea	Body-opens		rs5532821	48
cg0871767	0.225727	0.471253	13.87584	1.12E-06	0.000418	6.311173	0.35839	0.584117	-0.22573	14	1E+08	F	II	EML1	Body	opensea	Body-opens		rs1147682	43
cg1212603	0.263236	0.605583	13.87369	1.12E-06	0.000418	6.310075	0.473965	0.737201	-0.26324	20	33658033	F	II	TRPC4AP	Body	opensea	Body-opens		rs1841684	14;31
cg1572703	0.207847	0.667641	13.87081	1.12E-06	0.000419	6.308608	0.563718	0.771565	-0.20785	21	33020644	F	II		IGR	opensea	IGR-opense		rs5332796	12
cg0591920	0.216154	0.465117	13.86753	1.12E-06	0.000419	6.306938	0.35704	0.573195	-0.21615	4	5914761	R	II		IGR	opensea	IGR-opense		rs5412842	45
cg1757834	0.201562	0.58461	13.86177	1.13E-06	0.00042	6.304002	0.48383	0.685391	-0.20156	7	81420133	F	II		IGR	opensea	IGR-opense		rs5545652	27
cg1076797	0.201439	0.616507	13.8579	1.13E-06	0.00042	6.302031	0.515787	0.717227	-0.20144	13	45431256	F	II		IGR	opensea	IGR-opense		rs1836364	0;16
cg0742232	0.254952	0.583731	13.852	1.13E-06	0.00042	6.299023	0.456255	0.711207	-0.25495	1	1.87E+08	F	II	PTGS2	3'UTR	opensea	3'UTR-oper		rs1848452	0;1
cg1093052	0.206046	0.444686	13.84554	1.14E-06	0.000421	6.295729	0.341663	0.547709	-0.20605	1	7991549	F	II	TNFRSF9	Body	opensea	Body-opens		rs1390706	0;2
cg0905712	0.2148	0.61923	13.84179	1.14E-06	0.000421	6.293811	0.51183	0.72663	-0.2148	1	1E+08	R	II		IGR	opensea	IGR-opense		rs5737878	20
cg2475952	0.200703	0.502431	13.83998	1.14E-06	0.000421	6.29289	0.402079	0.602782	-0.2007	3	1.43E+08	F	II		IGR	opensea	IGR-opense		rs3711001	45
cg0757520	0.249438	0.465463	13.83386	1.14E-06	0.000422	6.289763	0.340744	0.590183	-0.24944	16	75046592	R	II	ZNRF1	Body	opensea	Body-opens		rs5499192	12;11;2;1
cg0765096	0.208104	0.636519	13.82926	1.15E-06	0.000422	6.287413	0.532468	0.740571	-0.2081	18	40883481	R	II		IGR	opensea	IGR-opense		rs1477321	50;49
cg2073479	0.237138	0.536578	13.82343	1.15E-06	0.000422	6.284431	0.418009	0.655147	-0.23714	7	40564790	F	II	SUGCT	Body	opensea	Body-opens		rs1831569	0;11
cg1945782	0.220222	0.654196	13.82222	1.15E-06	0.000422	6.283812	0.544085	0.764307	-0.22022	5	1.43E+08	R	II	NR3C1	Body	opensea	Body-opens		rs1411339	33;1
cg0411410	0.220965	0.44657	13.82194	1.15E-06	0.000422	6.283672	0.336087	0.557052	-0.22096	3	13339321	F	II		IGR	opensea	IGR-opense		rs6058874	0;4
cg0985234	0.260352	0.543889	13.8216	1.15E-06	0.000422	6.283497	0.413714	0.674065	-0.26035	6	1.12E+08	R	II	REV3L	Body	opensea	Body-opens		rs1860621	1
cg0426768	0.260856	0.584557	13.81315	1.16E-06	0.000423	6.279175	0.454129	0.714985	-0.26086	6	1.06E+08	F	II		IGR	opensea	IGR-opense		rs1866413	0;24
cg2688470	0.213581	0.543193	13.80381	1.16E-06	0.000424	6.274391	0.436402	0.649983	-0.21358	1	32378506	R	II	PTP4A2	Body	opensea	Body-opens			
cg0111221	0.240784	0.580178	13.79864	1.16E-06	0.000424	6.27174	0.459786	0.70057	-0.24078	14	89285377	R	II		IGR	opensea	IGR-opense		rs5735944	20
cg0010506	0.203828	0.616922	13.79614	1.17E-06	0.000424	6.270459	0.515008	0.718836	-0.20383	7	1.48E+08	R	II	CNTNAP2	Body	opensea	Body-opens		rs1390169	21;2
cg2613719	0.222169	0.53741	13.7955	1.17E-06	0.000424	6.270134	0.426326	0.648494	-0.22217	2	69453290	F	II	ANTXR1	Body	opensea	Body-opens		rs5643436	38
cg1935533	0.214595	0.497876	13.79438	1.17E-06	0.000424	6.269558	0.390578	0.605174	-0.2146	7	1.42E+08	R	II		IGR	opensea	IGR-opense			
cg1244328	0.217624	0.530959	13.79054	1.17E-06	0.000424	6.267589	0.422147	0.639771	-0.21762	11	1.11E+08	R	II		IGR	opensea	IGR-opense		rs5703954	14;11;5
cg2354888	0.225126	0.550119	13.78964	1.17E-06	0.000424	6.267126	0.437556	0.662682	-0.22513	4	1.24E+08	R	II		IGR	opensea	IGR-opense		rs5374667	2
cg1467645	0.224338	0.601477	13.78767	1.17E-06	0.000424	6.26612	0.489308	0.713646	-0.22434	4	1.59E+08	F	II		IGR	opensea	IGR-opense			
cg0667498	0.232802	0.480956	13.78765	1.17E-06	0.000424	6.266108	0.364555	0.597357	-0.2328	7	89251345	R	II		IGR	opensea	IGR-opense		rs7370923	2;1
cg0311642	0.207676	0.547633	13.78635	1.17E-06	0.000424	6.265441	0.443795	0.651471	-0.20768	4	14411456	R	II		IGR	opensea	IGR-opense		rs3683112	34;2;1
cg1443827	0.252234	0.492381	13.78605	1.17E-06	0.000424	6.265286	0.366264	0.618498	-0.25223	5	1.43E+08	F	II	NR3C1	5'UTR	opensea	5'UTR-oper			
cg2752944	0.202037	0.539118	13.78336	1.17E-06	0.000424	6.263908	0.438099	0.640136	-0.20204	6	33185552	R	II		IGR	opensea	IGR-opense		rs5502290	12
cg0866614	0.216527	0.583559	13.78205	1.17E-06	0.000424	6.263233	0.475295	0.691822	-0.21653	20	49952074	R	II		IGR	opensea	IGR-opense		rs5718245	20;6
cg0425806	0.243805	0.523244	13.78152	1.18E-06	0.000424	6.262964	0.401342	0.645147	-0.24381	4	1.75E+08	F	II		IGR	opensea	IGR-opense			
cg0067293	0.215273	0.635996	13.7737	1.18E-06	0.000424	6.258946	0.52836	0.743632	-0.21527	8	1.31E+08	F	II	CCDC26	Body	opensea	Body-opens		rs5291254	22;39
cg0636469	0.204191	0.682501	13.77351	1.18E-06	0.000424	6.25885	0.580406	0.784597	-0.20419	8	1.3E+08	F	II		IGR	opensea	IGR-opense		rs1488491	30
cg0759973	0.232528	0.520477	13.77295	1.18E-06	0.000424	6.258562	0.404213	0.636741	-0.23253	9	35929835	F	II		IGR	opensea	IGR-opense		rs5355350	6
cg2279714	0.216218	0.543516	13.76816	1.18E-06	0.000425	6.256099	0.435407	0.651626	-0.21622	6	88557431	F	II	LOC10192	Body	opensea	Body-opens		rs1896441	1;33
cg2735231	0.22602	0.497969	13.76628	1.18E-06	0.000425	6.255136	0.384959	0.610979	-0.22602	17	19453200	R	II	SLC47A1	Body	opensea	Body-opens		rs5718886	6;1
cg0857274	0.214645	0.627068	13.76376	1.19E-06	0.000425	6.253841	0.519746	0.734391	-0.21464	5	89664894	R	II		IGR	opensea	IGR-opense		rs1194810	23;1
cg0413740	0.213352	0.396057	13.76244	1.19E-06	0.000425	6.253162	0.289381	0.502733	-0.21335	14	62414451	R	II		IGR	opensea	IGR-opense		rs3750408	16
cg1576126	0.277283	0.498994	13.7566	1.19E-06	0.000426	6.250159	0.360352	0.637635	-0.27728	11	1.1E+08	F	II		IGR	opensea	IGR-opense		rs5636425	8;46
cg0111083	0.226334	0.53959	13.75403	1.19E-06	0.000426	6.248836	0.426423	0.652758	-0.22633	13	50383231	R	II		IGR	opensea	IGR-opense		rs1999409	27;1
cg2721143	0.238408	0.510816	13.74416	1.2E-06	0.000427	6.243753	0.391612	0.63002	-0.23841	15	71095215	F	II		IGR	opensea	IGR-opense		rs1114452	4
cg1828803	0.232932	0.604494	13.74322	1.2E-06	0.000427	6.24327	0.488028	0.72096	-0.23293	5	1.58E+08	F	II	EBF1	Body	opensea	Body-opens		rs5305717	0;22;30
cg2591888	0.239702	0.575784	13.73902	1.2E-06	0.000427	6.241107	0.455933	0.695635	-0.2397	17	36576950	R	II		IGR	shore	IGR-shore	chr17:365	rs5555333	10;1
cg0071851	0.233335	0.528769	13.73849	1.2E-06	0.000427	6.240834	0.412101	0.645437	-0.23334	2	89215272	R	II		IGR	opensea	IGR-opense		rs1458485	1
cg0930471	0.208045	0.565054	13.73756	1.2E-06	0.000427	6.240355	0.461031	0.669076	-0.20804	5	60712031	F	II	ZSWIM6	Body	opensea	Body-opens		rs5651363	16;43
cg0696885	0.227169	0.520935	13.73443	1.21E-06	0.000427	6.238742	0.407351	0.63452	-0.22717	2	80724209	F	II	CTNNA2	Body	opensea	Body-opens			
cg1148441	0.232363	0.505494	13.72984	1.21E-06	0.000427	6.236377	0.389312	0.621676	-0.23236	11	1.28E+08	F	II		IGR	opensea	IGR-opense		rs1465307	0;1;22
cg1043453	0.250224	0.572098	13.72907	1.21E-06	0.000427	6.23598	0.446986	0.69721	-0.25022	8	1.04E+08	R	II		IGR	opensea	IGR-opense		rs5329185	29
cg0840856	0.312624	0.518635	13.72859	1.21E-06	0.000427	6.235732	0.362323	0.674946	-0.31262	5	67067213	R	II		IGR	opensea	IGR-opense		rs5292816	45;2
cg0456297	0.205283	0.637178	13.72221	1.21E-06	0.000428	6.232443	0.534536	0.739819	-0.20528	16	30459538	R	II		IGR	shelf	IGR-shelf	chr16:304	rs5508574	45;31;9
cg2460310	0.233302	0.526795	13.72107	1.21E-06	0.000428	6.231855	0.410144	0.643446	-0.2333	8	1.19E+08	F	II	EXT1	Body	opensea	Body-opens		rs1923670	0;27;50
cg0633917	0.210223	0.441876	13.71896	1.22E-06	0.000428	6.230764	0.336765	0.546987	-0.21022	3	61873593	F	II	PTPRG	Body	opensea	Body-opens		rs1901962	1;47;47
cg2228244	0.200037	0.418663	13.7136	1.22E-06	0.000429	6.228	0.318645	0.518681	-0.20004	5	1.1E+08	F	II	TMEM232	Body	opensea	Body-opens		rs6237608	16;49;50
cg1812182	0.248303	0.485209	13.71225	1.22E-06	0.000429	6.227304	0.361058	0.60936	-0.2483	11	8705790	R	II	RPL27A	Body	shore	Body-shore	chr11:870	rs1496398	4
cg0550472	0.207917	0.508524	13.71089	1.22E-06	0.000429	6.226601	0.404565	0.612482	-0.20792	16	89399490	F	II	ANKRD11	5'UTR	opensea	5'UTR-oper			
cg1083838	0.267067	0.616002	13.70823	1.22E-06	0.000429	6.225224	0.482468	0.749535	-0.26707	10	1156113	R	I	WDR37	Body	opensea	Body-opens		rs5400188	35;6
cg1131562	0.259761	0.492516	13.69879	1.23E-06	0.00043	6.220347	0.362635	0.622396	-0.25976	17	57829517	R	II	VMP1	Body	opensea	Body-opens		rs5682089	49;34;15;1
cg0580948	0.214887	0.625534	13.69675	1.23E-06	0.00043	6.219291	0.518091	0.732978	-0.21489	1	1.57E+08	F	II	ISG20L2	Body	shore	Body-shore	chr1:1566	rs1823711	5;25
cg0236508	0.221732	0.57585	13.69474	1.23E-06	0.00043	6.218255	0.464984	0.686716	-0.22173	1	27701171	R	II	FCN3	Body	opensea	Body-opens		rs5465843	8;7;1
cg2555650	0.209453	0.480046	13.68973	1.23E-06	0.00043	6.215662	0.375319	0.584772	-0.20945	13	25766431	F	II		IGR	opensea	IGR-opense			
cg0694330	0.227734	0.578557	13.68764	1.24E-06	0.00043	6.214582	0.46469	0.692424	-0.22773	1	1.63E+08	R	II		IGR	shore	IGR-shore	chr1:1633	rs5583261	45
cg0784606	0.257307	0.469349	13.68719	1.24E-06	0.00043	6.214346	0.340695	0.598002	-0.25731	16	74732434	F	II	MLKL	5'UTR	shore	5'UTR-shor	chr16:747	rs1179046	4
cg0010015	0.212025	0.581349	13.68645	1.24E-06	0.00043	6.213968	0.475336	0.687361	-0.21202	5	42873127	F	II		IGR	opensea	IGR-opense		rs5708244	11;47;48
cg1696029	0.241728	0.483807	13.68493	1.24E-06	0.00043	6.213179	0.362943	0.604671	-0.24173	7	50663419	F	II	GRB10	Body	opensea	Body-opens		rs1155562	26
cg0166972	0.231676	0.441046	13.68488	1.24E-06	0.00043	6.213153	0.325208	0.556884	-0.23168	14	55155256	R	II	SAMD4A	Body	opensea	Body-opens		rs5678832	39;11;2
cg0974327	0.200397	0.547188	13.68084	1.24E-06	0.000431	6.211062	0.44699	0.647387	-0.2004	1	1.13E+08	R	II		IGR	opensea	IGR-opense		rs5475963	46
cg2733813	0.20267	0.431439	13.68033	1.24E-06	0.000431	6.210796	0.330103	0.532774	-0.20267	2	95939667	R	II	PROM2	TSS1500	opensea	TSS1500-op		rs5514338	1
cg1412218	0.25646	0.600507	13.67575	1.24E-06	0.000431	6.208425	0.472276	0.728737	-0.25646	8	1.3E+08	F	II		IGR	opensea	IGR-opense		rs5596979	1;5;44
cg2671266	0.237293	0.434708	13.67513	1.24E-06	0.000431	6.208105	0.316062	0.553355	-0.23729	11	1.21E+08	R	II	TECTA	Body	opensea	Body-opens		rs5503063	39;29;17;1
cg2237217	0.262212	0.525957	13.67438	1.24E-06	0.000431	6.207716	0.39485	0.657063	-0.26221	20	24941563	F	II		IGR	opensea	IGR-opense		rs1466871	0;42
cg2593923	0.223118	0.460782	13.67383	1.24E-06	0.000431	6.20743	0.349223	0.572342	-0.22312	18	6967140	R	II	LAMA1	Body	opensea	Body-opens			
cg1631831	0.237884	0.603669	13.65998	1.25E-06	0.000432	6.200249	0.484727	0.722611	-0.23788	12	96113161	R	II	NTN4	Body	opensea	Body-opens			
cg1757944	0.214826	0.525575	13.6577	1.26E-06	0.000432	6.19907	0.418162	0.632988	-0.21483	17	7217367	R	II	GPS2	Body	shore	Body-shore	chr17:721	rs2015341	47;29;27;4;2
cg2713620	0.234686	0.52731	13.65706	1.26E-06	0.000432	6.198736	0.409966	0.644653	-0.23469	1	1.94E+08	R	II		IGR	opensea	IGR-opense		rs1501856	45;44;34;30
cg2319552	0.212044	0.553076	13.64541	1.26E-06	0.000434	6.192691	0.447054	0.659099	-0.21204	11	85775563	F	II	PICALM	5'UTR	opensea	5'UTR-oper		rs1169264	20;26;40
cg1411779	0.230062	0.548974	13.64298	1.27E-06	0.000434	6.191426	0.433942	0.664005	-0.23006	7	1.21E+08	R	II	CPED1	Body	opensea	Body-opens		rs5315564	43;21
cg0088332	0.222037	0.454325	13.64192	1.27E-06	0.000434	6.190877	0.343306	0.565343	-0.22204	17	33204221	F	II		IGR	opensea	IGR-opense			
cg2347390	0.210514	0.514518	13.64038	1.27E-06	0.000434	6.190073	0.409261	0.619775	-0.21051	6	3523483	F	II		IGR	opensea	IGR-opense		rs5736365	0;30;48
cg2404408	0.202608	0.646573	13.63904	1.27E-06	0.000434	6.189381	0.545269	0.747877	-0.20261	17	41930333	F	II	CD300LG	Body	opensea	Body-opens		rs1460424	0;1;40
cg0694067	0.210721	0.512227	13.63835	1.27E-06	0.000434	6.189021	0.406867	0.617587	-0.21072	20	4528299	F	II		IGR	opensea	IGR-opense		rs5712967	46;47
cg1124367	0.222612	0.621539	13.63596	1.27E-06	0.000434	6.18778	0.510233	0.732845	-0.22261	5	1.65E+08	F	II		IGR	opensea	IGR-opense		rs1820373	0
cg1866188	0.269048	0.55146	13.63594	1.27E-06	0.000434	6.187767	0.416936	0.685984	-0.26905	4	833876	R	II		IGR	opensea	IGR-opense		rs5406829	24;1
cg2363851	0.256471	0.515808	13.63475	1.27E-06	0.000434	6.187149	0.387572	0.644044	-0.25647	6	39080067	F	II	C6orf64	Body	shelf	Body-shelf	chr6:3908	rs1415820	15;32;49
cg0950104	0.242218	0.550152	13.63421	1.27E-06	0.000434	6.18687	0.429043	0.671261	-0.24222	1	1.65E+08	R	II	PBX1	Body	opensea	Body-opens		rs1897560	18
cg0216474	0.265839	0.407002	13.63265	1.27E-06	0.000434	6.186056	0.274082	0.539921	-0.26584	6	76206048	F	II		IGR	opensea	IGR-opense		rs1172187	1
cg0234365	0.282126	0.528344	13.62866	1.28E-06	0.000435	6.18398	0.387281	0.669407	-0.28213	21	30476730	R	II	MAP3K7CL	5'UTR	opensea	5'UTR-oper			
cg0735311	0.236556	0.492349	13.62834	1.28E-06	0.000435	6.183816	0.374072	0.610627	-0.23656	8	29028701	R	II	KIF13B	Body	opensea	Body-opens		rs5781544	34;26;23;1
cg1959048	0.211253	0.427659	13.62783	1.28E-06	0.000435	6.183549	0.322032	0.533286	-0.21125	19	36623725	R	II		IGR	opensea	IGR-opense		rs5692523	29;1
cg0062543	0.264275	0.530272	13.62629	1.28E-06	0.000435	6.182748	0.398134	0.66241	-0.26428	11	34706961	R	II		IGR	opensea	IGR-opense		rs5525057	48;42;1
cg2143825	0.211104	0.733011	13.62487	1.28E-06	0.000435	6.182012	0.627459	0.838563	-0.2111	1	28904846	F	II	TRNAU1AP	Body	shelf	Body-shelf	chr1:2890	rs5289206	40;47;50
cg1458016	0.254872	0.578689	13.62447	1.28E-06	0.000435	6.181801	0.451253	0.706125	-0.25487	12	26286940	R	II		IGR	opensea	IGR-opense			
cg0016767	0.222373	0.496358	13.62386	1.28E-06	0.000435	6.181483	0.385172	0.607544	-0.22237	4	13922944	R	II		IGR	opensea	IGR-opense		rs7379853	51;2
cg0655191	0.22081	0.58338	13.61825	1.28E-06	0.000436	6.178566	0.472975	0.693785	-0.22081	4	1.09E+08	F	II	SGMS2	5'UTR	opensea	5'UTR-oper		rs1840247	35
cg0554769	0.212266	0.562733	13.61736	1.28E-06	0.000436	6.178101	0.4566	0.668866	-0.21227	9	1.07E+08	F	II		IGR	opensea	IGR-opense		rs1901437	1;7;27
cg1073741	0.230586	0.533863	13.61665	1.28E-06	0.000436	6.177733	0.41857	0.649156	-0.23059	15	34190524	F	II	AVEN	Body	opensea	Body-opens		rs1148690	0;25;34
cg2047008	0.211438	0.519901	13.61225	1.29E-06	0.000436	6.175439	0.414183	0.62562	-0.21144	12	1.13E+08	R	II	MIR1302-1	TSS1500	opensea	TSS1500-op			
cg0563820	0.216701	0.486403	13.61128	1.29E-06	0.000436	6.174933	0.378053	0.594753	-0.2167	5	41372068	R	II	PLCXD3	Body	opensea	Body-opens			
cg1525389	0.21243	0.411908	13.6096	1.29E-06	0.000436	6.174059	0.305693	0.518123	-0.21243	6	1.51E+08	F	II	MTHFD1L	Body	opensea	Body-opens			
cg1036884	0.201963	0.54077	13.60948	1.29E-06	0.000436	6.173997	0.439788	0.641751	-0.20196	10	1.16E+08	F	II	PLEKHS1	1stExon	opensea	1stExon-op		rs1900998	4;7;24;35;46;50
cg1329062	0.235866	0.661923	13.60292	1.29E-06	0.000436	6.170578	0.54399	0.779856	-0.23587	15	68840772	R	II		IGR	opensea	IGR-opense		rs5448290	37;2;1
cg0941579	0.245598	0.483545	13.60091	1.29E-06	0.000436	6.169529	0.360747	0.606344	-0.2456	3	23800187	F	II		IGR	opensea	IGR-opense		rs5727585	4;17
cg2608180	0.227041	0.571841	13.59543	1.3E-06	0.000436	6.16667	0.45832	0.685361	-0.22704	3	1.01E+08	R	II	ABI3BP	Body	opensea	Body-opens		rs7761511	17;6;2;1
cg1199606	0.220743	0.67621	13.59346	1.3E-06	0.000436	6.165646	0.565839	0.786582	-0.22074	9	93635561	R	II	SYK	Body	opensea	Body-opens		rs3749133	1
cg2722690	0.246651	0.653204	13.58746	1.3E-06	0.000436	6.162512	0.529879	0.77653	-0.24665	4	1.25E+08	R	II		IGR	opensea	IGR-opense		rs1405181	22;1
cg1391263	0.217773	0.6176	13.58523	1.31E-06	0.000436	6.161348	0.508714	0.726487	-0.21777	5	1.48E+08	F	II		IGR	opensea	IGR-opense		rs1383841	12
cg1463790	0.218021	0.485569	13.58411	1.31E-06	0.000436	6.160766	0.376559	0.59458	-0.21802	10	1.04E+08	F	II	C10orf76	Body	opensea	Body-opens		rs1872004	18;28;41
cg1176439	0.230789	0.569399	13.57649	1.31E-06	0.000438	6.156782	0.454005	0.684794	-0.23079	21	47864791	F	II	PCNT	Body	opensea	Body-opens		rs1512361	0;4;22;25;32;47
cg2591613	0.222756	0.43127	13.57167	1.32E-06	0.000438	6.154266	0.319892	0.542648	-0.22276	2	59071761	F	II		IGR	opensea	IGR-opense		rs5364716	0;24;47
cg0777642	0.210141	0.533868	13.5704	1.32E-06	0.000438	6.1536	0.428798	0.638939	-0.21014	10	5713058	R	II		IGR	opensea	IGR-opense		rs5572781	42;2;1
cg0702868	0.254747	0.603784	13.56991	1.32E-06	0.000438	6.153346	0.47641	0.731157	-0.25475	6	1.14E+08	F	II	LOC10192	Body	opensea	Body-opens		rs1427248	26;47
cg1850601	0.204577	0.589363	13.5576	1.33E-06	0.000439	6.146903	0.487074	0.691651	-0.20458	19	44285974	F	II	KCNN4	TSS1500	opensea	TSS1500-op		rs5745012	5;13;27;31
cg0842866	0.210945	0.44033	13.55694	1.33E-06	0.000439	6.146562	0.334857	0.545802	-0.21094	16	28011265	F	II	GSG1L	Body	opensea	Body-opens		rs1424953	0;8

cg1230507	0.220054	0.516792	13.55332	1.33E-06	0.00044	6.144664	0.406765	0.626819	-0.22005	7	78368756	F	II	MAGI2	Body	opensea	Body-opens		rs5655999	43
cg1646195	0.201971	0.5412	13.55001	1.33E-06	0.00044	6.142933	0.440214	0.642185	-0.20197	16	58936076	F	II		IGR	opensea	IGR-opense		rs1897237	0
cg2469968	0.25952	0.610765	13.54955	1.33E-06	0.00044	6.142689	0.481005	0.740525	-0.25952	1	95268325	F	II		IGR	opensea	IGR-opense			
cg1693496	0.252705	0.451735	13.54887	1.33E-06	0.00044	6.142335	0.325383	0.578088	-0.2527	2	1.74E+08	R	II	RAPGEF4	Body	opensea	Body-opens		rs1851433	36
cg0734841	0.23723	0.567626	13.53714	1.34E-06	0.000442	6.136186	0.449011	0.686241	-0.23723	1	44903330	F	II	RNF220	Body	opensea	Body-opens		rs5603200	17;50
cg2423267	0.204395	0.630394	13.53617	1.34E-06	0.000442	6.135678	0.528196	0.732591	-0.20439	1	2.19E+08	R	II	LYPLAL1-AS	Body	opensea	Body-opens		rs5563090	25;2;1
cg1689009	0.207714	0.497215	13.53577	1.34E-06	0.000442	6.135466	0.393358	0.601072	-0.20771	13	96162551	R	II	CLDN10-AS	Body	opensea	Body-opens		rs1487420	26
cg2525296	0.252045	0.489168	13.52832	1.35E-06	0.000442	6.13156	0.363146	0.615191	-0.25204	12	8695028	F	II	CLEC4E	TSS1500	opensea	TSS1500-op			
cg1371996	0.213052	0.36382	13.52378	1.35E-06	0.000443	6.129175	0.257294	0.470346	-0.21305	8	1.34E+08	R	II	MIR7848	TSS200	opensea	TSS200-ope		rs1126474	42;2
cg1083408	0.223214	0.568885	13.52058	1.35E-06	0.000443	6.127499	0.457278	0.680492	-0.22321	15	80500594	R	II		IGR	opensea	IGR-opense		rs5698519	29;2;1
cg2406430	0.203721	0.574889	13.51431	1.36E-06	0.000444	6.124202	0.473029	0.676749	-0.20372	8	1.02E+08	F	II		IGR	opensea	IGR-opense		rs5681487	0;2
cg1900584	0.2177	0.476601	13.51047	1.36E-06	0.000444	6.122183	0.367751	0.585451	-0.2177	6	29399774	R	II		IGR	opensea	IGR-opense			
cg1705548	0.215227	0.44791	13.50868	1.36E-06	0.000444	6.121243	0.340296	0.555523	-0.21523	6	1.44E+08	R	II	LOC28574(	Body	opensea	Body-opens		rs1461213	1
cg0887749	0.229285	0.620075	13.50696	1.36E-06	0.000445	6.120343	0.505433	0.734718	-0.22928	8	77895589	R	II	PEX2	Body	opensea	Body-opens			
cg2659627	0.218571	0.426805	13.5047	1.36E-06	0.000445	6.119151	0.317519	0.53609	-0.21857	1	2.06E+08	R	II	AVPR1B	Body	shelf	Body-shelf	chr1:2062:	rs5333455	10
cg0868389	0.215698	0.588581	13.49653	1.37E-06	0.000445	6.114854	0.480732	0.69643	-0.2157	12	1.31E+08	R	II		IGR	opensea	IGR-opense		rs1163038	2
cg1557429	0.257351	0.488898	13.49567	1.37E-06	0.000445	6.1144	0.360223	0.617573	-0.25735	7	25590861	F	II		IGR	opensea	IGR-opense		rs1778270	29;47
cg2684898	0.236932	0.537667	13.49119	1.37E-06	0.000445	6.112044	0.419201	0.656133	-0.23693	1	1.86E+08	F	II	HMCN1	Body	opensea	Body-opens		rs1401494	0;31
cg2191019	0.20868	0.559592	13.49079	1.37E-06	0.000445	6.111834	0.455252	0.663932	-0.20868	14	72402619	F	II	RGS6	5'UTR	shelf	5'UTR-shelf	chr14:723	rs1995191	40
cg1984122	0.259673	0.55996	13.48428	1.38E-06	0.000446	6.108404	0.430123	0.689796	-0.25967	6	1.12E+08	F	II	FYN	5'UTR	opensea	5'UTR-oper		rs7563665	49
cg0325433	0.227427	0.529957	13.48227	1.38E-06	0.000446	6.107345	0.416244	0.643671	-0.22743	10	1.15E+08	F	II		IGR	opensea	IGR-opense		rs5484135	6
cg0507224	0.236789	0.626009	13.4808	1.38E-06	0.000446	6.106571	0.507615	0.744404	-0.23679	6	27781799	R	II	HIST1H2BN	TSS1500	shore	TSS1500-sh	chr6:2778:	rs3709405	14;1
cg2659144	0.208586	0.484437	13.47721	1.38E-06	0.000446	6.104681	0.380144	0.588729	-0.20859	10	61366602	F	II		IGR	opensea	IGR-opense		rs1903495	23
cg0775890	0.210376	0.491636	13.47021	1.39E-06	0.000447	6.100987	0.386448	0.596824	-0.21038	15	31523838	F	II	LOC28371(	TSS1500	opensea	TSS1500-op		rs3736801	0
cg2709776	0.254997	0.514017	13.46989	1.39E-06	0.000447	6.10082	0.386518	0.641515	-0.255	14	21923087	R	II		IGR	shore	IGR-shore	chr14:219:	rs7912057	9
cg1035214	0.207601	0.502354	13.46978	1.39E-06	0.000447	6.100762	0.398554	0.606154	-0.2076	18	57052410	R	II		IGR	opensea	IGR-opense		rs5693219	1
cg1356446	0.250773	0.615126	13.46372	1.39E-06	0.000448	6.097563	0.489739	0.740512	-0.25077	3	1.86E+08	R	II	LOC25357:	Body	opensea	Body-opens		rs1164472	9;2
cg1060127	0.216797	0.487985	13.46255	1.4E-06	0.000448	6.096947	0.379587	0.596384	-0.2168	1	1.1E+08	R	II		IGR	opensea	IGR-opense		rs1869738	46
cg2089747	0.214675	0.559167	13.4598	1.4E-06	0.000448	6.095495	0.451829	0.666504	-0.21468	11	12292577	R	II		IGR	opensea	IGR-opense		rs1388526	38;2
cg2437971	0.239192	0.570817	13.45805	1.4E-06	0.000448	6.094572	0.451221	0.690413	-0.23919	6	1.41E+08	F	II		IGR	opensea	IGR-opense		rs5541648	1;8;9;10;45
cg2622431	0.230055	0.556318	13.45736	1.4E-06	0.000448	6.094207	0.441291	0.671345	-0.23005	13	81746461	F	II		IGR	opensea	IGR-opense		rs1386506	19;25
cg0207074	0.206285	0.681744	13.45511	1.4E-06	0.000448	6.093016	0.578602	0.784887	-0.20628	1	1.47E+08	R	II	CHD1L	Body	opensea	Body-opens		rs3753423	48;22;5;2;1
cg0059709	0.210508	0.490362	13.44687	1.41E-06	0.000449	6.088665	0.385108	0.595616	-0.21051	16	76839538	R	II		IGR	opensea	IGR-opense		rs1902602	34;25;20;11
cg1533448	0.221847	0.536485	13.44654	1.41E-06	0.000449	6.088493	0.425562	0.647409	-0.22185	1	64578142	F	II	ROR1-AS1	Body	opensea	Body-opens			
cg2078216	0.222548	0.559316	13.43989	1.41E-06	0.000449	6.084976	0.448041	0.67059	-0.22255	4	1.58E+08	R	II		IGR	opensea	IGR-opense		rs1919654	24
cg1263923	0.209508	0.591507	13.43871	1.41E-06	0.000449	6.08435	0.486753	0.696261	-0.20951	8	18248964	F	II	NAT2	5'UTR	shelf	5'UTR-shelf	chr8:1824	rs5423625	0;18;32
cg1297559	0.24092	0.622552	13.43817	1.41E-06	0.000449	6.084067	0.502092	0.743012	-0.24092	6	22897079	F	II		IGR	opensea	IGR-opense		rs5654362	0
cg2330542	0.262959	0.449821	13.43571	1.42E-06	0.000449	6.082764	0.318341	0.5813	-0.26296	11	1.1E+08	R	II		IGR	opensea	IGR-opense		rs5731021	51;32;31;2
cg1596231	0.215245	0.576248	13.42032	1.43E-06	0.000451	6.074615	0.468626	0.683871	-0.21525	11	1.27E+08	R	II	KIRREL3	Body	opensea	Body-opens		rs1473837	9;2
cg1419104	0.20868	0.571739	13.41771	1.43E-06	0.000451	6.07323	0.467399	0.676078	-0.20868	10	96745681	R	II	CYP2C9	Body	opensea	Body-opens		rs1834552	50;42;35;2
cg1138195	0.233401	0.534879	13.41644	1.43E-06	0.000451	6.072558	0.418179	0.65158	-0.2334	4	1.02E+08	F	II	PPP3CA	Body	opensea	Body-opens		rs5776410	3;35
cg0519850	0.226204	0.419849	13.41184	1.43E-06	0.000451	6.070119	0.306747	0.532951	-0.2262	5	1.39E+08	R	II	SPATA24	Body	opensea	Body-opens		rs7891733	37;25;1
cg0801791	0.271808	0.682409	13.41178	1.44E-06	0.000451	6.070088	0.546505	0.818313	-0.27181	4	84526934	R	II	AGPAT9	3'UTR	opensea	3'UTR-oper			
cg0215619	0.212458	0.621279	13.41058	1.44E-06	0.000451	6.069453	0.51505	0.727509	-0.21246	20	11668904	F	II		IGR	shore	IGR-shore	chr20:116(	rs5319090	28
cg2396941	0.20223	0.414566	13.40913	1.44E-06	0.000451	6.068682	0.313451	0.51568	-0.20223	2	5313194	R	II		IGR	opensea	IGR-opense		rs1404616	51;28;2
cg1677277	0.204538	0.455498	13.40812	1.44E-06	0.000451	6.06815	0.353229	0.557767	-0.20454	14	62721064	R	II		IGR	opensea	IGR-opense		rs5579411	23;11;1
cg0742919	0.212529	0.523374	13.40795	1.44E-06	0.000451	6.068056	0.41711	0.629639	-0.21253	5	9918811	F	II		IGR	opensea	IGR-opense			
cg0802385	0.210193	0.513853	13.40694	1.44E-06	0.000451	6.067522	0.408757	0.61895	-0.21019	3	65864381	F	II	MAGI1	Body	opensea	Body-opens		rs1488223	45;46
cg0342632	0.20307	0.490705	13.40655	1.44E-06	0.000451	6.067318	0.38917	0.59224	-0.20307	21	39627382	R	II	KCNJ15	TSS1500	opensea	TSS1500-op		rs7654592	7
cg0405506	0.223944	0.542202	13.40083	1.44E-06	0.000451	6.064279	0.43023	0.654174	-0.22394	3	25382277	F	II	RARB	TSS200	opensea	TSS200-ope		rs1459922	25
cg1826409	0.200464	0.518544	13.40069	1.44E-06	0.000451	6.064209	0.418312	0.618776	-0.20046	10	6183575	F	II		IGR	shelf	IGR-shelf	chr10:618(	rs7998593	0;17;28
cg0378798	0.200361	0.592833	13.39985	1.44E-06	0.000451	6.063761	0.492653	0.693013	-0.20036	11	10602522	F	II	MRVI1	Body	opensea	Body-opens		rs5780562	2;48
cg2758254	0.213953	0.541938	13.39581	1.45E-06	0.000451	6.061614	0.434961	0.648914	-0.21395	1	1062237	R	II		IGR	opensea	IGR-opense		rs5771966	32
cg1494799	0.204792	0.492289	13.39448	1.45E-06	0.000451	6.06091	0.389893	0.594685	-0.20479	6	1.07E+08	R	II		IGR	opensea	IGR-opense		rs8029972	18
cg1596769	0.23087	0.53039	13.39418	1.45E-06	0.000451	6.060753	0.414955	0.645825	-0.23087	14	1.01E+08	F	II	SNHG24	Body	opensea	Body-opens			
cg2281371	0.213426	0.504566	13.38824	1.45E-06	0.000452	6.057596	0.397853	0.611279	-0.21343	1	87595171	F	II	LOC33952	TSS1500	shelf	TSS1500-sh	chr1:8759:	rs1447539	36;42
cg0488447	0.220949	0.476628	13.38748	1.45E-06	0.000452	6.05719	0.366153	0.587102	-0.22095	17	60266252	F	II		IGR	opensea	IGR-opense		rs1852967	1
cg0933585	0.246108	0.47597	13.38578	1.46E-06	0.000452	6.056288	0.352916	0.599024	-0.24611	3	5307317	R	II		IGR	opensea	IGR-opense		rs5388905	1
cg0298628	0.223077	0.689451	13.38483	1.46E-06	0.000452	6.055786	0.577913	0.80099	-0.22308	11	2798509	R	II	KCNQ1	Body	opensea	Body-opens			
cg0191966	0.202521	0.596485	13.38436	1.46E-06	0.000452	6.055534	0.495224	0.697745	-0.20252	11	62782872	R	II	SLC22A8	5'UTR	opensea	5'UTR-oper			
cg0319103	0.223938	0.592634	13.38296	1.46E-06	0.000452	6.054789	0.480665	0.704603	-0.22394	3	1.86E+08	R	II		IGR	opensea	IGR-opense		rs1141473	35
cg0271199	0.266039	0.508263	13.38251	1.46E-06	0.000452	6.05455	0.375243	0.641283	-0.26604	4	70716021	F	II	SULT1E1	Body	opensea	Body-opens		rs1157377	1;48
cg0805005	0.205091	0.572512	13.37889	1.46E-06	0.000452	6.052628	0.469967	0.675057	-0.20509	3	1.06E+08	R	II		IGR	opensea	IGR-opense		rs340058;r	48;36;31;20;10
cg1262485	0.221326	0.598949	13.3713	1.47E-06	0.000454	6.048591	0.488286	0.709612	-0.22133	19	16231013	R	II	RAB8A	Body	opensea	Body-opens		rs5691306	9;2
cg2098666	0.223	0.474908	13.36761	1.47E-06	0.000454	6.046628	0.363408	0.586408	-0.223	1	22552245	R	II		IGR	opensea	IGR-opense		rs1414776	38
cg1615890	0.296041	0.542502	13.36592	1.47E-06	0.000454	6.045726	0.394481	0.690522	-0.29604	7	1.14E+08	R	II		IGR	opensea	IGR-opense		rs1416447	24;2
cg0567350	0.258601	0.560047	13.36233	1.47E-06	0.000455	6.043813	0.430747	0.689348	-0.2586	14	97176849	F	II		IGR	opensea	IGR-opense			
cg1563945	0.202062	0.554342	13.36191	1.47E-06	0.000455	6.04359	0.453311	0.655373	-0.20206	9	19377072	R	II	RPS6	Body	shelf	Body-shelf	chr9:1937	rs5386515	33;28
cg2299401	0.236348	0.60723	13.3601	1.48E-06	0.000455	6.042626	0.489056	0.725403	-0.23635	11	10044327	F	II	SBF2	Body	opensea	Body-opens		rs5703060	0;1
cg0698629	0.244923	0.52428	13.35865	1.48E-06	0.000455	6.041855	0.401818	0.646741	-0.24492	8	1.31E+08	R	II	CCDC26	Body	opensea	Body-opens		rs1881361	46
cg0049637	0.210319	0.499815	13.35548	1.48E-06	0.000455	6.040166	0.394656	0.604975	-0.21032	1	2.21E+08	F	II		IGR	opensea	IGR-opense			
cg1752905	0.222582	0.490984	13.35541	1.48E-06	0.000455	6.040131	0.379693	0.602275	-0.22258	17	72886179	R	II	FADS6	Body	shelf	Body-shelf	chr17:7288	rs5369868	39;2
cg1876334	0.222744	0.476479	13.35273	1.48E-06	0.000455	6.0387	0.365107	0.587852	-0.22274	2	1.93E+08	F	II		IGR	opensea	IGR-opense		rs5723109	0;11;33;43;47
cg0181867	0.241413	0.591411	13.34874	1.49E-06	0.000456	6.036572	0.470705	0.712117	-0.24141	4	8257758	R	II		IGR	opensea	IGR-opense		rs1313854	48;1
cg0363445	0.218189	0.453791	13.34595	1.49E-06	0.000456	6.035085	0.344696	0.562885	-0.21819	15	71626380	F	II	THSD4	Body	opensea	Body-opens		rs5602230	1;5;9
cg0296589	0.213516	0.455633	13.34269	1.49E-06	0.000456	6.033345	0.348875	0.562391	-0.21352	11	5020172	F	II	OR51L1	TSS200	opensea	TSS200-ope		rs1418062	6;17
cg2621968	0.230914	0.465424	13.34139	1.49E-06	0.000456	6.032652	0.349967	0.580881	-0.23091	2	58568596	F	II		IGR	opensea	IGR-opense		rs1812388	8;15;29
cg1578234	0.226855	0.484	13.33814	1.49E-06	0.000456	6.03092	0.370572	0.597428	-0.22686	2	69016925	R	II	ARHGAP25	Body	opensea	Body-opens		rs5694987	27
cg0366689	0.28318	0.613098	13.33341	1.5E-06	0.000456	6.028393	0.471508	0.754688	-0.28318	11	76847277	R	II	MYO7A	Body	shore	Body-shore	chr11:768	rs1841666	37
cg1111414	0.229882	0.524354	13.33103	1.5E-06	0.000456	6.027124	0.409413	0.639295	-0.22988	2	2.07E+08	F	II	NRP2	Body	opensea	Body-opens		rs7972957	46;50
cg1725378	0.2515	0.635624	13.3244	1.51E-06	0.000456	6.023585	0.509874	0.761374	-0.2515	8	67587258	F	II	C8orf44	TSS1500	opensea	TSS1500-op		rs5315788	0;1;3;9
cg0174076	0.248058	0.634788	13.32387	1.51E-06	0.000456	6.023298	0.510759	0.758817	-0.24806	3	15541901	R	II	COLQ	Body	opensea	Body-opens			
cg1465512	0.201339	0.660634	13.31462	1.51E-06	0.000457	6.018353	0.559964	0.761304	-0.20134	1	21970665	R	II	RAP1GAP	Body	opensea	Body-opens		rs5277795	49;35;25;23;1
cg1769193	0.229884	0.507255	13.3143	1.51E-06	0.000457	6.018185	0.392313	0.622197	-0.22988	7	48568677	R	II	ABCA13	Body	opensea	Body-opens		rs1842984	46;18;15;2;1
cg2382102	0.283746	0.482484	13.31254	1.52E-06	0.000457	6.017241	0.340611	0.624357	-0.28375	13	99962835	F	II	UBAC2	Body	opensea	Body-opens			
cg0363480	0.226669	0.567069	13.30594	1.52E-06	0.000458	6.013713	0.453735	0.680404	-0.22667	18	54777662	F	II		IGR	opensea	IGR-opense		rs5771064	37
cg0293509	0.213571	0.579609	13.30427	1.52E-06	0.000458	6.012819	0.472824	0.686394	-0.21357	1	92414782	F	II	BRDT	TSS200	island	TSS200-isla	chr1:9241	rs1806778	0;25;41;49
cg2684911	0.236712	0.542983	13.30287	1.52E-06	0.000458	6.012066	0.424627	0.661339	-0.23671	3	1.24E+08	F	II	KALRN	3'UTR	opensea	3'UTR-oper		rs1906726	0;1;25
cg0084995	0.215293	0.613398	13.30271	1.52E-06	0.000458	6.011982	0.505751	0.721045	-0.21529	6	1.12E+08	F	II	FYN	5'UTR	opensea	5'UTR-oper		rs5778525	15
cg0846984	0.204579	0.539783	13.30104	1.52E-06	0.000458	6.01109	0.437494	0.642073	-0.20458	17	39676096	R	II	KRT15	TSS1500	opensea	TSS1500-op		rs5767407	47;40;10;7
cg1899189	0.239014	0.539423	13.30094	1.53E-06	0.000458	6.011034	0.419916	0.65893	-0.23901	3	1.23E+08	F	II	SEMA5B	Body	opensea	Body-opens		rs5280798	0;10;14;22
cg2610446	0.200168	0.557205	13.3	1.53E-06	0.000458	6.010533	0.457121	0.65729	-0.20017	9	1.13E+08	F	II		IGR	opensea	IGR-opense		rs1145435	1;23
cg1168652	0.206574	0.435231	13.29874	1.53E-06	0.000458	6.009855	0.331944	0.538518	-0.20657	5	24554038	F	II	CDH10	Body	opensea	Body-opens		rs1414052	9;11
cg0764559	0.214976	0.472442	13.2986	1.53E-06	0.000458	6.009783	0.364954	0.579929	-0.21498	2	1.55E+08	R	II	LOC10014	TSS1500	opensea	TSS1500-op		rs5287913	34
cg1588328	0.21805	0.498781	13.2968	1.53E-06	0.000458	6.008817	0.389757	0.607806	-0.21805	14	85853554	R	II		IGR	opensea	IGR-opense		rs1459251	4
cg0745449	0.230434	0.600003	13.29439	1.53E-06	0.000458	6.007527	0.484786	0.71522	-0.23043	6	31665801	F	II	ABHD16A	Body	opensea	Body-opens		rs5301782	3
cg1869174	0.216972	0.550928	13.29091	1.53E-06	0.000459	6.005663	0.442443	0.659414	-0.21697	9	83264554	R	II		IGR	opensea	IGR-opense		rs1461765	13;9
cg1392620	0.223348	0.478049	13.2838	1.54E-06	0.00046	6.001851	0.366375	0.589724	-0.22335	4	15239597	R	II	LOC10192	Body	opensea	Body-opens		rs1835845	40;6
cg1593690	0.242758	0.569355	13.2806	1.54E-06	0.00046	6.000136	0.447976	0.690734	-0.24276	17	30024653	F	II		IGR	opensea	IGR-opense		rs1413264	31
cg1955303	0.24754	0.544674	13.27856	1.54E-06	0.000461	5.999042	0.420904	0.668444	-0.24754	4	26196680	F	II		IGR	opensea	IGR-opense		rs5390600	0;1;10;48
cg0701959	0.275544	0.460061	13.2773	1.55E-06	0.000461	5.998366	0.322289	0.597834	-0.27554	17	34207481	R	II	CCL5	TSS200	opensea	TSS200-ope		rs5359131	1
cg2340567	0.233237	0.515586	13.27469	1.55E-06	0.000461	5.996961	0.398967	0.632204	-0.23324	20	14690830	F	II	MACROD2	Body	opensea	Body-opens		rs5441572	18;22;34
cg0217392	0.218329	0.559182	13.27111	1.55E-06	0.000461	5.995044	0.450017	0.668346	-0.21833	20	44769380	R	II		IGR	opensea	IGR-opense			
cg0183376	0.242069	0.537296	13.27101	1.55E-06	0.000461	5.994989	0.416262	0.65833	-0.24207	9	1.09E+08	R	II		IGR	opensea	IGR-opense		rs1917919	21;1
cg1315620	0.215179	0.506532	13.2663	1.55E-06	0.000462	5.992458	0.398943	0.614122	-0.21518	2	28873060	R	II		IGR	opensea	IGR-opense		rs1378763	49;13
cg0259644	0.21275	0.596955	13.26011	1.56E-06	0.000462	5.989136	0.49058	0.70333	-0.21275	14	1.02E+08	F	II		IGR	opensea	IGR-opense		rs7560170	40
cg2077409	0.222744	0.526379	13.25937	1.56E-06	0.000462	5.988738	0.415007	0.637751	-0.22274	20	23087608	R	II		IGR	opensea	IGR-opense		rs1832262	1
cg1405756	0.208337	0.441249	13.2578	1.56E-06	0.000462	5.98789	0.337081	0.545418	-0.20834	2	17305179	F	II		IGR	opensea	IGR-opense		rs1443950	0;28;32
cg0001641	0.224901	0.677089	13.25667	1.56E-06	0.000462	5.987287	0.564639	0.78954	-0.2249	5	82840805	F	II	VCAN	Body	opensea	Body-opens		rs1816685	38
cg2542336	0.259125	0.563246	13.25605	1.56E-06	0.000462	5.986952	0.433683	0.692808	-0.25912	14	73480891	R	II	ZFYVE1	Body	opensea	Body-opens		rs1393819	50
cg1606021	0.2275	0.503464	13.25217	1.57E-06	0.000463	5.984864	0.389714	0.617214	-0.2275	4	86936666	F	II	MAPK10	3'UTR	opensea	3'UTR-oper		rs1382285	1;4
cg1988860	0.208079	0.489727	13.25107	1.57E-06	0.000463	5.984273	0.385687	0.593767	-0.20808	6	22443354	F	II		IGR	opensea	IGR-opense			
cg1384635	0.255109	0.502194	13.2475	1.57E-06	0.000463	5.982352	0.37464	0.629749	-0.25511	3	1.51E+08	R	II	MIR548H2	Body	opensea	Body-opens		rs5719518	1
cg0897342	0.210038	0.635078	13.24506	1.57E-06	0.000463	5.981042	0.530059	0.740097	-0.21004	21	40401238	F	II		IGR	opensea	IGR-opense		rs1132281	27
cg1393150	0.233137	0.599888	13.24325	1.57E-06	0.000463	5.980065	0.48332	0.716457	-0.23314	2	1.7E+08	F	II	ABCB11	ExonBnd	opensea	ExonBnd-op		rs1815336	0
cg1064265	0.216907	0.506778	13.24255	1.58E-06	0.000463	5.979688	0.398324	0.615231	-0.21691	1	76077469	R	II	SLC44A5	TSS1500	shelf	TSS1500-sh	chr1:7608(	rs1843122	35
cg2359676	0.250353	0.4858	13.23997	1.58E-06	0.000463	5.978303	0.360624	0.610976	-0.25035	8	1.21E+08	R	II		IGR	opensea	IGR-opense		rs5430849	6
cg0538340	0.238933	0.661597	13.23714	1.58E-06	0.000463	5.976775	0.54213	0.781063	-0.23893	15	65319353	R	II	MTFMT	Body	shelf	Body-shelf	chr15:653:	rs2021922	50;22;6
cg1923628	0.208359	0.556406	13.2356	1.58E-06	0.000464	5.975946	0.452226	0.660585	-0.20836	20	23215597	F	II		IGR	opensea	IGR-opense		rs7267349	19;44

cg0229647	0.240295	0.518303	13.22833	1.59E-06	0.000464	5.972034	0.398155	0.638451	-0.2403	8	1.04E+08	R	II	AZIN1-AS1	Body	opensea	Body-opens		rs5525803	32;10
cg2343227	0.211958	0.654274	13.22818	1.59E-06	0.000464	5.97195	0.548295	0.760253	-0.21196	3	19431834	R	II	KCNH8	Body	opensea	Body-opens			
cg1681883	0.200239	0.474608	13.22799	1.59E-06	0.000464	5.971848	0.374489	0.574728	-0.20024	17	37379606	F	II	STAC2	Body	shore	Body-shore	chr17:3738	rs5544646	10;22
cg1052984	0.218258	0.421015	13.22652	1.59E-06	0.000464	5.971057	0.311886	0.530144	-0.21826	16	49522337	R	II		IGR	shelf	IGR-shelf	chr16:4952	rs3727160	51;32;2;1
cg0849819	0.212524	0.501821	13.22583	1.59E-06	0.000464	5.970682	0.395559	0.608083	-0.21252	7	83271654	F	II	SEMA3E	TSS1500	opensea	TSS1500-op		rs5382559	45
cg1943713	0.229693	0.461474	13.22025	1.59E-06	0.000464	5.967677	0.346627	0.576321	-0.22969	22	42015675	F	II	XRCC6	TSS1500	shore	TSS1500-sh	chr22:420	rs7254742	32
cg2527017	0.216811	0.476357	13.21791	1.6E-06	0.000464	5.966411	0.367951	0.584762	-0.21681	22	44293765	F	II		IGR	opensea	IGR-opense		rs1152022	0;22;49
cg2286795	0.212924	0.575635	13.21718	1.6E-06	0.000464	5.966021	0.469173	0.682097	-0.21292	8	9274794	R	II		IGR	opensea	IGR-opense		rs5370310	35
cg0612819	0.202127	0.52623	13.21693	1.6E-06	0.000464	5.965885	0.425167	0.627294	-0.20213	5	1.68E+08	F	II		IGR	opensea	IGR-opense		rs1145056	0;18;19;36;48
cg1339872	0.209038	0.555847	13.2166	1.6E-06	0.000464	5.965707	0.451328	0.660366	-0.20904	20	47405977	R	II	PREX1	Body	opensea	Body-opens			
cg0728277	0.230747	0.566279	13.2124	1.6E-06	0.000465	5.963441	0.450906	0.681653	-0.23075	10	80391121	R	II		IGR	opensea	IGR-opense		rs5763727	23;1
cg0076349	0.200794	0.579682	13.20609	1.61E-06	0.000465	5.960034	0.479285	0.680079	-0.20079	4	22714863	R	II	GBA3	TSS1500	opensea	TSS1500-op		rs2863469	29
cg0605760	0.202094	0.52401	13.2046	1.61E-06	0.000465	5.959233	0.422963	0.625057	-0.20209	11	79322178	R	II		IGR	opensea	IGR-opense		rs1775717	49;35
cg2666795	0.212205	0.692367	13.20074	1.61E-06	0.000465	5.957143	0.586265	0.798469	-0.2122	10	11362901	R	II	CELF2-AS1	Body	opensea	Body-opens		rs1857024	42
cg1237261	0.21559	0.497574	13.2004	1.61E-06	0.000465	5.956963	0.389779	0.605369	-0.21559	1	2.12E+08	R	II		IGR	opensea	IGR-opense		rs5493077	48;47;44
cg0259309	0.22472	0.529885	13.19869	1.61E-06	0.000465	5.95604	0.417526	0.642245	-0.22472	1	27011393	F	II		IGR	opensea	IGR-opense			
cg0982838	0.230236	0.569266	13.19818	1.61E-06	0.000465	5.955765	0.454148	0.684384	-0.23024	12	63177404	R	II	PPM1H	Body	opensea	Body-opens		rs5478263	2;1
cg1141199	0.25581	0.491085	13.19067	1.62E-06	0.000466	5.951701	0.36318	0.61899	-0.25581	10	90878257	F	II		IGR	opensea	IGR-opense		rs5370986	46
cg1766271	0.200427	0.534761	13.18753	1.62E-06	0.000466	5.950006	0.434548	0.634975	-0.20043	8	24261632	R	II	ADAMDEC1	Body	opensea	Body-opens		rs1380687	32;2
cg1867012	0.205251	0.63405	13.18488	1.63E-06	0.000467	5.94857	0.531424	0.736675	-0.20525	4	5865698	F	II	CRMP1	Body	opensea	Body-opens		rs5452239	0;8;21
cg1379532	0.219386	0.446764	13.18182	1.63E-06	0.000467	5.946913	0.337072	0.556457	-0.21939	15	71398102	R	II		IGR	opensea	IGR-opense		rs8040775	25
cg2650930	0.208773	0.695836	13.18132	1.63E-06	0.000467	5.946646	0.59145	0.800223	-0.20877	2	28083763	F	II	RBKS	Body	opensea	Body-opens		rs1416994	23
cg2152610	0.242716	0.499516	13.17997	1.63E-06	0.000467	5.945917	0.378158	0.620874	-0.24272	15	32392647	R	II	CHRNA7	Body	opensea	Body-opens			
cg2010263	0.253142	0.589359	13.17585	1.63E-06	0.000467	5.943683	0.462788	0.71593	-0.25314	4	1.24E+08	R	II		IGR	opensea	IGR-opense		rs309388	40
cg1464559	0.214903	0.549554	13.17129	1.64E-06	0.000467	5.941215	0.442103	0.657006	-0.2149	8	21595402	F	II	GFRA2	Body	opensea	Body-opens		rs5281585	50
cg2266899	0.221807	0.537675	13.17006	1.64E-06	0.000467	5.940548	0.426772	0.648579	-0.22181	3	1.69E+08	F	II		IGR	opensea	IGR-opense		rs5307477	1;20
cg0321442	0.207961	0.590958	13.16958	1.64E-06	0.000467	5.940292	0.486978	0.694939	-0.20796	19	44257589	R	II	C19orf61	5'UTR	shore	5'UTR-shor	chr19:442	rs5405488	49;15;7
cg1338368	0.235923	0.599922	13.16924	1.64E-06	0.000467	5.940104	0.481961	0.717884	-0.23592	2	1.12E+08	R	II	LOC40099	TSS200	shore	TSS200-sho	chr2:1118	rs5601839	41;7;1
cg1584409	0.240816	0.594957	13.16715	1.64E-06	0.000467	5.938972	0.474549	0.715365	-0.24082	4	1.24E+08	F	II	IL21	Body	opensea	Body-opens			
cg0715138	0.256195	0.515019	13.16701	1.64E-06	0.000467	5.938896	0.386921	0.643117	-0.2562	5	1.27E+08	F	II		IGR	opensea	IGR-opense		rs5715783	40
cg0178546	0.212833	0.545458	13.16664	1.64E-06	0.000467	5.938696	0.439041	0.651874	-0.21283	2	40066183	R	II		IGR	opensea	IGR-opense		rs5451667	33;28;19;8;6
cg0664565	0.262226	0.591399	13.16564	1.64E-06	0.000467	5.938154	0.460287	0.722512	-0.26223	2	1.53E+08	F	II	CACNB4	Body	opensea	Body-opens		rs1488132	0;1;30;36;43;51
cg1353759	0.293762	0.574349	13.16323	1.65E-06	0.000467	5.936849	0.427467	0.72123	-0.29376	12	26451968	R	II		IGR	opensea	IGR-opense		rs5385523	48;1
cg0852998	0.218223	0.569089	13.16259	1.65E-06	0.000467	5.936499	0.459978	0.678201	-0.21822	14	1E+08	R	II		IGR	opensea	IGR-opense			
cg1620640	0.209045	0.458491	13.15605	1.65E-06	0.000469	5.932954	0.353969	0.563013	-0.20904	6	1.41E+08	F	II		IGR	opensea	IGR-opense		rs1879432	18;23
cg0624256	0.226458	0.523543	13.15171	1.66E-06	0.000469	5.930602	0.410314	0.636772	-0.22646	2	2.25E+08	R	II		IGR	opensea	IGR-opense			
cg0148521	0.256761	0.545865	13.1502	1.66E-06	0.000469	5.929779	0.417484	0.674245	-0.25676	10	93557062	F	II	TNKS2	TSS1500	shore	TSS1500-sh	chr10:935	rs5538520	13;50
cg0123544	0.209004	0.590351	13.14988	1.66E-06	0.000469	5.929609	0.485849	0.694853	-0.209	14	51940896	R	II	FRMD6-AS2	Body	opensea	Body-opens		rs5760060	2
cg0470047	0.208309	0.524597	13.14958	1.66E-06	0.000469	5.929442	0.420442	0.628751	-0.20831	6	1.11E+08	R	II		IGR	opensea	IGR-opense		rs6933588	43;31
cg0224029	0.237101	0.541064	13.14401	1.66E-06	0.000469	5.926422	0.422513	0.659615	-0.2371	17	59883075	R	II	BRIP1	Body	opensea	Body-opens			
cg0456751	0.247448	0.665088	13.14009	1.67E-06	0.000469	5.924292	0.541364	0.788812	-0.24745	5	71035990	R	II		IGR	opensea	IGR-opense		rs1424137	45;3
cg2388037	0.202813	0.470919	13.13988	1.67E-06	0.000469	5.924176	0.369513	0.572326	-0.20281	10	1.24E+08	R	II	BTBD16	Body	opensea	Body-opens		rs1894388	6;2
cg0115580	0.201546	0.500145	13.13877	1.67E-06	0.000469	5.923576	0.399372	0.600918	-0.20155	6	54905378	F	II		IGR	opensea	IGR-opense		rs1386069	1;24;38;49
cg2632726	0.25353	0.630583	13.1376	1.67E-06	0.000469	5.92294	0.503818	0.757348	-0.25353	15	48710196	F	II	FBN1	Body	opensea	Body-opens		rs1430775	0;3;14;16
cg2523733	0.245655	0.570295	13.13708	1.67E-06	0.000469	5.922657	0.447467	0.693123	-0.24566	6	17993827	R	II		IGR	opensea	IGR-opense			
cg0062729	0.231037	0.601532	13.13642	1.67E-06	0.000469	5.922302	0.486013	0.71705	-0.23104	14	1.01E+08	F	II	SNHG24	Body	opensea	Body-opens		rs5551177	0;32;35
cg1300849	0.261347	0.553884	13.13606	1.67E-06	0.000469	5.922101	0.42321	0.684558	-0.26135	3	1.19E+08	R	II	POPDC2	Body	opensea	Body-opens		rs5292043	2
cg0437394	0.221709	0.503086	13.13503	1.67E-06	0.000469	5.921546	0.392232	0.61394	-0.22171	12	90806795	F	II		IGR	opensea	IGR-opense		rs5704448	1;3;45
cg0845203	0.256826	0.51763	13.13282	1.67E-06	0.00047	5.920343	0.389217	0.646043	-0.25683	22	28426107	F	II	TTC28	Body	opensea	Body-opens		rs5692076	1;21
cg2632586	0.206802	0.454092	13.12861	1.68E-06	0.00047	5.918056	0.350691	0.557493	-0.2068	6	41398693	F	II		IGR	shelf	IGR-shelf	chr6:4139	rs1121964	9;41;49
cg1366330	0.227175	0.593321	13.12494	1.68E-06	0.00047	5.916057	0.479734	0.706909	-0.22718	5	35046992	F	II	AGXT2	Body	opensea	Body-opens		rs5414041	38
cg2288665	0.232614	0.472224	13.12042	1.69E-06	0.00047	5.913602	0.355917	0.588531	-0.23261	6	498807	R	II	EXOC2	Body	opensea	Body-opens		rs5451429	1
cg0468358	0.208558	0.458537	13.11638	1.69E-06	0.00047	5.911401	0.354258	0.562816	-0.20856	9	37679969	R	II	FRMPD1	5'UTR	opensea	5'UTR-oper		rs5490864	46;10
cg2423852	0.205404	0.444209	13.11391	1.69E-06	0.00047	5.910056	0.341507	0.546911	-0.2054	6	56597743	F	II	RNU6-71P	Body	opensea	Body-opens		rs5694198	0
cg2359805	0.210233	0.495485	13.1125	1.69E-06	0.00047	5.909289	0.390368	0.600602	-0.21023	4	1.77E+08	R	II	SPCS3	Body	shore	Body-shore	chr4:1772	rs1394977	8
cg0479413	0.2048	0.585462	13.10816	1.7E-06	0.00047	5.906926	0.483062	0.687862	-0.2048	18	60750880	F	II		IGR	opensea	IGR-opense		rs5434339	27;42;45;49
cg0008981	0.200592	0.61749	13.10708	1.7E-06	0.00047	5.906339	0.517194	0.717786	-0.20059	3	72641008	R	II		IGR	opensea	IGR-opense		rs5618881	16;2;1
cg0298611	0.210025	0.473605	13.10597	1.7E-06	0.00047	5.905731	0.368593	0.578618	-0.21002	15	59785759	R	II	FAM81A	Body	opensea	Body-opens		rs7600966	51;11
cg1159152	0.209391	0.528606	13.10492	1.7E-06	0.00047	5.905162	0.42391	0.633301	-0.20939	4	1.24E+08	F	II	IL21	TSS200	opensea	TSS200-ope		rs1462051	43
cg1121459	0.220347	0.611341	13.10305	1.7E-06	0.00047	5.904144	0.501168	0.721515	-0.22035	5	28287931	F	II		IGR	opensea	IGR-opense		rs5466210	5;6;27
cg1551810	0.203245	0.459164	13.10062	1.7E-06	0.00047	5.902814	0.357542	0.560786	-0.20324	3	53836983	R	II	CACNA1D	Body	opensea	Body-opens		rs2359132	48;32;2
cg2315923	0.206161	0.685151	13.09962	1.71E-06	0.00047	5.902271	0.58207	0.788231	-0.20616	22	46571660	F	II	PPARA	5'UTR	opensea	5'UTR-oper		rs5535064	8;36
cg1822709	0.232119	0.527381	13.09953	1.71E-06	0.00047	5.902223	0.411322	0.643441	-0.23212	18	36854676	R	II	LINC00669	Body	opensea	Body-opens		rs5333694	47;36;18;14;8
cg0144088	0.240364	0.481379	13.09386	1.71E-06	0.00047	5.899133	0.361197	0.601561	-0.24036	16	54169984	F	II		IGR	opensea	IGR-opense			
cg1424183	0.228446	0.57084	13.09376	1.71E-06	0.00047	5.899076	0.456617	0.685063	-0.22845	7	48124921	R	II		IGR	shelf	IGR-shelf	chr7:4812	rs5325277	1
cg2553185	0.205215	0.438954	13.09289	1.71E-06	0.00047	5.898603	0.336346	0.541561	-0.20522	20	47897451	F	II	C20orf199	Body	shelf	Body-shelf	chr20:478	rs1393377	0;15;35
cg0437525	0.246493	0.395163	13.09128	1.71E-06	0.00047	5.897723	0.271916	0.518409	-0.24649	5	66241308	F	II	MAST4	Body	opensea	Body-opens		rs1150686	0;23;42
cg1285706	0.203694	0.531876	13.09042	1.71E-06	0.00047	5.897252	0.43003	0.633723	-0.20369	8	1.1E+08	F	II		IGR	opensea	IGR-opense		rs5779529	0;27
cg2632961	0.209371	0.491653	13.08892	1.72E-06	0.00047	5.896435	0.386968	0.596339	-0.20937	5	36302704	F	II	RANBP3L	TSS1500	opensea	TSS1500-op		rs5647919	7
cg2482429	0.201428	0.479518	13.08678	1.72E-06	0.00047	5.895266	0.378804	0.580232	-0.20143	8	1.18E+08	R	II		IGR	opensea	IGR-opense		rs5444816	15;2
cg2358540	0.244755	0.641538	13.08636	1.72E-06	0.00047	5.895037	0.51916	0.763916	-0.24476	2	38243638	R	II	RMDN2	Body	opensea	Body-opens		rs5293081	47;40;1
cg1358082	0.210138	0.547329	13.08582	1.72E-06	0.00047	5.894745	0.44226	0.652398	-0.21014	9	72081155	F	II	APBA1	Body	opensea	Body-opens			
cg0864299	0.206148	0.510547	13.08293	1.72E-06	0.00047	5.893166	0.407473	0.613621	-0.20615	2	66914032	R	II	LOC10050	Body	opensea	Body-opens		rs1762856	10;2;1
cg0240107	0.217012	0.444112	13.0819	1.72E-06	0.00047	5.892605	0.335606	0.552618	-0.21701	10	49660642	F	II	ARHGAP22	Body	shore	Body-shore	chr10:496	rs1511527	0;1
cg0672513	0.222315	0.526612	13.08007	1.72E-06	0.00047	5.891606	0.415454	0.63777	-0.22232	21	36044374	F	II	CLIC6	Body	shore	Body-shore	chr21:360		
cg0570272	0.229814	0.475198	13.07721	1.73E-06	0.00047	5.890043	0.360291	0.590105	-0.22981	4	1.12E+08	R	II	PITX2	TSS1500	shelf	TSS1500-sh	chr4:1115	rs5411311	29;1
cg1312395	0.206389	0.543121	13.07637	1.73E-06	0.00047	5.889583	0.439927	0.646316	-0.20639	2	2.23E+08	F	II		IGR	opensea	IGR-opense		rs5386987	32;46
cg0703339	0.223016	0.57526	13.0748	1.73E-06	0.00047	5.888729	0.463752	0.686769	-0.22302	1	28906538	R	II	SNHG12	Body	shore	Body-shore	chr1:2890	rs1926234	2
cg0399333	0.254496	0.52509	13.07475	1.73E-06	0.00047	5.8887	0.397842	0.652338	-0.2545	16	24863047	R	II	SLC5A11	5'UTR	opensea	5'UTR-oper		rs1178622	2
cg2668918	0.228235	0.568013	13.07399	1.73E-06	0.00047	5.888286	0.453895	0.68213	-0.22824	7	33301429	F	II	BBS9	Body	opensea	Body-opens		rs1830557	35
cg2763394	0.200467	0.61903	13.07077	1.73E-06	0.000471	5.886524	0.518797	0.719263	-0.20047	2	1.52E+08	R	II		IGR	opensea	IGR-opense		rs5343530	40
cg0173130	0.207141	0.448685	13.07009	1.73E-06	0.000471	5.886152	0.345115	0.552256	-0.20714	9	85164256	F	II		IGR	opensea	IGR-opense		rs1135072	0;5;30
cg0076122	0.224933	0.566822	13.06573	1.74E-06	0.000471	5.883769	0.454356	0.679289	-0.22493	13	25851201	R	II	MTMR6	Body	opensea	Body-opens		rs1461722	38;19
cg1095514	0.224813	0.423749	13.06461	1.74E-06	0.000471	5.88316	0.311342	0.536155	-0.22481	4	90790505	R	II		IGR	opensea	IGR-opense		rs1916283	24
cg0218696	0.22196	0.574524	13.06398	1.74E-06	0.000471	5.882815	0.463544	0.685504	-0.22196	14	68996759	F	II	RAD51B	Body	opensea	Body-opens		rs1909867	0
cg1386103	0.258312	0.497421	13.06388	1.74E-06	0.000471	5.882758	0.368265	0.626576	-0.25831	11	5385322	R	II	OR51B5	Body	opensea	Body-opens		rs1846079	49;32
cg0118993	0.256439	0.60871	13.06376	1.74E-06	0.000471	5.882694	0.48049	0.736929	-0.25644	13	43385958	R	II	LINC01050	ExonBnd	opensea	ExonBnd-op		rs1919289	45;24;1
cg2671274	0.209945	0.393188	13.06154	1.74E-06	0.000471	5.881481	0.288215	0.498161	-0.20995	10	1E+08	F	II	PYROXD2	Body	opensea	Body-opens		rs1844302	0;19
cg2632895	0.218188	0.484732	13.05891	1.74E-06	0.000471	5.88004	0.375638	0.593826	-0.21819	1	28906514	R	II	SNHG12	Body	shore	Body-shore	chr1:2890		
cg1919076	0.201292	0.508917	13.05671	1.75E-06	0.000471	5.878834	0.408271	0.609562	-0.20129	13	44806055	R	II		IGR	opensea	IGR-opense		rs5654910	2
cg1488340	0.239253	0.479845	13.05399	1.75E-06	0.000471	5.877345	0.360218	0.599471	-0.23925	4	1.24E+08	F	II		IGR	opensea	IGR-opense			
cg1459529	0.208725	0.441108	13.05077	1.75E-06	0.000472	5.875583	0.336745	0.545471	-0.20873	11	35993855	F	II	LDLRAD3	5'UTR	opensea	5'UTR-oper		rs5563810	13;42
cg1516049	0.222486	0.576604	13.04956	1.75E-06	0.000472	5.874923	0.465361	0.687847	-0.22249	1	68346041	R	II	GNG12-AS1	Body	opensea	Body-opens		rs5639797	42;10;2;1
cg1190240	0.207956	0.57302	13.04677	1.76E-06	0.000472	5.873396	0.469042	0.676998	-0.20796	3	14859299	F	II	FGD5	TSS1500	opensea	TSS1500-op		rs1925837	1;19;26
cg0815404	0.243971	0.482986	13.04464	1.76E-06	0.000472	5.872226	0.361	0.604971	-0.24397	7	1.05E+08	F	II	ATXN7L1	Body	opensea	Body-opens		rs5725654	41
cg1648675	0.231281	0.664558	13.03905	1.76E-06	0.000472	5.869162	0.548917	0.780198	-0.23128	7	5624786	R	II		IGR	opensea	IGR-opense		rs5480984	36;17;8;1
cg1348046	0.249672	0.503531	13.02261	1.78E-06	0.000474	5.860147	0.378695	0.628366	-0.24967	4	16795757	F	II	LDB2	Body	opensea	Body-opens		rs5746426	0;5;11
cg1389638	0.218089	0.506271	13.02096	1.78E-06	0.000474	5.859242	0.397227	0.615316	-0.21809	11	1.22E+08	R	II		IGR	opensea	IGR-opense		rs5754021	42;28
cg2310304	0.211417	0.541875	13.0152	1.79E-06	0.000475	5.856078	0.436167	0.647584	-0.21142	17	69646723	F	II		IGR	opensea	IGR-opense		rs1864745	35;41;45
cg2724441	0.216254	0.593374	13.00384	1.8E-06	0.000477	5.849838	0.485247	0.701501	-0.21625	10	78045133	F	II	C10orf11	Body	opensea	Body-opens		rs5629782	0;42
cg2519123	0.219879	0.410308	13.0036	1.8E-06	0.000477	5.849704	0.300368	0.520248	-0.21988	12	1.26E+08	R	II		IGR	opensea	IGR-opense		rs1861873	51;38;1
cg0414523	0.202653	0.489551	13.00353	1.8E-06	0.000477	5.849667	0.388224	0.590878	-0.20265	13	98854715	R	II	FARP1	5'UTR	opensea	5'UTR-oper		rs5295191	29
cg1494508	0.220671	0.493801	12.9957	1.81E-06	0.000477	5.845357	0.383465	0.604136	-0.22067	18	2657780	R	II	SMCHD1	Body	shore	Body-shore	chr18:265	rs5403109	49;26;16
cg0950695	0.202229	0.65852	12.99379	1.81E-06	0.000477	5.844309	0.557405	0.759634	-0.20223	19	13841688	R	II		IGR	opensea	IGR-opense		rs1480241	49;5
cg0072278	0.231921	0.603041	12.99276	1.81E-06	0.000477	5.843741	0.48708	0.719001	-0.23192	6	22008819	R	II	CASC15	Body	opensea	Body-opens		rs1878181	1
cg2310201	0.207236	0.399099	12.99271	1.81E-06	0.000477	5.843712	0.295481	0.502717	-0.20724	15	70574295	F	II		IGR	opensea	IGR-opense			
cg2390210	0.249144	0.544153	12.99233	1.81E-06	0.000477	5.843505	0.419581	0.668725	-0.24914	6	22009091	F	II	FLJ22536	Body	opensea	Body-opens		rs5421717	1;19
cg0549381	0.200938	0.528081	12.98945	1.81E-06	0.000477	5.841919	0.427612	0.62855	-0.20094	21	47157612	R	II		IGR	opensea	IGR-opense		rs1901306	25
cg1757201	0.229211	0.625104	12.98916	1.81E-06	0.000477	5.841759	0.510498	0.739709	-0.22921	5	1.5E+08	F	II		IGR	opensea	IGR-opense		rs5345037	0;42
cg0081273	0.206787	0.438292	12.98727	1.82E-06	0.000477	5.840719	0.334899	0.541686	-0.20679	4	77679152	R	II	SHROOM3	Body	opensea	Body-opens		rs5586544	36
cg1507068	0.205975	0.52398	12.98582	1.82E-06	0.000477	5.839918	0.420993	0.626968	-0.20597	4	83209071	R	II		IGR	shelf	IGR-shelf	chr4:8320	rs5337055	51;3
cg1725171	0.218538	0.427875	12.98189	1.82E-06	0.000477	5.837754	0.318606	0.537144	-0.21854	18	61443038	F	II	SERPINB7	5'UTR	opensea	5'UTR-oper		rs1905693	0;45
cg2299294	0.2056	0.480644	12.97997	1.82E-06	0.000477	5.836698	0.377844	0.583444	-0.2056	8	30354402	R	II	RBPMS	Body	opensea	Body-opens		rs1762796	28;26;16;2
cg2548125	0.265004	0.496963	12.9791	1.82E-06	0.000477	5.836218	0.36446	0.629465	-0.265	7	1.42E+08	F	II	TAS2R38	1stExon	opensea	1stExon-op		rs1411968	0;1;13;14
cg1179840	0.247969	0.574099	12.97259	1.83E-06	0.000477	5.832632	0.450114	0.698083	-0.24797	21	40145361	R	II	NCRNA001	Body	opensea	Body-opens		rs5352195	15
cg0186125	0.210218	0.525493	12.96965	1.83E-06	0.000477	5.831011	0.420383	0.630602	-0.21022	2	2.38E+08	R	II		IGR	opensea	IGR-opense		rs5717819	47
cg0539294	0.256067	0.560231	12.9673	1.84E-06	0.000478	5.82971	0.432197	0.688264	-0.25607	13	77091333	R	II		IGR	opensea	IGR-opense		rs1905198	46;44;38;2
cg2718340	0.224288	0.398669	12.9648	1.84E-06	0.000478	5.82833	0.286525	0.510813	-0.22429	18	47315112	R	II	ACAA2	Body	opensea	Body-opens			
cg1184878	0.218999	0.535982	12.9628	1.84E-06	0.000478	5.827232	0.426482	0.645481	-0.219	1	39041399	F	II		IGR	shelf	IGR-shelf	chr1:3904	rs5455271	7

cg1549361	0.239108	0.524258	12.96183	1.84E-06	0.000478	5.826694	0.404704	0.643812	-0.23911	3	1.43E+08	F	II	SLC9A9	Body	opensea	Body-opens		rs5545630	37
cg1922816	0.218099	0.51009	12.96116	1.84E-06	0.000478	5.826327	0.401041	0.61914	-0.2181	12	13100222	R	II	GPRC5D	Body	opensea	Body-opens		rs1410230	4;1
cg1288198	0.24168	0.542342	12.96084	1.84E-06	0.000478	5.82615	0.421502	0.663183	-0.24168	4	13915406	F	II	LINC01182	Body	opensea	Body-opens		rs1858916	27
cg1466071	0.208761	0.768743	12.95913	1.85E-06	0.000478	5.825204	0.664362	0.873123	-0.20876	18	42217877	F	II		IGR	opensea	IGR-opensea		rs5329342	0
cg0331707	0.226334	0.587995	12.95786	1.85E-06	0.000478	5.824502	0.474828	0.701162	-0.22633	4	80928989	F	II	ANTXR2	Body	opensea	Body-opens		rs5327060	0;3;7;48
cg1613368	0.242156	0.52457	12.95333	1.85E-06	0.000479	5.822003	0.403492	0.645648	-0.24216	9	79039563	R	II		IGR	opensea	IGR-opensea		rs5675232	2
cg1628628	0.20596	0.483051	12.95308	1.85E-06	0.000479	5.821863	0.380071	0.586031	-0.20596	5	1.24E+08	R	II	ZNF608	Body	opensea	Body-opens			
cg0761049	0.21794	0.439644	12.94465	1.86E-06	0.00048	5.817206	0.330674	0.548614	-0.21794	10	6312774	R	II		IGR	opensea	IGR-opensea		rs7972136	45;36;20;1
cg0602410	0.245751	0.467432	12.94386	1.86E-06	0.00048	5.816768	0.344557	0.590308	-0.24575	1	1.54E+08	R	II	SNAPIN	TSS1500	shore	TSS1500-sh	chr1:15363		
cg1939081	0.209927	0.570623	12.94312	1.86E-06	0.00048	5.816361	0.46566	0.675587	-0.20993	10	71001680	R	II	HKDC1	Body	opensea	Body-opens		rs5590203	1
cg2058787	0.218069	0.434559	12.9414	1.86E-06	0.00048	5.815408	0.325525	0.543593	-0.21807	2	1.79E+08	F	II	MIR548N	Body	opensea	Body-opens		rs5776743	0;7
cg0832127	0.22538	0.571813	12.93865	1.87E-06	0.00048	5.81389	0.459122	0.684503	-0.22538	6	89928426	F	II	GABRR1	TSS1500	opensea	TSS1500-op		rs1420232	0
cg2410884	0.205189	0.553673	12.93737	1.87E-06	0.00048	5.813178	0.451079	0.656268	-0.20519	20	39651831	R	II		IGR	opensea	IGR-opensea		rs5452803	37;19
cg0032537	0.210341	0.509174	12.93422	1.87E-06	0.000481	5.811436	0.404003	0.614344	-0.21034	5	56797893	R	II		IGR	opensea	IGR-opensea		rs5696474	42
cg0065993	0.208439	0.628464	12.93016	1.88E-06	0.000481	5.809193	0.524244	0.732683	-0.20844	15	1.02E+08	R	II	PCSK6	Body	opensea	Body-opens		rs1391393	26;1
cg0003321	0.215481	0.50553	12.92778	1.88E-06	0.000481	5.807874	0.397789	0.61327	-0.21548	10	7773345	R	II	ITIH2	Body	opensea	Body-opens		rs5408954	24
cg1864660	0.213849	0.521446	12.92299	1.88E-06	0.000481	5.805219	0.414521	0.62837	-0.21385	6	1.47E+08	R	II	STXBP5-AS1	Body	opensea	Body-opens		rs1810092	48;31;23;1
cg0916880	0.217555	0.47123	12.92196	1.88E-06	0.000482	5.804649	0.362452	0.580007	-0.21755	8	97056373	R	II		IGR	opensea	IGR-opensea			
cg1966979	0.211774	0.5414	12.92131	1.89E-06	0.000482	5.80429	0.435513	0.647287	-0.21177	1	57154147	F	II	PRKAA2	Body	opensea	Body-opens			
cg2478404	0.235323	0.410989	12.9191	1.89E-06	0.000482	5.803064	0.293327	0.52865	-0.23532	3	33034475	F	II		IGR	opensea	IGR-opensea		rs5465351	1;4;25;36;49
cg1033807	0.213417	0.685461	12.91818	1.89E-06	0.000482	5.802555	0.578753	0.79217	-0.21342	11	63302736	F	II		IGR	opensea	IGR-opensea		rs1828207	12;22
cg1267518	0.21721	0.449257	12.90732	1.9E-06	0.000484	5.796537	0.340652	0.557861	-0.21721	11	1.33E+08	F	II	OPCML	Body	opensea	Body-opens		rs3733347	3;32
cg1952637	0.205856	0.510294	12.90542	1.9E-06	0.000484	5.795485	0.407365	0.613222	-0.20586	21	31130470	R	II	GRIK1-AS1	Body	opensea	Body-opens		rs1395360	41;1
cg0613436	0.273228	0.634025	12.89857	1.91E-06	0.000484	5.791679	0.497411	0.770639	-0.27323	3	1.25E+08	F	II	SLC12A8	Body	opensea	Body-opens		rs5506197	1;5;13;48
cg1166730	0.212589	0.610015	12.89703	1.91E-06	0.000484	5.790825	0.503721	0.716309	-0.21259	2	2.03E+08	R	II	SNORD70	Body	opensea	Body-opens		rs2014848	42;36;27;22;20;2;1
cg0886490	0.201777	0.501747	12.89614	1.91E-06	0.000484	5.790333	0.400859	0.602635	-0.20178	8	79472625	R	II	PKIA	5'UTR	opensea	5'UTR-open		rs5754158	25;2
cg0040453	0.249452	0.531449	12.8942	1.91E-06	0.000484	5.789254	0.406723	0.656175	-0.24945	20	8702075	F	II	PLCB1	Body	opensea	Body-opens		rs5583118	27;28;40;48
cg1716401	0.212203	0.498534	12.89226	1.92E-06	0.000484	5.788177	0.392433	0.604636	-0.2122	8	1.11E+08	F	II		IGR	shelf	IGR-shelf	chr8:11098	rs5624441	0
cg0847771	0.227683	0.548608	12.89197	1.92E-06	0.000484	5.788015	0.434767	0.66245	-0.22768	17	41671170	R	II		IGR	opensea	IGR-opensea			
cg1730289	0.20974	0.513509	12.89037	1.92E-06	0.000484	5.78713	0.408639	0.618379	-0.20974	5	1.5E+08	F	II		IGR	shelf	IGR-shelf	chr5:14954	rs5478520	1;42
cg1894088	0.235842	0.478769	12.8885	1.92E-06	0.000484	5.786092	0.360848	0.59669	-0.23584	8	62800933	R	II		IGR	opensea	IGR-opensea		rs5393993	42;41;25
cg1454301	0.20465	0.386229	12.88698	1.92E-06	0.000484	5.785247	0.283904	0.488554	-0.20465	20	44573011	R	II	PCIF1	Body	shore	Body-shore	chr20:4457	rs5491227	44;17;1
cg0235836	0.233067	0.538027	12.88564	1.92E-06	0.000485	5.784502	0.421493	0.654561	-0.23307	5	1.72E+08	R	II	NEURL1B	Body	shore	Body-shore	chr5:17209	rs1444149	9
cg0587810	0.201965	0.518442	12.88357	1.93E-06	0.000485	5.783352	0.41746	0.619425	-0.20196	13	63057588	F	II		IGR	opensea	IGR-opensea			
cg0789872	0.26033	0.514476	12.88241	1.93E-06	0.000485	5.782707	0.384311	0.644641	-0.26033	5	1.09E+08	R	II		IGR	opensea	IGR-opensea		rs5609340	14;2;1
cg1269377	0.213554	0.541791	12.8759	1.93E-06	0.000485	5.779086	0.435014	0.648567	-0.21355	7	20572677	R	II		IGR	opensea	IGR-opensea		rs1841477	23;13
cg1749995	0.267745	0.486775	12.87091	1.94E-06	0.000486	5.776308	0.352903	0.620648	-0.26775	12	40562179	F	II		IGR	opensea	IGR-opensea		rs5725517	0;15;39
cg2603683	0.224672	0.611911	12.86786	1.94E-06	0.000486	5.77461	0.499574	0.724247	-0.22467	10	13723418	F	II	FRMD4A	Body	opensea	Body-opens		rs1414371	0
cg1596509	0.211407	0.410702	12.86785	1.94E-06	0.000486	5.774608	0.304998	0.516406	-0.21141	9	1.1E+08	F	II		IGR	opensea	IGR-opensea		rs7435796	1;42;48
cg0931965	0.200965	0.588373	12.86727	1.94E-06	0.000486	5.774282	0.48789	0.688855	-0.20096	1	2.05E+08	R	II	KLHDC8A	5'UTR	shelf	5'UTR-shelf	chr1:20531	rs5551829	36;10;2
cg1157642	0.226738	0.40592	12.86343	1.95E-06	0.000486	5.772145	0.292552	0.519289	-0.22674	5	1.32E+08	F	II	C5orf56	5'UTR	shelf	5'UTR-shelf	chr5:13174		
cg1239967	0.220313	0.684078	12.86234	1.95E-06	0.000487	5.771541	0.573921	0.794234	-0.22031	2	2.09E+08	R	II		IGR	opensea	IGR-opensea		rs1828354	40;18;8
cg1870074	0.276513	0.402938	12.84924	1.96E-06	0.000488	5.764241	0.264682	0.541195	-0.27651	12	1.13E+08	R	II	NAA25	Body	opensea	Body-opens			
cg0690695	0.255217	0.549925	12.84828	1.97E-06	0.000488	5.763703	0.422317	0.677534	-0.25522	5	71614854	R	II	PTCD2	TSS1500	shore	TSS1500-sh	chr5:71616	rs5669637	6
cg2460449	0.201462	0.569444	12.84782	1.97E-06	0.000488	5.76345	0.468713	0.670175	-0.20146	3	31752002	F	II	OSBPL10-A	Body	opensea	Body-opens		rs5316077	35
cg1159481	0.256056	0.509773	12.84324	1.97E-06	0.000488	5.760892	0.381745	0.637801	-0.25606	11	1.15E+08	R	II	CADM1	Body	opensea	Body-opens		rs3774602	2;14;20;21;23
cg1241854	0.216811	0.523667	12.84304	1.97E-06	0.000488	5.760781	0.415261	0.632073	-0.21681	1	2.08E+08	F	II		IGR	opensea	IGR-opensea		rs1470586	0
cg1459780	0.245378	0.607948	12.8426	1.97E-06	0.000488	5.760538	0.485259	0.730637	-0.24538	5	1.32E+08	R	II	RAD50	Body	opensea	Body-opens		rs5387155	47;2
cg0692942	0.20449	0.537907	12.84213	1.97E-06	0.000488	5.760272	0.435662	0.640152	-0.20449	7	84315906	F	II		IGR	opensea	IGR-opensea		rs5566819	0;1;4;9;40
cg0894620	0.208018	0.520764	12.84167	1.97E-06	0.000488	5.760019	0.416755	0.624773	-0.20802	18	10169877	F	II		IGR	opensea	IGR-opensea		rs1147123	1
cg1686204	0.200313	0.43179	12.84034	1.97E-06	0.000488	5.759276	0.331634	0.531947	-0.20031	4	11705905	R	II		IGR	opensea	IGR-opensea			
cg2080808	0.243928	0.515248	12.83999	1.97E-06	0.000488	5.759078	0.393284	0.637213	-0.24393	2	6962835	R	II		IGR	opensea	IGR-opensea		rs1834726	25;14;1
cg1554998	0.203615	0.433366	12.83874	1.98E-06	0.000488	5.758384	0.331559	0.535173	-0.20361	4	1.39E+08	R	II	LINC00616	Body	opensea	Body-opens		rs7974621	1
cg2529610	0.271443	0.490284	12.83848	1.98E-06	0.000488	5.758237	0.354563	0.626006	-0.27144	2	1.45E+08	R	II	ZEB2	Body	opensea	Body-opens		rs5374792	46;43;21
cg0539421	0.205691	0.515695	12.83333	1.98E-06	0.000488	5.755361	0.41285	0.618541	-0.20569	11	31130488	F	II		IGR	opensea	IGR-opensea			
cg2009805	0.203175	0.610435	12.82818	1.99E-06	0.000489	5.752488	0.508847	0.712022	-0.20317	17	53325360	R	II		IGR	opensea	IGR-opensea			
cg0534223	0.239832	0.609571	12.82596	1.99E-06	0.000489	5.751247	0.489655	0.729488	-0.23983	10	70426406	R	II	TET1	Body	opensea	Body-opens		rs5412749	35
cg0298761	0.200108	0.546037	12.82471	1.99E-06	0.000489	5.750547	0.445983	0.646091	-0.20011	10	30054988	R	II		IGR	opensea	IGR-opensea		rs5310867	26;22;2
cg2528657	0.209934	0.484561	12.82164	2E-06	0.00049	5.748836	0.379594	0.589527	-0.20993	1	56069436	R	II		IGR	opensea	IGR-opensea		rs5569243	47;45;13;1
cg0655815	0.205418	0.681013	12.81782	2E-06	0.00049	5.746697	0.578304	0.783722	-0.20542	8	64144576	F	II		IGR	opensea	IGR-opensea		rs5473866	44
cg0405862	0.226612	0.452186	12.81686	2E-06	0.00049	5.74616	0.33888	0.565492	-0.22661	20	48285160	F	II	B4GALT5	Body	opensea	Body-opens			
cg2258181	0.205636	0.576854	12.81336	2E-06	0.00049	5.744205	0.474036	0.679673	-0.20564	13	43815115	F	II	ENOX1	Body	opensea	Body-opens		rs1846153	1;8;26;51
cg0800092	0.20825	0.698553	12.80878	2.01E-06	0.00049	5.741639	0.594428	0.802678	-0.20825	7	26676945	F	II	C7orf71	TSS1500	opensea	TSS1500-op		rs1419815	14;34
cg0946360	0.209713	0.420909	12.80705	2.01E-06	0.00049	5.740674	0.316053	0.525766	-0.20971	1	84255079	R	II	LOC10192	Body	opensea	Body-opens		rs1139648	34
cg0348736	0.245911	0.521693	12.80671	2.01E-06	0.00049	5.740482	0.398737	0.644648	-0.24591	12	50643418	R	II	LIMA1	5'UTR	opensea	5'UTR-open		rs5521908	37;1
cg1396472	0.20921	0.49713	12.80468	2.01E-06	0.00049	5.739347	0.392525	0.601735	-0.20921	9	71062328	R	II	PGM5	Body	opensea	Body-opens		rs1884635	2
cg2419631	0.239554	0.559406	12.79511	2.03E-06	0.000491	5.733985	0.439629	0.679183	-0.23955	21	33086923	F	II	SCAF4	Body	opensea	Body-opens		rs5496552	1;25;38;42
cg1768500	0.247945	0.535749	12.78883	2.03E-06	0.000492	5.730467	0.411777	0.659721	-0.24794	2	2.17E+08	R	II		IGR	opensea	IGR-opensea		rs5281621	19;8;2;1
cg0700071	0.249676	0.456577	12.78826	2.03E-06	0.000492	5.730148	0.331739	0.581415	-0.24968	12	62463441	R	II	FAM19A2	5'UTR	opensea	5'UTR-open		rs348645	31
cg0466933	0.209613	0.473815	12.78607	2.04E-06	0.000492	5.728923	0.369009	0.578621	-0.20961	2	1.33E+08	F	II		IGR	opensea	IGR-opensea		rs4018100	8;17;19;25;26
cg1535912	0.216272	0.362616	12.78331	2.04E-06	0.000492	5.727373	0.25448	0.470752	-0.21627	11	1.14E+08	R	II	ZBTB16	Body	opensea	Body-opens		rs5371618	14
cg2464755	0.202883	0.677206	12.78279	2.04E-06	0.000492	5.727079	0.575764	0.778647	-0.20288	1	1.71E+08	F	II	PRRX1	Body	opensea	Body-opens			
cg0537474	0.23077	0.635997	12.78223	2.04E-06	0.000492	5.726768	0.520612	0.751382	-0.23077	10	13770043	F	II	FRMD4A	Body	opensea	Body-opens		rs6602676	22
cg1975365	0.225861	0.553596	12.78113	2.04E-06	0.000492	5.726147	0.440666	0.666527	-0.22586	8	38584462	F	II	TACC1	TSS1500	opensea	TSS1500-op		rs1131941	14;51
cg0982740	0.258065	0.456682	12.77897	2.04E-06	0.000492	5.724937	0.32765	0.585715	-0.25807	3	1.95E+08	F	II		IGR	opensea	IGR-opensea		rs7788498	17;48
cg1044898	0.216625	0.546541	12.77881	2.04E-06	0.000492	5.724847	0.438228	0.654854	-0.21663	14	86041336	R	II	FLRT2	5'UTR	opensea	5'UTR-open		rs4899873	45;39
cg2619051	0.251558	0.476931	12.77868	2.04E-06	0.000492	5.724778	0.351152	0.60271	-0.25156	3	1.09E+08	R	II	MORC1	Body	opensea	Body-opens		rs1115950	16;26
cg2191982	0.222239	0.473307	12.77847	2.05E-06	0.000492	5.724656	0.362187	0.584427	-0.22224	7	28081543	F	II	JAZF1	Body	opensea	Body-opens		rs1503020	0;7
cg1368319	0.218676	0.522817	12.77793	2.05E-06	0.000492	5.724357	0.413478	0.632155	-0.21868	9	1.04E+08	R	II	C9orf125	3'UTR	opensea	3'UTR-open			
cg2610620	0.227825	0.548447	12.77752	2.05E-06	0.000492	5.724125	0.434535	0.66236	-0.22782	1	1.93E+08	R	II	RGS13	Body	opensea	Body-opens		rs5531883	35;14
cg0966597	0.268642	0.552047	12.77651	2.05E-06	0.000492	5.723558	0.417726	0.686368	-0.26864	2	1.73E+08	F	II	SLC25A12	Body	opensea	Body-opens		rs5507590	0
cg0522477	0.234722	0.498642	12.77329	2.05E-06	0.000492	5.72175	0.381281	0.616004	-0.23472	12	68553819	R	II	IFNG	TSS1500	opensea	TSS1500-op			
cg1527837	0.235414	0.466291	12.7732	2.05E-06	0.000492	5.721699	0.348584	0.583997	-0.23541	12	1.32E+08	F	I	GPR133	Body	shelf	Body-shelf	chr12:1315	rs7346876	0;6;18;37;40
cg1768949	0.260133	0.49822	12.76898	2.06E-06	0.000493	5.71933	0.368153	0.628286	-0.26013	10	1.29E+08	F	II	DOCK1	Body	opensea	Body-opens			
cg2266425	0.262985	0.5115	12.76333	2.06E-06	0.000494	5.716159	0.380007	0.642992	-0.26299	12	25836256	R	II		IGR	opensea	IGR-opensea		rs1236732	41
cg0697069	0.220139	0.538699	12.76172	2.06E-06	0.000494	5.715254	0.42863	0.648769	-0.22014	7	2117046	R	II	MAD1L1	Body	shore	Body-shore	chr7:2116	rs5768059	29;27
cg2049537	0.244984	0.350003	12.76026	2.07E-06	0.000494	5.714432	0.227511	0.472495	-0.24498	14	23266481	F	II	SLC7A7	Body	opensea	Body-opens		rs1428133	0;7;23;41;44;45
cg1789494	0.226028	0.62614	12.75812	2.07E-06	0.000494	5.713229	0.513126	0.739154	-0.22603	14	67600512	R	II	GPHN	Body	opensea	Body-opens		rs5728979	23;1
cg2150728	0.202083	0.588404	12.75762	2.07E-06	0.000494	5.712949	0.487363	0.689445	-0.20208	14	59992203	F	II	CCDC175	Body	opensea	Body-opens		rs5697248	1;17
cg1039558	0.21393	0.554681	12.7562	2.07E-06	0.000494	5.712151	0.447716	0.661646	-0.21393	14	32964719	R	II	AKAP6	Body	opensea	Body-opens		rs5533906	9;2
cg0799434	0.20668	0.503449	12.75465	2.07E-06	0.000494	5.711275	0.400109	0.606789	-0.20668	7	19969042	R	II	LOC10192	Body	opensea	Body-opens		rs1913321	41;38;7
cg2018245	0.20296	0.37605	12.75431	2.07E-06	0.000494	5.711088	0.27457	0.47753	-0.20296	10	7662882	F	II	ITIH5	TSS1500	opensea	TSS1500-op			
cg2185896	0.244664	0.694101	12.75295	2.08E-06	0.000494	5.710323	0.571769	0.816433	-0.24466	6	52148119	F	II	MCM3	Body	shore	Body-shore	chr6:5214		
cg0438546	0.231983	0.505219	12.75272	2.08E-06	0.000494	5.710191	0.389227	0.62121	-0.23198	4	55796460	R	II		IGR	opensea	IGR-opensea			
cg1735734	0.264372	0.428438	12.75031	2.08E-06	0.000494	5.708838	0.296251	0.560624	-0.26437	2	58603854	F	II		IGR	opensea	IGR-opensea		rs5549182	0;1;29
cg1108959	0.227166	0.421538	12.7458	2.08E-06	0.000494	5.706302	0.307955	0.535121	-0.22717	6	1.55E+08	R	II		IGR	opensea	IGR-opensea		rs1816465	48;47;40
cg0137458	0.225042	0.552041	12.74559	2.08E-06	0.000494	5.706182	0.43952	0.664562	-0.22504	15	62521056	F	II		IGR	opensea	IGR-opensea		rs5484915	0;4
cg1171460	0.233269	0.53969	12.74262	2.09E-06	0.000494	5.704507	0.423056	0.656325	-0.23327	5	1.32E+08	R	II	RAD50	Body	opensea	Body-opens		rs5550471	50;39;7;4
cg0435054	0.209907	0.606589	12.74078	2.09E-06	0.000494	5.703471	0.501635	0.711542	-0.20991	4	33937903	F	II	LOC10192	Body	opensea	Body-opens		rs5576123	1;16
cg1772759	0.248297	0.453567	12.73386	2.1E-06	0.000494	5.699574	0.329418	0.577716	-0.2483	5	1.18E+08	F	II		IGR	opensea	IGR-opensea			
cg1410635	0.20306	0.568622	12.73317	2.1E-06	0.000494	5.699189	0.467093	0.670152	-0.20306	4	1.07E+08	R	II		IGR	opensea	IGR-opensea		rs5324613	11;4;3;1
cg1884558	0.23691	0.512775	12.73288	2.1E-06	0.000494	5.699023	0.39432	0.631231	-0.23691	9	1.26E+08	F	II		IGR	opensea	IGR-opensea		rs5415831	21
cg1911164	0.203913	0.595352	12.73229	2.1E-06	0.000494	5.698692	0.493395	0.697308	-0.20391	1	2.18E+08	R	II	LOC10192	TSS200	opensea	TSS200-ope		rs5375190	44;30;21;1
cg2421959	0.225807	0.636172	12.7305	2.1E-06	0.000494	5.697683	0.523269	0.749076	-0.22581	1	1.83E+08	F	II		IGR	opensea	IGR-opensea		rs5752094	8;16;18
cg2194756	0.233709	0.564585	12.72633	2.11E-06	0.000494	5.695332	0.44773	0.68144	-0.23371	20	37650634	R	II	DHX35	Body	opensea	Body-opens		rs3679595	34
cg1996482	0.249557	0.593166	12.72613	2.11E-06	0.000494	5.695217	0.468387	0.717945	-0.24956	2	1.83E+08	F	II	PPP1R1C	Body	opensea	Body-opens		rs5542558	0;11;49
cg1805372	0.217311	0.476139	12.71814	2.12E-06	0.000495	5.690715	0.367484	0.584795	-0.21731	12	47788146	R	II		IGR	opensea	IGR-opensea		rs5340433	45;8;7
cg0287102	0.217997	0.543388	12.71708	2.12E-06	0.000495	5.690115	0.434389	0.652387	-0.218	11	10715715	R	II	MRVI1	TSS1500	opensea	TSS1500-op			
cg2162220	0.206166	0.648093	12.71592	2.12E-06	0.000495	5.689463	0.54501	0.751176	-0.20617	2	1.74E+08	F	II	CDCA7	Body	shelf	Body-shelf	chr2:1742		
cg2417691	0.220849	0.536716	12.71464	2.12E-06	0.000495	5.688741	0.426292	0.64714	-0.22085	12	42522401	R	II	GXYLT1	Body	opensea	Body-opens		rs1425515	2
cg0310911	0.213488	0.471208	12.71211	2.12E-06	0.000495	5.687311	0.364464	0.577952	-0.21349	14	71647081	F	II		IGR	opensea	IGR-opensea		rs4902911	37;51
cg1034232	0.247793	0.470327	12.70981	2.13E-06	0.000495	5.686009	0.346431	0.594224	-0.24779	14	52171238	F	II	FRMD6	Body	opensea	Body-opens		rs5523043	0;1;28;45;50
cg2201555	0.234838	0.529461	12.7091	2.13E-06	0.000495	5.685611	0.412042	0.64688	-0.23484	15	78358486	F	II	TBC1D2B	Body	opensea	Body-opens		rs5443766	1
cg1645443	0.237667	0.588598	12.70646	2.13E-06	0.000495	5.684118	0.469764	0.707432	-0.23767	2	2.36E+08	R	II		IGR	opensea	IGR-opensea		rs5457479	8
cg1507897	0.263562	0.588481	12.70424	2.13E-06	0.000495	5.682866	0.4567	0.720261	-0.26356	11	85958536	F	II	EED	Body	shelf	Body-shelf	chr11:8595	rs7932677	6
cg0093571	0.240072	0.502911	12.70272	2.14E-06	0.000495	5.682008	0.382875	0.622947	-0.24007	8	40432238	F	II	ZMAT4	Body	opensea	Body-opens			
cg0082663	0.302607	0.508574	12.70238	2.14E-06	0.000495	5.681814	0.357271	0.659878	-0.30261	19	46969832	F	II	PNMAL1	3'UTR	opensea	3'UTR-open		rs5392116	0;15
cg1415402	0.246262	0.603506	12.70108	2.14E-06	0.000495	5.68108	0.480375	0.726638	-0.24626	21	26800400	F	II	LINC00158	Body	opensea	Body-opens		rs5343394	4
cg1671391	0.232192	0.519459	12.69961	2.14E-06	0.000495	5.680252	0.403363	0.635555	-0.23219	10	4289162	R	II		IGR	opensea	IGR-opensea		rs3717233	47

cg1377140	0.22391	0.519847	12.69611	2.14E-06	0.000496	5.678271	0.407891	0.631802	-0.22391	15	82172817	R	II		IGR	opensea	IGR-opense		rs1396732	51;33;32;20;11;2
cg1675590	0.232781	0.524934	12.69281	2.15E-06	0.000496	5.676404	0.408543	0.641325	-0.23278	10	44567089	F	II		IGR	opensea	IGR-opense		rs5622879	0
cg1858092	0.208815	0.588474	12.69131	2.15E-06	0.000496	5.675559	0.484066	0.692882	-0.20882	17	45336851	F	II	ITGB3	Body	opensea	Body-opens			
cg2376265	0.216956	0.398367	12.691	2.15E-06	0.000496	5.67538	0.289889	0.506845	-0.21696	1	20914109	R	II	CDA	TSS1500	opensea	TSS1500-op			
cg0325222	0.245974	0.53403	12.68444	2.16E-06	0.000497	5.671671	0.411043	0.657017	-0.24597	9	22214533	R	II		IGR	opensea	IGR-opense		rs5750912	1
cg0022024	0.215656	0.656471	12.68349	2.16E-06	0.000497	5.671133	0.548643	0.764299	-0.21566	15	38164845	F	II		IGR	opensea	IGR-opense			
cg0593378	0.247146	0.468252	12.68264	2.16E-06	0.000497	5.670655	0.344679	0.591825	-0.24715	2	97190408	F	II		IGR	shelf	IGR-shelf	chr2:9719	rs5493021	0;42;45
cg0667392	0.223513	0.487094	12.6816	2.16E-06	0.000497	5.670065	0.375337	0.59885	-0.22351	10	13130374	F	II	CCDC3	5'UTR	opensea	5'UTR-oper		rs5387966	0;5;15
cg1508029	0.203148	0.413418	12.6775	2.17E-06	0.000498	5.667743	0.311844	0.514992	-0.20315	22	27619629	R	II		IGR	opensea	IGR-opense		rs5539595	50;41;25
cg0782625	0.233074	0.530451	12.67558	2.17E-06	0.000498	5.666653	0.413914	0.646988	-0.23307	17	48243707	R	II	SGCA	Body	opensea	Body-opens		rs3755041	47;33
cg0500748	0.241972	0.45237	12.67457	2.17E-06	0.000498	5.666081	0.331384	0.573356	-0.24197	19	31399134	F	II		IGR	opensea	IGR-opense		rs5544075	0;1;44
cg2741724	0.263542	0.558366	12.67059	2.18E-06	0.000498	5.66383	0.426595	0.690137	-0.26354	7	20323695	F	II		IGR	opensea	IGR-opense		rs2529019	10
cg0849255	0.234652	0.497154	12.67042	2.18E-06	0.000498	5.663734	0.379827	0.61448	-0.23465	12	1.11E+08	R	II		IGR	opensea	IGR-opense		rs5707240	20;2
cg1449397	0.201351	0.532065	12.66976	2.18E-06	0.000498	5.663359	0.43139	0.632741	-0.20135	11	63879234	R	II	FLRT1	5'UTR	opensea	5'UTR-oper		rs5441288	37;1
cg2035054	0.231834	0.46953	12.66949	2.18E-06	0.000498	5.663205	0.353612	0.585447	-0.23183	3	1.49E+08	F	II		IGR	opensea	IGR-opense		rs5740788	36
cg2665295	0.258927	0.444013	12.66899	2.18E-06	0.000498	5.662922	0.314549	0.573476	-0.25893	8	24823645	R	II		IGR	opensea	IGR-opense		rs5708701	6;1
cg1169795	0.206444	0.586451	12.6679	2.18E-06	0.000498	5.662307	0.483229	0.689673	-0.20644	2	40332823	F	II	SLC8A1-AS	Body	opensea	Body-opens		rs5636610	18;28;47
cg0723974	0.210106	0.583663	12.66723	2.18E-06	0.000498	5.661924	0.47861	0.688716	-0.21011	4	19757311	F	II		IGR	opensea	IGR-opense		rs1405355	1;14
cg1081194	0.207851	0.60281	12.66721	2.18E-06	0.000498	5.661911	0.498885	0.706736	-0.20785	1	1.57E+08	R	II	SH2D2A	Body	shore	Body-shore	chr1:1567	rs3773306	50;2
cg2730500	0.223813	0.510635	12.66448	2.18E-06	0.000499	5.660367	0.398728	0.622542	-0.22381	2	1.75E+08	R	II	WIPF1	5'UTR	opensea	5'UTR-oper		rs1416899	12
cg0390607	0.228752	0.512524	12.66247	2.19E-06	0.000499	5.659225	0.398148	0.6269	-0.22875	7	22894898	F	II	SNORD93	TSS1500	shore	TSS1500-sh	chr7:2289	rs5768626	5
cg1515884	0.226987	0.564052	12.65968	2.19E-06	0.000499	5.657643	0.450559	0.677545	-0.22699	8	53021899	F	II		IGR	opensea	IGR-opense		rs1503000	0;1;38;45
cg1104285	0.219508	0.55229	12.65842	2.19E-06	0.000499	5.65693	0.442536	0.662045	-0.21951	1	91524842	F	II		IGR	opensea	IGR-opense		rs5461138	16;41
cg1459898	0.206551	0.407444	12.65575	2.19E-06	0.000499	5.655412	0.304169	0.51072	-0.20655	1	68819247	F	II		IGR	opensea	IGR-opense		rs1828373	5;8;47;51
cg0446688	0.215022	0.55163	12.65182	2.2E-06	0.000499	5.653185	0.444119	0.659141	-0.21502	7	19064177	F	II		IGR	opensea	IGR-opense		rs5542940	17;27;47
cg0475630	0.217082	0.465883	12.64775	2.2E-06	0.0005	5.650872	0.357342	0.574424	-0.21708	4	1.71E+08	F	II		IGR	opensea	IGR-opense		rs1435162	43
cg1617579	0.248564	0.514484	12.64722	2.21E-06	0.0005	5.650572	0.390202	0.638766	-0.24856	1	1.2E+08	R	II	HSD3B1	Body	opensea	Body-opens		rs3751422	29;13;12;1
cg0816166	0.209549	0.464146	12.64457	2.21E-06	0.0005	5.649069	0.359372	0.568921	-0.20955	11	13034897	F	II		IGR	shelf	IGR-shelf	chr11:130	rs1895698	2
cg2624913	0.212044	0.507688	12.64222	2.21E-06	0.0005	5.647734	0.401667	0.61371	-0.21204	6	32026147	R	II	TNXB	Body	opensea	Body-opens		rs5362265	49;42;2
cg2385234	0.202761	0.653984	12.63961	2.22E-06	0.000501	5.64625	0.552604	0.755365	-0.20276	11	1.04E+08	R	II	PDGFD	Body	shelf	Body-shelf	chr11:104	rs5611737	50;43;21;13
cg1671935	0.239158	0.488646	12.63698	2.22E-06	0.000501	5.644757	0.369067	0.608225	-0.23916	1	92218200	F	II	TGFBR3	Body	opensea	Body-opens		rs1757348	45
cg0513394	0.23187	0.571421	12.63648	2.22E-06	0.000501	5.644472	0.455486	0.687356	-0.23187	14	1.05E+08	R	II		IGR	opensea	IGR-opense			
cg1902060	0.208346	0.510326	12.63509	2.22E-06	0.000501	5.643683	0.406153	0.614499	-0.20835	2	30974108	F	II	CAPN13	ExonBnd	opensea	ExonBnd-op		rs1820813	0;8;49
cg1494892	0.201624	0.562164	12.63447	2.22E-06	0.000501	5.643327	0.461353	0.662976	-0.20162	17	43453180	F	II		IGR	shelf	IGR-shelf	chr17:434	rs5555452	0
cg2704142	0.223969	0.472952	12.63269	2.22E-06	0.000501	5.642315	0.360968	0.584937	-0.22397	12	6232979	F	II	VWF	5'UTR	opensea	5'UTR-oper		rs1812516	1
cg0071840	0.211779	0.615116	12.63195	2.22E-06	0.000501	5.641895	0.509226	0.721005	-0.21178	6	41131863	R	II	TREM2	TSS1500	opensea	TSS1500-op		rs1382223	42;23;1
cg0992466	0.228895	0.58807	12.63041	2.23E-06	0.000501	5.64102	0.473623	0.702518	-0.22889	1	31976989	F	II		IGR	opensea	IGR-opense		rs5277479	1;12
cg2217812	0.214914	0.590846	12.62833	2.23E-06	0.000501	5.639838	0.483389	0.698303	-0.21491	4	25022049	F	II	LGI2	Body	opensea	Body-opens		rs5368579	0
cg0143989	0.229849	0.505313	12.62661	2.23E-06	0.000501	5.638857	0.390389	0.620238	-0.22985	11	72949060	F	II	P2RY2	3'UTR	shelf	3'UTR-shelf	chr11:729		
cg0753579	0.266184	0.517361	12.62648	2.23E-06	0.000501	5.638782	0.384269	0.650454	-0.26618	3	1.15E+08	R	II	ZBTB20	Body	opensea	Body-opens		rs1060854	3
cg2256631	0.257453	0.520459	12.62193	2.24E-06	0.000501	5.636196	0.391733	0.649186	-0.25745	7	40515317	F	II	SUGCT	Body	opensea	Body-opens			
cg2189452	0.266133	0.608423	12.61818	2.24E-06	0.000502	5.634058	0.475357	0.74149	-0.26613	1	1.85E+08	R	II	FAM129A	Body	opensea	Body-opens		rs5773163	40
cg0819749	0.222219	0.48277	12.61717	2.24E-06	0.000502	5.633483	0.371661	0.593879	-0.22222	11	82838289	R	II		IGR	opensea	IGR-opense		rs1922466	47;38
cg2082269	0.219152	0.519346	12.61687	2.24E-06	0.000502	5.633315	0.40977	0.628922	-0.21915	2	2.31E+08	R	II	SLC16A14	TSS1500	shore	TSS1500-sh	chr2:2309	rs5721295	39;32;9
cg1813195	0.244301	0.592457	12.61492	2.25E-06	0.000502	5.632206	0.470307	0.714608	-0.2443	2	1.06E+08	R	II	FHL2	5'UTR	opensea	5'UTR-oper		rs5466022	16;1
cg0735868	0.216213	0.655715	12.61347	2.25E-06	0.000502	5.631379	0.547609	0.763822	-0.21621	17	7948375	F	II	ALOX15B	Body	opensea	Body-opens		rs5392147	0;1;3;50
cg1255177	0.217089	0.668362	12.61098	2.25E-06	0.000502	5.629958	0.559818	0.776906	-0.21709	13	75939861	R	II	TBC1D4	Body	opensea	Body-opens		rs5343833	37;32;15
cg1454236	0.208311	0.560102	12.61017	2.25E-06	0.000502	5.6295	0.455946	0.664257	-0.20831	9	12704964	R	II	TYRP1	Body	opensea	Body-opens		rs5358841	23
cg0210287	0.207166	0.506376	12.6092	2.25E-06	0.000502	5.628949	0.402793	0.609959	-0.20717	4	79338837	R	II	FRAS1	Body	opensea	Body-opens		rs5371754	51;44;38
cg2599294	0.212709	0.537594	12.60401	2.26E-06	0.000503	5.625989	0.431239	0.643948	-0.21271	4	1.41E+08	R	II	MAML3	Body	opensea	Body-opens		rs1464663	44;16;1
cg0285142	0.225358	0.489295	12.60327	2.26E-06	0.000503	5.625568	0.376616	0.601974	-0.22536	18	75272747	F	II		IGR	opensea	IGR-opense		rs7614663	7;43
cg0170897	0.209443	0.641981	12.59975	2.27E-06	0.000503	5.623559	0.53726	0.746702	-0.20944	2	1.83E+08	R	II		IGR	opensea	IGR-opense		rs5671963	34;7
cg2586738	0.26545	0.51274	12.59951	2.27E-06	0.000503	5.623422	0.380015	0.645464	-0.26545	8	27936225	R	II	NUGGC	5'UTR	opensea	5'UTR-oper		rs5599172	6;2;1
cg0832329	0.22679	0.520682	12.59833	2.27E-06	0.000503	5.622747	0.407287	0.634077	-0.22679	13	80563028	R	II		IGR	opensea	IGR-opense		rs5279103	12;1
cg2507813	0.207686	0.540104	12.59503	2.27E-06	0.000503	5.620866	0.436261	0.643948	-0.20769	17	46698598	R	II	HOXB9	3'UTR	shore	3'UTR-shore	chr17:466	rs5688627	2
cg1615611	0.235752	0.571519	12.59292	2.28E-06	0.000503	5.619665	0.453644	0.689395	-0.23575	2	1.23E+08	R	II		IGR	opensea	IGR-opense		rs1417181	45
cg0991444	0.240396	0.537632	12.58575	2.29E-06	0.000505	5.615571	0.417434	0.65783	-0.2404	1	46972183	F	II	DMBX1	TSS1500	opensea	TSS1500-op		rs5537484	1;31;46
cg1652994	0.246227	0.667358	12.58167	2.29E-06	0.000506	5.613241	0.544244	0.790472	-0.24623	10	98896466	F	II	SLIT1	Body	opensea	Body-opens		rs1427190	0;45
cg1960210	0.263622	0.594133	12.57781	2.3E-06	0.000506	5.611037	0.462321	0.725944	-0.26362	4	90800500	F	II		IGR	opensea	IGR-opense		rs1893177	27
cg0605737	0.219802	0.356256	12.57708	2.3E-06	0.000506	5.610616	0.246355	0.466158	-0.2198	1	89507082	R	II		IGR	opensea	IGR-opense		rs1508799	32
cg0244969	0.200797	0.62793	12.57657	2.3E-06	0.000506	5.610327	0.527532	0.728329	-0.2008	1	1.96E+08	R	II		IGR	opensea	IGR-opense		rs5413744	43;40;1
cg2538832	0.253959	0.513327	12.57473	2.3E-06	0.000506	5.609275	0.386348	0.640307	-0.25396	14	32461050	F	II		IGR	opensea	IGR-opense		rs1497213	11;15
cg2661327	0.23415	0.625189	12.57409	2.3E-06	0.000506	5.608906	0.508114	0.742263	-0.23415	8	1.08E+08	R	II		IGR	opensea	IGR-opense		rs1472481	39;31;7
cg2539299	0.229473	0.540486	12.57375	2.3E-06	0.000506	5.608715	0.425749	0.655222	-0.22947	22	50625926	R	II	TRABD	5'UTR	shore	5'UTR-shore	chr22:506		
cg1129186	0.256129	0.512281	12.56953	2.31E-06	0.000506	5.606303	0.384216	0.640345	-0.25613	2	1.74E+08	R	II		IGR	opensea	IGR-opense		rs7640536	31;28;11;1
cg0625205	0.235811	0.578027	12.56474	2.31E-06	0.000507	5.603561	0.460121	0.695932	-0.23581	11	68923361	F	I	LOC33869	Body	shore	Body-shore	chr11:689	rs5729407	26;28;31;43
cg0030342	0.213642	0.462376	12.5639	2.31E-06	0.000507	5.603084	0.355555	0.569197	-0.21364	2	2.18E+08	F	II		IGR	opensea	IGR-opense		rs1505899	44
cg0481141	0.270663	0.377747	12.56221	2.32E-06	0.000507	5.602113	0.242415	0.513079	-0.27066	1	1.74E+08	R	II	SNORD80	TSS1500	shelf	TSS1500-sh	chr1:1738	rs5374956	24;7;2
cg2432934	0.214069	0.596028	12.56167	2.32E-06	0.000507	5.601803	0.488994	0.703063	-0.21407	5	7822907	R	II	ADCY2	Body	opensea	Body-opens		rs5331038	2
cg1282317	0.224919	0.604897	12.5609	2.32E-06	0.000507	5.601364	0.492438	0.717357	-0.22492	16	78930322	R	II	WWOX	Body	opensea	Body-opens		rs5395586	37;32;26;17
cg0684445	0.217064	0.608791	12.55983	2.32E-06	0.000507	5.600753	0.50026	0.717323	-0.21706	2	1.59E+08	F	II	CCDC148-A	TSS1500	opensea	TSS1500-op		rs1441109	1;49
cg0905838	0.211538	0.562116	12.55781	2.32E-06	0.000507	5.599596	0.456347	0.667885	-0.21154	13	1.09E+08	R	II		IGR	opensea	IGR-opense		rs5449910	50;49;44;15;1
cg0717553	0.267491	0.482076	12.55558	2.33E-06	0.000507	5.59832	0.34833	0.615821	-0.26749	16	15991691	F	II		IGR	opensea	IGR-opense		rs5528229	0
cg1301362	0.205858	0.708509	12.5555	2.33E-06	0.000507	5.598274	0.60558	0.811438	-0.20586	1	1.7E+08	R	II		IGR	shelf	IGR-shelf	chr1:1700	rs5300714	9;1
cg1114179	0.247364	0.550155	12.5486	2.34E-06	0.000507	5.594319	0.426473	0.673837	-0.24736	4	57967183	R	II	IGFBP7	Body	opensea	Body-opens		rs5537207	2
cg0757910	0.223241	0.584804	12.5465	2.34E-06	0.000508	5.593119	0.473184	0.696424	-0.22324	15	53910786	R	II	WDR72	Body	opensea	Body-opens		rs5676651	44;42
cg2068419	0.25413	0.466197	12.54426	2.34E-06	0.000508	5.591834	0.339133	0.593262	-0.25413	5	1.42E+08	F	II	FGF1	5'UTR	opensea	5'UTR-oper		rs1880151	47;51
cg2223911	0.213543	0.556464	12.53952	2.35E-06	0.000509	5.589116	0.449693	0.663236	-0.21354	4	92370268	F	II	CCSER1	Body	opensea	Body-opens		rs5644317	0;26
cg0581245	0.204197	0.45632	12.53634	2.35E-06	0.000509	5.587292	0.354221	0.558418	-0.2042	3	1.68E+08	F	II	EGFEM1P	Body	opensea	Body-opens		rs5344868	17;31
cg2702232	0.232298	0.623245	12.53609	2.35E-06	0.000509	5.58715	0.507096	0.739394	-0.2323	14	1.02E+08	F	II	PPP2R5C	3'UTR	shore	3'UTR-shore	chr14:102	rs1850586	1;8;29;46
cg0955115	0.246208	0.54509	12.53539	2.35E-06	0.000509	5.586745	0.421986	0.668194	-0.24621	1	2.1E+08	F	II		IGR	opensea	IGR-opense		rs5762574	0;10;41
cg0423459	0.252878	0.541693	12.53191	2.36E-06	0.00051	5.584752	0.415254	0.668132	-0.25288	5	3851581	R	II		IGR	shelf	IGR-shelf	chr5:3854	rs5374055	6
cg0361901	0.225221	0.508768	12.52805	2.36E-06	0.000511	5.582533	0.396157	0.621378	-0.22522	18	53536662	F	II		IGR	opensea	IGR-opense			
cg0660205	0.204337	0.453552	12.5272	2.36E-06	0.000511	5.582048	0.351384	0.555721	-0.20434	10	73409742	F	II	CDH23	Body	opensea	Body-opens		rs5289018	0;8;23
cg1689091	0.221402	0.395161	12.52411	2.37E-06	0.000511	5.580274	0.28446	0.505861	-0.2214	5	1.25E+08	F	II		IGR	opensea	IGR-opense		rs1470751	0
cg1178407	0.239952	0.573303	12.52073	2.37E-06	0.000512	5.578331	0.453327	0.693279	-0.23995	7	79125480	R	II		IGR	opensea	IGR-opense		rs5515014	21;13;2;1
cg1800712	0.251887	0.594361	12.51939	2.38E-06	0.000512	5.577565	0.468417	0.720304	-0.25189	3	1.55E+08	F	II	PLCH1	TSS1500	opensea	TSS1500-op			
cg0649086	0.251881	0.538774	12.51347	2.38E-06	0.000513	5.57416	0.412833	0.664715	-0.25188	10	79725233	R	II		IGR	opensea	IGR-opense			
cg2018704	0.264481	0.514816	12.51306	2.38E-06	0.000513	5.573924	0.382575	0.647056	-0.26448	3	28982939	R	II		IGR	opensea	IGR-opense			
cg1538547	0.214711	0.592852	12.51214	2.39E-06	0.000513	5.573398	0.485496	0.700207	-0.21471	1	1.96E+08	R	II		IGR	opensea	IGR-opense			
cg0830795	0.206084	0.629745	12.50753	2.39E-06	0.000513	5.570744	0.526703	0.732786	-0.20608	15	99329769	R	II	IGF1R	Body	opensea	Body-opens		rs3756299	13;11
cg2618953	0.241471	0.473909	12.49579	2.41E-06	0.000515	5.563988	0.353173	0.594644	-0.24147	18	56791106	F	II		IGR	opensea	IGR-opense			
cg0524182	0.253849	0.540464	12.49576	2.41E-06	0.000515	5.56397	0.41354	0.667388	-0.25385	4	85648511	R	II	WDFY3	Body	opensea	Body-opens		rs5579855	48;36
cg2493700	0.210555	0.526129	12.49535	2.41E-06	0.000515	5.563738	0.420851	0.631407	-0.21056	17	56783942	F	II	RAD51C	Body	opensea	Body-opens		rs5504479	29
cg1495224	0.219388	0.624306	12.49419	2.41E-06	0.000515	5.563068	0.514612	0.734	-0.21939	12	1.17E+08	R	II		IGR	opensea	IGR-opense		rs1834661	32;23;1
cg2108053	0.224569	0.514207	12.49278	2.41E-06	0.000515	5.562258	0.401923	0.626492	-0.22457	6	49755105	R	II	PGK2	TSS200	opensea	TSS200-ope		rs1470929	49;33;18
cg1213475	0.23816	0.419045	12.49271	2.41E-06	0.000515	5.562219	0.299965	0.538125	-0.23816	19	5249431	R	II	PTPRS	Body	shore	Body-shore	chr19:525		
cg0327843	0.200946	0.54	12.49117	2.41E-06	0.000515	5.561328	0.439526	0.640473	-0.20095	4	1.83E+08	R	II		IGR	opensea	IGR-opense		rs5444408	47;27
cg1568714	0.205882	0.522156	12.48854	2.42E-06	0.000515	5.559816	0.419215	0.625097	-0.20588	4	91570600	F	II	FAM190A	Body	opensea	Body-opens		rs5290941	1;47
cg0355697	0.246435	0.557998	12.48751	2.42E-06	0.000515	5.55922	0.43478	0.681215	-0.24643	13	24249366	F	II	TNFRSF19	3'UTR	opensea	3'UTR-oper		rs5426259	0
cg0951245	0.217757	0.46521	12.48728	2.42E-06	0.000515	5.559091	0.356332	0.574088	-0.21776	11	62120239	R	II	ASRGL1	Body	opensea	Body-opens			
cg1020569	0.204774	0.597685	12.486	2.42E-06	0.000515	5.558348	0.495298	0.700072	-0.20477	3	1.73E+08	R	II	NLGN1	Body	opensea	Body-opens		rs6776920	50;1
cg0429245	0.228971	0.521562	12.48508	2.42E-06	0.000515	5.55782	0.407076	0.636047	-0.22897	5	14444521	R	II	TRIO	Body	opensea	Body-opens		rs5448693	39;16
cg1645512	0.202692	0.482575	12.48497	2.42E-06	0.000515	5.557759	0.381229	0.583921	-0.20269	19	54903669	F	II		IGR	opensea	IGR-opense		rs5679651	14;15;36
cg1004051	0.201082	0.466119	12.48408	2.42E-06	0.000515	5.557244	0.365578	0.56666	-0.20108	2	2.06E+08	F	II	PARD3B	Body	opensea	Body-opens		rs1413005	13;16
cg1264556	0.264082	0.4391	12.47896	2.43E-06	0.000516	5.554295	0.307059	0.571141	-0.26408	1	51339561	R	II	FAF1	Body	opensea	Body-opens		rs1921246	37;2
cg1748173	0.202864	0.542883	12.47789	2.43E-06	0.000516	5.553674	0.441451	0.644315	-0.20286	11	1.17E+08	R	II		IGR	opensea	IGR-opense		rs1807190	24;14
cg0959775	0.242602	0.410443	12.47733	2.43E-06	0.000516	5.553351	0.289142	0.531744	-0.2426	14	74162875	R	II	DNAL1	3'UTR	opensea	3'UTR-oper		rs8003105	32;30;27
cg0828850	0.218432	0.620501	12.47702	2.43E-06	0.000516	5.553172	0.511285	0.729717	-0.21843	3	16889869	F	II		IGR	opensea	IGR-opense		rs5722420	14;41
cg2664967	0.203645	0.494674	12.47159	2.44E-06	0.000516	5.55004	0.392852	0.596497	-0.20365	2	2.29E+08	R	II	SLC19A3	3'UTR	opensea	3'UTR-oper		rs1129571	50;29
cg0897367	0.200001	0.372167	12.4677	2.45E-06	0.000517	5.547795	0.272167	0.472168	-0.2	3	87210807	R	II		IGR	opensea	IGR-opense			
cg1039390	0.202352	0.530198	12.4673	2.45E-06	0.000517	5.547567	0.429023	0.631374	-0.20235	12	1.11E+08	F	II		IGR	opensea	IGR-opense		rs5428273	0;18
cg1555249	0.20922	0.469008	12.46148	2.46E-06	0.000518	5.544202	0.364398	0.573618	-0.20922	2	1.02E+08	R	II	MAP4K4	Body	opensea	Body-opens		rs5380878	2
cg0016345	0.275269	0.562105	12.4596	2.46E-06	0.000518	5.543119	0.42447	0.699739	-0.27527	20	10411528	R	II	MKKS	5'UTR	shelf	5'UTR-shelf	chr20:104		
cg1946604	0.239532	0.454322	12.45685	2.46E-06	0.000518	5.541532	0.334556	0.574088	-0.23953	12	1.19E+08	F	II	TAOK3	5'UTR	opensea	5'UTR-oper			
cg0239230	0.207988	0.393278	12.45581	2.47E-06	0.000518	5.54093	0.289284	0.497272	-0.20799	10	99781741	F	II	CRTAC1	Body	opensea	Body-opens		rs5558096	3;23;49
cg1559208	0.237297	0.512323	12.45335	2.47E-06	0.000518	5.539507	0.393674	0.630971	-0.2373	16	49683803	R	II	ZNF423	Body	opensea	Body-opens		rs7593493	37;25
cg1450006	0.219897	0.66865	12.45086	2.47E-06	0.000518	5.538071	0.558702	0.778599	-0.2199	5	82656554	F	II		IGR	opensea	IGR-opense		rs1480356	11;31;39;46
cg1377996	0.210725	0.553228	12.45083	2.47E-06	0.000518	5.538052	0.447866	0.658591	-0.21072	6	17903072	R	II	KIF13A	Body	opensea	Body-opens		rs5383663	46;1

cg0627179	0.213381	0.621143	12.44768	2.48E-06	0.000519	5.53623	0.514452	0.727833	-0.21338	1	84409003	F	II	TTLL7	Body	opensea	Body-opens		rs7646997	46;51
cg1969380	0.207074	0.643129	12.44719	2.48E-06	0.000519	5.535946	0.539592	0.746666	-0.20707	5	1.19E+08	R	II	TNFAIP8	TSS1500	opensea	TSS1500-op		rs5747966	34;11;1
cg2230094	0.250403	0.541894	12.44503	2.48E-06	0.000519	5.534696	0.416692	0.667095	-0.2504	3	35710038	F	II	ARPP21	5'UTR	opensea	5'UTR-oper			
cg2104493	0.214199	0.52488	12.44265	2.48E-06	0.000519	5.533323	0.41778	0.63198	-0.2142	14	58442152	F	II		IGR	opensea	IGR-opense		rs5449148	0;11;25;37
cg2663244	0.250156	0.463162	12.44105	2.49E-06	0.00052	5.532396	0.338084	0.58824	-0.25016	11	18302340	F	II	HPS5	Body	opensea	Body-opens			
cg0834360	0.26319	0.501879	12.43946	2.49E-06	0.00052	5.531472	0.370284	0.633474	-0.26319	10	43894095	F	II	HNRNPF	5'UTR	shore	5'UTR-shor	chr10:4389	rs5523722	1;13;17;37
cg2103363	0.259234	0.528491	12.43734	2.49E-06	0.00052	5.530245	0.398874	0.658107	-0.25923	5	64486421	F	II	ADAMTS6	Body	opensea	Body-opens		rs5699740	1;22
cg0449678	0.240996	0.497538	12.43618	2.49E-06	0.00052	5.529578	0.37704	0.618036	-0.241	3	31741190	F	II	OSBPL10	Body	opensea	Body-opens		rs1130016	37
cg0075719	0.26821	0.430957	12.43574	2.49E-06	0.00052	5.52932	0.296853	0.565062	-0.26821	3	1.85E+08	F	II	IGF2BP2	Body	opensea	Body-opens		rs1811918	1
cg0928642	0.225735	0.467096	12.43357	2.5E-06	0.00052	5.528067	0.354229	0.579964	-0.22574	4	55339975	F	II		IGR	opensea	IGR-opense		rs1414050	0;1;20
cg0691891	0.205693	0.555117	12.43331	2.5E-06	0.00052	5.527917	0.452271	0.657964	-0.20569	10	11547855	R	II	USP6NL	Body	opensea	Body-opens		rs5579903	34;1
cg1678976	0.228999	0.398346	12.43162	2.5E-06	0.00052	5.526938	0.283847	0.512846	-0.229	14	89066213	F	II	ZC3H14	Body	opensea	Body-opens		rs7145665	48
cg1951536	0.260771	0.523776	12.43002	2.5E-06	0.00052	5.526007	0.39339	0.654161	-0.26077	3	1.56E+08	R	II	KCNAB1	Body	opensea	Body-opens		rs5580411	37;36;12;2;1
cg1174325	0.247048	0.508282	12.4299	2.5E-06	0.00052	5.525942	0.384758	0.631806	-0.24705	4	14784843	F	II	LINC00504	Body	opensea	Body-opens		rs5578152	0;40;50
cg2547879	0.245118	0.617235	12.42646	2.51E-06	0.00052	5.523945	0.494676	0.739794	-0.24512	8	1.41E+08	R	II	TRAPPC9	Body	opensea	Body-opens		rs5703298	34;30;10
cg1090054	0.22304	0.568329	12.41919	2.52E-06	0.000521	5.519734	0.456809	0.679849	-0.22304	4	1.69E+08	R	II	PALLD	Body	opensea	Body-opens		rs1127052	2
cg0808941	0.207767	0.608147	12.41916	2.52E-06	0.000521	5.519718	0.504263	0.71203	-0.20777	7	1.2E+08	F	II		IGR	opensea	IGR-opense			
cg0010324	0.212288	0.601808	12.41735	2.52E-06	0.000521	5.518668	0.495664	0.707952	-0.21229	1	2.37E+08	R	II	RYR2	Body	opensea	Body-opens		rs5638006	29;21
cg0434091	0.23324	0.506804	12.41547	2.52E-06	0.000521	5.517576	0.390185	0.623424	-0.23324	8	19577028	R	II		IGR	opensea	IGR-opense		rs1495708	42
cg2528383	0.242189	0.536256	12.4127	2.53E-06	0.000521	5.515968	0.415161	0.65735	-0.24219	11	92132845	R	II	FAT3	Body	opensea	Body-opens			
cg0188619	0.202733	0.512253	12.41148	2.53E-06	0.000521	5.515259	0.410886	0.613619	-0.20273	13	73976090	R	II		IGR	opensea	IGR-opense		rs1147952	12;3;1
cg0668961	0.234635	0.668711	12.41056	2.53E-06	0.000521	5.514725	0.551394	0.786029	-0.23463	4	99935464	R	II	METAP1	Body	opensea	Body-opens		rs5309003	29;2;1
cg1953867	0.220691	0.536598	12.4096	2.53E-06	0.000521	5.514173	0.426253	0.646944	-0.22069	3	27575726	F	II		IGR	opensea	IGR-opense		rs3737936	13;32;33
cg1460992	0.210018	0.466089	12.40537	2.54E-06	0.000522	5.511718	0.36108	0.571098	-0.21002	1	2.2E+08	R	II		IGR	opensea	IGR-opense			
cg0106085	0.223554	0.447959	12.40147	2.54E-06	0.000522	5.509452	0.336182	0.559736	-0.22355	6	37227779	R	II	TBC1D22B	Body	shelf	Body-shelf	chr6:3722		
cg2251201	0.22576	0.480548	12.40021	2.55E-06	0.000522	5.508722	0.367668	0.593428	-0.22576	13	77402717	F	II		IGR	opensea	IGR-opense		rs1473678	0;1
cg2144677	0.215174	0.55146	12.3977	2.55E-06	0.000522	5.507264	0.443873	0.659047	-0.21517	2	2.23E+08	R	II		IGR	opensea	IGR-opense		rs5305531	2
cg1520078	0.20877	0.520322	12.39446	2.56E-06	0.000523	5.505378	0.415937	0.624707	-0.20877	10	79285311	R	II	KCNMA1	Body	opensea	Body-opens			
cg2488790	0.202975	0.58608	12.39423	2.56E-06	0.000523	5.505243	0.484593	0.687568	-0.20298	12	4124630	F	II		IGR	opensea	IGR-opense		rs5618800	4;14
cg2101092	0.208418	0.628453	12.39296	2.56E-06	0.000523	5.504506	0.524245	0.732662	-0.20842	11	44659065	F	II		IGR	opensea	IGR-opense		rs1390885	0
cg2255650	0.246123	0.479453	12.38881	2.56E-06	0.000523	5.502094	0.356392	0.602515	-0.24612	7	1.48E+08	R	II	CNTNAP2	Body	opensea	Body-opens		rs5278384	48;2
cg1487909	0.276103	0.545629	12.38747	2.57E-06	0.000523	5.501317	0.407578	0.683681	-0.2761	1	1.85E+08	F	II	FAM129A	Body	opensea	Body-opens		rs5294297	1;4;11;24;44
cg0701441	0.219573	0.366244	12.38361	2.57E-06	0.000523	5.49907	0.256458	0.47603	-0.21957	2	38396113	R	II	CYP1B1-AS	Body	opensea	Body-opens		rs5480533	25;1
cg0946822	0.201448	0.387245	12.3813	2.58E-06	0.000524	5.497725	0.286521	0.487969	-0.20145	2	2.32E+08	R	II		IGR	opensea	IGR-opense		rs3714545	50;5
cg1884269	0.206791	0.568422	12.37924	2.58E-06	0.000524	5.496527	0.465026	0.671817	-0.20679	11	60112913	F	II		IGR	opensea	IGR-opense		rs3724330	20;38
cg1297382	0.210184	0.566646	12.37868	2.58E-06	0.000524	5.496203	0.461555	0.671738	-0.21018	10	73795916	F	II		IGR	opensea	IGR-opense		rs5462227	1
cg2534578	0.206916	0.375548	12.37719	2.58E-06	0.000524	5.495337	0.27209	0.479005	-0.20692	8	50425076	F	II		IGR	opensea	IGR-opense		rs1327952	1;16;41
cg1074958	0.208217	0.590457	12.37524	2.58E-06	0.000524	5.494199	0.486349	0.694566	-0.20822	7	80253343	R	II	CD36	5'UTR	opensea	5'UTR-oper		rs5513450	19;17;14
cg0560042	0.227391	0.551183	12.37478	2.59E-06	0.000524	5.493932	0.437488	0.664879	-0.22739	11	30597981	F	II	MPPED2	Body	opensea	Body-opens		rs5697113	0
cg2178638	0.200263	0.370602	12.37466	2.59E-06	0.000524	5.493861	0.270471	0.470734	-0.20026	5	1.48E+08	F	II		IGR	opensea	IGR-opense		rs5457259	51
cg2605851	0.205763	0.536245	12.36751	2.6E-06	0.000525	5.489701	0.433363	0.639127	-0.20576	15	88260042	F	II		IGR	opensea	IGR-opense			
cg0840302	0.224714	0.593299	12.36527	2.6E-06	0.000525	5.488393	0.480942	0.705656	-0.22471	15	65361131	F	II	RASL12	TSS1500	shore	TSS1500-sh	chr15:653	rs5352876	10;16;41;47
cg1838544	0.24868	0.419003	12.36448	2.6E-06	0.000525	5.487935	0.294663	0.543343	-0.24868	10	1.06E+08	R	II	GSTO2	TSS1500	shore	TSS1500-sh	chr10:106	rs5625682	24;15;13
cg0880667	0.223182	0.563143	12.36307	2.6E-06	0.000525	5.487109	0.451552	0.674733	-0.22318	10	80383717	F	II		IGR	opensea	IGR-opense		rs1418948	1;38;44
cg2389549	0.220283	0.495515	12.36202	2.6E-06	0.000525	5.486502	0.385373	0.605656	-0.22028	7	47539837	F	II	TNS3	5'UTR	opensea	5'UTR-oper			
cg0048206	0.211553	0.628561	12.36139	2.61E-06	0.000525	5.486135	0.522784	0.734337	-0.21155	1	61478983	F	II		IGR	opensea	IGR-opense		rs1486563	23;29
cg0932503	0.243934	0.486739	12.36026	2.61E-06	0.000525	5.485476	0.364772	0.608706	-0.24393	17	32307434	R	II	ASIC2	Body	opensea	Body-opens		rs5307188	37;36;2
cg0255072	0.201134	0.48105	12.36001	2.61E-06	0.000525	5.485327	0.380484	0.581617	-0.20113	6	32048679	F	II	TNXB	Body	shore	Body-shore	chr6:3204	rs1389520	1
cg2044907	0.224741	0.489844	12.35992	2.61E-06	0.000525	5.485276	0.377473	0.602214	-0.22474	7	10658637	F	II		IGR	opensea	IGR-opense		rs1139827	0;10;18;51
cg1323720	0.287302	0.487719	12.35808	2.61E-06	0.000525	5.484202	0.344068	0.63137	-0.2873	13	98192250	F	II		IGR	opensea	IGR-opense		rs5746394	1
cg0086445	0.207718	0.63226	12.35435	2.62E-06	0.000525	5.482028	0.528401	0.736119	-0.20772	8	56985462	R	II	RPS20	Body	shore	Body-shore	chr8:5698	rs1164726	51;42;13
cg2380665	0.251271	0.499377	12.35203	2.62E-06	0.000526	5.480675	0.373741	0.625013	-0.25127	7	1.11E+08	R	II	IMMP2L	Body	opensea	Body-opens		rs1150644	46
cg0563579	0.206217	0.59036	12.35121	2.62E-06	0.000526	5.480197	0.487252	0.693469	-0.20622	18	573825	R	II		IGR	opensea	IGR-opense		rs5600412	43
cg2710004	0.243035	0.537942	12.34881	2.63E-06	0.000526	5.478796	0.416424	0.659459	-0.24304	20	49311875	F	II		IGR	opensea	IGR-opense		rs5286891	0;1;2;26;26;29
cg1268735	0.240221	0.564837	12.34571	2.63E-06	0.000526	5.476989	0.444726	0.684947	-0.24022	3	6937063	R	II	GRM7-AS2	TSS1500	opensea	TSS1500-op		rs5580675	21
cg1803815	0.281335	0.511564	12.34541	2.63E-06	0.000526	5.476812	0.370896	0.652232	-0.28134	8	1.04E+08	R	II		IGR	opensea	IGR-opense			
cg1564307	0.220577	0.51464	12.34533	2.63E-06	0.000526	5.476764	0.404352	0.624929	-0.22058	12	22562854	R	II		IGR	opensea	IGR-opense		rs5322045	42
cg0687157	0.201285	0.470421	12.34513	2.63E-06	0.000526	5.476648	0.369779	0.571064	-0.20129	1	1.12E+08	R	II	LINC01160	Body	opensea	Body-opens		rs5553117	11;2
cg0541703	0.234743	0.457906	12.34371	2.63E-06	0.000527	5.475819	0.340534	0.575277	-0.23474	2	18150633	F	II		IGR	opensea	IGR-opense		rs5776842	30
cg0380295	0.241732	0.512445	12.34214	2.64E-06	0.000527	5.474904	0.391579	0.633311	-0.24173	2	45411645	R	II	LINC01121	Body	opensea	Body-opens		rs1703916	24;5;1
cg1077746	0.221666	0.649971	12.34051	2.64E-06	0.000527	5.473948	0.539138	0.760804	-0.22167	5	1.25E+08	R	II		IGR	opensea	IGR-opense			
cg0355649	0.26673	0.56953	12.33488	2.65E-06	0.000528	5.470664	0.436165	0.702895	-0.26673	3	1.85E+08	F	II	EHHADH	Body	opensea	Body-opens		rs5299869	32
cg0601916	0.244891	0.603591	12.33329	2.65E-06	0.000528	5.469735	0.481146	0.726037	-0.24489	6	1.56E+08	R	II		IGR	opensea	IGR-opense		rs1138755	49;24
cg0247627	0.245291	0.604817	12.33312	2.65E-06	0.000528	5.469631	0.482171	0.727462	-0.24529	1	1.56E+08	R	II		IGR	opensea	IGR-opense		rs1842924	50
cg1983404	0.242951	0.538451	12.33255	2.65E-06	0.000528	5.469301	0.416975	0.659926	-0.24295	3	1.6E+08	R	II	SCHIP1	Body	opensea	Body-opens		rs5479757	49;46;36;19;1
cg1692288	0.220131	0.496381	12.33241	2.65E-06	0.000528	5.469219	0.386315	0.606446	-0.22013	3	14825290	R	II		IGR	opensea	IGR-opense		rs829192;r	38;21;9
cg2423732	0.227931	0.532526	12.33109	2.65E-06	0.000528	5.468444	0.41856	0.646491	-0.22793	14	70007511	R	II		IGR	opensea	IGR-opense		rs1441235	27
cg1066239	0.260166	0.573891	12.32967	2.65E-06	0.000528	5.467619	0.443808	0.703974	-0.26017	1	24046776	R	II		IGR	opensea	IGR-opense		rs1150834	14;2
cg2025632	0.205895	0.58252	12.32882	2.66E-06	0.000528	5.467119	0.479572	0.685468	-0.2059	11	28679066	R	II		IGR	opensea	IGR-opense		rs1882407	14;7
cg0239364	0.235696	0.457564	12.32867	2.66E-06	0.000528	5.467031	0.339716	0.575412	-0.2357	7	1.36E+08	F	II	LUZP6	5'UTR	shore	5'UTR-shor	chr7:1356	rs5641132	29
cg0078623	0.204065	0.483373	12.32714	2.66E-06	0.000528	5.466137	0.381341	0.585405	-0.20406	3	1.54E+08	R	II		IGR	shelf	IGR-shelf	chr3:1541	rs7798497	48;26;23;14;2
cg2313423	0.200742	0.487504	12.32515	2.66E-06	0.000528	5.464975	0.387133	0.587875	-0.20074	3	1.5E+08	R	II		IGR	opensea	IGR-opense		rs5615278	37;2;1
cg0039025	0.216548	0.611953	12.31651	2.68E-06	0.00053	5.459917	0.503679	0.720227	-0.21655	3	50241300	F	II	SLC38A3	TSS1500	shore	TSS1500-sh	chr3:5024	rs5761197	0
cg1785788	0.210174	0.397567	12.31441	2.68E-06	0.00053	5.458691	0.292481	0.502654	-0.21017	19	49993008	R	II	RPL13A	5'UTR	shore	5'UTR-shor	chr19:499	rs5284760	42;12;6
cg0538088	0.22203	0.526454	12.31221	2.68E-06	0.00053	5.457402	0.415439	0.637469	-0.22203	1	1.83E+08	F	II	NMNAT2	Body	opensea	Body-opens		rs5776214	23
cg2496545	0.235914	0.543552	12.30994	2.69E-06	0.00053	5.456073	0.425595	0.661509	-0.23591	2	7578495	F	II	LOC10050	Body	opensea	Body-opens		rs5498317	46
cg1930614	0.20827	0.595256	12.30928	2.69E-06	0.00053	5.455683	0.491121	0.699391	-0.20827	7	1.34E+08	F	II	EXOC4	Body	opensea	Body-opens		rs5314338	0
cg1513737	0.225318	0.517683	12.30893	2.69E-06	0.00053	5.455481	0.405023	0.630342	-0.22532	12	23729510	F	II	SOX5	Body	opensea	Body-opens		rs1860090	0;1
cg1828500	0.229857	0.548129	12.30711	2.69E-06	0.00053	5.454414	0.433201	0.663058	-0.22986	10	11873962	R	II	PROSER2	5'UTR	opensea	5'UTR-oper		rs5371935	14
cg2454471	0.215585	0.485697	12.30678	2.69E-06	0.00053	5.454222	0.377905	0.59349	-0.21558	14	88339690	R	II		IGR	opensea	IGR-opense		rs5602415	48
cg2395696	0.20483	0.549083	12.30597	2.69E-06	0.00053	5.453745	0.446668	0.651498	-0.20483	7	79758274	F	II		IGR	opensea	IGR-opense		rs5459771	0
cg1482573	0.237685	0.485278	12.30553	2.69E-06	0.00053	5.453486	0.366436	0.604121	-0.23768	5	1.42E+08	R	II	FGF1	TSS1500	opensea	TSS1500-op			
cg1407641	0.215168	0.5994	12.30421	2.7E-06	0.00053	5.452714	0.491816	0.706984	-0.21517	5	43412601	F	II	CCL28	TSS200	opensea	TSS200-ope		rs3750556	3;7;27;34;51
cg2025600	0.206695	0.671997	12.30399	2.7E-06	0.00053	5.452586	0.568649	0.775344	-0.2067	1	1.53E+08	F	II	S100A8	5'UTR	opensea	5'UTR-oper		rs1495738	1;27;42
cg1641519	0.220811	0.668711	12.29909	2.7E-06	0.000531	5.449712	0.558305	0.779116	-0.22081	17	33408053	R	II	RFFL	Body	opensea	Body-opens		rs1828087	31
cg0017029	0.206268	0.471043	12.29724	2.71E-06	0.000531	5.448632	0.367909	0.574177	-0.20627	6	46720370	F	II	LOC10028	Body	opensea	Body-opens		rs1817823	0
cg0790031	0.24423	0.607542	12.29696	2.71E-06	0.000531	5.448465	0.485427	0.729657	-0.24423	22	36362528	R	II	RBFOX2	Body	opensea	Body-opens		rs5418011	51;27;4;1
cg0376422	0.22834	0.517414	12.29558	2.71E-06	0.000531	5.44766	0.403245	0.631584	-0.22834	20	30942880	F	II		IGR	shelf	IGR-shelf	chr20:309	rs1437152	26;38
cg1230017	0.245085	0.508877	12.28666	2.72E-06	0.000532	5.442424	0.386335	0.63142	-0.24508	3	70362865	R	II		IGR	opensea	IGR-opense		rs5688947	51;29;24;21;1
cg1647310	0.232974	0.539595	12.28417	2.73E-06	0.000533	5.440961	0.423108	0.656082	-0.23297	5	1.31E+08	F	II		IGR	opensea	IGR-opense		rs5610944	1;23;30
cg0693457	0.208021	0.630089	12.28358	2.73E-06	0.000533	5.440614	0.526078	0.734099	-0.20802	2	9458925	R	II	ASAP2	Body	shore	Body-shore	chr2:9459		
cg0131990	0.233848	0.666414	12.28333	2.73E-06	0.000533	5.440472	0.54949	0.783338	-0.23385	1	1.14E+08	F	II		IGR	opensea	IGR-opense			
cg0424368	0.234655	0.634274	12.2812	2.73E-06	0.000533	5.43922	0.516946	0.751601	-0.23466	6	86395706	F	II		IGR	opensea	IGR-opense		rs5544356	0;1;7;25;39
cg0276710	0.209644	0.640319	12.2811	2.73E-06	0.000533	5.439161	0.535497	0.745141	-0.20964	10	34793792	F	II	PARD3	Body	opensea	Body-opens		rs5367342	0;41;42;43
cg1669858	0.252207	0.580304	12.27989	2.73E-06	0.000533	5.438452	0.454201	0.706407	-0.25221	9	1.08E+08	R	II		IGR	shelf	IGR-shelf	chr9:1077		
cg0281326	0.229539	0.540774	12.27958	2.73E-06	0.000533	5.438265	0.426005	0.655544	-0.22954	2	10670665	R	II		IGR	shelf	IGR-shelf	chr2:1066	rs1641139	49;1
cg1400128	0.226494	0.531996	12.2795	2.74E-06	0.000533	5.438219	0.418749	0.645243	-0.22649	14	72978188	F	II	RGS6	Body	opensea	Body-opens		rs1883641	15;29;37
cg0665891	0.200745	0.606135	12.27683	2.74E-06	0.000533	5.436651	0.505762	0.706507	-0.20074	6	1.18E+08	R	II	ROS1	TSS1500	opensea	TSS1500-op		rs1475969	1
cg0002527	0.205026	0.591446	12.27607	2.74E-06	0.000533	5.436206	0.488933	0.693958	-0.20503	11	17429271	R	II	ABCC8	Body	opensea	Body-opens		rs5732361	50;8;1
cg2513297	0.24542	0.632035	12.27162	2.75E-06	0.000534	5.433591	0.509325	0.754745	-0.24542	12	94403370	R	II		IGR	opensea	IGR-opense		rs1924857	35
cg2366092	0.202084	0.569045	12.27128	2.75E-06	0.000534	5.43339	0.468002	0.670087	-0.20208	1	2.42E+08	F	II		IGR	shelf	IGR-shelf	chr1:2420	rs5758907	0;1;13;14
cg2249308	0.2076	0.634006	12.2671	2.76E-06	0.000535	5.430933	0.530206	0.737807	-0.2076	5	1.58E+08	R	II	EBF1	Body	opensea	Body-opens		rs5387253	31;2
cg0871775	0.216121	0.444665	12.26534	2.76E-06	0.000535	5.429898	0.336604	0.552726	-0.21612	13	37881008	F	II		IGR	opensea	IGR-opense		rs1912434	1;44
cg0715804	0.228777	0.564995	12.26425	2.76E-06	0.000535	5.42926	0.450606	0.679383	-0.22878	2	1.91E+08	R	II	HIBCH	TSS1500	shore	TSS1500-sh	chr2:1911		
cg2270285	0.203996	0.409891	12.26311	2.76E-06	0.000535	5.428588	0.307893	0.511889	-0.204	12	4269985	R	II		IGR	shelf	IGR-shelf	chr12:427	rs5466354	6;1
cg1102966	0.244584	0.53274	12.261	2.77E-06	0.000536	5.427347	0.410448	0.655032	-0.24458	11	1.32E+08	F	II		IGR	opensea	IGR-opense		rs5676464	0;1;32
cg1911501	0.209289	0.530326	12.25989	2.77E-06	0.000536	5.426693	0.425682	0.634971	-0.20929	11	15229502	F	II	INSC	Body	opensea	Body-opens		rs7675458	33
cg0386177	0.235437	0.571214	12.25728	2.77E-06	0.000536	5.425156	0.453496	0.688932	-0.23544	3	31796070	F	II	OSBPL10	Body	opensea	Body-opens		rs5748557	20
cg1606257	0.213063	0.383079	12.25607	2.77E-06	0.000536	5.424444	0.276547	0.48961	-0.21306	12	69491491	F	II		IGR	opensea	IGR-opense		rs3717578	1;47
cg0142010	0.255844	0.506703	12.25602	2.77E-06	0.000536	5.424415	0.378781	0.634625	-0.25584	5	94237541	R	II	MCTP1	Body	opensea	Body-opens		rs1440062	13;2
cg1603809	0.20087	0.57005	12.25535	2.77E-06	0.000536	5.424023	0.469615	0.670486	-0.20087	15	79087784	R	II	ADAMTS7	Body	opensea	Body-opens			
cg2453800	0.236743	0.587984	12.25501	2.78E-06	0.000536	5.423819	0.469612	0.706356	-0.23674	1	17558947	R	II	PADI1	Body	opensea	Body-opens		rs5626957	39;30;29;2
cg0265937	0.269913	0.648779	12.25299	2.78E-06	0.000536	5.422632	0.513823	0.783736	-0.26991	2	1.91E+08	F	II	PMS1	ExonBnd	opensea	ExonBnd-op			
cg0390213	0.210192	0.621044	12.25042	2.78E-06	0.000536	5.421121	0.515949	0.72614	-0.21019	3	13672669	R	II	FBLN2	Body	opensea	Body-opens		rs5566371	51;50;41;2
cg0675529	0.237002	0.536865	12.24955	2.78E-06	0.000536	5.420604	0.418364	0.655366	-0.237	15	98199925	F	II		IGR	shelf	IGR-shelf	chr15:981	rs7999971	15;19
cg0461659	0.20963	0.56061	12.24567	2.79E-06	0.000537	5.418322	0.455795	0.665425	-0.20963	8	50042978	R	II		IGR	opensea	IGR-opense		rs5632845	34;6
cg2237196	0.238726	0.476012	12.24393	2.79E-06	0.000537	5.417294	0.356649	0.595375	-0.23873	1	1.69E+08	F	II	NME7	Body	opensea	Body-opens		rs5699895	0;1
cg1035105	0.208634	0.547429	12.2439	2.79E-06	0.000537	5.417281	0.443112	0.651746	-0.20863	5	39703340	F	II		IGR	opensea	IGR-opense		rs5773205	2;29
cg2570899	0.206253	0.576515	12.23746	2.8E-06	0.000538	5.413481	0.473388	0.679641	-0.20625	1	2.03E+08	F	II	PRELP	TSS1500	opensea	TSS1500-op		rs5530721	50

cg0179622	0.236555	0.603257	12.23574	2.81E-06	0.000538	5.412465	0.48498	0.721535	-0.23656	11	89954258	R	II	CHORDC1	Body	shore	Body-shore	chr11:899!	rs5706306	47;44;37;28;26;6
cg2013488	0.218818	0.451993	12.23569	2.81E-06	0.000538	5.412439	0.342584	0.561402	-0.21882	7	5622551	F	II		IGR	opensea	IGR-opensea		rs5409319	0;1
cg2461595	0.213903	0.397419	12.23156	2.81E-06	0.000539	5.410003	0.290468	0.504371	-0.2139	17	29253798	R	II	ADAP2	Body	shelf	Body-shelf	chr17:292	rs5402375	7
cg2089185	0.2211	0.632743	12.22915	2.82E-06	0.000539	5.40858	0.522193	0.743293	-0.2211	10	1.19E+08	R	II	SHTN1	Body	opensea	Body-opens		rs5321389	47;15;2
cg2757160	0.208153	0.514008	12.22828	2.82E-06	0.000539	5.408069	0.409931	0.618084	-0.20815	10	80722277	R	II	ZMIZ1-AS1	Body	opensea	Body-opens		rs7563087	17;16;2
cg0891979	0.239217	0.453332	12.22354	2.83E-06	0.00054	5.405268	0.333724	0.572941	-0.23922	1	1.74E+08	F	II	RABGAP1L	5'UTR	opensea	5'UTR-oper			
cg0664451	0.217834	0.389214	12.21981	2.83E-06	0.000541	5.403066	0.280297	0.49813	-0.21783	1	1.74E+08	R	II	SNORD47	TSS1500	shelf	TSS1500-sh	chr1:1738	rs5368350	15
cg0875273	0.242298	0.438388	12.21798	2.84E-06	0.000541	5.401985	0.31724	0.559537	-0.2423	22	32313898	F	II		IGR	opensea	IGR-opensea		rs5339811	28;35;43
cg1526895	0.200055	0.42035	12.21547	2.84E-06	0.000541	5.400504	0.320322	0.520377	-0.20005	2	1.89E+08	F	II	GULP1	Body	opensea	Body-opens		rs5755538	26
cg0141639	0.235949	0.43484	12.21455	2.84E-06	0.000541	5.399961	0.316865	0.552814	-0.23595	20	55806433	R	II	BMP7	Body	opensea	Body-opens		rs5555885	36;14
cg2354251	0.217122	0.539415	12.21161	2.85E-06	0.000542	5.398222	0.430854	0.647976	-0.21712	7	1.18E+08	R	II		IGR	opensea	IGR-opensea		rs5408492	2
cg0224285	0.246352	0.581036	12.21052	2.85E-06	0.000542	5.39758	0.45786	0.704212	-0.24635	10	13768464	F	II	FRMD4A	Body	opensea	Body-opens			
cg1244399	0.206424	0.524657	12.20727	2.86E-06	0.000542	5.395658	0.421445	0.627868	-0.20642	10	52610612	R	II	A1CF	Body	opensea	Body-opens		rs5371524	22;3
cg1775502	0.238958	0.589534	12.20421	2.86E-06	0.000543	5.393851	0.470055	0.709013	-0.23896	3	1.89E+08	F	II		IGR	opensea	IGR-opensea		rs5613843	14;18;46;47
cg1817038	0.211565	0.523393	12.19526	2.88E-06	0.000544	5.388554	0.417611	0.629175	-0.21156	10	330411	F	II	DIP2C	Body	opensea	Body-opens		rs5610125	2;25
cg2440171	0.256821	0.522577	12.19233	2.88E-06	0.000545	5.386819	0.394166	0.650987	-0.25682	9	71532723	F	II	PIP5K1B	Body	opensea	Body-opens		rs1398280	1;16;31
cg0865681	0.235735	0.370794	12.19085	2.88E-06	0.000545	5.385945	0.252926	0.488661	-0.23573	17	34207454	R	II	CCL5	TSS200	opensea	TSS200-ope		rs2280788	51;50
cg0521588	0.219898	0.589723	12.19035	2.88E-06	0.000545	5.385649	0.479774	0.699672	-0.2199	15	58670807	R	II		IGR	opensea	IGR-opensea		rs5281284	16;14;1
cg1809126	0.245548	0.601247	12.18979	2.89E-06	0.000545	5.385318	0.478473	0.724021	-0.24555	17	63822838	R	II	CCDC46	TSS200	opensea	TSS200-ope		rs5713764	14;12;10;9;2;1
cg0501921	0.203837	0.432558	12.18567	2.89E-06	0.000545	5.382877	0.330639	0.534476	-0.20384	22	33343603	F	II	SYN3	Body	opensea	Body-opens		rs5635371	11
cg1171126	0.228436	0.535625	12.18531	2.89E-06	0.000545	5.382661	0.421407	0.649843	-0.22844	19	528571	F	II		IGR	shelf	IGR-shelf	chr19:531	rs5740459	0;20;33
cg2427808	0.20482	0.609228	12.18508	2.89E-06	0.000545	5.382525	0.506818	0.711637	-0.20482	11	65904369	R	II	PACS1	Body	opensea	Body-opens		rs5650765	2
cg0923660	0.226378	0.380235	12.18162	2.9E-06	0.000546	5.380473	0.267046	0.493424	-0.22638	7	35559711	F	II		IGR	opensea	IGR-opensea			
cg0969010	0.216992	0.431615	12.18046	2.9E-06	0.000546	5.379788	0.323119	0.540111	-0.21699	4	14603924	R	II		IGR	opensea	IGR-opensea			
cg2572386	0.201543	0.629675	12.17919	2.9E-06	0.000546	5.379038	0.528904	0.730446	-0.20154	15	50483002	F	II	SLC27A2	Body	opensea	Body-opens		rs5668995	51
cg2666567	0.205011	0.553539	12.1787	2.9E-06	0.000546	5.378747	0.451034	0.656045	-0.20501	5	1.38E+08	F	II		IGR	opensea	IGR-opensea		rs5485519	32;44
cg1984081	0.211214	0.498876	12.17776	2.91E-06	0.000546	5.378186	0.393269	0.604483	-0.21121	10	80764672	F	II	ZMIZ1-AS1	Body	opensea	Body-opens			
cg0796525	0.250566	0.525478	12.17556	2.91E-06	0.000546	5.376883	0.400195	0.650761	-0.25057	15	38342973	R	II	LOC10192	Body	opensea	Body-opens			
cg0541049	0.204128	0.471636	12.1746	2.91E-06	0.000546	5.376311	0.369572	0.5737	-0.20413	6	56825978	R	II	BEND6	5'UTR	opensea	5'UTR-oper			
cg2009047	0.205458	0.469586	12.1726	2.91E-06	0.000546	5.375126	0.366857	0.572315	-0.20546	2	9403086	R	II	ASAP2	Body	opensea	Body-opens		rs5320819	33
cg1837953	0.217095	0.417058	12.17176	2.92E-06	0.000546	5.374632	0.30851	0.525605	-0.21709	20	17973048	F	II		IGR	opensea	IGR-opensea		rs5660656	12
cg2691356	0.261361	0.576276	12.17014	2.92E-06	0.000546	5.37367	0.445595	0.706956	-0.26136	5	1.73E+08	F	II	C5orf41	Body	opensea	Body-opens		rs5624478	15;21
cg2303242	0.208791	0.502722	12.16969	2.92E-06	0.000546	5.373399	0.398326	0.607117	-0.20879	3	3152038	F	II	IL5RA	5'UTR	opensea	5'UTR-oper		rs5736176	6
cg0744005	0.204661	0.347628	12.16752	2.92E-06	0.000546	5.372114	0.245297	0.449958	-0.20466	17	69899186	R	II		IGR	opensea	IGR-opensea		rs9898912	35;24
cg1531947	0.248254	0.515692	12.16686	2.92E-06	0.000546	5.371723	0.391565	0.639819	-0.24825	12	62546183	R	II	FAM19A2	5'UTR	opensea	5'UTR-oper			
cg2580792	0.220283	0.607604	12.16661	2.93E-06	0.000546	5.371572	0.497463	0.717745	-0.22028	14	47663593	R	II	MDGA2	Body	opensea	Body-opens		rs1455626	48;41;36;16;6
cg2639179	0.235601	0.419946	12.16608	2.93E-06	0.000546	5.371258	0.302146	0.537747	-0.2356	13	49323899	R	II		IGR	opensea	IGR-opensea		rs1917753	40
cg0195007	0.221815	0.469225	12.16515	2.93E-06	0.000546	5.370708	0.358317	0.580133	-0.22182	6	1.1E+08	F	II		IGR	shore	IGR-shore	chr6:1096	rs3700363	16;29;37
cg1170089	0.248238	0.482404	12.1642	2.93E-06	0.000546	5.370143	0.358285	0.606523	-0.24824	7	1.55E+08	R	II		IGR	opensea	IGR-opensea			
cg0785156	0.273308	0.537373	12.16367	2.93E-06	0.000546	5.36983	0.400719	0.674026	-0.27331	18	48695911	R	II		IGR	opensea	IGR-opensea			
cg2145022	0.204167	0.654055	12.16364	2.93E-06	0.000546	5.369813	0.551971	0.756138	-0.20417	3	23727711	R	II		IGR	opensea	IGR-opensea		rs5640644	2
cg1873597	0.238349	0.485458	12.16058	2.94E-06	0.000546	5.367993	0.366283	0.604632	-0.23835	1	2.08E+08	R	II	CD46	Body	shelf	Body-shelf	chr1:2079	rs1178757	26;1
cg0932086	0.227054	0.634582	12.1603	2.94E-06	0.000546	5.367829	0.521055	0.748109	-0.22705	5	1.09E+08	R	II		IGR	shore	IGR-shore	chr5:1092	rs1890273	2;1
cg0496677	0.217962	0.572944	12.15856	2.94E-06	0.000546	5.366795	0.463963	0.681925	-0.21796	20	35483358	F	II	SOGA1	Body	opensea	Body-opens		rs3768439	0
cg0332694	0.241463	0.610122	12.15625	2.94E-06	0.000546	5.365423	0.489391	0.730854	-0.24146	9	1E+08	F	II	LOC28635	TSS1500	opensea	TSS1500-op		rs5340745	1;5;45
cg0192661	0.215973	0.416114	12.15586	2.94E-06	0.000546	5.365192	0.308127	0.524101	-0.21597	11	1.16E+08	F	II		IGR	opensea	IGR-opensea		rs5650529	0
cg2007303	0.205045	0.551951	12.15554	2.94E-06	0.000546	5.364998	0.449429	0.654474	-0.20504	16	8774580	F	II	ABAT	5'UTR	opensea	5'UTR-oper		rs5518878	7
cg2509012	0.260069	0.537504	12.15491	2.95E-06	0.000546	5.364627	0.407469	0.667539	-0.26007	17	43407096	R	II		IGR	opensea	IGR-opensea		rs5696653	40;39;1
cg0593990	0.218608	0.522763	12.15458	2.95E-06	0.000546	5.36443	0.413459	0.632067	-0.21861	11	45346903	R	II		IGR	opensea	IGR-opensea		rs5518508	44;2
cg0212291	0.206158	0.657535	12.15255	2.95E-06	0.000546	5.363225	0.554456	0.760614	-0.20616	2	30359207	R	II		IGR	opensea	IGR-opensea		rs5507642	23
cg0842731	0.203749	0.618538	12.14947	2.96E-06	0.000547	5.361394	0.516664	0.720413	-0.20375	4	1.42E+08	F	II	RNF150	Body	opensea	Body-opens		rs5696306	0;10;18
cg0823910	0.203061	0.55444	12.14535	2.96E-06	0.000547	5.358942	0.452909	0.65597	-0.20306	11	16623818	F	II		IGR	shelf	IGR-shelf	chr11:166	rs5465365	1;39
cg0009078	0.20963	0.550506	12.1452	2.96E-06	0.000547	5.358855	0.445691	0.655321	-0.20963	15	66795345	R	II	RPL4	Body	opensea	Body-opens		rs5646791	50;32
cg2389809	0.23995	0.628644	12.14476	2.96E-06	0.000547	5.358595	0.508669	0.748619	-0.23995	6	1.67E+08	R	II		IGR	opensea	IGR-opensea		rs5578234	2
cg1992710	0.207975	0.499048	12.14453	2.96E-06	0.000547	5.358459	0.39506	0.603035	-0.20797	15	68902085	R	II	CORO2B	Body	opensea	Body-opens		rs5333926	49;15;2
cg0085442	0.219566	0.570402	12.14449	2.96E-06	0.000547	5.358431	0.460619	0.680185	-0.21957	7	65520870	R	II		IGR	opensea	IGR-opensea		rs5778363	39
cg1358352	0.200536	0.565613	12.14393	2.97E-06	0.000547	5.358102	0.465345	0.665882	-0.20054	5	24764190	R	II		IGR	opensea	IGR-opensea			
cg0603513	0.206492	0.476777	12.13972	2.97E-06	0.000548	5.355598	0.373531	0.580023	-0.20649	8	93108556	F	II	RUNX1T1	TSS1500	opensea	TSS1500-op		rs5609561	15
cg2346182	0.255009	0.578695	12.13712	2.98E-06	0.000548	5.354049	0.451191	0.7062	-0.25501	7	1.35E+08	R	II	CALD1	Body	opensea	Body-opens			
cg1989077	0.201012	0.662263	12.13449	2.98E-06	0.000548	5.352481	0.561757	0.762768	-0.20101	11	1.23E+08	R	II		IGR	opensea	IGR-opensea		rs1121227	41;3
cg2232520	0.210499	0.469394	12.13425	2.98E-06	0.000548	5.352342	0.364145	0.574644	-0.2105	20	48785237	R	II		IGR	opensea	IGR-opensea			
cg2469337	0.216969	0.605211	12.13327	2.98E-06	0.000548	5.351758	0.496726	0.713695	-0.21697	10	77541764	R	II	C10orf11	TSS1500	opensea	TSS1500-op		rs1862959	11;2;1
cg0966893	0.235338	0.557725	12.13007	2.99E-06	0.000549	5.349852	0.440056	0.675394	-0.23534	1	49196418	F	II	BEND5	Body	opensea	Body-opens			
cg0680522	0.210791	0.587612	12.12961	2.99E-06	0.000549	5.349579	0.482217	0.693008	-0.21079	12	1.31E+08	F	II	RIMBP2	5'UTR	opensea	5'UTR-oper		rs5522926	0;37;46
cg1313973	0.200369	0.618316	12.12835	2.99E-06	0.000549	5.34883	0.518132	0.7185	-0.20037	1	1.92E+08	F	II		IGR	opensea	IGR-opensea		rs5450720	1;22;32
cg0801055	0.207291	0.455191	12.1276	2.99E-06	0.000549	5.348381	0.351545	0.558837	-0.20729	22	26146031	R	II	MYO18B	5'UTR	opensea	5'UTR-oper		rs1447005	24
cg2195874	0.231671	0.553926	12.125	3E-06	0.000549	5.346831	0.438091	0.669761	-0.23167	12	79832188	F	II	SYT1	Body	opensea	Body-opens			
cg2070167	0.229786	0.418033	12.12407	3E-06	0.000549	5.34628	0.30314	0.532927	-0.22979	2	64782942	F	II	AFTPH	Body	opensea	Body-opens		rs5682482	9;20
cg0238148	0.20732	0.513849	12.12395	3E-06	0.000549	5.346204	0.410189	0.617509	-0.20732	10	80679729	F	II		IGR	opensea	IGR-opensea			
cg0742707	0.2778	0.481477	12.12293	3E-06	0.000549	5.345599	0.342577	0.620377	-0.2778	3	1.89E+08	F	II		IGR	opensea	IGR-opensea		rs5750090	10
cg1950469	0.217489	0.514203	12.12196	3E-06	0.000549	5.345024	0.405458	0.622948	-0.21749	10	98474121	R	II	PIK3AP1	Body	opensea	Body-opens		rs5557920	33;33;19
cg0763889	0.249841	0.474905	12.12162	3.01E-06	0.000549	5.344816	0.349984	0.599825	-0.24984	5	1.24E+08	R	II	ZNF608	Body	opensea	Body-opens		rs5313074	31
cg2344467	0.232893	0.534221	12.12117	3.01E-06	0.000549	5.344548	0.417774	0.650667	-0.23289	9	96617890	R	II		IGR	opensea	IGR-opensea			
cg0017471	0.215157	0.514657	12.11938	3.01E-06	0.00055	5.343486	0.407079	0.622236	-0.21516	6	47136110	R	II		IGR	opensea	IGR-opensea		rs5596330	41;5;2
cg0192938	0.217716	0.51999	12.11808	3.01E-06	0.00055	5.34271	0.411132	0.628848	-0.21772	12	71631626	F	II		IGR	opensea	IGR-opensea		rs7539868	1;20
cg0364650	0.207039	0.510269	12.11705	3.01E-06	0.00055	5.342092	0.406749	0.613788	-0.20704	3	16145303	R	II		IGR	opensea	IGR-opensea			
cg2709656	0.247135	0.566219	12.11563	3.02E-06	0.00055	5.341249	0.442651	0.689787	-0.24714	1	33419301	R	II	RNF19B	Body	opensea	Body-opens		rs5327542	2;1
cg0720394	0.205368	0.664066	12.11195	3.02E-06	0.00055	5.339055	0.561382	0.76675	-0.20537	4	99976972	R	II	METAP1	Body	opensea	Body-opens		rs3678976	16;1
cg1311613	0.210417	0.598986	12.11144	3.02E-06	0.00055	5.33875	0.493777	0.704194	-0.21042	12	51582638	R	II	POU6F1	3'UTR	opensea	3'UTR-oper		rs5439318	50;43;14
cg1017229	0.220793	0.632069	12.10665	3.03E-06	0.00055	5.335889	0.521673	0.742465	-0.22079	2	2.01E+08	F	II	SPATS2L	5'UTR	opensea	5'UTR-oper			
cg0519290	0.209227	0.562459	12.10596	3.03E-06	0.00055	5.335479	0.457845	0.667072	-0.20923	6	25910691	R	II		IGR	opensea	IGR-opensea		rs7671058	36;2;1
cg1583832	0.206097	0.631338	12.10489	3.04E-06	0.00055	5.334839	0.52829	0.734387	-0.2061	20	49461129	R	II	BCAS4	Body	shelf	Body-shelf	chr20:494	rs1873228	44;7
cg2119784	0.207274	0.678413	12.10473	3.04E-06	0.00055	5.334747	0.574776	0.78205	-0.20727	8	86959593	R	II		IGR	opensea	IGR-opensea		rs5749833	46;7;2
cg1359080	0.217783	0.436984	12.10446	3.04E-06	0.00055	5.334582	0.328093	0.545876	-0.21778	18	57369634	R	II		IGR	opensea	IGR-opensea		rs5288077	1
cg2047508	0.21573	0.604116	12.10311	3.04E-06	0.00055	5.333778	0.496251	0.711981	-0.21573	2	1.29E+08	R	II		IGR	opensea	IGR-opensea		rs1390073	2
cg0919961	0.240566	0.437716	12.10286	3.04E-06	0.00055	5.33363	0.317432	0.557999	-0.24057	5	56946761	R	II	LOC10192	Body	opensea	Body-opens		rs5338328	1
cg2071048	0.2158	0.614861	12.09643	3.05E-06	0.000551	5.329789	0.506961	0.722761	-0.2158	4	42462320	R	II	ATP8A1	Body	opensea	Body-opens		rs1118653	13;3;1
cg2509657	0.226646	0.650194	12.09515	3.05E-06	0.000552	5.329023	0.536871	0.763517	-0.22665	14	74937693	F	II		IGR	opensea	IGR-opensea			
cg1406739	0.234502	0.494627	12.09489	3.05E-06	0.000552	5.328867	0.377376	0.611878	-0.2345	22	37644539	F	II		IGR	opensea	IGR-opensea		rs1504825	0
cg2557668	0.215602	0.63567	12.09244	3.06E-06	0.000552	5.327406	0.527869	0.743471	-0.2156	6	1.32E+08	R	II	ENPP3	Body	opensea	Body-opens		rs1492663	43;40;39;18;17;10
cg0893605	0.203981	0.60137	12.08901	3.06E-06	0.000553	5.325354	0.49938	0.703361	-0.20398	12	4511796	F	II		IGR	opensea	IGR-opensea			
cg1106907	0.23027	0.479784	12.0889	3.06E-06	0.000553	5.325291	0.364649	0.594919	-0.23027	14	1.02E+08	R	II	MIR496	TSS1500	opensea	TSS1500-op		rs1176270	43
cg1212026	0.279491	0.458537	12.08663	3.07E-06	0.000553	5.323932	0.318791	0.598283	-0.27949	5	1.26E+08	R	II	ALDH7A1	Body	opensea	Body-opens		rs1437451	0;29;32
cg0516987	0.21437	0.473963	12.08035	3.08E-06	0.000554	5.320174	0.366778	0.581147	-0.21437	12	96199841	F	II		IGR	opensea	IGR-opensea		rs1736836	41
cg0101951	0.226831	0.590834	12.07922	3.08E-06	0.000554	5.3195	0.477418	0.70425	-0.22683	18	36276187	R	II		IGR	opensea	IGR-opensea			
cg0561296	0.200177	0.476298	12.0787	3.08E-06	0.000554	5.319189	0.376209	0.576386	-0.20018	10	88106244	F	II	GRID1	Body	opensea	Body-opens		rs1854215	1;6;17;19
cg2326487	0.208037	0.411891	12.06508	3.11E-06	0.000557	5.311033	0.307873	0.51591	-0.20804	13	92210112	R	II	GPC5	Body	opensea	Body-opens		rs1469974	37
cg0044676	0.221403	0.55837	12.06428	3.11E-06	0.000557	5.310558	0.447668	0.669072	-0.2214	3	1.25E+08	R	II	MUC13	3'UTR	opensea	3'UTR-oper		rs5483293	50;18;2
cg2736557	0.248259	0.546134	12.0642	3.11E-06	0.000557	5.310505	0.422005	0.670263	-0.24826	2	1.47E+08	F	II		IGR	opensea	IGR-opensea		rs5407420	0;18;51
cg1073478	0.251062	0.491828	12.0636	3.11E-06	0.000557	5.310151	0.366297	0.617359	-0.25106	8	19833012	F	II		IGR	opensea	IGR-opensea		rs5616270	0;14;49
cg0074614	0.262798	0.60214	12.06343	3.11E-06	0.000557	5.310044	0.470741	0.733539	-0.2628	20	39551744	F	II		IGR	opensea	IGR-opensea		rs1864539	47
cg1517559	0.218491	0.552643	12.05739	3.12E-06	0.000557	5.306426	0.443397	0.661888	-0.21849	20	11833806	F	II	LINC00687	Body	opensea	Body-opens			
cg0314052	0.217327	0.628353	12.0568	3.12E-06	0.000557	5.30607	0.51969	0.737017	-0.21733	18	70663038	R	II		IGR	opensea	IGR-opensea		rs5652413	34;2
cg2353300	0.203933	0.625384	12.05305	3.13E-06	0.000557	5.303825	0.523418	0.727351	-0.20393	11	1.12E+08	R	II		IGR	opensea	IGR-opensea			
cg1103558	0.220697	0.398658	12.05175	3.13E-06	0.000557	5.303043	0.288309	0.509006	-0.2207	21	33613069	R	II		IGR	opensea	IGR-opensea		rs1490327	1
cg2278616	0.209135	0.498925	12.05154	3.13E-06	0.000557	5.302917	0.394357	0.603492	-0.20914	7	82701979	R	II	PCLO	Body	opensea	Body-opens		rs5489647	1
cg0016906	0.235428	0.514651	12.05144	3.13E-06	0.000557	5.302857	0.396938	0.632365	-0.23543	12	66089047	R	II		IGR	opensea	IGR-opensea		rs1117586	27;7
cg2070828	0.273959	0.567427	12.05075	3.14E-06	0.000557	5.302445	0.430448	0.704407	-0.27396	5	1.27E+08	R	II		IGR	opensea	IGR-opensea		rs1147652	1
cg1503632	0.2259	0.466817	12.05063	3.14E-06	0.000557	5.302371	0.353867	0.579766	-0.2259	17	27048708	F	II	RPL23A	Body	shore	Body-shore	chr17:270	rs1418104	1
cg1581418	0.210182	0.718516	12.04989	3.14E-06	0.000557	5.301931	0.613425	0.823607	-0.21018	7	39726374	F	II	RALA	ExonBnd	opensea	ExonBnd-op			
cg1254855	0.200584	0.634691	12.04761	3.14E-06	0.000557	5.300558	0.534399	0.734983	-0.20058	2	42411128	F	II	EML4	Body	opensea	Body-opens		rs1394893	0;40;42
cg1441216	0.235154	0.597356	12.04739	3.14E-06	0.000557	5.300429	0.479779	0.714933	-0.23515	4	81957210	R	II	BMP3	Body	opensea	Body-opens		rs1003156	26;1
cg2053821	0.250902	0.58313	12.04536	3.15E-06	0.000557	5.29921	0.457679	0.708581	-0.2509	4	58027287	R	II		IGR	shelf	IGR-shelf	chr4:5802	rs1493992	47;46;35;2
cg1256916	0.202785	0.538537	12.04205	3.15E-06	0.000557	5.297225	0.437144	0.639929	-0.20279	3	1.75E+08	R	II	NAALADL2-	TSS1500	opensea	TSS1500-op			
cg1770905	0.245289	0.503836	12.04187	3.15E-06	0.000557	5.297117	0.381192	0.62648	-0.24529	4	95238351	F	II	HPGDS	Body	opensea	Body-opens		rs5526325	5;22
cg0231666	0.218106	0.687738	12.04187	3.15E-06	0.000557	5.297113	0.578684	0.796791	-0.21811	3	1.82E+08	R	II		IGR	opensea	IGR-opensea		rs923341;r	49;42;1
cg1048965	0.200002	0.385393	12.04164	3.15E-06	0.000557	5.296978	0.285392	0.485394	-0.2	18	3512188	R	II	DLGAP1	Body	opensea	Body-opens			
cg0532314	0.208722	0.568131	12.04092	3.15E-06	0.000557	5.296546	0.46377	0.672492	-0.20872	20	52592404	F	II	BCAS1	Body	opensea	Body-opens		rs1899145	1;8
cg0799602	0.267326	0.553334	12.04086	3.16E-06	0.000557	5.29651	0.419671	0.686997	-0.26733	12	6270392	R	II		IGR	opensea	IGR-opensea		rs5383760	44;43;28;22;13

cg1342383	0.263861	0.46737	12.04077	3.16E-06	0.000557	5.296456	0.335439	0.5993	-0.26386	10	4818881	F	II		IGR	opensea	IGR-opense		rs1408021	31
cg0792195	0.217977	0.652935	12.03844	3.16E-06	0.000557	5.295055	0.543947	0.761924	-0.21798	12	14107199	F	II	GRIN2B	5'UTR	opensea	5'UTR-open		rs5560964	19
cg0922012	0.212553	0.490527	12.03535	3.17E-06	0.000558	5.293201	0.384251	0.596803	-0.21255	1	1.17E+08	F	II	CD58	Body	opensea	Body-opens			
cg0186272	0.212735	0.643861	12.03129	3.17E-06	0.000559	5.290761	0.537494	0.750229	-0.21274	1	1.58E+08	R	II	CD1D	TSS1500	shore	TSS1500-sh	chr1:1581	rs5510497	10
cg1784747	0.220681	0.519745	12.03121	3.17E-06	0.000559	5.29071	0.409405	0.630086	-0.22068	16	78802628	F	II	WWOX	Body	opensea	Body-opens		rs1460566	45
cg1973227	0.230078	0.419499	12.03084	3.17E-06	0.000559	5.290492	0.30446	0.534538	-0.23008	17	8856818	F	II	PIK3R5	5'UTR	opensea	5'UTR-open			
cg0623543	0.21818	0.445353	12.02823	3.18E-06	0.000559	5.288921	0.336263	0.554443	-0.21818	16	30485966	F	II	ITGAL	Body	shore	Body-shore	chr16:3048	rs5437776	3;47
cg2620159	0.204316	0.482325	12.02786	3.18E-06	0.000559	5.288699	0.380167	0.584483	-0.20432	10	766041	F	II		IGR	opensea	IGR-opense		rs5445642	18;22;44
cg2594416	0.224231	0.644002	12.02533	3.18E-06	0.000559	5.287179	0.531886	0.756117	-0.22423	2	88862221	F	II	EIF2AK3	Body	opensea	Body-opens		rs1997527	20;40
cg2481332	0.227451	0.477129	12.02287	3.19E-06	0.000559	5.285698	0.363404	0.590855	-0.22745	12	63242527	F	II	PPM1H	Body	opensea	Body-opens		rs7912170	1;40
cg2573412	0.225604	0.672882	12.0215	3.19E-06	0.000559	5.284876	0.56008	0.785684	-0.2256	2	1.67E+08	R	II	LOC10192	Body	opensea	Body-opens		rs6008473	37;2
cg0707109	0.214264	0.5916	12.02013	3.19E-06	0.00056	5.284051	0.484468	0.698732	-0.21426	14	58461392	F	II		IGR	opensea	IGR-opense		rs2747095	11
cg1268281	0.265449	0.468144	12.01724	3.2E-06	0.00056	5.282309	0.335419	0.600869	-0.26545	2	1.69E+08	R	II	STK39	Body	opensea	Body-opens		rs1861657	42
cg1115656	0.23489	0.460331	12.01689	3.2E-06	0.00056	5.282102	0.342886	0.577776	-0.23489	17	42816286	R	II	DBF4B	Body	opensea	Body-opens		rs1495850	38;15
cg0585947	0.214885	0.540297	12.01621	3.2E-06	0.00056	5.281694	0.432855	0.647739	-0.21488	21	42600919	F	II	BACE2	Body	opensea	Body-opens		rs5976047	15;51
cg1722059	0.20614	0.587018	12.00688	3.22E-06	0.000562	5.276076	0.483949	0.690088	-0.20614	4	26522849	R	II		IGR	opensea	IGR-opense		rs5767219	2
cg1959835	0.204645	0.635278	12.00669	3.22E-06	0.000562	5.275958	0.532955	0.7376	-0.20465	20	33556046	F	II	MYH7B	5'UTR	opensea	5'UTR-open		rs5312238	0
cg1908300	0.208342	0.636919	12.00299	3.23E-06	0.000563	5.27373	0.532748	0.741091	-0.20834	6	46293862	R	II	RCAN2	TSS1500	opensea	TSS1500-op		rs1155800	49;25
cg1630950	0.20009	0.579968	12.00189	3.23E-06	0.000563	5.273066	0.479923	0.680013	-0.20009	12	60651018	F	II		IGR	opensea	IGR-opense		rs5403461	19;49
cg1018119	0.210857	0.530891	12.00172	3.23E-06	0.000563	5.272964	0.425462	0.636319	-0.21086	1	56840643	R	II		IGR	opensea	IGR-opense		rs1437660	48
cg0245852	0.218028	0.622507	12.0014	3.23E-06	0.000563	5.272774	0.513493	0.731521	-0.21803	14	24166813	R	II		IGR	opensea	IGR-opense		rs6071922	50;35;1
cg0789347	0.203803	0.528012	11.99918	3.24E-06	0.000563	5.271431	0.42611	0.629913	-0.2038	3	1.96E+08	R	II	C3orf43	TSS200	opensea	TSS200-ope		rs5492922	1
cg1587121	0.202602	0.565779	11.99815	3.24E-06	0.000563	5.270815	0.464478	0.66708	-0.2026	7	37939591	R	II	NME8	3'UTR	opensea	3'UTR-open		rs5691420	3;2;1
cg0909719	0.24294	0.432298	11.994	3.25E-06	0.000564	5.26831	0.310829	0.553768	-0.24294	22	26145960	R	II	MYO18B	5'UTR	opensea	5'UTR-open		rs5439446	2
cg0129997	0.240807	0.593403	11.99277	3.25E-06	0.000564	5.267568	0.473	0.713807	-0.24081	1	1.87E+08	R	II		IGR	opensea	IGR-opense		rs5354874	50;1
cg0857356	0.204452	0.485056	11.98976	3.25E-06	0.000565	5.265753	0.38283	0.587282	-0.20445	6	1.6E+08	F	II		IGR	opensea	IGR-opense		rs9457586	18;33
cg0135953	0.233642	0.456588	11.98973	3.25E-06	0.000565	5.265733	0.339767	0.573409	-0.23364	15	75045126	F	II	CYP1A2	Body	opensea	Body-opens		rs5685129	13;19;22
cg2082131	0.252891	0.48948	11.98869	3.26E-06	0.000565	5.265108	0.363035	0.615926	-0.25289	2	10950732	F	II	PDIA6	Body	shore	Body-shore	chr2:1095	rs1136054	10
cg1027487	0.241138	0.297094	11.98545	3.26E-06	0.000566	5.26315	0.176525	0.417663	-0.24114	18	57469286	F	II		IGR	opensea	IGR-opense		rs5566038	0;2;34;47
cg1695968	0.261129	0.548178	11.98107	3.27E-06	0.000566	5.260511	0.417613	0.678742	-0.26113	12	77350297	R	II		IGR	opensea	IGR-opense		rs7754387	32;8
cg1456679	0.201229	0.619814	11.97966	3.27E-06	0.000566	5.259656	0.519199	0.720428	-0.20123	20	20186234	R	II	CFAP61	Body	opensea	Body-opens			
cg0093423	0.203829	0.653234	11.97833	3.28E-06	0.000567	5.258854	0.55132	0.755149	-0.20383	8	1.04E+08	R	II	BAALC	Body	opensea	Body-opens		rs5275041	2;1
cg1927272	0.209455	0.493808	11.97381	3.29E-06	0.000567	5.256123	0.38908	0.598535	-0.20946	1	2.04E+08	R	II	PLEKHA6	5'UTR	opensea	5'UTR-open		rs3704024	23
cg0182513	0.213166	0.509887	11.97318	3.29E-06	0.000567	5.255744	0.403305	0.61647	-0.21317	9	38360365	F	II		IGR	opensea	IGR-opense		rs1392485	1;40
cg1729397	0.22321	0.601713	11.973	3.29E-06	0.000567	5.255635	0.490108	0.713318	-0.22321	2	39948248	F	II		IGR	opensea	IGR-opense		rs1832524	21
cg0908827	0.26019	0.541301	11.97067	3.29E-06	0.000568	5.254228	0.411206	0.671396	-0.26019	17	25609077	F	II		IGR	opensea	IGR-opense		rs4794872	20
cg1293727	0.234071	0.575793	11.97044	3.29E-06	0.000568	5.254085	0.458757	0.692828	-0.23407	7	47925161	R	II	PKD1L1	Body	opensea	Body-opens		rs5546554	51;36
cg0878600	0.258147	0.584917	11.97007	3.29E-06	0.000568	5.253861	0.455844	0.713991	-0.25815	1	1.58E+08	R	II	FCRL3	TSS200	opensea	TSS200-ope		rs1893792	50;2;1
cg2508595	0.206957	0.659667	11.96793	3.3E-06	0.000568	5.252567	0.556188	0.763145	-0.20696	1	28692779	R	II		IGR	shelf	IGR-shelf	chr1:2869	rs5326994	19;1
cg0129419	0.20638	0.467759	11.96103	3.31E-06	0.000569	5.248399	0.364569	0.570949	-0.20638	1	71368022	F	II	PTGER3	3'UTR	opensea	3'UTR-open		rs1161229	45
cg2047500	0.243493	0.594041	11.95955	3.31E-06	0.000569	5.2475	0.472295	0.715788	-0.24349	7	1.23E+08	R	II	WASL	Body	opensea	Body-opens			
cg0559687	0.201998	0.507392	11.95892	3.32E-06	0.000569	5.24712	0.406393	0.608392	-0.202	15	29969720	R	II		IGR	shelf	IGR-shelf	chr15:299	rs5522949	1
cg0249336	0.255732	0.396848	11.95868	3.32E-06	0.000569	5.246978	0.268982	0.524714	-0.25573	19	36204278	R	II	ZBTB32	5'UTR	shelf	5'UTR-shelf	chr19:362	rs5393570	2
cg2078184	0.235733	0.605274	11.95854	3.32E-06	0.000569	5.246889	0.487407	0.72314	-0.23573	19	55067917	F	II		IGR	opensea	IGR-opense		rs8111581	1;4;16;42
cg1325220	0.223863	0.610771	11.95781	3.32E-06	0.000569	5.24645	0.49884	0.722703	-0.22386	8	16970768	R	II	EFHA2	Body	opensea	Body-opens		rs5663466	33;7
cg0030562	0.206093	0.578053	11.9571	3.32E-06	0.000569	5.24602	0.475007	0.6811	-0.20609	19	2237672	F	II	PLEKHJ1	TSS1500	shore	TSS1500-sh	chr19:223	rs5701175	28;39
cg0732047	0.211752	0.640834	11.95605	3.32E-06	0.000569	5.245383	0.534958	0.74671	-0.21175	7	41857293	R	II		IGR	opensea	IGR-opense		rs7928731	51;36;1
cg2207053	0.217168	0.637299	11.95439	3.32E-06	0.000569	5.244382	0.528714	0.745883	-0.21717	4	1.78E+08	F	II		IGR	opensea	IGR-opense			
cg0978165	0.207655	0.483136	11.95338	3.33E-06	0.000569	5.243768	0.379308	0.586963	-0.20765	16	66285054	F	II		IGR	opensea	IGR-opense		rs5543153	40;43
cg2039409	0.230452	0.466697	11.95219	3.33E-06	0.000569	5.243048	0.351471	0.581923	-0.23045	22	26361316	R	II	MYO18B	Body	opensea	Body-opens		rs5534232	10;2
cg0956048	0.238427	0.430235	11.9514	3.33E-06	0.00057	5.242566	0.311022	0.549449	-0.23843	8	1.07E+08	F	II	OXR1	5'UTR	opensea	5'UTR-open		rs7489222	51
cg0846287	0.20461	0.570283	11.95034	3.33E-06	0.00057	5.24193	0.467978	0.672588	-0.20461	1	66458116	F	II	PDE4B	Body	opensea	Body-opens		rs1113486	26
cg2226401	0.21723	0.528273	11.95011	3.33E-06	0.00057	5.241786	0.419658	0.636889	-0.21723	18	1774470	F	II		IGR	opensea	IGR-opense		rs5412068	23;40
cg2584009	0.219495	0.551392	11.9501	3.33E-06	0.00057	5.241783	0.441645	0.66114	-0.2195	1	1.97E+08	R	II	CFHR5	TSS1500	opensea	TSS1500-op			
cg0335825	0.251881	0.519135	11.94975	3.33E-06	0.00057	5.241571	0.393194	0.645075	-0.25188	2	1.83E+08	R	II	PPP1R1C	Body	opensea	Body-opens		rs1146932	28;2
cg2469383	0.209666	0.569641	11.94492	3.34E-06	0.000571	5.238642	0.464808	0.674474	-0.20967	7	85260179	R	II		IGR	opensea	IGR-opense		rs7370391	9
cg0050264	0.23764	0.557521	11.94464	3.34E-06	0.000571	5.238477	0.438701	0.676341	-0.23764	2	1.65E+08	F	II		IGR	opensea	IGR-opense		rs5434351	29;38
cg1006630	0.226061	0.467298	11.94138	3.35E-06	0.000571	5.236497	0.354267	0.580329	-0.22606	8	1.42E+08	R	II	PTK2	Body	opensea	Body-opens		rs5452132	35;28;22
cg0251038	0.202316	0.347556	11.93954	3.35E-06	0.000571	5.235384	0.246398	0.448714	-0.20232	2	25815015	R	II	DTNB	Body	opensea	Body-opens			
cg1041486	0.211237	0.590497	11.93761	3.36E-06	0.000571	5.234214	0.484878	0.696115	-0.21124	1	1.7E+08	R	II		IGR	opensea	IGR-opense		rs1510838	36;27
cg0936174	0.210877	0.582242	11.93717	3.36E-06	0.000571	5.233949	0.476803	0.68768	-0.21088	7	71682306	R	II	CALN1	Body	opensea	Body-opens		rs5673275	46
cg0855455	0.220647	0.489913	11.93632	3.36E-06	0.000571	5.233433	0.37959	0.600237	-0.22065	20	62330618	F	II	ARFRP1	3'UTR	shelf	3'UTR-shelf	chr20:623	rs1930438	0;22;33;34
cg2702702	0.206643	0.516947	11.93544	3.36E-06	0.000571	5.232898	0.413626	0.620268	-0.20664	18	53756914	F	II	LINC01539	Body	opensea	Body-opens		rs5481666	0;1;20
cg0783346	0.253675	0.532959	11.93537	3.36E-06	0.000571	5.232856	0.406121	0.659796	-0.25367	12	11716844	R	II	LINC01252	Body	opensea	Body-opens		rs5761767	17;6
cg1189979	0.232356	0.647463	11.93521	3.36E-06	0.000571	5.232757	0.531285	0.763641	-0.23236	18	53297089	F	II	TCF4	Body	opensea	Body-opens		rs5681957	4;8;49
cg2417844	0.226323	0.614996	11.93483	3.36E-06	0.000571	5.232532	0.501835	0.728157	-0.22632	3	1.49E+08	R	II		IGR	opensea	IGR-opense		rs1828597	15
cg0668587	0.20248	0.476327	11.92588	3.38E-06	0.000572	5.227099	0.375087	0.577567	-0.20248	21	47170388	R	II		IGR	shelf	IGR-shelf	chr21:471	rs1497875	33
cg2438565	0.231006	0.528161	11.92523	3.38E-06	0.000572	5.226702	0.412658	0.643664	-0.23101	10	50329828	R	II		IGR	opensea	IGR-opense		rs5742252	6
cg0551627	0.225844	0.598424	11.92456	3.39E-06	0.000572	5.226297	0.485502	0.711346	-0.22584	10	60237728	R	II		IGR	opensea	IGR-opense		rs5423898	11
cg1173365	0.200563	0.60266	11.92446	3.39E-06	0.000572	5.226238	0.502378	0.702941	-0.20056	3	1.23E+08	F	II	ADCY5	Body	opensea	Body-opens		rs5612065	1
cg1104932	0.22742	0.536668	11.92343	3.39E-06	0.000572	5.225612	0.422958	0.650378	-0.22742	8	77492619	F	II		IGR	opensea	IGR-opense		rs1905965	1;11;22
cg0067126	0.209493	0.465262	11.92096	3.39E-06	0.000572	5.224112	0.360515	0.570008	-0.20949	4	21792865	F	II	KCNIP4	5'UTR	opensea	5'UTR-open		rs5643437	9
cg0676046	0.211429	0.563547	11.92009	3.39E-06	0.000572	5.223585	0.457832	0.669261	-0.21143	6	29496514	F	II		IGR	opensea	IGR-opense		rs1395917	0;28
cg1821172	0.217487	0.589097	11.91993	3.4E-06	0.000572	5.223489	0.480353	0.697841	-0.21749	18	55832105	R	II	NEDD4L	Body	opensea	Body-opens		rs1824183	46;35;28;10
cg0785557	0.213458	0.496981	11.91809	3.4E-06	0.000572	5.222371	0.390253	0.60371	-0.21346	5	25393894	R	II		IGR	opensea	IGR-opense		rs5717252	28;2;1
cg2332475	0.230039	0.576217	11.91793	3.4E-06	0.000572	5.222271	0.461198	0.691237	-0.23004	18	31384904	R	II		IGR	opensea	IGR-opense			
cg0425408	0.202726	0.57104	11.91585	3.4E-06	0.000572	5.221008	0.469677	0.672403	-0.20273	13	51899383	R	II		IGR	opensea	IGR-opense		rs1470810	48
cg1902718	0.237981	0.575561	11.91218	3.41E-06	0.000572	5.218779	0.45657	0.694552	-0.23798	2	2.36E+08	R	II		IGR	opensea	IGR-opense		rs5752790	3
cg2075237	0.25226	0.452739	11.91176	3.41E-06	0.000572	5.218525	0.326609	0.578869	-0.25226	11	34645224	F	II	EHF	5'UTR	opensea	5'UTR-open		rs1486289	37
cg2045424	0.221881	0.59503	11.91166	3.41E-06	0.000572	5.218463	0.48409	0.705971	-0.22188	2	2.18E+08	R	II		IGR	opensea	IGR-opense		rs1875421	49
cg1097376	0.204006	0.45383	11.91032	3.42E-06	0.000572	5.217647	0.351827	0.555833	-0.20401	15	48625931	R	II	DUT	Body	shore	Body-shore	chr15:486	rs1501363	43;3
cg0924761	0.20385	0.444366	11.91017	3.42E-06	0.000572	5.217558	0.342441	0.54629	-0.20385	1	1.99E+08	F	II	PTPRC	Body	opensea	Body-opens		rs5658577	0;8
cg1006804	0.226331	0.430427	11.90971	3.42E-06	0.000572	5.217278	0.317262	0.543593	-0.22633	20	8721036	F	II	PLCB1	Body	opensea	Body-opens		rs2021992	0
cg1366429	0.250112	0.485632	11.90944	3.42E-06	0.000572	5.217111	0.360576	0.610688	-0.25011	11	9587813	F	II		IGR	opensea	IGR-opense		rs1502162	0
cg2610636	0.241078	0.537361	11.9086	3.42E-06	0.000572	5.216604	0.416823	0.6579	-0.24108	19	38530902	F	II	SIPA1L3	5'UTR	opensea	5'UTR-open			
cg0165597	0.219342	0.562146	11.90734	3.42E-06	0.000572	5.215838	0.452475	0.671817	-0.21934	2	1.21E+08	F	II		IGR	opensea	IGR-opense		rs5776112	20
cg1410981	0.22477	0.526128	11.90451	3.43E-06	0.000573	5.214119	0.413743	0.638513	-0.22477	3	69963596	F	II	MITF	Body	opensea	Body-opens		rs5379503	0
cg0585605	0.211898	0.615283	11.90352	3.43E-06	0.000573	5.213516	0.509334	0.721232	-0.2119	17	3989372	R	II	ZZEF1	Body	opensea	Body-opens			
cg2722153	0.213333	0.597307	11.90311	3.43E-06	0.000573	5.213263	0.490641	0.703974	-0.21333	6	51154725	R	II		IGR	opensea	IGR-opense			
cg0860608	0.231989	0.500658	11.90114	3.43E-06	0.000573	5.212069	0.384663	0.616652	-0.23199	3	1.97E+08	R	II		IGR	opensea	IGR-opense		rs5671365	13
cg2478011	0.210752	0.580839	11.90106	3.43E-06	0.000573	5.212021	0.475463	0.686215	-0.21075	3	1.46E+08	F	II		IGR	opensea	IGR-opense		rs7653582	1
cg2667824	0.269022	0.590773	11.90036	3.44E-06	0.000573	5.211589	0.456262	0.725284	-0.26902	4	88876673	R	II		IGR	opensea	IGR-opense		rs5613737	43;27
cg2592475	0.204914	0.747247	11.89985	3.44E-06	0.000573	5.211279	0.64479	0.849704	-0.20491	3	1.94E+08	F	II		IGR	opensea	IGR-opense		rs1461456	0;4;11;25;49;50
cg0606613	0.215176	0.648878	11.89981	3.44E-06	0.000573	5.211257	0.54129	0.756466	-0.21518	13	24325453	F	II	MIPEP	Body	opensea	Body-opens		rs1415071	24
cg2632425	0.20952	0.402757	11.89432	3.45E-06	0.000574	5.207915	0.297997	0.507517	-0.20952	20	40612816	R	II		IGR	opensea	IGR-opense		rs5746162	9
cg0938427	0.209377	0.523028	11.89051	3.46E-06	0.000574	5.205594	0.41834	0.627717	-0.20938	1	39738033	F	II	MACF1	Body	opensea	Body-opens			
cg0490660	0.241925	0.663758	11.88974	3.46E-06	0.000574	5.205127	0.542795	0.78472	-0.24192	15	88670452	F	II	NTRK3	ExonBnd	opensea	ExonBnd-op		rs1436171	0;1;24;25;29;47
cg1351376	0.22567	0.645654	11.88894	3.46E-06	0.000574	5.204642	0.53282	0.758489	-0.22567	3	1.52E+08	R	II		IGR	opensea	IGR-opense		rs5696451	47
cg0626699	0.234701	0.442279	11.88891	3.46E-06	0.000574	5.204625	0.324929	0.55963	-0.2347	2	1.96E+08	R	II		IGR	opensea	IGR-opense		rs1888201	18
cg0459915	0.219483	0.65951	11.88835	3.46E-06	0.000574	5.204279	0.549769	0.769252	-0.21948	1	50763351	F	II		IGR	opensea	IGR-opense		rs1914776	0
cg1423115	0.21938	0.516724	11.8869	3.46E-06	0.000574	5.203399	0.407034	0.626414	-0.21938	1	2.22E+08	F	II		IGR	opensea	IGR-opense		rs1457584	25
cg0075313	0.207076	0.641595	11.88672	3.46E-06	0.000574	5.203288	0.538057	0.745133	-0.20708	3	1.78E+08	R	II		IGR	opensea	IGR-opense		rs5422065	51;11;1
cg2181199	0.201932	0.636212	11.88134	3.48E-06	0.000575	5.200012	0.535246	0.737178	-0.20193	4	1.47E+08	R	II		IGR	opensea	IGR-opense		rs5466790	45;1
cg0945954	0.222066	0.619197	11.8808	3.48E-06	0.000575	5.199684	0.508164	0.73023	-0.22207	5	1.2E+08	F	II	PRR16	Body	opensea	Body-opens		rs1524562	30;34;36
cg0981151	0.230283	0.692447	11.88058	3.48E-06	0.000575	5.199546	0.577306	0.807589	-0.23028	3	1.6E+08	F	II	SCHIP1	Body	opensea	Body-opens			
cg1570487	0.248642	0.481429	11.87965	3.48E-06	0.000575	5.198982	0.357108	0.60575	-0.24864	2	1.03E+08	R	II	SLC9A2	Body	shelf	Body-shelf	chr2:1032	rs1833148	16;1
cg1846163	0.205117	0.523614	11.87928	3.48E-06	0.000575	5.198757	0.421056	0.626173	-0.20512	3	46990143	R	II	CCDC12	Body	opensea	Body-opens			
cg1236700	0.260616	0.420723	11.87782	3.48E-06	0.000575	5.197868	0.290416	0.551031	-0.26062	18	55673626	F	II		IGR	opensea	IGR-opense		rs1821329	21
cg1507366	0.224773	0.386876	11.87695	3.49E-06	0.000575	5.197336	0.274489	0.499262	-0.22477	11	66659874	F	II	PC	5'UTR	opensea	5'UTR-open		rs1428603	13
cg0954506	0.219048	0.451913	11.87694	3.49E-06	0.000575	5.197332	0.342389	0.561437	-0.21905	11	1.22E+08	R	II		IGR	opensea	IGR-opense		rs1425546	22;5;2
cg2018127	0.217712	0.594082	11.87259	3.49E-06	0.000575	5.194676	0.485226	0.702938	-0.21771	7	20147323	F	II	LOC10192	Body	opensea	Body-opens		rs5633761	14;18
cg0917806	0.257016	0.522962	11.87252	3.5E-06	0.000575	5.194631	0.394454	0.65147	-0.25702	3	34437393	R	II		IGR	opensea	IGR-opense		rs1511982	2;1
cg1821569	0.203063	0.551748	11.87219	3.5E-06	0.000575	5.194433	0.450216	0.653279	-0.20306	7	30890848	F	II	FAM188B	Body	opensea	Body-opens		rs1505083	0;7
cg0864427	0.238276	0.490615	11.87171	3.5E-06	0.000575	5.194137	0.371478	0.609753	-0.23828	2	2.27E+08	R	II		IGR	opensea	IGR-opense		rs5466826	30;25;1
cg1775002	0.24987	0.497716	11.87131	3.5E-06	0.000575	5.193897	0.372781	0.622651	-0.24987	1	1.76E+08	R	II	TNR	5'UTR	opensea	5'UTR-open		rs5577201	18;9
cg0386559	0.223637	0.529485	11.86944	3.5E-06	0.000575	5.192754	0.417666	0.641303	-0.22364	6	39792251	R	II	DAAM2	5'UTR	opensea	5'UTR-open		rs5691648	39;31;2;1
cg0052807	0.209101	0.403835	11.86904	3.5E-06	0.000575	5.192512	0.299285	0.508386	-0.2091	16	10060873	R	II	GRIN2A	Body	opensea	Body-opens		rs1925348	14;1
cg2466387	0.227791	0.566628	11.8688	3.5E-06	0.000575	5.192361	0.452733	0.680524	-0.22779	5	32692179	F	II		IGR	opensea	IGR-opense		rs7274062	41
cg2333439	0.205395	0.488633	11.86284	3.52E-06	0.000576	5.188724	0.385935	0.591331	-0.2054	2	1.3E+08	F	II		IGR	opensea	IGR-opense		rs5646429	1

cg0151664	0.268573	0.553262	11.85852	3.53E-06	0.000577	5.186086	0.418976	0.687549	-0.26857	1	28471495	F	II		IGR	opensea	IGR-opensea			
cg2760639	0.239642	0.557886	11.85792	3.53E-06	0.000577	5.18572	0.438066	0.677707	-0.23964	2	1.73E+08	R	II		IGR	opensea	IGR-opensea		rs1140498	51;24;2;1
cg1035216	0.208946	0.437396	11.85749	3.53E-06	0.000577	5.185461	0.332923	0.541869	-0.20895	4	1.27E+08	F	II		IGR	opensea	IGR-opensea		rs3545190	8;47
cg1105786	0.236182	0.613273	11.85725	3.53E-06	0.000577	5.185309	0.495182	0.731364	-0.23618	1	92414793	F	II	BRDT	TSS200	island	TSS200-isla	chr1:92414	rs5283840	14;30;38;43;46
cg1941954	0.220004	0.585577	11.85687	3.53E-06	0.000577	5.185079	0.475575	0.695579	-0.22	11	1.06E+08	R	II		IGR	opensea	IGR-opensea			
cg2761724	0.203581	0.638609	11.85541	3.53E-06	0.000577	5.184188	0.536818	0.740399	-0.20358	3	1.24E+08	R	II	KALRN	TSS1500	opensea	TSS1500-op		rs5331345	46;16;1
cg2238860	0.223694	0.581052	11.85527	3.53E-06	0.000577	5.184105	0.469205	0.692899	-0.22369	6	49917254	F	II	DEFB133	TSS200	opensea	TSS200-ope		rs9369926	10
cg1147433	0.221553	0.678407	11.85527	3.53E-06	0.000577	5.184105	0.56763	0.789183	-0.22155	3	1.79E+08	R	II		IGR	opensea	IGR-opensea		rs7305470	17
cg2305105	0.221662	0.58299	11.85251	3.54E-06	0.000577	5.182419	0.47216	0.693821	-0.22166	8	89072565	R	II	MMP16	Body	opensea	Body-opens		rs5700754	1
cg0148083	0.257143	0.468137	11.84877	3.55E-06	0.000578	5.180133	0.339566	0.596709	-0.25714	3	1.14E+08	F	II	ZBTB20	Body	opensea	Body-opens			
cg2080080	0.222338	0.481945	11.84661	3.55E-06	0.000578	5.178811	0.370776	0.593114	-0.22234	1	1.13E+08	F	II	CAPZA1	Body	shore	Body-shore	chr1:11316		
cg2241600	0.204108	0.579235	11.84579	3.55E-06	0.000578	5.17831	0.477181	0.681289	-0.20411	22	39713062	R	II	RPL3	Body	shelf	Body-shelf	chr22:3971	rs5376181	45;2
cg1348438	0.203982	0.580239	11.84513	3.55E-06	0.000578	5.177903	0.478248	0.68223	-0.20398	1	88865486	R	II		IGR	opensea	IGR-opensea			
cg2247831	0.206628	0.426654	11.84221	3.56E-06	0.000578	5.176118	0.32334	0.529968	-0.20663	2	1.1E+08	R	II		IGR	opensea	IGR-opensea		rs5479719	2
cg0425883	0.258877	0.540491	11.84104	3.56E-06	0.000579	5.175406	0.411052	0.669929	-0.25888	2	1.03E+08	F	II	IL1R1	5'UTR	opensea	5'UTR-open		rs7462859	33;42;50
cg1090454	0.232811	0.513085	11.84058	3.56E-06	0.000579	5.175124	0.396679	0.62949	-0.23281	15	58262083	F	II	ALDH1A2	Body	opensea	Body-opens		rs5339963	0;1;18
cg0849631	0.214282	0.510782	11.83597	3.57E-06	0.000579	5.172302	0.403641	0.617924	-0.21428	4	1.29E+08	F	II		IGR	opensea	IGR-opensea		rs5566420	0;3
cg0302648	0.21734	0.634378	11.83534	3.58E-06	0.000579	5.171915	0.525707	0.743048	-0.21734	17	40404781	R	II	STAT5B	5'UTR	opensea	5'UTR-open			
cg1363859	0.202935	0.464907	11.83148	3.58E-06	0.00058	5.169552	0.36344	0.566375	-0.20294	13	67240484	R	II	PCDH9	Body	opensea	Body-opens		rs5529964	33;2;1
cg0460133	0.261272	0.519094	11.83054	3.59E-06	0.00058	5.168977	0.388457	0.64973	-0.26127	12	26209446	R	II	RASSF8	Body	opensea	Body-opens		rs5428438	2;1
cg1796966	0.244268	0.583936	11.82943	3.59E-06	0.00058	5.168295	0.461801	0.70607	-0.24427	1	2.21E+08	R	II		IGR	opensea	IGR-opensea		rs5754031	26;7;6
cg0463459	0.241735	0.435613	11.82933	3.59E-06	0.00058	5.168237	0.314746	0.556481	-0.24173	20	21417020	R	II		IGR	opensea	IGR-opensea		rs1841806	2;1
cg1453036	0.215079	0.652307	11.82875	3.59E-06	0.00058	5.167882	0.544767	0.759847	-0.21508	8	1.07E+08	R	II	ZFPM2	Body	opensea	Body-opens		rs1388776	49;37;29;19;1
cg0465414	0.237917	0.607913	11.8287	3.59E-06	0.00058	5.16785	0.488954	0.726871	-0.23792	9	1.34E+08	R	II	ABL1	Body	opensea	Body-opens		rs5404333	30;1
cg2566088	0.230606	0.592725	11.82803	3.59E-06	0.00058	5.167436	0.477422	0.708028	-0.23061	18	57324466	R	II	CCBE1	Body	opensea	Body-opens		rs3722289	41;40;36;13;2
cg1699668	0.225362	0.522079	11.82706	3.59E-06	0.00058	5.166844	0.409398	0.63476	-0.22536	1	71830814	R	II		IGR	opensea	IGR-opensea		rs1425663	48;40;32;23;10
cg1222439	0.219384	0.49238	11.82702	3.59E-06	0.00058	5.166824	0.382688	0.602072	-0.21938	14	79084021	R	II	NRXN3	5'UTR	opensea	5'UTR-open		rs5629127	51;46;43;42
cg0608004	0.203547	0.691497	11.82629	3.6E-06	0.00058	5.166374	0.589724	0.793271	-0.20355	1	45038374	F	II	RNF220	Body	opensea	Body-opens		rs5353194	1
cg0238449	0.244422	0.558287	11.82501	3.6E-06	0.00058	5.165588	0.436076	0.680498	-0.24442	1	1.62E+08	R	II	NOS1AP	Body	opensea	Body-opens			
cg2248247	0.223039	0.528615	11.82389	3.6E-06	0.000581	5.164902	0.417095	0.640135	-0.22304	12	13364350	R	II	EMP1	5'UTR	opensea	5'UTR-open		rs1421687	43;19
cg1797482	0.200223	0.619175	11.8233	3.6E-06	0.000581	5.164543	0.519063	0.719287	-0.20022	21	20502091	F	II		IGR	opensea	IGR-opensea		rs5354549	0;4;6;23
cg1929169	0.225081	0.566876	11.82275	3.6E-06	0.000581	5.164203	0.454335	0.679416	-0.22508	13	27769437	R	II		IGR	opensea	IGR-opensea		rs5363224	22;14;9;1
cg2633640	0.208037	0.469505	11.82241	3.6E-06	0.000581	5.163993	0.365487	0.573524	-0.20804	20	55508704	R	II		IGR	opensea	IGR-opensea			
cg1585415	0.20728	0.578421	11.82094	3.61E-06	0.000581	5.163097	0.474781	0.682061	-0.20728	10	73592958	R	II	PSAP	Body	opensea	Body-opens		rs1931756	26;14
cg0202077	0.224378	0.523471	11.81953	3.61E-06	0.000581	5.162234	0.411282	0.63566	-0.22438	5	1.39E+08	R	II	UBE2D2	5'UTR	opensea	5'UTR-open		rs5492604	1
cg2587658	0.220818	0.534501	11.81882	3.61E-06	0.000581	5.161793	0.424092	0.64491	-0.22082	8	75260202	R	II		IGR	shelf	IGR-shelf	chr8:75262		
cg1551234	0.24721	0.61264	11.81612	3.62E-06	0.000582	5.160141	0.489035	0.736245	-0.24721	8	1.31E+08	R	II	CCDC26	Body	opensea	Body-opens		rs1895508	21
cg2243478	0.226562	0.550174	11.81421	3.62E-06	0.000582	5.158966	0.436893	0.663455	-0.22656	14	59537154	F	II		IGR	opensea	IGR-opensea		rs8008708	1
cg0586867	0.216719	0.508708	11.81405	3.62E-06	0.000582	5.158872	0.400348	0.617067	-0.21672	10	1.15E+08	F	II		IGR	opensea	IGR-opensea		rs1400140	0;26;41;47;48
cg0737516	0.20078	0.602111	11.81323	3.62E-06	0.000582	5.158369	0.501721	0.702501	-0.20078	8	1.02E+08	R	II	ANKRD46	5'UTR	opensea	5'UTR-open		rs5584302	7;3
cg2692676	0.211512	0.39474	11.81079	3.63E-06	0.000583	5.156869	0.288983	0.500496	-0.21151	6	54127349	F	II	C6orf142	Body	opensea	Body-opens		rs1871060	0;23
cg1573538	0.233729	0.657947	11.80923	3.63E-06	0.000583	5.155916	0.541083	0.774811	-0.23373	8	1.25E+08	R	II	FER1L6	Body	opensea	Body-opens		rs5575712	44;27;2
cg2713019	0.246692	0.571676	11.80789	3.64E-06	0.000583	5.155089	0.44833	0.695022	-0.24669	8	29028955	F	II	KIF13B	Body	opensea	Body-opens		rs1827352	22;36
cg0654593	0.269577	0.582621	11.80757	3.64E-06	0.000583	5.154896	0.447832	0.717409	-0.26958	19	39107006	R	II	MAP4K1	Body	shelf	Body-shelf	chr19:3910	rs1784769	29
cg0504672	0.218843	0.509437	11.80739	3.64E-06	0.000583	5.154785	0.400016	0.618859	-0.21884	1	15208255	R	II	KIAA1026	Body	opensea	Body-opens		rs5570861	46;45;32
cg0064031	0.242261	0.528804	11.805	3.64E-06	0.000584	5.153318	0.407674	0.649935	-0.24226	8	67835853	R	II	SNORD87	TSS1500	shore	TSS1500-sh	chr8:67837		
cg0044251	0.206679	0.514514	11.80365	3.65E-06	0.000584	5.152492	0.411175	0.617854	-0.20668	11	1.2E+08	R	II	POU2F3	Body	opensea	Body-opens		rs1477423	36;2;1
cg2752709	0.208788	0.33814	11.80349	3.65E-06	0.000584	5.152388	0.233746	0.442534	-0.20879	1	2.07E+08	R	II		IGR	opensea	IGR-opensea		rs1885881	46;23;12
cg2136222	0.232668	0.626885	11.80037	3.65E-06	0.000584	5.150478	0.510551	0.743219	-0.23267	8	1864982	R	II	ARHGEF10	Body	opensea	Body-opens		rs1857418	50;47;2
cg1313628	0.209727	0.419911	11.7995	3.66E-06	0.000584	5.149941	0.315048	0.524775	-0.20973	6	3868172	R	II		IGR	opensea	IGR-opensea			
cg0702643	0.222715	0.60204	11.79796	3.66E-06	0.000584	5.148995	0.490682	0.713398	-0.22272	1	1.95E+08	F	II		IGR	opensea	IGR-opensea		rs7619827	43
cg0564172	0.212296	0.528731	11.79687	3.66E-06	0.000585	5.148325	0.422583	0.634879	-0.2123	11	57212298	R	II		IGR	opensea	IGR-opensea		rs1474802	49
cg2060051	0.219862	0.573971	11.7964	3.66E-06	0.000585	5.148037	0.46404	0.683902	-0.21986	4	1.46E+08	R	II	SMAD1	Body	opensea	Body-opens			
cg1114292	0.227798	0.531795	11.79318	3.67E-06	0.000585	5.146058	0.417896	0.645694	-0.2278	2	2.38E+08	F	II		IGR	opensea	IGR-opensea		rs1506133	1;36
cg1836773	0.225489	0.486395	11.78809	3.68E-06	0.000586	5.142932	0.37365	0.599139	-0.22549	2	1.83E+08	R	II		IGR	opensea	IGR-opensea		rs5418478	41;2
cg2143898	0.249887	0.369222	11.78759	3.68E-06	0.000586	5.142621	0.244278	0.494165	-0.24989	19	55787902	F	II	HSPBP1	Body	shelf	Body-shelf	chr19:5579		
cg1607949	0.235669	0.543902	11.78701	3.68E-06	0.000586	5.142263	0.426067	0.661736	-0.23567	5	94244779	R	II	MCTP1	Body	opensea	Body-opens		rs5705747	15
cg2568778	0.214707	0.500745	11.7853	3.69E-06	0.000586	5.141214	0.393391	0.608099	-0.21471	4	76933117	F	II	ART3	5'UTR	opensea	5'UTR-open			
cg2695547	0.21509	0.550094	11.78325	3.69E-06	0.000586	5.139951	0.442549	0.657639	-0.21509	1	1.65E+08	R	II	PBX1	Body	opensea	Body-opens		rs5640356	30;29;2
cg0880370	0.266795	0.55803	11.78322	3.69E-06	0.000586	5.139934	0.424632	0.691428	-0.2668	16	57273293	F	II	RSPRY1	3'UTR	opensea	3'UTR-open		rs1417706	2;41
cg1134696	0.204392	0.625806	11.78274	3.69E-06	0.000586	5.139641	0.52361	0.728002	-0.20439	11	5266120	R	II	HBBP1	TSS1500	opensea	TSS1500-op			
cg0454711	0.214692	0.526277	11.78202	3.69E-06	0.000586	5.139198	0.418931	0.633623	-0.21469	18	72112867	F	II	FAM69C	Body	opensea	Body-opens		rs5397187	1;4;12;23;30;34
cg2440156	0.226883	0.539551	11.7811	3.7E-06	0.000587	5.13863	0.42611	0.652992	-0.22688	10	97152743	F	II	SORBS1	Body	opensea	Body-opens			
cg0032703	0.222614	0.47395	11.77989	3.7E-06	0.000587	5.137889	0.362643	0.585256	-0.22261	12	59575337	F	II		IGR	opensea	IGR-opensea		rs1497378	0;36
cg1986577	0.218519	0.491634	11.7794	3.7E-06	0.000587	5.137582	0.382374	0.600893	-0.21852	1	2.22E+08	F	II	LOC40080	TSS1500	opensea	TSS1500-op		rs7852111	0;1;3;8;44
cg1465315	0.213842	0.548486	11.77937	3.7E-06	0.000587	5.137569	0.441565	0.655407	-0.21384	8	80263514	R	II		IGR	opensea	IGR-opensea		rs1426279	47;41
cg0714748	0.203116	0.502622	11.77607	3.71E-06	0.000587	5.135533	0.401064	0.60418	-0.20312	10	55236933	R	II		IGR	opensea	IGR-opensea		rs1820405	37;13
cg1692589	0.220552	0.599553	11.77461	3.71E-06	0.000587	5.134636	0.489277	0.709829	-0.22055	13	1.03E+08	R	II		IGR	opensea	IGR-opensea		rs5711842	1
cg1821008	0.218487	0.577854	11.77388	3.71E-06	0.000587	5.134185	0.468611	0.687097	-0.21849	1	1.76E+08	F	II		IGR	opensea	IGR-opensea			
cg1720682	0.206569	0.483786	11.77254	3.72E-06	0.000587	5.133362	0.380501	0.587071	-0.20657	2	1.04E+08	F	II		IGR	opensea	IGR-opensea		rs1868500	1;22;35;42;51
cg1768394	0.242605	0.497478	11.77185	3.72E-06	0.000587	5.132937	0.376176	0.618781	-0.24261	15	65763347	F	II	DPP8	Body	opensea	Body-opens		rs5557354	18;20;48
cg0190075	0.226976	0.55379	11.77145	3.72E-06	0.000587	5.132688	0.440303	0.667278	-0.22698	6	64350180	R	II	PHF3	5'UTR	shelf	5'UTR-shelf	chr6:6434!	rs5360852	38;22;9
cg0138379	0.210241	0.705824	11.77086	3.72E-06	0.000588	5.132327	0.600704	0.810945	-0.21024	12	1.25E+08	R	II		IGR	opensea	IGR-opensea		rs1105787	45;38;25;2
cg0794895	0.234043	0.546482	11.7702	3.72E-06	0.000588	5.131921	0.42946	0.663503	-0.23404	6	45649741	R	II		IGR	opensea	IGR-opensea		rs1432177	2
cg1711828	0.220057	0.670669	11.7691	3.72E-06	0.000588	5.131243	0.560641	0.780698	-0.22006	1	2.19E+08	R	II	TGFB2	Body	opensea	Body-opens		rs1923330	30;1
cg0311214	0.210858	0.501959	11.76898	3.72E-06	0.000588	5.131173	0.39653	0.607388	-0.21086	8	77455657	R	II		IGR	opensea	IGR-opensea		rs5323073	24;2;1
cg0681348	0.246096	0.613567	11.76713	3.73E-06	0.000588	5.130033	0.490519	0.736615	-0.2461	20	20499757	R	II	RALGAPA2	Body	opensea	Body-opens		rs5620859	46;7;1
cg2295218	0.24901	0.545826	11.76596	3.73E-06	0.000588	5.12931	0.421321	0.670331	-0.24901	11	9091264	F	II	SCUBE2	Body	opensea	Body-opens		rs5540114	42
cg0146845	0.218308	0.531044	11.76369	3.74E-06	0.000588	5.127912	0.42189	0.640198	-0.21831	17	43024214	R	II	KIF18B	5'UTR	shore	5'UTR-shor	chr17:4302	rs1392967	49;23;1
cg0785106	0.212911	0.575563	11.76039	3.74E-06	0.000589	5.125878	0.469108	0.682019	-0.21291	18	66774794	R	II		IGR	opensea	IGR-opensea		rs5662208	34
cg1088951	0.20765	0.555639	11.75887	3.75E-06	0.000589	5.124942	0.451814	0.659464	-0.20765	7	17532256	R	II	LOC10192	Body	opensea	Body-opens		rs5736554	24;20;1
cg2103629	0.205592	0.48101	11.75805	3.75E-06	0.000589	5.124432	0.378214	0.583806	-0.20559	11	86105321	R	II	CCDC81	Body	opensea	Body-opens		rs5351736	34
cg0521474	0.206442	0.532317	11.75379	3.76E-06	0.00059	5.121807	0.429096	0.635538	-0.20644	10	86367946	F	II		IGR	opensea	IGR-opensea		rs7531330	1;26;36
cg2590242	0.25272	0.570633	11.75059	3.77E-06	0.00059	5.119833	0.444273	0.696993	-0.25272	12	94773488	R	II	CEP83	Body	opensea	Body-opens			
cg0361514	0.223366	0.567455	11.75016	3.77E-06	0.00059	5.11957	0.455773	0.679138	-0.22337	3	1.07E+08	R	II	LOC34459	Body	opensea	Body-opens		rs1464169	49
cg0049654	0.203779	0.527707	11.74756	3.77E-06	0.000591	5.117963	0.425817	0.629596	-0.20378	9	32819000	F	II		IGR	opensea	IGR-opensea		rs5286463	18;32
cg1643831	0.263328	0.496048	11.74627	3.78E-06	0.000591	5.117167	0.364384	0.627712	-0.26333	7	1E+08	R	II	AGFG2	Body	shelf	Body-shelf	chr7:1001	rs5641192	1;15;35
cg1481659	0.259282	0.500003	11.74624	3.78E-06	0.000591	5.117151	0.370362	0.629644	-0.25928	3	1.42E+08	F	II	ATP1B3	Body	opensea	Body-opens		rs7819298	9;36;37;43
cg0847439	0.232683	0.710518	11.74491	3.78E-06	0.000591	5.11633	0.594176	0.826859	-0.23268	16	27237656	F	II	NSMCE1	Body	opensea	Body-opens			
cg1091934	0.22223	0.571621	11.74252	3.79E-06	0.000591	5.114852	0.460506	0.682736	-0.22223	11	59210634	F	II	OR5A1	TSS200	opensea	TSS200-ope		rs3750093	0;1;2;12
cg1512478	0.222234	0.561032	11.74186	3.79E-06	0.000591	5.114447	0.449915	0.672149	-0.22223	3	5288430	R	II		IGR	opensea	IGR-opensea		rs5608083	51;49;1
cg0504072	0.235847	0.554138	11.74109	3.79E-06	0.000591	5.113968	0.436214	0.672061	-0.23585	4	1.84E+08	F	II	CLDN22	TSS1500	opensea	TSS1500-op		rs1475780	30
cg2607781	0.204482	0.36298	11.74076	3.79E-06	0.000591	5.113766	0.260739	0.465221	-0.20448	11	1.19E+08	R	II	USP2	Body	shelf	Body-shelf	chr11:119	rs5780654	40
cg0697204	0.216707	0.53845	11.74006	3.79E-06	0.000591	5.113334	0.430096	0.646803	-0.21671	6	21348636	R	II		IGR	opensea	IGR-opensea		rs1125834	32;22;4
cg0188287	0.225046	0.63123	11.73963	3.79E-06	0.000591	5.113067	0.518707	0.743753	-0.22505	17	32575548	R	II		IGR	opensea	IGR-opensea		rs5632281	7
cg1489409	0.22149	0.545427	11.73955	3.79E-06	0.000591	5.113021	0.434682	0.656172	-0.22149	2	2.27E+08	F	II		IGR	opensea	IGR-opensea		rs5637669	26;47
cg0847861	0.248345	0.549314	11.73621	3.8E-06	0.000591	5.110954	0.425142	0.673487	-0.24834	10	48372128	R	II	ZNF488	3'UTR	shore	3'UTR-shor	chr10:483	rs5355257	2
cg2452598	0.208227	0.565756	11.73595	3.8E-06	0.000591	5.110797	0.461643	0.66987	-0.20823	4	19134792	R	II		IGR	opensea	IGR-opensea			
cg1118387	0.222207	0.505512	11.73585	3.8E-06	0.000591	5.110735	0.394408	0.616616	-0.22221	2	38341436	F	II		IGR	opensea	IGR-opensea		rs1901289	15
cg2689157	0.224505	0.478563	11.7332	3.81E-06	0.000591	5.109093	0.36631	0.590815	-0.22451	1	56288471	F	II		IGR	opensea	IGR-opensea		rs1856375	1
cg0943944	0.236172	0.526128	11.73239	3.81E-06	0.000591	5.108592	0.408042	0.644214	-0.23617	3	1.75E+08	R	II	NAALADL2	Body	opensea	Body-opens		rs5398480	51
cg1700769	0.211829	0.542616	11.73115	3.81E-06	0.000591	5.107828	0.436701	0.64853	-0.21183	18	12041356	R	II		IGR	shore	IGR-shore	chr18:120	rs5657928	51;2
cg0089229	0.276192	0.465661	11.72995	3.82E-06	0.000591	5.107084	0.327565	0.603757	-0.27619	13	49323918	F	II		IGR	opensea	IGR-opensea		rs5729498	32
cg0875526	0.207285	0.524951	11.72328	3.83E-06	0.000592	5.10296	0.421308	0.628593	-0.20728	6	3325053	R	II	SLC22A23	Body	opensea	Body-opens		rs5492652	51;47;12
cg0422807	0.207506	0.517515	11.72055	3.84E-06	0.000592	5.10127	0.413762	0.621268	-0.20751	12	1.14E+08	R	II		IGR	shelf	IGR-shelf	chr12:113		
cg2493362	0.210271	0.612086	11.7181	3.84E-06	0.000592	5.099757	0.506951	0.717221	-0.21027	9	38024422	F	II	SHB	Body	opensea	Body-opens		rs5705596	13;36;51
cg1648578	0.237496	0.556517	11.71634	3.85E-06	0.000593	5.09867	0.437769	0.675265	-0.2375	7	82013475	F	II	CACNA2D1	Body	opensea	Body-opens		rs1842346	27;46
cg1998549	0.225681	0.568876	11.71526	3.85E-06	0.000593	5.098001	0.456035	0.681716	-0.22568	16	83073842	F	II	CDH13	5'UTR	opensea	5'UTR-open		rs5349397	0;6;7;36;49
cg0174281	0.203599	0.612685	11.71519	3.85E-06	0.000593	5.097957	0.510885	0.714484	-0.2036	1	70508118	F	II	LRRC7	Body	opensea	Body-opens		rs1461028	0;22;23;30
cg2082318	0.219245	0.497232	11.71461	3.85E-06	0.000593	5.097599	0.38761	0.606855	-0.21924	1	39318494	F	II	RRAGC	Body	opensea	Body-opens		rs1181072	1;29
cg1269676	0.255057	0.415117	11.71042	3.86E-06	0.000593	5.095001	0.287588	0.542645	-0.25506	18	60848167	F	II	BCL2	Body	opensea	Body-opens		rs5664592	4;17;28
cg0688042	0.218043	0.571097	11.70848	3.87E-06	0.000594	5.093802	0.462076	0.680118	-0.21804	1	2E+08	R	II	NR5A2	Body	shelf	Body-shelf	chr1:2001	rs5405584	50;34;26
cg0902787	0.222076	0.614462	11.70693	3.87E-06	0.000594	5.092838	0.503424	0.725501	-0.22208	1	25914071	F	II		IGR	opensea	IGR-opensea		rs1821826	1;30;48
cg1638717	0.202754	0.534699	11.7052	3.87E-06	0.000594	5.09177	0.433322	0.636076	-0.20275	18	44048991	F	II		IGR	opensea	IGR-opensea		rs1260714	17
cg2215925	0.239412	0.532776	11.70485	3.88E-06	0.000594	5.091552	0.413071	0.652482	-0.23941	1	65064166	R	II	CACHD1	Body	opensea	Body-opens		rs5303520	42
cg2305109	0.230063	0.658122	11.69943	3.89E-06	0.000595	5.088193	0.54309	0.773153	-0.23006	8	1.22E+08	F	II		IGR	opensea	IGR-opensea		rs1655004	38;48
cg1152502	0.223678	0.491265	11.69888	3.89E-06	0.000595	5.08785	0.379426	0.603104	-0.22368	20	17674233	F	II	BANF2	TSS200	opensea	TSS200-ope		rs5750991	0;28;41
cg1580658	0.233592	0.487871	11.69831	3.89E-06	0.000595	5.0875	0.371075	0.604667	-0.23359	13	29177556	F	II		IGR	opensea	IGR-opensea		rs1421115	1
cg2524080	0.225775	0.436914	11.69721	3.89E-06	0.000595	5.086816	0.324027	0.549802	-0.22577	5	89195843	R	II		IGR	opensea	IGR-opensea		rs1380979	31;16

cg2166088	0.214468	0.501906	11.69481	3.9E-06	0.000595	5.085329	0.394672	0.60914	-0.21447	3	78210011	R	II		IGR	opensea	IGR-opense		rs1812710 40;34;29;15;8
cg2437161	0.228164	0.474642	11.69351	3.9E-06	0.000596	5.084518	0.36056	0.588724	-0.22816	8	74276808	F	II		IGR	opensea	IGR-opense		rs5598781 0;1;21;35;46
cg1528023	0.23179	0.632337	11.69226	3.91E-06	0.000596	5.083747	0.516442	0.748233	-0.23179	1	61737456	R	II	NFIA	Body	opensea	Body-opens		rs7574300 2
cg2193219	0.235279	0.579147	11.69143	3.91E-06	0.000596	5.08323	0.461508	0.696786	-0.23528	1	2.29E+08	F	II		IGR	opensea	IGR-opense		
cg1345131	0.215807	0.668686	11.69141	3.91E-06	0.000596	5.08322	0.560783	0.77659	-0.21581	2	1.27E+08	F	II		IGR	opensea	IGR-opense		rs5654319 30
cg1018969	0.202563	0.431592	11.68981	3.91E-06	0.000596	5.082224	0.330311	0.532874	-0.20256	6	44458366	F	II		IGR	opensea	IGR-opense		rs7959621 8;22;35;44;49
cg0703796	0.200055	0.531982	11.68959	3.91E-06	0.000596	5.082089	0.431954	0.63201	-0.20006	6	43265846	R	II	SLC22A7	TSS200	opensea	TSS200-ope		rs5320850 49;46
cg2679563	0.209415	0.546037	11.68867	3.91E-06	0.000596	5.08152	0.44133	0.650745	-0.20942	4	30869870	R	II	PCDH7	Body	opensea	Body-opens		rs5295335 50;45;43;30
cg0816730	0.205557	0.512119	11.68797	3.92E-06	0.000596	5.081082	0.409341	0.614898	-0.20556	2	2.23E+08	F	II	SGPP2	Body	opensea	Body-opens		
cg1478879	0.201713	0.592226	11.68572	3.92E-06	0.000596	5.079688	0.491369	0.693082	-0.20171	11	1.24E+08	F	II		IGR	opensea	IGR-opense		rs7892408 0;28
cg1787765	0.242272	0.588857	11.6853	3.92E-06	0.000596	5.079425	0.467721	0.709993	-0.24227	10	98751367	R	II		IGR	opensea	IGR-opense		rs1123526 27;1
cg0755123	0.222682	0.529726	11.68522	3.92E-06	0.000596	5.079378	0.418385	0.641068	-0.22268	17	17253796	F	II		IGR	opensea	IGR-opense		rs7706664 1;42
cg1579607	0.235755	0.61715	11.68315	3.93E-06	0.000597	5.078092	0.499273	0.735027	-0.23575	2	1.06E+08	F	II	TGFBRAP1	5'UTR	opensea	5'UTR-oper		rs5297386 0;18;49
cg1500140	0.208166	0.530815	11.68008	3.94E-06	0.000597	5.076186	0.426732	0.634898	-0.20817	12	1.31E+08	F	II	RIMBP2	Body	opensea	Body-opens		rs1409323 40
cg1348801	0.203088	0.390654	11.67983	3.94E-06	0.000597	5.076029	0.28911	0.492198	-0.20309	15	81466841	R	II		IGR	opensea	IGR-opense		rs5767794 33;7;1
cg2321105	0.269271	0.450264	11.6788	3.94E-06	0.000597	5.075388	0.315628	0.584899	-0.26927	3	1.68E+08	F	II	EGFEM1P	Body	opensea	Body-opens		rs5340285 0;26;34;41
cg0966605	0.211572	0.560037	11.67766	3.94E-06	0.000597	5.074682	0.45425	0.665823	-0.21157	4	41611109	R	II	LIMCH1	Body	opensea	Body-opens		rs5660973 10
cg1698913	0.21059	0.523813	11.67562	3.95E-06	0.000597	5.073416	0.418518	0.629107	-0.21059	5	1.6E+08	R	II		IGR	opensea	IGR-opense		
cg0653900	0.235298	0.467505	11.67402	3.95E-06	0.000598	5.072418	0.349856	0.585154	-0.2353	2	2.08E+08	F	II	CPO	Body	opensea	Body-opens		rs1150623 0;29
cg0129692	0.22324	0.595926	11.67239	3.95E-06	0.000598	5.071406	0.484306	0.707546	-0.22324	2	82120996	F	II		IGR	opensea	IGR-opense		rs1500159 13
cg2543279	0.204999	0.545563	11.6699	3.96E-06	0.000598	5.069857	0.443064	0.648063	-0.205	2	23546167	F	II		IGR	opensea	IGR-opense		rs1420694 38
cg2506513	0.22145	0.658446	11.66942	3.96E-06	0.000598	5.069561	0.54772	0.769171	-0.22145	10	93100919	R	II	LOC100188	Body	opensea	Body-opens		rs5422173 41;1
cg0428196	0.201699	0.636361	11.66925	3.96E-06	0.000598	5.069456	0.535511	0.73721	-0.2017	15	53917915	R	II	WDR72	Body	opensea	Body-opens		rs1420246 40;1
cg0942073	0.238006	0.603704	11.66851	3.96E-06	0.000598	5.068996	0.484701	0.722707	-0.23801	7	25705835	R	II		IGR	opensea	IGR-opense		rs1899908 36;7;1
cg1048541	0.209271	0.468841	11.66304	3.98E-06	0.000599	5.065594	0.364206	0.573477	-0.20927	2	26586435	F	II	EPT1	Body	opensea	Body-opens		rs1854841 1
cg0643990	0.236082	0.581842	11.66283	3.98E-06	0.000599	5.06546	0.463801	0.699883	-0.23608	2	1.68E+08	F	II	XIRP2	TSS200	opensea	TSS200-ope		rs7741461 27;34
cg0423802	0.208889	0.489369	11.66188	3.98E-06	0.000599	5.064868	0.384924	0.593814	-0.20889	4	20842253	F	II	KCNIP4	Body	opensea	Body-opens		rs7324251 51
cg1813907	0.22768	0.536733	11.65974	3.99E-06	0.0006	5.063537	0.422893	0.650572	-0.22768	9	1.08E+08	R	II	ABCA1	5'UTR	opensea	5'UTR-oper		rs5624434 26;11;2
cg2288944	0.202172	0.688548	11.65871	3.99E-06	0.0006	5.062895	0.587462	0.789634	-0.20217	19	6886962	F	II	EMR1	TSS1500	opensea	TSS1500-op		rs5441143 26
cg2535176	0.217776	0.571172	11.65696	3.99E-06	0.0006	5.061809	0.462284	0.68006	-0.21778	5	71724363	F	II		IGR	opensea	IGR-opense		rs1821856 1;31;32;44;51
cg0019540	0.236378	0.506878	11.65352	4E-06	0.0006	5.059667	0.388689	0.625067	-0.23638	10	95140036	F	II	MYOF	Body	opensea	Body-opens		rs5625012 1
cg2661399	0.235744	0.525142	11.64946	4.01E-06	0.000601	5.057135	0.40727	0.643013	-0.23574	4	5412385	F	II	STK32B	Body	opensea	Body-opens		rs1143265 0
cg2190362	0.203349	0.515922	11.64931	4.01E-06	0.000601	5.057043	0.414248	0.617597	-0.20335	1	1.52E+08	R	II	CRCT1	TSS1500	shelf	TSS1500-sh	chr1:15248	rs1066646 0
cg1548545	0.21982	0.622439	11.64891	4.01E-06	0.000601	5.056797	0.512529	0.732349	-0.21982	21	40266015	R	II	LOC400867	Body	opensea	Body-opens		rs1869794 27;2;1
cg0789462	0.230866	0.598947	11.64699	4.02E-06	0.000601	5.055599	0.483514	0.71438	-0.23087	5	58265816	R	II	PDE4D	3'UTR	opensea	3'UTR-oper		rs5659539 50;2
cg0225064	0.273585	0.567672	11.64669	4.02E-06	0.000601	5.055409	0.43088	0.704464	-0.27358	15	45020024	R	II	LOC100419	TSS1500	opensea	TSS1500-op		rs1381315 40;1
cg0871003	0.204481	0.551845	11.64587	4.02E-06	0.000601	5.0549	0.449604	0.654085	-0.20448	1	64475514	F	II	ROR1	Body	opensea	Body-opens		rs1445814 1;20
cg1123654	0.208527	0.502322	11.64536	4.02E-06	0.000601	5.054583	0.398058	0.606585	-0.20853	21	27777262	R	II		IGR	opensea	IGR-opense		rs1140161 11
cg0064838	0.221071	0.570543	11.64485	4.02E-06	0.000601	5.054263	0.460008	0.681079	-0.22107	1	1.67E+08	F	II	DUSP27	Body	shelf	Body-shelf	chr1:16705	rs4657633 27;29
cg1042384	0.225745	0.583407	11.64459	4.02E-06	0.000601	5.054102	0.470534	0.696279	-0.22574	6	56200769	F	II		IGR	opensea	IGR-opense		rs1903798 46;51
cg0603378	0.242286	0.577643	11.64214	4.03E-06	0.000601	5.052576	0.4565	0.698786	-0.24229	8	1.06E+08	F	II		IGR	opensea	IGR-opense		rs1138761 0;17;34
cg2366847	0.205337	0.601309	11.642	4.03E-06	0.000601	5.05249	0.49864	0.703978	-0.20534	7	65715904	F	II	TPST1	Body	opensea	Body-opens		rs5412710 14;30
cg0255025	0.222403	0.647525	11.6414	4.03E-06	0.000601	5.052114	0.536324	0.758727	-0.2224	2	2.22E+08	R	II		IGR	opensea	IGR-opense		rs5615221 51;48;47;2
cg0440442	0.208919	0.405302	11.64101	4.03E-06	0.000601	5.05187	0.300843	0.509761	-0.20892	8	1.24E+08	R	II		IGR	opensea	IGR-opense		rs5681075 14;8;2;1
cg0515968	0.201197	0.46619	11.64059	4.03E-06	0.000601	5.051612	0.365591	0.566788	-0.2012	2	9514871	F	II	ASAP2	Body	island	Body-island	chr2:95147	rs2021450 0;15;46;49
cg1600159	0.253835	0.577248	11.64053	4.03E-06	0.000601	5.051571	0.450331	0.704166	-0.25384	10	3800109	R	II		IGR	opensea	IGR-opense		rs1895452 37;35;2;1
cg1842271	0.228256	0.419737	11.63924	4.04E-06	0.000601	5.050768	0.305609	0.533865	-0.22826	7	19392033	R	II		IGR	opensea	IGR-opense		rs1023334 27;8;1
cg0349925	0.217954	0.536177	11.6338	4.05E-06	0.000602	5.047379	0.4272	0.645154	-0.21795	8	72912611	R	II	MSC-AS1	Body	opensea	Body-opens		rs5699988 8;2
cg1145495	0.209624	0.41876	11.63371	4.05E-06	0.000602	5.047319	0.313948	0.523572	-0.20962	2	1.93E+08	R	II	TMEFF2	Body	opensea	Body-opens		rs5699901 51
cg2106517	0.225553	0.615893	11.63275	4.05E-06	0.000602	5.046724	0.503117	0.728669	-0.22555	7	84730904	F	II	SEMA3D	Body	opensea	Body-opens		
cg0489321	0.235322	0.618199	11.62801	4.06E-06	0.000603	5.043763	0.500538	0.73586	-0.23532	9	14310315	R	II	NFIB	Body	shelf	Body-shelf	chr9:14312	
cg2491693	0.244587	0.484794	11.62662	4.07E-06	0.000603	5.042897	0.362501	0.607088	-0.24459	1	1.01E+08	R	II	DBT	TSS1500	opensea	TSS1500-op		
cg0373284	0.218894	0.573486	11.62542	4.07E-06	0.000603	5.042149	0.464039	0.682933	-0.21889	18	11096230	F	II	PIEZO2	Body	opensea	Body-opens		rs1505555 0;20
cg1756676	0.215171	0.499791	11.62396	4.08E-06	0.000603	5.041232	0.392206	0.607376	-0.21517	21	31607097	F	II		IGR	opensea	IGR-opense		rs5455152 0;1;3;6;19;47
cg2627266	0.210181	0.551605	11.62338	4.08E-06	0.000604	5.040872	0.446514	0.656696	-0.21018	11	85977891	R	II	EED	Body	opensea	Body-opens		rs1511443 20;11;2
cg1805371	0.224085	0.574326	11.62058	4.08E-06	0.000604	5.039122	0.462283	0.686368	-0.22409	13	99049874	F	II	FARP1	Body	opensea	Body-opens		rs7652923 1;30;33;40
cg0912776	0.229345	0.600401	11.61929	4.09E-06	0.000604	5.038315	0.485729	0.715074	-0.22935	6	1.47E+08	F	II	STXBP5-AS:	Body	opensea	Body-opens		rs5434843 0;33;37
cg0040214	0.20009	0.493714	11.61906	4.09E-06	0.000604	5.038176	0.393669	0.593759	-0.20009	2	53631176	R	II		IGR	opensea	IGR-opense		rs1136020 47;2;1
cg0290254	0.229671	0.541858	11.61894	4.09E-06	0.000604	5.038102	0.427023	0.656693	-0.22967	16	50136493	R	II	HEATR3	Body	opensea	Body-opens		rs5563205 46;1
cg1345506	0.210791	0.47751	11.61807	4.09E-06	0.000604	5.037554	0.372114	0.582906	-0.21079	4	7385791	R	II	SORCS2	Body	opensea	Body-opens		rs5540836 36
cg1654826	0.213979	0.654463	11.61481	4.1E-06	0.000604	5.035519	0.547473	0.761452	-0.21398	14	1.07E+08	R	II		IGR	opensea	IGR-opense		rs5877581 1
cg0712987	0.20168	0.649012	11.61448	4.1E-06	0.000604	5.035313	0.548172	0.749852	-0.20168	3	1.44E+08	R	II		IGR	opensea	IGR-opense		rs1485673 16
cg1489303	0.223823	0.571836	11.61233	4.1E-06	0.000605	5.033966	0.459925	0.683748	-0.22382	8	26323465	R	II		IGR	opensea	IGR-opense		rs5744952 48;1
cg2202426	0.227832	0.569977	11.60867	4.11E-06	0.000606	5.031683	0.456061	0.683893	-0.22783	21	26799096	R	II	LINC00158	Body	opensea	Body-opens		rs5596907 37;9
cg0163006	0.258083	0.467075	11.60763	4.12E-06	0.000606	5.031031	0.338034	0.596117	-0.25808	8	1.02E+08	R	II	SNX31	Body	opensea	Body-opens		rs7929575 41;13;10
cg1049509	0.208498	0.497562	11.60603	4.12E-06	0.000606	5.03003	0.393313	0.601811	-0.2085	6	80517072	F	II	C6orf7	Body	opensea	Body-opens		rs1819798 1;37
cg1481374	0.252202	0.543665	11.606	4.12E-06	0.000606	5.03001	0.417564	0.669766	-0.2522	21	19859651	R	II		IGR	opensea	IGR-opense		rs1145617 1
cg1179597	0.233706	0.635285	11.60442	4.13E-06	0.000607	5.02902	0.518432	0.752138	-0.23371	11	1.22E+08	R	II	BLID	TSS1500	opensea	TSS1500-op		rs5506605 46
cg1674404	0.22488	0.612779	11.60402	4.13E-06	0.000607	5.028772	0.50034	0.725219	-0.22488	2	39924833	F	II	TMEM178A	5'UTR	opensea	5'UTR-oper		rs1853893 0
cg2580667	0.245027	0.526675	11.60324	4.13E-06	0.000607	5.028283	0.404162	0.649189	-0.24503	2	58867041	F	II	LINC01122	Body	opensea	Body-opens		rs1143399 0;7;18;20;37;38
cg2718215	0.219349	0.505276	11.60305	4.13E-06	0.000607	5.028166	0.395601	0.614951	-0.21935	6	33241226	F	II	RPS18	Body	shore	Body-shore	chr6:33239	rs5705039 33
cg2231396	0.201601	0.706969	11.60291	4.13E-06	0.000607	5.028077	0.606169	0.807769	-0.2016	7	13117510	R	II		IGR	opensea	IGR-opense		rs5658181 28
cg1100096	0.21046	0.659648	11.60239	4.13E-06	0.000607	5.027753	0.554418	0.764878	-0.21046	17	75337274	R	II	SEPT9	Body	opensea	Body-opens		
cg1208130	0.227244	0.518249	11.60203	4.13E-06	0.000607	5.027525	0.404628	0.631871	-0.22724	2	38346853	F	II		IGR	opensea	IGR-opense		rs5649776 0;20;49
cg1241687	0.22343	0.503768	11.60056	4.14E-06	0.000607	5.026606	0.392053	0.615484	-0.22343	3	23431400	F	II	UBE2E2	Body	opensea	Body-opens		rs1485225 46
cg2476166	0.242376	0.483087	11.59986	4.14E-06	0.000607	5.026169	0.3619	0.604275	-0.24238	2	1.09E+08	F	II		IGR	opensea	IGR-opense		
cg0805963	0.261876	0.425424	11.59951	4.14E-06	0.000607	5.025952	0.294486	0.556362	-0.26188	8	6274876	R	II	MCPH1	Body	opensea	Body-opens		rs5367941 38
cg2717115	0.20636	0.521253	11.5973	4.14E-06	0.000607	5.024568	0.418073	0.624433	-0.20636	2	71698651	F	II	DYSF	Body	opensea	Body-opens		rs1861231 37
cg1765401	0.223575	0.724041	11.59635	4.15E-06	0.000607	5.023971	0.612253	0.835828	-0.22357	6	1.44E+08	R	II	PLAGL1	5'UTR	opensea	5'UTR-oper		rs1858199 35
cg2069078	0.226765	0.624081	11.5955	4.15E-06	0.000607	5.023437	0.510699	0.737463	-0.22676	1	26937762	R	II		IGR	opensea	IGR-opense		rs5696233 2
cg0010212	0.227911	0.58255	11.59546	4.15E-06	0.000607	5.023411	0.468595	0.696506	-0.22791	8	1.31E+08	F	II	FAM49B	Body	opensea	Body-opens		rs5756457 1;19
cg2206572	0.212084	0.574343	11.58995	4.16E-06	0.000608	5.019963	0.468301	0.680385	-0.21208	10	61769702	R	II		IGR	opensea	IGR-opense		rs5681602 8;2
cg1581352	0.217346	0.505875	11.58989	4.16E-06	0.000608	5.019929	0.397202	0.614548	-0.21735	11	48028299	R	II	PTPRJ	Body	opensea	Body-opens		
cg1512253	0.214406	0.796574	11.58985	4.16E-06	0.000608	5.019903	0.689371	0.903777	-0.21441	11	1.12E+08	R	II	LOC28314(	TSS1500	opensea	TSS1500-op		
cg2199342	0.224188	0.543028	11.58583	4.17E-06	0.000609	5.017383	0.430934	0.655122	-0.22419	9	1.17E+08	F	II	ZNF618	Body	opensea	Body-opens		rs5378378 22;44
cg2660789	0.246612	0.568635	11.58143	4.18E-06	0.000609	5.014625	0.445328	0.691941	-0.24661	1	1.68E+08	R	II	CREG1	3'UTR	opensea	3'UTR-oper		rs1927989 26;38
cg0450891	0.203429	0.58912	11.58003	4.19E-06	0.000609	5.013746	0.487405	0.690834	-0.20343	7	16844714	R	II	AGR2	1stExon	opensea	1stExon-op		rs1895509 51;46;35;3
cg0679159	0.27818	0.449296	11.57955	4.19E-06	0.000609	5.013446	0.310206	0.588385	-0.27818	6	32823292	R	II	PSMB9	Body	shore	Body-shore	chr6:32820	rs3763346 37;1
cg2207403	0.203086	0.479169	11.57859	4.19E-06	0.000609	5.012845	0.377626	0.580712	-0.20309	15	61356067	F	II	RORA	Body	opensea	Body-opens		rs5386520 3
cg2640154	0.290149	0.54057	11.57551	4.2E-06	0.00061	5.010909	0.395495	0.685644	-0.29015	6	91078974	F	II		IGR	opensea	IGR-opense		rs5483666 42;47
cg1102082	0.231345	0.571401	11.57508	4.2E-06	0.00061	5.010642	0.455728	0.687073	-0.23134	15	79128547	R	II		IGR	shelf	IGR-shelf	chr15:7917	rs4539564 50;28;2
cg1981577	0.241413	0.634729	11.57486	4.2E-06	0.00061	5.010503	0.514023	0.755435	-0.24141	14	1.03E+08	R	II	MOK	Body	opensea	Body-opens		rs5731819 0;1
cg0591741	0.210196	0.571085	11.57327	4.21E-06	0.00061	5.009506	0.465987	0.676183	-0.2102	5	1.24E+08	R	II	ZNF608	Body	opensea	Body-opens		
cg0195197	0.237568	0.59867	11.57269	4.21E-06	0.00061	5.009142	0.479886	0.717455	-0.23757	9	1.1E+08	F	II	ZNF462	5'UTR	opensea	5'UTR-oper		rs1382992 0;1;30;50
cg0645082	0.212466	0.468337	11.57248	4.21E-06	0.00061	5.009012	0.362104	0.57457	-0.21247	16	77810038	F	II		IGR	opensea	IGR-opense		rs3691572 1;10;33
cg1201989	0.258525	0.44417	11.57242	4.21E-06	0.00061	5.008973	0.314907	0.573432	-0.25852	3	42950090	R	II	ZNF662	Body	shore	Body-shore	chr3:42947	rs1510392 21;18;1
cg0834277	0.207011	0.485375	11.56901	4.22E-06	0.000611	5.006834	0.381869	0.58888	-0.20701	5	1.37E+08	F	II	SPOCK1	Body	opensea	Body-opens		rs1133823 1
cg0470618	0.217413	0.52729	11.5684	4.22E-06	0.000611	5.006452	0.418583	0.635996	-0.21741	12	13259327	F	II		IGR	opensea	IGR-opense		rs7315035 51
cg0255026	0.203446	0.463173	11.56829	4.22E-06	0.000611	5.006379	0.36145	0.564896	-0.20345	22	21926094	F	II	UBE2L3	Body	shelf	Body-shelf	chr22:2192	
cg1626680	0.262506	0.485557	11.56635	4.22E-06	0.000612	5.005164	0.354304	0.61681	-0.26251	20	30943371	R	II		IGR	shelf	IGR-shelf	chr20:3094	
cg0200080	0.231197	0.611361	11.56489	4.23E-06	0.000612	5.004245	0.495763	0.72696	-0.2312	7	27156062	F	II	HOXA3	5'UTR	shore	5'UTR-shor	chr7:27154	rs5701181 0;51
cg0525129	0.238123	0.419025	11.56439	4.23E-06	0.000612	5.003934	0.299964	0.538087	-0.23812	17	25799447	F	II	KSR1	5'UTR	opensea	5'UTR-oper		rs5563231 2;14;45
cg1390021	0.208936	0.57483	11.56289	4.23E-06	0.000612	5.00299	0.470362	0.679298	-0.20894	21	35881375	R	II	KCNE1	5'UTR	opensea	5'UTR-oper		rs5581689 51
cg1343667	0.20837	0.431273	11.56197	4.24E-06	0.000612	5.002412	0.327088	0.535458	-0.20837	9	1.12E+08	R	II		IGR	shelf	IGR-shelf	chr9:11208	rs5313338 30
cg2232573	0.200254	0.49869	11.56112	4.24E-06	0.000612	5.001882	0.398563	0.598817	-0.20025	20	23236654	R	II		IGR	opensea	IGR-opense		rs5599491 46;15
cg2273954	0.209227	0.64762	11.55979	4.24E-06	0.000612	5.001041	0.543007	0.752233	-0.20923	2	1.09E+08	F	II	LINC01594	TSS1500	opensea	TSS1500-op		rs1926302 1;40
cg1878183	0.206542	0.599076	11.55933	4.24E-06	0.000612	5.000756	0.495805	0.702347	-0.20654	2	2.43E+08	R	II		IGR	opensea	IGR-opense		
cg0377503	0.266218	0.428593	11.55849	4.25E-06	0.000612	5.000224	0.295484	0.561702	-0.26622	2	88874141	R	II	LOC101928	Body	opensea	Body-opens		rs5445031 50;28;1
cg1004135	0.223378	0.555153	11.5556	4.25E-06	0.000613	4.998411	0.443465	0.666842	-0.22338	6	21557585	R	II		IGR	opensea	IGR-opense		
cg1867560	0.210753	0.61684	11.55177	4.26E-06	0.000614	4.996003	0.511463	0.722216	-0.21075	8	19145981	R	II		IGR	opensea	IGR-opense		rs5473031 2
cg2708356	0.249817	0.512077	11.54641	4.28E-06	0.000615	4.99263	0.387168	0.636985	-0.24982	18	34522301	F	II	KIAA1328	Body	opensea	Body-opens		rs5519001 42
cg1197735	0.204051	0.432577	11.54526	4.28E-06	0.000615	4.991906	0.330551	0.534603	-0.20405	8	32611018	R	II	NRG1	Body	opensea	Body-opens		rs1434686 5
cg0604758	0.229944	0.514998	11.54326	4.29E-06	0.000615	4.990649	0.400026	0.629971	-0.22994	3	1.17E+08	F	II		IGR	opensea	IGR-opense		rs1475468 34;39
cg2160774	0.218755	0.646706	11.54163	4.29E-06	0.000615	4.989623	0.537328	0.756083	-0.21876	12	22613314	F	II	C2CD5	Body	opensea	Body-opens		rs5665212 30
cg0750432	0.25091	0.562871	11.54115	4.29E-06	0.000615	4.989322	0.437416	0.688325	-0.25091	2	98860544	F	II	VWA3B	Body	opensea	Body-opens		rs7773337 18;29
cg1368113	0.22854	0.598448	11.54092	4.29E-06	0.000615	4.989179	0.484178	0.712718	-0.22854	11	18600705	F	II	UEVLD	5'UTR	opensea	5'UTR-oper		rs5352393 20;36;37;50
cg2027015	0.236992	0.44257	11.54067	4.29E-06	0.000615	4.989019	0.324075	0.561066	-0.23699	5	32542511	R	II		IGR	opensea	IGR-opense		rs5486926 1
cg2349386	0.228365	0.541521	11.53953	4.3E-06	0.000615	4.988303	0.427339	0.655703	-0.22836	4	1.2E+08	R	II	SYNPO2	Body	opensea	Body-opens		rs5426688 27;7
cg1372761	0.247276	0.589872	11.53883	4.3E-06	0.000615	4.98786	0.466234	0.71351	-0.24728	19	7145317	F	II	INSR	Body	shelf	Body-shelf	chr19:7142	

cg0510677	0.240323	0.53662	11.53867	4.3E-06	0.000615	4.987762	0.416459	0.656782	-0.24032	8	97598420	R	II	SDC2	Body	opensea	Body-opens		rs3721959 2
cg0433349	0.284219	0.4762	11.53617	4.31E-06	0.000616	4.98619	0.334091	0.61831	-0.28422	17	18763778	R	II	PRPSAP2	5'UTR	shore	5'UTR-shore	chr17:1876	
cg1484244	0.201932	0.65257	11.53514	4.31E-06	0.000616	4.985538	0.551604	0.753536	-0.20193	2	28080488	F	II	RBKS	Body	opensea	Body-opens		rs5493976 0
cg0176122	0.219624	0.552986	11.53261	4.31E-06	0.000616	4.983945	0.443174	0.662798	-0.21962	2	1.15E+08	F	II	DPP10	Body	shelf	Body-shelf	chr2:11541	
cg1934366	0.202839	0.691009	11.53035	4.32E-06	0.000617	4.982521	0.58959	0.792429	-0.20284	8	1.42E+08	R	II	PTK2	Body	opensea	Body-opens		rs8016894 24;2;1
cg2227482	0.217965	0.492067	11.52968	4.32E-06	0.000617	4.982103	0.383084	0.601049	-0.21796	7	83702993	F	II	SEMA3A	Body	opensea	Body-opens		rs1821651 0;26;39
cg0778728	0.203178	0.585922	11.5295	4.32E-06	0.000617	4.981985	0.484333	0.687511	-0.20318	15	45613679	R	II		IGR	opensea	IGR-opense		rs1444828 43;33;26;5;2;1
cg1189300	0.233645	0.496686	11.52891	4.33E-06	0.000617	4.981613	0.379864	0.613509	-0.23365	9	1.14E+08	F	II	LPAR1	5'UTR	opensea	5'UTR-open		rs5516977 5
cg2036635	0.216646	0.471807	11.52722	4.33E-06	0.000618	4.980547	0.363484	0.58013	-0.21665	2	67730094	R	II		IGR	opensea	IGR-opense		rs5408008 17
cg1327889	0.223304	0.519889	11.52606	4.33E-06	0.000618	4.979816	0.408237	0.631541	-0.2233	13	84451164	R	II		IGR	shelf	IGR-shelf	chr13:8445	rs5749292 31;1
cg2731918	0.215383	0.522888	11.52458	4.34E-06	0.000618	4.978888	0.415196	0.630579	-0.21538	11	65880175	R	II	PACS1	Body	opensea	Body-opens		
cg1711034	0.222476	0.582972	11.5245	4.34E-06	0.000618	4.978836	0.471735	0.69421	-0.22248	20	61554899	R	II	DIDO1	5'UTR	shelf	5'UTR-shelf	chr20:615	rs5582710 36;14
cg0063840	0.215043	0.576035	11.52285	4.34E-06	0.000618	4.977793	0.468513	0.683556	-0.21504	2	80471121	F	II	CTNNA2	Body	opensea	Body-opens		rs1844198 0;1;8;26
cg0114319	0.21257	0.541856	11.52146	4.35E-06	0.000618	4.976917	0.435571	0.648141	-0.21257	11	56000767	F	II	OR5T2	TSS200	opensea	TSS200-ope		rs5339991 28;43
cg1508728	0.209071	0.633083	11.52034	4.35E-06	0.000618	4.97621	0.528547	0.737618	-0.20907	6	89854631	F	II	PM20D2	TSS1500	shore	TSS1500-sh	chr6:8985	rs5339162 6
cg1509836	0.20553	0.597785	11.51992	4.35E-06	0.000618	4.97595	0.49502	0.70055	-0.20553	15	92106338	F	II		IGR	opensea	IGR-opense		rs1429757 1;13;42
cg1595050	0.221981	0.606354	11.51927	4.35E-06	0.000618	4.97554	0.495363	0.717344	-0.22198	13	91145493	F	II		IGR	opensea	IGR-opense		rs1401838 12;17
cg0137681	0.208468	0.639536	11.5181	4.35E-06	0.000618	4.9748	0.535302	0.74377	-0.20847	1	2.38E+08	R	II	ZP4	TSS200	opensea	TSS200-ope		rs5690251 47;16;1
cg1589295	0.206976	0.611008	11.51794	4.35E-06	0.000618	4.974697	0.50752	0.714497	-0.20698	8	23168643	R	II	LOXL2	Body	opensea	Body-opens		
cg2205627	0.201335	0.423918	11.51748	4.36E-06	0.000618	4.97441	0.323251	0.524586	-0.20133	7	77612433	F	II		IGR	opensea	IGR-opense		rs1922993 0
cg2311252	0.217236	0.471243	11.51669	4.36E-06	0.000618	4.973909	0.362625	0.579861	-0.21724	22	30943828	R	II	SEC14L6	TSS1500	opensea	TSS1500-op		rs1698876 9;2
cg2132488	0.201214	0.554301	11.51479	4.36E-06	0.000619	4.972713	0.453695	0.654908	-0.20121	3	64801329	R	II	MIR548A2	Body	opensea	Body-opens		rs1833378 43;18
cg0297050	0.2276	0.480258	11.51367	4.37E-06	0.000619	4.972006	0.366458	0.594058	-0.2276	3	1.02E+08	F	II	LOC15222	Body	opensea	Body-opens		rs5472781 8
cg2588911	0.208881	0.516809	11.51294	4.37E-06	0.000619	4.971542	0.41236	0.62125	-0.20888	11	1.25E+08	R	II	ROBO4	Body	opensea	Body-opens		rs1397883 15
cg2119404	0.254857	0.479034	11.51282	4.37E-06	0.000619	4.971467	0.351606	0.606463	-0.25486	3	11862088	R	II	TAMM41	Body	opensea	Body-opens		
cg1937166	0.206655	0.39818	11.51108	4.37E-06	0.000619	4.97037	0.294852	0.501507	-0.20665	1	2.05E+08	F	II	LRRN2	5'UTR	opensea	5'UTR-open		rs5429887 1;20;49
cg0590491	0.235316	0.661924	11.51074	4.37E-06	0.000619	4.970154	0.544266	0.779582	-0.23532	6	1.56E+08	R	II		IGR	opensea	IGR-opense		rs5567461 2
cg2597035	0.240777	0.481453	11.51001	4.38E-06	0.000619	4.969693	0.361065	0.601842	-0.24078	15	59514661	F	II	MYO1E	Body	opensea	Body-opens		rs5762771 35;46
cg0534437	0.219099	0.45185	11.50971	4.38E-06	0.000619	4.969508	0.342301	0.561399	-0.2191	12	60735995	R	II		IGR	opensea	IGR-opense		rs5663435 1
cg2423935	0.239514	0.576307	11.50709	4.38E-06	0.000619	4.96785	0.45655	0.696064	-0.23951	14	47812319	R	II	MDGA2	1stExon	opensea	1stExon-op		rs1471909 46;32;10
cg0539749	0.226147	0.62554	11.50334	4.4E-06	0.00062	4.965484	0.512467	0.738614	-0.22615	13	33105476	R	II	N4BP2L2	Body	opensea	Body-opens		rs1497398 31;18;13;8
cg1354054	0.253013	0.538296	11.50251	4.4E-06	0.00062	4.964961	0.411789	0.664802	-0.25301	19	16424005	R	II		IGR	opensea	IGR-opense		rs1440038 48;28
cg2463970	0.270643	0.548447	11.50218	4.4E-06	0.00062	4.964753	0.413126	0.683768	-0.27064	1	64986155	R	II	CACHD1	Body	opensea	Body-opens		rs5298374 13
cg0888747	0.228635	0.578365	11.50122	4.4E-06	0.00062	4.964146	0.464047	0.692682	-0.22864	22	22516233	R	II		IGR	opensea	IGR-opense		rs5583233 19
cg0744899	0.219466	0.621619	11.50094	4.4E-06	0.00062	4.96397	0.511887	0.731352	-0.21947	7	77779958	F	II	MAGI2	Body	opensea	Body-opens		rs5650020 0;1
cg1277418	0.261238	0.397902	11.49626	4.41E-06	0.000621	4.961013	0.267283	0.528521	-0.26124	1	1.92E+08	F	II		IGR	opensea	IGR-opense		rs1124379 44;47
cg1875823	0.216387	0.565977	11.49484	4.42E-06	0.000621	4.960115	0.457784	0.674171	-0.21639	17	53626325	F	II		IGR	opensea	IGR-opense		
cg0825018	0.2153	0.505546	11.49354	4.42E-06	0.000621	4.959289	0.397896	0.613195	-0.2153	2	17482455	F	II		IGR	opensea	IGR-opense		rs7645488 17
cg2616901	0.213698	0.58338	11.49253	4.43E-06	0.000621	4.958653	0.47653	0.690229	-0.2137	6	74270238	R	II		IGR	opensea	IGR-opense		rs1904439 28;4
cg1293093	0.233529	0.504487	11.49189	4.43E-06	0.000621	4.958249	0.387723	0.621252	-0.23353	11	5372503	R	II	OR51B6	TSS1500	opensea	TSS1500-op		rs5640550 47;45;41;35;25;12;2;1
cg1100313	0.263201	0.473412	11.49184	4.43E-06	0.000621	4.958216	0.341812	0.605013	-0.2632	1	1.59E+08	R	II	AIM2	5'UTR	opensea	5'UTR-open		rs4126445 15;10;2;1
cg0531250	0.23394	0.479866	11.4915	4.43E-06	0.000621	4.958001	0.362896	0.596836	-0.23394	18	11114858	R	II	PIEZO2	Body	opensea	Body-opens		rs5781550 42
cg1447887	0.225213	0.46896	11.4905	4.43E-06	0.000621	4.957371	0.356353	0.581566	-0.22521	1	2.26E+08	F	II		IGR	opensea	IGR-opense		rs1879971 37
cg0527938	0.204759	0.581634	11.48863	4.44E-06	0.000621	4.956188	0.479255	0.684014	-0.20476	22	21321253	R	II	AIFM3	5'UTR	shore	5'UTR-shore	chr22:213	rs5453283 44;9
cg2740564	0.264257	0.457298	11.48711	4.44E-06	0.000621	4.955227	0.325169	0.589426	-0.26426	5	42547112	F	II	GHR	TSS1500	opensea	TSS1500-op		
cg1212852	0.20398	0.654689	11.48707	4.44E-06	0.000621	4.955199	0.552699	0.756679	-0.20398	7	93381385	F	II		IGR	opensea	IGR-opense		rs1489232 0;34
cg1219727	0.205844	0.592067	11.48685	4.44E-06	0.000621	4.955059	0.489145	0.694989	-0.20584	10	75037719	F	II	CFAP70	Body	opensea	Body-opens		rs5453849 6
cg1334694	0.208746	0.561546	11.48466	4.45E-06	0.000621	4.953678	0.457173	0.665919	-0.20875	17	40207636	R	II		IGR	opensea	IGR-opense		
cg1040201	0.209934	0.525327	11.47948	4.46E-06	0.000623	4.950398	0.42036	0.630294	-0.20993	14	70375748	R	II	SMOC1	Body	opensea	Body-opens		
cg1036965	0.202768	0.478211	11.47834	4.46E-06	0.000623	4.949676	0.376827	0.579595	-0.20277	3	38679751	F	II	SCN5A	5'UTR	opensea	5'UTR-open		rs3764189 0;17
cg0986915	0.245284	0.624145	11.47781	4.47E-06	0.000623	4.949339	0.501503	0.746787	-0.24528	8	6405472	R	II	ANGPT2	Body	opensea	Body-opens		rs7607168 51;49;2
cg2675727	0.207675	0.635524	11.47695	4.47E-06	0.000623	4.948796	0.531686	0.739361	-0.20768	16	85238227	R	II		IGR	opensea	IGR-opense		rs5533314 40
cg1025952	0.207547	0.439997	11.47624	4.47E-06	0.000623	4.948342	0.336223	0.54377	-0.20755	15	79428573	R	II		IGR	opensea	IGR-opense		rs1837946 14;12;11
cg0603061	0.203979	0.59188	11.47512	4.47E-06	0.000623	4.947635	0.489891	0.69387	-0.20398	1	37903193	F	II		IGR	opensea	IGR-opense		
cg1382107	0.239079	0.494316	11.47462	4.48E-06	0.000623	4.947318	0.374776	0.613855	-0.23908	6	68936152	F	II		IGR	opensea	IGR-opense		rs5329317 0
cg1713688	0.219336	0.522162	11.47447	4.48E-06	0.000623	4.947223	0.412494	0.631829	-0.21934	9	682998	F	II	KANK1	Body	opensea	Body-opens		rs1850826 23;26
cg0710254	0.209857	0.578601	11.47358	4.48E-06	0.000623	4.94666	0.473672	0.68353	-0.20986	9	1.34E+08	R	II	ABL1	Body	opensea	Body-opens		rs5341822 31
cg1197920	0.208662	0.549782	11.47304	4.48E-06	0.000623	4.946316	0.445451	0.654112	-0.20866	11	69006333	F	II		IGR	opensea	IGR-opense		rs5672761 21
cg1521259	0.203387	0.519467	11.47128	4.48E-06	0.000623	4.945203	0.417774	0.621161	-0.20339	1	2.05E+08	F	II	NFASC	Body	opensea	Body-opens		rs5284974 1
cg0230191	0.225993	0.507797	11.46829	4.49E-06	0.000623	4.943312	0.394801	0.620794	-0.22599	1	1.63E+08	R	II		IGR	opensea	IGR-opense		rs5680174 2
cg1089681	0.221782	0.541768	11.46738	4.5E-06	0.000623	4.942733	0.430877	0.652659	-0.22178	10	77800235	R	II	C10orf11	Body	opensea	Body-opens		rs1150156 40;14;11
cg2432769	0.231011	0.493326	11.46595	4.5E-06	0.000624	4.941828	0.37782	0.608831	-0.23101	2	1.52E+08	R	II		IGR	opensea	IGR-opense		rs7850628 48;34;18
cg1761894	0.216662	0.580438	11.46459	4.5E-06	0.000624	4.940965	0.472107	0.688769	-0.21666	19	2668273	F	II	GNG7	5'UTR	opensea	5'UTR-open		rs1913362 0;20;25;44
cg1133484	0.2068	0.627087	11.46421	4.5E-06	0.000624	4.940722	0.523687	0.730428	-0.2068	13	80276946	R	II		IGR	opensea	IGR-opense		rs1464036 10;2
cg1502845	0.208015	0.489438	11.46409	4.51E-06	0.000624	4.940645	0.38543	0.593445	-0.20801	3	58860290	R	I	C3orf67	Body	opensea	Body-opens		rs5576609 2
cg0493642	0.23562	0.428385	11.46225	4.51E-06	0.000624	4.939482	0.310576	0.546195	-0.23562	3	1.46E+08	R	II		IGR	opensea	IGR-opense		rs1685787 27;26;8
cg1429281	0.226254	0.555183	11.46209	4.51E-06	0.000624	4.93938	0.442056	0.668309	-0.22625	1	1.73E+08	F	II		IGR	opensea	IGR-opense		rs7613012 1;2;10;34
cg1181121	0.218575	0.519642	11.46037	4.52E-06	0.000625	4.938289	0.410355	0.628929	-0.21857	5	1.79E+08	F	II	ADAMTS2	Body	shore	Body-shore	chr5:1786	rs5558319 6;19
cg0662675	0.209697	0.52157	11.45928	4.52E-06	0.000625	4.937597	0.416722	0.626419	-0.2097	17	56082885	F	II	SFRS1	3'UTR	shore	3'UTR-shore	chr17:560	rs1420721 23
cg0285707	0.209441	0.435939	11.45453	4.53E-06	0.000626	4.934581	0.331218	0.540659	-0.20944	7	81746161	R	II	CACNA2D1	Body	opensea	Body-opens		rs1817546 43
cg2239427	0.219776	0.582244	11.45404	4.53E-06	0.000626	4.93427	0.472356	0.692132	-0.21978	2	2.08E+08	R	II		IGR	opensea	IGR-opense		rs1887091 47;21;2;1
cg1574050	0.206509	0.675319	11.45332	4.54E-06	0.000626	4.933815	0.572064	0.778573	-0.20651	3	99988576	F	II	TBC1D23	Body	opensea	Body-opens		
cg2251422	0.243845	0.640429	11.45327	4.54E-06	0.000626	4.933783	0.518506	0.762351	-0.24384	1	94118054	R	II	BCAR3	Body	opensea	Body-opens		rs1851198 18;2
cg2119172	0.25556	0.582011	11.45058	4.54E-06	0.000626	4.932076	0.454231	0.709791	-0.25556	20	46086203	F	II		IGR	opensea	IGR-opense		rs1180112 1;50
cg1310783	0.233766	0.528535	11.45011	4.55E-06	0.000626	4.931776	0.411652	0.645418	-0.23377	1	14847711	F	II		IGR	opensea	IGR-opense		rs1921651 1;16;43;44
cg0248501	0.253543	0.535638	11.4483	4.55E-06	0.000626	4.93063	0.408866	0.662409	-0.25354	10	1.26E+08	F	II		IGR	opensea	IGR-opense		rs1402642 0;1;47
cg0875742	0.217447	0.627511	11.44727	4.55E-06	0.000626	4.929974	0.518787	0.736234	-0.21745	9	97879242	F	II	FANCC	Body	opensea	Body-opens		
cg2017199	0.23866	0.513462	11.44662	4.56E-06	0.000626	4.929561	0.394132	0.632792	-0.23866	8	67343066	R	II		IGR	shore	IGR-shore	chr8:6734	rs3524340 27;22
cg1358859	0.210317	0.668158	11.44543	4.56E-06	0.000627	4.928808	0.563	0.773317	-0.21032	9	1.31E+08	F	II	SLC27A4	TSS1500	shore	TSS1500-sh	chr9:1311	rs1135998 40
cg1617835	0.224283	0.607642	11.44362	4.56E-06	0.000627	4.927657	0.4955	0.719783	-0.22428	5	81630266	R	II		IGR	opensea	IGR-opense		rs5642772 22
cg2398990	0.222479	0.519687	11.44305	4.57E-06	0.000627	4.927295	0.408448	0.630927	-0.22248	7	1.37E+08	F	II	DGKI	Body	opensea	Body-opens		rs5726609 29
cg1671347	0.212775	0.657101	11.44088	4.57E-06	0.000627	4.925913	0.550713	0.763488	-0.21278	3	1.29E+08	R	II		IGR	opensea	IGR-opense		rs1838689 2;1
cg1713618	0.259725	0.536059	11.43441	4.59E-06	0.000629	4.921801	0.406197	0.665921	-0.25972	5	6030096	R	II		IGR	opensea	IGR-opense		rs1880286 30;2
cg2119057	0.237626	0.395502	11.43408	4.59E-06	0.000629	4.921596	0.276689	0.514314	-0.23763	13	74818020	F	II		IGR	opensea	IGR-opense		rs5415324 15
cg0197900	0.224116	0.588647	11.43278	4.6E-06	0.000629	4.920765	0.476589	0.700705	-0.22412	2	1.82E+08	F	II	SCHLAP1	Body	opensea	Body-opens		rs1865940 17;24
cg2705779	0.222829	0.557396	11.43237	4.6E-06	0.000629	4.920504	0.445982	0.668811	-0.22283	20	19569677	R	II	SLC24A3	Body	opensea	Body-opens		rs3686492 46;44
cg1865030	0.211257	0.521037	11.43174	4.6E-06	0.000629	4.920104	0.415408	0.626666	-0.21126	4	17809468	F	II	DCAF16	5'UTR	shelf	5'UTR-shelf	chr4:1781	rs1478995 1;41
cg0856240	0.202634	0.53933	11.43124	4.6E-06	0.000629	4.91979	0.438013	0.640646	-0.20263	3	1.27E+08	F	II		IGR	opensea	IGR-opense		rs5596938 0;1;7;15;22;45
cg0348271	0.227811	0.42977	11.42994	4.6E-06	0.000629	4.91896	0.315864	0.543675	-0.22781	1	2.27E+08	R	II		IGR	opensea	IGR-opense		rs5735485 22
cg2629417	0.223044	0.62706	11.42993	4.6E-06	0.000629	4.918956	0.515538	0.738582	-0.22304	18	36583611	F	II		IGR	opensea	IGR-opense		rs1132145 0
cg0571077	0.236067	0.651642	11.4294	4.61E-06	0.000629	4.91862	0.533608	0.769675	-0.23607	2	65089821	F	II	LOC10192	Body	shelf	Body-shelf	chr2:6509	rs5470309 5;20
cg0876638	0.222231	0.572392	11.428	4.61E-06	0.000629	4.91773	0.461277	0.683507	-0.22223	17	31107923	F	II	MYO1D	Body	opensea	Body-opens		rs5532090 36
cg2266034	0.234605	0.519608	11.4267	4.61E-06	0.000629	4.916897	0.402306	0.636911	-0.23461	20	48322124	R	II	B4GALT5	Body	opensea	Body-opens		rs1509631 38;20;16;11;2;1
cg1343487	0.238665	0.560856	11.42624	4.61E-06	0.000629	4.916607	0.441523	0.680188	-0.23867	1	1.01E+08	F	II		IGR	opensea	IGR-opense		rs1152743 22;38
cg0053100	0.203825	0.552149	11.4251	4.62E-06	0.000629	4.915884	0.450237	0.654061	-0.20382	2	2.36E+08	F	II		IGR	opensea	IGR-opense		rs1449725 1;21
cg1808751	0.27007	0.548336	11.42479	4.62E-06	0.000629	4.915686	0.413301	0.683372	-0.27007	1	2.42E+08	F	II	KMO	Body	opensea	Body-opens		rs7709607 1;36
cg2400540	0.224728	0.61834	11.42351	4.62E-06	0.000629	4.914871	0.505976	0.730704	-0.22473	7	23060529	F	II		IGR	opensea	IGR-opense		rs1879437 1;17;49
cg0685883	0.20922	0.511352	11.42321	4.62E-06	0.000629	4.914681	0.406742	0.615962	-0.20922	6	1.47E+08	F	II	C6orf103	Body	opensea	Body-opens		rs1167077 1;20;33;43
cg0173965	0.215834	0.482778	11.4203	4.63E-06	0.00063	4.912825	0.374861	0.590695	-0.21583	8	29755226	F	II		IGR	opensea	IGR-opense		rs1896177 51
cg1274612	0.251072	0.425033	11.41801	4.64E-06	0.00063	4.911369	0.299497	0.550569	-0.25107	7	1.29E+08	R	II	SMO	Body	opensea	Body-opens		rs1157402 1
cg0685816	0.237205	0.600062	11.41713	4.64E-06	0.00063	4.910806	0.48146	0.718664	-0.2372	4	1.1E+08	R	II	COL25A1	Body	opensea	Body-opens		rs1866193 51;14
cg0780428	0.204499	0.527768	11.4131	4.65E-06	0.000631	4.908238	0.425518	0.630017	-0.2045	1	1.53E+08	R	II	SPRR2C	TSS1500	opensea	TSS1500-op		rs3730071 22;21;2;1
cg1771102	0.212629	0.581854	11.41127	4.66E-06	0.000632	4.907074	0.47554	0.688168	-0.21263	2	2.39E+08	R	II		IGR	opensea	IGR-opense		rs5289543 4
cg1833247	0.231791	0.592367	11.41108	4.66E-06	0.000632	4.906956	0.476471	0.708262	-0.23179	11	77437756	R	II	RSF1	Body	opensea	Body-opens		rs5675824 47
cg1342797	0.246494	0.585844	11.40555	4.68E-06	0.000633	4.903428	0.462598	0.709091	-0.24649	1	94615946	F	II		IGR	opensea	IGR-opense		
cg1528653	0.250988	0.572863	11.40372	4.68E-06	0.000633	4.902263	0.447369	0.698357	-0.25099	16	19823027	F	II	IQCK	Body	opensea	Body-opens		rs1470942 1;33
cg0900561	0.261957	0.474054	11.40315	4.68E-06	0.000633	4.901897	0.343076	0.605033	-0.26196	1	1.86E+08	R	II	HMCN1	Body	opensea	Body-opens		rs5664036 1
cg2709657	0.226479	0.592625	11.40153	4.69E-06	0.000633	4.900863	0.479386	0.705865	-0.22648	18	73141041	F	II	C18orf62	TSS1500	opensea	TSS1500-op		rs5658107 22;34
cg1191258	0.247042	0.54904	11.40112	4.69E-06	0.000633	4.900603	0.425519	0.672561	-0.24704	1	2.03E+08	F	II	MGAT4EP	Body	opensea	Body-opens		rs1092048 13;18;35;42
cg1848553	0.220755	0.628576	11.39951	4.69E-06	0.000634	4.899576	0.518198	0.738953	-0.22076	13	59547037	R	II		IGR	opensea	IGR-opense		rs5337434 2
cg0566178	0.23201	0.542818	11.39841	4.7E-06	0.000634	4.898877	0.426813	0.658823	-0.23201	1	1.16E+08	F	II	CASQ2	TSS1500	opensea	TSS1500-op		rs5631624 0
cg0548643	0.22292	0.620197	11.3982	4.7E-06	0.000634	4.898742	0.508737	0.731656	-0.22292	1	1.62E+08	R	II	NOS1AP	Body	opensea	Body-opens		rs5761393 1
cg1888038	0.224946	0.656331	11.39719	4.7E-06	0.000634	4.8981	0.543858	0.768804	-0.22495	6	32365113	R	II	BTNL2	Body	opensea	Body-opens		rs1178304 40;29;24;22;15;2;1
cg0073366	0.214937	0.555851	11.39635	4.7E-06	0.000634	4.897558	0.448382	0.663319	-0.21494	11	8274959	R	II	LMO1	Body	opensea	Body-opens		rs1171650 41
cg2239611	0.255637	0.606856	11.39466	4.71E-06	0.000634	4.896485	0.479037	0.734674	-0.25564	8	1.22E+08	F	II		IGR	opensea	IGR-opense		rs1825037 32
cg0994140	0.238792	0.57403	11.39339	4.71E-06	0.000634	4.89567	0.454634	0.693427	-0.23879	7	18901296	R	II	HDAC9	Body	opensea	Body-opens		rs1174447 45;40;8;2
cg2365346	0.242485	0.522983	11.39286	4.71E-06	0.000634	4.895335	0.40174	0.644225	-0.24248	2	1.42E+08	F	II	LRP1B	Body	opensea	Body-opens		
cg1619354	0.240285	0.490779	11.39234	4.71E-06	0.000634	4.895002	0.370636	0.610921	-0.24028	17	53536548	F	II		IGR	opensea	IGR-opense		rs5460189 10
cg2530440	0.225425	0.514938	11.39163	4.72E-06	0.000634	4.89455	0.402226	0.62765	-0.22542	17	32485152	R	II	ACCN1	TSS1500	shore	TSS1500-sh	chr17:324	rs1862167 41;1

cg2343967	0.2399	0.595575	11.39085	4.72E-06	0.000634	4.894053	0.475626	0.715525	-0.2399	8	97597395	F	II	SDC2	Body	opensea	Body-opens		rs5768048 6;7;13;23
cg0294431	0.208735	0.571089	11.3886	4.73E-06	0.000635	4.892614	0.466721	0.675456	-0.20873	7	3207719	R	II		IGR	opensea	IGR-opense		rs5409243 48;37;35
cg1386284	0.205113	0.540226	11.38708	4.73E-06	0.000635	4.891642	0.437669	0.642782	-0.20511	13	44272416	R	II	ENOX1	5'UTR	opensea	5'UTR-oper		rs5457994 30;20;8;4
cg0602256	0.249789	0.501309	11.38444	4.74E-06	0.000635	4.889958	0.376415	0.626203	-0.24979	2	2.43E+08	R	I	D2HGDH	Body	opensea	Body-opens		rs5707745 25;17;4;2
cg2313905	0.209159	0.667919	11.38058	4.75E-06	0.000636	4.887488	0.56334	0.772499	-0.20916	11	9847030	F	II	SBF2	Body	opensea	Body-opens		rs1902214 0;42;44;46;51
cg1550970	0.212141	0.332194	11.38039	4.75E-06	0.000636	4.88737	0.226124	0.438265	-0.21214	20	36363187	F	II	CTNNBL1	Body	opensea	Body-opens		rs1913148 1;15
cg0822905	0.208778	0.638584	11.38016	4.75E-06	0.000636	4.887221	0.534195	0.742973	-0.20878	13	25296292	R	II		IGR	shelf	IGR-shelf	chr13:2529	rs1449496 45;27;15
cg0530968	0.237903	0.564	11.37783	4.76E-06	0.000636	4.885732	0.445048	0.682951	-0.2379	18	34107658	R	II	FHOD3	Body	opensea	Body-opens		
cg2642744	0.210221	0.462039	11.37634	4.76E-06	0.000636	4.884779	0.356928	0.56715	-0.21022	20	30922353	R	II	KIF3B	3'UTR	opensea	3'UTR-oper		rs5356068 35;19
cg2542698	0.225551	0.502051	11.37598	4.76E-06	0.000636	4.88455	0.389275	0.614826	-0.22555	18	61683198	F	II		IGR	opensea	IGR-opense		rs1511701 28;46
cg2201455	0.228785	0.526098	11.37368	4.77E-06	0.000637	4.883079	0.411706	0.640491	-0.22879	7	1.23E+08	F	II		IGR	opensea	IGR-opense		rs1496829 1;12;31;34;34;49
cg2181093	0.223626	0.53822	11.37192	4.78E-06	0.000637	4.881956	0.426407	0.650033	-0.22363	15	62149916	F	II	VPS13C	Body	opensea	Body-opens		rs1136019 8
cg2251384	0.216053	0.518312	11.36806	4.79E-06	0.000638	4.879486	0.410285	0.626338	-0.21605	2	1.62E+08	F	II		IGR	opensea	IGR-opense		rs1140303 0;35
cg0386298	0.207831	0.43527	11.36767	4.79E-06	0.000638	4.879236	0.331355	0.539185	-0.20783	4	76596784	R	II	G3BP2	5'UTR	shore	5'UTR-shor	chr4:7659	
cg0724389	0.294451	0.471	11.36497	4.8E-06	0.000639	4.877509	0.323775	0.618226	-0.29445	3	18195180	F	II	LOC33986	Body	opensea	Body-opens		rs5592653 0;3
cg0494575	0.242243	0.485109	11.36346	4.8E-06	0.000639	4.876542	0.363988	0.606231	-0.24224	2	2.01E+08	F	II	SPATS2L	Body	opensea	Body-opens		rs5755708 1;32;46
cg0467723	0.201231	0.462662	11.36299	4.8E-06	0.000639	4.87624	0.362047	0.563278	-0.20123	11	45274796	R	II	SYT13	Body	opensea	Body-opens		rs5530070 22;2;1
cg1913935	0.238065	0.502486	11.36276	4.8E-06	0.000639	4.876093	0.383453	0.621518	-0.23806	5	1.11E+08	F	II		IGR	opensea	IGR-opense		rs1404316 1;40
cg2023087	0.220138	0.541244	11.36049	4.81E-06	0.000639	4.87464	0.431175	0.651313	-0.22014	2	1.21E+08	R	II		IGR	opensea	IGR-opense		rs5531886 2
cg0885261	0.236975	0.579821	11.35983	4.81E-06	0.000639	4.874214	0.461333	0.698308	-0.23697	4	1.58E+08	F	II	PDGFC	Body	opensea	Body-opens		rs5337306 7;40
cg0401142	0.249188	0.554686	11.35558	4.83E-06	0.000639	4.871498	0.430092	0.67928	-0.24919	3	1.41E+08	R	II	ZBTB38	5'UTR	opensea	5'UTR-oper		rs5310161 47;23;13;5
cg0068933	0.200621	0.708698	11.35488	4.83E-06	0.000639	4.871049	0.608387	0.809008	-0.20062	13	50024281	F	II	SETDB2	TSS1500	opensea	TSS1500-o		rs5332914 0;12;13
cg2550934	0.203624	0.625813	11.35472	4.83E-06	0.000639	4.870943	0.524001	0.727625	-0.20362	5	1.72E+08	F	II		IGR	opensea	IGR-opense		rs5637879 1
cg0101926	0.224617	0.618703	11.35452	4.83E-06	0.000639	4.870819	0.506395	0.731012	-0.22462	1	1.55E+08	F	II		IGR	shelf	IGR-shelf	chr1:1550	rs5329139 0;25
cg1188980	0.244845	0.496295	11.35267	4.84E-06	0.00064	4.869628	0.373873	0.618718	-0.24485	15	86253551	F	II	AKAP13	Body	opensea	Body-opens		rs5277685 1
cg1646274	0.216613	0.652205	11.35084	4.84E-06	0.00064	4.868455	0.543899	0.760512	-0.21661	14	59744940	F	II	DAAM1	Body	opensea	Body-opens		rs5352338 1
cg0434433	0.210532	0.522742	11.34995	4.84E-06	0.00064	4.867885	0.417475	0.628008	-0.21053	1	97109279	F	II		IGR	opensea	IGR-opense		
cg2635344	0.203497	0.448692	11.3499	4.84E-06	0.00064	4.867859	0.346944	0.550441	-0.2035	1	2.49E+08	F	II	OR2T4	TSS1500	opensea	TSS1500-o		rs1544175 26;42
cg1989766	0.206736	0.49778	11.34977	4.84E-06	0.00064	4.867775	0.394412	0.601148	-0.20674	21	28484462	F	II		IGR	opensea	IGR-opense		rs5293696 9;37
cg0591194	0.250482	0.557861	11.34969	4.84E-06	0.00064	4.867721	0.432621	0.683102	-0.25048	11	1.3E+08	F	II		IGR	opensea	IGR-opense		rs5380282 14;27;40
cg1023643	0.227245	0.504119	11.34638	4.85E-06	0.00064	4.865598	0.390497	0.617742	-0.22725	12	1.24E+08	F	I	SNRNP35	TSS1500	shore	TSS1500-sh	chr12:123	rs6646610 40;50
cg0778849	0.211642	0.574967	11.34452	4.86E-06	0.00064	4.86441	0.469146	0.680787	-0.21164	15	86236291	R	II	AKAP13	Body	opensea	Body-opens		rs7533160 2
cg0482289	0.258223	0.647471	11.34297	4.87E-06	0.00064	4.863413	0.518359	0.776582	-0.25822	10	16612764	R	II		IGR	opensea	IGR-opense		rs5866176 30;4
cg1174411	0.213373	0.664435	11.34294	4.87E-06	0.00064	4.863393	0.557749	0.771121	-0.21337	7	1.42E+08	R	II	TAS2R38	TSS200	opensea	TSS200-op		rs3718434 2
cg2266912	0.239889	0.534033	11.34232	4.87E-06	0.00064	4.862994	0.414089	0.653978	-0.23989	12	5024586	F	II	KCNA1	3'UTR	shelf	3'UTR-shelf	chr12:501	rs1883772 42
cg0971123	0.211755	0.5368	11.34224	4.87E-06	0.00064	4.862943	0.430922	0.642677	-0.21176	12	68553709	R	II	IFNG	TSS200	opensea	TSS200-op		rs2069710 36;12;8
cg0712465	0.229157	0.57005	11.34196	4.87E-06	0.00064	4.862766	0.455472	0.684629	-0.22916	5	96891229	F	II	LINC01340	Body	opensea	Body-opens		rs5488278 19
cg1709893	0.223538	0.678817	11.34022	4.87E-06	0.000641	4.861651	0.567048	0.790586	-0.22354	7	17746902	F	II		IGR	opensea	IGR-opense		rs5276122 4;51
cg1694082	0.200211	0.469927	11.33846	4.88E-06	0.000641	4.860518	0.369822	0.570033	-0.20021	1	2.25E+08	F	II		IGR	opensea	IGR-opense		rs1901611 7
cg0250099	0.204539	0.537925	11.33794	4.88E-06	0.000641	4.860186	0.435656	0.640195	-0.20454	10	45937578	R	II	ALOX5	Body	shore	Body-shore	chr10:4593	rs1420449 20;2
cg1534887	0.247786	0.460813	11.33686	4.88E-06	0.000641	4.859491	0.33692	0.584706	-0.24779	2	17854374	R	II	SMC6	Body	opensea	Body-opens		rs1505478 5
cg1629226	0.206589	0.700575	11.33537	4.89E-06	0.000641	4.858535	0.597281	0.80387	-0.20659	18	21797951	F	II	OSBPL1A	Body	opensea	Body-opens		
cg1880656	0.224392	0.594654	11.33371	4.89E-06	0.000641	4.857472	0.482458	0.706851	-0.22439	3	23803797	R	II		IGR	opensea	IGR-opense		
cg1385278	0.210833	0.462576	11.3335	4.89E-06	0.000641	4.857334	0.357159	0.567992	-0.21083	9	1.01E+08	R	II	GABBR2	Body	opensea	Body-opens		rs5639870 45;1
cg1381544	0.268193	0.533438	11.33246	4.9E-06	0.000641	4.856671	0.399342	0.667534	-0.26819	11	88028475	R	II	CTSC	Body	opensea	Body-opens		rs5606178 21;18;8
cg1568147	0.215554	0.507434	11.33182	4.9E-06	0.000641	4.856258	0.399657	0.615211	-0.21555	11	45128880	F	II	PRDM11	Body	opensea	Body-opens		rs5720915 19;21
cg0336129	0.222983	0.532356	11.33136	4.9E-06	0.000641	4.85596	0.420864	0.643847	-0.22298	14	57051003	F	II	TMEM260	Body	opensea	Body-opens		rs1820326 10
cg1114287	0.212523	0.546981	11.33065	4.9E-06	0.000641	4.855508	0.440719	0.653242	-0.21252	7	39971006	R	II		IGR	opensea	IGR-opense		
cg1417491	0.211819	0.293032	11.33019	4.91E-06	0.000641	4.855211	0.187123	0.398942	-0.21182	10	3824516	F	II	KLF6	Body	shore	Body-shore	chr10:382	rs1490189 20;39
cg1244016	0.261046	0.410838	11.33014	4.91E-06	0.000641	4.855177	0.280315	0.54136	-0.26105	15	57822016	F	II	CGNL1	Body	opensea	Body-opens		rs1435686 1;34;47
cg1095474	0.204401	0.559387	11.32979	4.91E-06	0.000641	4.854956	0.457186	0.661587	-0.2044	8	1.11E+08	F	II	KCNV1	Body	shelf	Body-shelf	chr8:1109	rs1917402 0;1;22
cg1163416	0.235254	0.627018	11.32936	4.91E-06	0.000641	4.854676	0.509391	0.744645	-0.23525	10	31893331	F	II		IGR	shore	IGR-shore	chr10:318	
cg1024167	0.216149	0.443975	11.32865	4.91E-06	0.000641	4.854223	0.335901	0.55205	-0.21615	13	31942320	F	II		IGR	opensea	IGR-opense		rs5280044 42
cg0525937	0.255045	0.526269	11.32665	4.92E-06	0.000641	4.852934	0.398746	0.653792	-0.25505	13	60308743	R	II	DIAPH3	Body	opensea	Body-opens		rs1180984 49;28;5;2
cg2263700	0.232924	0.455171	11.32502	4.92E-06	0.000641	4.851889	0.338708	0.571633	-0.23292	2	706036	R	II		IGR	opensea	IGR-opense		
cg1648023	0.238808	0.459628	11.32044	4.94E-06	0.000642	4.848949	0.340224	0.579033	-0.23881	20	9160245	F	II	PLCB4	5'UTR	opensea	5'UTR-oper		rs1509038 0
cg0815153	0.200204	0.645408	11.31586	4.95E-06	0.000642	4.846	0.545306	0.74551	-0.2002	18	53053356	F	II	TCF4	Body	opensea	Body-opens		rs7349294 42
cg0957039	0.211546	0.578752	11.31406	4.96E-06	0.000642	4.844843	0.472979	0.684525	-0.21155	11	34646080	F	II	EHF	5'UTR	opensea	5'UTR-oper		rs1476292 26
cg1887541	0.224201	0.602643	11.31399	4.96E-06	0.000642	4.844799	0.490543	0.714743	-0.2242	4	1.69E+08	R	II	PALLD	Body	opensea	Body-opens		
cg1644042	0.226342	0.560741	11.31381	4.96E-06	0.000642	4.844681	0.44757	0.673912	-0.22634	3	1.6E+08	F	II	IL12A-AS1	TSS1500	opensea	TSS1500-o		rs9852490 14;35
cg2386315	0.223135	0.501038	11.31308	4.96E-06	0.000642	4.844213	0.38947	0.612605	-0.22313	4	1.67E+08	R	II		IGR	opensea	IGR-opense		rs5318933 44;25
cg2525444	0.21945	0.646397	11.31238	4.96E-06	0.000642	4.843763	0.536672	0.756122	-0.21945	9	21408013	F	II	IFNA8	TSS1500	opensea	TSS1500-o		rs1494319 2;38;42
cg2490781	0.242455	0.618111	11.31237	4.96E-06	0.000642	4.843758	0.496884	0.739339	-0.24246	1	1.13E+08	F	II	CAPZA1	Body	opensea	Body-opens		rs5333111 24
cg1286460	0.215068	0.611282	11.31119	4.96E-06	0.000643	4.842999	0.503748	0.718816	-0.21507	11	1.15E+08	F	II	CADM1	Body	opensea	Body-opens		rs1482046 1;43
cg0547808	0.215998	0.529986	11.3105	4.97E-06	0.000643	4.842555	0.421987	0.637985	-0.216	7	1.34E+08	F	II	AKR1B15	TSS200	opensea	TSS200-op		rs5777005 0;25;27;32
cg1944131	0.206147	0.49733	11.30924	4.97E-06	0.000643	4.841743	0.394257	0.600404	-0.20615	3	1.24E+08	R	II	MYLK	Body	opensea	Body-opens		
cg0845623	0.205595	0.625501	11.30901	4.97E-06	0.000643	4.841597	0.522704	0.728298	-0.20559	1	2.04E+08	F	II		IGR	opensea	IGR-opense		rs5493951 0
cg2615013	0.212258	0.619709	11.3087	4.97E-06	0.000643	4.8414	0.51358	0.725838	-0.21226	5	1.23E+08	F	II		IGR	opensea	IGR-opense		rs5677587 1
cg2378620	0.223488	0.464616	11.30713	4.98E-06	0.000643	4.840384	0.352872	0.576359	-0.22349	10	77057425	R	II		IGR	shelf	IGR-shelf	chr10:770	
cg0345071	0.227442	0.470411	11.30658	4.98E-06	0.000643	4.840034	0.35669	0.584132	-0.22744	2	1.03E+08	R	II	IL18RAP	Body	opensea	Body-opens		rs1511331 40;29
cg2722246	0.203804	0.70751	11.30604	4.98E-06	0.000643	4.839684	0.605608	0.809412	-0.2038	3	1.12E+08	F	II	SLC9C1	Body	opensea	Body-opens		
cg0517476	0.208106	0.563884	11.30578	4.98E-06	0.000643	4.839519	0.459831	0.667937	-0.20811	11	84880848	F	II	DLG2	Body	opensea	Body-opens		rs5717830 1;35
cg0457547	0.23226	0.436623	11.3046	4.99E-06	0.000643	4.838761	0.320493	0.552753	-0.23226	6	1.27E+08	R	II		IGR	opensea	IGR-opense		rs1321172 21;1
cg1476379	0.211688	0.482824	11.302	4.99E-06	0.000644	4.837084	0.37698	0.588668	-0.21169	2	1.33E+08	F	II	NCKAP5	Body	shelf	Body-shelf	chr2:1334	
cg0725537	0.210049	0.488371	11.30116	5E-06	0.000644	4.836542	0.383346	0.593395	-0.21005	1	1.58E+08	R	II		IGR	opensea	IGR-opense		rs5570282 38;25;22;8
cg1530350	0.211746	0.39675	11.30006	5E-06	0.000644	4.835832	0.290877	0.502623	-0.21175	3	1.51E+08	R	II	CLRN1	3'UTR	opensea	3'UTR-oper		rs7771890 46;35;21;1
cg1759146	0.204364	0.699897	11.29946	5E-06	0.000644	4.83545	0.597715	0.802079	-0.20436	4	87102408	F	II	LOC10192	Body	opensea	Body-opens		rs1379720 0
cg0642216	0.211906	0.485224	11.29737	5.01E-06	0.000644	4.8341	0.379271	0.591177	-0.21191	1	26406870	F	II		IGR	opensea	IGR-opense		rs7664787 10;34
cg1280287	0.246656	0.454168	11.29668	5.01E-06	0.000644	4.833654	0.33084	0.577496	-0.24666	8	72751354	F	II		IGR	shelf	IGR-shelf	chr8:7275	rs5663303 0;1;30;36;46
cg1194257	0.220942	0.597665	11.29602	5.01E-06	0.000644	4.833229	0.487194	0.708136	-0.22094	2	48952008	R	II	LHCGR	Body	opensea	Body-opens		rs4366951 39;21;20;1
cg1015870	0.31373	0.534867	11.29595	5.01E-06	0.000644	4.833188	0.378002	0.691732	-0.31373	5	1.16E+08	F	II		IGR	opensea	IGR-opense		rs5320644 1;44
cg1172540	0.21222	0.577445	11.29512	5.02E-06	0.000644	4.832654	0.471335	0.683555	-0.21222	14	21625063	F	II	OR5AU1	TSS1500	opensea	TSS1500-o		rs5390788 23;37
cg0264221	0.216475	0.670689	11.29286	5.02E-06	0.000645	4.831199	0.562451	0.778926	-0.21648	4	89388747	R	II	HERC5	Body	opensea	Body-opens		rs5768238 25;24
cg2315639	0.241729	0.533388	11.29193	5.03E-06	0.000645	4.830598	0.412523	0.654252	-0.24173	3	1.7E+08	F	II	SAMD7	Body	opensea	Body-opens		rs9870311 18;48
cg1066914	0.246704	0.551082	11.29169	5.03E-06	0.000645	4.830439	0.427729	0.674434	-0.2467	3	1.51E+08	R	II	CLRN1	Body	opensea	Body-opens		rs5769908 38;22
cg0852779	0.223417	0.465032	11.29111	5.03E-06	0.000645	4.830071	0.353324	0.57674	-0.22342	8	21274126	R	II		IGR	opensea	IGR-opense		rs5554869 7;1
cg1669734	0.236199	0.454849	11.28951	5.03E-06	0.000645	4.829036	0.336749	0.572948	-0.2362	18	3319784	F	II		IGR	opensea	IGR-opense		rs1050231 13;32;38;41;51
cg2387466	0.211005	0.547399	11.28641	5.04E-06	0.000645	4.827037	0.441896	0.652901	-0.21101	1	1.63E+08	F	II	DDR2	TSS1500	opensea	TSS1500-o		rs1212633 40
cg2243225	0.200618	0.57203	11.28573	5.05E-06	0.000645	4.826596	0.471721	0.672339	-0.20062	1	2.19E+08	F	II	MIR548F3	Body	opensea	Body-opens		
cg1927312	0.227231	0.53253	11.2845	5.05E-06	0.000646	4.825808	0.418914	0.646146	-0.22723	10	60380342	F	II	BICC1	Body	opensea	Body-opens		rs1886466 0;24
cg1216198	0.229652	0.56545	11.28343	5.05E-06	0.000646	4.825114	0.450624	0.680276	-0.22965	10	49802919	R	II	ARHGAP22	Body	opensea	Body-opens		rs5622957 47;14
cg1735227	0.248902	0.573632	11.28071	5.06E-06	0.000647	4.823361	0.449181	0.698083	-0.2489	15	66795208	R	II	SNORD16	Body	opensea	Body-opens		rs2022393 49;42;38;24;2
cg1628421	0.20241	0.682285	11.27917	5.07E-06	0.000647	4.822368	0.58108	0.78349	-0.20241	18	9741480	R	II	RAB31	Body	opensea	Body-opens		rs1149817 34;2
cg2739742	0.202498	0.565464	11.27683	5.08E-06	0.000648	4.820859	0.464215	0.666713	-0.2025	2	1.53E+08	F	II	CACNB4	Body	opensea	Body-opens		rs5502444 4;12;15;24
cg2333028	0.229578	0.541758	11.27603	5.08E-06	0.000648	4.820344	0.426969	0.656547	-0.22958	11	19372234	R	II	NAV2	TSS200	opensea	TSS200-op		rs1816913 47;2
cg1104978	0.233895	0.618456	11.2757	5.08E-06	0.000648	4.820128	0.501508	0.735403	-0.23389	1	1.78E+08	F	II	RASAL2	Body	opensea	Body-opens		rs1275590 42
cg2305127	0.228362	0.521579	11.2754	5.08E-06	0.000648	4.819933	0.407398	0.63576	-0.22836	17	55253027	R	II		IGR	opensea	IGR-opense		rs7399179 42;37;26;23;1
cg1777513	0.219303	0.567429	11.27495	5.08E-06	0.000648	4.819646	0.457778	0.67708	-0.2193	5	59089139	F	II	PDE4D	Body	opensea	Body-opens		rs6133261 1;42;45
cg1230198	0.257822	0.505793	11.27472	5.08E-06	0.000648	4.819492	0.376882	0.634704	-0.25782	2	1E+08	R	II	AFF3	Body	opensea	Body-opens		rs1479200 47
cg1480000	0.208889	0.458995	11.27427	5.08E-06	0.000648	4.819202	0.354551	0.56344	-0.20889	6	1.12E+08	R	II	FYN	5'UTR	opensea	5'UTR-oper		rs5566641 42
cg0896456	0.223349	0.50514	11.27423	5.08E-06	0.000648	4.819179	0.393465	0.616814	-0.22335	7	1.16E+08	F	II	MET	5'UTR	opensea	5'UTR-oper		
cg0820668	0.200978	0.535284	11.27096	5.09E-06	0.000648	4.817067	0.434795	0.635772	-0.20098	21	45420795	R	II		IGR	opensea	IGR-opense		rs5445879 28;20;18;2;1
cg0624324	0.238171	0.437779	11.26956	5.1E-06	0.000648	4.816163	0.318694	0.556865	-0.23817	9	89988940	F	II		IGR	opensea	IGR-opense		
cg0042921	0.236582	0.628697	11.26948	5.1E-06	0.000648	4.816112	0.510405	0.746988	-0.23658	2	1.91E+08	R	II	PMS1	5'UTR	opensea	5'UTR-oper		rs5661803 47;32;20;2
cg0801466	0.211291	0.591092	11.26947	5.1E-06	0.000648	4.816103	0.485446	0.696737	-0.21129	6	22334619	R	II		IGR	opensea	IGR-opense		rs1820185 44
cg0512343	0.206329	0.541171	11.26933	5.1E-06	0.000648	4.816012	0.438006	0.644336	-0.20633	12	81392156	F	II		IGR	opensea	IGR-opense		rs1824781 4;34
cg1592756	0.22866	0.477526	11.26747	5.11E-06	0.000648	4.814812	0.363196	0.591856	-0.22866	4	42635853	F	II	ATP8A1	Body	opensea	Body-opens		rs5583712 1;15
cg1491417	0.241242	0.630004	11.2665	5.11E-06	0.000648	4.81419	0.509383	0.750626	-0.24124	3	31045929	R	II		IGR	opensea	IGR-opense		rs5330639 47
cg1936333	0.260029	0.496866	11.26488	5.11E-06	0.000649	4.813141	0.366851	0.62688	-0.26003	8	77897727	F	II	PEX2	5'UTR	opensea	5'UTR-oper		
cg1343457	0.222983	0.655482	11.26446	5.12E-06	0.000649	4.812868	0.543991	0.766973	-0.22298	1	2.06E+08	F	II	CTSE	TSS1500	opensea	TSS1500-o		
cg2099448	0.238446	0.483334	11.26215	5.12E-06	0.000649	4.811373	0.364111	0.602557	-0.23845	6	1.68E+08	R	II	TTLL2	Body	opensea	Body-opens		
cg0953026	0.22664	0.639727	11.25864	5.13E-06	0.00065	4.809108	0.526407	0.753047	-0.22664	10	1.05E+08	R	II		IGR	opensea	IGR-opense		rs5373852 2
cg1618153	0.225247	0.538401	11.25648	5.14E-06	0.00065	4.807711	0.425778	0.651024	-0.22525	18	28629471	F	II		IGR	opensea	IGR-opense		rs5716031 6
cg0157176	0.210098	0.697731	11.25609	5.14E-06	0.00065	4.807458	0.592682	0.80278	-0.2101	11	9973545	R	II	SBF2	Body	opensea	Body-opens		rs7938570 36;27;16
cg2189934	0.223069	0.522223	11.25557	5.15E-06	0.00065	4.807121	0.410689	0.633758	-0.22307	1	68419196	R	II	GNG12-AS1	Body	opensea	Body-opens		rs7567508 34;21
cg0969476	0.205439	0.596338	11.25446	5.15E-06	0.00065	4.806401	0.493618	0.699058	-0.20544	14	59408465	R	II	LINC01500	Body	opensea	Body-opens		rs5691589 44;39
cg1692580	0.232903	0.532553	11.25442	5.15E-06	0.00065	4.806377	0.416102	0.649005	-0.2329	14	73939631	R	II		IGR	opensea	IGR-opense		
cg0775576	0.21769	0.55351	11.25431	5.15E-06	0.00065	4.806305	0.444665	0.662355	-0.21769	12	22563748	R	II		IGR	opensea	IGR-opense		rs1857020 49;2;1
cg2753885	0.233874	0.56143	11.25366	5.15E-06	0.00065	4.805882	0.444493	0.678367	-0.23387	1	15106700	R	II	KIAA1026	Body	opensea	Body-opens		rs5565177 21

cg2082675	0.229678	0.566776	11.24779	5.17E-06	0.000651	4.80209	0.451937	0.681615	-0.22968	8	1.42E+08	R	II	PTK2	Body	opensea	Body-opens			
cg1665176	0.217356	0.546971	11.24774	5.17E-06	0.000651	4.802056	0.438293	0.655649	-0.21736	1	2.22E+08	F	II		IGR	opensea	IGR-opense			
cg1439369	0.215236	0.473002	11.24741	5.17E-06	0.000651	4.801842	0.365384	0.58062	-0.21524	6	1.66E+08	R	II	PDE10A	Body	opensea	Body-opens		rs5733046	41;37;2
cg0220880	0.22659	0.586143	11.24638	5.18E-06	0.000651	4.801175	0.472848	0.699438	-0.22659	15	52298012	R	II		IGR	opensea	IGR-opense		rs1381601	6;1
cg1079874	0.223667	0.359016	11.24542	5.18E-06	0.000651	4.800552	0.247182	0.47085	-0.22367	1	1.46E+08	R	II	CD160	TSS200	shore	TSS200-sho	chr1:1457	rs1859238	44;2
cg1443813	0.204374	0.571124	11.24502	5.18E-06	0.000651	4.800297	0.468936	0.673311	-0.20437	21	40170587	R	II		IGR	opensea	IGR-opense		rs5620753	30;5;2;1
cg1923518	0.259473	0.412361	11.24482	5.18E-06	0.000651	4.800167	0.282624	0.542097	-0.25947	3	1.56E+08	R	II	KCNAB1-AS	TSS200	opensea	TSS200-ope			
cg0385333	0.206274	0.506152	11.24412	5.18E-06	0.000651	4.799714	0.403015	0.609289	-0.20627	7	1.39E+08	F	II	HIPK2	Body	opensea	Body-opens		rs1133252	42
cg0744629	0.219313	0.426266	11.24223	5.19E-06	0.000651	4.798485	0.316609	0.535922	-0.21931	9	1.33E+08	F	II		IGR	opensea	IGR-opense		rs5381093	36
cg1852069	0.250253	0.496993	11.24212	5.19E-06	0.000651	4.798414	0.371866	0.622119	-0.25025	17	4631822	R	II		IGR	shelf	IGR-shelf	chr17:4634	rs5272813	2
cg2150863	0.225282	0.449659	11.24138	5.19E-06	0.000651	4.797936	0.337018	0.5623	-0.22528	17	54667590	R	II		IGR	shelf	IGR-shelf	chr17:5467	rs5690778	7
cg1901021	0.20037	0.495847	11.23984	5.2E-06	0.000651	4.796936	0.395662	0.596033	-0.20037	2	1.06E+08	F	II	NCK2	Body	opensea	Body-opens		rs5342305	0;1;11
cg0650921	0.243954	0.552909	11.2397	5.2E-06	0.000651	4.796847	0.430932	0.674886	-0.24395	18	42855350	F	II	SLC14A2	5'UTR	opensea	5'UTR-oper		rs1458994	0;34;49
cg1566192	0.266959	0.576673	11.23963	5.2E-06	0.000651	4.7968	0.443194	0.710153	-0.26696	21	15919643	R	II	SAMSN1	TSS1500	opensea	TSS1500-op			
cg1199649	0.248713	0.615259	11.23898	5.2E-06	0.000651	4.796385	0.490903	0.739616	-0.24871	3	37026597	F	II		IGR	opensea	IGR-opense		rs1879520	1;3
cg1821882	0.206489	0.636251	11.23886	5.2E-06	0.000651	4.796304	0.533007	0.739496	-0.20649	22	38729789	F	II	LOC40092	5'UTR	opensea	5'UTR-oper		rs5598088	0
cg2027564	0.266456	0.429985	11.23879	5.2E-06	0.000651	4.79626	0.296757	0.563213	-0.26646	10	10911683	R	II		IGR	opensea	IGR-opense		rs1382590	2
cg0458489	0.241451	0.654169	11.23591	5.21E-06	0.000652	4.794393	0.533444	0.774895	-0.24145	2	1.03E+08	F	II	SLC9A2	Body	opensea	Body-opens			
cg0885563	0.210469	0.575828	11.23398	5.22E-06	0.000652	4.793146	0.470594	0.681063	-0.21047	3	68978377	R	II	FAM19A4	5'UTR	shelf	5'UTR-shelf	chr3:6898	rs1899606	42;13;2
cg2625204	0.21654	0.642468	11.23216	5.22E-06	0.000653	4.791964	0.534198	0.750738	-0.21654	11	34256703	F	II	ABTB2	Body	opensea	Body-opens		rs5645527	0
cg1600822	0.201704	0.456239	11.23194	5.22E-06	0.000653	4.791819	0.355387	0.557091	-0.2017	20	39122112	R	II		IGR	opensea	IGR-opense		rs5453137	42;9;1
cg1447842	0.209566	0.648177	11.2315	5.23E-06	0.000653	4.791534	0.543394	0.75296	-0.20957	2	2.34E+08	F	II	NGEF	1stExon	opensea	1stExon-op		rs5781575	12;38
cg2615549	0.216993	0.469487	11.23079	5.23E-06	0.000653	4.791074	0.360991	0.577983	-0.21699	1	1.1E+08	F	II	GPR61	TSS200	opensea	TSS200-ope		rs1702324	14
cg1388508	0.210056	0.515044	11.22756	5.24E-06	0.000653	4.788981	0.410016	0.620071	-0.21006	22	42823184	F	II	NFAM1	Body	opensea	Body-opens			
cg2278362	0.225457	0.625546	11.22726	5.24E-06	0.000653	4.788785	0.512818	0.738275	-0.22546	11	64914347	F	II		IGR	opensea	IGR-opense		rs5386908	1;51
cg1713117	0.207347	0.454923	11.22703	5.24E-06	0.000653	4.788639	0.351249	0.558596	-0.20735	16	78802689	F	II	WWOX	Body	opensea	Body-opens		rs5765530	0;6;10;39
cg1074089	0.207953	0.637147	11.22547	5.25E-06	0.000653	4.787625	0.533171	0.741124	-0.20795	20	43838292	R	II	SEMG1	3'UTR	opensea	3'UTR-oper		rs6032056	1
cg0667677	0.229118	0.591112	11.22543	5.25E-06	0.000653	4.787596	0.476552	0.705671	-0.22912	5	1.48E+08	R	II		IGR	shelf	IGR-shelf	chr5:1482	rs5335151	34;20
cg1372453	0.225631	0.565467	11.22464	5.25E-06	0.000653	4.787087	0.452651	0.678282	-0.22563	15	57737565	R	II	CGNL1	Body	opensea	Body-opens		rs1420332	1
cg2282782	0.217489	0.511055	11.22429	5.25E-06	0.000653	4.786861	0.40231	0.619799	-0.21749	2	1.98E+08	F	II	HSPE1	Body	shelf	Body-shelf	chr2:1983	rs5560777	0;15;18;25
cg1560418	0.202996	0.57497	11.22366	5.25E-06	0.000653	4.786447	0.473472	0.676468	-0.203	2	62687964	R	II		IGR	shelf	IGR-shelf	chr2:6268	rs5680639	33
cg0526678	0.20059	0.693894	11.22363	5.25E-06	0.000653	4.786432	0.593599	0.794189	-0.20059	10	49742271	F	II	ARHGAP22	Body	opensea	Body-opens		rs1402868	15
cg0991180	0.223372	0.42089	11.22346	5.25E-06	0.000653	4.786321	0.309204	0.532576	-0.22337	9	1.14E+08	F	II	LPAR1	Body	opensea	Body-opens		rs1380223	0;1;7;21;47;50
cg1492172	0.213197	0.560722	11.22158	5.26E-06	0.000653	4.785103	0.454124	0.667321	-0.2132	3	1.6E+08	F	II	IQCJ-SCHIP	Body	opensea	Body-opens		rs1506652	32;36;38
cg2741756	0.218994	0.372831	11.22103	5.26E-06	0.000654	4.784742	0.263334	0.482328	-0.21899	19	51711261	F	II	MIR8074	TSS1500	opensea	TSS1500-op		rs8014857	1;42
cg0842307	0.213971	0.645525	11.21894	5.27E-06	0.000654	4.783389	0.53854	0.752511	-0.21397	4	1.65E+08	R	II	MARCH1	Body	opensea	Body-opens		rs5777040	51;23;8
cg0420541	0.204033	0.663586	11.21881	5.27E-06	0.000654	4.783304	0.561569	0.765602	-0.20403	17	54785335	R	II		IGR	opensea	IGR-opense		rs227711;r	45;33;27;26
cg2408217	0.237602	0.542298	11.21816	5.27E-06	0.000654	4.782882	0.423497	0.661099	-0.2376	3	1.87E+08	F	II	SNORA63	TSS1500	shore	TSS1500-sh	chr3:1865	rs5352836	1;2
cg0261469	0.201464	0.461203	11.217	5.27E-06	0.000654	4.782126	0.360471	0.561935	-0.20146	4	10262707	R	II		IGR	opensea	IGR-opense		rs1883481	47;4
cg2029844	0.21749	0.413137	11.21438	5.28E-06	0.000655	4.780425	0.304392	0.521882	-0.21749	4	47536255	R	II	ATP10D	Body	opensea	Body-opens		rs5633395	1
cg2301470	0.242739	0.487715	11.21366	5.29E-06	0.000655	4.77996	0.366346	0.609085	-0.24274	5	1.69E+08	F	II		IGR	opensea	IGR-opense			
cg0411210	0.271798	0.516289	11.21317	5.29E-06	0.000655	4.779642	0.38039	0.652188	-0.2718	2	1.44E+08	R	II	KYNU	Body	opensea	Body-opens			
cg2656803	0.247778	0.599671	11.2131	5.29E-06	0.000655	4.779597	0.475782	0.723561	-0.24778	10	91313380	F	II		IGR	opensea	IGR-opense		rs1429255	1;39
cg0978058	0.239646	0.577925	11.21124	5.29E-06	0.000655	4.778386	0.458102	0.697748	-0.23965	3	55540579	R	II		IGR	opensea	IGR-opense		rs5714728	21
cg0706929	0.227744	0.560583	11.20931	5.3E-06	0.000656	4.777135	0.446711	0.674454	-0.22774	8	80735125	R	II	LOC10192	Body	opensea	Body-opens		rs5589720	40
cg2745554	0.200657	0.55362	11.20902	5.3E-06	0.000656	4.776943	0.453292	0.653949	-0.20066	2	18866125	R	II		IGR	opensea	IGR-opense		rs1403968	50;38;1
cg1493693	0.233509	0.581181	11.20872	5.3E-06	0.000656	4.776751	0.464427	0.697936	-0.23351	8	1.22E+08	R	II		IGR	opensea	IGR-opense		rs1832738	46;7
cg2540564	0.241116	0.579167	11.20827	5.3E-06	0.000656	4.776459	0.458609	0.699725	-0.24112	2	2.18E+08	F	II	DIRC3	Body	opensea	Body-opens		rs5431954	11;22
cg1115030	0.261864	0.416871	11.20523	5.31E-06	0.000656	4.774484	0.285939	0.547803	-0.26186	1	1.98E+08	F	II		IGR	opensea	IGR-opense		rs5311340	0
cg1977192	0.227828	0.542064	11.20306	5.32E-06	0.000657	4.773075	0.42815	0.655978	-0.22783	2	1.74E+08	F	II	RAPGEF4	Body	opensea	Body-opens		rs5464038	14;36
cg2003832	0.221088	0.523573	11.20166	5.33E-06	0.000657	4.77216	0.413029	0.634117	-0.22109	1	2.01E+08	F	II	CAMSAP2	Body	opensea	Body-opens		rs5681402	14;38;46
cg0247919	0.201075	0.591295	11.2013	5.33E-06	0.000657	4.771931	0.490757	0.691833	-0.20108	11	84997870	F	II	DLG2	Body	opensea	Body-opens		rs7880970	1
cg0465098	0.228395	0.559247	11.19854	5.34E-06	0.000657	4.770136	0.44505	0.673445	-0.2284	6	1.59E+08	F	II		IGR	opensea	IGR-opense			
cg2364103	0.220542	0.460021	11.19617	5.35E-06	0.000657	4.768594	0.34975	0.570292	-0.22054	1	1.75E+08	R	II	TNR	5'UTR	opensea	5'UTR-oper		rs5510252	5
cg2214428	0.246295	0.66769	11.19502	5.35E-06	0.000658	4.767846	0.544543	0.790838	-0.2463	5	1.69E+08	R	II		IGR	opensea	IGR-opense		rs1904611	38;32;6;1
cg1626185	0.2258	0.504736	11.19321	5.36E-06	0.000658	4.766666	0.391836	0.617636	-0.2258	18	57634307	F	II		IGR	shelf	IGR-shelf	chr18:576	rs7654253	0;3;41
cg0013344	0.2048	0.45716	11.19148	5.36E-06	0.000658	4.765541	0.35476	0.55956	-0.2048	7	46824233	R	II		IGR	opensea	IGR-opense			
cg2069894	0.218717	0.601731	11.18896	5.37E-06	0.000659	4.763898	0.492373	0.71109	-0.21872	1	51910707	R	II	EPS15	Body	opensea	Body-opens		rs1158090	47;43;40
cg0393736	0.200775	0.491715	11.18753	5.38E-06	0.000659	4.762969	0.391327	0.592102	-0.20078	1	64330272	F	II	ROR1	Body	opensea	Body-opens			
cg2544408	0.243598	0.510701	11.18462	5.39E-06	0.000659	4.761078	0.388902	0.632499	-0.2436	20	11852131	R	II	LINC00687	TSS1500	opensea	TSS1500-op		rs5289717	51;45;44;29
cg1631377	0.227837	0.50383	11.18196	5.39E-06	0.000659	4.759345	0.389911	0.617749	-0.22784	8	1.19E+08	R	II		IGR	opensea	IGR-opense		rs5468156	1;25;45
cg1410938	0.213199	0.527246	11.18119	5.4E-06	0.000659	4.758839	0.420647	0.633846	-0.2132	4	25940163	F	II		IGR	opensea	IGR-opense		rs5781876	0;2;46
cg1258355	0.223166	0.462876	11.18054	5.4E-06	0.000659	4.758417	0.351293	0.574459	-0.22317	6	33242487	R	II	RPS18	Body	shelf	Body-shelf	chr6:3324	rs3746162	28;12
cg0113421	0.227885	0.586653	11.17854	5.41E-06	0.00066	4.757115	0.471211	0.700596	-0.22789	18	6363442	F	II	L3MBTL4	5'UTR	opensea	5'UTR-oper		rs1143902	35
cg0697503	0.202477	0.443333	11.17556	5.42E-06	0.00066	4.755174	0.342094	0.544571	-0.20248	5	50230491	R	II		IGR	opensea	IGR-opense		rs5310363	39;9
cg1060582	0.238975	0.574159	11.17153	5.43E-06	0.000661	4.752547	0.454672	0.693646	-0.23897	9	72087222	F	II	APBA1	Body	opensea	Body-opens		rs4744901	14;16
cg1503058	0.227816	0.476285	11.17094	5.43E-06	0.000661	4.752159	0.362377	0.590193	-0.22782	13	27927676	R	II		IGR	opensea	IGR-opense		rs5498135	37;24
cg0139778	0.310646	0.505352	11.17078	5.43E-06	0.000661	4.752054	0.350029	0.660675	-0.31065	10	3894712	R	II		IGR	opensea	IGR-opense		rs5388411	1
cg1080789	0.228688	0.378085	11.17031	5.44E-06	0.000661	4.75175	0.26374	0.492429	-0.22869	12	1.25E+08	R	II		IGR	opensea	IGR-opense		rs5274990	23
cg1700267	0.215645	0.47168	11.16883	5.44E-06	0.000661	4.750781	0.363857	0.579503	-0.21565	7	1.55E+08	F	II	RBM33	Body	opensea	Body-opens			
cg2525591	0.219559	0.732083	11.16659	5.45E-06	0.000661	4.749321	0.622304	0.841863	-0.21956	2	29685363	F	II	ALK	Body	opensea	Body-opens		rs5761336	42;43
cg0625673	0.242255	0.516613	11.16135	5.47E-06	0.000663	4.745901	0.395486	0.637741	-0.24225	12	8815894	R	II	MFAP5	TSS1500	opensea	TSS1500-op			
cg2525943	0.244708	0.532273	11.15853	5.48E-06	0.000663	4.744065	0.409919	0.654627	-0.24471	11	23100280	R	II		IGR	opensea	IGR-opense		rs1168649	18;8;2
cg2421389	0.203133	0.654715	11.1578	5.48E-06	0.000663	4.743584	0.553148	0.756281	-0.20313	20	1924201	F	II		IGR	shelf	IGR-shelf	chr20:192	rs5624914	1;8
cg0148241	0.258591	0.521363	11.15717	5.48E-06	0.000663	4.743179	0.392068	0.650658	-0.25859	1	2.26E+08	F	II		IGR	opensea	IGR-opense		rs5579066	7;27
cg0248820	0.267898	0.472198	11.1569	5.48E-06	0.000663	4.743	0.338249	0.606147	-0.2679	7	1.32E+08	F	II	PLXNA4	Body	opensea	Body-opens		rs7530553	0;19;38
cg2091982	0.211296	0.551922	11.15661	5.48E-06	0.000663	4.74281	0.446274	0.657569	-0.2113	22	47490983	R	II	TBC1D22A	Body	opensea	Body-opens		rs1141926	35;31;20;7
cg1478625	0.228202	0.548327	11.15461	5.49E-06	0.000664	4.741503	0.434226	0.662429	-0.2282	8	20038608	F	II	SLC18A1	5'UTR	opensea	5'UTR-oper		rs5612287	0;6;14;18;41
cg1484724	0.219639	0.585148	11.14969	5.51E-06	0.000665	4.738288	0.475328	0.694967	-0.21964	3	40158292	R	II	MYRIP	5'UTR	opensea	5'UTR-oper			
cg2600268	0.205618	0.327203	11.14942	5.51E-06	0.000665	4.738115	0.224394	0.430013	-0.20562	22	26019477	R	II	ADRBK2	Body	opensea	Body-opens		rs5679184	2
cg2634187	0.205249	0.624278	11.14423	5.53E-06	0.000666	4.734721	0.521653	0.726902	-0.20525	10	74714969	F	II	PLA2G12B	TSS1500	opensea	TSS1500-op			
cg0663094	0.239852	0.523169	11.14387	5.53E-06	0.000666	4.734485	0.403243	0.643096	-0.23985	5	93330995	R	II	FAM172A	Body	opensea	Body-opens		rs5391995	26;1
cg2432979	0.204454	0.536284	11.14307	5.53E-06	0.000666	4.733962	0.434057	0.638511	-0.20445	22	27592445	F	II		IGR	opensea	IGR-opense		rs742060;r	0;43
cg1567386	0.204263	0.444533	11.14258	5.53E-06	0.000666	4.73364	0.342402	0.546665	-0.20426	11	87590493	R	II		IGR	opensea	IGR-opense		rs5689870	30;1
cg0817608	0.206575	0.595469	11.14111	5.54E-06	0.000666	4.732683	0.492182	0.698757	-0.20657	2	1E+08	R	II	REV1	5'UTR	opensea	5'UTR-oper		rs3723457	44;27;2
cg1565788	0.212123	0.600258	11.13871	5.55E-06	0.000667	4.731114	0.494196	0.706319	-0.21212	2	1.14E+08	R	II	IL1F9	5'UTR	opensea	5'UTR-oper		rs1893364	49;25;11
cg1635673	0.217365	0.544473	11.13641	5.56E-06	0.000667	4.729604	0.43579	0.653155	-0.21736	6	25729805	R	II		IGR	shelf	IGR-shelf	chr6:2573		
cg1340007	0.214766	0.55853	11.13582	5.56E-06	0.000667	4.729221	0.451147	0.665913	-0.21477	1	1.62E+08	R	II	NOS1AP	Body	opensea	Body-opens		rs6692600	13;5
cg1576875	0.222395	0.678968	11.1358	5.56E-06	0.000667	4.72921	0.56777	0.790165	-0.22239	2	3626969	F	II	RPS7	Body	shelf	Body-shelf	chr2:3622	rs1163652	33
cg1361317	0.203586	0.520158	11.13234	5.57E-06	0.000668	4.726945	0.418365	0.621952	-0.20359	2	2.43E+08	R	II	D2HGDH	Body	opensea	Body-opens		rs5506462	33
cg2291434	0.20196	0.475128	11.12972	5.58E-06	0.000668	4.725226	0.374148	0.576108	-0.20196	10	91187065	R	II		IGR	opensea	IGR-opense		rs1907593	2
cg1997837	0.214018	0.50596	11.12882	5.58E-06	0.000668	4.724637	0.398951	0.612969	-0.21402	6	31542671	R	II	TNF	TSS1500	opensea	TSS1500-op			
cg1261996	0.221314	0.656188	11.12563	5.6E-06	0.000669	4.722552	0.545531	0.766845	-0.22131	6	16677204	R	II	ATXN1	5'UTR	opensea	5'UTR-oper		rs1432200	28
cg0503126	0.207167	0.513932	11.12511	5.6E-06	0.000669	4.722212	0.410348	0.617515	-0.20717	18	33959405	F	II	FHOD3	Body	opensea	Body-opens		rs1898046	36
cg1448826	0.220171	0.545428	11.1233	5.6E-06	0.000669	4.721024	0.435342	0.655513	-0.22017	7	1.07E+08	F	II		IGR	opensea	IGR-opense		rs1715427	30
cg1476447	0.237453	0.568754	11.12221	5.61E-06	0.000669	4.720307	0.450028	0.687481	-0.23745	5	1.43E+08	F	II		IGR	opensea	IGR-opense		rs1469596	5;31
cg0879753	0.217852	0.478768	11.11985	5.62E-06	0.000669	4.718764	0.369842	0.587694	-0.21785	4	98815580	R	II	STPG2	Body	opensea	Body-opens		rs5536621	7
cg1383702	0.218731	0.574202	11.11905	5.62E-06	0.000669	4.71824	0.464837	0.683567	-0.21873	5	1.49E+08	R	II		IGR	opensea	IGR-opense		rs5586504	51;36;1
cg0581017	0.205861	0.418982	11.11872	5.62E-06	0.000669	4.718021	0.316051	0.521912	-0.20586	7	1.34E+08	R	II		IGR	opensea	IGR-opense		rs1489571	26;2
cg1344054	0.230825	0.505863	11.11621	5.63E-06	0.00067	4.71638	0.390451	0.621276	-0.23082	14	52792228	R	II	PTGER2	Body	opensea	Body-opens		rs5482129	13;2
cg2581616	0.208521	0.467591	11.11526	5.63E-06	0.00067	4.715758	0.363331	0.571852	-0.20852	1	93053203	F	II	EVI5	Body	opensea	Body-opens		rs1821519	0
cg1134818	0.217211	0.577304	11.11378	5.64E-06	0.000671	4.714786	0.468699	0.685909	-0.21721	2	2.23E+08	F	II		IGR	opensea	IGR-opense		rs5350210	51
cg1156043	0.213722	0.599129	11.11285	5.64E-06	0.000671	4.714177	0.492269	0.70599	-0.21372	6	3416406	F	II	SLC22A23	Body	opensea	Body-opens		rs5363056	1;25
cg0445125	0.209812	0.598834	11.11281	5.64E-06	0.000671	4.714149	0.493928	0.70374	-0.20981	11	35358924	F	II	SLC1A2	Body	opensea	Body-opens		rs1161679	1;21;33;34
cg1270539	0.226634	0.486769	11.11171	5.65E-06	0.000671	4.713428	0.373452	0.600086	-0.22663	6	12586532	F	II		IGR	opensea	IGR-opense		rs1897145	49
cg1260084	0.204308	0.582788	11.10961	5.65E-06	0.000671	4.712052	0.480634	0.684942	-0.20431	6	30179881	R	II	TRIM26	5'UTR	shore	5'UTR-shor	chr6:3018	rs5570285	48;2
cg2091834	0.203104	0.551311	11.10726	5.66E-06	0.000671	4.710505	0.449759	0.652863	-0.2031	7	51101539	F	II	COBL	Body	opensea	Body-opens		rs5313094	0;25;38
cg0871296	0.205244	0.503241	11.10725	5.66E-06	0.000671	4.710503	0.400619	0.605863	-0.20524	15	84283688	R	II	SH3GL3	Body	opensea	Body-opens		rs1451892	1
cg0250039	0.236448	0.520061	11.10658	5.66E-06	0.000671	4.710063	0.401837	0.638285	-0.23645	6	1.39E+08	F	II	PBOV1	1stExon	opensea	1stExon-op		rs7377468	0;28;46
cg1063306	0.233579	0.585163	11.10611	5.67E-06	0.000671	4.709754	0.468374	0.701953	-0.23358	3	4047981	F	II		IGR	opensea	IGR-opense		rs1484908	9
cg2617187	0.220769	0.62685	11.10331	5.68E-06	0.000672	4.707916	0.516465	0.737235	-0.22077	5	1.21E+08	F	II		IGR	opensea	IGR-opense		rs1382860	1;4;21
cg0648837	0.207212	0.599141	11.10279	5.68E-06	0.000672	4.707574	0.495535	0.702747	-0.20721	1	24392896	F	II	MYOM3	Body	opensea	Body-opens			
cg0525157	0.201818	0.530639	11.09845	5.69E-06	0.000673	4.704724	0.42973	0.631548	-0.20182	13	1.12E+08	F	II		IGR	opensea	IGR-opense		rs5316275	0;6;29
cg0981061	0.20863	0.543721	11.0972	5.7E-06	0.000673	4.703905	0.439406	0.648037	-0.20863	8	92919702	R	II		IGR	opensea	IGR-opense		rs1392579	1
cg0252498	0.250406	0.665167	11.09069	5.72E-06	0.000674	4.699631	0.539964	0.79037	-0.25041	3	1.88E+08	F	I	LPP	5'UTR	opensea	5'UTR-oper		rs5550028	0;20;37
cg0615338	0.229998	0.57475	11.08888	5.73E-06	0.000675	4.69844	0.459751	0.689749	-0.23	15	70644758	F	II		IGR	opensea	IGR-opense		rs5452017	51
cg2753727	0.254334	0.398861	11.08883	5.73E-06	0.000675	4.698408	0.271693	0.526028	-0.25433	3	1.48E+08	R	II	AGTR1	5'UTR	opensea	5'UTR-oper		rs5446051	50;35
cg0200939	0.212905	0.4327	11.08876	5.73E-06	0.000675	4.69836	0.326247	0.539153	-0.21291	16	20702744	R	II	ACSM1	TSS200	opensea	TSS200-ope		rs5632856	44;39;2
cg1868018	0.235597	0.63263	11.08586	5.74E-06	0.000675	4.696456	0.514831	0.750429	-0.2356	14	35629311	R	II	KIAA0391	Body	opensea	Body-opens		rs1907458	10

cg0076599	0.224206	0.564449	11.08583	5.74E-06	0.000675	4.696432	0.452347	0.676552	-0.22421	1	2.1E+08	R	II		IGR	shelf	IGR-shelf	chr1:2104(	rs5715619	23;2
cg2516858	0.220608	0.629916	11.08539	5.74E-06	0.000675	4.696141	0.519613	0.74022	-0.22061	10	13276816	F	II	UCMA	TSS1500	opensea	TSS1500-op		rs5291127	39
cg0119027	0.222451	0.594702	11.08395	5.75E-06	0.000675	4.6952	0.483476	0.705928	-0.22245	3	24284272	F	II	THRB	5'UTR	opensea	5'UTR-open		rs5455478	27
cg0468063	0.214409	0.467058	11.0823	5.75E-06	0.000675	4.694112	0.359854	0.574263	-0.21441	3	1.81E+08	R	II	SOX2-OT	Body	opensea	Body-opens		rs3767475	1
cg0106202	0.201677	0.44701	11.08006	5.76E-06	0.000676	4.692636	0.346171	0.547848	-0.20168	3	49065979	F	II	IMPDH2	Body	shore	Body-shore	chr3:4906(	rs1841723	0;12;19;50
cg0726963	0.221273	0.612592	11.07982	5.76E-06	0.000676	4.692484	0.501956	0.723229	-0.22127	7	18561537	F	II	HDAC9	Body	opensea	Body-opens		rs1853911	39
cg2217105	0.217242	0.600778	11.07893	5.77E-06	0.000676	4.691893	0.492158	0.709399	-0.21724	1	62905816	R	II	USP1	Body	shelf	Body-shelf	chr1:6290:	rs1841788	31;29
cg0842205	0.200369	0.6318	11.07854	5.77E-06	0.000676	4.691637	0.531616	0.731985	-0.20037	3	1.01E+08	R	II		IGR	opensea	IGR-opense		rs5682335	43;15;5
cg1586213	0.225613	0.463171	11.07522	5.78E-06	0.000677	4.689455	0.350364	0.575977	-0.22561	13	42980762	F	II		IGR	opensea	IGR-opense			
cg1729538	0.201636	0.594252	11.07443	5.78E-06	0.000677	4.688937	0.493434	0.69507	-0.20164	6	1.11E+08	F	II	DDO	Body	shelf	Body-shelf	chr6:1107:		
cg0392903	0.241155	0.58485	11.07432	5.78E-06	0.000677	4.688863	0.464273	0.705428	-0.24115	6	84668020	R	II	CYB5R4	Body	opensea	Body-opens		rs6175692	20;17
cg0118875	0.249095	0.527807	11.07419	5.79E-06	0.000677	4.688774	0.403259	0.652354	-0.2491	20	47897068	R	II	C20orf199	Body	shore	Body-shore	chr20:478(		
cg0738987	0.218414	0.616283	11.07416	5.79E-06	0.000677	4.688755	0.507076	0.725491	-0.21841	1	1.11E+08	R	II	SLC16A4	Body	opensea	Body-opens		rs1440269	29;2
cg1350261	0.204727	0.51374	11.07234	5.79E-06	0.000677	4.687561	0.411377	0.616104	-0.20473	1	1.81E+08	F	II	XPR1	Body	opensea	Body-opens		rs5453750	9;24
cg1998339	0.292268	0.468728	11.07224	5.79E-06	0.000677	4.687492	0.322594	0.614862	-0.29227	13	29115045	R	II		IGR	opensea	IGR-opense		rs5451385	38;2
cg1666395	0.222025	0.572126	11.07196	5.79E-06	0.000677	4.687311	0.461113	0.683139	-0.22203	12	24352279	R	II	SOX5	5'UTR	opensea	5'UTR-open		rs5698956	1
cg1871601	0.225562	0.618441	11.07067	5.8E-06	0.000677	4.686457	0.50566	0.731223	-0.22556	1	47125994	R	II	ATPAF1	5'UTR	opensea	5'UTR-open		rs1180131	30
cg1720931	0.204917	0.511908	11.06981	5.8E-06	0.000677	4.685891	0.40945	0.614367	-0.20492	20	43930688	F	II	MATN4	Body	shelf	Body-shelf	chr20:439:	rs5470182	14;22;39;41;48
cg2114400	0.21168	0.548104	11.06855	5.81E-06	0.000678	4.685066	0.442264	0.653945	-0.21168	1	2.08E+08	F	II	PLXNA2	Body	opensea	Body-opens		rs5396226	23
cg1350104	0.236287	0.46351	11.06731	5.81E-06	0.000678	4.684247	0.345366	0.581654	-0.23629	6	75308261	F	II	LOC10192(	Body	opensea	Body-opens		rs1508934	0
cg0783036	0.258233	0.506746	11.0671	5.81E-06	0.000678	4.684107	0.377629	0.635862	-0.25823	3	1.54E+08	R	II		IGR	opensea	IGR-opense		rs5384325	21;2
cg1465487	0.20304	0.567433	11.06371	5.83E-06	0.000679	4.681873	0.465913	0.668953	-0.20304	2	2.3E+08	R	II	DNER	Body	opensea	Body-opens		rs1907058	34;18;8
cg0586595	0.227499	0.462308	11.063	5.83E-06	0.000679	4.681408	0.348558	0.576057	-0.2275	10	63224906	R	II	TMEM26-A:	Body	opensea	Body-opens		rs1151823	48;1
cg1112934	0.217905	0.545062	11.06294	5.83E-06	0.000679	4.681367	0.43611	0.654015	-0.21791	11	19870761	F	II	NAV2	Body	opensea	Body-opens			
cg2070750	0.238924	0.602104	11.06056	5.84E-06	0.000679	4.679801	0.482642	0.721566	-0.23892	2	38663934	F	II		IGR	opensea	IGR-opense		rs925256	27
cg2738139	0.201148	0.583191	11.06023	5.84E-06	0.000679	4.679579	0.482617	0.683765	-0.20115	15	1.02E+08	R	II	PCSK6	Body	opensea	Body-opens		rs5666729	29
cg2361388	0.234828	0.530978	11.05895	5.84E-06	0.000679	4.678739	0.413564	0.648392	-0.23483	10	1.23E+08	F	II		IGR	shelf	IGR-shelf	chr10:123:	rs1460271	0;35;36
cg1255076	0.201588	0.640241	11.05673	5.85E-06	0.000679	4.677275	0.539447	0.741034	-0.20159	1	87213417	R	II	SH3GLB1	3'UTR	opensea	3'UTR-open		rs1495226	42;7
cg0225174	0.217691	0.54191	11.05614	5.85E-06	0.000679	4.676887	0.433065	0.650756	-0.21769	2	1.88E+08	R	II	CALCRL	5'UTR	opensea	5'UTR-open		rs5665288	50;48;2
cg1503198	0.214773	0.703441	11.05523	5.86E-06	0.000679	4.676285	0.596054	0.810827	-0.21477	6	1.53E+08	R	II	RGS17	5'UTR	opensea	5'UTR-open		rs6925834	40;1
cg0453100	0.227184	0.627152	11.05518	5.86E-06	0.000679	4.676255	0.51356	0.740743	-0.22718	2	1.28E+08	F	II		IGR	opensea	IGR-opense			
cg1003973	0.255539	0.560838	11.05423	5.86E-06	0.00068	4.675626	0.433068	0.688607	-0.25554	10	95139986	R	II	MYOF	Body	opensea	Body-opens			
cg2319617	0.217544	0.527612	11.05059	5.88E-06	0.00068	4.673226	0.41884	0.636384	-0.21754	6	29446963	F	II		IGR	opensea	IGR-opense		rs3685810	1
cg2602181	0.212768	0.556187	11.05026	5.88E-06	0.00068	4.67301	0.449803	0.662571	-0.21277	13	23895705	R	II	SGCG	Body	opensea	Body-opens		rs7344110	7;2
cg0432634	0.201748	0.576768	11.04959	5.88E-06	0.00068	4.672564	0.475894	0.677642	-0.20175	10	1.25E+08	F	II		IGR	opensea	IGR-opense		rs5415409	8;34
cg1441313	0.205472	0.415207	11.0484	5.88E-06	0.000681	4.671779	0.312471	0.517943	-0.20547	18	47860601	F	II		IGR	opensea	IGR-opense		rs5632125	10;18;19;33;41
cg0265823	0.268555	0.449705	11.04828	5.88E-06	0.000681	4.671697	0.315428	0.583983	-0.26856	2	2.25E+08	R	II	CUL3	TSS1500	opensea	TSS1500-op			
cg0201227	0.212223	0.578799	11.04733	5.89E-06	0.000681	4.671071	0.472688	0.684911	-0.21222	17	37974860	R	II	IKZF3	Body	opensea	Body-opens		rs1915667	49;22
cg1355417	0.248967	0.609152	11.04674	5.89E-06	0.000681	4.670682	0.484669	0.733636	-0.24897	6	79780164	R	II	PHIP	Body	opensea	Body-opens		rs5509525	29;4
cg1040873	0.219787	0.636444	11.04572	5.89E-06	0.000681	4.67001	0.52655	0.746337	-0.21979	6	1.01E+08	R	II		IGR	opensea	IGR-opense		rs5458902	36;5;2;1
cg0282524	0.223121	0.441995	11.04547	5.89E-06	0.000681	4.669844	0.330435	0.553556	-0.22312	2	1.97E+08	R	II		IGR	opensea	IGR-opense		rs5754973	22;1
cg0238931	0.264668	0.502428	11.0453	5.9E-06	0.000681	4.669734	0.370094	0.634762	-0.26467	12	76577488	F	II		IGR	opensea	IGR-opense		rs3697068	1
cg1099033	0.205395	0.535589	11.04149	5.91E-06	0.000681	4.667219	0.432892	0.638287	-0.2054	2	42941352	R	II	MTA3	Body	opensea	Body-opens		rs5665201	2
cg1751157	0.202375	0.613843	11.03848	5.92E-06	0.000682	4.665229	0.512655	0.71503	-0.20238	2	1.22E+08	F	II	CLASP1	Body	opensea	Body-opens		rs1857801	6;38
cg0450792	0.258589	0.483531	11.03599	5.93E-06	0.000682	4.663584	0.354237	0.612825	-0.25859	2	2.11E+08	R	II	UNC80	Body	opensea	Body-opens		rs5480498	20;2
cg1346559	0.231153	0.571819	11.03537	5.93E-06	0.000682	4.663176	0.456243	0.687396	-0.23115	8	38401333	F	II		IGR	opensea	IGR-opense			
cg2260532	0.218938	0.56824	11.03398	5.94E-06	0.000683	4.662255	0.458771	0.677709	-0.21894	3	1.31E+08	R	II		IGR	opensea	IGR-opense		rs5620064	46;18;2
cg1008503	0.219551	0.512104	11.03357	5.94E-06	0.000683	4.661986	0.402328	0.621879	-0.21955	14	99504935	F	II		IGR	opensea	IGR-opense		rs5745578	1;3;6;24;28
cg2490257	0.219917	0.455112	11.03221	5.95E-06	0.000683	4.661089	0.345154	0.565071	-0.21992	16	66984552	R	II		IGR	shore	IGR-shore	chr16:669(	rs5621028	29;10;2
cg1754029	0.202871	0.506113	11.03168	5.95E-06	0.000683	4.660739	0.404678	0.607549	-0.20287	14	20835697	F	II	TEP1	3'UTR	opensea	3'UTR-open			
cg1129831	0.210466	0.485133	11.03084	5.95E-06	0.000683	4.660181	0.3799	0.590366	-0.21047	10	1.24E+08	R	II	TACC2	5'UTR	opensea	5'UTR-open		rs5845758	31
cg2155174	0.22104	0.637525	11.03002	5.95E-06	0.000683	4.65964	0.527005	0.748044	-0.22104	18	46548378	F	II		IGR	opensea	IGR-opense		rs1819298	2;32
cg1589791	0.22019	0.537131	11.02873	5.96E-06	0.000683	4.658785	0.427036	0.647226	-0.22019	8	6198768	F	II		IGR	opensea	IGR-opense		rs5600836	13;47
cg1077992	0.244745	0.518715	11.02727	5.97E-06	0.000683	4.657824	0.396343	0.641088	-0.24474	3	1.67E+08	R	II	WDR49	Body	opensea	Body-opens			
cg0142056	0.214882	0.448166	11.02692	5.97E-06	0.000683	4.657591	0.340726	0.555607	-0.21488	2	1.79E+08	R	II	OSBPL6	5'UTR	opensea	5'UTR-open		rs5276858	47;25
cg1155901	0.228559	0.693804	11.02689	5.97E-06	0.000683	4.657572	0.579525	0.808084	-0.22856	18	61576332	F	II		IGR	opensea	IGR-opense		rs1707207	22;45
cg0590584	0.204728	0.412181	11.02598	5.97E-06	0.000684	4.656972	0.309817	0.514545	-0.20473	7	1.36E+08	F	II		IGR	opensea	IGR-opense		rs1864184	1;6
cg0468498	0.23242	0.506892	11.02421	5.98E-06	0.000684	4.655796	0.390682	0.623102	-0.23242	2	1.71E+08	F	II	CCDC173	Body	opensea	Body-opens			
cg2043608	0.212958	0.599781	11.022	5.99E-06	0.000684	4.654334	0.493302	0.70626	-0.21296	10	71615835	F	II	COL13A1	Body	opensea	Body-opens		rs5501884	1;22
cg2434215	0.248332	0.538303	11.02087	5.99E-06	0.000684	4.653588	0.414137	0.662469	-0.24833	8	1.25E+08	F	II	FER1L6	5'UTR	opensea	5'UTR-open		rs1446023	0;3;18
cg0690516	0.207232	0.515317	11.02049	5.99E-06	0.000684	4.653335	0.411701	0.618933	-0.20723	16	27096840	R	II		IGR	opensea	IGR-opense		rs8050140	27;17;4;2
cg1692971	0.223589	0.668212	11.01946	6E-06	0.000685	4.652659	0.556418	0.780006	-0.22359	3	73024037	R	II	GXYLT2	Body	opensea	Body-opens		rs5573931	2
cg2517992	0.2374	0.698106	11.01929	6E-06	0.000685	4.652544	0.579406	0.816806	-0.2374	8	16959530	R	II	MICU3	Body	opensea	Body-opens		rs5549638	51;9;5
cg1601997	0.255035	0.553233	11.01839	6E-06	0.000685	4.651949	0.425716	0.68075	-0.25503	5	1.43E+08	R	II		IGR	opensea	IGR-opense		rs3691189	2;1
cg1000809	0.21465	0.607974	11.01765	6E-06	0.000685	4.651457	0.50065	0.715299	-0.21465	11	93469286	R	II	SNORA40	TSS1500	opensea	TSS1500-op		rs1507924	2
cg2422244	0.202726	0.520986	11.01734	6E-06	0.000685	4.651251	0.419623	0.622349	-0.20273	13	43986058	F	II	ENOX1	ExonBnd	opensea	ExonBnd-op		rs7682457	5
cg1038464	0.213008	0.55336	11.0155	6.01E-06	0.000685	4.650037	0.446856	0.659864	-0.21301	9	84982365	F	II		IGR	opensea	IGR-opense		rs5631459	0;31;33
cg2604550	0.228582	0.658584	11.01513	6.01E-06	0.000685	4.649794	0.544293	0.772876	-0.22858	18	11309047	F	II		IGR	opensea	IGR-opense		rs5467269	0
cg0192008	0.223924	0.418882	11.01508	6.01E-06	0.000685	4.649759	0.30692	0.530844	-0.22392	20	9966720	F	II	LOC10192(	TSS1500	opensea	TSS1500-op		rs5734145	1
cg0155875	0.201969	0.493988	11.01449	6.02E-06	0.000685	4.649368	0.393003	0.594972	-0.20197	6	73450879	F	II	KCNQ5	Body	opensea	Body-opens		rs5576439	0;43
cg2628869	0.231338	0.526412	11.01128	6.03E-06	0.000685	4.647244	0.410743	0.64208	-0.23134	1	1.2E+08	F	II		IGR	opensea	IGR-opense			
cg1627957	0.206003	0.549818	11.00983	6.03E-06	0.000685	4.646283	0.446817	0.65282	-0.206	6	12275455	R	II		IGR	opensea	IGR-opense		rs5295188	22
cg0024824	0.218694	0.590944	11.00954	6.04E-06	0.000685	4.646089	0.481597	0.700291	-0.21869	2	2.41E+08	R	II		IGR	shore	IGR-shore	chr2:2408(		
cg1828239	0.233188	0.553994	11.00939	6.04E-06	0.000685	4.64599	0.4374	0.670588	-0.23319	2	1.12E+08	R	II	ACOXL	Body	opensea	Body-opens		rs5517676	1;41
cg1884235	0.204843	0.464585	11.00911	6.04E-06	0.000686	4.645807	0.362163	0.567006	-0.20484	13	97794052	F	II		IGR	opensea	IGR-opense		rs5468635	17
cg0221656	0.217109	0.600803	11.0084	6.04E-06	0.000686	4.645333	0.492248	0.709357	-0.21711	10	96857781	F	II		IGR	opensea	IGR-opense		rs1506831	12;14;44
cg1734574	0.204519	0.467321	11.00737	6.04E-06	0.000686	4.64465	0.365062	0.569581	-0.20452	14	69472652	F	II		IGR	opensea	IGR-opense		rs1920510	8
cg1617038	0.2311	0.475764	11.00446	6.06E-06	0.000686	4.642727	0.360214	0.591314	-0.2311	8	1.04E+08	F	II	AZIN1-AS1	Body	opensea	Body-opens		rs5350708	0;7;41
cg1372540	0.211397	0.655738	11.00377	6.06E-06	0.000686	4.642265	0.55004	0.761437	-0.2114	3	1.25E+08	R	II	MUC13	Body	opensea	Body-opens		rs5277627	44;32;11
cg2197568	0.230689	0.530784	11.00303	6.06E-06	0.000687	4.641774	0.415439	0.646128	-0.23069	3	1.87E+08	R	II	MASP1	Body	opensea	Body-opens		rs710473;r	42;29;8
cg1255533	0.201342	0.488912	11.00184	6.07E-06	0.000687	4.640988	0.388241	0.589582	-0.20134	7	56120290	F	II	CCT6A	Body	shore	Body-shore	chr7:5611(		
cg1703169	0.251267	0.405116	11.00129	6.07E-06	0.000687	4.640619	0.279483	0.53075	-0.25127	12	94602306	F	II	PLXNC1	Body	opensea	Body-opens		rs1925419	0;1;10;27
cg1269294	0.206806	0.57195	10.99748	6.08E-06	0.000687	4.638093	0.468547	0.675353	-0.20681	4	1.41E+08	F	II		IGR	opensea	IGR-opense		rs5427686	25;30
cg0293916	0.201156	0.548967	10.9969	6.09E-06	0.000687	4.637712	0.448389	0.649545	-0.20116	10	60828539	R	II		IGR	opensea	IGR-opense		rs3197	46
cg0845474	0.226265	0.592022	10.9969	6.09E-06	0.000687	4.637709	0.47889	0.705155	-0.22626	1	2.46E+08	F	II	KIF26B	Body	opensea	Body-opens		rs5582636	2;41
cg1807982	0.231838	0.495233	10.99645	6.09E-06	0.000687	4.637413	0.379314	0.611151	-0.23184	11	36614820	R	II	RAG2	Body	opensea	Body-opens		rs5459108	51;12;2
cg1262900	0.228031	0.558708	10.99631	6.09E-06	0.000687	4.637322	0.444693	0.672724	-0.22803	1	58625006	F	II	DAB1	5'UTR	opensea	5'UTR-open		rs1878761	1;39
cg0273250	0.203917	0.527811	10.99615	6.09E-06	0.000687	4.637213	0.425852	0.62977	-0.20392	16	89628853	R	II	RPL13	Body	shore	Body-shore	chr16:896:	rs1894763	40;18;14
cg0193671	0.202437	0.663735	10.99429	6.1E-06	0.000687	4.635983	0.562516	0.764953	-0.20244	10	4144300	R	II		IGR	opensea	IGR-opense		rs5564631	27;1
cg1088085	0.206299	0.713037	10.99326	6.1E-06	0.000687	4.635299	0.609887	0.816186	-0.2063	3	35833333	R	II	ARPP21	Body	opensea	Body-opens		rs1441666	43;6
cg0238442	0.205454	0.4991	10.9914	6.11E-06	0.000687	4.634063	0.396373	0.601827	-0.20545	11	1.3E+08	R	II	ST14	Body	opensea	Body-opens		rs5537089	37;3;1
cg0516207	0.222235	0.521295	10.98929	6.12E-06	0.000687	4.632663	0.410177	0.632413	-0.22224	11	13142205	R	II		IGR	opensea	IGR-opense		rs1901609	11;10
cg1269932	0.205484	0.53321	10.98857	6.12E-06	0.000688	4.632183	0.430468	0.635952	-0.20548	3	1.17E+08	R	II		IGR	opensea	IGR-opense		rs9881096	34;24;7;1
cg1179589	0.240925	0.692548	10.98835	6.12E-06	0.000688	4.632037	0.572085	0.81301	-0.24092	6	87855840	F	II		IGR	opensea	IGR-opense		rs5569807	43
cg1500269	0.210653	0.563803	10.98759	6.12E-06	0.000688	4.631533	0.458477	0.66913	-0.21065	7	1.38E+08	F	II	AKR1D1	TSS200	opensea	TSS200-ope		rs7779865	0;10;35;45
cg1130363	0.235759	0.55456	10.98745	6.12E-06	0.000688	4.631445	0.436681	0.67244	-0.23576	2	1.92E+08	R	II	STAT4	Body	opensea	Body-opens		rs5676133	2
cg0304340	0.202926	0.577786	10.98703	6.12E-06	0.000688	4.631165	0.476323	0.679249	-0.20293	1	45242356	R	II	RPS8	Body	shore	Body-shore	chr1:4524:	rs2014406	35;25;22
cg0972064	0.230156	0.436348	10.98671	6.13E-06	0.000688	4.630952	0.32127	0.551426	-0.23016	16	78929792	R	II	WWOX	Body	opensea	Body-opens		rs5703876	16
cg0512105	0.202394	0.500606	10.98373	6.14E-06	0.000689	4.628974	0.399409	0.601803	-0.20239	22	33313979	F	II	SYN3	Body	opensea	Body-opens		rs5315165	4;29;34
cg0594446	0.203301	0.719531	10.98334	6.14E-06	0.000689	4.628711	0.61788	0.821181	-0.2033	15	74661351	F	II	CYP11A1	TSS1500	shelf	TSS1500-sh	chr15:746(		
cg1026960	0.245138	0.494473	10.98248	6.14E-06	0.000689	4.628141	0.371903	0.617042	-0.24514	4	1.2E+08	R	II	USP53	5'UTR	opensea	5'UTR-open		rs1469883	1
cg2327482	0.225874	0.480544	10.98204	6.14E-06	0.000689	4.627851	0.367607	0.593481	-0.22587	8	67104670	F	II	LINC00967	Body	opensea	Body-opens		rs5411038	1;18;22;40
cg0567661	0.218319	0.39222	10.982	6.15E-06	0.000689	4.627822	0.283061	0.501379	-0.21832	12	7173728	F	II	C1S	Body	opensea	Body-opens			
cg0792073	0.237106	0.540011	10.98141	6.15E-06	0.000689	4.627433	0.421457	0.658564	-0.23711	12	14405823	R	II		IGR	opensea	IGR-opense			
cg0519913	0.218541	0.599661	10.97909	6.16E-06	0.000689	4.625891	0.49039	0.708931	-0.21854	3	14288295	R	II		IGR	opensea	IGR-opense		rs1508489	1
cg1291565	0.216739	0.524461	10.97754	6.16E-06	0.000689	4.624859	0.416092	0.632831	-0.21674	4	1.24E+08	F	II		IGR	opensea	IGR-opense		rs5972865	1
cg0867552	0.235107	0.546844	10.97609	6.17E-06	0.00069	4.623896	0.42929	0.664398	-0.23511	11	18716666	F	II		IGR	shelf	IGR-shelf	chr11:187:	rs1497659	41
cg1011133	0.210152	0.507715	10.97603	6.17E-06	0.00069	4.623858	0.40264	0.612791	-0.21015	6	1.06E+08	F	II		IGR	opensea	IGR-opense			
cg0942667	0.21735	0.569303	10.97411	6.18E-06	0.00069	4.622578	0.460628	0.677978	-0.21735	9	5644909	R	II	RIC1	Body	opensea	Body-opens		rs3677883	6
cg2550332	0.233418	0.633216	10.97266	6.18E-06	0.00069	4.621614	0.516507	0.749925	-0.23342	7	36567098	R	II	AOAH	Body	opensea	Body-opens			
cg0629595	0.217743	0.420079	10.97117	6.19E-06	0.000691	4.620626	0.311208	0.528951	-0.21774	18	71019915	R	II		IGR	opensea	IGR-opense		rs5421295	49
cg0415077	0.238647	0.601847	10.96875	6.2E-06	0.000691	4.619017	0.482524	0.721171	-0.23865	7	40587972	F	II	SUGCT	Body	opensea	Body-opens		rs2011954	17
cg0469999	0.200043	0.719917	10.96745	6.2E-06	0.000691	4.618154	0.619896	0.819938	-0.20004	22	36231914	F	II	RBFOX2	Body	opensea	Body-opens			
cg1773615	0.250086	0.480865	10.96678	6.21E-06	0.000691	4.61771	0.355821	0.605908	-0.25009	1	31661388	F	II	NKAIN1	Body	opensea	Body-opens		rs5560152	1;5
cg0101009	0.23245	0.660227	10.96555	6.21E-06	0.000691	4.616889	0.544002	0.776452	-0.23245	14	22292347	F	II		IGR	opensea	IGR-opense		rs1158986	1;35
cg0116733	0.210119	0.599104	10.96508	6.21E-06	0.000691	4.616579	0.494044	0.704163	-0.21012	10	1.05E+08	F	II		IGR	opensea	IGR-opense			
cg2719364	0.245702	0.434599	10.96401	6.22E-06	0.000691	4.615865	0.311747	0.55745	-0.2457	21	26549225	F	II		IGR	opensea	IGR-opense			
cg1712275	0.224477	0.554229	10.96384	6.22E-06	0.000691	4.61575	0.441991	0.666467	-0.22448	2	65804208	F	II		IGR	opensea	IGR-opense		rs1505672	29;33;36;50
cg1478297	0.206047	0.568843	10.9597	6.24E-06	0.000693	4.612995	0.46582	0.671867	-0.20605	12	53189960	R	II	KRT3	TSS200	opensea	TSS200-ope		rs5397848	31;28;14

cg2435939	0.239485	0.498865	10.95933	6.24E-06	0.000693	4.61275	0.379123	0.618607	-0.23948	9	1.36E+08	F	II	TMEM8C	TSS1500	opensea	TSS1500-op		rs1500020	15
cg1197929	0.235766	0.471281	10.959	6.24E-06	0.000693	4.612534	0.353398	0.589165	-0.23577	3	1.02E+08	F	II	LOC15222	Body	opensea	Body-opens		rs5594694	1;25
cg2126947	0.213847	0.542826	10.95808	6.24E-06	0.000693	4.611919	0.435903	0.64975	-0.21385	14	1.03E+08	R	II	MOK	Body	opensea	Body-opens		rs1427032	16;2
cg0964495	0.209436	0.574025	10.95744	6.24E-06	0.000693	4.611494	0.469307	0.678744	-0.20944	3	1.22E+08	F	II	CD86	TSS1500	opensea	TSS1500-op		rs1192234	18;30
cg0552025	0.265508	0.621261	10.95645	6.25E-06	0.000693	4.610833	0.488507	0.754015	-0.26551	5	88104209	R	II	MEF2C	Body	opensea	Body-opens		rs5637046	37;36
cg0295212	0.215768	0.519476	10.95571	6.25E-06	0.000694	4.61034	0.411592	0.62736	-0.21577	2	2.3E+08	F	II	DNER	Body	opensea	Body-opens		rs1147231	13
cg2510899	0.25483	0.626975	10.95466	6.26E-06	0.000694	4.609644	0.49956	0.75439	-0.25483	2	1.14E+08	F	II		IGR	opensea	IGR-opense		rs1483016	39
cg0915235	0.224403	0.52201	10.95289	6.26E-06	0.000694	4.608466	0.409809	0.634212	-0.2244	2	10134152	R	II	GRHL1	Body	opensea	Body-opens		rs7843484	45;38;1
cg0545138	0.21869	0.448036	10.95223	6.27E-06	0.000694	4.608025	0.338691	0.557381	-0.21869	3	1.98E+08	R	II	LRCH3	Body	shore	Body-shore	chr3:1975		
cg1139061	0.208899	0.479329	10.95223	6.27E-06	0.000694	4.608023	0.374879	0.583778	-0.2089	7	46589190	F	II		IGR	opensea	IGR-opense		rs1829823	1;46
cg2402922	0.218735	0.552864	10.95152	6.27E-06	0.000694	4.607549	0.443496	0.662232	-0.21874	13	50257914	R	II	EBPL	Body	opensea	Body-opens		rs3751274	39;38;14;3;1
cg0807976	0.214373	0.680145	10.95008	6.28E-06	0.000694	4.606592	0.572958	0.787331	-0.21437	7	1.3E+08	F	II	COPG2	Body	opensea	Body-opens		rs5731596	0
cg0613362	0.286172	0.564363	10.94722	6.29E-06	0.000694	4.604688	0.421277	0.707449	-0.28617	4	1.01E+08	R	II	EMCN	Body	opensea	Body-opens		rs3611005	50;2;1
cg1349344	0.236355	0.612425	10.94664	6.29E-06	0.000694	4.604297	0.494247	0.730602	-0.23635	3	5833716	F	II		IGR	opensea	IGR-opense		rs5601762	0;1
cg2762831	0.251858	0.583293	10.94512	6.3E-06	0.000694	4.603285	0.457364	0.709222	-0.25186	2	1.1E+08	R	II	SH3RF3	Body	opensea	Body-opens		rs1125545	50;43;41;16
cg1608030	0.226488	0.478435	10.94508	6.3E-06	0.000694	4.603262	0.365191	0.591679	-0.22649	21	45087640	R	II	RRP1B	Body	opensea	Body-opens		rs5415435	47;2
cg0279093	0.225751	0.565528	10.94373	6.3E-06	0.000694	4.602362	0.452652	0.678403	-0.22575	3	23373256	F	II	UBE2E2	Body	opensea	Body-opens		rs5544304	0;1;41
cg2588468	0.209673	0.601554	10.94338	6.3E-06	0.000694	4.602123	0.496718	0.70639	-0.20967	3	24587682	F	II		IGR	opensea	IGR-opense		rs5539913	40;47
cg1781085	0.221284	0.597032	10.94167	6.31E-06	0.000695	4.600985	0.48639	0.707674	-0.22128	6	42369411	R	II	TRERF1	5'UTR	opensea	5'UTR-oper		rs260282;r	22;15;2
cg0005222	0.227688	0.632533	10.94167	6.31E-06	0.000695	4.600984	0.518689	0.746377	-0.22769	12	15783053	F	II	EPS8	Body	opensea	Body-opens		rs5569469	0;31;48
cg1419365	0.228219	0.594935	10.94131	6.31E-06	0.000695	4.600746	0.480825	0.709044	-0.22822	9	94178868	R	II	NFIL3	5'UTR	opensea	5'UTR-oper			
cg1023666	0.247655	0.585361	10.93959	6.32E-06	0.000695	4.599598	0.461533	0.709188	-0.24765	3	1.88E+08	R	II	LPP	Body	opensea	Body-opens		rs5615344	34;2
cg2122365	0.292188	0.431253	10.93629	6.33E-06	0.000695	4.5974	0.285159	0.577347	-0.29219	10	15873568	R	II	FAM188A	Body	opensea	Body-opens		rs1153725	24
cg0500709	0.236661	0.57767	10.93471	6.34E-06	0.000696	4.596346	0.45934	0.696001	-0.23666	16	77322779	R	II	ADAMTS18	Body	opensea	Body-opens		rs1164331	24;1
cg2737039	0.210658	0.461656	10.93238	6.35E-06	0.000697	4.59479	0.356327	0.566985	-0.21066	3	1.78E+08	R	II		IGR	opensea	IGR-opense		rs1840133	39;21
cg0197424	0.21939	0.479148	10.93048	6.36E-06	0.000697	4.593524	0.369453	0.588843	-0.21939	3	1.07E+08	F	II		IGR	opensea	IGR-opense		rs3693876	13
cg0500627	0.231177	0.585665	10.92758	6.37E-06	0.000697	4.591583	0.470077	0.701254	-0.23118	9	14243730	R	II	NFIB	Body	opensea	Body-opens		rs7870033	51;32;22;14
cg2427744	0.229707	0.574953	10.92474	6.38E-06	0.000698	4.58969	0.4601	0.689807	-0.22971	14	52796897	F	II		IGR	opensea	IGR-opense		rs1834354	27;43
cg1942051	0.212915	0.643848	10.92417	6.38E-06	0.000698	4.589311	0.537391	0.750305	-0.21291	12	41124066	R	II	CNTN1	5'UTR	opensea	5'UTR-oper		rs5331366	41;24
cg0240876	0.259444	0.634348	10.92226	6.39E-06	0.000698	4.588033	0.504626	0.76407	-0.25944	6	1.49E+08	F	II	UST	Body	opensea	Body-opens		rs5621830	0;1;21;41;42;49
cg0256260	0.205105	0.590487	10.9198	6.4E-06	0.000699	4.586388	0.487934	0.693039	-0.2051	2	1.45E+08	R	II		IGR	opensea	IGR-opense		rs5419051	42;12
cg0289942	0.215034	0.442008	10.91906	6.4E-06	0.000699	4.585893	0.334491	0.549525	-0.21503	10	55072007	R	II		IGR	opensea	IGR-opense			
cg1324787	0.210799	0.655626	10.91642	6.42E-06	0.0007	4.584131	0.550227	0.761026	-0.2108	8	12868246	R	II	KIAA1456	Body	opensea	Body-opens		rs5778392	44;29;8;7
cg0908796	0.229547	0.459048	10.9161	6.42E-06	0.0007	4.583916	0.344274	0.573821	-0.22955	7	23503778	R	II	IGF2BP3	Body	opensea	Body-opens		rs1095094	26;15
cg0345980	0.217502	0.52832	10.91446	6.42E-06	0.0007	4.58282	0.419569	0.637072	-0.2175	1	2.26E+08	R	II	EPHX1	1stExon	opensea	1stExon-op		rs5717112	2
cg1061078	0.204973	0.522061	10.91422	6.43E-06	0.0007	4.582656	0.419574	0.624547	-0.20497	2	2.07E+08	F	II		IGR	opensea	IGR-opense		rs5520493	3
cg0543725	0.211323	0.611509	10.91203	6.43E-06	0.0007	4.581198	0.505847	0.717171	-0.21132	4	95612104	R	II		IGR	opensea	IGR-opense		rs5554483	39;29;21
cg1982627	0.202831	0.443404	10.91128	6.44E-06	0.0007	4.580692	0.341989	0.54482	-0.20283	16	80617770	R	II		IGR	opensea	IGR-opense		rs1417916	44;27
cg0815309	0.214188	0.640174	10.91116	6.44E-06	0.0007	4.580616	0.533079	0.747268	-0.21419	3	1.51E+08	F	II	CLRN1	TSS1500	opensea	TSS1500-op		rs1409066	0
cg1148737	0.230403	0.477612	10.90998	6.44E-06	0.0007	4.579826	0.36241	0.592813	-0.2304	2	9428037	F	II	ASAP2	Body	opensea	Body-opens			
cg1531706	0.200331	0.597245	10.9095	6.45E-06	0.0007	4.579504	0.497079	0.69741	-0.20033	10	73741408	F	II	CHST3	5'UTR	opensea	5'UTR-oper		rs5683318	16;40
cg1493880	0.203482	0.556562	10.90648	6.46E-06	0.000701	4.577482	0.454822	0.658303	-0.20348	3	40141086	R	II	MYRIP	TSS1500	opensea	TSS1500-op		rs5704957	37;2;1
cg0683366	0.217005	0.37443	10.90528	6.46E-06	0.000702	4.576678	0.265928	0.482932	-0.217	1	54154751	R	II	GLIS1	5'UTR	opensea	5'UTR-oper		rs882879	27
cg0535283	0.218536	0.444178	10.90239	6.48E-06	0.000702	4.574748	0.33491	0.553446	-0.21854	6	33384391	R	II	CUTA	3'UTR	shore	3'UTR-shor	chr6:3338	rs5402820	13;3
cg2532645	0.205558	0.573406	10.89444	6.51E-06	0.000705	4.569423	0.470627	0.676185	-0.20556	5	1.63E+08	F	II		IGR	opensea	IGR-opense		rs5618837	27
cg0172632	0.205826	0.455378	10.8929	6.52E-06	0.000705	4.568393	0.352465	0.558291	-0.20583	5	1.49E+08	R	II	MIR143HG	TSS200	opensea	TSS200-ope		rs5682818	28;27;14
cg0659515	0.220601	0.604684	10.89253	6.52E-06	0.000706	4.568146	0.494383	0.714985	-0.2206	11	10716164	F	II	MRVI1	TSS1500	opensea	TSS1500-op		rs1408737	1;46
cg0287762	0.228998	0.602765	10.89135	6.52E-06	0.000706	4.567356	0.488266	0.717264	-0.229	2	33239383	F	II	LTBP1	Body	opensea	Body-opens		rs1494473	0;16;31;46
cg1555263	0.212334	0.604807	10.89111	6.52E-06	0.000706	4.567191	0.49864	0.710974	-0.21233	22	33297618	R	II	SYN3	Body	opensea	Body-opens		rs5669720	13;1
cg1190442	0.225036	0.479856	10.8881	6.54E-06	0.000706	4.565174	0.367338	0.592374	-0.22504	19	6592554	F	II	CD70	TSS1500	shore	TSS1500-sh	chr19:659	rs5442269	0
cg1773680	0.262153	0.391484	10.88772	6.54E-06	0.000706	4.564918	0.260408	0.522561	-0.26215	13	44875634	F	II		IGR	opensea	IGR-opense		rs5407378	12;13;44
cg2196169	0.209702	0.6069	10.88723	6.54E-06	0.000707	4.564591	0.502049	0.711752	-0.2097	11	62947565	R	II	SLC22A25	Body	opensea	Body-opens		rs5425688	43;29;15
cg2013930	0.20751	0.447019	10.8866	6.54E-06	0.000707	4.564172	0.343264	0.550774	-0.20751	20	34497362	F	II	PHF20	Body	opensea	Body-opens		rs5368041	0
cg2352116	0.281508	0.52019	10.88532	6.55E-06	0.000707	4.563309	0.379436	0.660944	-0.28151	14	77287324	F	II		IGR	opensea	IGR-opense		rs5457683	1;41
cg0588887	0.211727	0.440142	10.88467	6.55E-06	0.000707	4.562873	0.334278	0.546005	-0.21173	3	1.72E+08	F	II		IGR	opensea	IGR-opense		rs5297540	0;1;10
cg2017993	0.201782	0.456002	10.88426	6.55E-06	0.000707	4.562601	0.355111	0.556894	-0.20178	11	1.26E+08	R	II	KIRREL3	Body	opensea	Body-opens			
cg1605552	0.202718	0.53123	10.88399	6.56E-06	0.000707	4.562423	0.42987	0.632589	-0.20272	6	33083287	F	II	HLA-DPB2	Body	shore	Body-shore	chr6:3308	rs1444821	1;42
cg1369627	0.202566	0.558329	10.88389	6.56E-06	0.000707	4.562354	0.457046	0.659612	-0.20257	17	9950632	F	II	GAS7	Body	opensea	Body-opens		rs1473170	0;29;35
cg1686035	0.223999	0.543098	10.88307	6.56E-06	0.000707	4.561801	0.431098	0.655097	-0.224	22	26650819	R	II	SEZ6L	Body	opensea	Body-opens		rs1472734	19;1
cg1011435	0.234871	0.585294	10.88249	6.56E-06	0.000707	4.561414	0.467859	0.70273	-0.23487	11	35993896	F	II	LDLRAD3	5'UTR	opensea	5'UTR-oper		rs5701750	1
cg2487480	0.203726	0.554979	10.88188	6.56E-06	0.000707	4.561008	0.453116	0.656842	-0.20373	5	1.5E+08	F	II	CAMK2A	Body	opensea	Body-opens		rs5318526	0
cg1205212	0.204825	0.567378	10.8809	6.57E-06	0.000707	4.560347	0.464965	0.66979	-0.20482	10	80451110	F	II		IGR	opensea	IGR-opense			
cg1856212	0.201301	0.55563	10.88	6.57E-06	0.000708	4.559743	0.454979	0.65628	-0.2013	2	27076093	R	II	DPYSL5	5'UTR	shelf	5'UTR-shelf	chr2:2707		
cg1832293	0.22003	0.451451	10.87802	6.58E-06	0.000708	4.558415	0.341436	0.561466	-0.22003	6	1.17E+08	R	II	RSPH4A	Body	opensea	Body-opens		rs5578883	37;11;1
cg0058264	0.21936	0.629536	10.87731	6.58E-06	0.000708	4.557937	0.519856	0.739216	-0.21936	1	26331449	R	II		IGR	opensea	IGR-opense		rs5536089	49
cg0956311	0.270945	0.433486	10.87631	6.59E-06	0.000708	4.557268	0.298013	0.568959	-0.27095	14	1E+08	F	II		IGR	opensea	IGR-opense		rs1897442	0;2
cg0990441	0.239872	0.506194	10.87602	6.59E-06	0.000708	4.557073	0.386259	0.62613	-0.23987	4	1.41E+08	F	II	SCOC	5'UTR	opensea	5'UTR-oper		rs1882566	20;36;45;48
cg1733369	0.231828	0.555202	10.87431	6.6E-06	0.000709	4.555929	0.439288	0.671116	-0.23183	4	1.73E+08	F	II		IGR	shore	IGR-shore	chr4:1727	rs5441605	0;1;6
cg0373337	0.213768	0.430855	10.87366	6.6E-06	0.000709	4.555491	0.323971	0.537738	-0.21377	3	1.85E+08	F	II	LIPH	TSS200	opensea	TSS200-ope		rs5450177	0;50
cg0419877	0.212966	0.536208	10.87135	6.61E-06	0.000709	4.553937	0.429724	0.642691	-0.21297	5	1.7E+08	R	II		IGR	opensea	IGR-opense		rs1914812	39;28;2;1
cg2642996	0.255997	0.66598	10.868	6.62E-06	0.00071	4.551689	0.537981	0.793979	-0.256	15	83867672	F	II	HDGFRP3	Body	opensea	Body-opens		rs5557750	24
cg1821657	0.210849	0.643405	10.86292	6.65E-06	0.00071	4.548281	0.53798	0.74883	-0.21085	3	1.41E+08	F	II	ZBTB38	5'UTR	opensea	5'UTR-oper		rs5582900	19;25
cg1174807	0.201824	0.589473	10.86155	6.65E-06	0.00071	4.54736	0.488561	0.690385	-0.20182	4	1.66E+08	F	II		IGR	opensea	IGR-opense		rs5277709	0;49
cg2691213	0.243468	0.497411	10.86117	6.65E-06	0.00071	4.547101	0.375677	0.619145	-0.24347	7	1.3E+08	F	II		IGR	opensea	IGR-opense			
cg0914171	0.228614	0.545188	10.85701	6.67E-06	0.000711	4.544308	0.430881	0.659494	-0.22861	9	97428981	R	II		IGR	opensea	IGR-opense		rs1848910	49;46;27;11
cg0275371	0.204672	0.498264	10.85468	6.68E-06	0.000712	4.542742	0.395928	0.6006	-0.20467	17	66657633	R	II		IGR	opensea	IGR-opense		rs5679909	47
cg2495059	0.2051	0.495151	10.85456	6.68E-06	0.000712	4.542657	0.392601	0.597701	-0.2051	17	33302061	R	II		IGR	opensea	IGR-opense		rs1900712	39;36;11
cg0603332	0.225831	0.655386	10.85437	6.68E-06	0.000712	4.542534	0.542471	0.768302	-0.22583	1	86011409	R	II	DDAH1	5'UTR	opensea	5'UTR-oper		rs5673949	46;42
cg0322855	0.233738	0.579508	10.85417	6.69E-06	0.000712	4.542395	0.462639	0.696377	-0.23374	19	45174912	R	II	CEACAM19	5'UTR	opensea	5'UTR-oper			
cg0685792	0.205706	0.487709	10.85389	6.69E-06	0.000712	4.542206	0.384856	0.590562	-0.20571	1	37084266	F	II		IGR	opensea	IGR-opense		rs5292390	3
cg0116599	0.21653	0.454151	10.85296	6.69E-06	0.000712	4.541585	0.345886	0.562416	-0.21653	2	2.01E+08	R	II		IGR	opensea	IGR-opense		rs7664089	43;30;23;1
cg0548369	0.226476	0.590976	10.85153	6.7E-06	0.000712	4.540622	0.477738	0.704214	-0.22648	12	216093	F	II	IQSEC3	5'UTR	shore	5'UTR-shor	chr12:213		
cg2761753	0.208895	0.529823	10.84778	6.71E-06	0.000713	4.538098	0.425375	0.63427	-0.20889	5	1.35E+08	F	II		IGR	opensea	IGR-opense		rs1893815	1;9;25;41
cg1217651	0.207491	0.542913	10.84708	6.72E-06	0.000713	4.537628	0.439168	0.646659	-0.20749	12	66912585	R	II	GRIP1	Body	opensea	Body-opens		rs1422283	28;2;1
cg0593884	0.201484	0.516745	10.84513	6.73E-06	0.000713	4.536313	0.416003	0.617487	-0.20148	16	4207303	F	II		IGR	opensea	IGR-opense			
cg0236905	0.21105	0.605768	10.84305	6.74E-06	0.000714	4.534917	0.500243	0.711294	-0.21105	15	92941926	F	II	ST8SIA2	Body	shelf	Body-shelf	chr15:929	rs1472141	1
cg1607464	0.202904	0.580002	10.84263	6.74E-06	0.000714	4.534634	0.47855	0.681454	-0.2029	6	1.16E+08	F	II	NT5DC1	Body	shore	Body-shore	chr6:1164	rs1196819	0
cg1403406	0.248493	0.510923	10.83965	6.75E-06	0.000714	4.532622	0.386676	0.63517	-0.24849	22	39282561	R	II		IGR	opensea	IGR-opense		rs5630978	9
cg0429653	0.237788	0.404361	10.8388	6.75E-06	0.000714	4.532051	0.285467	0.523255	-0.23779	14	95778707	F	II	CLMN	Body	opensea	Body-opens		rs1928476	0;1;7;26
cg0199013	0.248657	0.582953	10.83786	6.76E-06	0.000714	4.531418	0.458625	0.707282	-0.24866	19	51898727	F	II		IGR	shore	IGR-shore	chr19:518	rs3724930	1;4;33
cg2625162	0.233324	0.590103	10.83776	6.76E-06	0.000714	4.531355	0.47344	0.706765	-0.23332	7	28682097	R	II	CREB5	Body	opensea	Body-opens		rs1816551	43;18;2
cg0609930	0.202155	0.397585	10.83725	6.76E-06	0.000714	4.531008	0.296508	0.498662	-0.20215	1	96672496	R	II		IGR	opensea	IGR-opense		rs1406150	26;22;21;18
cg1707316	0.255864	0.507857	10.83691	6.76E-06	0.000714	4.530779	0.379925	0.635788	-0.25586	3	1.11E+08	F	II		IGR	opensea	IGR-opense		rs5601347	29;37
cg1635452	0.208652	0.581611	10.83464	6.77E-06	0.000714	4.529251	0.477285	0.685937	-0.20865	1	1.53E+08	F	II	LCE2A	3'UTR	opensea	3'UTR-oper			
cg2509020	0.212067	0.45597	10.83452	6.77E-06	0.000714	4.529169	0.349937	0.562004	-0.21207	7	1.48E+08	F	II		IGR	opensea	IGR-opense		rs1819861	0;6;19
cg0915712	0.236849	0.602515	10.83225	6.78E-06	0.000715	4.527638	0.484091	0.72094	-0.23685	2	69201416	R	II	GKN1	TSS1500	opensea	TSS1500-op		rs7976194	23;2;1
cg0087016	0.21195	0.412875	10.82851	6.8E-06	0.000715	4.525116	0.3069	0.51885	-0.21195	12	12584171	F	II	LOH12CR1	Body	opensea	Body-opens		rs1923269	0;20;31
cg1239310	0.252442	0.578114	10.82725	6.81E-06	0.000715	4.524267	0.451893	0.704335	-0.25244	11	1.24E+08	R	II		IGR	opensea	IGR-opense		rs1866220	51;37;16
cg2421564	0.237812	0.600006	10.82714	6.81E-06	0.000715	4.524193	0.4811	0.718912	-0.23781	10	91001364	R	II	LIPA	Body	opensea	Body-opens		rs1896579	17
cg1240563	0.217189	0.546815	10.8271	6.81E-06	0.000715	4.524167	0.43822	0.655409	-0.21719	7	72745074	R	II	FKBP6	Body	shelf	Body-shelf	chr7:7274	rs1445205	1
cg0909492	0.213724	0.397784	10.82684	6.81E-06	0.000715	4.523993	0.290922	0.504646	-0.21372	8	86456883	R	II		IGR	opensea	IGR-opense		rs5641763	17;1
cg2591816	0.220923	0.661014	10.82365	6.82E-06	0.000716	4.521841	0.550553	0.771476	-0.22092	2	1.46E+08	F	II		IGR	opensea	IGR-opense		rs5406500	1;34;44;46
cg0628253	0.207404	0.390402	10.82355	6.82E-06	0.000716	4.521773	0.2867	0.494105	-0.2074	8	1.44E+08	R	II		IGR	opensea	IGR-opense		rs5318236	33
cg0916132	0.202684	0.610493	10.82347	6.82E-06	0.000716	4.521723	0.50915	0.711835	-0.20268	8	1054717	R	II	ERICH1-AS1	Body	shore	Body-shore	chr8:1053	rs1134022	11;2
cg0800904	0.219859	0.636676	10.82304	6.83E-06	0.000716	4.521431	0.526747	0.746605	-0.21986	1	2.46E+08	R	II	KIF26B	Body	opensea	Body-opens			
cg1114618	0.223986	0.652509	10.82267	6.83E-06	0.000716	4.521181	0.540515	0.764502	-0.22399	15	39593169	F	II		IGR	opensea	IGR-opense		rs3708730	1;26
cg2002378	0.202295	0.359874	10.8214	6.83E-06	0.000716	4.520323	0.258727	0.461022	-0.20229	2	2.22E+08	R	II		IGR	opensea	IGR-opense		rs3758192	5
cg2153667	0.215961	0.491921	10.82063	6.84E-06	0.000716	4.519801	0.383941	0.599902	-0.21596	1	55951958	R	II		IGR	opensea	IGR-opense		rs5317256	14;2
cg2175155	0.25292	0.457777	10.81937	6.84E-06	0.000716	4.518956	0.331317	0.584238	-0.25292	21	32451135	F	II		IGR	opensea	IGR-opense		rs5522770	1;24;26;39
cg0402421	0.215623	0.538935	10.81845	6.85E-06	0.000716	4.518334	0.431124	0.646747	-0.21562	10	35003357	R	II	PARD3	Body	opensea	Body-opens		rs1819344	19;1
cg0579250	0.231893	0.571465	10.81788	6.85E-06	0.000717	4.517945	0.455518	0.687411	-0.23189	9	1.13E+08	F	II	PALM2	Body	opensea	Body-opens		rs1237552	7;9;18
cg0131373	0.214718	0.595512	10.8172	6.85E-06	0.000717	4.517491	0.488153	0.702871	-0.21472	12	10252119	R	II	CLEC1A	TSS1500	opensea	TSS1500-op			
cg0702059	0.218736	0.542689	10.81643	6.86E-06	0.000717	4.516967	0.433322	0.652057	-0.21874	10	69146234	R	II	CTNNA3	Body	opensea	Body-opens			
cg1146309	0.20265	0.459746	10.81639	6.86E-06	0.000717	4.516943	0.358421	0.561071	-0.20265	9	1.02E+08	R	II	COL15A1	Body	shelf	Body-shelf	chr9:1017		
cg2358011	0.269824	0.484164	10.81608	6.86E-06	0.000717	4.516736	0.349252	0.619076	-0.26982	12	68511699	F	II		IGR	opensea	IGR-opense		rs1460827	0
cg1038784	0.212288	0.58663	10.81295	6.87E-06	0.000717	4.514618	0.480486	0.692774	-0.21229	18	29757472	F	II		IGR	opensea	IGR-opense		rs5348795	1
cg0701742	0.226149	0.61243	10.81253	6.87E-06	0.000717	4.514339	0.499355	0.725504	-0.22615	7	3247724	R	II		IGR	opensea	IGR-opense		rs1886997	48;36;2
cg1227908	0.219431	0.499654	10.81105	6.88E-06	0.000718	4.513339	0.389938	0.60937	-0.21943	3	1.17E+08	R	II		IGR	opensea	IGR-opense		rs5488651	34;1
cg0024799	0.216203	0.549279	10.81085	6.88E-06	0.000718	4.5132	0.441177	0.65738	-0.2162	5	7529982	R	II	ADCY2	Body	opensea	Body-opens		rs1901776	1

cg0472171	0.217769	0.515206	10.8085	6.89E-06	0.000718	4.511615	0.406321	0.62409	-0.21777	11	32416490	R	II	WT1	Body	opensea	Body-opens		rs1466356	26;15;1
cg0234670	0.200609	0.519576	10.80766	6.9E-06	0.000718	4.511049	0.419272	0.619881	-0.20061	15	1.02E+08	R	II		IGR	opensea	IGR-opense		rs5649056	29;10;1
cg2117411	0.215646	0.527781	10.8076	6.9E-06	0.000718	4.511006	0.419958	0.635604	-0.21565	3	1.5E+08	F	II	LINC01213	TSS200	opensea	TSS200-ope			
cg0871134	0.25332	0.485648	10.80725	6.9E-06	0.000718	4.510773	0.358989	0.612308	-0.25332	9	1.24E+08	F	II	TRAF1	Body	opensea	Body-opens		rs1850025	1;34
cg1009587	0.201169	0.575416	10.80719	6.9E-06	0.000718	4.51073	0.474831	0.676001	-0.20117	10	75523308	F	II	SEC24C	Body	opensea	Body-opens			
cg0824346	0.230943	0.522361	10.80707	6.9E-06	0.000718	4.510652	0.406889	0.637832	-0.23094	20	56274036	F	II	PMEPA1	5'UTR	opensea	5'UTR-oper		rs5772691	23
cg1435537	0.225584	0.613664	10.80671	6.9E-06	0.000718	4.510409	0.500872	0.726457	-0.22558	10	3850160	R	II		IGR	opensea	IGR-opense		rs1809953	36;6;2
cg1369649	0.253002	0.488134	10.80629	6.9E-06	0.000718	4.510122	0.361633	0.614634	-0.253	3	1.51E+08	F	II	LOC20165	TSS1500	opensea	TSS1500-op		rs1144074	1
cg0373131	0.238694	0.389513	10.80615	6.9E-06	0.000718	4.510029	0.270165	0.50886	-0.23869	6	1.49E+08	F	II	UST	Body	opensea	Body-opens		rs9498156	19;49
cg1188541	0.319135	0.492537	10.80583	6.9E-06	0.000718	4.509812	0.332969	0.652104	-0.31914	21	30715207	R	II	BACH1	3'UTR	opensea	3'UTR-oper		rs5398539	13
cg0410368	0.215131	0.570757	10.8058	6.9E-06	0.000718	4.509795	0.463191	0.678322	-0.21513	21	37999823	F	II		IGR	opensea	IGR-opense		rs5734896	0
cg1633727	0.202333	0.677027	10.80463	6.91E-06	0.000718	4.509003	0.575861	0.778193	-0.20233	3	1.17E+08	R	II		IGR	opensea	IGR-opense			
cg0212370	0.236159	0.501877	10.80404	6.91E-06	0.000718	4.508601	0.383798	0.619956	-0.23616	6	1.36E+08	F	II	AHI1	Body	opensea	Body-opens			
cg2518976	0.24924	0.569868	10.80132	6.92E-06	0.000719	4.506765	0.445248	0.694488	-0.24924	6	1.12E+08	R	II	FYN	5'UTR	opensea	5'UTR-oper		rs1916832	41;17;14;11
cg1707431	0.216568	0.60257	10.80028	6.93E-06	0.000719	4.506059	0.494285	0.710854	-0.21657	6	1.4E+08	R	II	LOC10050	Body	opensea	Body-opens		rs5738424	48
cg1747448	0.260761	0.46843	10.79936	6.93E-06	0.000719	4.50544	0.338049	0.59881	-0.26076	12	20047827	F	II		IGR	opensea	IGR-opense		rs5284874	44
cg1633283	0.207459	0.458085	10.79832	6.94E-06	0.00072	4.504733	0.354355	0.561815	-0.20746	15	1.01E+08	R	II	LRRK1	Body	opensea	Body-opens		rs3724335	47
cg0647766	0.242171	0.651046	10.79755	6.94E-06	0.00072	4.504214	0.52996	0.772131	-0.24217	13	46757415	R	I	LCP1	TSS1500	opensea	TSS1500-op		rs1895407	24;22;16;11;4;1
cg1074314	0.252207	0.617498	10.79663	6.95E-06	0.00072	4.503591	0.491394	0.743601	-0.25221	13	94446931	F	II	GPC6	Body	opensea	Body-opens		rs5623907	1;8;35
cg0864450	0.205472	0.589883	10.79601	6.95E-06	0.00072	4.503172	0.487147	0.692619	-0.20547	22	20771324	R	II		IGR	opensea	IGR-opense		rs1876399	8
cg1833451	0.204659	0.561416	10.79584	6.95E-06	0.00072	4.503057	0.459087	0.663746	-0.20466	10	56245086	R	II	PCDH15	Body	opensea	Body-opens		rs5509436	24
cg0891463	0.237976	0.454843	10.79307	6.96E-06	0.000721	4.501186	0.335855	0.573831	-0.23798	3	1.21E+08	F	II	HCLS1	Body	opensea	Body-opens		rs5422666	1;24
cg0969506	0.213532	0.425961	10.79305	6.96E-06	0.000721	4.501174	0.319196	0.532727	-0.21353	1	51661252	F	II		IGR	opensea	IGR-opense			
cg1001548	0.228476	0.662639	10.7884	6.98E-06	0.000722	4.498025	0.548401	0.776877	-0.22848	15	39685589	F	II		IGR	opensea	IGR-opense		rs5649919	0;32
cg0024017	0.220422	0.565442	10.78811	6.99E-06	0.000722	4.497829	0.455231	0.675652	-0.22042	6	74232108	R	II	EEF1A1	TSS1500	shore	TSS1500-sh	chr6:7423	rs5353778	35
cg2502092	0.223999	0.48281	10.78724	6.99E-06	0.000722	4.497238	0.370811	0.59481	-0.224	8	42742938	R	II	RNF170	Body	opensea	Body-opens		rs1486119	45
cg0994242	0.221915	0.648469	10.78691	6.99E-06	0.000722	4.497017	0.537511	0.759426	-0.22191	17	74913419	F	II	MGAT5B	Body	opensea	Body-opens		rs1171965	0;28
cg2258150	0.209279	0.71331	10.78636	6.99E-06	0.000722	4.496647	0.608671	0.817949	-0.20928	5	1.7E+08	R	II	CTD-2270F	Body	opensea	Body-opens		rs1498547	37;34;28;4;1
cg1075131	0.227818	0.418608	10.7863	6.99E-06	0.000722	4.496602	0.304699	0.532517	-0.22782	20	57557998	F	II	NELFCD	Body	shore	Body-shore	chr20:575	rs1859013	11;12;17
cg0152100	0.206873	0.596977	10.78618	7E-06	0.000722	4.496523	0.493541	0.700414	-0.20687	10	90759822	F	II	FAS	Body	opensea	Body-opens		rs9658715	14;20;24
cg2717396	0.21433	0.582511	10.78375	7.01E-06	0.000723	4.494877	0.475346	0.689676	-0.21433	1	68841715	R	II		IGR	opensea	IGR-opense		rs1827353	49;24;19
cg2502675	0.210266	0.604757	10.78257	7.01E-06	0.000723	4.494078	0.499623	0.70989	-0.21027	5	57702195	F	II		IGR	opensea	IGR-opense		rs1840512	24
cg0117780	0.231062	0.481904	10.78098	7.02E-06	0.000724	4.493004	0.366373	0.597435	-0.23106	17	39042031	F	II	KRT20	TSS1500	opensea	TSS1500-op		rs1397193	32;41
cg2175419	0.225851	0.578625	10.77864	7.03E-06	0.000724	4.491415	0.465699	0.69155	-0.22585	4	57393820	F	II		IGR	shelf	IGR-shelf	chr4:5739	rs5595802	1;39
cg1231838	0.207569	0.431871	10.77508	7.05E-06	0.000725	4.489007	0.328087	0.535655	-0.20757	10	79000235	F	II	KCNMA1	Body	opensea	Body-opens		rs5731436	30
cg0637765	0.228285	0.664147	10.77362	7.05E-06	0.000726	4.488014	0.550005	0.77829	-0.22829	3	1.06E+08	R	II		IGR	opensea	IGR-opense		rs5365641	49;2
cg2297873	0.206085	0.536315	10.77172	7.06E-06	0.000726	4.486725	0.433272	0.639357	-0.20608	3	1.95E+08	F	II		IGR	opensea	IGR-opense		rs1153288	20;26
cg1344260	0.206026	0.574836	10.77158	7.06E-06	0.000726	4.486633	0.471823	0.677849	-0.20603	11	3071269	R	II	CARS	Body	opensea	Body-opens		rs5403079	51;37;36;26
cg0107608	0.27027	0.504343	10.77076	7.07E-06	0.000726	4.486074	0.369208	0.639478	-0.27027	3	1.78E+08	F	II	KCNMB2	5'UTR	opensea	5'UTR-oper		rs5316963	0;29
cg1370210	0.225721	0.606203	10.76894	7.08E-06	0.000727	4.484841	0.493342	0.719063	-0.22572	16	68774709	R	II	CDH1	Body	shelf	Body-shelf	chr16:687		
cg0002493	0.213871	0.492231	10.76819	7.08E-06	0.000727	4.484332	0.385296	0.599167	-0.21387	1	1.93E+08	F	II		IGR	opensea	IGR-opense			
cg1413258	0.208873	0.532605	10.76713	7.08E-06	0.000727	4.483616	0.428168	0.637041	-0.20887	5	95513202	R	II	LOC10192	Body	opensea	Body-opens		rs1499386	35;29;1
cg0384596	0.208044	0.562866	10.76701	7.09E-06	0.000727	4.483533	0.458843	0.666888	-0.20804	3	65880266	F	II	MAGI1	Body	opensea	Body-opens		rs1853162	30
cg2175575	0.206773	0.541549	10.76278	7.11E-06	0.000728	4.480666	0.438163	0.644936	-0.20677	8	1.19E+08	R	II	SAMD12	Body	opensea	Body-opens		rs5627921	35;31
cg1851990	0.225568	0.512012	10.76156	7.11E-06	0.000728	4.479832	0.399228	0.624796	-0.22557	11	95289759	F	II		IGR	opensea	IGR-opense		rs5507908	29;37
cg0573248	0.200613	0.587729	10.76126	7.11E-06	0.000728	4.479633	0.487422	0.688035	-0.20061	13	94709668	F	II	GPC6	Body	opensea	Body-opens		rs1829557	21
cg1549113	0.245645	0.59771	10.75975	7.12E-06	0.000729	4.478608	0.474888	0.720532	-0.24564	2	40545033	R	II	SLC8A1	Body	opensea	Body-opens		rs1159447	25
cg1307468	0.201779	0.552948	10.75964	7.12E-06	0.000729	4.478531	0.452058	0.653838	-0.20178	15	40113167	R	II	GPR176	Body	opensea	Body-opens		rs1500213	11
cg0139411	0.225652	0.448708	10.75907	7.12E-06	0.000729	4.478144	0.335882	0.561534	-0.22565	5	1.5E+08	R	II	RBM22	TSS1500	shore	TSS1500-sh	chr5:1500	rs5471269	48;20
cg2085848	0.253953	0.501885	10.75849	7.13E-06	0.000729	4.477753	0.374908	0.628861	-0.25395	15	52369365	F	II		IGR	opensea	IGR-opense		rs5521638	1
cg1715152	0.206985	0.61724	10.75845	7.13E-06	0.000729	4.477723	0.513748	0.720733	-0.20698	3	99246483	F	II		IGR	opensea	IGR-opense		rs5355101	0;14
cg0575044	0.210007	0.546608	10.75683	7.13E-06	0.000729	4.476623	0.441604	0.651611	-0.21001	11	36208284	R	II	LDLRAD3	Body	opensea	Body-opens		rs1861512	49;48;41;1
cg0475346	0.213781	0.442363	10.75508	7.14E-06	0.00073	4.475438	0.335473	0.549253	-0.21378	12	53569794	R	II	CSAD	5'UTR	opensea	5'UTR-oper		rs1839856	15;13;1
cg0624666	0.208709	0.473914	10.75394	7.15E-06	0.00073	4.474661	0.36956	0.578269	-0.20871	8	26712348	F	II	ADRA1A	Body	opensea	Body-opens		rs5309310	0;26
cg1676068	0.261141	0.644956	10.7532	7.15E-06	0.00073	4.474161	0.514385	0.775527	-0.26114	6	1.35E+08	F	II		IGR	opensea	IGR-opense			
cg1518798	0.230291	0.57069	10.74991	7.17E-06	0.000731	4.471923	0.455545	0.685835	-0.23029	17	42675980	F	II		IGR	opensea	IGR-opense		rs5557443	12
cg0541570	0.211831	0.549101	10.74939	7.17E-06	0.000731	4.471571	0.443186	0.655016	-0.21183	9	27109023	F	II	TEK	TSS200	opensea	TSS200-ope		rs5671624	0;12;30
cg2657471	0.21593	0.634086	10.74925	7.17E-06	0.000731	4.471477	0.526121	0.742051	-0.21593	3	93881733	F	II		IGR	opensea	IGR-opense		rs1162539	0
cg1212624	0.239394	0.605284	10.74751	7.18E-06	0.000731	4.470294	0.485587	0.724981	-0.23939	14	59742285	F	II	DAAM1	Body	opensea	Body-opens		rs5550038	1;19;44
cg0159468	0.216573	0.571499	10.74678	7.18E-06	0.000731	4.469798	0.463213	0.679785	-0.21657	16	3115053	R	II	IL32	TSS1500	opensea	TSS1500-op		rs1882202	46;2
cg0024209	0.241132	0.518802	10.74643	7.18E-06	0.000731	4.469558	0.398236	0.639368	-0.24113	1	2.36E+08	F	II		IGR	opensea	IGR-opense		rs5608731	0;21;26
cg1167509	0.238513	0.452848	10.74612	7.18E-06	0.000731	4.469352	0.333592	0.572105	-0.23851	1	1.18E+08	R	II		IGR	opensea	IGR-opense		rs5586714	27;7
cg2203500	0.208357	0.655484	10.74609	7.19E-06	0.000731	4.469328	0.551306	0.759663	-0.20836	5	58647658	F	II	PDE4D	Body	opensea	Body-opens			
cg1065205	0.237611	0.546066	10.74589	7.19E-06	0.000731	4.469195	0.427261	0.664872	-0.23761	13	43649126	R	II	DNAJC15	Body	opensea	Body-opens		rs1430925	36;35;28;11
cg0620957	0.20561	0.554104	10.74502	7.19E-06	0.000731	4.468605	0.451299	0.656909	-0.20561	4	4890431	R	II		IGR	opensea	IGR-opense		rs5564162	47;41
cg0799309	0.212576	0.507992	10.74331	7.2E-06	0.000731	4.467437	0.401704	0.61428	-0.21258	7	1.17E+08	R	II	CTTNBP2	Body	opensea	Body-opens		rs5476321	46
cg0195289	0.246864	0.576009	10.74328	7.2E-06	0.000731	4.467419	0.452577	0.699441	-0.24686	5	1.11E+08	R	II	STARD4-AS	Body	opensea	Body-opens		rs1457719	7
cg1789544	0.214066	0.533553	10.74016	7.21E-06	0.000732	4.4653	0.42652	0.640586	-0.21407	4	1.74E+08	R	II	GALNTL6	Body	opensea	Body-opens		rs1155799	13;5;2
cg0697566	0.218953	0.541674	10.74009	7.21E-06	0.000732	4.465249	0.432197	0.65115	-0.21895	5	83491377	F	II	EDIL3	Body	opensea	Body-opens		rs5778190	1;8
cg2495066	0.202435	0.666241	10.73658	7.23E-06	0.000733	4.462862	0.565023	0.767458	-0.20244	2	33066042	R	II	LINC00486	Body	opensea	Body-opens		rs1886177	49;35;1
cg1120928	0.20376	0.504764	10.73489	7.24E-06	0.000733	4.46171	0.402884	0.606644	-0.20376	3	1.38E+08	F	II	MRAS	Body	opensea	Body-opens			
cg0788221	0.213595	0.342494	10.73452	7.24E-06	0.000733	4.461458	0.235696	0.449291	-0.21359	8	21541227	F	II		IGR	opensea	IGR-opense		rs5336409	1;2
cg1828198	0.222432	0.599068	10.73401	7.24E-06	0.000733	4.461115	0.487852	0.710284	-0.22243	20	6699284	R	II		IGR	opensea	IGR-opense			
cg1823485	0.212677	0.616888	10.73279	7.25E-06	0.000733	4.460281	0.51055	0.723226	-0.21268	3	1.06E+08	R	II	CBLB	Body	opensea	Body-opens		rs1163046	19;4;2
cg1532026	0.212167	0.576765	10.73179	7.25E-06	0.000733	4.4596	0.470681	0.682848	-0.21217	5	1.58E+08	F	II		IGR	opensea	IGR-opense		rs1480885	0
cg2434887	0.237209	0.381835	10.73158	7.26E-06	0.000733	4.459462	0.26323	0.500439	-0.23721	6	1.49E+08	F	II		IGR	opensea	IGR-opense		rs1485131	0;1
cg1244663	0.201064	0.527755	10.73047	7.26E-06	0.000733	4.458702	0.427223	0.628287	-0.20106	1	87636203	F	II		IGR	opensea	IGR-opense		rs5630288	30
cg0297927	0.256697	0.652417	10.73045	7.26E-06	0.000733	4.458688	0.524068	0.780766	-0.2567	8	20367570	R	II		IGR	opensea	IGR-opense		rs7805047	33;31;1
cg1769722	0.225132	0.610761	10.72987	7.26E-06	0.000733	4.458298	0.498195	0.723327	-0.22513	8	1.1E+08	R	II		IGR	opensea	IGR-opense		rs5536167	37;15;11;10
cg2068497	0.209621	0.457451	10.72945	7.27E-06	0.000733	4.458011	0.35264	0.562261	-0.20962	7	1.23E+08	F	II		IGR	opensea	IGR-opense		rs5437201	5;16
cg0192617	0.219777	0.462325	10.72756	7.27E-06	0.000734	4.456722	0.352437	0.572214	-0.21978	8	1.29E+08	R	II		IGR	opensea	IGR-opense		rs5753607	4;1
cg1088448	0.238684	0.498298	10.7264	7.28E-06	0.000734	4.455933	0.378956	0.61764	-0.23868	2	1.92E+08	R	II		IGR	opensea	IGR-opense		rs5449776	46;23;16;2
cg0094335	0.245665	0.541203	10.7262	7.28E-06	0.000734	4.455797	0.41837	0.664035	-0.24567	8	13222600	R	II	DLC1	Body	opensea	Body-opens		rs5424827	1
cg0339334	0.219766	0.507781	10.72584	7.28E-06	0.000734	4.455555	0.397898	0.617664	-0.21977	10	72751056	R	II		IGR	opensea	IGR-opense		rs5553028	22
cg2012773	0.20451	0.46558	10.72411	7.29E-06	0.000734	4.454375	0.363325	0.567835	-0.20451	10	33635645	R	II		IGR	opensea	IGR-opense		rs1400317	2
cg2456290	0.234005	0.577885	10.72307	7.3E-06	0.000735	4.453665	0.460883	0.694888	-0.234	9	1.14E+08	F	II		IGR	opensea	IGR-opense			
cg0733514	0.214689	0.582351	10.72067	7.31E-06	0.000735	4.45203	0.475006	0.689695	-0.21469	1	1.1E+08	F	II		IGR	opensea	IGR-opense		rs1846597	18;19;40
cg0758278	0.220493	0.501041	10.72057	7.31E-06	0.000735	4.451961	0.390795	0.611288	-0.22049	6	53171642	F	II	ELOVL5	5'UTR	opensea	5'UTR-oper		rs9395853	0;16;32
cg1565502	0.229678	0.602953	10.7202	7.31E-06	0.000735	4.451712	0.488114	0.717792	-0.22968	8	41575780	F	II	ANK1	Body	opensea	Body-opens		rs1823488	0;1;14
cg0280788	0.220324	0.576449	10.72017	7.31E-06	0.000735	4.451693	0.466287	0.686611	-0.22032	6	1.26E+08	F	II	TPD52L1	5'UTR	opensea	5'UTR-oper		rs5728543	21
cg1061548	0.229807	0.611679	10.71945	7.31E-06	0.000735	4.451198	0.496775	0.726582	-0.22981	2	31181446	F	II	GALNT14	Body	opensea	Body-opens		rs7929270	1;2
cg2294925	0.228525	0.475622	10.7193	7.32E-06	0.000735	4.4511	0.361359	0.589884	-0.22852	1	67135552	F	II	SGIP1	Body	opensea	Body-opens		rs5766792	0;10;27;48
cg0736959	0.219254	0.609931	10.71922	7.32E-06	0.000735	4.451044	0.500304	0.719557	-0.21925	13	31712722	R	II	HSPH1	ExonBnd	opensea	ExonBnd-op		rs5706973	1
cg0587888	0.21827	0.660311	10.71891	7.32E-06	0.000735	4.450829	0.551176	0.769446	-0.21827	3	99620389	F	II	C3orf26	Body	opensea	Body-opens		rs1512481	42;47
cg1148183	0.214809	0.680843	10.71569	7.33E-06	0.000735	4.448637	0.573439	0.788247	-0.21481	17	36075377	F	II	HNF1B	Body	opensea	Body-opens		rs5600340	47
cg0097132	0.201735	0.520198	10.71495	7.34E-06	0.000735	4.448132	0.41933	0.621065	-0.20173	3	1.19E+08	R	II	MAATS1	Body	opensea	Body-opens		rs7489862	42
cg1266814	0.217913	0.571019	10.71464	7.34E-06	0.000735	4.447923	0.462062	0.679975	-0.21791	2	56229195	R	II	MIR216B	TSS1500	opensea	TSS1500-op		rs5428739	15
cg0862835	0.213364	0.616091	10.71427	7.34E-06	0.000735	4.447669	0.509409	0.722774	-0.21336	14	51989095	F	II	FRMD6-AS2	Body	opensea	Body-opens		rs5621596	0;1;32
cg1473265	0.214222	0.558139	10.7128	7.35E-06	0.000736	4.446668	0.451028	0.66525	-0.21422	4	1.07E+08	F	II		IGR	opensea	IGR-opense		rs5319931	1;24;40
cg0924980	0.208132	0.630176	10.71221	7.35E-06	0.000736	4.446266	0.52611	0.734242	-0.20813	16	84846936	F	II		IGR	opensea	IGR-opense		rs5582057	0;46
cg1637104	0.216299	0.51105	10.71207	7.35E-06	0.000736	4.446172	0.402901	0.6192	-0.2163	1	51975433	R	II	EPS15	Body	opensea	Body-opens		rs5761075	25;17
cg0932678	0.211308	0.591211	10.71148	7.35E-06	0.000736	4.445765	0.485557	0.696865	-0.21131	3	1.93E+08	R	II	MB21D2	Body	opensea	Body-opens		rs3677982	19;11;1
cg0601211	0.20897	0.736863	10.71099	7.36E-06	0.000736	4.445433	0.632377	0.841348	-0.20897	8	11341573	F	II		IGR	opensea	IGR-opense		rs5701738	34;49
cg1801628	0.227733	0.519597	10.7098	7.36E-06	0.000736	4.444622	0.405731	0.633464	-0.22773	13	95834131	R	II	ABCC4	Body	opensea	Body-opens		rs1912913	49;1
cg2609118	0.226275	0.343899	10.70968	7.36E-06	0.000736	4.444541	0.230761	0.457037	-0.22628	1	77415478	R	II	ST6GALNAC	Body	opensea	Body-opens		rs5507793	1
cg0857159	0.232691	0.504206	10.70756	7.37E-06	0.000736	4.443096	0.38786	0.620551	-0.23269	6	64225663	F	II		IGR	opensea	IGR-opense			
cg0170453	0.208595	0.643045	10.70548	7.38E-06	0.000737	4.441676	0.538748	0.747343	-0.20859	4	40518024	R	II	RBM47	5'UTR	opensea	5'UTR-oper		rs5332106	14;2
cg2542317	0.224554	0.576187	10.70495	7.39E-06	0.000737	4.441313	0.46391	0.688464	-0.22455	3	12236303	R	II		IGR	opensea	IGR-opense		rs5663822	50;2
cg1607439	0.221902	0.439168	10.70384	7.39E-06	0.000737	4.440556	0.328217	0.550119	-0.2219	15	58256507	R	II	ALDH1A2	Body	opensea	Body-opens		rs5738941	28;20
cg0390929	0.205602	0.601639	10.70299	7.4E-06	0.000738	4.439973	0.498839	0.70444	-0.2056	5	61086467	R	II		IGR	opensea	IGR-opense		rs1154519	46;33;25;12;2;1
cg2153801	0.209202	0.585747	10.70095	7.41E-06	0.000738	4.438584	0.481145	0.690348	-0.2092	1	2.3E+08	F	II		IGR	opensea	IGR-opense		rs1454227	0;2;31;34
cg0664362	0.202277	0.569027	10.70002	7.41E-06	0.000739	4.437945	0.467889	0.670166	-0.20228	7	1.17E+08	F	II	CTTNBP2	Body	opensea	Body-opens		rs1142494	0
cg1771941	0.202154	0.554755	10.69904	7.42E-06	0.000739	4.437279	0.453678	0.655833	-0.20215	18	73139433	R	II	SMIM21	1stExon	opensea	1stExon-op		rs5714971	25;1
cg1090057	0.234045	0.479224	10.69822	7.42E-06	0.000739	4.436717	0.362201	0.596246	-0.23404	8	1.36E+08	R	II	LOC10192	Body	opensea	Body-opens			
cg0384565	0.202838	0.520245	10.69767	7.42E-06	0.000739	4.436341	0.418826	0.621663	-0.20284	2	1.53E+08	R	II	FMNL2	Body	opensea	Body-opens			
cg0298100	0.226354	0.29351	10.69735	7.42E-06	0.000739	4.43612	0.180333	0.406687	-0.22635	10	1.35E+08	R	II	GPR123	Body	shore	Body-shore	chr10:134	rs1906855	35;10
cg1457147	0.244705	0.563784	10.69647	7.43E-06	0.000739	4.435523	0.441432	0.686137	-0.2447	1	2.18E+08	R	II	GPATCH2	Body	opensea	Body-opens		rs1895635	33;17;12;7
cg0341396	0.253618	0.531645	10.69573	7.43E-06	0.000739	4.435019	0.404836	0.658454	-0.25362	22	31136755	F	II	OSBP2	Body	opensea	Body-opens		rs1413962	0;2
cg0106105	0.230443	0.478598	10.6957	7.43E-06	0.000739	4.434999	0.363376	0.593819	-0.23044	6	84090476	F	II	ME1	Body	opensea	Body-opens		rs1909110	1;47

cg2122504	0.214026	0.573467	10.69499	7.44E-06	0.000739	4.434513	0.466454	0.68048	-0.21403	4	32147668	R	II	LOC10272	ExonBnd	opensea	ExonBnd-o			
cg0411937	0.201496	0.595757	10.69467	7.44E-06	0.000739	4.434291	0.495009	0.696505	-0.2015	14	1.01E+08	F	II		IGR	opensea	IGR-opense		rs5510676	22
cg1460604	0.212773	0.629188	10.69421	7.44E-06	0.000739	4.433982	0.522802	0.735575	-0.21277	3	1.13E+08	R	II		IGR	opensea	IGR-opense			
cg0697467	0.208905	0.595519	10.69227	7.45E-06	0.00074	4.432656	0.491067	0.699971	-0.2089	2	1.29E+08	R	II		IGR	opensea	IGR-opense		rs3698753	23;22;9;7
cg1202448	0.209679	0.590617	10.69178	7.45E-06	0.00074	4.432321	0.485777	0.695456	-0.20968	11	97527648	R	II		IGR	opensea	IGR-opense		rs7147549	15
cg0362698	0.202744	0.524874	10.69116	7.45E-06	0.00074	4.431898	0.423502	0.626246	-0.20274	7	1.44E+08	R	II	TPK1	Body	opensea	Body-open		rs1022570	31;7
cg2596957	0.224125	0.508118	10.68996	7.46E-06	0.00074	4.431078	0.396055	0.62018	-0.22412	6	1.51E+08	F	II		IGR	opensea	IGR-opense		rs1921948	0
cg2718131	0.249908	0.544719	10.6898	7.46E-06	0.00074	4.430966	0.419764	0.669673	-0.24991	7	1.48E+08	R	II		IGR	opensea	IGR-opense		rs1456714	48
cg225483C	0.20294	0.41462	10.68723	7.47E-06	0.000741	4.429206	0.31315	0.51609	-0.20294	4	1.1E+08	F	II	COL25A1	Body	opensea	Body-open		rs5564564	0
cg0631445	0.207669	0.395325	10.68681	7.48E-06	0.000741	4.428924	0.29149	0.499159	-0.20767	2	1.21E+08	R	II	RALB	Body	opensea	Body-open		rs7631671	47
cg2475479	0.228214	0.519493	10.68627	7.48E-06	0.000741	4.42855	0.405385	0.6336	-0.22821	4	1.74E+08	F	II		IGR	opensea	IGR-opense		rs5702959	0
cg2692858	0.256951	0.599821	10.68553	7.48E-06	0.000741	4.428047	0.471346	0.728297	-0.25695	7	25591490	F	II		IGR	opensea	IGR-opense			
cg1527473	0.206031	0.632583	10.68504	7.49E-06	0.000741	4.427715	0.529568	0.735599	-0.20603	18	46057682	F	II		IGR	opensea	IGR-opense		rs5390337	43;50
cg1188784	0.220508	0.582022	10.68415	7.49E-06	0.000741	4.427105	0.471767	0.692276	-0.22051	15	32392710	F	II	CHRNA7	Body	opensea	Body-open		rs5615183	38
cg0128404	0.224836	0.61516	10.68407	7.49E-06	0.000741	4.427049	0.502742	0.727578	-0.22484	10	23081250	R	II		IGR	opensea	IGR-opense		rs5566667	39;23;8;1
cg2538342	0.245145	0.546686	10.68394	7.49E-06	0.000741	4.426958	0.424114	0.669259	-0.24515	21	40259710	R	II	LOC40086	Body	opensea	Body-open			
cg2417153	0.227844	0.512059	10.68346	7.49E-06	0.000741	4.426635	0.398137	0.625982	-0.22784	8	55361122	F	II		IGR	opensea	IGR-opense			
cg0325642	0.229401	0.609876	10.68168	7.5E-06	0.000742	4.425415	0.495175	0.724576	-0.2294	12	13099884	F	II	GPRC5D	Body	opensea	Body-open		rs1821890	0;15;35
cg0718864	0.21177	0.589363	10.6796	7.51E-06	0.000742	4.423994	0.483478	0.695248	-0.21177	17	34208012	R	II	CCL5	TSS1500	opensea	TSS1500-o		rs5307775	27;2
cg1652481	0.224954	0.549574	10.67874	7.52E-06	0.000742	4.423406	0.437097	0.662051	-0.22495	1	2.36E+08	R	II	NID1	Body	opensea	Body-open		rs5548831	18;2
cg2351698	0.20573	0.535928	10.67873	7.52E-06	0.000742	4.423393	0.433062	0.638793	-0.20573	5	14356625	F	II	TRIO	Body	opensea	Body-open			
cg239181C	0.220644	0.598192	10.6781	7.52E-06	0.000742	4.422968	0.48787	0.708514	-0.22064	1	1.17E+08	F	II		IGR	opensea	IGR-opense		rs1870394	0
cg1119641	0.212443	0.656023	10.67657	7.53E-06	0.000742	4.421915	0.549802	0.762245	-0.21244	9	17131746	R	II		IGR	shelf	IGR-shelf	chr9:1713	rs966406	49
cg1108453	0.217847	0.565775	10.67508	7.54E-06	0.000743	4.420898	0.456851	0.674698	-0.21785	6	1.32E+08	R	II		IGR	shelf	IGR-shelf	chr6:1322		
cg2146428	0.209447	0.587887	10.67061	7.56E-06	0.000743	4.417838	0.483164	0.692611	-0.20945	4	41256169	R	II		IGR	shelf	IGR-shelf	chr4:4125	rs1870612	51;42;36;7
cg1962262	0.242492	0.451058	10.67031	7.56E-06	0.000743	4.417634	0.329812	0.572303	-0.24249	12	86230825	R	II	RASSF9	TSS1500	opensea	TSS1500-o		rs5705035	28;27
cg263779C	0.201246	0.422722	10.67014	7.56E-06	0.000743	4.417514	0.322099	0.523345	-0.20125	11	41876134	R	II		IGR	opensea	IGR-opense		rs5549067	42
cg1504861	0.261098	0.462332	10.66914	7.57E-06	0.000743	4.416829	0.331783	0.592881	-0.2611	12	32487523	F	II	BICD1	Body	opensea	Body-open		rs1125298	0
cg1625582	0.225455	0.587211	10.66859	7.57E-06	0.000743	4.416453	0.474483	0.699939	-0.22546	2	1.79E+08	R	II	PDE11A	Body	opensea	Body-open		rs5480387	26
cg1573342	0.213235	0.625358	10.66764	7.57E-06	0.000743	4.415804	0.518741	0.731976	-0.21323	7	18501832	R	II	HDAC9	Body	opensea	Body-open		rs5550225	28;24
cg1779702	0.250961	0.45644	10.66743	7.57E-06	0.000743	4.41566	0.330959	0.581921	-0.25096	1	68348868	F	II	GNG12-AS1	Body	opensea	Body-open		rs5698563	0;34;51
cg1821131	0.228356	0.488658	10.66574	7.58E-06	0.000744	4.414506	0.37448	0.602836	-0.22836	8	98437059	F	II	LOC10192	Body	opensea	Body-open		rs3685368	0;21;27;36;37
cg1436371	0.220367	0.639507	10.66555	7.58E-06	0.000744	4.414374	0.529323	0.749691	-0.22037	3	1.1E+08	R	II		IGR	opensea	IGR-opense		rs1861785	49
cg0229028	0.250933	0.583728	10.66416	7.59E-06	0.000744	4.413417	0.458261	0.709195	-0.25093	7	26479853	R	II	LOC44120	Body	opensea	Body-open		rs5646655	8
cg0370258	0.216027	0.57599	10.66415	7.59E-06	0.000744	4.413412	0.467976	0.684003	-0.21603	12	1.08E+08	R	II	BTBD11	Body	opensea	Body-open		rs5402955	44;30;10
cg1388815	0.211899	0.500819	10.66182	7.6E-06	0.000744	4.411818	0.39487	0.606769	-0.2119	17	73310148	F	II		IGR	opensea	IGR-opense		rs5716442	1;14
cg1511091	0.242288	0.681399	10.66121	7.61E-06	0.000744	4.4114	0.560255	0.802543	-0.24229	7	7959339	F	II		IGR	opensea	IGR-opense			
cg0051171	0.227562	0.439064	10.65698	7.63E-06	0.000745	4.408499	0.325283	0.552845	-0.22756	7	1.32E+08	F	II		IGR	opensea	IGR-opense		rs5704445	29
cg1715501	0.215509	0.389901	10.65604	7.63E-06	0.000745	4.407858	0.282146	0.497656	-0.21551	7	1.31E+08	R	II	LOC10050	Body	opensea	Body-open			
cg1107041	0.218621	0.575423	10.65505	7.64E-06	0.000745	4.407177	0.466113	0.684734	-0.21862	1	2.07E+08	F	II	C4BPA	TSS1500	opensea	TSS1500-o			
cg1126746	0.271375	0.591867	10.65439	7.64E-06	0.000745	4.406723	0.45618	0.727555	-0.27137	7	1.38E+08	F	II		IGR	opensea	IGR-opense			
cg1062322	0.206219	0.56929	10.65371	7.65E-06	0.000745	4.406257	0.466181	0.6724	-0.20622	3	1.73E+08	F	II	NLGN1	5'UTR	shelf	5'UTR-shelf	chr3:1731		
cg1750734	0.2017	0.540256	10.65351	7.65E-06	0.000745	4.406118	0.439405	0.641106	-0.2017	20	39357379	F	II		IGR	opensea	IGR-opense		rs5730159	12
cg1950076	0.210245	0.463178	10.6522	7.65E-06	0.000746	4.405224	0.358055	0.568301	-0.21025	2	41370011	F	II		IGR	opensea	IGR-opense		rs5591459	11;21;45;46
cg0597777	0.209432	0.539558	10.64922	7.67E-06	0.000746	4.403174	0.434842	0.644274	-0.20943	2	3741732	F	II	ALLC	Body	opensea	Body-open		rs1816107	1;35;40
cg1568382	0.284506	0.544068	10.64437	7.69E-06	0.000748	4.399851	0.401815	0.686321	-0.28451	6	37111556	R	II		IGR	opensea	IGR-opense		rs1137070	1
cg0597327	0.200151	0.63853	10.63966	7.72E-06	0.000749	4.396615	0.538454	0.738605	-0.20015	22	32860714	F	II		IGR	opensea	IGR-opense			
cg0671994	0.208455	0.615009	10.6387	7.72E-06	0.000749	4.395954	0.510782	0.719237	-0.20846	15	39448875	R	II		IGR	opensea	IGR-opense		rs5473811	44;36;27
cg0636023	0.237445	0.445251	10.63827	7.73E-06	0.000749	4.395657	0.326528	0.563974	-0.23745	21	33251408	F	II	HUNK	Body	opensea	Body-open		rs5400186	0;1;47
cg0183896	0.215312	0.487837	10.63536	7.74E-06	0.00075	4.393659	0.380181	0.595493	-0.21531	6	1.27E+08	R	II		IGR	opensea	IGR-opense		rs7771076	2
cg0457686	0.240637	0.594094	10.6331	7.75E-06	0.00075	4.392108	0.473775	0.714413	-0.24064	2	1.45E+08	R	II	GTDC1	5'UTR	opensea	5'UTR-oper		rs5588044	18;4;2;1
cg1084331	0.207365	0.557781	10.63286	7.75E-06	0.00075	4.391945	0.454099	0.661464	-0.20736	4	1.52E+08	R	II	FAM160A1	5'UTR	opensea	5'UTR-oper		rs1830246	45
cg0724243	0.210797	0.41105	10.63273	7.75E-06	0.00075	4.391854	0.305651	0.516448	-0.2108	2	35687894	R	II		IGR	opensea	IGR-opense		rs5577222	1
cg0848016	0.287765	0.495074	10.63268	7.75E-06	0.00075	4.39182	0.351192	0.638957	-0.28776	15	41036515	F	II	RMDN3	Body	opensea	Body-open			
cg2099411	0.225402	0.46924	10.63244	7.76E-06	0.00075	4.391654	0.356539	0.581941	-0.2254	1	2.1E+08	R	II	CAMK1G	Body	opensea	Body-open		rs1824074	23;1
cg2335242	0.217384	0.602486	10.63127	7.76E-06	0.00075	4.390848	0.493794	0.711178	-0.21738	13	44566114	F	II		IGR	opensea	IGR-opense			
cg2390687	0.200207	0.525442	10.63032	7.77E-06	0.00075	4.390199	0.425338	0.625545	-0.20021	6	24324774	R	II	DCDC2	Body	opensea	Body-open		rs5512372	21
cg1427502	0.220164	0.557666	10.6302	7.77E-06	0.00075	4.390112	0.447584	0.667748	-0.22016	3	467930	F	II		IGR	opensea	IGR-opense			
cg2189466	0.223629	0.384978	10.62939	7.77E-06	0.000751	4.389556	0.273164	0.496793	-0.22363	2	1.65E+08	R	II	GRB14	Body	opensea	Body-open			
cg1361461	0.222317	0.468742	10.62864	7.78E-06	0.000751	4.389042	0.357583	0.5799	-0.22232	15	65656635	R	II	IGDCC3	Body	shelf	Body-shelf	chr15:656	rs5379129	39;7
cg0313489	0.204635	0.643138	10.62796	7.78E-06	0.000751	4.388576	0.54082	0.745455	-0.20464	7	66343288	R	II		IGR	opensea	IGR-opense		rs5752921	1
cg1810658	0.20087	0.40166	10.62561	7.79E-06	0.000752	4.386955	0.301225	0.502095	-0.20087	5	53816023	F	II	SNX18	1stExon	shore	1stExon-sh	chr5:5381		
cg2619676	0.212465	0.582449	10.62518	7.79E-06	0.000752	4.386662	0.476216	0.688681	-0.21247	18	42451768	R	II	SETBP1	Body	opensea	Body-open		rs5405230	30;17
cg2300407	0.219342	0.60065	10.62439	7.8E-06	0.000752	4.386119	0.490979	0.710321	-0.21934	13	67551521	F	II	PCDH9-AS3	Body	opensea	Body-open		rs5719902	3;14
cg0568218	0.251144	0.544498	10.62352	7.8E-06	0.000752	4.38552	0.418926	0.67007	-0.25114	1	1.87E+08	F	II		IGR	opensea	IGR-opense		rs5327333	32
cg0023207	0.240658	0.52496	10.62309	7.8E-06	0.000752	4.385224	0.404631	0.645289	-0.24066	4	26753673	R	II	TBC1D19	Body	opensea	Body-open		rs7669392	34
cg0855836	0.230605	0.562293	10.62228	7.81E-06	0.000753	4.384668	0.446991	0.677596	-0.2306	1	94544894	R	II	ABCA4	ExonBnd	opensea	ExonBnd-o			
cg1942528	0.22111	0.460761	10.6202	7.82E-06	0.000753	4.383237	0.350206	0.571316	-0.22111	5	34917257	F	II	RAD1	TSS1500	shore	TSS1500-sh	chr5:3491	rs4127066	0;36
cg2643367	0.206798	0.664249	10.61931	7.82E-06	0.000753	4.382625	0.56085	0.767648	-0.2068	4	15230035	F	II	LOC10192	Body	opensea	Body-open		rs1148342	1;18;29;32;43
cg0509667	0.244741	0.514666	10.61772	7.83E-06	0.000753	4.381527	0.392295	0.637036	-0.24474	3	1.96E+08	F	II	SLC51A	Body	opensea	Body-open		rs1383308	1;5;33
cg1165804	0.222587	0.52395	10.61742	7.83E-06	0.000753	4.381321	0.412657	0.635244	-0.22259	8	18667350	R	II	PSD3	TSS1500	opensea	TSS1500-o		rs5315207	50;45
cg0332687	0.217216	0.612434	10.61659	7.84E-06	0.000753	4.380749	0.503826	0.721042	-0.21722	10	48332552	R	II		IGR	opensea	IGR-opense			
cg1275514	0.215129	0.587394	10.61658	7.84E-06	0.000753	4.380742	0.47983	0.694958	-0.21513	8	1.31E+08	F	II		IGR	opensea	IGR-opense		rs1931326	11
cg1337959	0.214857	0.513079	10.61629	7.84E-06	0.000753	4.380543	0.40565	0.620507	-0.21486	6	5981984	R	II		IGR	opensea	IGR-opense		rs5874004	41;29;15;2
cg2258652	0.210226	0.621467	10.6157	7.84E-06	0.000753	4.380139	0.516354	0.726579	-0.21023	11	16499411	F	II	SOX6	TSS1500	opensea	TSS1500-o		rs5435093	12;42
cg0017109	0.216993	0.490955	10.61556	7.84E-06	0.000753	4.380041	0.382459	0.599452	-0.21699	3	1.25E+08	F	II	ITGB5	Body	opensea	Body-open		rs1150094	0;47
cg1863003	0.261278	0.651132	10.61555	7.84E-06	0.000753	4.380036	0.520493	0.781771	-0.26128	17	60900969	R	II	MIR548W	Body	opensea	Body-open			
cg0728715	0.215185	0.573679	10.61544	7.85E-06	0.000753	4.379958	0.466087	0.681272	-0.21519	21	39579277	F	II	DSCR10	Body	opensea	Body-open		rs5544613	0;1;46
cg0871383	0.212051	0.671606	10.61426	7.85E-06	0.000754	4.37915	0.565581	0.777631	-0.21205	18	56009524	R	II	NEDD4L	Body	opensea	Body-open		rs5727202	10;2;1
cg0258409	0.242594	0.534232	10.61366	7.85E-06	0.000754	4.378734	0.412935	0.655529	-0.24259	17	6461275	F	II	PITPNM3	TSS1500	shore	TSS1500-sh	chr17:646	rs1411379	5;8;37;44
cg092810C	0.243175	0.594752	10.6135	7.86E-06	0.000754	4.378626	0.473165	0.71634	-0.24317	10	34919180	F	II	PARD3	Body	opensea	Body-open		rs1928373	13
cg0360647	0.206533	0.507068	10.61277	7.86E-06	0.000754	4.378121	0.403801	0.610334	-0.20653	3	1.03E+08	R	II		IGR	opensea	IGR-opense			
cg1398041	0.210731	0.622793	10.61172	7.87E-06	0.000754	4.3774	0.517428	0.728159	-0.21073	5	1.47E+08	R	II		IGR	opensea	IGR-opense		rs1400692	22
cg1660849	0.200398	0.542944	10.61102	7.87E-06	0.000754	4.376918	0.442745	0.643143	-0.2004	5	1.59E+08	R	II	RNF145	Body	opensea	Body-open		rs1841215	51;39;25;2;1
cg0823841	0.217696	0.434351	10.60925	7.88E-06	0.000754	4.375698	0.325503	0.543199	-0.2177	7	55416476	R	II		IGR	shelf	IGR-shelf	chr7:5541	rs5595578	20
cg168222C	0.210982	0.633584	10.60852	7.88E-06	0.000754	4.375197	0.528093	0.739075	-0.21098	6	1.49E+08	F	II		IGR	opensea	IGR-opense			
cg1344517	0.234238	0.560733	10.60796	7.89E-06	0.000754	4.374806	0.443614	0.677852	-0.23424	1	1.52E+08	R	II	S100A10	TSS1500	shore	TSS1500-sh	chr1:1519	rs1439642	11
cg1878103	0.225864	0.470395	10.60748	7.89E-06	0.000754	4.37448	0.357463	0.583327	-0.22586	5	1.28E+08	F	II	FBN2	Body	opensea	Body-open		rs7334137	14;33
cg2588314	0.242719	0.500687	10.60696	7.89E-06	0.000754	4.374119	0.379327	0.622046	-0.24272	2	2.33E+08	F	II	MIR1471	TSS200	opensea	TSS200-ope		rs5737045	0
cg1371356	0.206149	0.463152	10.60559	7.9E-06	0.000754	4.373173	0.360077	0.566227	-0.20615	10	78314461	F	II	C10orf11	Body	opensea	Body-open		rs1453935	43
cg2155738	0.214856	0.580517	10.60425	7.9E-06	0.000755	4.372255	0.473089	0.687945	-0.21486	12	31715602	F	II	DENND5B	Body	opensea	Body-open		rs5471249	2
cg0473612	0.215104	0.622744	10.60384	7.91E-06	0.000755	4.37197	0.515192	0.730296	-0.2151	4	1E+08	F	II		IGR	opensea	IGR-opense			
cg1416871	0.248212	0.550336	10.60352	7.91E-06	0.000755	4.37175	0.42623	0.674442	-0.24821	10	60579479	R	II	BICC1	Body	opensea	Body-open		rs7902375	24
cg2552254	0.210851	0.490802	10.60239	7.91E-06	0.000755	4.370967	0.385376	0.596227	-0.21085	5	17691839	R	II		IGR	opensea	IGR-opense		rs5339786	1
cg2326521	0.250383	0.556347	10.60235	7.92E-06	0.000755	4.370944	0.431156	0.681538	-0.25038	11	94536536	R	II	AMOTL1	Body	opensea	Body-open		rs1499675	46
cg0515603	0.214489	0.639446	10.59717	7.94E-06	0.000756	4.367371	0.532202	0.74669	-0.21449	5	82673625	R	II		IGR	opensea	IGR-opense		rs1690037	30
cg1905532	0.236049	0.453961	10.59611	7.95E-06	0.000756	4.366642	0.335937	0.571986	-0.23605	17	39341434	F	II	KRTAP4-1	TSS1500	opensea	TSS1500-o		rs1244931	39
cg0639825	0.216506	0.52498	10.59567	7.95E-06	0.000756	4.366334	0.416727	0.633233	-0.21651	3	1.84E+08	F	II	EIF4G1	Body	shore	Body-shore	chr3:1840	rs1854435	37;44;45
cg0008862	0.229547	0.514982	10.59501	7.95E-06	0.000756	4.365881	0.400208	0.629756	-0.22955	16	73224998	R	II		IGR	opensea	IGR-opense		rs5729082	2;1
cg1570034	0.202295	0.634641	10.59396	7.96E-06	0.000756	4.365156	0.533493	0.735788	-0.20229	5	24841861	R	II	LOC34010	TSS1500	opensea	TSS1500-o		rs5336971	1
cg0697454	0.211241	0.383002	10.5935	7.96E-06	0.000756	4.364838	0.277381	0.488622	-0.21124	14	34800530	R	II		IGR	opensea	IGR-opense		rs7749188	51;41;27;6
cg0283017	0.255289	0.506311	10.59341	7.96E-06	0.000756	4.364779	0.378666	0.633956	-0.25529	2	25576057	R	II		IGR	opensea	IGR-opense		rs5537198	5
cg0148503	0.228331	0.544631	10.59326	7.96E-06	0.000756	4.364673	0.430465	0.658796	-0.22833	7	3192834	F	II	LOC10012	Body	opensea	Body-open		rs5777472	1;30
cg1766168	0.200641	0.582609	10.59234	7.97E-06	0.000756	4.364041	0.482289	0.682929	-0.20064	8	73986912	F	II	C8orf84	Body	opensea	Body-open		rs1389332	0;14;32
cg1179774	0.215124	0.712586	10.59214	7.97E-06	0.000756	4.363902	0.605024	0.820148	-0.21512	10	73755332	R	II	CHST3	5'UTR	opensea	5'UTR-oper		rs1821934	2
cg083714C	0.22952	0.462484	10.59026	7.98E-06	0.000757	4.362602	0.347724	0.577243	-0.22952	9	6598558	F	II	GLDC	Body	opensea	Body-open		rs5289862	11;29;33;50
cg106944C	0.228119	0.525683	10.58716	8E-06	0.000758	4.36046	0.411624	0.639743	-0.22812	7	1.44E+08	F	II	OR2F1	TSS1500	opensea	TSS1500-o		rs1811382	1;23;51
cg1310641	0.205212	0.524957	10.58401	8.01E-06	0.000758	4.35829	0.422351	0.627563	-0.20521	6	1.11E+08	R	II		IGR	opensea	IGR-opense		rs5505408	7;2
cg1513811	0.246293	0.437051	10.58401	8.01E-06	0.000758	4.358286	0.313904	0.560197	-0.24629	6	1.5E+08	F	II	PPP1R14C	Body	opensea	Body-open			
cg0381857	0.225019	0.454495	10.58327	8.02E-06	0.000759	4.357774	0.341985	0.567004	-0.22502	11	40312660	F	II	LRRC4C	5'UTR	opensea	5'UTR-oper		rs5770543	0;18;31;51
cg085262C	0.210288	0.492959	10.58293	8.02E-06	0.000759	4.357545	0.387814	0.598103	-0.21029	3	1.39E+08	R	II		IGR	shelf	IGR-shelf	chr3:1393	rs1470572	43;1
cg261640C	0.235707	0.596639	10.5826	8.02E-06	0.000759	4.357315	0.478786	0.714493	-0.23571	6	1.23E+08	R	II	HSF2	Body	opensea	Body-open		rs1451035	1
cg1832181	0.201948	0.462099	10.58136	8.03E-06	0.000759	4.356457	0.361125	0.563073	-0.20195	14	59436794	R	II	LINC01500	Body	opensea	Body-open		rs1926250	41;32;31;22
cg030860C	0.244787	0.584958	10.58039	8.03E-06	0.000759	4.355791	0.462565	0.707352	-0.24479	11	1.2E+08	R	II		IGR	shelf	IGR-shelf	chr11:120	rs1228514	42;16;2;1
cg0295432	0.204303	0.640991	10.57999	8.04E-06	0.00076	4.355514	0.538839	0.743142	-0.2043	3	67045043	R	II		IGR	shelf	IGR-shelf	chr3:6704	rs5746379	12;2;1
cg1658805	0.216631	0.48349	10.57952	8.04E-06	0.00076	4.355184	0.375175	0.591806	-0.21663	5	1.23E+08	R	II		IGR	opensea	IGR-opense			
cg0004098	0.214976	0.654076	10.57881	8.04E-06	0.00076	4.354699	0.546587	0.761564	-0.21498	1	1.57E+08	F	II	ARHGEF11	Body	opensea	Body-open		rs5499228	0;42;49
cg0257374	0.213939	0.650538	10.57692	8.05E-06	0.00076	4.353391	0.543568	0.757507	-0.21394	15	56120298	R	II	NEDD4	3'UTR	opensea	3'UTR-oper			
cg1745231	0.201803	0.669946	10.57543	8.06E-06	0.000761	4.35236	0.569045	0.770847	-0.2018	13	36884806	F	II	SPG20	Body	opensea	Body-open		rs5321284	31;43

cg2710294	0.221288	0.556084	10.56735	8.11E-06	0.000762	4.346771	0.44544	0.666727	-0.22129	15	99679368	F	II	TTC23	Body	opensea	Body-opens		rs1466673	26;31;35
cg0215324	0.202005	0.553693	10.56712	8.11E-06	0.000762	4.346616	0.452691	0.654696	-0.20201	8	1.42E+08	R	II	PTK2	Body	opensea	Body-opens			
cg1175829	0.20759	0.516368	10.56701	8.11E-06	0.000762	4.34654	0.412573	0.620163	-0.20759	2	2.32E+08	F	II		IGR	shelf	IGR-shelf	chr2:2319(	rs7740275	46
cg1593015	0.202552	0.428169	10.56528	8.12E-06	0.000763	4.345341	0.326893	0.529445	-0.20255	10	30185196	F	II		IGR	opensea	IGR-opense		rs5406191	28
cg2497619	0.240372	0.459934	10.56195	8.14E-06	0.000763	4.343036	0.339748	0.580119	-0.24037	4	1.01E+08	R	II		IGR	opensea	IGR-opense		rs5511034	2;1
cg0738703	0.211442	0.700064	10.55921	8.15E-06	0.000764	4.34114	0.594343	0.805785	-0.21144	12	66233746	F	II	HMGA2	Body	opensea	Body-opens		rs1826071	1;29
cg1305106	0.201993	0.518039	10.55814	8.16E-06	0.000764	4.3404	0.417043	0.619036	-0.20199	8	1.22E+08	F	II		IGR	opensea	IGR-opense		rs5369976	0;1;29
cg2017140	0.224891	0.491109	10.55811	8.16E-06	0.000764	4.340379	0.378664	0.603554	-0.22489	12	1.21E+08	F	II	RPLP0	ExonBnd	shore	ExonBnd-sh	chr12:120(	rs1493381	14;31;35
cg1504515	0.210998	0.552278	10.55743	8.16E-06	0.000764	4.33991	0.446779	0.657777	-0.211	3	1.9E+08	R	II	LEPREL1	Body	opensea	Body-opens		rs5671274	22;7;2
cg1478688	0.20761	0.661339	10.55711	8.16E-06	0.000764	4.339687	0.557534	0.765144	-0.20761	10	1.02E+08	R	II	ERLIN1	Body	opensea	Body-opens		rs1180503	5;1
cg2280322	0.213856	0.570711	10.55675	8.16E-06	0.000764	4.339437	0.463783	0.677639	-0.21386	12	64587031	R	II	C12orf66	3'UTR	opensea	3'UTR-oper		rs5731463	22;7;1
cg1405869	0.217212	0.57825	10.55652	8.17E-06	0.000764	4.339278	0.469644	0.686856	-0.21721	13	63200547	R	II		IGR	opensea	IGR-opense		rs5633557	22
cg1712732	0.210201	0.590871	10.55597	8.17E-06	0.000764	4.338896	0.485771	0.695972	-0.2102	4	88812922	F	II		IGR	opensea	IGR-opense		rs7249025	35;50
cg2466792	0.231845	0.498573	10.55557	8.17E-06	0.000764	4.338623	0.382651	0.614496	-0.23185	21	27764738	F	II		IGR	opensea	IGR-opense		rs5615840	1;26;28
cg1484248	0.218609	0.584619	10.55522	8.17E-06	0.000764	4.338379	0.475314	0.693923	-0.21861	11	1.19E+08	R	II	CBL	Body	opensea	Body-opens			
cg1227175	0.20727	0.622715	10.5549	8.17E-06	0.000764	4.338153	0.51908	0.72635	-0.20727	2	40468548	R	II	SLC8A1	Body	opensea	Body-opens		rs1479865	28
cg2276313	0.218888	0.640545	10.55383	8.18E-06	0.000764	4.337412	0.531101	0.749989	-0.21889	4	1.41E+08	R	II	MAML3	Body	opensea	Body-opens		rs1490649	26;2
cg2738896	0.223104	0.55484	10.55191	8.19E-06	0.000765	4.336085	0.443288	0.666392	-0.2231	10	4705434	F	II	LOC10021(	Body	opensea	Body-opens			
cg0065844	0.24624	0.563775	10.55104	8.2E-06	0.000765	4.335482	0.440655	0.686895	-0.24624	19	39511868	R	II		IGR	opensea	IGR-opense		rs5320097	44
cg1057708	0.243901	0.619026	10.55062	8.2E-06	0.000765	4.33519	0.497075	0.740977	-0.2439	6	1.26E+08	R	II	TPD52L1	Body	opensea	Body-opens			
cg1822716	0.21344	0.628308	10.5499	8.2E-06	0.000765	4.334694	0.521588	0.735028	-0.21344	1	2.39E+08	F	II		IGR	opensea	IGR-opense		rs5511405	5;8
cg2009550	0.221786	0.569184	10.5477	8.21E-06	0.000766	4.333169	0.458291	0.680077	-0.22179	10	3027087	F	II		IGR	opensea	IGR-opense		rs1435156	6;41;49
cg2625158	0.219985	0.377948	10.54732	8.22E-06	0.000766	4.332901	0.267956	0.487941	-0.21998	7	11456440	R	II	THSD7A	Body	opensea	Body-opens		rs3710485	47;40;18;8
cg0953010	0.2353	0.626768	10.54707	8.22E-06	0.000766	4.332728	0.509118	0.744418	-0.2353	2	36586710	F	II	CRIM1	Body	shore	Body-shore	chr2:3658⁄		
cg0983779	0.205827	0.552091	10.54431	8.23E-06	0.000767	4.330815	0.449177	0.655005	-0.20583	3	82023856	F	II		IGR	opensea	IGR-opense		rs5575524	0;1;35
cg1406224	0.219461	0.539141	10.5439	8.24E-06	0.000767	4.330531	0.42941	0.648871	-0.21946	3	1.7E+08	R	II		IGR	opensea	IGR-opense		rs5714090	3
cg2701111	0.245771	0.540005	10.54311	8.24E-06	0.000767	4.329985	0.417119	0.66289	-0.24577	8	30179144	F	II		IGR	opensea	IGR-opense		rs5706757	4;22;25;35;47
cg0896230	0.227358	0.613714	10.54209	8.25E-06	0.000767	4.32928	0.500035	0.727393	-0.22736	3	46622647	R	II	LRRC2	TSS1500	shelf	TSS1500-sh	chr3:4661ℓ	rs5347370	48;47;29;23;20;1
cg0612614	0.206133	0.634056	10.5409	8.25E-06	0.000767	4.32845	0.530989	0.737122	-0.20613	14	51834564	F	II		IGR	opensea	IGR-opense		rs5336747	0;8;25
cg0157003	0.200496	0.543929	10.54011	8.26E-06	0.000768	4.327902	0.443681	0.644177	-0.2005	8	40226431	R	II		IGR	opensea	IGR-opense			
cg2642637	0.206101	0.494532	10.53802	8.27E-06	0.000768	4.326456	0.391481	0.597582	-0.2061	2	2.17E+08	R	II		IGR	opensea	IGR-opense			
cg2625413	0.212827	0.559948	10.53647	8.28E-06	0.000768	4.32538	0.453534	0.666361	-0.21283	2	69912852	F	II		IGR	opensea	IGR-opense		rs5510334	0;25;43
cg1196532	0.205646	0.425474	10.53375	8.29E-06	0.000769	4.323492	0.32265	0.528297	-0.20565	6	32997101	R	II		IGR	opensea	IGR-opense		rs1492649	1
cg1613077	0.206632	0.613987	10.53326	8.3E-06	0.000769	4.323152	0.510671	0.717303	-0.20663	12	94652584	F	II	PLXNC1	Body	opensea	Body-opens		rs7690655	1
cg0406541	0.227785	0.552346	10.53314	8.3E-06	0.000769	4.323071	0.438454	0.666238	-0.22778	3	62799139	R	II	CADPS	Body	opensea	Body-opens		rs5713159	45;31;19;7;2
cg2532078	0.21411	0.58024	10.53232	8.3E-06	0.00077	4.322501	0.473185	0.687295	-0.21411	1	1.7E+08	R	II	LINC01142	Body	opensea	Body-opens			
cg1989796	0.208723	0.542024	10.5323	8.3E-06	0.00077	4.322487	0.437663	0.646386	-0.20872	20	56416164	F	II		IGR	opensea	IGR-opense		rs7329123	1;15
cg0833475	0.206416	0.445828	10.53184	8.3E-06	0.00077	4.322164	0.34262	0.549035	-0.20642	13	1.03E+08	R	II	FGF14	Body	opensea	Body-opens		rs1452450	14;2
cg1664743	0.209632	0.454996	10.53125	8.31E-06	0.00077	4.321758	0.35018	0.559812	-0.20963	21	37025385	R	II		IGR	opensea	IGR-opense		rs5744716	43;1
cg0713661	0.219618	0.540969	10.53031	8.31E-06	0.00077	4.321107	0.43116	0.650778	-0.21962	2	1.61E+08	R	II		IGR	opensea	IGR-opense		rs1472762	2
cg1829692	0.21754	0.582117	10.52677	8.33E-06	0.000771	4.318644	0.473347	0.690887	-0.21754	1	1.01E+08	F	II	CDC14A	Body	opensea	Body-opens		rs1460256	10;17
cg1742535	0.202974	0.526452	10.52474	8.34E-06	0.000771	4.317237	0.424965	0.627939	-0.20297	11	1.11E+08	R	II		IGR	opensea	IGR-opense			
cg0458915	0.251647	0.562328	10.52297	8.35E-06	0.000772	4.316008	0.436504	0.688151	-0.25165	6	1.48E+08	F	II		IGR	opensea	IGR-opense		rs5291172	12;48
cg2480777	0.219414	0.551931	10.52222	8.36E-06	0.000772	4.315485	0.442225	0.661638	-0.21941	14	1.01E+08	R	II	SNORD113	TSS1500	opensea	TSS1500-op		rs3404079	22;11
cg2141059	0.236844	0.575544	10.52178	8.36E-06	0.000772	4.315176	0.457122	0.693967	-0.23684	17	42625246	R	II		IGR	opensea	IGR-opense		rs5636295	1
cg1981829	0.242208	0.567839	10.52111	8.36E-06	0.000772	4.314715	0.446735	0.688943	-0.24221	9	1.03E+08	R	II	INVS	Body	opensea	Body-opens		rs1840393	28;3
cg2396735	0.233762	0.565624	10.52012	8.37E-06	0.000772	4.314023	0.448743	0.682505	-0.23376	5	1.06E+08	R	II	LOC10246	Body	opensea	Body-opens		rs1924286	36;14
cg2431571	0.226048	0.55795	10.5199	8.37E-06	0.000772	4.31387	0.444926	0.670974	-0.22605	3	1.3E+08	R	II	COL29A1	3'UTR	opensea	3'UTR-oper			
cg2095731	0.259606	0.488598	10.51961	8.37E-06	0.000772	4.31367	0.358795	0.618401	-0.25961	2	38976409	F	II	SFRS7	Body	shore	Body-shore	chr2:3897		
cg0577856	0.216866	0.45335	10.51769	8.38E-06	0.000772	4.312334	0.344917	0.561782	-0.21687	3	1.73E+08	F	II	NLGN1	Body	opensea	Body-opens		rs1463579	1;50
cg1610290	0.216237	0.631983	10.51692	8.39E-06	0.000773	4.311803	0.523865	0.740102	-0.21624	15	85212333	R	II		IGR	opensea	IGR-opense		rs1397521	2;1
cg2308927	0.205007	0.500945	10.51689	8.39E-06	0.000773	4.311781	0.398442	0.603448	-0.20501	15	39985431	R	II	FSIP1	Body	opensea	Body-opens		rs5759349	8;1
cg1889061	0.205063	0.605589	10.51512	8.4E-06	0.000773	4.310546	0.503058	0.70812	-0.20506	1	1.92E+08	F	II		IGR	opensea	IGR-opense		rs5466387	1
cg1036590	0.223862	0.547125	10.51267	8.41E-06	0.000774	4.308843	0.435193	0.659056	-0.22386	11	78568009	R	II	ODZ4	Body	opensea	Body-opens		rs5317268	46;2
cg0609670	0.212419	0.488784	10.51081	8.42E-06	0.000774	4.307555	0.382575	0.594994	-0.21242	8	1.38E+08	R	II		IGR	opensea	IGR-opense			
cg1803359	0.213193	0.469564	10.51056	8.42E-06	0.000774	4.307377	0.362967	0.576161	-0.21319	11	75537636	F	II	UVRAG	Body	opensea	Body-opens		rs5485900	10
cg0692894	0.218686	0.591068	10.51005	8.43E-06	0.000774	4.307022	0.481725	0.70041	-0.21869	6	90023128	R	II	GABRR2	Body	opensea	Body-opens		rs1812250	44;32;22
cg0340324	0.241265	0.609307	10.50822	8.44E-06	0.000775	4.305749	0.488674	0.729939	-0.24126	3	1.95E+08	R	II		IGR	opensea	IGR-opense		rs1485277	37;25;12
cg0900416	0.201851	0.432817	10.50735	8.44E-06	0.000775	4.305143	0.331891	0.533743	-0.20185	1	17053983	F	II		IGR	shelf	IGR-shelf	chr1:1705	rs7556167	0
cg0177794	0.209875	0.466049	10.50729	8.44E-06	0.000775	4.305102	0.361111	0.570986	-0.20987	22	35248469	R	II		IGR	opensea	IGR-opense		rs1869058	18;4
cg1883338	0.258135	0.541525	10.50591	8.45E-06	0.000775	4.304142	0.412457	0.670592	-0.25813	1	10343921	F	II	KIF1B	Body	opensea	Body-opens		rs5416456	49
cg0535580	0.223749	0.613218	10.4997	8.49E-06	0.000777	4.299818	0.501344	0.725093	-0.22375	1	55088543	F	II	FAM151A	Body	opensea	Body-opens		rs1618859	27;43
cg1577649	0.222581	0.444926	10.49914	8.49E-06	0.000777	4.299431	0.333636	0.556217	-0.22258	16	89428646	F	II	ANKRD11	Body	opensea	Body-opens		rs5306578	9;49
cg2460485	0.209264	0.603959	10.4978	8.5E-06	0.000777	4.298496	0.499327	0.708591	-0.20926	7	35762816	F	II		IGR	opensea	IGR-opense		rs5738899	14;42
cg0999443	0.200081	0.615149	10.49562	8.51E-06	0.000778	4.296977	0.515109	0.71519	-0.20008	20	62404778	F	II	ZBTB46	Body	shore	Body-shore	chr20:624(		
cg0183050	0.212867	0.504625	10.49122	8.54E-06	0.000779	4.293909	0.398191	0.611058	-0.21287	5	1.41E+08	F	II		IGR	opensea	IGR-opense			
cg0273598	0.213863	0.488405	10.49093	8.54E-06	0.000779	4.293707	0.381474	0.595337	-0.21386	20	11125380	R	II		IGR	opensea	IGR-opense		rs5452027	45;33;20;2
cg0762620	0.21659	0.481203	10.48885	8.55E-06	0.000779	4.292258	0.372908	0.589498	-0.21659	7	55200657	F	II	EGFR	Body	opensea	Body-opens		rs7787752	0;44
cg1388154	0.223147	0.575344	10.48865	8.55E-06	0.000779	4.292118	0.46377	0.686917	-0.22315	17	40545151	F	II		IGR	opensea	IGR-opense		rs5377535	15;16;17
cg2443075	0.204167	0.594474	10.48801	8.56E-06	0.000779	4.291676	0.492391	0.696558	-0.20417	2	2.32E+08	F	II	NMUR1	TSS1500	shore	TSS1500-sh	chr2:2323	rs5545105	1;7
cg1585803	0.208252	0.507352	10.48775	8.56E-06	0.000779	4.291493	0.403226	0.611478	-0.20825	12	54978014	F	II	PPP1R1A	Body	shelf	Body-shelf	chr12:549(	rs1996585	1
cg2489933	0.278219	0.453643	10.48729	8.56E-06	0.000779	4.291174	0.314533	0.592752	-0.27822	3	1.68E+08	F	II	GOLIM4	Body	opensea	Body-opens		rs5725839	31
cg0738930	0.259748	0.497733	10.48648	8.56E-06	0.00078	4.290608	0.367859	0.627607	-0.25975	2	2.32E+08	F	II		IGR	opensea	IGR-opense		rs3702063	8;36;41
cg0711592	0.21624	0.63943	10.48529	8.57E-06	0.00078	4.289778	0.53131	0.74755	-0.21624	7	30295722	R	II		IGR	opensea	IGR-opense		rs5713368	41;20
cg2558710	0.223616	0.573994	10.48357	8.58E-06	0.00078	4.288577	0.462186	0.685802	-0.22362	17	68078868	F	II	KCNJ16	5'UTR	opensea	5'UTR-oper		rs2158970	18
cg2601248	0.221642	0.614885	10.48244	8.59E-06	0.000781	4.287791	0.504064	0.725706	-0.22164	2	8423155	F	II		IGR	opensea	IGR-opense		rs5341908	1
cg2547689	0.214854	0.460566	10.48164	8.59E-06	0.000781	4.287232	0.353139	0.567994	-0.21485	13	34335944	R	II		IGR	opensea	IGR-opense		rs1486120	38;24
cg1872872	0.212711	0.612075	10.48117	8.6E-06	0.000781	4.286903	0.50572	0.718431	-0.21271	1	2E+08	F	II		IGR	opensea	IGR-opense		rs1401983	0
cg2693160	0.203556	0.372243	10.48109	8.6E-06	0.000781	4.286844	0.270465	0.474021	-0.20356	1	67030196	F	II	SGIP1	Body	opensea	Body-opens		rs5410696	28;44;48
cg1079838	0.219489	0.490235	10.48075	8.6E-06	0.000781	4.286607	0.380491	0.59998	-0.21949	17	10325221	R	II	MYH8	5'UTR	opensea	5'UTR-oper			
cg0554349	0.265042	0.59754	10.48057	8.6E-06	0.000781	4.286481	0.465019	0.730061	-0.26504	2	1.51E+08	R	II		IGR	opensea	IGR-opense			
cg2400067	0.206321	0.566495	10.4803	8.6E-06	0.000781	4.286294	0.463335	0.669656	-0.20632	9	1.17E+08	F	II		IGR	opensea	IGR-opense		rs5648299	0;1
cg2390252	0.201128	0.656524	10.47839	8.61E-06	0.000781	4.284965	0.555959	0.757088	-0.20113	10	55892708	F	II	PCDH15	Body	opensea	Body-opens		rs1391047	1;7;8
cg0270138	0.216931	0.676274	10.47723	8.62E-06	0.000782	4.284152	0.567808	0.784739	-0.21693	21	17798271	R	II	MIR99AHG	Body	opensea	Body-opens		rs5363924	1
cg0221173	0.201525	0.480733	10.4746	8.63E-06	0.000782	4.282317	0.37997	0.581495	-0.20153	11	5142602	R	II	OR52A4	1stExon	opensea	1stExon-op		rs1479478	39
cg0087500	0.214429	0.537979	10.47376	8.64E-06	0.000782	4.281731	0.430764	0.645193	-0.21443	6	16657919	F	II	ATXN1	5'UTR	opensea	5'UTR-oper		rs5290082	12;18
cg1470355	0.230477	0.495974	10.4726	8.65E-06	0.000782	4.280923	0.380735	0.611212	-0.23048	1	48670405	F	II		IGR	opensea	IGR-opense		rs1390769	1;4;10;48;49
cg1455596	0.222871	0.512583	10.47248	8.65E-06	0.000782	4.280841	0.401147	0.624018	-0.22287	2	1.98E+08	R	II	RFTN2	Body	opensea	Body-opens		rs1189965	19
cg2707937	0.223464	0.478318	10.47158	8.65E-06	0.000782	4.280207	0.366586	0.59005	-0.22346	21	19165713	F	II	C21orf91	3'UTR	opensea	3'UTR-oper		rs5332645	0;41
cg1111353	0.255712	0.564748	10.47082	8.66E-06	0.000782	4.279676	0.436892	0.692604	-0.25571	11	1.16E+08	R	II		IGR	opensea	IGR-opense		rs1262591	43;42
cg0867000	0.226635	0.579806	10.46948	8.66E-06	0.000782	4.27874	0.466489	0.693124	-0.22663	14	56738739	R	II	PELI2	Body	opensea	Body-opens		rs3769656	2
cg2537369	0.208672	0.567774	10.46935	8.67E-06	0.000782	4.278653	0.463438	0.67211	-0.20867	9	33236792	R	II		IGR	opensea	IGR-opense		rs1885560	29;4;2
cg1290696	0.204651	0.400778	10.46875	8.67E-06	0.000782	4.278236	0.298452	0.503103	-0.20465	3	3807867	R	II		IGR	opensea	IGR-opense		rs5648868	1
cg2170532	0.201944	0.559899	10.4673	8.68E-06	0.000783	4.277219	0.458927	0.660871	-0.20194	4	24200012	R	II		IGR	opensea	IGR-opense		rs5483907	40;21
cg0811669	0.212138	0.511246	10.46704	8.68E-06	0.000783	4.277036	0.405177	0.617315	-0.21214	4	77675652	F	II	SHROOM3	Body	opensea	Body-opens		rs1431798	0;1;13;21;29
cg1017779	0.200841	0.515627	10.46618	8.68E-06	0.000783	4.276436	0.415207	0.616048	-0.20084	13	78180325	F	II	SCEL-AS1	Body	opensea	Body-opens		rs5609414	4;39
cg2753643	0.201163	0.638112	10.46592	8.69E-06	0.000783	4.276257	0.537531	0.738693	-0.20116	20	34713354	R	II	EPB41L1	ExonBnd	opensea	ExonBnd-op		rs1897719	38;23
cg2101774	0.236299	0.616272	10.46547	8.69E-06	0.000783	4.275943	0.498123	0.734421	-0.2363	4	1.6E+08	F	II	FNIP2	Body	opensea	Body-opens			
cg0062310	0.222665	0.68408	10.46492	8.69E-06	0.000783	4.275559	0.572748	0.795413	-0.22266	3	1.93E+08	F	II	ATP13A5	Body	opensea	Body-opens		rs5633467	40;46
cg0736249	0.203133	0.559164	10.46141	8.71E-06	0.000784	4.273104	0.457597	0.66073	-0.20313	1	2.33E+08	R	II		IGR	opensea	IGR-opense		rs5278525	9;8
cg1935952	0.231289	0.609563	10.46008	8.72E-06	0.000784	4.272175	0.493919	0.725207	-0.23129	16	31414218	F	II	ITGAD	Body	opensea	Body-opens		rs5619104	39
cg1352743	0.213415	0.663705	10.46006	8.72E-06	0.000784	4.272157	0.556997	0.770412	-0.21341	17	17304659	R	II		IGR	opensea	IGR-opense		rs5720626	36
cg1773312	0.234606	0.530788	10.45882	8.73E-06	0.000784	4.271295	0.413485	0.648091	-0.23461	2	24807724	R	II	NCOA1	5'UTR	opensea	5'UTR-oper		rs5461710	14
cg1756034	0.242265	0.46818	10.45878	8.73E-06	0.000784	4.271262	0.347048	0.589313	-0.24227	17	684490	F	II	RNMTL1	TSS1500	shore	TSS1500-sh	chr17:685	rs7397552	7;31;44
cg0609580	0.217327	0.605556	10.45867	8.73E-06	0.000784	4.27119	0.496892	0.714219	-0.21733	11	1.03E+08	R	II		IGR	opensea	IGR-opense		rs5452979	1
cg1888229	0.235307	0.626881	10.45705	8.74E-06	0.000785	4.270052	0.509228	0.744535	-0.23531	7	83674657	F	II	SEMA3A	Body	opensea	Body-opens			
cg1201711	0.229315	0.623566	10.45564	8.75E-06	0.000785	4.269072	0.508909	0.738223	-0.22931	9	90271285	R	II	DAPK1	Body	shelf	Body-shelf	chr9:9027⁄	rs5538890	26;11
cg0763755	0.208957	0.614729	10.45467	8.75E-06	0.000785	4.268393	0.510251	0.719208	-0.20896	6	32179971	R	II	NOTCH4	Body	opensea	Body-opens		rs5409355	21;2;1
cg1819086	0.219778	0.638422	10.45363	8.76E-06	0.000785	4.267664	0.528533	0.748311	-0.21978	12	52689098	R	II		IGR	shelf	IGR-shelf	chr12:526		
cg1228909	0.240625	0.539348	10.45313	8.76E-06	0.000785	4.267316	0.419036	0.659661	-0.24062	2	97009820	R	II	NCAPH	Body	opensea	Body-opens		rs5763965	10;2
cg0997423	0.222776	0.641188	10.45246	8.77E-06	0.000785	4.266842	0.5298	0.752576	-0.22278	10	1.02E+08	F	II	ABCC2	Body	opensea	Body-opens		rs5671642	1;5;29
cg2044078	0.271148	0.618682	10.45243	8.77E-06	0.000785	4.266826	0.483108	0.754256	-0.27115	6	1.09E+08	F	II		IGR	opensea	IGR-opense		rs5453437	7;42
cg0082960	0.23428	0.602483	10.45179	8.77E-06	0.000785	4.266374	0.485343	0.719623	-0.23428	16	84678923	F	II		IGR	shelf	IGR-shelf	chr16:846(	rs5322773	9;15;26
cg0091978	0.218152	0.446518	10.4514	8.77E-06	0.000785	4.2661	0.337442	0.555594	-0.21815	7	1.22E+08	R	II	CADPS2	Body	opensea	Body-opens		rs5431863	0;12
cg2063510	0.225851	0.646775	10.45027	8.78E-06	0.000785	4.265308	0.533849	0.7597	-0.22585	12	26578619	F	II	ITPR2	Body	opensea	Body-opens		rs5298000	0;18
cg2755654	0.234153	0.529826	10.44499	8.81E-06	0.000787	4.261612	0.412749	0.646902	-0.23415	19	44846179	R	II	ZNF112	Body	opensea	Body-opens		rs1171690	26;8
cg1415532	0.241929	0.525649	10.44448	8.81E-06	0.000787	4.261256	0.404685	0.646614	-0.24193	4	87235054	R	II	MAPK10	5'UTR	opensea	5'UTR-oper		rs1814814	12
cg0160408	0.217274	0.524991	10.44219	8.83E-06	0.000787	4.259657	0.416354	0.633627	-0.21727	6	88446851	F	II		IGR	opensea	IGR-opense		rs5748027	1;6
cg2630424	0.252989	0.433761	10.44166	8.83E-06	0.000788	4.259285	0.307267	0.560256	-0.25299	8	62288433	R	II	CLVS1	Body	opensea	Body-opens		rs5630298	30;1

cg1984494	0.228973	0.656158	10.44153	8.83E-06	0.000788	4.259191	0.541671	0.770644	-0.22897	2	2.01E+08	R	II	TYW5	Body	opensea	Body-opens		
cg0897166	0.20999	0.619051	10.43806	8.85E-06	0.000789	4.256764	0.514056	0.724047	-0.20999	7	79087104	R	II	MAGI2-AS3	Body	shelf	Body-shelf	chr7:79082	
cg2592925	0.256285	0.508805	10.43664	8.86E-06	0.000789	4.255769	0.380663	0.636948	-0.25629	4	36211731	F	II	ARAP2	Body	opensea	Body-opens		rs3679273 0;1;36
cg1265080	0.214473	0.595364	10.43566	8.87E-06	0.000789	4.255082	0.488128	0.7026	-0.21447	7	81662291	F	II	CACNA2D1	Body	opensea	Body-opens		rs1895659 32;45
cg1038472	0.239336	0.512812	10.43554	8.87E-06	0.000789	4.254992	0.393144	0.63248	-0.23934	11	63035103	F	II		IGR	opensea	IGR-opense		rs1121841 31
cg1905121	0.221698	0.49653	10.43387	8.88E-06	0.000789	4.253828	0.385681	0.607379	-0.2217	2	1.28E+08	R	I	IWS1	Body	shelf	Body-shelf	chr2:12828	rs2017355 4
cg2155418	0.249907	0.538122	10.43313	8.88E-06	0.00079	4.253305	0.413168	0.663076	-0.24991	1	1.16E+08	R	II	CASQ2	Body	opensea	Body-opens		
cg2689540	0.218	0.586436	10.43195	8.89E-06	0.00079	4.252478	0.477436	0.695436	-0.218	11	35941694	F	II		IGR	opensea	IGR-opense		rs5724710 1;5;26
cg1792675	0.204175	0.518693	10.43186	8.89E-06	0.00079	4.252412	0.416605	0.620781	-0.20418	3	55217960	F	II		IGR	opensea	IGR-opense		rs1482264 7;23
cg1845352	0.230013	0.583973	10.43157	8.89E-06	0.00079	4.252209	0.468967	0.69898	-0.23001	1	1.59E+08	R	II	OR6K2	TSS1500	opensea	TSS1500-op		rs2023613 45;24;5
cg0001330	0.203647	0.63354	10.42834	8.91E-06	0.00079	4.249947	0.531717	0.735364	-0.20365	3	1.5E+08	F	II		IGR	opensea	IGR-opense		rs5306522 0;1;38
cg1761148	0.214212	0.54879	10.4283	8.91E-06	0.00079	4.249922	0.441684	0.655896	-0.21421	4	1.11E+08	F	II	EGF	Body	opensea	Body-opens		rs3723039 13;15;28;29;44
cg1235228	0.22836	0.602652	10.4279	8.92E-06	0.00079	4.249638	0.488472	0.716832	-0.22836	8	97744293	F	II	CPQ	5'UTR	opensea	5'UTR-oper		rs5649052 15
cg0340756	0.256303	0.580308	10.42623	8.93E-06	0.00079	4.248467	0.452157	0.708459	-0.2563	8	1682921	R	II		IGR	opensea	IGR-opense		rs5464896 50;47;37;35
cg1924952	0.204784	0.60372	10.42487	8.93E-06	0.00079	4.247512	0.501328	0.706112	-0.20478	15	33029102	R	II		IGR	opensea	IGR-opense		
cg0368190	0.243181	0.583285	10.42487	8.93E-06	0.00079	4.247511	0.461695	0.704876	-0.24318	4	1.59E+08	F	II		IGR	opensea	IGR-opense		
cg1858166	0.207516	0.649764	10.42407	8.94E-06	0.00079	4.246953	0.546007	0.753522	-0.20752	7	29873076	F	II	WIPF3	TSS1500	opensea	TSS1500-op		
cg1069586	0.200434	0.482318	10.42328	8.94E-06	0.00079	4.246396	0.382101	0.582535	-0.20043	13	33954699	F	II	STARD13	Body	opensea	Body-opens		rs1426363 0;48;50
cg1376087	0.219159	0.464084	10.42241	8.95E-06	0.00079	4.24579	0.354505	0.573664	-0.21916	3	69815765	F	II	MITF	Body	opensea	Body-opens		rs5474510 0;47
cg0732657	0.240524	0.578832	10.4224	8.95E-06	0.00079	4.245779	0.45857	0.699094	-0.24052	7	51292092	F	II	COBL	Body	opensea	Body-opens		rs5325896 0
cg1730821	0.206793	0.605625	10.42186	8.95E-06	0.00079	4.245403	0.502228	0.709021	-0.20679	14	59642351	R	II		IGR	opensea	IGR-opense		
cg1201923	0.216956	0.645893	10.42129	8.96E-06	0.000791	4.244999	0.537415	0.754371	-0.21696	11	12628283	F	II		IGR	opensea	IGR-opense		rs5342089 39
cg2096612	0.221937	0.626674	10.42036	8.96E-06	0.000791	4.244347	0.515706	0.737643	-0.22194	11	93469940	R	II	TAF1D	Body	opensea	Body-opens		rs3459161 50;34;1
cg1107944	0.230319	0.512121	10.42009	8.96E-06	0.000791	4.24416	0.396962	0.627281	-0.23032	8	1.31E+08	F	II	ASAP1	5'UTR	opensea	5'UTR-oper		rs5293081 15;30;37;40
cg1954714	0.243302	0.590004	10.41982	8.97E-06	0.000791	4.243969	0.468353	0.711655	-0.2433	11	1.18E+08	F	II	MLL	Body	opensea	Body-opens		
cg1511864	0.221138	0.655312	10.41913	8.97E-06	0.000791	4.243481	0.544743	0.765881	-0.22114	22	50602187	R	II		IGR	shelf	IGR-shelf	chr22:5060	rs5740809 19;15
cg0161364	0.247161	0.496673	10.419	8.97E-06	0.000791	4.243392	0.373093	0.620254	-0.24716	10	28271425	R	II	ARMC4	TSS1500	opensea	TSS1500-op		rs5310922 51;23;1
cg0624651	0.25239	0.451976	10.41818	8.98E-06	0.000791	4.242821	0.325781	0.578171	-0.25239	8	1.02E+08	R	II		IGR	opensea	IGR-opense		rs5723783 48;42;39
cg1628319	0.207134	0.674403	10.41816	8.98E-06	0.000791	4.242804	0.570836	0.77797	-0.20713	5	73929025	F	II	ENC1	3'UTR	opensea	3'UTR-oper		
cg0546756	0.212226	0.647741	10.41778	8.98E-06	0.000791	4.242534	0.541628	0.753854	-0.21223	7	90595609	R	II	CDK14	Body	opensea	Body-opens		rs5537235 34;7;1
cg0471725	0.200552	0.647051	10.4154	8.99E-06	0.000791	4.240863	0.546775	0.747327	-0.20055	5	1.39E+08	R	II		IGR	opensea	IGR-opense		rs5580758 21
cg0499070	0.207674	0.345822	10.41536	8.99E-06	0.000791	4.240837	0.241985	0.449659	-0.20767	10	1.28E+08	R	II		IGR	opensea	IGR-opense		rs5749146 40
cg1750065	0.226701	0.539396	10.41461	9E-06	0.000791	4.240309	0.426045	0.652747	-0.2267	5	58145887	R	II	RAB3C	Body	opensea	Body-opens		rs5618114 36
cg0244114	0.209443	0.587143	10.41194	9.01E-06	0.000792	4.238438	0.482422	0.691864	-0.20944	13	1.07E+08	R	II	EFNB2	Body	opensea	Body-opens		rs7411303 51;2;1
cg2176672	0.200497	0.603951	10.41146	9.02E-06	0.000792	4.238098	0.503702	0.704199	-0.2005	4	1.83E+08	R	II		IGR	opensea	IGR-opense		rs1491020 1
cg1674421	0.253521	0.55346	10.41125	9.02E-06	0.000792	4.237949	0.426699	0.68022	-0.25352	14	49872206	F	II		IGR	opensea	IGR-opense		rs1500094 1;8
cg2445764	0.231773	0.502161	10.41122	9.02E-06	0.000792	4.237926	0.386274	0.618047	-0.23177	1	64668350	R	II	UBE2U	TSS1500	opensea	TSS1500-op		rs1417404 48;45;45;14;2
cg1812171	0.255367	0.487571	10.40939	9.03E-06	0.000793	4.236645	0.359888	0.615255	-0.25537	2	17738759	F	II	VSNL1	5'UTR	opensea	5'UTR-oper		rs5559629 0;8;15;32
cg1882710	0.207778	0.636021	10.40879	9.03E-06	0.000793	4.236223	0.532132	0.73991	-0.20778	12	86230957	F	II	RASSF9	TSS1500	opensea	TSS1500-op		rs1385821 7;17
cg1915486	0.216299	0.636049	10.40857	9.03E-06	0.000793	4.236065	0.5279	0.744199	-0.2163	1	1.54E+08	R	II	NUP210L	Body	opensea	Body-opens		rs5683381 42;1
cg0970454	0.20717	0.566549	10.40826	9.04E-06	0.000793	4.235852	0.462964	0.670134	-0.20717	15	85616433	R	II	PDE8A	Body	opensea	Body-opens		rs3724782 39;19
cg0110938	0.237824	0.452971	10.40758	9.04E-06	0.000793	4.235369	0.334059	0.571883	-0.23782	21	34302076	R	II		IGR	opensea	IGR-opense		rs1818884 2
cg0242657	0.206865	0.623281	10.40677	9.05E-06	0.000793	4.234801	0.519849	0.726713	-0.20686	12	60205342	R	II		IGR	opensea	IGR-opense		rs5584549 31
cg1409865	0.215502	0.457721	10.4054	9.05E-06	0.000794	4.233839	0.349971	0.565472	-0.2155	1	1.12E+08	F	II	KCND3-AS1	TSS1500	opensea	TSS1500-op		rs5345913 1;35;36;43;46
cg0959040	0.217516	0.612684	10.40433	9.06E-06	0.000794	4.233086	0.503926	0.721442	-0.21752	2	2.19E+08	R	I	VIL1	5'UTR	opensea	5'UTR-oper		
cg2058886	0.217203	0.600113	10.40428	9.06E-06	0.000794	4.233049	0.491511	0.708714	-0.2172	10	95551111	R	II	LGI1	Body	opensea	Body-opens		rs5388509 36;2;1
cg0637296	0.240795	0.584118	10.40401	9.06E-06	0.000794	4.232863	0.463721	0.704516	-0.2408	1	43351860	R	II		IGR	shore	IGR-shore	chr1:4335	rs1922801 1
cg0469308	0.226944	0.619762	10.40398	9.06E-06	0.000794	4.232844	0.50629	0.733233	-0.22694	3	87383039	F	II		IGR	opensea	IGR-opense		rs1923906 0;38
cg1215447	0.211874	0.680284	10.40395	9.06E-06	0.000794	4.23282	0.574347	0.786221	-0.21187	11	68196085	R	II	LRP5	Body	shelf	Body-shelf	chr11:681	rs3679333 44
cg0393557	0.210609	0.62967	10.40314	9.07E-06	0.000794	4.232253	0.524365	0.734975	-0.21061	1	72035562	R	II	NEGR1	Body	opensea	Body-opens		rs5596504 11;9
cg1595193	0.205941	0.657875	10.40303	9.07E-06	0.000794	4.23217	0.554905	0.760846	-0.20594	16	19819871	R	II	IQCK	Body	opensea	Body-opens		
cg1316439	0.214507	0.575758	10.40302	9.07E-06	0.000794	4.232169	0.468505	0.683011	-0.21451	1	1.81E+08	F	II		IGR	shelf	IGR-shelf	chr1:1808	rs1848585 15
cg1610507	0.227094	0.470045	10.4029	9.07E-06	0.000794	4.232082	0.356497	0.583592	-0.22709	2	1.68E+08	R	II	XIRP2	5'UTR	opensea	5'UTR-oper		rs5772581 37;18
cg0473385	0.211708	0.582217	10.40204	9.08E-06	0.000794	4.23148	0.476363	0.688071	-0.21171	3	24961439	F	II		IGR	opensea	IGR-opense		rs1856269 8;11;35
cg2156702	0.213163	0.535121	10.40015	9.09E-06	0.000795	4.230147	0.428539	0.641703	-0.21316	11	93067324	F	II	CCDC67	Body	shelf	Body-shelf	chr11:930	rs1173211 6;20;31
cg0726797	0.276349	0.462064	10.39983	9.09E-06	0.000795	4.229919	0.32389	0.600239	-0.27635	8	62539945	F	II	ASPH	Body	opensea	Body-opens		rs1920400 0;24;27;51
cg1948522	0.229855	0.599264	10.39904	9.09E-06	0.000795	4.229366	0.484336	0.714192	-0.22986	1	68216572	R	II	GNG12	5'UTR	opensea	5'UTR-oper		rs7787932 43;1
cg0681745	0.2241	0.549853	10.39893	9.1E-06	0.000795	4.229288	0.437803	0.661903	-0.2241	1	48360454	F	II		IGR	opensea	IGR-opense		rs5758648 0;1;33;46
cg2281027	0.200764	0.635378	10.39841	9.1E-06	0.000795	4.228924	0.534996	0.73576	-0.20076	20	37899981	R	II		IGR	opensea	IGR-opense		rs1512108 44;14;2;1
cg2510667	0.259381	0.614207	10.39783	9.1E-06	0.000795	4.228518	0.484516	0.743897	-0.25938	21	17452671	R	II	MIR99AHG	Body	opensea	Body-opens		
cg0874755	0.219205	0.595434	10.39545	9.12E-06	0.000796	4.226844	0.485831	0.705036	-0.2192	11	1.03E+08	F	II	MMP8	3'UTR	opensea	3'UTR-oper		rs1422861 0;1;36
cg0515504	0.207728	0.60159	10.39424	9.12E-06	0.000797	4.225993	0.497726	0.705454	-0.20773	5	168719	F	II	PLEKHG4B	Body	shore	Body-shore	chr5:1695	rs5488733 1;18;19
cg0565892	0.266903	0.539845	10.39347	9.13E-06	0.000797	4.225447	0.406393	0.673296	-0.2669	7	13191068	R	II		IGR	opensea	IGR-opense		rs5354259 20
cg1734156	0.237335	0.486657	10.39321	9.13E-06	0.000797	4.225263	0.367989	0.605325	-0.23734	2	2.16E+08	R	II	LINC00607	Body	opensea	Body-opens		rs5580152 46;40;39;37;27;6
cg0795913	0.20602	0.611966	10.39312	9.13E-06	0.000797	4.225204	0.508956	0.714976	-0.20602	1	87601970	F	II	LOC339524	Body	shelf	Body-shelf	chr1:8759	rs5327347 5;10;26
cg2300904	0.235442	0.393837	10.39219	9.14E-06	0.000797	4.224547	0.276116	0.511558	-0.23544	3	47603786	F	II	CSPG5	3'UTR	opensea	3'UTR-oper		rs5600080 14
cg1908424	0.203828	0.648327	10.39202	9.14E-06	0.000797	4.224428	0.546413	0.750241	-0.20383	18	9835632	F	II	RAB31	Body	opensea	Body-opens		rs1404911 3;41
cg1648925	0.223405	0.523847	10.39102	9.14E-06	0.000797	4.223721	0.412145	0.63555	-0.22341	10	32438418	R	II		IGR	opensea	IGR-opense		rs7628711 42;26;25;1
cg0295698	0.231084	0.678087	10.38836	9.16E-06	0.000798	4.22185	0.562546	0.793629	-0.23108	1	74929125	F	II	TNNI3K	ExonBnd	opensea	ExonBnd-o		rs1458406 1;6;7;21;26;41;45
cg0594566	0.211758	0.512084	10.3864	9.17E-06	0.000798	4.220469	0.406205	0.617963	-0.21176	5	1.54E+08	F	II		IGR	shelf	IGR-shelf	chr5:1539	
cg1583477	0.271795	0.500053	10.38544	9.18E-06	0.000799	4.219795	0.364156	0.635951	-0.2718	17	43919576	R	II		IGR	opensea	IGR-opense		
cg2222444	0.247159	0.554832	10.38348	9.19E-06	0.000799	4.218412	0.431252	0.678411	-0.24716	2	1.88E+08	F	II		IGR	opensea	IGR-opense		rs1415868 1;5;29
cg2245431	0.242918	0.544629	10.38094	9.21E-06	0.0008	4.216622	0.42317	0.666088	-0.24292	1	2.02E+08	R	II	NAV1	Body	opensea	Body-opens		rs5676465 49;35
cg1206124	0.238153	0.544871	10.38062	9.21E-06	0.000801	4.2164	0.425795	0.663948	-0.23815	3	1.26E+08	R	II		IGR	opensea	IGR-opense		
cg2535716	0.215188	0.713511	10.37994	9.22E-06	0.000801	4.215917	0.605917	0.821105	-0.21519	1	39323275	F	II	RRAGC	Body	shore	Body-shore	chr1:3932	rs5501648 10;24
cg1205674	0.22526	0.598238	10.37957	9.22E-06	0.000801	4.215656	0.485608	0.710868	-0.22526	5	59786348	R	II	PART1	Body	opensea	Body-opens		
cg1614170	0.222701	0.562034	10.37942	9.22E-06	0.000801	4.215551	0.450683	0.673384	-0.2227	15	25651598	R	II	UBE3A	TSS1500	opensea	TSS1500-op		
cg0088449	0.208402	0.64905	10.37874	9.22E-06	0.000801	4.215073	0.544848	0.753251	-0.2084	7	6245952	F	II	CYTH3	Body	opensea	Body-opens		rs1827325 41;44
cg0531364	0.208811	0.543463	10.37734	9.23E-06	0.000801	4.214089	0.439058	0.647868	-0.20881	17	65027466	R	II	CACNG4	3'UTR	opensea	3'UTR-oper		rs5720962 37;17
cg0274071	0.247678	0.491949	10.37673	9.24E-06	0.000801	4.213659	0.368109	0.615788	-0.24768	5	1.51E+08	R	II	SLC36A1	TSS1500	opensea	TSS1500-op		rs1390628 0;44;47
cg2743881	0.2235	0.430346	10.37638	9.24E-06	0.000801	4.213409	0.318596	0.542096	-0.2235	12	1.08E+08	F	II		IGR	opensea	IGR-opense		
cg0522884	0.243626	0.480345	10.37619	9.24E-06	0.000801	4.213274	0.358533	0.602158	-0.24363	14	55925656	R	II		IGR	opensea	IGR-opense		rs1014008 29;23;2
cg2197484	0.223515	0.636154	10.37577	9.24E-06	0.000801	4.212981	0.524396	0.747911	-0.22352	1	1.93E+08	R	II		IGR	opensea	IGR-opense		rs5773605 4
cg0389455	0.216156	0.623549	10.37384	9.25E-06	0.000802	4.21162	0.515471	0.731627	-0.21616	15	52851392	R	II	ARPP19	Body	opensea	Body-opens		rs5286399 44;25;1
cg0305872	0.202201	0.506325	10.37234	9.26E-06	0.000802	4.210563	0.405225	0.607426	-0.2022	11	1.21E+08	F	II	TECTA	Body	opensea	Body-opens		rs1384975 5;39;43
cg2225642	0.209568	0.593417	10.3718	9.27E-06	0.000802	4.210179	0.488633	0.698201	-0.20957	10	4060366	F	II		IGR	opensea	IGR-opense		rs5768434 22;48
cg0157857	0.233382	0.533787	10.37092	9.27E-06	0.000802	4.209559	0.417096	0.650478	-0.23338	5	1.73E+08	R	II		IGR	opensea	IGR-opense		rs5516099 15
cg0972055	0.20276	0.495996	10.36771	9.29E-06	0.000803	4.207297	0.394616	0.597376	-0.20276	2	1.76E+08	F	II	ATF2	Body	opensea	Body-opens		rs1152091 0;35
cg0438079	0.203702	0.508174	10.36691	9.3E-06	0.000803	4.206727	0.406323	0.610025	-0.2037	1	61664754	F	II	NFIA	Body	opensea	Body-opens		rs5435736 34;41
cg0755235	0.238947	0.494839	10.36495	9.31E-06	0.000804	4.205344	0.375365	0.614312	-0.23895	4	90226782	R	II	GPRIN3	5'UTR	shore	5'UTR-shor	chr4:9022	rs7608871 25;9
cg1270040	0.220216	0.561139	10.36494	9.31E-06	0.000804	4.205341	0.451032	0.671247	-0.22022	10	1.04E+08	F	II	ARL3	Body	shelf	Body-shelf	chr10:104	rs5714780 18
cg1643564	0.230298	0.404614	10.36433	9.31E-06	0.000804	4.204912	0.289464	0.519763	-0.2303	6	1.28E+08	R	II	THEMIS	5'UTR	opensea	5'UTR-oper		rs5667728 19
cg1654318	0.220036	0.558992	10.36376	9.32E-06	0.000804	4.20451	0.448974	0.66901	-0.22004	21	35874035	R	II	KCNE1	5'UTR	opensea	5'UTR-oper		rs5655336 2
cg2165055	0.205284	0.441063	10.36367	9.32E-06	0.000804	4.204439	0.338421	0.543705	-0.20528	3	1.58E+08	F	II		IGR	opensea	IGR-opense		rs1431706 19;30
cg2654637	0.2235	0.54827	10.36311	9.32E-06	0.000804	4.204048	0.436519	0.66002	-0.2235	5	1.54E+08	R	II	LARP1	Body	opensea	Body-opens		rs1891514 35
cg1689043	0.242462	0.462712	10.36285	9.32E-06	0.000804	4.203867	0.341482	0.583943	-0.24246	5	1.49E+08	R	II	AFAP1L1	Body	opensea	Body-opens		rs1444141 17;2
cg0615260	0.209384	0.562272	10.36198	9.33E-06	0.000804	4.203252	0.45758	0.666965	-0.20938	17	80444748	F	II	NARF	Body	opensea	Body-opens		rs1886721 0
cg0481378	0.215431	0.499271	10.35963	9.35E-06	0.000805	4.201587	0.391556	0.606986	-0.21543	6	1.68E+08	R	II		IGR	opensea	IGR-opense		rs7493573 47;23
cg1655372	0.226348	0.464796	10.35878	9.35E-06	0.000805	4.200991	0.351622	0.57797	-0.22635	19	6668742	F	II	TNFSF14	Body	opensea	Body-opens		rs5647949 6
cg1913932	0.251929	0.534318	10.35751	9.36E-06	0.000805	4.200093	0.408354	0.660282	-0.25193	2	2.18E+08	R	II		IGR	opensea	IGR-opense		
cg2303375	0.227698	0.504684	10.35667	9.36E-06	0.000806	4.199502	0.390835	0.618533	-0.2277	1	65062243	F	II	CACHD1	Body	opensea	Body-opens		
cg1823321	0.202959	0.698888	10.35554	9.37E-06	0.000806	4.198699	0.597409	0.800368	-0.20296	13	73687268	R	II		IGR	opensea	IGR-opense		rs1143369 30;21
cg1041531	0.246422	0.615818	10.35375	9.38E-06	0.000807	4.197438	0.492607	0.739029	-0.24642	14	33949227	R	II	NPAS3	Body	opensea	Body-opens		rs5294392 39;19
cg2504587	0.238733	0.603948	10.35342	9.39E-06	0.000807	4.197203	0.484581	0.723314	-0.23873	21	18188931	F	II		IGR	opensea	IGR-opense		rs5672681 4;7;43;45
cg0287838	0.243345	0.518198	10.35045	9.4E-06	0.000807	4.195108	0.396526	0.639871	-0.24334	4	1.68E+08	R	II	SPOCK3	5'UTR	opensea	5'UTR-oper		rs5707342 2
cg0866300	0.224707	0.539846	10.34999	9.41E-06	0.000808	4.194781	0.427493	0.652199	-0.22471	1	1.17E+08	R	II		IGR	opensea	IGR-opense		rs5288995 19;45;49
cg2497848	0.216889	0.62718	10.34943	9.41E-06	0.000808	4.194386	0.518736	0.735625	-0.21689	12	89866458	F	II	POC1B	Body	opensea	Body-opens		rs1912900 21;45
cg0526632	0.249157	0.591195	10.34739	9.42E-06	0.000808	4.192943	0.466616	0.715773	-0.24916	3	46401927	R	II	CCR2	3'UTR	opensea	3'UTR-oper		rs5721842 8
cg1349731	0.217948	0.649099	10.34596	9.43E-06	0.000809	4.191933	0.540126	0.758073	-0.21795	15	39796985	F	II		IGR	opensea	IGR-opense		rs5520052 0;1;19
cg1448540	0.256135	0.471364	10.34559	9.44E-06	0.000809	4.191667	0.343296	0.599431	-0.25613	2	45063342	R	II		IGR	opensea	IGR-opense		rs1137184 51;50;34;16;2
cg0394361	0.254038	0.557562	10.34518	9.44E-06	0.000809	4.191376	0.430544	0.684581	-0.25404	3	3243751	R	II		IGR	opensea	IGR-opense		rs5272629 24;16
cg1252109	0.232217	0.484393	10.3445	9.44E-06	0.000809	4.1909	0.368284	0.600502	-0.23222	1	2.05E+08	R	II	LRRN2	5'UTR	opensea	5'UTR-oper		rs5760123 8
cg0921353	0.237393	0.544411	10.34444	9.44E-06	0.000809	4.190856	0.425715	0.663108	-0.23739	8	8852726	R	II		IGR	opensea	IGR-opense		rs5685227 50;46;22
cg2575121	0.217749	0.508736	10.34439	9.44E-06	0.000809	4.190822	0.399861	0.61761	-0.21775	13	1.07E+08	F	II		IGR	opensea	IGR-opense		
cg1236703	0.204057	0.595967	10.34057	9.47E-06	0.00081	4.188117	0.493938	0.697995	-0.20406	21	44933622	R	II		IGR	opensea	IGR-opense		rs5824868 13
cg0967665	0.232165	0.563245	10.33969	9.48E-06	0.000811	4.187497	0.447162	0.679327	-0.23216	3	1.68E+08	F	II		IGR	opensea	IGR-opense		rs5575972 1
cg0882484	0.204044	0.464694	10.33918	9.48E-06	0.000811	4.18713	0.362672	0.566716	-0.20404	11	35052388	F	II		IGR	opensea	IGR-opense		
cg1787283	0.223322	0.621693	10.33897	9.48E-06	0.000811	4.186984	0.510032	0.733354	-0.22332	13	52607339	F	II	UTP14C	3'UTR	opensea	3'UTR-oper		rs1875070 0;30;50
cg1746018	0.23585	0.536804	10.33766	9.49E-06	0.000811	4.186057	0.41888	0.654729	-0.23585	10	48370158	R	II	ZNF488	5'UTR	shore	5'UTR-shor	chr10:483	rs5351060 6

cg1270647	0.202282	0.568603	10.33584	9.5E-06	0.000812	4.184767	0.467462	0.669744	-0.20228	1	2.11E+08	F	II	KCNH1	Body	shore	Body-shore	chr1:2108!	rs1440997	0;29;51
cg1402791	0.200908	0.572793	10.33312	9.52E-06	0.000812	4.182845	0.472339	0.673248	-0.20091	3	1.24E+08	R	II	ITGB5	Body	opensea	Body-opens		rs1160223	36;32;2
cg0486668	0.249609	0.598331	10.33287	9.52E-06	0.000812	4.182669	0.473527	0.723135	-0.24961	3	35973060	F	II		IGR	opensea	IGR-opensea		rs1854203	25
cg0761875	0.218156	0.464644	10.33252	9.52E-06	0.000812	4.182422	0.355566	0.573722	-0.21816	6	18551644	F	II		IGR	opensea	IGR-opensea		rs7794763	8;12;30
cg1913809	0.20326	0.611449	10.33244	9.52E-06	0.000812	4.182361	0.509819	0.713079	-0.20326	3	1.8E+08	R	II	PEX5L	5'UTR	opensea	5'UTR-open			
cg1131391	0.225573	0.464477	10.33225	9.52E-06	0.000812	4.182225	0.351691	0.577264	-0.22557	13	23331520	R	II		IGR	opensea	IGR-opensea		rs1408204	51;48;20;16;1
cg1030706	0.229934	0.504501	10.33104	9.53E-06	0.000812	4.181373	0.389534	0.619468	-0.22993	10	64144210	R	II	ZNF365	Body	opensea	Body-opens		rs7075904	30;2
cg2179209	0.205479	0.53426	10.33033	9.54E-06	0.000812	4.18087	0.43152	0.636999	-0.20548	7	24611752	R	II	MPP6	TSS1500	opensea	TSS1500-op		rs5697444	44;19
cg2047598	0.209777	0.63446	10.32969	9.54E-06	0.000812	4.180418	0.529571	0.739349	-0.20978	12	42332113	F	II		IGR	opensea	IGR-opensea		rs1458238	19;43
cg0702963	0.201377	0.570614	10.32902	9.55E-06	0.000812	4.179942	0.469925	0.671302	-0.20138	10	20253868	F	II	PLXDC2	Body	opensea	Body-opens		rs5496323	0
cg1034920	0.201049	0.450344	10.32889	9.55E-06	0.000812	4.179848	0.34982	0.550869	-0.20105	7	37590645	F	II		IGR	opensea	IGR-opensea		rs5681701	8;49
cg2074494	0.210523	0.682064	10.32787	9.55E-06	0.000812	4.179129	0.576803	0.787326	-0.21052	5	1.27E+08	F	II	CTXN3	5'UTR	opensea	5'UTR-open		rs1492638	31
cg1016682	0.228142	0.537786	10.32782	9.55E-06	0.000812	4.179092	0.423715	0.651858	-0.22814	5	1.25E+08	R	II		IGR	opensea	IGR-opensea			
cg0886897	0.225558	0.570974	10.32713	9.56E-06	0.000812	4.178602	0.458195	0.683753	-0.22556	2	1.11E+08	R	II	NPHP1	Body	opensea	Body-opens			
cg2150394	0.216996	0.637975	10.32566	9.57E-06	0.000813	4.177558	0.529477	0.746474	-0.217	10	75232826	R	II	PPP3CB	Body	opensea	Body-opens			
cg1608888	0.216698	0.574269	10.32565	9.57E-06	0.000813	4.177552	0.46592	0.682618	-0.2167	20	56042945	R	II		IGR	opensea	IGR-opensea			
cg0803614	0.257185	0.578898	10.32561	9.57E-06	0.000813	4.177523	0.450305	0.70749	-0.25719	17	8484764	R	II	MYH10	Body	opensea	Body-opens		rs1429598	29
cg1649668	0.224536	0.649322	10.32344	9.58E-06	0.000813	4.17599	0.537054	0.76159	-0.22454	9	1.13E+08	R	II	SVEP1	Body	opensea	Body-opens		rs5650024	3
cg0389964	0.212453	0.549684	10.32125	9.6E-06	0.000814	4.174433	0.443457	0.65591	-0.21245	1	90205170	F	II		IGR	opensea	IGR-opensea		rs1180276	39
cg1897247	0.213185	0.580545	10.32125	9.6E-06	0.000814	4.174431	0.473952	0.687138	-0.21319	10	1.09E+08	F	II		IGR	opensea	IGR-opensea		rs5334580	16;17;41
cg0311737	0.218497	0.485286	10.3201	9.6E-06	0.000814	4.173618	0.376038	0.594535	-0.2185	20	17943971	R	II	SNORD17	TSS1500	opensea	TSS1500-op		rs7510161	44;41;1
cg1987622	0.271569	0.408102	10.32008	9.6E-06	0.000814	4.173605	0.272317	0.543886	-0.27157	7	12641129	F	II	SCIN	Body	opensea	Body-opens		rs1882068	8;16;23
cg0749309	0.210859	0.554048	10.3182	9.62E-06	0.000814	4.172273	0.448619	0.659477	-0.21086	2	1.59E+08	F	II	UPP2	TSS1500	opensea	TSS1500-op		rs1842775	5;11
cg1677438	0.215791	0.530707	10.31467	9.64E-06	0.000815	4.169767	0.422811	0.638602	-0.21579	7	1.43E+08	F	II	OR6V1	TSS1500	opensea	TSS1500-op		rs5640307	0;14
cg0320804	0.259482	0.548686	10.31312	9.65E-06	0.000816	4.168669	0.418945	0.678427	-0.25948	8	59763635	F	II	TOX	Body	opensea	Body-opens		rs1125039	18;32
cg1633884	0.235753	0.557235	10.31172	9.66E-06	0.000816	4.167678	0.439358	0.675111	-0.23575	16	55738066	R	II	SLC6A2	Body	opensea	Body-opens		rs5588036	32;1;0
cg0934138	0.237846	0.489973	10.31153	9.66E-06	0.000816	4.167539	0.37105	0.608896	-0.23785	8	1.05E+08	R	II	RIMS2	Body	opensea	Body-opens		rs5355082	28
cg1960702	0.233008	0.516585	10.3101	9.67E-06	0.000817	4.166523	0.400081	0.633088	-0.23301	5	1.39E+08	R	II		IGR	opensea	IGR-opensea		rs1816952	2;1
cg2675329	0.226502	0.545047	10.30793	9.69E-06	0.000818	4.164985	0.431796	0.658298	-0.2265	2	1.89E+08	R	II	MIR561	TSS1500	shelf	TSS1500-sh	chr2:1891!	rs1840231	22
cg1416785	0.235747	0.537541	10.30707	9.69E-06	0.000818	4.164374	0.419667	0.655415	-0.23575	1	1.2E+08	F	II		IGR	opensea	IGR-opensea		rs5877748	6;21
cg0447436	0.225928	0.453652	10.30626	9.7E-06	0.000818	4.163802	0.340688	0.566616	-0.22593	3	1.87E+08	F	II	SNORA4	TSS200	shelf	TSS200-she	chr3:18650		
cg0661690	0.205695	0.602956	10.30467	9.71E-06	0.000819	4.162673	0.500108	0.705803	-0.2057	1	1.73E+08	F	II		IGR	opensea	IGR-opensea		rs7650428	40
cg2239202	0.218557	0.626013	10.30284	9.72E-06	0.000819	4.161369	0.516734	0.735292	-0.21856	3	1.41E+08	F	II		IGR	opensea	IGR-opensea		rs5423561	28;44
cg1683583	0.202899	0.663152	10.30235	9.72E-06	0.000819	4.161024	0.561703	0.764602	-0.2029	5	17269380	F	II	BASP1	5'UTR	opensea	5'UTR-open		rs5283578	23
cg0569325	0.202744	0.494781	10.30216	9.72E-06	0.000819	4.160891	0.393409	0.596152	-0.20274	8	90479388	F	II		IGR	opensea	IGR-opensea			
cg1729422	0.213642	0.535017	10.30191	9.73E-06	0.000819	4.160712	0.428196	0.641838	-0.21364	3	14865505	R	II	FGD5	Body	opensea	Body-opens		rs1144274	44;36;10
cg1977633	0.223568	0.572272	10.30108	9.73E-06	0.000819	4.16012	0.460489	0.684056	-0.22357	11	86636890	R	II	PRSS23	Body	opensea	Body-opens			
cg2290905	0.235449	0.561641	10.30094	9.73E-06	0.000819	4.160023	0.443917	0.679366	-0.23545	4	1.66E+08	R	II	APELA	3'UTR	opensea	3'UTR-open			
cg0418599	0.226453	0.440369	10.30016	9.74E-06	0.000819	4.15947	0.327142	0.553596	-0.22645	20	23380447	F	II	NAPB	Body	opensea	Body-opens		rs5730355	19
cg1134478	0.201132	0.672285	10.30002	9.74E-06	0.000819	4.159368	0.571719	0.772851	-0.20113	18	44689907	F	II	IER3IP1	Body	opensea	Body-opens		rs5760042	32;39
cg2107495	0.217138	0.600702	10.29974	9.74E-06	0.000819	4.159171	0.492134	0.709271	-0.21714	2	2.03E+08	R	II		IGR	opensea	IGR-opensea		rs5454042	26
cg1695905	0.206177	0.514164	10.298	9.75E-06	0.00082	4.157935	0.411076	0.617253	-0.20618	3	87256918	R	II		IGR	opensea	IGR-opensea		rs5644155	10;1
cg0642879	0.204411	0.61441	10.29744	9.76E-06	0.00082	4.157532	0.512204	0.716616	-0.20441	19	49996158	F	II		IGR	shelf	IGR-shelf	chr19:4999!	rs1921896	1;28;35;36;49
cg1535824	0.228803	0.605121	10.29387	9.78E-06	0.000821	4.154997	0.49072	0.719523	-0.2288	15	36737547	R	II		IGR	opensea	IGR-opensea		rs1120257	22
cg1086890	0.204437	0.459493	10.29162	9.8E-06	0.000821	4.153398	0.357274	0.561711	-0.20444	10	1.01E+08	R	II	CNNM1	Body	opensea	Body-opens		rs5605775	40;15
cg2149585	0.263493	0.379535	10.29133	9.8E-06	0.000821	4.15319	0.247789	0.511281	-0.26349	1	2.22E+08	R	II		IGR	opensea	IGR-opensea		rs1886349	42;2;1
cg0528743	0.217539	0.578597	10.2905	9.8E-06	0.000822	4.152601	0.469828	0.687367	-0.21754	3	16215248	R	II	GALNTL2	TSS1500	opensea	TSS1500-op			
cg1100522	0.206268	0.541739	10.2903	9.81E-06	0.000822	4.152462	0.438605	0.644873	-0.20627	1	67114525	R	II	SGIP1	Body	opensea	Body-opens		rs1915738	18;17;1
cg0449970	0.227562	0.576219	10.28775	9.82E-06	0.000822	4.150645	0.462438	0.689999	-0.22756	11	1.11E+08	R	II		IGR	opensea	IGR-opensea		rs5779001	50;2;1
cg1373868	0.274094	0.50737	10.28717	9.83E-06	0.000822	4.150235	0.370322	0.644417	-0.27409	3	1.86E+08	R	II		IGR	opensea	IGR-opensea		rs1918843	20;17
cg1521293	0.239349	0.448794	10.28368	9.85E-06	0.000823	4.14775	0.32912	0.568469	-0.23935	11	59223558	F	II	OR4D6	TSS1500	opensea	TSS1500-op		rs5547363	45
cg2759462	0.207833	0.545793	10.28024	9.87E-06	0.000824	4.1453	0.441877	0.64971	-0.20783	16	16002061	F	II		IGR	opensea	IGR-opensea		rs5735807	1;2;3
cg1327007	0.217719	0.583724	10.27656	9.9E-06	0.000825	4.142682	0.474864	0.692583	-0.21772	13	42987244	R	II		IGR	opensea	IGR-opensea		rs5351549	6;5;4;1
cg1311507	0.217515	0.546571	10.2755	9.91E-06	0.000825	4.141924	0.437813	0.655328	-0.21752	3	1.49E+08	F	II		IGR	opensea	IGR-opensea		rs6227769	51
cg0329028	0.200206	0.595616	10.27474	9.91E-06	0.000825	4.141383	0.495513	0.695719	-0.20021	6	2632607	F	II	C6orf195	5'UTR	shore	5'UTR-shore	chr6:2634:	rs1403560	5;19
cg0936781	0.287327	0.591184	10.27277	9.93E-06	0.000826	4.139984	0.44752	0.734847	-0.28733	21	30463648	R	II	MAP3K7CL	TSS1500	opensea	TSS1500-op		rs8131128	25;17;1
cg2095583	0.217173	0.466925	10.27263	9.93E-06	0.000826	4.139882	0.358339	0.575511	-0.21717	2	1.09E+08	R	II	SULT1C2	Body	opensea	Body-opens		rs5534434	29;5;1
cg1389797	0.226693	0.54101	10.27123	9.94E-06	0.000826	4.138883	0.427664	0.654356	-0.22669	12	88928761	F	II	KITLG	Body	opensea	Body-opens		rs1183567	0;25
cg1334070	0.218592	0.550435	10.27077	9.94E-06	0.000827	4.138556	0.441139	0.659731	-0.21859	18	30091235	F	II	WBP11P1	TSS1500	opensea	TSS1500-op		rs1159150	28;33;42;44
cg2456342	0.253396	0.609752	10.26999	9.95E-06	0.000827	4.137999	0.483054	0.73645	-0.2534	6	1.38E+08	R	II		IGR	opensea	IGR-opensea		rs5541432	1
cg1113124	0.228283	0.614773	10.26948	9.95E-06	0.000827	4.137638	0.500631	0.728914	-0.22828	20	1396853	F	II		IGR	opensea	IGR-opensea		rs1483980	0;21;34
cg1971160	0.236048	0.585395	10.26651	9.97E-06	0.000828	4.135518	0.467371	0.703419	-0.23605	13	50033894	R	II	SETDB2	Body	opensea	Body-opens		rs1495988	19
cg1361878	0.216454	0.477749	10.26521	9.98E-06	0.000828	4.134598	0.369523	0.585976	-0.21645	1	2.06E+08	F	II	CDK18	3'UTR	shore	3'UTR-shore	chr1:2054!	rs1124050	32;35
cg1803101	0.207716	0.643399	10.26474	9.98E-06	0.000828	4.134262	0.539542	0.747257	-0.20772	10	14499148	F	II		IGR	opensea	IGR-opensea		rs1404087	1;13;35
cg1706204	0.203509	0.665087	10.26306	9.99E-06	0.000828	4.133063	0.563332	0.766842	-0.20351	7	80803564	F	II		IGR	opensea	IGR-opensea		rs5462638	30;34;47
cg0474987	0.260779	0.570265	10.263	9.99E-06	0.000828	4.133016	0.439875	0.700655	-0.26078	8	71039880	R	II	NCOA2	Body	opensea	Body-opens		rs1146388	16;1
cg0972528	0.22857	0.617618	10.26047	1E-05	0.000829	4.131213	0.503333	0.731903	-0.22857	8	1.34E+08	R	II		IGR	opensea	IGR-opensea		rs1181422	25;22
cg1958505	0.25447	0.493061	10.25963	1E-05	0.000829	4.130617	0.365826	0.620296	-0.25447	7	85103687	R	II		IGR	opensea	IGR-opensea		rs2091086	18;1
cg1447205	0.227864	0.598135	10.25951	1E-05	0.000829	4.130527	0.484202	0.712067	-0.22786	2	2.07E+08	R	II	NRP2	Body	opensea	Body-opens		rs1904355	45;1
cg0264622	0.237375	0.502275	10.25946	1E-05	0.000829	4.130494	0.383588	0.620963	-0.23737	2	2.32E+08	F	II		IGR	opensea	IGR-opensea		rs5379568	0;1;6;15
cg0439675	0.215869	0.549207	10.2592	1E-05	0.000829	4.130311	0.441272	0.657142	-0.21587	2	1.14E+08	F	II		IGR	opensea	IGR-opensea			
cg1447270	0.2191	0.594301	10.25807	1E-05	0.000829	4.129503	0.484751	0.70385	-0.2191	3	1.02E+08	F	II	NXPE3	3'UTR	opensea	3'UTR-open		rs6795426	6;50
cg2669461	0.202994	0.740908	10.258	1E-05	0.000829	4.12945	0.639411	0.842405	-0.20299	9	21017267	R	II	HACD4	Body	opensea	Body-opens		rs5776822	35;34;29;21
cg2352040	0.21018	0.58193	10.25608	1E-05	0.00083	4.128084	0.47684	0.68702	-0.21018	4	41388199	R	II	LIMCH1	5'UTR	opensea	5'UTR-open			
cg1176591	0.21056	0.588197	10.25535	1E-05	0.00083	4.127559	0.482917	0.693477	-0.21056	19	7926080	F	II	EVI5L	Body	shore	Body-shore	chr19:792(	rs5391506	19
cg2006937	0.207567	0.495299	10.25469	1.01E-05	0.00083	4.12709	0.391516	0.599083	-0.20757	10	98117247	R	II	OPALIN	5'UTR	opensea	5'UTR-open		rs5600164	27
cg2658624	0.201805	0.603518	10.25436	1.01E-05	0.00083	4.126857	0.502616	0.704421	-0.20181	2	9731123	R	II	YWHAQ	Body	opensea	Body-opens		rs3722789	14;1
cg2279083	0.232258	0.528855	10.25295	1.01E-05	0.00083	4.125851	0.412725	0.644984	-0.23226	5	1.58E+08	R	II		IGR	opensea	IGR-opensea		rs5359846	38;15;1
cg1016731	0.240108	0.504281	10.25134	1.01E-05	0.000831	4.124698	0.384227	0.624335	-0.24011	15	70115869	R	II		IGR	opensea	IGR-opensea		rs1435769	45
cg1479476	0.222388	0.610178	10.25101	1.01E-05	0.000831	4.124467	0.498984	0.721372	-0.22239	8	1.25E+08	F	II	FER1L6	5'UTR	opensea	5'UTR-open			
cg1871373	0.201838	0.396808	10.25068	1.01E-05	0.000831	4.124227	0.295889	0.497727	-0.20184	22	28101490	F	II		IGR	opensea	IGR-opensea		rs5633913	9
cg0388950	0.208588	0.539953	10.25018	1.01E-05	0.000831	4.123873	0.435659	0.644247	-0.20859	5	33309433	R	II		IGR	opensea	IGR-opensea			
cg0647717	0.221866	0.530088	10.24967	1.01E-05	0.000831	4.123505	0.419155	0.641021	-0.22187	15	34533266	F	II	SLC12A6	Body	opensea	Body-opens		rs1861525	0
cg1609940	0.231641	0.550892	10.24812	1.01E-05	0.000832	4.1224	0.435071	0.666713	-0.23164	2	2.02E+08	R	II	STRADB	Body	opensea	Body-opens		rs1815644	35;7;2;1
cg2231890	0.245206	0.442101	10.24665	1.01E-05	0.000832	4.121347	0.319498	0.564704	-0.24521	13	34253578	R	II		IGR	opensea	IGR-opensea		rs7316399	11;2
cg0007597	0.221006	0.536708	10.24642	1.01E-05	0.000832	4.121188	0.426205	0.647211	-0.22101	1	2.3E+08	F	II	PGBD5	Body	shore	Body-shore	chr1:2304!	rs1833643	1;19
cg0143674	0.228683	0.605083	10.24498	1.01E-05	0.000832	4.12016	0.490742	0.719425	-0.22868	5	1.36E+08	R	II		IGR	opensea	IGR-opensea		rs5313048	29;19
cg1028815	0.222515	0.607118	10.24449	1.01E-05	0.000832	4.119809	0.49586	0.718376	-0.22252	3	1.1E+08	F	II		IGR	opensea	IGR-opensea		rs1390244	1
cg2239245	0.229757	0.510348	10.24414	1.01E-05	0.000832	4.119561	0.395469	0.625226	-0.22976	6	10839494	F	II	MAK	TSS1500	shore	TSS1500-sh	chr6:1083!	rs5466470	34
cg0876146	0.202757	0.56627	10.24395	1.01E-05	0.000832	4.119421	0.464891	0.667649	-0.20276	16	87118888	R	II		IGR	opensea	IGR-opensea		rs1459520	1
cg0759982	0.222231	0.429007	10.24189	1.01E-05	0.000833	4.117948	0.317892	0.540122	-0.22223	17	30053217	F	II		IGR	opensea	IGR-opensea			
cg1559860	0.230911	0.659965	10.24189	1.01E-05	0.000833	4.117948	0.544509	0.77542	-0.23091	18	52339396	F	II		IGR	opensea	IGR-opensea		rs1416627	0;1;25;43
cg1250217	0.21075	0.643254	10.24053	1.02E-05	0.000833	4.116975	0.537879	0.748629	-0.21075	12	1.08E+08	F	II		IGR	opensea	IGR-opensea		rs1427256	1;30
cg1630214	0.200554	0.506643	10.23946	1.02E-05	0.000834	4.116213	0.406366	0.60692	-0.20055	6	5094765	F	II	LOC10012!	Body	opensea	Body-opens		rs1442156	8;31
cg1273254	0.205259	0.587452	10.23942	1.02E-05	0.000834	4.116184	0.484823	0.690081	-0.20526	15	40631573	R	II	C15orf52	Body	opensea	Body-opens		rs5465050	35;1
cg1291542	0.212763	0.491024	10.2391	1.02E-05	0.000834	4.115955	0.384642	0.597406	-0.21276	3	28615891	F	II	LINC00693	TSS1500	shore	TSS1500-sh	chr3:2861(	rs5753153	0
cg1165092	0.221744	0.495254	10.23801	1.02E-05	0.000834	4.115178	0.384382	0.606125	-0.22174	1	68490241	F	II		IGR	opensea	IGR-opensea		rs5681466	9
cg1040107	0.232919	0.434527	10.23689	1.02E-05	0.000834	4.114378	0.318068	0.550987	-0.23292	1	66673501	R	II	PDE4B	Body	opensea	Body-opens		rs5399550	45;26;24;1
cg2076985	0.212399	0.540267	10.23411	1.02E-05	0.000835	4.112385	0.434068	0.646467	-0.2124	13	31422123	F	II		IGR	opensea	IGR-opensea		rs3735062	24;25;48
cg1611219	0.20483	0.431568	10.23384	1.02E-05	0.000835	4.112193	0.329153	0.533983	-0.20483	4	1.05E+08	F	II		IGR	opensea	IGR-opensea		rs1907632	1
cg2097229	0.20683	0.528961	10.23312	1.02E-05	0.000835	4.111679	0.425546	0.632377	-0.20683	11	1.33E+08	R	II	OPCML	Body	opensea	Body-opens		rs5310502	24
cg1177427	0.228521	0.569946	10.23286	1.02E-05	0.000835	4.111492	0.455686	0.684207	-0.22852	17	49110507	R	II	SPAG9	Body	opensea	Body-opens		rs5492084	2
cg1486067	0.211529	0.620397	10.23243	1.02E-05	0.000835	4.111188	0.514633	0.726162	-0.21153	20	48393157	R	II		IGR	opensea	IGR-opensea		rs5372613	28;14
cg2111986	0.214428	0.627966	10.2323	1.02E-05	0.000835	4.111094	0.520752	0.73518	-0.21443	8	82374723	F	II	FABP9	TSS1500	opensea	TSS1500-op		rs5747548	0;47
cg1382857	0.214562	0.587192	10.2314	1.02E-05	0.000835	4.110453	0.479911	0.694473	-0.21456	9	1.24E+08	F	II	GSN	Body	opensea	Body-opens		rs1172664	0
cg0266335	0.206035	0.615101	10.23112	1.02E-05	0.000835	4.110246	0.512083	0.718118	-0.20603	11	35167041	R	II	CD44	Body	opensea	Body-opens		rs5338040	1
cg1381538	0.215044	0.639115	10.23013	1.02E-05	0.000836	4.109542	0.531593	0.746637	-0.21504	5	32642264	F	II		IGR	opensea	IGR-opensea		rs818131	26
cg0167462	0.251489	0.456989	10.2298	1.02E-05	0.000836	4.109308	0.331245	0.582734	-0.25149	7	1.06E+08	R	II	NAMPT	Body	opensea	Body-opens		rs5367027	21
cg0307063	0.230057	0.473749	10.22859	1.02E-05	0.000836	4.108437	0.358721	0.588778	-0.23006	2	1991629	R	II	MYT1L	5'UTR	opensea	5'UTR-open		rs7459586	43;29;2
cg1270211	0.230057	0.509018	10.22678	1.02E-05	0.000836	4.107145	0.393989	0.624046	-0.23006	7	90337268	R	II	CDK14	TSS1500	opensea	TSS1500-op		rs5492741	15;1
cg2537656	0.206232	0.716311	10.22669	1.03E-05	0.000836	4.107077	0.613195	0.819427	-0.20623	2	60065508	F	II		IGR	opensea	IGR-opensea		rs5377228	7;23
cg1390098	0.239827	0.58929	10.22457	1.03E-05	0.000837	4.105565	0.469377	0.709204	-0.23983	14	90730393	R	II	PSMC1	ExonBnd	opensea	ExonBnd-op		rs1337938	43;2
cg1453356	0.274217	0.450676	10.22443	1.03E-05	0.000837	4.105464	0.313567	0.587784	-0.27422	3	52705872	R	II	PBRM1	Body	opensea	Body-opens		rs5497009	33;22
cg1307894	0.230393	0.572489	10.22356	1.03E-05	0.000837	4.104839	0.457292	0.687685	-0.23039	1	10463746	R	II	PGD	Body	shelf	Body-shelf	chr1:1045!		
cg1195974	0.219222	0.599453	10.22266	1.03E-05	0.000837	4.104193	0.489842	0.709064	-0.21922	12	50321909	R	II		IGR	opensea	IGR-opensea		rs1164898	16;2
cg1690517	0.214288	0.581104	10.22231	1.03E-05	0.000837	4.103946	0.47396	0.688248	-0.21429	10	27258099	R	II		IGR	opensea	IGR-opensea		rs3431952	37
cg0537267	0.202354	0.520802	10.2211	1.03E-05	0.000837	4.103079	0.419625	0.62198	-0.20235	15	48064730	F	II	SEMA6D	3'UTR	opensea	3'UTR-open		rs1119038	27
cg2084005	0.206203	0.624729	10.22078	1.03E-05	0.000837	4.102849	0.521628	0.727831	-0.2062	2	1.28E+08	R	II	LIMS2	Body	shore	Body-shore	chr2:1284:	rs1893590	14
cg0867858	0.203317	0.530104	10.2207	1.03E-05	0.000837	4.10279	0.428446	0.631763	-0.20332	21	38531638	R	II	TTC3	Body	opensea	Body-opens		rs1071263	23

cg0749071	0.213244	0.605812	10.22038	1.03E-05	0.000837	4.102561	0.49919	0.712434	-0.21324	2	39744451	R	II		IGR	opensea	IGR-opense		rs1866953	26
cg2573408	0.278311	0.424532	10.21984	1.03E-05	0.000837	4.102178	0.285377	0.563688	-0.27831	19	46969919	R	II	PNMAL1	3'UTR	opensea	3'UTR-oper		rs5658754	36
cg1009482	0.227623	0.450159	10.21913	1.03E-05	0.000838	4.101667	0.336348	0.563971	-0.22762	10	8642275	R	II		IGR	opensea	IGR-opense		rs5321476	40;38;25
cg2394234	0.204664	0.671366	10.21848	1.03E-05	0.000838	4.101203	0.569034	0.773698	-0.20466	12	57858027	R	II	GLI1	5'UTR	opensea	5'UTR-oper		rs5382328	33;21;18;14;13;6
cg2045528	0.21106	0.665955	10.2165	1.03E-05	0.000838	4.099784	0.560425	0.771485	-0.21106	20	2293532	R	II	TGM3	ExonBnd	opensea	ExonBnd-op		rs2003085	34;1
cg2709098	0.20711	0.630746	10.21572	1.03E-05	0.000838	4.099226	0.527191	0.734301	-0.20711	3	9789110	F	II	BRPF1	3'UTR	shelf	3'UTR-shelf	chr3:9791	rs5314948	48
cg0814986	0.226022	0.547266	10.21506	1.03E-05	0.000839	4.098752	0.434255	0.660277	-0.22602	21	24264513	F	II		IGR	opensea	IGR-opense			
cg0866164	0.22526	0.658781	10.21436	1.03E-05	0.000839	4.098253	0.546151	0.771411	-0.22526	8	19964604	R	II		IGR	opensea	IGR-opense		rs5661705	47;2
cg0791308	0.250974	0.679446	10.21424	1.03E-05	0.000839	4.098166	0.553959	0.804933	-0.25097	3	18278830	F	II	LOC33986	Body	opensea	Body-opens		rs5380826	0;39
cg1485645	0.221392	0.63626	10.21402	1.03E-05	0.000839	4.098008	0.525564	0.746955	-0.22139	17	36076623	F	II	HNF1B	Body	opensea	Body-opens		rs5411615	46
cg1669256	0.242261	0.60223	10.21358	1.03E-05	0.000839	4.097688	0.481099	0.72336	-0.24226	2	39135866	R	II		IGR	opensea	IGR-opense		rs1463480	20
cg2399175	0.230446	0.585818	10.21129	1.04E-05	0.00084	4.096046	0.470595	0.701042	-0.23045	21	30266046	R	II		IGR	opensea	IGR-opense		rs5626043	30
cg2029880	0.201694	0.633758	10.20774	1.04E-05	0.000841	4.093503	0.532911	0.734605	-0.20169	4	75955358	F	II	PARM1	Body	opensea	Body-opens		rs1700010	12
cg0677167	0.202304	0.624728	10.20536	1.04E-05	0.000841	4.0918	0.523576	0.72588	-0.2023	7	1.15E+08	F	II	LINC01393	TSS1500	opensea	TSS1500-op			
cg2110556	0.201868	0.564287	10.202	1.04E-05	0.000842	4.089385	0.463352	0.665221	-0.20187	11	19218301	F	II	CSRP3	5'UTR	opensea	5'UTR-oper		rs5717534	0
cg0724323	0.206306	0.651236	10.2	1.04E-05	0.000842	4.087952	0.548083	0.754389	-0.20631	5	80508236	R	II	RASGRF2	Body	opensea	Body-opens		rs1475515	22;14
cg0089004	0.205861	0.523758	10.19902	1.05E-05	0.000843	4.087247	0.420828	0.626688	-0.20586	6	1.16E+08	F	II		IGR	opensea	IGR-opense		rs5707477	0;1
cg1760500	0.229798	0.523778	10.19811	1.05E-05	0.000843	4.086592	0.408879	0.638677	-0.2298	2	20776435	F	II		IGR	opensea	IGR-opense		rs1828360	0;18;50
cg1326155	0.206287	0.534524	10.19772	1.05E-05	0.000843	4.086316	0.431381	0.637668	-0.20629	7	1.06E+08	F	II		IGR	opensea	IGR-opense		rs5290719	0;51
cg1165541	0.237376	0.569331	10.19772	1.05E-05	0.000843	4.086315	0.450643	0.688019	-0.23738	7	78870151	R	II	MAGI2	Body	opensea	Body-opens		rs5519685	49
cg0107075	0.206835	0.459047	10.19429	1.05E-05	0.000844	4.083856	0.355629	0.562464	-0.20683	6	12372429	F	II		IGR	opensea	IGR-opense		rs5320795	21;37
cg2599023	0.229429	0.577557	10.19418	1.05E-05	0.000844	4.083772	0.462842	0.692271	-0.22943	11	1.25E+08	R	II	ROBO4	Body	opensea	Body-opens		rs1489388	48;47;33;16;5;2;1
cg0173222	0.210344	0.570595	10.19375	1.05E-05	0.000844	4.083463	0.465423	0.675766	-0.21034	11	4659876	R	II	OR51D1	TSS1500	opensea	TSS1500-op		rs5618765	47;20
cg0637953	0.201448	0.705876	10.19364	1.05E-05	0.000844	4.08339	0.605152	0.8066	-0.20145	3	32898362	F	II	TRIM71	Body	opensea	Body-opens		rs5299820	17;20;22
cg1122651	0.220337	0.546305	10.19236	1.05E-05	0.000844	4.08247	0.436137	0.656474	-0.22034	12	55076393	R	II		IGR	opensea	IGR-opense		rs5504472	43;1
cg0070459	0.229608	0.583178	10.19035	1.05E-05	0.000845	4.081021	0.468375	0.697982	-0.22961	7	92534346	R	II	LOC10192	Body	opensea	Body-opens		rs5653493	20
cg2640892	0.227275	0.536546	10.1892	1.05E-05	0.000846	4.080195	0.422908	0.650183	-0.22728	3	53759723	F	II	CACNA1D	Body	opensea	Body-opens		rs5553138	0;26;34
cg1330420	0.237175	0.419563	10.18724	1.05E-05	0.000846	4.078793	0.300976	0.538151	-0.23717	4	40562130	F	II	RBM47	5'UTR	opensea	5'UTR-oper		rs3749506	0;14
cg0302600	0.206012	0.503678	10.18716	1.05E-05	0.000846	4.078733	0.400672	0.606684	-0.20601	18	6833417	R	II	ARHGAP28	TSS1500	opensea	TSS1500-op		rs5751656	42;2
cg2041103	0.210717	0.441643	10.18621	1.05E-05	0.000846	4.078047	0.336285	0.547002	-0.21072	2	1.9E+08	R	II		IGR	opensea	IGR-opense			
cg0832950	0.238023	0.377747	10.18556	1.05E-05	0.000846	4.077585	0.258735	0.496759	-0.23802	9	79056558	F	II	GCNT1	TSS200	opensea	TSS200-ope		rs5442034	0;5
cg1399762	0.22116	0.590375	10.18434	1.06E-05	0.000846	4.076703	0.479795	0.700955	-0.22116	15	51641258	F	II	GLDN	Body	opensea	Body-opens		rs1417915	1;23;25
cg0375227	0.205351	0.565813	10.18373	1.06E-05	0.000846	4.076267	0.463138	0.668489	-0.20535	12	2977347	F	II	FOXM1	Body	opensea	Body-opens		rs1927248	1;21;22
cg1483451	0.21466	0.569175	10.18229	1.06E-05	0.000846	4.075234	0.461845	0.676505	-0.21466	3	1.53E+08	F	II		IGR	opensea	IGR-opense			
cg1751370	0.223907	0.531836	10.18098	1.06E-05	0.000847	4.074292	0.419882	0.64379	-0.22391	1	2.48E+08	F	II	OR2G3	TSS1500	opensea	TSS1500-op		rs1425811	1;5
cg1899015	0.222051	0.509634	10.18036	1.06E-05	0.000847	4.073845	0.398609	0.62066	-0.22205	2	27166550	R	II	DPYSL5	Body	opensea	Body-opens		rs1880314	32;8
cg1026920	0.2042	0.446577	10.18032	1.06E-05	0.000847	4.073819	0.344477	0.548678	-0.2042	6	10237806	R	II		IGR	opensea	IGR-opense		rs5481839	33;28;1
cg1304895	0.209472	0.496672	10.17691	1.06E-05	0.000848	4.071365	0.391936	0.601408	-0.20947	7	1.42E+08	R	II		IGR	opensea	IGR-opense		rs7797904	30;19;3;2;1
cg2277189	0.2626	0.51584	10.17602	1.06E-05	0.000848	4.070724	0.384539	0.64714	-0.2626	8	17613996	R	II	MTUS1	5'UTR	opensea	5'UTR-oper		rs5499932	49;2;1
cg1035268	0.265884	0.56273	10.17522	1.06E-05	0.000848	4.070148	0.429788	0.695671	-0.26588	21	17406587	R	II		IGR	opensea	IGR-opense			
cg1168371	0.240946	0.583219	10.17516	1.06E-05	0.000848	4.070108	0.462746	0.703692	-0.24095	5	1.6E+08	R	II		IGR	opensea	IGR-opense		rs1497242	15;1
cg2478357	0.211294	0.615984	10.17508	1.06E-05	0.000848	4.070046	0.510336	0.721631	-0.21129	20	12297171	R	II		IGR	opensea	IGR-opense		rs1137941	43;33;19;1
cg2586731	0.214274	0.473198	10.1745	1.06E-05	0.000848	4.06963	0.366061	0.580335	-0.21427	17	40494745	F	II	STAT3	Body	opensea	Body-opens		rs5625963	27
cg1405720	0.23663	0.610823	10.17438	1.06E-05	0.000848	4.069544	0.492509	0.729138	-0.23663	2	39659093	F	II	MAP4K3	Body	opensea	Body-opens		rs7950591	1;15;24;45
cg2350783	0.245969	0.414151	10.17433	1.06E-05	0.000848	4.069507	0.291166	0.537136	-0.24597	3	1.84E+08	F	II		IGR	opensea	IGR-opense		rs1159349	0;1
cg0027582	0.228671	0.502629	10.17404	1.06E-05	0.000848	4.069297	0.388294	0.616965	-0.22867	17	39222690	R	II	KRTAP2-4	TSS1500	opensea	TSS1500-op		rs5367711	20;18;1
cg2277801	0.213391	0.589964	10.17297	1.06E-05	0.000848	4.068526	0.483269	0.69666	-0.21339	4	1.1E+08	R	II	COL25A1	Body	opensea	Body-opens			
cg0867599	0.248351	0.522115	10.17272	1.06E-05	0.000848	4.068349	0.39794	0.646291	-0.24835	7	1.31E+08	F	II	LINC-PINT	Body	opensea	Body-opens			
cg2082087	0.214262	0.740931	10.17134	1.07E-05	0.000849	4.067353	0.633799	0.848062	-0.21426	21	37741721	F	II	MORC3	Body	opensea	Body-opens		rs5714799	23;26
cg1548626	0.203692	0.548862	10.17123	1.07E-05	0.000849	4.067279	0.447016	0.650708	-0.20369	6	1.4E+08	R	II		IGR	opensea	IGR-opense		rs5581060	36;20
cg0393355	0.230272	0.66858	10.168	1.07E-05	0.000849	4.064949	0.553443	0.783716	-0.23027	18	55906325	F	II	NEDD4L	Body	opensea	Body-opens		rs3736775	1;25;26
cg2483613	0.206683	0.599236	10.16679	1.07E-05	0.00085	4.064079	0.495895	0.702578	-0.20668	15	95561441	F	II		IGR	opensea	IGR-opense		rs7565296	0
cg1492584	0.276519	0.449926	10.16677	1.07E-05	0.00085	4.064063	0.311667	0.588186	-0.27652	21	35790214	F	II		IGR	opensea	IGR-opense		rs5487386	6;14;32;34
cg2220713	0.20511	0.560973	10.16471	1.07E-05	0.00085	4.062585	0.458417	0.663528	-0.20511	15	59467736	F	II	MYO1E	Body	opensea	Body-opens		rs5634345	0;1;37
cg1199357	0.208784	0.549212	10.16411	1.07E-05	0.00085	4.062148	0.44482	0.653604	-0.20878	7	18509097	F	II	HDAC9	Body	opensea	Body-opens		rs2855830	5;13;17;29;44
cg0476219	0.22202	0.391799	10.16397	1.07E-05	0.00085	4.062053	0.280789	0.502809	-0.22202	1	1.81E+08	F	II		IGR	opensea	IGR-opense		rs1931272	20;43
cg0322263	0.214966	0.687679	10.16258	1.07E-05	0.00085	4.061048	0.580196	0.795162	-0.21497	11	1.11E+08	F	II	LAYN	3'UTR	opensea	3'UTR-oper			
cg0401147	0.20288	0.651992	10.16255	1.07E-05	0.00085	4.061024	0.550552	0.753432	-0.20288	2	28904455	R	II		IGR	opensea	IGR-opense		rs5478775	1
cg1385683	0.247707	0.608922	10.16242	1.07E-05	0.00085	4.060935	0.485069	0.732776	-0.24771	3	1.57E+08	F	II	LINC00886	Body	opensea	Body-opens		rs5392272	1
cg0923644	0.212454	0.520442	10.1615	1.07E-05	0.00085	4.06027	0.414215	0.626669	-0.21245	3	73591147	F	II	PDZRN3	Body	opensea	Body-opens		rs5326919	0;26
cg2487559	0.204246	0.657164	10.15991	1.07E-05	0.000851	4.059129	0.555042	0.759287	-0.20425	2	79363761	R	II	REG1P	Body	opensea	Body-opens		rs5471295	21;2
cg0376817	0.213144	0.561809	10.1589	1.07E-05	0.000851	4.0584	0.455238	0.668381	-0.21314	14	1.01E+08	R	II	MEG3	Body	shelf	Body-shelf	chr14:101		
cg1359041	0.247406	0.500516	10.15787	1.08E-05	0.000851	4.057658	0.376813	0.624219	-0.24741	20	43209782	R	II	PKIG	TSS1500	opensea	TSS1500-op		rs5624246	40;1
cg2699224	0.227643	0.497466	10.15596	1.08E-05	0.000852	4.056277	0.383644	0.611287	-0.22764	8	29848579	R	II		IGR	opensea	IGR-opense		rs5704616	48;32
cg2147730	0.22388	0.512762	10.155	1.08E-05	0.000852	4.055587	0.400822	0.624702	-0.22388	20	46701128	R	II		IGR	opensea	IGR-opense		rs5744122	2
cg2055938	0.23233	0.509087	10.15497	1.08E-05	0.000852	4.055567	0.392922	0.625252	-0.23233	6	1.06E+08	F	II		IGR	opensea	IGR-opense			
cg2038076	0.263204	0.591134	10.15452	1.08E-05	0.000852	4.055244	0.459532	0.722736	-0.2632	7	1.3E+08	F	II	MEST	TSS1500	shore	TSS1500-sh	chr7:1301	rs5505774	37
cg1410939	0.203088	0.392975	10.15449	1.08E-05	0.000852	4.055221	0.291431	0.494518	-0.20309	13	61390507	R	II		IGR	opensea	IGR-opense		rs1465286	19;8;2
cg1299544	0.219079	0.656658	10.15279	1.08E-05	0.000853	4.053991	0.547119	0.766197	-0.21908	6	1.23E+08	R	II	PKIB	Body	opensea	Body-opens		rs1837973	51;45;23
cg2635284	0.241553	0.514882	10.15278	1.08E-05	0.000853	4.053986	0.394106	0.635658	-0.24155	4	53689989	R	II		IGR	opensea	IGR-opense		rs5484781	33;30
cg0634978	0.209147	0.484202	10.15153	1.08E-05	0.000853	4.053087	0.379628	0.588776	-0.20915	2	1.23E+08	F	II		IGR	opensea	IGR-opense		rs1425285	1
cg0239670	0.220544	0.616216	10.15051	1.08E-05	0.000853	4.052348	0.505944	0.726488	-0.22054	18	56740117	R	II		IGR	opensea	IGR-opense		rs5598518	2
cg0931561	0.216729	0.370159	10.14701	1.08E-05	0.000854	4.049823	0.261794	0.478523	-0.21673	13	24154719	R	II	TNFRSF19	5'UTR	shore	5'UTR-shor	chr13:241	rs5583147	35;33
cg1784609	0.240241	0.462389	10.14292	1.09E-05	0.000856	4.046875	0.342269	0.58251	-0.24024	3	1.57E+08	F	II	LINC00880	Body	opensea	Body-opens		rs5338856	1;14;23;39
cg0129483	0.204368	0.597855	10.14272	1.09E-05	0.000856	4.046729	0.495671	0.700038	-0.20437	6	1.67E+08	R	II	RPS6KA2	Body	opensea	Body-opens		rs1167328	39;17
cg0094847	0.220544	0.429522	10.14216	1.09E-05	0.000856	4.046327	0.31925	0.539795	-0.22054	2	1.14E+08	R	II		IGR	opensea	IGR-opense		rs5407719	26;1
cg1534831	0.232441	0.618324	10.14096	1.09E-05	0.000856	4.045461	0.502104	0.734545	-0.23244	6	14282571	R	II		IGR	opensea	IGR-opense		rs1472479	30;2
cg0075212	0.209598	0.598193	10.14066	1.09E-05	0.000856	4.045239	0.493394	0.702991	-0.2096	2	25241029	R	II	DNAJC27-A	Body	opensea	Body-opens		rs4665757	10
cg0864526	0.227359	0.585283	10.13998	1.09E-05	0.000856	4.044754	0.471604	0.698963	-0.22736	17	19484616	R	II		IGR	shore	IGR-shore	chr17:194	rs5339464	1
cg0503699	0.211174	0.471192	10.13944	1.09E-05	0.000856	4.044364	0.365605	0.576779	-0.21117	2	1.55E+08	F	II	GALNT13	Body	opensea	Body-opens		rs1894686	0;9;13
cg0288308	0.2161	0.501655	10.13935	1.09E-05	0.000856	4.044296	0.393605	0.609705	-0.2161	10	1.3E+08	R	II		IGR	opensea	IGR-opense		rs1436066	1
cg2029368	0.253739	0.501957	10.13918	1.09E-05	0.000856	4.044173	0.375087	0.628826	-0.25374	19	2668369	F	II	GNG7	5'UTR	opensea	5'UTR-oper		rs1492075	17
cg0546513	0.204011	0.426	10.1382	1.09E-05	0.000857	4.043463	0.323994	0.528005	-0.20401	5	1.33E+08	F	II	FSTL4	Body	opensea	Body-opens		rs5655527	28
cg0612174	0.247298	0.660272	10.13783	1.09E-05	0.000857	4.0432	0.536623	0.783921	-0.2473	10	34511567	F	II	PARD3	Body	opensea	Body-opens		rs7456207	42;51
cg0565948	0.21823	0.540699	10.13661	1.09E-05	0.000857	4.042316	0.431584	0.649814	-0.21823	6	53792689	R	II		IGR	opensea	IGR-opense			
cg2759207	0.223471	0.564803	10.13656	1.09E-05	0.000857	4.042284	0.453068	0.676539	-0.22347	6	18016000	R	II		IGR	opensea	IGR-opense		rs1883930	36;18
cg0830665	0.207092	0.57708	10.13578	1.09E-05	0.000857	4.04172	0.473534	0.680626	-0.20709	7	1.08E+08	R	II	LAMB4	5'UTR	opensea	5'UTR-oper			
cg2403410	0.204092	0.492013	10.1346	1.09E-05	0.000857	4.040864	0.389967	0.594059	-0.20409	1	1.52E+08	F	II	LCE5A	TSS1500	opensea	TSS1500-op		rs5352463	1;7;29;36
cg0897642	0.227032	0.59023	10.13125	1.1E-05	0.000858	4.038445	0.476714	0.703746	-0.22703	4	1.55E+08	F	II		IGR	opensea	IGR-opense		rs5380372	15
cg1349476	0.255929	0.580787	10.13022	1.1E-05	0.000858	4.037698	0.452823	0.708751	-0.25593	1	2.44E+08	F	II	MIR4677	TSS1500	opensea	TSS1500-op		rs1924570	0;1
cg1677040	0.219042	0.630069	10.12976	1.1E-05	0.000858	4.037371	0.520548	0.73959	-0.21904	6	1.06E+08	F	II		IGR	opensea	IGR-opense		rs5716391	0
cg0855971	0.232766	0.454109	10.12976	1.1E-05	0.000858	4.037366	0.337727	0.570492	-0.23277	6	20978738	F	II	CDKAL1	Body	opensea	Body-opens		rs1893972	10;37;43
cg0315067	0.208438	0.529504	10.12938	1.1E-05	0.000858	4.037094	0.425285	0.633723	-0.20844	4	93186308	R	II	LOC10192	Body	opensea	Body-opens		rs1874233	2;1
cg0217068	0.246056	0.563739	10.12805	1.1E-05	0.000858	4.036136	0.440711	0.686766	-0.24606	2	60736594	F	II	BCL11A	Body	opensea	Body-opens			
cg1325913	0.203274	0.608154	10.12708	1.1E-05	0.000858	4.035435	0.506517	0.709791	-0.20327	18	29298554	R	II		IGR	opensea	IGR-opense		rs1446972	26;1
cg1786725	0.201265	0.736476	10.12701	1.1E-05	0.000858	4.035379	0.635844	0.837109	-0.20127	6	73364350	F	II	KCNQ5-IT1	Body	opensea	Body-opens			
cg1053671	0.267834	0.533585	10.12584	1.1E-05	0.000859	4.034538	0.399668	0.667502	-0.26783	2	86089041	R	II	ST3GAL5	Body	opensea	Body-opens		rs1436850	1
cg0865866	0.221319	0.664324	10.12572	1.1E-05	0.000859	4.034451	0.553665	0.774984	-0.22132	12	1.09E+08	F	II	SVOP	Body	opensea	Body-opens		rs5665863	19;45
cg2646553	0.205966	0.506703	10.12552	1.1E-05	0.000859	4.034306	0.40372	0.609686	-0.20597	10	79446426	F	II		IGR	opensea	IGR-opense		rs5602729	0;14;36;45
cg1643534	0.218999	0.449043	10.12372	1.1E-05	0.000859	4.033002	0.339544	0.558543	-0.219	13	1.07E+08	R	II		IGR	opensea	IGR-opense			
cg1535256	0.203494	0.686096	10.1236	1.1E-05	0.000859	4.032918	0.584349	0.787843	-0.20349	5	1.13E+08	R	II	MCC	Body	opensea	Body-opens		rs5726478	21;5
cg0705062	0.21936	0.515948	10.12346	1.1E-05	0.000859	4.032816	0.406268	0.625628	-0.21936	11	1.31E+08	R	II		IGR	opensea	IGR-opense		rs5476707	11;10;1
cg2476977	0.207114	0.627748	10.12295	1.1E-05	0.000859	4.032445	0.524191	0.731305	-0.20711	8	1.07E+08	F	II	OXR1	Body	opensea	Body-opens		rs5398726	10;49
cg2685686	0.210575	0.449462	10.12259	1.1E-05	0.000859	4.032188	0.344174	0.55475	-0.21058	11	11562311	F	II	GALNT18	Body	opensea	Body-opens			
cg1396398	0.239488	0.508846	10.12249	1.1E-05	0.000859	4.032115	0.389101	0.62859	-0.23949	1	2.05E+08	F	II		IGR	opensea	IGR-opense			
cg0574670	0.224921	0.564453	10.12164	1.1E-05	0.00086	4.031495	0.451992	0.676914	-0.22492	15	91221258	R	II	CRTC3-AS1	Body	opensea	Body-opens			
cg1736068	0.228256	0.619317	10.12155	1.1E-05	0.00086	4.03143	0.505189	0.733445	-0.22826	12	53069734	F	II	KRT1	Body	opensea	Body-opens		rs6651455	6;28;32;33;45
cg0081866	0.221686	0.506666	10.11983	1.1E-05	0.00086	4.030188	0.395823	0.617509	-0.22169	6	96752894	F	II		IGR	opensea	IGR-opense		rs1500512	12
cg0656440	0.207815	0.558746	10.11982	1.1E-05	0.00086	4.03018	0.454838	0.662653	-0.20782	9	1.36E+08	R	II	AK8	Body	opensea	Body-opens		rs1896269	39;38;19
cg1783618	0.209242	0.608626	10.11964	1.11E-05	0.00086	4.03005	0.504005	0.713247	-0.20924	21	36570494	F	II		IGR	opensea	IGR-opense		rs5651723	50;51
cg0550161	0.271245	0.495043	10.11903	1.11E-05	0.00086	4.029611	0.359421	0.630666	-0.27124	4	1.85E+08	R	II	IRF2	Body	opensea	Body-opens		rs6233775	34;13
cg2706628	0.207011	0.64975	10.11763	1.11E-05	0.000861	4.028599	0.546245	0.753256	-0.20701	1	1.62E+08	F	II	NOS1AP	Body	opensea	Body-opens		rs5304332	32;40;42
cg0547766	0.201187	0.703723	10.11743	1.11E-05	0.000861	4.028449	0.60313	0.804317	-0.20119	2	1.12E+08	F	II	LOC40099	TSS200	shore	TSS200-sho	chr2:1118	rs1862610	1;7;14;33
cg2713449	0.203117	0.447918	10.1172	1.11E-05	0.000861	4.028283	0.346359	0.549476	-0.20312	2	1.51E+08	R	II		IGR	opensea	IGR-opense		rs5376459	1
cg2220212	0.219668	0.45894	10.11612	1.11E-05	0.000861	4.027502	0.349106	0.568773	-0.21967	20	50547182	F	II		IGR	opensea	IGR-opense		rs1435090	1;23;25
cg0395267	0.268836	0.518275	10.11281	1.11E-05	0.000862	4.025107	0.383857	0.652693	-0.26884	21	45126620	F	II		IGR	opensea	IGR-opense		rs5581136	0;9;31;32
cg1185138	0.238616	0.506128	10.11275	1.11E-05	0.000862	4.025064	0.38682	0.625436	-0.23862	1	56996910	R	II	PPAP2B	Body	opensea	Body-opens		rs1180280	46;35;14;1
cg0358509	0.250328	0.547535	10.11154	1.11E-05	0.000863	4.024192	0.422371	0.672699	-0.25033	13	20287930	F	II	PSPC1	Body	opensea	Body-opens		rs1153460	0;24;34
cg2675466	0.212225	0.49147	10.10999	1.11E-05	0.000863	4.023068	0.385357	0.597583	-0.21223	11	1.25E+08	R	II	ROBO4	3'UTR	opensea	3'UTR-oper		rs5382698	50;30;1

cg0647669	0.209368	0.672093	10.10978	1.11E-05	0.000863	4.022912	0.567409	0.776777	-0.20937	11	1.3E+08	F	II	ADAMTS8	Body	opensea	Body-opens		rs1421741	1;37
cg1344919	0.212001	0.496621	10.109	1.11E-05	0.000863	4.022352	0.39062	0.602621	-0.212	13	73028751	F	II		IGR	opensea	IGR-opense		rs1846667	0;1
cg0806038	0.233143	0.580663	10.10797	1.11E-05	0.000863	4.021601	0.464091	0.697234	-0.23314	3	44637866	F	II		IGR	opensea	IGR-opense			
cg2562523	0.205646	0.557081	10.10752	1.11E-05	0.000863	4.021279	0.454258	0.659904	-0.20565	18	53304670	F	II	TCF4	TSS1500	opensea	TSS1500-op		rs5455126	0;27
cg0186402	0.219712	0.508234	10.10671	1.12E-05	0.000864	4.020692	0.398378	0.61809	-0.21971	15	1.02E+08	R	II		IGR	opensea	IGR-opense		rs1932433	19
cg0551999	0.206505	0.612391	10.1062	1.12E-05	0.000864	4.020319	0.509139	0.715644	-0.2065	11	94628792	R	II		IGR	opensea	IGR-opense			
cg0237467	0.207126	0.544188	10.10571	1.12E-05	0.000864	4.019967	0.440625	0.647751	-0.20713	18	34047739	R	II	FHOD3	Body	opensea	Body-opens		rs5321201	49;48;26
cg2546722	0.230779	0.539388	10.10329	1.12E-05	0.000864	4.018214	0.423998	0.654777	-0.23078	22	50218031	F	II	BRD1	5'UTR	shore	5'UTR-shore	chr22:502	rs5430723	29
cg2142163	0.203637	0.574555	10.10265	1.12E-05	0.000864	4.017746	0.472736	0.676374	-0.20364	1	2.04E+08	F	II	PLEKHA6	5'UTR	opensea	5'UTR-oper		rs5777281	1;20;42;46
cg2006364	0.208989	0.538059	10.10017	1.12E-05	0.000865	4.015951	0.433565	0.642554	-0.20899	20	2635105	R	II	NOP56	Body	shore	Body-shore	chr20:263	rs5726487	37;27;26
cg1537333	0.221334	0.558457	10.09465	1.12E-05	0.000868	4.011949	0.447791	0.669124	-0.22133	9	1.17E+08	R	II	COL27A1	Body	opensea	Body-opens		rs1914997	15;1
cg0970550	0.212103	0.650994	10.09452	1.12E-05	0.000868	4.011855	0.544943	0.757045	-0.2121	8	1.11E+08	F	II	SYBU	Body	opensea	Body-opens		rs5615227	1;41
cg0779551	0.213494	0.667228	10.09442	1.12E-05	0.000868	4.011785	0.560481	0.773975	-0.21349	8	1.42E+08	R	II	PTK2	Body	opensea	Body-opens		rs1902118	21;9
cg1564098	0.210113	0.465689	10.09332	1.13E-05	0.000868	4.010983	0.360632	0.570745	-0.21011	11	33434222	F	II		IGR	opensea	IGR-opense		rs5543156	3
cg1161135	0.226063	0.589138	10.09246	1.13E-05	0.000868	4.010362	0.476107	0.70217	-0.22606	1	56913679	R	II		IGR	opensea	IGR-opense		rs7577088	1
cg0936061	0.217105	0.572677	10.09174	1.13E-05	0.000868	4.009841	0.464125	0.68123	-0.21711	5	1.58E+08	R	II	EBF1	ExonBnd	opensea	ExonBnd-op		rs3739223	26;2;1
cg2149337	0.226532	0.495936	10.09151	1.13E-05	0.000868	4.009673	0.38267	0.609202	-0.22653	4	1.41E+08	F	II	SCOC	5'UTR	opensea	5'UTR-oper		rs1144346	1;24;38
cg2080400	0.20688	0.529444	10.09094	1.13E-05	0.000868	4.009257	0.426005	0.632884	-0.20688	3	13685146	F	II		IGR	opensea	IGR-opense		rs1132123	7;8;13;27;33
cg1965443	0.214249	0.556492	10.08986	1.13E-05	0.000868	4.008474	0.449367	0.663616	-0.21425	2	2.03E+08	R	II	CDK15	TSS200	opensea	TSS200-ope		rs5309332	49;1
cg2255777	0.2539	0.568425	10.08839	1.13E-05	0.000869	4.007408	0.441475	0.695376	-0.2539	11	36407062	R	II	PRR5L	5'UTR	opensea	5'UTR-oper		rs5357617	22;14;6
cg0647591	0.213188	0.565896	10.08635	1.13E-05	0.000869	4.005929	0.459302	0.67249	-0.21319	2	2.19E+08	R	II	RQCD1	Body	opensea	Body-opens		rs7936593	2
cg0968910	0.212684	0.616182	10.08574	1.13E-05	0.000869	4.005484	0.50984	0.722524	-0.21268	8	1.25E+08	R	II	FER1L6	5'UTR	opensea	5'UTR-oper			
cg1785835	0.267996	0.556698	10.08535	1.13E-05	0.000869	4.005203	0.4227	0.690696	-0.268	3	1.78E+08	R	II	KCNMB2-A	Body	opensea	Body-opens		rs5304138	40;25;13;2
cg2654472	0.236888	0.504517	10.08492	1.13E-05	0.000869	4.00489	0.386072	0.622961	-0.23689	4	65883972	F	II		IGR	opensea	IGR-opense		rs1428737	10;15;20
cg1037907	0.232433	0.578745	10.08463	1.13E-05	0.000869	4.004679	0.462529	0.694962	-0.23243	16	11076864	F	II	CLEC16A	Body	opensea	Body-opens		rs2011164	0;1
cg0004884	0.212575	0.47539	10.08319	1.13E-05	0.00087	4.00363	0.369103	0.581678	-0.21258	12	72473584	F	II		IGR	opensea	IGR-opense		rs7481614	0;18;23;28
cg1744996	0.242242	0.614582	10.08262	1.13E-05	0.00087	4.003221	0.493461	0.735704	-0.24224	7	1.27E+08	R	II	SND1	Body	opensea	Body-opens		rs3808106	44;43;19;15;2
cg0142571	0.222865	0.547875	10.08185	1.13E-05	0.00087	4.002655	0.436443	0.659308	-0.22287	1	81974159	R	II		IGR	opensea	IGR-opense		rs7895772	28;1
cg1517694	0.200526	0.443857	10.08132	1.14E-05	0.00087	4.002271	0.343594	0.54412	-0.20053	15	55569970	R	II	RAB27A	5'UTR	opensea	5'UTR-oper		rs1855015	40;1
cg1962860	0.209689	0.605585	10.08095	1.14E-05	0.00087	4.002008	0.500741	0.71043	-0.20969	3	71032216	R	II	FOXP1	Body	opensea	Body-opens		rs5449100	22;15;12;5
cg2159313	0.214302	0.625815	10.07995	1.14E-05	0.00087	4.001278	0.518664	0.732966	-0.2143	1	14844052	R	II		IGR	opensea	IGR-opense		rs5564002	28;1
cg1271036	0.20616	0.619861	10.0786	1.14E-05	0.00087	4.000298	0.516781	0.722941	-0.20616	6	64486271	F	II	EYS	Body	opensea	Body-opens		rs5319727	10
cg0875051	0.251943	0.510264	10.07755	1.14E-05	0.000871	3.999534	0.384293	0.636236	-0.25194	5	32537617	R	II		IGR	opensea	IGR-opense		rs1909435	29;27;13
cg0536889	0.238512	0.45425	10.07448	1.14E-05	0.000872	3.997307	0.334994	0.573507	-0.23851	1	1760171	R	II	GNB1	5'UTR	opensea	5'UTR-oper		rs1448951	47;44;32
cg0607257	0.21033	0.743045	10.07403	1.14E-05	0.000872	3.996977	0.63788	0.848209	-0.21033	12	10466134	R	II	KLRD1	Body	opensea	Body-opens		rs5570262	7;5
cg0582233	0.210121	0.588893	10.07355	1.14E-05	0.000872	3.996631	0.483832	0.693953	-0.21012	1	1.71E+08	R	II	FMO2	3'UTR	opensea	3'UTR-oper		rs1860992	50;2
cg1925093	0.221647	0.565298	10.07298	1.14E-05	0.000872	3.996214	0.454474	0.676121	-0.22165	20	1800881	R	II		IGR	opensea	IGR-opense		rs1888519	39;21;1
cg1152121	0.254778	0.615226	10.07007	1.14E-05	0.000873	3.994097	0.487837	0.742615	-0.25478	4	75366983	R	II		IGR	opensea	IGR-opense		rs7286118	39;27;14;2
cg1171960	0.234432	0.666414	10.06822	1.15E-05	0.000874	3.992753	0.549198	0.78363	-0.23443	14	51910138	R	II		IGR	opensea	IGR-opense		rs5693319	49;2
cg1761787	0.215204	0.524578	10.06796	1.15E-05	0.000874	3.992565	0.416975	0.63218	-0.2152	8	93434899	R	II		IGR	opensea	IGR-opense		rs5343275	21;5;1
cg2328204	0.224923	0.506009	10.06609	1.15E-05	0.000874	3.991207	0.393547	0.61847	-0.22492	17	39950347	F	II		IGR	opensea	IGR-opense		rs5289168	14;41
cg1719748	0.209913	0.493579	10.06232	1.15E-05	0.000875	3.988461	0.388623	0.598536	-0.20991	16	56483905	R	II	NUDT21	Body	shore	Body-shore	chr16:564		
cg0292348	0.206852	0.61496	10.06209	1.15E-05	0.000875	3.988295	0.511534	0.718387	-0.20685	1	2.24E+08	F	II	CAPN2	Body	opensea	Body-opens		rs5501610	1;11;13;37;45
cg2348978	0.200623	0.557037	10.06095	1.15E-05	0.000876	3.987462	0.456726	0.657348	-0.20062	8	80558042	F	II	STMN2	Body	opensea	Body-opens		rs5754301	0
cg1406992	0.201332	0.600165	10.06073	1.15E-05	0.000876	3.987308	0.4995	0.700831	-0.20133	14	1.07E+08	F	II		IGR	opensea	IGR-opense		rs1137256	12;17
cg2188496	0.218556	0.541612	10.05861	1.15E-05	0.000876	3.985763	0.432334	0.650889	-0.21856	2	2.18E+08	R	II	DIRC3	Body	opensea	Body-opens		rs5684500	40;35;1
cg1549354	0.204833	0.69942	10.05802	1.15E-05	0.000876	3.985331	0.597004	0.801837	-0.20483	4	1.85E+08	R	II	LOC10272	Body	opensea	Body-opens		rs5431581	42;15;1
cg2236336	0.221075	0.470848	10.05632	1.16E-05	0.000876	3.984093	0.36031	0.581385	-0.22108	2	59126860	F	II		IGR	opensea	IGR-opense		rs1139974	27;29
cg2208562	0.205148	0.515426	10.05559	1.16E-05	0.000876	3.983564	0.412852	0.618001	-0.20515	1	16091218	R	II	FBLIM1	TSS1500	opensea	TSS1500-op		rs5686476	45;5
cg0758849	0.20644	0.510771	10.05551	1.16E-05	0.000876	3.983505	0.407552	0.613991	-0.20644	5	1.42E+08	F	II		IGR	opensea	IGR-opense			
cg1744819	0.253768	0.403663	10.05542	1.16E-05	0.000876	3.983437	0.276779	0.530547	-0.25377	3	1.87E+08	R	II	ST6GAL1	5'UTR	opensea	5'UTR-oper		rs1848526	29;25
cg2023939	0.207356	0.537605	10.05528	1.16E-05	0.000876	3.983334	0.433927	0.641283	-0.20736	20	45030732	R	II	ELMO2	5'UTR	shelf	5'UTR-shelf	chr20:450		
cg1204667	0.202901	0.554941	10.05503	1.16E-05	0.000876	3.983155	0.453491	0.656391	-0.2029	12	2360663	F	II	CACNA1C	Body	opensea	Body-opens		rs1170686	0;5
cg2715625	0.21608	0.621844	10.05314	1.16E-05	0.000877	3.981776	0.513804	0.729883	-0.21608	12	1.21E+08	F	II	PXN	Body	opensea	Body-opens		rs5487413	12;19
cg1920306	0.206101	0.584382	10.05177	1.16E-05	0.000877	3.980781	0.481331	0.687432	-0.2061	1	95145415	R	II	LINC01057	Body	opensea	Body-opens		rs841354	13
cg1845177	0.201296	0.669728	10.05083	1.16E-05	0.000877	3.980094	0.56908	0.770376	-0.2013	21	40291514	F	II	LOC40086	Body	opensea	Body-opens		rs5325093	0;15;19
cg1851226	0.207008	0.577163	10.0504	1.16E-05	0.000877	3.979783	0.473659	0.680667	-0.20701	11	6049729	R	II	OR56A1	TSS1500	opensea	TSS1500-op		rs5646212	30;2;1
cg0742680	0.200531	0.372007	10.05026	1.16E-05	0.000877	3.97968	0.271741	0.472272	-0.20053	8	60885377	R	II		IGR	opensea	IGR-opense		rs1407293	43;41;36;34
cg0455071	0.212415	0.555124	10.04775	1.16E-05	0.000878	3.977853	0.448917	0.661332	-0.21242	5	1.41E+08	F	II		IGR	opensea	IGR-opense		rs5616819	46
cg0387681	0.253825	0.539889	10.04764	1.16E-05	0.000878	3.977771	0.412977	0.666801	-0.25382	1	2.44E+08	R	II	AKT3	Body	opensea	Body-opens			
cg0494728	0.204851	0.614739	10.04549	1.16E-05	0.000879	3.976202	0.512313	0.717164	-0.20485	15	43360134	F	II	UBR1	Body	opensea	Body-opens		rs1506170	8;25;40
cg2031810	0.217483	0.63102	10.04479	1.17E-05	0.000879	3.975693	0.522279	0.739761	-0.21748	1	2.1E+08	R	II		IGR	opensea	IGR-opense		rs1437442	39;15
cg2177377	0.212844	0.628596	10.04292	1.17E-05	0.000879	3.974328	0.522174	0.735017	-0.21284	10	5237570	R	II	AKR1C4	TSS1500	opensea	TSS1500-op		rs5352043	46;1
cg0578168	0.207022	0.613893	10.04222	1.17E-05	0.000879	3.973818	0.510382	0.717404	-0.20702	2	2.38E+08	R	II		IGR	opensea	IGR-opense		rs1907813	5;2;1
cg0503045	0.237876	0.47048	10.0417	1.17E-05	0.00088	3.973442	0.351542	0.589418	-0.23788	5	1.67E+08	F	II	ODZ2	Body	opensea	Body-opens		rs5702992	0;27;28;42
cg2210410	0.247445	0.574408	10.03691	1.17E-05	0.000881	3.96995	0.450685	0.698131	-0.24745	21	17565450	F	II	C21orf34	Body	opensea	Body-opens		rs5722588	1;27
cg0173693	0.229159	0.555572	10.03625	1.17E-05	0.000881	3.969469	0.440992	0.670151	-0.22916	2	1.06E+08	F	II	FHL2	TSS200	opensea	TSS200-ope		rs1122585	4;12;13
cg1182091	0.200929	0.566745	10.03039	1.18E-05	0.000882	3.965192	0.46628	0.667209	-0.20093	14	1.01E+08	F	II	BEGAIN	Body	opensea	Body-opens		rs5732874	10
cg2485231	0.258975	0.531645	10.03032	1.18E-05	0.000882	3.965141	0.402157	0.661132	-0.25897	6	1.13E+08	F	II		IGR	opensea	IGR-opense		rs5397393	25
cg2370631	0.234888	0.55691	10.02843	1.18E-05	0.000883	3.963756	0.439466	0.674353	-0.23489	5	1.38E+08	F	II	KDM3B	Body	opensea	Body-opens			
cg1606165	0.249562	0.497343	10.02755	1.18E-05	0.000884	3.963116	0.372561	0.622124	-0.24956	11	1.15E+08	R	II	CADM1	Body	opensea	Body-opens		rs7357213	51;34;11;1
cg1014929	0.221053	0.675752	10.0261	1.18E-05	0.000884	3.962057	0.565226	0.786279	-0.22105	2	20795912	R	II		IGR	opensea	IGR-opense		rs3743127	1
cg0070435	0.214307	0.634897	10.02577	1.18E-05	0.000884	3.961815	0.527743	0.74205	-0.21431	14	73869495	R	II	NUMB	5'UTR	opensea	5'UTR-oper			
cg0258221	0.207751	0.634739	10.02524	1.18E-05	0.000884	3.961429	0.530863	0.738615	-0.20775	2	59569659	R	II		IGR	opensea	IGR-opense		rs5423375	22;2
cg0589085	0.238152	0.473904	10.02405	1.18E-05	0.000885	3.960562	0.354828	0.59298	-0.23815	2	2.17E+08	R	II		IGR	opensea	IGR-opense		rs5586543	29;2
cg2372640	0.230444	0.663277	10.0236	1.18E-05	0.000885	3.960231	0.548055	0.778499	-0.23044	3	1.23E+08	F	II	MYLK	Body	opensea	Body-opens		rs1412857	0;22;43
cg0060337	0.236571	0.65577	10.02159	1.18E-05	0.000885	3.958761	0.537485	0.774056	-0.23657	1	1.11E+08	R	II		IGR	opensea	IGR-opense		rs1702586	23
cg0929501	0.223477	0.588621	10.02096	1.19E-05	0.000886	3.958305	0.476882	0.70036	-0.22348	8	99503021	F	II	STK3	Body	opensea	Body-opens		rs1828935	0;30;31
cg1378380	0.212125	0.671954	10.02058	1.19E-05	0.000886	3.958026	0.565891	0.778016	-0.21212	19	6489371	R	II		IGR	opensea	IGR-opense		rs7926943	39;35;28;18
cg0771283	0.208558	0.534464	10.0201	1.19E-05	0.000886	3.957672	0.430185	0.638743	-0.20856	18	74034689	F	II		IGR	opensea	IGR-opense		rs5617251	22;47
cg1279499	0.221172	0.598851	10.01916	1.19E-05	0.000886	3.956986	0.488265	0.709436	-0.22117	8	1.22E+08	F	II		IGR	opensea	IGR-opense			
cg0949788	0.221044	0.438812	10.0191	1.19E-05	0.000886	3.956942	0.32829	0.549334	-0.22104	9	95918949	R	II		IGR	opensea	IGR-opense		rs1923145	3
cg1984724	0.202787	0.64716	10.01775	1.19E-05	0.000886	3.955955	0.545767	0.748553	-0.20279	15	58246538	R	II	ALDH1A2	3'UTR	opensea	3'UTR-oper		rs1904748	33
cg0844433	0.212593	0.615155	10.01747	1.19E-05	0.000886	3.95575	0.508859	0.721451	-0.21259	6	38463839	R	II	BTBD9	Body	opensea	Body-opens		rs1868424	10;1
cg0965513	0.232503	0.454139	10.01614	1.19E-05	0.000886	3.954776	0.337887	0.57039	-0.2325	5	1.08E+08	R	II		IGR	opensea	IGR-opense		rs7784060	33;10
cg0779957	0.2289	0.526637	10.01603	1.19E-05	0.000886	3.954699	0.412187	0.641087	-0.2289	5	70309843	R	II	NAIP	Body	opensea	Body-opens		rs5691943	4
cg1217668	0.243132	0.603617	10.01526	1.19E-05	0.000886	3.954134	0.482051	0.725183	-0.24313	20	23018803	R	II		IGR	shore	IGR-shore	chr20:230	rs5463990	48
cg1813032	0.242999	0.587272	10.01486	1.19E-05	0.000886	3.953841	0.465773	0.708772	-0.243	12	65720609	R	II	MSRB3	ExonBnd	opensea	ExonBnd-op		rs1467330	41;7;1
cg0972298	0.21916	0.674974	10.01441	1.19E-05	0.000886	3.953512	0.565394	0.784554	-0.21916	12	26150525	F	II	RASSF8	5'UTR	opensea	5'UTR-oper			
cg1005038	0.219252	0.643194	10.01405	1.19E-05	0.000886	3.953248	0.533568	0.75282	-0.21925	6	1.65E+08	R	II		IGR	opensea	IGR-opense			
cg1927307	0.213303	0.609561	10.01398	1.19E-05	0.000886	3.953198	0.502909	0.716212	-0.2133	12	41832738	F	II	PDZRN4	Body	opensea	Body-opens		rs1123502	5;23;51
cg0495147	0.227074	0.607226	10.01244	1.19E-05	0.000887	3.952075	0.493689	0.720763	-0.22707	6	3848243	R	II	FAM50B	TSS1500	shore	TSS1500-sh	chr6:3849	rs5363587	51;2
cg2215286	0.262429	0.560499	10.01243	1.19E-05	0.000887	3.952064	0.429285	0.691714	-0.26243	22	24596179	R	II		IGR	opensea	IGR-opense			
cg0141092	0.25789	0.659851	10.01223	1.19E-05	0.000887	3.951922	0.530906	0.788796	-0.25789	2	1.92E+08	R	II	MYO1B	Body	opensea	Body-opens		rs1488319	51;31
cg0348697	0.207169	0.660594	10.01094	1.19E-05	0.000887	3.950976	0.557009	0.764178	-0.20717	2	2.03E+08	F	II		IGR	opensea	IGR-opense		rs5752636	1
cg2439138	0.209435	0.427322	10.01065	1.19E-05	0.000887	3.950762	0.322605	0.532039	-0.20943	2	65526509	R	II		IGR	opensea	IGR-opense		rs5602028	33
cg2414031	0.268857	0.546892	10.00996	1.19E-05	0.000887	3.950262	0.412463	0.68132	-0.26886	1	1.85E+08	R	II		IGR	opensea	IGR-opense		rs1503022	17;1
cg0331780	0.230603	0.602107	10.00867	1.2E-05	0.000887	3.949319	0.486805	0.717409	-0.2306	14	56331298	R	II		IGR	opensea	IGR-opense			
cg1346390	0.209594	0.650955	10.00793	1.2E-05	0.000887	3.948775	0.546158	0.755752	-0.20959	5	1.48E+08	R	II		IGR	opensea	IGR-opense		rs5518989	46;16;2;1
cg1968280	0.223028	0.679315	10.00698	1.2E-05	0.000888	3.94808	0.567801	0.790829	-0.22303	8	1.03E+08	F	II	NCALD	5'UTR	opensea	5'UTR-oper		rs5481098	13
cg2494357	0.211036	0.483959	10.00652	1.2E-05	0.000888	3.947743	0.378441	0.589476	-0.21104	2	15933717	F	II		IGR	opensea	IGR-opense		rs5494968	6;39;51
cg1257157	0.227343	0.635547	10.00646	1.2E-05	0.000888	3.947696	0.521876	0.749218	-0.22734	2	1.45E+08	R	II	ZEB2	Body	opensea	Body-opens		rs3756300	43;36
cg0417769	0.207487	0.459996	10.00571	1.2E-05	0.000888	3.947152	0.356253	0.563739	-0.20749	8	40850062	F	II		IGR	opensea	IGR-opense		rs1917635	0;14;16;48
cg1101549	0.20051	0.496055	10.00532	1.2E-05	0.000888	3.946863	0.3958	0.59631	-0.20051	2	38750277	F	II		IGR	opensea	IGR-opense		rs7748995	10;11;22;48
cg2049208	0.232778	0.549019	10.00476	1.2E-05	0.000888	3.946455	0.43263	0.665408	-0.23278	5	79112509	F	II		IGR	opensea	IGR-opense		rs1265503	1;28;42
cg0158282	0.256996	0.584148	10.00319	1.2E-05	0.000888	3.945303	0.45565	0.712646	-0.257	18	41825318	R	II		IGR	opensea	IGR-opense		rs1173793	2
cg1652420	0.215985	0.533804	10.0031	1.2E-05	0.000888	3.945242	0.425811	0.641797	-0.21599	12	71849440	R	II	LGR5	Body	opensea	Body-opens			
cg1761450	0.222432	0.409072	10.00285	1.2E-05	0.000888	3.945056	0.297856	0.520288	-0.22243	7	47803072	F	II		IGR	opensea	IGR-opense		rs1827841	3;10;38
cg1705982	0.240083	0.524512	10.00155	1.2E-05	0.000888	3.944103	0.40447	0.644554	-0.24008	15	39933961	R	II	FSIP1	Body	opensea	Body-opens		rs2898686	50
cg0929085	0.206813	0.56107	10.00107	1.2E-05	0.000889	3.943753	0.457664	0.664477	-0.20681	2	29373296	R	II	CLIP4	Body	opensea	Body-opens		rs5628350	36
cg1990306	0.231256	0.53181	9.997911	1.2E-05	0.00089	3.94144	0.416182	0.647438	-0.23126	7	1.11E+08	R	II	DOCK4	Body	opensea	Body-opens		rs7343208	28;25
cg0370800	0.223085	0.697126	9.997642	1.2E-05	0.00089	3.941243	0.585584	0.808669	-0.22309	18	52937080	R	II	TCF4	ExonBnd	opensea	ExonBnd-op		rs5507361	35
cg1675495	0.207148	0.641698	9.996013	1.21E-05	0.00089	3.940049	0.538124	0.745272	-0.20715	7	83932562	R	II		IGR	opensea	IGR-opense		rs5506667	44;43;32;1
cg1446094	0.216831	0.494326	9.995958	1.21E-05	0.00089	3.940009	0.38591	0.602741	-0.21683	18	37646175	R	II	LINC01477	TSS200	opensea	TSS200-ope		rs5685394	43;33
cg1189531	0.218751	0.6026	9.995245	1.21E-05	0.00089	3.939487	0.493224	0.711976	-0.21875	21	37268698	R	II		IGR	opensea	IGR-opense		rs5636874	4;3;2;1
cg0120872	0.239932	0.451298	9.994919	1.21E-05	0.000891	3.939248	0.331332	0.571264	-0.23993	5	6829916	F	II		IGR	opensea	IGR-opense			
cg1411413	0.200892	0.38297	9.993978	1.21E-05	0.000891	3.938558	0.282524	0.483416	-0.20089	6	10839408	F	II		IGR	shore	IGR-shore	chr6:1083	rs1923033	0;8
cg0340877	0.22895	0.551449	9.993947	1.21E-05	0.000891	3.938536	0.436974	0.665924	-0.22895	2	1.44E+08	R	II	ARHGAP15	Body	opensea	Body-opens		rs5766398	15;13
cg0304816	0.231438	0.463929	9.992963	1.21E-05	0.000891	3.937815	0.34821	0.579648	-0.23144	17	47558681	R	II		IGR	opensea	IGR-opense		rs5622757	25;5

cg0115010	0.228934	0.570087	9.992939	1.21E-05	0.000891	3.937797	0.45562	0.684554	-0.22893	3	1.75E+08	R	II	NAALADL2	Body	opensea	Body-opens		rs3733625	44;2;1
cg0952284	0.216333	0.684504	9.992799	1.21E-05	0.000891	3.937694	0.576338	0.792671	-0.21633	10	71613456	R	II	COL13A1	Body	opensea	Body-opens		rs1859084	11;4
cg0522457	0.20723	0.50837	9.992746	1.21E-05	0.000891	3.937656	0.404755	0.611985	-0.20723	15	45381921	R	II		IGR	opensea	IGR-opense		rs1918332	36
cg0251500	0.210424	0.517858	9.992523	1.21E-05	0.000891	3.937492	0.412646	0.62307	-0.21042	3	53569971	F	II	CACNA1D	Body	opensea	Body-opens		rs5281491	42
cg1685920	0.211318	0.499557	9.99236	1.21E-05	0.000891	3.937373	0.393898	0.605216	-0.21132	16	5022748	R	II	SEC14L5	Body	opensea	Body-opens		rs1428255	47;36;18;17;15;13;1
cg1962089	0.220839	0.484155	9.991186	1.21E-05	0.000891	3.936512	0.373735	0.594574	-0.22084	6	27694665	F	II		IGR	opensea	IGR-opense			
cg0877666	0.21683	0.520921	9.989664	1.21E-05	0.000892	3.935397	0.412506	0.629336	-0.21683	16	1670473	R	II	CRAMP1L	Body	opensea	Body-opens		rs1900052	38
cg1461723	0.231853	0.559265	9.988779	1.21E-05	0.000892	3.934748	0.443338	0.675191	-0.23185	6	1.39E+08	R	II	REPS1	Body	opensea	Body-opens		rs5373672	51;31
cg1824133	0.209938	0.437624	9.984557	1.22E-05	0.000893	3.931652	0.332655	0.542593	-0.20994	3	1.56E+08	R	II	SSR3	Body	shore	Body-shore	chr3:1562	rs5715222	2
cg0412472	0.223487	0.582935	9.983652	1.22E-05	0.000893	3.930988	0.471192	0.694678	-0.22349	7	79086680	R	II	MAGI2-AS3	Body	shelf	Body-shelf	chr7:7908		
cg0229993	0.21795	0.483284	9.981054	1.22E-05	0.000894	3.929082	0.37431	0.592259	-0.21795	17	4631697	R	II		IGR	shelf	IGR-shelf	chr17:463	rs1395372	34;21
cg0494537	0.239141	0.588768	9.980702	1.22E-05	0.000894	3.928824	0.469197	0.708338	-0.23914	16	10970200	R	II	CIITA	TSS1500	shelf	TSS1500-sh	chr16:109	rs5564576	50;32;29;2;1
cg0572729	0.221662	0.502025	9.979366	1.22E-05	0.000894	3.927844	0.391194	0.612856	-0.22166	6	44929009	F	II	SUPT3H	Body	opensea	Body-opens			
cg1417899	0.244864	0.542737	9.979165	1.22E-05	0.000894	3.927696	0.420306	0.665169	-0.24486	4	76440699	R	II	RCHY1	TSS1500	shore	TSS1500-sh	chr4:7643	rs1402333	24;5
cg1612010	0.234455	0.523795	9.978597	1.22E-05	0.000894	3.92728	0.406567	0.641022	-0.23446	7	93112319	R	II	MIR653	TSS200	opensea	TSS200-ope		rs5643076	30;1
cg1884490	0.207669	0.653864	9.978559	1.22E-05	0.000894	3.927252	0.55003	0.757698	-0.20767	18	12742595	F	II	LOC10099	Body	opensea	Body-opens		rs1421013	0
cg1288273	0.219908	0.386222	9.97673	1.22E-05	0.000895	3.92591	0.276268	0.496176	-0.21991	10	11251269	R	II	CELF2	Body	opensea	Body-opens			
cg0398502	0.220752	0.59195	9.975273	1.22E-05	0.000895	3.92484	0.481574	0.702326	-0.22075	1	1.87E+08	R	II		IGR	opensea	IGR-opense		rs5618600	32
cg0205626	0.209455	0.629153	9.975258	1.22E-05	0.000895	3.924828	0.524425	0.73388	-0.20946	1	2.48E+08	R	II	OR2T8	TSS1500	opensea	TSS1500-op		rs5756982	13
cg2066343	0.249296	0.458461	9.97511	1.22E-05	0.000895	3.92472	0.333813	0.583109	-0.2493	12	5696809	F	II	ANO2	Body	opensea	Body-opens		rs5295548	40;48
cg0473539	0.260336	0.594208	9.974545	1.23E-05	0.000895	3.924305	0.46404	0.724377	-0.26034	3	35906672	R	II		IGR	opensea	IGR-opense		rs1432372	50;15
cg0639355	0.206223	0.448372	9.973101	1.23E-05	0.000896	3.923245	0.34526	0.551483	-0.20622	11	69982916	R	II	ANO1	Body	opensea	Body-opens			
cg0338230	0.210447	0.596047	9.972934	1.23E-05	0.000896	3.923122	0.490823	0.70127	-0.21045	15	66097959	R	II		IGR	opensea	IGR-opense			
cg1955885	0.249987	0.370464	9.971747	1.23E-05	0.000896	3.922251	0.245471	0.495458	-0.24999	15	60507509	R	II		IGR	opensea	IGR-opense			
cg1403488	0.205951	0.581961	9.971417	1.23E-05	0.000896	3.922009	0.478985	0.684936	-0.20595	3	37637413	R	II	ITGA9	Body	opensea	Body-opens			
cg2753192	0.251255	0.432661	9.970952	1.23E-05	0.000896	3.921667	0.307033	0.558288	-0.25126	13	98598462	R	II		IGR	opensea	IGR-opense		rs5741080	1
cg2373248	0.225972	0.422971	9.970311	1.23E-05	0.000896	3.921196	0.309984	0.535957	-0.22597	3	48965611	F	II	ARIH2	Body	opensea	Body-opens		rs5640411	18
cg2706224	0.2011	0.546431	9.967817	1.23E-05	0.000896	3.919364	0.445881	0.64698	-0.2011	10	1.15E+08	R	II	TCF7L2	Body	opensea	Body-opens		rs5521350	43
cg0253679	0.254918	0.518048	9.96765	1.23E-05	0.000896	3.919241	0.390589	0.645507	-0.25492	5	1.68E+08	R	II	TENM2	Body	opensea	Body-opens		rs5635933	17;1
cg2680230	0.220669	0.692859	9.966059	1.23E-05	0.000897	3.918072	0.582525	0.803194	-0.22067	11	61042752	F	II	VWCE	Body	opensea	Body-opens		rs5645552	0;8;23
cg2664680	0.204983	0.697665	9.964899	1.23E-05	0.000897	3.91722	0.595174	0.800157	-0.20498	5	68785451	R	II		IGR	shelf	IGR-shelf	chr5:6878	rs1871023	49;8
cg2617833	0.200357	0.348977	9.964858	1.23E-05	0.000897	3.91719	0.248799	0.449156	-0.20036	4	78508595	F	II	CXCL13	5'UTR	opensea	5'UTR-oper		rs5578376	0
cg1907149	0.228868	0.553489	9.963882	1.23E-05	0.000898	3.916473	0.439055	0.667924	-0.22887	1	63604560	F	II		IGR	opensea	IGR-opense		rs1118257	1
cg2512942	0.230497	0.538738	9.963673	1.23E-05	0.000898	3.916318	0.42349	0.653987	-0.2305	3	1.41E+08	R	II		IGR	opensea	IGR-opense		rs5393237	50;46;43;33;17
cg0861276	0.200025	0.619628	9.962528	1.24E-05	0.000898	3.915477	0.519616	0.719641	-0.20002	3	36449636	R	II	STAC	Body	opensea	Body-opens		rs1138406	32
cg2070646	0.203111	0.66868	9.962374	1.24E-05	0.000898	3.915364	0.567125	0.770235	-0.20311	6	1.11E+08	F	II		IGR	opensea	IGR-opense		rs1810031	0
cg2032478	0.217092	0.632078	9.961142	1.24E-05	0.000899	3.914459	0.523532	0.740624	-0.21709	10	74436268	F	II		IGR	opensea	IGR-opense		rs5385150	38
cg0631362	0.210596	0.613831	9.960975	1.24E-05	0.000899	3.914335	0.508533	0.719129	-0.2106	18	72146136	F	II		IGR	opensea	IGR-opense			
cg0780769	0.205338	0.459337	9.960249	1.24E-05	0.000899	3.913802	0.356669	0.562006	-0.20534	9	75406192	R	II	TMC1	Body	opensea	Body-opens		rs5571643	3
cg1984814	0.227196	0.608918	9.959139	1.24E-05	0.0009	3.912985	0.49532	0.722516	-0.2272	2	1.69E+08	R	II	STK39	Body	opensea	Body-opens		rs1409336	2
cg1814715	0.232255	0.578208	9.957132	1.24E-05	0.0009	3.91151	0.46208	0.694335	-0.23226	10	92683987	R	II		IGR	opensea	IGR-opense		rs5781180	2;1
cg2108022	0.226643	0.569927	9.956903	1.24E-05	0.0009	3.911341	0.456606	0.683248	-0.22664	11	95933005	R	II	MAML2	Body	opensea	Body-opens		rs1898167	20
cg1556715	0.221597	0.56858	9.956645	1.24E-05	0.0009	3.911152	0.457781	0.679378	-0.2216	8	75014592	F	II		IGR	opensea	IGR-opense		rs1009061	6;34
cg1607293	0.212114	0.613169	9.956243	1.24E-05	0.0009	3.910855	0.507112	0.719226	-0.21211	5	64344256	F	II		IGR	opensea	IGR-opense		rs1882792	0;10;15;31
cg1664957	0.222688	0.510689	9.954858	1.24E-05	0.000901	3.909837	0.399344	0.622033	-0.22269	13	85182015	R	II		IGR	opensea	IGR-opense		rs1451908	37
cg1266113	0.207077	0.634296	9.953373	1.24E-05	0.000901	3.908744	0.530757	0.737834	-0.20708	10	11759774	R	II		IGR	opensea	IGR-opense		rs7511215	45
cg2038859	0.2084	0.547812	9.953336	1.24E-05	0.000901	3.908717	0.443612	0.652012	-0.2084	10	36915266	F	II		IGR	opensea	IGR-opense		rs5462803	11;28;50
cg1352425	0.209551	0.591253	9.95327	1.24E-05	0.000901	3.908668	0.486478	0.696029	-0.20955	10	80698358	R	II		IGR	opensea	IGR-opense		rs1924181	26;19;2
cg1720284	0.248394	0.576072	9.95302	1.24E-05	0.000901	3.908485	0.451875	0.700269	-0.24839	13	43294719	F	II		IGR	opensea	IGR-opense		rs1501921	19
cg0644224	0.225862	0.591242	9.952075	1.24E-05	0.000901	3.90779	0.478311	0.704173	-0.22586	16	19129228	R	II	ITPRIPL2	1stExon	shore	1stExon-sh	chr16:191	rs5577708	2
cg2141606	0.252018	0.515575	9.951002	1.25E-05	0.000901	3.907	0.389566	0.641584	-0.25202	7	81645196	F	II	LOC10192	Body	opensea	Body-opens			
cg2290008	0.237406	0.5762	9.94968	1.25E-05	0.000902	3.906026	0.457497	0.694903	-0.23741	3	1.01E+08	F	II		IGR	opensea	IGR-opense		rs1854332	0;1;29
cg2696715	0.204891	0.659421	9.947749	1.25E-05	0.000902	3.904605	0.556976	0.761867	-0.20489	13	45062312	F	II	TSC22D1	Body	opensea	Body-opens		rs1881057	19;37
cg0327688	0.222785	0.42825	9.946582	1.25E-05	0.000902	3.903746	0.316857	0.539643	-0.22279	4	1.04E+08	F	II	BDH2	TSS1500	opensea	TSS1500-op		rs1814926	1;50
cg0536338	0.211939	0.452786	9.946098	1.25E-05	0.000902	3.90339	0.346816	0.558755	-0.21194	17	45767653	R	II		IGR	shelf	IGR-shelf	chr17:457		
cg0002290	0.216499	0.555671	9.944247	1.25E-05	0.000903	3.902027	0.447421	0.66392	-0.2165	8	94143534	R	II		IGR	opensea	IGR-opense		rs1822086	38
cg1048344	0.205834	0.587752	9.943787	1.25E-05	0.000903	3.901687	0.484836	0.690669	-0.20583	13	87782107	F	II		IGR	opensea	IGR-opense		rs5636335	1;21
cg2153275	0.22719	0.639782	9.943716	1.25E-05	0.000903	3.901636	0.526187	0.753378	-0.22719	9	1.08E+08	F	II	ABCA1	Body	opensea	Body-opens		rs5427660	18
cg0799358	0.237397	0.568674	9.943162	1.25E-05	0.000903	3.901227	0.449975	0.687372	-0.2374	7	1.57E+08	F	II	DNAJB6	Body	shore	Body-shore	chr7:1572	rs5777873	0;6;41
cg2002889	0.202647	0.4163	9.943082	1.25E-05	0.000903	3.901169	0.314976	0.517623	-0.20265	4	1.14E+08	R	II	ANK2	5'UTR	opensea	5'UTR-oper		rs1406853	2
cg0802794	0.213564	0.557812	9.942715	1.25E-05	0.000903	3.900898	0.45103	0.664593	-0.21356	14	53833982	F	II		IGR	opensea	IGR-opense		rs5710567	0;1;11;23;30
cg0630489	0.207075	0.57833	9.942451	1.25E-05	0.000903	3.900704	0.474793	0.681868	-0.20708	5	63870294	R	II	RGS7BP	TSS1500	opensea	TSS1500-op		rs5511678	40;4
cg1936278	0.205142	0.637654	9.94212	1.25E-05	0.000903	3.90046	0.535083	0.740225	-0.20514	9	82046357	F	II		IGR	opensea	IGR-opense			
cg0365158	0.225789	0.604382	9.938971	1.26E-05	0.000905	3.89814	0.491487	0.717276	-0.22579	7	40879777	R	II	SUGCT	Body	opensea	Body-opens			
cg0565887	0.209228	0.645668	9.936539	1.26E-05	0.000905	3.896348	0.541054	0.750282	-0.20923	4	74910375	F	II		IGR	opensea	IGR-opense		rs1855008	15
cg1366277	0.200012	0.555246	9.935952	1.26E-05	0.000905	3.895915	0.45524	0.655252	-0.20001	4	1.56E+08	R	II	FGG	5'UTR	opensea	5'UTR-oper			
cg2127271	0.212191	0.536405	9.934875	1.26E-05	0.000906	3.895121	0.430309	0.6425	-0.21219	12	79194558	R	II		IGR	shelf	IGR-shelf	chr12:791		
cg0737039	0.203879	0.595506	9.932712	1.26E-05	0.000906	3.893527	0.493567	0.697445	-0.20388	10	8704402	R	II		IGR	opensea	IGR-opense		rs5765391	1;24
cg0049646	0.232853	0.547774	9.931205	1.26E-05	0.000907	3.892416	0.431348	0.664201	-0.23285	7	93577610	F	II		IGR	opensea	IGR-opense		rs1492379	22
cg2158004	0.237652	0.420867	9.928984	1.27E-05	0.000908	3.890778	0.302041	0.539693	-0.23765	17	17929070	F	II	ATPAF2	Body	opensea	Body-opens		rs1134836	36
cg1831170	0.204209	0.399	9.928477	1.27E-05	0.000908	3.890403	0.296895	0.501104	-0.20421	12	52730954	R	II		IGR	opensea	IGR-opense			
cg1131128	0.213303	0.676174	9.92678	1.27E-05	0.000908	3.889152	0.569523	0.782826	-0.2133	12	63088234	F	II	PPM1H	Body	opensea	Body-opens		rs5726652	0
cg2055327	0.268358	0.535653	9.926421	1.27E-05	0.000908	3.888887	0.401474	0.669832	-0.26836	4	96336870	R	II	UNC5C	Body	opensea	Body-opens		rs1828039	50;31;21;20;14;1
cg0491212	0.206739	0.401816	9.926018	1.27E-05	0.000908	3.888589	0.298446	0.505186	-0.20674	6	1.54E+08	F	II	OPRM1	TSS1500	opensea	TSS1500-op		rs1163440	44
cg0122835	0.2282	0.528869	9.924796	1.27E-05	0.000909	3.887687	0.414769	0.642969	-0.2282	2	1.06E+08	R	II		IGR	opensea	IGR-opense		rs5538231	1;22;39;43;51
cg1138986	0.217248	0.520783	9.924618	1.27E-05	0.000909	3.887556	0.412159	0.629407	-0.21725	11	69240780	R	II		IGR	opensea	IGR-opense		rs5526613	1
cg0537693	0.24281	0.475135	9.922876	1.27E-05	0.000909	3.886271	0.353731	0.59654	-0.24281	1	2.25E+08	R	II	CNIH3	Body	opensea	Body-opens		rs5764090	32;33;47
cg0682353	0.24467	0.590824	9.92088	1.27E-05	0.00091	3.884798	0.468489	0.713159	-0.24467	4	38719348	F	II		IGR	opensea	IGR-opense		rs1136911	0
cg0350995	0.201045	0.610846	9.919766	1.27E-05	0.00091	3.883975	0.510323	0.711368	-0.20105	12	3544062	F	II	PRMT8	Body	opensea	Body-opens		rs1886563	0;5;25;45
cg0524249	0.203115	0.397554	9.919601	1.27E-05	0.00091	3.883853	0.295997	0.499112	-0.20311	5	1.24E+08	F	II		IGR	opensea	IGR-opense		rs5701278	1;17
cg0489002	0.218462	0.537694	9.919384	1.27E-05	0.00091	3.883693	0.428463	0.646926	-0.21846	18	74842101	F	II	MBP	5'UTR	shore	5'UTR-shor	chr18:748	rs1917729	31
cg2486488	0.201382	0.626944	9.917416	1.28E-05	0.000911	3.88224	0.526252	0.727635	-0.20138	18	7447886	F	II		IGR	opensea	IGR-opense		rs5499384	1;36;37
cg1190732	0.219968	0.611809	9.91589	1.28E-05	0.000912	3.881113	0.501825	0.721793	-0.21997	8	1.23E+08	F	II		IGR	opensea	IGR-opense		rs5548119	15;17
cg2668968	0.21203	0.464515	9.91558	1.28E-05	0.000912	3.880883	0.3585	0.57053	-0.21203	5	86418216	R	II	LOC10192	Body	opensea	Body-opens			
cg0349097	0.215424	0.597821	9.91472	1.28E-05	0.000912	3.880248	0.490109	0.705533	-0.21542	3	1.19E+08	R	II	UPK1B	5'UTR	opensea	5'UTR-oper		rs5526797	31
cg0624530	0.22027	0.426499	9.914124	1.28E-05	0.000912	3.879808	0.316364	0.536634	-0.22027	1	48227883	R	II	TRABD2B	3'UTR	opensea	3'UTR-oper		rs1849128	11
cg2145592	0.211999	0.611623	9.913974	1.28E-05	0.000912	3.879697	0.505624	0.717622	-0.212	3	57326068	R	II	ASB14	5'UTR	opensea	5'UTR-oper		rs1460061	37;5;1
cg1130024	0.251496	0.606308	9.91241	1.28E-05	0.000912	3.878542	0.48056	0.732056	-0.2515	9	1.37E+08	F	II		IGR	opensea	IGR-opense		rs1466424	1;3;5;15;42;46
cg0791577	0.203504	0.55338	9.911991	1.28E-05	0.000912	3.878232	0.451628	0.655131	-0.2035	2	1.9E+08	R	II		IGR	opensea	IGR-opense		rs1163616	44;1
cg1680604	0.230848	0.661315	9.910985	1.28E-05	0.000912	3.877489	0.545891	0.776739	-0.23085	13	39880988	R	II		IGR	opensea	IGR-opense		rs5682308	51
cg1886038	0.235689	0.438554	9.910537	1.28E-05	0.000912	3.877158	0.320709	0.556398	-0.23569	16	73147208	F	II		IGR	opensea	IGR-opense		rs5528517	0;6
cg1318612	0.237753	0.563034	9.91001	1.28E-05	0.000912	3.876768	0.444158	0.68191	-0.23775	12	40117933	F	II		IGR	opensea	IGR-opense		rs5497262	18;23
cg2642669	0.200979	0.402263	9.907893	1.28E-05	0.000913	3.875204	0.301774	0.502753	-0.20098	10	93861824	R	II	CPEB3	Body	opensea	Body-opens		rs1842588	34
cg1369789	0.251715	0.526481	9.907801	1.29E-05	0.000913	3.875136	0.400623	0.652338	-0.25171	17	73312128	F	II		IGR	opensea	IGR-opense		rs1141066	44
cg1992919	0.248559	0.55374	9.907586	1.29E-05	0.000913	3.874977	0.42946	0.67802	-0.24856	7	22390378	R	II	RAPGEF5	5'UTR	opensea	5'UTR-oper		rs1445035	28;1
cg0659015	0.232638	0.526352	9.905981	1.29E-05	0.000914	3.87379	0.410033	0.64267	-0.23264	4	1.24E+08	F	II	IL21-AS1	Body	opensea	Body-opens		rs5442322	0;15;40
cg2270687	0.200397	0.618274	9.905434	1.29E-05	0.000914	3.873386	0.518076	0.718473	-0.2004	10	74634711	R	II	MCU	Body	opensea	Body-opens		rs5424242	34;3
cg2617391	0.215869	0.684219	9.905079	1.29E-05	0.000914	3.873124	0.576285	0.792154	-0.21587	3	19387759	R	II	KCNH8	Body	opensea	Body-opens		rs1860406	24;4
cg0673878	0.242543	0.419452	9.902176	1.29E-05	0.000915	3.870976	0.298181	0.540723	-0.24254	20	19265058	R	II	LOC10013	Body	opensea	Body-opens		rs3692381	49;42
cg1800798	0.221613	0.607456	9.9019	1.29E-05	0.000915	3.870773	0.496649	0.718263	-0.22161	7	95022330	R	II	PON3	Body	shelf	Body-shelf	chr7:9502	rs1777416	16
cg0023220	0.203981	0.536015	9.900043	1.29E-05	0.000915	3.869399	0.434024	0.638005	-0.20398	9	1.14E+08	R	II	LPAR1	5'UTR	opensea	5'UTR-oper		rs3761046	45;21
cg0544974	0.202516	0.51912	9.899336	1.29E-05	0.000916	3.868876	0.417862	0.620378	-0.20252	13	1.1E+08	R	II		IGR	opensea	IGR-opense		rs1841110	2
cg2555797	0.208484	0.624125	9.898368	1.29E-05	0.000916	3.868159	0.519883	0.728367	-0.20848	13	78119389	F	II	SCEL	5'UTR	opensea	5'UTR-oper		rs5670435	47
cg0616910	0.214378	0.619394	9.897177	1.29E-05	0.000916	3.867278	0.512205	0.726584	-0.21438	1	1.57E+08	R	II		IGR	opensea	IGR-opense		rs1396870	5;2
cg1029532	0.203319	0.567294	9.89642	1.3E-05	0.000917	3.866718	0.465635	0.668954	-0.20332	11	66814384	R	II	SYT12	Body	opensea	Body-opens		rs5314605	10;4
cg1186772	0.210631	0.665829	9.896364	1.3E-05	0.000917	3.866676	0.560514	0.771145	-0.21063	22	45903851	R	II	FBLN1	Body	opensea	Body-opens		rs5515877	48;44
cg0995329	0.216244	0.554228	9.895137	1.3E-05	0.000917	3.865769	0.446107	0.66235	-0.21624	6	4347415	R	II		IGR	opensea	IGR-opense		rs5372538	2
cg0272960	0.211052	0.505707	9.895078	1.3E-05	0.000917	3.865725	0.40018	0.611233	-0.21105	7	94995740	R	II	PON3	Body	opensea	Body-opens		rs5409639	16;1
cg1178633	0.25208	0.505904	9.894135	1.3E-05	0.000918	3.865027	0.379864	0.631944	-0.25208	18	56809717	F	II	SEC11C	Body	shelf	Body-shelf	chr18:568	rs1260467	26;35
cg0318592	0.213489	0.725978	9.893504	1.3E-05	0.000918	3.864559	0.619233	0.832722	-0.21349	14	1E+08	F	II	CYP46A1	Body	opensea	Body-opens		rs1881045	0
cg1763013	0.200862	0.675452	9.891877	1.3E-05	0.000918	3.863355	0.575021	0.775883	-0.20086	3	1.55E+08	R	II		IGR	shelf	IGR-shelf	chr3:1550	rs5676239	34;21
cg0312283	0.213355	0.586301	9.890711	1.3E-05	0.000919	3.862492	0.479623	0.692978	-0.21336	18	332912	R	II	COLEC12	Body	opensea	Body-opens		rs5734366	34
cg0789420	0.228318	0.530397	9.890322	1.3E-05	0.000919	3.862204	0.416238	0.644556	-0.22832	12	78481296	F	II	NAV3	Body	opensea	Body-opens		rs1282375	0;1;23
cg1363129	0.20653	0.617933	9.890067	1.3E-05	0.000919	3.862015	0.514668	0.721198	-0.20653	5	1.78E+08	R	II		IGR	opensea	IGR-opense		rs1491261	48;8;3;2;1
cg0475802	0.249392	0.498374	9.887131	1.3E-05	0.00092	3.85984	0.373678	0.62307	-0.24939	2	1.52E+08	R	II		IGR	opensea	IGR-opense		rs5318671	49;24;2
cg1137435	0.207917	0.533583	9.882631	1.31E-05	0.000921	3.856506	0.429625	0.637542	-0.20792	19	39925660	R	II	RPS16	Body	shore	Body-shore	chr19:399	rs7303338	41
cg2453305	0.210906	0.415899	9.881221	1.31E-05	0.000922	3.855461	0.310446	0.521352	-0.21091	7	1.15E+08	R	II	LINC01392	TSS200	opensea	TSS200-ope		rs5475150	50;7;1
cg0745785	0.208812	0.46555	9.879578	1.31E-05	0.000922	3.854244	0.361144	0.569955	-0.20881	10	77028890	R	II		IGR	opensea	IGR-opense		rs5360403	27
cg1692030	0.257658	0.582246	9.878756	1.31E-05	0.000923	3.853634	0.453417	0.711076	-0.25766	4	74548513	R	II		IGR	opensea	IGR-opense		rs5495018	51;41;41;37;2;1

cg0112073	0.28872	0.455863	9.876402	1.31E-05	0.000923	3.851889	0.311504	0.600223	-0.28872	19	19080651	R	II		IGR	opensea	IGR-opense		rs1464859 41;27
cg0171264	0.21729	0.538757	9.87533	1.32E-05	0.000923	3.851093	0.430112	0.647402	-0.21729	2	1.6E+08	R	II		IGR	opensea	IGR-opense		rs6218219 23;16;2
cg208381C	0.216631	0.582697	9.875083	1.32E-05	0.000923	3.85091	0.474382	0.691013	-0.21663	9	37820310	F	II	DCAF10	Body	opensea	Body-opens		rs1818606 0;1;41;46
cg1188356	0.218216	0.425761	9.87476	1.32E-05	0.000923	3.850671	0.316653	0.534869	-0.21822	6	1.12E+08	F	II	FYN	5'UTR	opensea	5'UTR-oper		rs1463810 33;37
cg108750C	0.215798	0.657921	9.87144	1.32E-05	0.000924	3.848208	0.550022	0.76582	-0.2158	4	85799002	F	II	WDFY3	5'UTR	opensea	5'UTR-oper		
cg064305C	0.210091	0.495801	9.871138	1.32E-05	0.000924	3.847984	0.390756	0.600847	-0.21009	21	44853470	F	II		IGR	opensea	IGR-opense		
cg1637991	0.209724	0.551667	9.871021	1.32E-05	0.000924	3.847897	0.446805	0.656529	-0.20972	9	1.14E+08	F	II	MUSK	Body	opensea	Body-opens		rs5379414 20
cg1448565	0.219559	0.635033	9.869277	1.32E-05	0.000925	3.846603	0.525254	0.744813	-0.21956	3	23622488	R	II	UBE2E2	Body	opensea	Body-opens		rs5777691 43;1
cg0490235	0.201478	0.706224	9.867902	1.32E-05	0.000926	3.845582	0.605485	0.806963	-0.20148	8	30357101	R	II	RBPMS	Body	opensea	Body-opens		rs5585956 6;1
cg0292602	0.225797	0.619513	9.867849	1.32E-05	0.000926	3.845543	0.506615	0.732412	-0.2258	1	1.78E+08	F	II	RASAL2	Body	opensea	Body-opens		rs1906417 0;50
cg1149821	0.229143	0.630862	9.867384	1.32E-05	0.000926	3.845198	0.516291	0.745434	-0.22914	1	2.1E+08	R	II		IGR	opensea	IGR-opense		rs6182036 49;10;1
cg0415319	0.212955	0.451397	9.866545	1.32E-05	0.000926	3.844575	0.344919	0.557874	-0.21295	2	38264963	R	II	RMDN2-AS	TSS1500	opensea	TSS1500-op		rs5482063 45;2
cg0589202	0.221041	0.614035	9.866322	1.32E-05	0.000926	3.844409	0.503514	0.724555	-0.22104	7	553412	F	II	PDGFA	Body	shelf	Body-shelf	chr7:5566	rs1918144 0;1;21
cg0629795	0.213027	0.514656	9.865789	1.32E-05	0.000926	3.844014	0.408142	0.621169	-0.21303	15	39985286	R	II	FSIP1	Body	opensea	Body-opens		rs1163582 7;6
cg0611689	0.218987	0.506964	9.865736	1.32E-05	0.000926	3.843974	0.39747	0.616457	-0.21899	6	41372569	R	II		IGR	shore	IGR-shore	chr6:4137	rs5706286 35;4
cg187425C	0.208349	0.550526	9.865434	1.32E-05	0.000926	3.84375	0.446352	0.6547	-0.20835	9	33185416	R	II		IGR	opensea	IGR-opense		rs1915474 38;26;1
cg1768146	0.230503	0.687773	9.862977	1.33E-05	0.000927	3.841926	0.572521	0.803024	-0.2305	4	10131207	R	II		IGR	opensea	IGR-opense		rs6228660 48;44;43;40;16;8
cg1284999	0.241196	0.525838	9.86278	1.33E-05	0.000927	3.84178	0.40524	0.646435	-0.2412	15	51970513	R	II		IGR	shelf	IGR-shelf	chr15:519	rs5601462 26
cg1876435	0.205016	0.550568	9.862433	1.33E-05	0.000927	3.841522	0.44806	0.653076	-0.20502	7	85138606	R	II		IGR	opensea	IGR-opense		rs1844212 33;2
cg1615848	0.20518	0.671392	9.86177	1.33E-05	0.000927	3.84103	0.568802	0.773982	-0.20518	1	33891592	R	II		IGR	opensea	IGR-opense		
cg032495C	0.202461	0.436021	9.861491	1.33E-05	0.000927	3.840823	0.334791	0.537252	-0.20246	2	2.09E+08	F	II		IGR	opensea	IGR-opense		rs5274170 0;6;45
cg1929626	0.211082	0.535942	9.861037	1.33E-05	0.000927	3.840485	0.430401	0.641483	-0.21108	6	25636039	R	II		IGR	opensea	IGR-opense		
cg1413626	0.201172	0.563322	9.859638	1.33E-05	0.000928	3.839446	0.462736	0.663908	-0.20117	11	73711382	R	II	UCP3	3'UTR	opensea	3'UTR-oper		
cg1988188	0.2308	0.581009	9.858422	1.33E-05	0.000928	3.838542	0.465609	0.696409	-0.2308	7	12362656	F	II		IGR	opensea	IGR-opense		rs5761410 6;7;12;19;31
cg1180034	0.20067	0.653689	9.858113	1.33E-05	0.000928	3.838313	0.553354	0.754024	-0.20067	9	1.14E+08	F	II	LPAR1	Body	opensea	Body-opens		rs1869473 1;2
cg2130398	0.231534	0.437362	9.856812	1.33E-05	0.000928	3.837346	0.321595	0.553129	-0.23153	2	1.06E+08	R	II		IGR	opensea	IGR-opense		rs1016902 39;24;5;1
cg2292642	0.212242	0.566196	9.855823	1.33E-05	0.000929	3.836612	0.460075	0.672317	-0.21224	12	70721074	F	II	CNOT2	Body	opensea	Body-opens		rs5307862 18;37
cg2296235	0.258769	0.581004	9.854794	1.34E-05	0.000929	3.835847	0.45162	0.710388	-0.25877	18	42982855	R	II	SLC14A2	5'UTR	opensea	5'UTR-oper		
cg0784314	0.241125	0.509088	9.85466	1.34E-05	0.000929	3.835747	0.388525	0.62965	-0.24112	5	38925921	F	II	OSMR	Body	opensea	Body-opens		rs5732044 1
cg196400C	0.21723	0.632106	9.854545	1.34E-05	0.000929	3.835661	0.523491	0.740721	-0.21723	4	1.47E+08	R	II	C4orf51	TSS1500	opensea	TSS1500-op		
cg2577325	0.205311	0.664887	9.853223	1.34E-05	0.000929	3.834679	0.562231	0.767542	-0.20531	12	94019073	R	II		IGR	opensea	IGR-opense		rs7213823 44;21;2
cg2359465	0.211377	0.667077	9.853178	1.34E-05	0.000929	3.834646	0.561389	0.772766	-0.21138	7	65796392	R	II	TPST1	Body	opensea	Body-opens		
cg013812C	0.25418	0.493214	9.852322	1.34E-05	0.00093	3.834009	0.366124	0.620304	-0.25418	12	2985630	F	II	FOXM1	5'UTR	shore	5'UTR-shor	chr12:298	rs5745077 23;25
cg0528405	0.20821	0.630918	9.85204	1.34E-05	0.00093	3.8338	0.526813	0.735023	-0.20821	10	80665368	R	II		IGR	opensea	IGR-opense		rs5540503 24;15;2;1
cg2563256	0.222149	0.630011	9.851818	1.34E-05	0.00093	3.833634	0.518937	0.741086	-0.22215	5	36422692	R	II		IGR	opensea	IGR-opense		rs1493826 39
cg1840833	0.219224	0.507396	9.851662	1.34E-05	0.00093	3.833518	0.397783	0.617008	-0.21922	18	3305544	R	II		IGR	opensea	IGR-opense		rs5344272 42
cg0876398	0.217012	0.53213	9.849603	1.34E-05	0.000931	3.831988	0.423624	0.640636	-0.21701	10	44872504	R	II	CXCL12	Body	opensea	Body-opens		rs5344946 39;32
cg2612123	0.202108	0.453081	9.848885	1.34E-05	0.000931	3.831454	0.352027	0.554135	-0.20211	3	13899618	F	II	WNT7A	Body	shelf	Body-shelf	chr3:1389	rs1863682 1;51
cg2349849	0.224643	0.56968	9.847741	1.34E-05	0.000932	3.830603	0.457359	0.682002	-0.22464	7	90095672	F	II		IGR	opensea	IGR-opense		
cg1204492	0.209974	0.407512	9.846361	1.34E-05	0.000932	3.829577	0.302525	0.512499	-0.20997	4	5207312	F	II	STK32B	Body	opensea	Body-opens		rs3725833 15;27;49
cg0866541	0.234249	0.468698	9.845353	1.34E-05	0.000932	3.828827	0.351573	0.585822	-0.23425	19	46969849	R	II	PNMAL1	3'UTR	opensea	3'UTR-oper		rs5392116 19;4
cg0287977	0.218611	0.525332	9.844752	1.34E-05	0.000932	3.828379	0.416027	0.634638	-0.21861	10	80407110	F	II		IGR	opensea	IGR-opense		rs8023439 7;10
cg0724743	0.242033	0.529621	9.842395	1.35E-05	0.000933	3.826626	0.408604	0.650637	-0.24203	18	9809551	F	II	RAB31	Body	opensea	Body-opens		rs1163111 50
cg1527404	0.233477	0.508118	9.840226	1.35E-05	0.000934	3.825011	0.391379	0.624856	-0.23348	19	1102570	F	II	GPX4	TSS1500	shore	TSS1500-sh	chr19:110	rs5655066 3;24;41
cg1237805	0.236071	0.587838	9.839319	1.35E-05	0.000935	3.824336	0.469802	0.705873	-0.23607	1	74929525	R	II	FPGT-TNNI	Body	opensea	Body-opens		rs1709537 41;4
cg1920563	0.224093	0.651777	9.838658	1.35E-05	0.000935	3.823844	0.539731	0.763824	-0.22409	11	59282123	F	II	OR4D9	TSS1500	opensea	TSS1500-op		rs5641148 0;31
cg2544918	0.204623	0.463112	9.838654	1.35E-05	0.000935	3.823841	0.360801	0.565424	-0.20462	3	70115097	F	II		IGR	opensea	IGR-opense		rs5751266 31
cg2499067	0.239795	0.56761	9.838255	1.35E-05	0.000935	3.823544	0.447712	0.687507	-0.2398	20	8048453	R	II		IGR	opensea	IGR-opense		rs5287949 39;2;1
cg2698671	0.24602	0.542749	9.838171	1.35E-05	0.000935	3.823481	0.419739	0.665759	-0.24602	3	1.22E+08	R	II	ILDR1	Body	opensea	Body-opens		rs5571512 2
cg0759225	0.226481	0.497903	9.836872	1.35E-05	0.000936	3.822514	0.384663	0.611144	-0.22648	16	57910413	F	II		IGR	opensea	IGR-opense		rs1724095 30
cg1585213	0.230603	0.583173	9.836292	1.35E-05	0.000936	3.822082	0.467871	0.698475	-0.2306	18	12102699	F	II	ANKRD62	Body	opensea	Body-opens		
cg1806555	0.207705	0.561366	9.834636	1.35E-05	0.000936	3.82085	0.457514	0.665219	-0.20771	3	58596027	F	II	FAM107A	Body	opensea	Body-opens		rs5435428 22;28;33;40
cg1985361	0.212659	0.474985	9.834499	1.35E-05	0.000936	3.820747	0.368656	0.581314	-0.21266	5	1.22E+08	F	II	LOC10192	Body	opensea	Body-opens		rs1827566 1;39
cg0239652	0.223191	0.550434	9.83422	1.36E-05	0.000936	3.82054	0.438838	0.662029	-0.22319	2	1.96E+08	F	II		IGR	opensea	IGR-opense		rs1456292 15
cg1358593	0.240741	0.509833	9.832559	1.36E-05	0.000936	3.819302	0.389462	0.630203	-0.24074	10	72027357	R	II	NPFFR1	TSS1500	opensea	TSS1500-op		rs5657026 5;2
cg1355908	0.26055	0.543497	9.831923	1.36E-05	0.000937	3.818829	0.413222	0.673772	-0.26055	15	94441198	F	II		IGR	opensea	IGR-opense		rs5305341 0
cg2385341	0.203144	0.587887	9.830165	1.36E-05	0.000937	3.817519	0.486314	0.689459	-0.20314	10	45065077	F	II		IGR	opensea	IGR-opense		rs5563500 0;1
cg0403988	0.206979	0.466977	9.829853	1.36E-05	0.000937	3.817287	0.363487	0.570466	-0.20698	1	2.39E+08	R	II		IGR	opensea	IGR-opense		rs7866368 15;12
cg1221957	0.2217	0.617334	9.828576	1.36E-05	0.000938	3.816335	0.506484	0.728184	-0.2217	2	1.74E+08	F	II	ZAK	Body	opensea	Body-opens		rs5762998 27
cg1926877	0.215339	0.628222	9.826819	1.36E-05	0.000938	3.815026	0.520552	0.735891	-0.21534	10	52041044	R	II		IGR	opensea	IGR-opense		rs5343551 39;37
cg1289842	0.207604	0.429025	9.826727	1.36E-05	0.000938	3.814957	0.325223	0.532827	-0.2076	8	1.23E+08	F	II		IGR	opensea	IGR-opense		rs1479250 25
cg1830159	0.222402	0.592732	9.82621	1.36E-05	0.000938	3.814572	0.481531	0.703933	-0.2224	11	1.27E+08	F	II	KIRREL3	Body	opensea	Body-opens		rs5507860 0
cg1941213	0.223351	0.642697	9.824122	1.37E-05	0.000939	3.813015	0.531022	0.754373	-0.22335	6	16747686	F	II	ATXN1	5'UTR	opensea	5'UTR-oper		rs5732894 3
cg2153281	0.20928	0.435674	9.823454	1.37E-05	0.000939	3.812517	0.331034	0.540314	-0.20928	1	1.59E+08	R	II	CADM3	Body	opensea	Body-opens		rs5578988 44;1
cg2648412	0.232736	0.594656	9.821967	1.37E-05	0.00094	3.811408	0.478287	0.711024	-0.23274	18	10094391	R	II		IGR	opensea	IGR-opense		rs3728334 1
cg2494306	0.200554	0.411036	9.821324	1.37E-05	0.00094	3.810929	0.310759	0.511313	-0.20055	21	27009142	R	II		IGR	shelf	IGR-shelf	chr21:270	
cg0464507	0.250012	0.449562	9.821323	1.37E-05	0.00094	3.810928	0.324556	0.574568	-0.25001	11	34393106	F	II		IGR	opensea	IGR-opense		rs5693732 0;4;43
cg0362811	0.251237	0.618217	9.820541	1.37E-05	0.00094	3.810344	0.492599	0.743836	-0.25124	12	68553980	F	II	IFNG	TSS1500	opensea	TSS1500-op		rs5514063 2;48
cg2165957	0.210238	0.515538	9.820173	1.37E-05	0.00094	3.81007	0.410419	0.620657	-0.21024	17	47467409	F	II		IGR	opensea	IGR-opense		rs5290923 0;2
cg0454131	0.220102	0.42841	9.819878	1.37E-05	0.00094	3.80985	0.318359	0.538461	-0.2201	15	33897878	F	II	RYR3	Body	opensea	Body-opens		rs5532466 7;43
cg1100103	0.213467	0.619213	9.819721	1.37E-05	0.00094	3.809733	0.51248	0.725946	-0.21347	12	13306175	R	II		IGR	opensea	IGR-opense		rs5305450 41;2
cg1740838	0.207309	0.648088	9.819151	1.37E-05	0.000941	3.809308	0.544433	0.751743	-0.20731	17	27049137	F	II	RPL23A	Body	shore	Body-shore	chr17:270	rs5429103 4;14;35
cg1334271	0.239434	0.633999	9.818217	1.37E-05	0.000941	3.808611	0.514282	0.753716	-0.23943	3	1.23E+08	F	II	MYLK	Body	opensea	Body-opens		rs1124707 3
cg0014294	0.202858	0.664322	9.816961	1.37E-05	0.000942	3.807674	0.562893	0.765751	-0.20286	10	90646294	F	II	STAMBPL1	5'UTR	opensea	5'UTR-oper		rs1386509 0;16;25;47
cg0831577	0.200698	0.763686	9.81573	1.37E-05	0.000942	3.806755	0.663337	0.864035	-0.2007	4	1.06E+08	F	II		IGR	opensea	IGR-opense		rs5297381 0;11;12
cg2120364	0.235196	0.697337	9.814719	1.37E-05	0.000943	3.806001	0.579738	0.814935	-0.2352	6	1.57E+08	R	II		IGR	opensea	IGR-opense		rs1435589 40;24;2
cg0943517	0.232057	0.502286	9.814343	1.37E-05	0.000943	3.805721	0.386258	0.618315	-0.23206	4	1.84E+08	F	II		IGR	opensea	IGR-opense		rs5337337 0;25;33
cg0759819	0.226951	0.637576	9.813565	1.38E-05	0.000943	3.80514	0.5241	0.751051	-0.22695	4	1.2E+08	R	II	MYOZ2	TSS200	opensea	TSS200-ope		rs5365590 36;16;10;2
cg0965895	0.203316	0.335113	9.813238	1.38E-05	0.000943	3.804896	0.233455	0.436771	-0.20332	8	56904259	R	II	LYN	Body	opensea	Body-opens		rs2667977 17;14;2
cg1576159	0.223483	0.557357	9.812243	1.38E-05	0.000943	3.804153	0.445616	0.669099	-0.22348	9	86754339	R	II		IGR	shore	IGR-shore	chr9:8675	rs5299860 31;30;22
cg0594948	0.219963	0.527452	9.81205	1.38E-05	0.000943	3.804009	0.417471	0.637434	-0.21996	7	1.42E+08	R	II		IGR	opensea	IGR-opense		rs5759277 41;27;20;16
cg1510757	0.230272	0.504673	9.81159	1.38E-05	0.000943	3.803666	0.389537	0.619809	-0.23027	8	1.34E+08	F	II		IGR	opensea	IGR-opense		rs1924869 0
cg1735582	0.243861	0.447924	9.810518	1.38E-05	0.000943	3.802866	0.325993	0.569855	-0.24386	14	72516174	R	II	RGS6	5'UTR	opensea	5'UTR-oper		rs5482225 22;16;4;2
cg2196265	0.200651	0.503877	9.809461	1.38E-05	0.000943	3.802076	0.403551	0.604202	-0.20065	8	21657302	F	II		IGR	opensea	IGR-opense		
cg0790323	0.203527	0.633766	9.809293	1.38E-05	0.000943	3.801951	0.532003	0.73553	-0.20353	7	20704865	F	II	ABCB5	Body	opensea	Body-opens		rs6949607 15;32
cg1807249	0.215639	0.596634	9.809055	1.38E-05	0.000943	3.801773	0.488815	0.704454	-0.21564	12	40944308	F	II	MUC19	Body	opensea	Body-opens		
cg1097871	0.223654	0.520738	9.808313	1.38E-05	0.000943	3.801219	0.408911	0.632565	-0.22365	15	56992113	F	II	ZNF280D	Body	opensea	Body-opens		rs1427468 0;29
cg0791446	0.210378	0.579284	9.808042	1.38E-05	0.000934	3.801016	0.474095	0.684473	-0.21038	1	1.59E+08	R	II	PYHIN1	Body	opensea	Body-opens		rs1424249 0;18
cg1002665	0.224458	0.487724	9.807559	1.38E-05	0.000943	3.800656	0.375495	0.599953	-0.22446	6	1.56E+08	F	II		IGR	opensea	IGR-opense		rs5534548 18;36;43
cg060973C	0.255189	0.476053	9.802991	1.39E-05	0.000945	3.797243	0.348458	0.603647	-0.25519	21	21749792	F	II		IGR	opensea	IGR-opense		
cg0006136	0.208241	0.573198	9.801283	1.39E-05	0.000946	3.795967	0.469077	0.677318	-0.20824	1	16196968	F	II	MIR5096	TSS1500	opensea	TSS1500-op		rs5304696 0
cg2024406	0.213116	0.552471	9.800476	1.39E-05	0.000946	3.795364	0.445913	0.659029	-0.21312	6	71108549	R	II	EVADR	Body	opensea	Body-opens		rs1901974 8;2
cg0680952	0.232071	0.495579	9.798249	1.39E-05	0.000947	3.7937	0.379543	0.611614	-0.23207	14	1.03E+08	R	II		IGR	opensea	IGR-opense		rs7155198 39;26;24
cg0854286	0.206596	0.612857	9.797608	1.39E-05	0.000947	3.79322	0.509558	0.716155	-0.2066	2	85440664	F	II	TCF7L1	Body	opensea	Body-opens		rs5384209 0;33
cg0330265	0.218235	0.570301	9.795782	1.39E-05	0.000948	3.791855	0.461184	0.679418	-0.21823	6	56674105	R	II	DST	Body	opensea	Body-opens		
cg2467654	0.216819	0.611963	9.795577	1.39E-05	0.000948	3.791702	0.503554	0.720373	-0.21682	4	86323787	F	II		IGR	opensea	IGR-opense		
cg1859814	0.248706	0.491796	9.793808	1.4E-05	0.000948	3.790379	0.367443	0.616149	-0.24871	20	47896448	R	II	SNORD12B	TSS1500	shore	TSS1500-sh	chr20:478	rs7361137 1
cg020208C	0.246839	0.58809	9.793222	1.4E-05	0.000948	3.789941	0.464671	0.711509	-0.24684	9	93893852	R	II		IGR	opensea	IGR-opense		
cg0041542	0.209669	0.480153	9.792785	1.4E-05	0.000948	3.789614	0.375319	0.584987	-0.20967	2	40622490	R	II	SLC8A1	Body	opensea	Body-opens		rs5778839 49;37
cg0431586	0.24341	0.407654	9.792011	1.4E-05	0.000948	3.789035	0.285948	0.529359	-0.24341	13	47103926	F	II		IGR	opensea	IGR-opense		rs5452154 43
cg0110091	0.208542	0.551359	9.790109	1.4E-05	0.000949	3.787612	0.447088	0.65563	-0.20854	18	60848285	F	II	BCL2	Body	opensea	Body-opens		rs5416113 18
cg0588025	0.232285	0.591817	9.788112	1.4E-05	0.00095	3.786118	0.475674	0.707959	-0.23229	1	2.09E+08	F	II		IGR	opensea	IGR-opense		rs1906265 14;27
cg1466827	0.207666	0.640996	9.785903	1.4E-05	0.00095	3.784465	0.537162	0.744829	-0.20767	8	72743243	R	II		IGR	opensea	IGR-opense		rs5632145 44;6
cg0208484	0.222983	0.528818	9.7845	1.4E-05	0.00095	3.783414	0.417326	0.640309	-0.22298	16	69380204	R	II	TMED6	Body	opensea	Body-opens		rs7593405 17;2
cg1700564	0.253086	0.566982	9.784294	1.41E-05	0.00095	3.78326	0.440438	0.693525	-0.25309	7	23304848	R	II	GPNMB	Body	opensea	Body-opens		
cg029206C	0.205425	0.53775	9.782302	1.41E-05	0.000951	3.781769	0.435038	0.640463	-0.20543	15	59484170	R	II	MYO1E	Body	opensea	Body-opens		rs5446820 31
cg2642308	0.217587	0.640671	9.782123	1.41E-05	0.000951	3.781635	0.531877	0.749464	-0.21759	12	2126032	R	II		IGR	opensea	IGR-opense		
cg2425654	0.258999	0.51316	9.781311	1.41E-05	0.000951	3.781027	0.38366	0.642659	-0.259	12	1.02E+08	R	II		IGR	opensea	IGR-opense		rs1122641 49;21;2
cg0928358	0.240987	0.437112	9.779613	1.41E-05	0.000951	3.779755	0.316618	0.557605	-0.24099	16	80380376	R	II	LOC10272	Body	opensea	Body-opens		rs5286320 2;1
cg276351C	0.211506	0.659786	9.778774	1.41E-05	0.000952	3.779127	0.554033	0.765539	-0.21151	2	59468572	R	II	LOC10192	Body	opensea	Body-opens		rs5507799 31;18
cg1410435	0.20591	0.642102	9.77872	1.41E-05	0.000952	3.779086	0.539147	0.745057	-0.20591	6	15715552	R	II		IGR	opensea	IGR-opense		rs1444822 2;1
cg0506968	0.24109	0.53401	9.778611	1.41E-05	0.000952	3.779005	0.413465	0.654555	-0.24109	7	1.42E+08	R	II	MGAM	Body	opensea	Body-opens		
cg0064256	0.216415	0.716817	9.77704	1.41E-05	0.000952	3.777828	0.60861	0.825025	-0.21641	16	81877508	R	II	PLCG2	Body	opensea	Body-opens		rs5353567 47;40;37;32
cg1507524	0.231354	0.654617	9.776493	1.41E-05	0.000952	3.777418	0.53894	0.770294	-0.23135	2	10587052	R	II	SNORA80B	TSS200	shore	TSS200-sho	chr2:1058	rs5777315 35
cg1537312	0.211077	0.617464	9.776476	1.41E-05	0.000952	3.777405	0.511925	0.723002	-0.21108	10	89462893	F	II	PAPSS2	Body	opensea	Body-opens		rs5330837 1;6
cg2528102	0.203847	0.580948	9.776187	1.41E-05	0.000952	3.777189	0.479025	0.682872	-0.20385	12	88969462	F	II	KITLG	Body	shelf	Body-shelf	chr12:889	rs5775858 15;37
cg0622784	0.214144	0.636141	9.774562	1.42E-05	0.000953	3.775971	0.529069	0.743213	-0.21414	1	2.07E+08	F	II		IGR	opensea	IGR-opense		rs5401475 15;26
cg1741129	0.211457	0.589319	9.773449	1.42E-05	0.000953	3.775137	0.483591	0.695048	-0.21146	9	71624788	F	II		IGR	shelf	IGR-shelf	chr9:7162	rs5501538 46
cg1750881	0.214845	0.550318	9.772991	1.42E-05	0.000953	3.774794	0.442896	0.657741	-0.21485	8	38423639	R	II		IGR	opensea	IGR-opense		

cg1455272	0.231553	0.607601	9.770474	1.42E-05	0.000954	3.772907	0.491825	0.723378	-0.23155	3	1.51E+08	R	II	IGSF10	TSS200	opensea	TSS200-ope		rs5651631	3
cg0035801	0.206823	0.466839	9.768946	1.42E-05	0.000955	3.771762	0.363427	0.57025	-0.20682	1	2.21E+08	F	II		IGR	opensea	IGR-opense		rs3762937	31;42
cg0723719	0.209606	0.556373	9.76813	1.42E-05	0.000955	3.77115	0.45157	0.661176	-0.20961	2	1.6E+08	R	II	TANC1	5'UTR	opensea	5'UTR-oper		rs1424512	42;14;2
cg0965940	0.231114	0.503397	9.767905	1.42E-05	0.000955	3.770981	0.38784	0.618954	-0.23111	20	43744673	F	II	WFDC5	TSS1500	opensea	TSS1500-op		rs5376589	0;23;36
cg2353327	0.21951	0.550342	9.766322	1.42E-05	0.000956	3.769794	0.440587	0.660097	-0.21951	10	78635553	R	II	KCNMA1	3'UTR	opensea	3'UTR-oper		rs1139814	43;11;2;1
cg0551192	0.200304	0.566273	9.766215	1.42E-05	0.000956	3.769714	0.466121	0.666425	-0.2003	2	2.18E+08	R	II	DIRC3	Body	opensea	Body-opens		rs7449567	50;1
cg0604215	0.207136	0.565281	9.764609	1.43E-05	0.000956	3.768509	0.461713	0.668849	-0.20714	7	55661735	F	II		IGR	opensea	IGR-opense		rs5734746	0;1;6;9;18;30
cg0881596	0.237238	0.612181	9.762956	1.43E-05	0.000957	3.767269	0.493562	0.7308	-0.23724	3	1.44E+08	F	II	SLC9A9	Body	opensea	Body-opens		rs5561481	11;27;29
cg0159357	0.277898	0.468791	9.762442	1.43E-05	0.000957	3.766883	0.329843	0.60774	-0.2779	17	60818877	R	II	MARCH10	Body	opensea	Body-opens			
cg1631287	0.203368	0.71947	9.76134	1.43E-05	0.000957	3.766057	0.617786	0.821153	-0.20337	1	40343411	F	II	TRIT1	Body	opensea	Body-opens		rs1078904	21;22;30
cg1676016	0.219037	0.591676	9.759234	1.43E-05	0.000957	3.764476	0.482157	0.701194	-0.21904	15	38901688	F	II		IGR	opensea	IGR-opense		rs1878676	42
cg1568627	0.207154	0.541904	9.75908	1.43E-05	0.000957	3.764361	0.438327	0.645481	-0.20715	9	92807373	F	II		IGR	opensea	IGR-opense		rs5709701	0;27;33;49
cg2405057	0.200553	0.49702	9.759033	1.43E-05	0.000957	3.764325	0.396744	0.597297	-0.20055	13	34574802	F	II		IGR	opensea	IGR-opense			
cg1256778	0.210048	0.616598	9.758873	1.43E-05	0.000957	3.764205	0.511574	0.721622	-0.21005	4	1.14E+08	R	II	ANK2	Body	opensea	Body-opens		rs5761994	48;40;2
cg1058138	0.221156	0.525686	9.758551	1.43E-05	0.000957	3.763963	0.415108	0.636264	-0.22116	2	2.39E+08	R	II		IGR	opensea	IGR-opense			
cg2183647	0.258022	0.486935	9.757164	1.43E-05	0.000957	3.762922	0.357924	0.615946	-0.25802	13	76535057	R	II		IGR	opensea	IGR-opense		rs1417420	20
cg2761593	0.222846	0.538532	9.756903	1.43E-05	0.000958	3.762726	0.427109	0.649956	-0.22285	17	16344574	F	II	NCRNA001	Body	shore	Body-shore	chr17:1634	rs5606759	11;25;26;27
cg2185525	0.239699	0.557177	9.756633	1.43E-05	0.000958	3.762523	0.437327	0.677026	-0.2397	1	1.59E+08	F	II	IFI16	5'UTR	opensea	5'UTR-oper		rs5560662	11;24;30;37;46
cg2019818	0.204623	0.553681	9.756125	1.43E-05	0.000958	3.762143	0.451369	0.655992	-0.20462	10	49832794	F	II		IGR	opensea	IGR-opense		rs5335526	12;30
cg2041986	0.239797	0.497666	9.75433	1.44E-05	0.000959	3.760794	0.377767	0.617565	-0.2398	3	1.46E+08	F	II		IGR	opensea	IGR-opense		rs5429045	1;30
cg0018217	0.258413	0.571376	9.752189	1.44E-05	0.000959	3.759187	0.442169	0.700582	-0.25841	9	21852933	R	II	MTAP	Body	opensea	Body-opens			
cg1921828	0.203058	0.646349	9.752106	1.44E-05	0.000959	3.759124	0.54482	0.747878	-0.20306	14	30765767	F	II		IGR	opensea	IGR-opense		rs1378915	32
cg2304713	0.267139	0.471051	9.752101	1.44E-05	0.000959	3.75912	0.337482	0.60462	-0.26714	3	24089778	R	II		IGR	opensea	IGR-opense		rs1497249	34;29;18
cg0090263	0.213437	0.378471	9.751435	1.44E-05	0.000959	3.75862	0.271752	0.485189	-0.21344	12	1.23E+08	R	II	PITPNM2	Body	shelf	Body-shelf	chr12:1234	rs5561508	48;19
cg0872787	0.225219	0.521166	9.751266	1.44E-05	0.000959	3.758493	0.408557	0.633776	-0.22522	17	6427324	R	II	PITPNM3	Body	opensea	Body-opens		rs5352479	47;29
cg2039281	0.200992	0.496779	9.750657	1.44E-05	0.000959	3.758036	0.396283	0.597275	-0.20099	10	67469425	F	II	LINC01515	Body	opensea	Body-opens		rs3759903	0;28;51
cg2120280	0.204375	0.504377	9.750441	1.44E-05	0.000959	3.757873	0.40219	0.606565	-0.20438	13	52107056	R	II		IGR	opensea	IGR-opense		rs5362691	42;40
cg1103568	0.203711	0.632332	9.749267	1.44E-05	0.00096	3.756992	0.530476	0.734188	-0.20371	7	20338338	F	II	LOC10192	Body	opensea	Body-opens		rs1827705	8;21
cg0455231	0.205348	0.564198	9.748893	1.44E-05	0.00096	3.756711	0.461524	0.666872	-0.20535	18	5420951	R	II	EPB41L3	Body	opensea	Body-opens		rs1963827	45;39
cg1385408	0.221939	0.641967	9.748215	1.44E-05	0.00096	3.756201	0.530998	0.752937	-0.22194	14	78205329	R	II	SNW1	Body	opensea	Body-opens		rs5559488	1
cg1341076	0.245595	0.468996	9.746542	1.44E-05	0.00096	3.754944	0.346199	0.591794	-0.2456	9	90407888	R	II		IGR	opensea	IGR-opense		rs1819948	22
cg0513986	0.215139	0.644154	9.745028	1.45E-05	0.000961	3.753806	0.536584	0.751724	-0.21514	6	44007826	F	II		IGR	opensea	IGR-opense		rs5420632	22
cg2449933	0.208925	0.570664	9.740971	1.45E-05	0.000962	3.750756	0.466201	0.675127	-0.20893	5	1.72E+08	F	II	SH3PXD2B	Body	opensea	Body-opens			
cg0793717	0.273117	0.470297	9.739044	1.45E-05	0.000963	3.749307	0.333738	0.606855	-0.27312	1	2.19E+08	R	II		IGR	opensea	IGR-opense		rs5416872	1
cg0801919	0.238579	0.458014	9.738642	1.45E-05	0.000963	3.749004	0.338724	0.577304	-0.23858	1	12495618	F	II	VPS13D	Body	opensea	Body-opens			
cg0999405	0.212601	0.546009	9.738482	1.45E-05	0.000963	3.748884	0.439708	0.65231	-0.2126	10	14598259	F	II	FAM107B	5'UTR	opensea	5'UTR-oper			
cg0375274	0.212074	0.550249	9.737509	1.45E-05	0.000963	3.748153	0.444212	0.656286	-0.21207	15	84168459	F	II	SH3GL3	Body	opensea	Body-opens		rs1052057	12
cg1778568	0.268337	0.437383	9.736327	1.46E-05	0.000964	3.747263	0.303215	0.571552	-0.26834	18	21322159	F	II	LAMA3	Body	opensea	Body-opens		rs5669774	26
cg0055461	0.236726	0.510496	9.733819	1.46E-05	0.000964	3.745376	0.392132	0.628859	-0.23673	16	79747651	R	II		IGR	opensea	IGR-opense		rs5353848	48;32;1
cg2370375	0.209363	0.610995	9.732331	1.46E-05	0.000965	3.744256	0.506313	0.715676	-0.20936	8	6635325	F	II		IGR	opensea	IGR-opense		rs1406505	7;16;18;21;44;51
cg2750038	0.204744	0.511743	9.730303	1.46E-05	0.000965	3.74273	0.409371	0.614115	-0.20474	1	1.78E+08	R	II		IGR	opensea	IGR-opense		rs5317199	1
cg2531162	0.241516	0.599446	9.72855	1.46E-05	0.000966	3.74141	0.478688	0.720204	-0.24152	1	95375671	R	II	CNN3	Body	opensea	Body-opens			
cg1855357	0.203749	0.640208	9.728325	1.46E-05	0.000966	3.741241	0.538334	0.742083	-0.20375	16	19491814	R	II	TMC5	Body	opensea	Body-opens		rs5308603	51;4;1
cg0585460	0.245091	0.592255	9.728138	1.46E-05	0.000966	3.7411	0.46971	0.714801	-0.24509	2	40351367	R	II	SLC8A1	Body	opensea	Body-opens		rs5483373	23;15;5;4;2;1
cg1182858	0.206394	0.714391	9.727439	1.46E-05	0.000966	3.740574	0.611194	0.817588	-0.20639	1	2E+08	R	II	NR5A2	5'UTR	opensea	5'UTR-oper		rs1419838	2
cg0979330	0.257459	0.522199	9.727182	1.46E-05	0.000966	3.74038	0.393469	0.650928	-0.25746	1	2.13E+08	R	II	ATF3	5'UTR	opensea	5'UTR-oper		rs1435368	19;2
cg1391520	0.207997	0.507207	9.725398	1.47E-05	0.000967	3.739037	0.403209	0.611206	-0.208	14	96963698	R	II		IGR	opensea	IGR-opense		rs7944544	47;40
cg1789812	0.202036	0.458415	9.723755	1.47E-05	0.000968	3.737799	0.357397	0.559433	-0.20204	1	1.13E+08	R	II	KCND3	5'UTR	shelf	5'UTR-shelf	chr1:1125	rs1446361	26
cg1231117	0.252827	0.548174	9.721914	1.47E-05	0.000969	3.736412	0.421761	0.674588	-0.25283	4	1.19E+08	F	II	NDST3	Body	opensea	Body-opens		rs1384381	28
cg0502931	0.209265	0.732107	9.720419	1.47E-05	0.000969	3.735286	0.627474	0.836739	-0.20926	4	22476976	R	II	GPR125	Body	opensea	Body-opens		rs7537473	14;1
cg1058367	0.203805	0.399737	9.719348	1.47E-05	0.000969	3.734479	0.297835	0.501639	-0.2038	18	68157112	R	II		IGR	opensea	IGR-opense		rs5433954	33;28;24;2
cg2686950	0.21507	0.624355	9.719221	1.47E-05	0.000969	3.734383	0.516821	0.73189	-0.21507	12	98912949	F	II	TMPO	Body	shelf	Body-shelf	chr12:989(		
cg1000928	0.216704	0.450911	9.718327	1.47E-05	0.00097	3.733709	0.342559	0.559263	-0.2167	17	8657989	F	II	SPDYE4	Body	opensea	Body-opens		rs5551996	0;16;44;50
cg0044832	0.207342	0.592647	9.716849	1.48E-05	0.00097	3.732595	0.488977	0.696318	-0.20734	2	1.9E+08	R	II		IGR	opensea	IGR-opense			
cg1975577	0.222973	0.474329	9.7168	1.48E-05	0.00097	3.732558	0.362843	0.585816	-0.22297	6	29067386	R	II		IGR	opensea	IGR-opense		rs1813841	42;7
cg0575869	0.255574	0.431164	9.713805	1.48E-05	0.000971	3.7303	0.303377	0.55895	-0.25557	6	4790999	F	II	CDYL	Body	opensea	Body-opens		rs1876937	12
cg1155666	0.228389	0.419058	9.712029	1.48E-05	0.000971	3.72896	0.304863	0.533252	-0.22839	8	29499870	R	II		IGR	opensea	IGR-opense		rs5676593	2
cg0681018	0.215959	0.662663	9.710482	1.48E-05	0.000972	3.727794	0.554684	0.770643	-0.21596	2	1.88E+08	R	II		IGR	opensea	IGR-opense		rs1441983	1
cg0670599	0.251204	0.615038	9.709708	1.48E-05	0.000972	3.72721	0.489436	0.74064	-0.2512	8	30248467	F	II	RBPMS	Body	opensea	Body-opens			
cg2622192	0.206971	0.563201	9.709543	1.48E-05	0.000972	3.727085	0.459716	0.666687	-0.20697	1	27701067	R	II	FCN3	Body	opensea	Body-opens		rs1834299	50;6
cg1094245	0.203392	0.570043	9.708427	1.48E-05	0.000973	3.726243	0.468347	0.671739	-0.20339	3	1.73E+08	R	II	NLGN1	5'UTR	opensea	5'UTR-oper		rs7492996	31
cg0970313	0.225375	0.580623	9.707508	1.49E-05	0.000973	3.72555	0.467935	0.69331	-0.22537	4	1.74E+08	R	II		IGR	opensea	IGR-opense		rs5720625	2
cg0153647	0.214556	0.64204	9.706463	1.49E-05	0.000973	3.724762	0.534763	0.749318	-0.21456	3	24208364	R	II	LOC10192	Body	opensea	Body-opens		rs5663483	39;2;1
cg1065557	0.226484	0.674352	9.705886	1.49E-05	0.000973	3.724326	0.56111	0.787594	-0.22648	3	5063461	F	II		IGR	opensea	IGR-opense		rs1175083	27;39;40
cg2430804	0.216802	0.579702	9.704873	1.49E-05	0.000974	3.723562	0.471301	0.688102	-0.2168	10	49967889	R	II	WDFY4	Body	opensea	Body-opens		rs5725684	48;26;2;1
cg1223880	0.209212	0.594766	9.702357	1.49E-05	0.000974	3.721662	0.49016	0.699372	-0.20921	1	93300695	F	II	RPL5	Body	shelf	Body-shelf	chr1:9329	rs5477591	11
cg1615153	0.206246	0.498169	9.701415	1.49E-05	0.000975	3.720952	0.395046	0.601292	-0.20625	20	898238	F	II	ANGPT4	TSS1500	opensea	TSS1500-op		rs7718607	0;26;33;40
cg0360130	0.215477	0.550174	9.700032	1.49E-05	0.000975	3.719907	0.442436	0.657912	-0.21548	13	95134454	R	II		IGR	opensea	IGR-opense		rs5309531	7
cg2347489	0.209064	0.465054	9.699087	1.5E-05	0.000975	3.719193	0.360522	0.569586	-0.20906	12	1.22E+08	R	I	BCL7A	Body	opensea	Body-opens		rs5437491	19;11
cg1904017	0.231961	0.580045	9.697649	1.5E-05	0.000976	3.718107	0.464065	0.696026	-0.23196	17	61356602	F	II	TANC2	Body	opensea	Body-opens		rs1909842	11;48
cg1183422	0.207165	0.633158	9.697141	1.5E-05	0.000976	3.717723	0.529575	0.73674	-0.20716	1	2.26E+08	F	II	ENAH	Body	opensea	Body-opens		rs1464635	1
cg2259389	0.250784	0.572075	9.695393	1.5E-05	0.000976	3.716404	0.446683	0.697467	-0.25078	20	1781910	F	II		IGR	shore	IGR-shore	chr20:178	rs5504520	0;2;34;35;48
cg0416119	0.202826	0.444166	9.695297	1.5E-05	0.000976	3.716331	0.342753	0.545579	-0.20283	8	1.01E+08	R	II	RNF19A	5'UTR	opensea	5'UTR-oper		rs5643177	40
cg0147856	0.205263	0.627031	9.694495	1.5E-05	0.000977	3.715725	0.5244	0.729663	-0.20526	10	64630758	R	II		IGR	opensea	IGR-opense		rs5681084	13
cg0115394	0.217573	0.494345	9.694462	1.5E-05	0.000977	3.7157	0.385559	0.603132	-0.21757	20	37062621	F	II	LOC38879(	Body	shore	Body-shore	chr20:370(	rs5473665	0;24;41;51
cg2633623	0.244844	0.597704	9.69437	1.5E-05	0.000977	3.715631	0.475282	0.720126	-0.24484	2	38838729	F	II		IGR	opensea	IGR-opense		rs3741799	8
cg1287001	0.219926	0.629508	9.692901	1.5E-05	0.000977	3.71452	0.519545	0.739471	-0.21993	10	3847895	F	II		IGR	opensea	IGR-opense			
cg1288577	0.234597	0.575989	9.69264	1.5E-05	0.000977	3.714323	0.458691	0.693288	-0.2346	7	47725484	R	II		IGR	opensea	IGR-opense		rs1379622	36;33;18;1
cg0144950	0.206473	0.67484	9.692357	1.5E-05	0.000977	3.714109	0.571604	0.778077	-0.20647	11	60637361	F	II	ZP1	ExonBnd	opensea	ExonBnd-op		rs5710446	17;25;31
cg2359588	0.209613	0.621482	9.691355	1.5E-05	0.000977	3.713352	0.516676	0.726289	-0.20961	4	82430098	R	II		IGR	opensea	IGR-opense		rs5678540	43;36;35;2
cg1229911	0.221502	0.59214	9.691224	1.5E-05	0.000977	3.713253	0.481389	0.702892	-0.2215	3	72282244	R	II		IGR	opensea	IGR-opense		rs3595045	34;7;4;2
cg1200668	0.213072	0.531733	9.691222	1.5E-05	0.000977	3.713251	0.425197	0.638268	-0.21307	4	57960841	F	II	IGFBP7	Body	opensea	Body-opens		rs5707530	0
cg1837178	0.203165	0.498678	9.690936	1.5E-05	0.000978	3.713035	0.397095	0.600261	-0.20317	19	45116368	F	II	IGSF23	TSS1500	opensea	TSS1500-op		rs5601789	7
cg1249621	0.217295	0.615094	9.690563	1.5E-05	0.000978	3.712753	0.506446	0.723742	-0.2173	12	2193060	F	II	CACNA1C	Body	opensea	Body-opens		rs1137336	18;49
cg1907474	0.230818	0.430873	9.690461	1.5E-05	0.000978	3.712676	0.315464	0.546282	-0.23082	4	4639746	R	II	STX18-AS1	Body	opensea	Body-opens		rs5860264	36;16;2
cg2747935	0.249136	0.553375	9.689801	1.51E-05	0.000978	3.712177	0.428807	0.677943	-0.24914	8	19969536	F	II		IGR	opensea	IGR-opense		rs5762644	5
cg0531405	0.231473	0.521337	9.689264	1.51E-05	0.000978	3.711772	0.4056	0.637073	-0.23147	5	1.79E+08	R	II		IGR	opensea	IGR-opense		rs5615569	28;22
cg1571468	0.218239	0.639966	9.688966	1.51E-05	0.000978	3.711546	0.530847	0.749086	-0.21824	17	39217766	R	II	LOC73075	TSS1500	opensea	TSS1500-op			
cg0071394	0.204444	0.446944	9.688908	1.51E-05	0.000978	3.711502	0.344722	0.549166	-0.20444	8	39713316	R	II		IGR	opensea	IGR-opense		rs5756466	49;35
cg1283896	0.213509	0.596831	9.68779	1.51E-05	0.000978	3.710657	0.490076	0.703586	-0.21351	5	1.53E+08	F	II		IGR	opensea	IGR-opense		rs5432901	4
cg0668165	0.227724	0.56841	9.685174	1.51E-05	0.000979	3.708679	0.454548	0.682272	-0.22772	20	22777067	F	II		IGR	opensea	IGR-opense		rs5396566	1;6;18;42
cg0101112	0.223961	0.485613	9.684803	1.51E-05	0.00098	3.708398	0.373633	0.597593	-0.22396	2	2.25E+08	R	II	SERPINE2	Body	opensea	Body-opens		rs5414171	15
cg0488574	0.204988	0.579836	9.684557	1.51E-05	0.00098	3.708212	0.477342	0.68233	-0.20499	12	64855513	F	II	TBK1	Body	opensea	Body-opens			
cg2192351	0.214554	0.581834	9.683794	1.51E-05	0.00098	3.707635	0.474557	0.689111	-0.21455	19	42106562	R	II		IGR	opensea	IGR-opense		rs5713883	3;2;1
cg2372824	0.240694	0.503566	9.682714	1.51E-05	0.00098	3.706818	0.383219	0.623913	-0.24069	5	1.2E+08	F	II		IGR	opensea	IGR-opense		rs1450986	11
cg1519040	0.231495	0.538797	9.682259	1.51E-05	0.000981	3.706474	0.42305	0.654545	-0.23149	7	1.39E+08	R	II	HIPK2	Body	opensea	Body-opens		rs5484615	23
cg0304449	0.213663	0.576903	9.679733	1.52E-05	0.000982	3.704563	0.470071	0.683734	-0.21366	11	1.22E+08	R	II		IGR	opensea	IGR-opense		rs5300313	49;2;1
cg1825839	0.211668	0.586332	9.679156	1.52E-05	0.000982	3.704126	0.480498	0.692166	-0.21167	5	1.5E+08	R	II		IGR	opensea	IGR-opense		rs5441438	37;7
cg2463681	0.242736	0.534326	9.678444	1.52E-05	0.000982	3.703588	0.412958	0.655693	-0.24274	2	1.92E+08	R	II	STAT4	Body	opensea	Body-opens		rs5296159	43
cg2427643	0.208393	0.511908	9.678133	1.52E-05	0.000982	3.703352	0.407712	0.616105	-0.20839	1	1.96E+08	R	II		IGR	opensea	IGR-opense		rs5420867	35;19
cg1018287	0.202497	0.630808	9.675779	1.52E-05	0.000983	3.70157	0.529559	0.732056	-0.2025	11	1.04E+08	R	II		IGR	opensea	IGR-opense		rs5345902	2
cg1940605	0.228472	0.701615	9.674357	1.52E-05	0.000983	3.700494	0.587379	0.815851	-0.22847	6	42204675	R	II	TRERF1	Body	opensea	Body-opens		rs9462785	31;2;1
cg1113326	0.252044	0.581436	9.674141	1.52E-05	0.000983	3.70033	0.455414	0.707458	-0.25204	1	64540593	F	II	ROR1	Body	opensea	Body-opens		rs5613434	1;42
cg1750212	0.234516	0.507636	9.673767	1.52E-05	0.000983	3.700047	0.390378	0.624894	-0.23452	5	1.42E+08	F	II	FGF1	TSS200	opensea	TSS200-ope		rs5414802	11;17;37
cg1957866	0.243931	0.469712	9.673753	1.52E-05	0.000983	3.700036	0.347747	0.591678	-0.24393	10	3511600	R	II		IGR	opensea	IGR-opense			
cg1671735	0.238543	0.570251	9.673007	1.52E-05	0.000983	3.699471	0.450979	0.689522	-0.23854	8	1.18E+08	F	II		IGR	opensea	IGR-opense		rs3774966	0;4;35;40
cg0452798	0.2387	0.617094	9.672764	1.52E-05	0.000983	3.699287	0.497744	0.736444	-0.2387	5	71614752	R	II	PTCD2	TSS1500	shore	TSS1500-sh	chr5:7161(	rs5304664	14
cg1748963	0.206116	0.574948	9.671526	1.53E-05	0.000984	3.69835	0.47189	0.678006	-0.20612	7	38714215	R	II		IGR	opensea	IGR-opense		rs5289843	23
cg1314519	0.257051	0.422412	9.671017	1.53E-05	0.000984	3.697964	0.293886	0.550937	-0.25705	7	1.06E+08	F	II	NAMPT	TSS1500	shore	TSS1500-sh	chr7:1059	rs6133008	48
cg1708490	0.248394	0.496447	9.670788	1.53E-05	0.000984	3.697791	0.37225	0.620644	-0.24839	13	73160215	R	II		IGR	opensea	IGR-opense			
cg1488650	0.24317	0.525174	9.670697	1.53E-05	0.000984	3.697722	0.403589	0.646759	-0.24317	6	23447204	R	II		IGR	opensea	IGR-opense		rs5334853	27;2;1
cg0056541	0.241938	0.59947	9.668022	1.53E-05	0.000985	3.695696	0.478501	0.720439	-0.24194	21	26933554	F	II	MIR155HG	TSS1500	shore	TSS1500-sh	chr21:269		
cg2684231	0.23493	0.510506	9.66757	1.53E-05	0.000985	3.695353	0.39304	0.627971	-0.23493	2	16177920	F	II		IGR	opensea	IGR-opense		rs5662893	0;20
cg1666930	0.214603	0.525286	9.666417	1.53E-05	0.000986	3.69448	0.417984	0.632587	-0.2146	9	1.35E+08	R	II	MED27	Body	opensea	Body-opens			
cg2048666	0.208777	0.565038	9.666379	1.53E-05	0.000986	3.694451	0.46065	0.669427	-0.20878	12	1.28E+08	F	II		IGR	opensea	IGR-opense			
cg2407925	0.225142	0.652718	9.66535	1.53E-05	0.000987	3.693671	0.540147	0.765289	-0.22514	17	9274424	R	II	STX8	Body	opensea	Body-opens		rs1925376	27;26;8
cg1355181	0.202927	0.487565	9.664606	1.53E-05	0.000987	3.693107	0.386101	0.589028	-0.20293	2	20688997	R	II		IGR	opensea	IGR-opense		rs5463016	43;2
cg2172108	0.234729	0.528901	9.664058	1.53E-05	0.000987	3.692692	0.411537	0.646265	-0.23473	9	37667895	R	II	FRMPD1	5'UTR	opensea	5'UTR-oper		rs5434572	32;17
cg0316539	0.274314	0.42807	9.664019	1.53E-05	0.000987	3.692662	0.290913	0.565227	-0.27431	12	1.2E+08	R	II	CIT	Body	opensea	Body-opens		rs5832550	31;4;1

cg1207680	0.241836	0.545883	9.659182	1.54E-05	0.000988	3.688995	0.424965	0.666801	-0.24184	12	94699253	R	II	PLXNC1	Body	opensea	Body-opens		rs1494753	34
cg0997402	0.218591	0.626485	9.658339	1.54E-05	0.000989	3.688355	0.51719	0.735781	-0.21859	4	75346037	R	II		IGR	opensea	IGR-opense		rs5581345	44;34
cg0763872	0.204717	0.537796	9.657905	1.54E-05	0.000989	3.688027	0.435438	0.640155	-0.20472	3	1.34E+08	R	II		IGR	opensea	IGR-opense		rs5531784	46;2
cg1409488	0.227599	0.627692	9.657856	1.54E-05	0.000989	3.68799	0.513892	0.741491	-0.2276	3	42199807	R	II	TRAK1	Body	opensea	Body-opens		rs5699876	26
cg1721729	0.253791	0.403937	9.65733	1.54E-05	0.000989	3.687591	0.277042	0.530833	-0.25379	1	1.59E+08	F	II	AIM2	TSS1500	opensea	TSS1500-op		rs1162896	1;8;42
cg1543753	0.20094	0.602242	9.65612	1.54E-05	0.000989	3.686673	0.501772	0.702712	-0.20094	5	33236365	R	II		IGR	opensea	IGR-opense		rs1401825	16
cg1810095	0.281389	0.549288	9.655239	1.54E-05	0.00099	3.686004	0.408593	0.689982	-0.28139	2	2.14E+08	F	II		IGR	opensea	IGR-opense		rs6218803	18;24;25
cg0676195	0.226691	0.634767	9.655232	1.54E-05	0.00099	3.685999	0.521421	0.748112	-0.22669	2	47453931	R	II	LOC10192	Body	opensea	Body-opens		rs5328998	20;1
cg0950461	0.21612	0.474102	9.65424	1.55E-05	0.00099	3.685246	0.366042	0.582162	-0.21612	10	1.16E+08	R	II		IGR	opensea	IGR-opense		rs7337370	38
cg2534275	0.283445	0.537846	9.653719	1.55E-05	0.00099	3.684851	0.396123	0.679569	-0.28345	16	11473669	F	II		IGR	opensea	IGR-opense		rs5380570	0;13;15;38;39
cg1037197	0.230642	0.673632	9.652124	1.55E-05	0.000991	3.683641	0.558311	0.788954	-0.23064	12	42737595	F	I	PPHLN1	Body	opensea	Body-opens		rs1496070	1;17;40
cg1372002	0.210496	0.671774	9.651798	1.55E-05	0.000991	3.683393	0.566526	0.777022	-0.2105	18	36752967	R	II		IGR	opensea	IGR-opense		rs5390107	43
cg1270771	0.212955	0.529983	9.651528	1.55E-05	0.000991	3.683189	0.423506	0.636461	-0.21296	4	57137351	F	II	KIAA1211	5'UTR	opensea	5'UTR-oper		rs5459434	1
cg2691974	0.225655	0.645056	9.650973	1.55E-05	0.000991	3.682767	0.532228	0.757883	-0.22565	4	1.01E+08	F	II	LOC25688	Body	opensea	Body-opens		rs1809371	0;26
cg1838630	0.204559	0.48182	9.648887	1.55E-05	0.000992	3.681184	0.379541	0.5841	-0.20456	21	44741771	R	II		IGR	opensea	IGR-opense		rs8022542	39
cg1068408	0.203809	0.588052	9.648447	1.55E-05	0.000992	3.68085	0.486148	0.689957	-0.20381	9	92804632	F	II	LOC28637	TSS1500	opensea	TSS1500-op		rs5650213	15
cg0669018	0.240449	0.677271	9.6473	1.55E-05	0.000993	3.679979	0.557047	0.797495	-0.24045	18	77960781	F	II	PARD6G	Body	opensea	Body-opens		rs1130568	27
cg1685386	0.247824	0.340977	9.645771	1.55E-05	0.000993	3.678818	0.217066	0.464889	-0.24782	6	32823116	R	II	PSMB9	Body	shore	Body-shore	chr6:3282		
cg1360732	0.224802	0.563851	9.645614	1.56E-05	0.000993	3.678699	0.45145	0.676252	-0.2248	5	1.25E+08	F	II		IGR	opensea	IGR-opense		rs5328300	41
cg0665510	0.226518	0.656913	9.644892	1.56E-05	0.000993	3.67815	0.543654	0.770172	-0.22652	7	56165514	F	II		IGR	opensea	IGR-opense			
cg0387500	0.211828	0.523704	9.644164	1.56E-05	0.000994	3.677598	0.417789	0.629618	-0.21183	5	1.03E+08	F	II		IGR	opensea	IGR-opense			
cg0624809	0.261617	0.488664	9.644134	1.56E-05	0.000994	3.677575	0.357856	0.619473	-0.26162	18	6004245	F	II	L3MBTL4	Body	opensea	Body-opens		rs5430257	0
cg1326755	0.20099	0.531264	9.643911	1.56E-05	0.000994	3.677405	0.430769	0.631759	-0.20099	6	30915371	R	II	DPCR1	Body	opensea	Body-opens		rs1407903	1
cg0850799	0.219306	0.568677	9.643022	1.56E-05	0.000994	3.676731	0.459024	0.67833	-0.21931	20	52646082	F	II	BCAS1	Body	opensea	Body-opens		rs1504209	27;43
cg1731674	0.234792	0.59133	9.641547	1.56E-05	0.000994	3.67561	0.473934	0.708726	-0.23479	2	2.18E+08	F	II	DIRC3	Body	opensea	Body-opens		rs1391444	1;2;32;36;48
cg0491117	0.20481	0.597671	9.641336	1.56E-05	0.000994	3.67545	0.495266	0.700076	-0.20481	3	1.94E+08	R	II		IGR	opensea	IGR-opense		rs1309035	29
cg1499231	0.208008	0.356507	9.640912	1.56E-05	0.000994	3.675128	0.252503	0.460511	-0.20801	7	1.55E+08	R	II		IGR	opensea	IGR-opense			
cg1840210	0.216321	0.673259	9.640467	1.56E-05	0.000995	3.674789	0.565098	0.781419	-0.21632	3	72059061	F	II		IGR	opensea	IGR-opense		rs5510570	0;16;37
cg1798205	0.20939	0.644213	9.637677	1.56E-05	0.000995	3.67267	0.539518	0.748908	-0.20939	8	1.16E+08	F	II	TRPS1	Body	opensea	Body-opens		rs5273751	21;25
cg1839301	0.223534	0.553388	9.637135	1.56E-05	0.000995	3.672257	0.441621	0.665156	-0.22353	8	23651954	R	II		IGR	opensea	IGR-opense		rs1828605	24;1
cg1191361	0.238082	0.426576	9.636618	1.57E-05	0.000995	3.671864	0.307535	0.545617	-0.23808	2	60761127	R	II	BCL11A	Body	opensea	Body-opens		rs5374692	24;1
cg1710442	0.240425	0.519807	9.636478	1.57E-05	0.000995	3.671758	0.399594	0.640019	-0.24042	20	572111	F	II		IGR	opensea	IGR-opense		rs5529930	0;7;48
cg0892413	0.200302	0.590036	9.63635	1.57E-05	0.000995	3.671661	0.489885	0.690188	-0.2003	22	43438584	R	II	LOC10050	Body	opensea	Body-opens		rs1161208	1
cg2364646	0.225778	0.646279	9.635394	1.57E-05	0.000996	3.670934	0.53339	0.759168	-0.22578	13	1.15E+08	R	II	UPF3A	Body	opensea	Body-opens		rs5297055	29;15
cg0221560	0.21806	0.629093	9.633164	1.57E-05	0.000997	3.669238	0.520064	0.738123	-0.21806	4	1.46E+08	R	II	HHIP	Body	opensea	Body-opens			
cg2638662	0.209417	0.69819	9.632937	1.57E-05	0.000997	3.669066	0.593482	0.802899	-0.20942	8	17974394	F	II		IGR	opensea	IGR-opense		rs5679226	0;1
cg0341514	0.202585	0.57775	9.632871	1.57E-05	0.000997	3.669016	0.476458	0.679043	-0.20258	11	12037969	R	II		IGR	opensea	IGR-opense		rs5293012	44;23;22;2;1
cg1458535	0.210571	0.501447	9.632644	1.57E-05	0.000997	3.668843	0.396161	0.606732	-0.21057	17	38023679	F	II	ZPBP2	TSS1500	shore	TSS1500-sh	chr17:380		
cg1220737	0.205555	0.709332	9.630843	1.57E-05	0.000997	3.667474	0.606554	0.81211	-0.20556	12	7655803	R	II	CD163	Body	opensea	Body-opens		rs1180787	46
cg2540513	0.233587	0.545648	9.630206	1.57E-05	0.000998	3.666989	0.428854	0.662441	-0.23359	10	34488427	R	II	PARD3	Body	opensea	Body-opens			
cg0297436	0.207798	0.515905	9.629154	1.57E-05	0.000998	3.666189	0.412006	0.619804	-0.2078	1	96840725	F	II	LOC10192	TSS1500	opensea	TSS1500-op		rs3756807	0;12
cg2131713	0.20314	0.601604	9.628331	1.57E-05	0.000998	3.665563	0.500033	0.703174	-0.20314	18	74781907	F	II	MBP	Body	opensea	Body-opens		rs5680482	3;36
cg0535597	0.234911	0.628686	9.628166	1.58E-05	0.000998	3.665438	0.511231	0.746142	-0.23491	20	18792370	R	II	C20orf78	Body	opensea	Body-opens		rs5478406	2
cg0501795	0.272398	0.57228	9.627675	1.58E-05	0.000998	3.665064	0.436081	0.708479	-0.2724	3	1.51E+08	F	II	GPR87	5'UTR	opensea	5'UTR-oper		rs1192614	31;36
cg1858463	0.20389	0.513118	9.627433	1.58E-05	0.000998	3.66488	0.411173	0.615063	-0.20389	5	1.03E+08	F	II		IGR	opensea	IGR-opense		rs5615105	1;17;42
cg1074835	0.205135	0.61205	9.626025	1.58E-05	0.000999	3.663809	0.509483	0.714618	-0.20513	9	84204292	F	II	TLE1	Body	opensea	Body-opens		rs5514030	9;19;40;46
cg0238763	0.22787	0.362318	9.625871	1.58E-05	0.000999	3.663691	0.248383	0.476253	-0.22787	8	50084756	R	II		IGR	opensea	IGR-opense		rs5564741	13;1
cg1671041	0.203788	0.438071	9.625245	1.58E-05	0.001	3.663215	0.336177	0.539965	-0.20379	7	93707553	R	II		IGR	opensea	IGR-opense		rs5709207	24
cg0913166	0.235053	0.530379	9.624893	1.58E-05	0.001	3.662947	0.412852	0.647905	-0.23505	3	54076817	R	II		IGR	opensea	IGR-opense		rs1500469	2
cg1227489	0.200966	0.546754	9.624844	1.58E-05	0.001	3.66291	0.446271	0.647237	-0.20097	1	1.54E+08	F	II	S100A16	TSS1500	opensea	TSS1500-op		rs5303739	36;42
cg2200351	0.209631	0.399284	9.623292	1.58E-05	0.001	3.661729	0.294469	0.5041	-0.20963	7	90530573	F	II	CDK14	Body	opensea	Body-opens		rs1457372	0;1
cg2035619	0.208742	0.529607	9.62262	1.58E-05	0.001	3.661217	0.425236	0.633978	-0.20874	6	1.01E+08	R	II		IGR	opensea	IGR-opense		rs5707073	40
cg1185391	0.201227	0.539522	9.620265	1.58E-05	0.001002	3.659425	0.438909	0.640136	-0.20123	6	17159487	R	II		IGR	opensea	IGR-opense		rs1429702	2
cg0815499	0.202014	0.690487	9.617226	1.59E-05	0.001002	3.65711	0.58948	0.791494	-0.20201	1	53540497	F	II	PODN	Body	shelf	Body-shelf	chr1:5354		
cg0177578	0.223792	0.478857	9.616924	1.59E-05	0.001002	3.65688	0.366962	0.590753	-0.22379	11	16419252	F	II	SOX6	Body	opensea	Body-opens			
cg0406119	0.224671	0.403213	9.615607	1.59E-05	0.001003	3.655877	0.290877	0.515549	-0.22467	7	25591247	F	II		IGR	opensea	IGR-opense		rs5316005	0;1;41
cg1684714	0.24656	0.539062	9.615525	1.59E-05	0.001003	3.655815	0.415783	0.662342	-0.24656	8	98988226	R	II	MATN2	Body	opensea	Body-opens		rs5497152	10
cg1612790	0.215806	0.590356	9.615159	1.59E-05	0.001003	3.655536	0.482453	0.698259	-0.21581	6	1.17E+08	F	II	GPRC6A	TSS1500	opensea	TSS1500-op		rs5443362	24;32
cg0518977	0.206014	0.613656	9.612541	1.59E-05	0.001004	3.653541	0.510649	0.716664	-0.20601	21	38646501	F	II		IGR	opensea	IGR-opense		rs9977897	41
cg1352402	0.202551	0.440801	9.612134	1.59E-05	0.001004	3.653231	0.339526	0.542077	-0.20255	1	2.34E+08	F	II	KCNK1	Body	opensea	Body-opens		rs5431954	0;1;27;33
cg2313001	0.208713	0.635796	9.611889	1.59E-05	0.001004	3.653044	0.531439	0.740153	-0.20871	2	1.66E+08	R	II	COBLL1	Body	opensea	Body-opens		rs1116346	43
cg0735576	0.234017	0.536717	9.61173	1.59E-05	0.001004	3.652923	0.419709	0.653726	-0.23402	5	1.28E+08	F	II	FBN2	Body	shelf	Body-shelf	chr5:1278	rs5373949	0;1;36
cg0979904	0.204666	0.454902	9.610764	1.6E-05	0.001004	3.652187	0.352569	0.557235	-0.20467	7	47486641	F	II	TNS3	5'UTR	opensea	5'UTR-oper		rs1477464	22;23;26;26;35;36
cg2030434	0.246629	0.599376	9.60919	1.6E-05	0.001005	3.650987	0.476061	0.72269	-0.24663	10	13739925	R	II	FRMD4A	Body	opensea	Body-opens		rs1396537	49;16
cg1735724	0.203025	0.580673	9.607343	1.6E-05	0.001006	3.649579	0.479161	0.682186	-0.20303	11	30462469	F	II	MPPED2	Body	opensea	Body-opens		rs5618390	0;21
cg1269626	0.242408	0.511622	9.605276	1.6E-05	0.001006	3.648004	0.390418	0.632825	-0.24241	14	67446489	R	II	GPHN	Body	opensea	Body-opens		rs5423226	32;1
cg2374342	0.256965	0.706424	9.604463	1.6E-05	0.001007	3.647383	0.577942	0.834907	-0.25696	8	13170741	R	II	DLC1	Body	opensea	Body-opens		rs5633304	26
cg0459252	0.235468	0.593054	9.601857	1.61E-05	0.001008	3.645396	0.475319	0.710788	-0.23547	1	1.58E+08	R	II	CD1C	TSS1500	opensea	TSS1500-op		rs5630113	50
cg2338199	0.283833	0.507977	9.598785	1.61E-05	0.001009	3.643052	0.366061	0.649894	-0.28383	1	88867081	R	II		IGR	opensea	IGR-opense		rs5368460	22
cg0234589	0.203981	0.5794	9.598663	1.61E-05	0.001009	3.642959	0.477409	0.681391	-0.20398	20	31608207	F	II	BPIFB2	ExonBnd	opensea	ExonBnd-op		rs6057703	43
cg1384446	0.201818	0.353498	9.597762	1.61E-05	0.001009	3.642271	0.252589	0.454407	-0.20182	11	35436672	R	II	SLC1A2	Body	shelf	Body-shelf	chr11:354	rs5674451	19
cg1046725	0.200493	0.613903	9.597401	1.61E-05	0.001009	3.641996	0.513656	0.71415	-0.20049	20	24483119	F	II	SYNDIG1	5'UTR	opensea	5'UTR-oper		rs1386376	1
cg2085728	0.218018	0.491619	9.597396	1.61E-05	0.001009	3.641992	0.38261	0.600628	-0.21802	15	51632905	R	II	GLDN	TSS1500	shore	TSS1500-sh	chr15:516	rs1118695	46;27
cg2377719	0.210234	0.586374	9.595534	1.61E-05	0.00101	3.64057	0.481257	0.691491	-0.21023	5	88361885	R	II		IGR	opensea	IGR-opense		rs1416695	14;8
cg0396131	0.233548	0.607138	9.594327	1.61E-05	0.00101	3.639649	0.490364	0.723912	-0.23355	2	2.4E+08	F	II	HDAC4	Body	opensea	Body-opens		rs5274099	1;39;50
cg2306568	0.210517	0.627515	9.593718	1.62E-05	0.001011	3.639184	0.522257	0.732773	-0.21052	5	97643638	R	II		IGR	opensea	IGR-opense		rs5276784	29;13
cg2522099	0.20876	0.58812	9.59348	1.62E-05	0.001011	3.639003	0.48374	0.6925	-0.20876	5	4206669	R	II		IGR	opensea	IGR-opense		rs5328821	42;2
cg1350662	0.225028	0.63585	9.590853	1.62E-05	0.001012	3.636996	0.523336	0.748364	-0.22503	3	45131153	R	II	CDCP1	Body	opensea	Body-opens		rs1493718	2
cg0525422	0.244191	0.4917	9.588546	1.62E-05	0.001012	3.635235	0.369604	0.613795	-0.24419	10	61050062	R	II	FAM13C	Body	opensea	Body-opens		rs5729288	25;5
cg1262951	0.227468	0.649104	9.586292	1.62E-05	0.001013	3.633512	0.53537	0.762838	-0.22747	6	27859811	R	II	HIST1H3J	TSS1500	shore	TSS1500-sh	chr6:2786	rs1860837	39
cg1718967	0.202008	0.42276	9.586204	1.62E-05	0.001013	3.633445	0.321756	0.523764	-0.20201	8	61846477	F	II		IGR	opensea	IGR-opense		rs5479358	6;23;50
cg0543255	0.246145	0.498094	9.58569	1.63E-05	0.001013	3.633052	0.375022	0.621167	-0.24614	17	62632136	R	II	SMURF2	Body	opensea	Body-opens		rs5554456	29;7;2
cg1662966	0.231229	0.579768	9.585558	1.63E-05	0.001013	3.632952	0.464154	0.695383	-0.23123	12	31584191	R	II	DENND5B	Body	opensea	Body-opens		rs1430659	2
cg1278988	0.218791	0.595954	9.585089	1.63E-05	0.001013	3.632594	0.486558	0.70535	-0.21879	3	1.76E+08	F	II		IGR	opensea	IGR-opense		rs5366562	0
cg0577435	0.21212	0.651511	9.584647	1.63E-05	0.001013	3.632256	0.545451	0.757571	-0.21212	4	1.2E+08	F	II		IGR	opensea	IGR-opense			
cg1536003	0.220556	0.641118	9.584165	1.63E-05	0.001013	3.631887	0.530839	0.751396	-0.22056	3	41002920	R	II		IGR	opensea	IGR-opense		rs1389610	46;35;2
cg1127349	0.238282	0.540403	9.583914	1.63E-05	0.001013	3.631695	0.421262	0.659544	-0.23828	1	52239157	F	II	OSBPL9	Body	opensea	Body-opens		rs1482992	19;36;39;49
cg0083112	0.214695	0.575415	9.583119	1.63E-05	0.001013	3.631088	0.468068	0.682762	-0.21469	1	23038925	R	II	EPHB2	Body	shore	Body-shore	chr1:2303		
cg2217613	0.244998	0.553371	9.581583	1.63E-05	0.001014	3.629914	0.430872	0.67587	-0.245	10	33442301	F	II		IGR	opensea	IGR-opense		rs1864286	23;39
cg2423421	0.211357	0.425537	9.581413	1.63E-05	0.001014	3.629784	0.319859	0.531216	-0.21136	6	45678425	F	II		IGR	opensea	IGR-opense		rs5603071	21;26;28
cg2454516	0.214765	0.358767	9.581357	1.63E-05	0.001014	3.629741	0.251384	0.46615	-0.21477	6	1.55E+08	R	II	IPCEF1	5'UTR	opensea	5'UTR-oper		rs1892452	50;21;2;1
cg1333963	0.218657	0.500962	9.579466	1.63E-05	0.001015	3.628295	0.391634	0.61029	-0.21866	5	1.79E+08	R	II	RNF130	Body	opensea	Body-opens		rs5660575	48;18
cg0264033	0.202827	0.562841	9.579084	1.63E-05	0.001015	3.628003	0.461427	0.664254	-0.20283	2	2.06E+08	R	II	PARD3B	Body	opensea	Body-opens		rs5567470	21
cg1142136	0.216921	0.512839	9.578548	1.63E-05	0.001015	3.627593	0.404379	0.6213	-0.21692	6	1.46E+08	R	II	LOC10050	Body	opensea	Body-opens		rs1818323	21
cg0205215	0.259187	0.472762	9.57718	1.64E-05	0.001015	3.626547	0.343168	0.602355	-0.25919	8	26211126	F	II	PPP2R2A	Body	opensea	Body-opens			
cg2462348	0.20485	0.515179	9.576417	1.64E-05	0.001015	3.625964	0.412754	0.617604	-0.20485	18	6719757	R	II		IGR	opensea	IGR-opense		rs5322106	36
cg0615932	0.203013	0.567079	9.57474	1.64E-05	0.001016	3.624681	0.465573	0.668586	-0.20301	4	74516954	R	II		IGR	opensea	IGR-opense		rs1865624	2
cg0092963	0.226655	0.530784	9.574727	1.64E-05	0.001016	3.624671	0.417456	0.644111	-0.22666	1	1379667	R	II		IGR	opensea	IGR-opense		rs1386918	37
cg2763896	0.20627	0.460753	9.57433	1.64E-05	0.001016	3.624367	0.357618	0.563888	-0.20627	1	2.19E+08	F	II		IGR	opensea	IGR-opense		rs5708386	27;49
cg1583814	0.277821	0.444971	9.573712	1.64E-05	0.001016	3.623895	0.306061	0.583882	-0.27782	10	28700847	R	II		IGR	opensea	IGR-opense			
cg0620457	0.214177	0.641651	9.573604	1.64E-05	0.001016	3.623812	0.534562	0.748739	-0.21418	6	44645167	F	II		IGR	opensea	IGR-opense		rs1494794	0;1;29;33
cg0229832	0.217707	0.671067	9.57229	1.64E-05	0.001017	3.622806	0.562213	0.779921	-0.21771	2	69180527	R	II	GKN2	TSS1500	opensea	TSS1500-op		rs7393545	43;18;9;2
cg0631187	0.232939	0.666299	9.572085	1.64E-05	0.001017	3.62265	0.549829	0.782769	-0.23294	12	93427724	F	II		IGR	opensea	IGR-opense		rs1502121	12
cg1719706	0.21299	0.601573	9.571249	1.64E-05	0.001018	3.62201	0.495078	0.708068	-0.21299	4	80327523	R	II	GK2	1stExon	opensea	1stExon-op		rs5664194	41;39;35
cg0326751	0.216064	0.729951	9.57086	1.64E-05	0.001018	3.621713	0.621919	0.837983	-0.21606	8	21796065	R	II	XPO7	Body	opensea	Body-opens			
cg1498563	0.231106	0.562676	9.568702	1.65E-05	0.001018	3.62006	0.447123	0.678229	-0.23111	4	1.16E+08	F	II	NDST4	TSS1500	opensea	TSS1500-op		rs1868936	0;36
cg0950395	0.223272	0.529583	9.56569	1.65E-05	0.00102	3.617755	0.417947	0.641219	-0.22327	10	50565220	F	II		IGR	opensea	IGR-opense		rs7974075	1
cg0280755	0.259194	0.538735	9.562478	1.65E-05	0.001021	3.615295	0.409138	0.668332	-0.25919	2	1.55E+08	R	II	LOC10014	TSS1500	opensea	TSS1500-op			
cg0289036	0.210723	0.637425	9.562234	1.65E-05	0.001021	3.615108	0.532063	0.742786	-0.21072	1	1.52E+08	R	II	TUFT1	Body	opensea	Body-opens		rs5521988	1
cg1518025	0.202656	0.548366	9.562135	1.65E-05	0.001021	3.615032	0.447038	0.649694	-0.20266	10	24535313	F	II	KIAA1217	Body	opensea	Body-opens			
cg1753908	0.250688	0.486975	9.561775	1.65E-05	0.001021	3.614757	0.361631	0.612319	-0.25069	1	42265746	F	II	HIVEP3	5'UTR	opensea	5'UTR-oper		rs1450071	32;42
cg1327042	0.229373	0.544367	9.561413	1.65E-05	0.001021	3.614479	0.429681	0.659054	-0.22937	4	1.22E+08	F	II	PRDM5	Body	opensea	Body-opens			
cg0794972	0.277965	0.413362	9.56083	1.66E-05	0.001021	3.614033	0.274379	0.552344	-0.27797	17	32576670	F	II		IGR	opensea	IGR-opense		rs5612776	41
cg1354721	0.256551	0.477276	9.55777	1.66E-05	0.001023	3.611688	0.349001	0.605551	-0.25655	2	2.24E+08	R	II		IGR	opensea	IGR-opense		rs5561144	25
cg1054649	0.216252	0.525608	9.557576	1.66E-05	0.001023	3.611539	0.417482	0.633734	-0.21625	5	1.33E+08	R	II	FSTL4	ExonBnd	opensea	ExonBnd-op			
cg1462230	0.216259	0.567131	9.557192	1.66E-05	0.001023	3.611245	0.459001	0.67526	-0.21626	21	40149168	F	II		IGR	opensea	IGR-opense			
cg2111041	0.218227	0.587696	9.556791	1.66E-05	0.001023	3.610938	0.478582	0.696809	-0.21823	6	1.07E+08	F	II	AIM1	Body	opensea	Body-opens		rs5384906	1;32;38;51
cg1467143	0.220339	0.484138	9.556451	1.66E-05	0.001023	3.610677	0.373968	0.594307	-0.22034	8	9216252	F	II		IGR	opensea	IGR-opense		rs5278302	36;40
cg1650362	0.207181	0.648056	9.556314	1.66E-05	0.001023	3.610573	0.544465	0.751647	-0.20718	3	1.92E+08	R	II	FGF12	Body	opensea	Body-opens		rs5624892	33

cg1414372	0.227468	0.682556	9.555375	1.66E-05	0.001023	3.609853	0.568822	0.79629	-0.22747	3	27638612	F	II		IGR	opensea	IGR-opense			
cg1401807	0.229442	0.471908	9.554648	1.66E-05	0.001023	3.609295	0.357187	0.586629	-0.22944	1	8157705	R	II		IGR	opensea	IGR-opense		rs5579865	46;12
cg0807655	0.237737	0.592765	9.554624	1.66E-05	0.001023	3.609277	0.473897	0.711634	-0.23774	3	1.75E+08	F	II	NAALADL2-	Body	opensea	Body-opens		rs5411959	0
cg2754160	0.205082	0.456257	9.554563	1.66E-05	0.001023	3.60923	0.353716	0.558798	-0.20508	1	1.59E+08	R	II	AIM2	5'UTR	opensea	5'UTR-oper		rs2298802	48;38;32;23;10;1
cg2289271	0.233604	0.65064	9.553903	1.66E-05	0.001023	3.608724	0.533839	0.767442	-0.2336	6	22252037	R	II		IGR	opensea	IGR-opense		rs5285784	43;40;35;2
cg1141175	0.203369	0.58825	9.5534	1.66E-05	0.001024	3.608338	0.486565	0.689934	-0.20337	6	52822464	F	II		IGR	opensea	IGR-opense		rs1455683	0;48
cg0210889	0.20605	0.711407	9.553195	1.66E-05	0.001024	3.608181	0.608382	0.814432	-0.20605	10	1.29E+08	F	II	DOCK1	Body	opensea	Body-opens		rs5736690	19;40
cg0967344	0.20031	0.4831	9.55268	1.67E-05	0.001024	3.607787	0.382944	0.583255	-0.20031	2	34578659	R	II		IGR	opensea	IGR-opense		rs1833988	44
cg0650981	0.21885	0.582569	9.551828	1.67E-05	0.001024	3.607133	0.473144	0.691994	-0.21885	8	1.42E+08	R	II	PTK2	Body	opensea	Body-opens		rs5586205	49
cg0040294	0.200994	0.493774	9.55089	1.67E-05	0.001024	3.606414	0.393277	0.594271	-0.20099	10	36783791	R	II		IGR	opensea	IGR-opense		rs5374088	23
cg2241067	0.211708	0.645359	9.548419	1.67E-05	0.001025	3.604519	0.539505	0.751213	-0.21171	14	25476564	F	II	STXBP6	5'UTR	opensea	5'UTR-oper		rs1414717	5
cg2033301	0.205391	0.532995	9.548209	1.67E-05	0.001025	3.604357	0.4303	0.63569	-0.20539	11	67442126	R	II	ALDH3B2	5'UTR	opensea	5'UTR-oper		rs1166382	1
cg1884543	0.21164	0.464961	9.547892	1.67E-05	0.001025	3.604114	0.359141	0.570781	-0.21164	12	53193325	R	II		IGR	opensea	IGR-opense		rs5377218	38;1
cg0351413	0.218163	0.473743	9.547458	1.67E-05	0.001026	3.603782	0.364662	0.582825	-0.21816	7	50732341	F	II	GRB10	Body	opensea	Body-opens		rs5326268	4
cg0687856	0.225919	0.68079	9.547084	1.67E-05	0.001026	3.603495	0.567831	0.793749	-0.22592	14	51649150	F	II		IGR	opensea	IGR-opense		rs5477329	4
cg1297939	0.217265	0.606223	9.546846	1.67E-05	0.001026	3.603312	0.49759	0.714855	-0.21727	17	76569921	F	II	DNAH17	Body	opensea	Body-opens		rs1178810	1;20;23
cg0576650	0.229557	0.640334	9.546518	1.67E-05	0.001026	3.603061	0.525555	0.755113	-0.22956	2	2.08E+08	F	II	DYTN	Body	opensea	Body-opens		rs5351007	1;39
cg1557884	0.22169	0.338619	9.546421	1.67E-05	0.001026	3.602986	0.227774	0.449464	-0.22169	1	79839273	F	II		IGR	opensea	IGR-opense		rs5456866	1;46
cg1782008	0.220209	0.472026	9.545362	1.67E-05	0.001026	3.602173	0.361921	0.58213	-0.22021	2	1.63E+08	R	II	FAP	TSS1500	opensea	TSS1500-op		rs5695446	35
cg0141047	0.209625	0.391521	9.54505	1.68E-05	0.001026	3.601934	0.286709	0.496334	-0.20963	14	27286128	F	II	LOC102724	ExonBnd	opensea	ExonBnd-op		rs5439380	29
cg1083556	0.205029	0.61805	9.542166	1.68E-05	0.001027	3.599721	0.515536	0.720565	-0.20503	20	13035458	F	II	SPTLC3	Body	opensea	Body-opens		rs1927100	0;1;35;51
cg0888432	0.226462	0.641244	9.541764	1.68E-05	0.001028	3.599412	0.528013	0.754475	-0.22646	18	9354514	F	II	TWSG1	Body	opensea	Body-opens		rs5314704	5
cg0587810	0.205731	0.66336	9.54158	1.68E-05	0.001028	3.599271	0.560494	0.766225	-0.20573	3	1.7E+08	R	II	PRKCI	Body	opensea	Body-opens		rs5508848	29;14
cg1815668	0.246375	0.389494	9.541523	1.68E-05	0.001028	3.599227	0.266306	0.512681	-0.24638	6	53097064	R	II		IGR	opensea	IGR-opense		rs1490449	16;4;1
cg2291678	0.209105	0.506998	9.540553	1.68E-05	0.001028	3.598482	0.402446	0.611551	-0.20911	2	47946343	R	II		IGR	opensea	IGR-opense		rs1867275	2
cg0138156	0.212648	0.483196	9.535915	1.69E-05	0.00103	3.594921	0.376872	0.58952	-0.21265	2	2.26E+08	F	II	NYAP2	Body	opensea	Body-opens		rs5701942	0
cg2229115	0.220282	0.552505	9.533149	1.69E-05	0.001031	3.592796	0.442365	0.662646	-0.22028	13	27232582	F	II	WASF3	Body	opensea	Body-opens		rs3734997	1;16;31
cg0700971	0.219506	0.48228	9.532921	1.69E-05	0.001031	3.592621	0.372527	0.592033	-0.21951	1	2.04E+08	R	II	ATP2B4	Body	opensea	Body-opens		rs6182565	21;1
cg1447547	0.221775	0.545029	9.532505	1.69E-05	0.001031	3.592302	0.434142	0.655917	-0.22177	10	1.05E+08	R	II	NEURL	Body	opensea	Body-opens		rs5615084	1
cg1281210	0.231654	0.441713	9.530919	1.69E-05	0.001032	3.591083	0.325885	0.55754	-0.23165	9	27715148	R	II		IGR	opensea	IGR-opense			
cg0200593	0.259144	0.631461	9.530002	1.69E-05	0.001032	3.590378	0.501889	0.761033	-0.25914	13	74137411	F	II	LINC00392	TSS1500	opensea	TSS1500-op		rs5739170	18
cg2454594	0.210776	0.628023	9.528722	1.7E-05	0.001032	3.589394	0.522635	0.733411	-0.21078	2	1.83E+08	F	II	PPP1R1C	TSS1500	opensea	TSS1500-op			
cg1800332	0.246154	0.524181	9.528702	1.7E-05	0.001032	3.589379	0.401104	0.647258	-0.24615	12	32138012	F	II	KIAA1551	Body	opensea	Body-opens		rs7694315	15;31;46
cg1439379	0.227584	0.621621	9.527922	1.7E-05	0.001032	3.588779	0.507829	0.735413	-0.22758	15	45379815	F	II		IGR	opensea	IGR-opense		rs1466085	40
cg2119722	0.22701	0.577066	9.527865	1.7E-05	0.001032	3.588736	0.46356	0.690571	-0.22701	17	56082780	R	II	SFRS1	3'UTR	shore	3'UTR-shore	chr17:5608		
cg1618796	0.202475	0.62601	9.526804	1.7E-05	0.001032	3.58792	0.524773	0.727248	-0.20247	20	40332967	R	II		IGR	opensea	IGR-opense		rs1503732	7
cg1799699	0.25224	0.458614	9.52641	1.7E-05	0.001032	3.587617	0.332493	0.584734	-0.25224	11	86651974	F	II		IGR	opensea	IGR-opense		rs5448632	1
cg1974727	0.261892	0.489867	9.526306	1.7E-05	0.001032	3.587537	0.358921	0.620813	-0.26189	6	28553390	R	II	SCAND3	Body	shore	Body-shore	chr6:2855	rs1865280	2
cg0383232	0.200558	0.391211	9.525293	1.7E-05	0.001033	3.586758	0.290932	0.49149	-0.20056	18	28955953	R	II	DSG4	TSS1500	opensea	TSS1500-op		rs5291773	46;2
cg0294457	0.267511	0.660896	9.524567	1.7E-05	0.001033	3.5862	0.527141	0.794652	-0.26751	14	63762854	F	II		IGR	opensea	IGR-opense		rs7455086	4;5
cg2097580	0.221016	0.619886	9.52375	1.7E-05	0.001033	3.585571	0.509378	0.730394	-0.22102	1	76254925	R	II	RABGGTB	Body	opensea	Body-opens		rs5334552	43
cg1134131	0.204624	0.689791	9.522714	1.7E-05	0.001033	3.584775	0.587479	0.792104	-0.20462	11	18285729	F	II		IGR	opensea	IGR-opense		rs5434781	1;6;45
cg2288964	0.227078	0.40676	9.52227	1.7E-05	0.001033	3.584433	0.293221	0.520299	-0.22708	8	29107750	R	II	KIF13B	Body	opensea	Body-opens		rs1865629	28;27;18;2
cg0820239	0.214981	0.564468	9.520492	1.71E-05	0.001034	3.583066	0.456978	0.671959	-0.21498	7	1.4E+08	F	II	TBXAS1	5'UTR	opensea	5'UTR-oper		rs5580594	0;38
cg2587955	0.211508	0.604007	9.519741	1.71E-05	0.001034	3.582488	0.498253	0.709761	-0.21151	19	58620305	R	II	ZSCAN18	Body	opensea	Body-opens		rs2554981	34
cg0826803	0.272895	0.426734	9.51823	1.71E-05	0.001034	3.581326	0.290286	0.563181	-0.2729	1	2.3E+08	R	II		IGR	opensea	IGR-opense			
cg1241207	0.206251	0.472906	9.516693	1.71E-05	0.001035	3.580143	0.369781	0.576032	-0.20625	13	24566933	R	II	SPATA13	Body	opensea	Body-opens		rs9553131	35;3;1
cg0880180	0.239733	0.610766	9.515993	1.71E-05	0.001035	3.579604	0.4909	0.730633	-0.23973	1	2.44E+08	F	II		IGR	opensea	IGR-opense		rs5313164	1;30
cg2532673	0.230454	0.551492	9.515495	1.71E-05	0.001035	3.579221	0.436265	0.666719	-0.23045	4	2714321	R	II	FAM193A	Body	opensea	Body-opens		rs1126529	17;1
cg2400448	0.206022	0.662908	9.51458	1.71E-05	0.001035	3.578517	0.559897	0.765919	-0.20602	1	944783	R	I		IGR	shelf	IGR-shelf	chr1:9486	rs5542646	36;6
cg1423285	0.20953	0.43865	9.51458	1.71E-05	0.001035	3.578516	0.333885	0.543414	-0.20953	9	80111634	F	II	GNA14	Body	opensea	Body-opens		rs1156759	0
cg0643061	0.222124	0.579521	9.513325	1.71E-05	0.001036	3.57755	0.468459	0.690583	-0.22212	1	2.15E+08	F	II		IGR	opensea	IGR-opense		rs1931379	5;14;37
cg2024342	0.21756	0.506001	9.512514	1.72E-05	0.001036	3.576926	0.397221	0.614781	-0.21756	6	32059605	F	II	TNXB	Body	shelf	Body-shelf	chr6:3206		
cg0789186	0.202974	0.686311	9.512313	1.72E-05	0.001036	3.576771	0.584824	0.787798	-0.20297	8	1.42E+08	F	II	PTK2	Body	opensea	Body-opens		rs5586205	0
cg1327437	0.219389	0.706443	9.512264	1.72E-05	0.001036	3.576734	0.596748	0.816137	-0.21939	2	47516134	R	II	LOC10192	Body	opensea	Body-opens		rs1441624	8;2
cg1483498	0.201826	0.607219	9.511515	1.72E-05	0.001036	3.576157	0.506306	0.708132	-0.20183	18	43218779	F	II	SLC14A2	Body	opensea	Body-opens		rs5626695	1;27
cg0150329	0.244332	0.576604	9.508526	1.72E-05	0.001037	3.573855	0.454438	0.69877	-0.24433	4	2963264	F	I	NOP14	Body	shore	Body-shore	chr4:2964	rs7379210	14;21;41;45
cg2190475	0.215355	0.615752	9.508347	1.72E-05	0.001037	3.573717	0.508074	0.723429	-0.21535	12	87233925	R	II	MGAT4C	TSS1500	opensea	TSS1500-op		rs1932128	23;12
cg1113614	0.218533	0.563474	9.507919	1.72E-05	0.001037	3.573387	0.454207	0.67274	-0.21853	16	72988399	F	II	ZFHX3	Body	opensea	Body-opens			
cg0301394	0.202193	0.553325	9.507902	1.72E-05	0.001037	3.573374	0.452228	0.654421	-0.20219	20	44142512	R	II	SPINT3	Body	opensea	Body-opens		rs1810173	2
cg0065088	0.211993	0.572576	9.506225	1.72E-05	0.001038	3.572082	0.46658	0.678573	-0.21199	11	30533945	R	II	MPPED2	Body	opensea	Body-opens		rs1476658	39;21
cg1344953	0.215046	0.619623	9.505419	1.73E-05	0.001038	3.571461	0.5121	0.727145	-0.21505	1	1101348	R	I	MIR200B	TSS1500	shore	TSS1500-sh	chr1:1098	rs8029603	48;32
cg1701360	0.209413	0.706289	9.505084	1.73E-05	0.001038	3.571203	0.601582	0.810995	-0.20941	15	33435087	F	II	FMN1	Body	opensea	Body-opens		rs5423124	0;12
cg0476329	0.206083	0.602327	9.504209	1.73E-05	0.001039	3.570529	0.499285	0.705368	-0.20608	1	2.35E+08	R	II		IGR	opensea	IGR-opense		rs5455814	1
cg0278161	0.213567	0.502601	9.503911	1.73E-05	0.001039	3.570299	0.395818	0.609385	-0.21357	1	28843701	R	II	RCC1	TSS1500	shore	TSS1500-sh	chr1:2884		
cg1669479	0.201896	0.528984	9.50314	1.73E-05	0.001039	3.569705	0.428036	0.629932	-0.2019	4	88866784	R	II		IGR	opensea	IGR-opense		rs5491224	44;39
cg1295426	0.202721	0.635365	9.502718	1.73E-05	0.001039	3.56938	0.534004	0.736725	-0.20272	6	33093109	R	II	HLA-DPB2	Body	opensea	Body-opens		rs3750244	50;20;3
cg1202397	0.230973	0.501332	9.501711	1.73E-05	0.00104	3.568604	0.385845	0.616818	-0.23097	12	1.06E+08	F	II	NUAK1	Body	opensea	Body-opens		rs6094283	31
cg0424457	0.242159	0.606543	9.501611	1.73E-05	0.00104	3.568527	0.485464	0.727623	-0.24216	3	34323792	F	II		IGR	opensea	IGR-opense		rs5465365	25
cg1186386	0.205018	0.592471	9.500752	1.73E-05	0.00104	3.567865	0.489961	0.69498	-0.20502	17	75706410	R	II		IGR	opensea	IGR-opense		rs1494688	29;23
cg0260019	0.277543	0.529799	9.500502	1.73E-05	0.00104	3.567672	0.391028	0.668571	-0.27754	8	1.36E+08	R	II		IGR	opensea	IGR-opense		rs1861060	16;2
cg2437212	0.20941	0.573082	9.499235	1.73E-05	0.00104	3.566695	0.468377	0.677787	-0.20941	1	1.85E+08	R	II	FAM129A	Body	opensea	Body-opens		rs5379848	35;11
cg1008139	0.210687	0.548139	9.498546	1.73E-05	0.001041	3.566164	0.442795	0.653482	-0.21069	12	1.08E+08	R	II		IGR	opensea	IGR-opense		rs5359015	2
cg1320973	0.200717	0.680818	9.498287	1.73E-05	0.001041	3.565964	0.58046	0.781176	-0.20072	12	8995557	R	II	A2ML1	Body	opensea	Body-opens		rs7137125	36
cg1436210	0.206407	0.65174	9.497821	1.73E-05	0.001041	3.565605	0.548536	0.754944	-0.20641	1	91000048	R	II		IGR	opensea	IGR-opense		rs1135501	46;33
cg2443605	0.228234	0.431492	9.497277	1.74E-05	0.001041	3.565185	0.317375	0.545609	-0.22823	7	1.55E+08	R	II	DPP6	Body	opensea	Body-opens		rs5374288	31;29;16;12;2;1
cg0583581	0.242939	0.565675	9.49686	1.74E-05	0.001041	3.564864	0.444205	0.687144	-0.24294	1	1.54E+08	R	II		IGR	opensea	IGR-opense		rs5612743	35;9;6
cg1222066	0.208084	0.642396	9.495291	1.74E-05	0.001042	3.563654	0.538354	0.746439	-0.20808	2	20194085	F	II	MATN3	Body	shelf	Body-shelf	chr2:2018	rs5312931	32
cg1511024	0.202872	0.481266	9.49522	1.74E-05	0.001042	3.563599	0.37983	0.582702	-0.20287	11	78501744	F	II	ODZ4	Body	opensea	Body-opens		rs5355961	1
cg1717844	0.21348	0.622799	9.494397	1.74E-05	0.001042	3.562964	0.516059	0.729539	-0.21348	10	23701242	R	II		IGR	opensea	IGR-opense		rs2004404	48;7
cg1607334	0.229139	0.537509	9.491002	1.74E-05	0.001043	3.560345	0.422939	0.652078	-0.22914	20	31631564	R	II	BPIFB6	Body	opensea	Body-opens		rs7942346	46
cg1332058	0.251092	0.44184	9.490881	1.74E-05	0.001043	3.560251	0.316294	0.567386	-0.25109	12	79812901	R	II	MIR1252	TSS200	opensea	TSS200-ope		rs5295216	46;2
cg2346748	0.219496	0.534155	9.488442	1.75E-05	0.001044	3.558369	0.424408	0.643903	-0.2195	1	1.75E+08	F	II	RABGAP1L	Body	opensea	Body-opens		rs1812494	26;46
cg1147324	0.209878	0.604213	9.486663	1.75E-05	0.001045	3.556996	0.499274	0.709152	-0.20988	17	39553960	R	II	KRT31	TSS200	opensea	TSS200-ope			
cg1022538	0.230884	0.442197	9.485604	1.75E-05	0.001045	3.556178	0.326755	0.557639	-0.23088	6	1.09E+08	F	II	ARMC2	5'UTR	opensea	5'UTR-oper		rs5521452	2;4
cg2111359	0.255875	0.53552	9.484502	1.75E-05	0.001046	3.555327	0.407582	0.663457	-0.25588	14	31858067	R	II	HEATR5A	1stExon	opensea	1stExon-op		rs3887865	44;23;2
cg0205154	0.207521	0.711741	9.483459	1.75E-05	0.001046	3.554522	0.607981	0.815502	-0.20752	1	2.4E+08	R	II		IGR	shelf	IGR-shelf	chr1:2401	rs5772036	47;46
cg1472936	0.206654	0.394452	9.482502	1.75E-05	0.001046	3.553782	0.291125	0.497779	-0.20665	16	77364437	F	II	ADAMTS18	Body	opensea	Body-opens		rs274530;r	16;17;46
cg2734309	0.219614	0.605926	9.480912	1.76E-05	0.001047	3.552554	0.496118	0.715733	-0.21961	2	86025560	R	II		IGR	opensea	IGR-opense		rs5503525	2
cg0658392	0.230017	0.484437	9.480464	1.76E-05	0.001047	3.552209	0.369429	0.599446	-0.23002	3	1.93E+08	R	II		IGR	opensea	IGR-opense			
cg0677265	0.200443	0.480389	9.480256	1.76E-05	0.001047	3.552048	0.380168	0.58061	-0.20044	5	38048847	F	II		IGR	opensea	IGR-opense			
cg2354609	0.204682	0.508491	9.479879	1.76E-05	0.001047	3.551756	0.40615	0.610832	-0.20468	16	15740943	F	II	NDE1	5'UTR	shelf	5'UTR-shelf	chr16:157	rs1465391	17;22;30;37;40
cg1626593	0.260008	0.445822	9.47956	1.76E-05	0.001047	3.55151	0.315818	0.575825	-0.26001	10	1.12E+08	R	II	RBM20	Body	opensea	Body-opens		rs5287368	41
cg0806342	0.224469	0.63927	9.479323	1.76E-05	0.001047	3.551327	0.527035	0.751504	-0.22447	2	2.18E+08	F	II		IGR	opensea	IGR-opense		rs1442196	0
cg1792263	0.209302	0.504172	9.47882	1.76E-05	0.001047	3.550938	0.399521	0.608823	-0.2093	1	69988995	F	II		IGR	opensea	IGR-opense		rs1861992	36
cg2065544	0.200643	0.523203	9.478788	1.76E-05	0.001047	3.550914	0.422881	0.623524	-0.20064	13	86240791	F	II		IGR	opensea	IGR-opense		rs4324043	41
cg0475943	0.22591	0.40581	9.478513	1.76E-05	0.001047	3.550701	0.292855	0.518764	-0.22591	8	50242329	F	II		IGR	opensea	IGR-opense		rs1843899	0;27
cg0082985	0.28173	0.47357	9.478448	1.76E-05	0.001047	3.550651	0.332705	0.614435	-0.28173	2	1.79E+08	F	II	OSBPL6	5'UTR	opensea	5'UTR-oper		rs5694126	0;1
cg1274324	0.213357	0.415142	9.47791	1.76E-05	0.001047	3.550235	0.308464	0.52182	-0.21336	8	49636840	R	II	EFCAB1	3'UTR	opensea	3'UTR-oper		rs5582310	28;22;17
cg2082502	0.201272	0.480695	9.475794	1.76E-05	0.001048	3.5486	0.38006	0.581331	-0.20127	2	1.25E+08	R	II	CNTNAP5	Body	opensea	Body-opens		rs1882126	1
cg0396225	0.253546	0.523242	9.472327	1.77E-05	0.001049	3.54592	0.39647	0.650015	-0.25355	13	96211557	R	II	CLDN10	Body	opensea	Body-opens		rs5545256	24
cg0452743	0.202711	0.573136	9.4716	1.77E-05	0.001049	3.545358	0.47178	0.674491	-0.20271	11	1.12E+08	F	II	DLAT	Body	opensea	Body-opens		rs3699024	0;25
cg2549646	0.219426	0.523581	9.471255	1.77E-05	0.001049	3.545091	0.413868	0.633293	-0.21943	15	76258485	R	II	NRG4	Body	opensea	Body-opens		rs5429589	28;10;9
cg1987159	0.220037	0.493113	9.470258	1.77E-05	0.001049	3.54432	0.383094	0.603132	-0.22004	13	59592287	F	II		IGR	opensea	IGR-opense			
cg1511478	0.20871	0.478162	9.470024	1.77E-05	0.001049	3.544139	0.373807	0.582517	-0.20871	5	31745603	F	II		IGR	opensea	IGR-opense		rs5633689	0;6;12;32
cg1740408	0.218462	0.483272	9.469302	1.77E-05	0.001049	3.543581	0.374041	0.592503	-0.21846	3	39449544	F	II	SNORA6	TSS1500	shore	TSS1500-sh	chr3:3944	rs1810418	1
cg0408314	0.237928	0.476157	9.468528	1.77E-05	0.001049	3.542982	0.357193	0.59512	-0.23793	17	64028625	R	II	CEP112	Body	opensea	Body-opens		rs5435131	42;2
cg0801496	0.22195	0.475943	9.467875	1.77E-05	0.00105	3.542477	0.364968	0.586918	-0.22195	17	10372972	F	II	MYH4	TSS200	opensea	TSS200-ope		rs1814821	1
cg1318615	0.21895	0.481275	9.467503	1.77E-05	0.00105	3.542189	0.371799	0.59075	-0.21895	1	86063729	R	II		IGR	opensea	IGR-opense		rs1849554	49;48
cg0688211	0.20319	0.506376	9.467176	1.78E-05	0.00105	3.541936	0.404781	0.607971	-0.20319	12	39725994	R	II	KIF21A	Body	opensea	Body-opens		rs7834430	2
cg0449163	0.268863	0.532912	9.465714	1.78E-05	0.00105	3.540805	0.39848	0.667344	-0.26886	2	1.74E+08	F	II	ZAK	Body	opensea	Body-opens		rs1116036	0;21
cg0250734	0.201334	0.62064	9.465226	1.78E-05	0.00105	3.540428	0.519973	0.721307	-0.20133	13	40714464	R	II		IGR	opensea	IGR-opense		rs5359402	25;24
cg0001410	0.229755	0.560633	9.463853	1.78E-05	0.001051	3.539365	0.445756	0.675511	-0.22976	22	22220367	R	II	MAPK1	Body	shore	Body-shore	chr22:222	rs5293634	2
cg2213009	0.238412	0.611324	9.46384	1.78E-05	0.001051	3.539355	0.492118	0.73053	-0.23841	2	59098148	F	II	LINC01122	Body	opensea	Body-opens		rs7553148	0;6;26
cg0784263	0.232468	0.52817	9.46367	1.78E-05	0.001051	3.539223	0.411936	0.644404	-0.23247	13	47862393	F	II		IGR	opensea	IGR-opense		rs1409901	46
cg1238629	0.215222	0.513408	9.462742	1.78E-05	0.001051	3.538506	0.405797	0.621018	-0.21522	1	2.1E+08	R	II	LOC64258	Body	opensea	Body-opens		rs1174186	2
cg2453318	0.204188	0.575055	9.461064	1.78E-05	0.001052	3.537207	0.472961	0.677149	-0.20419	1	2.1E+08	F	II		IGR	opensea	IGR-opense		rs1437442	21;45
cg2393984	0.255781	0.469867	9.459359	1.79E-05	0.001053	3.535887	0.341977	0.597758	-0.25578	6	3585854	R	II		IGR	opensea	IGR-opense		rs7372105	13;12
cg2660857	0.203219	0.518075	9.459331	1.79E-05	0.001053	3.535865	0.416465	0.619684	-0.20322	1	2.06E+08	F	II	SLC26A9	Body	opensea	Body-opens		rs1467772	27;43;45

cg0151816	0.23272	0.647749	9.4591	1.79E-05	0.001053	3.535686	0.531389	0.764109	-0.23272	6	92828798	F	II		IGR	opensea	IGR-opense		rs1174980	1;3;44
cg1221499	0.206656	0.606989	9.458588	1.79E-05	0.001053	3.53529	0.50366	0.710317	-0.20666	10	49764906	R	II	ARHGAP22	Body	opensea	Body-opens			
cg2454543	0.21652	0.610226	9.458402	1.79E-05	0.001053	3.535146	0.501966	0.718486	-0.21652	2	2.07E+08	R	II	ADAM23	Body	opensea	Body-opens		rs5765901	33;27
cg0397805	0.215692	0.693094	9.456842	1.79E-05	0.001053	3.533938	0.585248	0.800941	-0.21569	2	26740200	F	II	OTOF	Body	opensea	Body-opens			
cg0193448	0.217764	0.556476	9.452999	1.79E-05	0.001055	3.530961	0.447594	0.665358	-0.21776	8	1.41E+08	R	II	TRAPPC9	Body	opensea	Body-opens		rs7666046	12
cg2522615	0.218673	0.466458	9.452441	1.79E-05	0.001056	3.530529	0.357122	0.575795	-0.21867	10	89554130	R	II	ATAD1	Body	opensea	Body-opens			
cg0178974	0.217181	0.588683	9.452375	1.79E-05	0.001056	3.530478	0.480093	0.697274	-0.21718	1	2.36E+08	F	II	NID1	Body	shelf	Body-shelf	chr1:2361!	rs5351212	5;31
cg1335025	0.24582	0.583829	9.45181	1.8E-05	0.001056	3.530041	0.460919	0.706739	-0.24582	15	42344200	F	II	PLA2G4E	TSS1500	opensea	TSS1500-op		rs5443423	0;17
cg2435067	0.217577	0.4841	9.45113	1.8E-05	0.001056	3.529513	0.375312	0.592889	-0.21758	7	83878136	F	II		IGR	opensea	IGR-opense		rs5476220	26;43
cg1722122	0.244294	0.451466	9.449419	1.8E-05	0.001056	3.528188	0.329319	0.573613	-0.24429	11	64414192	F	II	NRXN2	Body	shelf	Body-shelf	chr11:644(	rs1813472	0;10;15
cg0999221	0.244371	0.443633	9.448873	1.8E-05	0.001057	3.527764	0.321447	0.565818	-0.24437	11	32353565	R	II		IGR	shore	IGR-shore	chr11:323!		
cg0581590	0.208188	0.449012	9.448091	1.8E-05	0.001057	3.527158	0.344918	0.553106	-0.20819	9	18472812	F	II	ADAMTSL1	TSS1500	opensea	TSS1500-op		rs5661056	31;34;36
cg1283376	0.215552	0.427266	9.446916	1.8E-05	0.001057	3.526247	0.31949	0.535042	-0.21555	2	69470878	F	II	ANTXR1	Body	opensea	Body-opens		rs1436090	1;18;46
cg0662591	0.302976	0.517093	9.445908	1.8E-05	0.001058	3.525466	0.365605	0.668581	-0.30298	4	1.2E+08	F	II	SEC24D	Body	opensea	Body-opens			
cg0518983	0.20187	0.657591	9.445322	1.8E-05	0.001058	3.525012	0.556656	0.758527	-0.20187	1	2.2E+08	F	II	RNU5F-1	Body	opensea	Body-opens		rs5674268	1
cg2337668	0.240866	0.589659	9.444995	1.8E-05	0.001058	3.524758	0.469227	0.710092	-0.24087	6	3324763	F	II	SLC22A23	Body	opensea	Body-opens		rs5282426	0;7;38
cg0357743	0.207009	0.596301	9.443642	1.81E-05	0.001059	3.523709	0.492797	0.699805	-0.20701	2	1.75E+08	F	II		IGR	opensea	IGR-opense			
cg2631452	0.244331	0.472568	9.443493	1.81E-05	0.001059	3.523594	0.350402	0.594733	-0.24433	5	1.63E+08	R	II		IGR	opensea	IGR-opense			
cg1670340	0.210859	0.672458	9.443315	1.81E-05	0.001059	3.523455	0.567028	0.777887	-0.21086	15	64540345	R	II	CSNK1G1	Body	opensea	Body-opens		rs1389649	6;2
cg2476296	0.202786	0.656335	9.442595	1.81E-05	0.001059	3.522897	0.554942	0.757728	-0.20279	5	60937901	R	II	FLJ37543	5'UTR	opensea	5'UTR-oper			
cg1562530	0.204273	0.621209	9.442579	1.81E-05	0.001059	3.522885	0.519073	0.723346	-0.20427	5	1.43E+08	R	II		IGR	opensea	IGR-opense		rs5446488	14;1
cg2062715	0.20661	0.436303	9.44085	1.81E-05	0.00106	3.521543	0.332998	0.539608	-0.20661	8	1.11E+08	R	II		IGR	opensea	IGR-opense		rs1050514	25;1
cg1965882	0.241857	0.594539	9.440667	1.81E-05	0.001061	3.521401	0.47361	0.715467	-0.24186	3	1.88E+08	R	II		IGR	opensea	IGR-opense			
cg0557979	0.206568	0.459107	9.438312	1.81E-05	0.001062	3.519574	0.355823	0.562391	-0.20657	10	1.04E+08	R	II		IGR	opensea	IGR-opense		rs5277668	50;27
cg1866446	0.211558	0.453814	9.437176	1.82E-05	0.001062	3.518693	0.348035	0.559593	-0.21156	8	58163581	R	II		IGR	opensea	IGR-opense		rs5628264	15;1
cg2206927	0.213694	0.505478	9.437068	1.82E-05	0.001062	3.518609	0.398631	0.612325	-0.21369	13	67205408	R	II	PCDH9	Body	opensea	Body-opens		rs1458948	24
cg0853044	0.23362	0.520746	9.437058	1.82E-05	0.001062	3.518601	0.403936	0.637556	-0.23362	1	55944299	R	II		IGR	opensea	IGR-opense		rs5666229	27;1
cg1498181	0.234207	0.583385	9.436347	1.82E-05	0.001063	3.51805	0.466282	0.700489	-0.23421	7	96597711	F	II		IGR	opensea	IGR-opense		rs5549519	22
cg0791683	0.203285	0.434556	9.435432	1.82E-05	0.001063	3.51734	0.332913	0.536199	-0.20329	5	89517850	R	II	LINC01339	Body	opensea	Body-opens		rs1005973	19;17;10;7
cg1048805	0.225032	0.682125	9.434138	1.82E-05	0.001064	3.516335	0.569609	0.794641	-0.22503	7	1.33E+08	F	II	CHCHD3	Body	opensea	Body-opens			
cg0192854	0.20085	0.504601	9.433621	1.82E-05	0.001064	3.515934	0.404176	0.605026	-0.20085	4	48972614	R	II		IGR	opensea	IGR-opense		rs5642916	25;1
cg2116803	0.201787	0.597703	9.433128	1.82E-05	0.001064	3.515551	0.49681	0.698597	-0.20179	13	74703602	R	II	KLF12	5'UTR	opensea	5'UTR-oper		rs5390619	32
cg0843448	0.208648	0.670827	9.432818	1.82E-05	0.001064	3.51531	0.566502	0.775151	-0.20865	14	34771014	F	II		IGR	opensea	IGR-opense		rs1481305	0;1;11;23
cg2568743	0.213636	0.569371	9.432804	1.82E-05	0.001064	3.515299	0.462553	0.676189	-0.21364	18	72006048	F	II	C18orf63	ExonBnd	opensea	ExonBnd-op		rs1155169	17
cg2487546	0.210283	0.62707	9.42992	1.83E-05	0.001065	3.51306	0.521928	0.732212	-0.21028	10	34864942	F	II	PARD3	Body	opensea	Body-opens		rs5671771	6;42
cg0936726	0.245611	0.583584	9.429806	1.83E-05	0.001065	3.512972	0.460778	0.706389	-0.24561	6	6643814	F	II	LY86	Body	opensea	Body-opens		rs1903988	41
cg0486617	0.201732	0.46598	9.429763	1.83E-05	0.001065	3.512938	0.365114	0.566846	-0.20173	2	1.06E+08	F	II	FHL2	Body	opensea	Body-opens		rs1838274	17;42;46
cg2700415	0.209064	0.600834	9.427962	1.83E-05	0.001066	3.51154	0.496301	0.705366	-0.20906	8	82261965	R	II		IGR	opensea	IGR-opense			
cg2159389	0.222707	0.496482	9.426707	1.83E-05	0.001066	3.510565	0.385129	0.607835	-0.22271	12	1.09E+08	R	II	CORO1C	Body	opensea	Body-opens		rs1914517	1
cg0747313	0.227213	0.620295	9.426106	1.83E-05	0.001066	3.510098	0.506688	0.733901	-0.22721	10	1.22E+08	F	II		IGR	shelf	IGR-shelf	chr10:122;		
cg1000358	0.230331	0.543095	9.424973	1.83E-05	0.001067	3.509217	0.427929	0.65826	-0.23033	6	56755793	R	II	DST	Body	opensea	Body-opens		rs5579069	48;41;32;22
cg0042833	0.200598	0.554885	9.42194	1.84E-05	0.001067	3.50686	0.454585	0.655184	-0.2006	5	59553800	F	II	PDE4D	5'UTR	opensea	5'UTR-oper			
cg0005069	0.219513	0.468922	9.421734	1.84E-05	0.001067	3.5067	0.359165	0.578678	-0.21951	2	25524877	R	II	DNMT3A	Body	opensea	Body-opens		rs1813232	33
cg0740829	0.239629	0.636028	9.420175	1.84E-05	0.001068	3.505489	0.516213	0.755843	-0.23963	17	67038755	F	II	ABCA9	Body	opensea	Body-opens		rs5772285	24;28
cg0984015	0.216528	0.616416	9.418139	1.84E-05	0.001069	3.503906	0.508152	0.72468	-0.21653	11	10716592	F	II	MRVI1	TSS1500	opensea	TSS1500-op		rs5506164	0
cg0096624	0.222281	0.438193	9.418139	1.84E-05	0.001069	3.503906	0.327053	0.549334	-0.22228	19	11819352	R	II		IGR	opensea	IGR-opense		rs5316481	36;17;8
cg1203661	0.278659	0.480439	9.417307	1.84E-05	0.001069	3.503258	0.341109	0.619768	-0.27866	4	80929171	R	II	ANTXR2	Body	opensea	Body-opens		rs5659504	28;2;1
cg0874102	0.219829	0.490778	9.416767	1.84E-05	0.001069	3.502839	0.380863	0.600692	-0.21983	11	76455305	F	II		IGR	opensea	IGR-opense		rs1807350	19;20;27
cg1534855	0.254013	0.503734	9.415528	1.85E-05	0.001069	3.501875	0.376728	0.63074	-0.25401	10	12163898	R	II	DHTKD1	3'UTR	opensea	3'UTR-oper		rs1150130	35;23
cg2166535	0.245226	0.567825	9.414976	1.85E-05	0.00107	3.501446	0.445212	0.690437	-0.24523	6	1.56E+08	R	II		IGR	opensea	IGR-opense			
cg0963330	0.213136	0.589528	9.410818	1.85E-05	0.001072	3.498211	0.48296	0.696096	-0.21314	2	65678133	R	II		IGR	opensea	IGR-opense			
cg2688618	0.207055	0.619515	9.409677	1.85E-05	0.001072	3.497323	0.515987	0.723042	-0.20706	6	4251695	F	II		IGR	shelf	IGR-shelf	chr6:4254!	rs3683205	0;1;17;30;39;40;46
cg0271466	0.205313	0.608372	9.409665	1.85E-05	0.001072	3.497314	0.505716	0.711029	-0.20531	3	16131767	R	II		IGR	opensea	IGR-opense		rs1837935	45
cg0033459	0.208753	0.614914	9.409506	1.85E-05	0.001072	3.49719	0.510538	0.71929	-0.20875	13	31641914	F	II		IGR	opensea	IGR-opense		rs5612970	0;20
cg2595419	0.220401	0.634124	9.409383	1.85E-05	0.001072	3.497094	0.523923	0.744324	-0.2204	5	1.32E+08	R	II	IL5	TSS200	opensea	TSS200-ope		rs1906949	43;1
cg1110815	0.221008	0.583589	9.408507	1.85E-05	0.001072	3.496412	0.473085	0.694093	-0.22101	13	86373073	R	II	SLITRK6	5'UTR	opensea	5'UTR-oper			
cg1258523	0.212042	0.590142	9.40732	1.86E-05	0.001073	3.495489	0.484121	0.696164	-0.21204	10	44918789	F	II		IGR	opensea	IGR-opense		rs5372645	1
cg0203851	0.204333	0.575858	9.405797	1.86E-05	0.001073	3.494303	0.473692	0.678025	-0.20433	2	1.82E+08	F	II	SCHLAP1	Body	opensea	Body-opens		rs1906597	1;2;49
cg0357137	0.225376	0.423483	9.405364	1.86E-05	0.001073	3.493966	0.310795	0.536171	-0.22538	22	22935973	F	II		IGR	opensea	IGR-opense			
cg0105970	0.208854	0.693646	9.4041	1.86E-05	0.001073	3.492982	0.589219	0.798074	-0.20885	5	34914428	R	II	RAD1	Body	shore	Body-shore	chr5:3491!	rs2069470	26
cg1546355	0.244299	0.4736	9.402061	1.86E-05	0.001074	3.491394	0.35145	0.595749	-0.2443	13	21904723	F	II	LINC00539	ExonBnd	opensea	ExonBnd-op		rs5703387	0;29;41;42
cg0683222	0.224308	0.634222	9.401423	1.86E-05	0.001075	3.490897	0.522068	0.746376	-0.22431	6	14192840	F	II		IGR	opensea	IGR-opense		rs5569941	47
cg0964430	0.20367	0.647061	9.4013	1.86E-05	0.001075	3.490801	0.545226	0.748896	-0.20367	13	72022552	F	II	DACH1	Body	opensea	Body-opens		rs5722599	6
cg1415368	0.217627	0.468266	9.400567	1.87E-05	0.001075	3.49023	0.359453	0.57708	-0.21763	4	10732403	R	II		IGR	opensea	IGR-opense		rs1897782	47;1
cg0251033	0.214621	0.463639	9.400021	1.87E-05	0.001075	3.489805	0.356329	0.57095	-0.21462	6	1186717	F	II		IGR	opensea	IGR-opense		rs1154428	35;40;51
cg1395975	0.238775	0.639419	9.399284	1.87E-05	0.001075	3.48923	0.520032	0.758807	-0.23877	2	2.07E+08	F	II	ADAM23	Body	opensea	Body-opens		rs1112685	31
cg1954657	0.23097	0.540918	9.399217	1.87E-05	0.001075	3.489179	0.425433	0.656403	-0.23097	6	1.01E+08	R	II		IGR	shore	IGR-shore	chr6:1006;	rs1415675	25
cg0528680	0.250573	0.418622	9.398885	1.87E-05	0.001075	3.48892	0.293336	0.543908	-0.25057	4	78274985	R	II		IGR	opensea	IGR-opense		rs7285981	12;2;1
cg0624433	0.222673	0.577733	9.397783	1.87E-05	0.001076	3.488062	0.466396	0.689069	-0.22267	6	29555026	R	II	OR2H2	TSS1500	opensea	TSS1500-op		rs5777860	10
cg2178131	0.206968	0.586508	9.397542	1.87E-05	0.001076	3.487873	0.483024	0.689992	-0.20697	17	61020813	F	II	MIR633	TSS1500	opensea	TSS1500-op		rs5419856	29;35
cg0156178	0.245869	0.670553	9.395176	1.87E-05	0.001077	3.48603	0.547619	0.793487	-0.24587	9	1.01E+08	F	II	GABBR2	Body	opensea	Body-opens			
cg0079154	0.206661	0.606257	9.395142	1.87E-05	0.001077	3.486003	0.502927	0.709588	-0.20666	2	30966534	R	II	CAPN13	Body	opensea	Body-opens		rs1420063	32;31;8;2
cg0257500	0.22146	0.538628	9.394677	1.87E-05	0.001077	3.485641	0.427899	0.649358	-0.22146	2	46897669	R	II		IGR	opensea	IGR-opense		rs1448545	1
cg0967648	0.212502	0.601672	9.394615	1.87E-05	0.001077	3.485593	0.495421	0.707923	-0.2125	17	49232155	R	II	NME1	5'UTR	shore	5'UTR-shor	chr17:492;	rs5778731	48;42;20;2
cg1996939	0.216475	0.431972	9.393783	1.88E-05	0.001077	3.484944	0.323734	0.54021	-0.21648	18	24167674	F	II	KCTD1	Body	opensea	Body-opens		rs5292305	28
cg2260817	0.206661	0.583634	9.393256	1.88E-05	0.001077	3.484533	0.480304	0.686964	-0.20666	19	39290548	F	II		IGR	opensea	IGR-opense			
cg1069826	0.200165	0.649422	9.392004	1.88E-05	0.001078	3.483557	0.54934	0.749504	-0.20016	5	1.58E+08	F	II	EBF1	Body	opensea	Body-opens		rs1164709	9;28
cg0386613	0.22385	0.363205	9.390603	1.88E-05	0.001078	3.482464	0.251281	0.47513	-0.22385	5	1.33E+08	F	II		IGR	opensea	IGR-opense		rs1124217	10;15
cg0522162	0.216867	0.582364	9.389681	1.88E-05	0.001079	3.481746	0.47393	0.690797	-0.21687	5	55593516	F	II		IGR	opensea	IGR-opense		rs5656334	35
cg1725160	0.207875	0.556572	9.389199	1.88E-05	0.001079	3.48137	0.452635	0.66051	-0.20787	3	65489574	R	II	MAGI1	Body	opensea	Body-opens		rs5378932	42;12
cg1549470	0.2135	0.501399	9.38753	1.88E-05	0.00108	3.480068	0.394649	0.608149	-0.2135	15	45268816	R	II	C15orf43	Body	opensea	Body-opens			
cg1384513	0.222771	0.546406	9.384062	1.89E-05	0.001081	3.477362	0.43502	0.657791	-0.22277	1	1.65E+08	R	II	PBX1	Body	shore	Body-shore	chr1:1645		
cg1680729	0.255723	0.495932	9.38243	1.89E-05	0.001082	3.476089	0.36807	0.623793	-0.25572	16	14375926	F	II		IGR	shelf	IGR-shelf	chr16:143;	rs1850731	0;3
cg1968397	0.209205	0.53147	9.381711	1.89E-05	0.001082	3.475528	0.426868	0.636073	-0.2092	2	57725263	R	II		IGR	opensea	IGR-opense		rs5293954	23;2
cg2669568	0.222295	0.625558	9.380916	1.89E-05	0.001082	3.474907	0.51441	0.736705	-0.2223	17	64269194	F	II		IGR	opensea	IGR-opense		rs5301052	25;50
cg1892925	0.239099	0.488334	9.380191	1.89E-05	0.001082	3.474341	0.368785	0.607884	-0.2391	7	1.08E+08	R	II	LAMB4	Body	opensea	Body-opens		rs5446010	34;26;2;1
cg1943907	0.202839	0.503406	9.37952	1.9E-05	0.001082	3.473817	0.401987	0.604826	-0.20284	17	74557625	F	II	SNORD1A	TSS200	shelf	TSS200-she	chr17:745!	rs1871478	1;5
cg0608288	0.21449	0.533424	9.379446	1.9E-05	0.001082	3.473759	0.426179	0.640669	-0.21449	16	89686744	R	II	DPEP1	5'UTR	opensea	5'UTR-oper		rs7488343	28;2
cg0122137	0.26347	0.455543	9.378041	1.9E-05	0.001083	3.472662	0.323808	0.587278	-0.26347	17	1263732	F	II	YWHAE	Body	opensea	Body-opens		rs7863643	27
cg2181490	0.219827	0.525483	9.37603	1.9E-05	0.001084	3.471092	0.41557	0.635397	-0.21983	10	13812297	F	II	FRMD4A	Body	opensea	Body-opens		rs1886019	0;26;27;44
cg1255718	0.225796	0.657851	9.374497	1.9E-05	0.001085	3.469894	0.544952	0.770749	-0.2258	2	1.9E+08	F	II		IGR	opensea	IGR-opense		rs5658390	0
cg1916317	0.223988	0.363687	9.372945	1.91E-05	0.001085	3.468682	0.251693	0.475681	-0.22399	8	1.21E+08	F	II		IGR	opensea	IGR-opense		rs5693639	0;47
cg1340841	0.258172	0.59922	9.37279	1.91E-05	0.001085	3.468561	0.470134	0.728306	-0.25817	1	1.53E+08	F	II		IGR	opensea	IGR-opense			
cg2472374	0.244884	0.541353	9.371798	1.91E-05	0.001086	3.467786	0.418911	0.663794	-0.24488	9	1.01E+08	F	II	TRIM14	Body	opensea	Body-opens			
cg0728549	0.262968	0.518765	9.371791	1.91E-05	0.001086	3.467781	0.387281	0.650249	-0.26297	1	32071681	F	II		IGR	opensea	IGR-opense		rs5399555	24;49
cg2097140	0.225719	0.542173	9.371698	1.91E-05	0.001086	3.467708	0.429313	0.655032	-0.22572	3	5022392	R	II	BHLHE40	Body	shore	Body-shore	chr3:5019!		
cg1197613	0.218398	0.564612	9.371272	1.91E-05	0.001086	3.467375	0.455413	0.673811	-0.2184	15	63113869	R	II	TLN2	Body	opensea	Body-opens		rs1149510	2
cg0632549	0.235066	0.685673	9.370963	1.91E-05	0.001086	3.467134	0.56814	0.803206	-0.23507	8	10767745	R	II	XKR6	Body	opensea	Body-opens		rs1410685	1
cg0228710	0.219586	0.573434	9.37031	1.91E-05	0.001086	3.466624	0.463641	0.683227	-0.21959	6	32115284	R	I		IGR	shore	IGR-shore	chr6:3211(	rs1380605	47;17;13;2
cg1963737	0.245954	0.505474	9.370116	1.91E-05	0.001086	3.466472	0.382497	0.628451	-0.24595	17	57907234	F	II	VMP1	Body	opensea	Body-opens		rs1143354	0;1
cg0227554	0.248008	0.60594	9.369101	1.91E-05	0.001086	3.465678	0.481936	0.729944	-0.24801	8	19040543	R	II		IGR	opensea	IGR-opense		rs5608581	1
cg2266935	0.212949	0.631507	9.368187	1.91E-05	0.001086	3.464965	0.525033	0.737982	-0.21295	5	1.51E+08	R	II		IGR	opensea	IGR-opense		rs1386673	5
cg0476630	0.223965	0.643275	9.367299	1.91E-05	0.001087	3.464271	0.531292	0.755257	-0.22396	6	18047219	R	II		IGR	opensea	IGR-opense		rs3748844	27;26;1
cg0179274	0.22226	0.475185	9.365742	1.92E-05	0.001087	3.463053	0.364055	0.586315	-0.22226	3	38835179	R	II	SCN10A	Body	opensea	Body-opens		rs5436902	29;9;8
cg0207907	0.269017	0.474324	9.364868	1.92E-05	0.001088	3.462369	0.339816	0.608833	-0.26902	1	2.08E+08	R	II		IGR	opensea	IGR-opense		rs5520171	9;8
cg2714917	0.221272	0.635236	9.364487	1.92E-05	0.001088	3.462072	0.5246	0.745872	-0.22127	11	86451848	F	II		IGR	opensea	IGR-opense		rs5414569	38;48
cg1155448	0.214345	0.614357	9.363709	1.92E-05	0.001088	3.461464	0.507185	0.72153	-0.21434	1	1.88E+08	F	II		IGR	opensea	IGR-opense		rs5536702	25;29
cg1736534	0.207071	0.659218	9.361071	1.92E-05	0.00109	3.459401	0.555682	0.762754	-0.20707	4	1.07E+08	F	II	ARHGEF38	3'UTR	opensea	3'UTR-oper		rs7709654	38
cg0235075	0.23323	0.584038	9.360654	1.92E-05	0.00109	3.459075	0.467423	0.700653	-0.23323	4	55898322	R	II		IGR	opensea	IGR-opense		rs1382338	37;1
cg0959553	0.208444	0.550513	9.35924	1.93E-05	0.00109	3.457968	0.446292	0.654735	-0.20844	18	66597897	F	II	CCDC102B	Body	opensea	Body-opens			
cg0429259	0.247709	0.374788	9.357272	1.93E-05	0.001091	3.456428	0.250933	0.498642	-0.24771	9	1.13E+08	F	II	PALM2	Body	opensea	Body-opens		rs5661767	0;28
cg0287964	0.266059	0.616182	9.356934	1.93E-05	0.001091	3.456163	0.483152	0.749211	-0.26606	2	2.34E+08	F	II	KCNJ13	5'UTR	opensea	5'UTR-oper			
cg2137475	0.207468	0.57973	9.356547	1.93E-05	0.001091	3.455861	0.475996	0.683464	-0.20747	3	42452862	F	II	LYZL4	TSS1500	opensea	TSS1500-op		rs5660270	37
cg1264048	0.204289	0.556148	9.356502	1.93E-05	0.001091	3.455826	0.454004	0.658293	-0.20429	1	28905573	F	II	SNHG12	Body	shelf	Body-shelf	chr1:2890!	rs1829755	0;18
cg0764149	0.234674	0.565943	9.355828	1.93E-05	0.001091	3.455299	0.448606	0.68328	-0.23467	22	33195343	F	II	SYN3	Body	shelf	Body-shelf	chr22:331!	rs1395135	0
cg2169950	0.205058	0.533472	9.355408	1.93E-05	0.001091	3.45497	0.430943	0.636001	-0.20506	4	80786707	F	II		IGR	opensea	IGR-opense		rs1389780	0;1
cg2189686	0.228811	0.462679	9.355343	1.93E-05	0.001091	3.454919	0.348274	0.577085	-0.22881	1	32625023	R	II	KPNA6	Body	opensea	Body-opens			
cg2144174	0.214724	0.581831	9.354069	1.93E-05	0.001092	3.453921	0.474469	0.689193	-0.21472	5	1.22E+08	R	I	LOC10050!	Body	opensea	Body-opens		rs7329152	48;30;16;2
cg1735143	0.202413	0.577684	9.35399	1.93E-05	0.001092	3.45386	0.476478	0.67889	-0.20241	13	93888067	R	II	GPC6	Body	opensea	Body-opens		rs1436027	2

cg1140446	0.264518	0.432603	9.353462	1.93E-05	0.001092	3.453446	0.300344	0.564862	-0.26452	2	1.06E+08	R	II		IGR	opensea	IGR-opense			
cg2526116	0.203509	0.731296	9.353431	1.93E-05	0.001092	3.453422	0.629542	0.833051	-0.20351	11	47123246	R	II	C11orf49	Body	opensea	Body-opens		rs5575349	1
cg0191877	0.235079	0.536748	9.353241	1.93E-05	0.001092	3.453273	0.419209	0.654287	-0.23508	15	40144688	R	II	GPR176	Body	opensea	Body-opens		rs7668831	21;16
cg0818939	0.201592	0.526253	9.351962	1.94E-05	0.001092	3.452272	0.425457	0.627049	-0.20159	10	1.14E+08	F	II		IGR	opensea	IGR-opense		rs5419473	0;10;24
cg1406875	0.213068	0.571992	9.351735	1.94E-05	0.001092	3.452095	0.465458	0.678526	-0.21307	5	59019044	F	II	PDE4D	Body	opensea	Body-opens		rs5730788	47
cg0348331	0.202473	0.590313	9.351599	1.94E-05	0.001092	3.451988	0.489077	0.69155	-0.20247	8	1.02E+08	R	II		IGR	opensea	IGR-opense		rs1467471	21;2
cg0951117	0.218905	0.607824	9.351324	1.94E-05	0.001093	3.451772	0.498371	0.717276	-0.21891	1	2.48E+08	R	II	C1orf150	3'UTR	opensea	3'UTR-oper		rs1999612	32;9;2
cg0547735	0.233088	0.471118	9.349796	1.94E-05	0.001093	3.450576	0.354574	0.587662	-0.23309	3	1.32E+08	R	II	CPNE4	Body	opensea	Body-opens			
cg1070765	0.238905	0.50664	9.34932	1.94E-05	0.001093	3.450203	0.387188	0.626092	-0.2389	3	1.84E+08	F	II	LOC101928	TSS200	opensea	TSS200-ope		rs5774671	26
cg1983671	0.211977	0.52727	9.348736	1.94E-05	0.001093	3.449746	0.421281	0.633259	-0.21198	11	17464823	R	II	ABCC8	Body	opensea	Body-opens		rs2021837	18;17
cg1975239	0.24589	0.513851	9.347415	1.94E-05	0.001094	3.448711	0.390906	0.636796	-0.24589	2	1.09E+08	R	II	LIMS1	5'UTR	opensea	5'UTR-oper		rs7867094	32;14;2
cg0264397	0.241908	0.612984	9.346602	1.94E-05	0.001094	3.448075	0.49203	0.733938	-0.24191	17	68071130	R	II	KCNJ16	TSS1500	opensea	TSS1500-op		rs1145580	48;41;24;22;19;18
cg0558807	0.220834	0.438128	9.345458	1.95E-05	0.001095	3.447178	0.327711	0.548544	-0.22083	20	17486490	F	II	BFSP1	Body	opensea	Body-opens		rs5340175	10;41
cg2436060	0.205475	0.668403	9.344968	1.95E-05	0.001095	3.446794	0.565666	0.771141	-0.20548	17	8837741	R	II	PIK3R5	5'UTR	opensea	5'UTR-oper		rs1478987	48;43
cg0365104	0.253261	0.570554	9.344275	1.95E-05	0.001095	3.446251	0.443923	0.697185	-0.25326	14	55542565	R	II		IGR	opensea	IGR-opense			
cg2761332	0.212163	0.569583	9.343432	1.95E-05	0.001096	3.445591	0.463501	0.675664	-0.21216	13	42943425	R	II		IGR	opensea	IGR-opense		rs3690180	49;38;20;14;2
cg0111307	0.242574	0.57566	9.342287	1.95E-05	0.001096	3.444694	0.454373	0.696947	-0.24257	6	1.56E+08	F	II		IGR	opensea	IGR-opense		rs5275182	31;44
cg1639160	0.200121	0.685721	9.341758	1.95E-05	0.001096	3.444278	0.585661	0.785782	-0.20012	1	1.56E+08	R	II	SEMA4A	Body	opensea	Body-opens		rs5409797	50
cg2224368	0.214902	0.637167	9.34062	1.95E-05	0.001097	3.443387	0.529716	0.744617	-0.2149	17	41052543	R	II	G6PC	TSS1500	opensea	TSS1500-op		rs1396895	51;41;4
cg1985456	0.227363	0.677881	9.340311	1.95E-05	0.001097	3.443145	0.5642	0.791563	-0.22736	12	66989336	F	II	GRIP1	Body	opensea	Body-opens		rs5668670	32;46
cg2739491	0.232732	0.455475	9.339011	1.95E-05	0.001097	3.442125	0.339109	0.571841	-0.23273	16	23304561	R	II		IGR	opensea	IGR-opense			
cg2276423	0.218206	0.44979	9.336856	1.96E-05	0.001098	3.440435	0.340687	0.558893	-0.21821	2	17796973	R	II	VSNL1	Body	opensea	Body-opens			
cg2453022	0.203632	0.60258	9.336658	1.96E-05	0.001098	3.440281	0.500764	0.704396	-0.20363	2	1.03E+08	F	II	IL1R1	5'UTR	opensea	5'UTR-oper		rs1459900	1;8;13
cg0833873	0.233922	0.5091	9.336412	1.96E-05	0.001098	3.440087	0.39214	0.626061	-0.23392	4	1.13E+08	F	II		IGR	opensea	IGR-opense		rs1417029	31
cg1279363	0.200065	0.570199	9.334473	1.96E-05	0.001099	3.438566	0.470166	0.670231	-0.20006	6	1.35E+08	F	II	LOC101928	TSS1500	opensea	TSS1500-op		rs1879965	0;13;45
cg1650943	0.229158	0.518715	9.334287	1.96E-05	0.001099	3.438421	0.404136	0.633294	-0.22916	6	16699516	R	II	ATXN1	5'UTR	opensea	5'UTR-oper		rs7372521	43;3
cg1429397	0.209437	0.578626	9.333121	1.96E-05	0.0011	3.437506	0.473908	0.683345	-0.20944	5	90167401	R	II	ADGRV1	Body	opensea	Body-opens		rs5578921	30
cg0516130	0.207264	0.61494	9.331822	1.97E-05	0.0011	3.436487	0.511308	0.718572	-0.20726	10	16158824	R	II		IGR	opensea	IGR-opense		rs1138041	25;14
cg1991750	0.232458	0.521645	9.33177	1.97E-05	0.0011	3.436446	0.405416	0.637874	-0.23246	18	56208955	R	II	ALPK2	Body	opensea	Body-opens		rs5411683	51;30;11
cg2452184	0.223398	0.547164	9.331281	1.97E-05	0.0011	3.436062	0.435465	0.658863	-0.2234	4	1.03E+08	R	II	BANK1	Body	opensea	Body-opens		rs1864329	18;12;2
cg2235498	0.244459	0.455413	9.330889	1.97E-05	0.0011	3.435755	0.333184	0.577643	-0.24446	21	33344786	R	II	HUNK	Body	opensea	Body-opens			
cg1743935	0.201471	0.702414	9.329585	1.97E-05	0.001101	3.434731	0.601679	0.80315	-0.20147	21	45653242	R	II	ICOSLG	Body	opensea	Body-opens		rs5542423	2
cg2172956	0.207356	0.420039	9.329543	1.97E-05	0.001101	3.434699	0.31636	0.523717	-0.20736	16	80661293	R	II	CDYL2	Body	opensea	Body-opens		rs5573937	32
cg2042310	0.209004	0.607205	9.327416	1.97E-05	0.001102	3.433029	0.502703	0.711708	-0.209	6	28919021	F	II		IGR	opensea	IGR-opense		rs1164161	15
cg0044976	0.253804	0.488789	9.32608	1.97E-05	0.001102	3.43198	0.361887	0.61569	-0.2538	12	31559410	F	II	DENND5B	Body	opensea	Body-opens			
cg0259391	0.200046	0.536335	9.324266	1.98E-05	0.001102	3.430557	0.436312	0.636359	-0.20005	1	22164880	R	II	HSPG2	Body	opensea	Body-opens		rs7980394	43
cg2269665	0.207402	0.667914	9.322221	1.98E-05	0.001103	3.42895	0.564214	0.771615	-0.2074	1	64255970	R	II	ROR1	Body	opensea	Body-opens		rs5680831	36;29;14
cg2445215	0.261478	0.51216	9.32109	1.98E-05	0.001104	3.428062	0.381421	0.642899	-0.26148	2	12433867	R	II	LOC100506	Body	opensea	Body-opens		rs5563541	1
cg1638160	0.208415	0.490264	9.319937	1.98E-05	0.001104	3.427156	0.386057	0.594472	-0.20841	11	70002745	F	II	ANO1	Body	shore	Body-shore	chr11:7000		
cg1717817	0.20951	0.44503	9.319897	1.98E-05	0.001104	3.427125	0.340275	0.549785	-0.20951	1	1.84E+08	F	II		IGR	opensea	IGR-opense		rs1395128	29
cg1981114	0.202226	0.544225	9.319794	1.98E-05	0.001104	3.427044	0.443112	0.645338	-0.20223	18	46286151	R	II	KIAA0427	Body	shore	Body-shore	chr18:4628	rs5485708	39;1
cg2431204	0.222026	0.526609	9.318052	1.99E-05	0.001104	3.425675	0.415596	0.637622	-0.22203	1	22672625	R	II		IGR	shelf	IGR-shelf	chr1:2266	rs5511470	29;25;2;1
cg0636735	0.217009	0.617905	9.317834	1.99E-05	0.001104	3.425504	0.5094	0.72641	-0.21701	3	8363442	F	II		IGR	opensea	IGR-opense		rs5419719	29;46
cg1397632	0.217854	0.670571	9.316704	1.99E-05	0.001105	3.424616	0.561644	0.779498	-0.21785	10	34604934	R	II	PARD3	Body	opensea	Body-opens		rs1100973	41;30;26
cg0724544	0.209121	0.678637	9.316524	1.99E-05	0.001105	3.424475	0.574077	0.783198	-0.20912	1	1.56E+08	F	II	PMF1	Body	shore	Body-shore	chr1:1561		
cg1373383	0.238915	0.63147	9.315739	1.99E-05	0.001105	3.423857	0.512013	0.750928	-0.23891	10	9059161	F	II		IGR	opensea	IGR-opense		rs5575364	0
cg2205860	0.217546	0.610808	9.31555	1.99E-05	0.001105	3.42371	0.502035	0.719581	-0.21755	13	43935238	R	II	ENOX1	Body	opensea	Body-opens		rs5768211	43
cg0036892	0.210749	0.552732	9.315399	1.99E-05	0.001105	3.423591	0.447358	0.658106	-0.21075	9	1.02E+08	F	II	GALNT12	Body	shelf	Body-shelf	chr9:1015	rs5617446	1;27;40
cg1420807	0.228026	0.464066	9.315229	1.99E-05	0.001105	3.423457	0.350053	0.578079	-0.22803	2	81724589	R	II		IGR	opensea	IGR-opense		rs1895720	2
cg2738266	0.217414	0.618501	9.314701	1.99E-05	0.001105	3.423042	0.509794	0.727208	-0.21741	4	1.2E+08	F	II	MYOZ2	TSS1500	opensea	TSS1500-op		rs1163808	8
cg1266330	0.221735	0.460361	9.313993	1.99E-05	0.001106	3.422485	0.349493	0.571228	-0.22174	6	1.14E+08	R	II		IGR	opensea	IGR-opense		rs5779602	42;14;4;2;1
cg2655769	0.244231	0.498658	9.313924	1.99E-05	0.001106	3.422431	0.376543	0.620774	-0.24423	1	2.21E+08	F	II		IGR	opensea	IGR-opense		rs5310098	0;38
cg2452416	0.205449	0.404716	9.313475	1.99E-05	0.001106	3.422078	0.301992	0.507441	-0.20545	2	2.04E+08	R	II		IGR	opensea	IGR-opense		rs5756511	1
cg0665056	0.235265	0.541911	9.313435	1.99E-05	0.001106	3.422047	0.424279	0.659543	-0.23526	2	1.66E+08	F	II	SLC38A11	Body	opensea	Body-opens		rs7548124	12;26
cg1861368	0.21456	0.626058	9.313275	1.99E-05	0.001106	3.421921	0.518778	0.733338	-0.21456	19	5282167	F	II	PTPRS	Body	opensea	Body-opens		rs3756982	1;29;30;49
cg0719208	0.240302	0.494033	9.31258	1.99E-05	0.001106	3.421375	0.373882	0.614184	-0.2403	14	75981936	F	II		IGR	opensea	IGR-opense			
cg0698545	0.208178	0.541448	9.311343	2E-05	0.001107	3.420402	0.437359	0.645537	-0.20818	14	95653488	R	II	CLMN	3'UTR	opensea	3'UTR-oper		rs5291332	2;1
cg0323704	0.217937	0.65518	9.310946	2E-05	0.001107	3.42009	0.546212	0.764149	-0.21794	5	77175909	R	II		IGR	opensea	IGR-opense		rs1821166	35;34;1
cg0372915	0.204368	0.525918	9.309976	2E-05	0.001107	3.419327	0.423734	0.628102	-0.20437	7	19961027	F	II		IGR	opensea	IGR-opense		rs5542116	0;3;27;51
cg1275694	0.218146	0.577927	9.307114	2E-05	0.001109	3.417076	0.468854	0.687	-0.21815	7	1.57E+08	F	II	PTPRN2	Body	shore	Body-shore	chr7:1574	rs1812796	1;19;21
cg1719897	0.216212	0.587384	9.306166	2E-05	0.001109	3.41633	0.479278	0.69549	-0.21621	16	72277225	R	II		IGR	opensea	IGR-opense		rs5449548	28;27;11;6
cg2094485	0.239962	0.559554	9.305919	2E-05	0.001109	3.416136	0.439573	0.679535	-0.23996	12	81172316	R	II		IGR	opensea	IGR-opense		rs1811075	23;1
cg2300002	0.230938	0.508383	9.305308	2.01E-05	0.001109	3.415655	0.392914	0.623852	-0.23094	12	93707765	R	II	LOC64333	Body	opensea	Body-opens		rs5449117	45;2
cg0282572	0.213259	0.561174	9.304396	2.01E-05	0.00111	3.414938	0.454544	0.667803	-0.21326	21	31540204	F	II	CLDN17	TSS1500	opensea	TSS1500-op		rs5428913	3;8;26;34
cg2689516	0.215513	0.600803	9.299101	2.01E-05	0.001111	3.41077	0.493047	0.70856	-0.21551	14	91010168	F	II	TTC7B	Body	shelf	Body-shelf	chr14:910		
cg1981830	0.208684	0.490458	9.298724	2.02E-05	0.001111	3.410474	0.386116	0.5948	-0.20868	14	75946745	R	II		IGR	opensea	IGR-opense		rs1425667	18
cg0926467	0.204735	0.512418	9.298691	2.02E-05	0.001111	3.410448	0.410051	0.614786	-0.20474	16	20684636	F	II	ACSM1	Body	opensea	Body-opens		rs5551033	20;37;46
cg0159768	0.214514	0.629245	9.298614	2.02E-05	0.001111	3.410387	0.521988	0.736502	-0.21451	10	54739651	F	II		IGR	opensea	IGR-opense		rs1849449	1;8;18;29;33;48
cg2327498	0.21225	0.6219	9.298373	2.02E-05	0.001112	3.410197	0.515774	0.728025	-0.21225	2	1.3E+08	F	II		IGR	opensea	IGR-opense		rs5621905	1;40;51
cg0394712	0.225435	0.538335	9.294557	2.02E-05	0.001113	3.407192	0.425617	0.651052	-0.22544	6	22926357	R	II		IGR	opensea	IGR-opense			
cg2588287	0.21057	0.698301	9.294106	2.02E-05	0.001113	3.406836	0.593016	0.803586	-0.21057	22	17701547	R	II	CECR1	TSS1500	opensea	TSS1500-op		rs1896588	45;19
cg1159060	0.216986	0.477404	9.291091	2.03E-05	0.001115	3.404461	0.368911	0.585897	-0.21699	8	52169322	R	II		IGR	opensea	IGR-opense		rs7653671	23;5
cg1634204	0.242599	0.565469	9.290633	2.03E-05	0.001115	3.4041	0.444169	0.686768	-0.2426	1	1.99E+08	R	II		IGR	opensea	IGR-opense		rs3032430	43;37;34;33;22;1
cg1319523	0.22529	0.693528	9.290587	2.03E-05	0.001115	3.404065	0.580883	0.806173	-0.22529	7	1.38E+08	F	II	SVOPL	Body	opensea	Body-opens		rs1426466	10;25
cg1597187	0.239477	0.426607	9.290448	2.03E-05	0.001115	3.403955	0.306868	0.546345	-0.23948	2	2.27E+08	R	II		IGR	opensea	IGR-opense		rs1380049	20;2;1
cg0850513	0.21881	0.572182	9.290239	2.03E-05	0.001115	3.40379	0.462777	0.681587	-0.21881	1	2.05E+08	F	II	LEMD1	1stExon	opensea	1stExon-op			
cg0941529	0.235217	0.576687	9.289626	2.03E-05	0.001116	3.403307	0.459078	0.694296	-0.23522	14	99309749	R	II		IGR	opensea	IGR-opense			
cg1357321	0.202942	0.497162	9.289514	2.03E-05	0.001116	3.403219	0.395691	0.598633	-0.20294	9	1.26E+08	R	II	CRB2	Body	shore	Body-shore	chr9:1261	rs1453242	15;2
cg1316349	0.257935	0.642702	9.289058	2.03E-05	0.001116	3.402859	0.513734	0.771669	-0.25793	1	2.16E+08	R	II	KCTD3	Body	opensea	Body-opens		rs5289969	10;1
cg0262137	0.213594	0.549312	9.288773	2.03E-05	0.001116	3.402635	0.442515	0.656109	-0.21359	1	85461303	R	II	MCOLN2	Body	shore	Body-shore	chr1:8546	rs2389792	49;35
cg0886514	0.203651	0.592571	9.288561	2.03E-05	0.001116	3.402467	0.490745	0.694396	-0.20365	10	71507307	R	II		IGR	opensea	IGR-opense		rs1433531	33;2
cg0274778	0.217025	0.689866	9.288425	2.03E-05	0.001116	3.40236	0.581353	0.798378	-0.21702	5	32531247	F	II		IGR	opensea	IGR-opense		rs1503571	0;5;36
cg0680570	0.213721	0.543374	9.288235	2.03E-05	0.001116	3.402211	0.436513	0.650234	-0.21372	16	81160871	F	II	PKD1L2	Body	opensea	Body-opens		rs3408037	11
cg0871926	0.251697	0.491203	9.285738	2.04E-05	0.001117	3.400242	0.365354	0.617051	-0.2517	4	1.29E+08	R	II	INTU	Body	opensea	Body-opens		rs5681101	1
cg0116279	0.238935	0.584248	9.28412	2.04E-05	0.001118	3.398966	0.464781	0.703716	-0.23893	1	64293256	R	II	ROR1	Body	opensea	Body-opens		rs5512446	27
cg0569049	0.217745	0.595421	9.283779	2.04E-05	0.001118	3.398698	0.486548	0.704294	-0.21775	1	2.25E+08	F	II	CNIH3	Body	opensea	Body-opens		rs5573136	1;38;41;42
cg1813354	0.224387	0.541904	9.282718	2.04E-05	0.001118	3.397861	0.429711	0.654098	-0.22439	14	33682124	F	II	NPAS3	Body	opensea	Body-opens		rs5528915	16;24;29
cg0468019	0.247364	0.605602	9.281598	2.04E-05	0.001118	3.396978	0.48192	0.729284	-0.24736	9	1.17E+08	F	II		IGR	opensea	IGR-opense			
cg0116536	0.227174	0.474445	9.280941	2.04E-05	0.001119	3.39646	0.360858	0.588032	-0.22717	1	2.12E+08	F	II	LPGAT1	Body	opensea	Body-opens		rs5356322	26
cg1957609	0.208352	0.427435	9.279164	2.05E-05	0.001119	3.395058	0.323259	0.531611	-0.20835	6	1.07E+08	F	II		IGR	shelf	IGR-shelf	chr6:1065	rs1427808	20;33
cg0456022	0.214452	0.533746	9.27889	2.05E-05	0.00112	3.394841	0.42652	0.640972	-0.21445	20	50098437	R	II	NFATC2	Body	opensea	Body-opens			
cg2073684	0.2602	0.471003	9.278714	2.05E-05	0.00112	3.394702	0.340903	0.601103	-0.2602	2	73902031	F	II	ALMS1P	ExonBnd	opensea	ExonBnd-op		rs2010199	0;1
cg1355150	0.200882	0.606124	9.27732	2.05E-05	0.00112	3.393602	0.505683	0.706565	-0.20088	12	2014983	R	II	CACNA2D4	Body	shelf	Body-shelf	chr12:201	rs1489500	17;2
cg0428642	0.200863	0.597923	9.277242	2.05E-05	0.00112	3.393541	0.497492	0.698354	-0.20086	22	28513008	F	II	TTC28	Body	opensea	Body-opens		rs5581824	1;33
cg1614453	0.207931	0.5405	9.276238	2.05E-05	0.00112	3.392749	0.436535	0.644465	-0.20793	1	87677564	R	II	LOC10192	TSS1500	opensea	TSS1500-op			
cg0895992	0.219039	0.460464	9.274939	2.05E-05	0.001121	3.391723	0.350945	0.569984	-0.21904	10	92806758	F	II	LINC00502	ExonBnd	opensea	ExonBnd-op		rs5273723	1
cg1428599	0.214236	0.590283	9.274327	2.05E-05	0.001121	3.39124	0.483165	0.6974	-0.21424	7	1.15E+08	R	II	LINC01393	Body	opensea	Body-opens		rs5479742	38;1
cg2689995	0.212912	0.46964	9.273886	2.05E-05	0.001121	3.390892	0.363184	0.576096	-0.21291	4	8649781	R	II		IGR	shelf	IGR-shelf	chr4:8645	rs5562054	42
cg1206217	0.223047	0.631697	9.271521	2.06E-05	0.001122	3.389025	0.520174	0.743221	-0.22305	6	56734221	F	II	DST	Body	opensea	Body-opens		rs1827318	1
cg0347861	0.226409	0.609366	9.269673	2.06E-05	0.001123	3.387566	0.496161	0.72257	-0.22641	3	1.36E+08	R	II	PPP2R3A	5'UTR	opensea	5'UTR-oper		rs1141954	33;1
cg0104830	0.22027	0.5806	9.264774	2.07E-05	0.001126	3.383696	0.470465	0.690734	-0.22027	8	17762019	F	II		IGR	opensea	IGR-opense		rs5581583	35;48
cg1223222	0.268013	0.571534	9.263862	2.07E-05	0.001126	3.382975	0.437528	0.705541	-0.26801	13	98825268	F	II	FARP1	5'UTR	shelf	5'UTR-shelf	chr13:988	rs7410869	0;1;2;51
cg0320317	0.224054	0.5593	9.261651	2.07E-05	0.001127	3.381228	0.447273	0.671327	-0.22405	1	92275181	R	II	TGFBR3	Body	opensea	Body-opens		rs1788198	31;23
cg1593312	0.237951	0.482532	9.261628	2.07E-05	0.001127	3.38121	0.363557	0.601508	-0.23795	14	1.02E+08	R	II		IGR	shore	IGR-shore	chr14:102	rs4906136	13;2
cg1366458	0.230078	0.476911	9.260602	2.07E-05	0.001127	3.380399	0.361872	0.59195	-0.23008	17	74557494	F	II	SNORD1A	TSS1500	shelf	TSS1500-sh	chr17:745		
cg1278796	0.217143	0.487887	9.260309	2.08E-05	0.001127	3.380167	0.379316	0.596458	-0.21714	4	1.5E+08	R	II		IGR	opensea	IGR-opense		rs7295998	2
cg0503477	0.218771	0.549451	9.259192	2.08E-05	0.001128	3.379284	0.440065	0.658836	-0.21877	1	2.1E+08	R	II		IGR	opensea	IGR-opense		rs5710818	32;17;13;1
cg0829168	0.224688	0.483085	9.257777	2.08E-05	0.001129	3.378166	0.370741	0.595429	-0.22469	22	49749753	F	II		IGR	opensea	IGR-opense		rs5325898	37
cg1071826	0.201176	0.585953	9.257648	2.08E-05	0.001129	3.378063	0.485365	0.686541	-0.20118	2	9783004	F	II		IGR	opensea	IGR-opense		rs1922104	1;18
cg0595445	0.203289	0.592108	9.256129	2.08E-05	0.001129	3.376862	0.490464	0.693753	-0.20329	14	52064259	F	II	FRMD6-AS2	Body	opensea	Body-opens		rs5430772	0;1
cg0748235	0.232578	0.601986	9.255404	2.08E-05	0.00113	3.376288	0.485697	0.718275	-0.23258	9	1982266	F	II		IGR	opensea	IGR-opense		rs5697162	0;1;6;20;27;29
cg1937112	0.210894	0.60642	9.255266	2.08E-05	0.00113	3.37618	0.500973	0.711867	-0.21089	1	1.75E+08	R	II	TNN	Body	shore	Body-shore	chr1:1750	rs5510594	48;2
cg1437750	0.206183	0.51481	9.252513	2.09E-05	0.001131	3.374002	0.411719	0.617902	-0.20618	11	26744743	R	II	SLC5A12	TSS1500	opensea	TSS1500-op		rs5470070	49
cg2264678	0.243171	0.60454	9.250395	2.09E-05	0.001132	3.372326	0.482955	0.726125	-0.24317	6	1.45E+08	R	II	UTRN	Body	opensea	Body-opens		rs5298981	44
cg0189525	0.222009	0.613901	9.248172	2.09E-05	0.001132	3.370567	0.502896	0.724905	-0.22201	7	55133046	R	II	EGFR	Body	opensea	Body-opens		rs5309502	35;18
cg1980503	0.210968	0.682518	9.246964	2.1E-05	0.001133	3.369611	0.577034	0.788002	-0.21097	10	13766768	R	II	FRMD4A	Body	opensea	Body-opens		rs1814195	32
cg2482679	0.219679	0.626218	9.246039	2.1E-05	0.001133	3.368878	0.516379	0.736058	-0.21968	2	65825029	R	II		IGR	opensea	IGR-opense		rs5326276	19;13;10
cg0159414	0.205525	0.663434	9.244017	2.1E-05	0.001134	3.367277	0.560671	0.766196	-0.20553	2	47414734	R	II		IGR	opensea	IGR-opense		rs1841327	2;1
cg2330235	0.203599	0.57804	9.24165	2.1E-05	0.001135	3.365403	0.476241	0.67984	-0.2036	17	27372891	R	II	PIPOX	Body	opensea	Body-opens		rs7597223	42;41;4;2

cg2027496	0.209015	0.697821	9.240982	2.11E-05	0.001135	3.364873	0.593313	0.802328	-0.20901	18	59989045	F	II		IGR	shelf	IGR-shelf	chr18:5999	rs5410808	0;20;26;38
cg2056150	0.211855	0.710374	9.240654	2.11E-05	0.001135	3.364614	0.604446	0.816301	-0.21186	14	79715920	R	II	NRXN3	Body	opensea	Body-opens		rs1449897	7;2
cg0844271	0.20673	0.518885	9.240457	2.11E-05	0.001135	3.364457	0.41552	0.62225	-0.20673	5	1.18E+08	R	II		IGR	opensea	IGR-opense		rs5314279	23;19
cg1330871	0.24857	0.443618	9.240279	2.11E-05	0.001135	3.364317	0.319333	0.567903	-0.24857	7	70065077	F	II	AUTS2	Body	shelf	Body-shelf	chr7:7006(	rs1845834	0;3;51
cg2282215	0.215622	0.635723	9.239472	2.11E-05	0.001136	3.363677	0.527912	0.743534	-0.21562	6	64486333	R	II	EYS	Body	opensea	Body-opens		rs7578366	10
cg2134803	0.250768	0.563127	9.238498	2.11E-05	0.001136	3.362905	0.437743	0.688511	-0.25077	5	1.29E+08	F	II	ADAMTS19	Body	opensea	Body-opens		rs1494714	0;21
cg1332043	0.201487	0.548502	9.237476	2.11E-05	0.001137	3.362095	0.447759	0.649246	-0.20149	8	55138014	F	II		IGR	opensea	IGR-opense		rs7359489	40
cg0068613	0.202063	0.625296	9.236978	2.11E-05	0.001137	3.361701	0.524265	0.726327	-0.20206	6	1.68E+08	F	II	MLLT4	Body	opensea	Body-opens			
cg0753924	0.20252	0.726864	9.236637	2.11E-05	0.001137	3.361431	0.625604	0.828125	-0.20252	7	93919388	F	II		IGR	opensea	IGR-opense		rs5531540	7;9;16;36
cg2636216	0.204014	0.6111	9.235051	2.12E-05	0.001138	3.360174	0.509093	0.713106	-0.20401	1	2.31E+08	R	II		IGR	opensea	IGR-opense		rs5498040	2
cg2727324	0.236686	0.518327	9.234654	2.12E-05	0.001138	3.35986	0.399985	0.63667	-0.23669	6	3802297	F	II		IGR	opensea	IGR-opense		rs5780338	1
cg0372532	0.254095	0.416392	9.23404	2.12E-05	0.001138	3.359373	0.289344	0.543439	-0.2541	20	50015815	F	II	NFATC2	Body	opensea	Body-opens		rs7545307	0;11;49
cg2645014	0.213366	0.680199	9.232689	2.12E-05	0.001138	3.358301	0.573516	0.786882	-0.21337	7	47375903	F	II	TNS3	Body	opensea	Body-opens			
cg1802326	0.22984	0.545422	9.232447	2.12E-05	0.001138	3.35811	0.430502	0.660342	-0.22984	11	84403466	R	II	DLG2	Body	opensea	Body-opens		rs5309154	2
cg1197032	0.222459	0.614141	9.232047	2.12E-05	0.001139	3.357793	0.502912	0.725371	-0.22246	7	40529988	R	II	SUGCT	Body	opensea	Body-opens		rs1474069	2
cg0411214	0.201045	0.562806	9.232036	2.12E-05	0.001139	3.357784	0.462284	0.663328	-0.20104	2	2E+08	F	II		IGR	opensea	IGR-opense		rs1840751	9
cg1694025	0.222424	0.577489	9.231875	2.12E-05	0.001139	3.357656	0.466277	0.688701	-0.22242	1	2.36E+08	F	II	GPR137B	Body	opensea	Body-opens		rs5433993	0;1;9
cg2675391	0.230893	0.516634	9.230681	2.12E-05	0.001139	3.356709	0.401188	0.632081	-0.23089	14	24540526	R	II	CPNE6	TSS200	opensea	TSS200-ope			
cg1353075	0.245838	0.650778	9.230331	2.12E-05	0.001139	3.356432	0.527859	0.773697	-0.24584	20	20406628	F	II	RALGAPA2	Body	opensea	Body-opens		rs1405844	15;19
cg0337754	0.231706	0.5664	9.22872	2.13E-05	0.00114	3.355154	0.450546	0.682253	-0.23171	6	47170067	F	II		IGR	opensea	IGR-opense		rs5530303	0;33;43;47
cg1196211	0.225313	0.639992	9.228082	2.13E-05	0.00114	3.354648	0.527335	0.752648	-0.22531	3	1.53E+08	F	II		IGR	opensea	IGR-opense		rs5704644	1;2;35
cg2184301	0.200121	0.507827	9.226984	2.13E-05	0.00114	3.353777	0.407766	0.607887	-0.20012	1	1.53E+08	R	II	PGLYRP3	TSS1500	opensea	TSS1500-op		rs2987761	46;37;24
cg0532484	0.206371	0.623396	9.226619	2.13E-05	0.00114	3.353488	0.52021	0.726581	-0.20637	13	97550987	F	II		IGR	opensea	IGR-opense		rs1921852	1;43
cg1015845	0.233293	0.610403	9.226112	2.13E-05	0.001141	3.353086	0.493756	0.727049	-0.23329	1	93937840	R	II	FNBP1L	Body	opensea	Body-opens		rs1880176	16;2
cg0506431	0.220903	0.531175	9.225958	2.13E-05	0.001141	3.352963	0.420723	0.641626	-0.2209	5	1.33E+08	F	II	FSTL4	Body	opensea	Body-opens		rs5451529	0;3;10;28;44
cg1458062	0.207832	0.504384	9.225521	2.13E-05	0.001141	3.352616	0.400468	0.608301	-0.20783	5	43602116	R	II	NNT	TSS1500	shore	TSS1500-sh	chr5:4360	rs5735963	49;16
cg0888230	0.21307	0.467711	9.223561	2.13E-05	0.001142	3.351061	0.361176	0.574246	-0.21307	19	29489929	R	II		IGR	opensea	IGR-opense		rs1404326	46;24;2
cg2680858	0.214829	0.60076	9.222684	2.14E-05	0.001142	3.350365	0.493345	0.708174	-0.21483	1	1.53E+08	F	II	S100A12	Body	opensea	Body-opens		rs2233864	0;1
cg2688131	0.227726	0.556525	9.222562	2.14E-05	0.001142	3.350269	0.442662	0.670388	-0.22773	13	95719669	F	II	ABCC4	Body	opensea	Body-opens		rs1162551	1
cg1107098	0.216685	0.401932	9.220522	2.14E-05	0.001142	3.348649	0.293589	0.510275	-0.21669	6	1.14E+08	F	II		IGR	opensea	IGR-opense		rs5780015	0;1;30;32
cg0226149	0.208934	0.570037	9.220337	2.14E-05	0.001142	3.348503	0.46557	0.674505	-0.20893	10	80192517	R	II		IGR	opensea	IGR-opense		rs1505463	39
cg0075643	0.235865	0.541527	9.218083	2.14E-05	0.001143	3.346713	0.423594	0.65946	-0.23587	5	1.69E+08	F	II		IGR	opensea	IGR-opense		rs1842809	49
cg0941859	0.21597	0.48082	9.217407	2.14E-05	0.001143	3.346176	0.372835	0.588805	-0.21597	7	81112712	F	II		IGR	opensea	IGR-opense		rs5485935	4
cg2057825	0.251416	0.561408	9.216602	2.15E-05	0.001143	3.345537	0.4357	0.687116	-0.25142	10	97732488	F	II		IGR	opensea	IGR-opense		rs5757202	34
cg0053320	0.230441	0.475462	9.215195	2.15E-05	0.001143	3.344419	0.360241	0.590682	-0.23044	9	1E+08	F	II	LOC10049	Body	opensea	Body-opens		rs5578909	10
cg0750555	0.243888	0.655021	9.214601	2.15E-05	0.001144	3.343947	0.533077	0.776965	-0.24389	5	36648568	R	II	SLC1A3	Body	opensea	Body-opens		rs5560676	48;24;7
cg1539212	0.206713	0.633897	9.213923	2.15E-05	0.001144	3.343409	0.530541	0.737254	-0.20671	5	1.24E+08	F	II		IGR	opensea	IGR-opense		rs5708426	9
cg1398805	0.257382	0.60935	9.213089	2.15E-05	0.001144	3.342746	0.480658	0.738041	-0.25738	7	83792237	F	II	SEMA3A	Body	opensea	Body-opens		rs5497181	21;40
cg1085597	0.250195	0.526857	9.21077	2.16E-05	0.001144	3.340904	0.40176	0.651955	-0.2502	8	23780241	F	II		IGR	opensea	IGR-opense		rs5753882	1
cg2277903	0.212162	0.573773	9.210567	2.16E-05	0.001144	3.340743	0.467692	0.679854	-0.21216	4	52921007	F	II	SPATA18	Body	shelf	Body-shelf	chr4:5291		
cg0251785	0.200513	0.425661	9.210352	2.16E-05	0.001144	3.340572	0.325405	0.525918	-0.20051	3	1.71E+08	F	II		IGR	opensea	IGR-opense		rs3724153	0;2;10;17;37;49
cg0170680	0.226801	0.511922	9.210045	2.16E-05	0.001144	3.340328	0.398522	0.625323	-0.2268	4	1.46E+08	R	II		IGR	opensea	IGR-opense		rs1811951	1
cg1360235	0.258245	0.645051	9.209968	2.16E-05	0.001144	3.340267	0.515928	0.774173	-0.25825	3	14885560	F	II	FGD5	Body	opensea	Body-opens		rs5469159	33
cg0832971	0.220276	0.441855	9.209509	2.16E-05	0.001145	3.339902	0.331717	0.551993	-0.22028	1	15114228	F	II	KAZN	Body	opensea	Body-opens		rs1885493	42
cg2053628	0.231898	0.642785	9.209235	2.16E-05	0.001145	3.339684	0.526836	0.758735	-0.2319	4	1.48E+08	F	II		IGR	opensea	IGR-opense		rs1438863	0;24
cg0846176	0.24453	0.477385	9.206133	2.16E-05	0.001146	3.337218	0.35512	0.59965	-0.24453	16	80212320	F	II	LOC10272	Body	opensea	Body-opens		rs5603573	1;13;47
cg2155641	0.221805	0.684866	9.205973	2.16E-05	0.001146	3.337091	0.573964	0.795769	-0.2218	18	53399036	F	II		IGR	opensea	IGR-opense		rs1868557	0;1;11
cg1437288	0.209905	0.521258	9.203564	2.17E-05	0.001147	3.335175	0.416306	0.626211	-0.20991	3	1.51E+08	F	II		IGR	opensea	IGR-opense		rs5496071	1;23
cg2089999	0.200726	0.421091	9.203506	2.17E-05	0.001147	3.335129	0.320728	0.521454	-0.20073	12	33023315	F	II	PKP2	Body	opensea	Body-opens			
cg0084800	0.210535	0.619991	9.201138	2.17E-05	0.001148	3.333246	0.514723	0.725258	-0.21053	12	68553396	F	II	IFNG	1stExon	opensea	1stExon-op		rs1814075	4;26;37
cg2651127	0.22102	0.494885	9.200975	2.17E-05	0.001148	3.333117	0.384375	0.605394	-0.22102	4	1.7E+08	F	II		IGR	opensea	IGR-opense		rs4443239	19
cg0836117	0.227085	0.334233	9.19939	2.17E-05	0.001148	3.331855	0.22069	0.447776	-0.22709	9	90079331	R	II		IGR	opensea	IGR-opense		rs1175023	22;18;2
cg1717095	0.215659	0.59326	9.198634	2.18E-05	0.001149	3.331254	0.48543	0.70109	-0.21566	8	99056508	F	II	RPL30	Body	shore	Body-shore	chr8:9905	rs1867525	36;44
cg1775378	0.242499	0.589247	9.1981	2.18E-05	0.001149	3.330829	0.467998	0.710496	-0.2425	10	81026766	F	II	ZMIZ1	Body	opensea	Body-opens		rs5323573	1;14;47
cg0938982	0.221711	0.455537	9.197773	2.18E-05	0.001149	3.330569	0.344682	0.566393	-0.22171	10	31883099	F	II		IGR	opensea	IGR-opense		rs8010784	35
cg1115420	0.235103	0.606188	9.197579	2.18E-05	0.001149	3.330414	0.488636	0.723739	-0.2351	12	5757554	R	II	ANO2	Body	opensea	Body-opens		rs1148484	1
cg1860235	0.209477	0.441858	9.197262	2.18E-05	0.001149	3.330162	0.33712	0.546597	-0.20948	10	3269930	R	II		IGR	opensea	IGR-opense		rs1403675	47;38;8
cg1898921	0.207409	0.497191	9.197178	2.18E-05	0.001149	3.330095	0.393486	0.600895	-0.20741	13	73034831	R	II		IGR	opensea	IGR-opense		rs3706356	1
cg1371980	0.200538	0.676314	9.194398	2.18E-05	0.00115	3.327882	0.576045	0.776583	-0.20054	21	40168528	F	II		IGR	opensea	IGR-opense		rs5687085	6;34;39
cg0189707	0.214098	0.418729	9.193739	2.18E-05	0.00115	3.327358	0.31168	0.525778	-0.2141	13	1.07E+08	R	II	ARGLU1	Body	opensea	Body-opens		rs5706313	51;33;1
cg1462326	0.208672	0.637349	9.193102	2.18E-05	0.00115	3.326851	0.533013	0.741685	-0.20867	13	86374678	F	II	SLITRK6	TSS1500	opensea	TSS1500-op		rs5730180	41
cg0610234	0.206563	0.568478	9.190647	2.19E-05	0.001151	3.324896	0.465196	0.67176	-0.20656	6	8859229	F	II		IGR	opensea	IGR-opense		rs1886097	3;8;33;48
cg2260816	0.216149	0.573845	9.190335	2.19E-05	0.001152	3.324648	0.46577	0.68192	-0.21615	1	95352630	R	II	SLC44A3	Body	opensea	Body-opens		rs5689956	4;2
cg2671027	0.241774	0.495945	9.18883	2.19E-05	0.001152	3.323449	0.375057	0.616832	-0.24177	7	86374484	R	II	GRM3	5'UTR	opensea	5'UTR-oper			
cg2165775	0.228578	0.512507	9.188367	2.19E-05	0.001152	3.323081	0.398218	0.626796	-0.22858	11	11535011	R	II	GALNT18	Body	opensea	Body-opens		rs1883311	46;21;17;14;8;1
cg0937189	0.202035	0.682218	9.188096	2.19E-05	0.001152	3.322864	0.581201	0.783235	-0.20203	11	66788739	R	II	SYT12	TSS1500	shore	TSS1500-sh	chr11:667	rs5457259	2
cg1122457	0.235707	0.507225	9.187529	2.19E-05	0.001152	3.322413	0.389371	0.625078	-0.23571	10	34794060	R	II	PARD3	Body	opensea	Body-opens		rs773973;r	44;37
cg1541263	0.2218	0.644725	9.187345	2.19E-05	0.001152	3.322266	0.533825	0.755625	-0.2218	21	36422800	R	II	RUNX1	TSS1500	opensea	TSS1500-op		rs1160822	31;5;1
cg0222000	0.213213	0.697331	9.187124	2.19E-05	0.001152	3.32209	0.590725	0.803938	-0.21321	6	13192742	F	II	PHACTR1	Body	opensea	Body-opens		rs3864311	17;29
cg1518630	0.227631	0.550299	9.186895	2.19E-05	0.001152	3.321908	0.436484	0.664115	-0.22763	13	55110248	R	II		IGR	opensea	IGR-opense		rs5697292	42;31;26;1
cg1172943	0.238115	0.484022	9.18645	2.2E-05	0.001152	3.321554	0.364964	0.603079	-0.23811	2	2.09E+08	F	II	CRYGA	TSS200	opensea	TSS200-ope			
cg0109933	0.204758	0.396745	9.184559	2.2E-05	0.001153	3.320047	0.294366	0.499123	-0.20476	7	1.18E+08	F	II		IGR	opensea	IGR-opense		rs1731757	19;38;40;43
cg1023641	0.218912	0.594031	9.182244	2.2E-05	0.001154	3.318202	0.484575	0.703487	-0.21891	7	24789868	R	II	DFNA5	5'UTR	opensea	5'UTR-oper		rs1410942	31;24
cg2474346	0.21806	0.604214	9.182114	2.2E-05	0.001154	3.318098	0.495184	0.713244	-0.21806	13	65669420	R	II		IGR	opensea	IGR-opense		rs5744790	29
cg1802333	0.225227	0.532817	9.181703	2.2E-05	0.001155	3.31777	0.420203	0.64543	-0.22523	11	1.31E+08	R	II	C11orf44	TSS1500	opensea	TSS1500-op			
cg1008108	0.200089	0.674271	9.181071	2.2E-05	0.001155	3.317267	0.574226	0.774316	-0.20009	15	66417557	F	II	MEGF11	Body	opensea	Body-opens		rs1159554	0;50
cg1215317	0.245041	0.656004	9.180526	2.21E-05	0.001155	3.316832	0.533484	0.778525	-0.24504	4	40477836	F	II	RBM47	5'UTR	opensea	5'UTR-oper		rs5309442	41
cg2343906	0.215179	0.542957	9.179197	2.21E-05	0.001156	3.315773	0.435368	0.650547	-0.21518	12	20422450	F	II		IGR	opensea	IGR-opense		rs5687916	21
cg2497687	0.209206	0.440523	9.178695	2.21E-05	0.001156	3.315372	0.33592	0.545126	-0.20921	17	47792648	R	II	FAM117A	Body	opensea	Body-opens			
cg1974459	0.228879	0.52627	9.177925	2.21E-05	0.001156	3.314758	0.411831	0.64071	-0.22888	4	81226861	R	II		IGR	opensea	IGR-opense		rs1150348	33;30
cg2600187	0.231479	0.563173	9.177246	2.21E-05	0.001156	3.314217	0.447434	0.678913	-0.23148	13	60053878	F	II		IGR	opensea	IGR-opense		rs1506114	1;4;11;36
cg1731275	0.201952	0.393697	9.176502	2.21E-05	0.001156	3.313624	0.292721	0.494673	-0.20195	13	1.07E+08	F	II		IGR	opensea	IGR-opense			
cg0363145	0.217268	0.636158	9.176468	2.21E-05	0.001156	3.313597	0.527524	0.744792	-0.21727	10	20230737	F	II	PLXDC2	Body	opensea	Body-opens		rs5671935	18;51
cg2026014	0.216107	0.591246	9.175991	2.21E-05	0.001157	3.313216	0.483192	0.6993	-0.21611	17	18017367	R	II	MYO15A	5'UTR	opensea	5'UTR-oper		rs5745443	41
cg0253396	0.216328	0.585997	9.175308	2.21E-05	0.001157	3.312672	0.477833	0.694161	-0.21633	13	87781746	R	II		IGR	opensea	IGR-opense			
cg1269862	0.210643	0.629729	9.175271	2.21E-05	0.001157	3.312642	0.524408	0.735051	-0.21064	17	32581466	F	II	CCL2	TSS1500	opensea	TSS1500-op		rs1492104	1;43
cg1339900	0.20856	0.566836	9.175132	2.21E-05	0.001157	3.312531	0.462556	0.671115	-0.20856	10	4386826	F	II		IGR	opensea	IGR-opense			
cg2700016	0.210745	0.580163	9.174018	2.22E-05	0.001157	3.311642	0.474791	0.685536	-0.21074	5	91633920	F	II		IGR	opensea	IGR-opense		rs1505382	1
cg0738337	0.279952	0.463409	9.172074	2.22E-05	0.001158	3.310091	0.323433	0.603385	-0.27995	7	40766405	R	II	SUGCT	Body	opensea	Body-opens		rs1875599	1
cg1457836	0.205712	0.76232	9.1715	2.22E-05	0.001159	3.309633	0.659464	0.865176	-0.20571	17	42785078	F	II	DBF4B	TSS1500	shore	TSS1500-sh	chr17:427	rs1835658	1;7;39
cg1416167	0.225095	0.644447	9.168574	2.23E-05	0.00116	3.307298	0.5319	0.756995	-0.22509	1	32118219	F	II	COL16A1	3'UTR	opensea	3'UTR-oper		rs1813187	28;45
cg2224544	0.221584	0.626896	9.167868	2.23E-05	0.001161	3.306735	0.516104	0.737688	-0.22158	2	2.18E+08	F	II	DIRC3	Body	opensea	Body-opens		rs1164498	1;31
cg0979936	0.20739	0.683543	9.165826	2.23E-05	0.001162	3.305104	0.579848	0.787239	-0.20739	20	51268046	F	II		IGR	opensea	IGR-opense		rs5594264	0;36;39
cg0118670	0.203934	0.688738	9.165661	2.23E-05	0.001162	3.304973	0.586771	0.790705	-0.20393	6	1.58E+08	F	II		IGR	opensea	IGR-opense		rs1160596	1
cg1566195	0.22249	0.63639	9.165046	2.23E-05	0.001162	3.304481	0.525145	0.747635	-0.22249	15	82172767	R	II		IGR	opensea	IGR-opense		rs1160227	2;1
cg0683400	0.234328	0.569636	9.164242	2.23E-05	0.001163	3.30384	0.452472	0.686799	-0.23433	10	22499738	R	II		IGR	opensea	IGR-opense			
cg0405038	0.210128	0.656039	9.163433	2.23E-05	0.001163	3.303194	0.550974	0.761103	-0.21013	3	1.84E+08	F	II		IGR	opensea	IGR-opense		rs5755120	0
cg1703664	0.231913	0.497183	9.162868	2.24E-05	0.001163	3.302742	0.381226	0.613139	-0.23191	3	52723544	F	II	GNL3	Body	shelf	Body-shelf	chr3:5271	rs7610171	33
cg0562692	0.245028	0.507409	9.162008	2.24E-05	0.001163	3.302055	0.384895	0.629923	-0.24503	4	14362239	R	II		IGR	opensea	IGR-opense		rs5280285	41;30
cg1721053	0.247089	0.582158	9.16187	2.24E-05	0.001163	3.301945	0.458613	0.705702	-0.24709	3	1.83E+08	F	II	MCF2L2	Body	opensea	Body-opens		rs5675522	43
cg1007333	0.243666	0.44761	9.159045	2.24E-05	0.001164	3.299688	0.325777	0.569443	-0.24367	10	89521994	R	II	ATAD1	Body	opensea	Body-opens		rs5710980	2
cg2070796	0.238245	0.502044	9.159021	2.24E-05	0.001164	3.299669	0.382921	0.621166	-0.23825	12	1.31E+08	R	II	ADGRD1	Body	opensea	Body-opens		rs2021903	6;4
cg1830353	0.237929	0.574352	9.158954	2.24E-05	0.001164	3.299615	0.455388	0.693316	-0.23793	5	1.58E+08	F	II		IGR	opensea	IGR-opense		rs5765614	0;11;47;49
cg0359342	0.222492	0.636735	9.158556	2.24E-05	0.001164	3.299298	0.525489	0.747981	-0.22249	18	24337465	R	II		IGR	opensea	IGR-opense		rs527616;r	43;26;2
cg2084084	0.239512	0.547805	9.15843	2.24E-05	0.001164	3.299197	0.428049	0.66756	-0.23951	4	16025934	F	II	PROM1	ExonBnd	opensea	ExonBnd-op		rs1871160	0;1
cg0370108	0.229076	0.557349	9.158329	2.24E-05	0.001164	3.299117	0.442811	0.671886	-0.22908	8	52680434	R	II	PXDNL	Body	opensea	Body-opens			
cg2075878	0.217248	0.549286	9.158238	2.24E-05	0.001164	3.299044	0.440663	0.65791	-0.21725	15	82206091	F	II		IGR	opensea	IGR-opense		rs1840791	1
cg0801418	0.217631	0.469401	9.157821	2.24E-05	0.001165	3.29871	0.360586	0.578217	-0.21763	6	33241594	R	II	RPS18	Body	shore	Body-shore	chr6:3323	rs1178140	51;50;36
cg1420804	0.215488	0.585186	9.15751	2.25E-05	0.001165	3.298462	0.477443	0.69293	-0.21549	15	40080369	F	II		IGR	opensea	IGR-opense		rs5737836	0;4;39
cg1721097	0.206491	0.604441	9.157202	2.25E-05	0.001165	3.298216	0.501196	0.707686	-0.20649	21	31869664	F	II	KRTAP19-4	TSS1500	opensea	TSS1500-op		rs1880154	1;3;5;16
cg0573010	0.206506	0.530204	9.157159	2.25E-05	0.001165	3.298182	0.426951	0.633457	-0.20651	3	2933815	F	II	CNTN4	TSS200	opensea	TSS200-ope		rs5684547	0;35;37
cg2600384	0.243669	0.668742	9.156807	2.25E-05	0.001165	3.2979	0.546907	0.790576	-0.24367	5	78445588	R	II		IGR	opensea	IGR-opense			
cg2186208	0.227256	0.539462	9.15654	2.25E-05	0.001165	3.297687	0.425834	0.65309	-0.22726	14	77769687	R	II	POMT2	Body	opensea	Body-opens		rs8035559	29;9;1
cg1546622	0.239234	0.535077	9.155298	2.25E-05	0.001166	3.296694	0.41546	0.654694	-0.23923	7	1.52E+08	F	II	KMT2C	Body	opensea	Body-opens		rs1841775	39;50
cg1750959	0.225305	0.619438	9.155059	2.25E-05	0.001166	3.296503	0.506786	0.732091	-0.22531	1	1.02E+08	R	II		IGR	opensea	IGR-opense		rs4459133	38;31;1
cg0950702	0.210955	0.477176	9.153119	2.25E-05	0.001167	3.294952	0.371699	0.582654	-0.21095	13	93895610	R	II	GPC6	Body	opensea	Body-opens			
cg0246939	0.252089	0.599479	9.152889	2.25E-05	0.001167	3.294768	0.473435	0.725524	-0.25209	8	412261	R	II	FBXO25	Body	opensea	Body-opens		rs5674489	31
cg2670772	0.227013	0.558539	9.152411	2.25E-05	0.001167	3.294386	0.445032	0.672046	-0.22701	7	1.42E+08	F	II	CLEC5A	TSS1500	opensea	TSS1500-op		rs1381434	28;32
cg0457031	0.246057	0.670269	9.149646	2.26E-05	0.001169	3.292175	0.547241	0.793297	-0.24606	6	24776902	R	II	GMNN	5'UTR	shore	5'UTR-shore	chr6:2477		

cg2611323	0.202793	0.561694	9.149524	2.26E-05	0.001169	3.292078	0.460297	0.663091	-0.20279	5	1.75E+08	F	II		IGR	opensea	IGR-opense		rs5484021	0
cg1351365	0.217809	0.507922	9.149414	2.26E-05	0.001169	3.291989	0.399017	0.616827	-0.21781	1	48671325	R	II		IGR	opensea	IGR-opense		rs1486751	45;1
cg0626547	0.202017	0.646415	9.149273	2.26E-05	0.001169	3.291877	0.545406	0.747424	-0.20202	5	1.03E+08	R	II	LOC10246	Body	opensea	Body-opens		rs1119039	51;4
cg1550648	0.227679	0.570776	9.148973	2.26E-05	0.001169	3.291637	0.456937	0.684616	-0.22768	20	14782540	R	II	MACROD2	Body	opensea	Body-opens		rs5397076	32;11;1
cg0531854	0.226389	0.604897	9.148781	2.26E-05	0.001169	3.291483	0.491703	0.718092	-0.22639	2	2.22E+08	R	II		IGR	opensea	IGR-opense			
cg1648806	0.246623	0.532357	9.148128	2.26E-05	0.001169	3.290961	0.409046	0.655669	-0.24662	1	2.05E+08	R	II		IGR	shore	IGR-shore	chr1:2046	rs5549247	32;27
cg0250272	0.201905	0.560371	9.147851	2.26E-05	0.001169	3.290739	0.459418	0.661323	-0.20191	20	11780146	F	II		IGR	opensea	IGR-opense		rs1138817	25
cg1056898	0.205306	0.63267	9.147804	2.26E-05	0.001169	3.290701	0.530018	0.735323	-0.20531	15	80521890	F	II	LINC01314	Body	opensea	Body-opens		rs1487441	24;31
cg2736706	0.232388	0.511153	9.147108	2.26E-05	0.00117	3.290145	0.394959	0.627347	-0.23239	15	34662635	F	II		IGR	shelf	IGR-shelf	chr15:346	rs8042929	34
cg1185691	0.214817	0.613761	9.145823	2.27E-05	0.00117	3.289117	0.506352	0.721169	-0.21482	5	1.6E+08	F	II	FABP6	Body	opensea	Body-opens		rs1120587	0;12
cg1243958	0.207058	0.593361	9.145822	2.27E-05	0.00117	3.289116	0.489832	0.69689	-0.20706	2	2.17E+08	F	II	LINC00607	Body	opensea	Body-opens		rs5636087	1;3;24
cg0758636	0.243741	0.522387	9.145656	2.27E-05	0.00117	3.288983	0.400517	0.644257	-0.24374	17	54620995	R	II		IGR	opensea	IGR-opense		rs1493894	43;25;11
cg2738053	0.217128	0.57029	9.145491	2.27E-05	0.00117	3.288851	0.461725	0.678854	-0.21713	1	1.62E+08	R	II	NOS1AP	Body	opensea	Body-opens		rs1820874	42;33;2;1
cg0483769	0.211555	0.525756	9.145005	2.27E-05	0.001171	3.288462	0.419979	0.631533	-0.21155	6	35742999	F	II	CLPSL2	TSS1500	shore	TSS1500-sh	chr6:3574	rs1139058	0;41
cg0335173	0.23448	0.457381	9.144715	2.27E-05	0.001171	3.28823	0.340141	0.574621	-0.23448	18	21717196	F	II		IGR	shore	IGR-shore	chr18:217	rs7949387	24
cg0999803	0.207692	0.563226	9.144277	2.27E-05	0.001171	3.287879	0.45938	0.667072	-0.20769	10	75654563	F	II		IGR	opensea	IGR-opense		rs5530564	36;46
cg2324728	0.209961	0.547375	9.144215	2.27E-05	0.001171	3.28783	0.442394	0.652355	-0.20996	17	60937887	F	II	MIR548W	Body	opensea	Body-opens		rs1896087	23
cg1333212	0.207962	0.615588	9.143834	2.27E-05	0.001171	3.287525	0.511607	0.719568	-0.20796	5	1.57E+08	F	II	CYFIP2	Body	opensea	Body-opens		rs1882697	1
cg1337635	0.283798	0.48651	9.143093	2.27E-05	0.001171	3.286932	0.344611	0.628409	-0.2838	6	87892453	F	II	ZNF292	Body	opensea	Body-opens		rs1178603	7;21;32
cg0496491	0.210542	0.543515	9.142826	2.27E-05	0.001171	3.286718	0.438244	0.648786	-0.21054	2	1.59E+08	F	II	CCDC148	Body	opensea	Body-opens		rs5667910	0;1;45;48;49
cg0240039	0.207823	0.646319	9.142424	2.27E-05	0.001171	3.286396	0.542408	0.750231	-0.20782	10	23392945	R	II	MSRB2	Body	opensea	Body-opens		rs1426276	18;2
cg2091862	0.203456	0.455441	9.140759	2.27E-05	0.001172	3.285064	0.353712	0.557169	-0.20346	12	78800882	F	II		IGR	opensea	IGR-opense		rs1828029	1;41
cg0218959	0.217245	0.509986	9.140568	2.27E-05	0.001172	3.284911	0.401363	0.618609	-0.21725	15	60881071	F	II	RORA	Body	shelf	Body-shelf	chr15:608	rs5778999	5;41
cg0135229	0.205142	0.673638	9.139593	2.28E-05	0.001172	3.28413	0.571067	0.776208	-0.20514	6	1.1E+08	F	II	CCDC162	TSS1500	shelf	TSS1500-sh	chr6:1096	rs1833220	19;51
cg2568091	0.224511	0.589572	9.139183	2.28E-05	0.001172	3.283802	0.477317	0.701828	-0.22451	1	29288906	R	II	EPB41	5'UTR	opensea	5'UTR-oper		rs1511929	45;43;3
cg2102515	0.204981	0.705483	9.139173	2.28E-05	0.001172	3.283794	0.602992	0.807973	-0.20498	1	86901291	R	II	CLCA2	Body	opensea	Body-opens		rs5673549	1
cg1736307	0.204176	0.568177	9.139052	2.28E-05	0.001172	3.283697	0.466089	0.670265	-0.20418	5	37802433	R	II		IGR	opensea	IGR-opense			
cg0772723	0.204891	0.536636	9.13857	2.28E-05	0.001173	3.283311	0.434191	0.639081	-0.20489	13	46726752	F	II	LCP1	Body	opensea	Body-opens			
cg2524096	0.226607	0.534622	9.138083	2.28E-05	0.001173	3.282922	0.421318	0.647926	-0.22661	4	79339974	R	II	FRAS1	Body	opensea	Body-opens		rs1449887	48;45;26
cg1082900	0.214634	0.553322	9.137306	2.28E-05	0.001173	3.282299	0.446005	0.660639	-0.21463	14	60179825	F	II	RTN1	Body	opensea	Body-opens		rs5530072	29
cg2744212	0.206885	0.732768	9.136227	2.28E-05	0.001173	3.281435	0.629325	0.83621	-0.20689	14	93043537	F	II	RIN3	Body	opensea	Body-opens		rs5679090	38
cg0203202	0.203383	0.638888	9.135879	2.28E-05	0.001174	3.281157	0.537196	0.740579	-0.20338	6	1.32E+08	R	II	ENPP3	Body	opensea	Body-opens		rs1875462	43;2
cg0588739	0.221255	0.598113	9.133574	2.29E-05	0.001175	3.27931	0.487485	0.70874	-0.22126	3	58709278	F	II		IGR	opensea	IGR-opense		rs5555093	0;11
cg0073860	0.215881	0.532361	9.132348	2.29E-05	0.001175	3.278327	0.424421	0.640302	-0.21588	6	1.14E+08	R	II		IGR	opensea	IGR-opense		rs3768844	40;12;2
cg0322499	0.290116	0.441388	9.131841	2.29E-05	0.001176	3.277921	0.29633	0.586446	-0.29012	1	51750976	F	II		IGR	opensea	IGR-opense		rs5510240	11;47
cg2731544	0.210573	0.663226	9.131599	2.29E-05	0.001176	3.277728	0.55794	0.768513	-0.21057	7	79770081	F	II	GNAI1	5'UTR	opensea	5'UTR-oper		rs3769406	10
cg1384023	0.213195	0.569008	9.130939	2.29E-05	0.001176	3.277198	0.462411	0.675606	-0.21319	9	89218392	F	II		IGR	opensea	IGR-opense		rs5695931	19;48
cg2752207	0.222497	0.572663	9.130853	2.29E-05	0.001176	3.27713	0.461415	0.683912	-0.2225	1	22290005	R	II		IGR	opensea	IGR-opense		rs3747667	20
cg0787438	0.26488	0.558333	9.130715	2.29E-05	0.001176	3.277019	0.425893	0.690773	-0.26488	5	1.23E+08	F	II		IGR	opensea	IGR-opense		rs5399479	17
cg1028289	0.251514	0.616218	9.130105	2.29E-05	0.001176	3.27653	0.490461	0.741974	-0.25151	5	73611882	F	II		IGR	opensea	IGR-opense		rs5614390	46
cg2756978	0.249268	0.508056	9.129928	2.29E-05	0.001176	3.276388	0.383422	0.63269	-0.24927	6	27078427	R	II		IGR	opensea	IGR-opense		rs1394768	18
cg0839874	0.225054	0.52497	9.129465	2.29E-05	0.001176	3.276017	0.412443	0.637497	-0.22505	9	96819093	R	II	PTPDC1	5'UTR	opensea	5'UTR-oper		rs5487173	43;7;1
cg1528914	0.221216	0.456312	9.129263	2.29E-05	0.001176	3.275855	0.345704	0.56692	-0.22122	12	91311493	R	II	LINC00615	TSS1500	opensea	TSS1500-op		rs5279504	46;7
cg0301729	0.216723	0.553354	9.129193	2.29E-05	0.001176	3.275799	0.444992	0.661715	-0.21672	11	15886708	R	II		IGR	opensea	IGR-opense		rs1470609	1
cg1403332	0.205026	0.653346	9.127805	2.3E-05	0.001177	3.274687	0.550833	0.755859	-0.20503	12	1.16E+08	F	II		IGR	opensea	IGR-opense		rs1452936	1;39
cg2171391	0.234718	0.507745	9.12703	2.3E-05	0.001177	3.274065	0.390386	0.625103	-0.23472	5	1.04E+08	F	II		IGR	opensea	IGR-opense		rs1472804	35;42
cg1693605	0.221035	0.615408	9.126601	2.3E-05	0.001177	3.273721	0.504891	0.725925	-0.22103	11	1.26E+08	R	II		IGR	opensea	IGR-opense		rs5512982	49;46;29;21;10;2;1
cg1259425	0.281439	0.576177	9.12659	2.3E-05	0.001177	3.273712	0.435457	0.716896	-0.28144	17	8902590	R	II		IGR	shelf	IGR-shelf	chr17:890	rs5738899	48;40;31;25
cg1090352	0.241787	0.508957	9.126211	2.3E-05	0.001177	3.273409	0.388064	0.629851	-0.24179	5	1.57E+08	F	II	ADAM19	Body	opensea	Body-opens		rs7476710	28;48
cg0826743	0.205015	0.623879	9.126041	2.3E-05	0.001177	3.273272	0.521371	0.726386	-0.20502	1	2.06E+08	R	II	MFSD4	Body	opensea	Body-opens		rs5562286	4;1
cg2329986	0.238824	0.590622	9.12461	2.3E-05	0.001178	3.272125	0.47121	0.710034	-0.23882	1	53326448	F	II	ZYG11A	Body	opensea	Body-opens		rs5273807	0;1
cg2216303	0.217032	0.604099	9.124429	2.3E-05	0.001178	3.27198	0.495583	0.712616	-0.21703	4	80781490	R	II	PCAT4	Body	opensea	Body-opens		rs7653619	46
cg2761666	0.2487	0.480569	9.123779	2.3E-05	0.001178	3.271458	0.356219	0.604919	-0.2487	2	1.68E+08	R	II		IGR	opensea	IGR-opense		rs3719208	48;2
cg1507405	0.230135	0.594864	9.123659	2.3E-05	0.001178	3.271362	0.479796	0.709931	-0.23014	2	2.17E+08	F	II	LINC00607	Body	opensea	Body-opens		rs5375507	13
cg1861800	0.277259	0.649429	9.122568	2.31E-05	0.001178	3.270488	0.510799	0.788058	-0.27726	5	1.23E+08	R	II		IGR	opensea	IGR-opense		rs1851255	38
cg1121709	0.206327	0.527902	9.121815	2.31E-05	0.001178	3.269883	0.424739	0.631065	-0.20633	1	33560126	R	II	AZIN2	Body	opensea	Body-opens		rs1165054	44;15;13;2
cg1136292	0.201167	0.50207	9.121346	2.31E-05	0.001178	3.269507	0.401486	0.602653	-0.20117	16	87041023	F	II		IGR	opensea	IGR-opense		rs1152986	0;25;29;49
cg1189220	0.233193	0.478739	9.120841	2.31E-05	0.001179	3.269102	0.362142	0.595335	-0.23319	20	44572943	F	II	PCIF1	Body	shore	Body-shore	chr20:445	rs1850555	13;26
cg1074724	0.219494	0.387832	9.120763	2.31E-05	0.001179	3.269039	0.278085	0.497579	-0.21949	21	45033804	R	II	HSF2BP	Body	opensea	Body-opens		rs1403877	50;42;36;27;2
cg1940336	0.214244	0.592952	9.120074	2.31E-05	0.001179	3.268487	0.48583	0.700075	-0.21424	5	67611940	F	II		IGR	opensea	IGR-opense			
cg2164639	0.205445	0.609732	9.118094	2.31E-05	0.00118	3.266897	0.507009	0.712454	-0.20544	3	23710303	F	II		IGR	opensea	IGR-opense		rs5295698	0;1;46;50;51
cg0247739	0.212732	0.603068	9.117421	2.32E-05	0.00118	3.266357	0.496702	0.709434	-0.21273	8	1.05E+08	R	II	DCSTAMP	5'UTR	opensea	5'UTR-oper		rs3697949	23;1
cg1458625	0.206675	0.557388	9.117406	2.32E-05	0.00118	3.266346	0.45405	0.660725	-0.20667	19	43160953	F	II		IGR	opensea	IGR-opense		rs1450077	0;27
cg0849143	0.243143	0.519808	9.116894	2.32E-05	0.00118	3.265935	0.398237	0.64138	-0.24314	1	78442533	R	II	FUBP1	Body	shore	Body-shore	chr1:7844		
cg2426611	0.201615	0.681595	9.116294	2.32E-05	0.00118	3.265454	0.580787	0.782402	-0.20162	1	72263819	F	II	NEGR1-IT1	Body	opensea	Body-opens		rs5434976	8
cg1387661	0.205894	0.623447	9.114164	2.32E-05	0.001181	3.263743	0.5205	0.726394	-0.20589	2	16999102	F	II		IGR	opensea	IGR-opense		rs1162780	0
cg2737971	0.253609	0.549131	9.113326	2.32E-05	0.001181	3.263071	0.422326	0.675935	-0.25361	3	16171610	R	II		IGR	opensea	IGR-opense			
cg0628172	0.208614	0.657807	9.112399	2.32E-05	0.001182	3.262327	0.5535	0.762114	-0.20861	7	20686712	F	II	ABCB5	TSS1500	opensea	TSS1500-op			
cg0666507	0.240292	0.609531	9.111372	2.33E-05	0.001182	3.261502	0.489385	0.729677	-0.24029	14	1.01E+08	R	II	WDR25	Body	opensea	Body-opens		rs1475404	30
cg1776096	0.243431	0.555729	9.11051	2.33E-05	0.001183	3.26081	0.434013	0.677444	-0.24343	7	39012419	F	II		IGR	shelf	IGR-shelf	chr7:3901	rs5274543	1;12;13;23
cg2668325	0.218585	0.671745	9.110297	2.33E-05	0.001183	3.260638	0.562453	0.781038	-0.21859	17	54802123	F	I		IGR	opensea	IGR-opense		rs5635290	16;17;40
cg2385185	0.235336	0.501166	9.108358	2.33E-05	0.001184	3.259081	0.383498	0.618834	-0.23534	15	44380216	R	II	FRMD5	Body	opensea	Body-opens		rs5306438	48;42
cg1301985	0.221911	0.609369	9.108227	2.33E-05	0.001184	3.258976	0.498413	0.720324	-0.22191	2	70945292	R	II	ADD2	5'UTR	opensea	5'UTR-oper		rs3755361	43;30;2
cg2626808	0.221103	0.561152	9.108159	2.33E-05	0.001184	3.258921	0.450601	0.671704	-0.2211	10	98117676	F	II	OPALIN	5'UTR	opensea	5'UTR-oper		rs7415364	33
cg0752192	0.205019	0.384006	9.106666	2.33E-05	0.001185	3.257722	0.281497	0.486515	-0.20502	5	1.4E+08	R	II	SLC4A9	Body	shore	Body-shore	chr5:1397	rs5515573	40;8
cg0874315	0.22726	0.692479	9.106627	2.33E-05	0.001185	3.257691	0.57885	0.806109	-0.22726	22	36346442	F	II	RBFOX2	Body	opensea	Body-opens			
cg0786541	0.206965	0.734358	9.105747	2.34E-05	0.001185	3.256983	0.630875	0.837841	-0.20697	7	22702505	F	II	LOC40131	Body	shelf	Body-shelf	chr7:2270	rs5607884	50
cg1174230	0.220981	0.466786	9.103918	2.34E-05	0.001185	3.255513	0.356296	0.577276	-0.22098	6	1.68E+08	F	II	MLLT4	5'UTR	shelf	5'UTR-shelf	chr6:1682		
cg2052907	0.201168	0.663387	9.10386	2.34E-05	0.001185	3.255467	0.562803	0.763971	-0.20117	4	20286834	R	II	SLIT2	Body	opensea	Body-opens			
cg1961950	0.233046	0.421988	9.103286	2.34E-05	0.001186	3.255006	0.305465	0.538511	-0.23305	11	85850481	F	II		IGR	opensea	IGR-opense		rs1504211	10;30;36;43
cg0238494	0.241454	0.430669	9.103159	2.34E-05	0.001186	3.254903	0.309942	0.551396	-0.24145	3	1.96E+08	R	II		IGR	opensea	IGR-opense			
cg1449182	0.208734	0.582129	9.102546	2.34E-05	0.001186	3.254411	0.477762	0.686496	-0.20873	18	36124953	F	II		IGR	opensea	IGR-opense		rs5415594	42
cg1834540	0.207811	0.683228	9.101803	2.34E-05	0.001186	3.253814	0.579322	0.787133	-0.20781	4	1.52E+08	R	II	FAM160A1	5'UTR	opensea	5'UTR-oper			
cg0879770	0.203227	0.535663	9.101261	2.34E-05	0.001186	3.253378	0.434049	0.637276	-0.20323	16	65692605	F	II		IGR	opensea	IGR-opense			
cg1161645	0.252417	0.372722	9.099903	2.35E-05	0.001186	3.252286	0.246514	0.498931	-0.25242	4	38525723	R	II	LINC01258	TSS1500	opensea	TSS1500-op		rs1832261	33
cg0130344	0.215358	0.658537	9.098516	2.35E-05	0.001187	3.251171	0.550858	0.766215	-0.21536	6	1.47E+08	R	II	ADGB	Body	opensea	Body-opens		rs1844026	48;20;10;1
cg1017703	0.205217	0.452743	9.098202	2.35E-05	0.001187	3.250918	0.350134	0.555351	-0.20522	20	47897207	R	II	C20orf199	Body	shelf	Body-shelf	chr20:478	rs5637059	11
cg1975451	0.257378	0.558571	9.0956	2.35E-05	0.001188	3.248825	0.429882	0.68726	-0.25738	2	2.25E+08	F	II	WDFY1	ExonBnd	opensea	ExonBnd-op		rs1407775	0;2;6
cg1636605	0.222427	0.640395	9.094995	2.36E-05	0.001188	3.248339	0.529181	0.751608	-0.22243	7	93365934	F	II		IGR	opensea	IGR-opense			
cg1385884	0.216458	0.589287	9.094746	2.36E-05	0.001189	3.248139	0.481058	0.697516	-0.21646	10	1.29E+08	R	II	DOCK1	Body	opensea	Body-opens		rs2010144	49;47
cg2029166	0.200648	0.443059	9.094581	2.36E-05	0.001189	3.248006	0.342736	0.543383	-0.20065	7	1.16E+08	R	II		IGR	opensea	IGR-opense		rs5576818	37
cg2319801	0.224861	0.618476	9.094148	2.36E-05	0.001189	3.247658	0.506045	0.730906	-0.22486	1	1.62E+08	R	II		IGR	opensea	IGR-opense		rs1505133	46;31;30
cg0606759	0.224955	0.610042	9.093666	2.36E-05	0.001189	3.247269	0.497564	0.722519	-0.22496	4	1.77E+08	R	II	GPM6A	5'UTR	shelf	5'UTR-shelf	chr4:1769	rs5297090	2
cg2113981	0.204252	0.72849	9.09341	2.36E-05	0.001189	3.247064	0.626364	0.830616	-0.20425	8	75911816	R	II	CRISPLD1	TSS1500	opensea	TSS1500-op		rs5578140	17
cg1643668	0.218142	0.626852	9.093346	2.36E-05	0.001189	3.247012	0.517781	0.735923	-0.21814	1	1.86E+08	F	II	HMCN1	Body	opensea	Body-opens		rs5560106	0;14;35
cg0507523	0.210484	0.688598	9.092984	2.36E-05	0.001189	3.246721	0.583356	0.793841	-0.21048	6	1.26E+08	R	II	LOC64362	TSS1500	opensea	TSS1500-op		rs6940748	34;1
cg2368895	0.21874	0.654578	9.09279	2.36E-05	0.001189	3.246565	0.545208	0.763948	-0.21874	1	2.3E+08	F	II		IGR	opensea	IGR-opense		rs1128375	13;28;48
cg0730179	0.244405	0.39227	9.092149	2.36E-05	0.001189	3.246049	0.270068	0.514473	-0.24441	3	1.68E+08	F	II	LINC01330	Body	opensea	Body-opens		rs1491570	0;1;51
cg2391616	0.200797	0.53734	9.091071	2.36E-05	0.00119	3.245182	0.436942	0.637739	-0.2008	5	59326529	R	II	PDE4D	Body	opensea	Body-opens			
cg0498924	0.216769	0.417441	9.090163	2.36E-05	0.00119	3.244451	0.309056	0.525826	-0.21677	2	1.39E+08	F	II		IGR	opensea	IGR-opense			
cg2726697	0.21278	0.623779	9.08982	2.37E-05	0.00119	3.244175	0.517389	0.730169	-0.21278	3	1.24E+08	F	II	MYLK	5'UTR	opensea	5'UTR-oper		rs5585121	31
cg2064263	0.217394	0.610523	9.089611	2.37E-05	0.00119	3.244007	0.501826	0.71922	-0.21739	8	22846006	F	II	RHOBTB2	5'UTR	opensea	5'UTR-oper		rs5300857	13
cg1635485	0.229709	0.657492	9.088551	2.37E-05	0.00119	3.243154	0.542637	0.772347	-0.22971	12	19218990	F	II		IGR	opensea	IGR-opense		rs5481738	12
cg1435470	0.205811	0.57471	9.087346	2.37E-05	0.00119	3.242183	0.471805	0.677615	-0.20581	11	7985276	R	II	NLRP10	TSS1500	opensea	TSS1500-op		rs1147846	51;25
cg0785917	0.203163	0.556112	9.085392	2.37E-05	0.001191	3.24061	0.45453	0.657693	-0.20316	15	57098497	R	II		IGR	opensea	IGR-opense		rs1120718	1
cg1756448	0.215836	0.649785	9.08469	2.37E-05	0.001192	3.240045	0.541867	0.757703	-0.21584	12	1.11E+08	R	II		IGR	opensea	IGR-opense		rs5566322	1
cg2166463	0.257763	0.511192	9.084453	2.38E-05	0.001192	3.239854	0.382311	0.640074	-0.25776	3	70432664	R	I		IGR	opensea	IGR-opense		rs1820123	13;3
cg1785186	0.255222	0.470031	9.083941	2.38E-05	0.001192	3.239442	0.34242	0.597642	-0.25522	2	58748306	F	II		IGR	opensea	IGR-opense		rs1864157	0;3;44
cg0315534	0.222247	0.638999	9.079909	2.38E-05	0.001194	3.236194	0.527876	0.750123	-0.22225	4	11642410	F	II		IGR	opensea	IGR-opense		rs3675930	21;45
cg0502469	0.201128	0.616799	9.07926	2.38E-05	0.001194	3.235671	0.516234	0.717363	-0.20113	4	1.6E+08	R	II		IGR	opensea	IGR-opense		rs5278146	28;25;2
cg2406791	0.250227	0.540693	9.076158	2.39E-05	0.001196	3.23317	0.415579	0.665806	-0.25023	6	16729610	F	II	ATXN1	5'UTR	opensea	5'UTR-oper			
cg1172047	0.20885	0.632922	9.075539	2.39E-05	0.001196	3.232672	0.528497	0.737347	-0.20885	1	82481416	F	II		IGR	opensea	IGR-opense			
cg1046790	0.202248	0.667561	9.075093	2.39E-05	0.001196	3.232312	0.566437	0.768685	-0.20225	10	1.22E+08	R	II	LINC01561	Body	opensea	Body-opens		rs5305244	31;6
cg0535941	0.21091	0.554747	9.075006	2.39E-05	0.001196	3.232242	0.449292	0.660202	-0.21091	1	2.08E+08	R	II	CR1	Body	opensea	Body-opens		rs5529182	35;1
cg0505081	0.22079	0.484531	9.074034	2.39E-05	0.001197	3.231459	0.374137	0.594926	-0.22079	6	42745007	R	II		IGR	opensea	IGR-opense		rs5606735	34;33;14
cg2638131	0.223765	0.742997	9.073257	2.4E-05	0.001197	3.230832	0.631114	0.85488	-0.22377	1	93042953	R	II	EVI5	Body	opensea	Body-opens		rs5717128	5;2
cg0087044	0.200887	0.558818	9.072888	2.4E-05	0.001197	3.230534	0.458374	0.659262	-0.20089	2	40508306	R	II	SLC8A1	Body	opensea	Body-opens		rs5623954	45;26;7
cg0603162	0.228951	0.599995	9.072478	2.4E-05	0.001197	3.230204	0.48552	0.714471	-0.22895	20	35923593	F	II	MANBAL	5'UTR	opensea	5'UTR-oper			
cg0946539	0.227573	0.670633	9.07041	2.4E-05	0.001198	3.228536	0.556847	0.784419	-0.22757	4	1.41E+08	R	II	MAML3	Body	opensea	Body-opens		rs5616773	38;2

cg0895383	0.201884	0.484809	9.069068	2.4E-05	0.001199	3.227454	0.383867	0.58575	-0.20188	20	56704172	F	II		IGR	opensea	IGR-opense		rs1151134	28
cg2637715	0.204041	0.701847	9.068418	2.41E-05	0.001199	3.22693	0.599826	0.803867	-0.20404	1	1.51E+08	R	II	HORMAD1	Body	opensea	Body-opens		rs5876837	22
cg2734106	0.214939	0.583395	9.06787	2.41E-05	0.001199	3.226488	0.475926	0.690864	-0.21494	20	10055284	R	II	SNAP25-AS	Body	opensea	Body-opens		rs5693178	33
cg1256389	0.205555	0.555725	9.067805	2.41E-05	0.001199	3.226435	0.452948	0.658503	-0.20555	22	32978938	R	II	SYN3	Body	opensea	Body-opens		rs5630659	49
cg0939011	0.223556	0.431226	9.067104	2.41E-05	0.001199	3.22587	0.319448	0.543004	-0.22356	1	1.97E+08	F	II	CFH	Body	opensea	Body-opens		rs1905743	12;48
cg0357090	0.229473	0.614809	9.066381	2.41E-05	0.0012	3.225286	0.500073	0.729546	-0.22947	10	7682790	R	II	ITIH5	Body	opensea	Body-opens		rs5705894	34;22;1
cg0203986	0.227739	0.582649	9.065246	2.41E-05	0.0012	3.22437	0.468779	0.696518	-0.22774	10	97518677	F	II	ENTPD1	Body	opensea	Body-opens		rs5328523	0;22
cg1530636	0.215303	0.653934	9.063898	2.41E-05	0.001201	3.223282	0.546282	0.761585	-0.2153	1	2.07E+08	R	II		IGR	opensea	IGR-opense		rs1392161	50
cg0053117	0.20361	0.639072	9.063603	2.41E-05	0.001201	3.223044	0.537267	0.740877	-0.20361	8	28814510	F	II	HMBOX1	5'UTR	opensea	5'UTR-open			
cg1362024	0.269424	0.450379	9.063432	2.41E-05	0.001201	3.222906	0.315667	0.585091	-0.26942	9	1.26E+08	F	II	DENND1A	Body	opensea	Body-opens		rs5630428	48
cg2515918	0.232949	0.542602	9.063156	2.41E-05	0.001201	3.222683	0.426128	0.659077	-0.23295	2	2.31E+08	R	II	TRIP12	Body	opensea	Body-opens			
cg1420924	0.237526	0.600031	9.06114	2.42E-05	0.001202	3.221056	0.481267	0.718794	-0.23753	3	14307176	F	II		IGR	opensea	IGR-opense		rs5690595	31
cg2521788	0.213477	0.537821	9.061051	2.42E-05	0.001202	3.220984	0.431082	0.64456	-0.21348	19	14975658	R	II		IGR	opensea	IGR-opense		rs5425417	7;6;2
cg0521867	0.2001	0.564915	9.060742	2.42E-05	0.001202	3.220735	0.464865	0.664965	-0.2001	6	46081541	F	II		IGR	opensea	IGR-opense			
cg0078232	0.211471	0.616258	9.060685	2.42E-05	0.001202	3.220688	0.510523	0.721994	-0.21147	12	30351139	F	II		IGR	shelf	IGR-shelf	chr12:303!	rs1482863	0
cg0150781	0.202698	0.476664	9.058534	2.42E-05	0.001202	3.218951	0.375315	0.578014	-0.2027	17	8035482	R	II		IGR	opensea	IGR-opense		rs5441448	31;27;19
cg1908577	0.211783	0.685692	9.058286	2.42E-05	0.001202	3.218751	0.5798	0.791584	-0.21178	10	90022495	R	II		IGR	opensea	IGR-opense			
cg1379557	0.250357	0.543448	9.058264	2.42E-05	0.001202	3.218734	0.418269	0.668626	-0.25036	3	35747962	R	II	ARPP21	Body	opensea	Body-opens		rs5677037	7
cg2219921	0.204897	0.525071	9.056784	2.43E-05	0.001203	3.217539	0.422623	0.62752	-0.2049	9	7569630	R	II		IGR	opensea	IGR-opense		rs1268613	22;2;1
cg1889441	0.208172	0.654795	9.055834	2.43E-05	0.001203	3.216771	0.550709	0.758881	-0.20817	10	99053792	R	II	ARHGAP19	TSS1500	shore	TSS1500-sh	chr10:990!	rs3572887	31
cg2644808	0.231501	0.513219	9.055535	2.43E-05	0.001203	3.21653	0.397469	0.62897	-0.2315	5	1.71E+08	R	II	FBXW11	Body	opensea	Body-opens			
cg0935305	0.252171	0.438505	9.055022	2.43E-05	0.001203	3.216115	0.312419	0.56459	-0.25217	2	1.67E+08	R	II	SCN1A	3'UTR	opensea	3'UTR-open			
cg0391251	0.226944	0.566454	9.053785	2.43E-05	0.001204	3.215115	0.452982	0.679926	-0.22694	8	19221205	R	II	SH2D4A	Body	opensea	Body-opens			
cg0829978	0.201734	0.512291	9.052844	2.43E-05	0.001204	3.214356	0.411423	0.613158	-0.20173	2	48920986	F	II	GTF2A1L	Body	opensea	Body-opens		rs1875015	25
cg2736037	0.230906	0.616548	9.051005	2.44E-05	0.001205	3.212869	0.501095	0.732001	-0.23091	2	1.67E+08	R	II		IGR	opensea	IGR-opense		rs5608819	1
cg0881151	0.208804	0.537828	9.049249	2.44E-05	0.001205	3.21145	0.433427	0.64223	-0.2088	13	35043968	R	II	LINC00457	Body	opensea	Body-opens		rs5483855	47
cg0570946	0.22514	0.605789	9.049046	2.44E-05	0.001205	3.211286	0.493219	0.718359	-0.22514	18	21794764	F	II	OSBPL1A	Body	opensea	Body-opens		rs1424704	1;15
cg0207725	0.232043	0.47293	9.049031	2.44E-05	0.001205	3.211274	0.356909	0.588951	-0.23204	17	15133981	R	II	PMP22	3'UTR	opensea	3'UTR-open			
cg0718021	0.227741	0.551824	9.048092	2.44E-05	0.001206	3.210514	0.437953	0.665695	-0.22774	17	74557703	F	II	SNORD1A	TSS200	shelf	TSS200-she	chr17:745!	rs2021779	1;15;18
cg1372021	0.224813	0.664706	9.047901	2.44E-05	0.001206	3.21036	0.552299	0.777112	-0.22481	6	1.28E+08	R	II	PTPRK	Body	opensea	Body-opens		rs1463301	31;18;1
cg0846138	0.215772	0.506444	9.047619	2.44E-05	0.001206	3.210132	0.398558	0.614329	-0.21577	12	1.09E+08	F	II		IGR	opensea	IGR-opense		rs3736354	7;49
cg2299056	0.221554	0.499584	9.046906	2.45E-05	0.001206	3.209556	0.388807	0.610362	-0.22155	9	1.2E+08	R	II	ASTN2	Body	opensea	Body-opens		rs1868995	1
cg1076599	0.221601	0.678206	9.046145	2.45E-05	0.001206	3.208941	0.567405	0.789006	-0.2216	15	99722217	R	II	TTC23	Body	opensea	Body-opens		rs1512321	15;11;8;7
cg0762800	0.230131	0.543161	9.045035	2.45E-05	0.001207	3.208043	0.428095	0.658226	-0.23013	1	61365275	F	II		IGR	opensea	IGR-opense			
cg2085111	0.211725	0.585694	9.043904	2.45E-05	0.001207	3.207128	0.479831	0.691556	-0.21172	8	1.3E+08	F	II		IGR	opensea	IGR-opense		rs7898632	1
cg0086820	0.248395	0.471073	9.043622	2.45E-05	0.001207	3.2069	0.346876	0.595271	-0.2484	6	1.5E+08	F	II	TAB2	Body	shore	Body-shore	chr6:1496:	rs1445708	34;51
cg1278569	0.20319	0.599246	9.043255	2.45E-05	0.001208	3.206603	0.497651	0.700842	-0.20319	18	57146966	R	II	CCBE1	Body	opensea	Body-opens		rs1789476	45;43;1
cg0838841	0.244508	0.540418	9.041867	2.46E-05	0.001208	3.20548	0.418165	0.662672	-0.24451	2	2.26E+08	R	II	DOCK10	Body	opensea	Body-opens		rs5635179	7
cg0222857	0.227024	0.611202	9.040702	2.46E-05	0.001209	3.204538	0.49769	0.724714	-0.22702	12	94441067	R	II		IGR	opensea	IGR-opense		rs1388971	40
cg1513519	0.293903	0.649838	9.039613	2.46E-05	0.001209	3.203656	0.502886	0.796789	-0.2939	6	1.33E+08	F	II	VNN2	Body	opensea	Body-opens		rs1472691	1;4;35;37
cg0431896	0.222375	0.716669	9.038081	2.46E-05	0.00121	3.202417	0.605481	0.827856	-0.22237	12	46005881	R	II		IGR	opensea	IGR-opense		rs1895940	19;9;6
cg1761272	0.20015	0.550926	9.03499	2.47E-05	0.001211	3.199915	0.450852	0.651001	-0.20015	8	81235210	R	II		IGR	opensea	IGR-opense		rs5492705	42;2;1
cg1766458	0.221774	0.522978	9.034089	2.47E-05	0.001211	3.199186	0.412091	0.633865	-0.22177	16	23853554	R	II	PRKCB	Body	opensea	Body-opens		rs7195232	35;31;1
cg0548188	0.208657	0.53447	9.034079	2.47E-05	0.001211	3.199177	0.430142	0.638799	-0.20866	3	1.51E+08	F	II		IGR	opensea	IGR-opense		rs5666432	30;42
cg1273357	0.213675	0.4116	9.033665	2.47E-05	0.001211	3.198842	0.304763	0.518438	-0.21367	12	5261497	F	II		IGR	opensea	IGR-opense		rs1158490	0;1;19;44
cg0466172	0.236931	0.502135	9.033403	2.47E-05	0.001211	3.19863	0.38367	0.620601	-0.23693	5	83007630	F	II	HAPLN1	5'UTR	opensea	5'UTR-open		rs5496193	9;26
cg1354600	0.242147	0.558936	9.030398	2.48E-05	0.001213	3.196197	0.437862	0.680009	-0.24215	1	1.93E+08	F	II	LINC01031	Body	opensea	Body-opens			
cg1854628	0.203281	0.574952	9.029444	2.48E-05	0.001214	3.195424	0.473312	0.676592	-0.20328	2	2.34E+08	R	II	NGEF	Body	opensea	Body-opens		rs1878709	14
cg1113246	0.235439	0.584877	9.029397	2.48E-05	0.001214	3.195386	0.467158	0.702597	-0.23544	8	1.42E+08	R	II	PTP4A3	5'UTR	shelf	5'UTR-shelf	chr8:1424:	rs1832582	44;26
cg2266603	0.259769	0.518895	9.028685	2.48E-05	0.001214	3.194809	0.389011	0.64878	-0.25977	1	1.5E+08	F	II	VPS45	Body	opensea	Body-opens		rs1419694	0;27
cg0528493	0.218794	0.547921	9.027729	2.48E-05	0.001215	3.194034	0.438524	0.657317	-0.21879	2	2.18E+08	R	II		IGR	opensea	IGR-opense			
cg2224770	0.234389	0.630186	9.027372	2.48E-05	0.001215	3.193745	0.512992	0.747381	-0.23439	13	39953175	F	II	LHFP	Body	opensea	Body-opens		rs1492705	3;20;26;39;47
cg0100476	0.202671	0.580245	9.027044	2.48E-05	0.001215	3.193479	0.478909	0.68158	-0.20267	9	34956991	R	II	KIAA1045	TSS1500	shore	TSS1500-sh	chr9:3495:	rs5706344	5
cg1294863	0.228957	0.508989	9.026282	2.49E-05	0.001215	3.192862	0.394511	0.623467	-0.22896	6	23891030	R	II		IGR	opensea	IGR-opense		rs3718337	34;11;9
cg1008439	0.228538	0.560115	9.026059	2.49E-05	0.001215	3.192681	0.445846	0.674384	-0.22854	3	35822275	R	II	ARPP21	Body	opensea	Body-opens		rs5390510	27;18;8
cg0216785	0.208872	0.581132	9.025789	2.49E-05	0.001215	3.192463	0.476695	0.685568	-0.20887	2	1.23E+08	R	II		IGR	opensea	IGR-opense		rs1896881	32;31;30;12;2
cg2758322	0.217636	0.61124	9.02532	2.49E-05	0.001215	3.192083	0.502423	0.720058	-0.21764	16	3064377	F	II	CLDN9	3'UTR	shore	3'UTR-shor	chr16:306:	rs5589085	48
cg0166344	0.232799	0.571801	9.02481	2.49E-05	0.001215	3.191669	0.455402	0.688201	-0.2328	12	1056096	R	II	RAD52	5'UTR	shelf	5'UTR-shelf	chr12:105!	rs5578862	22
cg1679919	0.224262	0.591298	9.021489	2.49E-05	0.001217	3.188977	0.479167	0.703429	-0.22426	3	1.12E+08	R	II		IGR	opensea	IGR-opense		rs1848708	47;13;1
cg1668296	0.220295	0.644985	9.019918	2.5E-05	0.001217	3.187704	0.534837	0.755132	-0.2203	3	1.75E+08	F	II	NAALADL2-	TSS1500	opensea	TSS1500-op		rs1437869	0;14
cg2116168	0.205577	0.642409	9.019236	2.5E-05	0.001218	3.187151	0.53962	0.745197	-0.20558	5	1.6E+08	R	II	ATP10B	Body	opensea	Body-opens		rs5338251	42;10;1
cg1545269	0.275289	0.597897	9.018472	2.5E-05	0.001218	3.186531	0.460252	0.735541	-0.27529	3	1.02E+08	R	II		IGR	opensea	IGR-opense		rs5704379	51;47;46;22;11
cg1671876	0.216592	0.581445	9.017423	2.5E-05	0.001219	3.18568	0.473149	0.689741	-0.21659	3	55721067	R	II	ERC2	Body	opensea	Body-opens			
cg1606205	0.221789	0.685082	9.017202	2.5E-05	0.001219	3.185501	0.574188	0.795977	-0.22179	3	1.68E+08	F	II	MIR551B	TSS1500	opensea	TSS1500-op		rs5506286	11
cg2583286	0.200448	0.665009	9.016853	2.5E-05	0.001219	3.185218	0.564785	0.765233	-0.20045	12	22952457	R	II		IGR	opensea	IGR-opense		rs5556303	48;36;33;7
cg1108772	0.246514	0.580677	9.016109	2.5E-05	0.00122	3.184614	0.45742	0.703934	-0.24651	5	56894899	R	II		IGR	opensea	IGR-opense		rs1136506	48
cg1983189	0.23087	0.692066	9.015294	2.51E-05	0.00122	3.183953	0.576631	0.807501	-0.23087	2	37313112	R	II	GPATCH11	5'UTR	shore	5'UTR-shor	chr2:3731:	rs1505764	49;35;2
cg0303542	0.258138	0.50293	9.014662	2.51E-05	0.00122	3.183441	0.37386	0.631999	-0.25814	2	2.02E+08	R	II	ORC2	Body	opensea	Body-opens			
cg2577774	0.201658	0.650515	9.013046	2.51E-05	0.00122	3.182129	0.549686	0.751344	-0.20166	20	55626400	F	II		IGR	opensea	IGR-opense		rs5643636	0;22
cg2494268	0.23781	0.66188	9.012911	2.51E-05	0.00122	3.18202	0.542975	0.780785	-0.23781	13	77518644	R	II		IGR	opensea	IGR-opense			
cg1931887	0.224841	0.549083	9.011949	2.51E-05	0.001221	3.181238	0.436663	0.661504	-0.22484	18	5413514	F	II	EPB41L3	Body	opensea	Body-opens		rs5582564	2;7;34;40
cg0351582	0.204732	0.473136	9.011809	2.51E-05	0.001221	3.181126	0.37077	0.575502	-0.20473	7	1.34E+08	F	II		IGR	opensea	IGR-opense		rs5742849	0;22;34
cg2489195	0.224696	0.525675	9.010358	2.52E-05	0.001222	3.179948	0.413327	0.638022	-0.2247	19	2669059	F	II	GNG7	5'UTR	opensea	5'UTR-open		rs5986768	32
cg1800663	0.248426	0.485062	9.007812	2.52E-05	0.001223	3.177881	0.360849	0.609275	-0.24843	4	89713820	F	II	FAM13A	Body	opensea	Body-opens			
cg1416162	0.203825	0.761577	9.007777	2.52E-05	0.001223	3.177852	0.659664	0.863489	-0.20383	1	1.75E+08	R	II	LOC10192!	Body	opensea	Body-opens			
cg2507933	0.23216	0.654649	9.007207	2.52E-05	0.001223	3.177389	0.538569	0.770729	-0.23216	8	1.28E+08	F	II	PRNCR1	Body	opensea	Body-opens		rs5596037	13;23;47
cg2259067	0.217363	0.612822	9.006524	2.52E-05	0.001223	3.176835	0.50414	0.721503	-0.21736	5	1.45E+08	F	II	SH3RF2	Body	opensea	Body-opens		rs5577051	0;5;18
cg1083207	0.2244	0.476042	9.006517	2.52E-05	0.001223	3.176829	0.363842	0.588241	-0.2244	12	21418929	F	II	SLCO1A2	3'UTR	opensea	3'UTR-open		rs5359474	1;10;40
cg1441989	0.207329	0.594199	9.005744	2.53E-05	0.001224	3.176201	0.490534	0.697863	-0.20733	4	1.41E+08	R	II	MAML3	Body	opensea	Body-opens		rs5277616	27;1
cg1968802	0.2135	0.676664	9.005447	2.53E-05	0.001224	3.17596	0.569914	0.783414	-0.2135	14	90922528	R	II	LINC00642	Body	opensea	Body-opens		rs5304790	32;31;30;18;17;15;14;1
cg0047456	0.217183	0.421392	9.005122	2.53E-05	0.001224	3.175696	0.312801	0.529984	-0.21718	6	1.35E+08	F	II	SGK1	Body	opensea	Body-opens		rs5497521	11
cg2202122	0.248314	0.422799	9.00506	2.53E-05	0.001224	3.175646	0.298642	0.546956	-0.24831	8	43015521	F	II	HGSNAT	Body	opensea	Body-opens		rs3700179	0;22
cg2461313	0.270132	0.469674	9.004832	2.53E-05	0.001224	3.175461	0.334608	0.60474	-0.27013	8	1.29E+08	R	II		IGR	opensea	IGR-opense			
cg2563552	0.206767	0.601808	9.004059	2.53E-05	0.001224	3.174833	0.498425	0.705192	-0.20677	2	2.18E+08	F	II		IGR	opensea	IGR-opense		rs1862718	1;44
cg1929346	0.205077	0.534416	9.003276	2.53E-05	0.001224	3.174197	0.431878	0.636955	-0.20508	17	1973400	F	I	SMG6	Body	opensea	Body-opens		rs5709369	16;44
cg1017954	0.237811	0.597875	9.003047	2.53E-05	0.001224	3.174011	0.47897	0.716781	-0.23781	20	43177026	R	II	PKIG	5'UTR	opensea	5'UTR-open			
cg2234576	0.218064	0.649191	9.002918	2.53E-05	0.001224	3.173906	0.540159	0.758223	-0.21806	3	37614806	R	II	ITGA9	Body	opensea	Body-opens		rs5718780	46;40;20;1
cg0102030	0.202289	0.547415	9.002874	2.53E-05	0.001224	3.17387	0.446271	0.64856	-0.20229	8	1.03E+08	R	II	GRHL2	3'UTR	opensea	3'UTR-open		rs5452438	1
cg2764885	0.216353	0.654342	8.997785	2.54E-05	0.001227	3.169735	0.546166	0.762519	-0.21635	1	2.48E+08	R	II		IGR	opensea	IGR-opense			
cg0224198	0.205966	0.433332	8.997451	2.54E-05	0.001227	3.169464	0.330349	0.536315	-0.20597	10	5566543	R	II	CALML3	TSS1500	shore	TSS1500-sh	chr10:556:	rs5377631	19
cg1998085	0.24173	0.576377	8.994838	2.55E-05	0.001228	3.16734	0.455512	0.697242	-0.24173	9	84947928	F	II		IGR	opensea	IGR-opense		rs1512624	9
cg1394377	0.215411	0.558699	8.994832	2.55E-05	0.001228	3.167335	0.450994	0.666405	-0.21541	3	1.27E+08	R	II		IGR	opensea	IGR-opense			
cg2442772	0.24596	0.555363	8.994791	2.55E-05	0.001228	3.167301	0.432384	0.678343	-0.24596	12	6234257	F	II	VWF	TSS1500	opensea	TSS1500-op		rs6175044	1;44
cg2037253	0.22071	0.623377	8.993392	2.55E-05	0.001228	3.166164	0.513022	0.733732	-0.22071	1	1.55E+08	R	II		IGR	opensea	IGR-opense		rs5504320	45
cg2125094	0.242084	0.531974	8.99269	2.55E-05	0.001228	3.165593	0.410932	0.653016	-0.24208	3	24181646	F	II	THRB	Body	opensea	Body-opens		rs5681678	26
cg1904007	0.207431	0.488508	8.989921	2.56E-05	0.00123	3.163341	0.384793	0.592224	-0.20743	2	2.43E+08	F	II	D2HGDH	Body	opensea	Body-opens		rs5636153	1;6;13;23;34
cg0248580	0.232706	0.57668	8.988062	2.56E-05	0.001231	3.161828	0.460327	0.693033	-0.23271	15	60834343	F	II	RORA-AS1	Body	opensea	Body-opens		rs5460707	0;29
cg1936975	0.232485	0.448926	8.98798	2.56E-05	0.001231	3.161762	0.332684	0.565168	-0.23248	7	17442601	R	II	KCCAT333	Body	opensea	Body-opens			
cg0679542	0.213518	0.578717	8.98761	2.56E-05	0.001231	3.16146	0.471958	0.685477	-0.21352	19	43857111	R	II	CD177	TSS1500	opensea	TSS1500-op		rs5484376	38;1
cg0210038	0.20957	0.497989	8.987509	2.56E-05	0.001231	3.161379	0.393204	0.602774	-0.20957	13	1.11E+08	F	II	COL4A2	Body	opensea	Body-opens		rs1886978	0;19;49
cg2307339	0.210316	0.645881	8.986292	2.56E-05	0.001231	3.160388	0.540723	0.751039	-0.21032	4	71740118	R	II		IGR	opensea	IGR-opense		rs5502072	1
cg1033721	0.20539	0.6254	8.985882	2.56E-05	0.001231	3.160054	0.522705	0.728095	-0.20539	8	89970130	F	II		IGR	opensea	IGR-opense		rs1901400	5;19;45
cg0896830	0.247844	0.600789	8.985829	2.56E-05	0.001231	3.160012	0.476867	0.724711	-0.24784	6	97098046	R	II		IGR	opensea	IGR-opense		rs5610783	37;31;22;6;2
cg2166747	0.207888	0.674818	8.984468	2.57E-05	0.001232	3.158904	0.570874	0.778762	-0.20789	4	15716937	F	II	BST1	Body	opensea	Body-opens		rs2021514	0
cg0912761	0.236016	0.63907	8.984056	2.57E-05	0.001232	3.158568	0.521062	0.757078	-0.23602	5	58551379	F	II	PDE4D	Body	opensea	Body-opens			
cg1731687	0.215434	0.515664	8.982622	2.57E-05	0.001233	3.157401	0.407947	0.623381	-0.21543	12	1.23E+08	R	II	PITPNM2	Body	shelf	Body-shelf	chr12:123	rs5374403	44;31;2
cg0578358	0.223775	0.619382	8.982575	2.57E-05	0.001233	3.157363	0.507494	0.731269	-0.22378	1	2.01E+08	F	II	TMEM9	Body	opensea	Body-opens			
cg0510486	0.201368	0.682805	8.982202	2.57E-05	0.001233	3.157059	0.58212	0.783489	-0.20137	4	1.7E+08	R	II	SH3RF1	Body	opensea	Body-opens		rs5643051	33
cg0037224	0.202993	0.550914	8.981915	2.57E-05	0.001233	3.156826	0.449417	0.65241	-0.20299	4	1.86E+08	F	II	ACSL1	5'UTR	shore	5'UTR-shor	chr4:1857	rs5429250	4
cg2417631	0.20736	0.56628	8.98136	2.57E-05	0.001234	3.156374	0.4626	0.66996	-0.20736	8	94495440	R	II	LINC00535	Body	opensea	Body-opens		rs5594403	27
cg0899990	0.227383	0.535583	8.980848	2.57E-05	0.001234	3.155957	0.421891	0.649274	-0.22738	12	91331583	F	II	LINC00615	Body	opensea	Body-opens		rs1474119	0
cg0314653	0.21143	0.676975	8.97962	2.58E-05	0.001234	3.154957	0.571259	0.78269	-0.21143	4	74587910	R	II		IGR	opensea	IGR-opense		rs5318357	16;1
cg1612803	0.230533	0.474943	8.977927	2.58E-05	0.001235	3.153578	0.359676	0.590209	-0.23053	3	53664611	F	II	CACNA1D	Body	opensea	Body-opens		rs1387787	22
cg1591565	0.220796	0.736393	8.976572	2.58E-05	0.001235	3.152474	0.625995	0.846791	-0.2208	11	65262358	F	II		IGR	shelf	IGR-shelf	chr11:652	rs5521616	9
cg0494922	0.255303	0.508285	8.975732	2.59E-05	0.001235	3.15179	0.380633	0.635936	-0.2553	13	50796845	F	II		IGR	opensea	IGR-opense		rs5384783	0;8;21;24
cg0749824	0.207183	0.647061	8.975605	2.59E-05	0.001235	3.151686	0.543469	0.750652	-0.20718	2	2E+08	R	II		IGR	shelf	IGR-shelf	chr2:2004		
cg0567437	0.215412	0.412135	8.975509	2.59E-05	0.001235	3.151608	0.304429	0.519841	-0.21541	20	43275684	R	II	ADA	Body	opensea	Body-opens			
cg2478129	0.20456	0.436713	8.974983	2.59E-05	0.001235	3.15118	0.334433	0.538993	-0.20456	9	1.12E+08	R	II		IGR	opensea	IGR-opense		rs5638965	43;18;5
cg1570253	0.248278	0.50896	8.974056	2.59E-05	0.001236	3.150424	0.384821	0.633099	-0.24828	5	1.27E+08	R	II	MEGF10	Body	opensea	Body-opens		rs31483;rs	45;33;31;26;17

cg1338251	0.228636	0.640351	8.972806	2.59E-05	0.001236	3.149406	0.526033	0.754669	-0.22864	10	52336353	R	II	SGMS1	5'UTR	opensea	5'UTR-oper		rs5513262	51;48;41;25
cg1905435	0.204003	0.618619	8.971959	2.59E-05	0.001236	3.148715	0.516617	0.720621	-0.204	5	78219426	R	II	ARSB	Body	opensea	Body-opens		rs1866801	51;20;2;1
cg195747C	0.213162	0.571872	8.971408	2.59E-05	0.001237	3.148266	0.465291	0.678453	-0.21316	12	67490706	F	II	LOC10272	TSS1500	opensea	TSS1500-o		rs5491007	4;46
cg2102646	0.20832	0.581273	8.970935	2.59E-05	0.001237	3.147881	0.477113	0.685434	-0.20832	3	13539059	F	II	HDAC11	Body	opensea	Body-opens		rs3683436	8
cg160662C	0.206799	0.530036	8.970779	2.6E-05	0.001237	3.147754	0.426636	0.633435	-0.2068	9	1.1E+08	F	II		IGR	opensea	IGR-opense		rs1868457	36;39;51
cg2280968	0.238647	0.559743	8.969584	2.6E-05	0.001237	3.146779	0.440419	0.679066	-0.23865	1	1.83E+08	F	II	LAMC1	Body	opensea	Body-opens		rs1463331	20;29
cg0769425	0.21824	0.603843	8.969534	2.6E-05	0.001237	3.146739	0.494723	0.712963	-0.21824	8	1.08E+08	R	II	ANGPT1	Body	opensea	Body-opens		rs2837370	44;30;16;1
cg1226215	0.207691	0.592932	8.968392	2.6E-05	0.001238	3.145808	0.489087	0.696778	-0.20769	5	1.07E+08	R	II	EFNA5	Body	opensea	Body-opens		rs5592572	49;26;1
cg2720525	0.225521	0.502478	8.967746	2.6E-05	0.001238	3.145281	0.389717	0.615238	-0.22552	9	1536731	R	II		IGR	opensea	IGR-opense		rs1388997	46;40;2;1
cg195284C	0.212828	0.71624	8.966781	2.6E-05	0.001239	3.144494	0.609826	0.822654	-0.21283	5	10140142	R	II		IGR	opensea	IGR-opense		rs5495528	38;25;1
cg1502903	0.217481	0.500755	8.964391	2.61E-05	0.00124	3.142545	0.392015	0.609496	-0.21748	4	1.47E+08	R	II	ZNF827	Body	opensea	Body-opens		rs5540705	44;41;2
cg1715162	0.203298	0.718252	8.963491	2.61E-05	0.00124	3.141811	0.616603	0.819901	-0.2033	3	1.86E+08	R	I	AHSG	TSS1500	opensea	TSS1500-o		rs5457082	47;43;42;40;14;13;1
cg2445228	0.210344	0.461852	8.963288	2.61E-05	0.001241	3.141645	0.35668	0.567024	-0.21034	6	31542740	R	II	TNF	TSS1500	opensea	TSS1500-o		rs4248160	49
cg0248893	0.216274	0.396514	8.963236	2.61E-05	0.001241	3.141602	0.288377	0.50465	-0.21627	1	1.56E+08	R	II		IGR	shelf	IGR-shelf	chr1:1564		
cg2459849	0.239599	0.473223	8.961835	2.61E-05	0.001241	3.14046	0.353423	0.593022	-0.2396	13	37064804	F	II		IGR	opensea	IGR-opense		rs1381921	1;13;41
cg134087C	0.201003	0.60492	8.961825	2.61E-05	0.001241	3.140451	0.504418	0.705421	-0.201	1	59664430	F	II		IGR	opensea	IGR-opense		rs1848312	19
cg0256897	0.228507	0.63404	8.960394	2.62E-05	0.001242	3.139283	0.519786	0.748293	-0.22851	7	36320660	R	II	EEPD1	Body	opensea	Body-opens		rs5485360	23
cg2462877	0.233967	0.47053	8.960301	2.62E-05	0.001242	3.139208	0.353547	0.587513	-0.23397	8	1.3E+08	F	II		IGR	opensea	IGR-opense		rs1448584	11;20;25;35
cg1674023	0.215086	0.6275	8.959722	2.62E-05	0.001243	3.138735	0.519957	0.735043	-0.21509	6	1.32E+08	R	II	MED23	Body	opensea	Body-opens			
cg0762325	0.219453	0.457724	8.959107	2.62E-05	0.001243	3.138234	0.347998	0.567451	-0.21945	2	67518001	R	II		IGR	opensea	IGR-opense		rs5627685	43;10;9;1
cg1276277	0.219862	0.498378	8.958756	2.62E-05	0.001243	3.137947	0.388447	0.608309	-0.21986	19	33137590	F	II	ANKRD27	Body	opensea	Body-opens		rs1130307	1;25;39
cg136260C	0.219955	0.448498	8.958636	2.62E-05	0.001243	3.137849	0.33852	0.558475	-0.21995	11	16177409	R	II	SOX6	Body	opensea	Body-opens			
cg2290299	0.230465	0.643794	8.958379	2.62E-05	0.001243	3.137639	0.528562	0.759027	-0.23046	2	70629122	R	II		IGR	opensea	IGR-opense			
cg1235111	0.227899	0.679074	8.958088	2.62E-05	0.001243	3.137402	0.565124	0.793023	-0.2279	4	74853485	F	II	PPBP	Body	opensea	Body-opens		rs5631200	0;1
cg2145658	0.231037	0.618799	8.957957	2.62E-05	0.001243	3.137295	0.50328	0.734317	-0.23104	8	65828722	F	II		IGR	opensea	IGR-opense		rs7823966	9
cg0742967	0.220135	0.630164	8.956337	2.62E-05	0.001244	3.135973	0.520097	0.740232	-0.22014	4	1.82E+08	R	II		IGR	opensea	IGR-opense		rs1169853	10
cg0347704	0.205144	0.541446	8.955953	2.63E-05	0.001244	3.135659	0.438874	0.644018	-0.20514	5	1.26E+08	F	II		IGR	opensea	IGR-opense			
cg1231687	0.224729	0.549266	8.955915	2.63E-05	0.001244	3.135628	0.436902	0.661631	-0.22473	1	71356710	R	II	PTGER3	3'UTR	opensea	3'UTR-oper		rs4147114	47;20;2
cg0972073	0.247624	0.523291	8.955884	2.63E-05	0.001244	3.135602	0.399479	0.647103	-0.24762	10	1.29E+08	R	II	DOCK1	ExonBnd	opensea	ExonBnd-o		rs1880210	17
cg0077666	0.205871	0.514666	8.955541	2.63E-05	0.001244	3.135323	0.41173	0.617601	-0.20587	2	1.08E+08	R	II		IGR	opensea	IGR-opense		rs5407897	27
cg1760405	0.20869	0.584212	8.954851	2.63E-05	0.001245	3.134759	0.479867	0.688557	-0.20869	2	2.39E+08	R	II	FAM132B	TSS1500	shore	TSS1500-sh	chr2:2390	rs1512462	25;2
cg1915617	0.200492	0.332888	8.954431	2.63E-05	0.001245	3.134416	0.232643	0.433134	-0.20049	17	70131300	R	II		IGR	opensea	IGR-opense		rs5419783	39;29;2
cg0452039	0.208382	0.423467	8.953329	2.63E-05	0.001245	3.133516	0.319276	0.527658	-0.20838	5	1E+08	R	II	ST8SIA4	Body	opensea	Body-opens		rs1385491	40
cg0540771	0.204565	0.566957	8.953275	2.63E-05	0.001245	3.133472	0.464674	0.669239	-0.20456	11	45722308	F	II		IGR	opensea	IGR-opense			
cg225407C	0.231676	0.435447	8.952756	2.63E-05	0.001245	3.133049	0.319609	0.551285	-0.23168	10	1.09E+08	R	II	SORCS1	Body	opensea	Body-opens		rs1878637	6;1
cg1354236	0.206603	0.544925	8.952707	2.63E-05	0.001245	3.133009	0.441624	0.648227	-0.2066	10	95049539	R	II		IGR	opensea	IGR-opense		rs5583929	49;34;12;6;2
cg2582767	0.257233	0.521276	8.951076	2.64E-05	0.001246	3.131676	0.392659	0.649893	-0.25723	17	15255513	F	II		IGR	opensea	IGR-opense		rs5677320	37;41
cg0382398	0.212837	0.354048	8.94908	2.64E-05	0.001247	3.130045	0.24763	0.460467	-0.21284	9	1E+08	R	II	TMOD1	Body	opensea	Body-opens		rs1455096	41;40;2
cg0976728	0.236182	0.617234	8.949002	2.64E-05	0.001247	3.129982	0.499143	0.735325	-0.23618	8	1.31E+08	R	II	CCDC26	TSS1500	opensea	TSS1500-o		rs5707536	41;2
cg1041784	0.218322	0.585565	8.944019	2.65E-05	0.001249	3.12591	0.476405	0.694726	-0.21832	4	74375460	F	II	LOC72804	ExonBnd	opensea	ExonBnd-o		rs1093807	13;26
cg1494855	0.226906	0.573055	8.942785	2.65E-05	0.00125	3.124901	0.459602	0.686508	-0.22691	4	1.24E+08	R	II	IL21	TSS1500	opensea	TSS1500-o		rs5512936	2;1
cg2287947	0.21644	0.637475	8.942106	2.65E-05	0.00125	3.124345	0.529255	0.745695	-0.21644	21	39114939	R	II	KCNJ6	Body	opensea	Body-opens		rs5708659	48;40;2
cg0069124	0.204701	0.615763	8.940248	2.66E-05	0.001251	3.122826	0.513413	0.718113	-0.2047	5	36458165	R	II		IGR	opensea	IGR-opense		rs1886378	50;42;32
cg0077966	0.204124	0.685629	8.939391	2.66E-05	0.001252	3.122125	0.583566	0.787691	-0.20412	4	1.42E+08	F	II	RNF150	Body	opensea	Body-opens		rs5329888	31;48
cg1169219	0.234771	0.632098	8.939252	2.66E-05	0.001252	3.122012	0.514712	0.749484	-0.23477	17	53639752	R	II		IGR	shore	IGR-shore	chr17:536	rs1911408	38;1
cg1775789	0.242088	0.505041	8.937834	2.66E-05	0.001253	3.120852	0.383997	0.626085	-0.24209	2	1.45E+08	F	II	ZEB2	Body	opensea	Body-opens		rs5428056	27
cg0763343	0.205747	0.563507	8.937288	2.66E-05	0.001253	3.120405	0.460634	0.666381	-0.20575	5	1.53E+08	F	II	GRIA1	TSS1500	opensea	TSS1500-o			
cg1431632	0.200659	0.57386	8.936548	2.67E-05	0.001253	3.1198	0.47353	0.674189	-0.20066	5	1.24E+08	F	II		IGR	opensea	IGR-opense			
cg1076455	0.224286	0.525675	8.935559	2.67E-05	0.001254	3.118991	0.413532	0.637818	-0.22429	11	12419531	R	II	PARVA	Body	opensea	Body-opens		rs1818671	50;44
cg0279154	0.215604	0.598236	8.934184	2.67E-05	0.001255	3.117866	0.490434	0.706039	-0.2156	3	31777556	F	II	OSBPL10	Body	opensea	Body-opens		rs1161721	36;43;47
cg2607931	0.222019	0.536848	8.933301	2.67E-05	0.001255	3.117143	0.425839	0.647857	-0.22202	4	1.39E+08	F	II	LINC00499	Body	opensea	Body-opens		rs5690028	29;39
cg0275174	0.211678	0.562503	8.933203	2.67E-05	0.001255	3.117063	0.456664	0.668342	-0.21168	6	52532536	R	II	LOC73010	Body	shelf	Body-shelf	chr6:5252		
cg1122425	0.211318	0.502462	8.933134	2.67E-05	0.001255	3.117006	0.396803	0.608121	-0.21132	12	6335277	F	II	CD9	Body	opensea	Body-opens		rs5636211	8
cg258186C	0.213325	0.547317	8.93293	2.67E-05	0.001255	3.11684	0.440654	0.653979	-0.21333	2	1.01E+08	R	II		IGR	opensea	IGR-opense		rs1379590	51
cg2400671	0.209581	0.585626	8.932647	2.67E-05	0.001255	3.116608	0.480836	0.690417	-0.20958	3	13568212	F	II		IGR	opensea	IGR-opense		rs5347239	0;37
cg0163323	0.213838	0.5073	8.93214	2.68E-05	0.001255	3.116193	0.400381	0.614219	-0.21384	2	2.17E+08	R	II	XRCC5	Body	opensea	Body-opens		rs5779289	12
cg0643387	0.214122	0.547529	8.932017	2.68E-05	0.001255	3.116092	0.440468	0.65459	-0.21412	9	26065861	R	II	LOC10050	TSS1500	opensea	TSS1500-o		rs5405646	26;24;15;1
cg050186C	0.20409	0.609501	8.931561	2.68E-05	0.001255	3.115719	0.507456	0.711546	-0.20409	9	71460554	F	II	PIP5K1B	Body	opensea	Body-opens		rs5754598	1
cg247780C	0.212301	0.669554	8.931198	2.68E-05	0.001256	3.115422	0.563404	0.775704	-0.2123	2	1.21E+08	F	II		IGR	opensea	IGR-opense		rs1339259	8;45;48
cg0208665	0.250634	0.522727	8.930206	2.68E-05	0.001256	3.114609	0.39741	0.648044	-0.25063	1	56756750	R	II		IGR	opensea	IGR-opense		rs1407779	1
cg0685877	0.203953	0.660172	8.929755	2.68E-05	0.001256	3.114241	0.558195	0.762148	-0.20395	13	72364593	F	II	DACH1	Body	opensea	Body-opens			
cg1376751	0.208241	0.383226	8.929072	2.68E-05	0.001257	3.113681	0.279105	0.487346	-0.20824	3	1.73E+08	R	II	SPATA16	Body	opensea	Body-opens			
cg252156C	0.21205	0.542874	8.928805	2.68E-05	0.001257	3.113463	0.436849	0.648899	-0.21205	10	44820772	F	II		IGR	opensea	IGR-opense		rs7956674	25
cg0825073	0.23351	0.643226	8.927676	2.68E-05	0.001257	3.112538	0.526471	0.759981	-0.23351	5	73772012	R	II		IGR	opensea	IGR-opense		rs5729064	28;23;14;8;1
cg1447432	0.220402	0.485515	8.92669	2.69E-05	0.001257	3.11173	0.375314	0.595717	-0.2204	18	71303654	F	II		IGR	opensea	IGR-opense		rs1473913	16
cg0063529	0.209949	0.629034	8.925976	2.69E-05	0.001257	3.111146	0.52406	0.734009	-0.20995	1	68850147	F	II		IGR	opensea	IGR-opense		rs5738757	14
cg1280232	0.22494	0.578828	8.924988	2.69E-05	0.001257	3.110336	0.466358	0.691298	-0.22494	18	21032446	R	II	RIOK3	TSS1500	shore	TSS1500-sh	chr18:210	rs1396406	1
cg141597C	0.21073	0.569945	8.924278	2.69E-05	0.001258	3.109756	0.46458	0.67531	-0.21073	9	89580839	R	II		IGR	opensea	IGR-opense			
cg1554257	0.23503	0.465474	8.924195	2.69E-05	0.001258	3.109687	0.347959	0.582989	-0.23503	6	1.51E+08	R	II		IGR	opensea	IGR-opense			
cg087568C	0.218947	0.557591	8.924166	2.69E-05	0.001258	3.109663	0.448117	0.667064	-0.21895	15	38087461	F	II		IGR	opensea	IGR-opense		rs5555044	1;29;32;51
cg2611047	0.207583	0.53431	8.923817	2.69E-05	0.001258	3.109378	0.430519	0.638102	-0.20758	11	32054712	R	II		IGR	opensea	IGR-opense		rs1925007	51;1
cg2279533	0.230163	0.512543	8.923557	2.69E-05	0.001258	3.109164	0.397462	0.627624	-0.23016	6	1.18E+08	F	II		IGR	opensea	IGR-opense			
cg1985875	0.200889	0.446285	8.923286	2.69E-05	0.001258	3.108943	0.34584	0.546729	-0.20089	5	1.56E+08	F	II	SGCD	Body	opensea	Body-opens		rs5710046	0;38;39;49
cg1459752	0.208101	0.526828	8.922042	2.7E-05	0.001259	3.107923	0.422777	0.630878	-0.2081	6	1.67E+08	F	II	LOC10192	Body	opensea	Body-opens		rs1918786	1;20;34
cg2750929	0.228464	0.586767	8.921805	2.7E-05	0.001259	3.107729	0.472535	0.700999	-0.22846	6	14919597	R	II		IGR	opensea	IGR-opense		rs1812149	46;45;2
cg1148391	0.228087	0.466695	8.918218	2.7E-05	0.001261	3.104789	0.352651	0.580738	-0.22809	3	58860839	F	II	C3orf67	Body	opensea	Body-opens		rs1163807	1
cg1160235	0.226016	0.740188	8.917882	2.71E-05	0.001261	3.104514	0.62718	0.853196	-0.22602	1	78137696	F	II	ZZZ3	5'UTR	opensea	5'UTR-oper		rs1133918	0;18
cg1230517	0.206062	0.580047	8.917165	2.71E-05	0.001261	3.103926	0.477016	0.683078	-0.20606	16	11119093	R	II	CLEC16A	Body	opensea	Body-opens		rs1903964	12;1
cg0079539	0.229507	0.580987	8.916142	2.71E-05	0.001262	3.103087	0.466234	0.69574	-0.22951	14	87113901	F	II		IGR	opensea	IGR-opense		rs5332678	8
cg2429475	0.249627	0.536286	8.915234	2.71E-05	0.001262	3.102343	0.411472	0.661099	-0.24963	12	46028329	F	II		IGR	opensea	IGR-opense		rs3744955	1;19
cg0862397	0.205676	0.547963	8.914853	2.71E-05	0.001262	3.10203	0.445124	0.650801	-0.20568	1	36936639	R	II	CSF3R	Body	opensea	Body-opens		rs5543295	51;40;20;3;2;1
cg0198853	0.219707	0.584334	8.911703	2.72E-05	0.001263	3.099447	0.47448	0.694187	-0.21971	8	1.15E+08	R	II		IGR	opensea	IGR-opense		rs5379764	41;2;1
cg1018169	0.218723	0.655596	8.910788	2.72E-05	0.001264	3.098696	0.546234	0.764958	-0.21872	12	58187866	R	II	TSFM	Body	opensea	Body-opens			
cg2450428	0.202092	0.653506	8.910032	2.72E-05	0.001264	3.098076	0.55246	0.754552	-0.20209	6	32061478	F	II	TNXB	Body	shelf	Body-shelf	chr6:3206	rs5666083	51
cg2539628	0.203117	0.345806	8.90892	2.72E-05	0.001264	3.097164	0.244248	0.447365	-0.20312	7	17301295	R	II		IGR	opensea	IGR-opense		rs5543470	48;10;4
cg1474745	0.211823	0.41395	8.908852	2.72E-05	0.001264	3.097108	0.308039	0.519862	-0.21182	1	40063592	R	II		IGR	opensea	IGR-opense		rs5500148	19;1
cg0716158	0.215109	0.571839	8.905787	2.73E-05	0.001266	3.094592	0.464285	0.679394	-0.21511	3	12779524	F	II	TMEM40	Body	opensea	Body-opens			
cg1696783	0.209518	0.642733	8.905264	2.73E-05	0.001267	3.094164	0.537974	0.747492	-0.20952	17	79467055	R	II		IGR	shore	IGR-shore	chr17:794	rs1176820	50;7
cg1625865	0.282708	0.509754	8.904259	2.73E-05	0.001267	3.093338	0.368401	0.651108	-0.28271	3	29338542	F	II	RBMS3	Body	opensea	Body-opens		rs8023379	0;27;51
cg0284788	0.220097	0.690616	8.903726	2.74E-05	0.001267	3.092901	0.580567	0.800664	-0.2201	10	13802920	F	II	FRMD4A	Body	opensea	Body-opens		rs5559958	30
cg1289585	0.201558	0.645298	8.903366	2.74E-05	0.001267	3.092605	0.544519	0.746077	-0.20156	6	1.4E+08	F	II		IGR	opensea	IGR-opense		rs1405407	27
cg2683801	0.220859	0.574133	8.902985	2.74E-05	0.001268	3.092292	0.463703	0.684562	-0.22086	4	78072602	F	II		IGR	opensea	IGR-opense		rs5583227	5;7;40
cg0757275	0.241826	0.525543	8.902925	2.74E-05	0.001268	3.092244	0.40463	0.646456	-0.24183	2	1.58E+08	R	II		IGR	opensea	IGR-opense		rs5331996	1
cg1901755	0.206188	0.59165	8.901829	2.74E-05	0.001268	3.091343	0.488556	0.694744	-0.20619	10	34817409	R	II	PARD3	Body	opensea	Body-opens		rs1826736	50
cg1243665	0.2119	0.564624	8.901485	2.74E-05	0.001269	3.091061	0.458674	0.670574	-0.2119	18	20787494	R	II	CABLES1	Body	opensea	Body-opens		rs1509924	49;39;35;18;8
cg254910€	0.222776	0.534944	8.901383	2.74E-05	0.001269	3.090977	0.423556	0.646332	-0.22278	15	58328952	F	II	ALDH1A2	Body	opensea	Body-opens		rs5609434	37
cg240288C	0.22964	0.45562	8.901104	2.74E-05	0.001269	3.090748	0.340801	0.57044	-0.22964	6	1.53E+08	R	II	RGS17	5'UTR	opensea	5'UTR-oper		rs5282674	47
cg1614551	0.22622	0.543671	8.900475	2.74E-05	0.001269	3.090232	0.430561	0.656781	-0.22622	3	72772498	F	II		IGR	opensea	IGR-opense		rs5587456	1;29;47
cg0371423	0.218237	0.629358	8.898309	2.75E-05	0.00127	3.088452	0.52024	0.738477	-0.21824	1	88066830	R	II		IGR	opensea	IGR-opense		rs1412040	40
cg1100785	0.205527	0.662497	8.897334	2.75E-05	0.00127	3.087651	0.559734	0.765261	-0.20553	2	1.29E+08	F	II		IGR	opensea	IGR-opense		rs5543523	40
cg1579117	0.204733	0.55327	8.896861	2.75E-05	0.00127	3.087262	0.450903	0.655636	-0.20473	1	2.19E+08	R	II	MIR548F3	Body	opensea	Body-opens		rs1899741	9
cg1249178	0.203866	0.657691	8.895499	2.75E-05	0.001271	3.086143	0.555758	0.759624	-0.20387	3	1.86E+08	R	II	FETUB	5'UTR	opensea	5'UTR-oper		rs5741689	43
cg0277746	0.211034	0.415171	8.895359	2.75E-05	0.001271	3.086028	0.309654	0.520688	-0.21103	13	63801462	F	II	LINC00376	Body	opensea	Body-opens			
cg1447552	0.218352	0.558245	8.895231	2.75E-05	0.001271	3.085923	0.44907	0.667421	-0.21835	8	43046589	R	II	HGSNAT	Body	opensea	Body-opens		rs1812906	27
cg2104582	0.200382	0.667942	8.894859	2.75E-05	0.001271	3.085618	0.567751	0.768133	-0.20038	8	89310065	F	II	MMP16	Body	opensea	Body-opens		rs1491187	0;27
cg0522945	0.213662	0.61293	8.894699	2.76E-05	0.001271	3.085486	0.506099	0.71976	-0.21366	12	7985739	F	II	SLC2A14	Body	opensea	Body-opens		rs2376906	12;13
cg2594725	0.247746	0.6135	8.894427	2.76E-05	0.001271	3.085263	0.489626	0.737373	-0.24775	20	9288423	R	II	PLCB4	TSS200	opensea	TSS200-ope			
cg085443C	0.203908	0.57178	8.893027	2.76E-05	0.001272	3.084112	0.469826	0.673734	-0.20391	8	28725934	R	II	INTS9	5'UTR	opensea	5'UTR-oper		rs1814370	13;1
cg2157979	0.236169	0.63853	8.89244	2.76E-05	0.001272	3.08363	0.520446	0.756614	-0.23617	8	18577100	R	II	PSD3	Body	opensea	Body-opens		rs7768848	39;2
cg0205874	0.212759	0.394654	8.889914	2.77E-05	0.001274	3.081553	0.288275	0.501034	-0.21276	1	94724340	R	II		IGR	opensea	IGR-opense		rs1146860	8
cg0363282	0.2041	0.557437	8.889069	2.77E-05	0.001274	3.080858	0.455387	0.659487	-0.2041	4	1.7E+08	R	II	PALLD	Body	opensea	Body-opens		rs5746345	48;40
cg2468691	0.211074	0.469985	8.888605	2.77E-05	0.001274	3.080476	0.364448	0.575522	-0.21107	1	2.22E+08	F	II		IGR	opensea	IGR-opense		rs5553421	14
cg2082118	0.216873	0.517905	8.887668	2.77E-05	0.001274	3.079706	0.409469	0.626342	-0.21687	1	2E+08	R	II		IGR	opensea	IGR-opense		rs1898721	1
cg1258058	0.202561	0.62551	8.887078	2.77E-05	0.001275	3.079221	0.524229	0.72679	-0.20256	12	13759540	F	II	GRIN2B	Body	opensea	Body-opens		rs1231359	15;37;47;48
cg1276764	0.201038	0.407501	8.886962	2.77E-05	0.001275	3.079125	0.306982	0.50802	-0.20104	18	62295302	F	II		IGR	opensea	IGR-opense		rs5606910	0;1;20
cg0602383	0.242383	0.495896	8.886523	2.77E-05	0.001275	3.078765	0.374704	0.617088	-0.24238	10	71273679	F	II		IGR	opensea	IGR-opense		rs5513129	0;17
cg2653094	0.2298	0.409441	8.885946	2.77E-05	0.001275	3.07829	0.294541	0.524341	-0.2298	2	2.32E+08	F	II	ARMC9	Body	shelf	Body-shelf	chr2:2321		
cg1976181	0.240329	0.416937	8.885446	2.78E-05	0.001275	3.077878	0.296772	0.537101	-0.24033	18	12255836	R	II	CIDEA	Body	shore	Body-shore	chr18:122		
cg2014617	0.251087	0.516163	8.885324	2.78E-05	0.001275	3.077778	0.39062	0.641707	-0.25109	8	1.23E+08	R	II	HAS2	Body	opensea	Body-opens		rs5386940	37;22;17;11

cg0176771	0.285601	0.46932	8.885234	2.78E-05	0.001275	3.077704	0.32652	0.612121	-0.2856	6	1.41E+08	F	II		IGR	opensea	IGR-opense			
cg1399560	0.228573	0.532187	8.885147	2.78E-05	0.001275	3.077633	0.417901	0.646473	-0.22857	2	1.47E+08	R	II		IGR	opensea	IGR-opense		rs1841940	41;27;10;7
cg1458109	0.2253	0.604509	8.883842	2.78E-05	0.001276	3.076559	0.491859	0.71716	-0.2253	13	52520116	R	II	ATP7B	Body	opensea	Body-opens		rs5353968	46
cg0335448	0.210104	0.591769	8.88258	2.78E-05	0.001277	3.07552	0.486717	0.696821	-0.2101	6	20039211	F	II		IGR	opensea	IGR-opense		rs5531747	0;30
cg0791467	0.212246	0.642149	8.882508	2.78E-05	0.001277	3.075461	0.536026	0.748272	-0.21225	1	1.65E+08	F	II	LOC40079	Body	opensea	Body-opens			
cg1255099	0.207338	0.603838	8.882308	2.78E-05	0.001277	3.075297	0.500169	0.707507	-0.20734	8	55318116	F	II		IGR	opensea	IGR-opense		rs5421168	26;45;47
cg1731113	0.213718	0.356832	8.881477	2.78E-05	0.001277	3.074613	0.249973	0.463691	-0.21372	12	88693546	R	II		IGR	opensea	IGR-opense		rs5493641	36;2;1
cg0688937	0.211556	0.680272	8.879829	2.79E-05	0.001278	3.073257	0.574494	0.78605	-0.21156	4	1.02E+08	R	II	PPP3CA	Body	opensea	Body-opens		rs5703326	1
cg0379385	0.255319	0.514995	8.879401	2.79E-05	0.001278	3.072904	0.387336	0.642655	-0.25532	13	76867659	F	II		IGR	opensea	IGR-opense		rs5769345	5;8;37
cg0150020	0.238177	0.580017	8.879196	2.79E-05	0.001278	3.072736	0.460928	0.699105	-0.23818	10	13144314	R	II	OPTN	5'UTR	shore	5'UTR-shor	chr10:131	rs478911;r	35;27;26
cg0022245	0.218501	0.482506	8.879127	2.79E-05	0.001278	3.072679	0.373255	0.591756	-0.2185	5	1.24E+08	R	II	LOC10192	Body	opensea	Body-opens		rs5644652	14
cg2464725	0.207329	0.561006	8.877585	2.79E-05	0.001279	3.071409	0.457341	0.664671	-0.20733	7	94997769	F	II	PON3	Body	opensea	Body-opens		rs5822130	48
cg2054701	0.27367	0.491091	8.877027	2.79E-05	0.001279	3.07095	0.354256	0.627926	-0.27367	12	1.11E+08	R	II	PPP1CC	Body	opensea	Body-opens		rs5500907	44;32;25;20
cg1574120	0.20314	0.683636	8.875598	2.8E-05	0.001279	3.069773	0.582066	0.785206	-0.20314	8	1.08E+08	R	II		IGR	opensea	IGR-opense			
cg0070468	0.224229	0.611609	8.875428	2.8E-05	0.001279	3.069633	0.499495	0.723724	-0.22423	19	30115207	R	II		IGR	opensea	IGR-opense		rs1414310	36;24;21;5;4;1
cg0640457	0.210763	0.533046	8.875375	2.8E-05	0.001279	3.069589	0.427665	0.638428	-0.21076	8	1.31E+08	F	II	ASAP1	Body	shelf	Body-shelf	chr8:1313	rs1441805	1
cg1296954	0.214654	0.584259	8.875051	2.8E-05	0.00128	3.069323	0.476932	0.691586	-0.21465	2	35695553	R	II		IGR	opensea	IGR-opense		rs5720417	45;43;2
cg0285467	0.210518	0.700764	8.874416	2.8E-05	0.00128	3.0688	0.595506	0.806023	-0.21052	8	21000500	R	II		IGR	opensea	IGR-opense		rs1884682	49;29;19;7
cg1232649	0.232267	0.52476	8.874077	2.8E-05	0.00128	3.068521	0.408626	0.640894	-0.23227	4	91549443	F	II	CCSER1	Body	opensea	Body-opens		rs5708374	35;44
cg0845233	0.204555	0.558423	8.873921	2.8E-05	0.00128	3.068392	0.456145	0.6607	-0.20455	2	1.42E+08	F	II	LRP1B	Body	opensea	Body-opens		rs5724573	8;19;29;31;36
cg0488514	0.249272	0.633091	8.873122	2.8E-05	0.00128	3.067734	0.508455	0.757727	-0.24927	9	1.1E+08	R	II	MIR548Q	Body	opensea	Body-opens		rs5409613	2
cg0633283	0.201711	0.520346	8.873084	2.8E-05	0.00128	3.067703	0.41949	0.621201	-0.20171	3	13941267	F	II		IGR	shelf	IGR-shelf	chr3:1393	rs5485284	21;37
cg0881731	0.23155	0.587243	8.872647	2.8E-05	0.00128	3.067343	0.471468	0.703019	-0.23155	20	31903641	R	II		IGR	opensea	IGR-opense		rs1455251	22;1
cg1819590	0.223405	0.573562	8.872215	2.8E-05	0.00128	3.066987	0.461859	0.685264	-0.2234	2	1.49E+08	F	II		IGR	opensea	IGR-opense		rs1902997	0;8
cg0722341	0.2368	0.520734	8.872203	2.8E-05	0.00128	3.066977	0.402334	0.639134	-0.2368	2	28668934	F	II		IGR	opensea	IGR-opense		rs6752004	15
cg0047939	0.207542	0.643413	8.871674	2.81E-05	0.00128	3.066541	0.539642	0.747184	-0.20754	9	1E+08	R	II	LOC10049	Body	opensea	Body-opens		rs5691454	43
cg0057419	0.210758	0.545769	8.871029	2.81E-05	0.001281	3.06601	0.44039	0.651148	-0.21076	1	22137992	F	II	LDLRAD2	TSS1500	shelf	TSS1500-sh	chr1:2214	rs5570268	0;13;30;47
cg2614402	0.267635	0.525238	8.870804	2.81E-05	0.001281	3.065825	0.39142	0.659055	-0.26764	3	69047209	F	II	EOGT	Body	opensea	Body-opens		rs1473270	1;30
cg1060029	0.245766	0.44257	8.870226	2.81E-05	0.001281	3.065349	0.319687	0.565453	-0.24577	20	1789945	F	II		IGR	opensea	IGR-opense		rs5665149	38
cg0550700	0.230571	0.627114	8.870165	2.81E-05	0.001281	3.065298	0.511828	0.742399	-0.23057	5	68728606	R	II	MARVELD2	Body	opensea	Body-opens			
cg0363414	0.226611	0.535525	8.870085	2.81E-05	0.001281	3.065232	0.422219	0.64883	-0.22661	6	484044	R	II		IGR	opensea	IGR-opense		rs1869715	1
cg1519364	0.231866	0.606034	8.869806	2.81E-05	0.001281	3.065002	0.490102	0.721967	-0.23187	3	1.5E+08	R	II	ANKUB1	Body	opensea	Body-opens		rs3750897	49;28;2;1
cg1884349	0.204444	0.556414	8.868233	2.81E-05	0.001281	3.063706	0.454192	0.658636	-0.20444	3	1.29E+08	R	II	H1FOO	TSS1500	opensea	TSS1500-op		rs1820435	9;8
cg0808681	0.215844	0.618216	8.867384	2.82E-05	0.001282	3.063007	0.510294	0.726138	-0.21584	6	1.3E+08	R	II	TMEM244	TSS1500	opensea	TSS1500-op		rs1118835	38;36;23;18;2;1
cg2302587	0.222139	0.588025	8.866941	2.82E-05	0.001282	3.062641	0.476956	0.699094	-0.22214	7	77487860	F	II	PHTF2	Body	opensea	Body-opens		rs5363026	41;41
cg1052339	0.220567	0.463282	8.866563	2.82E-05	0.001282	3.06233	0.352999	0.573565	-0.22057	10	12606492	F	II	CAMK1D	Body	opensea	Body-opens			
cg0700190	0.21564	0.658393	8.866493	2.82E-05	0.001282	3.062272	0.550573	0.766213	-0.21564	20	12160957	F	II		IGR	opensea	IGR-opense		rs5386042	33
cg1012413	0.213146	0.513175	8.864679	2.82E-05	0.001283	3.060777	0.406602	0.619749	-0.21315	6	1.38E+08	F	II		IGR	opensea	IGR-opense			
cg0284191	0.265237	0.442454	8.864243	2.82E-05	0.001283	3.060417	0.309836	0.575073	-0.26524	6	1.53E+08	R	II	SYNE1	5'UTR	shore	5'UTR-shor	chr6:1529	rs5615733	22;19;2;1
cg0205002	0.221366	0.563308	8.864146	2.82E-05	0.001283	3.060337	0.452625	0.673991	-0.22137	7	43156037	R	II	HECW1	5'UTR	shelf	5'UTR-shelf	chr7:4315	rs5330254	34
cg1090730	0.265573	0.663611	8.863886	2.82E-05	0.001283	3.060123	0.530825	0.796398	-0.26557	11	71852326	R	II		IGR	opensea	IGR-opense		rs5572563	34;8
cg0288154	0.208853	0.595083	8.862846	2.83E-05	0.001284	3.059265	0.490656	0.69951	-0.20885	4	1.7E+08	R	II	SH3RF1	Body	opensea	Body-opens			
cg1386118	0.211529	0.540248	8.860993	2.83E-05	0.001284	3.057738	0.434484	0.646013	-0.21153	9	1.4E+08	F	II	GRIN1	Body	shore	Body-shore	chr9:1400	rs1439268	33
cg2450930	0.215363	0.612013	8.86067	2.83E-05	0.001284	3.057471	0.504331	0.719695	-0.21536	2	1.7E+08	R	II		IGR	opensea	IGR-opense			
cg0267332	0.209788	0.673797	8.860367	2.83E-05	0.001284	3.057221	0.568903	0.778691	-0.20979	6	8087102	R	II	EEF1E1	Body	opensea	Body-opens		rs5276477	16;7;2
cg1095405	0.233232	0.625129	8.860149	2.83E-05	0.001284	3.057041	0.508513	0.741745	-0.23323	15	94425363	R	II		IGR	opensea	IGR-opense		rs7498408	28;13;1
cg0526102	0.202714	0.619531	8.860004	2.83E-05	0.001284	3.056921	0.518174	0.720887	-0.20271	10	55577995	R	II	PCDH15	Body	opensea	Body-opens		rs1159085	36;5
cg1685850	0.219775	0.624597	8.858534	2.83E-05	0.001285	3.055709	0.514709	0.734484	-0.21978	11	88347154	F	II	GRM5	Body	opensea	Body-opens		rs5370141	0;15;23;44
cg1223246	0.218389	0.546932	8.858202	2.84E-05	0.001285	3.055435	0.437737	0.656127	-0.21839	2	1.01E+08	F	II	LONRF2	Body	opensea	Body-opens		rs5711136	0;1;38
cg2234506	0.200957	0.773136	8.857108	2.84E-05	0.001286	3.054533	0.672657	0.873614	-0.20096	5	6795634	R	II		IGR	opensea	IGR-opense		rs5626137	29;8
cg0368009	0.202001	0.562704	8.856384	2.84E-05	0.001286	3.053935	0.461704	0.663705	-0.202	6	40774214	F	II		IGR	opensea	IGR-opense			
cg1824717	0.22271	0.555609	8.856116	2.84E-05	0.001286	3.053714	0.444253	0.666964	-0.22271	22	19436867	F	II	C22orf39	TSS1500	shore	TSS1500-sh	chr22:194	rs5413501	10
cg1517673	0.220157	0.577878	8.856043	2.84E-05	0.001286	3.053653	0.467799	0.687956	-0.22016	13	29078048	R	II		IGR	opensea	IGR-opense		rs5476535	40;4
cg0460421	0.200181	0.530376	8.855543	2.84E-05	0.001286	3.053241	0.430285	0.630466	-0.20018	8	1.07E+08	F	II	ZFPM2	Body	opensea	Body-opens		rs1443182	0;20;29;30
cg1483785	0.226487	0.425687	8.855299	2.84E-05	0.001286	3.05304	0.312444	0.538931	-0.22649	12	54916439	F	II	NCKAP1L	Body	opensea	Body-opens		rs1115973	19;35;49
cg1492413	0.247358	0.649454	8.853063	2.85E-05	0.001287	3.051194	0.525775	0.773133	-0.24736	13	74207962	R	II		IGR	opensea	IGR-opense		rs1829570	42;22
cg1409115	0.235944	0.548645	8.8528	2.85E-05	0.001287	3.050977	0.430673	0.666617	-0.23594	4	79358764	F	II	FRAS1	Body	opensea	Body-opens		rs5445028	33;44
cg0595946	0.203841	0.524114	8.852728	2.85E-05	0.001287	3.050918	0.422193	0.626034	-0.20384	8	70064456	R	II		IGR	opensea	IGR-opense		rs1899346	45;8
cg0844914	0.213894	0.586625	8.852216	2.85E-05	0.001288	3.050495	0.479678	0.693571	-0.21389	11	1857919	F	II	SYT8	Body	opensea	Body-opens		rs1466231	0;11;17;31;38
cg0242318	0.236189	0.529443	8.851763	2.85E-05	0.001288	3.050121	0.411348	0.647537	-0.23619	13	37337525	F	II		IGR	opensea	IGR-opense		rs5448666	16
cg0212342	0.268649	0.411737	8.851012	2.85E-05	0.001288	3.049501	0.277413	0.546062	-0.26865	8	74433128	R	II	STAU2	Body	opensea	Body-opens		rs5459806	1
cg1376036	0.211138	0.48541	8.850918	2.85E-05	0.001288	3.049424	0.379841	0.59098	-0.21114	15	80104644	R	II		IGR	opensea	IGR-opense		rs5743151	51
cg1530235	0.202194	0.657939	8.850648	2.85E-05	0.001288	3.0492	0.556842	0.759036	-0.20219	11	68180760	R	II	LRP5	Body	shore	Body-shore	chr11:681	rs5716977	33;26
cg2103874	0.208286	0.517145	8.849321	2.86E-05	0.001288	3.048104	0.413002	0.621288	-0.20829	2	1.55E+08	R	II		IGR	opensea	IGR-opense			
cg0895051	0.273652	0.564719	8.84915	2.86E-05	0.001288	3.047963	0.427893	0.701545	-0.27365	2	1.35E+08	R	II		IGR	opensea	IGR-opense		rs5540531	38;32;3
cg2445182	0.205293	0.497854	8.847972	2.86E-05	0.001288	3.046991	0.395207	0.6005	-0.20529	2	2.21E+08	F	II		IGR	opensea	IGR-opense		rs1461057	37
cg1589963	0.203913	0.634445	8.847676	2.86E-05	0.001288	3.046746	0.532489	0.736402	-0.20391	3	1.55E+08	R	II		IGR	opensea	IGR-opense		rs5704529	36;23;12;1
cg0365743	0.210663	0.5056	8.847509	2.86E-05	0.001288	3.046608	0.400269	0.610932	-0.21066	6	1.47E+08	F	II	STXBP5-AS	Body	opensea	Body-opens		rs1160426	8
cg2691763	0.215596	0.405817	8.84707	2.86E-05	0.001289	3.046246	0.298019	0.513615	-0.2156	3	1.84E+08	F	II		IGR	opensea	IGR-opense		rs7944696	1
cg1671069	0.233541	0.617347	8.846387	2.86E-05	0.001289	3.045681	0.500576	0.734118	-0.23354	11	60101357	R	II	MS4A6E	TSS1500	opensea	TSS1500-op			
cg2704945	0.219276	0.531899	8.845682	2.86E-05	0.001289	3.045099	0.42226	0.641537	-0.21928	7	37744254	F	II		IGR	opensea	IGR-opense		rs1915094	10;27
cg1767164	0.201852	0.577334	8.843912	2.87E-05	0.00129	3.043637	0.476409	0.67826	-0.20185	15	27706516	R	II	GABRG3	Body	opensea	Body-opens			
cg2317934	0.215176	0.693728	8.84381	2.87E-05	0.00129	3.043553	0.58614	0.801316	-0.21518	4	1E+08	F	II	ADH7	TSS1500	opensea	TSS1500-op		rs1831506	1;2;8
cg0618082	0.241017	0.432932	8.84218	2.87E-05	0.001291	3.042205	0.312424	0.553441	-0.24102	8	48812543	F	II	PRKDC	Body	opensea	Body-opens		rs1459568	0;6
cg2734259	0.222502	0.577271	8.841968	2.87E-05	0.001291	3.04203	0.46602	0.688522	-0.2225	5	1.24E+08	R	II		IGR	opensea	IGR-opense		rs1146037	1
cg0564701	0.215793	0.596289	8.840893	2.87E-05	0.001291	3.041142	0.488392	0.704186	-0.21579	11	1.25E+08	R	II	HEPACAM	TSS1500	opensea	TSS1500-op		rs5409619	1;9;13;23;24
cg1336341	0.203197	0.566702	8.840677	2.88E-05	0.001292	3.040964	0.465104	0.6683	-0.2032	11	1.31E+08	R	II		IGR	opensea	IGR-opense		rs5356158	1
cg2411852	0.224852	0.552426	8.840535	2.88E-05	0.001292	3.040846	0.44	0.664852	-0.22485	7	90397517	F	II	CDK14	Body	opensea	Body-opens		rs1848588	0;31
cg1567876	0.216499	0.618565	8.839934	2.88E-05	0.001292	3.040349	0.510316	0.726814	-0.2165	10	52287323	R	II	SGMS1	5'UTR	opensea	5'UTR-oper		rs5283948	10;1
cg2377685	0.212746	0.634965	8.83991	2.88E-05	0.001292	3.040329	0.528592	0.741338	-0.21275	2	59662053	F	II		IGR	opensea	IGR-opense		rs5562569	37
cg1437996	0.207188	0.561206	8.839872	2.88E-05	0.001292	3.040298	0.457612	0.6648	-0.20719	21	33968392	F	II		IGR	opensea	IGR-opense		rs5530376	43
cg2564530	0.217907	0.500043	8.839503	2.88E-05	0.001292	3.039993	0.39109	0.608997	-0.21791	16	67642367	F	II	CTCF	5'UTR	opensea	5'UTR-oper		rs5691246	9;38;40
cg1058235	0.231569	0.602794	8.838107	2.88E-05	0.001292	3.038839	0.487009	0.718578	-0.23157	2	33090774	F	II	LINC00486	Body	opensea	Body-opens		rs5777335	0
cg0125426	0.225106	0.641012	8.83439	2.89E-05	0.001294	3.035765	0.528459	0.753565	-0.22511	6	33205676	F	II		IGR	opensea	IGR-opense		rs5501420	24;36
cg1760134	0.214483	0.464085	8.833984	2.89E-05	0.001294	3.03543	0.356843	0.571327	-0.21448	7	1.16E+08	R	II		IGR	opensea	IGR-opense		rs5522345	14;2
cg2328743	0.204219	0.618903	8.832174	2.89E-05	0.001295	3.033932	0.516794	0.721013	-0.20422	4	57937220	R	II	IGFBP7	Body	opensea	Body-opens		rs1718841	51;41;27;24;19;18
cg2520248	0.224358	0.389676	8.831676	2.9E-05	0.001295	3.03352	0.277497	0.501854	-0.22436	9	1.12E+08	F	II		IGR	opensea	IGR-opense		rs1858313	0
cg0307282	0.230351	0.541934	8.831455	2.9E-05	0.001295	3.033337	0.426758	0.65711	-0.23035	3	34612693	R	II		IGR	opensea	IGR-opense		rs1852918	40;37;2
cg0018289	0.236848	0.670375	8.830804	2.9E-05	0.001295	3.032799	0.551951	0.788799	-0.23685	6	1.4E+08	R	II		IGR	opensea	IGR-opense			
cg0815524	0.201333	0.650624	8.829676	2.9E-05	0.001295	3.031866	0.549958	0.751291	-0.20133	5	72919936	R	II		IGR	shore	IGR-shore	chr5:7292	rs2112161	51;49;37;8
cg1208496	0.218682	0.559909	8.828761	2.9E-05	0.001296	3.031108	0.450568	0.669249	-0.21868	7	1.07E+08	F	II	SLC26A4	ExonBnd	opensea	ExonBnd-op		rs3777137	1;29;30
cg1844748	0.24219	0.581099	8.827778	2.9E-05	0.001296	3.030294	0.460004	0.702194	-0.24219	1	1.5E+08	R	II	PRPF3	5'UTR	shore	5'UTR-shor	chr1:1502	rs3677403	23
cg1689846	0.212536	0.545032	8.827386	2.91E-05	0.001297	3.02997	0.438764	0.6513	-0.21254	13	29680394	R	II	MTUS2	Body	opensea	Body-opens		rs5416829	40;2;1
cg2347501	0.209572	0.338425	8.826864	2.91E-05	0.001297	3.029537	0.233639	0.44321	-0.20957	3	1.24E+08	R	II	KALRN	Body	opensea	Body-opens		rs1168113	10
cg0475031	0.213622	0.593911	8.826243	2.91E-05	0.001297	3.029024	0.487099	0.700722	-0.21362	1	42935595	F	II	PPCS	Body	opensea	Body-opens		rs7406844	0;16
cg0811166	0.200128	0.425646	8.825748	2.91E-05	0.001297	3.028614	0.325582	0.525709	-0.20013	12	2223540	R	II	CACNA1C	Body	shore	Body-shore	chr12:222		
cg1463074	0.201134	0.500468	8.824503	2.91E-05	0.001298	3.027583	0.399901	0.601035	-0.20113	3	1.8E+08	R	II		IGR	opensea	IGR-opense		rs1879617	13
cg2270012	0.212331	0.710401	8.824188	2.91E-05	0.001298	3.027322	0.604236	0.816567	-0.21233	4	4272480	F	II	LYAR	Body	opensea	Body-opens		rs1093783	22
cg1386068	0.234269	0.555109	8.823851	2.91E-05	0.001298	3.027043	0.437975	0.672244	-0.23427	20	37650491	F	II	DHX35	ExonBnd	opensea	ExonBnd-op		rs5344168	27;28
cg1249091	0.207563	0.732905	8.823229	2.92E-05	0.001298	3.026529	0.629123	0.836686	-0.20756	7	6712945	F	II		IGR	opensea	IGR-opense		rs1483039	1
cg0004551	0.239687	0.542571	8.822756	2.92E-05	0.001298	3.026136	0.422727	0.662414	-0.23969	20	35723332	F	II	RBL1	Body	shore	Body-shore	chr20:357		
cg2194598	0.226277	0.572921	8.821533	2.92E-05	0.001299	3.025123	0.459783	0.68606	-0.22628	4	70826695	F	II	CSN2	1stExon	opensea	1stExon-op		rs2016252	8;12;19;29;42
cg2481527	0.208601	0.482958	8.820475	2.92E-05	0.0013	3.024247	0.378657	0.587258	-0.2086	4	23780009	R	II		IGR	opensea	IGR-opense			
cg0818291	0.221765	0.416353	8.820026	2.92E-05	0.0013	3.023876	0.305471	0.527236	-0.22177	3	1.88E+08	F	II		IGR	opensea	IGR-opense			
cg0724454	0.205186	0.539207	8.818147	2.93E-05	0.001301	3.022319	0.436614	0.6418	-0.20519	15	92983829	R	II	ST8SIA2	Body	opensea	Body-opens		rs5596691	35;13;1
cg0898748	0.218844	0.462607	8.817165	2.93E-05	0.001301	3.021505	0.353185	0.572029	-0.21884	1	10585305	R	II	PEX14	Body	opensea	Body-opens		rs5475825	2
cg0019729	0.203386	0.643594	8.816996	2.93E-05	0.001301	3.021365	0.541901	0.745287	-0.20339	2	2870610	F	II		IGR	opensea	IGR-opense		rs5621872	26;28;34;35;40
cg1116406	0.224332	0.548387	8.816111	2.93E-05	0.001302	3.020632	0.43622	0.660553	-0.22433	14	66171298	F	II	FUT8	Body	opensea	Body-opens		rs5436008	8
cg1349786	0.210149	0.569064	8.816026	2.93E-05	0.001302	3.020561	0.463989	0.674138	-0.21015	5	9524497	F	II	SEMA5A	5'UTR	opensea	5'UTR-oper		rs5318858	1;16;39
cg0741526	0.223889	0.629256	8.814511	2.94E-05	0.001303	3.019305	0.517311	0.7412	-0.22389	2	1.03E+08	F	II	IL18RAP	Body	opensea	Body-opens		rs5582622	27
cg0709885	0.234206	0.395064	8.813823	2.94E-05	0.001303	3.018735	0.277961	0.512167	-0.23421	17	27048437	F	II	SNORD4A	TSS1500	shore	TSS1500-sh	chr17:270	rs5410853	1;20;24;33
cg2647251	0.246404	0.438613	8.813779	2.94E-05	0.001303	3.018699	0.315411	0.561815	-0.2464	12	27332626	F	II		IGR	opensea	IGR-opense		rs5736890	1
cg0890996	0.209811	0.571467	8.813227	2.94E-05	0.001303	3.018241	0.466562	0.676373	-0.20981	1	76262984	R	II	MSH4	Body	shore	Body-shore	chr1:7626	rs5745312	46
cg2552844	0.20123	0.717893	8.811825	2.94E-05	0.001304	3.017079	0.617277	0.818508	-0.20123	1	2.17E+08	R	II	ESRRG	5'UTR	opensea	5'UTR-oper		rs5368377	2
cg2258314	0.241972	0.424615	8.81164	2.94E-05	0.001304	3.016925	0.303629	0.545601	-0.24197	3	44331824	F	II	C3orf77	Body	opensea	Body-opens		rs5561627	0
cg2502863	0.221947	0.649023	8.810952	2.94E-05	0.001304	3.016355	0.53805	0.759997	-0.22195	11	9884897	R	II	SBF2	Body	opensea	Body-opens			
cg1661353	0.210509	0.61355	8.8108	2.94E-05	0.001304	3.016229	0.508295	0.718804	-0.21051	4	1.76E+08	R	II		IGR	opensea	IGR-opense		rs1385533	51
cg1344721	0.202714	0.408373	8.810668	2.94E-05	0.001304	3.01612	0.307016	0.509731	-0.20271	19	51211846	R	II	SHANK1	Body	opensea	Body-opens		rs5583463	10
cg0138587	0.267656	0.49613	8.807357	2.95E-05	0.001306	3.013373	0.362302	0.629958	-0.26766	5	1.24E+08	F	II		IGR	opensea	IGR-opense		rs7378434	8

cg1160179	0.210496	0.496034	8.806703	2.95E-05	0.001307	3.012831	0.390786	0.601282	-0.2105	1	60891629	F	II		IGR	opensea	IGR-opense		rs1120760	13;39
cg1156448	0.211604	0.585863	8.805271	2.96E-05	0.001307	3.011643	0.480061	0.691666	-0.2116	21	40266574	F	II	LOC40086	Body	opensea	Body-opens		rs5498630	10;20
cg1244578	0.218539	0.533527	8.804631	2.96E-05	0.001308	3.011112	0.424258	0.642797	-0.21854	15	89533560	R	II		IGR	opensea	IGR-opense		rs1466176	25;15;2
cg1066137	0.223007	0.616643	8.804438	2.96E-05	0.001308	3.010952	0.50514	0.728146	-0.22301	1	2.18E+08	F	II	GPATCH2	Body	opensea	Body-opens		rs1867127	0;1
cg1753736	0.236359	0.577819	8.803975	2.96E-05	0.001308	3.010567	0.45964	0.695999	-0.23636	4	75260310	F	II		IGR	opensea	IGR-opense		rs5771241	1;2;22;27
cg0303648	0.201548	0.549761	8.803354	2.96E-05	0.001308	3.010053	0.448987	0.650534	-0.20155	10	82300151	R	II	SH2D4B	TSS1500	shelf	TSS1500-sh	chr10:8229	rs5650443	45;19;12
cg0399564	0.219789	0.614368	8.803218	2.96E-05	0.001308	3.00994	0.504474	0.724262	-0.21979	10	11425457	F	II		IGR	opensea	IGR-opense		rs1849379	1
cg0286018	0.256038	0.577587	8.802427	2.96E-05	0.001309	3.009283	0.449568	0.705606	-0.25604	21	39876250	R	II	ERG	Body	opensea	Body-opens			
cg2358205	0.235712	0.639757	8.802349	2.96E-05	0.001309	3.009218	0.521901	0.757613	-0.23571	3	3152201	R	II	IL5RA	TSS200	opensea	TSS200-ope		rs1132542	28;17;12;9
cg1449128	0.21979	0.503628	8.800948	2.97E-05	0.001309	3.008055	0.393733	0.613523	-0.21979	4	74846046	F	II		IGR	shore	IGR-shore	chr4:7484	rs1478434	1;28;29;42
cg0661569	0.200886	0.559634	8.800707	2.97E-05	0.001309	3.007856	0.459191	0.660077	-0.20089	13	78832424	F	II		IGR	opensea	IGR-opense		rs5646600	40
cg2163654	0.205123	0.566553	8.800702	2.97E-05	0.001309	3.007851	0.463991	0.669114	-0.20512	10	45804715	R	II	OR13A1	5'UTR	opensea	5'UTR-open			
cg1581934	0.20208	0.517826	8.800242	2.97E-05	0.001309	3.007469	0.416786	0.618866	-0.20208	7	28579607	F	II	CREB5	Body	opensea	Body-opens		rs5645345	0
cg0998867	0.230162	0.467294	8.799227	2.97E-05	0.00131	3.006627	0.352213	0.582375	-0.23016	4	8351738	F	II		IGR	shelf	IGR-shelf	chr4:8347	rs7795991	15;33
cg2063104	0.202065	0.651112	8.797922	2.97E-05	0.001311	3.005543	0.55008	0.752144	-0.20206	19	41255890	F	II	C19orf54	TSS200	shore	TSS200-sho	chr19:4125	rs5410414	14;17;30
cg0761315	0.221666	0.483428	8.797717	2.98E-05	0.001311	3.005373	0.372595	0.594261	-0.22167	14	1.04E+08	F	II	TDRD9	Body	opensea	Body-opens		rs2003308	20;44;50
cg0540574	0.212957	0.423411	8.797535	2.98E-05	0.001311	3.005222	0.316932	0.52989	-0.21296	14	93313134	R	II		IGR	opensea	IGR-opense			
cg0098335	0.221349	0.59877	8.796891	2.98E-05	0.001311	3.004687	0.488095	0.709444	-0.22135	8	1.08E+08	R	II		IGR	opensea	IGR-opense		rs5552767	21;1
cg2110886	0.219295	0.655195	8.796397	2.98E-05	0.001311	3.004277	0.545548	0.764843	-0.21929	7	28852950	F	II	CREB5	Body	opensea	Body-opens		rs1892503	0;39
cg0431731	0.209686	0.309559	8.796323	2.98E-05	0.001311	3.004215	0.204716	0.414402	-0.20969	12	76338627	F	II		IGR	opensea	IGR-opense			
cg1436033	0.208208	0.591437	8.795686	2.98E-05	0.001311	3.003687	0.487332	0.695541	-0.20821	2	1.1E+08	R	II		IGR	opensea	IGR-opense			
cg0461250	0.211811	0.445861	8.794931	2.98E-05	0.001311	3.003059	0.339956	0.551767	-0.21181	2	2.23E+08	F	II	SGPP2	Body	opensea	Body-opens		rs5350487	17;25
cg0813032	0.215298	0.724343	8.79485	2.98E-05	0.001311	3.002992	0.616694	0.831992	-0.2153	18	32401932	R	II	DTNA	Body	opensea	Body-opens		rs5317338	2;1
cg1248612	0.267054	0.491225	8.794777	2.98E-05	0.001311	3.002931	0.357698	0.624752	-0.26705	8	1.29E+08	F	II		IGR	opensea	IGR-opense		rs7957746	1
cg1793146	0.221223	0.448002	8.794605	2.98E-05	0.001312	3.002788	0.33739	0.558613	-0.22122	6	1.05E+08	F	II		IGR	opensea	IGR-opense		rs5320112	46
cg0692329	0.21058	0.593427	8.794294	2.98E-05	0.001312	3.00253	0.488137	0.698716	-0.21058	12	90456743	R	II		IGR	opensea	IGR-opense			
cg2047307	0.250718	0.444525	8.792385	2.99E-05	0.001312	3.000944	0.319166	0.569884	-0.25072	15	48106646	F	II	LINC01491	Body	opensea	Body-opens		rs5373693	0;17;30
cg2036028	0.209027	0.563221	8.791899	2.99E-05	0.001312	3.000541	0.458707	0.667734	-0.20903	7	47989345	F	II	PKD1L1	TSS1500	opensea	TSS1500-op			
cg2510088	0.219071	0.529923	8.791847	2.99E-05	0.001312	3.000497	0.420387	0.639458	-0.21907	11	5878019	F	II	OR52E8	1stExon	opensea	1stExon-op		rs1491428	16;31;40;44;46;50
cg2219210	0.21787	0.696604	8.790959	2.99E-05	0.001313	2.999759	0.587669	0.805539	-0.21787	15	83594012	F	II	HOMER2	Body	opensea	Body-opens		rs1511789	48
cg1436659	0.23102	0.506847	8.790911	2.99E-05	0.001313	2.999719	0.391338	0.622357	-0.23102	14	23841754	F	II	IL25	TSS1500	opensea	TSS1500-op		rs5619445	1
cg1914867	0.230625	0.521871	8.789112	3E-05	0.001314	2.998224	0.406559	0.637184	-0.23062	11	1.11E+08	F	II		IGR	opensea	IGR-opense		rs5741286	26;38
cg2503831	0.22726	0.483645	8.78785	3E-05	0.001315	2.997175	0.370015	0.597276	-0.22726	10	1.05E+08	R	II		IGR	opensea	IGR-opense		rs1439551	9
cg1242362	0.203336	0.734652	8.787595	3E-05	0.001315	2.996963	0.632984	0.83632	-0.20334	2	45469414	R	II		IGR	opensea	IGR-opense			
cg2006035	0.245023	0.4604	8.787396	3E-05	0.001315	2.996798	0.337888	0.582911	-0.24502	8	1.07E+08	F	II	ZFPM2-AS1	Body	opensea	Body-opens		rs1896580	4;26;26
cg1533581	0.20321	0.579826	8.787092	3E-05	0.001315	2.996545	0.478221	0.681431	-0.20321	6	37836577	F	II	ZFAND3	Body	opensea	Body-opens		rs1470998	0;29
cg1346400	0.23421	0.645082	8.786654	3E-05	0.001315	2.996181	0.527977	0.762188	-0.23421	17	38976709	F	II	TMEM99	5'UTR	opensea	5'UTR-open		rs5535599	1;7;15;20;38;45
cg2523201	0.200594	0.562327	8.786164	3E-05	0.001315	2.995774	0.46203	0.662624	-0.20059	21	25695067	R	II	LOC10192	TSS1500	opensea	TSS1500-op			
cg1496373	0.239335	0.525741	8.785925	3E-05	0.001315	2.995575	0.406074	0.645408	-0.23933	7	19752782	F	II		IGR	shelf	IGR-shelf	chr7:1974	rs1422947	0;1;23
cg1035030	0.201694	0.589298	8.785136	3.01E-05	0.001316	2.994919	0.488451	0.690145	-0.20169	8	19935591	R	II		IGR	opensea	IGR-opense		rs5323543	28;21;5;1
cg1659274	0.209458	0.612856	8.784743	3.01E-05	0.001316	2.994592	0.508127	0.717585	-0.20946	9	1.18E+08	R	II		IGR	opensea	IGR-opense		rs7699568	7;1
cg0853320	0.21525	0.618121	8.783411	3.01E-05	0.001316	2.993485	0.510496	0.725746	-0.21525	8	821387	F	II	ERICH1-AS1	Body	opensea	Body-opens		rs5694385	24
cg1644966	0.2277	0.465943	8.783316	3.01E-05	0.001316	2.993406	0.352093	0.579793	-0.2277	1	2.42E+08	R	II		IGR	opensea	IGR-opense		rs5285268	42;2;1
cg0685463	0.2165	0.668658	8.783211	3.01E-05	0.001316	2.993319	0.560408	0.776908	-0.2165	7	22702493	F	II	LOC40131	Body	shelf	Body-shelf	chr7:2270	rs3684775	1
cg2293877	0.200671	0.58646	8.782216	3.01E-05	0.001317	2.992491	0.486124	0.686795	-0.20067	10	73185294	R	II	CDH23	5'UTR	opensea	5'UTR-open		rs1421553	50
cg0724725	0.240099	0.592668	8.780315	3.02E-05	0.001318	2.990909	0.472618	0.712717	-0.2401	2	1.66E+08	R	II	CSRNP3	Body	opensea	Body-opens		rs5640375	49;35
cg2495115	0.218372	0.528068	8.778625	3.02E-05	0.001319	2.989503	0.418883	0.637254	-0.21837	6	70894520	F	II	COL19A1	Body	opensea	Body-opens		rs5354028	14;49
cg1755836	0.216684	0.686464	8.778229	3.02E-05	0.001319	2.989174	0.578122	0.794806	-0.21668	8	1.06E+08	F	II	ZFPM2	Body	opensea	Body-opens		rs1400871	2;14
cg2005885	0.241685	0.567429	8.777971	3.02E-05	0.001319	2.988959	0.446586	0.688271	-0.24168	2	2.25E+08	F	II	AP1S3	Body	opensea	Body-opens		rs3738281	1;19
cg0513215	0.200071	0.5987	8.777754	3.02E-05	0.001319	2.988779	0.498665	0.698736	-0.20007	4	1.08E+08	R	II		IGR	opensea	IGR-opense		rs6138977	47;3
cg0751754	0.221405	0.529851	8.775968	3.03E-05	0.00132	2.987292	0.419149	0.640554	-0.22141	5	1.35E+08	R	II		IGR	opensea	IGR-opense		rs1846596	28;22;14;2
cg1939315	0.221299	0.619596	8.775707	3.03E-05	0.00132	2.987075	0.508947	0.730246	-0.2213	3	14802578	R	II	C3orf20	Body	opensea	Body-opens		rs7573707	39;1
cg0641371	0.220252	0.567575	8.77565	3.03E-05	0.00132	2.987028	0.457449	0.677701	-0.22025	1	1.85E+08	F	II	FAM129A	Body	opensea	Body-opens		rs5555361	0
cg0709918	0.200002	0.63403	8.775649	3.03E-05	0.00132	2.987026	0.534029	0.734031	-0.2	3	61947083	R	II	PTPRG	Body	opensea	Body-opens		rs1891201	1
cg0584597	0.229771	0.526353	8.775484	3.03E-05	0.00132	2.986889	0.411467	0.641238	-0.22977	10	3634843	R	II	LOC10537	Body	opensea	Body-opens		rs5478352	50;49
cg2044501	0.229003	0.558508	8.775399	3.03E-05	0.00132	2.986818	0.444006	0.673009	-0.229	12	82987128	F	II		IGR	opensea	IGR-opense		rs1167473	8;9
cg1316052	0.204906	0.629323	8.774986	3.03E-05	0.00132	2.986474	0.52687	0.731776	-0.20491	7	28991987	R	II		IGR	shelf	IGR-shelf	chr7:2899	rs5674932	43;37;34;7;5
cg1602503	0.252686	0.637241	8.774242	3.03E-05	0.00132	2.985856	0.510898	0.763584	-0.25269	1	2.07E+08	R	II		IGR	opensea	IGR-opense		rs5453057	10;9
cg0762003	0.261963	0.469175	8.774201	3.03E-05	0.00132	2.985821	0.338194	0.600157	-0.26196	13	39771708	R	II		IGR	opensea	IGR-opense		rs5583166	24
cg1688693	0.21528	0.631863	8.774161	3.03E-05	0.00132	2.985788	0.524222	0.739503	-0.21528	2	2.4E+08	F	II		IGR	opensea	IGR-opense		rs5653095	0;1;11
cg1049965	0.210694	0.576466	8.772889	3.03E-05	0.001321	2.984729	0.471119	0.681813	-0.21069	3	12465415	F	II	PPARG	Body	opensea	Body-opens		rs1908012	12;35;37
cg2320181	0.20649	0.584631	8.772451	3.04E-05	0.001321	2.984364	0.481387	0.687876	-0.20649	19	40007922	R	II	SELV	Body	shore	Body-shore	chr19:400	rs1414039	2
cg2081477	0.201659	0.61873	8.772071	3.04E-05	0.001321	2.984048	0.517901	0.719559	-0.20166	4	10467319	R	II		IGR	shelf	IGR-shelf	chr4:1046	rs5656291	39;13;9;8
cg0413554	0.207257	0.492395	8.770757	3.04E-05	0.001322	2.982954	0.388767	0.596023	-0.20726	4	42296896	R	II		IGR	opensea	IGR-opense		rs5487525	51;2
cg0116889	0.202679	0.499416	8.768373	3.05E-05	0.001324	2.980968	0.398076	0.600755	-0.20268	22	26222189	F	II	MYO18B	Body	opensea	Body-opens		rs7856155	0
cg0832330	0.213319	0.64971	8.768127	3.05E-05	0.001324	2.980763	0.54305	0.756369	-0.21332	16	72201692	F	II	PMFBP1	Body	opensea	Body-opens			
cg1368974	0.204409	0.480173	8.768017	3.05E-05	0.001324	2.980672	0.377969	0.582377	-0.20441	6	76491683	F	II	MYO6	5'UTR	opensea	5'UTR-open		rs1473692	1;6;8;16
cg0870291	0.216487	0.492776	8.767314	3.05E-05	0.001324	2.980086	0.384532	0.601019	-0.21649	1	76253688	F	II	RABGGTB	Body	opensea	Body-opens		rs3743855	0
cg2075883	0.231591	0.443824	8.766744	3.05E-05	0.001324	2.979611	0.328029	0.55962	-0.23159	4	1.14E+08	F	II	ANK2	Body	opensea	Body-opens		rs5699997	31;46
cg0720663	0.230337	0.575857	8.766531	3.05E-05	0.001324	2.979433	0.460689	0.691025	-0.23034	5	1.42E+08	R	II	FGF1	5'UTR	opensea	5'UTR-open		rs1407738	15;6;1
cg2687683	0.237399	0.352128	8.766012	3.05E-05	0.001324	2.979001	0.233428	0.470828	-0.2374	16	2013573	F	I	SNHG9	TSS1500	shore	TSS1500-sh	chr16:201	rs5421878	0;15;28
cg2012459	0.204822	0.509944	8.765961	3.05E-05	0.001324	2.978959	0.407533	0.612355	-0.20482	14	35825453	R	II		IGR	opensea	IGR-opense		rs8005252	19;18;2;1
cg1629503	0.241991	0.627547	8.765764	3.05E-05	0.001324	2.978794	0.506551	0.748542	-0.24199	5	94784489	F	II	FAM81B	Body	opensea	Body-opens		rs5710572	12;50
cg0432779	0.244817	0.58694	8.764781	3.05E-05	0.001324	2.977976	0.464531	0.709348	-0.24482	21	46937803	F	II	SLC19A1	Body	opensea	Body-opens		rs1911766	14;15;35
cg1346727	0.215545	0.611116	8.764652	3.05E-05	0.001324	2.977868	0.503344	0.718889	-0.21555	4	96458630	R	II	UNC5C	Body	opensea	Body-opens		rs5300867	42;34
cg0636231	0.23064	0.543102	8.763549	3.06E-05	0.001325	2.976949	0.427782	0.658422	-0.23064	6	52819489	F	II		IGR	opensea	IGR-opense		rs1176404	1;4;12;21;45;48
cg1211123	0.211465	0.539123	8.762322	3.06E-05	0.001326	2.975926	0.433391	0.644856	-0.21147	1	2.08E+08	F	II		IGR	opensea	IGR-opense		rs7530217	1;5
cg0416363	0.214967	0.681722	8.761869	3.06E-05	0.001326	2.975549	0.574239	0.789206	-0.21497	2	16832247	R	II	FAM49A	5'UTR	opensea	5'UTR-open		rs7592753	7;1
cg0670299	0.201342	0.630498	8.760436	3.06E-05	0.001327	2.974354	0.529827	0.731169	-0.20134	11	92562727	R	II	FAT3	Body	opensea	Body-opens		rs5687594	4
cg1656616	0.242265	0.628027	8.758374	3.07E-05	0.001328	2.972634	0.506895	0.74916	-0.24227	4	1.54E+08	R	II	FHDC1	Body	shelf	Body-shelf	chr4:1538	rs5719589	8
cg0259658	0.231917	0.566287	8.757699	3.07E-05	0.001328	2.972071	0.450329	0.682246	-0.23192	11	1.2E+08	R	II		IGR	opensea	IGR-opense		rs1174593	22
cg1137518	0.248314	0.447905	8.757643	3.07E-05	0.001328	2.972025	0.323748	0.572062	-0.24831	1	2.37E+08	R	II	RYR2	Body	opensea	Body-opens		rs1463152	51;1
cg0449787	0.209662	0.481114	8.757589	3.07E-05	0.001328	2.97198	0.376283	0.585946	-0.20966	1	42277284	F	II	HIVEP3	5'UTR	opensea	5'UTR-open		rs5433217	1;14;32
cg1160510	0.237353	0.553614	8.757177	3.07E-05	0.001328	2.971636	0.434938	0.672291	-0.23735	1	2.21E+08	F	II		IGR	opensea	IGR-opense		rs5291798	18;19
cg1956611	0.210894	0.648122	8.756886	3.07E-05	0.001328	2.971393	0.542675	0.753569	-0.21089	1	2.05E+08	R	II	LRRN2	5'UTR	opensea	5'UTR-open		rs1477295	1
cg2445511	0.200024	0.488483	8.756534	3.07E-05	0.001328	2.9711	0.388471	0.588495	-0.20002	2	1.21E+08	R	II		IGR	opensea	IGR-opense		rs5721112	17
cg2315480	0.204244	0.551777	8.755881	3.08E-05	0.001329	2.970556	0.449655	0.653899	-0.20424	1	1.89E+08	F	II		IGR	opensea	IGR-opense		rs5467094	0;28
cg2564555	0.25329	0.46051	8.754127	3.08E-05	0.00133	2.969092	0.333865	0.587154	-0.25329	6	17470599	F	II	CAP2	Body	opensea	Body-opens		rs5680000	5;38
cg0795677	0.211759	0.499124	8.752556	3.08E-05	0.001331	2.967781	0.393245	0.605004	-0.21176	15	71839040	F	II	THSD4	TSS1500	opensea	TSS1500-op		rs5715089	0;11;22;35
cg2555491	0.21122	0.513804	8.752291	3.08E-05	0.001331	2.967561	0.408195	0.619414	-0.21122	3	1.15E+08	R	II		IGR	opensea	IGR-opense			
cg2545581	0.206173	0.596727	8.751783	3.09E-05	0.001331	2.967137	0.49364	0.699813	-0.20617	2	68157657	F	II		IGR	opensea	IGR-opense		rs5699698	1;41;50
cg1841833	0.229892	0.456584	8.750993	3.09E-05	0.001332	2.966477	0.341638	0.57153	-0.22989	6	65488422	R	II	EYS	Body	opensea	Body-opens		rs1421072	1
cg1868806	0.202949	0.354005	8.750398	3.09E-05	0.001332	2.96598	0.252531	0.45548	-0.20295	6	31146471	R	II	PSORS1C3	TSS1500	shore	TSS1500-sh	chr6:3114	rs1052986	34;2;1
cg2619848	0.242593	0.577403	8.749958	3.09E-05	0.001332	2.965613	0.456106	0.698699	-0.24259	13	1.04E+08	F	II		IGR	opensea	IGR-opense		rs5367734	1
cg2650816	0.229885	0.56592	8.749825	3.09E-05	0.001332	2.965502	0.450977	0.680862	-0.22989	2	1.83E+08	F	II		IGR	opensea	IGR-opense		rs5774855	0;1
cg1495926	0.223653	0.434773	8.748811	3.09E-05	0.001333	2.964656	0.322947	0.5466	-0.22365	4	9917839	F	II	SLC2A9	Body	opensea	Body-opens		rs5424328	0;39
cg2396697	0.22592	0.696537	8.748739	3.09E-05	0.001333	2.964596	0.583577	0.809498	-0.22592	3	57145711	R	II	IL17RD	Body	opensea	Body-opens		rs5469212	35
cg1315935	0.218827	0.630108	8.747092	3.1E-05	0.001333	2.963221	0.520695	0.739521	-0.21883	10	25040372	F	II		IGR	opensea	IGR-opense		rs5276501	0;16;27
cg0696263	0.232857	0.537856	8.746239	3.1E-05	0.001333	2.962508	0.421427	0.654284	-0.23286	18	61939606	F	II	LOC28429	Body	opensea	Body-opens		rs1492375	0;1;7;8
cg0925464	0.205483	0.668253	8.745274	3.1E-05	0.001334	2.961703	0.565512	0.770995	-0.20548	1	84378747	F	II	MIR548AP	Body	opensea	Body-opens		rs1397600	12
cg2081095	0.214001	0.624286	8.744825	3.1E-05	0.001334	2.961328	0.517286	0.731287	-0.214	14	56552265	R	II		IGR	opensea	IGR-opense		rs1184602	33
cg2025159	0.222588	0.625129	8.742646	3.11E-05	0.001335	2.959508	0.513835	0.736423	-0.22259	6	74232674	R	II		IGR	shore	IGR-shore	chr6:7422	rs1394399	40;32;16;15
cg1257071	0.203884	0.458289	8.742494	3.11E-05	0.001335	2.959381	0.356347	0.560231	-0.20388	10	3822379	R	II	KLF6	Body	shore	Body-shore	chr10:382	rs1864099	30
cg0311735	0.245256	0.50832	8.741893	3.11E-05	0.001335	2.958879	0.385692	0.630948	-0.24526	10	34653147	F	II	PARD3	Body	opensea	Body-opens		rs1166202	1;12;32;38
cg2321664	0.240126	0.640323	8.73981	3.12E-05	0.001336	2.957139	0.52026	0.760386	-0.24013	7	1.42E+08	R	II	MGAM	ExonBnd	opensea	ExonBnd-op			
cg2383968	0.256314	0.512346	8.737409	3.12E-05	0.001337	2.955132	0.384189	0.640503	-0.25631	7	56118238	F	II	CCT6A	TSS1500	shore	TSS1500-sh	chr7:5611		
cg2117058	0.214507	0.632464	8.736685	3.12E-05	0.001338	2.954527	0.525211	0.739718	-0.21451	5	1.25E+08	R	II		IGR	opensea	IGR-opense		rs5344286	13
cg0292486	0.223653	0.497885	8.736401	3.12E-05	0.001338	2.95429	0.386059	0.609712	-0.22365	6	1.28E+08	F	II		IGR	opensea	IGR-opense			
cg0293693	0.231754	0.650321	8.7359	3.13E-05	0.001338	2.953871	0.534444	0.766197	-0.23175	6	1.35E+08	F	II		IGR	opensea	IGR-opense		rs5372686	0
cg1749759	0.255017	0.512019	8.735617	3.13E-05	0.001338	2.953634	0.38451	0.639528	-0.25502	3	1.55E+08	F	II	LOC10050	TSS200	opensea	TSS200-ope		rs5758219	0
cg2624194	0.223841	0.617073	8.734467	3.13E-05	0.001339	2.952673	0.505153	0.728994	-0.22384	3	1.55E+08	R	II	PLCH1	Body	opensea	Body-opens		rs1479018	8
cg0854849	0.252705	0.478157	8.73414	3.13E-05	0.001339	2.952399	0.351804	0.604509	-0.2527	20	43883546	F	II	SLPI	TSS1500	opensea	TSS1500-op		rs5485454	14;27
cg1053393	0.204801	0.666431	8.733166	3.13E-05	0.001339	2.951585	0.564031	0.768832	-0.2048	14	76345499	F	II	TTLL5	Body	opensea	Body-opens			
cg0905667	0.201753	0.608651	8.731045	3.14E-05	0.00134	2.949812	0.507775	0.709528	-0.20175	5	88743831	R	II		IGR	opensea	IGR-opense		rs5564947	38
cg1850414	0.206101	0.55509	8.730534	3.14E-05	0.00134	2.949384	0.45204	0.658141	-0.2061	1	7219603	R	II	CAMTA1	Body	opensea	Body-opens		rs5591219	47;19;1
cg1676986	0.202752	0.62728	8.729559	3.14E-05	0.001341	2.948568	0.525904	0.728656	-0.20275	1	43487359	F	II		IGR	opensea	IGR-opense		rs5648672	1;46
cg0682838	0.212235	0.720864	8.728563	3.14E-05	0.001342	2.947736	0.614746	0.826981	-0.21223	11	84998667	R	II	DLG2	Body	opensea	Body-opens		rs1391787	50;44;2
cg1919746	0.254673	0.514962	8.726767	3.15E-05	0.001342	2.946233	0.387626	0.642298	-0.25467	1	1.86E+08	F	II		IGR	opensea	IGR-opense		rs1175151	11
cg2348474	0.205832	0.680462	8.725273	3.15E-05	0.001343	2.944982	0.577546	0.783378	-0.20583	5	1.43E+08	R	II		IGR	opensea	IGR-opense		rs1818658	35;18;2

cg1618861	0.223428	0.553425	8.725199	3.15E-05	0.001343	2.94492	0.441711	0.665139	-0.22343	2	2.22E+08	R	II		IGR	opensea	IGR-opensea		rs1489323 1
cg2148491	0.212649	0.626312	8.724204	3.16E-05	0.001343	2.944088	0.519988	0.732636	-0.21265	2	40097661	F	II		IGR	opensea	IGR-opensea		rs5557528 19;35;44
cg1814290	0.232653	0.613264	8.723889	3.16E-05	0.001343	2.943824	0.496938	0.729591	-0.23265	3	1.67E+08	F	II	WDR49	Body	opensea	Body-opensea		rs2020586 14
cg0872326	0.215352	0.524262	8.723797	3.16E-05	0.001343	2.943747	0.416586	0.631938	-0.21535	2	2.36E+08	R	II		IGR	opensea	IGR-opensea		rs7838170 41;2
cg1046580	0.229733	0.580634	8.723303	3.16E-05	0.001344	2.943333	0.465767	0.6955	-0.22973	8	1.35E+08	F	II	LOC10192	Body	opensea	Body-opensea		rs1904172 1;23;41;47
cg0982468	0.217066	0.485238	8.721743	3.16E-05	0.001344	2.942028	0.376705	0.593771	-0.21707	10	52298570	F	II	SGMS1	5'UTR	opensea	5'UTR-opensea		rs1896173 0;50
cg0414716	0.213516	0.466685	8.721656	3.16E-05	0.001344	2.941955	0.359927	0.573443	-0.21352	8	67835448	F	II	SNHG6	Body	shelf	Body-shelf	chr8:6783	rs1165600 1
cg1479384	0.255825	0.572686	8.720982	3.16E-05	0.001344	2.94139	0.444774	0.700599	-0.25583	2	1.01E+08	R	II	AFF3	Body	opensea	Body-opensea		rs5705603 51;10
cg1331626	0.202243	0.602444	8.720859	3.16E-05	0.001344	2.941288	0.501323	0.703566	-0.20224	2	1.9E+08	R	II	COL5A2	Body	opensea	Body-opensea		rs1876201 48
cg1455676	0.207	0.491641	8.720827	3.16E-05	0.001344	2.941261	0.388141	0.595141	-0.207	1	1.71E+08	R	II		IGR	opensea	IGR-opensea		rs1470501 29;2
cg0536560	0.202621	0.604389	8.720291	3.16E-05	0.001344	2.940812	0.503078	0.705699	-0.20262	13	55827087	R	II		IGR	opensea	IGR-opensea		
cg0413794	0.255918	0.523356	8.719256	3.17E-05	0.001345	2.939945	0.395397	0.651315	-0.25592	4	1.64E+08	R	II	NAF1	Body	shore	Body-shore	chr4:1640	rs5424135 46;36
cg1303504	0.252796	0.540813	8.717559	3.17E-05	0.001346	2.938524	0.414415	0.667211	-0.2528	1	2.21E+08	R	II		IGR	opensea	IGR-opensea		
cg1840820	0.215621	0.643424	8.717284	3.17E-05	0.001346	2.938294	0.535614	0.751235	-0.21562	2	193509	R	II		IGR	shelf	IGR-shelf	chr2:1974	rs5401746 51;2;1
cg0242632	0.216111	0.636816	8.715628	3.18E-05	0.001347	2.936907	0.52876	0.744871	-0.21611	2	2.43E+08	R	II	D2HGDH	Body	opensea	Body-opensea		rs5625708 28;4;1
cg0370812	0.21337	0.410245	8.713631	3.18E-05	0.001348	2.935233	0.30356	0.51693	-0.21337	2	2.11E+08	F	II	CPS1	Body	opensea	Body-opensea		rs5569192 0;3;21
cg1963364	0.276954	0.460143	8.713409	3.18E-05	0.001348	2.935047	0.321666	0.59862	-0.27695	15	76428015	R	II	C15orf27	Body	opensea	Body-opensea		rs885388;r 23;14;1
cg1073568	0.207798	0.452922	8.712974	3.18E-05	0.001348	2.934683	0.349023	0.556821	-0.2078	18	21032577	F	II	RIOK3	TSS1500	shore	TSS1500-sh	chr18:210	rs5669039 22
cg0682772	0.21545	0.576689	8.712722	3.18E-05	0.001349	2.934472	0.468964	0.684414	-0.21545	1	2.04E+08	F	II	ETNK2	Body	opensea	Body-opensea		rs5545969 1;27;49
cg1268564	0.212653	0.672413	8.712645	3.18E-05	0.001349	2.934407	0.566086	0.778739	-0.21265	1	2.09E+08	F	II		IGR	opensea	IGR-opensea		rs5285683 15
cg2345332	0.255901	0.515331	8.712624	3.18E-05	0.001349	2.934389	0.387381	0.643282	-0.2559	4	14235703	F	II		IGR	opensea	IGR-opensea		rs5392765 1;3;7;19
cg2451695	0.215692	0.432883	8.712514	3.18E-05	0.001349	2.934298	0.325037	0.540729	-0.21569	13	89889903	F	II	LINC01047	TSS1500	opensea	TSS1500-op		rs1433831 0;1;22;33;48
cg0229857	0.213919	0.479294	8.710937	3.19E-05	0.001349	2.932976	0.372334	0.586254	-0.21392	10	1.15E+08	R	II		IGR	opensea	IGR-opensea		rs1157682 15;21;41
cg1531713	0.217804	0.647478	8.710594	3.19E-05	0.00135	2.932689	0.538576	0.75638	-0.2178	12	7900595	F	II	CLEC4C	5'UTR	opensea	5'UTR-opensea		
cg0032221	0.20862	0.593641	8.710493	3.19E-05	0.00135	2.932604	0.489331	0.697951	-0.20862	22	28072555	R	II		IGR	shore	IGR-shore	chr22:280	
cg1782217	0.27029	0.541355	8.709124	3.19E-05	0.00135	2.931456	0.40621	0.6765	-0.27029	2	1.45E+08	F	II		IGR	opensea	IGR-opensea		rs5737327 0
cg0554359	0.200181	0.593644	8.708573	3.19E-05	0.001351	2.930994	0.493554	0.693735	-0.20018	2	21511899	R	II		IGR	opensea	IGR-opensea		
cg1385372	0.216091	0.417482	8.705051	3.2E-05	0.001353	2.928041	0.309437	0.525528	-0.21609	20	38192245	R	II		IGR	opensea	IGR-opensea		rs7926079 20
cg1162242	0.216887	0.665887	8.704313	3.21E-05	0.001353	2.927422	0.557444	0.77433	-0.21689	3	1.51E+08	F	II	P2RY14	5'UTR	opensea	5'UTR-opensea		rs5679373 0;22;47
cg1751534	0.273408	0.544164	8.70218	3.21E-05	0.001354	2.925633	0.407461	0.680868	-0.27341	1	1.59E+08	R	II	AIM2	TSS1500	opensea	TSS1500-op		rs5652591 46;45;10;3
cg1370134	0.214747	0.728353	8.701333	3.21E-05	0.001354	2.924922	0.620979	0.835726	-0.21475	15	81212443	R	II	CEMIP	ExonBnd	opensea	ExonBnd-op		rs3716661 28;1
cg0488099	0.217404	0.529807	8.701021	3.21E-05	0.001355	2.92466	0.421105	0.638509	-0.2174	12	52887566	R	II	KRT6A	TSS1500	opensea	TSS1500-op		
cg1949972	0.205648	0.54785	8.700959	3.21E-05	0.001355	2.924608	0.445026	0.650674	-0.20565	12	1.13E+08	F	II		IGR	opensea	IGR-opensea		rs5503662 2;33;51
cg2231328	0.201634	0.613966	8.700614	3.22E-05	0.001355	2.924318	0.513149	0.714783	-0.20163	5	78057829	F	II		IGR	opensea	IGR-opensea		rs5600384 0;36
cg2180802	0.227757	0.441999	8.70015	3.22E-05	0.001355	2.923929	0.328121	0.555877	-0.22776	18	11871622	F	II	GNAL	Body	opensea	Body-opensea		rs5325637 0
cg0939965	0.216721	0.473085	8.698074	3.22E-05	0.001356	2.922187	0.364725	0.581445	-0.21672	10	82773304	F	II		IGR	opensea	IGR-opensea		rs1448274 0;1;13
cg1610107	0.245701	0.5302	8.697399	3.22E-05	0.001356	2.92162	0.407349	0.65305	-0.2457	10	1.3E+08	F	II	CLRN3	3'UTR	opensea	3'UTR-opensea		rs2296785 27;40
cg0262308	0.203063	0.635351	8.69654	3.23E-05	0.001356	2.920899	0.533819	0.736882	-0.20306	2	31138302	R	II	GALNT14	Body	opensea	Body-opensea		rs5680433 48
cg1279609	0.220859	0.523438	8.69648	3.23E-05	0.001356	2.920849	0.413009	0.633868	-0.22086	2	67911314	R	II		IGR	opensea	IGR-opensea		rs5729674 29;2;1
cg1004704	0.212052	0.519995	8.695345	3.23E-05	0.001357	2.919896	0.413969	0.626021	-0.21205	12	3446781	F	II		IGR	opensea	IGR-opensea		rs5649109 42;43;44
cg0672062	0.212457	0.543004	8.695322	3.23E-05	0.001357	2.919877	0.436776	0.649233	-0.21246	8	12646117	F	II	LOC34035	Body	opensea	Body-opensea		rs4481594 8;17;20
cg0718821	0.208241	0.638636	8.693745	3.23E-05	0.001358	2.918553	0.534515	0.742756	-0.20824	3	36592561	R	II		IGR	opensea	IGR-opensea		rs5847923 51;10
cg2553234	0.222304	0.579975	8.692514	3.24E-05	0.001358	2.917519	0.468823	0.691127	-0.2223	14	78047326	F	II	SPTLC2	Body	opensea	Body-opensea		rs1468760 1;24;27
cg1785303	0.22357	0.624898	8.690769	3.24E-05	0.001359	2.916053	0.513113	0.736683	-0.22357	2	69084431	R	II		IGR	opensea	IGR-opensea		rs5613635 21
cg2686992	0.2054	0.637321	8.690657	3.24E-05	0.001359	2.915959	0.53462	0.740021	-0.2054	6	53528893	F	II	KLHL31	5'UTR	opensea	5'UTR-opensea		rs5758979 0;36
cg1400512	0.222952	0.564744	8.690437	3.24E-05	0.001359	2.915774	0.453268	0.67622	-0.22295	9	93652538	R	II	SYK	Body	opensea	Body-opensea		rs5311974 51;1
cg0347965	0.206224	0.630693	8.68945	3.24E-05	0.001359	2.914945	0.527581	0.733805	-0.20622	2	1.36E+08	F	II	ZRANB3	Body	opensea	Body-opensea		rs1840871 2
cg1829376	0.20535	0.729047	8.689422	3.24E-05	0.001359	2.914922	0.626372	0.831723	-0.20535	3	1.9E+08	F	II	P3H2-AS1	Body	shore	Body-shore	chr3:1898	
cg1996944	0.204969	0.710722	8.689139	3.25E-05	0.001359	2.914684	0.608238	0.813207	-0.20497	20	11791322	R	II	LINC00687	Body	opensea	Body-opensea		rs1898646 40;38;37;2;1
cg1204318	0.225578	0.581175	8.688493	3.25E-05	0.001359	2.914141	0.468386	0.693964	-0.22558	1	2.27E+08	F	II		IGR	opensea	IGR-opensea		rs1464454 1;8;30
cg0498743	0.202083	0.398706	8.686315	3.25E-05	0.001361	2.912311	0.297665	0.499748	-0.20208	20	42380335	F	II		IGR	opensea	IGR-opensea		rs5711956 1;44;45
cg0668237	0.254569	0.562821	8.685889	3.25E-05	0.001361	2.911953	0.435536	0.690105	-0.25457	3	32291082	R	II	CMTM8	Body	opensea	Body-opensea		rs5515548 49;1
cg1004261	0.241311	0.578891	8.685777	3.25E-05	0.001361	2.911859	0.458235	0.699546	-0.24131	2	1.62E+08	F	II		IGR	opensea	IGR-opensea		
cg0605948	0.201085	0.605373	8.685521	3.25E-05	0.001361	2.911643	0.50483	0.705915	-0.20108	10	46171106	F	II		IGR	shelf	IGR-shelf	chr10:461	
cg1937553	0.20707	0.557545	8.684814	3.26E-05	0.001361	2.91105	0.45401	0.66108	-0.20707	2	64008069	R	II		IGR	opensea	IGR-opensea		rs5301480 1
cg0365983	0.204774	0.584882	8.684462	3.26E-05	0.001362	2.910753	0.482495	0.687269	-0.20477	5	35058495	F	II	PRLR	Body	opensea	Body-opensea		rs5584100 0;8;29
cg2673785	0.214961	0.541097	8.68411	3.26E-05	0.001362	2.910458	0.433617	0.648578	-0.21496	12	18849720	R	II	PLCZ1	Body	opensea	Body-opensea		rs7887755 32
cg0845390	0.211913	0.563212	8.682831	3.26E-05	0.001363	2.909383	0.457255	0.669169	-0.21191	17	71688187	R	II		IGR	opensea	IGR-opensea		rs5509517 48;40;22;16
cg2734905	0.205236	0.720586	8.681224	3.27E-05	0.001363	2.908032	0.617968	0.823204	-0.20524	5	1.42E+08	F	II		IGR	opensea	IGR-opensea		rs1480598 0
cg1436406	0.210778	0.58546	8.6797	3.27E-05	0.001365	2.90675	0.480071	0.690848	-0.21078	11	74667918	R	II	SPCS2	Body	opensea	Body-opensea		rs1146544 39;17
cg1665803	0.256582	0.477699	8.679596	3.27E-05	0.001365	2.906662	0.349408	0.60599	-0.25658	10	22919045	F	II	PIP4K2A	Body	opensea	Body-opensea		rs1878955 0;40;42;44
cg0849883	0.220885	0.54524	8.678795	3.27E-05	0.001365	2.905989	0.434797	0.655682	-0.22088	3	1.49E+08	R	II	WWTR1	Body	opensea	Body-opensea		
cg2739840	0.223919	0.671171	8.678445	3.27E-05	0.001365	2.905694	0.559211	0.783131	-0.22392	1	56976548	F	II	PPAP2B	Body	opensea	Body-opensea		rs5439112 41
cg1622002	0.200694	0.619298	8.678388	3.27E-05	0.001365	2.905646	0.518951	0.719645	-0.20069	4	1.4E+08	R	II	ELF2	Body	shelf	Body-shelf	chr4:1400	rs5612697 46
cg2038872	0.246236	0.642009	8.676963	3.28E-05	0.001366	2.904448	0.518891	0.765127	-0.24624	15	99200492	F	II	IGF1R	Body	opensea	Body-opensea		
cg1797401	0.232649	0.542729	8.676658	3.28E-05	0.001366	2.904191	0.426404	0.659054	-0.23265	11	1.2E+08	R	II		IGR	opensea	IGR-opensea		
cg0662160	0.233756	0.481739	8.674315	3.28E-05	0.001367	2.90222	0.364862	0.598617	-0.23376	5	82999102	R	II	HAPLN1	5'UTR	opensea	5'UTR-opensea		
cg2033901	0.222862	0.675067	8.67258	3.29E-05	0.001368	2.90076	0.563636	0.786498	-0.22286	6	1.2E+08	F	II	LOC28576	Body	opensea	Body-opensea		
cg0073759	0.255095	0.612744	8.671452	3.29E-05	0.001369	2.89981	0.485197	0.740292	-0.2551	1	43422337	F	II	SLC2A1	Body	shore	Body-shore	chr1:4342	rs5319325 13;46
cg1223097	0.218188	0.645186	8.670096	3.3E-05	0.00137	2.898669	0.536092	0.75428	-0.21819	1	1.92E+08	F	II		IGR	opensea	IGR-opensea		rs5736131 0;3;19
cg0888178	0.238797	0.594907	8.669755	3.3E-05	0.00137	2.898382	0.475509	0.714306	-0.2388	3	1.55E+08	R	II		IGR	shelf	IGR-shelf	chr3:1554	rs5742892 46;44;29
cg1795101	0.258853	0.454476	8.669604	3.3E-05	0.00137	2.898255	0.325049	0.583902	-0.25885	11	59972522	F	II		IGR	opensea	IGR-opensea		rs1907343 29
cg2758252	0.240101	0.555972	8.668496	3.3E-05	0.001371	2.897322	0.435922	0.676023	-0.2401	1	15734285	F	II		IGR	shore	IGR-shore	chr1:1573	rs5475215 0;16
cg0062856	0.201856	0.599504	8.668122	3.3E-05	0.001371	2.897007	0.498576	0.700432	-0.20186	5	71749237	F	II	ZNF366	Body	opensea	Body-opensea		rs5724812 0
cg1211225	0.238983	0.588189	8.667475	3.3E-05	0.001371	2.896461	0.468698	0.707681	-0.23898	2	1.97E+08	F	II	HECW2	Body	opensea	Body-opensea		rs5283422 0;1;35
cg0847692	0.241376	0.544335	8.667102	3.3E-05	0.001371	2.896148	0.423647	0.665023	-0.24138	11	82690000	F	II	RAB30	3'UTR	opensea	3'UTR-opensea		rs5695125 19
cg0691947	0.262305	0.455378	8.666346	3.31E-05	0.001372	2.895511	0.324226	0.586531	-0.26231	2	1.74E+08	R	II	ZAK	Body	opensea	Body-opensea		rs5422204 33
cg1541500	0.220838	0.571723	8.665511	3.31E-05	0.001372	2.894808	0.461304	0.682142	-0.22084	12	1.03E+08	R	II		IGR	opensea	IGR-opensea		rs5343378 49;27;8
cg0557407	0.201781	0.609666	8.66545	3.31E-05	0.001372	2.894756	0.508775	0.710556	-0.20178	3	1.43E+08	F	II	PAQR9-AS1	Body	opensea	Body-opensea		rs5332156 1;31
cg0146552	0.202829	0.59395	8.665117	3.31E-05	0.001372	2.894475	0.492535	0.695364	-0.20283	6	29548535	F	II	SNORD32B	TSS1500	opensea	TSS1500-op		rs3723249 36
cg1785517	0.231607	0.546892	8.664878	3.31E-05	0.001372	2.894275	0.431089	0.662696	-0.23161	20	19967270	F	II	RIN2	Body	opensea	Body-opensea		rs5354627 0;31;37
cg0896065	0.237287	0.418063	8.66477	3.31E-05	0.001372	2.894183	0.29942	0.536707	-0.23729	6	1.67E+08	R	II	RPS6KA2	Body	opensea	Body-opensea		rs5563195 20;1
cg0259670	0.20302	0.638111	8.664731	3.31E-05	0.001372	2.894151	0.536601	0.739621	-0.20302	13	93457414	R	II	GPC5	Body	opensea	Body-opensea		rs5482627 35
cg0929206	0.240236	0.635954	8.664444	3.31E-05	0.001372	2.893909	0.515836	0.756072	-0.24024	4	77801348	F	II		IGR	opensea	IGR-opensea		rs1860110 0
cg1810865	0.237265	0.491781	8.664288	3.31E-05	0.001372	2.893777	0.373148	0.610414	-0.23727	1	1.13E+08	R	II	CAPZA1	Body	opensea	Body-opensea		
cg1182632	0.206993	0.694927	8.664141	3.31E-05	0.001372	2.893654	0.591431	0.798423	-0.20699	8	1.04E+08	F	II		IGR	opensea	IGR-opensea		rs5504851 23
cg0056367	0.210041	0.597469	8.663883	3.31E-05	0.001372	2.893436	0.492448	0.70249	-0.21004	4	1.42E+08	R	II	RNF150	Body	opensea	Body-opensea		rs5283362 13
cg2689368	0.207845	0.569159	8.663213	3.31E-05	0.001373	2.892872	0.465237	0.673082	-0.20784	6	2523615	F	II		IGR	opensea	IGR-opensea		rs1828131 0;1;24;44
cg1312114	0.277988	0.569063	8.662598	3.32E-05	0.001373	2.892354	0.430069	0.708057	-0.27799	15	33218656	R	II	FMN1	Body	opensea	Body-opensea		rs5462501 39
cg1158526	0.210544	0.596923	8.662326	3.32E-05	0.001373	2.892125	0.49165	0.702195	-0.21054	6	1.39E+08	R	II	NHSL1	Body	opensea	Body-opensea		rs1442366 29;25
cg0026589	0.210517	0.519358	8.65991	3.32E-05	0.001374	2.890088	0.4141	0.624617	-0.21052	3	1.71E+08	R	II	PLD1	5'UTR	opensea	5'UTR-opensea		rs4484217 29;2
cg2069617	0.204846	0.607259	8.658304	3.33E-05	0.001375	2.888735	0.504836	0.709682	-0.20485	13	21497272	R	II		IGR	opensea	IGR-opensea		rs5513343 31;30;1
cg2068149	0.226069	0.482275	8.657802	3.33E-05	0.001375	2.888311	0.36924	0.595309	-0.22607	6	23175909	F	II		IGR	opensea	IGR-opensea		rs5421236 0;28
cg1538494	0.233154	0.497329	8.657692	3.33E-05	0.001375	2.888219	0.380751	0.613906	-0.23315	2	1.7E+08	F	II		IGR	opensea	IGR-opensea		rs7732124 16;37
cg1881821	0.210438	0.634607	8.65682	3.33E-05	0.001375	2.887484	0.529388	0.739826	-0.21044	5	1.47E+08	F	II	C5orf46	TSS1500	opensea	TSS1500-op		rs5646655 26
cg1904552	0.206086	0.675666	8.655953	3.33E-05	0.001376	2.886753	0.572623	0.778709	-0.20609	7	41246129	F	II		IGR	opensea	IGR-opensea		rs7978499 14
cg0979028	0.26257	0.644651	8.655679	3.33E-05	0.001376	2.886522	0.513366	0.775936	-0.26257	7	1.24E+08	R	II	LOC10192	Body	opensea	Body-opensea		rs7843655 24;1
cg0858731	0.250246	0.549236	8.654535	3.34E-05	0.001377	2.885557	0.424113	0.674359	-0.25025	8	1.3E+08	R	II		IGR	opensea	IGR-opensea		rs5328739 49;5
cg0844007	0.202289	0.529659	8.653377	3.34E-05	0.001377	2.884581	0.428514	0.630803	-0.20229	2	2.42E+08	R	II		IGR	shore	IGR-shore	chr2:2418	rs1454877 1
cg0624352	0.200111	0.613356	8.652713	3.34E-05	0.001377	2.884021	0.5133	0.713411	-0.20011	7	12797219	F	II		IGR	opensea	IGR-opensea		rs5765150 0
cg1349718	0.224297	0.559865	8.652438	3.34E-05	0.001377	2.883789	0.447717	0.672014	-0.2243	8	1.08E+08	R	II	OXR1	Body	opensea	Body-opensea		rs1809485 42;41;1
cg0327318	0.200844	0.608753	8.652105	3.34E-05	0.001377	2.883507	0.508331	0.709175	-0.20084	10	90761751	F	II	FAS	Body	opensea	Body-opensea		rs5327705 0
cg0036256	0.216753	0.541916	8.651736	3.34E-05	0.001377	2.883196	0.433539	0.650292	-0.21675	6	1.35E+08	F	II		IGR	opensea	IGR-opensea		rs5626292 1;42
cg1380884	0.223479	0.522535	8.650585	3.35E-05	0.001378	2.882226	0.410796	0.634274	-0.22348	1	1.55E+08	F	II		IGR	opensea	IGR-opensea		
cg0060207	0.265325	0.636625	8.649864	3.35E-05	0.001378	2.881617	0.503963	0.769288	-0.26533	5	33245479	F	II		IGR	opensea	IGR-opensea		rs5540954 0;1
cg1182475	0.218443	0.552318	8.649708	3.35E-05	0.001378	2.881486	0.443097	0.66154	-0.21844	20	3888037	F	II	PANK2	5'UTR	opensea	5'UTR-opensea		rs5531432 0;50
cg1148884	0.200943	0.639258	8.649299	3.35E-05	0.001378	2.88114	0.538787	0.73973	-0.20094	2	1.09E+08	R	II	SULT1C2	Body	opensea	Body-opensea		rs7548875 13;2
cg2743602	0.224883	0.591351	8.648639	3.35E-05	0.001378	2.880583	0.47891	0.703793	-0.22488	8	98859997	F	II	LAPTM4B	Body	opensea	Body-opensea		rs1910273 1;30;31
cg0754823	0.204063	0.483495	8.648168	3.35E-05	0.001378	2.880186	0.381464	0.585527	-0.20406	5	6520444	R	II		IGR	opensea	IGR-opensea		rs1005745 32;13;5
cg1751488	0.212693	0.653666	8.64768	3.36E-05	0.001379	2.879774	0.547319	0.760012	-0.21269	2	2E+08	R	II	LOC10192	Body	opensea	Body-opensea		
cg1167507	0.207922	0.668754	8.647248	3.36E-05	0.001379	2.87941	0.564793	0.772715	-0.20792	6	18353160	F	II		IGR	opensea	IGR-opensea		rs5726984 1;44
cg1550401	0.206155	0.666303	8.647246	3.36E-05	0.001379	2.879408	0.563225	0.769381	-0.20616	11	32498257	F	II		IGR	opensea	IGR-opensea		rs5680680 0;42
cg1456481	0.219897	0.613758	8.647088	3.36E-05	0.001379	2.879274	0.50381	0.723707	-0.2199	4	70689006	R	II		IGR	opensea	IGR-opensea		rs1478176 1
cg1702159	0.21951	0.502284	8.647034	3.36E-05	0.001379	2.879229	0.392529	0.612039	-0.21951	1	66418454	F	II	PDE4B	Body	opensea	Body-opensea		rs5652084 1;20
cg2736871	0.230368	0.54475	8.645694	3.36E-05	0.001379	2.878098	0.429566	0.659934	-0.23037	18	3883519	F	II		IGR	shelf	IGR-shelf	chr18:387	rs5747800 4
cg0070513	0.223271	0.368837	8.645346	3.36E-05	0.00138	2.877804	0.257202	0.480473	-0.22327	21	35649196	R	II		IGR	opensea	IGR-opensea		rs5638940 9;4;2;1
cg0347647	0.224977	0.528245	8.645061	3.36E-05	0.00138	2.877564	0.415757	0.640733	-0.22498	8	81851811	F	II		IGR	opensea	IGR-opensea		rs1890938 0;2;32;41;50
cg0745092	0.222059	0.545774	8.644886	3.36E-05	0.00138	2.877416	0.434745	0.656803	-0.22206	22	44701799	F	II	KIAA1644	5'UTR	opensea	5'UTR-opensea		rs1389547 0
cg1493548	0.21383	0.600917	8.644299	3.36E-05	0.00138	2.876921	0.494002	0.707832	-0.21383	22	39316598	R	II		IGR	opensea	IGR-opensea		rs3693533 38;11;1

cg1815847	0.214268	0.613185	8.643264	3.37E-05	0.00138	2.876047	0.506052	0.720319	-0.21427	14	89334755	F	II	TTC8	Body	opensea	Body-opens		rs1482316 1;30;33
cg1052211	0.223755	0.642426	8.643125	3.37E-05	0.00138	2.87593	0.530548	0.754303	-0.22375	10	76859441	R	II	DUSP13	Body	opensea	Body-opens		rs5778187 39
cg0748887	0.207179	0.570858	8.642628	3.37E-05	0.001381	2.87551	0.467269	0.674447	-0.20718	2	16955374	F	II		IGR	opensea	IGR-opense		rs1885662 0;1;30;40;41;47;49
cg1418033	0.227679	0.461994	8.642384	3.37E-05	0.001381	2.875304	0.348154	0.575833	-0.22768	5	40224461	R	II		IGR	opensea	IGR-opense		
cg1178078	0.215043	0.605866	8.641439	3.37E-05	0.001381	2.874506	0.498344	0.713388	-0.21504	16	72206241	F	I	PMFBP1	TSS200	opensea	TSS200-ope		
cg0429368	0.21369	0.708127	8.640739	3.37E-05	0.001381	2.873914	0.601282	0.814972	-0.21369	2	75466188	R	II		IGR	opensea	IGR-opense		rs5508823 1
cg0372967	0.270803	0.449855	8.640557	3.37E-05	0.001381	2.873761	0.314454	0.585257	-0.2708	5	71855774	F	II	LOC10250	Body	opensea	Body-opens		
cg2340240	0.201589	0.54002	8.639567	3.38E-05	0.001382	2.872925	0.439226	0.640815	-0.20159	15	86651355	R	II	LINC01584	Body	opensea	Body-opens		rs5322039 41;16
cg1232214	0.256344	0.636399	8.639491	3.38E-05	0.001382	2.87286	0.508227	0.764571	-0.25634	3	29197502	R	II		IGR	opensea	IGR-opense		rs4680717 37;2
cg0517743	0.214282	0.565208	8.638838	3.38E-05	0.001382	2.872309	0.458067	0.672349	-0.21428	3	1.47E+08	F	II		IGR	opensea	IGR-opense		rs7521296 0;1;29;31
cg0743067	0.207644	0.603579	8.63695	3.38E-05	0.001383	2.870714	0.499757	0.707401	-0.20764	16	10633189	F	II	EMP2	Body	opensea	Body-opens		rs5573487 3;26;42
cg0166511	0.206053	0.687541	8.635913	3.39E-05	0.001384	2.869838	0.584514	0.790567	-0.20605	13	77502805	F	II	BTF3L1	Body	opensea	Body-opens		
cg1314118	0.225226	0.467511	8.635626	3.39E-05	0.001384	2.869595	0.354898	0.580124	-0.22523	5	1.48E+08	R	II	HTR4	TSS1500	opensea	TSS1500-o		rs1454926 8;2
cg0360741	0.217015	0.521643	8.634485	3.39E-05	0.001385	2.868631	0.413136	0.630151	-0.21701	5	89881642	R	II	GPR98	Body	opensea	Body-opens		rs1892834 12
cg0671110	0.225262	0.465351	8.634083	3.39E-05	0.001385	2.868291	0.35272	0.577982	-0.22526	11	5811025	F	II	OR52N1	TSS1500	opensea	TSS1500-o		rs1450838 7;41;51
cg1386292	0.219237	0.580335	8.632913	3.4E-05	0.001385	2.867302	0.470717	0.689954	-0.21924	9	72158091	R	II	APBA1	5'UTR	opensea	5'UTR-open		rs1159191 50;2
cg2711105	0.252766	0.441992	8.631294	3.4E-05	0.001386	2.865933	0.315609	0.568375	-0.25277	2	1.39E+08	R	II	SPOPL	5'UTR	shore	5'UTR-shor	chr2:1392	rs5460482 41
cg1964250	0.204891	0.627389	8.630832	3.4E-05	0.001386	2.865543	0.524944	0.729835	-0.20489	6	30224156	R	II		IGR	shelf	IGR-shelf	chr6:3022	
cg0644939	0.205094	0.504655	8.629501	3.41E-05	0.001387	2.864417	0.402108	0.607202	-0.20509	22	46382247	F	II		IGR	opensea	IGR-opense		rs5639225 1
cg1579649	0.210641	0.635441	8.62935	3.41E-05	0.001387	2.86429	0.530121	0.740762	-0.21064	10	92811782	R	II	LINC00502	Body	opensea	Body-opens		
cg1275392	0.226848	0.441239	8.627845	3.41E-05	0.001388	2.863017	0.327816	0.554663	-0.22685	3	59552298	F	II		IGR	opensea	IGR-opense		rs5505723 0;46
cg0798903	0.217304	0.547135	8.627841	3.41E-05	0.001388	2.863014	0.438483	0.655787	-0.2173	10	98032439	R	II	BLNK	TSS1500	opensea	TSS1500-o		rs7072251 39;24;2
cg1865467	0.221852	0.399342	8.627695	3.41E-05	0.001388	2.862891	0.288416	0.510268	-0.22185	7	92794982	R	II		IGR	opensea	IGR-opense		
cg0077899	0.201049	0.491892	8.626208	3.41E-05	0.001389	2.861633	0.391368	0.592416	-0.20105	8	6275498	F	II	MCPH1	Body	opensea	Body-opens		rs5407716 1;31;38;41
cg0774876	0.232213	0.585473	8.62481	3.42E-05	0.00139	2.86045	0.469366	0.701579	-0.23221	2	1.13E+08	F	II	MERTK	Body	shelf	Body-shelf	chr2:1126	
cg0867146	0.239116	0.448774	8.62353	3.42E-05	0.00139	2.859367	0.329216	0.568332	-0.23912	12	51912686	R	II		IGR	opensea	IGR-opense		rs5409346 41;37;9;6
cg1588740	0.222768	0.568939	8.623279	3.42E-05	0.00139	2.859155	0.457555	0.680324	-0.22277	7	47986919	F	II	PKD1L1	Body	opensea	Body-opens		rs5286767 32
cg0757506	0.202426	0.672093	8.622574	3.42E-05	0.001391	2.858558	0.57088	0.773306	-0.20243	2	1.93E+08	R	II		IGR	opensea	IGR-opense		rs1683433 1
cg0843483	0.213863	0.478424	8.622336	3.42E-05	0.001391	2.858356	0.371493	0.585356	-0.21386	4	86792581	R	II	ARHGAP24	Body	opensea	Body-opens		rs5580067 2;1
cg1367984	0.202283	0.638226	8.622189	3.43E-05	0.001391	2.858233	0.537084	0.739368	-0.20228	12	29090040	R	II		IGR	opensea	IGR-opense		rs1827185 21
cg1054341	0.23257	0.532528	8.621308	3.43E-05	0.001391	2.857486	0.416243	0.648813	-0.23257	3	37489393	R	II		IGR	opensea	IGR-opense		rs7628974 50;45;2
cg1359723	0.210582	0.70114	8.620715	3.43E-05	0.001391	2.856985	0.595849	0.806431	-0.21058	12	10458222	F	II	KLRD1	5'UTR	opensea	5'UTR-open		rs5387291 0
cg0469705	0.205371	0.4849	8.620346	3.43E-05	0.001391	2.856672	0.382215	0.587585	-0.20537	4	1.88E+08	R	II	FAT1	Body	opensea	Body-opens		rs5668229 2
cg2579483	0.212178	0.522523	8.620341	3.43E-05	0.001391	2.856668	0.416434	0.628612	-0.21218	18	29018594	F	II		IGR	opensea	IGR-opense		rs1771661 10;39
cg0751229	0.220173	0.55055	8.619355	3.43E-05	0.001392	2.855833	0.440463	0.660636	-0.22017	2	2.35E+08	R	II	UGT1A6	TSS1500	opensea	TSS1500-o		rs7563561 37;2
cg0013473	0.250789	0.615719	8.618689	3.43E-05	0.001392	2.85527	0.490324	0.741114	-0.25079	1	1.2E+08	R	II		IGR	opensea	IGR-opense		rs5876723 37
cg2017532	0.270075	0.487228	8.618049	3.44E-05	0.001393	2.854728	0.352191	0.622266	-0.27007	13	50858812	F	II	DLEU1	Body	opensea	Body-opens		rs5378112 0
cg2475965	0.239745	0.514743	8.617022	3.44E-05	0.001393	2.853859	0.394871	0.634616	-0.23975	17	76801065	R	II	USP36	Body	shelf	Body-shelf	chr17:767	rs7476313 41;13;1
cg0230174	0.208732	0.604469	8.616758	3.44E-05	0.001394	2.853635	0.500103	0.708835	-0.20873	7	80593517	R	II		IGR	opensea	IGR-opense		rs5715258 46;22
cg0840934	0.20478	0.576842	8.616702	3.44E-05	0.001394	2.853587	0.474452	0.679232	-0.20478	7	30241229	R	II		IGR	opensea	IGR-opense		rs5288359 27
cg0076858	0.209857	0.525511	8.615319	3.44E-05	0.001394	2.852416	0.420582	0.630439	-0.20986	14	1.01E+08	F	II		IGR	opensea	IGR-opense		rs5627792 1;7;50
cg0789753	0.235238	0.547292	8.615075	3.45E-05	0.001395	2.85221	0.429673	0.664911	-0.23524	6	97098016	R	II		IGR	opensea	IGR-opense		rs1853949 35;7;1
cg0024633	0.218235	0.585483	8.612938	3.45E-05	0.001395	2.8504	0.476366	0.694601	-0.21824	12	79809932	F	II	SYT1	Body	opensea	Body-opens		rs5381424 4;30
cg0192824	0.218615	0.647342	8.612787	3.45E-05	0.001395	2.850272	0.538035	0.75665	-0.21861	15	72613427	F	II	CELF6	TSS1500	shore	TSS1500-sh	chr15:726	rs5745545 0
cg1226682	0.218806	0.514476	8.612225	3.45E-05	0.001396	2.849795	0.405073	0.623879	-0.21881	12	19954094	F	II		IGR	opensea	IGR-opense		rs1856792 12;36
cg1227801	0.227551	0.445271	8.611768	3.45E-05	0.001396	2.849409	0.331495	0.559047	-0.22755	1	35266042	F	II		IGR	opensea	IGR-opense		rs7943634 17;35
cg0904619	0.214494	0.54188	8.610961	3.46E-05	0.001396	2.848724	0.434634	0.649127	-0.21449	10	96351402	R	II	HELLS	Body	opensea	Body-opens		rs5285467 45;4;1
cg2374576	0.254745	0.525227	8.610823	3.46E-05	0.001396	2.848608	0.397854	0.652599	-0.25474	3	53589382	R	II	CACNA1D	Body	opensea	Body-opens		rs1853499 32
cg2349995	0.20393	0.639513	8.610705	3.46E-05	0.001396	2.848508	0.537548	0.741478	-0.20393	1	1.54E+08	R	II	S100A16	5'UTR	opensea	5'UTR-open		rs5351632 49;44;1
cg0514181	0.243532	0.588996	8.608706	3.46E-05	0.001398	2.846814	0.46723	0.710762	-0.24353	1	2.48E+08	F	II	OR13G1	TSS1500	opensea	TSS1500-o		rs1809210 22
cg0528488	0.227356	0.494841	8.608443	3.46E-05	0.001398	2.846591	0.381163	0.608519	-0.22736	3	1.01E+08	F	II	ABI3BP	Body	opensea	Body-opens		
cg1853638	0.213783	0.715419	8.60792	3.47E-05	0.001398	2.846147	0.608528	0.82231	-0.21378	6	1.37E+08	R	II		IGR	opensea	IGR-opense		
cg2733558	0.258701	0.507433	8.607889	3.47E-05	0.001398	2.846121	0.378083	0.636784	-0.2587	5	95616198	F	II	LOC10192	Body	opensea	Body-opens		
cg0428065	0.221709	0.596852	8.6078	3.47E-05	0.001398	2.846046	0.485998	0.707706	-0.22171	10	1.26E+08	F	II	CHST15	Body	opensea	Body-opens		rs1448830 24;32
cg2599201	0.22579	0.539161	8.606215	3.47E-05	0.001398	2.844702	0.426267	0.652056	-0.22579	13	77387018	R	II		IGR	opensea	IGR-opense		rs3775467 26;2
cg2031087	0.212338	0.393254	8.605336	3.47E-05	0.001399	2.843957	0.287085	0.499423	-0.21234	17	73310221	R	II		IGR	opensea	IGR-opense		
cg1355136	0.236258	0.59281	8.603548	3.48E-05	0.0014	2.842441	0.474681	0.710939	-0.23626	5	57370189	R	II		IGR	opensea	IGR-opense		rs5446055 40
cg0720349	0.213938	0.493622	8.602981	3.48E-05	0.0014	2.84196	0.386653	0.600591	-0.21394	20	49283757	F	II	FAM65C	Body	opensea	Body-opens		rs5489768 38
cg2080240	0.214742	0.613948	8.602902	3.48E-05	0.0014	2.841894	0.506577	0.721319	-0.21474	7	95496933	F	II	DYNC1I1	Body	opensea	Body-opens		rs5393377 1;48
cg0453017	0.218877	0.637498	8.600941	3.48E-05	0.001402	2.840231	0.52806	0.746937	-0.21888	8	23656770	F	II		IGR	opensea	IGR-opense		rs1817663 1;28
cg1685210	0.217572	0.625222	8.599472	3.49E-05	0.001403	2.838984	0.516437	0.734008	-0.21757	15	63624154	R	II	CA12	Body	opensea	Body-opens		rs3465206 12;2
cg0172007	0.234649	0.494292	8.597473	3.49E-05	0.001404	2.837288	0.376968	0.611617	-0.23465	12	31835278	R	II	AMN1	Body	opensea	Body-opens		
cg1693735	0.211161	0.531721	8.596168	3.5E-05	0.001405	2.836181	0.426141	0.637302	-0.21116	1	25070163	R	II		IGR	shore	IGR-shore	chr1:2507	rs5279498 36
cg0527917	0.253561	0.577969	8.59612	3.5E-05	0.001405	2.83614	0.451188	0.70475	-0.25356	17	7113697	F	II	DLG4	Body	shelf	Body-shelf	chr17:711	rs1143752 24;30;32
cg2156052	0.232357	0.607532	8.593903	3.5E-05	0.001406	2.834258	0.491353	0.72371	-0.23236	13	60308688	R	II	DIAPH3	Body	opensea	Body-opens		
cg0934173	0.220603	0.479558	8.593483	3.51E-05	0.001407	2.833902	0.369256	0.58986	-0.2206	19	40269534	R	II	LEUTX	5'UTR	opensea	5'UTR-open		rs5359749 51;16
cg0021932	0.202997	0.45099	8.593377	3.51E-05	0.001407	2.833812	0.349491	0.552488	-0.203	13	75949597	F	II	TBC1D4	Body	opensea	Body-opens		rs5680877 0;12;39;47;51
cg1419511	0.207195	0.53166	8.592263	3.51E-05	0.001407	2.832866	0.428062	0.635258	-0.2072	12	1.09E+08	R	II	SVOP	Body	opensea	Body-opens		rs1512513 40
cg1832586	0.210689	0.613036	8.592193	3.51E-05	0.001407	2.832807	0.507691	0.71838	-0.21069	10	1.17E+08	F	II	FAM160B1	Body	shelf	Body-shelf	chr10:116	rs5704991 45;48
cg1592880	0.205235	0.603619	8.591335	3.51E-05	0.001408	2.832078	0.501001	0.706236	-0.20524	3	64188875	R	II	PRICKLE2	5'UTR	opensea	5'UTR-open		rs5623044 40
cg2477184	0.22054	0.429278	8.59103	3.51E-05	0.001408	2.831819	0.319008	0.539548	-0.22054	18	47717446	F	II	MYO5B	Body	shelf	Body-shelf	chr18:477	rs5598902 4;11
cg0840762	0.234877	0.642026	8.590899	3.51E-05	0.001408	2.831708	0.524587	0.759464	-0.23488	6	24743861	R	II		IGR	opensea	IGR-opense		rs1492210 46
cg0852138	0.231831	0.558293	8.590874	3.51E-05	0.001408	2.831687	0.442378	0.674209	-0.23183	2	1.66E+08	F	II	SCN3A	Body	opensea	Body-opens		rs1904262 24;27
cg1778734	0.209104	0.680592	8.590186	3.52E-05	0.001408	2.831102	0.576041	0.785144	-0.2091	6	1.59E+08	F	II	TULP4	TSS1500	opensea	TSS1500-o		
cg1320215	0.201347	0.637593	8.589924	3.52E-05	0.001408	2.83088	0.53692	0.738267	-0.20135	2	68053707	R	II	LOC10192	TSS1500	opensea	TSS1500-o		rs5548080 1
cg0090642	0.213787	0.590724	8.588634	3.52E-05	0.001409	2.829784	0.483831	0.697618	-0.21379	3	78917133	R	II	ROBO1	Body	opensea	Body-opens		rs1847155 51;36
cg1576210	0.212034	0.504118	8.588589	3.52E-05	0.001409	2.829746	0.398101	0.610135	-0.21203	1	15558548	R	II		IGR	opensea	IGR-opense		rs5288622 33;32;27;21;19;17;13;9;6;3;2
cg0959151	0.222268	0.622829	8.588545	3.52E-05	0.001409	2.829709	0.511695	0.733963	-0.22227	6	33241770	R	I	RPS18	Body	shore	Body-shore	chr6:3323	rs1053995 35;32;4;2
cg0507491	0.21408	0.671273	8.588277	3.52E-05	0.001409	2.829481	0.564233	0.778313	-0.21408	2	2.4E+08	F	II	HDAC4	Body	opensea	Body-opens		rs5773881 1;22
cg1350295	0.222096	0.537382	8.587962	3.52E-05	0.001409	2.829214	0.426334	0.64843	-0.2221	10	92180666	F	II	LOC10192	Body	opensea	Body-opens		rs3722415 0
cg0309030	0.202	0.443392	8.58672	3.53E-05	0.00141	2.828159	0.342391	0.544392	-0.202	4	88419748	F	II	SPARCL1	Body	opensea	Body-opens		rs1175932 8
cg1231234	0.209476	0.580415	8.586271	3.53E-05	0.00141	2.827777	0.475677	0.685153	-0.20948	18	29091783	F	II	DSG2	Body	opensea	Body-opens		rs5331341 0
cg0361593	0.204181	0.660764	8.584295	3.53E-05	0.001411	2.826098	0.558674	0.762855	-0.20418	5	1.43E+08	F	II	ARHGAP26	Body	opensea	Body-opens		rs3444719 29;43
cg0917455	0.219971	0.460941	8.583001	3.54E-05	0.001412	2.824998	0.350955	0.570926	-0.21997	1	1.53E+08	F	II	S100A8	TSS1500	opensea	TSS1500-o		rs1463697 39
cg1179548	0.220227	0.549337	8.582742	3.54E-05	0.001412	2.824779	0.439223	0.65945	-0.22023	1	65038492	R	II	CACHD1	Body	opensea	Body-opens		
cg0967422	0.209678	0.556483	8.581994	3.54E-05	0.001412	2.824143	0.451644	0.661322	-0.20968	16	58914345	R	II		IGR	opensea	IGR-opense		rs5709192 13
cg2645345	0.239866	0.514	8.581944	3.54E-05	0.001412	2.8241	0.394067	0.633933	-0.23987	12	6316657	F	II	CD9	Body	opensea	Body-opens		rs1931577 3;22
cg2199854	0.2219	0.699546	8.581601	3.54E-05	0.001412	2.823809	0.588596	0.810496	-0.2219	10	33605101	F	II	NRP1	Body	opensea	Body-opens		rs1128471 1
cg1504486	0.23849	0.509327	8.58116	3.54E-05	0.001413	2.823434	0.390082	0.628572	-0.23849	3	1.36E+08	F	II	STAG1	Body	opensea	Body-opens		rs5473781 2;47
cg1149640	0.280822	0.576559	8.580703	3.54E-05	0.001413	2.823045	0.436148	0.71697	-0.28082	6	88428902	R	II		IGR	opensea	IGR-opense		rs5540577 49;17;2
cg1943977	0.219629	0.614909	8.57914	3.55E-05	0.001413	2.821717	0.505094	0.724723	-0.21963	1	2.06E+08	F	II		IGR	opensea	IGR-opense		rs5285916 0;1
cg2386767	0.209768	0.416025	8.579024	3.55E-05	0.001413	2.821618	0.311141	0.520909	-0.20977	10	73404170	F	II	CDH23	Body	opensea	Body-opens		rs1393746 0
cg0931811	0.234197	0.570762	8.578868	3.55E-05	0.001414	2.821486	0.453663	0.687861	-0.2342	3	65817280	R	II	MAGI1	Body	opensea	Body-opens		rs5624730 11
cg2454027	0.210724	0.636666	8.575689	3.56E-05	0.001415	2.818783	0.531304	0.742028	-0.21072	10	63701252	R	II	ARID5B	Body	opensea	Body-opens		rs5669949 2
cg2189888	0.228141	0.542017	8.574947	3.56E-05	0.001416	2.818151	0.427946	0.656087	-0.22814	6	22568278	F	II	HDGFL1	TSS1500	shore	TSS1500-sh	chr6:2256	rs1393940 4;47
cg0269512	0.201427	0.606342	8.574566	3.56E-05	0.001416	2.817827	0.505628	0.707055	-0.20143	5	1.5E+08	F	II	CAMK2A	Body	opensea	Body-opens		rs1402044 0;39;47
cg1076628	0.200334	0.674577	8.574184	3.56E-05	0.001416	2.817502	0.57441	0.774744	-0.20033	6	1.07E+08	F	II		IGR	opensea	IGR-opense		rs5731956 35;36
cg1624435	0.203773	0.671769	8.573402	3.56E-05	0.001416	2.816837	0.569883	0.773656	-0.20377	21	27777555	F	II		IGR	opensea	IGR-opense		rs1815828 0
cg0390058	0.208808	0.656525	8.572194	3.57E-05	0.001417	2.815809	0.552121	0.760929	-0.20881	1	96468567	R	II		IGR	opensea	IGR-opense		rs5715203 48
cg0743293	0.245553	0.571717	8.571583	3.57E-05	0.001417	2.815289	0.44894	0.694493	-0.24555	3	1.93E+08	R	II	MB21D2	Body	opensea	Body-opens		rs5722651 10;2
cg1115058	0.200394	0.531278	8.570318	3.57E-05	0.001418	2.814213	0.431081	0.631475	-0.20039	2	1.9E+08	F	II		IGR	opensea	IGR-opense		
cg0041979	0.204037	0.598147	8.570156	3.57E-05	0.001418	2.814075	0.496129	0.700166	-0.20404	7	1.37E+08	F	II	DGKI	Body	opensea	Body-opens		rs5687644 11;31;42
cg0594812	0.217154	0.658984	8.569613	3.57E-05	0.001419	2.813613	0.550407	0.767561	-0.21715	7	1.08E+08	R	II	LAMB1	Body	opensea	Body-opens		rs1405634 36;31;2;1
cg2685273	0.226302	0.660107	8.569461	3.57E-05	0.001419	2.813483	0.546956	0.773258	-0.2263	1	2.07E+08	R	II	FCAMR	TSS200	opensea	TSS200-ope		rs1478641 38
cg1527215	0.279212	0.473238	8.56823	3.58E-05	0.00142	2.812436	0.333632	0.612844	-0.27921	4	38525916	F	II	LINC01258	TSS1500	opensea	TSS1500-o		rs7313995 38
cg2451142	0.274306	0.491201	8.568085	3.58E-05	0.00142	2.812312	0.354049	0.628354	-0.27431	7	1.11E+08	R	II		IGR	opensea	IGR-opense		rs5753398 12
cg0813188	0.212924	0.520277	8.567226	3.58E-05	0.00142	2.811581	0.413815	0.626739	-0.21292	7	1.35E+08	R	II	SLC13A4	TSS1500	opensea	TSS1500-o		rs5367003 1
cg0981239	0.214249	0.551631	8.567102	3.58E-05	0.00142	2.811475	0.444507	0.658756	-0.21425	15	30078357	F	II	TJP1	Body	opensea	Body-opens		rs1507866 16
cg0959462	0.241339	0.4227	8.566529	3.58E-05	0.00142	2.810987	0.30203	0.543369	-0.24134	5	1.19E+08	R	II	TNFAIP8	TSS1500	shore	TSS1500-sh	chr5:1186	rs7610611 27;21
cg0990375	0.2298	0.581953	8.565778	3.59E-05	0.001421	2.810348	0.467053	0.696853	-0.2298	8	81312506	R	II		IGR	opensea	IGR-opense		rs1913149 18
cg1916855	0.203921	0.628648	8.564389	3.59E-05	0.001421	2.809166	0.526687	0.730608	-0.20392	6	1.32E+08	F	II		IGR	opensea	IGR-opense		rs5752364 1;44
cg0518696	0.218315	0.651051	8.564076	3.59E-05	0.001421	2.808898	0.541893	0.760209	-0.21832	5	1.21E+08	F	II		IGR	opensea	IGR-opense		
cg2434380	0.233716	0.564262	8.563947	3.59E-05	0.001421	2.808789	0.447404	0.68112	-0.23372	2	53145796	F	II		IGR	opensea	IGR-opense		rs5707733 4;6;29
cg0015484	0.209683	0.513845	8.563211	3.59E-05	0.001422	2.808162	0.409004	0.618687	-0.20968	13	47418771	R	II	HTR2A	Body	opensea	Body-opens		rs1835734 45;40
cg1186739	0.23039	0.560035	8.562827	3.59E-05	0.001422	2.807835	0.444841	0.67523	-0.23039	15	51718133	R	II		IGR	opensea	IGR-opense		rs5387749 39;4;2
cg0421758	0.202961	0.647819	8.56225	3.6E-05	0.001422	2.807343	0.546339	0.7493	-0.20296	10	44495810	F	II		IGR	opensea	IGR-opense		
cg1906106	0.256335	0.53086	8.562025	3.6E-05	0.001422	2.807152	0.402692	0.659027	-0.25634	12	43703894	R	II		IGR	opensea	IGR-opense		rs5556477 41;38;8;5;2
cg1257692	0.233914	0.667214	8.561824	3.6E-05	0.001423	2.806981	0.550257	0.784171	-0.23391	12	65980573	F	II	LOC10050	Body	opensea	Body-opens		rs1812842 0;30;33
cg1155498	0.230692	0.643263	8.560694	3.6E-05	0.001423	2.806018	0.527917	0.758609	-0.23069	14	32937945	R	II	AKAP6	Body	opensea	Body-opens		rs1163562 40;34;11

cg1440724	0.200965	0.4262	8.560553	3.6E-05	0.001423	2.805898	0.325717	0.526682	-0.20097	5	1.72E+08	R	II		IGR	shelf	IGR-shelf	chr5:17206	
cg2123294	0.211993	0.549657	8.560427	3.6E-05	0.001423	2.805791	0.44366	0.655653	-0.21199	20	33509394	R	II	ACSS2	ExonBnd	opensea	ExonBnd-op		rs2006284 19;2
cg1222443	0.23062	0.597286	8.55959	3.6E-05	0.001424	2.805077	0.481975	0.712596	-0.23062	11	10616068	F	II	MRVI1-AS1	Body	opensea	Body-opens		rs1905889 27;41;49
cg2072800	0.239701	0.514302	8.559452	3.6E-05	0.001424	2.80496	0.394451	0.634152	-0.2397	5	57637089	F	II		IGR	opensea	IGR-opense		rs7310293 0
cg0749635	0.227619	0.49518	8.557743	3.61E-05	0.001424	2.803504	0.381371	0.60899	-0.22762	9	1536641	R	II		IGR	opensea	IGR-opense		rs5664636 33;2
cg1488430	0.214246	0.669746	8.557223	3.61E-05	0.001425	2.803061	0.562623	0.776869	-0.21425	13	1.06E+08	R	II		IGR	opensea	IGR-opense		rs5531118 37;1
cg0489729	0.233854	0.429663	8.557042	3.61E-05	0.001425	2.802906	0.312735	0.54659	-0.23385	6	21402890	R	II		IGR	opensea	IGR-opense		
cg0435315	0.239029	0.49394	8.555984	3.61E-05	0.001425	2.802005	0.374425	0.613454	-0.23903	15	77722632	F	II	HMG20A	5'UTR	opensea	5'UTR-oper		rs1892357 1
cg2308084	0.246421	0.539256	8.554957	3.62E-05	0.001426	2.801129	0.416045	0.662466	-0.24642	6	16413699	R	II	ATXN1	5'UTR	opensea	5'UTR-oper		rs4716067 32
cg0452976	0.221596	0.657268	8.553356	3.62E-05	0.001427	2.799764	0.54647	0.768066	-0.2216	9	19033888	R	II	SAXO1	TSS1500	opensea	TSS1500-op		rs5510150 16
cg1340348	0.200474	0.587816	8.552798	3.62E-05	0.001427	2.799289	0.487578	0.688053	-0.20047	17	19388360	F	II		IGR	opensea	IGR-opense		
cg0602996	0.225923	0.667676	8.55273	3.62E-05	0.001427	2.79923	0.554715	0.780638	-0.22592	21	35171352	R	II	ITSN1	Body	opensea	Body-opens		rs5590944 25;1
cg0056934	0.216113	0.618288	8.552578	3.62E-05	0.001427	2.799101	0.510232	0.726344	-0.21611	3	1.01E+08	F	II	ABI3BP	Body	opensea	Body-opens		rs5620966 0;1;6
cg2635848	0.24241	0.576289	8.552132	3.63E-05	0.001427	2.798721	0.455084	0.697494	-0.24241	22	42674091	F	II		IGR	opensea	IGR-opense		
cg2255772	0.215003	0.642626	8.551585	3.63E-05	0.001428	2.798254	0.535124	0.750127	-0.215	10	60368369	R	II	BICC1	Body	opensea	Body-opens		rs5702151 14
cg1122336	0.201319	0.503558	8.551576	3.63E-05	0.001428	2.798246	0.402898	0.604217	-0.20132	6	32023102	R	II	TNXB	Body	opensea	Body-opens		rs5742560 44;31
cg1293788	0.221732	0.612073	8.551294	3.63E-05	0.001428	2.798006	0.501207	0.722939	-0.22173	20	1761383	R	II	LOC10028	TSS1500	shelf	TSS1500-sh	chr20:1757	rs1124644 46;20;18;4
cg2018449	0.200044	0.538594	8.551247	3.63E-05	0.001428	2.797966	0.438572	0.638615	-0.20004	17	8888813	F	II		IGR	opensea	IGR-opense		
cg2426166	0.206565	0.428138	8.55062	3.63E-05	0.001428	2.797432	0.324855	0.53142	-0.20657	1	1.81E+08	R	II		IGR	opensea	IGR-opense		rs5468271 12
cg0456601	0.312986	0.534443	8.550569	3.63E-05	0.001428	2.797387	0.37795	0.690936	-0.31299	5	1.68E+08	R	II	TENM2	Body	opensea	Body-opens		rs5559666 1
cg2487211	0.209827	0.61103	8.550426	3.63E-05	0.001428	2.797266	0.506116	0.715943	-0.20983	19	42356072	F	II	DMRTC2	3'UTR	opensea	3'UTR-oper		rs5339324 16;29;50
cg2018658	0.210689	0.604511	8.549417	3.63E-05	0.001429	2.796405	0.499167	0.709856	-0.21069	3	1.94E+08	R	II		IGR	opensea	IGR-opense		rs6792878 47;2
cg2644472	0.20455	0.564221	8.549228	3.63E-05	0.001429	2.796244	0.461946	0.666496	-0.20455	11	11629695	F	II	GALNT18	Body	opensea	Body-opens		rs1911071 0
cg0225357	0.230863	0.634843	8.547766	3.64E-05	0.00143	2.794997	0.519412	0.750275	-0.23086	7	29953445	F	II	WIPF3	Body	opensea	Body-opens		rs1421366 0;9;10;39
cg0974989	0.211166	0.664108	8.547641	3.64E-05	0.00143	2.794891	0.558525	0.769691	-0.21117	16	73360136	F	II		IGR	opensea	IGR-opense		rs1440503 46
cg2058378	0.212148	0.637385	8.546824	3.64E-05	0.00143	2.794193	0.53131	0.743459	-0.21215	10	52569361	R	II	A1CF	Body	opensea	Body-opens		
cg0324181	0.215248	0.625727	8.546247	3.64E-05	0.00143	2.793701	0.518103	0.733351	-0.21525	6	6731222	R	II		IGR	opensea	IGR-opense		rs1470283 17;6;1
cg0325830	0.207299	0.491391	8.542403	3.65E-05	0.001432	2.79042	0.387742	0.595041	-0.2073	13	88869717	F	II	LOC10537	Body	opensea	Body-opens		rs5357958 12;20;35;41
cg0596377	0.227943	0.548375	8.542218	3.66E-05	0.001433	2.790262	0.434403	0.662346	-0.22794	1	46573359	R	II	PIK3R3	Body	opensea	Body-opens		rs5396646 17;5;1
cg0212959	0.203887	0.669166	8.540639	3.66E-05	0.001433	2.788915	0.567223	0.771109	-0.20389	17	36624613	R	II	ARHGAP23	Body	shelf	Body-shelf	chr17:3662	rs1244970 19;7;6
cg2254407	0.215273	0.576242	8.540266	3.66E-05	0.001434	2.788595	0.468605	0.683878	-0.21527	13	98736068	R	II		IGR	opensea	IGR-opense		rs7706857 50;47;2
cg0818466	0.238657	0.656012	8.539725	3.66E-05	0.001434	2.788134	0.536684	0.775341	-0.23866	3	1.01E+08	F	II	ABI3BP	ExonBnd	opensea	ExonBnd-op		rs1469223 0;26;40
cg1362767	0.203763	0.455699	8.538777	3.67E-05	0.001435	2.787325	0.353818	0.557581	-0.20376	9	17601150	R	II	SH3GL2	Body	opensea	Body-opens		rs5741654 24;14
cg1470281	0.203233	0.561768	8.538413	3.67E-05	0.001435	2.787013	0.460151	0.663385	-0.20323	21	19894763	R	II		IGR	opensea	IGR-opense		
cg1244317	0.204654	0.671607	8.538134	3.67E-05	0.001435	2.786776	0.56928	0.773934	-0.20465	3	70905636	F	II		IGR	opensea	IGR-opense		rs7383695 25
cg1756673	0.227866	0.529948	8.535898	3.67E-05	0.001436	2.784866	0.416015	0.643881	-0.22787	15	96456154	F	II		IGR	opensea	IGR-opense		rs1153943 3
cg1113159	0.237864	0.602363	8.535731	3.67E-05	0.001436	2.784723	0.483432	0.721295	-0.23786	1	2.04E+08	F	II	PLEKHA6	Body	shelf	Body-shelf	chr1:20427	rs5732606 30
cg2154666	0.2135	0.539967	8.535582	3.68E-05	0.001437	2.784596	0.433217	0.646717	-0.2135	11	15815167	R	II		IGR	opensea	IGR-opense		rs5610332 20;14
cg1671060	0.221341	0.555356	8.534336	3.68E-05	0.001437	2.783531	0.444686	0.666026	-0.22134	15	33424752	R	II		IGR	opensea	IGR-opense		rs5397189 30
cg2406231	0.226663	0.589634	8.534155	3.68E-05	0.001437	2.783377	0.476302	0.702966	-0.22666	4	41610944	R	II	LIMCH1	Body	opensea	Body-opens		
cg0073915	0.216072	0.565698	8.533188	3.68E-05	0.001438	2.78255	0.457661	0.673734	-0.21607	17	36832423	F	II	C17orf96	TSS1500	shore	TSS1500-sh	chr17:3682	
cg0463973	0.210191	0.565708	8.532367	3.68E-05	0.001438	2.781849	0.460613	0.670804	-0.21019	4	1.42E+08	F	II		IGR	opensea	IGR-opense		
cg1044210	0.218069	0.561832	8.532006	3.69E-05	0.001438	2.78154	0.452798	0.670866	-0.21807	7	16066532	R	II		IGR	opensea	IGR-opense		rs3684453 19;10
cg0373347	0.225998	0.429402	8.528633	3.7E-05	0.001441	2.778657	0.316403	0.542401	-0.226	3	1.97E+08	R	II	SENP5	Body	opensea	Body-opens		rs3425188 44;41;31;15;13;5;1
cg0613664	0.259958	0.54497	8.527065	3.7E-05	0.001441	2.777316	0.414991	0.674949	-0.25996	7	1.49E+08	F	II	ZNF282	Body	opensea	Body-opens		
cg0901140	0.231308	0.621021	8.525794	3.7E-05	0.001442	2.77623	0.505367	0.736675	-0.23131	1	2.17E+08	R	II	ESRRG	Body	opensea	Body-opens		rs1391875 33;2;1
cg1355382	0.253558	0.532454	8.525201	3.71E-05	0.001442	2.775722	0.405675	0.659233	-0.25356	4	1.22E+08	F	II		IGR	opensea	IGR-opense		rs5728741 41
cg1697269	0.201594	0.550096	8.523434	3.71E-05	0.001444	2.774211	0.449299	0.650893	-0.20159	15	58112679	F	II		IGR	opensea	IGR-opense		rs1483841 0;1
cg2295087	0.203913	0.645926	8.52241	3.71E-05	0.001444	2.773336	0.54397	0.747883	-0.20391	4	74540238	F	II		IGR	opensea	IGR-opense		rs1810110 0;1;43
cg2670300	0.280726	0.527888	8.521565	3.72E-05	0.001444	2.772612	0.387525	0.668251	-0.28073	15	55834750	F	II	PYGO1	3'UTR	opensea	3'UTR-oper		rs1849950 11;18;19
cg0250191	0.252253	0.583502	8.520584	3.72E-05	0.001445	2.771773	0.457375	0.709628	-0.25225	2	1.09E+08	R	II	LIMS1	Body	opensea	Body-opens		rs5487182 38;1
cg2086774	0.2069	0.481172	8.520262	3.72E-05	0.001445	2.771497	0.377722	0.584622	-0.2069	11	4871813	R	II	OR51S1	TSS1500	opensea	TSS1500-op		rs1850744 51;26;2
cg1322412	0.20101	0.483792	8.519813	3.72E-05	0.001445	2.771114	0.383287	0.584296	-0.20101	1	1.54E+08	R	II	S100A16	5'UTR	opensea	5'UTR-oper		rs2016962 37
cg2680938	0.204224	0.547046	8.519424	3.72E-05	0.001445	2.770781	0.444934	0.649158	-0.20422	2	2E+08	R	II		IGR	opensea	IGR-opense		rs7908649 39;31
cg0724376	0.200581	0.404334	8.518996	3.73E-05	0.001446	2.770414	0.304043	0.504624	-0.20058	12	1.25E+08	F	II		IGR	shelf	IGR-shelf	chr12:1250	rs1387979 0
cg0688722	0.219278	0.665731	8.516794	3.73E-05	0.001447	2.76853	0.556092	0.775369	-0.21928	4	15500173	F	II	CC2D2A	Body	opensea	Body-opens		rs5405426 14;29
cg1041114	0.225192	0.624634	8.516632	3.73E-05	0.001447	2.768391	0.512038	0.73723	-0.22519	21	26549459	F	II		IGR	opensea	IGR-opense		
cg2225036	0.200747	0.657794	8.515662	3.74E-05	0.001447	2.767561	0.55742	0.758168	-0.20075	1	44483866	R	II	SLC6A9	TSS1500	opensea	TSS1500-op		rs5533994 43;1
cg0423907	0.240334	0.576632	8.51444	3.74E-05	0.001448	2.766514	0.456465	0.696799	-0.24033	1	2.1E+08	F	II		IGR	opensea	IGR-opense		rs5669311 1;3
cg2200802	0.223195	0.596934	8.51383	3.74E-05	0.001448	2.765992	0.485336	0.708531	-0.22319	3	1.75E+08	R	II	MIR548AY	Body	opensea	Body-opens		rs1808168 3;46
cg2289536	0.260654	0.474132	8.51375	3.74E-05	0.001448	2.765923	0.343805	0.604459	-0.26065	22	39574449	F	II		IGR	opensea	IGR-opense		rs1173623 32;36
cg2700230	0.216998	0.551485	8.51369	3.74E-05	0.001448	2.765872	0.442986	0.659984	-0.217	10	93335196	R	II	LOC10018	Body	opensea	Body-opens		
cg0604966	0.210825	0.430662	8.51186	3.75E-05	0.001449	2.764305	0.32525	0.536074	-0.21082	13	1.12E+08	F	II	TEX29	Body	opensea	Body-opens		rs5358439 0;7;9;42
cg2395274	0.240447	0.602159	8.510919	3.75E-05	0.00145	2.763499	0.481935	0.722382	-0.24045	17	64463393	F	II	PRKCA	Body	opensea	Body-opens		rs7822765 34;44;49
cg0386112	0.207403	0.473007	8.51061	3.75E-05	0.00145	2.763234	0.369305	0.576709	-0.2074	16	73774662	R	II		IGR	opensea	IGR-opense		rs1882093 12;2
cg2579443	0.205367	0.545033	8.507477	3.76E-05	0.001452	2.76055	0.44235	0.647717	-0.20537	16	81558090	R	II	CMIP	Body	opensea	Body-opens		rs1137353 26
cg0405866	0.209123	0.485625	8.507164	3.76E-05	0.001452	2.760282	0.381064	0.590187	-0.20912	3	70410237	F	II		IGR	opensea	IGR-opense		rs1421702 15
cg2507188	0.204305	0.620219	8.50658	3.76E-05	0.001453	2.759782	0.518067	0.722372	-0.20431	7	91273415	R	II		IGR	opensea	IGR-opense		rs1458764 49;25;2;1
cg0819156	0.235762	0.563362	8.505409	3.77E-05	0.001454	2.758778	0.445481	0.681243	-0.23576	20	33643925	R	II	TRPC4AP	Body	opensea	Body-opens		rs3776738 37;16
cg0519907	0.247471	0.627505	8.50525	3.77E-05	0.001454	2.758642	0.50377	0.751241	-0.24747	6	47217441	F	II	TNFRSF21	Body	opensea	Body-opens		rs7373638 0;15
cg2280538	0.200786	0.526895	8.504719	3.77E-05	0.001454	2.758186	0.426501	0.627288	-0.20079	14	51760188	R	II		IGR	opensea	IGR-opense		rs5461088 45;11;7;2
cg2748049	0.232152	0.486798	8.504691	3.77E-05	0.001454	2.758162	0.370722	0.602874	-0.23215	13	48784568	F	II		IGR	opensea	IGR-opense		rs5536301 51
cg1885771	0.201992	0.673488	8.504187	3.77E-05	0.001454	2.75773	0.572492	0.774483	-0.20199	10	77288339	F	II	C10orf11	Body	opensea	Body-opens		rs2115913 11
cg2111161	0.224057	0.539566	8.502439	3.78E-05	0.001455	2.756232	0.427537	0.651594	-0.22406	14	32473981	R	II		IGR	opensea	IGR-opense		rs1013530 41;33;18
cg2331631	0.281232	0.522544	8.501134	3.78E-05	0.001456	2.755113	0.381928	0.66316	-0.28123	12	21606508	R	II	PYROXD1	Body	opensea	Body-opens		
cg0934587	0.23278	0.644552	8.500303	3.78E-05	0.001456	2.754401	0.528162	0.760942	-0.23278	1	1.7E+08	R	II		IGR	opensea	IGR-opense		
cg0445275	0.210582	0.501976	8.500249	3.78E-05	0.001456	2.754354	0.396685	0.607267	-0.21058	20	24483088	R	II	SYNDIG1	5'UTR	opensea	5'UTR-oper		rs1452803 20
cg0041312	0.20317	0.645288	8.499985	3.78E-05	0.001456	2.754128	0.543704	0.746873	-0.20317	1	1.64E+08	R	II		IGR	opensea	IGR-opense		
cg0685258	0.211245	0.572631	8.499954	3.78E-05	0.001456	2.754101	0.467008	0.678254	-0.21125	5	1.35E+08	F	II		IGR	opensea	IGR-opense		rs5421992 0;1;28;36;37;48
cg2033825	0.214062	0.633855	8.499083	3.79E-05	0.001457	2.753354	0.526824	0.740886	-0.21406	12	38940501	R	II		IGR	opensea	IGR-opense		rs5274351 21
cg1331954	0.241856	0.42625	8.498016	3.79E-05	0.001458	2.752439	0.305322	0.547178	-0.24186	2	71580473	F	II	ZNF638	Body	opensea	Body-opens		rs5751349 1
cg1619515	0.205703	0.664624	8.497197	3.79E-05	0.001459	2.751737	0.561773	0.767475	-0.2057	4	1.1E+08	F	II	COL25A1	Body	opensea	Body-opens		rs1890165 0;16;46
cg1591663	0.210522	0.449299	8.497163	3.79E-05	0.001459	2.751708	0.344038	0.55456	-0.21052	13	1.06E+08	F	II		IGR	opensea	IGR-opense		rs5685196 1
cg2591541	0.239281	0.553823	8.496424	3.79E-05	0.001459	2.751074	0.434182	0.673463	-0.23928	14	1.03E+08	F	II	HSP90AA1	Body	shore	Body-shore	chr14:1025	rs1892352 16;19;28;45
cg0766930	0.212983	0.566877	8.495895	3.8E-05	0.001459	2.75062	0.460385	0.673368	-0.21298	16	54691248	F	II		IGR	opensea	IGR-opense		rs5697727 9;17;25
cg2369118	0.231426	0.49028	8.495626	3.8E-05	0.001459	2.750389	0.374567	0.605993	-0.23143	22	29305360	R	II	ZNRF3	Body	opensea	Body-opens		rs5364290 1
cg2664523	0.215277	0.605108	8.495558	3.8E-05	0.001459	2.750331	0.497469	0.712746	-0.21528	5	1.58E+08	R	II		IGR	opensea	IGR-opense		
cg2171361	0.228759	0.543967	8.49547	3.8E-05	0.001459	2.750255	0.429587	0.658346	-0.22876	4	79126719	F	II	FRAS1	Body	opensea	Body-opens		
cg1308226	0.229675	0.544483	8.494895	3.8E-05	0.001459	2.749762	0.429646	0.659321	-0.22967	1	69843439	F	II		IGR	opensea	IGR-opense		
cg0176125	0.202253	0.681463	8.493988	3.8E-05	0.00146	2.748984	0.580337	0.78259	-0.20225	14	72243817	F	II		IGR	opensea	IGR-opense		rs5660441 0;3;4;18
cg2188791	0.230621	0.578217	8.49327	3.8E-05	0.00146	2.748367	0.462906	0.693528	-0.23062	5	1.53E+08	R	II	GRIA1	Body	opensea	Body-opens		rs1158951 47;1
cg2166486	0.210244	0.64739	8.492447	3.81E-05	0.001461	2.747661	0.542268	0.752512	-0.21024	2	24537140	R	II	ITSN2	Body	opensea	Body-opens		
cg0324883	0.213543	0.564483	8.491519	3.81E-05	0.001462	2.746864	0.457712	0.671255	-0.21354	12	55864950	F	II	OR6C70	TSS1500	opensea	TSS1500-op		rs5517369 0;25
cg0342850	0.26024	0.604768	8.491467	3.81E-05	0.001462	2.74682	0.474648	0.734888	-0.26024	1	2.33E+08	F	II		IGR	opensea	IGR-opense		rs1819431 0;19
cg2262883	0.206451	0.546532	8.491183	3.81E-05	0.001462	2.746576	0.443307	0.649758	-0.20645	3	1.48E+08	R	II	AGTR1	5'UTR	opensea	5'UTR-oper		rs5673547 29
cg1364462	0.226402	0.432602	8.490867	3.81E-05	0.001462	2.746304	0.319401	0.545803	-0.2264	9	1.13E+08	R	II	PALM2	Body	shelf	Body-shelf	chr9:11254	
cg2704018	0.224246	0.393346	8.489664	3.82E-05	0.001463	2.745272	0.281223	0.505469	-0.22425	7	93820405	R	II		IGR	opensea	IGR-opense		rs5752427 33;19
cg2106348	0.220244	0.59874	8.489284	3.82E-05	0.001463	2.744946	0.488618	0.708861	-0.22024	7	1.43E+08	R	II		IGR	opensea	IGR-opense		rs5293048 29
cg0447469	0.230669	0.574326	8.486914	3.82E-05	0.001465	2.74291	0.458992	0.689661	-0.23067	17	55158407	R	II		IGR	shelf	IGR-shelf	chr17:5516	rs1401916 2
cg1637121	0.255279	0.543447	8.485322	3.83E-05	0.001466	2.741544	0.415808	0.671087	-0.25528	5	95615672	F	II	LOC10192	Body	opensea	Body-opens		rs1486079 10;34
cg1848495	0.218769	0.595602	8.485245	3.83E-05	0.001466	2.741478	0.486218	0.704987	-0.21877	3	1.49E+08	F	II	WWTR1	1stExon	opensea	1stExon-op		rs1925365 43
cg0746983	0.218317	0.600354	8.484981	3.83E-05	0.001466	2.741251	0.491195	0.709512	-0.21832	2	60703084	R	II	BCL11A	Body	opensea	Body-opens		rs7606422 40;38
cg2283319	0.2005	0.623732	8.481572	3.84E-05	0.001468	2.738322	0.523481	0.723982	-0.2005	15	90643843	F	II	IDH2	1stExon	shore	1stExon-sh	chr15:9064	rs5333871 44
cg1205334	0.209942	0.596496	8.480568	3.84E-05	0.001468	2.737459	0.491526	0.701467	-0.20994	3	72536534	F	II		IGR	opensea	IGR-opense		rs1868339 2;15;33;42
cg2606083	0.212762	0.551711	8.48027	3.85E-05	0.001469	2.737203	0.44533	0.658091	-0.21276	21	19258310	R	II	NCRNA001	TSS1500	opensea	TSS1500-op		rs5557773 1
cg0350732	0.247314	0.580596	8.479485	3.85E-05	0.001469	2.736528	0.456938	0.704253	-0.24731	16	2801952	R	I	LOC10012	Body	island	Body-island	chr16:2801	rs1865435 41;16
cg0682599	0.242671	0.57391	8.478901	3.85E-05	0.00147	2.736026	0.452575	0.695246	-0.24267	22	33074131	R	II	SYN3	Body	opensea	Body-opens		
cg0010354	0.202849	0.555661	8.477694	3.85E-05	0.00147	2.734989	0.454237	0.657086	-0.20285	2	45225295	R	II		IGR	shelf	IGR-shelf	chr2:45227	rs5330276 41;2
cg2374134	0.208786	0.573906	8.477588	3.85E-05	0.00147	2.734898	0.469513	0.678299	-0.20879	2	69427498	R	II	ANTXR1	Body	opensea	Body-opens		rs3726491 26
cg1956516	0.206807	0.642161	8.477576	3.85E-05	0.00147	2.734887	0.538758	0.745565	-0.20681	7	1.4E+08	F	II	TBXAS1	Body	opensea	Body-opens		rs5725120 38;48
cg1901117	0.208832	0.58115	8.476761	3.86E-05	0.001471	2.734186	0.476734	0.685567	-0.20883	2	1.21E+08	F	II		IGR	opensea	IGR-opense		
cg0276024	0.200774	0.674909	8.476028	3.86E-05	0.001471	2.733556	0.574522	0.775296	-0.20077	18	46086207	F	II	CTIF	5'UTR	opensea	5'UTR-oper		rs5381602 0;21;32
cg1078425	0.228247	0.63546	8.475949	3.86E-05	0.001471	2.733488	0.521337	0.749584	-0.22825	6	56711206	R	II	DST	Body	shelf	Body-shelf	chr6:56708	rs5510378 48;35
cg2308076	0.27592	0.547174	8.475238	3.86E-05	0.001471	2.732877	0.409214	0.685134	-0.27592	1	1.71E+08	R	II	FMO1	TSS1500	opensea	TSS1500-op		rs2064078 10
cg1024005	0.209897	0.519395	8.474914	3.86E-05	0.001472	2.732598	0.414394	0.624343	-0.2099	19	52256222	F	II	FPR1	TSS1500	opensea	TSS1500-op		rs2864155 21
cg0689003	0.202737	0.436877	8.474423	3.86E-05	0.001472	2.732176	0.335509	0.538245	-0.20274	4	1.47E+08	R	II	ZNF827	Body	opensea	Body-opens		rs5402008 19
cg0893358	0.236328	0.494616	8.474271	3.86E-05	0.001472	2.732046	0.376451	0.61278	-0.23633	20	8108599	F	II		IGR	opensea	IGR-opense		rs7418354 0;7;10
cg0629179	0.231047	0.442346	8.473916	3.87E-05	0.001472	2.73174	0.326823	0.55787	-0.23105	13	49323968	R	II		IGR	opensea	IGR-opense		rs5729498 20
cg2485900	0.227187	0.662797	8.473893	3.87E-05	0.001472	2.731721	0.549203	0.77639	-0.22719	3	1.28E+08	F	II		IGR	opensea	IGR-opense		rs5637943 0

cg1703184	0.217096	0.529374	8.473735	3.87E-05	0.001472	2.731584	0.420826	0.637922	-0.2171	17	71646778	F	II		IGR	opensea	IGR-opense		rs1493104	0
cg0161707	0.219027	0.474487	8.472979	3.87E-05	0.001473	2.730935	0.364973	0.584	-0.21903	7	964629	R	II	ADAP1	Body	shore	Body-shore	chr7:9659	rs1433319	35;1
cg0755057	0.202439	0.500882	8.472745	3.87E-05	0.001473	2.730733	0.399663	0.602101	-0.20244	19	4832918	F	II	TICAM1	TSS1500	shore	TSS1500-sh	chr19:483		
cg1148919	0.218035	0.411853	8.472602	3.87E-05	0.001473	2.73061	0.302836	0.520871	-0.21804	11	35993771	F	II	LDLRAD3	5'UTR	opensea	5'UTR-open		rs1465846	0;29
cg1881480	0.204968	0.660095	8.472428	3.87E-05	0.001473	2.73046	0.557611	0.762579	-0.20497	5	1.44E+08	R	II	KCTD16	Body	opensea	Body-opens		rs1499364	36;26;1
cg0636027	0.206579	0.681625	8.472036	3.87E-05	0.001473	2.730124	0.578336	0.784915	-0.20658	11	85923678	F	II		IGR	opensea	IGR-opense		rs1407758	0
cg1695472	0.207271	0.607039	8.468698	3.88E-05	0.001475	2.727251	0.503403	0.710675	-0.20727	10	3440330	F	II		IGR	opensea	IGR-opense		rs5688946	47
cg0797421	0.20313	0.67133	8.46863	3.88E-05	0.001475	2.727193	0.569765	0.772895	-0.20313	4	91879768	R	II	CCSER1	Body	opensea	Body-opens		rs1454960	47;25;1
cg0393092	0.256923	0.562283	8.468447	3.88E-05	0.001475	2.727035	0.433822	0.690745	-0.25692	3	3862703	F	II	LRRN1	5'UTR	opensea	5'UTR-open		rs5373070	1;10
cg1737118	0.233899	0.405541	8.467544	3.89E-05	0.001476	2.726258	0.288591	0.52249	-0.2339	2	61555931	R	II	USP34	Body	opensea	Body-opens		rs1406279	46;45
cg0325730	0.202412	0.564384	8.467473	3.89E-05	0.001476	2.726198	0.463178	0.66559	-0.20241	13	30722898	F	II		IGR	opensea	IGR-opense		rs1410509	1
cg2703290	0.200984	0.624843	8.466947	3.89E-05	0.001476	2.725745	0.524351	0.725335	-0.20098	13	51878551	F	II		IGR	opensea	IGR-opense		rs5526076	0
cg1099233	0.219539	0.62879	8.46408	3.9E-05	0.001478	2.723277	0.51902	0.738559	-0.21954	4	42645776	R	II	ATP8A1	Body	opensea	Body-opens		rs5757187	30;2
cg0005819	0.215197	0.517517	8.463198	3.9E-05	0.001478	2.722518	0.409919	0.625115	-0.2152	13	83873782	R	II		IGR	opensea	IGR-opense			
cg1378598	0.208294	0.553036	8.46302	3.9E-05	0.001479	2.722364	0.44889	0.657183	-0.20829	13	61533956	F	II		IGR	opensea	IGR-opense		rs1388197	1;4;44;50
cg0189306	0.254872	0.552797	8.462393	3.9E-05	0.001479	2.721824	0.42536	0.680233	-0.25487	7	90254267	F	II		IGR	opensea	IGR-opense		rs5766203	19
cg0318591	0.207994	0.641385	8.461324	3.91E-05	0.001479	2.720904	0.537388	0.745383	-0.20799	2	75369629	F	II	TACR1	Body	opensea	Body-opens		rs5502934	47
cg2477507	0.203144	0.560372	8.459751	3.91E-05	0.00148	2.719549	0.4588	0.661944	-0.20314	10	45804640	R	II	OR13A1	5'UTR	opensea	5'UTR-open		rs1510570	30;17;9;7;1
cg1466672	0.227838	0.644032	8.459735	3.91E-05	0.00148	2.719535	0.530113	0.757951	-0.22784	2	1E+08	R	II	AFF3	Body	opensea	Body-opens		rs5724600	41
cg0443782	0.205939	0.68335	8.459342	3.91E-05	0.00148	2.719197	0.58038	0.786319	-0.20594	7	1.43E+08	F	II	TAS2R40	TSS1500	opensea	TSS1500-op			
cg0983315	0.207207	0.655373	8.458333	3.92E-05	0.00148	2.718328	0.551769	0.758977	-0.20721	14	51823006	R	II		IGR	opensea	IGR-opense		rs1396948	44
cg0372427	0.205447	0.610077	8.456713	3.92E-05	0.001482	2.716932	0.507354	0.7128	-0.20545	1	56636154	R	II		IGR	opensea	IGR-opense		rs5348957	36;34;24;9
cg2449410	0.226196	0.577902	8.45495	3.93E-05	0.001483	2.715413	0.464804	0.691	-0.2262	4	48015981	F	II	CNGA1	TSS1500	shelf	TSS1500-sh	chr4:4801		
cg1566614	0.263475	0.462966	8.454258	3.93E-05	0.001483	2.714816	0.331228	0.594703	-0.26348	3	1.3E+08	F	II		IGR	shelf	IGR-shelf	chr3:1300	rs5399514	21
cg2730012	0.228592	0.583792	8.453711	3.93E-05	0.001483	2.714345	0.469496	0.698088	-0.22859	2	64713000	R	II		IGR	opensea	IGR-opense		rs9631064	21;7
cg0799287	0.222441	0.392427	8.452721	3.93E-05	0.001483	2.713492	0.281206	0.503647	-0.22244	4	88441629	R	II	SPARCL1	5'UTR	opensea	5'UTR-open			
cg2653128	0.223875	0.349115	8.451797	3.94E-05	0.001484	2.712695	0.237178	0.461053	-0.22387	13	98799358	R	II	FARP1	5'UTR	shelf	5'UTR-shelf	chr13:987	rs5514094	22;11
cg0397272	0.229265	0.480228	8.451493	3.94E-05	0.001484	2.712433	0.365596	0.59486	-0.22926	6	1.6E+08	F	II		IGR	opensea	IGR-opense		rs1500909	1;8
cg2080645	0.201798	0.647403	8.451158	3.94E-05	0.001484	2.712144	0.546504	0.748302	-0.2018	11	94480929	R	II		IGR	opensea	IGR-opense		rs1168468	2
cg1335252	0.226387	0.608214	8.451052	3.94E-05	0.001484	2.712052	0.49502	0.721407	-0.22639	5	65418807	F	II		IGR	opensea	IGR-opense		rs5700541	1;51
cg0182461	0.205222	0.495128	8.450661	3.94E-05	0.001484	2.711715	0.392517	0.597739	-0.20522	16	85315212	R	II		IGR	shelf	IGR-shelf	chr16:853	rs1852429	36;34
cg0651660	0.20644	0.631957	8.447653	3.95E-05	0.001486	2.709122	0.528738	0.735177	-0.20644	2	8661038	R	II		IGR	opensea	IGR-opense			
cg2649853	0.245361	0.523699	8.447331	3.95E-05	0.001486	2.708843	0.401019	0.646379	-0.24536	2	1.98E+08	R	II	HSPD1	Body	shore	Body-shore	chr2:1983	rs1381707	28;27;14
cg1105687	0.201793	0.563039	8.446579	3.95E-05	0.001487	2.708195	0.462142	0.663935	-0.20179	3	87007970	R	II	VGLL3	Body	opensea	Body-opens		rs5700668	50;16
cg1986731	0.209066	0.63718	8.446003	3.96E-05	0.001487	2.707698	0.532647	0.741712	-0.20907	6	56228811	F	II	RNU6-71P	Body	opensea	Body-opens		rs5508484	1;17;20;51
cg1613161	0.220294	0.522483	8.44577	3.96E-05	0.001487	2.707497	0.412336	0.63263	-0.22029	6	49312802	F	II		IGR	opensea	IGR-opense		rs5675759	0;13;20
cg0738333	0.22519	0.642964	8.444614	3.96E-05	0.001488	2.7065	0.53037	0.755559	-0.22519	1	64380349	R	II	ROR1	Body	opensea	Body-opens			
cg1016666	0.233621	0.581096	8.444035	3.96E-05	0.001488	2.706001	0.464286	0.697907	-0.23362	5	60624229	R	II		IGR	shelf	IGR-shelf	chr5:6062	rs5541660	33;2
cg0381303	0.244394	0.646052	8.443583	3.96E-05	0.001489	2.705611	0.523855	0.768249	-0.24439	6	1.37E+08	F	II	MAP7	Body	opensea	Body-opens		rs1837341	1;50;51
cg1970739	0.216779	0.495825	8.443468	3.96E-05	0.001489	2.705511	0.387436	0.604214	-0.21678	2	1.59E+08	R	II	CCDC148	Body	opensea	Body-opens			
cg0882646	0.208567	0.614259	8.443362	3.96E-05	0.001489	2.70542	0.509976	0.718543	-0.20857	2	1.05E+08	R	II		IGR	opensea	IGR-opense		rs5771715	23;1
cg0985010	0.238075	0.551108	8.443	3.96E-05	0.001489	2.705108	0.432071	0.670146	-0.23808	14	60195307	R	II	RTN1	Body	shore	Body-shore	chr14:601	rs5424686	40;26
cg2110255	0.215397	0.653947	8.442378	3.97E-05	0.001489	2.704571	0.546248	0.761645	-0.2154	8	1.22E+08	R	II		IGR	opensea	IGR-opense			
cg0253801	0.202819	0.508625	8.441174	3.97E-05	0.00149	2.703532	0.407215	0.610035	-0.20282	2	1.77E+08	R	II		IGR	opensea	IGR-opense		rs2018005	49;13
cg2450234	0.215286	0.671734	8.440504	3.97E-05	0.00149	2.702953	0.564091	0.779377	-0.21529	14	24877938	F	II	NYNRIN	Body	opensea	Body-opens		rs1398198	14;20;31
cg1626926	0.202186	0.60889	8.438935	3.98E-05	0.001491	2.701599	0.507797	0.709983	-0.20219	16	78830399	F	II	WWOX	Body	opensea	Body-opens		rs1141166	8;16;24;25;37
cg0374915	0.222464	0.564519	8.438167	3.98E-05	0.001491	2.700936	0.453287	0.675751	-0.22246	1	31155158	R	II		IGR	shelf	IGR-shelf	chr1:3115	rs5369134	14;9;2
cg0563119	0.23917	0.397423	8.438027	3.98E-05	0.001491	2.700815	0.277838	0.517008	-0.23917	6	31146417	R	II	PSORS1C3	TSS1500	shore	TSS1500-sh	chr6:3114	rs1052989	16
cg0365568	0.225924	0.570895	8.437331	3.98E-05	0.001492	2.700214	0.457932	0.683857	-0.22592	16	1508785	F	II	CLCN7	Body	shore	Body-shore	chr16:150	rs1404939	1;13;33;34;46
cg1237491	0.203625	0.576658	8.436965	3.98E-05	0.001492	2.699898	0.474845	0.67847	-0.20362	9	1.3E+08	R	II	STXBP1	Body	opensea	Body-opens		rs1843155	37;22
cg1098914	0.216896	0.614287	8.43658	3.99E-05	0.001492	2.699566	0.505839	0.722735	-0.2169	3	1.88E+08	R	II		IGR	opensea	IGR-opense		rs5700548	26
cg0807988	0.206834	0.681671	8.436159	3.99E-05	0.001492	2.699202	0.578254	0.785088	-0.20683	2	1.71E+08	R	II		IGR	opensea	IGR-opense		rs5728681	34;7;2
cg1110665	0.227176	0.614588	8.435316	3.99E-05	0.001493	2.698474	0.501	0.728176	-0.22718	4	56919047	F	II		IGR	shelf	IGR-shelf	chr4:5691	rs5652267	25
cg1337518	0.238485	0.503106	8.435029	3.99E-05	0.001493	2.698226	0.383863	0.622348	-0.23848	15	39886363	F	II	THBS1	Body	opensea	Body-opens		rs1121651	20;39;43
cg1127601	0.201887	0.470938	8.434963	3.99E-05	0.001493	2.698169	0.369995	0.571882	-0.20189	7	1.14E+08	R	II	FOXP2	Body	opensea	Body-opens		rs5630771	36
cg1369595	0.219689	0.633025	8.433918	3.99E-05	0.001494	2.697267	0.523181	0.742869	-0.21969	3	36718919	F	II		IGR	opensea	IGR-opense		rs5747639	4
cg2472795	0.266446	0.465248	8.432209	4E-05	0.001495	2.695791	0.332025	0.598471	-0.26645	1	2.08E+08	F	II	CR2	Body	opensea	Body-opens		rs5761839	0;11;28
cg2223548	0.228372	0.616204	8.432047	4E-05	0.001495	2.695651	0.502018	0.73039	-0.22837	8	1.17E+08	F	II		IGR	opensea	IGR-opense		rs5634536	0;1
cg0917866	0.209823	0.574804	8.431717	4E-05	0.001495	2.695365	0.469892	0.679716	-0.20982	8	12894534	F	II		IGR	opensea	IGR-opense		rs5628519	14;48
cg2722861	0.207155	0.671982	8.431664	4E-05	0.001495	2.695319	0.568405	0.775559	-0.20715	13	31144200	F	II		IGR	opensea	IGR-opense		rs1902912	10;28;46
cg2015250	0.203099	0.491517	8.431365	4E-05	0.001495	2.695062	0.389967	0.593067	-0.2031	13	78602831	F	II	LINC00446	Body	opensea	Body-opens		rs5455778	0;32;40;41;42;45;48
cg1109670	0.21913	0.539735	8.43113	4E-05	0.001495	2.694858	0.43017	0.649299	-0.21913	11	1.24E+08	R	II	OR8G1	TSS1500	opensea	TSS1500-op		rs5593724	28
cg1988349	0.231817	0.571588	8.430875	4E-05	0.001495	2.694638	0.455679	0.687496	-0.23182	10	34733436	F	II	PARD3	Body	opensea	Body-opens			
cg0253416	0.217669	0.573439	8.430043	4.01E-05	0.001496	2.693918	0.464604	0.682273	-0.21767	11	1.1E+08	F	II		IGR	opensea	IGR-opense		rs7632258	1
cg0157197	0.230383	0.639913	8.429958	4.01E-05	0.001496	2.693845	0.524721	0.755104	-0.23038	7	84122752	R	II		IGR	opensea	IGR-opense		rs1862025	49;2
cg0171102	0.200363	0.593964	8.429771	4.01E-05	0.001496	2.693683	0.493782	0.694145	-0.20036	20	4116458	R	II		IGR	opensea	IGR-opense		rs5612935	40;31;24
cg1042498	0.200961	0.632733	8.428968	4.01E-05	0.001497	2.69299	0.532253	0.733214	-0.20096	1	29872399	R	II		IGR	opensea	IGR-opense		rs5611094	26;2;1
cg1033016	0.207711	0.676863	8.428388	4.01E-05	0.001497	2.692488	0.573008	0.780719	-0.20771	2	2.33E+08	F	II	DIS3L2	5'UTR	shelf	5'UTR-shelf	chr2:2328	rs5504933	4;26;30;47
cg1244661	0.203201	0.567695	8.424009	4.03E-05	0.0015	2.688703	0.466095	0.669295	-0.2032	5	1.59E+08	F	II		IGR	opensea	IGR-opense		rs5475928	0;34;44
cg2514111	0.228279	0.575478	8.423912	4.03E-05	0.0015	2.688619	0.461338	0.689617	-0.22828	8	96228655	R	II	LINC01298	TSS200	opensea	TSS200-ope		rs5335000	46;22;8;2
cg1642354	0.200176	0.634022	8.422019	4.03E-05	0.001502	2.686982	0.533934	0.73411	-0.20018	7	1.27E+08	F	II	GRM8	5'UTR	opensea	5'UTR-open		rs7775021	46
cg1289553	0.204999	0.662073	8.421311	4.04E-05	0.001502	2.68637	0.559574	0.764573	-0.205	16	58063504	F	II	MMP15	Body	shelf	Body-shelf	chr16:580	rs5505787	3;9
cg2655668	0.203694	0.455512	8.420062	4.04E-05	0.001502	2.685289	0.353666	0.557359	-0.20369	2	1.03E+08	F	II	IL1R1	5'UTR	opensea	5'UTR-open		rs1386494	0;19
cg2759689	0.264391	0.52239	8.42	4.04E-05	0.001502	2.685235	0.390194	0.654585	-0.26439	16	49621173	F	II	ZNF423	Body	opensea	Body-opens		rs5494576	0;7
cg1058117	0.227188	0.655918	8.419718	4.04E-05	0.001503	2.684992	0.542324	0.769512	-0.22719	9	37221268	F	II	ZCCHC7	Body	opensea	Body-opens			
cg0443638	0.206567	0.645942	8.419315	4.04E-05	0.001503	2.684643	0.542658	0.749225	-0.20657	4	87770357	F	II	SLC10A6	5'UTR	opensea	5'UTR-open		rs5614073	8;36;50
cg1317392	0.204492	0.411774	8.419069	4.04E-05	0.001503	2.68443	0.309527	0.51402	-0.20449	2	1.67E+08	R	II	SCN1A	5'UTR	opensea	5'UTR-open		rs5404730	16
cg0554552	0.223459	0.591066	8.419061	4.04E-05	0.001503	2.684424	0.479336	0.702796	-0.22346	1	93283109	R	II		IGR	opensea	IGR-opense		rs5293888	2;1
cg2063619	0.204291	0.618412	8.419009	4.04E-05	0.001503	2.684378	0.516266	0.720557	-0.20429	10	55071675	F	II		IGR	opensea	IGR-opense		rs1883035	0;33
cg1529259	0.23451	0.399922	8.418831	4.04E-05	0.001503	2.684224	0.282667	0.517177	-0.23451	2	36374912	F	II		IGR	opensea	IGR-opense		rs5597701	1
cg0189245	0.24148	0.566735	8.418505	4.05E-05	0.001503	2.683942	0.445994	0.687475	-0.24148	1	86527360	R	II	COL24A1	Body	opensea	Body-opens		rs5685354	1
cg1515796	0.216833	0.533479	8.417641	4.05E-05	0.001504	2.683194	0.425062	0.641896	-0.21683	11	5207652	R	II		IGR	opensea	IGR-opense		rs1494810	49;34;2;1
cg2331870	0.276908	0.457967	8.41577	4.05E-05	0.001505	2.681575	0.319512	0.596421	-0.27691	7	76895988	R	II	CCDC146	Body	opensea	Body-opens			
cg0832111	0.254078	0.509153	8.413779	4.06E-05	0.001506	2.679852	0.382114	0.636192	-0.25408	5	77701852	F	II	SCAMP1	Body	opensea	Body-opens		rs1824511	0
cg1696759	0.238569	0.636035	8.412573	4.07E-05	0.001506	2.678808	0.51675	0.755319	-0.23857	7	17049064	F	II		IGR	opensea	IGR-opense			
cg0620363	0.201196	0.551027	8.411959	4.07E-05	0.001507	2.678276	0.450429	0.651626	-0.2012	10	31933573	F	II		IGR	opensea	IGR-opense		rs5412204	22;40;51
cg0738744	0.235687	0.486934	8.411307	4.07E-05	0.001507	2.677712	0.369091	0.604777	-0.23569	16	49727717	F	II	ZNF423	Body	opensea	Body-opens		rs5328897	0;1;13
cg1257442	0.200963	0.631288	8.410843	4.07E-05	0.001507	2.67731	0.530807	0.73177	-0.20096	3	5023050	R	II	BHLHE40	Body	shore	Body-shore	chr3:5024	rs5769625	42
cg2330298	0.200826	0.748616	8.410613	4.07E-05	0.001507	2.677111	0.648203	0.849029	-0.20083	6	36516650	F	II	STK38	TSS1500	shore	TSS1500-sh	chr6:3651	rs5354203	8;18
cg1702657	0.20842	0.645569	8.410046	4.07E-05	0.001508	2.67662	0.541359	0.749778	-0.20842	1	1.93E+08	R	II	LINC01031	TSS1500	opensea	TSS1500-op		rs5685140	42
cg1514055	0.243009	0.525882	8.408044	4.08E-05	0.001509	2.674885	0.404377	0.647386	-0.24301	4	10098295	F	II	WDR1	Body	opensea	Body-opens		rs1150929	0;27
cg1323995	0.204383	0.437507	8.406389	4.09E-05	0.00151	2.673452	0.335315	0.539699	-0.20438	7	1.15E+08	R	II		IGR	opensea	IGR-opense		rs1829450	1
cg0427187	0.247703	0.594405	8.405788	4.09E-05	0.001511	2.672932	0.470553	0.718256	-0.2477	2	1.58E+08	R	II		IGR	opensea	IGR-opense		rs5467230	49;42;14
cg0444336	0.2289	0.553229	8.405771	4.09E-05	0.001511	2.672916	0.43878	0.667679	-0.2289	7	50633211	F	II	DDC	TSS200	opensea	TSS200-ope		rs5372511	1
cg0743915	0.228156	0.422796	8.405274	4.09E-05	0.001511	2.672486	0.308718	0.536874	-0.22816	7	28519337	R	II	CREB5	5'UTR	opensea	5'UTR-open		rs5633455	1
cg1910295	0.217878	0.675871	8.403814	4.1E-05	0.001512	2.671221	0.566932	0.78481	-0.21788	20	5928064	F	II	TRMT6	Body	shelf	Body-shelf	chr20:593	rs5634145	4;33;42
cg2487238	0.224987	0.602614	8.403612	4.1E-05	0.001512	2.671046	0.490121	0.715108	-0.22499	17	39323742	R	II	KRTAP4-3	3'UTR	opensea	3'UTR-open		rs5438521	47;17;16;2
cg1397459	0.208217	0.506747	8.40298	4.1E-05	0.001513	2.670498	0.402639	0.610856	-0.20822	11	1.19E+08	F	II	USP2	Body	shelf	Body-shelf	chr11:119	rs5780654	27
cg0862680	0.231148	0.441888	8.401537	4.1E-05	0.001513	2.669247	0.326314	0.557461	-0.23115	7	42979643	F	II		IGR	opensea	IGR-opense		rs1808090	4
cg0981798	0.228852	0.495022	8.401307	4.1E-05	0.001514	2.669048	0.380596	0.609449	-0.22885	20	25054507	R	II		IGR	shelf	IGR-shelf	chr20:250	rs5281957	20;1
cg0486177	0.203235	0.666116	8.400923	4.11E-05	0.001514	2.668715	0.564498	0.767733	-0.20324	16	72185281	F	II	PMFBP1	Body	opensea	Body-opens		rs5766144	0;4;25
cg1027992	0.211308	0.607431	8.400533	4.11E-05	0.001514	2.668377	0.501777	0.713085	-0.21131	2	45347089	R	II		IGR	opensea	IGR-opense		rs1433526	45;39;9
cg2586340	0.215466	0.677965	8.400475	4.11E-05	0.001514	2.668327	0.570232	0.785698	-0.21547	1	31970630	R	II	LINC01225	TSS1500	opensea	TSS1500-op		rs7608142	45;42;1
cg1198643	0.203237	0.56336	8.400175	4.11E-05	0.001514	2.668067	0.461742	0.664979	-0.20324	9	1.11E+08	R	II		IGR	opensea	IGR-opense		rs1847584	9;1
cg1869769	0.230219	0.59064	8.400164	4.11E-05	0.001514	2.668057	0.47553	0.705749	-0.23022	11	58989430	F	II		IGR	opensea	IGR-opense			
cg0979825	0.248716	0.527915	8.400102	4.11E-05	0.001514	2.668003	0.403557	0.652273	-0.24872	8	59763565	R	II	TOX	Body	opensea	Body-opens		rs5524859	30
cg2213831	0.216912	0.48972	8.399276	4.11E-05	0.001514	2.667287	0.381264	0.598176	-0.21691	12	597242	R	II	B4GALNT3	Body	opensea	Body-opens		rs5502837	28;27
cg2006372	0.215646	0.680083	8.399145	4.11E-05	0.001514	2.667173	0.57226	0.787906	-0.21565	7	553423	F	II	PDGFA	Body	shelf	Body-shelf	chr7:5566	rs5627986	10
cg1854403	0.229729	0.656934	8.398697	4.11E-05	0.001515	2.666785	0.54207	0.771798	-0.22973	2	8626420	R	II		IGR	opensea	IGR-opense		rs5584468	37;16;11
cg0734537	0.220913	0.538381	8.39813	4.11E-05	0.001515	2.666293	0.427924	0.648838	-0.22091	17	76772464	R	II	CYTH1	Body	opensea	Body-opens		rs5383006	2;1
cg2286901	0.203861	0.422613	8.397001	4.12E-05	0.001516	2.665314	0.320683	0.524544	-0.20386	2	1.79E+08	R	II	OSBPL6	5'UTR	opensea	5'UTR-open		rs5435155	38;26
cg2666954	0.202201	0.549566	8.396708	4.12E-05	0.001516	2.665061	0.448466	0.650667	-0.2022	3	1.46E+08	R	II		IGR	opensea	IGR-opense		rs1685869	51
cg2510762	0.211207	0.392066	8.396099	4.12E-05	0.001516	2.664532	0.286462	0.49767	-0.21121	7	79060196	R	II	MAGI2	Body	opensea	Body-opens		rs1130323	43;38;2;1
cg1124536	0.231317	0.45662	8.395976	4.12E-05	0.001516	2.664426	0.340962	0.572278	-0.23132	18	21580850	R	II	TTC39C-AS	Body	opensea	Body-opens		rs7515169	43;23
cg2188203	0.205881	0.610637	8.39417	4.13E-05	0.001517	2.662859	0.507696	0.713578	-0.20588	7	1.25E+08	R	II	LOC10192	TSS1500	opensea	TSS1500-op		rs3689663	48;16
cg0066122	0.228763	0.525312	8.394072	4.13E-05	0.001518	2.662774	0.410931	0.639694	-0.22876	7	20166735	F	II	LOC10192	Body	opensea	Body-opens		rs5409032	0;1
cg2185241	0.27271	0.548945	8.3938	4.13E-05	0.001518	2.662538	0.41259	0.6853	-0.27271	2	2.04E+08	F	II	ICA1L	Body	opensea	Body-opens		rs5511272	1;30
cg0913330	0.206477	0.57268	8.391696	4.14E-05	0.001519	2.660712	0.469441	0.675918	-0.20648	20	59915284	F	II	CDH4	Body	opensea	Body-opens		rs5671119	1;25
cg1257266	0.224076	0.63838	8.39165	4.14E-05	0.001519	2.660672	0.526342	0.750418	-0.22408	2	72788795	R	II	EXOC6B	Body	opensea	Body-opens		rs5449612	1

cg0868253	0.200347	0.61843	8.39087	4.14E-05	0.00152	2.659995	0.518256	0.718604	-0.20035	8	95262502	F	II	GEM	3'UTR	opensea	3'UTR-oper		rs3676077 0;15;26;41
cg0475107	0.236373	0.522119	8.3898	4.14E-05	0.00152	2.659067	0.403932	0.640305	-0.23637	2	1.66E+08	R	II		IGR	opensea	IGR-opense		rs5431236 37;1
cg2304845	0.204478	0.409355	8.387726	4.15E-05	0.001521	2.657267	0.307117	0.511594	-0.20448	5	1.76E+08	F	II	LOC64320:	Body	opensea	Body-opens		rs5669505 51
cg2612941	0.240214	0.55553	8.387042	4.15E-05	0.001522	2.656673	0.435423	0.675637	-0.24021	8	16015402	R	II	MSR1	Body	opensea	Body-opens		rs5610676 43;14
cg0210800	0.239833	0.43403	8.386459	4.15E-05	0.001522	2.656166	0.314113	0.553946	-0.23983	7	10000666	F	II		IGR	opensea	IGR-opense		rs5308826 0;36;43
cg2060345	0.253365	0.599577	8.385865	4.16E-05	0.001522	2.65565	0.472894	0.726259	-0.25336	9	1.04E+08	R	II		IGR	opensea	IGR-opense		rs5546834 8
cg0290723	0.242174	0.467869	8.38574	4.16E-05	0.001522	2.655542	0.346782	0.588955	-0.24217	1	2.45E+08	R	II	EFCAB2	Body	opensea	Body-opens		rs5501904 46
cg0681099	0.205677	0.645161	8.385122	4.16E-05	0.001523	2.655006	0.542322	0.747999	-0.20568	7	90795434	F	II	CDK14	3'UTR	opensea	3'UTR-oper		rs1817561 1;18
cg0705385	0.214345	0.567595	8.384947	4.16E-05	0.001523	2.654853	0.460422	0.674767	-0.21434	17	61352831	R	II	TANC2	Body	opensea	Body-opens		rs5502608 30;2
cg0434983	0.23709	0.517672	8.38314	4.17E-05	0.001523	2.653284	0.399127	0.636217	-0.23709	13	40257078	F	II	COG6	Body	opensea	Body-opens		rs5613422 10;20;26;36;49
cg0390635	0.226635	0.64152	8.383058	4.17E-05	0.001523	2.653212	0.528202	0.754838	-0.22664	4	1.57E+08	F	II		IGR	opensea	IGR-opense		rs5347112 15;17;27;43
cg0719783	0.209427	0.373428	8.381174	4.17E-05	0.001524	2.651577	0.268714	0.478141	-0.20943	2	27503157	F	II	DNAJC5G	3'UTR	opensea	3'UTR-oper		rs5668279 48
cg0200871	0.233757	0.595506	8.380829	4.17E-05	0.001524	2.651277	0.478628	0.712385	-0.23376	6	1.33E+08	F	II	LINC00326	Body	opensea	Body-opens		rs1929208 0;1;48
cg1579664	0.235204	0.535551	8.380286	4.18E-05	0.001525	2.650805	0.417949	0.653153	-0.2352	1	2.06E+08	F	II	SLC26A9	TSS1500	opensea	TSS1500-op		rs4951030 38;46
cg2569539	0.211372	0.481072	8.37891	4.18E-05	0.001526	2.649609	0.375386	0.586758	-0.21137	7	28077169	R	II	JAZF1	Body	opensea	Body-opens		rs1379729 2
cg1158746	0.204712	0.642974	8.378885	4.18E-05	0.001526	2.649588	0.540618	0.74533	-0.20471	1	2.46E+08	F	II	KIF26B	Body	shelf	Body-shelf	chr1:2458!	rs1136656 1
cg0446292	0.200927	0.54389	8.378453	4.18E-05	0.001526	2.649212	0.443426	0.644353	-0.20093	8	1.28E+08	F	II		IGR	opensea	IGR-opense		rs1445529 51
cg0087805	0.203628	0.619879	8.378033	4.18E-05	0.001526	2.648847	0.518065	0.721693	-0.20363	2	1.36E+08	R	II		IGR	opensea	IGR-opense		rs1843174 35;10
cg0219814	0.240575	0.545778	8.377773	4.18E-05	0.001526	2.648621	0.425491	0.666066	-0.24057	16	85462102	R	I		IGR	shelf	IGR-shelf	chr16:854(	rs1490565 26;24;22;16;16;15
cg0257365	0.239343	0.41142	8.377311	4.19E-05	0.001527	2.64822	0.291749	0.531092	-0.23934	13	78090644	R	II		IGR	opensea	IGR-opense		rs1506552 17
cg2359628	0.211582	0.500166	8.377054	4.19E-05	0.001527	2.647996	0.394375	0.605957	-0.21158	15	88797039	F	II	NTRK3-AS1	Body	shelf	Body-shelf	chr15:887!	rs1402692 33;39;41
cg1599033	0.209942	0.698456	8.376865	4.19E-05	0.001527	2.647832	0.593485	0.803427	-0.20994	11	1.12E+08	R	II	LOC28314(	Body	opensea	Body-opens		
cg2569523	0.207704	0.42163	8.376524	4.19E-05	0.001527	2.647536	0.317778	0.525482	-0.2077	2	2.02E+08	F	II	ALS2CR11	Body	opensea	Body-opens		
cg0444421	0.209823	0.589548	8.376201	4.19E-05	0.001527	2.647255	0.484636	0.694459	-0.20982	3	77666842	F	II	ROBO2	Body	opensea	Body-opens		
cg1282904	0.201029	0.463863	8.374934	4.19E-05	0.001528	2.646153	0.363348	0.564377	-0.20103	10	77228852	F	II		IGR	opensea	IGR-opense		rs5338317 1;30
cg2461998	0.223491	0.564451	8.3746	4.2E-05	0.001528	2.645863	0.452705	0.676197	-0.22349	4	28094243	F	II		IGR	opensea	IGR-opense		rs5493168 0;21
cg0390867	0.22378	0.546518	8.374167	4.2E-05	0.001528	2.645487	0.434628	0.658408	-0.22378	11	10590282	F	II	LYVE1	1stExon	opensea	1stExon-op		rs5372747 0;12
cg1002218	0.216968	0.578619	8.372585	4.2E-05	0.001529	2.644111	0.470134	0.687103	-0.21697	5	1.58E+08	R	II	EBF1	Body	opensea	Body-opens		rs5906405 24;7;2
cg1501882	0.207854	0.683252	8.370741	4.21E-05	0.00153	2.642507	0.579325	0.787179	-0.20785	1	24678976	R	II	GRHL3	Body	opensea	Body-opens		
cg1575852	0.250244	0.524205	8.370109	4.21E-05	0.00153	2.641957	0.399083	0.649327	-0.25024	7	1.4E+08	F	II		IGR	opensea	IGR-opense		rs5512579 7
cg2298449	0.20904	0.689124	8.370026	4.21E-05	0.00153	2.641885	0.584604	0.793644	-0.20904	5	1.15E+08	R	II	LVRN	Body	opensea	Body-opens		rs5595459 31;16
cg1511311	0.214427	0.499573	8.369762	4.21E-05	0.00153	2.641656	0.39236	0.606787	-0.21443	12	5724577	R	II	ANO2	Body	opensea	Body-opens		rs5494856 13;2
cg2652176	0.232043	0.596771	8.369499	4.21E-05	0.001531	2.641427	0.48075	0.712793	-0.23204	11	59295398	F	II		IGR	opensea	IGR-opense		
cg2133242	0.208238	0.580756	8.369113	4.21E-05	0.001531	2.641091	0.476637	0.684875	-0.20824	3	58191826	F	II	DNASE1L3	Body	opensea	Body-opens		
cg2571614	0.210009	0.542395	8.368655	4.22E-05	0.001531	2.640693	0.437391	0.6474	-0.21001	3	33763505	R	II		IGR	shelf	IGR-shelf	chr3:3375!	rs5389048 46;1
cg0571825	0.218375	0.592338	8.366791	4.22E-05	0.001533	2.639071	0.483151	0.701526	-0.21838	22	35790141	F	II	HMOX1	3'UTR	opensea	3'UTR-oper		rs1924344 1;7;27;33
cg1862204	0.243304	0.563326	8.366787	4.22E-05	0.001533	2.639068	0.441674	0.684978	-0.2433	1	1.75E+08	F	II	RABGAP1L	Body	opensea	Body-opens		rs5704418 5;11
cg1295628	0.207713	0.534007	8.366627	4.22E-05	0.001533	2.638928	0.43015	0.637864	-0.20771	1	1.16E+08	F	II		IGR	opensea	IGR-opense		rs5664741 0;1;16;46
cg0773471	0.21567	0.583533	8.365684	4.23E-05	0.001533	2.638108	0.475698	0.691368	-0.21567	13	85420495	F	II		IGR	opensea	IGR-opense		
cg2694796	0.243656	0.613013	8.364509	4.23E-05	0.001534	2.637085	0.491185	0.734841	-0.24366	17	54334619	F	II	ANKFN1	Body	opensea	Body-opens		rs1927016 0;8;50
cg1215999	0.231461	0.611758	8.364301	4.23E-05	0.001534	2.636904	0.496027	0.727489	-0.23146	12	1.02E+08	R	II	MYBPC1	Body	opensea	Body-opens		rs5301977 20;11;1
cg2594115	0.203189	0.536703	8.363129	4.24E-05	0.001534	2.635884	0.435108	0.638297	-0.20319	11	88910913	F	II	TYR	TSS200	opensea	TSS200-ope		rs5604534 1;10;43
cg0361979	0.329609	0.508557	8.362517	4.24E-05	0.001535	2.635351	0.343753	0.673361	-0.32961	2	2.35E+08	R	II		IGR	opensea	IGR-opense		rs4616477 24;1
cg1232016	0.211937	0.687177	8.361564	4.24E-05	0.001536	2.634522	0.581209	0.793146	-0.21194	17	58166202	R	II	LOC645638	TSS1500	opensea	TSS1500-op		
cg2629645	0.204503	0.598709	8.360822	4.24E-05	0.001536	2.633875	0.496457	0.70096	-0.2045	1	95198014	R	II	LINC01057	Body	opensea	Body-opens		rs5335966 45;30;20
cg2689691	0.209902	0.603533	8.357756	4.25E-05	0.001539	2.631206	0.498582	0.708484	-0.2099	3	45986498	R	II	CXCR6	5'UTR	opensea	5'UTR-oper		rs5480993 35;26
cg1091106	0.225239	0.449963	8.356425	4.26E-05	0.00154	2.630046	0.337343	0.562582	-0.22524	8	39960940	R	II		IGR	opensea	IGR-opense		rs5765358 47
cg0501141	0.209327	0.69619	8.356139	4.26E-05	0.00154	2.629796	0.591526	0.800854	-0.20933	14	51394359	R	II	PYGL	Body	opensea	Body-opens		
cg1658163	0.222373	0.524691	8.355281	4.26E-05	0.00154	2.629049	0.413505	0.635878	-0.22237	8	83170486	R	II		IGR	opensea	IGR-opense		rs5635237 27;25;16;8
cg2353703	0.215525	0.616028	8.3547	4.27E-05	0.001541	2.628543	0.508266	0.723791	-0.21552	6	24165998	F	II		IGR	opensea	IGR-opense		rs1402225 0;32
cg0888649	0.20169	0.591524	8.353755	4.27E-05	0.001541	2.62772	0.490679	0.692369	-0.20169	20	9197927	F	II	PLCB4	TSS200	opensea	TSS200-ope		rs2014272 24
cg0219342	0.202054	0.58837	8.353308	4.27E-05	0.001541	2.62733	0.487343	0.689397	-0.20205	6	3848280	R	II	FAM50B	TSS1500	shore	TSS1500-sh	chr6:3849:	rs1897350 39;18
cg1837985	0.230679	0.602353	8.353244	4.27E-05	0.001541	2.627274	0.487014	0.717693	-0.23068	15	52851536	R	II	ARPP19	Body	opensea	Body-opens		rs1430961 19
cg2624706	0.216346	0.555115	8.352019	4.27E-05	0.001543	2.626207	0.446942	0.663288	-0.21635	11	74466198	F	II	RNF169	Body	opensea	Body-opens		rs5779155 9;13;18;25
cg1380579	0.200369	0.714307	8.351003	4.28E-05	0.001543	2.625321	0.614123	0.814491	-0.20037	10	54515952	R	II		IGR	opensea	IGR-opense		rs5486821 38;1
cg2068792	0.254529	0.479839	8.350823	4.28E-05	0.001543	2.625164	0.352575	0.607103	-0.25453	8	88950581	R	II		IGR	opensea	IGR-opense		rs5694518 1
cg1628448	0.201673	0.548594	8.350788	4.28E-05	0.001543	2.625134	0.447757	0.64943	-0.20167	16	80266015	R	II	LOC10272(	Body	opensea	Body-opens		rs7990692 44;2
cg2435463	0.214541	0.551628	8.350384	4.28E-05	0.001543	2.624782	0.444358	0.658899	-0.21454	12	81771643	R	II	PPFIA2	Body	opensea	Body-opens		rs1921790 2;1
cg1916714	0.203119	0.506607	8.348742	4.29E-05	0.001544	2.62335	0.405047	0.608167	-0.20312	4	41299998	R	II		IGR	opensea	IGR-opense		rs5373096 2
cg1660465	0.239694	0.670195	8.347925	4.29E-05	0.001544	2.622638	0.550348	0.790041	-0.23969	12	64847188	F	II	TBK1	5'UTR	shore	5'UTR-shor(	chr12:648(	rs1432481 4;14;35
cg2648867	0.211413	0.584053	8.346843	4.29E-05	0.001545	2.621694	0.478347	0.689759	-0.21141	7	24301747	F	II		IGR	opensea	IGR-opense		rs1114251 1;20
cg1100983	0.20591	0.469831	8.343238	4.31E-05	0.001547	2.61855	0.366876	0.572786	-0.20591	9	85066797	R	II		IGR	opensea	IGR-opense		rs1379891 1
cg1623918	0.216811	0.494721	8.34189	4.31E-05	0.001548	2.617374	0.386316	0.603127	-0.21681	6	1.35E+08	F	II		IGR	opensea	IGR-opense		rs5473019 0;25;40
cg0118557	0.211162	0.563234	8.34157	4.31E-05	0.001548	2.617095	0.457653	0.668815	-0.21116	3	1.35E+08	R	II		IGR	opensea	IGR-opense		rs5669317 35;1
cg2234168	0.246661	0.630863	8.339278	4.32E-05	0.00155	2.615094	0.507532	0.754193	-0.24666	15	61342360	R	II	RORA	Body	opensea	Body-opens		
cg1880127	0.201264	0.728982	8.33769	4.33E-05	0.001551	2.613708	0.62835	0.829614	-0.20126	8	1.09E+08	R	II	EIF3E	Body	opensea	Body-opens		rs1892737 39;21;16;15;11;8
cg1648161	0.220064	0.578658	8.336426	4.33E-05	0.001553	2.612605	0.468626	0.688689	-0.22006	17	64225731	F	II	APOH	TSS200	opensea	TSS200-ope		rs5395602 1;17
cg1187223	0.234905	0.503406	8.335741	4.33E-05	0.001553	2.612007	0.385954	0.620859	-0.2349	2	1.45E+08	F	II	GTDC1	5'UTR	opensea	5'UTR-oper		rs1890021 0;8;26;30;49
cg2545657	0.202771	0.688614	8.335488	4.33E-05	0.001553	2.611786	0.587229	0.79	-0.20277	1	1.53E+08	F	II		IGR	opensea	IGR-opense		rs3760459 18
cg2167885	0.213639	0.694923	8.335146	4.34E-05	0.001553	2.611487	0.588104	0.801743	-0.21364	5	1.32E+08	R	II		IGR	opensea	IGR-opense		rs1818203 27;9
cg024121C	0.205407	0.472715	8.334744	4.34E-05	0.001554	2.611136	0.370012	0.575419	-0.20541	18	3367383	R	II		IGR	opensea	IGR-opense		rs5765360 33;28
cg0294818	0.201873	0.65735	8.334562	4.34E-05	0.001554	2.610978	0.556414	0.758286	-0.20187	10	33272417	R	II		IGR	shelf	IGR-shelf	chr10:332(	rs5762289 2
cg0491422	0.22643	0.591282	8.331188	4.35E-05	0.001556	2.608031	0.478067	0.704496	-0.22643	15	63116514	F	II	TLN2	Body	opensea	Body-opens		rs5278698 18
cg2437621	0.274837	0.501798	8.331023	4.35E-05	0.001556	2.607886	0.364379	0.639216	-0.27484	19	38918135	R	II	RASGRP4	TSS1500	opensea	TSS1500-op		rs5374606 22;8;2
cg1290647	0.208026	0.647569	8.330391	4.35E-05	0.001556	2.607334	0.543557	0.751582	-0.20803	10	27991214	F	II	MKX	Body	opensea	Body-opens		
cg1386454	0.211556	0.495751	8.32991	4.35E-05	0.001556	2.606914	0.389973	0.60153	-0.21156	6	1.18E+08	F	II		IGR	opensea	IGR-opense		
cg149134C	0.236168	0.66194	8.328303	4.36E-05	0.001557	2.60551	0.543856	0.780024	-0.23617	1	2.28E+08	R	II	SNAP47	Body	opensea	Body-opens		rs5562415 36;34;8
cg0423278	0.200651	0.766031	8.328137	4.36E-05	0.001557	2.605365	0.665705	0.866356	-0.20065	12	24513927	R	II	SOX5	5'UTR	opensea	5'UTR-oper		rs5406234 45;1
cg1336021	0.207819	0.611928	8.326431	4.37E-05	0.001558	2.603874	0.508018	0.715838	-0.20782	18	67997425	F	II	SOCS6	3'UTR	opensea	3'UTR-oper		rs5750107 0;1;43
cg1066025	0.221969	0.592788	8.325501	4.37E-05	0.001559	2.603061	0.481803	0.703773	-0.22197	12	7940546	F	II	NANOG	TSS1500	opensea	TSS1500-op		
cg0042284	0.209566	0.507911	8.324389	4.37E-05	0.00156	2.602089	0.403128	0.612694	-0.20957	18	11204146	R	II		IGR	opensea	IGR-opense		rs5780993 29;22;1
cg1566101	0.208216	0.616627	8.324366	4.37E-05	0.00156	2.602069	0.512519	0.720735	-0.20822	7	1.55E+08	R	II	RBM33	Body	opensea	Body-opens		rs5502210 0;18
cg023771C	0.211087	0.477536	8.324057	4.38E-05	0.00156	2.601799	0.371992	0.583079	-0.21109	7	1.35E+08	F	II		IGR	opensea	IGR-opense		rs7796064 11;18;27;33
cg0299693	0.213725	0.664589	8.323853	4.38E-05	0.00156	2.601621	0.557726	0.771452	-0.21373	2	1.66E+08	R	II	SCN3A	5'UTR	opensea	5'UTR-oper		rs5500265 38;29
cg1598631	0.242392	0.456784	8.323691	4.38E-05	0.00156	2.601479	0.335588	0.577981	-0.24239	6	24256161	R	II	DCDC2	Body	opensea	Body-opens		
cg1053283	0.207387	0.664183	8.323476	4.38E-05	0.00156	2.601291	0.560489	0.767876	-0.20739	5	1.47E+08	R	II	SPINK5	TSS1500	opensea	TSS1500-op		rs7997783 48;14
cg0180554	0.211148	0.302305	8.323452	4.38E-05	0.00156	2.60127	0.196731	0.407879	-0.21115	21	42626439	R	II	BACE2	Body	opensea	Body-opens		rs1146954 40
cg0465751	0.217196	0.722501	8.32246	4.38E-05	0.001561	2.600403	0.613903	0.831099	-0.2172	6	89351153	R	II	RNGTT	Body	opensea	Body-opens		rs1688098 42;1
cg0067608	0.234566	0.547295	8.322148	4.38E-05	0.001561	2.60013	0.430012	0.664578	-0.23457	12	52991945	R	II	KRT72	Body	shelf	Body-shelf	chr12:529!	rs5555223 2;1
cg1731447	0.222107	0.59472	8.320315	4.39E-05	0.001562	2.598527	0.483666	0.705773	-0.22211	17	34087282	F	II	C17orf50	TSS1500	shelf	TSS1500-sh	chr17:340!	rs5877294 7;16;46
cg1426334	0.276472	0.464495	8.319655	4.39E-05	0.001563	2.59795	0.326259	0.602731	-0.27647	7	1.51E+08	F	II	RHEB	Body	opensea	Body-opens		rs1150018 17;18
cg2053493	0.213074	0.489734	8.319589	4.39E-05	0.001563	2.597892	0.383197	0.596271	-0.21307	15	71972294	F	II	THSD4	Body	opensea	Body-opens		rs5433646 47
cg0069528	0.216017	0.636693	8.31876	4.39E-05	0.001563	2.597167	0.528685	0.744702	-0.21602	6	21354634	F	II		IGR	opensea	IGR-opense		rs1407304 1;45
cg0201085	0.20656	0.63061	8.318072	4.4E-05	0.001564	2.596565	0.52733	0.733889	-0.20656	9	1.26E+08	R	II	CRB2	TSS200	opensea	TSS200-ope		rs5356891 45;43;28;1
cg0949154	0.22963	0.647231	8.317535	4.4E-05	0.001564	2.596095	0.532416	0.762046	-0.22963	6	1.55E+08	R	II		IGR	shelf	IGR-shelf	chr6:1548:	rs1394675 7;4
cg2473842	0.213249	0.521274	8.317035	4.4E-05	0.001564	2.595658	0.414649	0.627898	-0.21325	6	1.69E+08	F	II		IGR	opensea	IGR-opense		rs1156358 0;1
cg1305344	0.240164	0.396611	8.316471	4.4E-05	0.001564	2.595165	0.276529	0.516692	-0.24016	17	45002105	F	II	GOSR2	Body	shore	Body-shore	chr17:450(	rs5401753 1
cg1010928	0.213116	0.539682	8.315928	4.41E-05	0.001565	2.594689	0.433124	0.64624	-0.21312	4	1.13E+08	F	II		IGR	opensea	IGR-opense		rs326837 48
cg2118582	0.209868	0.544849	8.314082	4.41E-05	0.001565	2.593074	0.439915	0.649783	-0.20987	14	85984042	F	II		IGR	opensea	IGR-opense		rs5551921 19
cg1312971	0.214059	0.530318	8.313841	4.41E-05	0.001565	2.592863	0.423288	0.637348	-0.21406	6	65014005	F	II	EYS	Body	opensea	Body-opens		rs5344324 1
cg2222353	0.205529	0.487495	8.313684	4.41E-05	0.001565	2.592725	0.38473	0.59026	-0.20553	15	92977495	F	II	ST8SIA2	ExonBnd	opensea	ExonBnd-o		rs1422336 1;43
cg2411298	0.219521	0.696025	8.312619	4.42E-05	0.001566	2.591793	0.586264	0.805785	-0.21952	9	1.21E+08	R	II		IGR	opensea	IGR-opense		rs5760698 45;28
cg0946256	0.225146	0.409787	8.311413	4.42E-05	0.001567	2.590737	0.297214	0.52236	-0.22515	6	1.59E+08	R	II		IGR	opensea	IGR-opense		rs5410837 39;22;20;5
cg2520695	0.222132	0.552492	8.311357	4.42E-05	0.001567	2.590689	0.441426	0.663558	-0.22213	21	40219706	R	II		IGR	opensea	IGR-opense		rs8012854 1
cg2498099	0.202106	0.588146	8.311195	4.42E-05	0.001567	2.590547	0.487093	0.6892	-0.20211	2	2.18E+08	F	II	DIRC3	Body	opensea	Body-opens		
cg096403C	0.226825	0.672713	8.310157	4.43E-05	0.001567	2.589638	0.5593	0.786125	-0.22683	6	7641144	F	II		IGR	opensea	IGR-opense		rs5319618 1
cg2137318	0.223087	0.633691	8.309964	4.43E-05	0.001567	2.589469	0.522148	0.745235	-0.22309	8	32559783	R	II	NRG1	Body	opensea	Body-opens		rs5683313 20;1
cg102816C	0.226171	0.561966	8.309639	4.43E-05	0.001568	2.589185	0.44888	0.675051	-0.22617	22	40732359	F	II		IGR	opensea	IGR-opense		rs5655206 33;38;43;47
cg0066001	0.225595	0.599897	8.308571	4.43E-05	0.001569	2.58825	0.487099	0.712694	-0.22559	2	1.25E+08	R	II	CNTNAP5	Body	opensea	Body-opens		rs1860893 32
cg0187653	0.241695	0.446944	8.30835	4.43E-05	0.001569	2.588056	0.326096	0.567792	-0.2417	1	2.08E+08	R	II	PLXNA2	5'UTR	opensea	5'UTR-oper		rs5768552 48
cg0132369	0.281282	0.401416	8.307829	4.44E-05	0.001569	2.587599	0.260775	0.542057	-0.28128	3	52705086	R	II	PBRM1	Body	opensea	Body-opens		rs1144923 2
cg0092685	0.230979	0.508975	8.305662	4.44E-05	0.001571	2.585701	0.393486	0.624465	-0.23098	5	80037111	R	II	MSH3	Body	opensea	Body-opens		rs1808152 50;21;17;3;1
cg1344708	0.214343	0.550579	8.305046	4.45E-05	0.001572	2.585162	0.443407	0.657751	-0.21434	8	1.31E+08	F	II		IGR	opensea	IGR-opense		rs1407191 36
cg0856258	0.206884	0.693014	8.304223	4.45E-05	0.001572	2.584441	0.589572	0.796456	-0.20688	9	1.18E+08	R	II	TNC	Body	opensea	Body-opens		rs1153530 37
cg1865775	0.257003	0.521473	8.303765	4.45E-05	0.001572	2.58404	0.392972	0.649975	-0.257	17	9834056	F	II	GAS7	Body	opensea	Body-opens		rs5534885 1;7;42;51
cg031121C	0.209479	0.429509	8.303631	4.45E-05	0.001572	2.583922	0.324769	0.534248	-0.20948	3	55469470	F	II		IGR	opensea	IGR-opense		rs1862543 0;41;44
cg0010809	0.215928	0.511586	8.29961	4.47E-05	0.001576	2.580398	0.403622	0.61955	-0.21593	3	1.23E+08	F	II	SEMA5B	Body	shelf	Body-shelf	chr3:1226(	rs1473692 40;51
cg0655166	0.222061	0.631366	8.299375	4.47E-05	0.001576	2.580192	0.520336	0.742397	-0.22206	10	89366915	F	II		IGR	opensea	IGR-opense		rs1999391 23
cg066152C	0.21662	0.532092	8.299005	4.47E-05	0.001576	2.579867	0.423782	0.640402	-0.21662	6	1.64E+08	R	II		IGR	opensea	IGR-opense		

cg1096644	0.23241	0.593385	8.298815	4.47E-05	0.001576	2.579701	0.47718	0.70959	-0.23241	11	1.33E+08	R	II	OPCML	Body	opensea	Body-opens		rs5435359	47;22;1;1
cg2272422	0.227939	0.564392	8.296284	4.48E-05	0.001578	2.577482	0.450422	0.678361	-0.22794	6	1.55E+08	F	II	CNKSR3	Body	shelf	Body-shelf	chr6:1548	rs1462925	1
cg2348559	0.239265	0.608677	8.295656	4.48E-05	0.001578	2.576931	0.489045	0.728309	-0.23926	7	25601773	F	II		IGR	opensea	IGR-opense		rs3718350	1;39
cg0234310	0.205811	0.571216	8.295613	4.48E-05	0.001578	2.576894	0.46831	0.674121	-0.20581	3	1.97E+08	F	II		IGR	opensea	IGR-opense		rs1114800	6;15
cg2311823	0.208627	0.638216	8.292978	4.49E-05	0.00158	2.574582	0.533902	0.742529	-0.20863	6	14232628	R	II		IGR	opensea	IGR-opense		rs3769729	49
cg2061736	0.203738	0.651491	8.292675	4.49E-05	0.00158	2.574316	0.549622	0.75336	-0.20374	6	56611699	R	II	RNU6-71P	Body	opensea	Body-opens		rs4520018	51;1
cg0057649	0.223953	0.706114	8.292066	4.49E-05	0.00158	2.573782	0.594137	0.818091	-0.22395	6	1.41E+08	R	II		IGR	opensea	IGR-opense			
cg1752966	0.205912	0.392363	8.290932	4.5E-05	0.001581	2.572787	0.289407	0.495319	-0.20591	4	1.15E+08	F	II		IGR	opensea	IGR-opense		rs1914994	2;42
cg0462533	0.232726	0.559324	8.290528	4.5E-05	0.001581	2.572433	0.442961	0.675686	-0.23273	7	55141971	R	I	EGFR	Body	opensea	Body-opens		rs5754260	37
cg1536290	0.208957	0.390878	8.28943	4.5E-05	0.001582	2.571469	0.2864	0.495357	-0.20896	3	1.46E+08	F	II		IGR	opensea	IGR-opense		rs1131061	1;29;32
cg2258905	0.224196	0.548937	8.288704	4.51E-05	0.001582	2.570832	0.436839	0.661035	-0.2242	8	86973512	R	II		IGR	opensea	IGR-opense			
cg2101677	0.202847	0.681791	8.288172	4.51E-05	0.001582	2.570365	0.580368	0.783215	-0.20285	15	65805260	F	II	DPP8	5'UTR	opensea	5'UTR-oper		rs5655750	3;6
cg2718405	0.204834	0.460888	8.287823	4.51E-05	0.001583	2.570059	0.358472	0.563305	-0.20483	9	1.14E+08	R	II		IGR	opensea	IGR-opense		rs1176204	12;9
cg0024515	0.223347	0.564189	8.287467	4.51E-05	0.001583	2.569746	0.452515	0.675862	-0.22335	15	82243353	F	II		IGR	opensea	IGR-opense		rs5680560	0;41
cg1501900	0.224307	0.565185	8.28601	4.52E-05	0.001584	2.568467	0.453032	0.677338	-0.22431	6	33096303	R	II	HLA-DPB2	Body	opensea	Body-opens		rs2836107	47;31
cg1010315	0.229905	0.686933	8.285666	4.52E-05	0.001585	2.568165	0.571981	0.801885	-0.2299	15	69659778	R	II	PAQR5	Body	opensea	Body-opens		rs1917555	40;32;2;1
cg0890623	0.205476	0.585363	8.285189	4.52E-05	0.001585	2.567747	0.482625	0.688101	-0.20548	7	47475653	F	II	TNS3	Body	opensea	Body-opens		rs5285631	0;1;23;33;41
cg0332012	0.205442	0.347965	8.285171	4.52E-05	0.001585	2.567731	0.245244	0.450686	-0.20544	15	28330686	F	II	OCA2	5'UTR	opensea	5'UTR-oper		rs1456822	1;33
cg0689539	0.204355	0.575189	8.28462	4.52E-05	0.001585	2.567247	0.473011	0.677366	-0.20436	4	26406431	R	II	RBPJ	Body	opensea	Body-opens		rs5549026	33
cg2127873	0.250081	0.606531	8.284287	4.52E-05	0.001585	2.566955	0.481491	0.731572	-0.25008	4	38797651	F	II		IGR	opensea	IGR-opense		rs1479840	0;12;35
cg1956838	0.231635	0.648165	8.283476	4.53E-05	0.001586	2.566242	0.532348	0.763983	-0.23164	4	2012845	F	II		IGR	shore	IGR-shore	chr4:2009	rs5473651	32
cg1475850	0.256747	0.514417	8.283399	4.53E-05	0.001586	2.566175	0.386043	0.64279	-0.25675	6	1.44E+08	R	II		IGR	opensea	IGR-opense		rs3702481	43;1
cg1176927	0.272866	0.622794	8.28204	4.53E-05	0.001587	2.564982	0.486361	0.759227	-0.27287	3	1.95E+08	F	II	XXYLT1	Body	opensea	Body-opens		rs5359511	1;45
cg1614399	0.202782	0.605627	8.281187	4.54E-05	0.001587	2.564232	0.504236	0.707019	-0.20278	3	1.37E+08	F	II		IGR	opensea	IGR-opense		rs5451298	29
cg2369121	0.204394	0.67662	8.280979	4.54E-05	0.001587	2.56405	0.574423	0.778816	-0.20439	15	99436798	R	II	IGF1R	Body	shore	Body-shore	chr15:994		
cg1595237	0.222172	0.51302	8.280823	4.54E-05	0.001587	2.563913	0.401934	0.624106	-0.22217	11	1.24E+08	R	II	OR8D2	TSS1500	opensea	TSS1500-op			
cg0059582	0.200415	0.68088	8.279821	4.54E-05	0.001588	2.563032	0.580673	0.781088	-0.20041	4	1.42E+08	R	II	TBC1D9	Body	opensea	Body-opens		rs1457301	50;34;18
cg2541810	0.206227	0.556827	8.279593	4.54E-05	0.001588	2.562832	0.453713	0.65994	-0.20623	12	1.32E+08	R	II	ADGRD1	Body	shelf	Body-shelf	chr12:131	rs1976930	31;19;16
cg2087211	0.203673	0.470304	8.279526	4.54E-05	0.001588	2.562774	0.368467	0.57214	-0.20367	9	1.11E+08	F	II		IGR	opensea	IGR-opense		rs1427120	30;31
cg0038329	0.228489	0.558167	8.278462	4.55E-05	0.001589	2.561838	0.443923	0.672412	-0.22849	8	1.41E+08	R	II	TRAPPC9	Body	opensea	Body-opens			
cg0095819	0.214391	0.443744	8.278236	4.55E-05	0.001589	2.56164	0.336548	0.550939	-0.21439	7	28474389	F	II	CREB5	TSS1500	opensea	TSS1500-op			
cg2259814	0.216935	0.547811	8.277968	4.55E-05	0.001589	2.561405	0.439344	0.656279	-0.21693	8	1.18E+08	R	II		IGR	opensea	IGR-opense		rs5350513	1
cg1353051	0.202445	0.579336	8.277149	4.55E-05	0.001589	2.560684	0.478113	0.680558	-0.20244	1	30951615	R	II		IGR	opensea	IGR-opense		rs6703542	42;39;14
cg1897212	0.218616	0.567122	8.276163	4.55E-05	0.00159	2.559818	0.457814	0.67643	-0.21862	10	54532059	R	II	MBL2	TSS1500	opensea	TSS1500-op		rs1100312	47;33;1
cg2263182	0.270945	0.462042	8.275994	4.55E-05	0.00159	2.55967	0.326569	0.597514	-0.27095	6	1.44E+08	R	II	ZC2HC1B	Body	opensea	Body-opens			
cg1518968	0.216284	0.596995	8.275657	4.56E-05	0.00159	2.559374	0.488853	0.705137	-0.21628	3	1.92E+08	R	II	FGF12	Body	opensea	Body-opens		rs5416124	4;2
cg0486878	0.209166	0.4855	8.274983	4.56E-05	0.001591	2.558781	0.380917	0.590083	-0.20917	2	2.34E+08	R	II	INPP5D	Body	opensea	Body-opens		rs1896125	49;40
cg2641831	0.223435	0.66987	8.274258	4.56E-05	0.001591	2.558144	0.558152	0.781587	-0.22344	1	2.48E+08	R	II	GCSAML	5'UTR	shelf	5'UTR-shelf	chr1:2476	rs5321191	21;19;5
cg2511010	0.200633	0.400869	8.274246	4.56E-05	0.001591	2.558134	0.300552	0.501186	-0.20063	4	84674460	R	II		IGR	opensea	IGR-opense		rs1808257	18;8
cg1875485	0.203754	0.535591	8.274229	4.56E-05	0.001591	2.558118	0.433714	0.637467	-0.20375	10	1.01E+08	R	II	HPSE2	Body	opensea	Body-opens		rs1914628	45
cg1606649	0.241512	0.633811	8.273938	4.56E-05	0.001591	2.557862	0.513055	0.754567	-0.24151	9	1.15E+08	F	II		IGR	opensea	IGR-opense		rs1478043	0
cg1017446	0.260221	0.651521	8.273374	4.56E-05	0.001592	2.557367	0.521411	0.781632	-0.26022	8	11739341	F	II		IGR	opensea	IGR-opense		rs1831214	21;29;47
cg0741815	0.270941	0.435084	8.272691	4.57E-05	0.001592	2.556766	0.299613	0.570554	-0.27094	19	13056052	R	II	RAD23A	TSS1500	shore	TSS1500-sh	chr19:130		
cg1488087	0.212396	0.35868	8.271985	4.57E-05	0.001593	2.556146	0.252482	0.464878	-0.2124	4	23271344	R	II	MIR548AJ2	Body	opensea	Body-opens		rs5566694	49;48;21
cg0408827	0.226466	0.641722	8.271764	4.57E-05	0.001593	2.555951	0.528489	0.754954	-0.22647	21	36675546	F	II		IGR	opensea	IGR-opense		rs1386258	1
cg0169214	0.208717	0.587323	8.271222	4.57E-05	0.001593	2.555475	0.482965	0.691682	-0.20872	17	33668674	F	II		IGR	opensea	IGR-opense		rs1807607	0;1;5;22;27
cg2257113	0.265487	0.441103	8.270396	4.58E-05	0.001594	2.554748	0.30836	0.573847	-0.26549	7	55377050	F	II		IGR	opensea	IGR-opense			
cg1874938	0.205782	0.529094	8.268634	4.58E-05	0.001595	2.553198	0.426203	0.631985	-0.20578	4	1.48E+08	R	II		IGR	opensea	IGR-opense		rs5442677	43;2;1
cg2698072	0.225353	0.496909	8.267087	4.59E-05	0.001597	2.551838	0.384232	0.609585	-0.22535	6	1.05E+08	R	II		IGR	opensea	IGR-opense		rs1464866	44;9
cg1523017	0.201351	0.670416	8.265916	4.59E-05	0.001597	2.550808	0.569741	0.771092	-0.20135	3	98488157	F	II	ST3GAL6	5'UTR	opensea	5'UTR-oper			
cg1916795	0.202223	0.497555	8.265598	4.59E-05	0.001597	2.550528	0.396444	0.598666	-0.20222	2	1.59E+08	F	II	UPP2	TSS1500	opensea	TSS1500-op		rs5560557	41
cg1189721	0.200612	0.507436	8.265353	4.6E-05	0.001597	2.550313	0.40713	0.607742	-0.20061	5	1.74E+08	R	II		IGR	opensea	IGR-opense		rs1918319	23;17
cg0090393	0.203355	0.660288	8.265155	4.6E-05	0.001597	2.550138	0.55861	0.761965	-0.20335	1	1.01E+08	F	II		IGR	opensea	IGR-opense		rs5482560	0;1;18;19
cg0611217	0.201489	0.480044	8.264987	4.6E-05	0.001597	2.54999	0.379299	0.580788	-0.20149	6	40324498	R	II		IGR	opensea	IGR-opense		rs5348713	31;2;1
cg1284737	0.265529	0.46738	8.263094	4.6E-05	0.001598	2.548324	0.334616	0.600145	-0.26553	19	13056058	R	II	RAD23A	TSS1500	shore	TSS1500-sh	chr19:130		
cg0857181	0.218461	0.456539	8.262879	4.61E-05	0.001598	2.548135	0.347308	0.565769	-0.21846	9	1.02E+08	F	II		IGR	opensea	IGR-opense		rs5411582	0;28
cg0865141	0.21188	0.695088	8.262344	4.61E-05	0.001599	2.547664	0.589148	0.801028	-0.21188	8	1.41E+08	R	II	TRAPPC9	Body	opensea	Body-opens			
cg1196400	0.242223	0.552398	8.262262	4.61E-05	0.001599	2.547592	0.431287	0.673509	-0.24222	10	53852689	F	II	PRKG1	Body	opensea	Body-opens		rs5474575	0;41;47
cg1410727	0.200176	0.484039	8.260638	4.61E-05	0.0016	2.546163	0.383951	0.584127	-0.20018	5	1.42E+08	F	II	ARHGAP26	Body	opensea	Body-opens		rs5708336	16;21;42
cg2569780	0.208109	0.500384	8.260253	4.62E-05	0.0016	2.545824	0.396329	0.604438	-0.20811	4	1.5E+08	F	II		IGR	opensea	IGR-opense		rs5454255	0;39
cg0046494	0.202125	0.342809	8.260096	4.62E-05	0.0016	2.545686	0.241746	0.443871	-0.20212	1	1.2E+08	R	II		IGR	shelf	IGR-shelf	chr1:1201	rs1153106	44;29;5
cg2645381	0.235375	0.485168	8.259742	4.62E-05	0.0016	2.545373	0.367481	0.602856	-0.23538	6	14755099	F	II		IGR	opensea	IGR-opense			
cg0723164	0.219314	0.600824	8.259274	4.62E-05	0.001601	2.544962	0.491167	0.710481	-0.21931	4	91014825	R	II		IGR	opensea	IGR-opense		rs1876035	17
cg2094743	0.209627	0.579581	8.259135	4.62E-05	0.001601	2.544839	0.474767	0.684394	-0.20963	4	96351973	F	II	UNC5C	Body	opensea	Body-opens		rs1896899	6;35;39
cg0265400	0.262974	0.559308	8.258383	4.62E-05	0.001601	2.544177	0.427821	0.690795	-0.26297	20	32810251	R	II		IGR	opensea	IGR-opense		rs5563497	8
cg1898333	0.204551	0.591259	8.25837	4.62E-05	0.001601	2.544165	0.488983	0.693534	-0.20455	17	19406038	R	II		IGR	opensea	IGR-opense		rs1394246	46
cg0840563	0.240582	0.483623	8.257362	4.63E-05	0.001602	2.543278	0.363332	0.603914	-0.24058	9	36383478	F	II	RNF38	Body	opensea	Body-opens		rs5365661	0;18;43
cg0537351	0.207555	0.709323	8.25619	4.63E-05	0.001603	2.542246	0.605546	0.813101	-0.20755	18	57459767	F	II		IGR	opensea	IGR-opense		rs5543024	41;50;51
cg1664269	0.209664	0.453042	8.255484	4.63E-05	0.001604	2.541624	0.34821	0.557874	-0.20966	8	67343633	F	II	ADHFE1	TSS1500	shore	TSS1500-sh	chr8:6734	rs5740842	15;47
cg2761879	0.208802	0.39985	8.255228	4.63E-05	0.001604	2.541398	0.295449	0.504251	-0.2088	6	1.33E+08	F	II	RPS12	Body	shelf	Body-shelf	chr6:1331	rs1462277	29;39
cg0814209	0.234782	0.416777	8.254839	4.64E-05	0.001604	2.541055	0.299386	0.534169	-0.23478	11	1.11E+08	R	II	C11orf53	TSS1500	opensea	TSS1500-op			
cg1321138	0.214524	0.619174	8.254197	4.64E-05	0.001605	2.54049	0.511912	0.726436	-0.21452	19	47440539	F	II	ARHGAP35	ExonBnd	opensea	ExonBnd-op			
cg0786972	0.201777	0.502228	8.253875	4.64E-05	0.001605	2.540206	0.40134	0.603117	-0.20178	4	1.72E+08	F	II		IGR	opensea	IGR-opense		rs5544149	0
cg0580438	0.212303	0.550573	8.25299	4.64E-05	0.001605	2.539427	0.444422	0.656725	-0.2123	2	1.67E+08	F	II	LOC10192	Body	opensea	Body-opens		rs1879475	1;7;25
cg0779180	0.202191	0.47605	8.252361	4.65E-05	0.001606	2.538873	0.374954	0.577146	-0.20219	8	34343944	F	II		IGR	opensea	IGR-opense		rs1854543	38
cg2201228	0.244862	0.487232	8.248502	4.66E-05	0.001608	2.535471	0.364801	0.609663	-0.24486	22	17895032	F	II		IGR	shelf	IGR-shelf	chr22:178	rs5736780	0;19;43;49
cg2256827	0.294754	0.437497	8.248192	4.66E-05	0.001608	2.535198	0.29012	0.584874	-0.29475	3	69811867	R	II	MITF	TSS1500	opensea	TSS1500-op		rs2014262	37
cg1406585	0.200651	0.530618	8.247754	4.66E-05	0.001608	2.534812	0.430292	0.630943	-0.20065	2	33436054	R	II	LTBP1	Body	opensea	Body-opens		rs1419021	17;9;2
cg1420853	0.202944	0.707085	8.247295	4.67E-05	0.001609	2.534408	0.605613	0.808557	-0.20294	7	90661477	R	II	CDK14	Body	opensea	Body-opens		rs1024188	36;2
cg1642814	0.211007	0.64843	8.246683	4.67E-05	0.001609	2.533867	0.542927	0.753934	-0.21101	16	4616356	R	II	C16orf96	Body	opensea	Body-opens			
cg1327653	0.219286	0.598264	8.246104	4.67E-05	0.00161	2.533357	0.488621	0.707907	-0.21929	4	91096255	F	II	CCSER1	5'UTR	opensea	5'UTR-oper		rs5704904	1;3;13
cg2609811	0.210742	0.640456	8.24584	4.67E-05	0.00161	2.533124	0.535085	0.745827	-0.21074	6	1.26E+08	R	II		IGR	opensea	IGR-opense		rs5499868	45;5;2;1
cg2739782	0.218622	0.560672	8.245439	4.67E-05	0.00161	2.532771	0.451361	0.669983	-0.21862	2	17150204	R	II		IGR	opensea	IGR-opense		rs5719107	33;11;1
cg0682587	0.249492	0.476334	8.244803	4.68E-05	0.001611	2.53221	0.351588	0.60108	-0.24949	7	75472540	F	II		IGR	opensea	IGR-opense		rs5679392	1;5;13
cg1599697	0.214554	0.618523	8.244737	4.68E-05	0.001611	2.532151	0.511246	0.7258	-0.21455	12	79450288	R	II	SYT1	5'UTR	opensea	5'UTR-oper		rs1416307	38;11;2
cg1381779	0.204719	0.612693	8.244478	4.68E-05	0.001611	2.531923	0.510334	0.715053	-0.20472	2	2.16E+08	R	II	BARD1	Body	opensea	Body-opens		rs5718730	22
cg1499618	0.210764	0.628604	8.24374	4.68E-05	0.001611	2.531273	0.523221	0.733986	-0.21076	3	1.49E+08	F	II		IGR	opensea	IGR-opense		rs1456784	18;24;27;28;35
cg1569759	0.207584	0.552683	8.24266	4.68E-05	0.001612	2.53032	0.448891	0.656475	-0.20758	1	6805149	F	II		IGR	opensea	IGR-opense		rs5465103	49
cg0282684	0.241778	0.699155	8.241827	4.69E-05	0.001612	2.529585	0.578266	0.820044	-0.24178	7	1.07E+08	R	II	PIK3CG	ExonBnd	opensea	ExonBnd-op		rs1129293	27;24;20
cg2166685	0.209626	0.579549	8.24134	4.69E-05	0.001613	2.529155	0.474736	0.684362	-0.20963	2	2.01E+08	R	II		IGR	opensea	IGR-opense		rs1504915	12
cg0210706	0.209664	0.651532	8.241151	4.69E-05	0.001613	2.528989	0.5467	0.756364	-0.20966	5	52281282	R	II		IGR	shelf	IGR-shelf	chr5:5228	rs5468890	41
cg0938403	0.277328	0.575107	8.239475	4.7E-05	0.001613	2.52751	0.436443	0.713771	-0.27733	8	1.04E+08	F	II		IGR	opensea	IGR-opense			
cg0667951	0.202983	0.604022	8.238995	4.7E-05	0.001614	2.527086	0.502531	0.705514	-0.20298	7	11467973	R	II	THSD7A	Body	opensea	Body-opens		rs1125260	2;1
cg0033922	0.207106	0.675405	8.238806	4.7E-05	0.001614	2.526919	0.571852	0.778958	-0.20711	3	1.26E+08	F	II	C3orf22	Body	opensea	Body-opens			
cg0070645	0.214138	0.633497	8.238139	4.7E-05	0.001615	2.526331	0.526428	0.740566	-0.21414	4	1.55E+08	F	II	FGB	TSS1500	opensea	TSS1500-op			
cg1229125	0.204958	0.667505	8.237564	4.7E-05	0.001615	2.525823	0.565026	0.769984	-0.20496	2	58997691	R	II	LINC01122	Body	opensea	Body-opens		rs5417051	21
cg1686537	0.220608	0.451623	8.237528	4.7E-05	0.001615	2.525791	0.341319	0.561928	-0.22061	6	1.26E+08	R	II	TPD52L1	5'UTR	opensea	5'UTR-oper		rs1513254	26;2
cg2501477	0.24891	0.495966	8.235336	4.71E-05	0.001616	2.523857	0.371511	0.620421	-0.24891	12	65951721	F	II	LOC10050	Body	opensea	Body-opens			
cg1014299	0.200713	0.632149	8.234189	4.72E-05	0.001617	2.522844	0.531793	0.732506	-0.20071	13	67240245	R	II	PCDH9	Body	opensea	Body-opens		rs5657809	43;14
cg2150870	0.207784	0.628617	8.233067	4.72E-05	0.001618	2.521854	0.524725	0.732509	-0.20778	12	51602241	R	II	POU6F1	Body	opensea	Body-opens		rs5782215	29;2
cg2040257	0.222577	0.630693	8.232745	4.72E-05	0.001618	2.521569	0.519405	0.741982	-0.22258	7	24813196	F	II		IGR	opensea	IGR-opense		rs5398209	1;4;30;35
cg1154800	0.234817	0.378161	8.231451	4.73E-05	0.001619	2.520426	0.260752	0.495569	-0.23482	8	48812660	R	II	PRKDC	Body	opensea	Body-opens		rs8178076	16;2
cg0750473	0.213049	0.38852	8.228744	4.74E-05	0.001621	2.518035	0.281996	0.495045	-0.21305	1	2.31E+08	R	II		IGR	opensea	IGR-opense		rs1818259	2;1
cg0205640	0.24144	0.574865	8.228739	4.74E-05	0.001621	2.51803	0.454145	0.695585	-0.24144	5	88263995	R	II	MEF2C-AS1	Body	opensea	Body-opens		rs5360346	16;2
cg0829201	0.209392	0.554302	8.228716	4.74E-05	0.001621	2.51801	0.449606	0.658998	-0.20939	18	53453900	R	II		IGR	opensea	IGR-opense		rs5317835	35;1
cg0856329	0.200097	0.663392	8.227459	4.74E-05	0.001622	2.5169	0.563344	0.763441	-0.2001	19	16922592	R	II	NWD1	Body	shelf	Body-shelf	chr19:169	rs1857637	15;2
cg0042566	0.220329	0.533077	8.226767	4.75E-05	0.001622	2.516288	0.422912	0.643241	-0.22033	14	38485787	R	II		IGR	opensea	IGR-opense		rs1846883	2
cg0749443	0.200397	0.457769	8.225916	4.75E-05	0.001623	2.515536	0.357571	0.557968	-0.2004	10	52287340	F	II	SGMS1	5'UTR	opensea	5'UTR-oper		rs5715778	0
cg0741838	0.225496	0.502611	8.225097	4.75E-05	0.001624	2.514813	0.389863	0.615359	-0.2255	1	2.4E+08	F	II	FMN2	Body	shelf	Body-shelf	chr1:2403	rs1829430	0;4
cg2112812	0.200064	0.520004	8.224194	4.76E-05	0.001624	2.514015	0.419972	0.620036	-0.20006	1	48522093	R	II		IGR	opensea	IGR-opense		rs3727894	2
cg0705378	0.241931	0.441969	8.223915	4.76E-05	0.001624	2.513768	0.321003	0.562934	-0.24193	3	1.79E+08	R	II	ZMAT3	3'UTR	opensea	3'UTR-oper		rs5568058	2
cg0235042	0.218359	0.659775	8.223885	4.76E-05	0.001624	2.513741	0.550595	0.768955	-0.21836	8	1.27E+08	F	II	LINC00861	Body	opensea	Body-opens			
cg2497434	0.222035	0.660111	8.222979	4.76E-05	0.001625	2.512941	0.549094	0.771129	-0.22203	10	13492017	F	II	BEND7	Body	opensea	Body-opens			
cg0988559	0.2115	0.659227	8.22285	4.76E-05	0.001625	2.512827	0.553477	0.764977	-0.2115	6	25727535	R	II	HIST1H2AA	TSS1500	shore	TSS1500-sh	chr6:2572	rs9358872	20;15;10
cg0157462	0.20711	0.617723	8.22255	4.76E-05	0.001626	2.512561	0.514168	0.721278	-0.20711	9	26797396	R	II		IGR	opensea	IGR-opense		rs1845307	30
cg1257414	0.222131	0.480737	8.222352	4.76E-05	0.001626	2.512386	0.369671	0.591802	-0.22213	2	1.54E+08	R	II		IGR	opensea	IGR-opense		rs7397027	44;40;2
cg2038995	0.21659	0.380538	8.22037	4.77E-05	0.001627	2.510633	0.272244	0.488833	-0.21659	11	62195515	F	II		IGR	opensea	IGR-opense		rs1440603	48
cg0424627	0.212349	0.559714	8.219732	4.78E-05	0.001627	2.510069	0.45354	0.665888	-0.21235	16	12847194	F	II	CPPED1	Body	opensea	Body-opens		rs5426247	1;24;45
cg0438195	0.217394	0.448616	8.21964	4.78E-05	0.001627	2.509988	0.339919	0.557313	-0.21739	3	16550461	R	II	RFTN1	5'UTR	shelf	5'UTR-shelf	chr3:1655	rs5602788	26;22

cg0683899	0.207732	0.613671	8.219459	4.78E-05	0.001627	2.509828	0.509805	0.717537	-0.20773	11	78788443	F	II	TENM4	5'UTR	opensea	5'UTR-oper			
cg0305523	0.21175	0.605671	8.219184	4.78E-05	0.001628	2.509585	0.499796	0.711546	-0.21175	5	1.19E+08	R	II	TNFAIP8	TSS1500	shore	TSS1500-sh	chr5:1186	rs1134299	25
cg1081640	0.205425	0.655216	8.218845	4.78E-05	0.001628	2.509285	0.552504	0.757928	-0.20542	20	11851441	F	II	LINC00687	TSS200	opensea	TSS200-ope		rs1842197	16;36;37
cg0359886	0.228101	0.484106	8.217805	4.78E-05	0.001629	2.508365	0.370055	0.598156	-0.2281	6	1.7E+08	R	II	LINC00242	Body	opensea	Body-opens		rs5422047	45
cg0135982	0.216436	0.673578	8.215692	4.79E-05	0.00163	2.506496	0.56536	0.781796	-0.21644	21	40176597	R	I	ETS2	TSS1500	shore	TSS1500-sh	chr21:401	rs5669270	48;38;20
cg2720023	0.237701	0.512387	8.214307	4.8E-05	0.001631	2.50527	0.393537	0.631238	-0.2377	4	24296454	F	II		IGR	opensea	IGR-opense			
cg1597608	0.210982	0.544758	8.213049	4.8E-05	0.001632	2.504157	0.439267	0.650249	-0.21098	15	47809041	R	II	SEMA6D	5'UTR	opensea	5'UTR-oper		rs1259452	41;36
cg1146988	0.229877	0.590255	8.21187	4.81E-05	0.001633	2.503114	0.475317	0.705194	-0.22988	1	9062256	R	II		IGR	opensea	IGR-opense		rs5410470	28;1
cg0622636	0.214852	0.683967	8.21185	4.81E-05	0.001633	2.503096	0.576542	0.791393	-0.21485	3	72200262	F	II	LINC00870	TSS200	opensea	TSS200-ope			
cg1136171	0.2537	0.605199	8.211712	4.81E-05	0.001633	2.502974	0.478349	0.732049	-0.2537	13	1E+08	R	II		IGR	opensea	IGR-opense		rs1454540	47;1
cg2627142	0.205472	0.63764	8.211662	4.81E-05	0.001633	2.50293	0.534904	0.740376	-0.20547	16	10767081	F	II	TEKT5	Body	opensea	Body-opens		rs1435022	0;1;28
cg0416643	0.221724	0.570986	8.21147	4.81E-05	0.001633	2.50276	0.460124	0.681848	-0.22172	3	1.67E+08	F	II	WDR49	Body	opensea	Body-opens		rs2020586	1
cg0323036	0.271162	0.519146	8.211419	4.81E-05	0.001633	2.502715	0.383565	0.654727	-0.27116	18	55681687	R	II		IGR	opensea	IGR-opense		rs1848742	45;41;35
cg2421121	0.218621	0.581158	8.211199	4.81E-05	0.001633	2.50252	0.471848	0.690469	-0.21862	2	1.6E+08	F	II		IGR	opensea	IGR-opense		rs5617902	37
cg2630166	0.20784	0.62494	8.21008	4.81E-05	0.001634	2.501529	0.52102	0.72886	-0.20784	6	57070622	F	II	RAB23	Body	opensea	Body-opens		rs5548356	16
cg1603577	0.223331	0.471562	8.209043	4.82E-05	0.001634	2.500611	0.359896	0.583227	-0.22333	1	2.06E+08	R	II	MFSD4	Body	opensea	Body-opens		rs5532740	2
cg0773825	0.202905	0.543008	8.208862	4.82E-05	0.001634	2.500451	0.441556	0.644461	-0.2029	1	64368601	F	II	ROR1	Body	opensea	Body-opens			
cg2406074	0.205609	0.533225	8.207937	4.82E-05	0.001635	2.499632	0.430421	0.636029	-0.20561	7	41045529	R	II		IGR	opensea	IGR-opense		rs5675103	38
cg2536799	0.214822	0.613957	8.206513	4.83E-05	0.001636	2.498371	0.506545	0.721368	-0.21482	11	1.14E+08	F	II		IGR	opensea	IGR-opense		rs1149298	10
cg1024632	0.215547	0.592441	8.20554	4.83E-05	0.001637	2.497509	0.484668	0.700215	-0.21555	9	96845038	F	II	PTPDC1	Body	opensea	Body-opens			
cg2623839	0.249649	0.588622	8.205129	4.83E-05	0.001637	2.497145	0.463797	0.713446	-0.24965	6	1.33E+08	R	II	VNN2	Body	opensea	Body-opens		rs3485606	33;32;20;1
cg0700794	0.200633	0.617378	8.202048	4.85E-05	0.001639	2.494415	0.517061	0.717694	-0.20063	2	2.14E+08	R	II	IKZF2	Body	opensea	Body-opens		rs5422946	44;30;11;2
cg2678396	0.270329	0.481376	8.202043	4.85E-05	0.001639	2.494411	0.346212	0.616541	-0.27033	6	1.33E+08	R	II		IGR	opensea	IGR-opense		rs5438982	37;8;3
cg2278647	0.264676	0.387432	8.201497	4.85E-05	0.00164	2.493927	0.255094	0.51977	-0.26468	4	26198975	F	II		IGR	opensea	IGR-opense		rs5473382	0
cg1053340	0.252816	0.661157	8.201069	4.85E-05	0.00164	2.493548	0.534749	0.787565	-0.25282	3	11766469	R	II		IGR	opensea	IGR-opense		rs5429350	23;1
cg0220948	0.244188	0.587184	8.199922	4.86E-05	0.001641	2.492531	0.46509	0.709278	-0.24419	4	1.86E+08	F	II		IGR	opensea	IGR-opense		rs4541559	34;39;44
cg0353561	0.20665	0.615873	8.199382	4.86E-05	0.001641	2.492053	0.512548	0.719198	-0.20665	21	39832552	F	II	ERG	Body	opensea	Body-opens		rs1826909	1;14
cg2135763	0.211868	0.502849	8.198218	4.86E-05	0.001642	2.491021	0.396916	0.608783	-0.21187	2	3800503	R	II	DCDC2C	ExonBnd	opensea	ExonBnd-op		rs2010040	38;9;1
cg2719373	0.240454	0.501723	8.197503	4.87E-05	0.001643	2.490387	0.381496	0.621951	-0.24045	7	90562815	F	II	CDK14	Body	opensea	Body-opens		rs5416637	1;39
cg2240167	0.225388	0.639862	8.19437	4.88E-05	0.001645	2.48761	0.527168	0.752556	-0.22539	1	95080047	F	II		IGR	opensea	IGR-opense		rs5666043	46
cg1441122	0.233415	0.53113	8.194085	4.88E-05	0.001645	2.487357	0.414423	0.647838	-0.23341	14	64064608	F	II	WDR89	3'UTR	shelf	3'UTR-shelf	chr14:640	rs5598791	28
cg2036707	0.221225	0.647406	8.194042	4.88E-05	0.001645	2.487318	0.536794	0.758018	-0.22122	11	19224117	R	II	CSRP3	TSS1500	opensea	TSS1500-op		rs5676342	48;39;27;13;2
cg1448133	0.240055	0.613851	8.194003	4.88E-05	0.001645	2.487284	0.493823	0.733878	-0.24006	17	57678746	R	II	DHX40	Body	opensea	Body-opens		rs5618236	44;32
cg0735043	0.201213	0.467558	8.19394	4.88E-05	0.001645	2.487228	0.366952	0.568165	-0.20121	5	1.69E+08	R	II		IGR	opensea	IGR-opense		rs5287797	34;26;2
cg0719877	0.285596	0.499364	8.193445	4.88E-05	0.001645	2.48679	0.356565	0.642162	-0.2856	7	92439762	R	II	CDK6	Body	opensea	Body-opens		rs1878481	40;32;28;1
cg0441044	0.272639	0.493873	8.193331	4.88E-05	0.001645	2.486688	0.357553	0.630193	-0.27264	6	1.44E+08	R	II	C6orf94	Body	opensea	Body-opens		rs5456474	38;2;1
cg2472711	0.20065	0.632142	8.19279	4.88E-05	0.001645	2.486208	0.531818	0.732467	-0.20065	4	1.25E+08	R	II	LOC28541	Body	opensea	Body-opens		rs5387228	15
cg2052272	0.203649	0.563706	8.192546	4.89E-05	0.001646	2.485992	0.461881	0.66553	-0.20365	4	3336511	F	II	RGS12	Body	opensea	Body-opens		rs5680465	0;12;48
cg1930400	0.23521	0.547656	8.192041	4.89E-05	0.001646	2.485544	0.430051	0.665261	-0.23521	2	30967383	R	II	CAPN13	Body	opensea	Body-opens		rs1872625	19
cg1552473	0.205918	0.555068	8.191992	4.89E-05	0.001646	2.4855	0.452109	0.658027	-0.20592	5	68267366	R	II	LOC10192	Body	opensea	Body-opens			
cg0682369	0.202073	0.618303	8.191971	4.89E-05	0.001646	2.485482	0.517266	0.719339	-0.20207	7	8424991	F	II		IGR	opensea	IGR-opense		rs1862577	0;13
cg2642588	0.201889	0.525974	8.191516	4.89E-05	0.001646	2.485078	0.425029	0.626919	-0.20189	2	1.56E+08	R	II		IGR	opensea	IGR-opense		rs5397362	44;26;4
cg1018477	0.219908	0.472609	8.190842	4.89E-05	0.001647	2.48448	0.362655	0.582563	-0.21991	17	62102410	R	II		IGR	opensea	IGR-opense		rs5323188	35;33;26;10;8
cg0995416	0.201167	0.640141	8.18885	4.9E-05	0.001648	2.482713	0.539558	0.740725	-0.20117	12	57737949	R	II		IGR	opensea	IGR-opense		rs5696223	2
cg1214444	0.206355	0.552583	8.188574	4.9E-05	0.001649	2.482468	0.449406	0.655761	-0.20635	4	1.64E+08	R	II		IGR	opensea	IGR-opense		rs1826711	25;17
cg1992625	0.23482	0.595682	8.188434	4.9E-05	0.001649	2.482344	0.478272	0.713092	-0.23482	3	1.51E+08	F	II	GPR87	5'UTR	opensea	5'UTR-oper		rs5653766	1;11;44
cg0395486	0.20204	0.556576	8.188408	4.9E-05	0.001649	2.482321	0.455555	0.657596	-0.20204	5	83323870	R	II	EDIL3	Body	opensea	Body-opens		rs5355648	35;20
cg1639387	0.209637	0.576933	8.187314	4.91E-05	0.001649	2.48135	0.472114	0.681751	-0.20964	22	22726103	F	II		IGR	opensea	IGR-opense		rs5528348	1
cg2483834	0.219689	0.550927	8.18658	4.91E-05	0.00165	2.480698	0.441082	0.660772	-0.21969	19	55314972	R	II	KIR2DL4	TSS200	opensea	TSS200-ope		rs1717310	25
cg1912346	0.220165	0.594232	8.186523	4.91E-05	0.00165	2.480648	0.484149	0.704314	-0.22016	18	28567554	R	II		IGR	opensea	IGR-opense		rs5451186	16;6
cg1731112	0.220168	0.452263	8.186279	4.91E-05	0.00165	2.480431	0.342179	0.562347	-0.22017	5	1.56E+08	R	II	SGCD	Body	opensea	Body-opens		rs5291471	29
cg0061707	0.230961	0.535998	8.186059	4.91E-05	0.00165	2.480235	0.420517	0.651478	-0.23096	4	1.03E+08	F	II	BANK1	Body	opensea	Body-opens		rs5337806	1;18;35;41
cg2127377	0.222544	0.646146	8.185708	4.91E-05	0.00165	2.479924	0.534874	0.757418	-0.22254	11	94726463	F	II	KDM4D	5'UTR	opensea	5'UTR-oper		rs5876593	0
cg1035730	0.230343	0.682351	8.18521	4.92E-05	0.00165	2.479483	0.567179	0.797522	-0.23034	6	1.38E+08	R	II		IGR	opensea	IGR-opense			
cg2090494	0.211524	0.532092	8.183355	4.92E-05	0.001652	2.477835	0.42633	0.637854	-0.21152	18	54176880	R	II		IGR	opensea	IGR-opense		rs5494113	12
cg2281193	0.23689	0.577222	8.183193	4.92E-05	0.001652	2.477691	0.458777	0.695667	-0.23689	18	10072795	R	II		IGR	opensea	IGR-opense		rs5395665	1
cg0238142	0.222178	0.588962	8.182888	4.93E-05	0.001652	2.47742	0.477873	0.700052	-0.22218	6	1.37E+08	R	II		IGR	shelf	IGR-shelf	chr6:1373	rs1196734	51;46
cg0970506	0.202458	0.608854	8.182359	4.93E-05	0.001652	2.476951	0.507625	0.710083	-0.20246	6	56729413	R	II	DST	Body	opensea	Body-opens		rs1124857	41;12;2
cg2123888	0.262573	0.595509	8.181591	4.93E-05	0.001653	2.476269	0.464222	0.726796	-0.26257	6	83796488	F	II	DOPEY1	5'UTR	opensea	5'UTR-oper			
cg1102206	0.208547	0.505145	8.181529	4.93E-05	0.001653	2.476214	0.400872	0.609419	-0.20855	15	83298969	F	II	CPEB1	Body	opensea	Body-opens			
cg0343116	0.302543	0.559445	8.180659	4.94E-05	0.001653	2.475441	0.408174	0.710717	-0.30254	3	1.22E+08	F	II	CD86	5'UTR	opensea	5'UTR-oper		rs1407765	0;6;14;15;29;35
cg2463917	0.25375	0.594903	8.180605	4.94E-05	0.001653	2.475393	0.468028	0.721778	-0.25375	6	12753473	R	II	PHACTR1	Body	shelf	Body-shelf	chr6:1274	rs1508855	30
cg2447594	0.234829	0.719891	8.179684	4.94E-05	0.001654	2.474575	0.602476	0.837305	-0.23483	1	1.79E+08	F	II	RALGPS2	Body	opensea	Body-opens			
cg2202305	0.234714	0.608679	8.179575	4.94E-05	0.001654	2.474478	0.491322	0.726036	-0.23471	2	1.82E+08	R	II	ITGA4	Body	opensea	Body-opens		rs5273759	47;1
cg0926710	0.207458	0.642793	8.178707	4.94E-05	0.001654	2.473707	0.539065	0.746522	-0.20746	13	78929949	F	II	RNF219-AS	Body	opensea	Body-opens		rs1406516	3;28;38
cg0931550	0.227347	0.382363	8.178367	4.94E-05	0.001655	2.473405	0.26869	0.496037	-0.22735	8	1.17E+08	F	II	LINC00536	Body	opensea	Body-opens		rs5635606	1;13
cg0423410	0.269663	0.473729	8.178324	4.95E-05	0.001655	2.473367	0.338897	0.60856	-0.26966	10	6051242	R	II		IGR	opensea	IGR-opense		rs4129467	32;19;2
cg0914562	0.209953	0.479852	8.176504	4.95E-05	0.001656	2.47175	0.374876	0.584829	-0.20995	12	81189688	F	II		IGR	opensea	IGR-opense			
cg1752428	0.218364	0.606814	8.17559	4.96E-05	0.001657	2.470938	0.497632	0.715997	-0.21836	1	1.12E+08	F	II	KCND3	Body	opensea	Body-opens			
cg0277062	0.205858	0.624875	8.174724	4.96E-05	0.001657	2.470169	0.521946	0.727804	-0.20586	8	67032303	F	II		IGR	opensea	IGR-opense		rs1847501	1;25
cg1810624	0.207396	0.460342	8.171774	4.97E-05	0.001659	2.467546	0.356643	0.56404	-0.2074	6	42158764	F	II	GUCA1B	Body	opensea	Body-opens		rs7343428	1
cg1210724	0.225131	0.616218	8.171032	4.98E-05	0.001659	2.466886	0.503653	0.728784	-0.22513	17	75759464	F	II		IGR	opensea	IGR-opense		rs5540388	3
cg0451754	0.204458	0.587823	8.171013	4.98E-05	0.001659	2.466869	0.485594	0.690052	-0.20446	17	46988488	R	II	UBE2Z	Body	shore	Body-shore	chr17:469	rs5395273	41;1
cg0442665	0.211034	0.558292	8.170652	4.98E-05	0.001659	2.466549	0.452774	0.663809	-0.21103	3	1.26E+08	F	II		IGR	shelf	IGR-shelf	chr3:1261	rs1474867	1;5;7;46
cg1526380	0.215061	0.412925	8.167409	4.99E-05	0.001662	2.463664	0.305394	0.520455	-0.21506	1	39318607	R	II	RRAGC	Body	opensea	Body-opens		rs1473615	2;1
cg1488762	0.215011	0.485011	8.166772	4.99E-05	0.001663	2.463098	0.377506	0.592517	-0.21501	18	53501191	F	II		IGR	opensea	IGR-opense		rs7563913	1
cg2003546	0.251673	0.395457	8.165832	5E-05	0.001663	2.462262	0.269621	0.521294	-0.25167	8	1.34E+08	F	II	PHF20L1	Body	opensea	Body-opens		rs1871335	18
cg2534396	0.206429	0.59481	8.165744	5E-05	0.001663	2.462184	0.491596	0.698025	-0.20643	11	1.13E+08	R	II	NCAM1	Body	opensea	Body-opens			
cg1030776	0.21416	0.695258	8.16567	5E-05	0.001663	2.462118	0.588178	0.802338	-0.21416	6	1.09E+08	R	II	FOXO3	Body	opensea	Body-opens		rs1499392	49
cg0765347	0.20455	0.593022	8.165047	5E-05	0.001664	2.461563	0.490747	0.695297	-0.20455	22	33080918	F	II	SYN3	Body	opensea	Body-opens		rs1122282	5
cg2451588	0.20419	0.377919	8.164866	5E-05	0.001664	2.461402	0.275824	0.480014	-0.20419	2	18494208	R	II		IGR	opensea	IGR-opense		rs5743664	25;7;2
cg1759649	0.205218	0.394564	8.164313	5E-05	0.001664	2.46091	0.291955	0.497173	-0.20522	13	24143068	F	II	TNFRSF19	TSS1500	opensea	TSS1500-op		rs5775225	0
cg0348971	0.235394	0.572654	8.162909	5.01E-05	0.001666	2.459661	0.454957	0.690351	-0.23539	7	1.43E+08	F	II	ZYX	TSS1500	shore	TSS1500-sh	chr7:1430	rs1500525	7;38
cg0000208	0.203901	0.587312	8.162579	5.01E-05	0.001666	2.459367	0.485361	0.689262	-0.2039	21	30390417	F	II	RWDD2B	Body	shore	Body-shore	chr21:303	rs1425495	29;33
cg1536705	0.245331	0.486659	8.161237	5.02E-05	0.001667	2.458172	0.363994	0.609325	-0.24533	7	92271711	R	II	CDK6	Body	opensea	Body-opens		rs5616818	33;26
cg1247378	0.209752	0.511176	8.159245	5.03E-05	0.001668	2.4564	0.4063	0.616052	-0.20975	5	1.5E+08	F	II	CDX1	Body	opensea	Body-opens		rs5503785	1;25
cg2204354	0.214333	0.531802	8.158675	5.03E-05	0.001668	2.455892	0.424636	0.638969	-0.21433	4	1.69E+08	R	II	PALLD	Body	opensea	Body-opens		rs1905231	1
cg1788004	0.232894	0.660218	8.157133	5.03E-05	0.00167	2.454519	0.543771	0.776665	-0.23289	12	1.11E+08	R	II		IGR	opensea	IGR-opense		rs1413333	36;31;28
cg0329333	0.221498	0.609548	8.155849	5.04E-05	0.001671	2.453376	0.498799	0.720296	-0.2215	2	42941234	R	II	MTA3	Body	opensea	Body-opens		rs1153952	46;33;31;23
cg0070554	0.213484	0.667932	8.154956	5.04E-05	0.001671	2.45258	0.56119	0.774674	-0.21348	6	1.29E+08	F	II	PTPRK	5'UTR	opensea	5'UTR-oper		rs5666790	42
cg0868021	0.208597	0.576833	8.154703	5.04E-05	0.001671	2.452355	0.472534	0.681131	-0.2086	14	89743161	R	II	FOXN3	Body	opensea	Body-opens		rs5540710	34;2;1
cg1890455	0.22714	0.621232	8.15411	5.05E-05	0.001672	2.451827	0.507662	0.734803	-0.22714	10	93624759	F	II	TNKS2	3'UTR	opensea	3'UTR-oper		rs5768482	11
cg1959269	0.210754	0.561243	8.153932	5.05E-05	0.001672	2.451668	0.455866	0.66662	-0.21075	7	1.36E+08	R	II	LUZP6	5'UTR	shore	5'UTR-shor	chr7:1356	rs5393968	45
cg1095753	0.240705	0.625211	8.152104	5.06E-05	0.001673	2.45004	0.504859	0.745564	-0.2407	18	29147253	F	II		IGR	opensea	IGR-opense		rs1481839	1;20
cg2044943	0.227676	0.602972	8.151506	5.06E-05	0.001674	2.449507	0.489134	0.71681	-0.22768	18	9595066	R	II	PPP4R1	Body	opensea	Body-opens		rs5590680	18;2
cg1418948	0.211247	0.558019	8.151052	5.06E-05	0.001674	2.449102	0.452395	0.663643	-0.21125	1	1.96E+08	F	II	KCNT2	Body	opensea	Body-opens		rs5604618	28
cg2111134	0.215323	0.608705	8.150523	5.06E-05	0.001674	2.448631	0.501044	0.716367	-0.21532	11	1.09E+08	F	II		IGR	opensea	IGR-opense		rs1489490	1;48
cg2130847	0.262266	0.39527	8.150342	5.06E-05	0.001674	2.448469	0.264138	0.526403	-0.26227	1	12911678	R	II		IGR	opensea	IGR-opense		rs1143835	45;44;35;26;2;1
cg0248243	0.210571	0.623182	8.149928	5.07E-05	0.001675	2.448101	0.517896	0.728467	-0.21057	6	49753577	F	II	PGK2	3'UTR	opensea	3'UTR-oper		rs5579241	8
cg0296844	0.253097	0.44084	8.148476	5.07E-05	0.001676	2.446806	0.314292	0.567389	-0.2531	2	68972158	F	II	ARHGAP25	Body	opensea	Body-opens		rs5400394	0;1
cg0028873	0.207044	0.675534	8.147869	5.07E-05	0.001676	2.446266	0.572012	0.779056	-0.20704	8	1.21E+08	R	II	COL14A1	5'UTR	shelf	5'UTR-shelf	chr8:1211	rs7448817	36
cg2191378	0.236794	0.517225	8.147749	5.07E-05	0.001676	2.446159	0.398828	0.635622	-0.23679	5	96789385	F	II		IGR	opensea	IGR-opense		rs5565847	20;48
cg0192867	0.232068	0.679084	8.147436	5.08E-05	0.001677	2.44588	0.56305	0.795118	-0.23207	12	2261178	R	II	CACNA1C	Body	opensea	Body-opens			
cg2130231	0.215852	0.490788	8.146963	5.08E-05	0.001677	2.445458	0.382863	0.598714	-0.21585	9	1.09E+08	R	I	LINC01505	Body	opensea	Body-opens		rs5664167	41;21;2
cg1697353	0.230968	0.672209	8.145542	5.08E-05	0.001678	2.444191	0.556725	0.787693	-0.23097	10	65460155	F	II		IGR	opensea	IGR-opense		rs1922008	32
cg1749257	0.230077	0.480887	8.145084	5.09E-05	0.001678	2.443783	0.365849	0.595925	-0.23008	6	1.59E+08	R	II		IGR	opensea	IGR-opense		rs5410837	2
cg2176834	0.20574	0.582434	8.144828	5.09E-05	0.001678	2.443555	0.479564	0.685304	-0.20574	2	16704265	R	II		IGR	opensea	IGR-opense		rs1465981	25
cg0158418	0.242298	0.529084	8.143125	5.09E-05	0.00168	2.442036	0.407935	0.650233	-0.2423	18	24136594	F	II	KCTD1	Body	opensea	Body-opens		rs2006677	23;36;48
cg2441858	0.23064	0.520073	8.143088	5.09E-05	0.00168	2.442003	0.404752	0.635393	-0.23064	5	1.25E+08	F	II		IGR	opensea	IGR-opense		rs7812317	0;1;50
cg1717454	0.218139	0.532795	8.142661	5.1E-05	0.00168	2.441622	0.423726	0.641865	-0.21814	5	58802616	F	II	PDE4D	Body	opensea	Body-opens		rs5476152	0;1;32
cg0007052	0.236031	0.5402	8.138881	5.11E-05	0.001682	2.43825	0.422184	0.658215	-0.23603	13	43986655	R	II	ENOX1	Body	opensea	Body-opens		rs5556257	44;1
cg1386880	0.259503	0.658705	8.13844	5.12E-05	0.001682	2.437856	0.528954	0.788457	-0.2595	9	87917961	F	II		IGR	opensea	IGR-opense		rs5392606	0;6;15;30
cg1245505	0.2008	0.693542	8.138363	5.12E-05	0.001682	2.437787	0.593142	0.793942	-0.2008	2	1.97E+08	R	II	HECW2	Body	opensea	Body-opens		rs5626588	20;2
cg0112372	0.237508	0.44666	8.138065	5.12E-05	0.001682	2.437522	0.327906	0.565415	-0.23751	12	20280903	F	II		IGR	opensea	IGR-opense			
cg2074569	0.213034	0.356256	8.137831	5.12E-05	0.001683	2.437313	0.249739	0.462773	-0.21303	1	26909537	R	II		IGR	opensea	IGR-opense			
cg2559435	0.211403	0.636789	8.135435	5.13E-05	0.001685	2.435174	0.531088	0.742491	-0.2114	8	97391560	R	II	LOC10272	Body	opensea	Body-opens		rs5582416	42;16
cg2045008	0.258344	0.510981	8.135379	5.13E-05	0.001685	2.435124	0.381809	0.640153	-0.25834	6	75857507	R	II	COL12A1	ExonBnd	opensea	ExonBnd-op		rs1135498	36;35;13
cg1487333	0.209558	0.596115	8.133658	5.14E-05	0.001686	2.433588	0.491336	0.700894	-0.20956	3	60280143	F	II	FHIT	Body	opensea	Body-opens		rs5342298	8
cg1158328	0.215273	0.631133	8.133651	5.14E-05	0.001686	2.433582	0.523496	0.738769	-0.21527	10	45911321	R	II	ALOX5	Body	shelf	Body-shelf	chr10:459	rs7328317	31;6;4;1

cg2748669	0.222469	0.653997	8.133278	5.14E-05	0.001686	2.433249	0.542763	0.765231	-0.22247	12	14765994	F	II	GUCY2C	3'UTR	opensea	3'UTR-open		rs5538918	0;1;30
cg0111444	0.202836	0.730083	8.132014	5.14E-05	0.001687	2.43212	0.628665	0.831501	-0.20284	9	4686293	R	II	CDC37L1	Body	opensea	Body-opens		rs5780031	25;6;2
cg0722224	0.21447	0.3874	8.130648	5.15E-05	0.001688	2.4309	0.280166	0.494635	-0.21447	7	1.06E+08	F	II		IGR	opensea	IGR-opense		rs5564955	0
cg2611175	0.204436	0.64855	8.130439	5.15E-05	0.001688	2.430714	0.546332	0.750768	-0.20444	20	31642120	F	II	C20orf185	TSS1500	opensea	TSS1500-op		rs1929384	0
cg1456156	0.217363	0.471476	8.130032	5.15E-05	0.001688	2.430351	0.362794	0.580157	-0.21736	20	3401214	R	II		IGR	opensea	IGR-opense		rs5321831	36;27;18;14
cg0189178	0.236891	0.667327	8.130021	5.15E-05	0.001688	2.43034	0.548881	0.785772	-0.23689	3	1.78E+08	R	II		IGR	opensea	IGR-opense		rs5278634	27;1
cg2225319	0.213343	0.698664	8.129716	5.15E-05	0.001688	2.430068	0.591992	0.805336	-0.21334	7	28793763	R	II	CREB5	Body	opensea	Body-opens		rs5592178	24;12
cg1481817	0.211068	0.625262	8.129474	5.15E-05	0.001689	2.429852	0.519728	0.730796	-0.21107	17	73148480	F	II	HN1	5'UTR	shore	5'UTR-shore	chr17:7314	rs5299714	1;9
cg0544807	0.213948	0.660044	8.128394	5.16E-05	0.00169	2.428887	0.55307	0.767018	-0.21395	5	60917015	R	II		IGR	opensea	IGR-opense			
cg2228165	0.206323	0.649472	8.12789	5.16E-05	0.00169	2.428437	0.54631	0.752633	-0.20632	17	62197507	F	II	ERN1	Body	opensea	Body-opens		rs1513344	0;34
cg0181928	0.238184	0.619523	8.127267	5.16E-05	0.00169	2.42788	0.500431	0.738615	-0.23818	12	46006284	R	II		IGR	opensea	IGR-opense			
cg0272169	0.218643	0.493677	8.126805	5.17E-05	0.001691	2.427468	0.384356	0.602999	-0.21864	1	1.83E+08	F	II	C1orf14	TSS200	shore	TSS200-sho	chr1:18292		
cg0747846	0.232889	0.624474	8.126284	5.17E-05	0.001691	2.427002	0.508029	0.740918	-0.23289	8	91712588	R	II		IGR	opensea	IGR-opense		rs5444706	45;36;35;27;13
cg1172873	0.235595	0.56685	8.126169	5.17E-05	0.001691	2.426899	0.449053	0.684648	-0.23559	6	21711700	F	II	FLJ22536	Body	opensea	Body-opens		rs1810510	1
cg0546240	0.212901	0.681772	8.126035	5.17E-05	0.001691	2.426779	0.575322	0.788223	-0.2129	11	59266110	R	II		IGR	opensea	IGR-opense		rs5323318	39
cg0050804	0.207675	0.535962	8.125811	5.17E-05	0.001692	2.426579	0.432124	0.6398	-0.20768	17	40485194	F	II	STAT3	Body	opensea	Body-opens		rs7224339	21;35;37;45;46
cg0253143	0.212763	0.677411	8.125139	5.17E-05	0.001692	2.425979	0.571029	0.783792	-0.21276	7	98701226	R	II	SMURF1	Body	opensea	Body-opens		rs5399115	2
cg0568766	0.200794	0.690302	8.124687	5.18E-05	0.001693	2.425575	0.589905	0.790699	-0.20079	8	1.07E+08	R	II		IGR	opensea	IGR-opense		rs5272444	25
cg1420924	0.225687	0.513305	8.123962	5.18E-05	0.001693	2.424927	0.400462	0.626149	-0.22569	9	72877684	F	II	SMC5	Body	shelf	Body-shelf	chr9:72873		
cg0408426	0.206186	0.534202	8.122203	5.19E-05	0.001695	2.423355	0.431109	0.637295	-0.20619	1	1.51E+08	R	II		IGR	opensea	IGR-opense		rs1416869	43;40;1
cg2316146	0.220539	0.702575	8.121028	5.19E-05	0.001696	2.422304	0.592305	0.812845	-0.22054	1	84713356	R	II		IGR	opensea	IGR-opense		rs5695477	36;1
cg1551452	0.232943	0.517847	8.11968	5.2E-05	0.001697	2.421099	0.401376	0.634319	-0.23294	11	85854937	F	II		IGR	opensea	IGR-opense		rs5558250	0;22;33
cg0101984	0.246218	0.492496	8.119197	5.2E-05	0.001697	2.420668	0.369387	0.615605	-0.24622	11	1.19E+08	F	II	ABCG4	Body	shelf	Body-shelf	chr11:1190	rs5737133	0;32;45
cg0056118	0.241064	0.685849	8.11768	5.21E-05	0.001698	2.41931	0.565317	0.806381	-0.24106	1	64509006	R	II	ROR1	Body	opensea	Body-opens		rs5688256	2;1
cg1001643	0.24064	0.59478	8.117438	5.21E-05	0.001698	2.419094	0.47446	0.7151	-0.24064	10	3784156	F	II	LOC105376	Body	opensea	Body-opens		rs3711630	1;33
cg0972146	0.200957	0.688643	8.117008	5.21E-05	0.001699	2.41871	0.588165	0.789121	-0.20096	16	47747381	F	II		IGR	opensea	IGR-opense		rs1836555	0;23
cg1343257	0.220738	0.557337	8.116803	5.21E-05	0.001699	2.418526	0.446968	0.667706	-0.22074	2	59873661	F	II		IGR	opensea	IGR-opense		rs1820511	0;1;23
cg1636288	0.221227	0.601	8.116566	5.21E-05	0.001699	2.418314	0.490386	0.711613	-0.22123	6	21836670	F	II	CASC15	Body	opensea	Body-opens			
cg1868670	0.218559	0.497961	8.115086	5.22E-05	0.0017	2.41699	0.388681	0.60724	-0.21856	7	46737984	R	II		IGR	opensea	IGR-opense		rs5741107	1
cg1812588	0.221435	0.561128	8.115041	5.22E-05	0.0017	2.41695	0.450411	0.671846	-0.22143	18	70709973	R	II		IGR	opensea	IGR-opense		rs746471;r	43;36
cg1398071	0.239072	0.640182	8.112967	5.23E-05	0.001702	2.415095	0.520646	0.759718	-0.23907	2	2.18E+08	F	II	TNP1	TSS1500	opensea	TSS1500-op		rs5327672	8;17;28
cg0547959	0.261479	0.625697	8.112863	5.23E-05	0.001703	2.415001	0.494957	0.756437	-0.26148	11	1.35E+08	R	II		IGR	opensea	IGR-opense		rs5655423	40;22;16;2
cg1593012	0.23441	0.491504	8.110049	5.24E-05	0.001706	2.412483	0.374299	0.608709	-0.23441	14	33131712	R	II	AKAP6	Body	opensea	Body-opens		rs5513607	21;13;2;1
cg2592281	0.236057	0.594928	8.109196	5.24E-05	0.001706	2.41172	0.476899	0.712956	-0.23606	7	1.3E+08	F	II	TMEM209	Body	opensea	Body-opens		rs1496216	10
cg2719865	0.245723	0.419055	8.109018	5.24E-05	0.001706	2.41156	0.296194	0.541916	-0.24572	1	1.6E+08	R	II		IGR	opensea	IGR-opense			
cg2228401	0.204183	0.517211	8.108987	5.24E-05	0.001706	2.411532	0.415119	0.619302	-0.20418	4	1.07E+08	F	II	ARHGEF38	Body	opensea	Body-opens			
cg2756433	0.215933	0.45116	8.108777	5.25E-05	0.001706	2.411344	0.343193	0.559126	-0.21593	7	3592212	F	II	SDK1	Body	opensea	Body-opens		rs5735135	0;23;39;41
cg1886962	0.239647	0.575697	8.108695	5.25E-05	0.001706	2.411271	0.455873	0.69552	-0.23965	19	2260400	R	II		IGR	opensea	IGR-opense		rs4806834	41;27
cg1249604	0.221165	0.643068	8.107527	5.25E-05	0.001707	2.410225	0.532485	0.75365	-0.22117	10	19980391	R	II	MALRD1	Body	opensea	Body-opens		rs1506785	37
cg0655216	0.204576	0.612357	8.106554	5.26E-05	0.001707	2.409353	0.510069	0.714646	-0.20458	2	1926724	R	II	MYT1L	Body	opensea	Body-opens		rs2002352	2
cg0828054	0.205454	0.487632	8.106032	5.26E-05	0.001708	2.408887	0.384905	0.59036	-0.20545	1	26506100	F	II	CNKSR1	5'UTR	opensea	5'UTR-open			
cg0597049	0.22654	0.538118	8.104762	5.26E-05	0.001709	2.407749	0.424848	0.651388	-0.22654	17	2876230	R	II	RAP1GAP2	Body	opensea	Body-opens		rs5574398	7;6
cg0241426	0.208136	0.603936	8.104461	5.26E-05	0.001709	2.40748	0.499868	0.708004	-0.20814	3	45802101	R	II	SLC6A20	Body	opensea	Body-opens		rs1492844	30;2
cg2471546	0.221462	0.56641	8.104314	5.27E-05	0.001709	2.407347	0.455678	0.677141	-0.22146	7	3239363	F	II		IGR	opensea	IGR-opense		rs1417349	0;5;20;32;43
cg0450575	0.211387	0.484712	8.103867	5.27E-05	0.001709	2.406947	0.379018	0.590405	-0.21139	1	12774815	F	II	AADACL3	TSS1500	opensea	TSS1500-op		rs5599864	0;23;43
cg0558737	0.208747	0.599483	8.100945	5.28E-05	0.001712	2.404329	0.49511	0.703857	-0.20875	4	88189110	F	II		IGR	opensea	IGR-opense		rs1385613	6
cg0914310	0.202335	0.636307	8.10042	5.28E-05	0.001712	2.403859	0.53514	0.737475	-0.20233	10	15286861	R	II	FAM171A1	Body	opensea	Body-opens		rs1447476	25;12
cg1404655	0.204873	0.64892	8.096982	5.3E-05	0.001715	2.400778	0.546484	0.751356	-0.20487	2	1.67E+08	F	II	SCN1A	TSS1500	opensea	TSS1500-op		rs1494897	1;28;33;51
cg1193295	0.209663	0.593683	8.09693	5.3E-05	0.001715	2.400731	0.488851	0.698514	-0.20966	20	45202478	R	II	SLC13A3	Body	opensea	Body-opens		rs1905585	35;23;15;14;1
cg0977782	0.204919	0.632712	8.095419	5.31E-05	0.001716	2.399376	0.530252	0.735172	-0.20492	4	69047717	F	II	FTLP10	TSS1500	shore	TSS1500-sh	chr4:69048	rs5779433	50
cg2602006	0.208874	0.610332	8.095334	5.31E-05	0.001716	2.3993	0.505895	0.714769	-0.20887	6	52382441	R	II	TRAM2	Body	opensea	Body-opens			
cg1502744	0.218281	0.398721	8.09501	5.31E-05	0.001716	2.399009	0.28958	0.507861	-0.21828	4	56484062	R	II	NMU	Body	opensea	Body-opens		rs5505163	2
cg0327546	0.202171	0.638284	8.092276	5.32E-05	0.001718	2.396558	0.537199	0.73937	-0.20217	3	70800230	F	II		IGR	opensea	IGR-opense		rs5429714	2
cg0530422	0.227078	0.483129	8.092236	5.32E-05	0.001718	2.396522	0.36959	0.596669	-0.22708	5	4234168	F	II		IGR	opensea	IGR-opense		rs5679892	1
cg1009312	0.249378	0.567655	8.088561	5.34E-05	0.001721	2.393226	0.442966	0.692344	-0.24938	17	1335117	F	II	CRK	Body	opensea	Body-opens			
cg0778785	0.201685	0.670663	8.088467	5.34E-05	0.001721	2.393141	0.56982	0.771505	-0.20168	5	1.21E+08	F	II	ZNF474	Body	opensea	Body-opens		rs1439537	0;1;33;39;48;51
cg0853476	0.208912	0.498691	8.087663	5.34E-05	0.001722	2.39242	0.394235	0.603147	-0.20891	2	1.67E+08	R	II	SCN1A	5'UTR	opensea	5'UTR-open		rs1168125	43;29;2
cg0861205	0.270255	0.566167	8.087502	5.34E-05	0.001722	2.392275	0.431039	0.701294	-0.27025	7	35595790	R	II		IGR	opensea	IGR-opense		rs5523128	45;16;11;5
cg2257697	0.206584	0.505581	8.087092	5.34E-05	0.001722	2.391907	0.402289	0.608873	-0.20658	19	56852136	F	II		IGR	shore	IGR-shore	chr19:5685	rs1385039	1;50
cg0688664	0.224946	0.657616	8.0857	5.35E-05	0.001723	2.390657	0.545143	0.770089	-0.22495	12	95833248	F	II		IGR	opensea	IGR-opense		rs5496939	12;36
cg0989429	0.203792	0.492535	8.084429	5.36E-05	0.001723	2.389517	0.390639	0.594431	-0.20379	21	23179036	F	II		IGR	opensea	IGR-opense		rs1876472	0
cg1757351	0.205576	0.569322	8.084404	5.36E-05	0.001723	2.389494	0.466534	0.67211	-0.20558	2	2.11E+08	R	II	MYL1	TSS1500	opensea	TSS1500-op		rs5427566	2
cg2339847	0.242049	0.652076	8.084316	5.36E-05	0.001723	2.389415	0.531051	0.7731	-0.24205	6	1.38E+08	R	II	LOC10272	Body	opensea	Body-opens		rs7756138	49;46;39;1
cg0066919	0.209931	0.576572	8.084032	5.36E-05	0.001723	2.38916	0.471607	0.681537	-0.20993	15	35774388	F	II	DPH6	Body	opensea	Body-opens		rs1450952	0;1;17
cg1983504	0.206863	0.548453	8.08225	5.37E-05	0.001725	2.387561	0.445022	0.651884	-0.20686	13	50801738	R	II	DLEU1	Body	opensea	Body-opens		rs1427649	1
cg0395404	0.21061	0.600153	8.081046	5.37E-05	0.001726	2.386479	0.494848	0.705458	-0.21061	12	43247617	F	II		IGR	opensea	IGR-opense		rs1475955	1;29;50;51
cg2552286	0.206208	0.392566	8.07929	5.38E-05	0.001727	2.384903	0.289462	0.49567	-0.20621	11	34236648	R	II	ABTB2	Body	opensea	Body-opens		rs1138027	35
cg0797171	0.221094	0.629868	8.078434	5.38E-05	0.001728	2.384134	0.519321	0.740415	-0.22109	1	2E+08	R	II		IGR	opensea	IGR-opense			
cg1532941	0.204891	0.726185	8.078402	5.38E-05	0.001728	2.384105	0.62374	0.828631	-0.20489	5	33857193	F	II	ADAMTS12	Body	opensea	Body-opens		rs5357070	1;51
cg1659445	0.230088	0.525474	8.077948	5.39E-05	0.001728	2.383697	0.41043	0.640518	-0.23009	11	5365093	R	II	OR51B5	Body	opensea	Body-opens		rs1129968	43;30;2
cg0573078	0.246939	0.605202	8.077247	5.39E-05	0.001729	2.383067	0.481733	0.728672	-0.24694	7	17193341	R	II		IGR	opensea	IGR-opense		rs1382460	5;2
cg0204653	0.211267	0.677764	8.07683	5.39E-05	0.001729	2.382692	0.57213	0.783397	-0.21127	20	207858	F	II	DEFB129	TSS200	opensea	TSS200-ope		rs1808406	0;24;31;45
cg0032739	0.210295	0.421341	8.076529	5.39E-05	0.001729	2.382422	0.316193	0.526488	-0.2103	16	48642773	R	II	MIR5095	Body	shore	Body-shore	chr16:4864	rs5733662	50;36
cg0067295	0.238249	0.570269	8.076154	5.39E-05	0.001729	2.382085	0.451145	0.689394	-0.23825	4	1.7E+08	F	II	SH3RF1	Body	opensea	Body-opens		rs1920605	0;36;43;49
cg1976466	0.240383	0.442469	8.075677	5.4E-05	0.00173	2.381657	0.322277	0.56266	-0.24038	2	2.31E+08	R	I	SP110	Body	opensea	Body-opens		rs2303540	51
cg1405407	0.211861	0.466129	8.075134	5.4E-05	0.00173	2.381169	0.360198	0.572059	-0.21186	5	1.18E+08	F	II	HRAT56	Body	opensea	Body-opens		rs1137365	1;33
cg1761361	0.205496	0.607352	8.074114	5.4E-05	0.001731	2.380252	0.504604	0.7101	-0.2055	6	1.31E+08	R	II		IGR	opensea	IGR-opense			
cg1158758	0.211696	0.532817	8.074045	5.4E-05	0.001731	2.38019	0.426969	0.638666	-0.2117	3	1.53E+08	R	II		IGR	opensea	IGR-opense		rs3767672	46;33;32;25;19;1
cg1411748	0.207709	0.73648	8.073294	5.41E-05	0.001731	2.379515	0.632626	0.840335	-0.20771	1	1.58E+08	R	II	KIRREL	Body	opensea	Body-opens		rs5502760	32;2
cg1750416	0.200569	0.583071	8.072701	5.41E-05	0.001731	2.378982	0.482786	0.683355	-0.20057	1	79653656	R	II		IGR	opensea	IGR-opense		rs5720827	12;2
cg2450622	0.433966	0.406305	8.072536	5.41E-05	0.001731	2.378834	0.189322	0.623287	-0.43397	1	1.1E+08	R	I	GSTM1	TSS200	island	TSS200-isla	chr1:1102	rs5737347	36;19;8
cg0846625	0.206059	0.606386	8.071986	5.41E-05	0.001731	2.378339	0.503357	0.709415	-0.20606	2	1.9E+08	F	II	COL5A2	Body	opensea	Body-opens			
cg0045893	0.201699	0.444552	8.071218	5.42E-05	0.001732	2.377649	0.343702	0.545401	-0.2017	2	2.08E+08	F	II		IGR	opensea	IGR-opense		rs1161038	19
cg2299864	0.216841	0.443901	8.071074	5.42E-05	0.001732	2.377519	0.335481	0.552321	-0.21684	11	85758883	R	II	PICALM	5'UTR	opensea	5'UTR-open		rs5584712	32;31
cg0256275	0.206846	0.422042	8.068329	5.43E-05	0.001735	2.375051	0.318619	0.525465	-0.20685	21	29629836	R	II		IGR	opensea	IGR-opense		rs5311632	47;6;2;1
cg1204735	0.200407	0.435255	8.067156	5.44E-05	0.001735	2.373996	0.335052	0.535459	-0.20041	6	49490175	R	II	GLYATL3	Body	opensea	Body-opens		rs5756915	23
cg0225242	0.206056	0.510074	8.065964	5.44E-05	0.001736	2.372924	0.407046	0.613102	-0.20606	5	1.28E+08	F	II		IGR	opensea	IGR-opense			
cg1833497	0.205031	0.633763	8.063566	5.45E-05	0.001738	2.370767	0.531247	0.736279	-0.20503	3	1.28E+08	F	II		IGR	shore	IGR-shore	chr3:1282	rs5552229	0;1;18
cg0152848	0.200111	0.594484	8.063499	5.45E-05	0.001738	2.370706	0.494429	0.69454	-0.20011	11	1.17E+08	R	II	BACE1	Body	opensea	Body-opens			
cg1683178	0.224183	0.634061	8.063164	5.45E-05	0.001738	2.370405	0.52197	0.746153	-0.22418	15	33539925	F	II	TMCO5B	TSS200	opensea	TSS200-ope		rs1443812	1;43
cg0036104	0.211142	0.391932	8.062741	5.46E-05	0.001738	2.370024	0.286361	0.497503	-0.21114	13	73354967	R	II	PIBF1	TSS1500	shore	TSS1500-sh	chr13:7335	rs5485960	43;23;3
cg1721099	0.242153	0.671087	8.062198	5.46E-05	0.001739	2.369536	0.55001	0.792163	-0.24215	1	2.25E+08	F	II		IGR	opensea	IGR-opense		rs1387259	1;7;20
cg0844840	0.22542	0.550538	8.061455	5.46E-05	0.001739	2.368867	0.437828	0.663248	-0.22542	10	54633831	R	II		IGR	shelf	IGR-shelf	chr10:5463	rs1864750	29;2;1
cg1765377	0.205753	0.59341	8.061321	5.46E-05	0.001739	2.368747	0.490534	0.696286	-0.20575	7	1.36E+08	F	II		IGR	shelf	IGR-shelf	chr7:1356	rs1847033	0;19
cg0683708	0.222567	0.634828	8.060796	5.46E-05	0.00174	2.368274	0.523544	0.746111	-0.22257	4	1.74E+08	F	II	GALNTL6	Body	opensea	Body-opens		rs5466470	0;43
cg2472869	0.221514	0.441755	8.060534	5.47E-05	0.00174	2.368038	0.330998	0.552512	-0.22151	15	97081353	F	II		IGR	opensea	IGR-opense		rs1423581	1;4
cg1324694	0.243874	0.531294	8.05974	5.47E-05	0.00174	2.367324	0.409357	0.65323	-0.24387	3	41878377	F	II	ULK4	Body	opensea	Body-opens		rs5439213	26
cg1745171	0.214019	0.591968	8.058762	5.47E-05	0.001741	2.366443	0.484959	0.698977	-0.21402	12	1.22E+08	R	II	HPD	Body	opensea	Body-opens		rs1848667	37;19;2
cg0625075	0.232606	0.583506	8.058352	5.48E-05	0.001741	2.366075	0.467203	0.699809	-0.23261	15	49329117	R	II	SECISBP2L	Body	opensea	Body-opens		rs5559597	46
cg0530739	0.218665	0.584932	8.057882	5.48E-05	0.001742	2.365651	0.4756	0.694265	-0.21867	2	62611092	F	II		IGR	opensea	IGR-opense		rs5698314	5;45
cg2555739	0.220566	0.573323	8.056725	5.48E-05	0.001742	2.364609	0.46304	0.683606	-0.22057	10	62296386	R	II	ANK3	Body	opensea	Body-opens		rs5646519	48;15
cg0409488	0.227941	0.416933	8.05662	5.48E-05	0.001743	2.364515	0.302962	0.530903	-0.22794	17	38023830	F	II	ZPBP2	TSS1500	shore	TSS1500-sh	chr17:3802	rs5679148	25
cg1492894	0.213756	0.50866	8.056539	5.48E-05	0.001743	2.364442	0.401781	0.615538	-0.21376	6	16420347	R	II	ATXN1	5'UTR	opensea	5'UTR-open		rs5391439	47;40;12;2
cg0296737	0.204202	0.582368	8.05537	5.49E-05	0.001743	2.363389	0.480267	0.684469	-0.2042	15	99152767	R	II		IGR	opensea	IGR-opense		rs5665746	20;14;1
cg1971875	0.222553	0.626245	8.05483	5.49E-05	0.001744	2.362903	0.514968	0.737521	-0.22255	15	93610211	F	II	RGMA	Body	shore	Body-shore	chr15:9361	rs5348100	0
cg2453564	0.225934	0.651681	8.053924	5.5E-05	0.001744	2.362087	0.538714	0.764648	-0.22593	1	1.08E+08	R	II		IGR	opensea	IGR-opense		rs7876118	26;19;5
cg1788306	0.238223	0.643515	8.050524	5.51E-05	0.001747	2.359024	0.524403	0.762627	-0.23822	15	57277634	R	II	TCF12	Body	opensea	Body-opens			
cg0993999	0.206227	0.615878	8.0501	5.52E-05	0.001747	2.358641	0.512764	0.718991	-0.20623	10	89434856	R	II	PAPSS2	Body	opensea	Body-opens		rs5684019	1
cg2708360	0.220691	0.580538	8.048958	5.52E-05	0.001748	2.357612	0.470193	0.690884	-0.22069	5	1.78E+08	R	II	COL23A1	Body	opensea	Body-opens		rs1764152	51;2;1
cg1182874	0.221498	0.621382	8.04502	5.54E-05	0.001752	2.354063	0.510632	0.732131	-0.2215	11	10715767	F	II	MRVI1	TSS1500	opensea	TSS1500-op		rs1167644	22;28
cg0445819	0.231889	0.582357	8.043686	5.55E-05	0.001753	2.35286	0.466413	0.698302	-0.23189	1	2.2E+08	R	II	RAB3GAP2	Body	opensea	Body-opens		rs1399811	12
cg1680170	0.245591	0.435076	8.041959	5.55E-05	0.001754	2.351302	0.31228	0.557871	-0.24559	5	64110063	R	II	CWC27	Body	opensea	Body-opens		rs1483034	23
cg2339463	0.241885	0.661702	8.041679	5.56E-05	0.001754	2.351049	0.54076	0.782645	-0.24189	2	2.08E+08	F	II	LOC10192	Body	opensea	Body-opens		rs1438199	0;15;44
cg0187952	0.235454	0.661135	8.041266	5.56E-05	0.001755	2.350678	0.543408	0.778862	-0.23545	3	1.14E+08	R	II	LOC10192	Body	opensea	Body-opens		rs1831695	8;4;2
cg0855121	0.201972	0.654874	8.040658	5.56E-05	0.001755	2.350129	0.553888	0.75586	-0.20197	7	90573393	R	II	CDK14	Body	opensea	Body-opens			
cg0340953	0.225343	0.559725	8.039248	5.57E-05	0.001756	2.348857	0.447053	0.672397	-0.22534	6	47071840	F	II		IGR	opensea	IGR-opense		rs1170269	0;26;32
cg1938713	0.203286	0.575243	8.038698	5.57E-05	0.001757	2.348361	0.4736	0.676886	-0.20329	1	1.58E+08	F	II		IGR	opensea	IGR-opense		rs1830832	23;50
cg1250226	0.209436	0.658565	8.038609	5.57E-05	0.001757	2.34828	0.553847	0.763283	-0.20944	8	1.25E+08	F	II	FER1L6	Body	opensea	Body-opens		rs5392929	0
cg0231997	0.210594	0.478969	8.03766	5.57E-05	0.001757	2.347424	0.373672	0.584266	-0.21059	15	62798895	R	II		IGR	opensea	IGR-opense		rs5474270	24;15;1

cg1201152	0.201867	0.324203	8.036383	5.58E-05	0.001759	2.346272	0.22327	0.425137	-0.20187	2	1.79E+08	F	II	OSBPL6	Body	opensea	Body-opens		rs5604498	1;41
cg2729146	0.227478	0.582249	8.036187	5.58E-05	0.001759	2.346095	0.46851	0.695988	-0.22748	2	9878816	F	I		IGR	opensea	IGR-opense		rs5676309	29
cg1503439	0.219812	0.422595	8.035677	5.58E-05	0.001759	2.345635	0.312689	0.532501	-0.21981	3	1.53E+08	R	II		IGR	opensea	IGR-opense		rs1847278	12;1
cg2523711	0.203217	0.599195	8.034492	5.59E-05	0.00176	2.344564	0.497586	0.700803	-0.20322	2	80551812	R	II	CTNNA2	Body	shelf	Body-shelf	chr2:8054	rs5339263	2
cg2570427	0.21407	0.688057	8.032855	5.6E-05	0.001761	2.343087	0.581022	0.795092	-0.21407	6	1.35E+08	R	II		IGR	opensea	IGR-opense		rs1846997	22
cg0530060	0.249023	0.629502	8.030636	5.61E-05	0.001763	2.341084	0.504991	0.754014	-0.24902	12	52235472	R	II		IGR	opensea	IGR-opense		rs1258068	14;1
cg1155472	0.200819	0.66185	8.030581	5.61E-05	0.001763	2.341034	0.561441	0.762259	-0.20082	15	83242028	F	II	CPEB1	TSS1500	opensea	TSS1500-op			
cg0444657	0.247075	0.574634	8.030195	5.61E-05	0.001763	2.340685	0.451097	0.698172	-0.24708	4	1.07E+08	R	II		IGR	opensea	IGR-opense		rs5289070	30;16;14;2
cg1668012	0.250029	0.500698	8.029194	5.62E-05	0.001764	2.339781	0.375684	0.625712	-0.25003	1	2.07E+08	R	II	LGTN	Body	shelf	Body-shelf	chr1:2067	rs5472347	41;2;1
cg0768959	0.220585	0.527053	8.028406	5.62E-05	0.001764	2.339069	0.416761	0.637346	-0.22058	15	48138213	R	II	LINC01491	Body	opensea	Body-opens		rs5277524	1
cg1078208	0.21122	0.494762	8.028014	5.62E-05	0.001765	2.338715	0.389152	0.600372	-0.21122	1	51554444	F	II		IGR	opensea	IGR-opense		rs1924008	22
cg0565908	0.290514	0.53571	8.027867	5.62E-05	0.001765	2.338582	0.390453	0.680967	-0.29051	7	1.14E+08	F	II		IGR	opensea	IGR-opense		rs5780346	1;28
cg2422363	0.221399	0.511047	8.025353	5.63E-05	0.001767	2.336311	0.400347	0.621747	-0.2214	5	7978936	F	II		IGR	opensea	IGR-opense		rs1137114	1
cg0917051	0.20903	0.4492	8.024709	5.64E-05	0.001768	2.335729	0.344685	0.553715	-0.20903	22	45067941	R	II	PRR5	5'UTR	shelf	5'UTR-shelf	chr22:450	rs5586922	51
cg1967559	0.20643	0.675822	8.023379	5.64E-05	0.001769	2.334528	0.572607	0.779038	-0.20643	6	1.39E+08	F	II		IGR	opensea	IGR-opense		rs1493102	1;30;39
cg0856193	0.20352	0.454805	8.021936	5.65E-05	0.00177	2.333223	0.353045	0.556565	-0.20352	5	1.31E+08	R	II	CDC42SE2	5'UTR	opensea	5'UTR-oper		rs7731860	36;18
cg1063440	0.216804	0.530386	8.021787	5.65E-05	0.00177	2.333088	0.421984	0.638788	-0.2168	12	19585266	R	II		IGR	opensea	IGR-opense			
cg1292364	0.221833	0.681071	8.020735	5.66E-05	0.00177	2.332137	0.570155	0.791987	-0.22183	20	20584339	F	II	RALGAPA2	Body	opensea	Body-opens		rs1857471	1;5;31;47
cg2421273	0.215876	0.528438	8.020163	5.66E-05	0.001771	2.33162	0.4205	0.636376	-0.21588	15	69396631	R	II	MIR548H4	Body	opensea	Body-opens		rs5666813	27;9
cg1459531	0.212655	0.547446	8.019673	5.66E-05	0.001771	2.331177	0.441119	0.653773	-0.21265	5	1.71E+08	R	II		IGR	opensea	IGR-opense		rs5284036	8;2
cg1547083	0.231185	0.598489	8.018933	5.66E-05	0.001772	2.330508	0.482897	0.714082	-0.23118	7	55603306	F	II	VOPP1	Body	opensea	Body-opens		rs5464996	25;38
cg1314774	0.205013	0.57325	8.018287	5.67E-05	0.001773	2.329924	0.470743	0.675756	-0.20501	1	55997258	F	II		IGR	opensea	IGR-opense		rs5413864	0;6;32;41
cg2752779	0.22169	0.56516	8.018099	5.67E-05	0.001773	2.329754	0.454315	0.676005	-0.22169	7	1773245	F	I	ELFN1	5'UTR	opensea	5'UTR-oper		rs5336539	0;17
cg1996834	0.211588	0.603781	8.016009	5.68E-05	0.001774	2.327863	0.497987	0.709575	-0.21159	11	34579495	R	II		IGR	opensea	IGR-opense		rs5638506	1
cg0122346	0.213647	0.575539	8.015947	5.68E-05	0.001774	2.327808	0.468715	0.682362	-0.21365	6	1.17E+08	F	II		IGR	opensea	IGR-opense		rs5606691	0;14;35
cg0648443	0.206283	0.657169	8.015834	5.68E-05	0.001774	2.327705	0.554027	0.76031	-0.20628	5	1.29E+08	F	II	ADAMTS19	Body	shelf	Body-shelf	chr5:1287	rs1424569	0;21
cg2514921	0.208908	0.595646	8.015789	5.68E-05	0.001774	2.327665	0.491192	0.7001	-0.20891	2	2.05E+08	F	II	PARD3B	Body	shore	Body-shore	chr2:2054	rs1473126	8;51
cg1825888	0.23312	0.477585	8.015326	5.68E-05	0.001774	2.327246	0.361026	0.594145	-0.23312	5	95403859	F	II	LOC10192	Body	opensea	Body-opens		rs6885863	5;17;40
cg1286782	0.229354	0.512497	8.014604	5.69E-05	0.001775	2.326593	0.39782	0.627174	-0.22935	6	1.45E+08	F	II	UTRN	Body	opensea	Body-opens		rs5557031	11
cg1365905	0.223875	0.555899	8.010619	5.71E-05	0.001778	2.322987	0.443961	0.667836	-0.22388	2	2.23E+08	R	II	SGPP2	Body	opensea	Body-opens		rs1917004	14;2
cg1904406	0.201623	0.661759	8.009865	5.71E-05	0.001778	2.322305	0.560948	0.762571	-0.20162	3	1.52E+08	R	II		IGR	opensea	IGR-opense		rs3702829	2
cg1785723	0.200558	0.527541	8.009545	5.71E-05	0.001779	2.322015	0.427262	0.62782	-0.20056	5	17213797	R	II	LOC28569	Body	shelf	Body-shelf	chr5:1721	rs1864125	38;2
cg0539009	0.223692	0.624814	8.008297	5.72E-05	0.00178	2.320884	0.512968	0.73666	-0.22369	4	48298462	R	II		IGR	opensea	IGR-opense		rs1403655	43;24;2
cg0509004	0.219754	0.508893	8.007515	5.72E-05	0.001781	2.320177	0.399017	0.61877	-0.21975	7	1.38E+08	F	II		IGR	opensea	IGR-opense		rs1135875	28;40
cg1782193	0.202509	0.697861	8.006359	5.73E-05	0.001782	2.31913	0.596607	0.799115	-0.20251	2	1.84E+08	R	II		IGR	opensea	IGR-opense		rs1387278	42
cg1599144	0.203236	0.67152	8.005839	5.73E-05	0.001782	2.318659	0.569902	0.773138	-0.20324	11	4721506	R	II		IGR	opensea	IGR-opense		rs5345676	2
cg1007731	0.201761	0.400101	8.00583	5.73E-05	0.001782	2.318651	0.29922	0.500981	-0.20176	2	1.68E+08	R	II	XIRP2-AS1	Body	opensea	Body-opens		rs5738105	24
cg1720857	0.217981	0.639517	8.005048	5.73E-05	0.001782	2.317943	0.530526	0.748508	-0.21798	9	1.11E+08	F	II		IGR	opensea	IGR-opense		rs5649433	13;27
cg1050461	0.212635	0.541996	8.003187	5.74E-05	0.001784	2.316257	0.435679	0.648314	-0.21264	2	59882837	R	II		IGR	opensea	IGR-opense		rs1128738	18
cg1245683	0.200653	0.690279	8.003068	5.74E-05	0.001784	2.31615	0.589953	0.790606	-0.20065	20	22994357	F	II		IGR	opensea	IGR-opense		rs5736643	1;19;43
cg0595491	0.23002	0.615761	8.00292	5.74E-05	0.001784	2.316015	0.500751	0.730771	-0.23002	1	66296098	F	II	PDE4B	5'UTR	opensea	5'UTR-oper			
cg0084846	0.211121	0.608854	8.001532	5.75E-05	0.001785	2.314758	0.503294	0.714414	-0.21112	5	1.77E+08	F	II	PRELID1	Body	shore	Body-shore	chr5:1767	rs7695015	0;13;14;29;30;46
cg1261325	0.272425	0.470073	8.001212	5.75E-05	0.001785	2.314468	0.333861	0.606286	-0.27242	20	20393632	F	II	RALGAPA2	Body	opensea	Body-opens		rs3685910	0
cg0472874	0.208019	0.499508	8.001026	5.75E-05	0.001785	2.3143	0.395498	0.603518	-0.20802	10	1.17E+08	R	II	ATRNL1	Body	opensea	Body-opens		rs1155226	51;50;36;7
cg0721440	0.218796	0.469334	8.000202	5.76E-05	0.001786	2.313553	0.359937	0.578732	-0.2188	1	16312238	R	II		IGR	opensea	IGR-opense		rs1841809	1
cg0563908	0.206339	0.570265	7.998176	5.77E-05	0.001787	2.311717	0.467096	0.673435	-0.20634	2	2.08E+08	F	II		IGR	opensea	IGR-opense		rs1138523	0
cg2213161	0.231433	0.560434	7.997769	5.77E-05	0.001787	2.311348	0.444717	0.67615	-0.23143	6	20669947	R	II	CDKAL1	Body	opensea	Body-opens			
cg2673695	0.245765	0.648287	7.997703	5.77E-05	0.001787	2.311288	0.525405	0.77117	-0.24576	11	34645679	R	II	EHF	5'UTR	opensea	5'UTR-oper		rs1729217	45;32;14
cg1665009	0.204877	0.70789	7.995217	5.78E-05	0.001789	2.309035	0.605451	0.810328	-0.20488	4	1.41E+08	F	II	MAML3	Body	opensea	Body-opens		rs6140140	36;50
cg1743561	0.260902	0.581311	7.994502	5.79E-05	0.00179	2.308386	0.450861	0.711762	-0.2609	22	38731297	F	II	LOC40092	5'UTR	opensea	5'UTR-oper		rs5558848	19;42
cg0445364	0.276577	0.535947	7.994176	5.79E-05	0.00179	2.308091	0.397659	0.674236	-0.27658	2	40579303	F	II	SLC8A1	Body	opensea	Body-opens		rs1858235	13;17;21
cg1380764	0.227353	0.614686	7.993777	5.79E-05	0.001791	2.307729	0.50101	0.728362	-0.22735	20	38785218	R	II		IGR	opensea	IGR-opense		rs1844193	24
cg2271301	0.214442	0.538968	7.993329	5.79E-05	0.001791	2.307323	0.431747	0.646189	-0.21444	5	32060539	R	II	PDZD2	Body	opensea	Body-opens		rs5422308	40;27;10
cg0843240	0.204677	0.419881	7.993161	5.79E-05	0.001791	2.30717	0.317543	0.52222	-0.20468	3	24531858	R	II	THRB	5'UTR	shelf	5'UTR-shelf	chr3:2453	rs1387251	33
cg1502524	0.250624	0.501174	7.992023	5.8E-05	0.001792	2.306138	0.375861	0.626486	-0.25062	2	74811430	R	II	C2orf65	Body	opensea	Body-opens			
cg1773152	0.219487	0.622726	7.989595	5.81E-05	0.001794	2.303936	0.512983	0.73247	-0.21949	3	27235572	R	II	NEK10	Body	opensea	Body-opens		rs1385442	51;15;6
cg1684510	0.207272	0.333053	7.989088	5.81E-05	0.001795	2.303476	0.229417	0.436689	-0.20727	22	17959388	F	II	CECR2	5'UTR	opensea	5'UTR-oper		rs5652003	1;15
cg1968543	0.216868	0.579981	7.988675	5.81E-05	0.001795	2.303101	0.471547	0.688415	-0.21687	18	20394356	F	II		IGR	opensea	IGR-opense		rs1911024	20;31
cg2139849	0.230152	0.609979	7.988562	5.81E-05	0.001795	2.302999	0.494903	0.725056	-0.23015	2	1.56E+08	R	II		IGR	opensea	IGR-opense		rs5479778	23;2
cg2693375	0.207647	0.551058	7.987634	5.82E-05	0.001796	2.302157	0.447234	0.654882	-0.20765	2	1.45E+08	R	II	GTDC1	Body	opensea	Body-opens		rs5468782	28;1
cg2026562	0.218722	0.642552	7.986638	5.82E-05	0.001796	2.301253	0.533191	0.751913	-0.21872	4	26324308	F	II	RBPJ	Body	shore	Body-shore	chr4:2632	rs5445254	14
cg0586317	0.240682	0.575392	7.986435	5.83E-05	0.001796	2.301069	0.455051	0.695733	-0.24068	4	3023500	R	II	GRK4	Body	opensea	Body-opens		rs5345231	18;2
cg0035872	0.208713	0.705275	7.986415	5.83E-05	0.001796	2.30105	0.600918	0.809631	-0.20871	3	1.83E+08	F	II		IGR	opensea	IGR-opense		rs1467870	0
cg0350823	0.206108	0.721527	7.984666	5.83E-05	0.001797	2.299463	0.618473	0.824581	-0.20611	14	23445994	R	II	JUB	5'UTR	shelf	5'UTR-shelf	chr14:234	rs2019591	51;7;1
cg2456211	0.2068	0.647523	7.984662	5.83E-05	0.001797	2.299459	0.544123	0.750923	-0.2068	13	80324480	F	II		IGR	opensea	IGR-opense		rs1914250	26
cg1236778	0.234222	0.460682	7.981081	5.85E-05	0.0018	2.296208	0.343571	0.577793	-0.23422	21	39290161	F	II	KCNJ6	TSS1500	shore	TSS1500-sh	chr21:392	rs5482287	5
cg1968698	0.207563	0.698137	7.98105	5.85E-05	0.0018	2.296181	0.594356	0.801919	-0.20756	6	13182594	F	II	PHACTR1	Body	opensea	Body-opens		rs1409113	0;1;14;45
cg0135965	0.21368	0.615548	7.978515	5.87E-05	0.001802	2.293878	0.508708	0.722388	-0.21368	9	1.19E+08	F	II		IGR	opensea	IGR-opense		rs5582480	7
cg0579016	0.202217	0.605453	7.978358	5.87E-05	0.001802	2.293735	0.504344	0.706561	-0.20222	10	72465428	R	II	ADAMTS14	Body	opensea	Body-opens		rs5630811	28;1
cg0664150	0.229911	0.637535	7.97824	5.87E-05	0.001802	2.293628	0.522579	0.75249	-0.22991	3	48959341	F	II	ARIH2	5'UTR	shelf	5'UTR-shelf	chr3:4895	rs5467614	9
cg0421535	0.220873	0.624432	7.977981	5.87E-05	0.001802	2.293392	0.513995	0.734869	-0.22087	2	1.53E+08	R	II	FMNL2	Body	opensea	Body-opens		rs5650518	2
cg0867448	0.206229	0.60951	7.977715	5.87E-05	0.001803	2.293151	0.506396	0.712624	-0.20623	17	14933074	F	II	CDRT7	TSS1500	opensea	TSS1500-op		rs5730357	0;8;38
cg1565415	0.239934	0.666096	7.975198	5.88E-05	0.001805	2.290865	0.546129	0.786063	-0.23993	11	9965512	R	II	SBF2	Body	opensea	Body-opens			
cg2161796	0.21126	0.573629	7.974851	5.88E-05	0.001805	2.29055	0.467999	0.679259	-0.21126	16	13532223	R	II		IGR	opensea	IGR-opense			
cg1568569	0.232495	0.552606	7.974614	5.89E-05	0.001805	2.290333	0.436359	0.668854	-0.23249	17	18247645	R	II	SHMT1	Body	opensea	Body-opens		rs1872239	33;15;1
cg1353101	0.23992	0.549956	7.97328	5.89E-05	0.001806	2.289121	0.429996	0.669916	-0.23992	2	50883765	R	II	NRXN1	Body	opensea	Body-opens		rs5690431	1
cg1279556	0.201151	0.617686	7.97303	5.89E-05	0.001806	2.288895	0.517111	0.718262	-0.20115	18	60020637	F	II	TNFRSF11A	Body	opensea	Body-opens		rs5696857	33;47;48
cg0138615	0.210329	0.589402	7.972312	5.9E-05	0.001807	2.288242	0.484238	0.694566	-0.21033	6	29403982	R	II		IGR	opensea	IGR-opense		rs1143653	2
cg2217504	0.240986	0.455141	7.971742	5.9E-05	0.001807	2.287724	0.334648	0.575634	-0.24099	7	37779764	R	II	GPR141	TSS1500	opensea	TSS1500-op		rs5422985	42;14;12;2
cg1083956	0.246773	0.639485	7.970795	5.9E-05	0.001808	2.286863	0.516098	0.762872	-0.24677	12	24893783	F	II		IGR	opensea	IGR-opense		rs1142764	1
cg0938343	0.20835	0.580687	7.970694	5.91E-05	0.001808	2.286771	0.476512	0.684862	-0.20835	8	1.3E+08	R	II		IGR	opensea	IGR-opense		rs5333346	8;2;1
cg0387267	0.202856	0.485228	7.969489	5.91E-05	0.001809	2.285675	0.3838	0.586655	-0.20286	6	253022	R	II		IGR	opensea	IGR-opense			
cg1219430	0.209446	0.732956	7.968746	5.91E-05	0.00181	2.284999	0.628233	0.837679	-0.20945	7	18598398	R	II	HDAC9	Body	opensea	Body-opens			
cg0287341	0.216315	0.481414	7.968478	5.92E-05	0.00181	2.284756	0.373257	0.589571	-0.21631	15	52545136	F	II	MYO5C	Body	opensea	Body-opens		rs7401564	1
cg0982766	0.234222	0.58841	7.967811	5.92E-05	0.001811	2.284149	0.471299	0.705521	-0.23422	3	1.12E+08	R	II	SLC35A5	5'UTR	shore	5'UTR-shor	chr3:1122	rs5474139	46;27;11;9
cg1695109	0.211562	0.640161	7.967646	5.92E-05	0.001811	2.283999	0.53438	0.745942	-0.21156	2	1.66E+08	F	II	SLC38A11	Body	opensea	Body-opens		rs5675547	4;46
cg2023086	0.200933	0.657666	7.966426	5.93E-05	0.001812	2.282889	0.5572	0.758133	-0.20093	2	1.71E+08	F	II	METTL5	Body	opensea	Body-opens		rs5503468	0;28;43
cg1479039	0.202546	0.677285	7.965825	5.93E-05	0.001812	2.282344	0.576012	0.778558	-0.20255	18	456806	F	II	COLEC12	Body	opensea	Body-opens			
cg1636965	0.212979	0.47103	7.965807	5.93E-05	0.001812	2.282327	0.364541	0.57752	-0.21298	5	1.49E+08	F	II	CSF1R	TSS200	opensea	TSS200-ope		rs5364286	1;37
cg0718708	0.205252	0.614458	7.963327	5.94E-05	0.001814	2.280071	0.511832	0.717084	-0.20525	20	1825226	F	II		IGR	opensea	IGR-opense		rs5592059	22
cg1308540	0.21022	0.644258	7.963159	5.94E-05	0.001814	2.279918	0.539148	0.749368	-0.21022	1	2.33E+08	R	II		IGR	opensea	IGR-opense			
cg0288994	0.215314	0.602389	7.962442	5.95E-05	0.001815	2.279266	0.494732	0.710046	-0.21531	22	36239415	R	II	RBFOX2	Body	opensea	Body-opens		rs5746166	24;23
cg0127180	0.201883	0.590434	7.962278	5.95E-05	0.001815	2.279116	0.489493	0.691376	-0.20188	3	55694954	F	II	C3orf51	TSS1500	opensea	TSS1500-op		rs5724973	0;2
cg0330572	0.227835	0.593798	7.961358	5.95E-05	0.001815	2.278279	0.479881	0.707716	-0.22783	11	8866125	R	II	ST5	5'UTR	opensea	5'UTR-oper			
cg1013919	0.214297	0.624888	7.961295	5.95E-05	0.001815	2.278222	0.51774	0.732037	-0.2143	2	2.17E+08	R	II	MREG	Body	opensea	Body-opens		rs1301439	20
cg1726612	0.251203	0.578122	7.961111	5.95E-05	0.001815	2.278055	0.452521	0.703724	-0.2512	21	30476810	R	II	MAP3K7CL	5'UTR	opensea	5'UTR-oper		rs1870250	6;2
cg1260253	0.223254	0.475385	7.958833	5.97E-05	0.001817	2.275981	0.363758	0.587012	-0.22325	18	4445094	R	II	DLGAP1	5'UTR	opensea	5'UTR-oper		rs5782587	48;14;1
cg1382815	0.228264	0.657802	7.957735	5.97E-05	0.001818	2.274981	0.543669	0.771934	-0.22826	6	56696399	R	II	DST	Body	opensea	Body-opens			
cg1065913	0.209155	0.665653	7.957033	5.98E-05	0.001818	2.274342	0.561076	0.770231	-0.20915	5	97949352	R	II		IGR	opensea	IGR-opense		rs7607706	11;1
cg0735220	0.202854	0.486345	7.956164	5.98E-05	0.001819	2.273551	0.384918	0.587772	-0.20285	4	1.23E+08	F	II		IGR	opensea	IGR-opense		rs3692593	1;31;47
cg1067124	0.205675	0.581045	7.955966	5.98E-05	0.001819	2.273371	0.478208	0.683883	-0.20568	20	58142036	F	II		IGR	opensea	IGR-opense		rs3848705	41;49;51
cg1688997	0.200424	0.532723	7.955102	5.99E-05	0.00182	2.272584	0.432511	0.632935	-0.20042	4	1.55E+08	R	II		IGR	opensea	IGR-opense		rs1166166	44;26;25
cg0666577	0.213179	0.636819	7.954971	5.99E-05	0.00182	2.272464	0.530229	0.743409	-0.21318	10	73152727	F	II		IGR	shelf	IGR-shelf	chr10:731	rs1486660	23
cg1586301	0.20733	0.492069	7.952477	6E-05	0.001823	2.270193	0.388404	0.595734	-0.20733	1	24915946	F	II	NCMAP	5'UTR	opensea	5'UTR-oper		rs1900311	1;38;39
cg2401896	0.205627	0.696699	7.952419	6E-05	0.001823	2.27014	0.593886	0.799512	-0.20563	15	83226022	F	II	CPEB1	Body	opensea	Body-opens		rs5488322	15
cg2188618	0.207593	0.510666	7.951783	6E-05	0.001823	2.26956	0.406869	0.614462	-0.20759	11	1.2E+08	F	II		IGR	shelf	IGR-shelf	chr11:120	rs1163508	12
cg1326246	0.219298	0.655189	7.950905	6.01E-05	0.001824	2.268761	0.545539	0.764838	-0.2193	3	1.7E+08	F	II	LRRIQ4	TSS1500	opensea	TSS1500-op		rs5505620	8
cg1832458	0.226847	0.566566	7.949342	6.02E-05	0.001825	2.267336	0.453142	0.679989	-0.22685	5	1.43E+08	F	II		IGR	opensea	IGR-opense		rs1493442	10
cg1880185	0.200403	0.649163	7.949184	6.02E-05	0.001825	2.267192	0.548962	0.749365	-0.2004	1	2.46E+08	F	II	SMYD3	Body	opensea	Body-opens		rs1843254	12;50
cg2080846	0.212442	0.617099	7.947981	6.02E-05	0.001826	2.266096	0.510878	0.72332	-0.21244	13	85853402	F	II		IGR	opensea	IGR-opense		rs5777550	0;11;30;32
cg0267636	0.212582	0.618485	7.945319	6.04E-05	0.001828	2.263669	0.512194	0.724776	-0.21258	3	44007655	R	II		IGR	opensea	IGR-opense		rs5478928	36;35;1
cg1484660	0.211082	0.490476	7.944435	6.04E-05	0.001829	2.262863	0.384935	0.596017	-0.21108	8	87718912	R	II	CNGB3	Body	opensea	Body-opens		rs5386991	39;23
cg0483384	0.207272	0.624456	7.944349	6.04E-05	0.001829	2.262785	0.52082	0.728092	-0.20727	19	44286076	F	II	KCNN4	TSS1500	opensea	TSS1500-op			
cg0675094	0.207515	0.695632	7.94294	6.05E-05	0.00183	2.2615	0.591874	0.799389	-0.20751	6	31019554	F	II		IGR	opensea	IGR-opense		rs2394427	8
cg2078018	0.219543	0.438539	7.941226	6.06E-05	0.001832	2.259937	0.328768	0.548311	-0.21954	5	1.22E+08	F	II	SNX24	Body	opensea	Body-opens		rs5673432	3;32;33
cg0141512	0.219907	0.565111	7.940959	6.06E-05	0.001832	2.259694	0.455157	0.675064	-0.21991	2	85929746	R	II		IGR	opensea	IGR-opense		rs1829442	43;1
cg0988707	0.209659	0.608285	7.940774	6.06E-05	0.001832	2.259525	0.503456	0.713114	-0.20966	1	24278297	F	II		IGR	opensea	IGR-opense		rs5599404	25
cg2214661	0.205701	0.617017	7.940176	6.06E-05	0.001833	2.258979	0.514167	0.719868	-0.2057	6	17715219	R	II		IGR	opensea	IGR-opense		rs1220946	39
cg1739479	0.203327	0.64031	7.939899	6.06E-05	0.001833	2.258726	0.538647	0.741974	-0.20333	9	96628794	R	II		IGR	opensea	IGR-opense		rs5634106	30;1

cg0575676	0.200308	0.35796	7.939665	6.07E-05	0.001833	2.258513	0.257806	0.458114	-0.20031	21	39531070	F	II		IGR	opensea	IGR-opensea		rs7511265 47
cg0802855	0.248235	0.548699	7.938433	6.07E-05	0.001834	2.257389	0.424581	0.672816	-0.24824	13	28588650	F	II	FLT3	Body	opensea	Body-opens		
cg1188819	0.24286	0.413706	7.937992	6.07E-05	0.001835	2.256986	0.292275	0.535136	-0.24286	2	1.75E+08	F	II		IGR	shore	IGR-shore	chr2:1748	rs5606579 17
cg0414816	0.218247	0.574299	7.936888	6.08E-05	0.001835	2.255979	0.465176	0.683423	-0.21825	10	65442289	F	II		IGR	opensea	IGR-opensea		rs1817340 0;1
cg1840297	0.215392	0.637939	7.936008	6.08E-05	0.001836	2.255176	0.530243	0.745636	-0.21539	4	37717801	R	II		IGR	opensea	IGR-opensea		rs5563310 2
cg0754633	0.227145	0.482077	7.935942	6.09E-05	0.001836	2.255115	0.368504	0.595649	-0.22714	3	1.53E+08	F	II		IGR	shelf	IGR-shelf	chr3:1525	rs5545593 1;33
cg0842344	0.214911	0.527619	7.935779	6.09E-05	0.001837	2.254968	0.420163	0.635075	-0.21491	20	56621504	F	II		IGR	opensea	IGR-opensea		rs1436762 1;44
cg0546244	0.247587	0.44593	7.935036	6.09E-05	0.001837	2.254289	0.322137	0.569724	-0.24759	16	77574072	F	II		IGR	opensea	IGR-opensea		rs1919234 7
cg1966095	0.22668	0.516689	7.934651	6.09E-05	0.001837	2.253938	0.403349	0.630029	-0.22668	20	12951544	F	II		IGR	opensea	IGR-opensea		
cg0897395	0.216933	0.437497	7.934062	6.1E-05	0.001838	2.2534	0.32903	0.545963	-0.21693	17	64510584	R	II	PRKCA	Body	opensea	Body-opens		
cg0455450	0.20277	0.573299	7.933813	6.1E-05	0.001838	2.253172	0.471914	0.674683	-0.20277	11	1.01E+08	F	II	TRPC6	3'UTR	opensea	3'UTR-open		rs5394479 0;35;40
cg1713183	0.219044	0.5296	7.933773	6.1E-05	0.001838	2.253136	0.420078	0.639122	-0.21904	12	66251661	R	II	HMGA2	Body	opensea	Body-opens		rs5764788 1
cg2703109	0.218355	0.566064	7.929661	6.12E-05	0.001841	2.249382	0.456887	0.675242	-0.21836	8	1.27E+08	R	II		IGR	opensea	IGR-opensea		rs5318907 2
cg0908919	0.201136	0.67621	7.929646	6.12E-05	0.001841	2.249368	0.575642	0.776779	-0.20114	3	28476626	F	II	ZCWPW2	Body	opensea	Body-opens		rs1415961 32;35;47
cg2103946	0.232832	0.565483	7.927881	6.13E-05	0.001843	2.247756	0.449067	0.6819	-0.23283	2	1.44E+08	F	II	KYNU	Body	opensea	Body-opens		
cg2109709	0.231318	0.495296	7.927879	6.13E-05	0.001843	2.247754	0.379638	0.610955	-0.23132	5	1.19E+08	R	II	TNFAIP8	Body	shelf	Body-shelf	chr5:1186	rs1896012 48;46;2
cg0295569	0.251125	0.536131	7.92681	6.13E-05	0.001844	2.246777	0.410568	0.661693	-0.25112	10	33443170	R	II		IGR	opensea	IGR-opensea		rs1181739 10
cg1608276	0.207364	0.638198	7.925675	6.14E-05	0.001845	2.245741	0.534516	0.74188	-0.20736	3	1.94E+08	F	II	LOC64732	Body	opensea	Body-opens		rs7603606 1;8;16
cg0290294	0.208003	0.462591	7.924892	6.14E-05	0.001845	2.245025	0.358589	0.566592	-0.208	20	57617168	F	II	SLMO2	Body	shore	Body-shore	chr20:576	rs1142706 28;49
cg2356486	0.221464	0.48487	7.924201	6.15E-05	0.001846	2.244393	0.374138	0.595602	-0.22146	2	2.28E+08	R	II		IGR	opensea	IGR-opensea		rs5664442 51
cg0714228	0.200293	0.687211	7.924193	6.15E-05	0.001846	2.244386	0.587065	0.787358	-0.20029	9	1.27E+08	F	II	NR6A1	Body	opensea	Body-opens		rs1428066 1;41
cg2678570	0.204366	0.515818	7.923182	6.15E-05	0.001847	2.243462	0.413635	0.618001	-0.20437	11	1.19E+08	F	II	FOXR1	Body	shelf	Body-shelf	chr11:118	rs1477760 32;45
cg2050645	0.2353	0.549439	7.921083	6.16E-05	0.001848	2.241544	0.431789	0.667089	-0.2353	14	21183181	F	II		IGR	opensea	IGR-opensea		rs1175952 1
cg0552636	0.214229	0.610282	7.919507	6.17E-05	0.00185	2.240103	0.503168	0.717397	-0.21423	3	1.29E+08	R	II	IFT122	Body	opensea	Body-opens		rs1130678 6;2;1
cg2345770	0.211987	0.549299	7.917863	6.18E-05	0.001852	2.2386	0.443305	0.655293	-0.21199	8	1.07E+08	R	II	ZFPM2-AS1	Body	opensea	Body-opens		
cg2648973	0.25852	0.604792	7.917576	6.18E-05	0.001852	2.238337	0.475532	0.734052	-0.25852	10	3930603	R	II		IGR	opensea	IGR-opensea		rs5466613 45;34;28
cg2692691	0.206608	0.482011	7.917386	6.18E-05	0.001852	2.238163	0.378707	0.585315	-0.20661	3	55709664	R	II	ERC2	3'UTR	opensea	3'UTR-open		
cg0164152	0.219577	0.529275	7.917238	6.18E-05	0.001852	2.238028	0.419487	0.639064	-0.21958	14	37917543	F	II	MIPOL1	Body	opensea	Body-opens		rs5417813 21;26
cg1994732	0.208693	0.425857	7.916946	6.19E-05	0.001852	2.237761	0.321511	0.530204	-0.20869	12	1.3E+08	R	II	LOC28335	TSS1500	opensea	TSS1500-op		rs1123974 50
cg1998966	0.21041	0.496632	7.916491	6.19E-05	0.001852	2.237345	0.391427	0.601837	-0.21041	7	1.16E+08	R	II		IGR	opensea	IGR-opensea		rs1436637 20;1
cg1939152	0.209263	0.530335	7.916428	6.19E-05	0.001852	2.237287	0.425703	0.634966	-0.20926	8	28173732	F	II	PNOC	TSS1500	opensea	TSS1500-op		rs5511833 0;19
cg2351333	0.236013	0.458731	7.914274	6.2E-05	0.001854	2.235316	0.340724	0.576738	-0.23601	10	24813690	R	II	KIAA1217	ExonBnd	opensea	ExonBnd-op		rs2297327 44;2;1
cg1163676	0.211438	0.546899	7.914263	6.2E-05	0.001854	2.235306	0.44118	0.652618	-0.21144	7	11747365	R	II	THSD7A	Body	opensea	Body-opens		rs7329459 48;47;27;8;2
cg0432782	0.209201	0.611225	7.914124	6.2E-05	0.001854	2.23518	0.506625	0.715826	-0.2092	3	37854791	F	II	ITGA9-AS1	Body	opensea	Body-opens		rs5503852 0;37;40
cg0511897	0.232378	0.643851	7.913995	6.2E-05	0.001854	2.235061	0.527662	0.76004	-0.23238	14	55361901	F	II	GCH1	Body	opensea	Body-opens		rs5297148 1
cg0750443	0.21847	0.433451	7.912044	6.21E-05	0.001856	2.233276	0.324216	0.542686	-0.21847	8	50081281	F	II		IGR	opensea	IGR-opensea		rs5275459 1
cg1972059	0.201107	0.566693	7.911804	6.21E-05	0.001856	2.233057	0.466139	0.667246	-0.20111	10	84649719	F	II	NRG3	Body	opensea	Body-opens		
cg2569891	0.231715	0.469183	7.910483	6.22E-05	0.001857	2.231848	0.353326	0.58504	-0.23171	6	47639679	F	II	ADGRF2	5'UTR	opensea	5'UTR-open		rs5284163 38
cg1611337	0.264005	0.55292	7.910304	6.22E-05	0.001857	2.231684	0.420917	0.684922	-0.264	5	66040195	R	II	MAST4	Body	opensea	Body-opens		rs5369264 2
cg1900330	0.236821	0.424401	7.909669	6.23E-05	0.001858	2.231102	0.305991	0.542812	-0.23682	11	1.15E+08	R	II	CADM1	Body	opensea	Body-opens		rs7357214 21;19;1
cg2429802	0.201354	0.709545	7.907349	6.24E-05	0.00186	2.228979	0.608868	0.810222	-0.20135	6	1.26E+08	R	II		IGR	opensea	IGR-opensea		rs5420932 7
cg0584896	0.226963	0.583198	7.903871	6.26E-05	0.001863	2.225794	0.469716	0.696679	-0.22696	2	1.74E+08	F	II	MLK7-AS1	Body	opensea	Body-opens		rs1397590 0;1;14;15;18;37
cg1807107	0.23033	0.517282	7.903727	6.26E-05	0.001864	2.225662	0.402117	0.632447	-0.23033	16	78930307	R	II	WWOX	Body	opensea	Body-opens		rs5293225 45;44;37;22;17;11;2
cg0808431	0.244543	0.684271	7.902722	6.26E-05	0.001864	2.224741	0.562	0.806543	-0.24454	8	1.27E+08	R	II		IGR	opensea	IGR-opensea		rs5338644 48;11;4
cg0621098	0.216567	0.593594	7.902237	6.27E-05	0.001865	2.224297	0.48531	0.701878	-0.21657	3	57440587	R	II	DNAH12	Body	opensea	Body-opens		
cg2689901	0.208322	0.442257	7.901896	6.27E-05	0.001865	2.223984	0.338096	0.546418	-0.20832	5	86042549	R	II		IGR	opensea	IGR-opensea		rs5764025 37;4
cg1247731	0.222546	0.591735	7.901046	6.27E-05	0.001866	2.223206	0.480462	0.703009	-0.22255	2	9452254	R	II	ASAP2	Body	opensea	Body-opens		rs5288289 43;2
cg1780415	0.206302	0.586958	7.900785	6.27E-05	0.001866	2.222966	0.483808	0.690109	-0.2063	11	1.24E+08	F	II		IGR	opensea	IGR-opensea		
cg0170819	0.201053	0.523448	7.900102	6.28E-05	0.001866	2.22234	0.422921	0.623974	-0.20105	9	71876783	F	II		IGR	opensea	IGR-opensea		rs5525364 0;22
cg1217208	0.211123	0.650319	7.900003	6.28E-05	0.001866	2.22225	0.544757	0.75588	-0.21112	6	1.36E+08	F	II		IGR	opensea	IGR-opensea		
cg2640007	0.221423	0.332685	7.899831	6.28E-05	0.001866	2.222093	0.221973	0.443396	-0.22142	10	96996533	R	II		IGR	opensea	IGR-opensea		
cg0943325	0.226415	0.414401	7.898515	6.29E-05	0.001868	2.220886	0.301193	0.527608	-0.22641	6	1.44E+08	R	II	LOC28574	Body	opensea	Body-opens		rs1846300 2
cg0419585	0.210281	0.669803	7.897821	6.29E-05	0.001868	2.22025	0.564662	0.774944	-0.21028	16	8839233	F	II	ABAT	Body	opensea	Body-opens		
cg1762304	0.224083	0.61402	7.897588	6.29E-05	0.001868	2.220037	0.501979	0.726062	-0.22408	1	1.51E+08	R	II		IGR	opensea	IGR-opensea		
cg1184341	0.203881	0.483915	7.89752	6.29E-05	0.001868	2.219975	0.381974	0.585856	-0.20388	7	1.01E+08	R	II		IGR	opensea	IGR-opensea		rs1148739 36;20;12
cg2250277	0.241056	0.584556	7.896015	6.3E-05	0.00187	2.218595	0.464028	0.705084	-0.24106	14	24540516	R	II	CPNE6	TSS200	opensea	TSS200-ope		
cg2745261	0.217552	0.444074	7.895465	6.3E-05	0.00187	2.218091	0.335298	0.552851	-0.21755	5	1.42E+08	F	II	ARHGAP26	Body	opensea	Body-opens		rs1196015 43
cg0870181	0.238709	0.63898	7.89515	6.3E-05	0.00187	2.217802	0.519626	0.758334	-0.23871	8	95278142	R	II		IGR	shelf	IGR-shelf	chr8:9527	rs1457100 32
cg2574624	0.235168	0.591208	7.894877	6.31E-05	0.00187	2.217552	0.473624	0.708792	-0.23517	7	36072104	R	II		IGR	opensea	IGR-opensea		rs5633585 41
cg0662299	0.207573	0.631443	7.893023	6.32E-05	0.001872	2.215852	0.527656	0.735229	-0.20757	6	34640204	R	II	C6orf106	Body	opensea	Body-opens		
cg0320677	0.205965	0.537174	7.892163	6.32E-05	0.001872	2.215063	0.434191	0.640156	-0.20597	3	1.18E+08	F	II		IGR	opensea	IGR-opensea		rs7295159 0;16
cg0233912	0.204544	0.558812	7.890519	6.33E-05	0.001874	2.213555	0.45654	0.661084	-0.20454	17	54245140	R	II	ANKFN1	Body	opensea	Body-opens		rs1394986 1
cg0740425	0.200695	0.577884	7.88814	6.34E-05	0.001875	2.211373	0.477536	0.678231	-0.2007	4	1.84E+08	R	II	WWC2-AS1	TSS200	opensea	TSS200-ope		
cg2607942	0.221308	0.64603	7.887808	6.35E-05	0.001875	2.211068	0.535376	0.756684	-0.22131	11	1.07E+08	F	II		IGR	opensea	IGR-opensea		rs1508921 0;19
cg1258485	0.209148	0.440252	7.887621	6.35E-05	0.001875	2.210896	0.335678	0.544826	-0.20915	11	1.1E+08	F	II	FDX1	Body	opensea	Body-opens		
cg0082630	0.247587	0.465552	7.8874	6.35E-05	0.001875	2.210693	0.341759	0.589346	-0.24759	18	12998806	R	II	CEP192	5'UTR	opensea	5'UTR-open		rs5427516 47;46;20;1
cg1544904	0.212056	0.657412	7.886723	6.35E-05	0.001876	2.210072	0.551384	0.76344	-0.21206	3	69812125	R	II	MITF	TSS1500	opensea	TSS1500-op		rs1476000 2
cg1850882	0.256851	0.465201	7.885796	6.36E-05	0.001876	2.209222	0.336775	0.593626	-0.25685	4	76440715	F	II	RCHY1	TSS1500	shore	TSS1500-sh	chr4:7643	rs1434152 13;19
cg1806517	0.201798	0.603065	7.885791	6.36E-05	0.001876	2.209217	0.502166	0.703964	-0.2018	15	63116127	F	II	TLN2	Body	opensea	Body-opens		rs1137415 1
cg0850089	0.22736	0.636751	7.885741	6.36E-05	0.001876	2.209171	0.523071	0.750431	-0.22736	2	1.71E+08	F	II	MYO3B	Body	opensea	Body-opens		rs7789980 1
cg2680932	0.23686	0.628965	7.883981	6.37E-05	0.001878	2.207556	0.510535	0.747395	-0.23686	18	23483574	F	II		IGR	opensea	IGR-opensea		rs1919463 0;21;46
cg1701145	0.205628	0.578955	7.883585	6.37E-05	0.001878	2.207192	0.476141	0.681769	-0.20563	1	81537650	R	II		IGR	opensea	IGR-opensea		rs5335451 9;1
cg2474245	0.201671	0.629768	7.883528	6.37E-05	0.001878	2.20714	0.528933	0.730604	-0.20167	11	1.14E+08	R	II	ZBTB16	Body	opensea	Body-opens		rs5715083 38;16;13;11;1
cg0663655	0.203709	0.550366	7.883204	6.37E-05	0.001878	2.206842	0.448511	0.65222	-0.20371	19	14878812	R	II	ADGRE2	Body	opensea	Body-opens		rs1927169 51
cg2687011	0.207901	0.65178	7.883036	6.37E-05	0.001878	2.206688	0.547829	0.755731	-0.2079	3	3687430	R	II		IGR	opensea	IGR-opensea		rs1263707 51
cg0498503	0.227856	0.601482	7.882964	6.37E-05	0.001878	2.206622	0.487554	0.71541	-0.22786	5	1.25E+08	R	II		IGR	opensea	IGR-opensea		rs1916231 34;25;2
cg1775768	0.205667	0.69104	7.88244	6.37E-05	0.001879	2.20614	0.588206	0.793873	-0.20567	18	45506924	R	II		IGR	opensea	IGR-opensea		rs1245483 36
cg0113021	0.220729	0.647311	7.882392	6.38E-05	0.001879	2.206097	0.536947	0.757676	-0.22073	4	74481549	R	II	RASSF6	5'UTR	opensea	5'UTR-open		rs5475403 44;2;1
cg0372673	0.213273	0.584923	7.882043	6.38E-05	0.001879	2.205777	0.478286	0.691559	-0.21327	5	1.68E+08	F	II	SLIT3	Body	opensea	Body-opens		rs1824963 1;19
cg1171816	0.207012	0.444042	7.881326	6.38E-05	0.00188	2.205118	0.340536	0.547548	-0.20701	15	70485495	F	II		IGR	opensea	IGR-opensea		rs7700552 46
cg0621623	0.20783	0.594721	7.879584	6.39E-05	0.001882	2.203518	0.490806	0.698636	-0.20783	10	28272751	R	II	ARMC4	ExonBnd	opensea	ExonBnd-op		rs5459800 49;39;33;24
cg0466162	0.203348	0.654758	7.879269	6.39E-05	0.001882	2.203229	0.553083	0.756432	-0.20335	12	28174838	R	II		IGR	opensea	IGR-opensea		rs5546425 40;23
cg1659491	0.226902	0.637214	7.878746	6.4E-05	0.001882	2.202749	0.523763	0.750664	-0.2269	12	5607455	R	II		IGR	shelf	IGR-shelf	chr12:560	rs5483925 47;1
cg1845547	0.242	0.580625	7.878681	6.4E-05	0.001882	2.202688	0.459625	0.701625	-0.242	17	17311855	R	II		IGR	opensea	IGR-opensea		rs5826278 49;40;14
cg2577236	0.217584	0.612574	7.878646	6.4E-05	0.001882	2.202657	0.503782	0.721366	-0.21758	6	1.41E+08	F	II		IGR	opensea	IGR-opensea		rs5517511 1;28
cg1609903	0.213896	0.646078	7.87844	6.4E-05	0.001883	2.202468	0.53913	0.753026	-0.2139	21	32832415	F	II	TIAM1	5'UTR	opensea	5'UTR-open		rs5561815 44
cg0202605	0.208833	0.56319	7.877859	6.4E-05	0.001883	2.201934	0.458773	0.667606	-0.20883	4	1.56E+08	R	II		IGR	opensea	IGR-opensea		rs1859618 5;2;1
cg1617177	0.232643	0.629724	7.876021	6.41E-05	0.001885	2.200245	0.513402	0.746045	-0.23264	3	34187750	F	II		IGR	opensea	IGR-opensea		rs1425645 33;44
cg1843436	0.213799	0.643619	7.875641	6.41E-05	0.001885	2.199896	0.53672	0.750518	-0.2138	6	45891455	F	I	CLIC5	Body	opensea	Body-opens		rs3737965 48
cg0232918	0.200503	0.446035	7.875638	6.41E-05	0.001885	2.199893	0.345783	0.546286	-0.2005	10	96996226	R	II		IGR	opensea	IGR-opensea		rs5620624 51;26;13
cg0153439	0.208235	0.593968	7.8753	6.41E-05	0.001885	2.199583	0.489851	0.698086	-0.20823	14	23265401	F	II	SLC7A7	Body	opensea	Body-opens		rs1136785 4
cg2236845	0.204039	0.549362	7.875223	6.42E-05	0.001885	2.199512	0.447343	0.651382	-0.20404	16	19528985	R	II	GDE1	Body	shelf	Body-shelf	chr16:195	rs5445670 38;11;2;1
cg0968888	0.202725	0.64993	7.874862	6.42E-05	0.001885	2.19918	0.548567	0.751292	-0.20273	2	1.29E+08	F	II		IGR	opensea	IGR-opensea		
cg0087672	0.218515	0.656659	7.874037	6.42E-05	0.001886	2.198422	0.547402	0.765917	-0.21852	5	10324567	F	II		IGR	opensea	IGR-opensea		rs5765551 0
cg0845007	0.205501	0.586278	7.87359	6.42E-05	0.001886	2.198011	0.483528	0.689029	-0.2055	15	81633740	F	II	TMC3	ExonBnd	opensea	ExonBnd-op		
cg0392093	0.289445	0.492725	7.871832	6.43E-05	0.001887	2.196396	0.348002	0.637447	-0.28945	2	55538851	F	II	CCDC88A	Body	opensea	Body-opens		
cg0921715	0.220654	0.493623	7.871778	6.43E-05	0.001887	2.196346	0.383296	0.60395	-0.22065	10	97624891	F	II	ENTPD1	Body	opensea	Body-opens		rs5341077 5;21
cg2418351	0.219984	0.611632	7.870854	6.44E-05	0.001888	2.195497	0.50164	0.721625	-0.21998	14	51863868	F	II		IGR	opensea	IGR-opensea		
cg2025039	0.215126	0.521506	7.870819	6.44E-05	0.001888	2.195464	0.413943	0.629069	-0.21513	14	92807379	R	II	SLC24A4	Body	opensea	Body-opens		rs1116005 51;45;2
cg2743411	0.219707	0.508092	7.869522	6.45E-05	0.001889	2.194272	0.398239	0.617945	-0.21971	12	66007516	F	II	LOC10050	Body	opensea	Body-opens		rs1477462 2;23
cg0609451	0.210917	0.614772	7.86891	6.45E-05	0.00189	2.193709	0.509313	0.72023	-0.21092	10	1.24E+08	R	II	DMBT1	Body	shelf	Body-shelf	chr10:124	rs1456131 30;16
cg0097523	0.212527	0.536851	7.866808	6.46E-05	0.001892	2.191776	0.430587	0.643115	-0.21253	17	54437076	R	II	ANKFN1	Body	opensea	Body-opens		rs5487527 5
cg1723595	0.205931	0.601336	7.86667	6.46E-05	0.001892	2.191649	0.498371	0.704302	-0.20593	21	36931515	R	II	LOC10050	Body	opensea	Body-opens		rs1844044 46;34;9;1
cg2692017	0.240065	0.508739	7.866152	6.47E-05	0.001892	2.191172	0.388706	0.628772	-0.24007	8	16197182	R	II		IGR	opensea	IGR-opensea		rs5686888 42;38;34;9;1
cg1983940	0.216996	0.544169	7.865542	6.47E-05	0.001893	2.190612	0.435671	0.652667	-0.217	8	1.37E+08	F	II	KHDRBS3	Body	opensea	Body-opens		rs5282668 0;30
cg0957545	0.20498	0.54849	7.865492	6.47E-05	0.001893	2.190565	0.446	0.65098	-0.20498	5	87546981	F	II	TMEM161B	Body	opensea	Body-opens		rs5649259 1;16
cg2169253	0.222529	0.347906	7.865256	6.47E-05	0.001893	2.190349	0.236642	0.459171	-0.22253	3	1.43E+08	R	II		IGR	shelf	IGR-shelf	chr3:1426	rs7386626 35
cg2524971	0.232102	0.516234	7.864852	6.47E-05	0.001893	2.189976	0.400183	0.632285	-0.2321	4	1.77E+08	R	II	WDR17	Body	opensea	Body-opens		rs5697697 37;19;1
cg1254757	0.223557	0.440905	7.864775	6.47E-05	0.001893	2.189906	0.329126	0.552683	-0.22356	10	6130325	R	II	RBM17	TSS1500	shore	TSS1500-sh	chr10:613	
cg0745409	0.217917	0.504626	7.863817	6.48E-05	0.001894	2.189025	0.395667	0.613584	-0.21792	13	52435964	F	II	CCDC70	TSS200	opensea	TSS200-ope		
cg0925107	0.209128	0.639832	7.863607	6.48E-05	0.001894	2.188831	0.535267	0.744396	-0.20913	15	59461599	R	II	MYO1E	Body	opensea	Body-opens		rs5660405 48;35;30
cg0745020	0.2082	0.604948	7.86304	6.48E-05	0.001894	2.188309	0.500848	0.709048	-0.2082	8	12827559	F	II	KIAA1456	5'UTR	opensea	5'UTR-open		rs5431077 25;33
cg0746129	0.203764	0.455156	7.861624	6.49E-05	0.001895	2.187007	0.353274	0.557038	-0.20376	13	1.1E+08	F	II	MYO16	Body	opensea	Body-opens		rs2014831 1;35
cg0712630	0.206382	0.5411	7.861519	6.49E-05	0.001895	2.18691	0.437909	0.644291	-0.20638	3	1.78E+08	R	II	LINC01014	Body	opensea	Body-opens		rs5819265 42;26;25;18;1
cg0196127	0.202751	0.650118	7.860254	6.5E-05	0.001896	2.185746	0.548743	0.751493	-0.20275	1	2.42E+08	F	II	OPN3	Body	opensea	Body-opens		rs3689539 11;32
cg1337049	0.218425	0.579298	7.860188	6.5E-05	0.001896	2.185685	0.470085	0.68851	-0.21842	19	39148693	R	II	ACTN4	Body	opensea	Body-opens		rs5371870 24
cg1355266	0.20424	0.387608	7.860162	6.5E-05	0.001896	2.185661	0.285488	0.489728	-0.20424	5	1.67E+08	R	II	TENM2	Body	opensea	Body-opens		rs1930371 47;46
cg0162757	0.205064	0.657534	7.859067	6.51E-05	0.001897	2.184653	0.555002	0.760066	-0.20506	3	12305467	F	II		IGR	opensea	IGR-opensea		rs5681525 36;40
cg1960470	0.247173	0.479515	7.858914	6.51E-05	0.001897	2.184512	0.355928	0.603102	-0.24717	4	16261642	F	I		IGR	shelf	IGR-shelf	chr4:1625	rs1808403 10;21;34

cg1141778	0.212031	0.556335	7.858178	6.51E-05	0.001898	2.183834	0.45032	0.662351	-0.21203	1	2.42E+08	F	II	OPN3	Body	opensea	Body-opens		rs5448102	1;24
cg1171218	0.208824	0.507057	7.858169	6.51E-05	0.001898	2.183826	0.402645	0.611469	-0.20882	1	10368286	F	II	KIF1B	3'UTR	opensea	3'UTR-oper		rs1507172	0;33
cg1592265	0.200887	0.671315	7.85677	6.52E-05	0.001899	2.182538	0.570871	0.771758	-0.20089	6	1.35E+08	R	II		IGR	opensea	IGR-opense			
cg0679853	0.201023	0.650977	7.85669	6.52E-05	0.001899	2.182464	0.550466	0.751489	-0.20102	11	47961100	F	II		IGR	opensea	IGR-opense			
cg0882507	0.220687	0.528558	7.855993	6.52E-05	0.001899	2.181823	0.418214	0.638901	-0.22069	16	3934709	R	II		IGR	shelf	IGR-shelf	chr16:393(		
cg2423602	0.211828	0.572519	7.855976	6.52E-05	0.001899	2.181807	0.466605	0.678432	-0.21183	6	874435	R	II	LOC10192;	Body	opensea	Body-opens		rs1889871	16;4;1
cg0411438	0.276876	0.594069	7.855257	6.53E-05	0.0019	2.181145	0.455631	0.732507	-0.27688	1	2.29E+08	F	II		IGR	opensea	IGR-opense		rs5284557	1;41
cg1212364	0.203643	0.682783	7.853974	6.54E-05	0.001901	2.179963	0.580961	0.784605	-0.20364	4	1.26E+08	R	II		IGR	opensea	IGR-opense			
cg0162812	0.200979	0.579815	7.852668	6.54E-05	0.001902	2.17876	0.479325	0.680304	-0.20098	16	15647369	R	II	C16orf45	Body	opensea	Body-opens		rs5513465	48;21;1
cg2153607	0.207847	0.533366	7.85192	6.55E-05	0.001903	2.178071	0.429443	0.637289	-0.20785	7	42105481	R	II	GLI3	Body	opensea	Body-opens		rs1445861	2;1
cg1965389	0.200216	0.6123	7.851874	6.55E-05	0.001903	2.178029	0.512192	0.712409	-0.20022	15	55547488	F	II	RAB27A	5'UTR	opensea	5'UTR-oper		rs1844333	0;45
cg2496511	0.214381	0.652148	7.85137	6.55E-05	0.001903	2.177564	0.544958	0.759339	-0.21438	11	1.03E+08	R	II		IGR	opensea	IGR-opense		rs1509119	43;2
cg0356458	0.204981	0.675742	7.850732	6.55E-05	0.001903	2.176977	0.573252	0.778233	-0.20498	10	49693340	R	II	ARHGAP22	Body	opensea	Body-opens		rs5815286	47;26;17;13
cg0038552	0.200283	0.678866	7.850646	6.55E-05	0.001904	2.176898	0.578725	0.779008	-0.20028	13	67209742	F	II	PCDH9	Body	opensea	Body-opens		rs5663277	19
cg2440142	0.203225	0.621142	7.84954	6.56E-05	0.001905	2.175878	0.51953	0.722755	-0.20323	5	77014952	R	II	TBCA	Body	opensea	Body-opens		rs5481307	1
cg0085205	0.216999	0.575622	7.849472	6.56E-05	0.001905	2.175816	0.467123	0.684122	-0.217	1	2.14E+08	F	II		IGR	opensea	IGR-opense		rs5710811	8
cg1043256	0.264889	0.556131	7.849464	6.56E-05	0.001905	2.175809	0.423687	0.688576	-0.26489	17	46709858	F	II	MIR196A1	Body	shore	Body-shore	chr17:467;	rs1864495	1;17;41
cg2399100	0.202182	0.57852	7.849286	6.56E-05	0.001905	2.175645	0.477429	0.679611	-0.20218	9	1.36E+08	R	II	FAM163B	TSS1500	shore	TSS1500-sh	chr9:1364		
cg0129702	0.239326	0.419625	7.849153	6.56E-05	0.001905	2.175522	0.299962	0.539288	-0.23933	13	1.06E+08	R	II	DAOA	TSS1500	opensea	TSS1500-op		rs5646619	38;17;2
cg1789762	0.236318	0.523278	7.845285	6.59E-05	0.001908	2.171957	0.405119	0.641437	-0.23632	17	9292737	F	II	STX8	Body	opensea	Body-opens		rs1446812	0;41;44
cg2084926	0.226369	0.485825	7.845161	6.59E-05	0.001908	2.171842	0.37264	0.599009	-0.22637	13	92376203	R	II	GPC5	Body	opensea	Body-opens		rs5767471	49;35;1
cg0722407	0.246586	0.496871	7.844121	6.59E-05	0.001909	2.170883	0.373578	0.620164	-0.24659	7	1.15E+08	F	II	LINC01392	Body	opensea	Body-opens		rs5672739	31;35;36;46
cg1965784	0.214378	0.55891	7.843945	6.59E-05	0.001909	2.170721	0.451721	0.666099	-0.21438	10	26156371	F	II		IGR	opensea	IGR-opense		rs5572579	0;11;45
cg0275766	0.237292	0.690944	7.843924	6.59E-05	0.001909	2.170702	0.572298	0.80959	-0.23729	21	35930789	F	II	RCAN1	Body	opensea	Body-opens			
cg2479469	0.237951	0.507692	7.843741	6.59E-05	0.001909	2.170533	0.388717	0.626667	-0.23795	3	18278987	R	II	LOC33986;	Body	opensea	Body-opens		rs1162570	18
cg1303385	0.235892	0.580147	7.843647	6.59E-05	0.001909	2.170446	0.462201	0.698094	-0.23589	12	1.09E+08	F	II	SSH1	Body	shelf	Body-shelf	chr12:109;	rs5662329	1;13
cg0022658	0.257474	0.505163	7.842869	6.6E-05	0.00191	2.169729	0.376426	0.6339	-0.25747	5	89000691	F	II		IGR	opensea	IGR-opense		rs5614567	1
cg1438348	0.208097	0.607454	7.842637	6.6E-05	0.00191	2.169515	0.503406	0.711503	-0.2081	2	1.68E+08	R	II	XIRP2	TSS200	opensea	TSS200-ope		rs5336660	30;19;1
cg2280144	0.22134	0.669331	7.840644	6.61E-05	0.001912	2.167676	0.55866	0.780001	-0.22134	10	91752521	F	II		IGR	opensea	IGR-opense		rs7070718	31
cg0975930	0.217709	0.369021	7.840555	6.61E-05	0.001912	2.167594	0.260167	0.477876	-0.21771	12	70573614	R	II		IGR	opensea	IGR-opense		rs5440404	18;8
cg1918907	0.213675	0.575245	7.83952	6.62E-05	0.001913	2.16664	0.468408	0.682083	-0.21368	3	40806781	R	II		IGR	opensea	IGR-opense		rs5645112	32;13;2
cg2432672	0.204504	0.489267	7.839026	6.62E-05	0.001913	2.166184	0.387015	0.591519	-0.2045	4	38310453	R	II		IGR	opensea	IGR-opense		rs5770492	18;9;1
cg0627869	0.214682	0.655422	7.838941	6.62E-05	0.001914	2.166106	0.548081	0.762763	-0.21468	20	48533196	R	II	SPATA2	TSS1500	shore	TSS1500-sh	chr20:485:	rs8115603	10
cg0532989	0.205262	0.665165	7.838265	6.63E-05	0.001914	2.165482	0.562534	0.767796	-0.20526	4	71384359	R	II	AMTN	1stExon	opensea	1stExon-op		rs5514551	29;25;24;2
cg1203379	0.208811	0.682226	7.837868	6.63E-05	0.001915	2.165115	0.57782	0.786631	-0.20881	2	88311157	R	II		IGR	opensea	IGR-opense		rs5777481	2;1
cg1418105	0.208006	0.604266	7.837855	6.63E-05	0.001915	2.165104	0.500262	0.708269	-0.20801	3	1.69E+08	R	II	MECOM	5'UTR	opensea	5'UTR-oper		rs5782158	48;1
cg0253824	0.224546	0.542653	7.835077	6.64E-05	0.001917	2.16254	0.43038	0.654926	-0.22455	16	75045643	R	II	ZNRF1	Body	opensea	Body-opens		rs1954956	20;1
cg1564955	0.214812	0.728687	7.834501	6.65E-05	0.001918	2.162008	0.621281	0.836093	-0.21481	3	40807682	R	II		IGR	opensea	IGR-opense		rs5665336	35;23
cg0275418	0.205262	0.748957	7.833568	6.65E-05	0.001918	2.161147	0.646326	0.851588	-0.20526	5	56992863	F	II		IGR	opensea	IGR-opense		rs1928101	1;9;36
cg1212831	0.221198	0.506216	7.832583	6.66E-05	0.001919	2.160237	0.395617	0.616816	-0.2212	5	1.57E+08	R	II		IGR	opensea	IGR-opense			
cg0916865	0.211833	0.494211	7.830819	6.67E-05	0.001921	2.158609	0.388295	0.600127	-0.21183	6	34070996	F	II	GRM4	Body	opensea	Body-opens		rs5428540	0;1;33;39
cg1954398	0.205106	0.59168	7.82952	6.68E-05	0.001922	2.157409	0.489128	0.694233	-0.20511	12	47313800	R	II		IGR	opensea	IGR-opense		rs5331063	41;1
cg1574704	0.216867	0.530607	7.82817	6.68E-05	0.001922	2.156162	0.422173	0.63904	-0.21687	10	14373423	F	II	FRMD4A	TSS1500	opensea	TSS1500-op		rs1887395	0;39;47
cg1876527	0.205693	0.509063	7.828156	6.68E-05	0.001922	2.156149	0.406216	0.611909	-0.20569	1	71170890	F	II	LOC10192;	TSS1500	opensea	TSS1500-op		rs1837094	12;19;31;34
cg2517378	0.203743	0.390784	7.828091	6.68E-05	0.001922	2.156089	0.288912	0.492656	-0.20374	6	52022616	R	II		IGR	opensea	IGR-opense		rs5697433	9
cg2521490	0.210313	0.589284	7.827656	6.69E-05	0.001923	2.155687	0.484127	0.69444	-0.21031	12	79693302	F	II	SYT1	Body	opensea	Body-opens			
cg0806734	0.269335	0.51506	7.825982	6.7E-05	0.001924	2.154141	0.380392	0.649728	-0.26934	12	1.02E+08	F	II	GNPTAB	Body	opensea	Body-opens		rs3755020	0
cg2607907	0.2416	0.501426	7.825978	6.7E-05	0.001924	2.154137	0.380626	0.622226	-0.2416	18	41361082	F	II		IGR	opensea	IGR-opense		rs5333896	22;51
cg0888890	0.203534	0.462544	7.825951	6.7E-05	0.001924	2.154112	0.360777	0.564311	-0.20353	21	47008098	F	II		IGR	shore	IGR-shore	chr21:470(	rs7391012	0;41
cg2597534	0.235428	0.650473	7.825541	6.7E-05	0.001925	2.153733	0.532759	0.768186	-0.23543	2	2.05E+08	F	II		IGR	opensea	IGR-opense		rs1174893	5
cg0907612	0.203065	0.408337	7.825313	6.7E-05	0.001925	2.153522	0.306804	0.509869	-0.20307	1	1.84E+08	F	II	NCF2	5'UTR	opensea	5'UTR-oper		rs5615569	1;31;32
cg1414432	0.211084	0.54456	7.825039	6.7E-05	0.001925	2.153269	0.439019	0.650102	-0.21108	2	31111880	F	II		IGR	opensea	IGR-opense		rs5318360	1;19;20;37
cg1322835	0.208344	0.523075	7.824738	6.7E-05	0.001926	2.152991	0.418902	0.627247	-0.20834	20	53223230	F	II	DOK5	Body	opensea	Body-opens		rs5711444	0;1;17;22;36
cg1268841	0.210889	0.419943	7.823029	6.71E-05	0.001927	2.151411	0.314498	0.525387	-0.21089	8	1.42E+08	R	II		IGR	opensea	IGR-opense		rs5740271	50;11
cg1236198	0.225551	0.574394	7.822799	6.72E-05	0.001927	2.151199	0.461619	0.687169	-0.22555	2	1.77E+08	R	II		IGR	opensea	IGR-opense		rs1812909	47;34;33
cg0585505	0.228764	0.505041	7.82215	6.72E-05	0.001928	2.150599	0.390659	0.619422	-0.22876	18	57736576	F	II		IGR	opensea	IGR-opense			
cg0369301	0.241603	0.559026	7.822017	6.72E-05	0.001928	2.150476	0.438224	0.679828	-0.2416	10	95715050	F	II		IGR	opensea	IGR-opense		rs3727602	1
cg1500454	0.222904	0.734563	7.821034	6.73E-05	0.001928	2.149567	0.623111	0.846016	-0.2229	2	1.45E+08	R	II	ZEB2	Body	opensea	Body-opens		rs1898991	1
cg0187470	0.235752	0.470027	7.820787	6.73E-05	0.001928	2.149339	0.352151	0.587903	-0.23575	2	1.75E+08	F	II		IGR	opensea	IGR-opense		rs1881149	24
cg2145331	0.221409	0.567554	7.819845	6.73E-05	0.001929	2.148468	0.45685	0.678259	-0.22141	6	80189716	F	II		IGR	opensea	IGR-opense		rs7989702	17;36
cg1278495	0.207827	0.653277	7.818761	6.74E-05	0.00193	2.147466	0.549363	0.75719	-0.20783	13	37454811	R	II	SMAD9	5'UTR	opensea	5'UTR-oper		rs5460662	49
cg0179741	0.223442	0.480928	7.818668	6.74E-05	0.00193	2.14738	0.369207	0.592649	-0.22344	4	1.48E+08	F	II		IGR	opensea	IGR-opense		rs1854144	14;20
cg1081744	0.21026	0.418809	7.817544	6.75E-05	0.001931	2.146341	0.31368	0.523939	-0.21026	6	32823728	R	II	PSMB9	Body	shore	Body-shore	chr6:3282(	rs1496751	2
cg2617000	0.216989	0.588037	7.817141	6.75E-05	0.001931	2.145968	0.479542	0.696532	-0.21699	15	61374778	F	II	RORA	Body	opensea	Body-opens		rs1412585	0;1;15
cg2403765	0.232283	0.679601	7.816768	6.75E-05	0.001932	2.145622	0.563459	0.795743	-0.23228	8	1.43E+08	F	II	TSNARE1	5'UTR	opensea	5'UTR-oper		rs1876016	1;14;25
cg0622451	0.214644	0.582029	7.815418	6.76E-05	0.001932	2.144374	0.474707	0.689351	-0.21464	10	7745454	R	II	ITIH2	1stExon	opensea	1stExon-op		rs1418981	51;45;44;4;2
cg2375226	0.212832	0.603121	7.815214	6.76E-05	0.001932	2.144185	0.496705	0.709537	-0.21283	11	27602844	R	II	BDNF-AS	Body	opensea	Body-opens		rs5346209	42;34;14
cg2257373	0.208639	0.591381	7.81455	6.76E-05	0.001933	2.143571	0.487062	0.695701	-0.20864	2	2.21E+08	F	II		IGR	opensea	IGR-opense		rs1132415	14
cg0537329	0.200547	0.604781	7.814382	6.77E-05	0.001933	2.143415	0.504508	0.705054	-0.20055	1	1.9E+08	F	II		IGR	opensea	IGR-opense		rs7585728	0;14;18
cg0025019	0.21096	0.484954	7.814331	6.77E-05	0.001933	2.143368	0.379474	0.590434	-0.21096	2	16836324	F	II	FAM49A	5'UTR	opensea	5'UTR-oper		rs5611534	32
cg2383661	0.224252	0.672502	7.812945	6.77E-05	0.001934	2.142086	0.560376	0.784628	-0.22425	4	1.69E+08	F	II	ANXA10	Body	opensea	Body-opens		rs5573384	0;1;10;30;32;42
cg1467180	0.203682	0.548722	7.812285	6.78E-05	0.001935	2.141476	0.446881	0.650563	-0.20368	3	55558213	R	II	ERC2	3'UTR	opensea	3'UTR-oper		rs5626643	40;2
cg1786575	0.201564	0.306485	7.811982	6.78E-05	0.001935	2.141195	0.205703	0.407267	-0.20156	3	1.87E+08	R	II		IGR	opensea	IGR-opense		rs5692964	2
cg2394153	0.20134	0.540526	7.810032	6.79E-05	0.001936	2.13939	0.439856	0.641196	-0.20134	13	73676019	R	II		IGR	opensea	IGR-opense		rs5420969	2;1
cg2504615	0.229048	0.690632	7.806336	6.81E-05	0.00194	2.135968	0.576108	0.805156	-0.22905	13	46256900	R	II	LINC01055	Body	opensea	Body-opens		rs1889187	47;42;14
cg1653451	0.201502	0.633804	7.80517	6.82E-05	0.001941	2.134888	0.533053	0.734555	-0.2015	10	1.17E+08	R	II		IGR	opensea	IGR-opense			
cg1282874	0.20492	0.39563	7.804946	6.82E-05	0.001941	2.134681	0.29317	0.49809	-0.20492	11	18600741	F	II	UEVLD	5'UTR	opensea	5'UTR-oper		rs5473990	0;1;14
cg0079007	0.219859	0.616428	7.802579	6.84E-05	0.001943	2.132488	0.506498	0.726357	-0.21986	2	1.03E+08	F	II		IGR	opensea	IGR-opense		rs1401551	0
cg1703401	0.230617	0.536967	7.800713	6.85E-05	0.001945	2.13076	0.421658	0.652275	-0.23062	2	24837421	R	II	NCOA1	5'UTR	opensea	5'UTR-oper		rs5717264	31;1
cg1915250	0.202539	0.608353	7.800137	6.85E-05	0.001945	2.130226	0.507084	0.709623	-0.20254	21	19165818	R	II	C21orf91	3'UTR	opensea	3'UTR-oper		rs1700208	25;2;1
cg1615131	0.204783	0.586651	7.798583	6.86E-05	0.001947	2.128786	0.48426	0.689043	-0.20478	13	47674949	R	II		IGR	opensea	IGR-opense		rs5584942	7;1
cg1029640	0.208267	0.438969	7.798461	6.86E-05	0.001947	2.128673	0.334836	0.543103	-0.20827	4	40182240	R	II		IGR	opensea	IGR-opense		rs1851849	47;46;44;32;10;1
cg1455528	0.223307	0.615368	7.798099	6.86E-05	0.001947	2.128337	0.503714	0.727022	-0.22331	4	1.84E+08	F	II	CLDN24	TSS1500	opensea	TSS1500-op		rs1395932	45
cg0257029	0.213038	0.569944	7.797717	6.87E-05	0.001947	2.127984	0.463425	0.676463	-0.21304	13	53618826	F	II	OLFM4	Body	opensea	Body-opens		rs5302772	43
cg2154802	0.201693	0.710272	7.794696	6.88E-05	0.00195	2.125183	0.609426	0.811119	-0.20169	8	15873055	F	II		IGR	opensea	IGR-opense		rs5474801	4;7;17;20;33
cg2696772	0.204243	0.678572	7.793862	6.89E-05	0.001951	2.124409	0.576451	0.780694	-0.20424	5	1.7E+08	F	II		IGR	opensea	IGR-opense		rs7994594	12;36;37
cg1371440	0.248978	0.448599	7.793581	6.89E-05	0.001951	2.124149	0.32411	0.573088	-0.24898	9	1.35E+08	F	II	RAPGEF1	Body	opensea	Body-opens		rs5474923	1;18
cg0005560	0.283379	0.591175	7.793404	6.89E-05	0.001951	2.123984	0.449485	0.732865	-0.28338	1	48508839	R	II		IGR	opensea	IGR-opense		rs5578441	33
cg1993819	0.219753	0.521081	7.791536	6.9E-05	0.001953	2.122252	0.411204	0.630958	-0.21975	15	63233164	F	II		IGR	opensea	IGR-opense		rs5500725	0;6;37
cg0843791	0.210234	0.58779	7.791164	6.91E-05	0.001953	2.121907	0.482673	0.692907	-0.21023	4	1.41E+08	R	II	MGST2	3'UTR	shelf	3'UTR-shelf	chr4:1406!	rs3689022	15;1
cg0833917	0.204261	0.595313	7.790687	6.91E-05	0.001953	2.121464	0.493182	0.697443	-0.20426	17	63692987	F	II	CCDC46	Body	opensea	Body-opens		rs1434994	1;3;20
cg1692831	0.206807	0.576252	7.790641	6.91E-05	0.001953	2.121422	0.472849	0.679656	-0.20681	2	1.21E+08	F	II	PTPN4	Body	opensea	Body-opens		rs5758058	0;14
cg2592293	0.204104	0.579697	7.789987	6.91E-05	0.001954	2.120815	0.477645	0.68175	-0.2041	1	2.41E+08	F	II	MIR1273E	Body	opensea	Body-opens		rs5758920	50
cg2246210	0.200787	0.625116	7.789234	6.92E-05	0.001955	2.120117	0.524722	0.725509	-0.20079	3	1.39E+08	R	II	NMNAT3	3'UTR	opensea	3'UTR-oper		rs1895897	1
cg1891709	0.270013	0.473769	7.788524	6.92E-05	0.001955	2.119457	0.338763	0.608776	-0.27001	2	2.02E+08	F	II	CFLAR	TSS1500	opensea	TSS1500-op			
cg2570521	0.240196	0.652617	7.788234	6.92E-05	0.001955	2.119188	0.532519	0.772714	-0.2402	5	1.19E+08	R	II	TNFAIP8	TSS1500	shore	TSS1500-sh	chr5:1186!	rs1897500	46
cg1417248	0.203438	0.591457	7.788053	6.92E-05	0.001955	2.11902	0.489738	0.693176	-0.20344	22	33141587	F	II	SYN3	Body	opensea	Body-opens		rs1418157	1;6;22;29
cg1198549	0.217573	0.606944	7.787834	6.93E-05	0.001955	2.118817	0.498157	0.71573	-0.21757	4	86681976	R	II	ARHGAP24	Body	opensea	Body-opens			
cg2666697	0.215091	0.505932	7.78648	6.93E-05	0.001957	2.117561	0.398387	0.613478	-0.21509	21	47038531	F	II		IGR	opensea	IGR-opense		rs1870848	1;15;46
cg2320409	0.201246	0.500455	7.786062	6.94E-05	0.001957	2.117173	0.399832	0.601078	-0.20125	6	1.7E+08	F	II		IGR	opensea	IGR-opense		rs5586488	0;49
cg2222394	0.204223	0.589681	7.785632	6.94E-05	0.001957	2.116774	0.487569	0.691792	-0.20422	4	87279531	R	II	MAPK10	5'UTR	opensea	5'UTR-oper		rs1886773	40;34;10;1
cg1282532	0.21687	0.574956	7.785558	6.94E-05	0.001957	2.116705	0.466521	0.683391	-0.21687	3	1.24E+08	R	II	MYLK	5'UTR	opensea	5'UTR-oper		rs1454333	21;2
cg0938537	0.230182	0.554408	7.784748	6.94E-05	0.001957	2.115954	0.439317	0.669499	-0.23018	6	1.43E+08	R	II	LOC15391(	TSS1500	opensea	TSS1500-op		rs5433593	2
cg2461034	0.230264	0.595109	7.78434	6.95E-05	0.001958	2.115574	0.479977	0.710242	-0.23026	7	98696198	R	II	SMURF1	Body	opensea	Body-opens			
cg1549939	0.20357	0.646817	7.78331	6.95E-05	0.001959	2.114619	0.545032	0.748602	-0.20357	2	7888944	R	II		IGR	opensea	IGR-opense		rs1303303	10
cg1037951	0.207916	0.647387	7.78322	6.95E-05	0.001959	2.114534	0.543429	0.751345	-0.20792	9	1.33E+08	F	II	USP20	Body	shelf	Body-shelf	chr9:1326:	rs1469473	0;15;31;40
cg0134106	0.212248	0.577724	7.781112	6.97E-05	0.001961	2.112578	0.4716	0.683848	-0.21225	8	1.22E+08	R	II		IGR	opensea	IGR-opense		rs7332842	42;38;27
cg1351825	0.221172	0.413805	7.780271	6.97E-05	0.001961	2.111796	0.303219	0.52439	-0.22117	8	6038136	F	II		IGR	opensea	IGR-opense		rs5681147	1;23;31;43
cg0583231	0.212212	0.543633	7.780221	6.97E-05	0.001961	2.11175	0.437527	0.649739	-0.21221	9	18767398	F	II	ADAMTSL1	Body	opensea	Body-opens			
cg0409615	0.207358	0.584703	7.777822	6.99E-05	0.001964	2.109521	0.481024	0.688382	-0.20736	15	40098188	R	II	GPR176	Body	opensea	Body-opens		rs5355506	51
cg0856215	0.220505	0.590871	7.777505	6.99E-05	0.001964	2.109227	0.480619	0.701123	-0.2205	8	18746006	F	II	PSD3	Body	opensea	Body-opens		rs1134737	0;12;43;51
cg2135605	0.20621	0.640048	7.774982	7E-05	0.001966	2.106883	0.536943	0.743153	-0.20621	2	2.02E+08	R	II	CFLAR	TSS1500	opensea	TSS1500-op		rs5429210	33
cg1891425	0.201093	0.596603	7.774795	7.01E-05	0.001966	2.106709	0.496056	0.697149	-0.20109	4	12903886	R	II		IGR	opensea	IGR-opense		rs5505973	2
cg0170205	0.226928	0.512073	7.774512	7.01E-05	0.001967	2.106446	0.39861	0.625537	-0.22693	6	13303065	F	II		IGR	opensea	IGR-opense		rs5776790	1;42;51
cg0327559	0.205569	0.629454	7.773861	7.01E-05	0.001967	2.105842	0.526669	0.732238	-0.20557	7	44675025	R	II	OGDH	Body	opensea	Body-opens		rs5313664	49;36;22
cg0177565	0.215751	0.624146	7.773413	7.01E-05	0.001968	2.105425	0.516271	0.732022	-0.21575	1	2.13E+08	R	II	FLVCR1	Body	opensea	Body-opens		rs1904578	15
cg0825993	0.233489	0.527648	7.771706	7.02E-05	0.001969	2.103838	0.410904	0.644393	-0.23349	17	59659383	R	II		IGR	opensea	IGR-opense		rs1811624	2
cg1108867	0.215169	0.60018	7.770183	7.03E-05	0.00197	2.102422	0.492596	0.707765	-0.21517	4	1.43E+08	R	II		IGR	opensea	IGR-opense		rs1932467	1
cg1404179	0.214129	0.674596	7.7694	7.04E-05	0.001971	2.101694	0.567531	0.78166	-0.21413	13	51754437	F	II		IGR	opensea	IGR-opense		rs5763797	0;42;44

cg0971180	0.203911	0.612583	7.769245	7.04E-05	0.001971	2.10155	0.510628	0.714538	-0.20391	10	61019750	R	II	FAM13C	Body	opensea	Body-opens		rs5762444	51;14
cg2606214	0.202187	0.683226	7.767986	7.05E-05	0.001972	2.100379	0.582133	0.78432	-0.20219	1	66561475	F	II	PDE4B	Body	opensea	Body-opens		rs1398066	9;47;48
cg0309263	0.20623	0.463464	7.766706	7.06E-05	0.001973	2.099189	0.360349	0.566579	-0.20623	10	33406660	F	II		IGR	opensea	IGR-opense		rs1485062	0;8;39
cg0366507	0.203166	0.465655	7.766149	7.06E-05	0.001974	2.09867	0.364072	0.567238	-0.20317	5	1.19E+08	F	II	TNFAIP8	Body	shore	Body-shore	chr5:1186	rs5655353	26
cg0565685	0.24408	0.570299	7.765903	7.06E-05	0.001974	2.098442	0.448259	0.692339	-0.24408	7	55584276	F	II	VOPP1	Body	opensea	Body-opens		rs5381132	1;43
cg0725253	0.218915	0.675013	7.765371	7.06E-05	0.001975	2.097947	0.565556	0.784471	-0.21891	12	59845805	R	II		IGR	opensea	IGR-opense			
cg2678283	0.205307	0.72679	7.764028	7.07E-05	0.001976	2.096697	0.624136	0.829443	-0.20531	2	1.29E+08	F	II	AMMECR1L	5'UTR	shore	5'UTR-shor	chr2:1286	rs5464834	13;47
cg2093193	0.215274	0.654089	7.763735	7.07E-05	0.001976	2.096425	0.546453	0.761726	-0.21527	2	1.36E+08	R	II	R3HDM1	Body	opensea	Body-opens			
cg1525495	0.200025	0.573397	7.763622	7.07E-05	0.001976	2.09632	0.473385	0.67341	-0.20003	19	8420745	F	II		IGR	opensea	IGR-opense		rs5651605	3;23
cg0858682	0.235019	0.609728	7.762917	7.08E-05	0.001977	2.095664	0.492219	0.727238	-0.23502	10	92762470	R	II		IGR	opensea	IGR-opense		rs1508977	34
cg0145556	0.204253	0.60558	7.762115	7.08E-05	0.001978	2.094917	0.503453	0.707706	-0.20425	12	46883863	F	II	LOC100288	Body	opensea	Body-opens		rs5470793	0;10
cg0817952	0.203446	0.633647	7.762033	7.08E-05	0.001978	2.094841	0.531923	0.73537	-0.20345	3	1.93E+08	R	II		IGR	opensea	IGR-opense			
cg2253531	0.222664	0.544082	7.761834	7.09E-05	0.001978	2.094656	0.43275	0.655414	-0.22266	1	90865905	F	II		IGR	opensea	IGR-opense		rs1158817	1;15;36;37
cg1389533	0.21573	0.619086	7.761478	7.09E-05	0.001978	2.094325	0.511221	0.726951	-0.21573	9	6418884	R	II	UHRF2	Body	opensea	Body-opens		rs1868079	47
cg1638611	0.212507	0.75073	7.759392	7.1E-05	0.00198	2.092383	0.644476	0.856983	-0.21251	3	1.43E+08	F	II		IGR	opensea	IGR-opense		rs1151775	1;37;51
cg0930447	0.270107	0.575996	7.759047	7.1E-05	0.00198	2.092061	0.440943	0.711049	-0.27011	18	76916901	R	II	ATP9B	Body	opensea	Body-opens		rs1145147	1
cg2352505	0.221635	0.555088	7.759035	7.1E-05	0.00198	2.09205	0.444271	0.665906	-0.22164	15	38852680	F	II	RASGRP1	Body	shelf	Body-shelf	chr15:3885	rs1481250	1
cg0728368	0.200338	0.639403	7.757933	7.11E-05	0.001981	2.091024	0.539234	0.739572	-0.20034	15	48177477	R	II		IGR	opensea	IGR-opense		rs1461625	1
cg1744534	0.225887	0.584011	7.757654	7.11E-05	0.001982	2.090765	0.471068	0.696954	-0.22589	4	1.64E+08	R	II	TKTL2	TSS1500	shore	TSS1500-sh	chr4:1643	rs5646972	37
cg0902208	0.201486	0.34976	7.757435	7.11E-05	0.001982	2.09056	0.249017	0.450503	-0.20149	1	2.36E+08	R	II		IGR	opensea	IGR-opense		rs1683330	30;8
cg0761917	0.220452	0.694913	7.755128	7.13E-05	0.001983	2.088412	0.584687	0.805139	-0.22045	2	1.92E+08	R	II	STAT4	Body	opensea	Body-opens		rs5381830	17;2
cg2333021	0.224066	0.535243	7.754322	7.13E-05	0.001984	2.087662	0.42321	0.647276	-0.22407	11	27672697	F	II	BDNFOS	Body	opensea	Body-opens		rs5751267	0;27
cg1258012	0.226318	0.580911	7.75301	7.14E-05	0.001985	2.086439	0.467752	0.69407	-0.22632	4	1.53E+08	F	II		IGR	opensea	IGR-opense		rs5774945	1;35
cg0705384	0.233817	0.495778	7.752458	7.14E-05	0.001986	2.085926	0.378869	0.612687	-0.23382	6	29012588	F	II	OR2W1	1stExon	opensea	1stExon-op			
cg0010036	0.230833	0.460289	7.749974	7.16E-05	0.001988	2.083611	0.344872	0.575705	-0.23083	12	1.11E+08	F	II	ATP2A2	Body	opensea	Body-opens		rs5707659	1
cg0849474	0.249209	0.580354	7.749597	7.16E-05	0.001988	2.08326	0.455749	0.704958	-0.24921	11	1.11E+08	R	II		IGR	opensea	IGR-opense		rs5740891	31;30
cg1458277	0.210525	0.616274	7.749495	7.16E-05	0.001988	2.083165	0.511011	0.721536	-0.21053	1	2.29E+08	F	II		IGR	opensea	IGR-opense		rs5337668	0;18;23;27
cg0255615	0.216913	0.511047	7.749244	7.17E-05	0.001988	2.08293	0.402591	0.619504	-0.21691	3	1.14E+08	R	II		IGR	opensea	IGR-opense		rs3741836	7
cg0648449	0.217843	0.441189	7.748532	7.17E-05	0.001989	2.082267	0.332267	0.55011	-0.21784	2	1.91E+08	F	II	HIBCH	TSS1500	shore	TSS1500-sh	chr2:1911		
cg0175158	0.23066	0.375853	7.747947	7.17E-05	0.001989	2.081722	0.260523	0.491183	-0.23066	9	1E+08	R	II	LOC28635	TSS1500	opensea	TSS1500-op		rs1098193	17;2
cg2679042	0.216104	0.633994	7.74693	7.18E-05	0.00199	2.080773	0.525942	0.742046	-0.2161	2	97363955	F	II	FER1L5	Body	opensea	Body-opens		rs1153958	0
cg2037079	0.207776	0.384666	7.74687	7.18E-05	0.00199	2.080718	0.280778	0.488554	-0.20778	9	79508831	F	II	PRUNE2	Body	opensea	Body-opens		rs3755230	0;1
cg0862801	0.23855	0.59782	7.745441	7.19E-05	0.001991	2.079385	0.478545	0.717095	-0.23855	5	1.76E+08	R	II		IGR	opensea	IGR-opense			
cg0955124	0.209301	0.574134	7.744682	7.19E-05	0.001992	2.078678	0.469483	0.678784	-0.2093	17	2796053	F	II	RAP1GAP2	Body	opensea	Body-opens		rs5751364	0;1;37
cg0153943	0.220245	0.629381	7.744642	7.19E-05	0.001992	2.07864	0.519259	0.739504	-0.22024	21	40176506	F	II	ETS2	TSS1500	shore	TSS1500-sh	chr21:401	rs5708481	0;6;16;45
cg0163207	0.208279	0.589209	7.744115	7.2E-05	0.001992	2.078149	0.48507	0.693349	-0.20828	9	28264289	F	II	LINGO2	5'UTR	opensea	5'UTR-oper		rs5710038	1;21;29
cg1224535	0.220608	0.552099	7.743894	7.2E-05	0.001992	2.077942	0.441796	0.662403	-0.22061	4	84468038	R	II	AGPAT9	Body	opensea	Body-opens		rs5497568	2
cg2532394	0.204538	0.722212	7.742944	7.21E-05	0.001993	2.077057	0.619943	0.824481	-0.20454	10	13737519	R	II	FRMD4A	Body	opensea	Body-opens		rs7359222	27;25;6
cg0486743	0.205826	0.632174	7.741735	7.21E-05	0.001994	2.07593	0.529261	0.735087	-0.20583	5	1.36E+08	F	II	SPOCK1	Body	opensea	Body-opens		rs3689442	0;16;19;23;47
cg0992364	0.215977	0.533049	7.741327	7.22E-05	0.001995	2.075549	0.42506	0.641037	-0.21598	10	722301	R	II	DIP2C	Body	opensea	Body-opens			
cg2277130	0.215123	0.479419	7.740917	7.22E-05	0.001995	2.075166	0.371858	0.586981	-0.21512	3	1.08E+08	R	II	HHLA2	1stExon	opensea	1stExon-op			
cg1152540	0.20321	0.441303	7.740376	7.22E-05	0.001996	2.074661	0.339698	0.542908	-0.20321	1	88054230	F	II		IGR	opensea	IGR-opense		rs1141340	42
cg0495034	0.205149	0.656726	7.739137	7.23E-05	0.001997	2.073505	0.554151	0.759301	-0.20515	3	30544522	F	II		IGR	opensea	IGR-opense		rs5402547	0
cg2390014	0.265662	0.566083	7.738934	7.23E-05	0.001997	2.073316	0.433252	0.698913	-0.26566	9	22142053	R	II		IGR	opensea	IGR-opense		rs5579372	1
cg1964475	0.200546	0.438406	7.7388	7.23E-05	0.001997	2.073191	0.338133	0.538679	-0.20055	3	1.72E+08	R	II	PLD1	5'UTR	opensea	5'UTR-oper		rs5603784	42
cg0894389	0.250236	0.535527	7.73784	7.24E-05	0.001998	2.072295	0.410409	0.660646	-0.25024	14	50146187	F	II	POLE2	Body	opensea	Body-opens		rs1411196	1
cg0290974	0.202255	0.695388	7.737683	7.24E-05	0.001998	2.072149	0.59426	0.796516	-0.20226	9	1.38E+08	R	II	LINC01502	Body	opensea	Body-opens		rs7678683	32
cg1579859	0.231248	0.535118	7.736866	7.24E-05	0.001999	2.071386	0.419494	0.650742	-0.23125	1	2.44E+08	F	II	AKT3	Body	opensea	Body-opens			
cg1589305	0.204013	0.649079	7.736527	7.25E-05	0.001999	2.07107	0.547073	0.751085	-0.20401	4	1.41E+08	F	II	SCOC	5'UTR	opensea	5'UTR-oper			
cg0061235	0.211052	0.684878	7.735392	7.25E-05	0.002	2.070011	0.579352	0.790405	-0.21105	1	85763605	F	II		IGR	opensea	IGR-opense		rs1843787	0;12;26
cg0646396	0.206401	0.648506	7.735198	7.25E-05	0.002	2.069829	0.545305	0.751706	-0.2064	9	1.17E+08	F	II	ZNF618	Body	opensea	Body-opens		rs5516485	17
cg2252170	0.220985	0.501899	7.735152	7.25E-05	0.002	2.069786	0.391407	0.612392	-0.22099	6	1.44E+08	R	II		IGR	opensea	IGR-opense			
cg0016783	0.214611	0.670277	7.734454	7.26E-05	0.002	2.069135	0.562972	0.777583	-0.21461	11	1.12E+08	F	II	C11orf34	TSS200	opensea	TSS200-ope		rs1491925	1;15;23;49
cg1777768	0.247568	0.429556	7.734253	7.26E-05	0.002	2.068948	0.305772	0.55334	-0.24757	2	2.02E+08	F	II	CFLAR	5'UTR	shelf	5'UTR-shelf	chr2:2019		
cg0728871	0.201411	0.73205	7.734158	7.26E-05	0.002	2.068859	0.631638	0.832756	-0.20141	12	64324997	F	II	SRGAP1	Body	opensea	Body-opens		rs5431679	8
cg1154971	0.280052	0.522228	7.734081	7.26E-05	0.002	2.068786	0.382202	0.662254	-0.28005	2	55001373	F	II	EML6	Body	opensea	Body-opens		rs5554022	0;32
cg2323984	0.252175	0.589101	7.733953	7.26E-05	0.002	2.068668	0.463013	0.715189	-0.25218	17	29412769	R	II		IGR	opensea	IGR-opense		rs5478881	36
cg0136493	0.257963	0.475529	7.733093	7.27E-05	0.002001	2.067864	0.346548	0.60451	-0.25796	16	73185687	R	II		IGR	opensea	IGR-opense		rs5382053	39;35;1
cg2470852	0.231049	0.568792	7.731979	7.28E-05	0.002002	2.066824	0.453268	0.684317	-0.23105	8	1.42E+08	R	II	PTK2	Body	opensea	Body-opens		rs1150455	39;5;1
cg2057634	0.213186	0.56845	7.731636	7.28E-05	0.002002	2.066504	0.461856	0.675043	-0.21319	8	1.07E+08	R	II	ZFPM2	Body	opensea	Body-opens		rs7656231	19
cg0816400	0.209592	0.635768	7.7308	7.28E-05	0.002003	2.065723	0.530972	0.740564	-0.20959	21	27544052	R	II	APP	TSS1500	shore	TSS1500-sh	chr21:275	rs364048	13
cg2299937	0.215932	0.585967	7.730022	7.29E-05	0.002004	2.064996	0.478001	0.693933	-0.21593	15	54774263	R	II	UNC13C	Body	opensea	Body-opens		rs5512720	32;12;2
cg1980658	0.207965	0.469978	7.728689	7.3E-05	0.002004	2.063751	0.365996	0.573961	-0.20797	15	39524983	R	II		IGR	opensea	IGR-opense		rs5642858	35
cg2240581	0.215542	0.732097	7.728151	7.3E-05	0.002005	2.063249	0.624326	0.839868	-0.21554	9	1.18E+08	R	II	DEC1	5'UTR	opensea	5'UTR-oper		rs5511469	18
cg2354861	0.236411	0.548869	7.727666	7.3E-05	0.002005	2.062796	0.430664	0.667075	-0.23641	7	1.48E+08	R	II	CNTNAP2	Body	opensea	Body-opens		rs5618143	30
cg0840422	0.231654	0.659902	7.72745	7.3E-05	0.002005	2.062594	0.544074	0.775729	-0.23165	3	3151899	R	II	IL5RA	5'UTR	opensea	5'UTR-oper			
cg1429741	0.244008	0.474363	7.727307	7.31E-05	0.002005	2.06246	0.352359	0.596367	-0.24401	2	63351486	F	II	WDPCP	Body	opensea	Body-opens		rs1482247	0;3;37;41
cg0436148	0.223786	0.522405	7.726944	7.31E-05	0.002006	2.062122	0.410512	0.634299	-0.22379	8	1.28E+08	F	II	CASC8	Body	opensea	Body-opens		rs5284540	46
cg0523991	0.203925	0.578145	7.724914	7.32E-05	0.002008	2.060224	0.476183	0.680107	-0.20392	5	1.09E+08	R	II		IGR	opensea	IGR-opense		rs1921791	29;45
cg0026832	0.214194	0.474168	7.724596	7.32E-05	0.002008	2.059927	0.367071	0.581265	-0.21419	3	1.26E+08	R	II	UROC1	TSS1500	opensea	TSS1500-op		rs5375263	48;41;23;1
cg1846902	0.212237	0.642166	7.722895	7.33E-05	0.002009	2.058337	0.536047	0.748285	-0.21224	7	11578661	R	II	THSD7A	Body	opensea	Body-opens		rs1826286	11;2;1
cg2183371	0.237922	0.574326	7.720514	7.35E-05	0.002012	2.056111	0.455365	0.693287	-0.23792	12	68222105	F	II		IGR	opensea	IGR-opense		rs1907381	1;3;36;47
cg0639986	0.237001	0.702694	7.719376	7.36E-05	0.002013	2.055047	0.584194	0.821194	-0.237	5	1.22E+08	R	II	LOC10192	Body	opensea	Body-opens		rs5750374	27;20
cg1532433	0.208887	0.627014	7.718776	7.36E-05	0.002014	2.054487	0.52257	0.731457	-0.20889	10	30085133	F	II		IGR	opensea	IGR-opense		rs1858761	0;1;40
cg1972260	0.215542	0.497825	7.714275	7.39E-05	0.002019	2.050276	0.390054	0.605596	-0.21554	1	17578380	R	II	PADI3	Body	opensea	Body-opens		rs1854752	24;17;1
cg2007363	0.224214	0.584585	7.713355	7.4E-05	0.00202	2.049415	0.472478	0.696692	-0.22421	17	53799323	R	II	TMEM100	5'UTR	opensea	5'UTR-oper			
cg0634612	0.265935	0.46793	7.712935	7.4E-05	0.00202	2.049022	0.334963	0.600897	-0.26593	19	50804360	F	II	MYH14	Body	opensea	Body-opens		rs5651906	0;38;39;46
cg1981199	0.21448	0.678747	7.712781	7.4E-05	0.00202	2.048878	0.571507	0.785987	-0.21448	3	50373641	R	II	RASSF1	Body	shore	Body-shore	chr3:5037	rs1462615	14
cg0307432	0.20025	0.680098	7.71235	7.4E-05	0.002021	2.048475	0.579973	0.780223	-0.20025	14	52163526	F	II	FRMD6	Body	opensea	Body-opens		rs1901693	1;37;46
cg0731392	0.228753	0.771522	7.711191	7.41E-05	0.002022	2.04739	0.657145	0.885898	-0.22875	2	2.16E+08	R	II	ABCA12	Body	opensea	Body-opens		rs5740235	36;18;17;2;1
cg0046573	0.208159	0.56993	7.710647	7.41E-05	0.002022	2.04688	0.46585	0.674009	-0.20816	13	25993555	F	II	ATP8A2	Body	opensea	Body-opens		rs5719736	1
cg2210654	0.22264	0.554107	7.710194	7.42E-05	0.002022	2.046457	0.442787	0.665427	-0.22264	6	74049525	R	II		IGR	opensea	IGR-opense		rs7294793	36
cg1659631	0.202889	0.594908	7.710123	7.42E-05	0.002022	2.04639	0.493463	0.696352	-0.20289	1	85755348	F	II		IGR	opensea	IGR-opense		rs1469183	0
cg1850274	0.209761	0.377055	7.709184	7.42E-05	0.002023	2.045511	0.272174	0.481935	-0.20976	3	46335416	R	II		IGR	opensea	IGR-opense		rs5446545	51;44;2;1
cg0798589	0.214355	0.69606	7.708009	7.43E-05	0.002025	2.044411	0.588882	0.803238	-0.21436	18	55758855	R	II	NEDD4L	Body	opensea	Body-opens		rs1856695	39
cg1397833	0.206751	0.503415	7.707882	7.43E-05	0.002025	2.044293	0.400039	0.60679	-0.20675	6	11825273	R	II		IGR	opensea	IGR-opense		rs1488279	28;1
cg2384581	0.226933	0.525634	7.707381	7.44E-05	0.002025	2.043823	0.412167	0.6391	-0.22693	3	1.55E+08	F	II		IGR	opensea	IGR-opense			
cg1579558	0.242922	0.421553	7.70665	7.44E-05	0.002026	2.043138	0.300092	0.543014	-0.24292	2	2.43E+08	F	II	DTYMK	TSS1500	shore	TSS1500-sh	chr2:2426	rs1486185	38;49
cg0441450	0.218656	0.623528	7.705975	7.44E-05	0.002026	2.042506	0.5142	0.732856	-0.21866	3	1.69E+08	F	II	MECOM	Body	opensea	Body-opens		rs1839573	1;29;46
cg0577070	0.201759	0.665291	7.705646	7.45E-05	0.002026	2.042198	0.564412	0.766171	-0.20176	1	2.47E+08	F	II	CNST	Body	opensea	Body-opens		rs5517429	7
cg0182970	0.253352	0.564594	7.705076	7.45E-05	0.002027	2.041665	0.437918	0.69127	-0.25335	22	32047972	R	II		IGR	opensea	IGR-opense		rs6223917	33;4
cg1549303	0.210294	0.591626	7.702848	7.47E-05	0.002029	2.039578	0.486479	0.696773	-0.21029	10	1.17E+08	R	II	FAM160B1	3'UTR	opensea	3'UTR-oper		rs5504773	32;31;22;7;3
cg1291317	0.206509	0.558774	7.702492	7.47E-05	0.002029	2.039244	0.455519	0.662028	-0.20651	7	69871719	R	II	AUTS2	Body	opensea	Body-opens		rs6967417	37;27;23;22;21
cg2522481	0.200403	0.553024	7.701864	7.47E-05	0.002029	2.038655	0.452822	0.653225	-0.2004	4	1.58E+08	F	II	PDGFC	Body	opensea	Body-opens		rs1405190	48
cg0807898	0.222323	0.517797	7.701283	7.48E-05	0.00203	2.038111	0.406635	0.628959	-0.22232	16	64663668	F	II		IGR	opensea	IGR-opense		rs1891686	0
cg2370015	0.228594	0.497631	7.700845	7.48E-05	0.00203	2.0377	0.383334	0.611928	-0.22859	3	1.31E+08	R	II	CPNE4	Body	opensea	Body-opens		rs5481271	19;7;1
cg0740672	0.215118	0.673794	7.697523	7.5E-05	0.002033	2.034587	0.566236	0.781353	-0.21512	5	1.15E+08	R	II		IGR	opensea	IGR-opense		rs5424351	1
cg1354910	0.239855	0.474901	7.69566	7.51E-05	0.002035	2.032841	0.354974	0.594829	-0.23985	1	67632012	R	II	IL23R	TSS200	opensea	TSS200-ope		rs1146575	34;8;2
cg1742212	0.209629	0.661211	7.694753	7.52E-05	0.002036	2.03199	0.556396	0.766026	-0.20963	12	2103365	R	II	DCP1B	Body	opensea	Body-opens		rs5565751	41;1
cg0508694	0.205035	0.603039	7.694684	7.52E-05	0.002036	2.031926	0.500522	0.705557	-0.20504	4	43149680	F	II		IGR	opensea	IGR-opense		rs4615203	27
cg2502736	0.201052	0.560901	7.694609	7.52E-05	0.002036	2.031855	0.460375	0.661427	-0.20105	17	63100218	R	II		IGR	shelf	IGR-shelf	chr17:630		
cg0130600	0.243012	0.60985	7.694327	7.52E-05	0.002036	2.03159	0.488344	0.731356	-0.24301	2	24802612	F	II		IGR	opensea	IGR-opense		rs5566489	0;17
cg2484473	0.242016	0.638144	7.690933	7.54E-05	0.002039	2.028407	0.517136	0.759152	-0.24202	21	47075042	F	II		IGR	opensea	IGR-opense		rs1918137	0;46
cg1928695	0.207415	0.667857	7.690174	7.55E-05	0.00204	2.027695	0.56415	0.771565	-0.20742	4	81917939	R	II		IGR	opensea	IGR-opense		rs1122795	4
cg2160895	0.235319	0.522603	7.689917	7.55E-05	0.00204	2.027454	0.404944	0.640263	-0.23532	21	16273878	R	II		IGR	opensea	IGR-opense		rs5433571	1
cg2329127	0.230887	0.602336	7.688837	7.56E-05	0.002041	2.026441	0.486893	0.717779	-0.23089	12	15866615	F	II	EPS8	5'UTR	opensea	5'UTR-oper		rs5346277	50
cg0204312	0.215994	0.54743	7.688164	7.56E-05	0.002042	2.025809	0.439433	0.655427	-0.21599	10	72299368	F	II	PALD1	Body	opensea	Body-opens		rs1440933	7;11;30;42
cg1735754	0.210056	0.568267	7.687556	7.57E-05	0.002042	2.025238	0.463239	0.673295	-0.21006	15	50429829	F	II		IGR	opensea	IGR-opense			
cg1457153	0.207517	0.491702	7.687395	7.57E-05	0.002043	2.025087	0.387943	0.59546	-0.20752	5	1.29E+08	F	II		IGR	opensea	IGR-opense		rs5593341	23;41
cg0120460	0.227697	0.459458	7.687229	7.57E-05	0.002043	2.024931	0.345609	0.573306	-0.2277	16	80619852	R	II		IGR	opensea	IGR-opense		rs5339601	27;21;14
cg1141732	0.218623	0.351095	7.686411	7.58E-05	0.002044	2.024164	0.241784	0.460407	-0.21862	1	41160271	F	II	NFYC	5'UTR	shelf	5'UTR-shelf	chr1:4115	rs1490175	1;10;26;32
cg0407068	0.22007	0.449935	7.685805	7.58E-05	0.002044	2.023595	0.3399	0.55997	-0.22007	10	75534560	F	II	FUT11	Body	shore	Body-shore	chr10:755		
cg1724572	0.213024	0.419293	7.685152	7.58E-05	0.002045	2.022982	0.312781	0.525805	-0.21302	1	1.81E+08	F	II		IGR	opensea	IGR-opense		rs1146795	0
cg1733049	0.257009	0.566935	7.684394	7.59E-05	0.002046	2.02227	0.438431	0.69544	-0.25701	13	67550124	F	II	PCDH9-AS3	TSS1500	opensea	TSS1500-op		rs5415131	32;46
cg0084184	0.223398	0.455157	7.68433	7.59E-05	0.002046	2.02221	0.343459	0.566856	-0.2234	2	8683604	R	II		IGR	opensea	IGR-opense		rs5480219	47
cg2466112	0.216532	0.634861	7.682392	7.6E-05	0.002048	2.020391	0.526595	0.743127	-0.21653	2	46295385	F	II	PRKCE	Body	opensea	Body-opens		rs5357549	0
cg1712397	0.217918	0.53582	7.681836	7.61E-05	0.002048	2.019868	0.426861	0.644778	-0.21792	8	38635420	F	II	TACC1	5'UTR	opensea	5'UTR-oper		rs1473601	22
cg1363601	0.218545	0.497439	7.68153	7.61E-05	0.002049	2.019581	0.388167	0.606712	-0.21855	9	84004731	F	II		IGR	opensea	IGR-opense		rs1113919	31;36;46;50
cg0853511	0.216377	0.543269	7.678887	7.63E-05	0.002051	2.017099	0.43508	0.651457	-0.21638	10	61418311	R	II	SLC16A9	Body	opensea	Body-opens		rs5726696	22;7;2

cg1868999	0.224569	0.540618	7.678472	7.63E-05	0.002051	2.016708	0.428333	0.652902	-0.22457	5	55164885	R	II	IL31RA	5'UTR	opensea	5'UTR-oper		rs5300594	23;22;5;1
cg0192820	0.225486	0.526578	7.677503	7.64E-05	0.002052	2.015798	0.413836	0.639321	-0.22549	2	1.78E+08	R	II		IGR	opensea	IGR-opense		rs5541598	19;4
cg1975726	0.200704	0.581678	7.674567	7.66E-05	0.002054	2.013039	0.481326	0.68203	-0.2007	10	44222463	F	II		IGR	shore	IGR-shore	chr10:442	rs5732988	22;23
cg2128108	0.277162	0.527943	7.673513	7.66E-05	0.002055	2.012049	0.389362	0.666525	-0.27716	5	55067187	R	II	DDX4	Body	opensea	Body-opens		rs5311283	28
cg1637462	0.214664	0.399516	7.673143	7.67E-05	0.002056	2.011701	0.292184	0.506848	-0.21466	4	90143489	R	II		IGR	opensea	IGR-opense		rs1701519	46;45;16
cg0158503	0.227318	0.530909	7.673055	7.67E-05	0.002056	2.011619	0.41725	0.644568	-0.22732	21	39811743	R	II	ERG	Body	opensea	Body-opens			
cg1974210	0.211292	0.63794	7.672079	7.67E-05	0.002057	2.010701	0.532294	0.743586	-0.21129	5	88426429	F	II		IGR	opensea	IGR-opense		rs7318108	0;3
cg1274217	0.210219	0.577926	7.668148	7.7E-05	0.00206	2.007005	0.472817	0.683036	-0.21022	12	1.03E+08	R	II	IGF1	Body	opensea	Body-opens		rs5401299	23;1
cg1891422	0.223878	0.587798	7.667296	7.71E-05	0.002061	2.006203	0.475859	0.699737	-0.22388	9	1.3E+08	R	II	RALGPS1	Body	opensea	Body-opens		rs2417044	15
cg0084963	0.212725	0.47373	7.667157	7.71E-05	0.002061	2.006072	0.367367	0.580092	-0.21273	15	50399974	F	II	ATP8B4	5'UTR	opensea	5'UTR-oper			
cg0981588	0.227864	0.60254	7.667081	7.71E-05	0.002061	2.006001	0.488608	0.716472	-0.22786	12	93478385	R	II	LOC64333	Body	opensea	Body-opens		rs1428527	38;28;7;2;1
cg0164875	0.2152	0.597327	7.667046	7.71E-05	0.002061	2.005968	0.489727	0.704927	-0.2152	5	38931978	F	II	OSMR	ExonBnd	opensea	ExonBnd-op		rs1483977	47;48
cg0915245	0.216638	0.490175	7.666981	7.71E-05	0.002061	2.005906	0.381856	0.598494	-0.21664	5	31742848	F	II		IGR	opensea	IGR-opense		rs1815480	44
cg1692232	0.221327	0.552669	7.665827	7.72E-05	0.002062	2.004821	0.442005	0.663333	-0.22133	2	1.7E+08	F	II	NOSTRIN	5'UTR	opensea	5'UTR-oper		rs5287805	26;51
cg2667585	0.204189	0.524271	7.662486	7.74E-05	0.002065	2.001678	0.422176	0.626366	-0.20419	9	1.01E+08	F	II		IGR	opensea	IGR-opense		rs5584921	0;12
cg0425581	0.212845	0.695031	7.661935	7.74E-05	0.002065	2.001158	0.588608	0.801453	-0.21284	6	1.09E+08	R	II	LINC00222	Body	opensea	Body-opens		rs1841963	32;18;5;2
cg1875677	0.254379	0.590896	7.661758	7.74E-05	0.002065	2.000992	0.463707	0.718086	-0.25438	10	1.12E+08	R	II	DUSP5	Body	shelf	Body-shelf	chr10:112	rs1382536	42;38;14;5
cg2023813	0.206953	0.680944	7.66027	7.75E-05	0.002066	1.999591	0.577468	0.78442	-0.20695	1	2.46E+08	R	II	KIF26B	Body	opensea	Body-opens		rs1136597	2
cg0603084	0.212907	0.561529	7.657945	7.77E-05	0.002069	1.997402	0.455075	0.667982	-0.21291	3	1.33E+08	F	II	TMEM108	Body	opensea	Body-opens		rs1858090	0;1
cg0536068	0.241023	0.410026	7.657725	7.77E-05	0.002069	1.997196	0.289514	0.530537	-0.24102	14	1.06E+08	R	II		IGR	opensea	IGR-opense		rs1857465	2
cg2223925	0.216726	0.527709	7.657637	7.77E-05	0.002069	1.997112	0.419347	0.636072	-0.21673	10	33271411	F	I		IGR	shore	IGR-shore	chr10:332	rs5452603	1;36
cg0140004	0.232533	0.499102	7.655158	7.79E-05	0.002071	1.994778	0.382836	0.615368	-0.23253	15	61091220	R	II	RORA	Body	opensea	Body-opens			
cg1945606	0.230795	0.524093	7.655086	7.79E-05	0.002071	1.99471	0.408696	0.639491	-0.2308	18	74076380	R	II	ZNF516	Body	opensea	Body-opens		rs1505394	12;6
cg2618836	0.223167	0.631602	7.654944	7.79E-05	0.002072	1.994576	0.520018	0.743185	-0.22317	7	1.37E+08	R	II	MIR490	TSS1500	opensea	TSS1500-op		rs1893552	51;38;27;2;1
cg0025780	0.216087	0.589226	7.651863	7.81E-05	0.002075	1.991674	0.481182	0.69727	-0.21609	2	57938135	R	II		IGR	opensea	IGR-opense		rs5446424	32;2;1
cg1165921	0.23117	0.346215	7.649545	7.83E-05	0.002078	1.989489	0.23063	0.4618	-0.23117	16	71642351	F	II		IGR	opensea	IGR-opense		rs5538458	11;14;30;33
cg1948221	0.252062	0.461339	7.649154	7.83E-05	0.002078	1.98912	0.335308	0.58737	-0.25206	14	1.01E+08	F	II	WDR25	Body	opensea	Body-opens		rs1392904	0;3;13
cg0841204	0.212355	0.478961	7.648005	7.84E-05	0.00208	1.988037	0.372783	0.585138	-0.21235	13	1.1E+08	R	II		IGR	opensea	IGR-opense		rs5584778	35
cg0768486	0.208002	0.480705	7.647799	7.84E-05	0.00208	1.987843	0.376704	0.584706	-0.208	7	94122172	R	II		IGR	opensea	IGR-opense		rs5603975	14;10
cg0772530	0.211598	0.650579	7.647643	7.84E-05	0.00208	1.987696	0.54478	0.756378	-0.2116	5	82730775	F	II		IGR	opensea	IGR-opense		rs1449082	1;33;42
cg2600305	0.206055	0.63111	7.647044	7.85E-05	0.00208	1.987131	0.528083	0.734138	-0.20605	5	89908195	F	II	GPR98	Body	opensea	Body-opens		rs1907576	1;28;35
cg2621775	0.205118	0.61488	7.646449	7.85E-05	0.002081	1.98657	0.512321	0.717439	-0.20512	15	40405659	F	II		IGR	opensea	IGR-opense		rs5595421	1;31
cg0612133	0.205174	0.651206	7.645735	7.85E-05	0.002081	1.985897	0.548619	0.753793	-0.20517	9	77230148	F	II	RORB	Body	opensea	Body-opens		rs1480493	1
cg0515929	0.209726	0.588772	7.644907	7.86E-05	0.002082	1.985116	0.483909	0.693635	-0.20973	10	89870787	F	II		IGR	opensea	IGR-opense		rs1924523	37
cg0654121	0.217135	0.618529	7.644618	7.86E-05	0.002082	1.984844	0.509962	0.727097	-0.21714	2	1.47E+08	F	II		IGR	opensea	IGR-opense		rs1453170	10
cg0423528	0.240251	0.601087	7.643776	7.87E-05	0.002083	1.98405	0.480962	0.721213	-0.24025	2	1.03E+08	R	II	SLC9A4	Body	opensea	Body-opens		rs1908159	46
cg1481912	0.216258	0.642173	7.643305	7.87E-05	0.002084	1.983605	0.534043	0.750302	-0.21626	2	1.93E+08	F	II		IGR	opensea	IGR-opense		rs5285125	1;23;37
cg0731654	0.202714	0.615264	7.641753	7.88E-05	0.002085	1.982142	0.513907	0.716621	-0.20271	9	1.1E+08	R	II		IGR	opensea	IGR-opense		rs5305100	29;17;16
cg0156940	0.212594	0.589436	7.640987	7.89E-05	0.002086	1.981419	0.483139	0.695733	-0.21259	17	8824661	F	II	PIK3R5	5'UTR	opensea	5'UTR-oper		rs1475678	0;1
cg2730929	0.216554	0.585288	7.640755	7.89E-05	0.002086	1.9812	0.477012	0.693565	-0.21655	10	71867356	F	II	H2AFY2	Body	opensea	Body-opens		rs5722344	0;1
cg2202005	0.240709	0.530685	7.640676	7.89E-05	0.002086	1.981125	0.410331	0.65104	-0.24071	6	88417020	F	II		IGR	opensea	IGR-opense		rs5637637	1;6
cg0072183	0.230513	0.645073	7.640066	7.89E-05	0.002087	1.98055	0.529816	0.760329	-0.23051	20	50033738	F	II	NFATC2	Body	opensea	Body-opens		rs8006186	11
cg0348942	0.216512	0.661317	7.639678	7.9E-05	0.002087	1.980184	0.553062	0.769573	-0.21651	8	1.2E+08	F	II		IGR	opensea	IGR-opense			
cg0679343	0.230296	0.596624	7.63795	7.91E-05	0.002089	1.978553	0.481476	0.711772	-0.2303	2	2.32E+08	R	II	ARMC9	Body	opensea	Body-opens		rs1906056	23;15;1
cg1623627	0.234646	0.448156	7.637915	7.91E-05	0.002089	1.97852	0.330833	0.565479	-0.23465	6	87891939	F	II	ZNF292	Body	opensea	Body-opens		rs5755990	10;21;22
cg0092220	0.208208	0.721317	7.636577	7.92E-05	0.002091	1.977257	0.617212	0.825421	-0.20821	7	83742233	F	II	SEMA3A	Body	opensea	Body-opens		rs1322360	1;32;33
cg0894579	0.2609	0.51415	7.635365	7.93E-05	0.002092	1.976114	0.3837	0.6446	-0.2609	13	27571183	R	II		IGR	opensea	IGR-opense		rs5447545	2
cg2101706	0.213457	0.533929	7.634047	7.94E-05	0.002093	1.974869	0.4272	0.640658	-0.21346	13	61001645	R	II	TDRD3	5'UTR	opensea	5'UTR-oper		rs1123258	26
cg0399232	0.233853	0.609466	7.631577	7.95E-05	0.002095	1.972536	0.492539	0.726392	-0.23385	1	2.24E+08	R	II	TP53BP2	Body	opensea	Body-opens		rs7473998	44;41;39
cg1639810	0.201089	0.646833	7.628865	7.97E-05	0.002098	1.969974	0.546289	0.747377	-0.20109	12	66331507	R	II	HMGA2	Body	opensea	Body-opens		rs5318896	24;2
cg0456484	0.204165	0.515394	7.628235	7.98E-05	0.002098	1.96938	0.413312	0.617476	-0.20416	17	67651487	F	II	LINC01483	Body	opensea	Body-opens		rs1385215	0;23;34
cg0291753	0.213555	0.546038	7.627255	7.99E-05	0.002098	1.968454	0.43926	0.652816	-0.21356	6	1.5E+08	F	II	TAB2	Body	shelf	Body-shelf	chr6:1496	rs5336620	14
cg2687632	0.226612	0.553905	7.627018	7.99E-05	0.002099	1.96823	0.440599	0.667211	-0.22661	1	2.45E+08	R	II		IGR	opensea	IGR-opense		rs5782582	13;2
cg1576533	0.235775	0.58615	7.624994	8E-05	0.0021	1.966317	0.468262	0.704037	-0.23578	17	25546334	F	II		IGR	opensea	IGR-opense		rs5694464	0;21;39;44
cg2285697	0.20233	0.65725	7.624645	8E-05	0.002101	1.965987	0.556085	0.758415	-0.20233	1	1.81E+08	R	II		IGR	opensea	IGR-opense		rs1155639	43;2
cg2437185	0.208444	0.646975	7.623824	8.01E-05	0.002101	1.965211	0.542753	0.751197	-0.20844	5	31882166	R	II	PDZD2	Body	opensea	Body-opens		rs6895128	27;26
cg1309345	0.202956	0.699119	7.621085	8.03E-05	0.002104	1.962622	0.59764	0.800597	-0.20296	16	83826869	F	II	CDH13	Body	opensea	Body-opens		rs7996293	44
cg2450249	0.224226	0.401812	7.620889	8.03E-05	0.002104	1.962436	0.289699	0.513924	-0.22423	2	20981591	F	II	LDAH	5'UTR	opensea	5'UTR-oper		rs5354175	1
cg1649647	0.220742	0.585987	7.619745	8.04E-05	0.002105	1.961354	0.475616	0.696358	-0.22074	2	1.15E+08	F	II	LOC10106	Body	opensea	Body-opens		rs5752980	1
cg0360757	0.230941	0.412285	7.619145	8.04E-05	0.002106	1.960786	0.296815	0.527756	-0.23094	11	93471889	R	II	TAF1D	Body	shelf	Body-shelf	chr11:934	rs5711861	50
cg0432415	0.212363	0.709529	7.616826	8.06E-05	0.002108	1.958593	0.603348	0.815711	-0.21236	3	1.68E+08	F	II		IGR	opensea	IGR-opense		rs1809462	0;17;20
cg0675476	0.202807	0.572484	7.616558	8.06E-05	0.002108	1.95834	0.471081	0.673888	-0.20281	6	72253845	R	II		IGR	opensea	IGR-opense		rs5607735	46;16
cg0332020	0.21565	0.567447	7.615804	8.07E-05	0.002109	1.957626	0.459622	0.675272	-0.21565	5	1.67E+08	F	II	TENM2	Body	opensea	Body-opens		rs5611869	1;14
cg1740312	0.206613	0.653271	7.615642	8.07E-05	0.002109	1.957473	0.549965	0.756577	-0.20661	11	6222458	F	II		IGR	opensea	IGR-opense		rs7708650	10;44;45
cg0589847	0.209224	0.556778	7.613147	8.09E-05	0.002111	1.955113	0.452166	0.66139	-0.20922	2	2.03E+08	R	II		IGR	opensea	IGR-opense		rs5753321	1
cg0970083	0.237281	0.46287	7.612308	8.09E-05	0.002112	1.954318	0.34423	0.58151	-0.23728	10	1.24E+08	F	II	ATE1	Body	opensea	Body-opens		rs5778852	3
cg1243151	0.267747	0.579245	7.611934	8.1E-05	0.002112	1.953964	0.445371	0.713118	-0.26775	7	26428084	R	II		IGR	opensea	IGR-opense		rs5350934	46;37
cg0557442	0.245585	0.439826	7.611071	8.1E-05	0.002113	1.953147	0.317033	0.562618	-0.24559	7	1.43E+08	R	II	OR6W1P	TSS1500	opensea	TSS1500-op		rs5717869	1
cg0793974	0.256059	0.371129	7.610276	8.11E-05	0.002114	1.952394	0.2431	0.499159	-0.25606	4	57507828	F	II		IGR	opensea	IGR-opense		rs5697734	1;3;10
cg1800797	0.226635	0.46791	7.61006	8.11E-05	0.002114	1.95219	0.354592	0.581227	-0.22663	19	38918129	F	II	RASGRP4	TSS1500	opensea	TSS1500-op		rs1401830	0;6;26;28;34
cg0827157	0.202273	0.589992	7.609788	8.11E-05	0.002114	1.951933	0.488856	0.691128	-0.20227	9	6683284	R	II		IGR	shore	IGR-shore	chr9:6680	rs5288425	45
cg0184545	0.200339	0.649515	7.608703	8.12E-05	0.002115	1.950905	0.549346	0.749685	-0.20034	21	40204504	R	II		IGR	opensea	IGR-opense		rs5598858	37;35
cg0476683	0.225535	0.588054	7.608553	8.12E-05	0.002115	1.950763	0.475287	0.700822	-0.22553	7	20647070	F	II		IGR	opensea	IGR-opense		rs1911644	1;25;38
cg2363569	0.214991	0.586307	7.607517	8.13E-05	0.002117	1.949782	0.478812	0.693803	-0.21499	22	23131902	F	II		IGR	opensea	IGR-opense		rs5626338	1
cg1233521	0.234979	0.519377	7.607167	8.13E-05	0.002117	1.949451	0.401888	0.636867	-0.23498	4	39661877	F	II		IGR	opensea	IGR-opense		rs1853423	0
cg0354841	0.279709	0.461009	7.606899	8.13E-05	0.002117	1.949197	0.321154	0.600863	-0.27971	11	1.12E+08	R	II		IGR	opensea	IGR-opense		rs5594888	49
cg0846056	0.204563	0.692165	7.606095	8.14E-05	0.002118	1.948435	0.589884	0.794447	-0.20456	7	29950752	R	II	WIPF3	Body	opensea	Body-opens		rs3688830	2
cg0842356	0.238755	0.465731	7.605047	8.15E-05	0.002119	1.947442	0.346353	0.585108	-0.23875	9	20355400	F	II	MLLT3	Body	opensea	Body-opens		rs5312661	25;48
cg1332223	0.211003	0.455196	7.604586	8.15E-05	0.00212	1.947006	0.349695	0.560698	-0.211	15	54025579	R	II	WDR72	5'UTR	opensea	5'UTR-oper		rs5406711	16;13;2;1
cg0614736	0.228452	0.665436	7.603711	8.16E-05	0.002121	1.946177	0.55121	0.779662	-0.22845	12	24689930	R	II	SOX5	5'UTR	opensea	5'UTR-oper			
cg2162972	0.222471	0.719089	7.603515	8.16E-05	0.002121	1.945992	0.607853	0.830324	-0.22247	10	20249114	R	II	PLXDC2	Body	opensea	Body-opens		rs5597220	31;27
cg0101536	0.216845	0.533539	7.603481	8.16E-05	0.002121	1.945959	0.425116	0.641962	-0.21685	3	1.85E+08	F	II	MAP3K13	5'UTR	opensea	5'UTR-oper		rs5492391	1
cg0491079	0.229097	0.561104	7.603093	8.16E-05	0.002122	1.945591	0.446556	0.675652	-0.2291	21	32585992	R	II	TIAM1	Body	opensea	Body-opens		rs3733863	5
[truncated: 665,908 more chars]
